# Polygenic risk scores for pan-cancer risk prediction in the Chinese population: A population-based cohort study based on the China Kadoorie Biobank

**DOI:** 10.1371/journal.pmed.1004534

**Published:** 2025-02-28

**Authors:** Meng Zhu, Xia Zhu, Yuting Han, Zhimin Ma, Chen Ji, Tianpei Wang, Caiwang Yan, Ci Song, Canqing Yu, Dianjianyi Sun, Yue Jiang, Jiaping Chen, Ling Yang, Yiping Chen, Huaidong Du, Robin Walters, Iona Y Millwood, Juncheng Dai, Hongxia Ma, Zhengdong Zhang, Zhengming Chen, Zhibin Hu, Jun Lv, Guangfu Jin, Liming Li, Hongbing Shen, on behalf of the China Kadoorie Biobank Collaborative Group

**Affiliations:** 1 Department of Epidemiology, Center for Global Health, School of Public Health, Nanjing Medical University, Nanjing, China; 2 Jiangsu Key Lab of Cancer Biomarkers, Prevention and Treatment, Collaborative Innovation Center for Cancer Medicine and China International Cooperation Center for Environment and Human Health, Nanjing Medical University, Nanjing, China; 3 Department of Chronic Non-Communicable Disease Control, The Affiliated Wuxi Center for Disease Control and Prevention of Nanjing Medical University, Wuxi Center for Disease Control and Prevention, Wuxi Medical Center, Nanjing Medical University, Wuxi, China; 4 Department of Epidemiology & Biostatistics, School of Public Health, Peking University, Beijing, China; 5 Peking University Center for Public Health and Epidemic Preparedness & Response, Beijing, China; 6 Key Laboratory of Epidemiology of Major Diseases (Peking University), Ministry of Education, Beijing, China; 7 Medical Research Council Population Health Research Unit at the University of Oxford, Oxford, United Kingdom; 8 Clinical Trial Service Unit & Epidemiological Studies Unit (CTSU), Nuffield Department of Population Health, University of Oxford, Oxford, United Kingdom; 9 Genomic Science and Precision Medicine Institute, Gusu School, Nanjing Medical University, Nanjing, China; 10 State Key Laboratory of Vascular Homeostasis and Remodeling, Peking University, Beijing, China; National Cancer Institute, UNITED STATES OF AMERICA

## Abstract

**Background:**

Polygenic risk scores (PRSs) have been extensively developed for cancer risk prediction in European populations, but their effectiveness in the Chinese population remains uncertain.

**Methods and findings:**

We constructed 80 PRSs for the 13 most common cancers using seven schemes and evaluated these PRSs in 100,219 participants from the China Kadoorie Biobank (CKB). The optimal PRSs with the highest discriminatory ability were used to define genetic risk, and their site-specific and cross-cancer associations were assessed. We modeled 10-year absolute risk trajectories for each cancer across risk strata defined by PRSs and modifiable risk scores and quantified the explained relative risk (ERR) of PRSs with modifiable risk factors for different cancers. More than 60% (50/80) of the PRSs demonstrated significant associations with the corresponding cancer outcomes. Optimal PRSs for nine common cancers were identified, with each standard deviation increase significantly associated with corresponding cancer risk (hazard ratios (HRs) ranging from 1.20 to 1.76). Compared with participants at low genetic risk and reduced modifiable risk scores, those with high genetic risk and elevated modifiable risk scores had the highest risk of incident cancer, with HRs ranging from 1.97 (95% confidence interval (CI): 1.11–3.48 for cervical cancer, *P* = 0.020) to 8.26 (95% CI: 1.92–35.46 for prostate cancer, *P* = 0.005). We observed nine significant cross-cancer associations for PRSs and found the integration of PRSs significantly increased the prediction accuracy for most cancers. The PRSs contributed 2.6%–20.3%, while modifiable risk factors explained 2.3%–16.7% of the ERR in the Chinese population.

**Conclusions:**

The integration of existing evidence has facilitated the development of PRSs associated with nine common cancer risks in the Chinese population, potentially improving clinical risk assessment.

## Author summary

**Why was this study done?:**

- Most polygenic risk scores (PRSs) for cancer risk prediction are developed based on European populations, limiting their applicability to other ancestries, such as the Chinese population.
- PRSs for lung and stomach cancers have been developed for Chinese populations, but they differ from European-derived PRSs in terms of genetic composition and effects.
- Due to smaller genome-wide association studies sample sizes and fewer identified genetic loci, optimal PRSs for other common cancers in the Chinese populations are still lacking.

**What did the researchers do and find?:**

- We developed 80 PRSs for 13 common cancers in Chinese populations and evaluated them in an independent prospective cohort of 100,000 participants.
- Nine optimal PRSs were identified, with improved accuracy for cancer risk prediction when incorporating East Asian-specific genetic loci, along with loci validated in Asian populations.
- We provided 10-year cancer risk projections, highlighting the combined impact of genetic and modifiable risk factors. PRSs significantly improved cancer risk prediction, particularly for colorectal, cervical, and prostate cancers.

**What do these findings mean?:**

- This study demonstrates the potential of leveraging existing genetic data to improve cancer risk assessment in Chinese populations.
- The findings provide valuable tools for cancer risk stratification, supporting the development of precision prevention strategies in China.
- Limitations include the relatively small number of cases for certain cancers and the lack of cancer subtype information, which may affect the generalizability of the results. Larger studies with detailed cancer classification are needed to validate these findings.

## Abstract 3

Meng Zhu and colleagues use data from the China Kadoorie Biobank to develop polygenic risk scores for cancer in Chinese populations.

## Introduction

Cancer is a leading cause of death worldwide, with more than one-fourth of new cases and one-third of cancer-related deaths occurring in China [1]. It is widely acknowledged that both heritable genetic factors and modifiable risk factors contribute to the development of cancer. In recent years, genome-wide association studies (GWASs) have unveiled a multitude of genetic variants associated with the risk of cancer, thereby providing insight into the genetic mechanisms of cancer susceptibility [2]. However, over 81% of participants in published GWAS are of European ancestry [3].

Panels of single-nucleotide polymorphisms (SNPs) from GWAS have been used to generate polygenic risk scores (PRSs) for quantifying an individual’s inherent risk. Multiple studies have demonstrated that PRS can effectively predict the incidence of cancer and improve cancer risk assessment in combination with modifiable exposures [4,5]. Given the ability of PRS to identify larger proportions of the population at comparable or elevated risk compared to rare monogenic mutations, it is generally accepted that PRS offers greater potential for cancer clinical practice [6]. The Polygenic Score (PGS) Catalog has documented 519 PRSs for 64 cancer types (MONDO_0004992), of which 506 are related to European ancestry by December 2023 [7]. The predictive performance of PRS diminishes across diverse populations, which hinders their clinical utility in diverse populations and would exacerbate healthcare disparities [8]. There is an urgent need to improve the accuracy of polygenic prediction in different ancestry populations to maximize the clinical potential of PRS.

Over the past decade, genetic studies in East Asian countries (e.g., China and Japan) have experienced some growth [9], with 65 PRSs related to eight cancer types recorded for East Asians in the PGS Catalog (MONDO_0004992) [7]. However, most of these PRSs (57/65) were derived from a multi-ancestry meta-analysis with limited representation of East Asian populations, and the performance of these PRSs remained to be improved. Recently, novel cross-population PRS construction methods (i.e., PRS-CSx) were developed and showed the ability to improve prediction accuracy across different populations [10]. In Chinese populations, we have developed a panel of PRSs for lung cancer (PRS-19) and gastric cancer (PRS-112) based on large-scale GWAS data, which showed substantial differences compared to those of European ancestry [11–13]. Nevertheless, due to insufficient GWAS data, the optimal PRSs for other cancer types remain to be explored among Chinese populations.

A crucial step in realizing the potential of PRS in precision medicine involves systematically evaluating the added value of genetic information compared to traditional risk factors and assessing its impact on lifetime risk trajectories. Moreover, a recent study demonstrated the cross-cancer portability of PRS in line with widespread pleiotropy of cancer susceptibility loci [14]. In this study, we compiled 80 PRSs for the 13 most common cancers with seven schemes in the Chinese population. We then assessed their predictive performance, explored cross-cancer associations, and evaluated their contribution to cancer risk prediction. The primary aim of this study is to determine whether PRSs can significantly enhance cancer risk prediction and stratification in the Chinese population, particularly in comparison to environmental risk factors, using data from a large cohort of 100,219 participants.

## Methods

### Study participants and design

The China Kadoorie Biobank (CKB) cohort is a nationwide prospective cohort in China. The study design, methods, and Institutional Review Board (IRB) approval for the CKB have been described previously [15,16]. In brief, a total of 512,726 Chinese adults aged 30−79 years were recruited from 10 geographically diverse regions across China between 2004 and 2008. All participants completed an interviewer-administered electronic questionnaire regarding lifestyle and other health-related information, as well as provided physical measurements and a blood sample at baseline. All eligible participants in CKB completed a written informed consent form approved by the IRB. The CKB cohort was approved by the Ethical Review Committee of the Chinese Center for Disease Control and Prevention (Beijing, China) (No. 005/2004) and the Oxford Tropical Research Ethics Committee at the University of Oxford (Oxford, UK) (No. 025-04).

A total of 100,640 participants were selected for genotyping based on a clustered random selection method and finally passed quality control. Individuals with cancers diagnosed at baseline were excluded from the analysis (*n* = 421), leaving 100,219 eligible participants in the final analysis. Details of genotyping, quality control, and imputation for the CKB cohort had been reported previously (S1 Text) [17].

**Fig 1:**
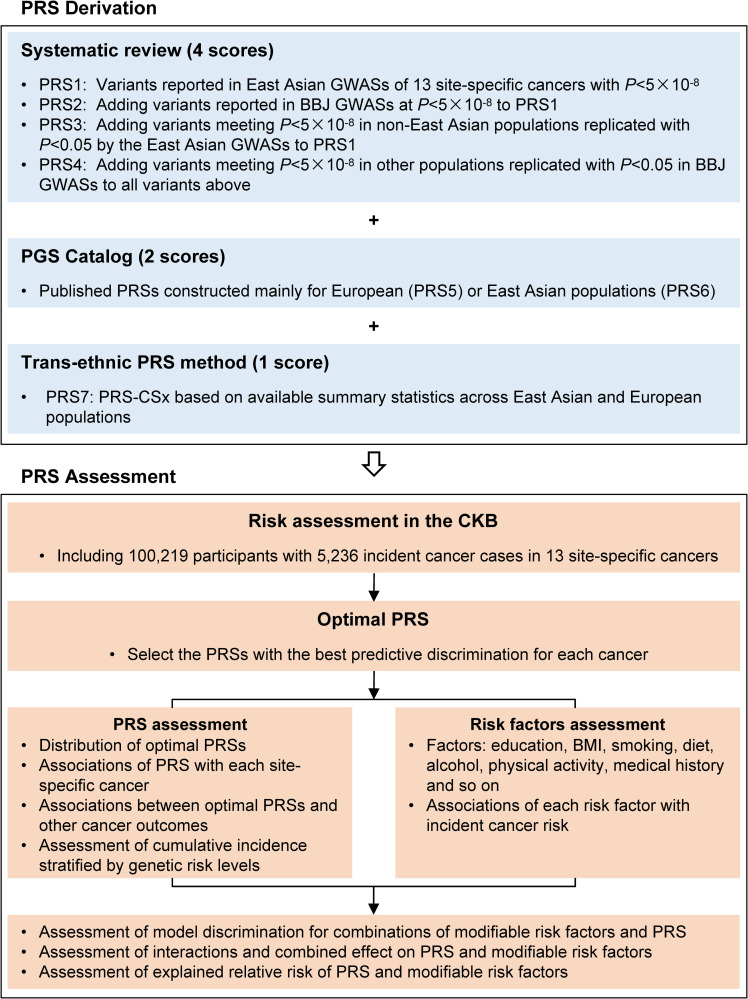
Study design and workflow. PRS, polygenic risk score; GWAS, genome-wide association study; BBJ, BioBank Japan Project; PGS, polygenic score; CKB, China Kadoorie Biobank; BMI, body mass index.

As shown in Fig 1, a two-stage analysis was included in this study. In the first stage, we constructed seven PRSs for each cancer based on published GWAS (4 scores), retrieved recorded PRSs from the PGS Catalog (2 scores), and used the PRS-CSx to construct cross-population PRSs (1 score). In the second stage, we evaluated the effectiveness of these PRSs in predicting the risk of corresponding cancers, exploring cross-cancer associations, and examining their combination with modifiable risk factors for cancer risk prediction in an independent prospective cohort of the CKB. None of the studies used to construct the PRS included data from the CKB cohort. This study is reported as per the Strengthening the Reporting of Genetic Association Studies (STREGA) guideline (S1 STREGA Checklist) [18].

### PRS construction

By using three strategies, we constructed seven PRSs for each of the 13 cancer types that were available in the CKB (S2 Text). In the first strategy, we initially conducted a comprehensive search for GWASs on each cancer type in populations of East Asian ancestry. Subsequently, we generated PRS for each cancer type using genetic variants with a significance threshold of *P* < 5 × 10^−8^ in East Asian GWASs (PRS1), additionally incorporated variants with *P* < 5 × 10^−8^ in BioBank Japan Project (BBJ) (PRS2) [19], added variants from other ethnic populations that were replicated with *P* < 0.05 in East Asian GWASs (PRS3), and finally added variants from other ethnic populations that were replicated with *P* < 0.05 in BBJ GWASs (PRS4). In the second strategy, we extracted parameters of PRS from the PGS Catalog constructed mainly for European (PRS5) or East Asian populations (PRS6). Details of PRSs for each cancer eligible from the PGS catalog are shown in S1 Table. In the third strategy, we applied a newly developed Bayesian polygenic modeling method, PRS-CSx, utilizing available GWAS summary statistics from both East Asian and European populations [10]. Details of the GWAS summary statistics can be found in S2 Table. We generated PRSs for the 13 cancer types by using shared variants in the two summary statistics (PRS7). We selected the PRSs with the best predictive discrimination for each outcome across different strategies and parameters as the optimal PRSs.

At the beginning of our study, there were no previously published PRSs for cancers of the head and neck, esophagus, liver, pancreas, cervix, endometrium, ovary, and bladder in East Asian populations. Additionally, there were no genome-wide significant SNPs identified for endometrial cancer in East Asian populations. Consequently, we generated 80 PRSs for 13 cancer types in our analyses.

### Assessment of risk factors

Risk factors in addition to age, sex (if applicable), and cancer family histories, such as environmental exposures, lifestyle factors, dietary patterns, and medical history were obtained from interviewer-administered electronic questionnaires at baseline in the CKB cohort (S3 Text). The selection of modifiable risk factors was based on a comprehensive literature review and reports. Furthermore, only established environmental and lifestyle-related characteristics that were collected and available in the CKB cohort were included. We selected socio-demographic characteristics (including highest education level and study region), lifestyles (including smoking status, alcohol consumption, physical activity, dietary habits, and body mass index), and medical conditions. Detailed information on the assessed risk factors in this study can be found in S3 Table.

### Outcomes

Participants in the CKB cohort were followed up for cancer events mainly through ongoing electronic linkage with official death certificates, chronic disease registries, and the Chinese National Health Insurance claim database semi-annually. This was supplemented with active follow-up by the study staff for local residential records annually. The trained study staff, who were masked to baseline information, described the outcomes according to the International Classification of Diseases 10th Revision codes. The complete follow-up was updated to December 31, 2017. We extracted the outcomes of 13 solid tumors with the highest incidence in the Chinese population, including head and neck cancer (C00–C14), esophageal cancer (C15), stomach cancer (C16), colorectal cancer (C18–C20), liver cancer (C22), pancreatic cancer (C25), lung cancer (C33–C34), breast cancer (C50), cervical cancer (C53), endometrial cancer (C54.1), ovarian cancer (C56), prostate cancer (C61), and bladder cancer (C67).

### Statistical analysis

Participants’ site-specific cancer risk was assessed from enrollment until the time of cancer diagnosis, death, loss to follow-up, or the end of follow-up, using Cox proportional hazards regression to estimate hazard ratios (HRs) with 95% confidence intervals (CIs) in the CKB cohort. For testing the proportional hazards assumption for PRSs and risk factors, we used the Schoenfeld residuals. The C-index was introduced by Harrell as a natural extension of the receiver operating characteristic curve area to survival analysis, which represents the predictive discrimination. The performance of a PRS-model, in distinguishing between those who will develop cancer from those who will remain unaffected can be quantified by Harrell’s C-index. The site-specific PRS with the highest predictive discrimination was selected as the optimal PRS for each cancer.

Each optimal PRS was then evaluated in multivariable Cox regression models specific to the cancer type for which it was developed, as well as for each of the other cancer types, adjusting for age, sex (except for sex-specific cancers), region, and the top 10 genetic ancestry principal components. Site-specific cancer genetic risk was categorized into low (the bottom quintile), intermediate (quintiles 2–4), and high (the top quintile) according to quintile cut-off points of the optimal PRSs in the CKB population, as previously described [11]. Modifiable risk factors were summarized by generating summary linear predictors based on risk factors in S3 Table [5], and individuals above the median of the risk score were considered to have elevated modifiable risk factors. Further, for testing the individual associations of genetic risk and risk factors with age at cancer onset, we dichotomized the study population by the age onset of cancer according to the median age of diagnosis within the Chinese population [20] and fitted Cox models allowing different association coefficients within different age groups of onsets.

The Cox regression model was used to estimate the 10-year absolute risk (*P*) of each cancer as follows:


$$P=1-S_{0}{\left(t\right)}^{\text{exp}\left(\overset{N}{\underset{i=1}{\sum }}{\text{β}}_{i}\left(x_{i}-M_{i}\right)\right)}.$$

where *S*_0_(*t*) is the baseline survival rate calculated at the mean values of the variables at time *t* (*t* = 10), *M*_*i*_ is the mean values of the variables, *β*_*i*_ is the regression coefficients, *x*_*i*_ represents variables, and *N* is the number of variables in the model. The 10-year absolute risk trajectories of each cancer across strata defined by genetic and modifiable risk factors were visualized by fitting linear models with smoothing splines to individual risk estimates as a function of age. We also estimated cumulative risk in each stratum with index age 40 up to age 80 years using cause-specific Cox proportional hazard models, treating death from any cause as a competing event.

Finally, we developed cancer-specific prediction models with three classes of risk factors progressively: (i) demographic factors (age, sex, and region) and cancer family history; (ii) modifiable risk factors; and (iii) genetic susceptibility. The improvement of risk discrimination was assessed based on the C-index and area under the curve (AUC) at 10 years. The continuous net reclassification index (NRI) was used to quantify improvements in reclassification, which is a more objective and versatile measure of improvement in risk prediction [21]. Sensitivity analyses were conducted: (i) excluding participants within the first year after recruitment; (ii) only including the first primary cancer. The relative predictive performance of the PRS and summarized linear risk factors for predicting the outcome was evaluated using Heller *R*^2^ for the explained relative risk (ERR) in the proportional hazards model using the “clinfun” software package in R. The ERR was selected because it provides a quantifiable measure for the importance of each variable in the model and it is robust to censoring that is independent of survival time conditional on the variables [22]. The CIs for ERR were estimated using 1,000 bootstrapped iterations. All *P*-values in this study were two-sided, and *P* < 0.05 was considered statistically significant. All statistical analyses were performed by using R version 4.3.1 (R Core Team, Vienna, Austria).

## Results

### Study population

Over a median follow-up of 11.33 years (interquartile range: 10.18–12.26), 5,236 incident cancer cases in 13 site-specific cancers were identified among 100,219 participants in the CKB cohort (Table 1). The mean age of incident cancer cases was older, more likely to be men, and had a higher prevalence of smoking and drinking at baseline than cancer-free individuals. The most common cancer types were lung (*n* = 1,540), stomach (*n* = 745), colorectal (*n* = 740), liver (*n* = 661), esophageal (n = 499), and breast cancers (*n* = 486), accounting for 83.58% of all newly diagnosed cancer events (S4 Table).

**Table 1:** Baseline characteristics of the participants in the CKB.

	Total (n = 100,219)	Status at the end of follow-up	
		Cancer-free (n = 94,983)	Incident cancer (n = 5,236)
Age at baseline (years)			
Mean (SD)	53.69 (11.01)	53.37 (10.97)	59.46 (9.93)
Sex			
Men	42,860 (42.8%)	40,142 (42.3%)	2,718 (51.9%)
Women	57,359 (57.2%)	54,841 (57.7%)	2,518 (48.1%)
Highest level of education			
College or up	5,950 (5.9%)	5,660 (6.0%)	290 (5.5%)
High school	14,185 (14.2%)	13,575 (14.3%)	610 (11.7%)
Middle school	26,856 (26.8%)	25,654 (27.0%)	1,202 (23.0%)
Primary school/no formal school	53,228 (53.1%)	50,094 (52.7%)	3,134 (59.9%)
Body-mass index (kg/m^2^)			
Mean (SD)	23.66 (3.49)	23.67 (3.49)	23.58 (3.60)
18.5 ≤ BMI < 24	50,861 (50.7%)	48,259 (50.8%)	2,602 (49.7%)
BMI < 18.5	5,192 (5.2%)	4,856 (5.1%)	336 (6.4%)
BMI ≥ 24	44,166 (44.1%)	41,868 (44.1%)	2,298 (43.9%)
Smoking status			
Never	65,736 (65.6%)	62,878 (66.2%)	2,858 (54.6%)
Smoker/ex-smoker	34,483 (34.4%)	32,105 (33.8%)	2,378 (45.4%)
Pack-years of smoking (<30)	22,093 (22.0%)	20,879 (22.0%)	1,214 (23.2%)
Pack-years of smoking (≥30)	12,390 (12.4%)	11,226 (11.8%)	1,164 (22.2%)
Alcohol status			
Never	80,158 (80.0%)	76,349 (80.4%)	3,809 (72.7%)
Drinker/abstainer	20,061 (20.0%)	18,634 (19.6%)	1,427 (27.3%)
Meat intake			
Monthly/never	17,183 (17.1%)	16,333 (17.2%)	850 (16.2%)
1–3 days/week	35,595 (35.5%)	33,644 (35.4%)	1,951 (37.3%)
≥4 days/week	47,441 (47.3%)	45,006 (47.4%)	2,435 (46.5%)
Salty vegetables intake			
≤3 days/week	77,143 (77.0%)	73,247 (77.1%)	3,896 (74.4%)
≥4 days/week	23,076 (23.0%)	21,736 (22.9%)	1,340 (25.6%)
Vegetables and fruits intake*			
Frequent	27,622 (27.6%)	26,150 (27.5%)	1,472 (28.1%)
Occasional	72,597 (72.4%)	68,833 (72.5%)	3,764 (71.9%)
Fruits intake			
≥1 day/week	59,372 (59.2%)	56,311 (59.3%)	3,061 (58.5%)
Monthly/never	40,847 (40.8%)	38,672 (40.7%)	2,175 (41.5%)
Physical activity (MET hours/day)			
Mean (SD)	19.89 (13.77)	20.05 (13.80)	17.03 (12.95)
Family history of cancer			
No	83,578 (83.4%)	79,403 (83.6%)	4,175 (79.7%)
Yes	16,641 (16.6%)	15,580 (16.4%)	1,061 (20.3%)

*Frequent intake of fresh vegetables and fruits was defined as eating vegetables every day and fruits ≥4 days per week or eating fruits every day and vegetables ≥4 days per week, otherwise was less frequent.

CKB, China Kadoorie Biobank; SD, standard deviation; BMI, body mass index.

### Construction of optimal cancer-specific PRS

**Fig 2:**
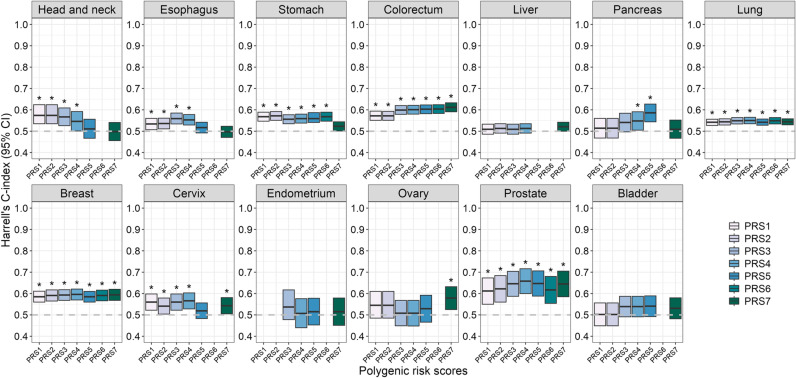
Assessment of discrimination based on Harrell’s C-index in the PRS-only model using Cox proportional hazards regression. The Harrell’s C-indices and their 95% CIs were estimated by Cox proportional hazard models. The statistical tests were two sides. The box limits represent 95% CIs and their centers represent the C-indices. Associations with statistically significant (*P*-value < 0.05) were annotated with an asterisk. PRS, polygenic risk score.

Among the 80 PRSs constructed using seven different schemes, over 60% (50/80) of PRSs were associated with the risk of cancer outcomes in the CKB cohort. At least one of the seven PRSs showed predictive performance in 10 different cancer types (Fig 2). We selected optimal site-specific PRSs with the best predictive discrimination with each cancer outcome according to the C-index in the PRS-only model. The optimal PRSs with the highest C-index were: PRS4 for lung, breast, cervical, and prostate cancers; PRS7 for colorectal and ovarian cancers; PRS1 for head and neck cancer; PRS2 for stomach cancer; PRS3 for esophageal cancer; and PRS5 for pancreatic cancer, which mostly showed the strongest association with each cancer outcome. After adjusting for potential confounders, no PRSs were associated with the incident risk of head and neck cancer. Therefore, we retained the remaining nine optimal PRSs for defining genetic risk in subsequent analyses (S5 Table). All weights, including comprehensive lists of sources of optimal PRSs, are provided in S1 Data.

The distribution of the nine optimal PRSs for each cancer site showed approximately normal distribution, with cancer-affected participants exhibiting higher PRSs than unaffected ones (S1 Fig). Each standard deviation increase in PRSs was significantly associated with the risk of the nine cancers (*P* < 0.05), with HRs ranging from 1.20 to 1.76 (Table 2). The risk of developing site-specific cancer significantly increased from quintile 1 to quintile 5 of PRSs in a positive dose–response manner (*P*_*trend*_ < 0.05). Compared with participants at low genetic risk (the bottom quintile of PRS), participants at high genetic risk (the top quintile) exhibited more than double for esophageal cancer (HR = 2.05, 95% CI: 1.55–2.71, *P* < 0.001), stomach cancer (HR = 2.04, 95% CI: 1.60–2.61, *P* < 0.001), colorectal cancer (HR = 3.25, 95% CI: 2.53–4.18, *P* < 0.001), pancreatic cancer (HR = 2.51, 95% CI: 1.47–4.27, *P* < 0.001), breast cancer (HR = 2.58, 95% CI: 1.88–3.53, *P* < 0.001), ovarian cancer (HR = 2.68, 95% CI: 1.29–5.54, *P* = 0.008), and prostate cancer (HR = 3.28, 95% CI: 1.62–6.63, *P* < 0.001) (S6 Table and S2 Fig). These results did not change significantly after additional adjustment for modifiable risk factors (S6 Table), exclusion of participants within the first year of follow-up, or only including the first primary cancer (S7 and S8 Tables). The 10-year AUC, sensitivity, and specificity of the nine optimal PRSs were shown in S9 Table. Cumulative incidence curves for different genetic risk groups demonstrated the expected risk stratification of site-specific cancer after adjusting for confounders (S3 Fig). Besides, individuals with high genetic risk exhibited a stronger association with stomach cancer (HR: 2.75 versus 1.53, *P*_*het*_ = 0.020) and cervix cancer (HR: 3.27 versus 1.35, *P*_*het*_ = 0.044) in patients with lower onset ages (S10 Table).

**Table 2:** The performance metrics of the optimal PRSs in the CKB cohort by cancer site.

Cancer site	Cancer events	Incidence rate (per 100,000 person-years)	HR per s.d. (95% CI)*	*P-*value *	C-index †	Top 20% vs. other 80% *	
						HR (95% CI)	*P-*value
Esophagus	499	46.53	1.28 (1.17–1.39)	<0.001	0.559	1.67 (1.38–2.04)	<0.001
Stomach	745	69.49	1.27 (1.18–1.37)	<0.001	0.572	1.48 (1.26–1.74)	<0.001
Colorectum	740	69.09	1.54 (1.44–1.66)	<0.001	0.612	2.19 (1.88–2.55)	<0.001
Pancreas	170	15.84	1.28 (1.11–1.49)	<0.001	0.586	1.57 (1.13–2.20)	0.008
Lung	1,540	143.76	1.22 (1.16–1.28)	<0.001	0.550	1.38 (1.23–1.55)	<0.001
Breast	486	77.49	1.41 (1.29–1.54)	<0.001	0.596	1.60 (1.31–1.95)	<0.001
Cervix	237	37.72	1.20 (1.06–1.36)	0.004	0.566	1.74 (1.31–2.29)	<0.001
Ovary	96	15.26	1.25 (1.02–1.53)	0.031	0.579	1.56 (1.00–2.44)	0.051
Prostate	95	21.40	1.76 (1.44–2.16)	<0.001	0.658	2.28 (1.50–3.46)	<0.001

*The model was adjusted for age, sex (if applicable), region, and the top 10 principal components.

†C-index in the Cox proportional hazards regression model with only the optimal PRS for each cancer.

PRS, polygenic risk score; CKB, China Kadoorie Biobank; HR, hazard ratio; CI, confidence interval.

### Cross-cancer PRS associations

**Fig 3:**
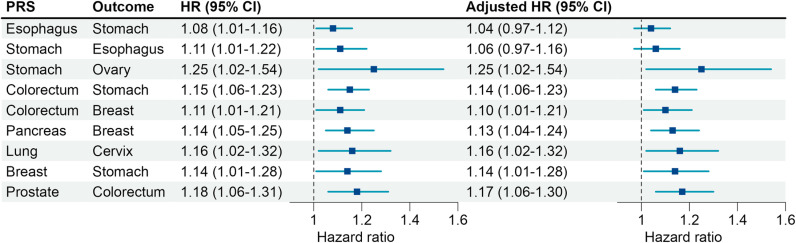
Hazard ratios for significant cross-cancer associations. HRs were estimated using a Cox regression model adjusted for age, sex (if applicable), region, and the top 10 principal components. The adjusted HRs were further adjusted for the corresponding site-specific PRSs. The error bars represent 95% CIs and their centers represent the HRs. PRS, polygenic risk score; HR, hazard ratio; CI, confidence interval.

In the CKB cohort, 20 site-specific PRS pairs showed small correlations with each other (*r* < 0.2) (S4 Fig). Most participants (78.9%, 79,075/100,219) were at high genetic risk for at least one of the nine cancer types (S11 Table). Cross-cancer associations between each PRS and the other eight cancer types revealed nine significant positive associations (Fig 3). After further adjusting for the corresponding site-specific PRSs, seven cross-cancer associations remained unchanged, and similar results were observed in men and women, respectively (Figs 3 and S5). Three associations between a PRS and cross-cancer outcome were found after correction for multiple testing (*P* < 0.05/9 = 0.0055): colorectal cancer PRS with stomach cancer (HR = 1.15, 95% CI: 1.06–1.23, *P* < 0.001); pancreatic cancer PRS with breast cancer (HR = 1.14, 95% CI: 1.05–1.25, *P* = 0.003); and prostate cancer PRS with colorectal cancer (HR = 1.18, 95% CI: 1.06–1.31, *P* = 0.002). Results remained materially unchanged correcting for the false discovery rate at *q* < 0.05 (S12 Table). Furthermore, after excluding shared SNPs or SNPs in strong linkage disequilibrium (*r*^2^ > 0.6), two associations were no longer significant, while the other associations were largely unchanged (S6 Fig).

### Associations of modifiable risk factors

The associations of established modifiable risk factors for the nine cancers in the CKB cohort were concordant with previous studies (S13 Table). Participants with elevated modifiable risk (above the median modifiable score) showed a significantly increased risk of incident cancer, with HRs ranging from 1.19 to 2.91 (S14 Table). Notably, stronger associations (HR ≥ 1.5) were observed for esophageal cancer (HR = 1.85, 95% CI: 1.44–2.38, *P* < 0.001), lung cancer (HR = 1.80, 95% CI: 1.58–2.05, *P* < 0.001), breast cancer (HR = 1.71, 95% CI: 1.36–2.13, *P* < 0.001), ovarian cancer (HR = 2.91, 95% CI: 1.79–4.74, *P* < 0.001), and prostate cancer (HR = 1.97, 95% CI: 1.17–3.31, *P* = 0.010). The associations between modifiable risk and incident cancer risk remained essentially unchanged after adjustment for the genetic risk (S14 Table). Compared with reduced modifiable risk, the elevated risk was borderline associated with a greater risk of esophageal cancer in individuals with lower onset ages (S15 Table).

### Risk stratification based on the genetic risk and modifiable risk factors

**Fig 4:**
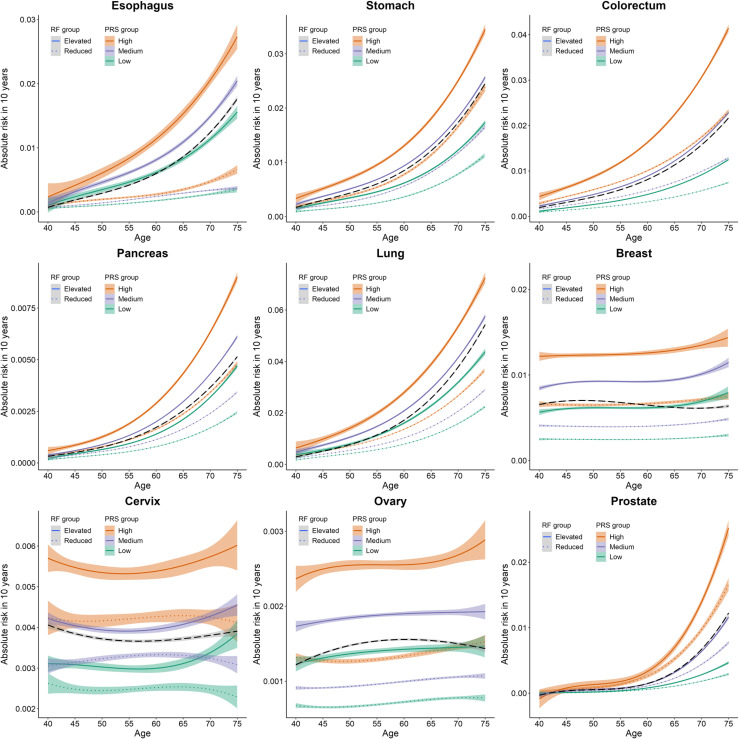
Predicted 10-year absolute risk trajectories across strata defined by PRS and modifiable risk factors. Participants were categorized into six groups according to genetic risk (high risk: the top quintile; medium: quintile 2–4, low risk: the bottom quintile) and modifiable risk factors (elevated: above the median; reduced: below the median). Average 10-year absolute risk trajectories across all individuals were visualized by black dashed lines. The error bars represent 95% CIs and their centers represent the average 10-year absolute risk. RF, modifiable risk factors; PRS, polygenic risk score.

To evaluate the joint impact of genetic risk and modifiable risk factors on individuals’ absolute risk with increasing age, we estimated the 10-year absolute risk trajectories and cumulative risk by age 80 for the nine cancers (Figs 4 and S7). The span of the 10-year absolute risk trajectory was increasingly notable with older age except for breast, cervical, and ovarian cancers (S8 Fig). For colorectal, prostate, and cervical cancers, participants with a high PRS were predicted to have an overall risk above average, even when they had reduced modifiable risk scores (Fig 4). Participants with high genetic risk and elevated modifiable risk scores (around 10% of total participants) had the highest risk of incident cancer, with HRs ranging from 1.97 (95% CI: 1.11–3.48 for cervical cancer, *P* = 0.020) to 8.26 (95% CI: 1.92–35.46 for prostate cancer, *P* = 0.005) compared with participants at low genetic risk and reduced modifiable risk scores (S16 Table). We also calculated the absolute risk reduction (ARR) according to genetic risk and modifiable risk factors. Change from an elevated risk profile to a reduced one provided 4.3-fold, 3.2-fold, and 2.8-fold greater ARRs for prostate, colorectal, and cervical cancers, respectively, among participants with high genetic risk than those with low genetic risk (S9 Fig). However, there were no significant interactions between genetic risk and modifiable risk scores for the nine cancers (S17 Table).

### Improvement in risk prediction

**Fig 5:**
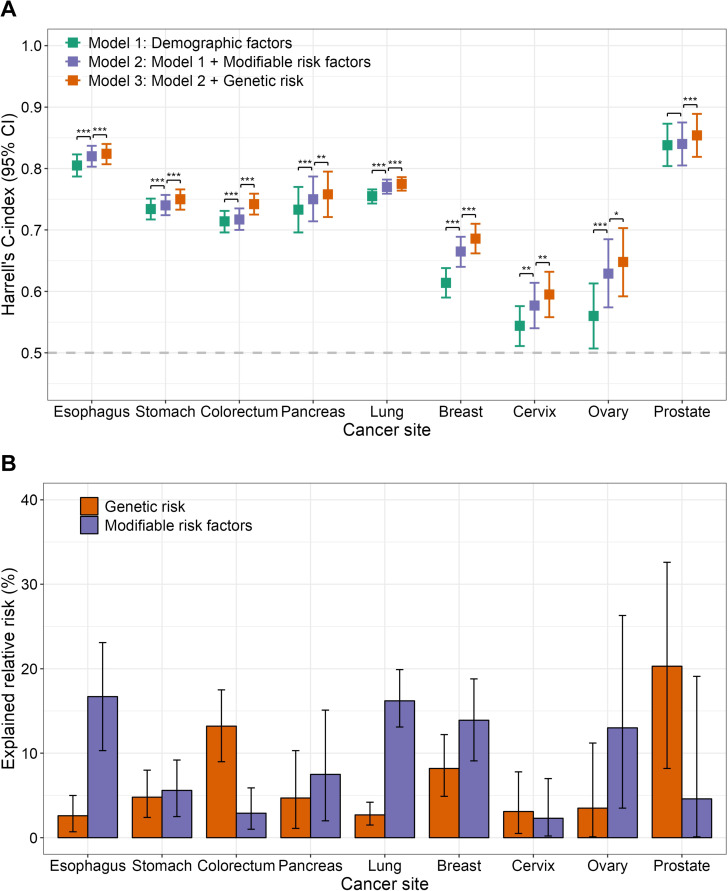
Assessment of model discrimination based on Harrell’s C-index (A) and the explained relative risk for PRS and summarized risk factors (B). **(A)** The Harrell’s C-indices and their 95% CIs were estimated by Cox proportional hazard models. Comparisons were conducted across nested models: Model 1: Including demographic factors (age, sex, and region) and family history of cancer. Model 2: Adding summarized modifiable risk factors to Model 1. Model 3: Adding genetic susceptibility, represented by the PRS to Model 2. The likelihood-ratio test was performed between Models 2 and 1, as well as between Models 3 and 2. The significance levels were denoted by asterisks as follows: *P-value < 0.05, **P-value < 0.01, and ***P-value < 0.001. The error bars represent 95% CIs and their centers represent the C-indices. **(B)** The explained relative risk was derived from Cox proportional hazard regression models that were adjusted for age, sex (if applicable), region, and family cancer history. The confidence intervals were estimated using 1,000 bootstrapped iterations. The error bars represent 95% CIs and their centers represent the explained relative risk.

The predictive discrimination of each risk model was assessed by Harrell’s C-index, and we also reported the AUC at 10 years of follow-up time for completeness. The C-index of the basic model based on demographic factors and cancer family history reached 0.60 for all cancers except for the cervix and ovary (S18 Table). The improvement in risk prediction of models incorporating the modifiable risk factors and the optimal site-specific PRSs compared with the basic model was variable (Fig 5A). For cancer sites with more available risk factors, incorporating this had a better impact on the C-index, such as esophagus (*C* = 0.820, Δ*C* = 0.015), lung (*C* = 0.770, ΔC = 0.015), breast (*C* = 0.665, Δ*C* = 0.051), and ovary (*C* = 0.629, Δ*C* = 0.069), and the same improvements in reclassification as indicated by continuous NRI were also observed. Next, adding PRSs to the risk models which have multiple risk factors resulted in a modest increase (Δ*C* ≥ 0.01) in C-index for stomach cancer (from 0.740 to 0.750), colorectal cancer (from 0.717 to 0.742), and breast cancer (from 0.665 to 0.686). A large increase in the C-index after incorporating the PRS was observed for prostate cancer (from 0.840 to 0.854) with few available predictors as well. Changes in the AUC at 10 years of follow-up were of similar magnitude. Continuous NRI values > 0.15 after incorporating the PRS were observed for colorectal, pancreatic, and prostate cancers (S18 Table). Interestingly, additional incorporation of the significant cross-cancer PRSs can lead to a further improvement of the model’s performance, such as the 10-year AUC of colorectal cancer in men increasing from 0.738 to 0.746 (DeLong’s test *P* = 0.009) after adding esophageal cancer PRS and prostate cancer PRS (S19 Table). The sensitivity analyses, excluding participants within the first year after recruitment or only including the first primary cancer, showed results comparable to the main findings (S20 and S21 Tables). In addition, the performance of cancer-specific prediction models was largely unchanged when considering the use of interactions, non-linear terms, or flexible parametric survival models (S22 Table).

The relative importance as measured by the ERR values for modifiable risk factors and site-specific PRSs are shown in Fig 5B. The identified modifiable risk factors and site-specific PRSs can explain 5.4%−25.0% of the observed relative risk in the CKB cohort. The contribution of genetic risk exceeded modifiable exposures for several cancers, such as colorectal cancer (13.2% versus 2.9%), cervical cancer (3.1% versus 2.3%), and prostate cancer (20.3% versus 4.6%). Esophageal cancer (16.7% versus 2.6%), lung cancer (16.2% versus 2.7%), and ovarian cancer (13.0% versus 3.5%) were among the cancers where modifiable risk factors had a substantially greater impact than PRS (S23 Table).

## Discussion

Variations in prediction accuracy across diverse populations have been observed for PRSs [23]. Due to the distinct cancer spectrum in China compared to North American and European countries, PRSs derived from European populations may not be directly applicable to precision medicine in Chinese populations. In the present study, we proposed multiple strategies to construct PRSs and evaluate cancer risk in the Chinese population based on existing GWAS findings and PRS resources. We quantified individuals’ genetic predisposition to nine types of cancer and revealed cross-cancer associations of PRS. Additionally, we elucidated the relative contributions of identified genetic and modifiable risk factors to cancer risk stratification and prediction in the Chinese population.

The cross-ethnic application of PRS has long been a significant scientific concern. Our study partially addresses questions on whether selected established PRSs that perform well in European-based populations have equal utility in Asian populations. Consistent with previous studies, our findings indicated that PRSs primarily developed for European ancestry (6/12) were significantly associated with the risk of corresponding cancer in the Chinese population, particularly for pancreatic cancer [24]. It is plausible that the PRS derived from European-ancestry GWAS performs better due to the lack of effective published pancreatic cancer PRS for East Asian populations and the weak predictive performance of currently identified genetic loci in East Asians. Our study also observed that two-thirds of the optimal PRSs were derived from East Asian GWAS or incorporated replicated SNPs from other ethnic populations, symmetrically emphasizing that prediction accuracy is consistently higher when using GWAS data from ancestry-matched populations. Developing new methods for constructing cross-ethnic PRS is a current research focus [10,25]. In our study, we demonstrated the value of this approach, showing that PRSs generated using PRS-CSx outperformed other methods for predicting the risk of colorectal cancer and ovarian cancer. Due to the lack of sufficient high-quality GWAS for head and neck, liver, endometrial, and bladder cancers among East Asian populations, we were unable to construct effective PRS for these cancers, highlighting the necessity for further GWAS in the Chinese population. In addition, heritability varies across different cancers and plays a crucial role in shaping the performance of PRSs. Cancers with higher heritability estimates from twin studies, such as prostate, colorectal, breast, and pancreatic cancers, demonstrated better predictive performance in our PRS-only model (S10 Fig) [26,27]. The systematic construction protocol and robust findings in our study can provide empirical guidance for developing cancer PRS for other ethnic groups, especially those with insufficient GWAS studies, potentially addressing current disparities in PRS research.

Cross-cancer GWAS analyses have observed relatively strong genome-wide genetic correlations across cancers, although many correlations were moderate. Genetic regions including 5p15.33 (TERT) and 6p21-22 (HLA) also showed wide evidence of pairwise pleiotropy in multiple cancer combinations for individual variants [14,28]. In our study, besides individual variants, we identified nine pairs of cross-cancer associations of PRS. Notably, two pairs (colorectal cancer PRS and breast cancer risk; pancreatic cancer PRS and breast cancer risk) were validated in a recent study in European populations [14]. Given the low incidence of stomach cancer in European populations, our study demonstrated cross-cancer associations between stomach PRS and ovarian cancer risk, as well as colorectal cancer PRS and breast cancer PRS with stomach cancer risk. Although genetic correlation analyses in European populations also indicated interrelations among these cancer types [29], our findings provide novel insights into genetic risk assessment and its application across different cancer types in Chinese populations, particularly through incorporating significant cross-cancer PRSs in cancer risk models. However, we only assessed the extent to which PRSs themselves show evidence of association with multiple cancer types. Further work is needed to understand the mechanisms driving these observed associations.

In China, identifying individuals at high risk of cancer is a crucial public health priority for both primary and secondary prevention efforts. Demographic factors (e.g., age and sex) alone could achieve modest risk discrimination for most cancers [30]. Consistent with previous studies, expanding predictors to include modifiable risk factors and PRSs could enhance the predictive performance of risk models in the Chinese population [5]. Modifiable risk factors contribute significantly to predictive discrimination for ovarian, breast, lung, pancreatic, and esophageal cancers, which aligns with their strong associations with these cancer types [31]. Including PRSs substantially enhances predictive discrimination for colorectal and breast cancers, in line with the wealth of genetic data available for these cancers in East Asian populations [32,33]. Although our study revealed a strong association between PRS and prostate cancer, the improvement in prediction performance was much weaker than that in European populations, likely due to differences in baseline model performance between Chinese populations (C-index of 0.868) and European populations (C-index of 0.716) [34]. We also observed that genetic risk is more strongly associated with stomach cancer and cervical cancer patients with lower onset ages, which underscores the importance of early interventions targeting modifiable risk factors and screening may need to be implemented for those with high genetic risk at younger ages.

A healthy lifestyle has been inversely associated with cancer risk in Chinese populations [31]. Our study addressed the absolute risk trajectories of common cancers across different modifiable and genetic risk groups in Chinese populations. The distinctly different trajectory patterns of lung, breast, cervical, and prostate cancers compared to those in European populations indicate variations in etiology among different ethnic groups [5,35]. Our study also indicated that maintaining a healthy lifestyle could lower cancer risk across genetic risk groups for nine common cancers. Beyond predictive performance and risk stratification, our findings offer a comprehensive understanding of the relative importance of modifiable risk factors and genetic risk in Chinese populations, informing potential future prevention policies.

There are several potential limitations of this study. Firstly, due to the unavailability of genome-wide summary statistics from the original East Asian GWAS, we primarily used data from the BBJ for PRS-CSx. The number of cases in BBJ is generally smaller than the original GWAS, which may lead to an underestimation of the efficiency of the PRS-CSx. Secondly, although we observed several pairs of cross-cancer associations, it is important to note that the associations were weak and some might be false positives. Thirdly, the “optimal” PRSs selected in this study may be only superficially superior in terms of C-index over other PRSs. Fourthly, several key risk factors of cancer were not available in the CKB cohort, such as *Helicobacter pylori* and human papillomavirus, possibly leading to an underestimation of modifiable risk factors’ contribution in the Chinese population. Fifthly, the low incidence of certain cancer types, such as prostate, ovarian, and pancreatic cancers, limits our ability to fully demonstrate the relationship between some modifiable risk factors and cancer risk in this study. Lastly, we acknowledge that the sensitivity and specificity of our PRS were suboptimal, which could result in false positives when identifying high-risk populations. Moving forward, it will be essential to refine risk stratification tools to reduce false positives and enhance the accuracy of identifying individuals genuinely at high risk.

In conclusion, our study derived nine optimal PRSs out of 80, showing superior performance for nine common cancers in Chinese populations. We also revealed the potential value of cross-cancer risk prediction using PRSs among Chinese populations. The absolute risk projections and the ERR of established modifiable and genetic risk factors significantly advance our understanding of cancer etiology and prevention in the Chinese population.

## Supporting information

S1 Text Genotyping and imputation in CKB. (DOCX)

S2 Text Construction of polygenic risk scores. (DOCX)

S3 Text Assessment of risk factors. (DOCX)

S4 Text Members of the China Kadoorie Biobank collaborative group. (DOCX)

S1 Table Details of polygenic risk scores for each cancer from the Polygenic Score (PGS) catalog used in this study. (DOCX)

S2 Table Details of the genome-wide association studies (GWAS) summary statistics applied for PRS-CSx in this study. EUR, European population; EAS, East Asian population; NJMU, GWAS summary statistics from Nanjing Medical University.
(DOCX)

S3 Table Modifiable risk factors assessed in this study in addition to age, sex (if applicable), region, and family history of cancer. BMI, body mass index.
(DOCX)

S4 Table Incidence rate of 13 cancers in the CKB cohort. (DOCX)

S5 Table Association details of the developed polygenic risk scores for each cancer type and corresponding C-index estimated by Cox regression models. PRS, polygenic risk score; HR, hazard ratio; CI, confidence interval; SNP, single-nucleotide polymorphism.
(DOCX)

S6 Table Association details of the optimal polygenic risk scores for the nine cancers in the CKB cohort. PRS, polygenic risk score; HR, hazard ratio; CI, confidence interval.
(DOCX)

S7 Table Association details of the optimal polygenic risk scores for the nine cancers in the CKB cohort after excluding all participants within the first year after recruitment. PRS, polygenic risk score; HR, hazard ratio; CI, confidence interval.
(DOCX)

S8 Table Association details of the optimal polygenic risk scores for the nine cancers in the CKB cohort after only including the first primary cancer. PRS, polygenic risk score; HR, hazard ratio; CI, confidence interval.
(DOCX)

S9 Table The 10-year AUC, sensitivity, and specificity of the nine optimal polygenic risk scores. AUC, area under the curve; PRS, polygenic risk score; CI, confidence interval.
(DOCX)

S10 Table Associations of genetic risk with different age onsets of each cancer type in the CKB cohort. HR, hazard ratio; CI, confidence interval.
(DOCX)

S11 Table Proportion of participants at high genetic risk of site-specific cancers. (DOCX)

S12 Table Association details between cancer-specific polygenic risk scores and other cancer outcomes. PRS, polygenic risk score; HR, hazard ratio; CI, confidence interval.
(DOCX)

S13 Table Association details for the modifiable risk factors with each cancer type in the CKB cohort. HR, hazard ratio; CI, confidence interval; BMI, body mass index.
(DOCX)

S14 Table Association details for the risk groups defined by modifiable risk factors in the CKB cohort. HR, hazard ratio; CI, confidence interval.
(DOCX)

S15 Table Associations of summarized risk factors with different age onsets of each cancer type in the CKB cohort. HR, hazard ratio; CI, confidence interval.
(DOCX)

S16 Table Assessment of the combined effect of polygenic risk score groups and modifiable risk factor groups in the CKB cohort. PRS, polygenic risk score; RF, modifiable risk factor; HR, hazard ratio; CI, confidence interval.
(DOCX)

S17 Table Assessment of multiplicative interaction between polygenic risk score groups and modifiable risk factor groups in the CKB cohort. PRS, polygenic risk score; RF, modifiable risk factor; HR, hazard ratio; CI, confidence interval.
(DOCX)

S18 Table Assessment of model discrimination for each cancer after incorporating modifiable risk factors and polygenic risk scores in the CKB cohort. PRS, polygenic risk score; CI, confidence interval; AUC, area under the curve; NRI, net reclassification improvement.
(DOCX)

S19 Table Assessment of model discrimination for each cancer after incorporating cross-cancer polygenic risk scores in the CKB cohort. PRS, polygenic risk score; CI, confidence interval; AUC, area under the curve.
(DOCX)

S20 Table Assessment of model discrimination for each cancer comparing different combinations of modifiable risk factors and polygenic risk scores after excluding all participants within the first year after recruitment. PRS, polygenic risk score; CI, confidence interval; AUC, area under the curve; NRI, net reclassification improvement.
(DOCX)

S21 Table Assessment of model discrimination for each cancer comparing different combinations of modifiable risk factors and polygenic risk scores after only including the first primary cancer. PRS, polygenic risk score; CI, confidence interval; AUC, area under the curve; NRI, net reclassification improvement.
(DOCX)

S22 Table Different approaches to construct predictive models based on modifiable risk factors and polygenic risk scores. AUC, area under the curve; CI, confidence interval.
(DOCX)

S23 Table Explained relative risk for the polygenic risk scores and summarized risk factors for each cancer type in the CKB cohort. ERR, explained relative risk; PRS, polygenic risk score; CI, confidence interval.
(DOCX)

S1 Fig Distribution of the nine optimal polygenic risk scores for each cancer type in the CKB cohort. PRS, polygenic risk score; CKB, China Kadoorie Biobank.
(DOCX)

S2 Fig The association of polygenic risk scores with individual cancer in the CKB cohort. Participants in the CKB cohort were divided into five equal groups according to their polygenic risk scores, and the HRs for each group were compared with those in quintile 1 (HR 1.0 [ref]) of the polygenic risk score with the adjustment of age, sex (if applicable), region, and the top 10 principal components. The error bars represent 95% CIs and their centers represent the HRs. PRS, polygenic risk score; CKB, China Kadoorie Biobank; HR, hazard ratio; CI, confidence interval.
(DOCX)

S3 Fig The adjusted cumulative incidence curves across strata defined by polygenic risk score. Low polygenic risk score (PRS) corresponds to the bottom quintile, medium PRS is defined as quintile 2–4, and high PRS includes individuals in the top quintile in the CKB cohort. Cumulative incidence was estimated using Cox regression models with the adjustment of age, sex (if applicable), region, and the top 10 principal components. PRS, polygenic risk score; CKB, China Kadoorie Biobank.
(DOCX)

S4 Fig Correlation heatmap among the nine optimal cancer-specific polygenic risk scores. The significance levels in the figure are denoted by asterisks as follows: * *P*-value < 0.05, **P-value < 0.01, and ***P-value < 0.001. PRS, polygenic risk score.
(DOCX)

S5 Fig Hazard ratios between cancer-specific polygenic risk scores and other cancer outcomes. HRs were estimated using a Cox regression model adjusted for age, sex (if applicable), region, and the top 10 principal components (left) and they were further adjusted for the corresponding site-specific PRSs (right). The significance levels in the figure are denoted by asterisks as follows: * *P*-value < 0.05, **P-value < 0.01, and ***P-value < 0.001. PRS, polygenic risk score; HR, hazard ratio.
(DOCX)

S6 Fig Hazard ratios for the significant cross-cancer associations after excluding shared SNPs or SNPs in high linkage disequilibrium (*r* ^2^ > 0.6) with those in the polygenic risk score of outcome cancer type. HRs were estimated using a Cox regression model adjusted for age, sex (if applicable), region, and the top 10 principal components. The adjusted HRs were further adjusted for the corresponding site-specific PRSs. No shared SNPs were found for stomach cancer PRS and ovarian cancer PRS, lung cancer PRS and cervical cancer PRS, and breast cancer PRS and stomach cancer PRS. The error bars represent 95% CIs and their centers represent the HRs. SNP, single-nucleotide polymorphism; PRS, polygenic risk score; HR, hazard ratio; CI, confidence interval.
(DOCX)

S7 Fig Cumulative risk by age 80 of the nine cancers across strata defined by polygenic risk scores and modifiable risk factors. Low PRS corresponds to the bottom quintile, medium PRS is defined as quintile 2–4, and high PRS includes individuals in the top quintile in the CKB cohort. Individuals above the median of risk factors risk score distribution were considered to have an elevated risk profile, whereas those below the median had reduced risk. Death from any cause was treated as a competing event. RF, modifiable risk factors; PRS, polygenic risk score.
(DOCX)

S8 Fig Boxplots of the 10-year absolute risk across strata defined by polygenic risk scores, modifiable risk factors and age categories. Low PRS corresponds to the bottom quintile, medium PRS is defined as quintile 2–4, and high PRS includes individuals in the top quintile in the CKB cohort. Individuals above the median of risk factors risk score distribution were considered to have an elevated risk profile, whereas those below the median had reduced risk. The box limits represent interquartile ranges (IQRs) and their centers represent the medians of the absolute risk. The boundaries of the whiskers are based on the 1.5 * IQR value and other observed points outside the boundary of the whiskers are plotted as outliers. RF, modifiable risk factors; PRS, polygenic risk score; CKB, China Kadoorie Biobank; IQR, interquartile range.
(DOCX)

S9 Fig Ten-year absolute risk reduction across strata defined by genetic risk and modifiable risk factors. Low PRS corresponds to the bottom quintile, medium PRS is defined as quintile 2–4, and high PRS includes individuals in the top quintile in the CKB cohort. Individuals above the median of risk factors risk score distribution were considered to have an elevated risk profile, whereas those below the median had reduced risk. The error bars represent interquartile ranges and their centers represent the medians of the absolute risk. RF, modifiable risk factors; PRS, polygenic risk score; ARR, absolute risk reduction; CKB, China Kadoorie Biobank.
(DOCX)

S10 Fig The correlation between the C-index and the heritability estimates. The C-index was from the PRS-only model of each cancer. The heritability estimates come from previous articles, mainly from two twin studies.
(DOCX)

S1 STREGA Checklist Reporting checklist of items for genetic association study based on the STREGA guidelines. (DOCX)

S1 Data All weights, including comprehensive lists of sources of nine optimal PRSs. (PDF)

## Abbreviations

ARR: absolute risk reduction
AUC: area under the curve
BBJ: BioBank Japan Project
CI: confidence interval
CKB: China Kadoorie Biobank
ERR: explained relative risk
GWASs: genome-wide association studies
HRs: hazard ratios
IRB: Institutional Review Board
NRI: net reclassification index
PGS: polygenic score
PRSs: polygenic risk scores
SNPs: single-nucleotide polymorphisms
STREGA: Strengthening the Reporting of Genetic Association Studies

## References

[1] Sung H, Ferlay J, Siegel RL, Laversanne M, Soerjomataram I, Jemal A, et al. Global cancer statistics 2020: GLOBOCAN estimates of incidence and mortality worldwide for 36 cancers in 185 countries. CA Cancer J Clin. 2021;71(3):209–49. doi: 10.3322/caac.21660 33538338

[2] Sud A, Kinnersley B, Houlston RS. Genome-wide association studies of cancer: current insights and future perspectives. Nat Rev Cancer. 2017;17(11):692–704. doi: 10.1038/nrc.2017.82 29026206

[3] Popejoy AB, Fullerton SM. Genomics is failing on diversity. Nature. 2016;538(7624):161–4. doi: 10.1038/538161a 27734877 PMC5089703

[4] Zhu M, Wang T, Huang Y, Zhao X, Ding Y, Zhu M, et al. Genetic risk for overall cancer and the benefit of adherence to a healthy lifestyle. Cancer Res. 2021;81(17):4618–27. doi: 10.1158/0008-5472.CAN-21-0836 34321244

[5] Kachuri L, Graff RE, Smith-Byrne K, Meyers TJ, Rashkin SR, Ziv E, et al. Pan-cancer analysis demonstrates that integrating polygenic risk scores with modifiable risk factors improves risk prediction. Nat Commun. 2020;11(1):6084. doi: 10.1038/s41467-020-19600-4 33247094 PMC7695829

[6] Khera AV, Chaffin M, Aragam KG, Haas ME, Roselli C, Choi SH, et al. Genome-wide polygenic scores for common diseases identify individuals with risk equivalent to monogenic mutations. Nat Genet. 2018;50(9):1219–24. doi: 10.1038/s41588-018-0183-z 30104762 PMC6128408

[7] Lambert SA, Gil L, Jupp S, Ritchie SC, Xu Y, Buniello A, et al. The polygenic score catalog as an open database for reproducibility and systematic evaluation. Nat Genet. 2021;53(4):420–5. doi: 10.1038/s41588-021-00783-5 33692568 PMC11165303

[8] Martin AR, Kanai M, Kamatani Y, Okada Y, Neale BM, Daly MJ. Clinical use of current polygenic risk scores may exacerbate health disparities. Nat Genet. 2019;51(4):584–91. doi: 10.1038/s41588-019-0379-x 30926966 PMC6563838

[9] Duncan L, Shen H, Gelaye B, Meijsen J, Ressler K, Feldman M, et al. Analysis of polygenic risk score usage and performance in diverse human populations. Nat Commun. 2019;10(1):3328. doi: 10.1038/s41467-019-11112-0 . 31346163 PMC6658471

[10] Ruan Y, Lin Y-F, Feng Y-CA, Chen C-Y, Lam M, Guo Z, et al. Improving polygenic prediction in ancestrally diverse populations. Nat Genet. 2022;54(5):573–80. doi: 10.1038/s41588-022-01054-7 35513724 PMC9117455

[11] Jin G, Lv J, Yang M, Wang M, Zhu M, Wang T, et al. Genetic risk, incident gastric cancer, and healthy lifestyle: a meta-analysis of genome-wide association studies and prospective cohort study. Lancet Oncol. 2020;21(10):1378–86. doi: 10.1016/S1470-2045(20)30460-5 33002439

[12] Dai J, Lv J, Zhu M, Wang Y, Qin N, Ma H, et al. Identification of risk loci and a polygenic risk score for lung cancer: a large-scale prospective cohort study in Chinese populations. Lancet Respir Med. 2019;7(10):881–91. doi: 10.1016/S2213-2600(19)30144-4 31326317 PMC7015703

[13] Zhu M, Lv J, Huang Y, Ma H, Li N, Wei X, et al. Ethnic differences of genetic risk and smoking in lung cancer: two prospective cohort studies. Int J Epidemiol. 2023;52(6):1815–25. doi: 10.1093/ije/dyad118 37676847

[14] Graff RE, Cavazos TB, Thai KK, Kachuri L, Rashkin SR, Hoffman JD, et al. Cross-cancer evaluation of polygenic risk scores for 16 cancer types in two large cohorts. Nat Commun. 2021;12(1):970. doi: 10.1038/s41467-021-21288-z 33579919 PMC7880989

[15] Chen Z, Lee L, Chen J, Collins R, Wu F, Guo Y, et al. Cohort profile: the Kadoorie Study of Chronic Disease in China (KSCDC). Int J Epidemiol. 2005;34(6):1243–9. doi: 10.1093/ije/dyi174 16131516

[16] Chen Z, Chen J, Collins R, Guo Y, Peto R, Wu F, et al. China Kadoorie Biobank of 0.5 million people: survey methods, baseline characteristics and long-term follow-up. Int J Epidemiol. 2011;40(6):1652–66. doi: 10.1093/ije/dyr120 22158673 PMC3235021

[17] Walters RG, Millwood IY, Lin K, Schmidt Valle D, McDonnell P, Hacker A, et al. Genotyping and population characteristics of the China Kadoorie Biobank. Cell Genom. 2023;3(8):100361. doi: 10.1016/j.xgen.2023.100361 37601966 PMC10435379

[18] Little J, Higgins JPT, Ioannidis JPA, Moher D, Gagnon F, von Elm E, et al. STrengthening the REporting of Genetic Association Studies (STREGA): an extension of the STROBE statement. PLoS Med. 2009;6(2):e22. doi: 10.1371/journal.pmed.1000022 19192942 PMC2634792

[19] Sakaue S, Kanai M, Tanigawa Y, Karjalainen J, Kurki M, Koshiba S, et al. A cross-population atlas of genetic associations for 220 human phenotypes. Nat Genet. 2021;53(10):1415–24. doi: 10.1038/s41588-021-00931-x 34594039 PMC12208603

[20] Zahed H, Feng X, Sheikh M, Bray F, Ferlay J, Ginsburg O, et al. Age at diagnosis for lung, colon, breast and prostate cancers: an international comparative study. Int J Cancer. 2024;154(1):28–40. doi: 10.1002/ijc.34671 37615573 PMC11153845

[21] Pencina MJ, D’Agostino RB Sr, Steyerberg EW. Extensions of net reclassification improvement calculations to measure usefulness of new biomarkers. Stat Med. 2011;30(1):11–21. doi: 10.1002/sim.4085 21204120 PMC3341973

[22] Heller G. A measure of explained risk in the proportional hazards model. Biostatistics. 2012;13(2):315–25. doi: 10.1093/biostatistics/kxr047 22190711 PMC3297826

[23] Kachuri L, Chatterjee N, Hirbo J, Schaid DJ, Martin I, Kullo IJ, et al. Principles and methods for transferring polygenic risk scores across global populations. Nat Rev Genet. 2024;25(1):8–25. doi: 10.1038/s41576-023-00637-2 37620596 PMC10961971

[24] Ho PJ, Tan IB, Chong DQ, Khor CC, Yuan J-M, Koh W-P, et al. Polygenic risk scores for the prediction of common cancers in East Asians: a population-based prospective cohort study. Elife. 2023;12:e82608. doi: 10.7554/eLife.82608 36971353 PMC10159619

[25] Hoggart CJ, Choi SW, García-González J, Souaiaia T, Preuss M, O’Reilly PF. BridgePRS leverages shared genetic effects across ancestries to increase polygenic risk score portability. Nat Genet. 2024;56(1):180–6. doi: 10.1038/s41588-023-01583-9 38123642 PMC10786716

[26] Lichtenstein P, Holm NV, Verkasalo PK, Iliadou A, Kaprio J, Koskenvuo M, et al. Environmental and heritable factors in the causation of cancer—analyses of cohorts of twins from Sweden, Denmark, and Finland. N Engl J Med. 2000;343(2):78–85. doi: 10.1056/NEJM200007133430201 10891514

[27] Mucci LA, Hjelmborg JB, Harris JR, Czene K, Havelick DJ, Scheike T, et al. Familial risk and heritability of cancer among twins in Nordic countries. JAMA. 2016;315(1):68–76. doi: 10.1001/jama.2015.17703 26746459 PMC5498110

[28] Lindström S, Wang L, Feng H, Majumdar A, Huo S, Macdonald J, et al. Genome-wide analyses characterize shared heritability among cancers and identify novel cancer susceptibility regions. J Natl Cancer Inst. 2023;115(6):712–32. doi: 10.1093/jnci/djad043 36929942 PMC10248849

[29] Rashkin SR, Graff RE, Kachuri L, Thai KK, Alexeeff SE, Blatchins MA, et al. Pan-cancer study detects genetic risk variants and shared genetic basis in two large cohorts. Nat Commun. 2020;11(1):4423. doi: 10.1038/s41467-020-18246-6 32887889 PMC7473862

[30] Laconi E, Marongiu F, DeGregori J. Cancer as a disease of old age: changing mutational and microenvironmental landscapes. Br J Cancer. 2020;122(7):943–52. doi: 10.1038/s41416-019-0721-1 32042067 PMC7109142

[31] Chen W, Xia C, Zheng R, Zhou M, Lin C, Zeng H, et al. Disparities by province, age, and sex in site-specific cancer burden attributable to 23 potentially modifiable risk factors in China: a comparative risk assessment. Lancet Glob Health. 2019;7(2):e257–69. doi: 10.1016/S2214-109X(18)30488-1 30683243

[32] Shu X, Long J, Cai Q, Kweon S-S, Choi J-Y, Kubo M, et al. Identification of novel breast cancer susceptibility loci in meta-analyses conducted among Asian and European descendants. Nat Commun. 2020;11(1):1217. doi: 10.1038/s41467-020-15046-w 32139696 PMC7057957

[33] Fernandez-Rozadilla C, Timofeeva M, Chen Z, Law P, Thomas M, Schmit S, et al. Deciphering colorectal cancer genetics through multi-omic analysis of 100,204 cases and 154,587 controls of European and east Asian ancestries. Nat Genet. 2023;55(1):89–99. doi: 10.1038/s41588-022-01222-9 36539618 PMC10094749

[34] Nyberg T, Brook MN, Ficorella L, Lee A, Dennis J, Yang X, et al. CanRisk-prostate: a comprehensive, externally validated risk model for the prediction of future prostate cancer. J Clin Oncol. 2023;41(5):1092–104. doi: 10.1200/JCO.22.01453 36493335 PMC9928632

[35] Plym A, Zhang Y, Stopsack KH, Delcoigne B, Wiklund F, Haiman C, et al. A healthy lifestyle in men at increased genetic risk for prostate cancer. Eur Urol. 2023;83(4):343–51. doi: 10.1016/j.eururo.2022.05.008 35637041 PMC10279925

