# Supplementary material for: Polygenic risk scores for pan-cancer risk prediction in the Chinese population: A population-based cohort study based on the China Kadoorie Biobank
Source: PLoS Med. 2025 Feb 28;22(2):e1004534. doi: 10.1371/journal.pmed.1004534 (PMC11870365; doi:10.1371/journal.pmed.1004534)
Supplement: S1 Text — (DOCX) [file pmed.1004534.s001.docx]

**S1 Text. Genotyping and imputation in CKB**

Details of genotyping, quality control, and imputation for the CKB cohort had been reported previously [1]. Briefly, participants in CKB were genotyped using a custom-designed Affymetrix Axiom® CKB array (optimized for usage of Han Chinese subjects), which consists of approximately 800,000 markers. After a series of quality control produces (exclude SNPs with call rate <0.98, plate effect *P*<10^-6^, batch effect *P*<10^-6^, Hardy-Weinberg equilibrium (HWE) deviations *P*<10^-6^ and minor allele frequency (MAF) difference from 1000 Genomes East Asian frequencies >0.2), the qualified genotype data were phased with SHAPEIT3 (autosomes) and SHAPEIT2 (chromosome X) and then imputed with IMPUTE4 (autosomes) and IMPUTE2 (chromosome X) with default parameters by using the 1000 Genomes phase3 as the reference. PCA was conducted using FlashPCA v2.1 after LD pruning and exclusion of regions of long-range LD which, if not excluded or otherwise accounted for, can interfere with PCA potentially leading to erroneous conclusions about population structure, or erroneous genetic association signals.

**References**

1. Walters RG, Millwood IY, Lin K, Schmidt Valle D, McDonnell P, Hacker A, et al. Genotyping and population characteristics of the China Kadoorie Biobank. Cell Genom. 2023;3(8):100361. doi: 10.1016/j.xgen.2023.100361. PMID: 37601966.
