# Supplementary material for: Polygenic risk scores for pan-cancer risk prediction in the Chinese population: A population-based cohort study based on the China Kadoorie Biobank
Source: PLoS Med. 2025 Feb 28;22(2):e1004534. doi: 10.1371/journal.pmed.1004534 (PMC11870365; doi:10.1371/journal.pmed.1004534)
Supplement: S2 Text — (DOCX) [file pmed.1004534.s002.docx]

**S2 Text. Construction of polygenic risk scores**

In this study, we developed 80 PRSs using seven different solutions as described in detail below and summarized in **Fig 1**.

**Strategy 1: PRSs using GWAS-identified risk SNPs (4 scores)**

For individual cancer types, we systematically searched GWASs of each cancer in populations of East Asian ancestry published before May 1, 2022, in PubMed, supplemented by searching the NHGRI-EBI GWAS Catalog [1]. For each eligible GWAS conducted in East Asians with the largest sample size, we reviewed the original manuscript and supplemental materials and extracted variants showing a significant association with cancer risk at *P*<5×10^-8^. Those variants that were newly identified with *P*<5×10^-8^ in other ethnic populations and replicated in a consistent association direction with *P*<0.05 by the largest GWASs in East Asians were kept for further analysis. Besides, the BioBank Japan Project (BBJ), which is a biobank that recruited approximately 200,000 patients with a diagnosis of at least one of 47 diseases in Japan, was also used in our study [2]. Through various combinations, we constructed four scores for each cancer type: (1) PRS1: genetic variants reported in East Asian GWAS at *P*<5×10^-8^; (2) PRS2: adding variants reported in BBJ GWAS at *P*<5×10^-8^ to PRS1; (3) PRS3: variants meeting *P*<5×10^-8^ in other ethnic populations and replicated with the significant associations (*P*<0.05) in East Asian GWAS; (4) PRS4: further adding variants meeting *P*<5×10^-8^ in other ethnic populations and replicated with the significant associations (*P*<0.05) in BBJ GWAS to all variants above. The corresponding effect sizes (i.e., OR, β) were extracted from the largest studies accordingly. For duplicated variants, we kept the published effect sizes from larger GWAS in terms of sample sizes. If multiple variants are in linkage disequilibrium (LD) with R^2^>0.2 in East Asians (1000 Genomes, Phase 3 v5), the variants with the smallest reported *P*-value were selected by using the linkage disequilibrium clumping procedure in PLINK [3].

**Strategy 2: Published PRSs from the PGS Catalog (2 scores)**

We retrieved published PRSs from the PGS Catalog, an open database of polygenic scores (retrieved on 4 August 2022) [4]. The parameters of PRS were extracted from the PGS Catalog constructed mainly for European (PRS5) or East Asian populations (PRS6), opting for studies with larger sample sizes. To note, the source of variant associations of PRS5 was not 100% European ancestry, and the source of variant associations of PRS6 was not 100% East Asian ancestry. For several cancers, both PRS5 and PRS6 were multi-ancestry scores with PRS5 having a higher proportion of European ancestry populations. Only PRSs with odds ratios or log odds ratios as weights were included. PRSs that used odds ratio over expected risk, inverse-variance weighting, and unweighted were excluded. **S1** **Table** shows details of polygenic risk scores for each cancer eligible on the Polygenic Score (PGS) catalog.

**Strategy 3: PRSs based on trans-ancestry construction algorithms (1 score)**

We used a recently proposed Bayesian polygenic modeling method, PRS-CSx, based on available GWAS summary statistics from East Asian and European populations [5]. PRS-CSx has been demonstrated to be useful for constructing trans-ancestry PRS and improving cross-population polygenic prediction. We curated the latest and largest sample size GWAS summary statistics data available for 13 types of cancer from the GWAS Catalog and previous literature. All accessible summary statistics data were separately compiled for European and East Asian populations. For each GWAS, the variants located within autosomal chromosomes meeting the thresholds of MAF≥0.01, INFO≥0.3, and *P*<0.05 were kept from the GWAS summary statistics data. Then, we utilized the PRS-CSx Python software to concurrently analyze GWAS summary statistics in the HapMap3 panel across two populations, employing a common continuous shrinkage prior in order to obtain more accurate effect size estimation. The analysis involved the utilization of pre-computed reference panels from the 1000 Genomes Project and a fully Bayesian algorithm for model fitting, allowing automatic learning of all model parameters without requiring hyperparameter tuning. We used 1000 Genomes EUR and EAS samples as the LD reference panel for European and East Asian populations’ summary statistics, respectively. Subsequently, population-specific posterior effect size estimates were combined using an inverse-variance-weighted meta-analysis within the Gibbs sampler (--meta). With the default parameters, we generated PRSs for the 13 cancer types by using shared variants in the two summary statistics (PRS7). **S2** **Table** details the GWAS summary statistics we applied for analysis.

The PRSs were generated for each cancer site using an additive model, which involved summing the dosage of each risk allele for each individual and then multiplying it by the respective effect size (log odds ratio, β) specific to each cancer type. We utilized PLINK1.9 to calculate each PRS as a weighted sum, using the --score sum function with default parameters. All weights, including comprehensive lists of sources, are provided in **S1 Data**.

**References**

1. Sollis E, Mosaku A, Abid A, Buniello A, Cerezo M, Gil L, et al. The NHGRI-EBI GWAS Catalog: knowledgebase and deposition resource. Nucleic Acids Res. 2023;51(D1):D977-D85. doi: 10.1093/nar/gkac1010. PMID: 36350656.

2. Sakaue S, Kanai M, Tanigawa Y, Karjalainen J, Kurki M, Koshiba S, et al. A cross-population atlas of genetic associations for 220 human phenotypes. Nat Genet. 2021;53(10):1415-24. doi: 10.1038/s41588-021-00931-x. PMID: 34594039.

3. Chang CC, Chow CC, Tellier LC, Vattikuti S, Purcell SM, Lee JJ. Second-generation PLINK: rising to the challenge of larger and richer datasets. Gigascience. 2015;4:7. doi: 10.1186/s13742-015-0047-8. PMID: 25722852.

4. Lambert SA, Gil L, Jupp S, Ritchie SC, Xu Y, Buniello A, et al. The Polygenic Score Catalog as an open database for reproducibility and systematic evaluation. Nat Genet. 2021;53(4):420-5. doi: 10.1038/s41588-021-00783-5. PMID: 33692568.

5. Ruan Y, Lin Y-F, Feng Y-CA, Chen C-Y, Lam M, Guo Z, et al. Improving polygenic prediction in ancestrally diverse populations. Nat Genet. 2022;54(5):573-80. doi: 10.1038/s41588-022-01054-7. PMID: 35513724.
