# Supplementary material for: Polygenic risk scores for pan-cancer risk prediction in the Chinese population: A population-based cohort study based on the China Kadoorie Biobank
Source: PLoS Med. 2025 Feb 28;22(2):e1004534. doi: 10.1371/journal.pmed.1004534 (PMC11870365; doi:10.1371/journal.pmed.1004534)
Supplement: S3 Text — (DOCX) [file pmed.1004534.s003.docx]

**S3 Text. Assessment of risk factors**

Risk factors as well as age, sex, and region were all collected from the baseline questionnaire of the CKB study. Education level was classified into four levels: primary school/no formal school, middle school, high school, college or up. Physical measurements included weight and height. BMI was calculated as weight in kilograms divided by the square of height in meters (kg/m^2^) and categorized based on the criteria of Chinese population [1].

During the baseline questionnaire, questions about tobacco smoking included how often the participants had smoked tobacco at the survey time. Smoking status was classified as never (smoked <100 cigarettes during lifetime), occasional, former, or regular smoker. Former smokers who had stopped smoking due to physical illness were still counted as smokers in the main analyses. Information on the duration (years) of smoking and cigarettes smoked per day for regular smokers and former smokers was also collected. We calculated pack-years as a measure of the cumulative burden of smoking by the product of the years of smoking (excluding years of quitting smoking) and the number of cigarette packs (the number of cigarettes divided by 20) smoked per day. Questions about alcohol consumption included typical drinking frequency in the past 12 months and drinking habits before. Drinking status was classified as never or occasional drinkers and regular drinkers or abstainers. For physical activity, the usual type and duration of occupational, commuting, domestic, and leisure time-related activities in the past 12 months were collected. To calculate the daily total physical activity level, we multiplied the metabolic equivalent of tasks (METs) for each activity by the hour spent on that activity and summed the MET-hours for all activities [2]. Respondents were also asked about the frequency of habitual dietary consumption, including fresh vegetables, fresh fruits, salty vegetables, and meat, during the previous 12 months and five categories of frequency (daily, 4-6 days per week, 1-3 days per week, monthly, or never or rarely) can be chosen.

For medical conditions, personal diagnoses of cancer, peptic ulcer, gallstone or gallbladder, cirrhosis or chronic hepatitis, emphysema or bronchitis, and frequent cough were all self-reported. Prevalent diabetes was defined as self-reported diabetes or screen-detected diabetes. The screen-detected diabetes was defined as measured fasting blood glucose >7.0 mmol/L or random blood glucose >11.1 mmol/L at baseline. The first-degree family members’ cancer history including fathers, mothers, and siblings had been collected for each participant. Those who reported at least one first-degree relative had cancer were classified as having a family history of cancer. For women, Information on age at menopause, number of pregnancies, and menopausal status was collected through questionnaires at baseline. One individual had missing values for BMI, while three individuals had missing values for age at menarche. Imputation was performed using the mean values.

**References**

1. Zhou B-F. Predictive values of body mass index and waist circumference for risk factors of certain related diseases in Chinese adults--study on optimal cut-off points of body mass index and waist circumference in Chinese adults. Biomed Environ Sci. 2002;15(1):83-96. PMID: 12046553.

2. Du H, Bennett D, Li L, Whitlock G, Guo Y, Collins R, et al. Physical activity and sedentary leisure time and their associations with BMI, waist circumference, and percentage body fat in 0.5 million adults: the China Kadoorie Biobank study. Am J Clin Nutr. 2013;97(3):487-96. doi: 10.3945/ajcn.112.046854. PMID: 23364014.
