# Supplementary material for: Polygenic risk scores for pan-cancer risk prediction in the Chinese population: A population-based cohort study based on the China Kadoorie Biobank
Source: PLoS Med. 2025 Feb 28;22(2):e1004534. doi: 10.1371/journal.pmed.1004534 (PMC11870365; doi:10.1371/journal.pmed.1004534)
Supplement: S1 Table — (DOCX) [file pmed.1004534.s005.docx]

**S1 Table. Details of polygenic risk scores for each cancer from the Polygenic Score (PGS) catalog used in this study**

| **PGS ID** | **Name** | **Reported Trait** | **Number of variants** | **Ancestry distribution (%)** | **Reference** |
| --- | --- | --- | --- | --- | --- |
| PGS000081 | CC_Oral | Oral cavity and pharyngeal cancers | 14 | European: 95.6%, Not Reported: 4.4% | Graff RE, et al. [1] |
| PGS002298 | PRS14_esophageal | Esophageal cancer | 14 | European: 100% | Choi J, et al. [2] |
| PGS002299 | PRS3_gastric | Gastric cancer | 3 | European: 100% | Choi J, et al. [2] |
| PGS000765 | PRS_CRC95 | Colorectal cancer | 95 | European: 95.8%, East Asian: 4.2% | Huyghe JR, et al. [3] |
| PGS002742 | PRS115_EAS | Colorectal cancer | 115 | European: 61.9%, East Asian: 36.5%, African: 1.3%, Additional Diverse Ancestries: 0.2% | Ping J, et al. [4] |
| PGS000663 | wGRS22 | Pancreatic cancer | 22 | European: 86.6%, Not Reported: 12.3%, Multi-ancestry (excluding European): 1.1% | Kim J, et al. [5] |
| PGS002270 | PRS33_LC | Lung cancer | 33 | European: 79.9%, East Asian: 20.1% | Dai J, et al. [6] |
| PGS000070 | PRS_LC_C | Lung cancer | 19 | European: 51.1%, East Asian: 48.9% | Dai J, et al. [6] |
| PGS000004 | PRS313_BC | Breast Cancer | 313 | European: 100% | Mavaddat N, et al. [7] |
| PGS002294 | PRS111 | Breast cancer | 111 | European: 88.3%, East Asian: 11.7% | Yang Y, et al. [8] |
| PGS001299 | GBE_cancer1041 | Cervical cancer | 24 | European: 100% | Tanigawa Y, et al. [9] |
| PGS000075 | CC_Endo | Endometrial cancer | 9 | European: 66.4%, Not Reported: 33.6% | Graff RE, et al. [1] |
| PGS000351 | PRS_EOC | Invasive epithelial ovarian cancer | 30 | European: 100% | Barnes DR, et al. [10] |
| PGS000662 | GRS.PCa.269 | Prostate Cancer | 269 | European: 75.8%, East Asian: 11.7%, Multi-ancestry (excluding European): 9.1%, Hispanic or Latin American: 3.4% | Conti DV, et al. [11] |
| PGS002268 | PRS_csPCa | Prostate cancer | 29 | East Asian: 100% | Song SH, et al. [12] |
| PGS000071 | CC_Bladder | Bladder cancer | 15 | European: 100% | Graff RE, et al. [1] |
| - | PRS112 | Gastric cancer | 112 | East Asian: 100% | Jin G, et al. [13] |

**References**

1. Graff RE, Cavazos TB, Thai KK, Kachuri L, Rashkin SR, Hoffman JD, et al. Cross-cancer evaluation of polygenic risk scores for 16 cancer types in two large cohorts. Nat Commun. 2021;12(1):970. doi: 10.1038/s41467-021-21288-z. PMID: 33579919.

2. Choi J, Jia G, Wen W, Long J, Zheng W. Evaluating polygenic risk scores in assessing risk of nine solid and hematologic cancers in European descendants. Int J Cancer. 2020;147(12):3416-23. doi: 10.1002/ijc.33176. PMID: 32588423.

3. Huyghe JR, Bien SA, Harrison TA, Kang HM, Chen S, Schmit SL, et al. Discovery of common and rare genetic risk variants for colorectal cancer. Nat Genet. 2019;51(1):76-87. doi: 10.1038/s41588-018-0286-6. PMID: 30510241.

4. Ping J, Yang Y, Wen W, Kweon S-S, Matsuda K, Jia W-H, et al. Developing and validating polygenic risk scores for colorectal cancer risk prediction in East Asians. Int J Cancer. 2022;151(10):1726-36. doi: 10.1002/ijc.34194. PMID: 35765848.

5. Kim J, Yuan C, Babic A, Bao Y, Clish CB, Pollak MN, et al. Genetic and Circulating Biomarker Data Improve Risk Prediction for Pancreatic Cancer in the General Population. Cancer Epidemiol Biomarkers Prev. 2020;29(5). doi: 10.1158/1055-9965.EPI-19-1389. PMID: 32321713.

6. Dai J, Lv J, Zhu M, Wang Y, Qin N, Ma H, et al. Identification of risk loci and a polygenic risk score for lung cancer: a large-scale prospective cohort study in Chinese populations. Lancet Respir Med. 2019;7(10):881-91. doi: 10.1016/S2213-2600(19)30144-4. PMID: 31326317.

7. Mavaddat N, Michailidou K, Dennis J, Lush M, Fachal L, Lee A, et al. Polygenic Risk Scores for Prediction of Breast Cancer and Breast Cancer Subtypes. Am J Hum Genet. 2019;104(1):21-34. doi: 10.1016/j.ajhg.2018.11.002. PMID: 30554720.

8. Yang Y, Tao R, Shu X, Cai Q, Wen W, Gu K, et al. Incorporating Polygenic Risk Scores and Nongenetic Risk Factors for Breast Cancer Risk Prediction Among Asian Women. JAMA Netw Open. 2022;5(3):e2149030. doi: 10.1001/jamanetworkopen.2021.49030. PMID: 35311964.

9. Tanigawa Y, Qian J, Venkataraman G, Justesen JM, Li R, Tibshirani R, et al. Significant sparse polygenic risk scores across 813 traits in UK Biobank. PLoS Genet. 2022;18(3):e1010105. doi: 10.1371/journal.pgen.1010105. PMID: 35324888.

10. Barnes DR, Rookus MA, McGuffog L, Leslie G, Mooij TM, Dennis J, et al. Polygenic risk scores and breast and epithelial ovarian cancer risks for carriers of BRCA1 and BRCA2 pathogenic variants. Genet Med. 2020;22(10):1653-66. doi: 10.1038/s41436-020-0862-x. PMID: 32665703.

11. Conti DV, Darst BF, Moss LC, Saunders EJ, Sheng X, Chou A, et al. Trans-ancestry genome-wide association meta-analysis of prostate cancer identifies new susceptibility loci and informs genetic risk prediction. Nat Genet. 2021;53(1):65-75. doi: 10.1038/s41588-020-00748-0. PMID: 33398198.

12. Song SH, Kim E, Woo E, Kwon E, Yoon S, Kim JK, et al. Prediction of clinically significant prostate cancer using polygenic risk models in Asians. Investig Clin Urol. 2022;63(1):42-52. doi: 10.4111/icu.20210305. PMID: 34983122.

13. Jin G, Lv J, Yang M, Wang M, Zhu M, Wang T, et al. Genetic risk, incident gastric cancer, and healthy lifestyle: a meta-analysis of genome-wide association studies and prospective cohort study. Lancet Oncol. 2020;21(10):1378-86. doi: 10.1016/S1470-2045(20)30460-5. PMID: 33002439.
