# Supplementary material for: Polygenic risk scores for pan-cancer risk prediction in the Chinese population: A population-based cohort study based on the China Kadoorie Biobank
Source: PLoS Med. 2025 Feb 28;22(2):e1004534. doi: 10.1371/journal.pmed.1004534 (PMC11870365; doi:10.1371/journal.pmed.1004534)
Supplement: S2 Table — EUR, European population; EAS, East Asian population; NJMU, GWAS summary statistics from Nanjing Medical University. (DOCX) [file pmed.1004534.s006.docx]

**S2 Table. Details of the GWAS summary statistics applied for PRS-CSx in this study**

| **Cancer site** | **Ancestry** | **Number of cases** | **Number of controls** | **Number of SNPs** | **Reference** | **Link** |
| --- | --- | --- | --- | --- | --- | --- |
| Head and neck | EUR | 2,497 | 2,928 | 7,296,579 | Lesseur C, et al. [1] | http://ftp.ebi.ac.uk/pub/databases/gwas/summary_statistics/GCST012001-GCST013000/GCST012235/ |
|  | EAS | 300 | 178,426 | 13,436,083 | Sakaue S, et al. [2] | <https://pheweb.jp/downloads> |
| Esophagus | EUR | 4,112 | 17,159 | 13,031,593 | Gharahkhani P, et al. [3] | http://ftp.ebi.ac.uk/pub/databases/gwas/summary_statistics/GCST003001-GCST004000/GCST003739/ |
|  | EAS | 1,388 | 159,201 | 13,433,288 | Sakaue S, et al. [2] | <https://pheweb.jp/downloads> |
| Stomach | EUR | 1,029 | 475,087 | 18,907,370 | Sakaue S, et al. [2] | https://pheweb.jp/downloads |
|  | EAS | 7,921 | 159,201 | 13,434,351 | Sakaue S, et al. [2] | <https://pheweb.jp/downloads> |
| Colorectum | EUR and EAS | 100,204 (78,473 EUR and 21,731 EAS) | 154,587 (107,143 EUR and 47,444 EAS) | 22,203,197 | Fernandez-Rozadilla C, et al. [4] | http://ftp.ebi.ac.uk/pub/databases/gwas/summary_statistics/GCST90129001-GCST90130000/GCST90129505/ |
|  | EAS | 8,305 | 159,386 | 13,434,399 | Sakaue S, et al. [2] | <https://pheweb.jp/downloads> |
| Liver | EAS | 2,122 | 159,201 | 13,433,487 | Sakaue S, et al. [2] | <https://pheweb.jp/downloads> |
|  | EAS | 1,538 | 1,465 | 7,447,250 | Han J, et al. [5] | NJMU |
| Pancreas | EUR | 3,851 | 3,934 | 543,436 | Petersen GM, et al. [6] | https://ftp.ncbi.nlm.nih.gov/dbgap/studies/phs000206/analyses/ |
|  | EAS | 2,039 | 32,592 | 7,914,378 | Lin Y, et al. [7] | [http://www.aichi-med-u.ac.jp/JaPAN/current_initiatives-e.html](https://pheweb.jp/downloads) |
| Lung | EUR | 29,266 | 56,450 | 7,884,164 | McKay JD, et al. [8] | http://ftp.ebi.ac.uk/pub/databases/gwas/summary_statistics/GCST004001-GCST005000/GCST004748/ |
|  | EAS | 4,444 | 174,282 | 13,436,083 | Sakaue S, et al. [2] | <https://pheweb.jp/downloads> |
| Breast | EUR | 122,977 | 105,974 | 10,760,767 | Zhang H, et al. [9] | <https://bcac.ccge.medschl.cam.ac.uk/bcacdata/oncoarray/oncoarray-and-combined-summary-result/gwas-summary-associations-breast-cancer-risk-2020/> |
|  | EAS | 6,325 | 73,225 | 13,407,080 | Sakaue S, et al. [2] | <https://pheweb.jp/downloads> |
| Cervix | EUR | 6,563 | 219,656 | 9,984,823 | Rashkin SR, et al. [10] | https://github.com/Wittelab/pancancer_pleiotropy |
|  | EAS | 967 | 60,614 | 13,387,808 | Sakaue S, et al. [2] | <https://pheweb.jp/downloads> |
| Endometrium | EUR | 12,906 | 108,979 | 9,529,047 | O'Mara TA, et al. [11] | http://ftp.ebi.ac.uk/pub/databases/gwas/summary_statistics/GCST006001-GCST007000/GCST006464/ |
|  | EAS | 1,200 | 60,614 | 13,387,811 | Sakaue S, et al. [2] | <https://pheweb.jp/downloads> |
| Ovary | EUR | 22,406 | 40,941 | 20,594,648 | Phelan CM, et al. [12] | <http://ftp.ebi.ac.uk/pub/databases/gwas/summary_statistics/GCST004001-GCST005000/GCST004462/> |
|  | EAS | 843 | 60,614 | 13,387,365 | Sakaue S, et al. [2] | <https://pheweb.jp/downloads> |
| Prostate | EUR | 79,148 | 61,106 | 20,370,946 | Schumacher FR, et al. [13] | http://practical.icr.ac.uk/blog/?page_id=8164 |
|  | EAS | 5,672 | 84,660 | 13,412,985 | Sakaue S, et al. [2] | <https://pheweb.jp/downloads> |
| Bladder | EUR | 2,242 | 410,350 | 9,987,520 | Rashkin SR, et al. [10] | https://github.com/Wittelab/pancancer_pleiotropy |
|  | EAS | 580 | 1,101 | 3,675,899 | Wang X, et al. [14] | NJMU |

EUR, European population; EAS, East Asian population; NJMU, GWAS summary statistics from Nanjing Medical University.

**References**

1. Lesseur C, Diergaarde B, Olshan AF, Wünsch-Filho V, Ness AR, Liu G, et al. Genome-wide association analyses identify new susceptibility loci for oral cavity and pharyngeal cancer. Nat Genet. 2016;48(12):1544-50. doi: 10.1038/ng.3685. PMID: 27749845.

2. Sakaue S, Kanai M, Tanigawa Y, Karjalainen J, Kurki M, Koshiba S, et al. A cross-population atlas of genetic associations for 220 human phenotypes. Nat Genet. 2021;53(10):1415-24. doi: 10.1038/s41588-021-00931-x. PMID: 34594039.

3. Gharahkhani P, Fitzgerald RC, Vaughan TL, Palles C, Gockel I, Tomlinson I, et al. Genome-wide association studies in oesophageal adenocarcinoma and Barrett's oesophagus: a large-scale meta-analysis. Lancet Oncol. 2016;17(10):1363-73. doi: 10.1016/S1470-2045(16)30240-6. PMID: 27527254.

4. Fernandez-Rozadilla C, Timofeeva M, Chen Z, Law P, Thomas M, Schmit S, et al. Deciphering colorectal cancer genetics through multi-omic analysis of 100,204 cases and 154,587 controls of European and east Asian ancestries. Nat Genet. 2023;55(1):89-99. doi: 10.1038/s41588-022-01222-9. PMID: 36539618.

5. Han J, Chen C, Wang C, Qin N, Huang M, Ma Z, et al. Transcriptome-wide association study for persistent hepatitis B virus infection and related hepatocellular carcinoma. Liver Int. 2020;40(9):2117-27. doi: 10.1111/liv.14577. PMID: 32574393.

6. Petersen GM, Amundadottir L, Fuchs CS, Kraft P, Stolzenberg-Solomon RZ, Jacobs KB, et al. A genome-wide association study identifies pancreatic cancer susceptibility loci on chromosomes 13q22.1, 1q32.1 and 5p15.33. Nat Genet. 2010;42(3):224-8. doi: 10.1038/ng.522. PMID: 20101243.

7. Lin Y, Nakatochi M, Hosono Y, Ito H, Kamatani Y, Inoko A, et al. Genome-wide association meta-analysis identifies GP2 gene risk variants for pancreatic cancer. Nat Commun. 2020;11(1):3175. doi: 10.1038/s41467-020-16711-w. PMID: 32581250.

8. McKay JD, Hung RJ, Han Y, Zong X, Carreras-Torres R, Christiani DC, et al. Large-scale association analysis identifies new lung cancer susceptibility loci and heterogeneity in genetic susceptibility across histological subtypes. Nat Genet. 2017;49(7):1126-32. doi: 10.1038/ng.3892. PMID: 28604730.

9. Zhang H, Ahearn TU, Lecarpentier J, Barnes D, Beesley J, Qi G, et al. Genome-wide association study identifies 32 novel breast cancer susceptibility loci from overall and subtype-specific analyses. Nat Genet. 2020;52(6):572-81. doi: 10.1038/s41588-020-0609-2. PMID: 32424353.

10. Rashkin SR, Graff RE, Kachuri L, Thai KK, Alexeeff SE, Blatchins MA, et al. Pan-cancer study detects genetic risk variants and shared genetic basis in two large cohorts. Nat Commun. 2020;11(1):4423. doi: 10.1038/s41467-020-18246-6. PMID: 32887889.

11. O'Mara TA, Glubb DM, Amant F, Annibali D, Ashton K, Attia J, et al. Identification of nine new susceptibility loci for endometrial cancer. Nat Commun. 2018;9(1):3166. doi: 10.1038/s41467-018-05427-7. PMID: 30093612.

12. Phelan CM, Kuchenbaecker KB, Tyrer JP, Kar SP, Lawrenson K, Winham SJ, et al. Identification of 12 new susceptibility loci for different histotypes of epithelial ovarian cancer. Nat Genet. 2017;49(5):680-91. doi: 10.1038/ng.3826. PMID: 28346442.

13. Schumacher FR, Al Olama AA, Berndt SI, Benlloch S, Ahmed M, Saunders EJ, et al. Association analyses of more than 140,000 men identify 63 new prostate cancer susceptibility loci. Nat Genet. 2018;50(7):928-36. doi: 10.1038/s41588-018-0142-8. PMID: 29892016.

14. Wang X, Guo Z, Zhu H, Xin J, Yuan L, Qin C, et al. Genetic variants in splicing factor genes and susceptibility to bladder cancer. Gene. 2022;809:146022. doi: 10.1016/j.gene.2021.146022. PMID: 34673209.
