# Supplementary material for: Polygenic risk scores for pan-cancer risk prediction in the Chinese population: A population-based cohort study based on the China Kadoorie Biobank
Source: PLoS Med. 2025 Feb 28;22(2):e1004534. doi: 10.1371/journal.pmed.1004534 (PMC11870365; doi:10.1371/journal.pmed.1004534)
Supplement: S3 Table — BMI, body mass index. (DOCX) [file pmed.1004534.s007.docx]

**S3 Table. Modifiable risk factors assessed in this study in addition to age, sex (if applicable), region, and family history of cancer**

| **Cancer site** | **Risk factors** | **Reference** |
| --- | --- | --- |
| Head and neck | | |
|  | Alcohol status (never vs. drinker/abstainer) | Kachuri L, et al. [1] |
|  | BMI (<18.5 vs. [18.5, 24) vs. ≥24) | Kim CS, et al. [2] |
|  | Pack-years of smoking (never vs. <30 vs. ≥30) | Yu VX, et al. [3] |
|  | Highest education (college or up vs. high school vs. middle school vs. primary school/no formal school) | Conway DI, et al. [4] |
| Esophagus | | |
|  | Pack-years of smoking (never vs. <30 vs. ≥30) | Kachuri L, et al. [1] |
|  | BMI (<18.5 vs. [18.5, 24) vs. ≥24) | Kachuri L, et al. [1];  Han Y, et al. [5] |
|  | Highest education (college or up vs. high school vs. middle school vs. primary school/no formal school) | Han Y, et al. [5] |
|  | Alcohol status (never vs. drinker/abstainer) | Han Y, et al. [5] |
|  | Physical activity (MET hours /day) | Han Y, et al. [5] |
|  | Fruits intake (≥1 day/week vs. monthly/never) | Han Y, et al. [5] |
| Stomach | | |
|  | Pack-years of smoking (never vs. <30 vs. ≥30) | Zhu X, et al. [6] |
|  | Alcohol status (never vs. drinker/abstainer) | Zhu X, et al. [6] |
|  | Salty vegetables intake (≤3 days/week vs. ≥4 days/week) | Zhu X, et al. [6] |
|  | Vegetables and fruits intake (frequent vs. occasional) | Zhu X, et al. [6] |
|  | History of peptic ulcer (no vs. yes) | Zhu X, et al. [6] |
|  | Highest education (college or up vs. high school vs. middle school vs. primary school/no formal school) | Zhu X, et al. [6] |
| Colorectum | | |
|  | BMI (<18.5 vs. [18.5, 24) vs. ≥24) | Abhari RE, et al. [7] |
|  | Pack-years of smoking (never/<30 vs. ≥30) | Kachuri L, et al. [1];  Abhari RE, et al. [7] |
|  | Alcohol status (never vs. drinker/abstainer) | Kachuri L, et al. [1];  Abhari RE, et al. [7] |
|  | Meat intake (monthly/never vs. 1-3 days/week vs. ≥4 days/week) | Kachuri L, et al. [1];  Aleksandrova K, et al. [8] |
|  | Physical activity (MET hours /day) | Kachuri L, et al. [1];  Abhari RE, et al. [7] |
|  | Diabetes diagnosis (no vs. yes) | Abhari RE, et al. [7] |
| Liver | | |
|  | Highest education (college or up vs. high school vs. middle school vs. primary school/no formal school) | Song C, et al. [9] |
|  | Diabetes diagnosis (no vs. yes) | Song C, et al. [9] |
|  | History of gallstone/gallbladder (no vs. yes) | Luo X, et al. [10] |
|  | BMI (<18.5 vs. [18.5, 24) vs. ≥24) | Song C, et al. [9] |
|  | Physical activity (MET hours /day) | Song C, et al. [9] |
|  | Smoking status (never vs. smoker/ex-smoker) | Song C, et al. [9] |
|  | Alcohol status (never vs. drinker/abstainer) | Song C, et al. [9] |
|  | History of cirrhosis/chronic hepatitis (no vs. yes) | Song C, et al. [9] |
| Pancreas | | |
|  | BMI (<18.5 vs. [18.5, 24) vs. ≥24) | Kachuri L, et al. [1];  Klein AP. [11] |
|  | Pack-years of smoking (never vs. <30 vs. ≥30) | Kachuri L, et al. [1];  Klein AP. [11] |
|  | Alcohol status (never vs. drinker/abstainer) | Klein AP. [11] |
|  | Diabetes diagnosis (no vs. yes) | Klein AP. [11] |
| Lung | | |
|  | Pack-years of smoking (never vs. <30 vs. ≥30) | Ma Z, et al. [12] |
|  | Highest education (college or up vs. high school vs. middle school vs. primary school/no formal school) | Ma Z, et al. [12] |
|  | Height (cm) | Ma Z, et al. [12] |
|  | Frequent cough (no vs. yes) | Ma Z, et al. [12] |
|  | History of emphysema/bronchitis (no vs. yes) | Ma Z, et al. [12] |
|  | BMI (<18.5 vs. [18.5, 24) vs. ≥24) | Ma Z, et al. [12] |
|  | Physical activity (MET hours /day) | Ma Z, et al. [12] |
| Breast | | |
|  | Number of pregnancies (nulliparous/na vs. 1 vs. 2 vs. ≥3) | Kachuri L, et al. [1];  Han Y, et al. [13] |
|  | Age at menarche (<12 vs. 13-14 vs. 15-16 vs. ≥17 years) | Kachuri L, et al. [1];  Han Y, et al. [13] |
|  | Menopausal status (no vs. current/post/na) | Kachuri L, et al. [1] |
|  | BMI (<18.5 vs. [18.5, 24) vs. ≥24) | Kachuri L, et al. [1];  Han Y, et al. [13] |
|  | Alcohol status (never vs. drinker/abstainer) | Kachuri L, et al. [1] |
|  | Height (cm) | Kachuri L, et al. [1] |
|  | Highest education (college or up vs. high school vs. middle school vs. primary school/no formal school) | Han Y, et al. [13] |
| Cervix | | |
|  | Number of pregnancies (nulliparous/na vs. 1 vs. 2 vs. ≥3) | Kachuri L, et al. [1] |
|  | BMI (<18.5 vs. [18.5, 24) vs. ≥24) | Clarke MA, et al. [14] |
|  | Age at menarche (<12 vs. 13-14 vs. 15-16 vs. ≥17 years) | Dunyo P, et al. [15] |
|  | Alcohol status (never vs. drinker/abstainer) | Weiderpass E, et al. [16] |
| Endometrium | | |
|  | Number of pregnancies (nulliparous/na vs. 1 vs. 2 vs. ≥3) | Kachuri L, et al. [1] |
|  | Age at menarche (<12 vs. 13-14 vs. 15-16 vs. ≥17 years) | Kachuri L, et al. [1] |
|  | BMI (<18.5 vs. [18.5, 24) vs. ≥24) | Kachuri L, et al. [1] |
|  | Menopausal status (no vs. current/post/na) | Kachuri L, et al. [1] |
|  | Diabetes diagnosis (no vs. yes) | Braun MM, et al. [17] |
| Ovary | | |
|  | Number of pregnancies (nulliparous/na vs. 1 vs. 2 vs. ≥3) | Kachuri L, et al. [1] |
|  | BMI (<18.5 vs. [18.5, 24) vs. ≥24) | Kachuri L, et al. [1] |
|  | Menopausal status (no vs. current/post/na) | Kachuri L, et al. [1] |
|  | Height (cm) | Dixon-Suen SC, et al. [18] |
|  | Highest education (college or up vs. high school vs. middle school vs. primary school/no formal school) | Alberg AJ, et al. [19] |
| Prostate | | |
|  | Smoking status (never vs. smoker/ex-smoker) | Gandaglia G, et al. [20] |
|  | BMI (<18.5 vs. [18.5, 24) vs. ≥24) | Gandaglia G, et al. [20] |
|  | Physical activity (MET hours /day) | Gandaglia G, et al. [20] |
| Bladder | | |
|  | Pack-years of smoking (never vs. <30 vs. ≥30) | Kachuri L, et al. [1];  Cumberbatch MGK, et al. [21] |
|  | BMI (<18.5 vs. [18.5, 24) vs. ≥24) | Kachuri L, et al. [1];  Cumberbatch MGK, et al. [21] |
|  | Meat intake (monthly/never vs. 1-3 days/week vs. ≥4 days/week) | Cumberbatch MGK, et al.[21] |

BMI, body mass index.

**References**

1. Kachuri L, Graff RE, Smith-Byrne K, Meyers TJ, Rashkin SR, Ziv E, et al. Pan-cancer analysis demonstrates that integrating polygenic risk scores with modifiable risk factors improves risk prediction. Nat Commun. 2020;11(1):6084. doi: 10.1038/s41467-020-19600-4. PMID: 33247094.

2. Kim C-S, Park J-O, Nam I-C, Park SJ, Lee D-H, Kim H-B, et al. Associations of Body Mass Index and Waist Circumference with the Risk of Head and Neck Cancer: A National Population-Based Study. Cancers. 2022;14(16). doi: 10.3390/cancers14163880. PMID: 36010881.

3. Yu VX, Long S, Tassler A. Smoking and Head and Neck Cancer. JAMA Otolaryngol Head Neck Surg. 2023;149(5):470. doi: 10.1001/jamaoto.2023.0195. PMID: 36995722.

4. Conway DI, Brenner DR, McMahon AD, Macpherson LMD, Agudo A, Ahrens W, et al. Estimating and explaining the effect of education and income on head and neck cancer risk: INHANCE consortium pooled analysis of 31 case-control studies from 27 countries. Int J Cancer. 2015;136(5):1125-39. doi: 10.1002/ijc.29063. PMID: 24996155.

5. Han Y, Zhu X, Hu Y, Yu C, Guo Y, Hang D, et al. Electronic Health Record-Based Absolute Risk Prediction Model for Esophageal Cancer in the Chinese Population: Model Development and External Validation. JMIR Public Health Surveill. 2023;9:e43725. doi: 10.2196/43725. PMID: 36781293.

6. Zhu X, Lv J, Zhu M, Yan C, Deng B, Yu C, et al. Development, validation, and evaluation of a risk assessment tool for personalized screening of gastric cancer in Chinese populations. BMC Med. 2023;21(1):159. doi: 10.1186/s12916-023-02864-0. PMID: 37106459.

7. Abhari RE, Thomson B, Yang L, Millwood I, Guo Y, Yang X, et al. External validation of models for predicting risk of colorectal cancer using the China Kadoorie Biobank. BMC Med. 2022;20(1):302. doi: 10.1186/s12916-022-02488-w. PMID: 36071519.

8. Aleksandrova K, Reichmann R, Kaaks R, Jenab M, Bueno-de-Mesquita HB, Dahm CC, et al. Development and validation of a lifestyle-based model for colorectal cancer risk prediction: the LiFeCRC score. BMC Med. 2021;19(1):1. doi: 10.1186/s12916-020-01826-0. PMID: 33390155.

9. Song C, Lv J, Yu C, Zhu M, Yu C, Guo Y, et al. Adherence to Healthy Lifestyle and Liver cancer in Chinese: a prospective cohort study of 0.5 million people. Br J Cancer. 2022;126(5):815-21. doi: 10.1038/s41416-021-01645-x. PMID: 34853434.

10. Luo X, Yang W, Joshi AD, Wu K, Simon TG, Yuan C, et al. Gallstones and risk of cancers of the liver, biliary tract and pancreas: a prospective study within two U.S. cohorts. Br J Cancer. 2022;127(6):1069-75. doi: 10.1038/s41416-022-01877-5. PMID: 35715632.

11. Klein AP. Pancreatic cancer epidemiology: understanding the role of lifestyle and inherited risk factors. Nat Rev Gastroenterol Hepatol. 2021;18(7):493-502. doi: 10.1038/s41575-021-00457-x. PMID: 34002083.

12. Ma Z, Lv J, Zhu M, Yu C, Ma H, Jin G, et al. Lung cancer risk score for ever and never smokers in China. Cancer Commun (Lond). 2023;43(8):877-95. doi: 10.1002/cac2.12463. PMID: 37410540.

13. Han Y, Lv J, Yu C, Guo Y, Bian Z, Hu Y, et al. Development and external validation of a breast cancer absolute risk prediction model in Chinese population. Breast Cancer Res. 2021;23(1):62. doi: 10.1186/s13058-021-01439-2. PMID: 34051827.

14. Clarke MA, Fetterman B, Cheung LC, Wentzensen N, Gage JC, Katki HA, et al. Epidemiologic Evidence That Excess Body Weight Increases Risk of Cervical Cancer by Decreased Detection of Precancer. J Clin Oncol. 2018;36(12):1184-91. doi: 10.1200/JCO.2017.75.3442. PMID: 29356609.

15. Dunyo P, Effah K, Udofia EA. Factors associated with late presentation of cervical cancer cases at a district hospital: a retrospective study. BMC Public Health. 2018;18(1):1156. doi: 10.1186/s12889-018-6065-6. PMID: 30285699.

16. Weiderpass E, Ye W, Tamimi R, Trichopolous D, Nyren O, Vainio H, et al. Alcoholism and risk for cancer of the cervix uteri, vagina, and vulva. Cancer Epidemiol Biomarkers Prev. 2001;10(8):899-901. PMID: 11489758.

17. Braun MM, Overbeek-Wager EA, Grumbo RJ. Diagnosis and Management of Endometrial Cancer. Am Fam Physician. 2016;93(6):468-74. PMID: 26977831.

18. Dixon-Suen SC, Nagle CM, Thrift AP, Pharoah PDP, Ewing A, Pearce CL, et al. Adult height is associated with increased risk of ovarian cancer: a Mendelian randomisation study. Br J Cancer. 2018;118(8):1123-9. doi: 10.1038/s41416-018-0011-3. PMID: 29555990.

19. Alberg AJ, Moorman PG, Crankshaw S, Wang F, Bandera EV, Barnholtz-Sloan JS, et al. Socioeconomic Status in Relation to the Risk of Ovarian Cancer in African-American Women: A Population-Based Case-Control Study. Am J Epidemiol. 2016;184(4):274-83. doi: 10.1093/aje/kwv450. PMID: 27492896.

20. Gandaglia G, Leni R, Bray F, Fleshner N, Freedland SJ, Kibel A, et al. Epidemiology and Prevention of Prostate Cancer. Eur Urol Oncol. 2021;4(6):877-92. doi: 10.1016/j.euo.2021.09.006. PMID: 34716119.

21. Cumberbatch MGK, Jubber I, Black PC, Esperto F, Figueroa JD, Kamat AM, et al. Epidemiology of Bladder Cancer: A Systematic Review and Contemporary Update of Risk Factors in 2018. Eur Urol. 2018;74(6):784-95. doi: 10.1016/j.eururo.2018.09.001. PMID: 30268659.
