# Supplementary material for: Polygenic risk scores for pan-cancer risk prediction in the Chinese population: A population-based cohort study based on the China Kadoorie Biobank
Source: PLoS Med. 2025 Feb 28;22(2):e1004534. doi: 10.1371/journal.pmed.1004534 (PMC11870365; doi:10.1371/journal.pmed.1004534)
Supplement: S4 Table — (DOCX) [file pmed.1004534.s008.docx]

**S4 Table. Incidence rate of 13 cancers in the CKB cohort**

| **Cancer site** | **Cases** | **Non-cases** | **Person-years** | **Incidence rate**  **(per 100,000 person-years)** |
| --- | --- | --- | --- | --- |
| Head and neck | 162 | 100,057 | 1,072,892 | 15.10 |
| Esophagus | 499 | 99,720 | 1,072,409 | 46.53 |
| Stomach | 745 | 99,474 | 1,072,029 | 69.49 |
| Colorectum | 740 | 99,479 | 1,071,084 | 69.09 |
| Liver | 661 | 99,558 | 1,072,817 | 61.61 |
| Pancreas | 170 | 100,049 | 1,073,278 | 15.84 |
| Lung | 1,540 | 98,679 | 1,071,248 | 143.76 |
| Breast | 486 | 56,873 | 627,180 | 77.49 |
| Cervix | 237 | 57,122 | 628,272 | 37.72 |
| Endometrium | 88 | 57,271 | 628,898 | 13.99 |
| Ovary | 96 | 57,263 | 628,957 | 15.26 |
| Prostate | 95 | 42,765 | 443,967 | 21.40 |
| Bladder | 149 | 100,070 | 1,072,912 | 13.89 |
