# Supplementary material for: Polygenic risk scores for pan-cancer risk prediction in the Chinese population: A population-based cohort study based on the China Kadoorie Biobank
Source: PLoS Med. 2025 Feb 28;22(2):e1004534. doi: 10.1371/journal.pmed.1004534 (PMC11870365; doi:10.1371/journal.pmed.1004534)
Supplement: S5 Table — PRS, polygenic risk score; HR, hazard ratio; CI, confidence interval; SNP, single-nucleotide polymorphism. (DOCX) [file pmed.1004534.s009.docx]

**S5 Table. Association details of the developed polygenic risk scores for each cancer type and corresponding C-index estimated by Cox regression models**

| **Cancer site** | **PRS** | **No. SNP** | **Model 1 ^*^** | | |  | **Model 2 ^†^** | | |
| --- | --- | --- | --- | --- | --- | --- | --- | --- | --- |
|  |  |  | **HR (95% CI)** | ***P-*value** | **C-index (95% CI)** |  | **HR (95% CI)** | ***P-*value** | **C-index (95% CI)** |
| Head and neck |  |  |  |  |  |  |  |  |  |
|  | PRS1 | 3 | 1.34 (1.15-1.57) | 2.30×10^-04^ | 0.574 (0.535-0.624) |  | 1.09 (0.92-1.28) | 0.320 | 0.752 (0.715-0.790) |
|  | PRS2 | 3 | 1.34 (1.15-1.57) | 2.30×10^-04^ | 0.574 (0.535-0.624) |  | 1.09 (0.92-1.28) | 0.320 | 0.752 (0.715-0.790) |
|  | PRS3 | 5 | 1.29 (1.11-1.50) | 0.001 | 0.567 (0.526-0.609) |  | 1.04 (0.89-1.23) | 0.603 | 0.752 (0.715-0.790) |
|  | PRS4 | 9 | 1.23 (1.06-1.44) | 0.008 | 0.546 (0.501-0.592) |  | 1.10 (0.94-1.28) | 0.238 | 0.752 (0.715-0.790) |
|  | PRS5 | 14 | 1.02 (0.88-1.19) | 0.763 | 0.511 (0.467-0.556) |  | 1.03 (0.88-1.20) | 0.719 | 0.752 (0.715-0.790) |
|  | PRS7 | 2,375 | 1.00 (0.86-1.17) | 0.982 | 0.498 (0.456-0.541) |  | 0.98 (0.84-1.14) | 0.779 | 0.753 (0.715-0.790) |
| Esophagus |  |  |  |  |  |  |  |  |  |
|  | PRS1 | 6 | 1.12 (1.03-1.22) | 0.010 | 0.534 (0.507-0.560) |  | 1.19 (1.09-1.30) | 7.58×10^-05^ | 0.874 (0.860-0.889) |
|  | PRS2 | 9 | 1.10 (1.01-1.20) | 0.024 | 0.536 (0.511-0.561) |  | 1.23 (1.12-1.34) | 5.26×10^-06^ | 0.876 (0.862-0.890) |
|  | PRS3 | 11 | 1.23 (1.13-1.34) | 3.32×10^-06^ | 0.559 (0.534-0.585) |  | 1.26 (1.16-1.38) | 1.33×10^-07^ | 0.876 (0.862-0.891) |
|  | PRS4 | 16 | 1.17 (1.07-1.27) | 3.32×10^-04^ | 0.553 (0.529-0.578) |  | 1.26 (1.15-1.37) | 2.39×10^-07^ | 0.877 (0.862-0.891) |
|  | PRS5 | 13 | 0.96 (0.88-1.05) | 0.332 | 0.517 (0.492-0.542) |  | 0.97 (0.89-1.06) | 0.494 | 0.874 (0.859-0.888) |
|  | PRS7 | 2,909 | 1.00 (0.92-1.09) | 0.961 | 0.497 (0.471-0.523) |  | 1.04 (0.95-1.13) | 0.435 | 0.874 (0.859-0.888) |
| Stomach |  |  |  |  |  |  |  |  |  |
|  | PRS1 | 8 | 1.29 (1.19-1.39) | 4.06×10^-11^ | 0.568 (0.547-0.589) |  | 1.28 (1.18-1.38) | 3.28×10^-10^ | 0.777 (0.762-0.793) |
|  | PRS2 | 12 | 1.31 (1.21-1.41) | 1.85×10^-12^ | 0.572 (0.551-0.592) |  | 1.27 (1.18-1.37) | 2.59×10^-10^ | 0.777 (0.762-0.793) |
|  | PRS3 | 38 | 1.22 (1.14-1.31) | 4.36×10^-08^ | 0.556 (0.535-0.577) |  | 1.21 (1.12-1.30) | 2.72×10^-07^ | 0.775 (0.759-0.791) |
|  | PRS4 | 41 | 1.24 (1.15-1.33) | 5.06×10^-09^ | 0.559 (0.538-0.580) |  | 1.21 (1.12-1.30) | 3.21×10^-07^ | 0.775 (0.759-0.791) |
|  | PRS5 | 3 | 1.23 (1.15-1.33) | 1.56×10^-08^ | 0.559 (0.541-0.587) |  | 1.21 (1.12-1.30) | 4.09×10^-07^ | 0.775 (0.759-0.791) |
|  | PRS6 | 112 | 1.27 (1.18-1.36) | 6.24×10^-11^ | 0.568 (0.547-0.589) |  | 1.24 (1.16-1.34) | 3.75×10^-09^ | 0.776 (0.760-0.792) |
|  | PRS7 | 2,797 | 1.06 (0.99-1.14) | 0.098 | 0.523 (0.501-0.544) |  | 1.05 (0.97-1.12) | 0.220 | 0.771 (0.755-0.787) |
| Colorectum |  |  |  |  |  |  |  |  |  |
|  | PRS1 | 33 | 1.29 (1.20-1.38) | 5.40×10^-12^ | 0.572 (0.551-0.594) |  | 1.29 (1.20-1.39) | 3.06×10^-12^ | 0.739 (0.722-0.757) |
|  | PRS2 | 38 | 1.28 (1.19-1.38) | 1.39×10^-11^ | 0.572 (0.551-0.593) |  | 1.29 (1.20-1.39) | 6.43×10^-12^ | 0.739 (0.721-0.756) |
|  | PRS3 | 99 | 1.43 (1.33-1.53) | 2.10×10^-22^ | 0.599 (0.579-0.620) |  | 1.44 (1.34-1.55) | 3.31×10^-23^ | 0.748 (0.731-0.765) |
|  | PRS4 | 114 | 1.42 (1.32-1.53) | 3.28×10^-22^ | 0.601 (0.580-0.621) |  | 1.45 (1.35-1.56) | 1.49×10^-23^ | 0.748 (0.731-0.765) |
|  | PRS5 | 91 | 1.44 (1.34-1.55) | 5.59×10^-23^ | 0.603 (0.582-0.623) |  | 1.46 (1.36-1.57) | 2.63×10^-24^ | 0.746 (0.728-0.763) |
|  | PRS6 | 115 | 1.45 (1.35-1.56) | 3.26×10^-24^ | 0.604 (0.583-0.624) |  | 1.47 (1.37-1.58) | 2.76×10^-25^ | 0.747 (0.730-0.764) |
|  | PRS7 | 11,462 | 1.51 (1.41-1.63) | 2.32×10^-29^ | 0.612 (0.591-0.633) |  | 1.54 (1.43-1.66) | 6.07×10^-31^ | 0.752 (0.735-0.769) |
| Liver |  |  |  |  |  |  |  |  |  |
|  | PRS1 | 4 | 1.04 (0.96-1.13) | 0.292 | 0.509 (0.486-0.533) |  | 1.04 (0.96-1.12) | 0.313 | 0.747 (0.729-0.766) |
|  | PRS2 | 8 | 1.06 (0.98-1.14) | 0.168 | 0.513 (0.491-0.535) |  | 1.05 (0.97-1.13) | 0.213 | 0.747 (0.729-0.766) |
|  | PRS3 | 4 | 1.04 (0.96-1.13) | 0.292 | 0.509 (0.486-0.533) |  | 1.04 (0.96-1.12) | 0.313 | 0.747 (0.729-0.766) |
|  | PRS4 | 8 | 1.06 (0.98-1.14) | 0.168 | 0.513 (0.491-0.535) |  | 1.05 (0.97-1.13) | 0.213 | 0.747 (0.729-0.766) |
|  | PRS7 | 80,890 | 1.05 (0.98-1.13) | 0.178 | 0.521 (0.500-0.542) |  | 1.63 (1.13-2.36) | 0.009 | 0.748 (0.730-0.767) |
| Pancreas |  |  |  |  |  |  |  |  |  |
|  | PRS1 | 8 | 1.03 (0.89-1.20) | 0.680 | 0.514 (0.468-0.560) |  | 1.02 (0.87-1.18) | 0.829 | 0.775 (0.743-0.806) |
|  | PRS2 | 8 | 1.03 (0.89-1.20) | 0.680 | 0.514 (0.468-0.560) |  | 1.02 (0.87-1.18) | 0.829 | 0.775 (0.743-0.806) |
|  | PRS3 | 17 | 1.14 (0.98-1.32) | 0.094 | 0.541 (0.497-0.584) |  | 1.12 (0.96-1.30) | 0.140 | 0.777 (0.745-0.808) |
|  | PRS4 | 24 | 1.17 (1.01-1.36) | 0.040 | 0.548 (0.505-0.591) |  | 1.16 (1.00-1.35) | 0.053 | 0.778 (0.747-0.810) |
|  | PRS5 | 21 | 1.29 (1.11-1.49) | 8.63×10^-04^ | 0.586 (0.544-0.627) |  | 1.28 (1.10-1.48) | 0.001 | 0.781 (0.748-0.814) |
|  | PRS7 | 1,329 | 1.05 (0.91-1.22) | 0.502 | 0.509 (0.467-0.552) |  | 1.03 (0.89-1.20) | 0.657 | 0.774 (0.742-0.806) |
| Lung |  |  |  |  |  |  |  |  |  |
|  | PRS1 | 19 | 1.17 (1.12-1.23) | 2.56×10^-10^ | 0.542 (0.527-0.556) |  | 1.17 (1.12-1.23) | 3.12×10^-10^ | 0.783 (0.772-0.794) |
|  | PRS2 | 22 | 1.18 (1.13-1.24) | 3.58×10^-11^ | 0.544 (0.529-0.558) |  | 1.18 (1.13-1.24) | 4.48×10^-11^ | 0.783 (0.772-0.794) |
|  | PRS3 | 39 | 1.21 (1.15-1.27) | 6.36×10^-14^ | 0.549 (0.534-0.564) |  | 1.21 (1.15-1.27) | 9.78×10^-14^ | 0.784 (0.773-0.795) |
|  | PRS4 | 42 | 1.21 (1.15-1.27) | 3.07×10^-14^ | 0.550 (0.535-0.565) |  | 1.22 (1.16-1.28) | 2.06×10^-14^ | 0.784 (0.773-0.795) |
|  | PRS5 | 32 | 1.16 (1.11-1.22) | 2.02×10^-09^ | 0.542 (0.528-0.557) |  | 1.17 (1.11-1.23) | 3.98×10^-10^ | 0.782 (0.771-0.793) |
|  | PRS6 | 19 | 1.20 (1.14-1.26) | 5.31×10^-13^ | 0.549 (0.534-0.564) |  | 1.22 (1.16-1.28) | 8.95×10^-15^ | 0.784 (0.773-0.795) |
|  | PRS7 | 4,260 | 1.18 (1.12-1.24) | 2.61×10^-10^ | 0.544 (0.530-0.558) |  | 1.17 (1.11-1.23) | 1.79×10^-09^ | 0.782 (0.771-0.793) |
| Breast |  |  |  |  |  |  |  |  |  |
|  | PRS1 | 12 | 1.38 (1.26-1.50) | 8.25×10^-13^ | 0.585 (0.560-0.611) |  | 1.37 (1.26-1.50) | 1.99×10^-12^ | 0.703 (0.680-0.725) |
|  | PRS2 | 14 | 1.41 (1.29-1.53) | 2.20×10^-14^ | 0.591 (0.565-0.617) |  | 1.40 (1.28-1.52) | 6.56×10^-14^ | 0.705 (0.682-0.728) |
|  | PRS3 | 104 | 1.41 (1.29-1.54) | 2.75×10^-14^ | 0.593 (0.568-0.618) |  | 1.40 (1.29-1.53) | 5.37×10^-14^ | 0.703 (0.681-0.726) |
|  | PRS4 | 109 | 1.42 (1.30-1.56) | 4.78×10^-15^ | 0.596 (0.570-0.621) |  | 1.42 (1.30-1.55) | 1.49×10^-14^ | 0.704 (0.681-0.727) |
|  | PRS5 | 289 | 1.38 (1.26-1.51) | 1.32×10^-12^ | 0.585 (0.560-0.610) |  | 1.36 (1.25-1.49) | 7.17×10^-12^ | 0.701 (0.678-0.724) |
|  | PRS6 | 110 | 1.38 (1.27-1.51) | 8.02×10^-13^ | 0.591 (0.566-0.616) |  | 1.36 (1.25-1.49) | 7.72×10^-12^ | 0.702 (0.680-0.725) |
|  | PRS7 | 7,688 | 1.42 (1.30-1.56) | 3.76×10^-15^ | 0.594 (0.568-0.620) |  | 1.41 (1.29-1.54) | 3.59×10^-14^ | 0.707 (0.684-0.729) |
| Cervix |  |  |  |  |  |  |  |  |  |
|  | PRS1 | 6 | 1.21 (1.07-1.38) | 0.003 | 0.560 (0.522-0.598) |  | 1.24 (1.09-1.40) | 0.001 | 0.705 (0.673-0.738) |
|  | PRS2 | 7 | 1.15 (1.01-1.30) | 0.036 | 0.541 (0.504-0.579) |  | 1.15 (1.01-1.30) | 0.034 | 0.700 (0.667-0.732) |
|  | PRS3 | 6 | 1.21 (1.07-1.38) | 0.003 | 0.560 (0.522-0.598) |  | 1.24 (1.09-1.40) | 0.001 | 0.705 (0.673-0.738) |
|  | PRS4 | 15 | 1.21 (1.07-1.38) | 0.002 | 0.566 (0.528-0.603) |  | 1.20 (1.06-1.36) | 0.004 | 0.703 (0.671-0.735) |
|  | PRS5 | 20 | 1.05 (0.93-1.20) | 0.422 | 0.519 (0.483-0.556) |  | 1.05 (0.92-1.19) | 0.469 | 0.695 (0.663-0.728) |
|  | PRS7 | 3,754 | 1.17 (1.03-1.33) | 0.017 | 0.543 (0.505-0.581) |  | 1.17 (1.03-1.33) | 0.013 | 0.700 (0.667-0.732) |
| Endometrium |  |  |  |  |  |  |  |  |  |
|  | PRS3 | 2 | 0.94 (0.76-1.15) | 0.530 | 0.537 (0.478-0.617) |  | 0.94 (0.77-1.16) | 0.572 | 0.705 (0.649-0.762) |
|  | PRS4 | 3 | 0.97 (0.79-1.20) | 0.799 | 0.507 (0.440-0.576) |  | 0.98 (0.80-1.21) | 0.853 | 0.704 (0.647-0.761) |
|  | PRS5 | 9 | 0.99 (0.80-1.22) | 0.917 | 0.515 (0.453-0.578) |  | 1.00 (0.81-1.23) | 0.994 | 0.704 (0.647-0.761) |
|  | PRS7 | 3,307 | 0.98 (0.79-1.21) | 0.835 | 0.515 (0.451-0.579) |  | 0.97 (0.79-1.20) | 0.798 | 0.704 (0.648-0.761) |
| Ovary |  |  |  |  |  |  |  |  |  |
|  | PRS1 | 3 | 1.15 (0.95-1.40) | 0.154 | 0.545 (0.485-0.610) |  | 1.15 (0.94-1.39) | 0.172 | 0.668 (0.611-0.725) |
|  | PRS2 | 3 | 1.15 (0.95-1.40) | 0.154 | 0.545 (0.485-0.610) |  | 1.15 (0.94-1.39) | 0.172 | 0.668 (0.611-0.725) |
|  | PRS3 | 15 | 1.09 (0.89-1.32) | 0.405 | 0.508 (0.449-0.568) |  | 1.09 (0.89-1.33) | 0.405 | 0.664 (0.606-0.723) |
|  | PRS4 | 19 | 1.09 (0.89-1.32) | 0.415 | 0.508 (0.448-0.568) |  | 1.08 (0.89-1.32) | 0.433 | 0.665 (0.608-0.723) |
|  | PRS5 | 26 | 1.08 (0.88-1.31) | 0.471 | 0.529 (0.466-0.592) |  | 1.08 (0.88-1.31) | 0.464 | 0.665 (0.607-0.723) |
|  | PRS7 | 2,802 | 1.25 (1.02-1.52) | 0.030 | 0.579 (0.525-0.632) |  | 1.25 (1.02-1.52) | 0.031 | 0.675 (0.618-0.732) |
| Prostate |  |  |  |  |  |  |  |  |  |
|  | PRS1 | 32 | 1.54 (1.26-1.90) | 3.37×10^-05^ | 0.612 (0.550-0.673) |  | 1.50 (1.22-1.84) | 1.26×10^-04^ | 0.887 (0.859-0.915) |
|  | PRS2 | 37 | 1.55 (1.27-1.90) | 2.14×10^-05^ | 0.622 (0.560-0.684) |  | 1.51 (1.24-1.85) | 6.37×10^-05^ | 0.889 (0.862-0.916) |
|  | PRS3 | 144 | 1.75 (1.43-2.13) | 5.17×10^-08^ | 0.646 (0.587-0.704) |  | 1.71 (1.39-2.09) | 2.82×10^-07^ | 0.890 (0.861-0.918) |
|  | PRS4 | 157 | 1.78 (1.46-2.17) | 1.39×10^-08^ | 0.658 (0.600-0.716) |  | 1.75 (1.43-2.15) | 6.22×10^-08^ | 0.893 (0.865-0.920) |
|  | PRS5 | 242 | 1.75 (1.43-2.13) | 5.35×10^-08^ | 0.647 (0.588-0.706) |  | 1.69 (1.38-2.07) | 3.89×10^-07^ | 0.891 (0.863-0.919) |
|  | PRS6 | 29 | 1.65 (1.35-2.02) | 8.74×10^-07^ | 0.617 (0.554-0.680) |  | 1.59 (1.29-1.94) | 8.55×10^-06^ | 0.887 (0.858-0.916) |
|  | PRS7 | 8,276 | 1.83 (1.51-2.22) | 1.07×10^-09^ | 0.645 (0.585-0.705) |  | 1.75 (1.44-2.13) | 2.02×10^-08^ | 0.892 (0.864-0.920) |
| Bladder |  |  |  |  |  |  |  |  |  |
|  | PRS1 | 4 | 1.00 (0.85-1.18) | 0.985 | 0.502 (0.449-0.556) |  | 1.00 (0.85-1.17) | 0.962 | 0.826 (0.792-0.860) |
|  | PRS2 | 4 | 1.00 (0.85-1.18) | 0.985 | 0.502 (0.449-0.556) |  | 1.00 (0.85-1.17) | 0.962 | 0.826 (0.792-0.860) |
|  | PRS3 | 18 | 1.09 (0.93-1.28) | 0.291 | 0.539 (0.491-0.587) |  | 1.10 (0.93-1.29) | 0.260 | 0.828 (0.794-0.862) |
|  | PRS4 | 18 | 1.09 (0.93-1.28) | 0.291 | 0.539 (0.491-0.587) |  | 1.10 (0.93-1.29) | 0.260 | 0.828 (0.794-0.862) |
|  | PRS5 | 15 | 1.17 (0.99-1.37) | 0.060 | 0.541 (0.492-0.589) |  | 1.18 (1.01-1.39) | 0.039 | 0.829 (0.796-0.862) |
|  | PRS7 | 2,582 | 1.11 (0.94-1.30) | 0.217 | 0.531 (0.482-0.580) |  | 1.11 (0.94-1.30) | 0.210 | 0.826 (0.792-0.861) |

PRS, polygenic risk score; HR, hazard ratio; CI, confidence interval; SNP, single nucleotide polymorphism.

^*^ PRS-only (unadjusted).

^†^ Adjusted for age, sex (if applicable), region, the top 10 principal components, family history of cancer, and modifiable risk factors.
