# Supplementary material for: Polygenic risk scores for pan-cancer risk prediction in the Chinese population: A population-based cohort study based on the China Kadoorie Biobank
Source: PLoS Med. 2025 Feb 28;22(2):e1004534. doi: 10.1371/journal.pmed.1004534 (PMC11870365; doi:10.1371/journal.pmed.1004534)
Supplement: S6 Table — PRS, polygenic risk score; HR, hazard ratio; CI, confidence interval. (DOCX) [file pmed.1004534.s010.docx]

**S6 Table. Association details of the optimal polygenic risk scores for the nine cancers in the CKB cohort**

| **Cancer site** | **PRS group ^*^** | **Cases** | **Person-years** | **Incidence rate ^†^** | **Model 1 ^‡^** | | |  | **Model 2 ^§^** | | |
| --- | --- | --- | --- | --- | --- | --- | --- | --- | --- | --- | --- |
|  |  |  |  |  | **HR (95% CI)** | ***P-*value** | ***P*_trend** |  | **HR (95% CI)** | ***P-*value** | ***P*_trend** |
| Esophagus |  |  |  |  |  |  |  |  |  |  |  |
|  | <20 | 76 | 214,926 | 35.36 | Ref | - |  |  | Ref | - |  |
|  | [20,40) | 84 | 214,381 | 39.18 | 1.11 (0.82-1.52) | 0.503 |  |  | 1.09 (0.80-1.49) | 0.588 |  |
|  | [40,60) | 103 | 214,527 | 48.01 | 1.44 (1.07-1.93) | 0.017 |  |  | 1.41 (1.05-1.90) | 0.023 |  |
|  | [60,80) | 96 | 214,400 | 44.78 | 1.37 (1.01-1.85) | 0.041 |  |  | 1.34 (0.99-1.81) | 0.057 |  |
|  | ≥80 | 140 | 214,175 | 65.37 | 2.05 (1.55-2.71) | 4.96×10^-07^ | 1.14×10^-07^ |  | 1.95 (1.47-2.58) | 2.87×10^-06^ | 6.22×10^-07^ |
| Stomach |  |  |  |  |  |  |  |  |  |  |  |
|  | <20 | 95 | 215,415 | 44.10 | Ref | - |  |  | Ref | - |  |
|  | [20,40) | 143 | 214,361 | 66.71 | 1.47 (1.13-1.91) | 0.004 |  |  | 1.47 (1.13-1.90) | 0.004 |  |
|  | [40,60) | 132 | 214,622 | 61.50 | 1.35 (1.04-1.76) | 0.026 |  |  | 1.35 (1.04-1.76) | 0.026 |  |
|  | [60,80) | 168 | 213,666 | 78.63 | 1.67 (1.30-2.16) | 6.45×10^-05^ |  |  | 1.67 (1.30-2.16) | 6.39×10^-05^ |  |
|  | ≥80 | 207 | 213,965 | 96.74 | 2.04 (1.60-2.61) | 1.02×10^-08^ | 8.33×10^-09^ |  | 2.05 (1.60-2.62) | 9.29×10^-09^ | 7.08×10^-09^ |
| Colorectum |  |  |  |  |  |  |  |  |  |  |  |
|  | <20 | 84 | 215,305 | 39.01 | Ref | - |  |  | Ref | - |  |
|  | [20,40) | 124 | 214,240 | 57.88 | 1.50 (1.14-1.98) | 0.004 |  |  | 1.49 (1.13-1.97) | 0.005 |  |
|  | [40,60) | 122 | 214,640 | 56.84 | 1.49 (1.13-1.97) | 0.005 |  |  | 1.49 (1.12-1.97) | 0.005 |  |
|  | [60,80) | 154 | 213,949 | 71.98 | 1.93 (1.48-2.52) | 1.44×10^-06^ |  |  | 1.93 (1.47-2.52) | 1.55×10^-06^ |  |
|  | ≥80 | 256 | 212,950 | 120.22 | 3.25 (2.53-4.18) | 2.18×10^-20^ | 1.06×10^-23^ |  | 3.23 (2.51-4.14) | 4.03×10^-20^ | 1.87×10^-23^ |
| Pancreas |  |  |  |  |  |  |  |  |  |  |  |
|  | <20 | 19 | 215,782 | 8.81 | Ref | - |  |  | Ref | - |  |
|  | [20,40) | 34 | 214,310 | 15.86 | 1.75 (1.00-3.07) | 0.050 |  |  | 1.75 (1.00-3.08) | 0.050 |  |
|  | [40,60) | 27 | 214,237 | 12.60 | 1.42 (0.79-2.55) | 0.245 |  |  | 1.41 (0.78-2.54) | 0.251 |  |
|  | [60,80) | 42 | 214,660 | 19.57 | 2.21 (1.28-3.80) | 0.004 |  |  | 2.20 (1.28-3.78) | 0.004 |  |
|  | ≥80 | 48 | 214,289 | 22.40 | 2.51 (1.47-4.27) | 6.98×10^-04^ | 3.72×10^-04^ |  | 2.48 (1.46-4.22) | 8.10×10^-04^ | 4.53×10^-04^ |
| Lung |  |  |  |  |  |  |  |  |  |  |  |
|  | <20 | 251 | 214,409 | 117.07 | Ref | - |  |  | Ref | - |  |
|  | [20,40) | 258 | 214,643 | 120.20 | 1.02 (0.86-1.22) | 0.808 |  |  | 1.02 (0.86-1.22) | 0.793 |  |
|  | [40,60) | 283 | 214,391 | 132.00 | 1.11 (0.93-1.31) | 0.241 |  |  | 1.11 (0.94-1.32) | 0.226 |  |
|  | [60,80) | 359 | 214,221 | 167.58 | 1.42 (1.21-1.67) | 1.79×10^-05^ |  |  | 1.42 (1.21-1.67) | 2.05×10^-05^ |  |
|  | ≥80 | 389 | 213,583 | 182.13 | 1.57 (1.34-1.84) | 2.43×10^-08^ | 3.79×10^-12^ |  | 1.58 (1.35-1.85) | 1.83×10^-08^ | 3.30×10^-12^ |
| Breast |  |  |  |  |  |  |  |  |  |  |  |
|  | <20 | 54 | 125,725 | 42.95 | Ref | - |  |  | Ref | - |  |
|  | [20,40) | 73 | 125,547 | 58.15 | 1.38 (0.97-1.96) | 0.076 |  |  | 1.38 (0.97-1.96) | 0.074 |  |
|  | [40,60) | 90 | 125,806 | 71.54 | 1.67 (1.19-2.35) | 0.003 |  |  | 1.66 (1.19-2.33) | 0.003 |  |
|  | [60,80) | 129 | 125,093 | 103.12 | 2.41 (1.75-3.31) | 6.11×10^-08^ |  |  | 2.41 (1.76-3.32) | 5.61×10^-08^ |  |
|  | ≥80 | 140 | 125,010 | 111.99 | 2.58 (1.88-3.53) | 3.32×10^-09^ | 8.97×10^-13^ |  | 2.59 (1.89-3.54) | 2.93×10^-09^ | 7.35×10^-13^ |
| Cervix |  |  |  |  |  |  |  |  |  |  |  |
|  | <20 | 37 | 126,126 | 29.34 | Ref | - |  |  | Ref | - |  |
|  | [20,40) | 37 | 125,743 | 29.43 | 0.99 (0.63-1.56) | 0.965 |  |  | 0.99 (0.63-1.56) | 0.968 |  |
|  | [40,60) | 49 | 125,839 | 38.94 | 1.29 (0.84-1.98) | 0.236 |  |  | 1.29 (0.84-1.98) | 0.239 |  |
|  | [60,80) | 42 | 125,458 | 33.48 | 1.10 (0.71-1.71) | 0.673 |  |  | 1.10 (0.70-1.71) | 0.685 |  |
|  | ≥80 | 72 | 125,105 | 57.55 | 1.90 (1.28-2.83) | 0.002 | 0.001 |  | 1.89 (1.27-2.82) | 0.002 | 0.001 |
| Ovary |  |  |  |  |  |  |  |  |  |  |  |
|  | <20 | 10 | 125,570 | 7.96 | Ref | - |  |  | Ref | - |  |
|  | [20,40) | 18 | 125,590 | 14.33 | 1.79 (0.82-3.87) | 0.141 |  |  | 1.81 (0.83-3.91) | 0.134 |  |
|  | [40,60) | 25 | 125,692 | 19.89 | 2.49 (1.20-5.19) | 0.015 |  |  | 2.52 (1.21-5.25) | 0.014 |  |
|  | [60,80) | 16 | 126,076 | 12.69 | 1.58 (0.72-3.49) | 0.254 |  |  | 1.60 (0.72-3.52) | 0.246 |  |
|  | ≥80 | 27 | 126,027 | 21.42 | 2.68 (1.29-5.54) | 0.008 | 0.024 |  | 2.70 (1.31-5.59) | 0.007 | 0.023 |
| Prostate |  |  |  |  |  |  |  |  |  |  |  |
|  | <20 | 10 | 89,090 | 11.22 | Ref | - |  |  | Ref | - |  |
|  | [20,40) | 9 | 89,070 | 10.10 | 0.82 (0.33-2.03) | 0.675 |  |  | 0.80 (0.33-1.98) | 0.636 |  |
|  | [40,60) | 14 | 88,634 | 15.80 | 1.37 (0.61-3.09) | 0.445 |  |  | 1.34 (0.60-3.02) | 0.479 |  |
|  | [60,80) | 27 | 88,726 | 30.43 | 2.57 (1.24-5.30) | 0.011 |  |  | 2.52 (1.22-5.20) | 0.013 |  |
|  | ≥80 | 35 | 88,447 | 39.57 | 3.28 (1.62-6.63) | 9.30×10^-04^ | 2.90×10^-06^ |  | 3.18 (1.57-6.44) | 0.001 | 4.00×10^-06^ |

PRS, polygenic risk score; HR, hazard ratio; CI, confidence interval.

^*^ Participants were divided into five parts according to the quintile of PRS. The HRs were estimated for each parts with a Cox regression model compared with participants at low genetic risk (the bottom quintile of PRS).

^†^ Per 100,000 person-years.

^‡^ Adjusted for age, sex (if applicable), region, and the top 10 principal components.

^§^ Adjusted for age, sex (if applicable), region, the top 10 principal components, family history of cancer, and modifiable risk factors.
