# Supplementary material for: Polygenic risk scores for pan-cancer risk prediction in the Chinese population: A population-based cohort study based on the China Kadoorie Biobank
Source: PLoS Med. 2025 Feb 28;22(2):e1004534. doi: 10.1371/journal.pmed.1004534 (PMC11870365; doi:10.1371/journal.pmed.1004534)
Supplement: S7 Table — PRS, polygenic risk score; HR, hazard ratio; CI, confidence interval. (DOCX) [file pmed.1004534.s011.docx]

**S7 Table. Association details of the optimal polygenic risk scores for the nine cancers in the CKB cohort after excluding all participants within the first year after recruitment**

| **Cancer site** | **PRS group ^*^** | **Cases** | **Person-years** | **Incidence rate ^†^** | **Model 1 ^‡^** | | |  | **Model 2 ^§^** | | |
| --- | --- | --- | --- | --- | --- | --- | --- | --- | --- | --- | --- |
|  |  |  |  |  | **HR (95% CI)** | ***P-*value** | ***P*_trend** |  | **HR (95% CI)** | ***P-*value** | ***P*_trend** |
| Esophagus |  |  |  |  |  |  |  |  |  |  |  |
|  | <20 | 70 | 214,836 | 32.58 | Ref | - |  |  | Ref | - |  |
|  | [20,40) | 81 | 214,098 | 37.83 | 1.16 (0.85-1.60) | 0.351 |  |  | 1.14 (0.83-1.57) | 0.421 |  |
|  | [40,60) | 97 | 214,640 | 45.19 | 1.47 (1.08-2.00) | 0.015 |  |  | 1.44 (1.06-1.96) | 0.021 |  |
|  | [60,80) | 88 | 214,236 | 41.08 | 1.36 (0.99-1.86) | 0.055 |  |  | 1.33 (0.97-1.82) | 0.075 |  |
|  | ≥80 | 129 | 214,139 | 60.24 | 2.05 (1.53-2.74) | 1.47×10^-06^ | 8.43×10^-07^ |  | 1.94 (1.45-2.60) | 7.91×10^-06^ | 4.11×10^-06^ |
| Stomach |  |  |  |  |  |  |  |  |  |  |  |
|  | <20 | 90 | 215,327 | 41.80 | Ref | - |  |  | Ref | - |  |
|  | [20,40) | 131 | 214,348 | 61.12 | 1.42 (1.09-1.86) | 0.010 |  |  | 1.42 (1.08-1.85) | 0.011 |  |
|  | [40,60) | 126 | 214,445 | 58.76 | 1.37 (1.04-1.79) | 0.024 |  |  | 1.36 (1.04-1.79) | 0.025 |  |
|  | [60,80) | 156 | 213,982 | 72.90 | 1.64 (1.27-2.13) | 1.92×10^-04^ |  |  | 1.64 (1.27-2.13) | 1.93×10^-04^ |  |
|  | ≥80 | 196 | 213,473 | 91.81 | 2.05 (1.59-2.63) | 2.21×10^-08^ | 1.23×10^-08^ |  | 2.05 (1.60-2.64) | 2.06×10^-08^ | 1.07×10^-08^ |
| Colorectum |  |  |  |  |  |  |  |  |  |  |  |
|  | <20 | 80 | 214,951 | 37.22 | Ref | - |  |  | Ref | - |  |
|  | [20,40) | 118 | 214,114 | 55.11 | 1.50 (1.13-1.99) | 0.005 |  |  | 1.49 (1.12-1.98) | 0.006 |  |
|  | [40,60) | 117 | 214,400 | 54.57 | 1.50 (1.13-1.99) | 0.005 |  |  | 1.49 (1.12-1.99) | 0.006 |  |
|  | [60,80) | 148 | 213,982 | 69.16 | 1.94 (1.48-2.56) | 1.98×10^-06^ |  |  | 1.94 (1.48-2.55) | 2.08×10^-06^ |  |
|  | ≥80 | 246 | 213,176 | 115.40 | 3.27 (2.53-4.22) | 1.12×10^-19^ | 5.17×10^-23^ |  | 3.24 (2.51-4.19) | 2.03×10^-19^ | 8.86×10^-23^ |
| Pancreas |  |  |  |  |  |  |  |  |  |  |  |
|  | <20 | 16 | 215,699 | 7.42 | Ref | - |  |  | Ref | - |  |
|  | [20,40) | 33 | 214,310 | 15.40 | 2.02 (1.11-3.67) | 0.021 |  |  | 2.02 (1.11-3.67) | 0.021 |  |
|  | [40,60) | 27 | 214,199 | 12.61 | 1.68 (0.91-3.12) | 0.100 |  |  | 1.68 (0.90-3.11) | 0.102 |  |
|  | [60,80) | 41 | 214,300 | 19.13 | 2.56 (1.44-4.56) | 0.001 |  |  | 2.55 (1.43-4.55) | 0.001 |  |
|  | ≥80 | 48 | 214,324 | 22.40 | 2.97 (1.69-5.23) | 1.64×10^-04^ | 8.71×10^-05^ |  | 2.94 (1.67-5.17) | 1.92×10^-04^ | 1.06×10^-04^ |
| Lung |  |  |  |  |  |  |  |  |  |  |  |
|  | <20 | 233 | 214,304 | 108.72 | Ref | - |  |  | Ref | - |  |
|  | [20,40) | 245 | 214,503 | 114.22 | 1.05 (0.87-1.25) | 0.622 |  |  | 1.05 (0.88-1.25) | 0.610 |  |
|  | [40,60) | 268 | 214,179 | 125.13 | 1.13 (0.95-1.35) | 0.173 |  |  | 1.13 (0.95-1.35) | 0.162 |  |
|  | [60,80) | 347 | 214,253 | 161.96 | 1.48 (1.25-1.75) | 3.56×10^-06^ |  |  | 1.48 (1.25-1.74) | 4.16×10^-06^ |  |
|  | ≥80 | 373 | 213,543 | 174.67 | 1.62 (1.38-1.91) | 7.08×10^-09^ | 6.79×10^-13^ |  | 1.63 (1.38-1.92) | 5.42×10^-09^ | 6.01×10^-13^ |
| Breast |  |  |  |  |  |  |  |  |  |  |  |
|  | <20 | 53 | 125,533 | 42.22 | Ref | - |  |  | Ref | - |  |
|  | [20,40) | 67 | 125,461 | 53.40 | 1.28 (0.90-1.84) | 0.173 |  |  | 1.29 (0.90-1.85) | 0.167 |  |
|  | [40,60) | 86 | 125,732 | 68.40 | 1.63 (1.16-2.30) | 0.005 |  |  | 1.62 (1.15-2.28) | 0.006 |  |
|  | [60,80) | 120 | 125,292 | 95.78 | 2.27 (1.65-3.14) | 6.34×10^-07^ |  |  | 2.28 (1.65-3.16) | 5.55×10^-07^ |  |
|  | ≥80 | 136 | 124,983 | 108.81 | 2.55 (1.86-3.50) | 7.48×10^-09^ | 2.13×10^-12^ |  | 2.56 (1.87-3.52) | 6.11×10^-09^ | 1.59×10^-12^ |
| Cervix |  |  |  |  |  |  |  |  |  |  |  |
|  | <20 | 33 | 126,093 | 26.17 | Ref | - |  |  | Ref | - |  |
|  | [20,40) | 36 | 125,732 | 28.63 | 1.08 (0.67-1.73) | 0.745 |  |  | 1.08 (0.67-1.74) | 0.743 |  |
|  | [40,60) | 47 | 125,779 | 37.37 | 1.39 (0.89-2.17) | 0.145 |  |  | 1.39 (0.89-2.17) | 0.147 |  |
|  | [60,80) | 42 | 125,370 | 33.50 | 1.23 (0.78-1.95) | 0.369 |  |  | 1.23 (0.78-1.94) | 0.379 |  |
|  | ≥80 | 68 | 125,123 | 54.35 | 2.02 (1.33-3.06) | 9.93×10^-04^ | 6.70×10^-04^ |  | 2.00 (1.32-3.03) | 0.001 | 7.92×10^-04^ |
| Ovary |  |  |  |  |  |  |  |  |  |  |  |
|  | <20 | 10 | 125,657 | 7.96 | Ref | - |  |  | Ref | - |  |
|  | [20,40) | 18 | 125,656 | 14.32 | 1.79 (0.83-3.87) | 0.141 |  |  | 1.81 (0.83-3.92) | 0.134 |  |
|  | [40,60) | 25 | 125,612 | 19.90 | 2.50 (1.20-5.20) | 0.014 |  |  | 2.53 (1.21-5.26) | 0.013 |  |
|  | [60,80) | 15 | 125,962 | 11.91 | 1.49 (0.67-3.32) | 0.330 |  |  | 1.50 (0.67-3.34) | 0.320 |  |
|  | ≥80 | 26 | 125,900 | 20.65 | 2.59 (1.25-5.38) | 0.011 | 0.037 |  | 2.61 (1.26-5.43) | 0.010 | 0.036 |
| Prostate |  |  |  |  |  |  |  |  |  |  |  |
|  | <20 | 9 | 89,035 | 10.11 | Ref | - |  |  | Ref | - |  |
|  | [20,40) | 8 | 89,014 | 8.99 | 0.81 (0.31-2.10) | 0.666 |  |  | 0.79 (0.31-2.06) | 0.634 |  |
|  | [40,60) | 14 | 88,689 | 15.79 | 1.51 (0.66-3.50) | 0.332 |  |  | 1.48 (0.64-3.43) | 0.357 |  |
|  | [60,80) | 25 | 88,623 | 28.21 | 2.64 (1.23-5.65) | 0.013 |  |  | 2.59 (1.21-5.55) | 0.015 |  |
|  | ≥80 | 35 | 88,328 | 39.62 | 3.66 (1.76-7.62) | 5.25×10^-04^ | 1.19×10^-06^ |  | 3.55 (1.71-7.41) | 7.13×10^-04^ | 1.65×10^-06^ |

PRS, polygenic risk score; HR, hazard ratio; CI, confidence interval.

^*^ Participants were divided into five parts according to the quintile of PRS after excluding all participants within the first year after recruitment. The HRs were estimated for each parts with a Cox regression model compared with participants at low genetic risk (the bottom quintile of PRS).

^†^ Per 100,000 person-years.

^‡^ Adjusted for age, sex (if applicable), region, and the top 10 principal components.

^§^ Adjusted for age, sex (if applicable), region, the top 10 principal components, family history of cancer, and modifiable risk factors.
