# Supplementary material for: Polygenic risk scores for pan-cancer risk prediction in the Chinese population: A population-based cohort study based on the China Kadoorie Biobank
Source: PLoS Med. 2025 Feb 28;22(2):e1004534. doi: 10.1371/journal.pmed.1004534 (PMC11870365; doi:10.1371/journal.pmed.1004534)
Supplement: S8 Table — PRS, polygenic risk score; HR, hazard ratio; CI, confidence interval. (DOCX) [file pmed.1004534.s012.docx]

**S8 Table. Association details of the optimal polygenic risk scores for the nine cancers in the CKB cohort after only including the first primary cancer**

| **Cancer site** | **PRS group ^*^** | **Cases** | **Person-years** | **Incidence rate ^†^** | **Model 1 ^‡^** | | |  | **Model 2 ^§^** | | |
| --- | --- | --- | --- | --- | --- | --- | --- | --- | --- | --- | --- |
|  |  |  |  |  | **HR (95% CI)** | ***P-*value** | ***P*_trend** |  | **HR (95% CI)** | ***P-*value** | ***P*_trend** |
| Esophagus |  |  |  |  |  |  |  |  |  |  |  |
|  | <20 | 70 | 214,893 | 32.57 | Ref | - |  |  | Ref | - |  |
|  | [20,40) | 76 | 214,162 | 35.49 | 1.10 (0.79-1.52) | 0.581 |  |  | 1.08 (0.78-1.49) | 0.661 |  |
|  | [40,60) | 88 | 214,560 | 41.01 | 1.33 (0.97-1.82) | 0.075 |  |  | 1.31 (0.96-1.79) | 0.093 |  |
|  | [60,80) | 87 | 214,278 | 40.60 | 1.34 (0.98-1.84) | 0.065 |  |  | 1.32 (0.96-1.81) | 0.085 |  |
|  | ≥80 | 122 | 214,107 | 56.98 | 1.93 (1.44-2.59) | 1.15×10^-05^ | 3.25×10^-06^ |  | 1.84 (1.37-2.47) | 5.09×10^-05^ | 1.35×10^-05^ |
| Stomach |  |  |  |  |  |  |  |  |  |  |  |
|  | <20 | 74 | 215,277 | 34.37 | Ref | - |  |  | Ref | - |  |
|  | [20,40) | 123 | 214,211 | 57.42 | 1.62 (1.21-2.16) | 0.001 |  |  | 1.62 (1.21-2.16) | 0.001 |  |
|  | [40,60) | 109 | 214,465 | 50.82 | 1.43 (1.07-1.93) | 0.017 |  |  | 1.43 (1.06-1.92) | 0.018 |  |
|  | [60,80) | 145 | 213,477 | 67.92 | 1.85 (1.40-2.46) | 1.71×10^-05^ |  |  | 1.86 (1.40-2.46) | 1.63×10^-05^ |  |
|  | ≥80 | 180 | 213,841 | 84.17 | 2.28 (1.74-2.99) | 3.02×10^-09^ | 2.88×10^-09^ |  | 2.29 (1.74-3.00) | 2.69×10^-09^ | 2.36×10^-09^ |
| Colorectum |  |  |  |  |  |  |  |  |  |  |  |
|  | <20 | 75 | 215,220 | 34.85 | Ref | - |  |  | Ref | - |  |
|  | [20,40) | 111 | 214,156 | 51.83 | 1.49 (1.11-2.00) | 0.007 |  |  | 1.49 (1.11-2.00) | 0.008 |  |
|  | [40,60) | 110 | 214,550 | 51.27 | 1.49 (1.11-2.01) | 0.007 |  |  | 1.49 (1.11-2.00) | 0.008 |  |
|  | [60,80) | 144 | 213,860 | 67.33 | 2.00 (1.51-2.66) | 1.21×10^-06^ |  |  | 2.00 (1.51-2.65) | 1.32×10^-06^ |  |
|  | ≥80 | 240 | 212,891 | 112.73 | 3.37 (2.59-4.39) | 1.16×10^-19^ | 1.16×10^-23^ |  | 3.35 (2.58-4.36) | 1.95×10^-19^ | 1.95×10^-23^ |
| Pancreas |  |  |  |  |  |  |  |  |  |  |  |
|  | <20 | 14 | 215,736 | 6.49 | Ref | - |  |  | Ref | - |  |
|  | [20,40) | 31 | 214,280 | 14.47 | 2.16 (1.15-4.06) | 0.017 |  |  | 2.16 (1.15-4.07) | 0.017 |  |
|  | [40,60) | 21 | 214,166 | 9.81 | 1.49 (0.76-2.93) | 0.248 |  |  | 1.48 (0.75-2.92) | 0.252 |  |
|  | [60,80) | 32 | 214,626 | 14.91 | 2.27 (1.21-4.26) | 0.010 |  |  | 2.26 (1.21-4.23) | 0.011 |  |
|  | ≥80 | 44 | 214,276 | 20.53 | 3.11 (1.70-5.68) | 2.18×10^-04^ | 3.42×10^-04^ |  | 3.09 (1.69-5.63) | 2.43×10^-04^ | 3.97×10^-04^ |
| Lung |  |  |  |  |  |  |  |  |  |  |  |
|  | <20 | 224 | 214,132 | 104.61 | Ref | - |  |  | Ref | - |  |
|  | [20,40) | 232 | 214,346 | 108.24 | 1.03 (0.86-1.24) | 0.757 |  |  | 1.03 (0.86-1.24) | 0.745 |  |
|  | [40,60) | 243 | 214,196 | 113.45 | 1.06 (0.89-1.28) | 0.504 |  |  | 1.07 (0.89-1.28) | 0.482 |  |
|  | [60,80) | 317 | 214,032 | 148.11 | 1.41 (1.19-1.67) | 8.85×10^-05^ |  |  | 1.41 (1.18-1.67) | 9.89×10^-05^ |  |
|  | ≥80 | 347 | 213,371 | 162.63 | 1.57 (1.33-1.86) | 1.33×10^-07^ | 8.42×10^-11^ |  | 1.58 (1.33-1.87) | 1.07×10^-07^ | 7.61×10^-11^ |
| Breast |  |  |  |  |  |  |  |  |  |  |  |
|  | <20 | 49 | 125,650 | 39.00 | Ref | - |  |  | Ref | - |  |
|  | [20,40) | 66 | 125,472 | 52.60 | 1.37 (0.95-1.98) | 0.095 |  |  | 1.37 (0.95-1.99) | 0.093 |  |
|  | [40,60) | 81 | 125,729 | 64.42 | 1.66 (1.17-2.37) | 0.005 |  |  | 1.65 (1.16-2.36) | 0.006 |  |
|  | [60,80) | 116 | 125,034 | 92.77 | 2.39 (1.71-3.33) | 3.33×10^-07^ |  |  | 2.38 (1.71-3.33) | 3.43×10^-07^ |  |
|  | ≥80 | 127 | 124,978 | 101.62 | 2.58 (1.86-3.59) | 1.74×10^-08^ | 1.07×10^-11^ |  | 2.59 (1.86-3.60) | 1.61×10^-08^ | 9.93×10^-12^ |
| Cervix |  |  |  |  |  |  |  |  |  |  |  |
|  | <20 | 34 | 126,105 | 26.96 | Ref | - |  |  | Ref | - |  |
|  | [20,40) | 33 | 125,667 | 26.26 | 0.96 (0.60-1.55) | 0.875 |  |  | 0.96 (0.60-1.56) | 0.880 |  |
|  | [40,60) | 43 | 125,835 | 34.17 | 1.24 (0.79-1.94) | 0.355 |  |  | 1.23 (0.79-1.93) | 0.362 |  |
|  | [60,80) | 36 | 125,405 | 28.71 | 1.02 (0.64-1.64) | 0.921 |  |  | 1.02 (0.64-1.63) | 0.936 |  |
|  | ≥80 | 64 | 125,053 | 51.18 | 1.84 (1.21-2.79) | 0.004 | 0.004 |  | 1.83 (1.20-2.77) | 0.005 | 0.004 |
| Ovary |  |  |  |  |  |  |  |  |  |  |  |
|  | <20 | 8 | 125,552 | 6.37 | Ref | - |  |  | Ref | - |  |
|  | [20,40) | 17 | 125,549 | 13.54 | 2.12 (0.91-4.92) | 0.080 |  |  | 2.15 (0.93-4.99) | 0.074 |  |
|  | [40,60) | 20 | 125,682 | 15.91 | 2.50 (1.10-5.67) | 0.029 |  |  | 2.53 (1.12-5.76) | 0.026 |  |
|  | [60,80) | 12 | 126,046 | 9.52 | 1.49 (0.61-3.66) | 0.380 |  |  | 1.52 (0.62-3.71) | 0.363 |  |
|  | ≥80 | 23 | 126,000 | 18.25 | 2.87 (1.28-6.43) | 0.010 | 0.051 |  | 2.91 (1.30-6.51) | 0.010 | 0.048 |
| Prostate |  |  |  |  |  |  |  |  |  |  |  |
|  | <20 | 10 | 89,090 | 11.22 | Ref | - |  |  | Ref | - |  |
|  | [20,40) | 9 | 89,025 | 10.11 | 0.83 (0.34-2.04) | 0.682 |  |  | 0.80 (0.33-1.98) | 0.636 |  |
|  | [40,60) | 13 | 88,631 | 14.67 | 1.27 (0.56-2.90) | 0.571 |  |  | 1.23 (0.54-2.80) | 0.626 |  |
|  | [60,80) | 22 | 88,721 | 24.80 | 2.10 (0.99-4.43) | 0.052 |  |  | 2.05 (0.97-4.34) | 0.059 |  |
|  | ≥80 | 32 | 88,419 | 36.19 | 2.98 (1.46-6.06) | 0.003 | 4.02×10^-05^ |  | 2.86 (1.41-5.84) | 0.004 | 5.55×10^-05^ |

PRS, polygenic risk score; HR, hazard ratio; CI, confidence interval.

^*^ Participants were divided into five parts according to the quintile of PRS after only including the first primary cancer. The HRs were estimated for each parts with a Cox regression model compared with participants at low genetic risk (the bottom quintile of PRS).

^†^ Per 100,000 person-years.

^‡^ Adjusted for age, sex (if applicable), region, and the top 10 principal components.

^§^ Adjusted for age, sex (if applicable), region, the top 10 principal components, family history of cancer, and modifiable risk factors.
