# Supplementary material for: Polygenic risk scores for pan-cancer risk prediction in the Chinese population: A population-based cohort study based on the China Kadoorie Biobank
Source: PLoS Med. 2025 Feb 28;22(2):e1004534. doi: 10.1371/journal.pmed.1004534 (PMC11870365; doi:10.1371/journal.pmed.1004534)
Supplement: S9 Table — AUC, area under the curve; PRS, polygenic risk score; CI, confidence interval. (DOCX) [file pmed.1004534.s013.docx]

**S9 Table. The 10-year AUC, sensitivity, and specificity of the nine optimal polygenic risk scores**

| **Cancer site** | **Cases** | **Incidence rate (per 100,000 person-years)** | **AUC (95% CI)** | **Sensitivity (95% CI)** | **Specificity (95% CI)** |
| --- | --- | --- | --- | --- | --- |
| Esophagus | 499 | 46.53 | 0.556 (0.529-0.583) | 0.664 (0.613-0.704) | 0.430 (0.373-0.461) |
| Stomach | 745 | 69.49 | 0.569 (0.547-0.591) | 0.500 (0.456-0.536) | 0.612 (0.558-0.638) |
| Colorectum | 740 | 69.09 | 0.610 (0.586-0.633) | 0.459 (0.418-0.496) | 0.719 (0.671-0.750) |
| Pancreas | 170 | 15.84 | 0.597 (0.550-0.643) | 0.703 (0.625-0.773) | 0.494 (0.343-0.550) |
| Lung | 1,540 | 143.76 | 0.550 (0.534-0.566) | 0.489 (0.460-0.514) | 0.598 (0.560-0.623) |
| Breast | 486 | 77.49 | 0.587 (0.559-0.614) | 0.591 (0.541-0.635) | 0.560 (0.492-0.604) |
| Cervix | 237 | 37.72 | 0.573 (0.533-0.614) | 0.451 (0.383-0.519) | 0.686 (0.594-0.735) |
| Ovary | 96 | 15.26 | 0.591 (0.532-0.649) | 0.688 (0.558-0.779) | 0.500 (0.348-0.555) |
| Prostate | 95 | 21.40 | 0.650 (0.588-0.713) | 0.571 (0.442-0.675) | 0.700 (0.579-0.764) |

AUC, area under the curve; PRS, polygenic risk score; CI, confidence interval.
