# Supplementary material for: Polygenic risk scores for pan-cancer risk prediction in the Chinese population: A population-based cohort study based on the China Kadoorie Biobank
Source: PLoS Med. 2025 Feb 28;22(2):e1004534. doi: 10.1371/journal.pmed.1004534 (PMC11870365; doi:10.1371/journal.pmed.1004534)
Supplement: S10 Table — HR, hazard ratio; CI, confidence interval. (DOCX) [file pmed.1004534.s014.docx]

**S10 Table. Associations of genetic risk with different age onsets of each cancer type in the CKB cohort**

| **Cancer site** | **Age group of onset** | **Case** | **Incidence rate (per 100,000 person-years)** | **Low ^*^** | **Intermediate ^*^** | | **High ^*^** | |
| --- | --- | --- | --- | --- | --- | --- | --- | --- |
|  |  |  |  | **HR** | **HR (95% CI)** | ***P*_het** | **HR (95% CI)** | ***P*_het** |
| Esophagus | <68 | 258 | 30.99 | 1.00 | 1.20 (0.86-1.70) |  | 1.74 (1.18-2.56) |  |
|  | ≥68 | 241 | 100.37 | 1.00 | 1.36 (0.94-1.99) | 0.628 | 2.28 (1.51-3.42) | 0.347 |
| Stomach | <68 | 386 | 46.37 | 1.00 | 1.85 (1.32-2.60) |  | 2.75 (1.92-3.95) |  |
|  | ≥68 | 359 | 149.62 | 1.00 | 1.24 (0.92-1.67) | 0.082 | 1.53 (1.09-2.15) | 0.020 |
| Colorectum | <68 | 381 | 45.79 | 1.00 | 1.59 (1.14-2.22) |  | 3.36 (2.37-4.75) |  |
|  | ≥68 | 359 | 150.00 | 1.00 | 1.68 (1.20-2.35) | 0.820 | 3.10 (2.16-4.44) | 0.753 |
| Pancreas | <70 | 84 | 9.54 | 1.00 | 1.56 (0.81-2.99) |  | 2.01 (0.97-4.15) |  |
|  | ≥70 | 86 | 44.59 | 1.00 | 2.11 (1.00-4.44) | 0.550 | 3.15 (1.42-6.96) | 0.414 |
| Lung | <68 | 690 | 82.92 | 1.00 | 1.25 (1.01-1.55) |  | 1.83 (1.44-2.32) |  |
|  | ≥68 | 850 | 355.01 | 1.00 | 1.14 (0.95-1.37) | 0.522 | 1.39 (1.12-1.73) | 0.095 |
| Breast | <53 | 148 | 65.99 | 1.00 | 1.85 (1.08-3.15) |  | 2.74 (1.55-4.86) |  |
|  | ≥53 | 338 | 83.85 | 1.00 | 1.80 (1.27-2.54) | 0.933 | 2.53 (1.74-3.68) | 0.819 |
| Cervix | <53 | 85 | 37.87 | 1.00 | 1.28 (0.64-2.57) |  | 3.27 (1.61-6.61) |  |
|  | ≥53 | 152 | 37.62 | 1.00 | 1.06 (0.69-1.64) | 0.652 | 1.35 (0.82-2.21) | 0.044 |
| Ovary | <53 | 28 | 12.46 | 1.00 | 1.09 (0.40-2.97) |  | 1.43 (0.45-4.53) |  |
|  | ≥53 | 68 | 16.81 | 1.00 | 2.86 (1.13-7.22) | 0.166 | 3.97 (1.49-10.59) | 0.186 |
| Prostate | <73 | 34 | 8.87 | 1.00 | 1.61 (0.55-4.75) |  | 2.84 (0.90-8.91) |  |
|  | ≥73 | 61 | 100.11 | 1.00 | 1.51 (0.63-3.63) | 0.928 | 3.37 (1.37-8.26) | 0.818 |

HR, hazard ratio; CI, confidence interval.

^*^ Low polygenic risk score (PRS) corresponds to the bottom quintile, medium PRS is defined as quintile 2-4, and high PRS includes individuals in the top quintile in the CKB cohort.
