# Supplementary material for: Polygenic risk scores for pan-cancer risk prediction in the Chinese population: A population-based cohort study based on the China Kadoorie Biobank
Source: PLoS Med. 2025 Feb 28;22(2):e1004534. doi: 10.1371/journal.pmed.1004534 (PMC11870365; doi:10.1371/journal.pmed.1004534)
Supplement: S11 Table — (DOCX) [file pmed.1004534.s015.docx]

**S11 Table. Proportion of participants at high genetic risk of site-specific cancers**

| **No. of high genetic risk cancers** | **Men (%)** | **Women (%)** | **All (%)** |
| --- | --- | --- | --- |
| 0 | 11,281 (26.32) | 9,863 (17.20) | 21,144 (21.10) |
| 1 | 16,588 (38.70) | 19,273 (33.60) | 35,861 (35.78) |
| 2 | 10,631 (24.80) | 16,616 (28.97) | 27,247 (27.19) |
| 3 | 3,585 (8.36) | 8,275 (14.43) | 11,860 (11.83) |
| 4 | 697 (1.63) | 2,686 (4.68) | 3,383 (3.38) |
| 5 | 74 (0.17) | 543 (0.95) | 617 (0.62) |
| 6 | 4 (0.01) | 96 (0.17) | 100 (0.10) |
| 7 | 0 (0.00) | 7 (0.01) | 7 (0.01) |
| Total | 42,860 (100.00) | 57,359 (100.00) | 100,219 (100.00) |
