# Supplementary material for: Polygenic risk scores for pan-cancer risk prediction in the Chinese population: A population-based cohort study based on the China Kadoorie Biobank
Source: PLoS Med. 2025 Feb 28;22(2):e1004534. doi: 10.1371/journal.pmed.1004534 (PMC11870365; doi:10.1371/journal.pmed.1004534)
Supplement: S12 Table — PRS, polygenic risk score; HR, hazard ratio; CI, confidence interval. (DOCX) [file pmed.1004534.s016.docx]

**S12 Table. Association details between cancer-specific polygenic risk scores and other cancer outcomes**

|  |  | **Model 1 ^*^** | | |  | **Model 2** ^†^ | | |
| --- | --- | --- | --- | --- | --- | --- | --- | --- |
| **PRS** | **Outcome** | **HR (95% CI)** | ***P*-value** | ***P*-value (FDR)** |  | **HR (95% CI)** | ***P*-value** | ***P*-value (FDR)** |
| Esophagus | Esophagus | 1.28 (1.17-1.39) | 2.45×10^-08^ | 2.21×10^-07^ |  | - | - | - |
| Esophagus | Stomach | 1.08 (1.01-1.16) | 0.033 | 0.147 |  | 1.04 (0.97-1.12) | 0.308 | 0.694 |
| Esophagus | Colorectum | 1.06 (0.99-1.14) | 0.098 | 0.293 |  | 1.06 (0.99-1.14) | 0.087 | 0.391 |
| Esophagus | Pancreas | 1.01 (0.87-1.18) | 0.882 | 0.900 |  | 1.02 (0.88-1.18) | 0.804 | 0.891 |
| Esophagus | Lung | 1.00 (0.95-1.05) | 0.900 | 0.900 |  | 1.00 (0.95-1.06) | 0.884 | 0.891 |
| Esophagus | Breast | 0.97 (0.89-1.06) | 0.503 | 0.900 |  | 0.95 (0.87-1.04) | 0.256 | 0.694 |
| Esophagus | Cervix | 1.02 (0.89-1.15) | 0.814 | 0.900 |  | 1.01 (0.89-1.15) | 0.891 | 0.891 |
| Esophagus | Ovary | 1.07 (0.87-1.30) | 0.536 | 0.900 |  | 1.06 (0.87-1.30) | 0.538 | 0.891 |
| Esophagus | Prostate | 1.03 (0.84-1.27) | 0.744 | 0.900 |  | 1.05 (0.86-1.29) | 0.615 | 0.891 |
| Stomach | Esophagus | 1.11 (1.01-1.22) | 0.024 | 0.098 |  | 1.06 (0.97-1.16) | 0.214 | 0.482 |
| Stomach | Stomach | 1.27 (1.18-1.37) | 3.09×10^-10^ | 2.78×10^-09^ |  | - | - | - |
| Stomach | Colorectum | 0.93 (0.87-1.00) | 0.051 | 0.114 |  | 0.92 (0.86-0.99) | 0.030 | 0.099 |
| Stomach | Pancreas | 0.96 (0.83-1.12) | 0.622 | 0.699 |  | 0.96 (0.83-1.12) | 0.620 | 0.698 |
| Stomach | Lung | 1.03 (0.98-1.08) | 0.318 | 0.522 |  | 1.02 (0.97-1.07) | 0.406 | 0.610 |
| Stomach | Breast | 1.00 (0.91-1.09) | 0.929 | 0.929 |  | 1.00 (0.91-1.09) | 0.991 | 0.991 |
| Stomach | Cervix | 0.96 (0.84-1.09) | 0.519 | 0.667 |  | 0.96 (0.84-1.09) | 0.489 | 0.629 |
| Stomach | Ovary | 1.25 (1.02-1.54) | 0.033 | 0.098 |  | 1.25 (1.02-1.54) | 0.033 | 0.099 |
| Stomach | Prostate | 1.10 (0.90-1.35) | 0.348 | 0.522 |  | 1.10 (0.90-1.35) | 0.361 | 0.610 |
| Colorectum | Esophagus | 1.01 (0.93-1.11) | 0.782 | 0.880 |  | 1.01 (0.93-1.11) | 0.761 | 0.880 |
| Colorectum | Stomach | 1.15 (1.06-1.23) | 2.75×10^-04^ | 0.001 |  | 1.14 (1.06-1.23) | 4.47×10^-04^ | 0.002 |
| Colorectum | Colorectum | 1.54 (1.44-1.66) | 3.06×10^-31^ | 2.76×10^-30^ |  | - | - | - |
| Colorectum | Pancreas | 1.16 (1.00-1.36) | 0.053 | 0.102 |  | 1.16 (1.00-1.35) | 0.057 | 0.103 |
| Colorectum | Lung | 0.99 (0.94-1.04) | 0.588 | 0.790 |  | 0.99 (0.94-1.04) | 0.783 | 0.880 |
| Colorectum | Breast | 1.11 (1.01-1.21) | 0.029 | 0.087 |  | 1.10 (1.01-1.21) | 0.033 | 0.100 |
| Colorectum | Cervix | 0.97 (0.85-1.10) | 0.615 | 0.790 |  | 0.97 (0.85-1.10) | 0.612 | 0.880 |
| Colorectum | Ovary | 1.01 (0.82-1.24) | 0.940 | 0.940 |  | 1.01 (0.82-1.24) | 0.939 | 0.939 |
| Colorectum | Prostate | 1.22 (0.99-1.49) | 0.057 | 0.102 |  | 1.22 (0.99-1.50) | 0.057 | 0.103 |
| Pancreas | Esophagus | 1.00 (0.92-1.09) | 0.954 | 0.954 |  | 1.01 (0.92-1.10) | 0.850 | 0.850 |
| Pancreas | Stomach | 1.06 (0.99-1.14) | 0.106 | 0.317 |  | 1.06 (0.99-1.14) | 0.103 | 0.310 |
| Pancreas | Colorectum | 1.01 (0.94-1.09) | 0.689 | 0.828 |  | 1.01 (0.94-1.09) | 0.782 | 0.850 |
| Pancreas | Pancreas | 1.28 (1.11-1.49) | 9.41×10^-04^ | 0.008 |  | - | - | - |
| Pancreas | Lung | 0.98 (0.93-1.03) | 0.483 | 0.828 |  | 1.02 (0.97-1.07) | 0.527 | 0.827 |
| Pancreas | Breast | 1.14 (1.05-1.25) | 0.003 | 0.015 |  | 1.13 (1.04-1.24) | 0.006 | 0.028 |
| Pancreas | Cervix | 0.96 (0.85-1.09) | 0.559 | 0.828 |  | 0.96 (0.85-1.09) | 0.551 | 0.827 |
| Pancreas | Ovary | 0.92 (0.75-1.12) | 0.401 | 0.828 |  | 0.92 (0.75-1.12) | 0.392 | 0.827 |
| Pancreas | Prostate | 1.04 (0.84-1.27) | 0.736 | 0.828 |  | 1.03 (0.84-1.26) | 0.772 | 0.850 |
| Lung | Esophagus | 0.92 (0.84-1.01) | 0.078 | 0.234 |  | 0.92 (0.84-1.01) | 0.076 | 0.229 |
| Lung | Stomach | 0.99 (0.92-1.06) | 0.753 | 0.847 |  | 0.98 (0.91-1.06) | 0.619 | 0.763 |
| Lung | Colorectum | 1.02 (0.95-1.10) | 0.558 | 0.837 |  | 1.04 (0.96-1.12) | 0.323 | 0.728 |
| Lung | Pancreas | 0.91 (0.78-1.06) | 0.231 | 0.519 |  | 0.95 (0.81-1.11) | 0.516 | 0.763 |
| Lung | Lung | 1.22 (1.16-1.28) | 1.85×10^-14^ | 1.66×10^-13^ |  | - | - | - |
| Lung | Breast | 0.98 (0.90-1.07) | 0.653 | 0.840 |  | 0.98 (0.90-1.07) | 0.679 | 0.763 |
| Lung | Cervix | 1.16 (1.02-1.32) | 0.022 | 0.099 |  | 1.16 (1.02-1.32) | 0.019 | 0.087 |
| Lung | Ovary | 1.00 (0.82-1.22) | 0.981 | 0.981 |  | 1.00 (0.82-1.22) | 0.991 | 0.991 |
| Lung | Prostate | 0.94 (0.76-1.15) | 0.524 | 0.837 |  | 0.93 (0.76-1.15) | 0.511 | 0.763 |
| Breast | Esophagus | 1.09 (0.93-1.27) | 0.307 | 0.819 |  | 1.07 (0.92-1.26) | 0.373 | 0.910 |
| Breast | Stomach | 1.14 (1.01-1.28) | 0.036 | 0.144 |  | 1.14 (1.01-1.28) | 0.034 | 0.138 |
| Breast | Colorectum | 1.04 (0.94-1.15) | 0.497 | 0.974 |  | 1.03 (0.93-1.14) | 0.534 | 0.910 |
| Breast | Pancreas | 1.03 (0.83-1.28) | 0.771 | 0.974 |  | 1.02 (0.83-1.27) | 0.828 | 0.974 |
| Breast | Lung | 1.02 (0.94-1.11) | 0.610 | 0.974 |  | 1.02 (0.94-1.11) | 0.569 | 0.910 |
| Breast | Breast | 1.41 (1.29-1.54) | 1.75×10^-14^ | 1.40×10^-13^ |  | - | - | - |
| Breast | Cervix | 1.00 (0.88-1.14) | 0.974 | 0.974 |  | 1.00 (0.88-1.14) | 0.978 | 0.978 |
| Breast | Ovary | 0.99 (0.81-1.21) | 0.893 | 0.974 |  | 0.98 (0.80-1.20) | 0.852 | 0.974 |
| Cervix | Esophagus | 1.07 (0.92-1.25) | 0.377 | 0.754 |  | 1.06 (0.91-1.24) | 0.428 | 0.855 |
| Cervix | Stomach | 1.02 (0.90-1.15) | 0.782 | 0.833 |  | 1.01 (0.90-1.14) | 0.867 | 0.867 |
| Cervix | Colorectum | 0.99 (0.89-1.10) | 0.826 | 0.833 |  | 0.99 (0.89-1.10) | 0.817 | 0.867 |
| Cervix | Pancreas | 0.86 (0.69-1.07) | 0.185 | 0.493 |  | 0.86 (0.69-1.07) | 0.185 | 0.493 |
| Cervix | Lung | 1.01 (0.93-1.09) | 0.833 | 0.833 |  | 1.01 (0.93-1.10) | 0.754 | 0.867 |
| Cervix | Breast | 0.94 (0.86-1.03) | 0.162 | 0.493 |  | 0.94 (0.86-1.02) | 0.155 | 0.493 |
| Cervix | Cervix | 1.20 (1.06-1.36) | 0.004 | 0.031 |  | - | - | - |
| Cervix | Ovary | 1.05 (0.86-1.28) | 0.635 | 0.833 |  | 1.05 (0.86-1.28) | 0.631 | 0.867 |
| Ovary | Esophagus | 1.06 (0.90-1.23) | 0.499 | 0.952 |  | 1.05 (0.90-1.23) | 0.508 | 0.996 |
| Ovary | Stomach | 1.02 (0.91-1.15) | 0.719 | 0.952 |  | 1.02 (0.91-1.15) | 0.738 | 0.996 |
| Ovary | Colorectum | 1.03 (0.93-1.14) | 0.611 | 0.952 |  | 1.03 (0.93-1.14) | 0.574 | 0.996 |
| Ovary | Pancreas | 1.03 (0.83-1.28) | 0.779 | 0.952 |  | 1.03 (0.83-1.28) | 0.780 | 0.996 |
| Ovary | Lung | 1.00 (0.92-1.08) | 0.952 | 0.952 |  | 0.99 (0.92-1.08) | 0.892 | 0.996 |
| Ovary | Breast | 1.01 (0.92-1.10) | 0.877 | 0.952 |  | 1.00 (0.91-1.09) | 0.996 | 0.996 |
| Ovary | Cervix | 1.13 (0.99-1.28) | 0.064 | 0.255 |  | 1.13 (0.99-1.28) | 0.063 | 0.252 |
| Ovary | Ovary | 1.25 (1.02-1.53) | 0.031 | 0.252 |  | - | - | - |
| Prostate | Esophagus | 1.00 (0.90-1.11) | 0.956 | 0.956 |  | 1.01 (0.91-1.12) | 0.914 | 0.914 |
| Prostate | Stomach | 0.98 (0.89-1.07) | 0.583 | 0.898 |  | 0.97 (0.89-1.07) | 0.573 | 0.859 |
| Prostate | Colorectum | 1.18 (1.06-1.31) | 0.002 | 0.005 |  | 1.17 (1.06-1.30) | 0.002 | 0.006 |
| Prostate | Pancreas | 0.98 (0.80-1.22) | 0.888 | 0.956 |  | 0.98 (0.80-1.21) | 0.867 | 0.914 |
| Prostate | Lung | 0.98 (0.92-1.05) | 0.599 | 0.898 |  | 0.98 (0.92-1.04) | 0.521 | 0.859 |
| Prostate | Prostate | 1.76 (1.44-2.16) | 3.79×10^-08^ | 2.27×10^-07^ |  | - | - | - |

PRS, polygenic risk score; HR, hazard ratio; CI, confidence interval.

^*^ Model 1: Adjusted for age, sex (if applicable), region, and the top 10 principal components.

^†^ Model 2: Adjusted for age, sex (if applicable), region, the top 10 principal components, and the corresponding site-specific PRSs.
