# Supplementary material for: Polygenic risk scores for pan-cancer risk prediction in the Chinese population: A population-based cohort study based on the China Kadoorie Biobank
Source: PLoS Med. 2025 Feb 28;22(2):e1004534. doi: 10.1371/journal.pmed.1004534 (PMC11870365; doi:10.1371/journal.pmed.1004534)
Supplement: S13 Table — HR, hazard ratio; CI, confidence interval; BMI, body mass index. (DOCX) [file pmed.1004534.s017.docx]

**S13 Table. Association details for the modifiable risk factors with each cancer type in the CKB cohort**

| **Cancer site** | **Risk factors** | **Model 1 ^*^** | |  | **Model 2 ^†^** | |
| --- | --- | --- | --- | --- | --- | --- |
|  |  | **HR (95% CI)** | ***P-*value** |  | **HR (95% CI)** | ***P-*value** |
| Head and neck |  |  |  |  |  |  |
|  | Pack-years of smoking (<30) | 1.56 (1.06-2.31) | 0.025 |  | 1.40 (0.85-2.31) | 0.183 |
|  | Pack-years of smoking (≥30) | 1.86 (1.17-2.94) | 0.008 |  | 1.19 (0.67-2.08) | 0.554 |
|  | Alcohol status (drinker/abstainer) | 1.03 (0.69-1.55) | 0.886 |  | 1.00 (0.66-1.51) | 0.998 |
|  | BMI (<18.5, kg/m^2^) | 1.96 (1.17-3.29) | 0.011 |  | 1.67 (0.99-2.81) | 0.056 |
|  | BMI (≥24, kg/m^2^) | 0.67 (0.47-0.94) | 0.020 |  | 0.70 (0.49-0.99) | 0.047 |
|  | Highest education (high school) | 1.59 (0.60-4.24) | 0.353 |  | 1.67 (0.62-4.49) | 0.305 |
|  | Highest education (middle school) | 1.87 (0.74-4.72) | 0.184 |  | 2.16 (0.85-5.53) | 0.106 |
|  | Highest education (primary school/no formal school) | 1.92 (0.78-4.74) | 0.157 |  | 2.20 (0.85-5.69) | 0.105 |
| Esophagus |  |  |  |  |  |  |
|  | BMI (<18.5, kg/m^2^) | 1.09 (0.77-1.54) | 0.627 |  | 1.38 (0.97-1.96) | 0.076 |
|  | BMI (≥24, kg/m^2^) | 0.83 (0.69-1.01) | 0.057 |  | 0.74 (0.61-0.91) | 0.003 |
|  | Pack-years of smoking (<30) | 2.09 (1.66-2.63) | 3.57×10^-10^ |  | 1.21 (0.92-1.58) | 0.177 |
|  | Pack-years of smoking (≥30) | 2.76 (2.16-3.53) | 5.73×10^-16^ |  | 1.89 (1.41-2.54) | 2.49×10^-05^ |
|  | Alcohol status (drinker/abstainer) | 1.55 (1.26-1.92) | 4.69×10^-05^ |  | 1.59 (1.28-1.97) | 3.35×10^-05^ |
|  | Highest education (high school) | 2.46 (0.96-6.32) | 0.062 |  | 1.61 (0.62-4.20) | 0.329 |
|  | Highest education (middle school) | 2.99 (1.21-7.39) | 0.018 |  | 1.73 (0.69-4.38) | 0.245 |
|  | Highest education (primary school/no formal school) | 7.11 (2.93-17.27) | 1.49×10^-05^ |  | 3.00 (1.20-7.52) | 0.019 |
|  | Fruits intake (monthly/never) | 2.10 (1.74-2.54) | 1.12×10^-14^ |  | 1.12 (0.91-1.37) | 0.293 |
|  | Physical activity (MET hours /day) | 0.98 (0.97-0.99) | 2.67×10^-09^ |  | 1.00 (0.99-1.01) | 0.744 |
| Stomach |  |  |  |  |  |  |
|  | Pack-years of smoking (<30) | 1.85 (1.54-2.23) | 5.17×10^-11^ |  | 1.15 (0.93-1.43) | 0.206 |
|  | Pack-years of smoking (≥30) | 2.33 (1.89-2.86) | 1.61×10^-15^ |  | 1.19 (0.93-1.52) | 0.159 |
|  | Alcohol status (drinker/abstainer) | 1.26 (1.05-1.51) | 0.012 |  | 1.17 (0.97-1.41) | 0.098 |
|  | Salty vegetables intake (≥4 days/week) | 1.51 (1.30-1.77) | 1.77×10^-07^ |  | 1.08 (0.90-1.28) | 0.420 |
|  | Vegetables and fruits intake (occasional) | 1.09 (0.91-1.30) | 0.355 |  | 1.25 (1.03-1.52) | 0.021 |
|  | History of peptic ulcer (yes) | 1.45 (1.08-1.95) | 0.014 |  | 1.29 (0.96-1.74) | 0.091 |
|  | Highest education (high school) | 1.01 (0.66-1.54) | 0.971 |  | 1.11 (0.72-1.70) | 0.649 |
|  | Highest education (middle school) | 1.17 (0.79-1.73) | 0.439 |  | 1.30 (0.87-1.95) | 0.200 |
|  | Highest education (primary school/no formal school) | 1.84 (1.26-2.69) | 0.002 |  | 1.27 (0.85-1.91) | 0.244 |
| Colorectum |  |  |  |  |  |  |
|  | BMI (<18.5, kg/m^2^) | 0.89 (0.60-1.32) | 0.560 |  | 0.74 (0.50-1.10) | 0.138 |
|  | BMI (≥24, kg/m^2^) | 1.24 (1.07-1.44) | 0.004 |  | 1.22 (1.04-1.42) | 0.014 |
|  | Pack-years of smoking (≥30) | 1.49 (1.22-1.82) | 1.07×10^-04^ |  | 1.10 (0.89-1.36) | 0.393 |
|  | Alcohol status (drinker/abstainer) | 1.34 (1.12-1.59) | 0.001 |  | 1.27 (1.05-1.55) | 0.016 |
|  | Meat intake (1-3 days/week) | 1.40 (1.09-1.80) | 0.008 |  | 1.13 (0.86-1.49) | 0.366 |
|  | Meat intake (≥4 days/week) | 1.46 (1.14-1.85) | 0.002 |  | 1.15 (0.87-1.52) | 0.325 |
|  | Physical activity (MET hours /day) | 0.98 (0.97-0.98) | 4.23×10^-15^ |  | 1.00 (0.99-1.00) | 0.475 |
|  | Diabetes diagnosis (yes) | 1.57 (1.24-1.99) | 1.59×10^-04^ |  | 1.19 (0.94-1.51) | 0.156 |
| Liver |  |  |  |  |  |  |
|  | Highest education (high school) | 0.78 (0.52-1.17) | 0.225 |  | 0.90 (0.60-1.36) | 0.614 |
|  | Highest education (middle school) | 0.94 (0.66-1.34) | 0.727 |  | 1.11 (0.76-1.61) | 0.593 |
|  | Highest education (primary school/no formal school) | 1.54 (1.10-2.15) | 0.012 |  | 1.40 (0.96-2.04) | 0.076 |
|  | Diabetes diagnosis (yes) | 1.83 (1.44-2.34) | 1.12×10^-06^ |  | 1.63 (1.27-2.09) | 1.03×10^-04^ |
|  | History of gallstone/gallbladder (yes) | 1.99 (1.57-2.53) | 1.32×10^-08^ |  | 1.99 (1.56-2.53) | 2.88×10^-08^ |
|  | BMI (<18.5, kg/m^2^) | 1.24 (0.90-1.69) | 0.185 |  | 1.13 (0.82-1.55) | 0.445 |
|  | BMI (≥24, kg/m^2^) | 0.86 (0.74-1.02) | 0.080 |  | 0.90 (0.76-1.06) | 0.198 |
|  | Alcohol status (drinker/abstainer) | 1.54 (1.28-1.85) | 6.25×10^-06^ |  | 1.34 (1.11-1.62) | 0.003 |
|  | Physical activity (MET hours /day) | 0.98 (0.98-0.99) | 4.93×10^-09^ |  | 0.99 (0.99-1.00) | 0.109 |
|  | Smoking status (smoker/ex-smoker) | 1.95 (1.64-2.34) | 1.54×10^-13^ |  | 1.08 (0.87-1.34) | 0.463 |
|  | History of cirrhosis/chronic hepatitis (yes) | 7.02 (5.38-9.16) | 9.98×10^-47^ |  | 6.96 (5.31-9.11) | 3.24×10^-45^ |
| Pancreas |  |  |  |  |  |  |
|  | BMI (<18.5, kg/m^2^) | 1.29 (0.62-2.68) | 0.494 |  | 0.99 (0.48-2.07) | 0.986 |
|  | BMI (≥24, kg/m^2^) | 1.39 (1.02-1.91) | 0.038 |  | 1.42 (1.02-1.96) | 0.037 |
|  | Pack-years of smoking (<30) | 1.19 (0.79-1.79) | 0.418 |  | 1.06 (0.66-1.72) | 0.807 |
|  | Pack-years of smoking (≥30) | 2.26 (1.48-3.46) | 1.78×10^-04^ |  | 1.46 (0.86-2.46) | 0.157 |
|  | Alcohol status (drinker/abstainer) | 1.13 (0.76-1.68) | 0.534 |  | 1.09 (0.72-1.65) | 0.675 |
|  | Diabetes diagnosis (yes) | 2.81 (1.85-4.26) | 1.13×10^-06^ |  | 1.99 (1.30-3.04) | 0.001 |
| Lung |  |  |  |  |  |  |
|  | BMI (<18.5, kg/m^2^) | 1.62 (1.35-1.94) | 1.76×10^-07^ |  | 1.44 (1.20-1.73) | 7.76×10^-05^ |
|  | BMI (≥24, kg/m^2^) | 0.91 (0.82-1.01) | 0.091 |  | 0.85 (0.76-0.95) | 0.004 |
|  | Physical activity (MET hours /day) | 0.98 (0.97-0.98) | 8.01×10^-30^ |  | 1.00 (0.99-1.00) | 0.729 |
|  | Highest education (high school) | 0.91 (0.69-1.21) | 0.515 |  | 1.20 (0.90-1.59) | 0.210 |
|  | Highest education (middle school) | 1.05 (0.81-1.35) | 0.723 |  | 1.47 (1.13-1.91) | 0.004 |
|  | Highest education (primary school/no formal school) | 1.57 (1.22-2.01) | 3.64×10^-04^ |  | 1.55 (1.19-2.01) | 0.001 |
|  | Frequent cough (yes) | 1.20 (1.03-1.38) | 0.015 |  | 1.24 (1.07-1.43) | 0.004 |
|  | History of emphysema/bronchitis (yes) | 1.78 (1.47-2.15) | 1.98×10^-09^ |  | 1.43 (1.18-1.73) | 2.04×10^-04^ |
|  | Pack-years of smoking (<30) | 1.96 (1.70-2.25) | 9.65×10^-21^ |  | 1.69 (1.44-2.00) | 4.20×10^-10^ |
|  | Pack-years of smoking (≥30) | 4.25 (3.72-4.86) | 2.02×10^-99^ |  | 3.03 (2.55-3.59) | 1.25×10^-36^ |
|  | Height (cm) | 1.00 (1.00-1.01) | 0.273 |  | 1.01 (1.01-1.02) | 0.002 |
| Breast |  |  |  |  |  |  |
|  | Number of pregnancies (n=1) | 0.41 (0.21-0.80) | 0.008 |  | 0.41 (0.21-0.80) | 0.009 |
|  | Number of pregnancies (n=2) | 0.52 (0.28-0.96) | 0.036 |  | 0.55 (0.30-1.02) | 0.060 |
|  | Number of pregnancies (n≥3) | 0.43 (0.23-0.79) | 0.006 |  | 0.45 (0.25-0.83) | 0.010 |
|  | Age at menarche (13-14, years) | 0.76 (0.53-1.08) | 0.129 |  | 0.74 (0.51-1.05) | 0.093 |
|  | Age at menarche (15-16, years) | 0.77 (0.54-1.09) | 0.140 |  | 0.73 (0.51-1.04) | 0.084 |
|  | Age at menarche (≥17, years) | 0.64 (0.44-0.94) | 0.022 |  | 0.62 (0.42-0.90) | 0.013 |
|  | Menopausal status (current/post/missing) | 1.40 (1.15-1.71) | 9.14×10^-04^ |  | 1.03 (0.78-1.37) | 0.815 |
|  | BMI (<18.5, kg/m^2^) | 0.68 (0.37-1.24) | 0.208 |  | 0.68 (0.37-1.26) | 0.223 |
|  | BMI (≥24, kg/m^2^) | 1.42 (1.18-1.71) | 1.92×10^-04^ |  | 1.32 (1.09-1.60) | 0.004 |
|  | Alcohol status (drinker/abstainer) | 1.07 (0.66-1.74) | 0.788 |  | 1.01 (0.62-1.66) | 0.955 |
|  | Height (cm) | 1.04 (1.03-1.06) | 2.75×10^-07^ |  | 1.03 (1.02-1.05) | 1.16×10^-04^ |
|  | Highest education (high school) | 0.79 (0.57-1.11) | 0.173 |  | 0.90 (0.64-1.26) | 0.528 |
|  | Highest education (middle school) | 0.53 (0.38-0.73) | 1.22×10^-04^ |  | 0.68 (0.48-0.95) | 0.024 |
|  | Highest education (primary school/no formal school) | 0.37 (0.27-0.51) | 2.18×10^-09^ |  | 0.57 (0.39-0.83) | 0.003 |
| Cervix |  |  |  |  |  |  |
|  | Number of pregnancies (n=1) | 2.32 (0.31-17.20) | 0.411 |  | 2.42 (0.32-18.00) | 0.389 |
|  | Number of pregnancies (n=2) | 2.38 (0.33-17.14) | 0.391 |  | 2.20 (0.30-15.92) | 0.434 |
|  | Number of pregnancies (n≥3) | 2.21 (0.31-15.77) | 0.431 |  | 2.03 (0.28-14.51) | 0.482 |
|  | Age at menarche (13-14, years) | 1.62 (0.81-3.24) | 0.168 |  | 1.50 (0.75-2.99) | 0.252 |
|  | Age at menarche (15-16, years) | 1.27 (0.64-2.52) | 0.503 |  | 1.18 (0.59-2.35) | 0.644 |
|  | Age at menarche (≥17, years) | 1.20 (0.60-2.41) | 0.611 |  | 1.11 (0.55-2.26) | 0.768 |
|  | Alcohol status (drinker/abstainer) | 1.36 (0.72-2.56) | 0.343 |  | 1.60 (0.84-3.06) | 0.154 |
|  | BMI (<18.5, kg/m^2^) | 1.14 (0.63-2.07) | 0.659 |  | 1.02 (0.56-1.86) | 0.940 |
|  | BMI (≥24, kg/m^2^) | 0.97 (0.75-1.26) | 0.831 |  | 1.10 (0.84-1.44) | 0.503 |
| Endometrium |  |  |  |  |  |  |
|  | Number of pregnancies (n=1) | 0.20 (0.05-0.80) | 0.023 |  | 0.20 (0.05-0.83) | 0.026 |
|  | Number of pregnancies (n=2) | 0.31 (0.09-1.01) | 0.052 |  | 0.33 (0.10-1.09) | 0.069 |
|  | Number of pregnancies (n≥3) | 0.24 (0.07-0.77) | 0.017 |  | 0.26 (0.08-0.83) | 0.023 |
|  | Age at menarche (13-14, years) | 0.76 (0.31-1.87) | 0.557 |  | 0.74 (0.30-1.82) | 0.513 |
|  | Age at menarche (15-16, years) | 0.76 (0.31-1.82) | 0.535 |  | 0.74 (0.30-1.79) | 0.503 |
|  | Age at menarche (≥17, years) | 0.84 (0.34-2.07) | 0.711 |  | 0.83 (0.33-2.07) | 0.682 |
|  | BMI (<18.5, kg/m^2^) | 1.06 (0.32-3.46) | 0.927 |  | 1.05 (0.32-3.47) | 0.931 |
|  | BMI (≥24, kg/m^2^) | 1.71 (1.10-2.67) | 0.017 |  | 1.61 (1.02-2.54) | 0.042 |
|  | Menopausal status (current/post/missing) | 0.84 (0.53-1.33) | 0.457 |  | 0.66 (0.34-1.30) | 0.229 |
|  | Diabetes diagnosis (yes) | 2.09 (1.10-3.99) | 0.025 |  | 1.80 (0.93-3.47) | 0.079 |
| Ovary |  |  |  |  |  |  |
|  | Number of pregnancies (n=1) | 0.29 (0.08-1.11) | 0.071 |  | 0.28 (0.07-1.05) | 0.059 |
|  | Number of pregnancies (n=2) | 0.34 (0.10-1.13) | 0.077 |  | 0.33 (0.10-1.10) | 0.070 |
|  | Number of pregnancies (n≥3) | 0.31 (0.10-0.99) | 0.049 |  | 0.29 (0.09-0.94) | 0.038 |
|  | BMI (<18.5, kg/m^2^) | 1.83 (0.77-4.35) | 0.171 |  | 1.78 (0.75-4.25) | 0.193 |
|  | BMI (≥24, kg/m^2^) | 1.45 (0.95-2.21) | 0.087 |  | 1.46 (0.95-2.25) | 0.086 |
|  | Menopausal status (current/post/missing) | 1.61 (1.02-2.55) | 0.042 |  | 1.42 (0.74-2.69) | 0.289 |
|  | Highest education (high school) | 0.90 (0.37-2.18) | 0.819 |  | 0.83 (0.34-2.04) | 0.684 |
|  | Highest education (middle school) | 0.81 (0.35-1.86) | 0.613 |  | 0.71 (0.30-1.70) | 0.445 |
|  | Highest education (primary school/no formal school) | 0.49 (0.21-1.13) | 0.093 |  | 0.41 (0.16-1.04) | 0.061 |
|  | Height (cm) | 1.02 (0.98-1.05) | 0.313 |  | 1.03 (0.99-1.07) | 0.128 |
| Prostate |  |  |  |  |  |  |
|  | Smoking status (smoker/ex-smoker) | 0.93 (0.60-1.46) | 0.755 |  | 0.93 (0.59-1.48) | 0.772 |
|  | BMI (<18.5, kg/m^2^) | 0.48 (0.12-1.99) | 0.314 |  | 0.32 (0.08-1.31) | 0.112 |
|  | BMI (≥24, kg/m^2^) | 1.25 (0.83-1.88) | 0.290 |  | 1.66 (1.07-2.56) | 0.023 |
|  | Physical activity (MET hours /day) | 0.95 (0.94-0.97) | 4.17×10^-07^ |  | 0.98 (0.96-1.00) | 0.082 |
| Bladder |  |  |  |  |  |  |
|  | BMI (<18.5, kg/m^2^) | 1.94 (1.05-3.59) | 0.035 |  | 1.59 (0.85-2.97) | 0.143 |
|  | BMI (≥24, kg/m^2^) | 1.32 (0.94-1.85) | 0.104 |  | 1.15 (0.81-1.65) | 0.433 |
|  | Pack-years of smoking (<30) | 2.07 (1.37-3.12) | 5.29×10^-04^ |  | 1.18 (0.72-1.94) | 0.517 |
|  | Pack-years of smoking (≥30) | 5.48 (3.77-7.98) | 6.86×10^-19^ |  | 2.10 (1.29-3.42) | 0.003 |
|  | Meat intake (1-3 days/week) | 1.63 (0.89-2.99) | 0.117 |  | 1.38 (0.71-2.67) | 0.344 |
|  | Meat intake (≥4 days/week) | 1.77 (0.98-3.18) | 0.058 |  | 1.38 (0.70-2.70) | 0.354 |

HR, hazard ratio; CI, confidence interval; BMI, body mass index.

^*^ Mutual adjusted.

^†^ Adjusted for age, sex (if applicable), region, and family history of cancer.
