# Supplementary material for: Polygenic risk scores for pan-cancer risk prediction in the Chinese population: A population-based cohort study based on the China Kadoorie Biobank
Source: PLoS Med. 2025 Feb 28;22(2):e1004534. doi: 10.1371/journal.pmed.1004534 (PMC11870365; doi:10.1371/journal.pmed.1004534)
Supplement: S14 Table — HR, hazard ratio; CI, confidence interval. (DOCX) [file pmed.1004534.s018.docx]

**S14 Table. Association details for the risk groups defined by modifiable risk factors in the CKB cohort**

| **Cancer site** | **Risk factors group** | **Cases** | **Person-years** | **Incidence rate ^*^** | **Model 1 ^†^** | |  | **Model 2 ^‡^** | |
| --- | --- | --- | --- | --- | --- | --- | --- | --- | --- |
|  |  |  |  |  | **HR (95% CI)** | ***P-*value** |  | **HR (95% CI)** | ***P-*value** |
| Esophagus | Reduced | 87 | 559,020 | 15.56 | Ref | - |  | Ref | - |
|  | Elevated | 412 | 513,389 | 80.25 | 1.85 (1.44-2.38) | 1.76×10^-06^ |  | 1.85 (1.44-2.38) | 1.85×10^-06^ |
| Stomach | Reduced | 142 | 380,369 | 37.33 | Ref | - |  | Ref | - |
|  | Elevated | 603 | 691,660 | 87.18 | 1.24 (1.01-1.52) | 0.038 |  | 1.24 (1.02-1.52) | 0.034 |
| Colorectum | Reduced | 242 | 546,077 | 44.32 | Ref | - |  | Ref | - |
|  | Elevated | 498 | 525,007 | 94.86 | 1.19 (1.00-1.42) | 0.049 |  | 1.18 (0.99-1.41) | 0.060 |
| Pancreas | Reduced | 53 | 482,242 | 10.99 | Ref | - |  | Ref | - |
|  | Elevated | 117 | 591,036 | 19.80 | 1.47 (1.05-2.06) | 0.024 |  | 1.47 (1.05-2.06) | 0.024 |
| Lung | Reduced | 361 | 558,793 | 64.60 | Ref | - |  | Ref | - |
|  | Elevated | 1179 | 512,456 | 230.07 | 1.80 (1.58-2.05) | 1.40×10^-18^ |  | 1.80 (1.58-2.05) | 1.63×10^-18^ |
| Breast | Reduced | 138 | 308,774 | 44.69 | Ref | - |  | Ref | - |
|  | Elevated | 348 | 318,406 | 109.29 | 1.71 (1.36-2.13) | 2.76×10^-06^ |  | 1.72 (1.37-2.15) | 2.16×10^-06^ |
| Cervix | Reduced | 82 | 257,421 | 31.85 | Ref | - |  | Ref | - |
|  | Elevated | 155 | 370,850 | 41.80 | 1.25 (0.95-1.65) | 0.108 |  | 1.25 (0.94-1.64) | 0.120 |
| Ovary | Reduced | 25 | 314,119 | 7.96 | Ref | - |  | Ref | - |
|  | Elevated | 71 | 314,839 | 22.55 | 2.91 (1.79-4.74) | 1.69×10^-05^ |  | 2.91 (1.79-4.74) | 1.82×10^-05^ |
| Prostate | Reduced | 24 | 226,922 | 10.58 | Ref | - |  | Ref | - |
|  | Elevated | 71 | 217,045 | 32.71 | 1.97 (1.17-3.31) | 0.010 |  | 1.91 (1.13-3.22) | 0.015 |

HR, hazard ratio; CI, confidence interval.

^*^ Per 100,000 person-years;

^†^ Adjusted for age, sex (if applicable), region, and family history of cancer;

^‡^ Adjusted for age, sex (if applicable), region, family history of cancer, site-specific PRS and the top 10 principal components.
