# Supplementary material for: Polygenic risk scores for pan-cancer risk prediction in the Chinese population: A population-based cohort study based on the China Kadoorie Biobank
Source: PLoS Med. 2025 Feb 28;22(2):e1004534. doi: 10.1371/journal.pmed.1004534 (PMC11870365; doi:10.1371/journal.pmed.1004534)
Supplement: S15 Table — HR, hazard ratio; CI, confidence interval. (DOCX) [file pmed.1004534.s019.docx]

**S15 Table. Associations of summarized risk factors with different age onsets of each cancer type in the CKB cohort**

| **Cancer site** | **Age group of onset** | **Case** | **Incidence rate (per 100,000 person-years)** | **Risk factors, HR (95% CI)** | | |
| --- | --- | --- | --- | --- | --- | --- |
|  |  |  |  | **Reduced** | **Elevated** | ***P*_het** |
| Esophagus | <68 | 258 | 30.99 | 1.00 | 2.14 (1.55-2.95) |  |
|  | ≥68 | 241 | 100.37 | 1.00 | 1.32 (0.89-1.96) | 0.063 |
| Stomach | <68 | 386 | 46.37 | 1.00 | 1.16 (0.89-1.52) |  |
|  | ≥68 | 359 | 149.62 | 1.00 | 1.34 (0.97-1.85) | 0.500 |
| Colorectum | <68 | 381 | 45.79 | 1.00 | 1.28 (1.02-1.60) |  |
|  | ≥68 | 359 | 150.00 | 1.00 | 0.98 (0.75-1.28) | 0.134 |
| Pancreas | <70 | 84 | 9.54 | 1.00 | 1.99 (1.21-3.29) |  |
|  | ≥70 | 86 | 44.59 | 1.00 | 1.09 (0.69-1.73) | 0.082 |
| Lung | <68 | 690 | 82.92 | 1.00 | 1.61 (1.35-1.92) |  |
|  | ≥68 | 850 | 355.01 | 1.00 | 1.93 (1.58-2.36) | 0.183 |
| Breast | <53 | 148 | 65.99 | 1.00 | 1.70 (1.12-2.57) |  |
|  | ≥53 | 338 | 83.85 | 1.00 | 1.75 (1.34-2.28) | 0.908 |
| Cervix | <53 | 85 | 37.87 | 1.00 | 1.11 (0.68-1.83) |  |
|  | ≥53 | 152 | 37.62 | 1.00 | 1.30 (0.93-1.80) | 0.603 |
| Ovary | <53 | 28 | 12.46 | 1.00 | 3.19 (1.30-7.85) |  |
|  | ≥53 | 68 | 16.81 | 1.00 | 2.64 (1.47-4.73) | 0.729 |
| Prostate | <73 | 34 | 8.87 | 1.00 | 1.14 (0.53-2.47) |  |
|  | ≥73 | 61 | 100.11 | 1.00 | 3.20 (1.45-7.06) | 0.067 |

HR, hazard ratio; CI, confidence interval.
