# Supplementary material for: Polygenic risk scores for pan-cancer risk prediction in the Chinese population: A population-based cohort study based on the China Kadoorie Biobank
Source: PLoS Med. 2025 Feb 28;22(2):e1004534. doi: 10.1371/journal.pmed.1004534 (PMC11870365; doi:10.1371/journal.pmed.1004534)
Supplement: S16 Table — PRS, polygenic risk score; RF, modifiable risk factor; HR, hazard ratio; CI, confidence interval. (DOCX) [file pmed.1004534.s020.docx]

**S16 Table. Assessment of the combined effect of polygenic risk score groups and modifiable risk factor groups in the CKB cohort**

| **Cancer site** | **Cases** | **Person-years** | **Incidence rate ^*^** | **Combined group** | **HR (95% CI) ^†^** | ***P-*value ^†^** |
| --- | --- | --- | --- | --- | --- | --- |
| Esophagus |  |  |  |  |  |  |
|  | 14 | 113,202 | 12.37 | Low PRS & Reduced RF | Ref | - |
|  | 62 | 101,724 | 60.95 | Low PRS & Elevated RF | 1.74 (0.97-3.14) | 0.065 |
|  | 45 | 333,952 | 13.47 | Medium PRS & Reduced RF | 1.10 (0.61-2.01) | 0.745 |
|  | 238 | 309,356 | 76.93 | Medium PRS & Elevated RF | 2.31 (1.33-3.99) | 0.003 |
|  | 28 | 111,866 | 25.03 | High PRS & Reduced RF | 2.24 (1.18-4.25) | 0.014 |
|  | 112 | 102,308 | 109.47 | High PRS & Elevated RF | 3.37 (1.92-5.94) | 2.49×10^-05^ |
| Stomach |  |  |  |  |  |  |
|  | 17 | 76,638 | 22.18 | Low PRS & Reduced RF | Ref | - |
|  | 78 | 138,778 | 56.21 | Low PRS & Elevated RF | 1.30 (0.76-2.21) | 0.341 |
|  | 83 | 227,950 | 36.41 | Medium PRS & Reduced RF | 1.53 (0.91-2.59) | 0.108 |
|  | 360 | 414,698 | 86.81 | Medium PRS & Elevated RF | 1.93 (1.18-3.17) | 0.009 |
|  | 42 | 75,781 | 55.42 | High PRS & Reduced RF | 2.18 (1.24-3.84) | 0.007 |
|  | 165 | 138,185 | 119.41 | High PRS & Elevated RF | 2.60 (1.56-4.32) | 2.28×10^-04^ |
| Colorectum |  |  |  |  |  |  |
|  | 30 | 106,227 | 28.24 | Low PRS & Reduced RF | Ref | - |
|  | 54 | 109,078 | 49.51 | Low PRS & Elevated RF | 0.98 (0.62-1.54) | 0.934 |
|  | 115 | 329,231 | 34.93 | Medium PRS & Reduced RF | 1.27 (0.85-1.90) | 0.247 |
|  | 285 | 313,598 | 90.88 | Medium PRS & Elevated RF | 1.81 (1.23-2.66) | 0.002 |
|  | 97 | 110,619 | 87.69 | High PRS & Reduced RF | 3.29 (2.18-4.96) | 1.38×10^-08^ |
|  | 159 | 102,332 | 155.38 | High PRS & Elevated RF | 3.13 (2.10-4.66) | 2.03×10^-08^ |
| Pancreas |  |  |  |  |  |  |
|  | 3 | 99,020 | 3.03 | Low PRS & Reduced RF | Ref | - |
|  | 16 | 116,762 | 13.70 | Low PRS & Elevated RF | 3.69 (1.07-12.70) | 0.039 |
|  | 37 | 287,683 | 12.86 | Medium PRS & Reduced RF | 4.16 (1.28-13.50) | 0.018 |
|  | 66 | 355,523 | 18.56 | Medium PRS & Elevated RF | 4.96 (1.55-15.82) | 0.007 |
|  | 13 | 95,539 | 13.61 | High PRS & Reduced RF | 4.44 (1.26-15.57) | 0.020 |
|  | 35 | 118,750 | 29.47 | High PRS & Elevated RF | 7.84 (2.40-25.59) | 6.39×10^-04^ |
| Lung |  |  |  |  |  |  |
|  | 54 | 111,083 | 48.61 | Low PRS & Reduced RF | Ref | - |
|  | 197 | 103,326 | 190.66 | Low PRS & Elevated RF | 1.99 (1.46-2.70) | 1.13×10^-05^ |
|  | 196 | 335,243 | 58.47 | Medium PRS & Reduced RF | 1.19 (0.88-1.61) | 0.250 |
|  | 704 | 308,013 | 228.56 | Medium PRS & Elevated RF | 2.34 (1.77-3.11) | 3.45×10^-09^ |
|  | 111 | 112,467 | 98.70 | High PRS & Reduced RF | 2.03 (1.47-2.81) | 1.95×10^-05^ |
|  | 278 | 101,117 | 274.93 | High PRS & Elevated RF | 2.87 (2.13-3.86) | 3.73×10^-12^ |
| Breast |  |  |  |  |  |  |
|  | 15 | 62,011 | 24.19 | Low PRS & Reduced RF | Ref | - |
|  | 39 | 63,713 | 61.21 | Low PRS & Elevated RF | 1.77 (0.97-3.24) | 0.065 |
|  | 81 | 185,495 | 43.67 | Medium PRS & Reduced RF | 1.81 (1.04-3.14) | 0.035 |
|  | 211 | 190,951 | 110.50 | Medium PRS & Elevated RF | 3.21 (1.88-5.48) | 1.85×10^-05^ |
|  | 42 | 61,267 | 68.55 | High PRS & Reduced RF | 2.80 (1.55-5.05) | 6.25×10^-04^ |
|  | 98 | 63,742 | 153.74 | High PRS & Elevated RF | 4.43 (2.55-7.71) | 1.40×10^-07^ |
| Cervix |  |  |  |  |  |  |
|  | 16 | 51,808 | 30.88 | Low PRS & Reduced RF | Ref | - |
|  | 21 | 74,318 | 28.26 | Low PRS & Elevated RF | 0.88 (0.46-1.69) | 0.699 |
|  | 43 | 154,070 | 27.91 | Medium PRS & Reduced RF | 0.88 (0.50-1.57) | 0.668 |
|  | 85 | 222,971 | 38.12 | Medium PRS & Elevated RF | 1.15 (0.67-1.98) | 0.601 |
|  | 23 | 51,543 | 44.62 | High PRS & Reduced RF | 1.45 (0.76-2.74) | 0.258 |
|  | 49 | 73,561 | 66.61 | High PRS & Elevated RF | 1.97 (1.11-3.48) | 0.020 |
| Ovary |  |  |  |  |  |  |
|  | 3 | 62,286 | 4.82 | Low PRS & Reduced RF | Ref | - |
|  | 7 | 63,285 | 11.06 | Low PRS & Elevated RF | 2.33 (0.59-9.13) | 0.225 |
|  | 14 | 188,029 | 7.45 | Medium PRS & Reduced RF | 1.52 (0.44-5.30) | 0.509 |
|  | 45 | 189,330 | 23.77 | Medium PRS & Elevated RF | 4.98 (1.53-16.26) | 0.008 |
|  | 8 | 63,803 | 12.54 | High PRS & Reduced RF | 2.54 (0.67-9.60) | 0.168 |
|  | 19 | 62,224 | 30.53 | High PRS & Elevated RF | 6.36 (1.86-21.74) | 0.003 |
| Prostate |  |  |  |  |  |  |
|  | 2 | 46,413 | 4.31 | Low PRS & Reduced RF | Ref | - |
|  | 8 | 42,677 | 18.75 | Low PRS & Elevated RF | 2.92 (0.61-14.05) | 0.180 |
|  | 13 | 135,878 | 9.57 | Medium PRS & Reduced RF | 2.18 (0.49-9.66) | 0.305 |
|  | 37 | 130,552 | 28.34 | Medium PRS & Elevated RF | 4.04 (0.95-17.10) | 0.058 |
|  | 9 | 44,630 | 20.17 | High PRS & Reduced RF | 4.63 (1.00-21.46) | 0.050 |
|  | 26 | 43,817 | 59.34 | High PRS & Elevated RF | 8.26 (1.92-35.46) | 0.005 |

PRS, polygenic risk score; RF, modifiable risk factor; HR, hazard ratio; CI, confidence interval.

^*^ Per 100,000 person-years.

^†^ Adjusted for age, sex (if applicable), region, family history of cancer, and the top 10 principal components.
