# Supplementary material for: Polygenic risk scores for pan-cancer risk prediction in the Chinese population: A population-based cohort study based on the China Kadoorie Biobank
Source: PLoS Med. 2025 Feb 28;22(2):e1004534. doi: 10.1371/journal.pmed.1004534 (PMC11870365; doi:10.1371/journal.pmed.1004534)
Supplement: S17 Table — PRS, polygenic risk score; RF, modifiable risk factor; HR, hazard ratio; CI, confidence interval. (DOCX) [file pmed.1004534.s021.docx]

**S17 Table. Assessment of multiplicative interaction between polygenic risk score groups and modifiable risk factor groups in the CKB cohort**

| **Cancer site** | **Interaction Combination** | **HR (95% CI) *** | ***P*_interaction *** |
| --- | --- | --- | --- |
| Esophagus | Medium PRS * Elevated RF | 1.20 (0.62-2.32) | 0.590 |
|  | High PRS * Elevated RF | 0.87 (0.42-1.77) | 0.691 |
| Stomach | Medium PRS * Elevated RF | 0.97 (0.55-1.73) | 0.920 |
|  | High PRS * Elevated RF | 0.92 (0.49-1.72) | 0.794 |
| Colorectum | Medium PRS * Elevated RF | 1.45 (0.89-2.39) | 0.139 |
|  | High PRS * Elevated RF | 0.97 (0.58-1.62) | 0.908 |
| Pancreas | Medium PRS * Elevated RF | 0.32 (0.09-1.18) | 0.088 |
|  | High PRS * Elevated RF | 0.48 (0.12-1.92) | 0.299 |
| Lung | Medium PRS * Elevated RF | 0.99 (0.70-1.39) | 0.948 |
|  | High PRS * Elevated RF | 0.71 (0.49-1.03) | 0.072 |
| Breast | Medium PRS * Elevated RF | 1.00 (0.52-1.92) | 0.991 |
|  | High PRS * Elevated RF | 0.90 (0.45-1.80) | 0.755 |
| Cervix | Medium PRS * Elevated RF | 1.49 (0.71-3.14) | 0.296 |
|  | High PRS * Elevated RF | 1.55 (0.68-3.51) | 0.294 |
| Ovary | Medium PRS * Elevated RF | 1.40 (0.32-6.17) | 0.653 |
|  | High PRS * Elevated RF | 1.07 (0.22-5.23) | 0.931 |
| Prostate | Medium PRS * Elevated RF | 0.63 (0.12-3.38) | 0.593 |
|  | High PRS * Elevated RF | 0.61 (0.11-3.42) | 0.574 |

PRS, polygenic risk score; RF, modifiable risk factor; HR, hazard ratio; CI, confidence interval.

^*^ Adjusted for age, sex (if applicable), region, family history of cancer, and the top 10 principal components.
