# Supplementary material for: Polygenic risk scores for pan-cancer risk prediction in the Chinese population: A population-based cohort study based on the China Kadoorie Biobank
Source: PLoS Med. 2025 Feb 28;22(2):e1004534. doi: 10.1371/journal.pmed.1004534 (PMC11870365; doi:10.1371/journal.pmed.1004534)
Supplement: S18 Table — PRS, polygenic risk score; CI, confidence interval; AUC, area under the curve; NRI, net reclassification improvement. (DOCX) [file pmed.1004534.s022.docx]

**S18 Table. Assessment of model discrimination for each cancer after incorporating modifiable risk factors and polygenic risk scores in the CKB cohort**

| **Cancer site** | **Cases** | **Non-cases** | **Model specification ^*^** | **C-index (95% CI)** | **ΔC ^†^** | ***P*_lr ^‡^** | **10-year AUC (95% CI)** | **ΔAUC ^†^** | ***P*_DeLong ^‡^** | **NRI (95% CI)** |
| --- | --- | --- | --- | --- | --- | --- | --- | --- | --- | --- |
| Esophagus | 499 | 99,720 | Model 1 | 0.805 (0.787-0.823) | - | - | 0.793 (0.773-0.812) | - | - | - |
|  |  |  | Model 2 | 0.820 (0.803-0.837) | 0.015 | 3.33×10^-16^ | 0.808 (0.790-0.827) | 0.016 | 5.31×10^-06^ | 19.8% (15.2%-23.6%) |
|  |  |  | Model 3 | 0.824 (0.807-0.840) | 0.004 | 1.07×10^-05^ | 0.811 (0.794-0.829) | 0.003 | 0.050 | 6.2% (0.3%-10.5%) |
| Stomach | 745 | 99,474 | Model 1 | 0.734 (0.717-0.751) | - | - | 0.717 (0.699-0.736) | - | - | - |
|  |  |  | Model 2 | 0.740 (0.724-0.757) | 0.006 | 2.52×10^-10^ | 0.724 (0.706-0.742) | 0.007 | 0.007 | 10.0% (6.5%-13.3%) |
|  |  |  | Model 3 | 0.750 (0.733-0.766) | 0.010 | 2.92×10^-11^ | 0.734 (0.716-0.752) | 0.010 | 1.40×10^-04^ | 8.8% (5.0%-12.7%) |
| Colorectum | 740 | 99,479 | Model 1 | 0.714 (0.696-0.731) | - | - | 0.700 (0.681-0.719) | - | - | - |
|  |  |  | Model 2 | 0.717 (0.700-0.735) | 0.003 | 9.50×10^-06^ | 0.704 (0.685-0.723) | 0.004 | 0.059 | 5.6% (2.0%-8.7%) |
|  |  |  | Model 3 | 0.742 (0.725-0.759) | 0.025 | <2.00×10^-16^ | 0.731 (0.712-0.749) | 0.027 | 6.27×10^-07^ | 15.3% (10.5%-19.8%) |
| Pancreas | 170 | 100,049 | Model 1 | 0.733 (0.696-0.770) | - | - | 0.724 (0.682-0.767) | - | - | - |
|  |  |  | Model 2 | 0.750 (0.714-0.787) | 0.017 | 1.72×10^-05^ | 0.738 (0.696-0.780) | 0.013 | 0.091 | 11.4% (0.1%-20.3%) |
|  |  |  | Model 3 | 0.758 (0.721-0.795) | 0.008 | 0.001 | 0.748 (0.705-0.791) | 0.010 | 0.036 | 17.4% (6.9%-27.1%) |
| Lung | 1,540 | 98,679 | Model 1 | 0.755 (0.743-0.766) | - | - | 0.736 (0.724-0.748) | - | - | - |
|  |  |  | Model 2 | 0.770 (0.759-0.782) | 0.015 | <2.00×10^-16^ | 0.753 (0.741-0.766) | 0.017 | 6.31×10^-12^ | 13.9% (10.8%-16.9%) |
|  |  |  | Model 3 | 0.775 (0.764-0.786) | 0.005 | 2.11×10^-14^ | 0.759 (0.746-0.771) | 0.005 | 7.15×10^-05^ | 7.6% (5.1%-10.3%) |
| Breast | 486 | 56,873 | Model 1 | 0.614 (0.590-0.638) | - | - | 0.620 (0.593-0.646) | - | - | - |
|  |  |  | Model 2 | 0.665 (0.640-0.689) | 0.051 | <2.00×10^-16^ | 0.666 (0.639-0.693) | 0.046 | 1.83×10^-06^ | 16.6% (11.7%-20.7%) |
|  |  |  | Model 3 | 0.686 (0.662-0.710) | 0.021 | 9.77×10^-15^ | 0.684 (0.658-0.710) | 0.018 | 0.012 | 15.0% (10.0%-19.3%) |
| Cervix | 237 | 57,122 | Model 1 | 0.544 (0.511-0.576) | - | - | 0.548 (0.513-0.582) | - | - | - |
|  |  |  | Model 2 | 0.577 (0.540-0.614) | 0.033 | 0.010 | 0.577 (0.539-0.616) | 0.030 | 0.063 | 5.9% (-0.1%-12.9%) |
|  |  |  | Model 3 | 0.595 (0.558-0.632) | 0.018 | 0.003 | 0.596 (0.558-0.635) | 0.019 | 0.170 | 11.2% (2.5%-18.6%) |
| Ovary | 96 | 57,263 | Model 1 | 0.560 (0.507-0.613) | - | - | 0.543 (0.484-0.603) | - | - | - |
|  |  |  | Model 2 | 0.629 (0.574-0.685) | 0.069 | 1.47×10^-04^ | 0.638 (0.577-0.699) | 0.095 | 0.005 | 20.8% (6.6%-33.2%) |
|  |  |  | Model 3 | 0.648 (0.592-0.703) | 0.019 | 0.027 | 0.662 (0.602-0.722) | 0.024 | 0.094 | 14.2% (1.9%-24.4%) |
| Prostate | 95 | 42,765 | Model 1 | 0.838 (0.804-0.873) | - | - | 0.809 (0.768-0.850) | - | - | - |
|  |  |  | Model 2 | 0.840 (0.805-0.875) | 0.002 | 0.095 | 0.810 (0.769-0.851) | 0.001 | 0.682 | 10.5% (-5.3%-18.2%) |
|  |  |  | Model 3 | 0.854 (0.819-0.889) | 0.014 | 5.23×10^-08^ | 0.829 (0.788-0.870) | 0.019 | 0.043 | 20.4% (10.9%-29.6%) |

PRS, polygenic risk score; CI, confidence interval; AUC, area under the curve; NRI, net reclassification improvement.

^*^ Model 1: Including demographic factors (age, sex, and region) and family history of cancer; Model 2: Adding summarized modifiable risk factors to Model 1; Model 3: Adding PRS to Model 2.

^†^ ΔC was the C-index difference between Model 2 and Model 1, as well as between Model 3 and Model 2; so was ΔAUC.

^‡^ The likelihood-ratio test was performed between Model 2 and Model 1, as well as between Model 3 and Model 2; so was the DeLong test.
