# Supplementary material for: Polygenic risk scores for pan-cancer risk prediction in the Chinese population: A population-based cohort study based on the China Kadoorie Biobank
Source: PLoS Med. 2025 Feb 28;22(2):e1004534. doi: 10.1371/journal.pmed.1004534 (PMC11870365; doi:10.1371/journal.pmed.1004534)
Supplement: S19 Table — PRS, polygenic risk score; CI, confidence interval; AUC, area under the curve. (DOCX) [file pmed.1004534.s023.docx]

**S19 Table. Assessment of model discrimination for each cancer after incorporating cross-cancer polygenic risk scores in the CKB cohort**

| **Group** | **Cancer site** | **Model specification ^*^** | **Added cross-cancer PRS** | **C-index (95% CI)** | **ΔC ^†^** | ***P*_lr ^‡^** | **10-year AUC (95% CI)** | **ΔAUC ^†^** | ***P*_Delong ^‡^** |
| --- | --- | --- | --- | --- | --- | --- | --- | --- | --- |
| Women | Esophagus | Model 1 | - | 0.819 (0.787-0.851) | - | - | 0.811 (0.776-0.846) | - | - |
|  |  | Model 2 | Stomach | 0.820 (0.788-0.852) | 0.001 | 0.057 | 0.812 (0.777-0.847) | 0.001 | 0.530 |
|  | Stomach | Model 1 | - | 0.722 (0.694-0.749) | - | - | 0.710 (0.681-0.740) | - | - |
|  |  | Model 2 | Esophagus, Colorectum, Breast | 0.728 (0.701-0.756) | 0.006 | 0.003 | 0.717 (0.688-0.747) | 0.007 | 0.180 |
|  | Colorectum | Model 1 | - | 0.727 (0.703-0.751) | - | - | 0.722 (0.696-0.749) | - | - |
|  |  | Model 2 | Esophagus | 0.727 (0.703-0.751) | 0.000 | 0.997 | 0.722 (0.696-0.749) | 0.000 | 0.456 |
|  | Pancreas | Model 1 | - | 0.760 (0.705-0.816) | - | - | 0.743 (0.677-0.809) | - | - |
|  |  | Model 2 | Colorectum | 0.762 (0.706-0.817) | 0.002 | 0.045 | 0.748 (0.683-0.812) | 0.005 | 0.454 |
|  | Breast | Model 1 | - | 0.686 (0.662-0.710) | - | - | 0.684 (0.658-0.710) | - | - |
|  |  | Model 2 | Colorectum, Pancreas | 0.691 (0.667-0.715) | 0.005 | 0.004 | 0.689 (0.663-0.715) | 0.005 | 0.082 |
|  | Cervix | Model 1 | - | 0.595 (0.558-0.632) | - | - | 0.596 (0.558-0.635) | - | - |
|  |  | Model 2 | Lung | 0.604 (0.566-0.641) | 0.009 | 0.016 | 0.609 (0.569-0.649) | 0.013 | 0.163 |
|  | Ovary | Model 1 | - | 0.648 (0.592-0.703) | - | - | 0.662 (0.602-0.722) | - | - |
|  |  | Model 2 | Stomach | 0.660 (0.607-0.713) | 0.012 | 0.033 | 0.674 (0.616-0.732) | 0.012 | 0.357 |
| Men | Esophagus | Model 1 | - | 0.784 (0.762-0.806) | - | - | 0.767 (0.744-0.791) | - | - |
|  |  | Model 2 | Stomach | 0.785 (0.763-0.807) | 0.001 | 0.071 | 0.769 (0.745-0.793) | 0.002 | 0.163 |
|  | Stomach | Model 1 | - | 0.726 (0.705-0.746) | - | - | 0.708 (0.685-0.730) | - | - |
|  |  | Model 2 | Esophagus, Colorectum | 0.729 (0.708-0.750) | 0.003 | 0.002 | 0.713 (0.691-0.736) | 0.005 | 0.045 |
|  | Colorectum | Model 1 | - | 0.755 (0.731-0.779) | - | - | 0.738 (0.712-0.765) | - | - |
|  |  | Model 2 | Esophagus, Prostate | 0.762 (0.738-0.785) | 0.007 | 3.21×10^-04^ | 0.746 (0.720-0.772) | 0.008 | 0.009 |
|  | Pancreas | Model 1 | - | 0.746 (0.698-0.795) | - | - | 0.744 (0.690-0.799) | - | - |
|  |  | Model 2 | Colorectum | 0.747 (0.701-0.794) | 0.001 | 0.270 | 0.744 (0.691-0.797) | 0.000 | 0.961 |
| Combined | Esophagus | Model 1 | - | 0.824 (0.807-0.840) | - | - | 0.811 (0.794-0.829) | - | - |
|  |  | Model 2 | Stomach | 0.825 (0.808-0.841) | 0.001 | 0.011 | 0.813 (0.795-0.831) | 0.001 | 0.193 |
|  | Stomach | Model 1 | - | 0.750 (0.733-0.766) | - | - | 0.734 (0.716-0.752) | - | - |
|  |  | Model 2 | Esophagus, Colorectum | 0.752 (0.736-0.769) | 0.002 | 1.62×10^-05^ | 0.738 (0.720-0.756) | 0.004 | 0.042 |
|  | Colorectum | Model 1 | - | 0.742 (0.725-0.759) | - | - | 0.731 (0.712-0.749) | - | - |
|  |  | Model 2 | Esophagus | 0.743 (0.726-0.760) | 0.001 | 0.065 | 0.731 (0.713-0.750) | 0.001 | 0.317 |
|  | Pancreas | Model 1 | - | 0.758 (0.721-0.795) | - | - | 0.748 (0.705-0.791) | - | - |
|  |  | Model 2 | Colorectum | 0.760 (0.723-0.796) | 0.002 | 0.028 | 0.750 (0.709-0.792) | 0.002 | 0.488 |

PRS, polygenic risk score; CI, confidence interval; AUC, area under the curve.

^*^ Model 1: Including demographic factors (age, sex, and region) and family history of cancer, summarized modifiable risk factors, and site-specific PRS; Model 2: further adding the significant cross-cancer PRS to Model 1. Cross-cancer sex-specific PRS was assessed only within the corresponding sex group.

^†^ ΔC was the C-index difference between Model 2 and Model 1; so was ΔAUC.

^‡^ The likelihood-ratio test was performed between Model 2 and Model 1; so was the DeLong test.
