# Supplementary material for: Polygenic risk scores for pan-cancer risk prediction in the Chinese population: A population-based cohort study based on the China Kadoorie Biobank
Source: PLoS Med. 2025 Feb 28;22(2):e1004534. doi: 10.1371/journal.pmed.1004534 (PMC11870365; doi:10.1371/journal.pmed.1004534)
Supplement: S20 Table — PRS, polygenic risk score; CI, confidence interval; AUC, area under the curve; NRI, net reclassification improvement. (DOCX) [file pmed.1004534.s024.docx]

**S20 Table. Assessment of model discrimination for each cancer comparing different combinations of modifiable risk factors and polygenic risk scores after excluding all participants within the first year after recruitment**

| **Cancer site** | **Cases** | **Non-cases** | **Model specification ^*^** | **C-index (95% CI)** | **ΔC ^†^** | ***P*_lr ^‡^** | **10-year AUC (95% CI)** | **ΔAUC ^†^** | ***P*_DeLong ^‡^** | **NRI (95% CI)** |
| --- | --- | --- | --- | --- | --- | --- | --- | --- | --- | --- |
| Esophagus | 465 | 98,939 | Model 1 | 0.804 (0.785-0.822) | - | - | 0.792 (0.772-0.813) | - | - |  |
|  |  |  | Model 2 | 0.821 (0.803-0.838) | 0.017 | 1.11×10^-16^ | 0.810 (0.791-0.829) | 0.018 | 3.16×10^-06^ | 20.5% (16.1%-24.9%) |
|  |  |  | Model 3 | 0.824 (0.807-0.841) | 0.003 | 3.24×10^-05^ | 0.813 (0.794-0.832) | 0.003 | 0.070 | 5.6% (0.1%-11.8%) |
| Stomach | 699 | 98,707 | Model 1 | 0.731 (0.714-0.749) | - | - | 0.715 (0.696-0.734) | - | - |  |
|  |  |  | Model 2 | 0.738 (0.720-0.755) | 0.007 | 1.06×10^-09^ | 0.722 (0.703-0.741) | 0.007 | 0.008 | 9.6% (6.3%-14.1%) |
|  |  |  | Model 3 | 0.748 (0.731-0.765) | 0.010 | 2.08×10^-11^ | 0.733 (0.714-0.751) | 0.011 | 1.35×10^-04^ | 9.4% (4.3%-12.5%) |
| Colorectum | 709 | 98,694 | Model 1 | 0.713 (0.695-0.731) | - | - | 0.700 (0.680-0.719) | - | - |  |
|  |  |  | Model 2 | 0.716 (0.698-0.734) | 0.003 | 3.75×10^-05^ | 0.703 (0.684-0.723) | 0.004 | 0.094 | 5.3% (0.9%-9.1%) |
|  |  |  | Model 3 | 0.741 (0.724-0.759) | 0.025 | <2.00×10^-16^ | 0.731 (0.711-0.750) | 0.027 | 1.09×10^-06^ | 15.5% (10.4%-19.9%) |
| Pancreas | 165 | 99,264 | Model 1 | 0.735 (0.698-0.772) | - | - | 0.728 (0.685-0.770) | - | - |  |
|  |  |  | Model 2 | 0.750 (0.714-0.786) | 0.015 | 1.01×10^-04^ | 0.739 (0.697-0.781) | 0.011 | 0.146 | 9.5% (-1.6%-18.3%) |
|  |  |  | Model 3 | 0.759 (0.722-0.796) | 0.009 | 3.16×10^-04^ | 0.752 (0.709-0.795) | 0.013 | 0.015 | 18.7% (9.9%-26.8%) |
| Lung | 1,466 | 97,921 | Model 1 | 0.752 (0.741-0.764) | - | - | 0.734 (0.721-0.747) | - | - |  |
|  |  |  | Model 2 | 0.767 (0.755-0.779) | 0.015 | <2.00×10^-16^ | 0.750 (0.737-0.763) | 0.017 | 1.75×10^-10^ | 13.6% (10.2%-17.1%) |
|  |  |  | Model 3 | 0.773 (0.761-0.784) | 0.006 | 3.77×10^-15^ | 0.756 (0.744-0.769) | 0.006 | 7.51×10^-05^ | 8.1% (5.3%-10.8%) |
| Breast | 462 | 56,576 | Model 1 | 0.614 (0.589-0.639) | - | - | 0.619 (0.592-0.646) | - | - |  |
|  |  |  | Model 2 | 0.665 (0.640-0.690) | 0.051 | <2.00×10^-16^ | 0.666 (0.638-0.693) | 0.047 | 3.02×10^-06^ | 16.9% (11.1%-22.1%) |
|  |  |  | Model 3 | 0.687 (0.662-0.711) | 0.022 | 1.79×10^-14^ | 0.684 (0.657-0.711) | 0.019 | 0.014 | 15.3% (8.6%-20.6%) |
| Cervix | 226 | 56,825 | Model 1 | 0.546 (0.513-0.580) | - | - | 0.551 (0.516-0.587) | - | - |  |
|  |  |  | Model 2 | 0.587 (0.550-0.625) | 0.041 | 0.004 | 0.586 (0.547-0.625) | 0.035 | 0.041 | 6.7% (1.4%-12.7%) |
|  |  |  | Model 3 | 0.602 (0.565-0.639) | 0.015 | 0.002 | 0.603 (0.564-0.643) | 0.017 | 0.195 | 11.9% (5.2%-17.6%) |
| Ovary | 94 | 56,965 | Model 1 | 0.561 (0.507-0.615) | - | - | 0.545 (0.484-0.606) | - | - |  |
|  |  |  | Model 2 | 0.635 (0.578-0.692) | 0.074 | 1.49×10^-04^ | 0.646 (0.583-0.708) | 0.100 | 0.004 | 21.1% (8.6%-30.2%) |
|  |  |  | Model 3 | 0.648 (0.592-0.705) | 0.013 | 0.043 | 0.663 (0.602-0.725) | 0.018 | 0.186 | 12.9% (4.6%-23.4%) |
| Prostate | 91 | 42,277 | Model 1 | 0.839 (0.803-0.875) | - | - | 0.810 (0.768-0.852) | - | - |  |
|  |  |  | Model 2 | 0.840 (0.803-0.876) | 0.001 | 0.154 | 0.810 (0.768-0.853) | 0.000 | 0.885 | 9.5% (-3.7%-18.8%) |
|  |  |  | Model 3 | 0.857 (0.821-0.893) | 0.017 | 1.41×10^-08^ | 0.832 (0.789-0.875) | 0.022 | 0.028 | 20.2% (10.5%-33.7%) |

PRS, polygenic risk score; CI, confidence interval; AUC, area under the curve; NRI, net reclassification improvement.

^*^ Model 1: Including demographic factors (age, sex, and region) and family history of cancer; Model 2: Adding summarized modifiable risk factors to Model 1; Model 3: Adding PRS to Model 2.

^†^ ΔC was the C-index difference between Model 2 and Model 1, as well as between Model 3 and Model 2; so was ΔAUC.

^‡^ The likelihood-ratio test was performed between Model 2 and Model 1, as well as between Model 3 and Model 2; so was the DeLong test.
