# Supplementary material for: Polygenic risk scores for pan-cancer risk prediction in the Chinese population: A population-based cohort study based on the China Kadoorie Biobank
Source: PLoS Med. 2025 Feb 28;22(2):e1004534. doi: 10.1371/journal.pmed.1004534 (PMC11870365; doi:10.1371/journal.pmed.1004534)
Supplement: S21 Table — PRS, polygenic risk score; CI, confidence interval; AUC, area under the curve; NRI, net reclassification improvement. (DOCX) [file pmed.1004534.s025.docx]

**S21 Table. Assessment of model discrimination for each cancer comparing different combinations of modifiable risk factors and polygenic risk scores after only including the first primary cancer**

| **Cancer site** | **Cases** | **Non-cases** | **Model specification ^*^** | **C-index (95% CI)** | **ΔC ^†^** | ***P*_lr ^‡^** | **10-year AUC (95% CI)** | **ΔAUC ^†^** | ***P*_DeLong ^‡^** | **NRI (95% CI)** |
| --- | --- | --- | --- | --- | --- | --- | --- | --- | --- | --- |
| Esophagus | 443 | 99,720 | Model 1 | 0.807 (0.788-0.826) | - | - | 0.793 (0.773-0.814) | - | - |  |
|  |  |  | Model 2 | 0.822 (0.804-0.839) | 0.015 | 4.09×10^-14^ | 0.809 (0.790-0.828) | 0.016 | 1.15×10^-05^ | 20.6% (16.1%-25.5%) |
|  |  |  | Model 3 | 0.825 (0.807-0.842) | 0.003 | 7.67×10^-05^ | 0.811 (0.792-0.830) | 0.003 | 0.092 | 5.9% (0.2%-11.1%) |
| Stomach | 631 | 99,474 | Model 1 | 0.732 (0.714-0.750) | - | - | 0.717 (0.697-0.736) | - | - |  |
|  |  |  | Model 2 | 0.738 (0.720-0.756) | 0.006 | 1.43×10^-08^ | 0.724 (0.704-0.743) | 0.007 | 0.011 | 10.8% (6.0%-14.6%) |
|  |  |  | Model 3 | 0.749 (0.731-0.766) | 0.011 | 2.66×10^-11^ | 0.735 (0.716-0.754) | 0.011 | 4.86×10^-04^ | 9.5% (4.8%-12.9%) |
| Colorectum | 680 | 99,479 | Model 1 | 0.718 (0.700-0.736) | - | - | 0.705 (0.685-0.724) | - | - |  |
|  |  |  | Model 2 | 0.721 (0.703-0.739) | 0.003 | 7.34×10^-05^ | 0.708 (0.688-0.728) | 0.004 | 0.075 | 4.9% (1.6%-10.0%) |
|  |  |  | Model 3 | 0.748 (0.730-0.766) | 0.027 | <2.00×10^-16^ | 0.737 (0.718-0.757) | 0.029 | 2.27×10^-07^ | 16.5% (11.5%-21.0%) |
| Pancreas | 142 | 100,049 | Model 1 | 0.750 (0.713-0.788) | - | - | 0.736 (0.693-0.780) | - | - |  |
|  |  |  | Model 2 | 0.762 (0.726-0.798) | 0.012 | 3.94×10^-04^ | 0.747 (0.705-0.790) | 0.011 | 0.134 | 13.5% (-0.1%-24.6%) |
|  |  |  | Model 3 | 0.770 (0.732-0.807) | 0.008 | 6.86×10^-04^ | 0.758 (0.714-0.802) | 0.010 | 0.072 | 15.7% (4.6%-24.9%) |
| Lung | 1,363 | 98,679 | Model 1 | 0.759 (0.747-0.770) | - | - | 0.742 (0.729-0.755) | - | - |  |
|  |  |  | Model 2 | 0.774 (0.763-0.786) | 0.015 | <2.00×10^-16^ | 0.759 (0.745-0.772) | 0.017 | 8.75×10^-11^ | 13.6% (10.3%-17.4%) |
|  |  |  | Model 3 | 0.779 (0.768-0.791) | 0.005 | 1.72×10^-13^ | 0.764 (0.751-0.777) | 0.005 | 4.26×10^-04^ | 7.4% (4.0%-10.2%) |
| Breast | 439 | 56,873 | Model 1 | 0.613 (0.588-0.639) | - | - | 0.619 (0.592-0.647) | - | - |  |
|  |  |  | Model 2 | 0.664 (0.639-0.690) | 0.051 | <2.00×10^-16^ | 0.664 (0.636-0.692) | 0.045 | 8.16×10^-06^ | 15.4% (9.9%-20.4%) |
|  |  |  | Model 3 | 0.686 (0.660-0.711) | 0.022 | 1.64×10^-13^ | 0.682 (0.655-0.710) | 0.018 | 0.021 | 14.7% (8.8%-18.8%) |
| Cervix | 210 | 57,122 | Model 1 | 0.540 (0.505-0.575) | - | - | 0.540 (0.503-0.578) | - | - |  |
|  |  |  | Model 2 | 0.572 (0.534-0.611) | 0.032 | 0.015 | 0.571 (0.531-0.612) | 0.031 | 0.056 | 5.7% (-1.1%-11.0%) |
|  |  |  | Model 3 | 0.594 (0.556-0.633) | 0.022 | 0.006 | 0.594 (0.553-0.635) | 0.023 | 0.109 | 10.3% (2.0%-16.6%) |
| Ovary | 80 | 57,263 | Model 1 | 0.557 (0.499-0.614) | - | - | 0.544 (0.480-0.608) | - | - |  |
|  |  |  | Model 2 | 0.635 (0.574-0.697) | 0.078 | 2.87×10^-04^ | 0.653 (0.587-0.719) | 0.109 | 0.002 | 19.9% (2.2%-30.4%) |
|  |  |  | Model 3 | 0.651 (0.590-0.712) | 0.016 | 0.048 | 0.674 (0.608-0.739) | 0.020 | 0.179 | 12.6% (0.0%-21.7%) |
| Prostate | 86 | 42,765 | Model 1 | 0.834 (0.797-0.871) | - | - | 0.804 (0.760-0.847) | - | - |  |
|  |  |  | Model 2 | 0.835 (0.797-0.873) | 0.001 | 0.047 | 0.804 (0.760-0.849) | 0.001 | 0.825 | 11.7% (-1.9%-18.1%) |
|  |  |  | Model 3 | 0.849 (0.811-0.886) | 0.014 | 5.02×10^-07^ | 0.823 (0.779-0.867) | 0.018 | 0.053 | 18.5% (9.7%-30.1%) |

PRS, polygenic risk score; CI, confidence interval; AUC, area under the curve; NRI, net reclassification improvement.

^*^ Model 1: Including demographic factors (age, sex, and region) and family history of cancer; Model 2: Adding summarized modifiable risk factors to Model 1; Model 3: Adding PRS to Model 2.

^†^ ΔC was the C-index difference between Model 2 and Model 1, as well as between Model 3 and Model 2; so was ΔAUC.

^‡^ The likelihood-ratio test was performed between Model 2 and Model 1, as well as between Model 3 and Model 2; so was the DeLong test.
