# Supplementary material for: Polygenic risk scores for pan-cancer risk prediction in the Chinese population: A population-based cohort study based on the China Kadoorie Biobank
Source: PLoS Med. 2025 Feb 28;22(2):e1004534. doi: 10.1371/journal.pmed.1004534 (PMC11870365; doi:10.1371/journal.pmed.1004534)
Supplement: S22 Table — AUC, area under the curve; CI, confidence interval. (DOCX) [file pmed.1004534.s026.docx]

**S22 Table. Different approaches to construct predictive models based on modifiable risk factors and polygenic risk scores**

| **Cancer site** | **Cases** | **Non-cases** | **Model specification** | **10-year AUC (95% CI)** | ***P_*DeLong** |
| --- | --- | --- | --- | --- | --- |
| Esophagus | 499 | 99,720 | Add direct terms | 0.811 (0.794-0.829) | - |
|  |  |  | Add interaction terms | 0.811 (0.793-0.829) | 0.728 |
|  |  |  | Add non-linear terms | 0.812 (0.794-0.829) | 0.915 |
|  |  |  | Flexible parametric model | 0.811 (0.794-0.829) | 0.980 |
| Stomach | 745 | 99,474 | Add direct terms | 0.734 (0.716-0.752) | - |
|  |  |  | Add interaction terms | 0.734 (0.716-0.752) | 0.743 |
|  |  |  | Add non-linear terms | 0.734 (0.716-0.752) | 0.378 |
|  |  |  | Flexible parametric model | 0.734 (0.716-0.752) | 0.941 |
| Colorectum | 740 | 99,479 | Add direct terms | 0.731 (0.712-0.749) | - |
|  |  |  | Add interaction terms | 0.731 (0.712-0.750) | 0.548 |
|  |  |  | Add non-linear terms | 0.732 (0.713-0.751) | 0.270 |
|  |  |  | Flexible parametric model | 0.731 (0.712-0.749) | 0.688 |
| Pancreas | 170 | 100,049 | Add direct terms | 0.748 (0.705-0.791) | - |
|  |  |  | Add interaction terms | 0.748 (0.705-0.791) | 0.935 |
|  |  |  | Add non-linear terms | 0.753 (0.710-0.795) | 0.129 |
|  |  |  | Flexible parametric model | 0.748 (0.705-0.791) | 0.997 |
| Lung | 1,540 | 98,679 | Add direct terms | 0.759 (0.746-0.771) | - |
|  |  |  | Add interaction terms | 0.760 (0.748-0.772) | 0.023 |
|  |  |  | Add non-linear terms | 0.760 (0.748-0.772) | 0.028 |
|  |  |  | Flexible parametric model | 0.759 (0.746-0.771) | 0.415 |
| Breast | 486 | 56,873 | Add direct terms | 0.684 (0.658-0.710) | - |
|  |  |  | Add interaction terms | 0.684 (0.658-0.710) | 0.784 |
|  |  |  | Add non-linear terms | 0.685 (0.659-0.711) | 0.212 |
|  |  |  | Flexible parametric model | 0.684 (0.658-0.710) | 0.620 |
| Cervix | 237 | 57,122 | Add direct terms | 0.596 (0.558-0.635) | - |
|  |  |  | Add interaction terms | 0.599 (0.561-0.638) | 0.657 |
|  |  |  | Add non-linear terms | 0.608 (0.570-0.647) | 0.145 |
|  |  |  | Flexible parametric model | 0.596 (0.558-0.635) | 0.847 |
| Ovary | 96 | 57,263 | Add direct terms | 0.662 (0.602-0.722) | - |
|  |  |  | Add interaction terms | 0.663 (0.602-0.723) | 0.458 |
|  |  |  | Add non-linear terms | 0.671 (0.612-0.731) | 0.276 |
|  |  |  | Flexible parametric model | 0.662 (0.602-0.722) | 0.525 |
| Prostate | 95 | 42,765 | Add direct terms | 0.829 (0.788-0.870) | - |
|  |  |  | Add interaction terms | 0.829 (0.787-0.870) | 0.616 |
|  |  |  | Add non-linear terms | 0.828 (0.787-0.870) | 0.788 |
|  |  |  | Flexible parametric model | 0.829 (0.787-0.870) | 0.644 |

AUC, area under the curve; CI, confidence interval.
