# Supplementary material for: Polygenic risk scores for pan-cancer risk prediction in the Chinese population: A population-based cohort study based on the China Kadoorie Biobank
Source: PLoS Med. 2025 Feb 28;22(2):e1004534. doi: 10.1371/journal.pmed.1004534 (PMC11870365; doi:10.1371/journal.pmed.1004534)
Supplement: S23 Table — ERR, explained relative risk; PRS, polygenic risk score; CI, confidence interval. (DOCX) [file pmed.1004534.s027.docx]

**S23 Table.** **Explained relative risk for the polygenic risk scores and summarized risk factors for each cancer type in the CKB cohort**

| **Cancer site** | **Genetic risk** | | |  | **Risk factors** | | |  | **Genetic risk + Risk factors** | |
| --- | --- | --- | --- | --- | --- | --- | --- | --- | --- | --- |
|  | **ERR ^*^** | **(95% CI) ^*^** | **Proportion, %** |  | **ERR ^*^** | **(95% CI) ^*^** | **Proportion, %** |  | **ERR ^*^** | **(95% CI) ^*^** |
| Esophagus | 0.026 | (0.007-0.050) | 13.47% |  | 0.167 | (0.103-0.231) | 86.53% |  | 0.193 | (0.127-0.258) |
| Stomach | 0.048 | (0.024-0.080) | 45.71% |  | 0.056 | (0.025-0.092) | 53.33% |  | 0.105 | (0.068-0.148) |
| Colorectum | 0.132 | (0.090-0.175) | 83.02% |  | 0.029 | (0.010-0.059) | 18.24% |  | 0.159 | (0.116-0.206) |
| Pancreas | 0.047 | (0.011-0.103) | 38.21% |  | 0.075 | (0.020-0.151) | 60.98% |  | 0.123 | (0.054-0.210) |
| Lung | 0.027 | (0.015-0.042) | 14.29% |  | 0.162 | (0.131-0.199) | 85.71% |  | 0.189 | (0.157-0.224) |
| Breast | 0.082 | (0.049-0.122) | 36.94% |  | 0.139 | (0.091-0.188) | 62.61% |  | 0.222 | (0.170-0.280) |
| Cervix | 0.031 | (0.005-0.078) | 57.41% |  | 0.023 | (0.002-0.070) | 42.59% |  | 0.054 | (0.016-0.119) |
| Ovary | 0.035 | (0.001-0.112) | 21.34% |  | 0.130 | (0.035-0.263) | 79.27% |  | 0.164 | (0.066-0.310) |
| Prostate | 0.203 | (0.082-0.326) | 81.20% |  | 0.046 | (0.001-0.191) | 18.40% |  | 0.250 | (0.142-0.387) |

ERR, explained relative risk; PRS, polygenic risk score; CI, confidence interval.

^*^ ERR estimates were derived from Cox proportional hazard regression models that were adjusted for age, sex (if applicable), region, and family cancer history. The confidence intervals for ERR were estimated using 1000 bootstrapped iterations.
