# Supplementary material for: Polygenic risk scores for pan-cancer risk prediction in the Chinese population: A population-based cohort study based on the China Kadoorie Biobank
Source: PLoS Med. 2025 Feb 28;22(2):e1004534. doi: 10.1371/journal.pmed.1004534 (PMC11870365; doi:10.1371/journal.pmed.1004534)

**S1 Fig. Distribution of the nine optimal polygenic risk scores for each cancer type in the CKB cohort.** PRS, polygenic risk score; CKB, China Kadoorie Biobank.


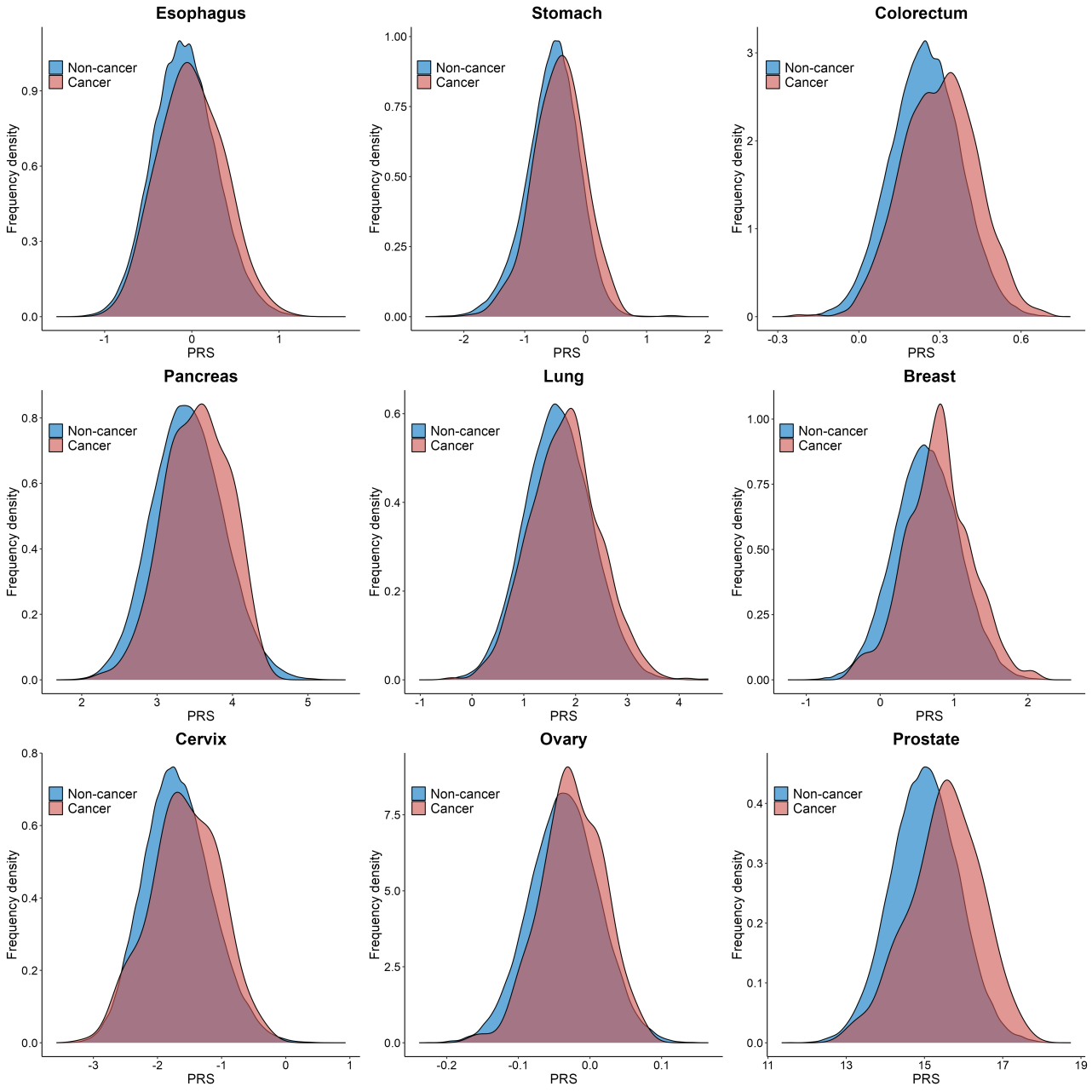

Supplement: S1 Fig — PRS, polygenic risk score; CKB, China Kadoorie Biobank. (DOCX) [file pmed.1004534.s028.docx]
