# Supplementary material for: Polygenic risk scores for pan-cancer risk prediction in the Chinese population: A population-based cohort study based on the China Kadoorie Biobank
Source: PLoS Med. 2025 Feb 28;22(2):e1004534. doi: 10.1371/journal.pmed.1004534 (PMC11870365; doi:10.1371/journal.pmed.1004534)

**S2 Fig. The association of polygenic risk scores with individual cancer in the CKB cohort.** Participants in the CKB cohort were divided into five equal groups according to their polygenic risk scores, and the HRs for each group were compared with those in quintile 1 (HR 1.0 [ref]) of the polygenic risk score with the adjustment of age, sex (if applicable), region, and the top 10 principal components. The error bars represent 95% CIs and their centers represent the HRs. PRS, polygenic risk score; CKB, China Kadoorie Biobank; HR, hazard ratio; CI, confidence interval.


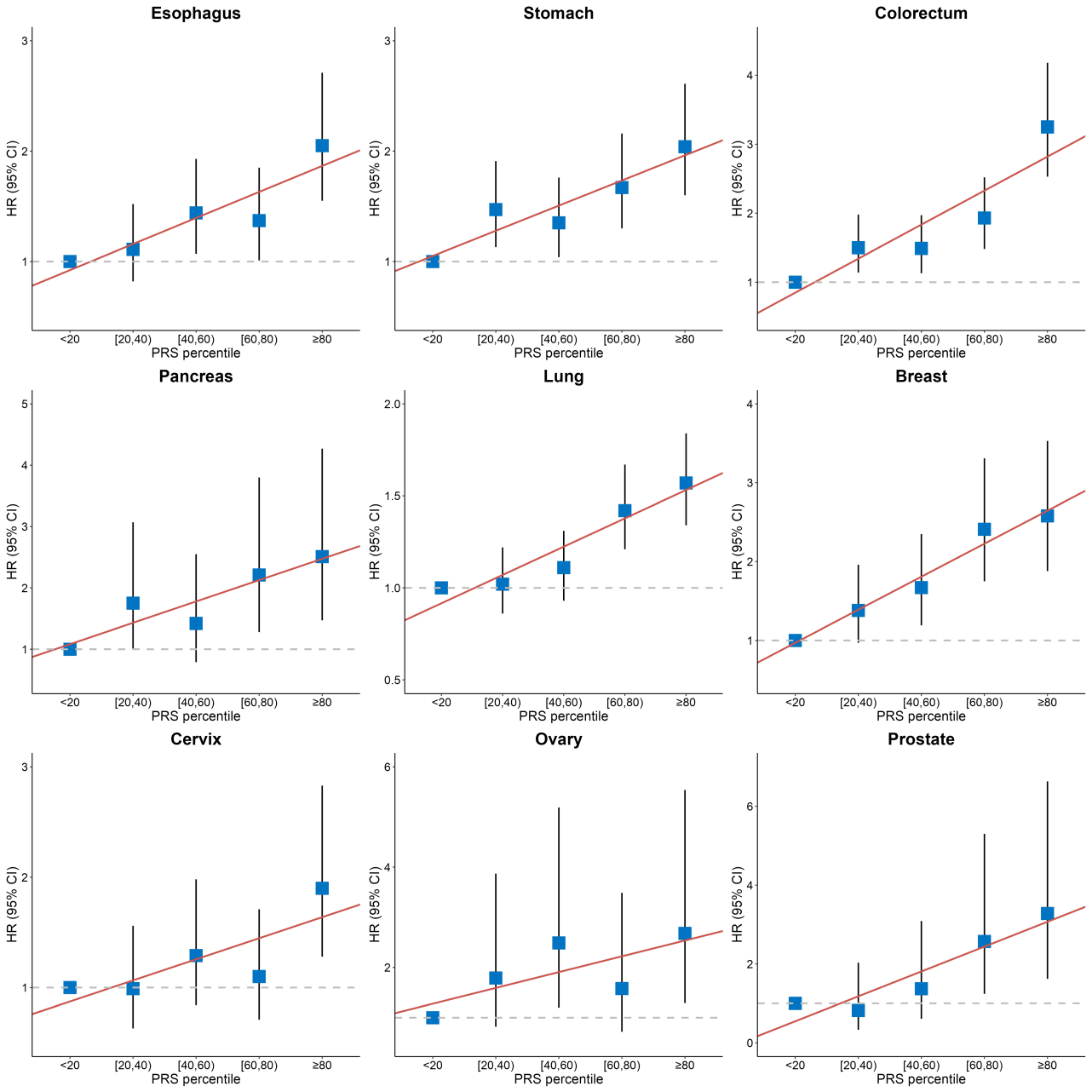

Supplement: S2 Fig — Participants in the CKB cohort were divided into five equal groups according to their polygenic risk scores, and the HRs for each group were compared with those in quintile 1 (HR 1.0 [ref]) of the polygenic risk score with the adjustment of age, sex (if applicable), region, and the top 10 principal components. The error bars represent 95% CIs and their centers represent the HRs. PRS, polygenic risk score; CKB, China Kadoorie Biobank; HR, hazard ratio; CI, confidence interval. (DOCX) [file pmed.1004534.s029.docx]
