# Supplementary material for: Polygenic risk scores for pan-cancer risk prediction in the Chinese population: A population-based cohort study based on the China Kadoorie Biobank
Source: PLoS Med. 2025 Feb 28;22(2):e1004534. doi: 10.1371/journal.pmed.1004534 (PMC11870365; doi:10.1371/journal.pmed.1004534)

**S3 Fig. The adjusted cumulative incidence curves across strata defined by polygenic risk score.** Low polygenic risk score (PRS) corresponds to the bottom quintile, medium PRS is defined as quintile 2-4, and high PRS includes individuals in the top quintile in the CKB cohort. Cumulative incidence was estimated using Cox regression models with the adjustment of age, sex (if applicable), region, and the top 10 principal components. PRS, polygenic risk score; CKB, China Kadoorie Biobank.


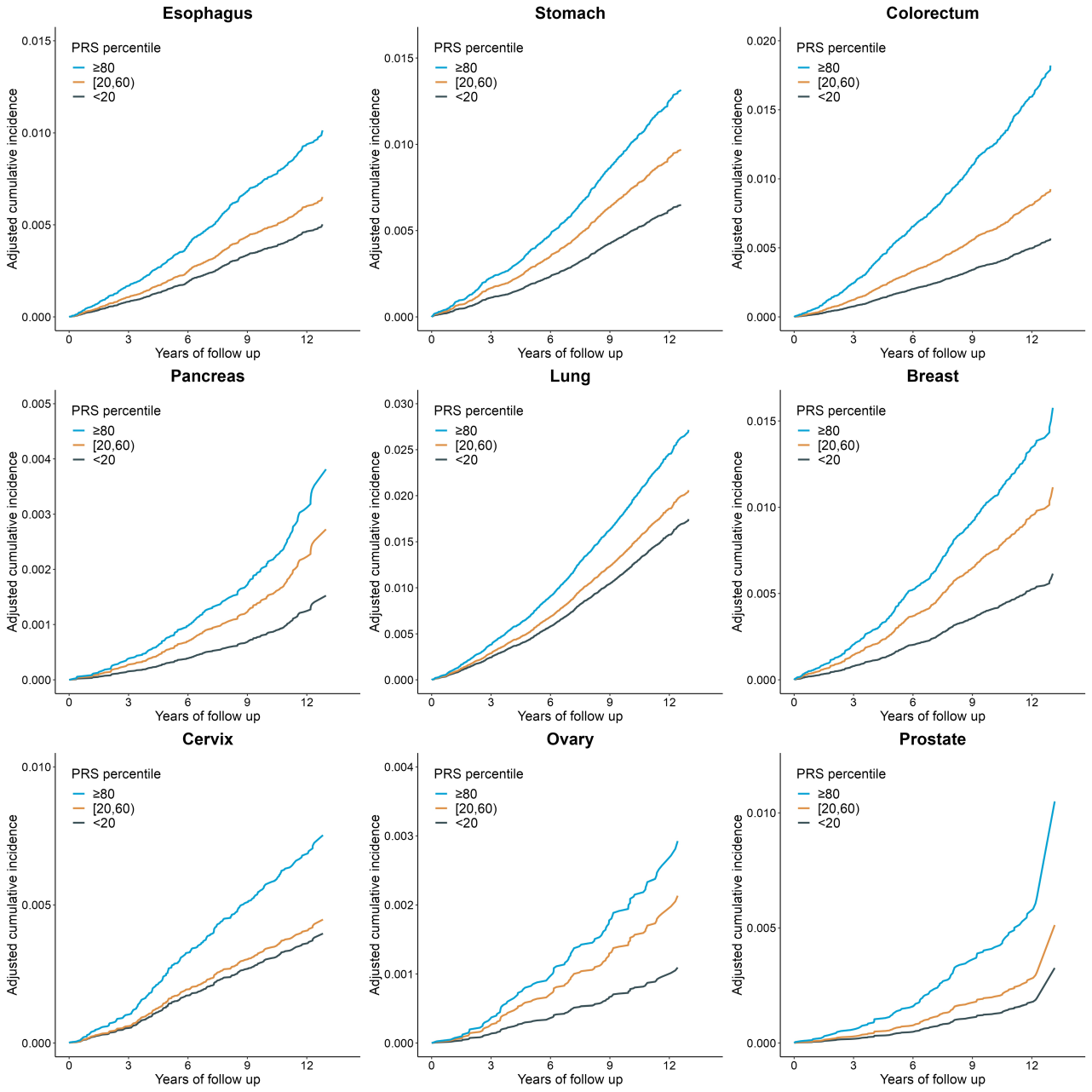

Supplement: S3 Fig — Low polygenic risk score (PRS) corresponds to the bottom quintile, medium PRS is defined as quintile 2–4, and high PRS includes individuals in the top quintile in the CKB cohort. Cumulative incidence was estimated using Cox regression models with the adjustment of age, sex (if applicable), region, and the top 10 principal components. PRS, polygenic risk score; CKB, China Kadoorie Biobank. (DOCX) [file pmed.1004534.s030.docx]
