# Supplementary material for: Polygenic risk scores for pan-cancer risk prediction in the Chinese population: A population-based cohort study based on the China Kadoorie Biobank
Source: PLoS Med. 2025 Feb 28;22(2):e1004534. doi: 10.1371/journal.pmed.1004534 (PMC11870365; doi:10.1371/journal.pmed.1004534)

**S4 Fig. Correlation heatmap among the nine optimal cancer-specific polygenic risk scores.** The significance levels in the figure are denoted by asterisks as follows: *: *P*-value<0.05, **: *P*-value<0.01, and ***: *P*-value<0.001. PRS, polygenic risk score.


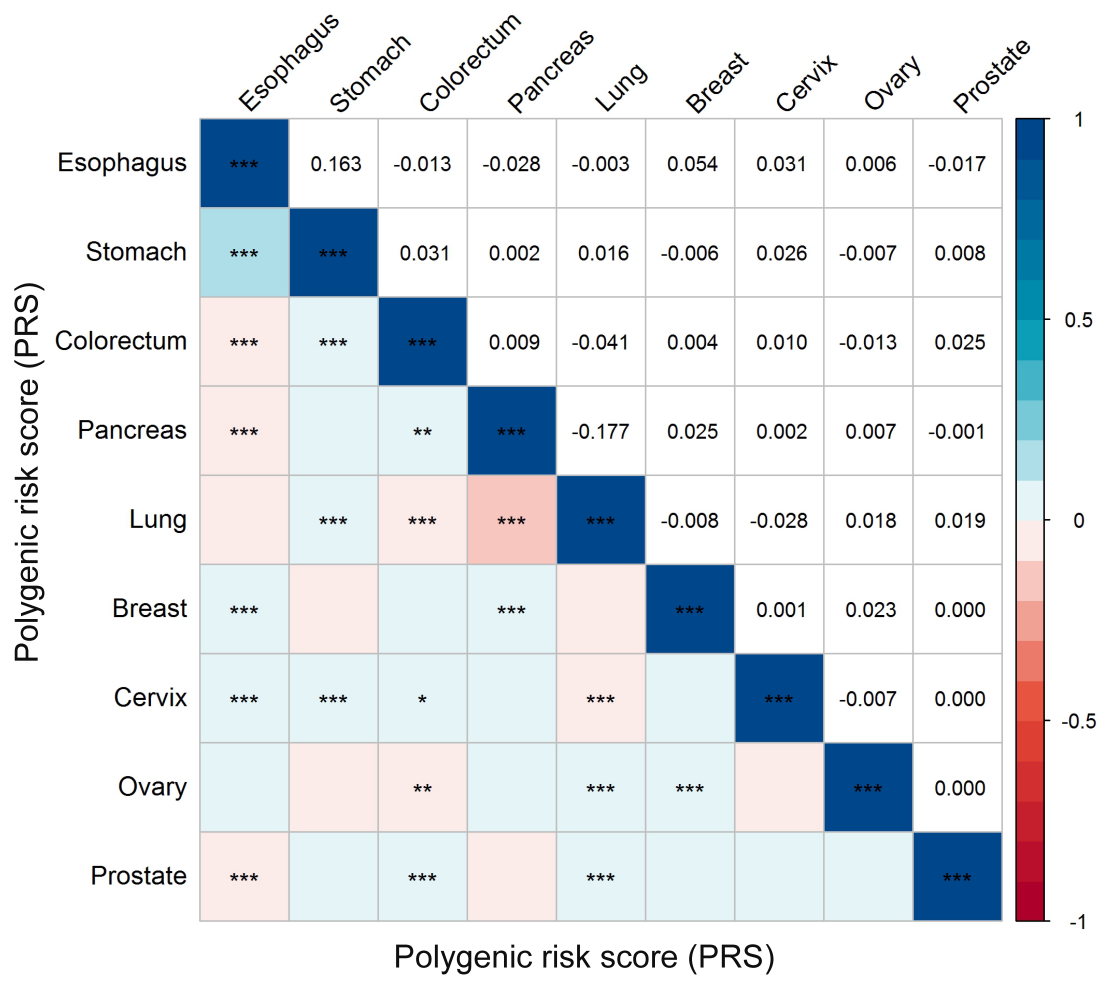

Supplement: S4 Fig — The significance levels in the figure are denoted by asterisks as follows: * P-value < 0.05, **P-value < 0.01, and ***P-value < 0.001. PRS, polygenic risk score. (DOCX) [file pmed.1004534.s031.docx]
