# Supplementary material for: Polygenic risk scores for pan-cancer risk prediction in the Chinese population: A population-based cohort study based on the China Kadoorie Biobank
Source: PLoS Med. 2025 Feb 28;22(2):e1004534. doi: 10.1371/journal.pmed.1004534 (PMC11870365; doi:10.1371/journal.pmed.1004534)

**S5 Fig. Hazard ratios between cancer-specific polygenic risk scores and other cancer outcomes.** HRs were estimated using a Cox regression model adjusted for age, sex (if applicable), region, and the top 10 principal components (left) and they were further adjusted for the corresponding site-specific PRSs (right). The significance levels in the figure are denoted by asterisks as follows: *: *P*-value<0.05, **: *P*-value<0.01, and ***: *P*-value<0.001. PRS, polygenic risk score; HR, hazard ratio.


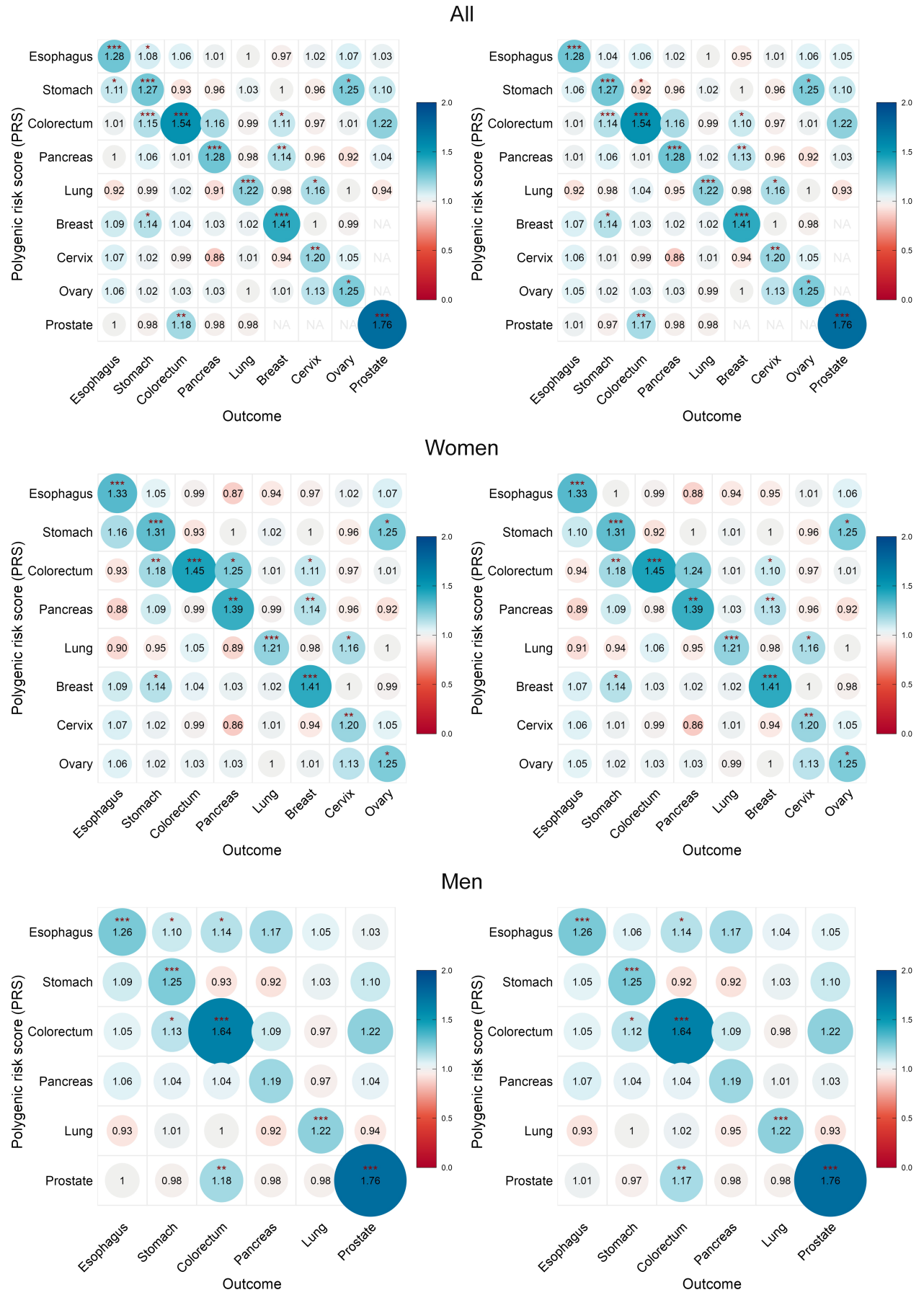

Supplement: S5 Fig — HRs were estimated using a Cox regression model adjusted for age, sex (if applicable), region, and the top 10 principal components (left) and they were further adjusted for the corresponding site-specific PRSs (right). The significance levels in the figure are denoted by asterisks as follows: * P-value < 0.05, **P-value < 0.01, and ***P-value < 0.001. PRS, polygenic risk score; HR, hazard ratio. (DOCX) [file pmed.1004534.s032.docx]
