# Supplementary material for: Polygenic risk scores for pan-cancer risk prediction in the Chinese population: A population-based cohort study based on the China Kadoorie Biobank
Source: PLoS Med. 2025 Feb 28;22(2):e1004534. doi: 10.1371/journal.pmed.1004534 (PMC11870365; doi:10.1371/journal.pmed.1004534)

**S6 Fig. Hazard ratios for the significant cross-cancer associations after excluding shared SNPs or SNPs in high linkage disequilibrium (r^2^>0.6) with those in the polygenic risk score of outcome cancer type.** HRs were estimated using a Cox regression model adjusted for age, sex (if applicable), region, and the top 10 principal components. The adjusted HRs were further adjusted for the corresponding site-specific PRSs. No shared SNPs were found for stomach cancer PRS and ovarian cancer PRS, lung cancer PRS and cervical cancer PRS, and breast cancer PRS and stomach cancer PRS. The error bars represent 95% CIs and their centers represent the HRs. SNP, single nucleotide polymorphism; PRS, polygenic risk score; HR, hazard ratio; CI, confidence interval.


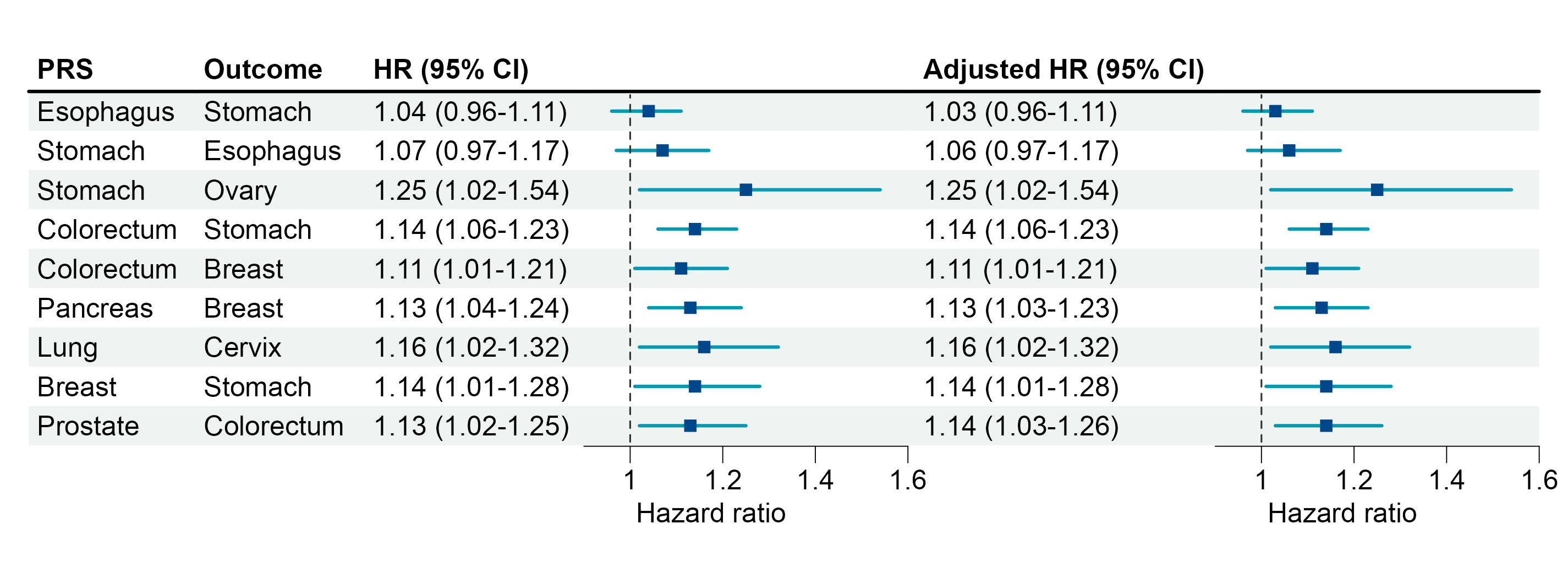

Supplement: S6 Fig — HRs were estimated using a Cox regression model adjusted for age, sex (if applicable), region, and the top 10 principal components. The adjusted HRs were further adjusted for the corresponding site-specific PRSs. No shared SNPs were found for stomach cancer PRS and ovarian cancer PRS, lung cancer PRS and cervical cancer PRS, and breast cancer PRS and stomach cancer PRS. The error bars represent 95% CIs and their centers represent the HRs. SNP, single-nucleotide polymorphism; PRS, polygenic risk score; HR, hazard ratio; CI, confidence interval. (DOCX) [file pmed.1004534.s033.docx]
