# Supplementary material for: Polygenic risk scores for pan-cancer risk prediction in the Chinese population: A population-based cohort study based on the China Kadoorie Biobank
Source: PLoS Med. 2025 Feb 28;22(2):e1004534. doi: 10.1371/journal.pmed.1004534 (PMC11870365; doi:10.1371/journal.pmed.1004534)

**S7 Fig. Cumulative risk by age 80 of the nine cancers across strata defined by polygenic risk scores and modifiable risk factors.** Low PRS corresponds to the bottom quintile, medium PRS is defined as quintile 2-4, and high PRS includes individuals in the top quintile in the CKB cohort. Individuals above the median of risk factors risk score distribution were considered to have an elevated risk profile, whereas those below the median had reduced risk. Death from any cause was treated as a competing event. RF, modifiable risk factors; PRS, polygenic risk score.


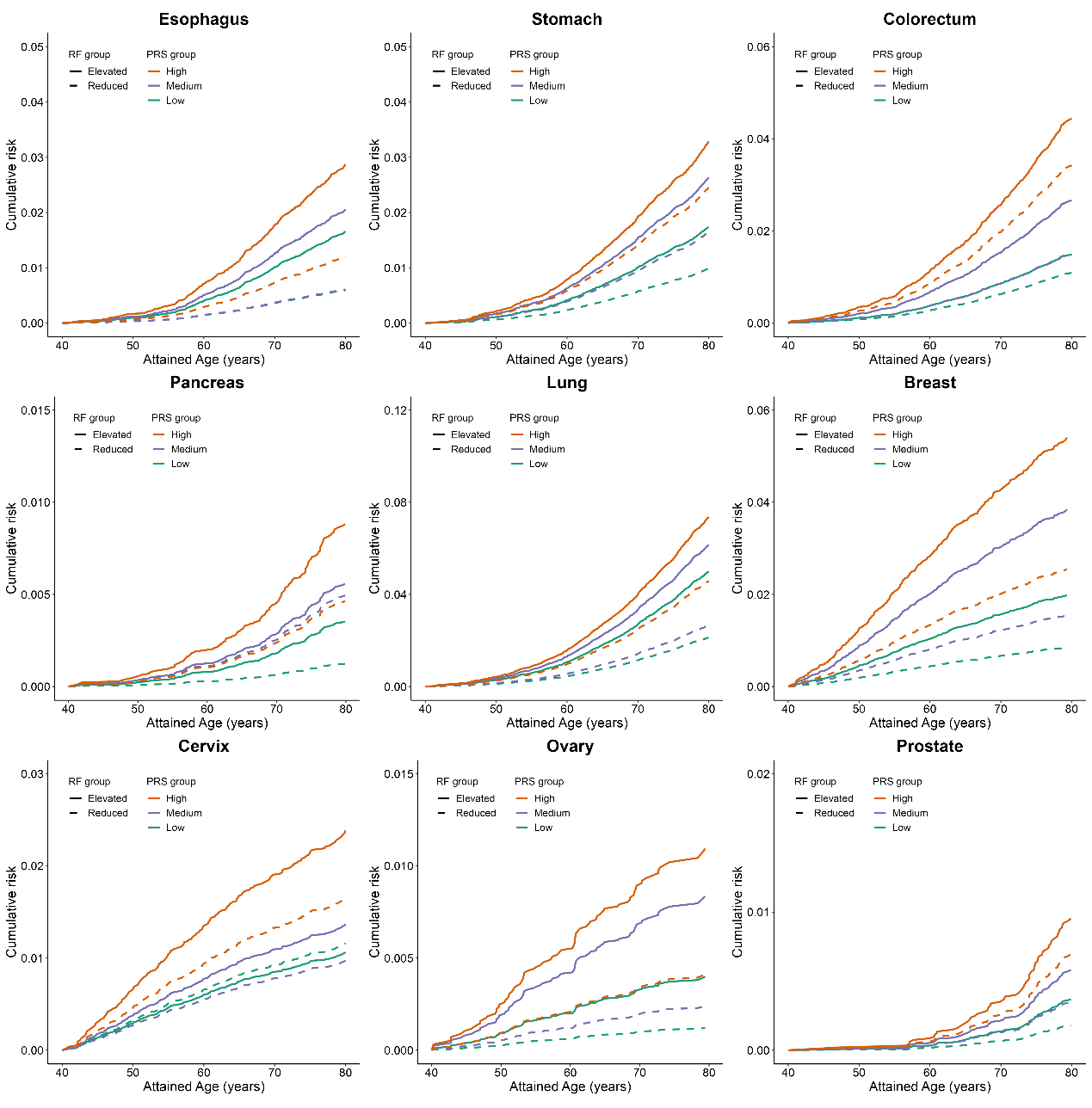

Supplement: S7 Fig — Low PRS corresponds to the bottom quintile, medium PRS is defined as quintile 2–4, and high PRS includes individuals in the top quintile in the CKB cohort. Individuals above the median of risk factors risk score distribution were considered to have an elevated risk profile, whereas those below the median had reduced risk. Death from any cause was treated as a competing event. RF, modifiable risk factors; PRS, polygenic risk score. (DOCX) [file pmed.1004534.s034.docx]
