# Supplementary material for: Polygenic risk scores for pan-cancer risk prediction in the Chinese population: A population-based cohort study based on the China Kadoorie Biobank
Source: PLoS Med. 2025 Feb 28;22(2):e1004534. doi: 10.1371/journal.pmed.1004534 (PMC11870365; doi:10.1371/journal.pmed.1004534)

**S8 Fig. Boxplots of the 10-year absolute risk across strata defined by polygenic risk scores, modifiable risk factors and age categories.** Low PRS corresponds to the bottom quintile, medium PRS is defined as quintile 2-4, and high PRS includes individuals in the top quintile in the CKB cohort. Individuals above the median of risk factors risk score distribution were considered to have an elevated risk profile, whereas those below the median had reduced risk. The box limits represent inter-quartile ranges (IQR) and their centers represent the medians of the absolute risk. The boundaries of the whiskers are based on the 1.5*IQR value and other observed points outside the boundary of the whiskers are plotted as outliers. RF, modifiable risk factors; PRS, polygenic risk score; CKB, China Kadoorie Biobank; IQR, interquartile range.


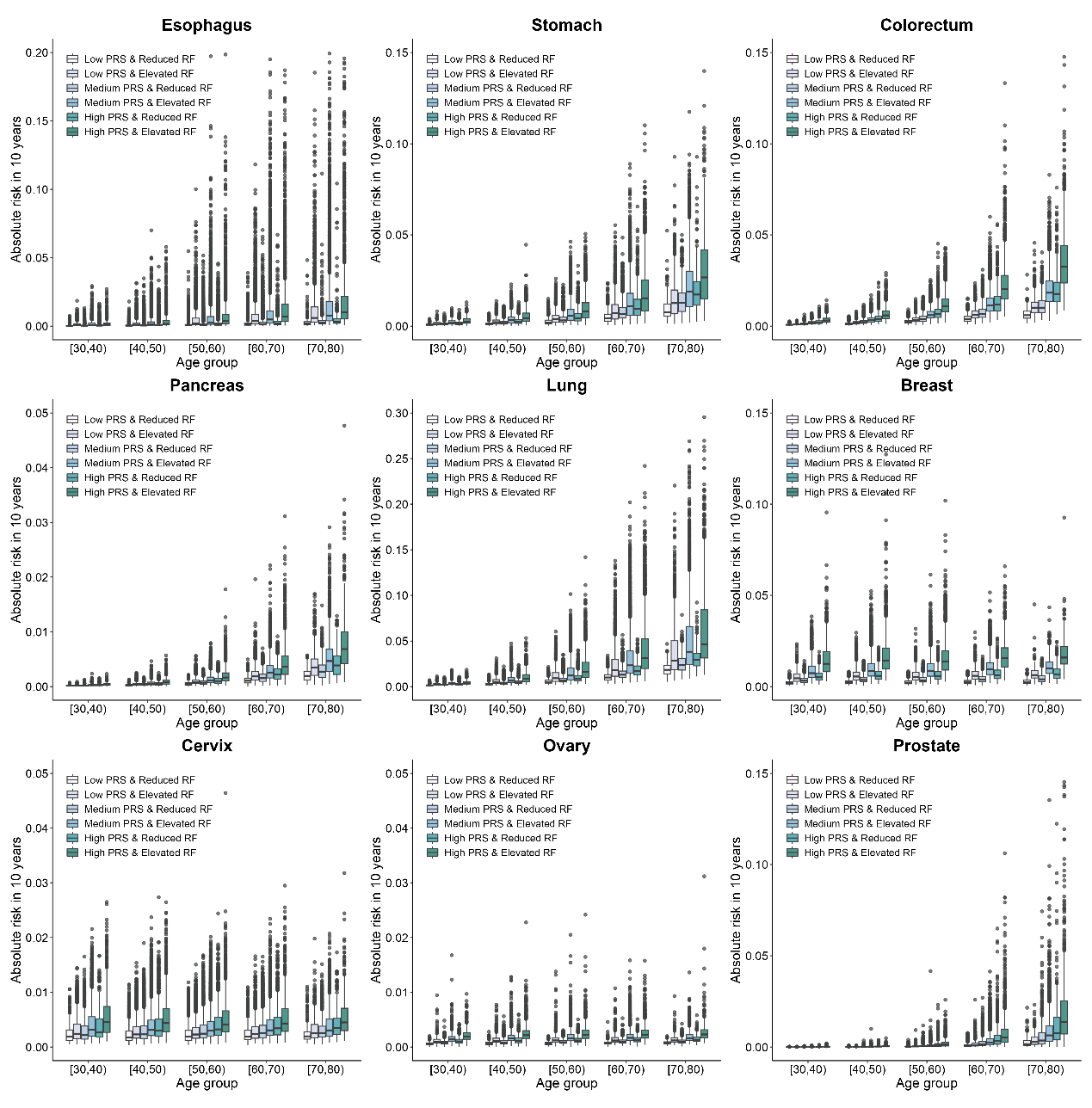

Supplement: S8 Fig — Low PRS corresponds to the bottom quintile, medium PRS is defined as quintile 2–4, and high PRS includes individuals in the top quintile in the CKB cohort. Individuals above the median of risk factors risk score distribution were considered to have an elevated risk profile, whereas those below the median had reduced risk. The box limits represent interquartile ranges (IQRs) and their centers represent the medians of the absolute risk. The boundaries of the whiskers are based on the 1.5 * IQR value and other observed points outside the boundary of the whiskers are plotted as outliers. RF, modifiable risk factors; PRS, polygenic risk score; CKB, China Kadoorie Biobank; IQR, interquartile range. (DOCX) [file pmed.1004534.s035.docx]
