# Supplementary material for: Polygenic risk scores for pan-cancer risk prediction in the Chinese population: A population-based cohort study based on the China Kadoorie Biobank
Source: PLoS Med. 2025 Feb 28;22(2):e1004534. doi: 10.1371/journal.pmed.1004534 (PMC11870365; doi:10.1371/journal.pmed.1004534)

**S9 Fig. 10-year absolute risk reduction across strata defined by genetic risk and modifiable risk factors**. Low PRS corresponds to the bottom quintile, medium PRS is defined as quintile 2-4, and high PRS includes individuals in the top quintile in the CKB cohort. Individuals above the median of risk factors risk score distribution were considered to have an elevated risk profile, whereas those below the median had reduced risk. The error bars represent interquartile ranges and their centers represent the medians of the absolute risk. RF, modifiable risk factors; PRS, polygenic risk score; ARR, absolute risk reduction; CKB, China Kadoorie Biobank.


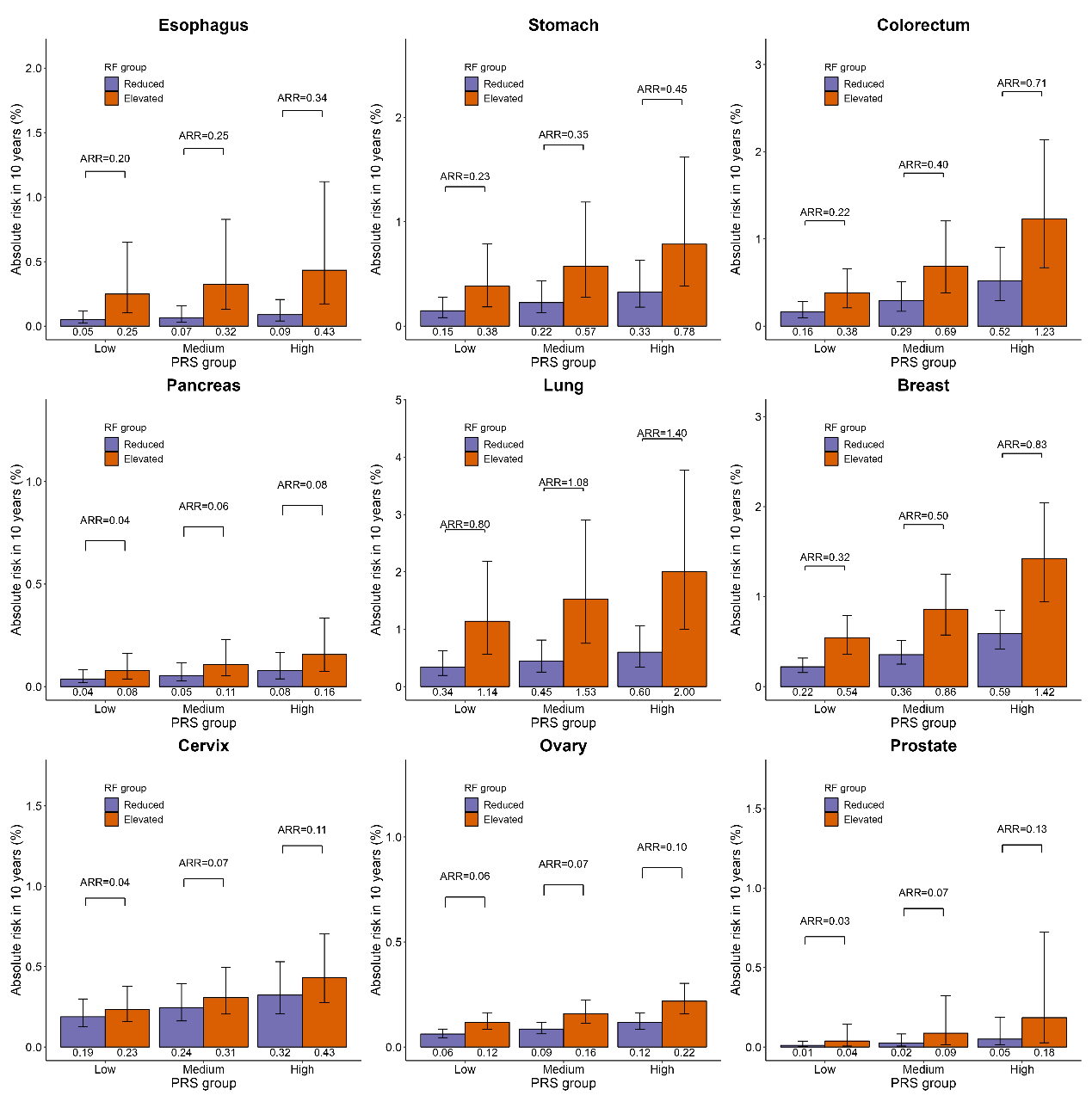

Supplement: S9 Fig — Low PRS corresponds to the bottom quintile, medium PRS is defined as quintile 2–4, and high PRS includes individuals in the top quintile in the CKB cohort. Individuals above the median of risk factors risk score distribution were considered to have an elevated risk profile, whereas those below the median had reduced risk. The error bars represent interquartile ranges and their centers represent the medians of the absolute risk. RF, modifiable risk factors; PRS, polygenic risk score; ARR, absolute risk reduction; CKB, China Kadoorie Biobank. (DOCX) [file pmed.1004534.s036.docx]
