# Supplementary material for: Polygenic risk scores for pan-cancer risk prediction in the Chinese population: A population-based cohort study based on the China Kadoorie Biobank
Source: PLoS Med. 2025 Feb 28;22(2):e1004534. doi: 10.1371/journal.pmed.1004534 (PMC11870365; doi:10.1371/journal.pmed.1004534)
Supplement: S10 Fig — The C-index was from the PRS-only model of each cancer. The heritability estimates come from previous articles, mainly from two twin studies. (DOCX) [file pmed.1004534.s037.docx]

**S10 Fig. The correlation between the C-index and the heritability estimates.** The C-index was from the PRS-only model of each cancer. The heritability estimates come from previous articles, mainly from two twin studies [1-4].

**
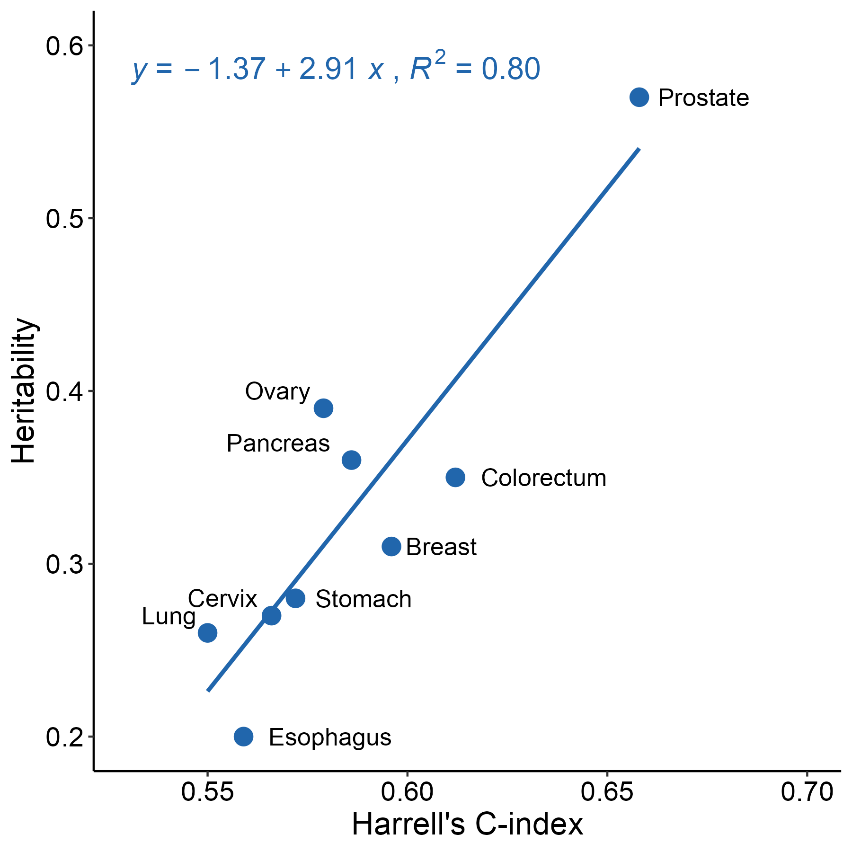
**

**References**

1. Lichtenstein P, Holm NV, Verkasalo PK, Iliadou A, Kaprio J, Koskenvuo M, et al. Environmental and heritable factors in the causation of cancer--analyses of cohorts of twins from Sweden, Denmark, and Finland. N Engl J Med. 2000;343(2):78-85. PMID: 10891514.

2. Mucci LA, Hjelmborg JB, Harris JR, Czene K, Havelick DJ, Scheike T, et al. Familial Risk and Heritability of Cancer Among Twins in Nordic Countries. JAMA. 2016;315(1):68-76. doi: 10.1001/jama.2015.17703. PMID: 26746459.

3. Dai J, Shen W, Wen W, Chang J, Wang T, Chen H, et al. Estimation of heritability for nine common cancers using data from genome-wide association studies in Chinese population. Int J Cancer. 2017;140(2):329-36. doi: 10.1002/ijc.30447. PMID: 27668986.

4. Magnusson PK, Lichtenstein P, Gyllensten UB. Heritability of cervical tumours. Int J Cancer. 2000;88(5):698-701. PMID: 11072236.
