# Supplementary material for: Polygenic risk scores for pan-cancer risk prediction in the Chinese population: A population-based cohort study based on the China Kadoorie Biobank
Source: PLoS Med. 2025 Feb 28;22(2):e1004534. doi: 10.1371/journal.pmed.1004534 (PMC11870365; doi:10.1371/journal.pmed.1004534)
Supplement: S1 Data — (PDF) [file pmed.1004534.s039.pdf]

Cancer site	PRS class	SNP rsID	CHR	Position (GRCh37)	Effect allele	Other allele	Beta	Study (PMID)
Esophagus	PRS3	rs13016963	2	202162811	G	A	-0.236	25129146
Esophagus	PRS3	rs2239612	3	186793242	A	G	0.113	25129146
Esophagus	PRS3	rs10052657	5	58407771	A	C	-0.223	25129146
Esophagus	PRS3	rs7447927	5	138861146	G	C	-0.163	25129146
Esophagus	PRS3	rs35597309	6	32589266	A	G	0.285	25129146
Esophagus	PRS3	rs10484761	6	40802261	C	T	0.131	25129146
Esophagus	PRS3	rs2274223	10	96066341	G	A	0.300	25129146
Esophagus	PRS3	rs4785204	16	50103734	T	C	0.140	25129146
Esophagus	PRS3	rs1642764	17	7557834	T	C	-0.128	25129146
Esophagus	PRS3	rs2014300	21	36357861	G	A	0.247	25129146
Esophagus	PRS3	rs2239815	22	29192670	C	T	-0.151	25129146
Stomach	PRS2	rs7366775	1	155168930	A	G	0.344	33002439
Stomach	PRS2	rs138554234	1	156089034	A	G	-0.446	33002439
Stomach	PRS2	rs11937064	4	125457123	A	G	-0.139	33002439
Stomach	PRS2	rs10074991	5	40790551	A	G	-0.248	33002439
Stomach	PRS2	rs28749114	6	29905452	A	G	0.131	33002439
Stomach	PRS2	rs10105842	8	143671615	T	C	-0.139	33002439
Stomach	PRS2	rs2585177	8	143776717	A	C	-0.223	33002439
Stomach	PRS2	rs10509670	10	96067947	A	G	-0.248	33002439
Stomach	PRS2	rs1050437	6	31239585	T	C	-0.111	34594039
Stomach	PRS2	rs9368777	6	33788637	C	G	0.176	34594039
Stomach	PRS2	rs375852527	13	81854872	C	T	1.290	34594039
Stomach	PRS2	rs1205528	X	108542014	C	T	-0.089	34594039
Colorectum	PRS7	rs10000081	4	17348363	C	T	-4.64E-04	PRS-CSx
Colorectum	PRS7	rs1000025	6	134085319	T	C	4.14E-04	PRS-CSx
Colorectum	PRS7	rs10000988	4	76010255	T	C	8.58E-04	PRS-CSx
Colorectum	PRS7	rs1000113	5	150240076	T	C	-5.01E-04	PRS-CSx
Colorectum	PRS7	rs1000140	5	129377047	G	A	-1.60E-04	PRS-CSx
Colorectum	PRS7	rs1000198	3	119113820	C	A	2.68E-04	PRS-CSx
Colorectum	PRS7	rs10002657	4	115404276	G	A	1.01E-04	PRS-CSx
Colorectum	PRS7	rs10003909	4	86915848	C	T	-4.67E-04	PRS-CSx
Colorectum	PRS7	rs10008568	4	89854192	G	A	2.09E-04	PRS-CSx
Colorectum	PRS7	rs10008902	4	140569503	C	A	-6.73E-04	PRS-CSx
Colorectum	PRS7	rs10010699	4	69810824	C	A	-1.59E-04	PRS-CSx
Colorectum	PRS7	rs10012087	4	151474920	C	T	1.25E-04	PRS-CSx
Colorectum	PRS7	rs10012313	4	17405513	A	G	1.84E-03	PRS-CSx
Colorectum	PRS7	rs10012720	4	151772410	G	A	5.57E-04	PRS-CSx
Colorectum	PRS7	rs1001348	7	45111943	C	A	-3.42E-04	PRS-CSx
Colorectum	PRS7	rs1001484	12	111536822	A	G	5.44E-05	PRS-CSx
Colorectum	PRS7	rs10019843	4	69786431	T	C	-9.04E-05	PRS-CSx
Colorectum	PRS7	rs10020962	4	130206071	G	T	-2.25E-04	PRS-CSx
Colorectum	PRS7	rs1002115	5	61039870	G	A	-9.14E-04	PRS-CSx
Colorectum	PRS7	rs10022844	4	151499440	G	A	6.04E-04	PRS-CSx
Colorectum	PRS7	rs10024717	4	89219645	A	G	1.33E-03	PRS-CSx
Colorectum	PRS7	rs10025001	4	14879929	T	C	1.86E-04	PRS-CSx
Colorectum	PRS7	rs10025675	4	118796265	T	G	1.01E-03	PRS-CSx
Colorectum	PRS7	rs10026528	4	105857952	G	A	-1.09E-03	PRS-CSx
Colorectum	PRS7	rs10027139	4	105937236	T	C	4.01E-04	PRS-CSx
Colorectum	PRS7	rs10027390	4	123368516	C	T	3.12E-04	PRS-CSx
Colorectum	PRS7	rs10030640	4	18943713	T	G	2.32E-04	PRS-CSx
Colorectum	PRS7	rs10032763	4	105860001	G	A	-1.14E-03	PRS-CSx
Colorectum	PRS7	rs10032829	4	23601001	T	G	-7.37E-04	PRS-CSx
Colorectum	PRS7	rs1003533	5	131755651	T	C	-1.98E-04	PRS-CSx
Colorectum	PRS7	rs1003563	12	6424577	G	A	4.20E-03	PRS-CSx
Colorectum	PRS7	rs10036904	5	129280920	A	G	-6.56E-05	PRS-CSx

Colorectum	PRS7	rs10037551	5	143667583	C	T	-2.69E-04	PRS-CSx
Colorectum	PRS7	rs10038587	5	81994098	A	G	-8.72E-04	PRS-CSx
Colorectum	PRS7	rs10038914	5	40078936	C	T	-2.63E-04	PRS-CSx
Colorectum	PRS7	rs1003985	3	85870303	A	G	8.10E-04	PRS-CSx
Colorectum	PRS7	rs1003986	3	85870363	T	G	5.34E-04	PRS-CSx
Colorectum	PRS7	rs10041021	5	58566164	A	G	5.51E-04	PRS-CSx
Colorectum	PRS7	rs10041072	5	150259642	C	T	-7.20E-04	PRS-CSx
Colorectum	PRS7	rs10041357	5	39696953	G	A	-3.15E-04	PRS-CSx
Colorectum	PRS7	rs10042299	5	139805611	A	G	9.64E-05	PRS-CSx
Colorectum	PRS7	rs10043258	5	128406110	A	G	-6.25E-04	PRS-CSx
Colorectum	PRS7	rs10043960	5	74266343	G	A	-7.57E-04	PRS-CSx
Colorectum	PRS7	rs10044567	5	38018463	T	C	4.92E-04	PRS-CSx
Colorectum	PRS7	rs1004467	10	104594507	G	A	1.47E-04	PRS-CSx
Colorectum	PRS7	rs10044688	5	75850020	G	A	2.38E-04	PRS-CSx
Colorectum	PRS7	rs10046242	6	23896587	A	C	-1.51E-04	PRS-CSx
Colorectum	PRS7	rs1004630	2	76504256	C	T	8.34E-04	PRS-CSx
Colorectum	PRS7	rs10046843	9	34631159	T	C	4.66E-04	PRS-CSx
Colorectum	PRS7	rs10047037	1	12803557	C	T	3.02E-04	PRS-CSx
Colorectum	PRS7	rs10047266	10	25811835	T	G	-8.11E-04	PRS-CSx
Colorectum	PRS7	rs10047355	10	70060078	T	C	-1.42E-04	PRS-CSx
Colorectum	PRS7	rs10047388	10	70060213	A	C	-1.87E-04	PRS-CSx
Colorectum	PRS7	rs10048300	18	42029836	C	A	4.50E-04	PRS-CSx
Colorectum	PRS7	rs10050268	4	155549354	T	C	1.93E-04	PRS-CSx
Colorectum	PRS7	rs10052080	5	150228833	A	G	2.40E-04	PRS-CSx
Colorectum	PRS7	rs10053640	5	8653345	A	G	-2.31E-05	PRS-CSx
Colorectum	PRS7	rs1005478	22	37478254	G	A	-5.39E-05	PRS-CSx
Colorectum	PRS7	rs1005510	11	57367222	C	T	6.79E-04	PRS-CSx
Colorectum	PRS7	rs1005511	11	57366656	C	T	7.09E-04	PRS-CSx
Colorectum	PRS7	rs10056197	5	143701824	T	C	-2.35E-04	PRS-CSx
Colorectum	PRS7	rs10056287	5	54954478	C	T	-7.56E-04	PRS-CSx
Colorectum	PRS7	rs10056694	5	150188072	C	T	4.31E-04	PRS-CSx
Colorectum	PRS7	rs10057304	5	54887522	T	G	-2.31E-03	PRS-CSx
Colorectum	PRS7	rs10059011	5	150226722	C	A	-3.79E-04	PRS-CSx
Colorectum	PRS7	rs1005902	12	112667675	T	G	3.59E-04	PRS-CSx
Colorectum	PRS7	rs10059203	5	40052085	A	C	-3.46E-04	PRS-CSx
Colorectum	PRS7	rs1005975	6	131442975	C	T	-6.14E-04	PRS-CSx
Colorectum	PRS7	rs1006049	2	71176660	T	C	-2.61E-03	PRS-CSx
Colorectum	PRS7	rs1006168	1	156946898	G	T	-4.88E-04	PRS-CSx
Colorectum	PRS7	rs10062356	5	39641964	C	T	2.86E-04	PRS-CSx
Colorectum	PRS7	rs10064543	5	75839369	G	A	2.91E-04	PRS-CSx
Colorectum	PRS7	rs1006489	12	115954822	G	A	-8.95E-04	PRS-CSx
Colorectum	PRS7	rs10064916	5	128397565	A	G	-5.24E-04	PRS-CSx
Colorectum	PRS7	rs10065424	5	75841888	T	C	4.00E-04	PRS-CSx
Colorectum	PRS7	rs10066258	5	160413823	T	C	2.24E-05	PRS-CSx
Colorectum	PRS7	rs10067755	5	67307462	A	G	5.85E-04	PRS-CSx
Colorectum	PRS7	rs1006974	10	73745476	T	C	-2.24E-04	PRS-CSx
Colorectum	PRS7	rs10070294	5	58564996	C	T	4.32E-04	PRS-CSx
Colorectum	PRS7	rs10070478	5	134547726	C	T	1.43E-04	PRS-CSx
Colorectum	PRS7	rs10071999	5	35263218	T	C	-9.72E-04	PRS-CSx
Colorectum	PRS7	rs1007219	8	65576786	A	G	-1.95E-04	PRS-CSx
Colorectum	PRS7	rs10073556	5	170275387	T	C	-2.54E-04	PRS-CSx
Colorectum	PRS7	rs1007514	14	59219037	G	A	5.27E-04	PRS-CSx
Colorectum	PRS7	rs10075696	5	129411386	G	A	-1.81E-04	PRS-CSx
Colorectum	PRS7	rs10076266	5	3331680	A	C	-6.41E-03	PRS-CSx
Colorectum	PRS7	rs10076745	5	8653313	G	A	3.63E-05	PRS-CSx
Colorectum	PRS7	rs10077333	5	150244041	G	T	2.69E-04	PRS-CSx

Colorectum	PRS7	rs1007738	11	46849360	A	G	2.76E-04	PRS-CSx
Colorectum	PRS7	rs10078088	5	80661696	G	A	1.14E-04	PRS-CSx
Colorectum	PRS7	rs10078635	5	129365347	G	T	-1.96E-04	PRS-CSx
Colorectum	PRS7	rs10079105	5	161238179	A	G	7.40E-04	PRS-CSx
Colorectum	PRS7	rs10081	11	214544	C	T	8.85E-04	PRS-CSx
Colorectum	PRS7	rs10081574	8	117841222	C	T	1.35E-03	PRS-CSx
Colorectum	PRS7	rs10082678	11	27320448	G	A	2.74E-04	PRS-CSx
Colorectum	PRS7	rs10083213	12	111654363	C	T	-2.57E-05	PRS-CSx
Colorectum	PRS7	rs1008477	1	12829219	T	G	5.72E-04	PRS-CSx
Colorectum	PRS7	rs10085777	7	16499253	A	G	-2.48E-04	PRS-CSx
Colorectum	PRS7	rs10085825	7	95376403	T	C	1.09E-03	PRS-CSx
Colorectum	PRS7	rs1008594	7	88852488	T	C	-2.24E-04	PRS-CSx
Colorectum	PRS7	rs10086541	8	96033950	C	T	7.15E-04	PRS-CSx
Colorectum	PRS7	rs10090464	8	78340413	C	A	9.75E-04	PRS-CSx
Colorectum	PRS7	rs10090519	8	129597629	G	T	-6.91E-04	PRS-CSx
Colorectum	PRS7	rs1009123	21	40207796	T	C	6.79E-04	PRS-CSx
Colorectum	PRS7	rs10091813	8	41311166	C	T	4.52E-04	PRS-CSx
Colorectum	PRS7	rs10092003	8	96036422	G	A	4.20E-04	PRS-CSx
Colorectum	PRS7	rs1009227	1	231480507	A	G	1.92E-04	PRS-CSx
Colorectum	PRS7	rs10093594	8	96033236	T	C	5.66E-04	PRS-CSx
Colorectum	PRS7	rs1009360	2	65276049	C	T	8.77E-04	PRS-CSx
Colorectum	PRS7	rs10094063	8	129202426	T	C	-8.12E-05	PRS-CSx
Colorectum	PRS7	rs10094220	8	83820608	G	A	-1.68E-04	PRS-CSx
Colorectum	PRS7	rs10094788	8	62247290	A	G	-1.82E-03	PRS-CSx
Colorectum	PRS7	rs1009532	10	111840725	G	A	-2.96E-04	PRS-CSx
Colorectum	PRS7	rs10097930	8	96025735	G	A	2.27E-04	PRS-CSx
Colorectum	PRS7	rs10098453	8	96029756	A	G	6.30E-04	PRS-CSx
Colorectum	PRS7	rs1010064	12	20000315	C	A	4.29E-04	PRS-CSx
Colorectum	PRS7	rs10101096	8	38292147	C	A	7.83E-04	PRS-CSx
Colorectum	PRS7	rs10106323	8	41311222	A	G	1.96E-04	PRS-CSx
Colorectum	PRS7	rs10106867	8	6169238	G	A	1.91E-03	PRS-CSx
Colorectum	PRS7	rs1010738	14	57239870	T	C	1.84E-03	PRS-CSx
Colorectum	PRS7	rs10108135	8	58672897	T	G	3.05E-04	PRS-CSx
Colorectum	PRS7	rs10108200	8	117841774	C	T	7.47E-04	PRS-CSx
Colorectum	PRS7	rs10109496	8	117838623	A	G	1.75E-03	PRS-CSx
Colorectum	PRS7	rs10111154	8	96040887	G	A	8.80E-04	PRS-CSx
Colorectum	PRS7	rs10111415	8	117843979	T	G	3.45E-03	PRS-CSx
Colorectum	PRS7	rs10112022	8	58674089	A	C	5.23E-04	PRS-CSx
Colorectum	PRS7	rs10112382	8	128784397	T	C	-5.21E-04	PRS-CSx
Colorectum	PRS7	rs10115595	9	139830787	C	A	-4.28E-04	PRS-CSx
Colorectum	PRS7	rs10116277	9	22081397	G	T	3.59E-04	PRS-CSx
Colorectum	PRS7	rs10118029	9	81589361	G	A	7.65E-04	PRS-CSx
Colorectum	PRS7	rs1012053	13	42653437	C	A	4.56E-04	PRS-CSx
Colorectum	PRS7	rs10120688	9	22056499	G	A	2.72E-04	PRS-CSx
Colorectum	PRS7	rs10123206	9	19246844	T	C	1.16E-04	PRS-CSx
Colorectum	PRS7	rs10123375	9	139746992	G	A	-2.40E-04	PRS-CSx
Colorectum	PRS7	rs10128255	10	114742835	G	A	-2.94E-04	PRS-CSx
Colorectum	PRS7	rs10128766	12	120441443	A	C	2.80E-04	PRS-CSx
Colorectum	PRS7	rs10129415	14	102036157	A	C	1.32E-03	PRS-CSx
Colorectum	PRS7	rs10129472	14	66188209	T	C	1.59E-03	PRS-CSx
Colorectum	PRS7	rs10130134	14	91677628	T	C	1.17E-03	PRS-CSx
Colorectum	PRS7	rs10130192	14	59353791	C	T	2.28E-04	PRS-CSx
Colorectum	PRS7	rs10131136	14	79035246	C	T	8.36E-04	PRS-CSx
Colorectum	PRS7	rs10131598	14	51358794	C	T	9.27E-05	PRS-CSx
Colorectum	PRS7	rs10132049	14	51376237	G	A	7.94E-04	PRS-CSx
Colorectum	PRS7	rs10132174	14	54722250	C	T	7.81E-04	PRS-CSx

Colorectum	PRS7	rs10132765	14	66242314	G	A	5.42E-04	PRS-CSx
Colorectum	PRS7	rs10132973	14	34525213	G	A	-8.68E-04	PRS-CSx
Colorectum	PRS7	rs10133192	14	75756816	C	T	1.22E-03	PRS-CSx
Colorectum	PRS7	rs10133305	14	58761129	A	G	1.16E-04	PRS-CSx
Colorectum	PRS7	rs10133577	14	54696739	T	C	7.73E-04	PRS-CSx
Colorectum	PRS7	rs10134186	14	26772228	C	T	1.30E-03	PRS-CSx
Colorectum	PRS7	rs10134508	14	59351820	T	C	1.77E-05	PRS-CSx
Colorectum	PRS7	rs10134619	14	33367658	C	T	1.31E-03	PRS-CSx
Colorectum	PRS7	rs10134969	14	34142191	A	G	-3.31E-04	PRS-CSx
Colorectum	PRS7	rs1013545	13	73971803	T	C	1.36E-04	PRS-CSx
Colorectum	PRS7	rs10136042	14	57665761	A	C	7.31E-04	PRS-CSx
Colorectum	PRS7	rs10137185	14	64775776	T	C	9.84E-06	PRS-CSx
Colorectum	PRS7	rs1013719	7	10554750	C	T	-1.08E-03	PRS-CSx
Colorectum	PRS7	rs10137379	14	57674867	C	T	6.24E-04	PRS-CSx
Colorectum	PRS7	rs10137475	14	58797953	A	G	1.26E-04	PRS-CSx
Colorectum	PRS7	rs10138053	14	51615152	T	C	7.29E-04	PRS-CSx
Colorectum	PRS7	rs1013963	11	94504880	C	A	4.13E-04	PRS-CSx
Colorectum	PRS7	rs10140858	14	51388969	A	G	-1.08E-03	PRS-CSx
Colorectum	PRS7	rs10141803	14	99102622	C	T	-7.00E-04	PRS-CSx
Colorectum	PRS7	rs10142539	14	99136677	G	A	-2.47E-04	PRS-CSx
Colorectum	PRS7	rs10142973	14	58799118	G	A	1.61E-04	PRS-CSx
Colorectum	PRS7	rs10143053	14	51407850	T	G	4.16E-05	PRS-CSx
Colorectum	PRS7	rs1014324	6	116887861	C	T	-5.10E-04	PRS-CSx
Colorectum	PRS7	rs10144051	14	103885931	A	C	3.84E-04	PRS-CSx
Colorectum	PRS7	rs10145280	14	58770055	A	G	1.82E-04	PRS-CSx
Colorectum	PRS7	rs10145629	14	59289864	G	A	1.27E-04	PRS-CSx
Colorectum	PRS7	rs1014640	3	64543615	T	C	2.39E-04	PRS-CSx
Colorectum	PRS7	rs10146738	14	95280151	C	T	-2.82E-03	PRS-CSx
Colorectum	PRS7	rs10147580	14	58714140	C	T	1.66E-04	PRS-CSx
Colorectum	PRS7	rs10147786	14	53836403	C	T	2.64E-04	PRS-CSx
Colorectum	PRS7	rs10147953	14	104624689	G	A	-1.14E-03	PRS-CSx
Colorectum	PRS7	rs10148555	14	54042952	C	T	-4.32E-04	PRS-CSx
Colorectum	PRS7	rs1014897	20	895457	T	C	1.34E-03	PRS-CSx
Colorectum	PRS7	rs10148970	14	103892000	G	A	4.41E-04	PRS-CSx
Colorectum	PRS7	rs10149867	14	51393929	T	C	-1.02E-04	PRS-CSx
Colorectum	PRS7	rs10150805	14	79976742	T	C	1.16E-03	PRS-CSx
Colorectum	PRS7	rs1015149	6	41658889	T	C	-1.24E-03	PRS-CSx
Colorectum	PRS7	rs10152028	14	51498099	C	T	5.66E-04	PRS-CSx
Colorectum	PRS7	rs10152383	15	52536273	G	A	4.92E-04	PRS-CSx
Colorectum	PRS7	rs10152467	15	102146151	G	A	4.90E-04	PRS-CSx
Colorectum	PRS7	rs1015249	12	113125311	T	G	4.73E-04	PRS-CSx
Colorectum	PRS7	rs10153322	18	49960407	T	C	7.86E-04	PRS-CSx
Colorectum	PRS7	rs10153500	19	38073267	C	T	6.50E-04	PRS-CSx
Colorectum	PRS7	rs1015735	18	41623323	T	C	3.31E-04	PRS-CSx
Colorectum	PRS7	rs10157433	1	194639414	T	C	1.18E-04	PRS-CSx
Colorectum	PRS7	rs1015868	6	29485373	T	C	-1.08E-04	PRS-CSx
Colorectum	PRS7	rs1015869	6	29484666	A	G	-2.16E-05	PRS-CSx
Colorectum	PRS7	rs1015897	11	17883404	C	T	-5.47E-04	PRS-CSx
Colorectum	PRS7	rs10159239	1	247607052	G	A	5.19E-04	PRS-CSx
Colorectum	PRS7	rs1016146	6	35445516	A	G	1.74E-04	PRS-CSx
Colorectum	PRS7	rs10162199	13	95838077	T	C	9.94E-04	PRS-CSx
Colorectum	PRS7	rs10163302	16	3392984	T	C	9.24E-04	PRS-CSx
Colorectum	PRS7	rs1016344	5	134572865	G	A	1.13E-03	PRS-CSx
Colorectum	PRS7	rs10163573	18	41661592	A	G	2.65E-04	PRS-CSx
Colorectum	PRS7	rs10165122	2	12208315	G	T	7.34E-04	PRS-CSx
Colorectum	PRS7	rs10166836	2	207039316	T	C	3.76E-04	PRS-CSx

Colorectum	PRS7	rs1016762	3	41206998	T	C	-1.77E-04	PRS-CSx
Colorectum	PRS7	rs1016792	4	146478779	C	T	-3.20E-04	PRS-CSx
Colorectum	PRS7	rs10169949	2	70833018	G	A	2.11E-03	PRS-CSx
Colorectum	PRS7	rs1017057	7	107494658	C	T	-8.63E-04	PRS-CSx
Colorectum	PRS7	rs10170896	2	12581138	A	G	2.27E-04	PRS-CSx
Colorectum	PRS7	rs10171517	2	28090059	T	C	3.77E-04	PRS-CSx
Colorectum	PRS7	rs10172694	2	154122856	C	T	4.15E-04	PRS-CSx
Colorectum	PRS7	rs1017305	5	68105402	A	G	9.15E-04	PRS-CSx
Colorectum	PRS7	rs10173529	2	108965676	A	G	-4.92E-04	PRS-CSx
Colorectum	PRS7	rs10173535	2	108965694	T	G	-4.92E-04	PRS-CSx
Colorectum	PRS7	rs10173714	2	225618741	A	G	2.03E-04	PRS-CSx
Colorectum	PRS7	rs1017406	2	163643407	A	C	2.83E-04	PRS-CSx
Colorectum	PRS7	rs10174247	2	160072795	A	G	-5.96E-04	PRS-CSx
Colorectum	PRS7	rs10174511	2	227348073	C	T	9.27E-04	PRS-CSx
Colorectum	PRS7	rs1017454	5	12975322	A	G	-6.21E-04	PRS-CSx
Colorectum	PRS7	rs10175176	2	160058776	C	T	1.37E-03	PRS-CSx
Colorectum	PRS7	rs10175898	2	182603382	G	A	-9.02E-04	PRS-CSx
Colorectum	PRS7	rs1017720	15	31768529	T	C	4.61E-04	PRS-CSx
Colorectum	PRS7	rs10177607	2	240226439	C	T	8.11E-04	PRS-CSx
Colorectum	PRS7	rs10177901	2	240473961	G	A	7.88E-04	PRS-CSx
Colorectum	PRS7	rs10178071	2	49417986	C	T	5.83E-04	PRS-CSx
Colorectum	PRS7	rs10179426	2	101649269	T	C	5.77E-04	PRS-CSx
Colorectum	PRS7	rs10179599	2	101649427	T	C	4.30E-04	PRS-CSx
Colorectum	PRS7	rs10179721	2	176433473	A	C	3.67E-04	PRS-CSx
Colorectum	PRS7	rs10181042	2	61224259	T	C	-9.76E-04	PRS-CSx
Colorectum	PRS7	rs10181622	2	225608689	T	C	-3.20E-04	PRS-CSx
Colorectum	PRS7	rs10182789	2	69410325	G	T	-2.02E-03	PRS-CSx
Colorectum	PRS7	rs10183527	2	182623490	G	A	-5.90E-04	PRS-CSx
Colorectum	PRS7	rs10184619	2	28582597	C	T	1.78E-04	PRS-CSx
Colorectum	PRS7	rs10185589	2	144587571	C	T	-6.65E-04	PRS-CSx
Colorectum	PRS7	rs10186075	2	124259490	A	G	-3.36E-04	PRS-CSx
Colorectum	PRS7	rs10186922	2	159731466	G	A	-2.96E-04	PRS-CSx
Colorectum	PRS7	rs10188217	2	61217542	C	T	-8.96E-04	PRS-CSx
Colorectum	PRS7	rs10189341	2	176435942	A	G	2.38E-04	PRS-CSx
Colorectum	PRS7	rs10190546	2	152934475	G	A	-2.69E-04	PRS-CSx
Colorectum	PRS7	rs10191848	2	69397157	A	G	8.27E-04	PRS-CSx
Colorectum	PRS7	rs10192196	2	225577135	G	A	1.31E-04	PRS-CSx
Colorectum	PRS7	rs10193918	2	8230485	C	T	-8.22E-04	PRS-CSx
Colorectum	PRS7	rs1019445	19	18453124	T	C	7.75E-04	PRS-CSx
Colorectum	PRS7	rs1019496	5	37773877	T	C	2.12E-04	PRS-CSx
Colorectum	PRS7	rs10196805	2	67316250	A	G	-6.35E-04	PRS-CSx
Colorectum	PRS7	rs1019736	5	134579744	T	G	6.60E-04	PRS-CSx
Colorectum	PRS7	rs10197382	2	225574650	T	C	1.90E-04	PRS-CSx
Colorectum	PRS7	rs10198160	2	38611930	C	T	-5.87E-04	PRS-CSx
Colorectum	PRS7	rs10198413	2	159955575	T	C	-3.08E-04	PRS-CSx
Colorectum	PRS7	rs10198809	2	225579262	T	C	-4.47E-04	PRS-CSx
Colorectum	PRS7	rs10199004	2	143244498	A	G	2.49E-03	PRS-CSx
Colorectum	PRS7	rs10199186	2	28662375	T	C	-4.74E-03	PRS-CSx
Colorectum	PRS7	rs1019987	15	79580877	T	G	-8.00E-04	PRS-CSx
Colorectum	PRS7	rs10199998	2	67308243	C	T	-5.38E-04	PRS-CSx
Colorectum	PRS7	rs10203544	2	225595932	A	G	-1.17E-04	PRS-CSx
Colorectum	PRS7	rs10207132	2	183300646	G	A	2.72E-04	PRS-CSx
Colorectum	PRS7	rs10208207	2	169074972	C	T	-4.55E-04	PRS-CSx
Colorectum	PRS7	rs10209234	2	144587743	A	G	-9.41E-04	PRS-CSx
Colorectum	PRS7	rs10210248	2	84673252	C	T	-7.11E-04	PRS-CSx
Colorectum	PRS7	rs10210419	2	130217485	G	A	-4.97E-04	PRS-CSx

Colorectum	PRS7	rs10210687	2	137389497	G	A	2.77E-04	PRS-CSx
Colorectum	PRS7	rs1021087	8	13456131	G	T	-1.37E-03	PRS-CSx
Colorectum	PRS7	rs1021090	2	183217590	A	G	6.68E-04	PRS-CSx
Colorectum	PRS7	rs1021662	3	192727562	G	A	-5.67E-04	PRS-CSx
Colorectum	PRS7	rs10218853	10	104796787	C	A	-5.46E-05	PRS-CSx
Colorectum	PRS7	rs10219203	11	74324923	T	C	7.24E-04	PRS-CSx
Colorectum	PRS7	rs10219739	12	54652654	A	G	-6.48E-04	PRS-CSx
Colorectum	PRS7	rs10220304	14	65931721	C	T	1.39E-04	PRS-CSx
Colorectum	PRS7	rs10221471	19	31929293	C	T	1.14E-03	PRS-CSx
Colorectum	PRS7	rs10222249	22	29257147	A	G	1.18E-04	PRS-CSx
Colorectum	PRS7	rs10224307	7	135313837	T	C	1.35E-04	PRS-CSx
Colorectum	PRS7	rs10224439	7	45180921	G	T	-3.50E-04	PRS-CSx
Colorectum	PRS7	rs1022477	22	45821887	G	A	-1.04E-04	PRS-CSx
Colorectum	PRS7	rs1022492	6	25579176	G	T	-4.06E-04	PRS-CSx
Colorectum	PRS7	rs102275	11	61557803	T	C	4.79E-04	PRS-CSx
Colorectum	PRS7	rs10228842	7	11965352	T	C	5.82E-04	PRS-CSx
Colorectum	PRS7	rs1023564	7	88841714	C	A	-3.40E-04	PRS-CSx
Colorectum	PRS7	rs10236415	7	35444548	A	C	-8.05E-04	PRS-CSx
Colorectum	PRS7	rs1023679	5	143766166	A	G	-1.73E-04	PRS-CSx
Colorectum	PRS7	rs10239141	7	132098335	C	T	-2.65E-04	PRS-CSx
Colorectum	PRS7	rs10239155	7	45181792	G	A	-2.76E-04	PRS-CSx
Colorectum	PRS7	rs10240045	7	88932387	T	C	-3.95E-04	PRS-CSx
Colorectum	PRS7	rs10240887	7	8790170	T	C	2.93E-04	PRS-CSx
Colorectum	PRS7	rs10242657	7	25882917	T	C	5.88E-04	PRS-CSx
Colorectum	PRS7	rs1024363	8	18045500	T	C	1.35E-03	PRS-CSx
Colorectum	PRS7	rs1024386	7	88121560	C	A	-3.69E-04	PRS-CSx
Colorectum	PRS7	rs1024467	12	113187354	C	T	-1.67E-05	PRS-CSx
Colorectum	PRS7	rs10245199	7	150758482	G	A	-3.96E-04	PRS-CSx
Colorectum	PRS7	rs10247993	7	127080377	C	T	1.19E-03	PRS-CSx
Colorectum	PRS7	rs1024890	2	71601522	C	T	-4.38E-04	PRS-CSx
Colorectum	PRS7	rs10249234	7	114129137	A	G	4.41E-04	PRS-CSx
Colorectum	PRS7	rs10251787	7	126842035	C	T	1.54E-04	PRS-CSx
Colorectum	PRS7	rs10252204	7	76820800	C	A	-5.24E-04	PRS-CSx
Colorectum	PRS7	rs1025261	5	134572376	T	C	1.94E-03	PRS-CSx
Colorectum	PRS7	rs10252811	7	96145647	A	G	-1.19E-03	PRS-CSx
Colorectum	PRS7	rs10253160	7	674233	A	C	-2.02E-03	PRS-CSx
Colorectum	PRS7	rs10254316	7	11964570	C	A	7.08E-04	PRS-CSx
Colorectum	PRS7	rs10258686	7	35427109	C	T	-9.10E-04	PRS-CSx
Colorectum	PRS7	rs1025905	17	52704312	C	T	-1.98E-04	PRS-CSx
Colorectum	PRS7	rs10259273	7	93714505	T	C	3.91E-04	PRS-CSx
Colorectum	PRS7	rs10261415	7	83760881	G	T	-4.92E-04	PRS-CSx
Colorectum	PRS7	rs10264280	7	45120453	T	C	-2.09E-04	PRS-CSx
Colorectum	PRS7	rs10266297	7	114143407	T	C	2.46E-04	PRS-CSx
Colorectum	PRS7	rs1026710	16	56241470	T	G	-2.97E-04	PRS-CSx
Colorectum	PRS7	rs10267212	7	99595422	C	T	-1.78E-04	PRS-CSx
Colorectum	PRS7	rs10267258	7	46168740	C	T	1.02E-05	PRS-CSx
Colorectum	PRS7	rs10269268	7	16627411	A	G	-7.13E-04	PRS-CSx
Colorectum	PRS7	rs1027204	6	26639613	T	C	-2.41E-04	PRS-CSx
Colorectum	PRS7	rs10274783	7	88941944	C	T	-2.48E-04	PRS-CSx
Colorectum	PRS7	rs10276536	7	2086960	T	C	-1.00E-03	PRS-CSx
Colorectum	PRS7	rs10278144	7	45171410	G	A	-2.22E-04	PRS-CSx
Colorectum	PRS7	rs1027880	2	38626307	C	T	-7.82E-04	PRS-CSx
Colorectum	PRS7	rs10279101	7	88848044	C	T	-2.16E-04	PRS-CSx
Colorectum	PRS7	rs10279499	7	92733766	A	C	-2.92E-04	PRS-CSx
Colorectum	PRS7	rs10280773	7	7304142	T	C	2.74E-03	PRS-CSx
Colorectum	PRS7	rs10281368	7	99655897	G	A	-2.80E-04	PRS-CSx

Colorectum	PRS7	rs10283129	8	117778673	A	G	7.73E-04	PRS-CSx
Colorectum	PRS7	rs1028564	11	33162009	A	G	-1.71E-04	PRS-CSx
Colorectum	PRS7	rs1028590	16	1858069	T	C	9.79E-04	PRS-CSx
Colorectum	PRS7	rs1029388	12	111926901	T	C	4.44E-04	PRS-CSx
Colorectum	PRS7	rs1029484	12	113285073	G	A	-2.06E-04	PRS-CSx
Colorectum	PRS7	rs1029830	17	17184097	C	A	2.79E-04	PRS-CSx
Colorectum	PRS7	rs10305514	6	39054138	T	G	-9.55E-04	PRS-CSx
Colorectum	PRS7	rs10305518	6	39055012	G	T	-2.04E-03	PRS-CSx
Colorectum	PRS7	rs1030809	3	173192043	T	G	8.04E-04	PRS-CSx
Colorectum	PRS7	rs1031293	2	207229821	C	T	-9.20E-05	PRS-CSx
Colorectum	PRS7	rs1031378	4	151712054	C	T	4.73E-04	PRS-CSx
Colorectum	PRS7	rs1031496	8	126591506	A	G	-5.73E-04	PRS-CSx
Colorectum	PRS7	rs1032072	17	40575688	T	C	1.71E-03	PRS-CSx
Colorectum	PRS7	rs1032202	18	69677805	G	A	-3.49E-04	PRS-CSx
Colorectum	PRS7	rs1032412	2	163639107	A	G	2.95E-04	PRS-CSx
Colorectum	PRS7	rs1032524	1	201790826	C	T	3.03E-04	PRS-CSx
Colorectum	PRS7	rs1033398	20	8138483	G	A	4.84E-04	PRS-CSx
Colorectum	PRS7	rs1033823	14	54522857	C	T	6.46E-04	PRS-CSx
Colorectum	PRS7	rs1034329	22	21943938	T	C	1.63E-04	PRS-CSx
Colorectum	PRS7	rs1034597	19	35799200	T	C	4.80E-04	PRS-CSx
Colorectum	PRS7	rs1034601	12	111647825	T	C	3.38E-05	PRS-CSx
Colorectum	PRS7	rs1034602	12	111649179	G	A	1.20E-05	PRS-CSx
Colorectum	PRS7	rs1034603	12	111649344	A	G	-3.76E-05	PRS-CSx
Colorectum	PRS7	rs1035077	17	70462887	G	A	9.66E-04	PRS-CSx
Colorectum	PRS7	rs1035209	10	101345366	T	C	6.58E-03	PRS-CSx
Colorectum	PRS7	rs1035380	5	85421790	C	T	9.66E-04	PRS-CSx
Colorectum	PRS7	rs1035421	5	167768114	C	T	1.67E-03	PRS-CSx
Colorectum	PRS7	rs1035445	19	33694158	T	C	-1.56E-03	PRS-CSx
Colorectum	PRS7	rs1035798	6	32151222	A	G	-1.20E-04	PRS-CSx
Colorectum	PRS7	rs1036322	12	95260442	T	C	-1.60E-04	PRS-CSx
Colorectum	PRS7	rs1036796	3	64886335	A	G	1.25E-05	PRS-CSx
Colorectum	PRS7	rs1036919	3	64527336	T	C	6.93E-05	PRS-CSx
Colorectum	PRS7	rs1037831	8	76323329	T	G	-1.03E-03	PRS-CSx
Colorectum	PRS7	rs1039481	11	48182237	G	A	4.83E-04	PRS-CSx
Colorectum	PRS7	rs10400277	11	49135301	C	T	-4.17E-04	PRS-CSx
Colorectum	PRS7	rs10400498	12	120494064	G	A	2.49E-04	PRS-CSx
Colorectum	PRS7	rs10403940	19	33476866	A	G	-5.00E-03	PRS-CSx
Colorectum	PRS7	rs1040427	22	43373570	C	A	9.51E-05	PRS-CSx
Colorectum	PRS7	rs1040558	6	20713706	G	A	-1.12E-04	PRS-CSx
Colorectum	PRS7	rs10405981	19	12560254	G	A	1.65E-05	PRS-CSx
Colorectum	PRS7	rs10409485	19	41920765	C	T	1.39E-04	PRS-CSx
Colorectum	PRS7	rs10411210	19	33532300	T	C	-7.25E-03	PRS-CSx
Colorectum	PRS7	rs10411735	19	33444707	T	C	4.09E-04	PRS-CSx
Colorectum	PRS7	rs10413885	19	4656355	G	A	3.65E-04	PRS-CSx
Colorectum	PRS7	rs10416642	19	1681480	G	A	7.25E-04	PRS-CSx
Colorectum	PRS7	rs10416763	19	8232734	T	C	1.30E-03	PRS-CSx
Colorectum	PRS7	rs10417204	19	38034722	A	G	-3.40E-04	PRS-CSx
Colorectum	PRS7	rs10418535	19	17850189	C	T	-9.76E-04	PRS-CSx
Colorectum	PRS7	rs10423119	19	4585171	C	A	5.07E-04	PRS-CSx
Colorectum	PRS7	rs10424568	19	39138948	A	G	2.16E-04	PRS-CSx
Colorectum	PRS7	rs10425117	19	995282	G	A	1.95E-03	PRS-CSx
Colorectum	PRS7	rs10426809	19	39186950	G	A	-3.49E-04	PRS-CSx
Colorectum	PRS7	rs1043128	9	113635929	G	A	5.54E-04	PRS-CSx
Colorectum	PRS7	rs10433340	3	122433930	A	G	-2.89E-04	PRS-CSx
Colorectum	PRS7	rs10433642	3	64605855	A	G	1.97E-04	PRS-CSx
Colorectum	PRS7	rs10433949	4	90038053	C	T	4.79E-04	PRS-CSx

Colorectum	PRS7	rs1043596	6	131161504	C	T	8.59E-05	PRS-CSx
Colorectum	PRS7	rs10438350	15	66979985	T	G	1.26E-03	PRS-CSx
Colorectum	PRS7	rs1043873	8	41367719	G	T	1.01E-03	PRS-CSx
Colorectum	PRS7	rs10439088	19	28502615	T	C	-4.45E-04	PRS-CSx
Colorectum	PRS7	rs10439137	19	28500957	C	T	-4.29E-04	PRS-CSx
Colorectum	PRS7	rs10439141	19	28501323	C	T	-4.19E-04	PRS-CSx
Colorectum	PRS7	rs10439229	2	6424385	C	A	5.72E-04	PRS-CSx
Colorectum	PRS7	rs10440630	5	58529636	G	A	1.31E-03	PRS-CSx
Colorectum	PRS7	rs10441090	7	38334818	T	C	-1.82E-03	PRS-CSx
Colorectum	PRS7	rs1044120	2	206988573	A	C	1.98E-04	PRS-CSx
Colorectum	PRS7	rs10441723	9	34082144	C	T	-3.80E-04	PRS-CSx
Colorectum	PRS7	rs1044218	21	34821570	G	A	9.60E-06	PRS-CSx
Colorectum	PRS7	rs10444715	14	52098340	A	G	-1.21E-03	PRS-CSx
Colorectum	PRS7	rs10446259	21	43355463	C	T	1.10E-03	PRS-CSx
Colorectum	PRS7	rs10447248	5	107915736	G	A	-6.68E-04	PRS-CSx
Colorectum	PRS7	rs1044983	7	16640300	G	A	6.71E-04	PRS-CSx
Colorectum	PRS7	rs10450561	11	27309956	C	T	7.41E-05	PRS-CSx
Colorectum	PRS7	rs10451228	17	75684250	A	G	6.00E-04	PRS-CSx
Colorectum	PRS7	rs10456362	6	28221816	A	G	-2.80E-04	PRS-CSx
Colorectum	PRS7	rs10456372	6	29341449	G	A	-2.69E-04	PRS-CSx
Colorectum	PRS7	rs10456373	6	29343333	C	T	-2.94E-04	PRS-CSx
Colorectum	PRS7	rs1045670	12	113335681	A	G	-8.38E-04	PRS-CSx
Colorectum	PRS7	rs10457242	6	111955435	G	A	-3.60E-04	PRS-CSx
Colorectum	PRS7	rs10458466	1	221207884	G	T	2.02E-04	PRS-CSx
Colorectum	PRS7	rs10459061	12	14073557	A	C	-9.72E-04	PRS-CSx
Colorectum	PRS7	rs10460398	2	200214742	T	C	-9.30E-05	PRS-CSx
Colorectum	PRS7	rs10460536	2	47283793	T	C	6.67E-04	PRS-CSx
Colorectum	PRS7	rs10461165	4	89770583	A	G	1.06E-04	PRS-CSx
Colorectum	PRS7	rs10463297	5	139936239	T	C	4.35E-04	PRS-CSx
Colorectum	PRS7	rs10463643	5	112032675	T	C	-4.24E-04	PRS-CSx
Colorectum	PRS7	rs10463861	5	129266529	G	A	-1.69E-04	PRS-CSx
Colorectum	PRS7	rs10468114	15	96118957	G	A	-1.36E-03	PRS-CSx
Colorectum	PRS7	rs1046909	19	41882712	G	A	7.42E-04	PRS-CSx
Colorectum	PRS7	rs10469881	2	65007992	C	T	4.13E-03	PRS-CSx
Colorectum	PRS7	rs10472268	5	35955525	T	C	-3.58E-05	PRS-CSx
Colorectum	PRS7	rs10473156	5	39960584	A	C	2.10E-04	PRS-CSx
Colorectum	PRS7	rs1047700	15	25924539	C	T	-4.64E-04	PRS-CSx
Colorectum	PRS7	rs10477239	5	143643808	A	G	-1.78E-04	PRS-CSx
Colorectum	PRS7	rs10477713	5	129343581	T	C	-3.75E-04	PRS-CSx
Colorectum	PRS7	rs10477768	5	134551759	C	T	-2.74E-05	PRS-CSx
Colorectum	PRS7	rs1047796	12	122628219	C	T	1.57E-04	PRS-CSx
Colorectum	PRS7	rs10482792	1	218605461	G	A	-5.94E-04	PRS-CSx
Colorectum	PRS7	rs10482796	1	218605635	T	C	-3.21E-04	PRS-CSx
Colorectum	PRS7	rs10483231	22	46178009	C	T	-4.58E-04	PRS-CSx
Colorectum	PRS7	rs10483628	14	54638401	T	C	1.52E-03	PRS-CSx
Colorectum	PRS7	rs10483698	14	58895456	G	A	3.24E-04	PRS-CSx
Colorectum	PRS7	rs10483708	14	59409089	T	C	3.54E-04	PRS-CSx
Colorectum	PRS7	rs10483807	14	68808824	G	A	-2.55E-04	PRS-CSx
Colorectum	PRS7	rs104843	16	1873087	C	A	-2.33E-04	PRS-CSx
Colorectum	PRS7	rs10484390	6	22073654	G	A	2.55E-03	PRS-CSx
Colorectum	PRS7	rs10484442	6	26555879	A	G	1.15E-04	PRS-CSx
Colorectum	PRS7	rs10484542	6	28309753	A	G	2.04E-04	PRS-CSx
Colorectum	PRS7	rs10484560	6	32298137	A	G	-1.60E-04	PRS-CSx
Colorectum	PRS7	rs10485717	20	7810099	G	A	9.30E-04	PRS-CSx
Colorectum	PRS7	rs10485719	20	8131904	T	C	3.14E-04	PRS-CSx
Colorectum	PRS7	rs10485767	20	13753470	A	G	3.98E-04	PRS-CSx

Colorectum	PRS7	rs10486414	7	25765401	G	A	-4.94E-04	PRS-CSx
Colorectum	PRS7	rs10486415	7	25771938	C	T	-2.32E-04	PRS-CSx
Colorectum	PRS7	rs10487483	7	127074138	T	C	8.22E-04	PRS-CSx
Colorectum	PRS7	rs10488040	7	77365365	C	T	-3.09E-04	PRS-CSx
Colorectum	PRS7	rs10488465	7	134561819	T	C	-1.89E-03	PRS-CSx
Colorectum	PRS7	rs10488466	7	134562744	G	A	-7.43E-04	PRS-CSx
Colorectum	PRS7	rs10488739	11	34675079	T	C	-6.99E-04	PRS-CSx
Colorectum	PRS7	rs1048904	11	34684543	A	C	5.09E-04	PRS-CSx
Colorectum	PRS7	rs10489193	1	167617699	G	A	-3.63E-03	PRS-CSx
Colorectum	PRS7	rs10489581	1	185121752	A	G	-3.10E-04	PRS-CSx
Colorectum	PRS7	rs10489960	1	221188122	T	G	-1.55E-03	PRS-CSx
Colorectum	PRS7	rs10490555	2	42522756	A	G	-7.94E-05	PRS-CSx
Colorectum	PRS7	rs10490910	10	118076455	T	C	3.82E-04	PRS-CSx
Colorectum	PRS7	rs10491120	17	34988444	G	A	-1.21E-03	PRS-CSx
Colorectum	PRS7	rs10491667	9	29438214	C	A	-1.81E-03	PRS-CSx
Colorectum	PRS7	rs10491693	9	327365	T	C	-2.39E-04	PRS-CSx
Colorectum	PRS7	rs10491985	12	11810134	G	A	2.44E-03	PRS-CSx
Colorectum	PRS7	rs10492018	12	113127404	A	G	3.25E-04	PRS-CSx
Colorectum	PRS7	rs10492024	12	113269412	A	G	-2.96E-04	PRS-CSx
Colorectum	PRS7	rs10492142	12	14045266	C	T	-1.95E-04	PRS-CSx
Colorectum	PRS7	rs10492252	12	108964885	A	C	8.92E-04	PRS-CSx
Colorectum	PRS7	rs10492438	13	42767706	A	C	3.16E-04	PRS-CSx
Colorectum	PRS7	rs10492939	1	3302871	G	A	6.51E-04	PRS-CSx
Colorectum	PRS7	rs10493375	1	65844654	T	G	-7.82E-04	PRS-CSx
Colorectum	PRS7	rs10493872	1	94890418	T	G	-8.86E-04	PRS-CSx
Colorectum	PRS7	rs10493900	1	98645213	G	A	-2.61E-04	PRS-CSx
Colorectum	PRS7	rs10495106	1	218951186	C	T	-1.23E-03	PRS-CSx
Colorectum	PRS7	rs10495152	1	220990355	A	C	2.63E-04	PRS-CSx
Colorectum	PRS7	rs10495194	1	222733971	T	C	-1.56E-04	PRS-CSx
Colorectum	PRS7	rs10495589	2	12207377	T	C	7.40E-04	PRS-CSx
Colorectum	PRS7	rs10495622	2	14021058	G	A	2.11E-04	PRS-CSx
Colorectum	PRS7	rs10496040	2	55307646	A	C	1.18E-03	PRS-CSx
Colorectum	PRS7	rs10496405	2	107259476	G	A	6.20E-04	PRS-CSx
Colorectum	PRS7	rs10496511	2	116661716	C	T	1.46E-03	PRS-CSx
Colorectum	PRS7	rs10497401	2	174089606	G	T	-6.15E-04	PRS-CSx
Colorectum	PRS7	rs10497442	2	176436826	T	C	5.22E-04	PRS-CSx
Colorectum	PRS7	rs10497597	2	183263753	T	C	-1.48E-04	PRS-CSx
Colorectum	PRS7	rs10497603	2	183336468	C	T	1.63E-04	PRS-CSx
Colorectum	PRS7	rs10497607	2	183458192	T	C	-1.74E-05	PRS-CSx
Colorectum	PRS7	rs10498074	2	221134279	T	C	1.37E-03	PRS-CSx
Colorectum	PRS7	rs10498172	2	225797167	T	G	-4.33E-04	PRS-CSx
Colorectum	PRS7	rs10498447	14	53718458	G	A	6.76E-04	PRS-CSx
Colorectum	PRS7	rs10498664	6	3844275	C	T	-9.95E-04	PRS-CSx
Colorectum	PRS7	rs10499162	6	129919010	C	T	1.94E-03	PRS-CSx
Colorectum	PRS7	rs10499177	6	131215821	G	A	6.16E-04	PRS-CSx
Colorectum	PRS7	rs10499470	7	16506133	A	G	-4.24E-04	PRS-CSx
Colorectum	PRS7	rs10500674	11	7058008	A	G	-2.94E-04	PRS-CSx
Colorectum	PRS7	rs10500822	11	16047917	A	G	5.00E-04	PRS-CSx
Colorectum	PRS7	rs10500825	11	16141354	T	C	6.90E-04	PRS-CSx
Colorectum	PRS7	rs10500898	11	21195352	T	C	-3.35E-04	PRS-CSx
Colorectum	PRS7	rs10500899	11	21195446	T	C	-4.60E-04	PRS-CSx
Colorectum	PRS7	rs10501417	11	74330980	G	A	-1.27E-03	PRS-CSx
Colorectum	PRS7	rs10501544	11	83334780	G	A	1.35E-04	PRS-CSx
Colorectum	PRS7	rs10502541	18	27853483	G	A	1.26E-03	PRS-CSx
Colorectum	PRS7	rs10502658	18	34106818	G	A	5.15E-04	PRS-CSx
Colorectum	PRS7	rs1050492	1	226820500	G	A	-1.95E-03	PRS-CSx

Colorectum	PRS7	rs10505287	8	117727850	C	T	-1.91E-04	PRS-CSx
Colorectum	PRS7	rs10505299	8	118426740	T	C	8.01E-04	PRS-CSx
Colorectum	PRS7	rs10505376	8	121072939	T	G	-1.39E-04	PRS-CSx
Colorectum	PRS7	rs10505473	8	128417936	T	C	4.85E-04	PRS-CSx
Colorectum	PRS7	rs10505475	8	128411457	G	T	2.26E-04	PRS-CSx
Colorectum	PRS7	rs10505476	8	128408116	T	C	2.27E-04	PRS-CSx
Colorectum	PRS7	rs10505477	8	128407443	A	G	7.74E-03	PRS-CSx
Colorectum	PRS7	rs10505509	8	129148754	A	G	1.51E-03	PRS-CSx
Colorectum	PRS7	rs10505554	8	131670595	T	C	-8.05E-04	PRS-CSx
Colorectum	PRS7	rs10505960	12	25273767	T	C	-2.69E-04	PRS-CSx
Colorectum	PRS7	rs10505980	12	25392443	T	C	-3.40E-04	PRS-CSx
Colorectum	PRS7	rs10506212	12	43096024	A	G	-4.70E-04	PRS-CSx
Colorectum	PRS7	rs10506213	12	43096128	T	C	-7.80E-04	PRS-CSx
Colorectum	PRS7	rs10506216	12	43130885	A	G	8.34E-04	PRS-CSx
Colorectum	PRS7	rs10506217	12	43131208	C	T	-8.49E-04	PRS-CSx
Colorectum	PRS7	rs10506220	12	43307405	T	C	3.49E-04	PRS-CSx
Colorectum	PRS7	rs10506606	12	71151654	C	A	-5.72E-04	PRS-CSx
Colorectum	PRS7	rs10506868	10	114319380	T	C	1.87E-03	PRS-CSx
Colorectum	PRS7	rs10507058	12	96098747	T	C	-2.66E-04	PRS-CSx
Colorectum	PRS7	rs10507382	13	28873144	C	T	-1.89E-04	PRS-CSx
Colorectum	PRS7	rs10507802	13	73019325	C	T	7.06E-04	PRS-CSx
Colorectum	PRS7	rs10508019	13	95895769	A	G	4.77E-04	PRS-CSx
Colorectum	PRS7	rs10508359	10	8631309	C	A	2.33E-04	PRS-CSx
Colorectum	PRS7	rs10508364	10	8768512	G	A	5.16E-04	PRS-CSx
Colorectum	PRS7	rs10508404	10	10565427	C	T	1.95E-03	PRS-CSx
Colorectum	PRS7	rs10508699	10	25821161	A	G	-5.32E-04	PRS-CSx
Colorectum	PRS7	rs10509264	10	68158254	T	C	1.17E-03	PRS-CSx
Colorectum	PRS7	rs10509419	10	82659941	T	C	-5.11E-04	PRS-CSx
Colorectum	PRS7	rs10509798	10	107503741	T	C	-9.69E-04	PRS-CSx
Colorectum	PRS7	rs10509907	10	111781715	C	T	-1.96E-04	PRS-CSx
Colorectum	PRS7	rs10510719	3	41390139	A	G	-1.85E-04	PRS-CSx
Colorectum	PRS7	rs10510879	3	62619770	C	T	-2.81E-03	PRS-CSx
Colorectum	PRS7	rs10511027	3	73791644	T	G	-2.94E-04	PRS-CSx
Colorectum	PRS7	rs1051105	9	101832442	G	A	3.94E-04	PRS-CSx
Colorectum	PRS7	rs10511319	3	112833698	C	T	-1.60E-03	PRS-CSx
Colorectum	PRS7	rs10511323	3	112958324	G	A	2.33E-04	PRS-CSx
Colorectum	PRS7	rs10511914	9	34107232	C	T	-5.90E-04	PRS-CSx
Colorectum	PRS7	rs10512262	9	101805008	A	G	-4.96E-04	PRS-CSx
Colorectum	PRS7	rs10512292	9	104608735	C	T	1.55E-03	PRS-CSx
Colorectum	PRS7	rs10512586	17	70415077	C	T	9.52E-03	PRS-CSx
Colorectum	PRS7	rs10512727	5	40193497	C	T	-5.57E-04	PRS-CSx
Colorectum	PRS7	rs10512751	5	40934832	C	A	-4.50E-04	PRS-CSx
Colorectum	PRS7	rs10512992	5	52069435	C	A	6.41E-04	PRS-CSx
Colorectum	PRS7	rs10513719	3	173510129	G	A	1.80E-04	PRS-CSx
Colorectum	PRS7	rs10513889	18	54011876	A	C	-2.92E-04	PRS-CSx
Colorectum	PRS7	rs10513892	14	87191137	T	C	6.23E-04	PRS-CSx
Colorectum	PRS7	rs10514259	5	82879340	A	G	-1.95E-03	PRS-CSx
Colorectum	PRS7	rs10514275	5	85434559	T	C	1.24E-03	PRS-CSx
Colorectum	PRS7	rs10514477	16	80024839	T	C	-8.75E-04	PRS-CSx
Colorectum	PRS7	rs10514478	16	80025027	C	T	6.02E-04	PRS-CSx
Colorectum	PRS7	rs10514623	2	108963492	C	T	-7.02E-04	PRS-CSx
Colorectum	PRS7	rs1051473	1	183114146	T	C	1.92E-04	PRS-CSx
Colorectum	PRS7	rs10514734	3	77604690	T	C	-1.27E-05	PRS-CSx
Colorectum	PRS7	rs10514804	2	47563573	A	G	3.12E-03	PRS-CSx
Colorectum	PRS7	rs10515072	17	52679112	A	G	-3.21E-04	PRS-CSx
Colorectum	PRS7	rs10515385	5	107528267	C	A	-4.77E-04	PRS-CSx

Colorectum	PRS7	rs10515388	5	107698701	T	C	-5.19E-04	PRS-CSx
Colorectum	PRS7	rs10515467	5	134543927	C	T	5.34E-05	PRS-CSx
Colorectum	PRS7	rs10515542	5	143758377	G	A	-2.88E-04	PRS-CSx
Colorectum	PRS7	rs10515576	5	146139979	G	T	1.07E-03	PRS-CSx
Colorectum	PRS7	rs10516470	4	102191755	A	G	-1.61E-04	PRS-CSx
Colorectum	PRS7	rs10516511	4	105821431	A	C	-6.12E-04	PRS-CSx
Colorectum	PRS7	rs10516603	4	115467459	G	A	3.81E-04	PRS-CSx
Colorectum	PRS7	rs10517021	4	23662176	C	A	-3.72E-05	PRS-CSx
Colorectum	PRS7	rs10517400	4	58497594	T	C	3.74E-04	PRS-CSx
Colorectum	PRS7	rs10517620	4	156676558	A	G	4.57E-04	PRS-CSx
Colorectum	PRS7	rs10517621	4	156677592	T	C	7.28E-04	PRS-CSx
Colorectum	PRS7	rs10517809	4	165499278	C	T	4.55E-03	PRS-CSx
Colorectum	PRS7	rs1051858	14	58831142	A	G	1.96E-04	PRS-CSx
Colorectum	PRS7	rs1051861	14	58838701	T	C	6.97E-05	PRS-CSx
Colorectum	PRS7	rs10518705	15	67363283	T	G	7.52E-04	PRS-CSx
Colorectum	PRS7	rs10518707	15	67365622	A	G	9.85E-04	PRS-CSx
Colorectum	PRS7	rs10519124	2	67965997	T	C	8.67E-04	PRS-CSx
Colorectum	PRS7	rs10519343	5	112592606	A	G	-5.26E-05	PRS-CSx
Colorectum	PRS7	rs10519346	5	112593030	A	G	-1.75E-04	PRS-CSx
Colorectum	PRS7	rs10519394	4	137604609	A	C	-1.27E-03	PRS-CSx
Colorectum	PRS7	rs10519400	5	114533832	C	A	-1.16E-03	PRS-CSx
Colorectum	PRS7	rs10519629	4	142682770	C	T	6.68E-04	PRS-CSx
Colorectum	PRS7	rs10519756	15	33148207	T	C	1.17E-02	PRS-CSx
Colorectum	PRS7	rs10520017	4	150233792	T	G	-4.06E-04	PRS-CSx
Colorectum	PRS7	rs10520447	4	181300520	A	G	9.57E-04	PRS-CSx
Colorectum	PRS7	rs10521307	16	53865701	G	A	2.72E-03	PRS-CSx
Colorectum	PRS7	rs1052651	12	96052721	A	G	-3.18E-03	PRS-CSx
Colorectum	PRS7	rs1052656	9	34087360	C	T	-2.86E-04	PRS-CSx
Colorectum	PRS7	rs1052878	1	109745618	T	C	1.71E-03	PRS-CSx
Colorectum	PRS7	rs1053862	2	61167761	G	A	-4.37E-04	PRS-CSx
Colorectum	PRS7	rs1055207	9	117428960	T	C	-6.90E-04	PRS-CSx
Colorectum	PRS7	rs1055447	11	47186424	A	C	4.47E-04	PRS-CSx
Colorectum	PRS7	rs1058177	1	183109171	G	A	-8.52E-04	PRS-CSx
Colorectum	PRS7	rs1059293	21	34809693	C	T	5.48E-05	PRS-CSx
Colorectum	PRS7	rs1059513	12	57489709	C	T	1.13E-03	PRS-CSx
Colorectum	PRS7	rs10600	11	33378209	A	G	-2.75E-04	PRS-CSx
Colorectum	PRS7	rs1060573	11	47179829	G	A	3.69E-04	PRS-CSx
Colorectum	PRS7	rs1060780	2	231852394	G	A	-1.28E-04	PRS-CSx
Colorectum	PRS7	rs1061485	2	26149502	G	A	-4.57E-04	PRS-CSx
Colorectum	PRS7	rs1061657	12	115108136	C	T	1.61E-03	PRS-CSx
Colorectum	PRS7	rs1061807	6	32136838	A	G	-1.85E-04	PRS-CSx
Colorectum	PRS7	rs1062044	1	183112412	A	G	3.17E-04	PRS-CSx
Colorectum	PRS7	rs1062059	2	101627852	T	C	4.58E-04	PRS-CSx
Colorectum	PRS7	rs1062158	5	141523000	C	T	4.76E-04	PRS-CSx
Colorectum	PRS7	rs1063192	9	22003367	G	A	8.08E-04	PRS-CSx
Colorectum	PRS7	rs1063497	17	3577146	T	C	8.32E-04	PRS-CSx
Colorectum	PRS7	rs1070446	5	40839228	T	C	1.89E-04	PRS-CSx
Colorectum	PRS7	rs1071882	5	178136040	T	C	5.47E-04	PRS-CSx
Colorectum	PRS7	rs10732824	10	123654025	A	G	8.63E-04	PRS-CSx
Colorectum	PRS7	rs10733471	9	33775367	T	C	-1.90E-04	PRS-CSx
Colorectum	PRS7	rs10733581	9	114731867	T	C	-4.19E-04	PRS-CSx
Colorectum	PRS7	rs10733843	10	70064509	A	G	-1.27E-04	PRS-CSx
Colorectum	PRS7	rs10734415	11	33314798	A	G	-3.41E-04	PRS-CSx
Colorectum	PRS7	rs10734548	11	46787573	C	T	8.46E-05	PRS-CSx
Colorectum	PRS7	rs10734549	11	46822686	A	C	1.71E-04	PRS-CSx
Colorectum	PRS7	rs10736313	10	85761914	A	C	4.57E-04	PRS-CSx

Colorectum	PRS7	rs10738604	9	22025493	G	A	2.95E-04	PRS-CSx
Colorectum	PRS7	rs10738607	9	22088094	A	G	2.51E-03	PRS-CSx
Colorectum	PRS7	rs10738813	9	29436044	C	T	-1.46E-03	PRS-CSx
Colorectum	PRS7	rs10738889	9	32458053	G	A	-5.06E-04	PRS-CSx
Colorectum	PRS7	rs10738890	9	32458081	C	T	-5.83E-04	PRS-CSx
Colorectum	PRS7	rs10740294	10	70061352	T	C	-1.68E-04	PRS-CSx
Colorectum	PRS7	rs10740376	10	73176278	C	T	3.04E-04	PRS-CSx
Colorectum	PRS7	rs10741092	10	25814527	T	G	1.88E-04	PRS-CSx
Colorectum	PRS7	rs10742174	11	27332291	G	A	2.03E-04	PRS-CSx
Colorectum	PRS7	rs10743476	12	23675294	C	T	-3.46E-04	PRS-CSx
Colorectum	PRS7	rs10744177	12	122630009	T	G	2.34E-04	PRS-CSx
Colorectum	PRS7	rs10744335	12	122612146	T	C	2.89E-04	PRS-CSx
Colorectum	PRS7	rs10744732	12	1078076	T	C	7.06E-04	PRS-CSx
Colorectum	PRS7	rs10744769	12	111650780	G	T	-4.56E-06	PRS-CSx
Colorectum	PRS7	rs10744770	12	111662578	G	A	2.28E-04	PRS-CSx
Colorectum	PRS7	rs10744790	12	113409794	T	C	2.43E-04	PRS-CSx
Colorectum	PRS7	rs10745351	1	109743122	T	C	1.55E-03	PRS-CSx
Colorectum	PRS7	rs10746096	12	108261731	G	A	-3.85E-04	PRS-CSx
Colorectum	PRS7	rs10746138	12	79930366	G	A	2.16E-04	PRS-CSx
Colorectum	PRS7	rs10747822	12	59449385	A	G	-3.68E-04	PRS-CSx
Colorectum	PRS7	rs10748784	10	101320120	G	A	-5.94E-05	PRS-CSx
Colorectum	PRS7	rs10749205	10	118046389	T	C	-2.93E-04	PRS-CSx
Colorectum	PRS7	rs10749435	10	123673339	T	C	1.01E-03	PRS-CSx
Colorectum	PRS7	rs10749771	1	67573730	A	G	2.51E-04	PRS-CSx
Colorectum	PRS7	rs10751134	11	85858887	G	A	-4.54E-04	PRS-CSx
Colorectum	PRS7	rs10751657	11	377795	A	G	-2.08E-04	PRS-CSx
Colorectum	PRS7	rs10751758	10	124515520	T	G	1.39E-03	PRS-CSx
Colorectum	PRS7	rs10752046	10	16317194	C	T	2.03E-03	PRS-CSx
Colorectum	PRS7	rs10752143	10	8646048	T	C	1.01E-04	PRS-CSx
Colorectum	PRS7	rs10752146	10	8775730	A	G	7.04E-04	PRS-CSx
Colorectum	PRS7	rs10752179	10	9946658	A	G	2.43E-03	PRS-CSx
Colorectum	PRS7	rs10752748	1	2544640	G	A	-3.58E-05	PRS-CSx
Colorectum	PRS7	rs10752881	1	182973491	A	G	5.08E-04	PRS-CSx
Colorectum	PRS7	rs10752893	1	183046261	C	T	7.42E-04	PRS-CSx
Colorectum	PRS7	rs10752897	1	183071024	C	T	5.52E-04	PRS-CSx
Colorectum	PRS7	rs10752898	1	183077896	T	C	3.56E-04	PRS-CSx
Colorectum	PRS7	rs10755057	2	242710662	T	G	1.16E-03	PRS-CSx
Colorectum	PRS7	rs10757048	9	19281099	G	A	1.32E-04	PRS-CSx
Colorectum	PRS7	rs10757264	9	22019732	A	G	3.48E-04	PRS-CSx
Colorectum	PRS7	rs10757270	9	22072719	A	G	3.50E-04	PRS-CSx
Colorectum	PRS7	rs10757272	9	22088260	C	T	2.05E-03	PRS-CSx
Colorectum	PRS7	rs10757893	9	29599001	A	G	1.11E-03	PRS-CSx
Colorectum	PRS7	rs10758225	9	33759300	A	G	-1.94E-04	PRS-CSx
Colorectum	PRS7	rs10758240	9	34098110	C	T	-6.37E-04	PRS-CSx
Colorectum	PRS7	rs10758242	9	34146776	A	G	-2.24E-04	PRS-CSx
Colorectum	PRS7	rs10758862	9	740472	A	G	-5.59E-04	PRS-CSx
Colorectum	PRS7	rs10760182	9	124412961	A	G	9.37E-04	PRS-CSx
Colorectum	PRS7	rs10760553	9	101620010	A	G	5.05E-04	PRS-CSx
Colorectum	PRS7	rs10760791	9	103878207	A	G	-2.20E-04	PRS-CSx
Colorectum	PRS7	rs1076118	22	43299710	T	C	5.54E-04	PRS-CSx
Colorectum	PRS7	rs10762210	10	70050617	G	T	-1.10E-04	PRS-CSx
Colorectum	PRS7	rs10762212	10	70054947	T	C	-3.50E-05	PRS-CSx
Colorectum	PRS7	rs10762224	10	70086313	T	C	-3.05E-04	PRS-CSx
Colorectum	PRS7	rs1076237	7	99585441	C	A	-1.30E-04	PRS-CSx
Colorectum	PRS7	rs10762498	10	73732665	C	T	-3.40E-04	PRS-CSx
Colorectum	PRS7	rs1076285	7	26585256	T	C	3.27E-05	PRS-CSx

Colorectum	PRS7	rs10762882	10	54496983	G	A	-5.15E-04	PRS-CSx
Colorectum	PRS7	rs10763668	10	29090749	A	G	5.11E-04	PRS-CSx
Colorectum	PRS7	rs10764228	10	20658827	A	G	-1.81E-04	PRS-CSx
Colorectum	PRS7	rs10764730	10	129767555	C	T	6.16E-04	PRS-CSx
Colorectum	PRS7	rs10766209	11	15126447	T	G	1.61E-04	PRS-CSx
Colorectum	PRS7	rs10766291	11	16011729	C	T	-6.17E-04	PRS-CSx
Colorectum	PRS7	rs10766292	11	16013614	T	C	-6.04E-04	PRS-CSx
Colorectum	PRS7	rs10767634	11	27332474	T	C	1.79E-04	PRS-CSx
Colorectum	PRS7	rs10768001	11	33233486	A	G	-2.07E-04	PRS-CSx
Colorectum	PRS7	rs10768003	11	33335130	G	T	-2.47E-04	PRS-CSx
Colorectum	PRS7	rs10768101	11	34811218	G	A	-9.55E-04	PRS-CSx
Colorectum	PRS7	rs10768739	11	41806690	T	C	8.66E-04	PRS-CSx
Colorectum	PRS7	rs10769205	11	46723603	A	G	4.24E-04	PRS-CSx
Colorectum	PRS7	rs10769208	11	46822240	C	T	8.18E-05	PRS-CSx
Colorectum	PRS7	rs10769211	11	46872873	A	G	2.29E-04	PRS-CSx
Colorectum	PRS7	rs10769226	11	46984803	G	A	2.58E-04	PRS-CSx
Colorectum	PRS7	rs10769558	11	49030187	T	C	-8.98E-05	PRS-CSx
Colorectum	PRS7	rs10769750	11	7023541	A	G	-3.60E-04	PRS-CSx
Colorectum	PRS7	rs1077026	13	38457720	C	T	-1.01E-03	PRS-CSx
Colorectum	PRS7	rs10772719	12	14068697	A	G	-9.70E-05	PRS-CSx
Colorectum	PRS7	rs10774214	12	4368352	T	C	7.39E-04	PRS-CSx
Colorectum	PRS7	rs10774223	12	4436632	A	G	7.55E-04	PRS-CSx
Colorectum	PRS7	rs10774542	12	120552851	T	C	3.07E-04	PRS-CSx
Colorectum	PRS7	rs10774623	12	111833589	A	G	4.16E-04	PRS-CSx
Colorectum	PRS7	rs10774667	12	113321965	C	T	-2.17E-04	PRS-CSx
Colorectum	PRS7	rs1077667	19	6668972	T	C	-3.18E-03	PRS-CSx
Colorectum	PRS7	rs10777648	12	95284561	A	G	-4.32E-04	PRS-CSx
Colorectum	PRS7	rs10777683	12	95699929	T	G	3.77E-06	PRS-CSx
Colorectum	PRS7	rs10777716	12	96057387	C	T	-1.51E-03	PRS-CSx
Colorectum	PRS7	rs10778620	12	108700608	C	T	5.23E-04	PRS-CSx
Colorectum	PRS7	rs10778677	12	79939895	C	A	3.24E-04	PRS-CSx
Colorectum	PRS7	rs10778678	12	79944776	G	A	4.79E-04	PRS-CSx
Colorectum	PRS7	rs10778683	12	80024020	G	A	3.40E-04	PRS-CSx
Colorectum	PRS7	rs10778686	12	80071955	A	G	4.64E-05	PRS-CSx
Colorectum	PRS7	rs10779637	1	215244510	T	C	-8.18E-04	PRS-CSx
Colorectum	PRS7	rs1078097	15	71008920	T	C	-6.24E-04	PRS-CSx
Colorectum	PRS7	rs10781516	9	139659901	G	A	3.73E-04	PRS-CSx
Colorectum	PRS7	rs10782046	18	53966763	C	T	-3.50E-04	PRS-CSx
Colorectum	PRS7	rs10782186	6	117823508	C	T	4.53E-04	PRS-CSx
Colorectum	PRS7	rs10783108	1	99551808	T	C	-6.83E-04	PRS-CSx
Colorectum	PRS7	rs10783347	12	50663386	G	A	2.97E-04	PRS-CSx
Colorectum	PRS7	rs10783387	12	51180143	T	C	1.13E-03	PRS-CSx
Colorectum	PRS7	rs10783812	12	57308723	T	C	5.77E-05	PRS-CSx
Colorectum	PRS7	rs10783813	12	57494360	G	T	-5.11E-04	PRS-CSx
Colorectum	PRS7	rs10783931	12	59436179	C	T	-2.83E-04	PRS-CSx
Colorectum	PRS7	rs10785393	12	43282869	A	C	-2.79E-04	PRS-CSx
Colorectum	PRS7	rs10785605	12	46477776	G	A	4.81E-04	PRS-CSx
Colorectum	PRS7	rs10785858	1	104807738	A	G	2.25E-04	PRS-CSx
Colorectum	PRS7	rs10785859	1	104807967	C	T	1.45E-04	PRS-CSx
Colorectum	PRS7	rs1078643	17	10707241	G	A	-1.02E-02	PRS-CSx
Colorectum	PRS7	rs10786744	10	104945028	A	C	5.42E-05	PRS-CSx
Colorectum	PRS7	rs1078679	6	26568741	C	T	-8.13E-05	PRS-CSx
Colorectum	PRS7	rs10786808	10	106384615	T	C	4.76E-04	PRS-CSx
Colorectum	PRS7	rs10788303	10	85761955	A	G	4.38E-04	PRS-CSx
Colorectum	PRS7	rs10788711	10	48550794	T	C	3.86E-04	PRS-CSx
Colorectum	PRS7	rs10789220	1	67551209	C	T	3.23E-04	PRS-CSx

Colorectum	PRS7	rs10790175	11	117034729	C	T	-4.00E-04	PRS-CSx
Colorectum	PRS7	rs10790381	11	120257495	G	A	-2.79E-04	PRS-CSx
Colorectum	PRS7	rs10790383	11	120344526	T	C	-1.60E-04	PRS-CSx
Colorectum	PRS7	rs10791200	11	100008296	T	C	-1.05E-03	PRS-CSx
Colorectum	PRS7	rs10793092	11	74278707	A	G	8.84E-04	PRS-CSx
Colorectum	PRS7	rs10793093	11	74290952	C	T	6.98E-04	PRS-CSx
Colorectum	PRS7	rs10794319	11	405360	A	G	3.04E-06	PRS-CSx
Colorectum	PRS7	rs10795668	10	8701219	A	G	-2.47E-03	PRS-CSx
Colorectum	PRS7	rs10795677	10	8803581	C	T	3.12E-04	PRS-CSx
Colorectum	PRS7	rs10795678	10	8813629	T	C	2.48E-04	PRS-CSx
Colorectum	PRS7	rs10796145	10	6474027	G	A	-9.17E-04	PRS-CSx
Colorectum	PRS7	rs10797432	1	2501338	C	T	-2.15E-04	PRS-CSx
Colorectum	PRS7	rs10797437	1	2539006	T	C	-2.31E-04	PRS-CSx
Colorectum	PRS7	rs10797440	1	2541269	A	G	-1.74E-04	PRS-CSx
Colorectum	PRS7	rs10797829	1	183033228	A	G	2.92E-04	PRS-CSx
Colorectum	PRS7	rs10797835	1	183072908	T	C	4.87E-04	PRS-CSx
Colorectum	PRS7	rs10797838	1	183077020	A	G	2.06E-04	PRS-CSx
Colorectum	PRS7	rs10797839	1	183077797	T	C	4.04E-04	PRS-CSx
Colorectum	PRS7	rs10797842	1	183081867	G	A	2.99E-04	PRS-CSx
Colorectum	PRS7	rs10797854	1	183106739	G	A	2.53E-04	PRS-CSx
Colorectum	PRS7	rs1079811	4	18925941	G	A	-2.86E-04	PRS-CSx
Colorectum	PRS7	rs10798670	1	179334934	G	T	2.66E-04	PRS-CSx
Colorectum	PRS7	rs10799573	1	224625075	T	C	4.05E-04	PRS-CSx
Colorectum	PRS7	rs10799574	1	224625548	T	G	1.93E-04	PRS-CSx
Colorectum	PRS7	rs10799749	1	22734591	A	G	-5.95E-04	PRS-CSx
Colorectum	PRS7	rs10800319	1	167553649	A	G	3.83E-04	PRS-CSx
Colorectum	PRS7	rs10800796	1	201886197	A	G	-1.21E-04	PRS-CSx
Colorectum	PRS7	rs10801344	1	194682062	C	A	-4.38E-04	PRS-CSx
Colorectum	PRS7	rs10801376	1	195019104	T	C	-7.29E-04	PRS-CSx
Colorectum	PRS7	rs10801687	1	89307261	A	G	-2.80E-04	PRS-CSx
Colorectum	PRS7	rs10801772	1	90194899	T	C	5.89E-04	PRS-CSx
Colorectum	PRS7	rs10801892	1	88909218	T	C	5.69E-04	PRS-CSx
Colorectum	PRS7	rs10803934	2	152931410	G	A	-4.79E-04	PRS-CSx
Colorectum	PRS7	rs10804630	3	133997989	T	C	3.59E-04	PRS-CSx
Colorectum	PRS7	rs10804849	3	172514294	G	A	-1.17E-03	PRS-CSx
Colorectum	PRS7	rs10805148	4	46615092	A	G	-3.70E-04	PRS-CSx
Colorectum	PRS7	rs10805421	5	67290836	A	G	5.60E-04	PRS-CSx
Colorectum	PRS7	rs10807922	7	45162209	A	G	-4.20E-04	PRS-CSx
Colorectum	PRS7	rs10808484	8	117679204	A	G	-2.24E-03	PRS-CSx
Colorectum	PRS7	rs10808555	8	128409511	G	A	4.16E-03	PRS-CSx
Colorectum	PRS7	rs10808556	8	128413147	C	T	2.95E-03	PRS-CSx
Colorectum	PRS7	rs10809695	9	1218440	C	T	-7.52E-04	PRS-CSx
Colorectum	PRS7	rs10811371	9	20581841	A	G	2.38E-04	PRS-CSx
Colorectum	PRS7	rs10811650	9	22067593	A	G	1.46E-04	PRS-CSx
Colorectum	PRS7	rs10814030	9	33685854	C	T	-1.14E-04	PRS-CSx
Colorectum	PRS7	rs10814035	9	33705620	T	C	-1.79E-04	PRS-CSx
Colorectum	PRS7	rs10814059	9	34008406	T	C	-2.12E-04	PRS-CSx
Colorectum	PRS7	rs10814062	9	34037738	T	C	-4.80E-05	PRS-CSx
Colorectum	PRS7	rs10814064	9	34051178	A	G	-1.09E-04	PRS-CSx
Colorectum	PRS7	rs10814127	9	34619397	A	G	4.10E-04	PRS-CSx
Colorectum	PRS7	rs10815567	9	733049	G	A	-1.04E-03	PRS-CSx
Colorectum	PRS7	rs10816434	9	109559157	G	A	-2.66E-04	PRS-CSx
Colorectum	PRS7	rs10816438	9	109580032	A	G	-3.61E-04	PRS-CSx
Colorectum	PRS7	rs10818014	9	119990556	A	G	-1.26E-04	PRS-CSx
Colorectum	PRS7	rs10818412	9	122755952	T	C	-6.62E-04	PRS-CSx
Colorectum	PRS7	rs10818583	9	124422261	A	G	-5.72E-04	PRS-CSx

Colorectum	PRS7	rs10819101	9	128768663	C	T	5.28E-04	PRS-CSx
Colorectum	PRS7	rs10820230	9	105446609	A	G	2.12E-04	PRS-CSx
Colorectum	PRS7	rs10820241	9	105464554	G	T	1.68E-04	PRS-CSx
Colorectum	PRS7	rs10820255	9	105489249	C	T	6.11E-04	PRS-CSx
Colorectum	PRS7	rs10820256	9	105489526	T	C	7.01E-04	PRS-CSx
Colorectum	PRS7	rs10820259	9	105501591	T	C	4.84E-04	PRS-CSx
Colorectum	PRS7	rs10821409	9	93173645	A	G	-1.49E-04	PRS-CSx
Colorectum	PRS7	rs10821602	10	61571336	C	T	2.62E-03	PRS-CSx
Colorectum	PRS7	rs10823171	10	70051928	A	G	9.81E-06	PRS-CSx
Colorectum	PRS7	rs10823741	10	73178389	C	T	3.39E-04	PRS-CSx
Colorectum	PRS7	rs10823743	10	73180767	T	C	2.24E-04	PRS-CSx
Colorectum	PRS7	rs10823892	10	73722349	T	C	-9.09E-05	PRS-CSx
Colorectum	PRS7	rs10823907	10	73960567	T	C	-7.72E-04	PRS-CSx
Colorectum	PRS7	rs10824779	10	54488855	A	G	-6.93E-04	PRS-CSx
Colorectum	PRS7	rs10826519	10	29090606	G	A	3.11E-04	PRS-CSx
Colorectum	PRS7	rs10826979	10	31987246	C	T	2.79E-03	PRS-CSx
Colorectum	PRS7	rs10827974	10	20443727	C	T	-2.17E-04	PRS-CSx
Colorectum	PRS7	rs10827997	10	20500743	T	G	-2.88E-04	PRS-CSx
Colorectum	PRS7	rs10828810	10	25788668	T	C	1.43E-04	PRS-CSx
Colorectum	PRS7	rs10832349	11	15081335	A	G	3.37E-04	PRS-CSx
Colorectum	PRS7	rs10832351	11	15082933	G	A	2.84E-04	PRS-CSx
Colorectum	PRS7	rs10832355	11	15094057	A	G	3.01E-04	PRS-CSx
Colorectum	PRS7	rs10833056	11	19105332	C	T	-5.24E-04	PRS-CSx
Colorectum	PRS7	rs10833470	11	21190026	A	C	-1.80E-04	PRS-CSx
Colorectum	PRS7	rs10833473	11	21197017	C	T	-3.09E-04	PRS-CSx
Colorectum	PRS7	rs10833989	11	23244871	A	G	-1.45E-03	PRS-CSx
Colorectum	PRS7	rs10835156	11	27325218	C	T	1.96E-04	PRS-CSx
Colorectum	PRS7	rs10835161	11	27338689	T	G	2.75E-04	PRS-CSx
Colorectum	PRS7	rs10836021	11	33118888	G	T	-2.06E-04	PRS-CSx
Colorectum	PRS7	rs10836026	11	33166062	T	G	-1.35E-04	PRS-CSx
Colorectum	PRS7	rs10836028	11	33169687	C	A	-2.39E-04	PRS-CSx
Colorectum	PRS7	rs10836036	11	33202683	G	A	-1.41E-04	PRS-CSx
Colorectum	PRS7	rs10836046	11	33291462	G	A	-2.81E-04	PRS-CSx
Colorectum	PRS7	rs10836049	11	33305142	A	G	-3.66E-04	PRS-CSx
Colorectum	PRS7	rs10836279	11	34718222	A	G	3.71E-04	PRS-CSx
Colorectum	PRS7	rs10836561	11	36457253	T	C	-8.29E-04	PRS-CSx
Colorectum	PRS7	rs10838447	11	45344554	A	G	-3.43E-04	PRS-CSx
Colorectum	PRS7	rs10838517	11	5695743	T	C	8.32E-04	PRS-CSx
Colorectum	PRS7	rs10838612	11	46701728	G	T	2.00E-04	PRS-CSx
Colorectum	PRS7	rs10838635	11	46945227	A	G	1.45E-04	PRS-CSx
Colorectum	PRS7	rs10838652	11	47077837	T	C	2.14E-04	PRS-CSx
Colorectum	PRS7	rs10838660	11	47152273	A	G	3.52E-04	PRS-CSx
Colorectum	PRS7	rs10838662	11	47184117	T	G	3.29E-05	PRS-CSx
Colorectum	PRS7	rs10838663	11	47184971	C	T	1.77E-04	PRS-CSx
Colorectum	PRS7	rs10838664	11	47203904	G	A	2.09E-04	PRS-CSx
Colorectum	PRS7	rs10838681	11	47275064	G	A	1.06E-04	PRS-CSx
Colorectum	PRS7	rs10839210	11	49033342	T	C	-1.84E-04	PRS-CSx
Colorectum	PRS7	rs10842458	12	25227302	T	C	-4.49E-04	PRS-CSx
Colorectum	PRS7	rs10842460	12	25227717	A	C	-5.22E-05	PRS-CSx
Colorectum	PRS7	rs10842496	12	25311489	T	G	-1.59E-04	PRS-CSx
Colorectum	PRS7	rs10842501	12	25343516	C	T	-4.95E-04	PRS-CSx
Colorectum	PRS7	rs10842514	12	25392282	C	T	-3.85E-04	PRS-CSx
Colorectum	PRS7	rs10843085	12	28231087	A	G	5.03E-04	PRS-CSx
Colorectum	PRS7	rs10843264	12	29002413	A	G	-8.95E-04	PRS-CSx
Colorectum	PRS7	rs10843986	12	31794885	C	T	-6.62E-04	PRS-CSx
Colorectum	PRS7	rs10845046	12	10269332	G	A	-8.98E-04	PRS-CSx

Colorectum	PRS7	rs10845827	12	13859064	G	A	-7.12E-04	PRS-CSx
Colorectum	PRS7	rs10845858	12	14045718	A	G	-3.32E-04	PRS-CSx
Colorectum	PRS7	rs10845861	12	14075637	C	T	-6.42E-04	PRS-CSx
Colorectum	PRS7	rs10845863	12	14078939	T	C	-3.05E-04	PRS-CSx
Colorectum	PRS7	rs10846392	12	16825623	T	C	-6.90E-04	PRS-CSx
Colorectum	PRS7	rs10849	3	37095070	T	C	-3.38E-04	PRS-CSx
Colorectum	PRS7	rs10849027	12	4376494	A	G	4.01E-03	PRS-CSx
Colorectum	PRS7	rs10849732	12	120439937	A	G	1.72E-04	PRS-CSx
Colorectum	PRS7	rs10849736	12	120480014	G	A	9.08E-05	PRS-CSx
Colorectum	PRS7	rs108499	11	61547237	C	T	5.02E-04	PRS-CSx
Colorectum	PRS7	rs10849918	12	111376921	C	T	3.07E-04	PRS-CSx
Colorectum	PRS7	rs10849920	12	111377051	T	C	3.00E-04	PRS-CSx
Colorectum	PRS7	rs10849938	12	111800059	T	C	2.45E-04	PRS-CSx
Colorectum	PRS7	rs10849944	12	111825389	T	C	4.63E-04	PRS-CSx
Colorectum	PRS7	rs10849949	12	111893537	A	G	3.75E-04	PRS-CSx
Colorectum	PRS7	rs10850031	12	112771063	T	G	2.31E-04	PRS-CSx
Colorectum	PRS7	rs10850075	12	113182298	G	A	-4.07E-05	PRS-CSx
Colorectum	PRS7	rs10850076	12	113183486	T	C	1.46E-04	PRS-CSx
Colorectum	PRS7	rs10850078	12	113209519	A	G	5.31E-05	PRS-CSx
Colorectum	PRS7	rs10850088	12	113319778	G	A	-2.67E-04	PRS-CSx
Colorectum	PRS7	rs10850089	12	113323067	C	T	-2.74E-04	PRS-CSx
Colorectum	PRS7	rs10850109	12	113414168	T	C	2.26E-04	PRS-CSx
Colorectum	PRS7	rs10850521	12	115932519	T	C	4.87E-04	PRS-CSx
Colorectum	PRS7	rs10850522	12	115932534	A	G	5.22E-04	PRS-CSx
Colorectum	PRS7	rs10850524	12	115932661	C	A	6.17E-04	PRS-CSx
Colorectum	PRS7	rs10850532	12	115960553	A	G	-6.19E-04	PRS-CSx
Colorectum	PRS7	rs10850535	12	116022506	C	T	-3.12E-04	PRS-CSx
Colorectum	PRS7	rs10850555	12	116155595	G	A	8.80E-05	PRS-CSx
Colorectum	PRS7	rs1085071	3	129561896	A	G	2.48E-04	PRS-CSx
Colorectum	PRS7	rs10850956	12	118518357	A	G	6.34E-04	PRS-CSx
Colorectum	PRS7	rs10851029	12	119191669	A	G	7.07E-04	PRS-CSx
Colorectum	PRS7	rs10851568	15	31758244	A	G	4.75E-04	PRS-CSx
Colorectum	PRS7	rs10851759	15	66794037	A	C	-2.99E-04	PRS-CSx
Colorectum	PRS7	rs10851840	15	71635928	T	C	5.32E-04	PRS-CSx
Colorectum	PRS7	rs10851884	15	76290042	G	A	-1.92E-04	PRS-CSx
Colorectum	PRS7	rs10851954	15	82087409	C	T	-1.70E-04	PRS-CSx
Colorectum	PRS7	rs10853517	18	42039010	A	G	3.17E-04	PRS-CSx
Colorectum	PRS7	rs10853751	19	41903220	A	G	1.99E-04	PRS-CSx
Colorectum	PRS7	rs10853966	19	3609005	A	G	5.93E-04	PRS-CSx
Colorectum	PRS7	rs10856903	4	94824607	A	G	3.07E-04	PRS-CSx
Colorectum	PRS7	rs10857139	4	130222259	C	T	8.30E-04	PRS-CSx
Colorectum	PRS7	rs10858081	1	109741516	A	C	2.06E-03	PRS-CSx
Colorectum	PRS7	rs10859871	12	95711876	C	A	-8.67E-05	PRS-CSx
Colorectum	PRS7	rs10859919	12	96054670	T	C	-1.37E-03	PRS-CSx
Colorectum	PRS7	rs10860151	12	97534818	T	C	1.27E-04	PRS-CSx
Colorectum	PRS7	rs10860152	12	97538337	A	G	1.41E-04	PRS-CSx
Colorectum	PRS7	rs10860645	12	101239426	T	C	-3.14E-04	PRS-CSx
Colorectum	PRS7	rs10860648	12	101245602	C	T	-5.84E-04	PRS-CSx
Colorectum	PRS7	rs10861719	12	107663554	A	G	5.58E-04	PRS-CSx
Colorectum	PRS7	rs10861950	12	108978949	C	T	7.76E-04	PRS-CSx
Colorectum	PRS7	rs10862007	12	80049965	G	A	1.91E-04	PRS-CSx
Colorectum	PRS7	rs10862805	12	84357221	C	T	5.02E-04	PRS-CSx
Colorectum	PRS7	rs10863595	1	221393875	T	C	3.23E-04	PRS-CSx
Colorectum	PRS7	rs10863651	1	221909193	G	A	1.02E-04	PRS-CSx
Colorectum	PRS7	rs10863668	1	222090944	A	G	-1.97E-04	PRS-CSx
Colorectum	PRS7	rs10864370	1	8985036	T	C	1.68E-03	PRS-CSx

Colorectum	PRS7	rs10865428	2	77429576	T	C	7.52E-04	PRS-CSx
Colorectum	PRS7	rs10866272	4	185884206	A	G	-1.04E-03	PRS-CSx
Colorectum	PRS7	rs10867960	9	85851428	C	T	5.42E-04	PRS-CSx
Colorectum	PRS7	rs10868132	9	86890592	G	T	6.16E-04	PRS-CSx
Colorectum	PRS7	rs10868134	9	86892119	G	A	1.19E-03	PRS-CSx
Colorectum	PRS7	rs10871349	16	78361149	A	G	-7.94E-04	PRS-CSx
Colorectum	PRS7	rs10871454	16	31048079	C	T	3.03E-04	PRS-CSx
Colorectum	PRS7	rs10872303	6	126255034	C	T	6.74E-04	PRS-CSx
Colorectum	PRS7	rs10873301	14	77507205	T	G	-4.60E-04	PRS-CSx
Colorectum	PRS7	rs10873325	14	80461289	C	T	4.72E-04	PRS-CSx
Colorectum	PRS7	rs10873326	14	80462899	G	T	2.77E-04	PRS-CSx
Colorectum	PRS7	rs10874471	2	96038093	C	T	-3.78E-04	PRS-CSx
Colorectum	PRS7	rs10875131	1	98645192	A	G	-2.79E-04	PRS-CSx
Colorectum	PRS7	rs10875328	1	101169328	A	G	2.17E-04	PRS-CSx
Colorectum	PRS7	rs10875560	5	150245724	G	A	-6.60E-04	PRS-CSx
Colorectum	PRS7	rs10875855	12	38974970	G	A	1.92E-04	PRS-CSx
Colorectum	PRS7	rs10876098	12	51220373	T	G	9.43E-04	PRS-CSx
Colorectum	PRS7	rs10876951	12	57306430	G	T	8.49E-05	PRS-CSx
Colorectum	PRS7	rs10876966	12	57543572	T	C	-1.51E-04	PRS-CSx
Colorectum	PRS7	rs1087838	15	56733662	G	T	9.04E-04	PRS-CSx
Colorectum	PRS7	rs10879020	12	70212497	C	A	3.37E-04	PRS-CSx
Colorectum	PRS7	rs10879178	12	71058733	G	A	8.33E-04	PRS-CSx
Colorectum	PRS7	rs10880386	12	43295999	T	C	5.80E-04	PRS-CSx
Colorectum	PRS7	rs10880908	12	46479056	C	T	5.66E-04	PRS-CSx
Colorectum	PRS7	rs10883365	10	101287764	G	A	-6.90E-04	PRS-CSx
Colorectum	PRS7	rs10883367	10	101287990	T	C	-5.94E-04	PRS-CSx
Colorectum	PRS7	rs10883368	10	101289998	G	A	5.80E-04	PRS-CSx
Colorectum	PRS7	rs10883371	10	101292455	C	A	-6.89E-04	PRS-CSx
Colorectum	PRS7	rs10883373	10	101292484	G	A	-6.63E-04	PRS-CSx
Colorectum	PRS7	rs10883477	10	102161396	G	A	-9.46E-05	PRS-CSx
Colorectum	PRS7	rs10883479	10	102162860	G	A	-1.59E-04	PRS-CSx
Colorectum	PRS7	rs10883483	10	102174244	C	T	-1.34E-04	PRS-CSx
Colorectum	PRS7	rs10883497	10	102228519	C	T	-2.27E-04	PRS-CSx
Colorectum	PRS7	rs10883820	10	104764661	A	C	4.93E-05	PRS-CSx
Colorectum	PRS7	rs10883839	10	104920341	G	A	-3.22E-04	PRS-CSx
Colorectum	PRS7	rs10885355	10	114258451	A	G	-1.02E-03	PRS-CSx
Colorectum	PRS7	rs10886048	10	118938882	T	C	1.18E-03	PRS-CSx
Colorectum	PRS7	rs10887193	10	85738811	T	C	1.62E-04	PRS-CSx
Colorectum	PRS7	rs10887195	10	85750023	C	T	2.22E-04	PRS-CSx
Colorectum	PRS7	rs10887272	10	86058516	T	C	-8.40E-04	PRS-CSx
Colorectum	PRS7	rs10887908	10	90856844	G	A	4.52E-04	PRS-CSx
Colorectum	PRS7	rs10888197	10	48547560	A	G	7.93E-04	PRS-CSx
Colorectum	PRS7	rs10889095	1	40442552	C	T	-1.24E-03	PRS-CSx
Colorectum	PRS7	rs10891240	11	111140462	G	A	-8.70E-04	PRS-CSx
Colorectum	PRS7	rs10891244	11	111165940	T	G	-1.51E-03	PRS-CSx
Colorectum	PRS7	rs10891245	11	111167792	G	T	-1.70E-03	PRS-CSx
Colorectum	PRS7	rs10892079	11	117016824	G	T	-5.80E-05	PRS-CSx
Colorectum	PRS7	rs10892563	11	120224544	C	T	-1.05E-04	PRS-CSx
Colorectum	PRS7	rs10892582	11	120347715	T	C	-2.31E-04	PRS-CSx
Colorectum	PRS7	rs10892774	11	121649479	T	C	1.09E-03	PRS-CSx
Colorectum	PRS7	rs10893354	11	125113503	A	G	5.48E-04	PRS-CSx
Colorectum	PRS7	rs10893360	11	125125225	A	C	7.20E-04	PRS-CSx
Colorectum	PRS7	rs10894151	11	99781356	A	G	6.99E-04	PRS-CSx
Colorectum	PRS7	rs10896016	11	65335705	G	A	-1.17E-04	PRS-CSx
Colorectum	PRS7	rs10897165	11	61277885	A	G	1.03E-03	PRS-CSx
Colorectum	PRS7	rs10897924	11	69921329	C	T	5.90E-04	PRS-CSx

Colorectum	PRS7	rs10898433	11	85780582	T	G	2.52E-04	PRS-CSx
Colorectum	PRS7	rs10898437	11	85847881	C	T	2.45E-04	PRS-CSx
Colorectum	PRS7	rs10898906	11	72917269	C	A	-3.34E-04	PRS-CSx
Colorectum	PRS7	rs10898995	11	74221170	T	C	2.42E-04	PRS-CSx
Colorectum	PRS7	rs10899013	11	74309542	T	C	-3.57E-04	PRS-CSx
Colorectum	PRS7	rs10899024	11	74358682	G	A	6.92E-04	PRS-CSx
Colorectum	PRS7	rs10901663	10	128390819	T	G	5.07E-04	PRS-CSx
Colorectum	PRS7	rs10902158	11	396308	G	A	7.68E-04	PRS-CSx
Colorectum	PRS7	rs10902164	11	432213	G	A	3.06E-04	PRS-CSx
Colorectum	PRS7	rs10902165	11	434659	G	A	3.11E-04	PRS-CSx
Colorectum	PRS7	rs10902877	10	124843587	G	A	-6.20E-04	PRS-CSx
Colorectum	PRS7	rs10905324	10	8263831	C	T	-6.18E-04	PRS-CSx
Colorectum	PRS7	rs10905416	10	8643129	T	G	-6.67E-05	PRS-CSx
Colorectum	PRS7	rs10905417	10	8645723	C	T	-1.17E-05	PRS-CSx
Colorectum	PRS7	rs10905431	10	8679215	T	C	-7.17E-05	PRS-CSx
Colorectum	PRS7	rs10905437	10	8695795	G	A	1.02E-03	PRS-CSx
Colorectum	PRS7	rs10905449	10	8728419	C	T	-1.52E-03	PRS-CSx
Colorectum	PRS7	rs10905453	10	8744021	A	G	7.86E-04	PRS-CSx
Colorectum	PRS7	rs10905455	10	8773143	C	T	1.27E-03	PRS-CSx
Colorectum	PRS7	rs10905530	10	9268122	G	A	-3.75E-04	PRS-CSx
Colorectum	PRS7	rs10905806	10	6152310	A	G	-5.56E-04	PRS-CSx
Colorectum	PRS7	rs10911184	1	182965667	T	C	5.77E-04	PRS-CSx
Colorectum	PRS7	rs10911191	1	182984524	T	C	6.88E-04	PRS-CSx
Colorectum	PRS7	rs10911194	1	182993025	C	T	3.70E-04	PRS-CSx
Colorectum	PRS7	rs10911232	1	183052533	C	T	8.01E-04	PRS-CSx
Colorectum	PRS7	rs10911233	1	183052585	G	A	8.60E-04	PRS-CSx
Colorectum	PRS7	rs10911260	1	183106230	C	A	3.45E-04	PRS-CSx
Colorectum	PRS7	rs10911269	1	183134719	A	G	-1.70E-04	PRS-CSx
Colorectum	PRS7	rs10915437	1	4183006	G	A	8.81E-04	PRS-CSx
Colorectum	PRS7	rs10916832	1	20945644	C	T	-8.11E-04	PRS-CSx
Colorectum	PRS7	rs10916833	1	20945686	A	G	-5.27E-04	PRS-CSx
Colorectum	PRS7	rs10917196	1	22622513	T	C	2.61E-04	PRS-CSx
Colorectum	PRS7	rs10917216	1	22665400	C	A	3.61E-04	PRS-CSx
Colorectum	PRS7	rs10917220	1	22683405	T	C	8.14E-04	PRS-CSx
Colorectum	PRS7	rs10917225	1	22733144	T	C	-3.87E-04	PRS-CSx
Colorectum	PRS7	rs10918081	1	164825891	C	T	6.35E-05	PRS-CSx
Colorectum	PRS7	rs10918091	1	164885666	A	C	3.88E-04	PRS-CSx
Colorectum	PRS7	rs10918095	1	164924633	G	A	2.39E-04	PRS-CSx
Colorectum	PRS7	rs10918667	1	167180137	C	T	4.06E-04	PRS-CSx
Colorectum	PRS7	rs10918695	1	167437953	T	C	-8.60E-05	PRS-CSx
Colorectum	PRS7	rs10919830	1	200154474	A	G	5.48E-04	PRS-CSx
Colorectum	PRS7	rs10921283	1	192870469	G	A	2.66E-04	PRS-CSx
Colorectum	PRS7	rs10921286	1	192889452	C	T	-1.14E-03	PRS-CSx
Colorectum	PRS7	rs10921643	1	194718327	T	C	-9.25E-04	PRS-CSx
Colorectum	PRS7	rs10921647	1	194724361	A	G	-4.00E-04	PRS-CSx
Colorectum	PRS7	rs10923081	1	88950775	C	T	-1.36E-04	PRS-CSx
Colorectum	PRS7	rs10924361	1	246014333	A	G	-6.96E-04	PRS-CSx
Colorectum	PRS7	rs10924366	1	246027410	C	T	1.28E-03	PRS-CSx
Colorectum	PRS7	rs10925076	1	247702886	A	G	-6.37E-04	PRS-CSx
Colorectum	PRS7	rs10926185	1	240424857	C	T	8.45E-04	PRS-CSx
Colorectum	PRS7	rs10927957	1	14249565	G	T	7.36E-04	PRS-CSx
Colorectum	PRS7	rs10928514	2	131800593	C	T	-2.05E-04	PRS-CSx
Colorectum	PRS7	rs10928516	2	131802332	A	G	-4.14E-04	PRS-CSx
Colorectum	PRS7	rs10928539	2	131889179	T	C	-3.95E-04	PRS-CSx
Colorectum	PRS7	rs10929543	2	8735215	C	A	-4.31E-04	PRS-CSx
Colorectum	PRS7	rs10929838	2	5958997	C	A	-6.10E-04	PRS-CSx

Colorectum	PRS7	rs10929934	2	159969451	T	C	-4.02E-04	PRS-CSx
Colorectum	PRS7	rs10929936	2	160030596	T	G	-5.71E-04	PRS-CSx
Colorectum	PRS7	rs10929989	2	161351779	A	G	1.73E-04	PRS-CSx
Colorectum	PRS7	rs10931822	2	199514757	T	G	1.90E-04	PRS-CSx
Colorectum	PRS7	rs10932127	2	206667756	G	T	-2.10E-03	PRS-CSx
Colorectum	PRS7	rs10932182	2	208070211	T	C	-1.50E-04	PRS-CSx
Colorectum	PRS7	rs10932629	2	216706843	T	C	-5.97E-04	PRS-CSx
Colorectum	PRS7	rs10933242	2	229071362	C	T	-3.73E-04	PRS-CSx
Colorectum	PRS7	rs10933363	2	231857076	A	G	-8.20E-05	PRS-CSx
Colorectum	PRS7	rs10934222	3	112872364	T	C	-9.91E-04	PRS-CSx
Colorectum	PRS7	rs10934223	3	112878536	G	A	-4.72E-04	PRS-CSx
Colorectum	PRS7	rs10934229	3	113146697	C	T	1.99E-04	PRS-CSx
Colorectum	PRS7	rs10935097	3	133822560	A	C	1.97E-04	PRS-CSx
Colorectum	PRS7	rs10935101	3	133863617	A	G	1.55E-04	PRS-CSx
Colorectum	PRS7	rs10936592	3	169425402	G	A	1.73E-04	PRS-CSx
Colorectum	PRS7	rs10936599	3	169492101	C	T	7.82E-04	PRS-CSx
Colorectum	PRS7	rs10936625	3	170094908	T	G	-5.19E-04	PRS-CSx
Colorectum	PRS7	rs10936840	3	175015289	G	T	7.60E-04	PRS-CSx
Colorectum	PRS7	rs10938449	4	46625274	G	A	-6.32E-04	PRS-CSx
Colorectum	PRS7	rs10940145	5	67290972	A	G	7.75E-04	PRS-CSx
Colorectum	PRS7	rs10941454	5	39737356	G	A	4.32E-04	PRS-CSx
Colorectum	PRS7	rs10946384	6	20495546	T	C	1.22E-03	PRS-CSx
Colorectum	PRS7	rs10946398	6	20661034	C	A	-7.06E-04	PRS-CSx
Colorectum	PRS7	rs10946403	6	20717404	G	A	-1.35E-04	PRS-CSx
Colorectum	PRS7	rs10946506	6	22074193	C	T	1.87E-03	PRS-CSx
Colorectum	PRS7	rs10946896	6	26988576	G	A	-4.11E-04	PRS-CSx
Colorectum	PRS7	rs10947075	6	11269466	C	A	5.65E-04	PRS-CSx
Colorectum	PRS7	rs10947670	6	11952845	G	A	4.02E-04	PRS-CSx
Colorectum	PRS7	rs10947819	6	12173757	G	T	1.28E-03	PRS-CSx
Colorectum	PRS7	rs10947982	6	12384972	A	G	4.91E-04	PRS-CSx
Colorectum	PRS7	rs10948108	6	43904294	C	T	-4.69E-04	PRS-CSx
Colorectum	PRS7	rs10948109	6	43904394	A	G	-8.10E-04	PRS-CSx
Colorectum	PRS7	rs10948110	6	43906364	T	C	-3.63E-04	PRS-CSx
Colorectum	PRS7	rs10948112	6	43910164	A	G	-4.91E-04	PRS-CSx
Colorectum	PRS7	rs10951100	7	25771151	C	T	-4.86E-04	PRS-CSx
Colorectum	PRS7	rs10951792	7	45086665	G	A	-5.42E-04	PRS-CSx
Colorectum	PRS7	rs10951794	7	45114962	A	G	-4.17E-04	PRS-CSx
Colorectum	PRS7	rs10951847	7	46226952	C	T	-9.42E-04	PRS-CSx
Colorectum	PRS7	rs10951911	7	47637239	C	T	-1.10E-03	PRS-CSx
Colorectum	PRS7	rs10953089	7	92728427	C	T	5.70E-04	PRS-CSx
Colorectum	PRS7	rs10954222	7	128795384	A	G	-4.83E-04	PRS-CSx
Colorectum	PRS7	rs10955140	8	99014550	T	C	-6.23E-04	PRS-CSx
Colorectum	PRS7	rs10955143	8	99016092	G	A	-4.42E-04	PRS-CSx
Colorectum	PRS7	rs10955793	8	117842055	C	T	1.18E-04	PRS-CSx
Colorectum	PRS7	rs10956368	8	128423650	T	C	2.95E-03	PRS-CSx
Colorectum	PRS7	rs10956694	8	134188948	A	G	-1.15E-03	PRS-CSx
Colorectum	PRS7	rs10956949	8	96436087	G	A	-7.28E-04	PRS-CSx
Colorectum	PRS7	rs10957017	8	58629835	A	G	5.75E-04	PRS-CSx
Colorectum	PRS7	rs10957100	8	60469380	G	A	-1.07E-03	PRS-CSx
Colorectum	PRS7	rs10960475	9	1218816	C	T	-8.79E-04	PRS-CSx
Colorectum	PRS7	rs10964075	9	19267851	C	T	3.76E-03	PRS-CSx
Colorectum	PRS7	rs10964573	9	20489026	G	A	4.65E-04	PRS-CSx
Colorectum	PRS7	rs10965215	9	22029445	G	A	3.09E-04	PRS-CSx
Colorectum	PRS7	rs10965219	9	22053687	A	G	1.79E-04	PRS-CSx
Colorectum	PRS7	rs10970464	9	326400	A	G	-2.39E-04	PRS-CSx
Colorectum	PRS7	rs10970468	9	326448	A	G	-1.66E-04	PRS-CSx

Colorectum	PRS7	rs10970986	9	32453278	C	T	-6.12E-04	PRS-CSx
Colorectum	PRS7	rs10971632	9	33665229	G	T	-1.53E-04	PRS-CSx
Colorectum	PRS7	rs10971677	9	33750237	T	C	-5.33E-05	PRS-CSx
Colorectum	PRS7	rs10971679	9	33751865	A	C	-2.04E-04	PRS-CSx
Colorectum	PRS7	rs10971709	9	33804813	C	T	-4.54E-04	PRS-CSx
Colorectum	PRS7	rs10971711	9	33815842	C	T	-3.79E-04	PRS-CSx
Colorectum	PRS7	rs10971720	9	33827153	G	A	-2.53E-04	PRS-CSx
Colorectum	PRS7	rs10971738	9	33855138	T	C	-3.20E-04	PRS-CSx
Colorectum	PRS7	rs10971745	9	33862508	A	G	-7.06E-04	PRS-CSx
Colorectum	PRS7	rs10971789	9	33915558	T	C	-3.58E-04	PRS-CSx
Colorectum	PRS7	rs10971836	9	33992591	C	T	-4.20E-04	PRS-CSx
Colorectum	PRS7	rs10972162	9	34600759	T	G	1.01E-03	PRS-CSx
Colorectum	PRS7	rs10972168	9	34619621	G	A	7.56E-04	PRS-CSx
Colorectum	PRS7	rs10972175	9	34625409	A	G	5.36E-04	PRS-CSx
Colorectum	PRS7	rs10972195	9	34683607	A	C	3.56E-04	PRS-CSx
Colorectum	PRS7	rs10972770	9	36169187	A	C	-4.79E-04	PRS-CSx
Colorectum	PRS7	rs10974007	9	38738620	C	T	1.39E-03	PRS-CSx
Colorectum	PRS7	rs10975135	9	5488002	C	T	-4.86E-04	PRS-CSx
Colorectum	PRS7	rs10978077	9	9908296	G	A	9.33E-04	PRS-CSx
Colorectum	PRS7	rs10978651	9	109579213	T	C	-3.09E-04	PRS-CSx
Colorectum	PRS7	rs10978653	9	109589226	T	C	-3.05E-04	PRS-CSx
Colorectum	PRS7	rs10978664	9	109640836	G	A	-2.89E-04	PRS-CSx
Colorectum	PRS7	rs10979018	9	110469181	T	C	-9.48E-04	PRS-CSx
Colorectum	PRS7	rs10980551	9	113504910	G	A	-5.71E-04	PRS-CSx
Colorectum	PRS7	rs10980564	9	113532450	A	G	-3.34E-04	PRS-CSx
Colorectum	PRS7	rs10980596	9	113619757	A	C	3.45E-04	PRS-CSx
Colorectum	PRS7	rs10980607	9	113638375	T	C	7.80E-04	PRS-CSx
Colorectum	PRS7	rs10982124	9	117015956	A	G	1.01E-03	PRS-CSx
Colorectum	PRS7	rs10982373	9	117463262	A	G	-5.89E-04	PRS-CSx
Colorectum	PRS7	rs10983582	9	120010216	A	G	-8.26E-04	PRS-CSx
Colorectum	PRS7	rs10984020	9	121169876	A	G	9.43E-04	PRS-CSx
Colorectum	PRS7	rs10990270	9	105503295	C	T	1.70E-04	PRS-CSx
Colorectum	PRS7	rs10991732	9	93724255	A	G	1.85E-03	PRS-CSx
Colorectum	PRS7	rs10993895	9	136887726	T	C	-4.47E-04	PRS-CSx
Colorectum	PRS7	rs10993907	9	136928644	C	T	6.82E-04	PRS-CSx
Colorectum	PRS7	rs10994023	10	61562977	G	A	1.97E-03	PRS-CSx
Colorectum	PRS7	rs10995315	10	64574571	A	G	2.53E-04	PRS-CSx
Colorectum	PRS7	rs11000213	10	73955819	C	T	-7.31E-04	PRS-CSx
Colorectum	PRS7	rs11000214	10	73960018	C	T	-8.80E-04	PRS-CSx
Colorectum	PRS7	rs11000218	10	73964589	C	T	-4.92E-04	PRS-CSx
Colorectum	PRS7	rs11003084	10	54488686	T	C	-2.66E-04	PRS-CSx
Colorectum	PRS7	rs11011827	10	20436081	A	C	-3.31E-04	PRS-CSx
Colorectum	PRS7	rs11011833	10	20445776	T	C	-2.65E-04	PRS-CSx
Colorectum	PRS7	rs11011852	10	20481020	G	A	-2.79E-04	PRS-CSx
Colorectum	PRS7	rs11011875	10	20503108	G	A	-5.03E-04	PRS-CSx
Colorectum	PRS7	rs11012730	10	21811487	A	G	3.17E-04	PRS-CSx
Colorectum	PRS7	rs11014581	10	25791510	T	G	2.38E-04	PRS-CSx
Colorectum	PRS7	rs11014603	10	25839112	G	A	1.05E-04	PRS-CSx
Colorectum	PRS7	rs11021294	11	95445705	T	C	-3.97E-04	PRS-CSx
Colorectum	PRS7	rs11023409	11	15026465	A	G	3.16E-04	PRS-CSx
Colorectum	PRS7	rs11023424	11	15095985	T	G	2.28E-04	PRS-CSx
Colorectum	PRS7	rs11023821	11	16022866	G	A	-6.74E-04	PRS-CSx
Colorectum	PRS7	rs11025240	11	3090062	A	C	-4.27E-04	PRS-CSx
Colorectum	PRS7	rs11029932	11	27328254	T	C	2.20E-04	PRS-CSx
Colorectum	PRS7	rs11032230	11	33362866	G	T	-1.74E-04	PRS-CSx
Colorectum	PRS7	rs11032742	11	34543942	T	G	5.68E-04	PRS-CSx

Colorectum	PRS7	rs11032858	11	34794889	A	G	4.12E-04	PRS-CSx
Colorectum	PRS7	rs11032877	11	34815099	C	T	4.93E-04	PRS-CSx
Colorectum	PRS7	rs11033617	11	36455949	C	T	9.24E-04	PRS-CSx
Colorectum	PRS7	rs11033799	11	36784768	T	C	6.40E-04	PRS-CSx
Colorectum	PRS7	rs11038642	11	5690003	T	C	1.21E-03	PRS-CSx
Colorectum	PRS7	rs11038977	11	46734384	C	T	2.98E-04	PRS-CSx
Colorectum	PRS7	rs11038993	11	46810916	A	C	2.63E-04	PRS-CSx
Colorectum	PRS7	rs11039002	11	46857279	T	C	5.84E-05	PRS-CSx
Colorectum	PRS7	rs11039014	11	46895378	A	G	1.09E-04	PRS-CSx
Colorectum	PRS7	rs11039024	11	46923168	C	T	1.73E-04	PRS-CSx
Colorectum	PRS7	rs11039035	11	46967415	T	G	1.79E-04	PRS-CSx
Colorectum	PRS7	rs11039097	11	47131881	A	G	2.48E-04	PRS-CSx
Colorectum	PRS7	rs11039112	11	47174296	C	T	3.43E-04	PRS-CSx
Colorectum	PRS7	rs11039119	11	47201924	G	A	3.08E-04	PRS-CSx
Colorectum	PRS7	rs11040780	11	6193544	C	T	-7.25E-04	PRS-CSx
Colorectum	PRS7	rs11040781	11	6194610	G	A	-1.12E-03	PRS-CSx
Colorectum	PRS7	rs11042686	11	10264122	G	A	4.50E-04	PRS-CSx
Colorectum	PRS7	rs11042689	11	10267618	C	T	4.93E-04	PRS-CSx
Colorectum	PRS7	rs11046719	12	23141333	T	C	5.04E-03	PRS-CSx
Colorectum	PRS7	rs1104748	9	34657663	T	C	1.03E-03	PRS-CSx
Colorectum	PRS7	rs11047887	12	25348672	A	C	-5.55E-04	PRS-CSx
Colorectum	PRS7	rs11047888	12	25348816	C	T	-3.87E-04	PRS-CSx
Colorectum	PRS7	rs1104880	15	91147512	G	A	-6.68E-04	PRS-CSx
Colorectum	PRS7	rs11051507	12	31795364	C	A	-5.85E-04	PRS-CSx
Colorectum	PRS7	rs11051508	12	31795383	C	T	-3.65E-04	PRS-CSx
Colorectum	PRS7	rs11053592	12	10270174	C	T	-1.42E-03	PRS-CSx
Colorectum	PRS7	rs11053597	12	10270938	T	G	-1.14E-03	PRS-CSx
Colorectum	PRS7	rs1105489	1	247710867	A	G	2.16E-03	PRS-CSx
Colorectum	PRS7	rs11054931	12	12643587	T	C	3.62E-04	PRS-CSx
Colorectum	PRS7	rs11055594	12	13857437	G	A	-8.25E-04	PRS-CSx
Colorectum	PRS7	rs11055595	12	13857554	T	C	-8.71E-04	PRS-CSx
Colorectum	PRS7	rs11055671	12	14052983	G	A	-1.63E-04	PRS-CSx
Colorectum	PRS7	rs11055687	12	14072223	C	T	-2.53E-04	PRS-CSx
Colorectum	PRS7	rs11055797	12	14294536	A	G	-5.14E-04	PRS-CSx
Colorectum	PRS7	rs11057042	12	16853443	T	C	-8.56E-04	PRS-CSx
Colorectum	PRS7	rs1105889	4	153860172	A	G	-2.28E-04	PRS-CSx
Colorectum	PRS7	rs11059094	12	122606837	C	T	2.45E-04	PRS-CSx
Colorectum	PRS7	rs11060369	12	129960197	C	A	7.45E-04	PRS-CSx
Colorectum	PRS7	rs11063069	12	4374373	G	A	-1.48E-02	PRS-CSx
Colorectum	PRS7	rs1106331	8	117617135	T	G	-2.30E-03	PRS-CSx
Colorectum	PRS7	rs11064983	12	120415088	A	G	4.30E-04	PRS-CSx
Colorectum	PRS7	rs11064996	12	120457783	A	G	1.83E-04	PRS-CSx
Colorectum	PRS7	rs11065014	12	120519124	C	T	3.16E-04	PRS-CSx
Colorectum	PRS7	rs11065287	12	110609714	C	T	1.26E-04	PRS-CSx
Colorectum	PRS7	rs11065774	12	111355326	A	G	-5.88E-03	PRS-CSx
Colorectum	PRS7	rs11066322	12	112922529	G	A	4.06E-04	PRS-CSx
Colorectum	PRS7	rs11066912	12	109931370	T	C	-3.74E-04	PRS-CSx
Colorectum	PRS7	rs11067150	12	109977418	C	A	-2.53E-04	PRS-CSx
Colorectum	PRS7	rs11067228	12	115094260	G	A	4.59E-03	PRS-CSx
Colorectum	PRS7	rs11067601	12	115883429	C	T	9.09E-04	PRS-CSx
Colorectum	PRS7	rs11067617	12	115926630	A	G	-1.92E-04	PRS-CSx
Colorectum	PRS7	rs11068110	12	110187992	A	C	6.82E-04	PRS-CSx
Colorectum	PRS7	rs11070887	15	52517737	A	C	6.08E-04	PRS-CSx
Colorectum	PRS7	rs11070888	15	52517953	T	C	8.75E-04	PRS-CSx
Colorectum	PRS7	rs11071070	15	31758161	T	C	4.01E-04	PRS-CSx
Colorectum	PRS7	rs11071075	15	31763609	G	T	3.52E-04	PRS-CSx

Colorectum	PRS7	rs11071094	15	31778654	A	G	7.07E-04	PRS-CSx
Colorectum	PRS7	rs11071351	15	58183308	A	G	4.01E-04	PRS-CSx
Colorectum	PRS7	rs11071895	15	66793480	C	T	-2.39E-04	PRS-CSx
Colorectum	PRS7	rs11072270	15	71606121	C	T	1.98E-04	PRS-CSx
Colorectum	PRS7	rs11072566	15	76293971	A	G	-1.58E-04	PRS-CSx
Colorectum	PRS7	rs11073016	15	82159980	G	T	-2.84E-04	PRS-CSx
Colorectum	PRS7	rs11073021	15	82180293	A	G	-6.92E-04	PRS-CSx
Colorectum	PRS7	rs11073184	15	36895630	G	T	5.23E-04	PRS-CSx
Colorectum	PRS7	rs11073911	15	90747977	G	A	-9.64E-04	PRS-CSx
Colorectum	PRS7	rs1107400	17	3615496	G	A	2.72E-04	PRS-CSx
Colorectum	PRS7	rs11075097	16	12671219	C	T	-2.43E-05	PRS-CSx
Colorectum	PRS7	rs11075100	16	12671579	A	G	-6.47E-05	PRS-CSx
Colorectum	PRS7	rs11075696	16	68731365	C	T	-1.08E-03	PRS-CSx
Colorectum	PRS7	rs11075721	16	69497810	T	C	4.90E-04	PRS-CSx
Colorectum	PRS7	rs11076164	16	56719404	C	T	3.80E-04	PRS-CSx
Colorectum	PRS7	rs11077107	16	6959803	G	T	-5.47E-04	PRS-CSx
Colorectum	PRS7	rs11078885	17	11105794	A	G	6.96E-04	PRS-CSx
Colorectum	PRS7	rs11078887	17	11112748	A	G	5.45E-04	PRS-CSx
Colorectum	PRS7	rs1107920	19	33690696	A	G	-9.83E-04	PRS-CSx
Colorectum	PRS7	rs1107930	15	47588129	T	C	-5.59E-04	PRS-CSx
Colorectum	PRS7	rs11079310	17	55684642	G	A	-8.30E-04	PRS-CSx
Colorectum	PRS7	rs11079317	17	55708795	A	G	-9.39E-04	PRS-CSx
Colorectum	PRS7	rs11081037	18	3353012	A	G	6.68E-04	PRS-CSx
Colorectum	PRS7	rs1108187	10	36186557	G	A	-9.10E-04	PRS-CSx
Colorectum	PRS7	rs11082334	18	41251372	T	C	-8.57E-04	PRS-CSx
Colorectum	PRS7	rs11082385	18	42040114	T	C	3.32E-04	PRS-CSx
Colorectum	PRS7	rs11083424	19	37993637	T	C	6.84E-04	PRS-CSx
Colorectum	PRS7	rs11083473	19	39179934	G	A	-1.94E-04	PRS-CSx
Colorectum	PRS7	rs11083616	19	41865643	A	G	7.74E-04	PRS-CSx
Colorectum	PRS7	rs1108405	12	96369440	T	G	-1.53E-03	PRS-CSx
Colorectum	PRS7	rs1108467	2	240475081	A	C	9.49E-04	PRS-CSx
Colorectum	PRS7	rs11084790	19	28496053	G	A	1.15E-03	PRS-CSx
Colorectum	PRS7	rs11084912	19	1859390	C	A	3.50E-04	PRS-CSx
Colorectum	PRS7	rs11084914	19	1873720	A	G	6.89E-04	PRS-CSx
Colorectum	PRS7	rs1108600	1	3241135	T	C	-4.95E-04	PRS-CSx
Colorectum	PRS7	rs11086668	20	57785057	C	T	-1.83E-03	PRS-CSx
Colorectum	PRS7	rs11088489	21	41001279	G	A	-1.36E-03	PRS-CSx
Colorectum	PRS7	rs11088588	21	18061292	A	G	-2.13E-03	PRS-CSx
Colorectum	PRS7	rs11089629	22	21958872	T	G	2.41E-04	PRS-CSx
Colorectum	PRS7	rs11089637	22	21979096	T	C	1.03E-04	PRS-CSx
Colorectum	PRS7	rs11089974	22	40543608	T	C	-6.40E-04	PRS-CSx
Colorectum	PRS7	rs11090115	22	43094877	G	A	3.37E-04	PRS-CSx
Colorectum	PRS7	rs1109020	10	132053000	T	G	-1.95E-03	PRS-CSx
Colorectum	PRS7	rs1109036	15	22933691	A	G	-1.49E-03	PRS-CSx
Colorectum	PRS7	rs1109515	17	21104627	A	G	-5.33E-04	PRS-CSx
Colorectum	PRS7	rs11096987	4	39259190	G	A	-1.06E-04	PRS-CSx
Colorectum	PRS7	rs11096990	4	39286949	T	C	-1.19E-04	PRS-CSx
Colorectum	PRS7	rs11097873	4	105883158	G	A	-6.25E-04	PRS-CSx
Colorectum	PRS7	rs11098254	4	115452456	A	G	2.02E-04	PRS-CSx
Colorectum	PRS7	rs11098375	4	118023183	C	T	-1.70E-03	PRS-CSx
Colorectum	PRS7	rs11099765	4	151432767	T	C	-3.69E-04	PRS-CSx
Colorectum	PRS7	rs11099853	4	153703306	C	T	-1.84E-04	PRS-CSx
Colorectum	PRS7	rs11099892	4	154553693	G	A	-1.68E-03	PRS-CSx
Colorectum	PRS7	rs11100654	4	167413008	C	T	8.88E-04	PRS-CSx
Colorectum	PRS7	rs11103429	9	137506578	G	A	-1.15E-03	PRS-CSx
Colorectum	PRS7	rs11106499	12	92699889	A	G	-2.98E-03	PRS-CSx

Colorectum	PRS7	rs11107212	12	94233250	A	G	-3.20E-03	PRS-CSx
Colorectum	PRS7	rs1110763	16	80157024	T	C	-2.75E-04	PRS-CSx
Colorectum	PRS7	rs11107782	12	95302658	T	C	-4.10E-04	PRS-CSx
Colorectum	PRS7	rs11107802	12	95319773	T	G	-1.95E-04	PRS-CSx
Colorectum	PRS7	rs11108193	12	96074323	T	C	3.92E-04	PRS-CSx
Colorectum	PRS7	rs11108834	12	97526310	G	A	9.04E-05	PRS-CSx
Colorectum	PRS7	rs11108835	12	97529761	T	C	2.12E-04	PRS-CSx
Colorectum	PRS7	rs11108836	12	97529985	A	G	2.49E-05	PRS-CSx
Colorectum	PRS7	rs1110956	4	153865930	A	G	-4.23E-04	PRS-CSx
Colorectum	PRS7	rs11112089	12	104919619	T	C	3.00E-03	PRS-CSx
Colorectum	PRS7	rs11114162	12	79931485	G	A	3.00E-04	PRS-CSx
Colorectum	PRS7	rs11114170	12	79954131	G	A	3.61E-04	PRS-CSx
Colorectum	PRS7	rs11114173	12	79957656	T	C	2.50E-04	PRS-CSx
Colorectum	PRS7	rs1111418	7	136633704	C	T	-8.85E-04	PRS-CSx
Colorectum	PRS7	rs11114185	12	79970339	G	A	7.10E-04	PRS-CSx
Colorectum	PRS7	rs11114218	12	80090750	C	T	2.13E-04	PRS-CSx
Colorectum	PRS7	rs11115156	12	77108186	T	G	-3.37E-04	PRS-CSx
Colorectum	PRS7	rs11115758	12	83761547	T	C	-6.12E-04	PRS-CSx
Colorectum	PRS7	rs1111583	2	98975985	C	A	-4.28E-04	PRS-CSx
Colorectum	PRS7	rs1111720	16	68698886	T	C	-8.05E-05	PRS-CSx
Colorectum	PRS7	rs1111721	16	68698461	A	G	-3.56E-05	PRS-CSx
Colorectum	PRS7	rs1111722	16	68698153	C	A	-5.86E-05	PRS-CSx
Colorectum	PRS7	rs11117450	16	86158321	A	G	2.83E-04	PRS-CSx
Colorectum	PRS7	rs11117451	16	86163221	A	G	1.73E-04	PRS-CSx
Colorectum	PRS7	rs11117853	1	217487940	C	T	1.57E-05	PRS-CSx
Colorectum	PRS7	rs11118609	1	220988490	G	A	4.42E-04	PRS-CSx
Colorectum	PRS7	rs11118610	1	220990086	C	A	8.01E-05	PRS-CSx
Colorectum	PRS7	rs11118642	1	221244372	T	C	3.16E-04	PRS-CSx
Colorectum	PRS7	rs11118647	1	221253830	A	G	1.58E-04	PRS-CSx
Colorectum	PRS7	rs11118858	1	221952958	T	G	6.93E-04	PRS-CSx
Colorectum	PRS7	rs11118882	1	222053234	T	C	-1.06E-04	PRS-CSx
Colorectum	PRS7	rs11118885	1	222075459	C	T	3.54E-04	PRS-CSx
Colorectum	PRS7	rs11118922	1	222161179	T	G	-9.97E-04	PRS-CSx
Colorectum	PRS7	rs11118924	1	222168110	T	C	-9.59E-04	PRS-CSx
Colorectum	PRS7	rs11121371	1	9392728	G	A	-2.20E-03	PRS-CSx
Colorectum	PRS7	rs11121950	1	12672149	C	T	-1.04E-03	PRS-CSx
Colorectum	PRS7	rs11121951	1	12676205	T	C	2.17E-03	PRS-CSx
Colorectum	PRS7	rs11121976	1	12833428	T	C	9.62E-05	PRS-CSx
Colorectum	PRS7	rs11122324	1	231859181	A	G	4.25E-04	PRS-CSx
Colorectum	PRS7	rs11123802	2	100487001	C	T	4.86E-04	PRS-CSx
Colorectum	PRS7	rs11125183	2	49013655	T	C	-6.97E-04	PRS-CSx
Colorectum	PRS7	rs11127142	2	28559717	C	T	9.04E-04	PRS-CSx
Colorectum	PRS7	rs11127144	2	28567705	A	G	3.68E-04	PRS-CSx
Colorectum	PRS7	rs11127145	2	28568016	G	A	4.39E-04	PRS-CSx
Colorectum	PRS7	rs11128120	3	69266821	G	A	-9.91E-04	PRS-CSx
Colorectum	PRS7	rs11128332	3	73430237	T	C	-6.59E-04	PRS-CSx
Colorectum	PRS7	rs11128357	3	73708530	C	A	-5.43E-04	PRS-CSx
Colorectum	PRS7	rs11129896	3	41295767	C	T	-4.85E-04	PRS-CSx
Colorectum	PRS7	rs11130875	3	62074171	A	G	-1.22E-03	PRS-CSx
Colorectum	PRS7	rs1113401	9	21012170	G	A	2.96E-04	PRS-CSx
Colorectum	PRS7	rs1113439	5	39907157	T	G	-8.23E-05	PRS-CSx
Colorectum	PRS7	rs1113851	11	111354161	A	G	-4.42E-04	PRS-CSx
Colorectum	PRS7	rs11139986	9	85853953	A	C	8.26E-04	PRS-CSx
Colorectum	PRS7	rs11140959	9	88020057	G	A	1.34E-03	PRS-CSx
Colorectum	PRS7	rs1114404	3	112562919	G	A	-5.14E-04	PRS-CSx
Colorectum	PRS7	rs1114463	18	69698895	G	A	-2.98E-04	PRS-CSx

Colorectum	PRS7	rs11148252	13	53009048	C	T	1.47E-04	PRS-CSx
Colorectum	PRS7	rs11149185	13	81561325	G	A	6.40E-04	PRS-CSx
Colorectum	PRS7	rs1114931	16	69076621	T	C	2.91E-04	PRS-CSx
Colorectum	PRS7	rs11149619	16	84080524	A	G	3.43E-04	PRS-CSx
Colorectum	PRS7	rs11150248	16	80202850	G	A	5.64E-04	PRS-CSx
Colorectum	PRS7	rs11151784	18	54021064	G	T	-4.94E-04	PRS-CSx
Colorectum	PRS7	rs11153618	6	116937394	T	G	-1.13E-04	PRS-CSx
Colorectum	PRS7	rs1115460	20	6398572	G	A	1.18E-03	PRS-CSx
Colorectum	PRS7	rs11154614	6	131405423	A	G	6.83E-04	PRS-CSx
Colorectum	PRS7	rs11155202	6	142092295	A	G	-1.01E-03	PRS-CSx
Colorectum	PRS7	rs1115646	6	15078289	C	A	8.28E-04	PRS-CSx
Colorectum	PRS7	rs11156808	14	34138233	G	T	-6.98E-04	PRS-CSx
Colorectum	PRS7	rs11156846	14	34975884	T	G	-5.83E-04	PRS-CSx
Colorectum	PRS7	rs11157780	14	51371130	T	C	1.10E-03	PRS-CSx
Colorectum	PRS7	rs11157781	14	51371150	T	C	9.32E-04	PRS-CSx
Colorectum	PRS7	rs11157782	14	51371524	T	C	1.23E-03	PRS-CSx
Colorectum	PRS7	rs11158135	14	57346872	G	A	-1.95E-03	PRS-CSx
Colorectum	PRS7	rs11158200	14	58649843	A	G	4.27E-04	PRS-CSx
Colorectum	PRS7	rs11158219	14	59282755	G	A	2.09E-04	PRS-CSx
Colorectum	PRS7	rs11158220	14	59282769	G	A	2.38E-04	PRS-CSx
Colorectum	PRS7	rs11158730	14	68812756	C	T	-1.93E-04	PRS-CSx
Colorectum	PRS7	rs11158907	14	71987065	G	A	5.01E-04	PRS-CSx
Colorectum	PRS7	rs11159247	14	77509872	A	G	-6.60E-04	PRS-CSx
Colorectum	PRS7	rs11160473	14	99129176	G	A	-5.85E-04	PRS-CSx
Colorectum	PRS7	rs11163895	1	84526329	T	C	1.03E-03	PRS-CSx
Colorectum	PRS7	rs11164125	2	96057129	T	C	-8.95E-05	PRS-CSx
Colorectum	PRS7	rs11165293	1	92174652	A	G	-3.21E-04	PRS-CSx
Colorectum	PRS7	rs11165294	1	92174737	T	C	-3.50E-04	PRS-CSx
Colorectum	PRS7	rs11166947	8	141065732	C	T	4.60E-04	PRS-CSx
Colorectum	PRS7	rs11167515	5	150239471	T	G	-3.72E-04	PRS-CSx
Colorectum	PRS7	rs11168074	5	148291305	T	C	-1.49E-03	PRS-CSx
Colorectum	PRS7	rs11169507	12	51015509	A	G	-1.47E-04	PRS-CSx
Colorectum	PRS7	rs11169567	12	51204938	C	T	8.06E-04	PRS-CSx
Colorectum	PRS7	rs11169571	12	51213765	C	T	9.99E-04	PRS-CSx
Colorectum	PRS7	rs11169578	12	51219701	A	G	7.62E-04	PRS-CSx
Colorectum	PRS7	rs11169761	12	51595505	A	G	-1.47E-03	PRS-CSx
Colorectum	PRS7	rs1117177	1	89787286	G	A	1.36E-03	PRS-CSx
Colorectum	PRS7	rs11172043	12	57293182	A	G	-2.81E-05	PRS-CSx
Colorectum	PRS7	rs11172047	12	57298080	C	T	4.80E-05	PRS-CSx
Colorectum	PRS7	rs11172056	12	57308975	T	C	1.04E-04	PRS-CSx
Colorectum	PRS7	rs11173671	12	40202795	T	C	-1.03E-03	PRS-CSx
Colorectum	PRS7	rs11177847	12	70180360	C	T	4.83E-04	PRS-CSx
Colorectum	PRS7	rs11178351	12	71054305	G	A	1.08E-03	PRS-CSx
Colorectum	PRS7	rs11183312	12	46454411	G	A	5.09E-04	PRS-CSx
Colorectum	PRS7	rs11184182	1	105027397	A	G	3.96E-04	PRS-CSx
Colorectum	PRS7	rs11186741	10	82493178	T	C	-1.92E-03	PRS-CSx
Colorectum	PRS7	rs11187646	10	95523820	T	C	-2.47E-04	PRS-CSx
Colorectum	PRS7	rs11190126	10	101271789	A	C	-4.35E-04	PRS-CSx
Colorectum	PRS7	rs11190134	10	101282200	G	A	-6.46E-04	PRS-CSx
Colorectum	PRS7	rs11190140	10	101291593	T	C	-6.73E-04	PRS-CSx
Colorectum	PRS7	rs11190164	10	101351704	G	A	2.11E-03	PRS-CSx
Colorectum	PRS7	rs11190169	10	101357635	A	G	3.70E-04	PRS-CSx
Colorectum	PRS7	rs11190513	10	102165238	G	A	-2.91E-04	PRS-CSx
Colorectum	PRS7	rs11190540	10	102202629	C	T	-8.98E-05	PRS-CSx
Colorectum	PRS7	rs11190541	10	102203071	G	A	-2.85E-04	PRS-CSx
Colorectum	PRS7	rs11190551	10	102204734	G	A	-2.65E-04	PRS-CSx

Colorectum	PRS7	rs11190552	10	102206607	A	G	-9.74E-05	PRS-CSx
Colorectum	PRS7	rs11190555	10	102209160	G	A	-1.31E-04	PRS-CSx
Colorectum	PRS7	rs11190568	10	102224277	T	C	-2.73E-04	PRS-CSx
Colorectum	PRS7	rs11190569	10	102227409	A	G	-1.60E-04	PRS-CSx
Colorectum	PRS7	rs11190578	10	102238953	T	C	-3.28E-04	PRS-CSx
Colorectum	PRS7	rs11191416	10	104604916	G	T	2.57E-04	PRS-CSx
Colorectum	PRS7	rs11191425	10	104625970	T	C	2.93E-04	PRS-CSx
Colorectum	PRS7	rs11191454	10	104660004	G	A	3.68E-04	PRS-CSx
Colorectum	PRS7	rs11191499	10	104764271	C	T	6.20E-04	PRS-CSx
Colorectum	PRS7	rs11191514	10	104773364	T	C	4.71E-04	PRS-CSx
Colorectum	PRS7	rs11191548	10	104846178	C	T	2.90E-04	PRS-CSx
Colorectum	PRS7	rs11191549	10	104846797	T	C	2.37E-05	PRS-CSx
Colorectum	PRS7	rs11191560	10	104869038	C	T	8.37E-04	PRS-CSx
Colorectum	PRS7	rs11191580	10	104906211	C	T	5.51E-04	PRS-CSx
Colorectum	PRS7	rs11191593	10	104939215	C	T	4.66E-04	PRS-CSx
Colorectum	PRS7	rs1119244	17	52690902	T	G	-4.78E-04	PRS-CSx
Colorectum	PRS7	rs11194951	10	111776122	C	T	-3.46E-04	PRS-CSx
Colorectum	PRS7	rs11194963	10	111806469	C	A	-4.99E-04	PRS-CSx
Colorectum	PRS7	rs11194981	10	111861054	C	T	-3.05E-04	PRS-CSx
Colorectum	PRS7	rs11197615	10	118027273	T	C	1.32E-03	PRS-CSx
Colorectum	PRS7	rs11200848	10	85856017	A	C	1.41E-03	PRS-CSx
Colorectum	PRS7	rs11202989	10	90880926	C	T	7.16E-04	PRS-CSx
Colorectum	PRS7	rs11203254	1	18326623	G	T	6.47E-04	PRS-CSx
Colorectum	PRS7	rs11203789	8	16357810	C	T	9.65E-04	PRS-CSx
Colorectum	PRS7	rs11206585	1	38568328	C	T	1.89E-04	PRS-CSx
Colorectum	PRS7	rs11207442	1	59845330	G	A	3.15E-04	PRS-CSx
Colorectum	PRS7	rs11207870	1	40789927	T	C	9.17E-04	PRS-CSx
Colorectum	PRS7	rs11208512	1	65170405	T	C	-1.13E-03	PRS-CSx
Colorectum	PRS7	rs11209002	1	67590461	T	C	2.76E-04	PRS-CSx
Colorectum	PRS7	rs11210870	1	38449910	C	T	2.68E-04	PRS-CSx
Colorectum	PRS7	rs11211576	1	48091748	A	G	3.19E-04	PRS-CSx
Colorectum	PRS7	rs11211827	11	106492521	C	T	-7.21E-04	PRS-CSx
Colorectum	PRS7	rs11211830	11	106501940	T	C	-8.44E-04	PRS-CSx
Colorectum	PRS7	rs1121252	5	39631732	G	A	3.20E-04	PRS-CSx
Colorectum	PRS7	rs11213821	11	111164651	C	T	-1.10E-03	PRS-CSx
Colorectum	PRS7	rs11216366	11	117231611	T	G	4.31E-04	PRS-CSx
Colorectum	PRS7	rs11216521	11	117614122	A	G	6.71E-04	PRS-CSx
Colorectum	PRS7	rs11217091	11	118793613	C	T	5.44E-03	PRS-CSx
Colorectum	PRS7	rs11217538	11	119718785	T	C	-2.21E-03	PRS-CSx
Colorectum	PRS7	rs11217821	11	120202099	C	T	-1.46E-04	PRS-CSx
Colorectum	PRS7	rs11217875	11	120325559	A	C	-2.60E-04	PRS-CSx
Colorectum	PRS7	rs11217878	11	120340383	A	G	-3.54E-04	PRS-CSx
Colorectum	PRS7	rs11219971	11	125079282	C	T	3.85E-04	PRS-CSx
Colorectum	PRS7	rs11220344	11	125956326	T	C	7.92E-04	PRS-CSx
Colorectum	PRS7	rs1122171	5	134509987	C	T	9.51E-04	PRS-CSx
Colorectum	PRS7	rs11222479	11	131001534	A	G	3.17E-03	PRS-CSx
Colorectum	PRS7	rs11222483	11	131004410	A	G	3.02E-03	PRS-CSx
Colorectum	PRS7	rs11222942	11	100009378	T	C	-4.19E-04	PRS-CSx
Colorectum	PRS7	rs11222971	11	132070057	C	A	2.03E-03	PRS-CSx
Colorectum	PRS7	rs1122373	11	73588240	A	G	-7.62E-04	PRS-CSx
Colorectum	PRS7	rs11225273	11	102370373	C	T	2.48E-03	PRS-CSx
Colorectum	PRS7	rs11227221	11	65319265	T	C	-2.03E-04	PRS-CSx
Colorectum	PRS7	rs11227226	11	65330510	T	C	-1.30E-03	PRS-CSx
Colorectum	PRS7	rs11227247	11	65422853	C	A	4.90E-04	PRS-CSx
Colorectum	PRS7	rs11227780	11	67136021	C	A	-7.68E-04	PRS-CSx
Colorectum	PRS7	rs11229087	11	57425699	C	T	2.50E-04	PRS-CSx

Colorectum	PRS7	rs1122961	5	82001631	T	C	-6.01E-04	PRS-CSx
Colorectum	PRS7	rs11230729	11	61278918	T	C	1.22E-03	PRS-CSx
Colorectum	PRS7	rs11232979	11	69921017	C	T	4.88E-04	PRS-CSx
Colorectum	PRS7	rs11233666	11	69935558	A	G	2.10E-03	PRS-CSx
Colorectum	PRS7	rs11234552	11	85846187	G	A	3.70E-04	PRS-CSx
Colorectum	PRS7	rs11234568	11	85876222	G	A	-5.34E-04	PRS-CSx
Colorectum	PRS7	rs11235909	11	73510645	G	A	-3.47E-04	PRS-CSx
Colorectum	PRS7	rs11235940	11	73604360	T	C	-3.35E-04	PRS-CSx
Colorectum	PRS7	rs11235948	11	73626955	T	C	-8.29E-04	PRS-CSx
Colorectum	PRS7	rs11236164	11	74294966	A	C	5.14E-04	PRS-CSx
Colorectum	PRS7	rs11236173	11	74332262	G	A	-1.06E-03	PRS-CSx
Colorectum	PRS7	rs11236187	11	74364566	C	A	8.84E-04	PRS-CSx
Colorectum	PRS7	rs11236188	11	74364730	G	T	8.57E-04	PRS-CSx
Colorectum	PRS7	rs11236208	11	74390321	T	G	-7.50E-04	PRS-CSx
Colorectum	PRS7	rs11237850	11	79179188	G	A	4.34E-04	PRS-CSx
Colorectum	PRS7	rs1124161	6	151492298	T	C	1.34E-03	PRS-CSx
Colorectum	PRS7	rs11241795	5	124540711	G	A	1.21E-03	PRS-CSx
Colorectum	PRS7	rs11242235	5	134551862	T	C	6.11E-05	PRS-CSx
Colorectum	PRS7	rs11242664	5	107584779	G	A	-4.03E-04	PRS-CSx
Colorectum	PRS7	rs11245997	11	204680	A	G	-2.78E-03	PRS-CSx
Colorectum	PRS7	rs11246002	11	211447	A	G	-1.94E-03	PRS-CSx
Colorectum	PRS7	rs11246159	11	436461	T	C	3.00E-04	PRS-CSx
Colorectum	PRS7	rs11248386	10	124843497	T	C	-8.27E-04	PRS-CSx
Colorectum	PRS7	rs11248389	10	124850994	T	C	-7.64E-04	PRS-CSx
Colorectum	PRS7	rs11248390	10	124851542	C	T	-4.43E-04	PRS-CSx
Colorectum	PRS7	rs11249	2	174086672	A	G	-9.12E-04	PRS-CSx
Colorectum	PRS7	rs11250741	10	1739576	C	T	-1.75E-03	PRS-CSx
Colorectum	PRS7	rs1125184	2	183320846	A	G	-2.81E-06	PRS-CSx
Colorectum	PRS7	rs1125326	10	91592198	G	A	-4.37E-04	PRS-CSx
Colorectum	PRS7	rs11255759	10	8626617	T	C	4.54E-04	PRS-CSx
Colorectum	PRS7	rs11255760	10	8626815	A	C	2.48E-04	PRS-CSx
Colorectum	PRS7	rs11255763	10	8630568	G	A	1.13E-04	PRS-CSx
Colorectum	PRS7	rs11255785	10	8676366	T	C	5.98E-04	PRS-CSx
Colorectum	PRS7	rs11255862	10	8753882	T	C	4.45E-04	PRS-CSx
Colorectum	PRS7	rs11255866	10	8757444	C	T	2.38E-03	PRS-CSx
Colorectum	PRS7	rs11255870	10	8763262	A	G	5.35E-04	PRS-CSx
Colorectum	PRS7	rs11255882	10	8770847	C	A	3.20E-04	PRS-CSx
Colorectum	PRS7	rs11255898	10	8793259	A	G	-9.24E-04	PRS-CSx
Colorectum	PRS7	rs11255908	10	8802912	T	G	3.42E-04	PRS-CSx
Colorectum	PRS7	rs1125642	1	233279349	G	A	3.16E-05	PRS-CSx
Colorectum	PRS7	rs11256433	10	6077845	G	T	2.25E-05	PRS-CSx
Colorectum	PRS7	rs11256448	10	6079479	G	A	1.02E-05	PRS-CSx
Colorectum	PRS7	rs11260584	1	1239339	T	G	1.39E-03	PRS-CSx
Colorectum	PRS7	rs11261300	1	223699609	A	G	2.74E-03	PRS-CSx
Colorectum	PRS7	rs1126384	13	73595944	G	A	2.72E-04	PRS-CSx
Colorectum	PRS7	rs11263956	1	37445740	C	T	2.25E-03	PRS-CSx
Colorectum	PRS7	rs11264593	1	156965948	G	A	-2.94E-04	PRS-CSx
Colorectum	PRS7	rs1127149	1	246930874	A	G	8.42E-04	PRS-CSx
Colorectum	PRS7	rs112750	11	114328285	A	G	-4.88E-05	PRS-CSx
Colorectum	PRS7	rs1129101	9	19372990	A	G	2.29E-04	PRS-CSx
Colorectum	PRS7	rs1129640	6	31506624	T	C	2.82E-05	PRS-CSx
Colorectum	PRS7	rs1130010	7	42948900	A	G	-5.73E-04	PRS-CSx
Colorectum	PRS7	rs1130861	6	160511354	A	G	5.98E-04	PRS-CSx
Colorectum	PRS7	rs1131262	3	133941320	T	C	-5.63E-04	PRS-CSx
Colorectum	PRS7	rs1132185	1	12820870	T	C	1.68E-04	PRS-CSx
Colorectum	PRS7	rs1132336	5	178140090	T	C	6.07E-04	PRS-CSx

Colorectum	PRS7	rs1133790	7	99632982	C	T	4.01E-04	PRS-CSx
Colorectum	PRS7	rs1135426	1	9787508	T	C	2.34E-03	PRS-CSx
Colorectum	PRS7	rs1140681	12	54651471	G	A	6.71E-04	PRS-CSx
Colorectum	PRS7	rs1140809	6	30611676	C	A	-1.36E-04	PRS-CSx
Colorectum	PRS7	rs1144383	13	78357782	G	T	-4.66E-04	PRS-CSx
Colorectum	PRS7	rs1144789	11	65389631	A	G	-1.05E-03	PRS-CSx
Colorectum	PRS7	rs1144841	1	226819073	A	G	-8.96E-04	PRS-CSx
Colorectum	PRS7	rs1144927	11	65287816	G	T	-1.63E-03	PRS-CSx
Colorectum	PRS7	rs1147148	2	168497830	G	A	-8.25E-04	PRS-CSx
Colorectum	PRS7	rs1147155	2	168503269	G	A	-9.64E-04	PRS-CSx
Colorectum	PRS7	rs11485595	1	38411350	T	C	-7.17E-04	PRS-CSx
Colorectum	PRS7	rs11488569	1	38338795	G	A	-1.20E-04	PRS-CSx
Colorectum	PRS7	rs1148930	11	34732225	T	C	-1.33E-04	PRS-CSx
Colorectum	PRS7	rs1150735	6	30045199	A	G	1.33E-04	PRS-CSx
Colorectum	PRS7	rs1150739	6	30031345	C	T	2.19E-04	PRS-CSx
Colorectum	PRS7	rs1150742	6	30028831	C	T	4.74E-05	PRS-CSx
Colorectum	PRS7	rs11514118	12	51312472	C	T	6.46E-04	PRS-CSx
Colorectum	PRS7	rs1151511	11	65756283	A	G	-3.02E-04	PRS-CSx
Colorectum	PRS7	rs1151514	11	65758854	G	A	-3.80E-04	PRS-CSx
Colorectum	PRS7	rs11527412	10	73717915	T	C	-7.59E-05	PRS-CSx
Colorectum	PRS7	rs1153287	21	30685611	A	G	-2.94E-04	PRS-CSx
Colorectum	PRS7	rs1153294	21	30701096	C	T	-1.11E-04	PRS-CSx
Colorectum	PRS7	rs1154818	12	68841276	A	G	2.60E-03	PRS-CSx
Colorectum	PRS7	rs11556635	17	4796286	C	T	1.53E-04	PRS-CSx
Colorectum	PRS7	rs11557154	9	34107505	C	T	-2.46E-03	PRS-CSx
Colorectum	PRS7	rs11558475	7	99654600	G	A	1.16E-04	PRS-CSx
Colorectum	PRS7	rs11558476	7	99654689	A	G	2.14E-04	PRS-CSx
Colorectum	PRS7	rs1156058	1	212869536	C	A	1.15E-03	PRS-CSx
Colorectum	PRS7	rs1156526	11	7022717	T	C	-2.63E-04	PRS-CSx
Colorectum	PRS7	rs11567762	5	35873201	G	A	3.69E-04	PRS-CSx
Colorectum	PRS7	rs1156970	6	82394320	G	T	2.11E-03	PRS-CSx
Colorectum	PRS7	rs11575812	4	123371049	G	A	2.79E-04	PRS-CSx
Colorectum	PRS7	rs1157611	22	27438196	G	A	8.32E-06	PRS-CSx
Colorectum	PRS7	rs11576196	1	164884440	A	G	2.92E-04	PRS-CSx
Colorectum	PRS7	rs11576508	1	201824390	A	G	-2.57E-04	PRS-CSx
Colorectum	PRS7	rs11578291	1	59883358	C	T	9.87E-04	PRS-CSx
Colorectum	PRS7	rs1157980	9	31191614	C	A	-1.18E-04	PRS-CSx
Colorectum	PRS7	rs11580249	1	22656863	T	G	7.71E-04	PRS-CSx
Colorectum	PRS7	rs11580545	1	58838246	G	A	3.78E-03	PRS-CSx
Colorectum	PRS7	rs11581256	1	55252514	T	C	1.17E-02	PRS-CSx
Colorectum	PRS7	rs11581606	1	221339380	T	C	6.52E-05	PRS-CSx
Colorectum	PRS7	rs11581623	1	221934718	T	C	-3.99E-04	PRS-CSx
Colorectum	PRS7	rs11583924	1	59858582	A	G	8.64E-04	PRS-CSx
Colorectum	PRS7	rs11584915	1	59860256	A	G	5.30E-04	PRS-CSx
Colorectum	PRS7	rs11585362	1	3428608	A	G	-1.98E-03	PRS-CSx
Colorectum	PRS7	rs1158586	7	136637136	A	G	-8.35E-04	PRS-CSx
Colorectum	PRS7	rs1158600	5	40143849	T	C	4.67E-04	PRS-CSx
Colorectum	PRS7	rs11588152	1	58845683	A	C	2.64E-03	PRS-CSx
Colorectum	PRS7	rs11588217	1	64727239	A	C	6.61E-04	PRS-CSx
Colorectum	PRS7	rs11588939	1	164884624	A	G	4.59E-04	PRS-CSx
Colorectum	PRS7	rs11590198	1	2551900	A	G	1.88E-04	PRS-CSx
Colorectum	PRS7	rs11591699	10	8638101	A	C	3.14E-04	PRS-CSx
Colorectum	PRS7	rs11592638	10	89399224	A	C	-1.90E-04	PRS-CSx
Colorectum	PRS7	rs11593068	10	27198815	C	T	-1.98E-03	PRS-CSx
Colorectum	PRS7	rs1159666	5	88334993	A	G	3.78E-04	PRS-CSx
Colorectum	PRS7	rs1159790	2	84685889	T	C	-8.64E-04	PRS-CSx

Colorectum	PRS7	rs11600292	11	46958698	T	C	9.58E-05	PRS-CSx
Colorectum	PRS7	rs11600668	11	47193566	G	A	2.28E-04	PRS-CSx
Colorectum	PRS7	rs11601767	11	65316382	C	T	-2.07E-04	PRS-CSx
Colorectum	PRS7	rs11603425	11	125080695	C	T	2.05E-04	PRS-CSx
Colorectum	PRS7	rs11604876	11	46768041	A	G	1.40E-04	PRS-CSx
Colorectum	PRS7	rs11604951	11	74335121	T	C	6.74E-04	PRS-CSx
Colorectum	PRS7	rs11606813	11	69018588	T	C	-5.88E-03	PRS-CSx
Colorectum	PRS7	rs11608412	12	116168751	G	T	1.55E-04	PRS-CSx
Colorectum	PRS7	rs11609105	12	115102482	C	A	1.67E-03	PRS-CSx
Colorectum	PRS7	rs11609860	12	95572027	A	G	-2.38E-04	PRS-CSx
Colorectum	PRS7	rs11610836	12	113202967	C	T	1.07E-04	PRS-CSx
Colorectum	PRS7	rs11611254	12	59317804	G	A	-7.21E-04	PRS-CSx
Colorectum	PRS7	rs11614295	12	113196733	A	G	7.19E-05	PRS-CSx
Colorectum	PRS7	rs11618218	13	51689586	T	C	-6.17E-05	PRS-CSx
Colorectum	PRS7	rs11619427	13	111060475	G	A	1.42E-04	PRS-CSx
Colorectum	PRS7	rs11620090	13	114742017	T	C	6.40E-04	PRS-CSx
Colorectum	PRS7	rs11620856	14	78241502	G	A	-4.87E-04	PRS-CSx
Colorectum	PRS7	rs11621185	14	54528067	T	C	6.12E-04	PRS-CSx
Colorectum	PRS7	rs11621593	14	78224205	T	C	-7.42E-04	PRS-CSx
Colorectum	PRS7	rs11621659	14	38047443	A	G	-6.79E-04	PRS-CSx
Colorectum	PRS7	rs11622493	14	77517045	G	A	-6.84E-04	PRS-CSx
Colorectum	PRS7	rs11623828	14	58669403	A	G	1.13E-04	PRS-CSx
Colorectum	PRS7	rs11624377	14	39058109	T	C	-1.01E-03	PRS-CSx
Colorectum	PRS7	rs11624899	14	96660336	T	C	9.46E-04	PRS-CSx
Colorectum	PRS7	rs11626845	14	39081318	T	C	-7.47E-04	PRS-CSx
Colorectum	PRS7	rs11627199	14	58853006	C	T	2.72E-04	PRS-CSx
Colorectum	PRS7	rs11627851	14	77991260	A	G	4.57E-04	PRS-CSx
Colorectum	PRS7	rs11628520	14	58669409	T	G	2.13E-04	PRS-CSx
Colorectum	PRS7	rs11631717	15	102113153	A	G	-6.61E-04	PRS-CSx
Colorectum	PRS7	rs11632070	15	71689059	C	T	1.14E-04	PRS-CSx
Colorectum	PRS7	rs11632094	15	66620971	C	T	-1.66E-04	PRS-CSx
Colorectum	PRS7	rs11632225	15	66725345	A	G	-2.45E-04	PRS-CSx
Colorectum	PRS7	rs11632680	15	71668140	G	T	8.61E-04	PRS-CSx
Colorectum	PRS7	rs11632784	15	22918281	T	C	-1.01E-03	PRS-CSx
Colorectum	PRS7	rs11632955	15	31549117	T	C	-1.49E-03	PRS-CSx
Colorectum	PRS7	rs11632964	15	67363573	T	C	-9.13E-04	PRS-CSx
Colorectum	PRS7	rs11634024	15	71647862	G	A	6.33E-04	PRS-CSx
Colorectum	PRS7	rs11634062	15	66774559	A	G	-3.46E-04	PRS-CSx
Colorectum	PRS7	rs11634896	15	47547177	T	C	-2.67E-04	PRS-CSx
Colorectum	PRS7	rs11634908	15	82138303	A	G	-2.02E-04	PRS-CSx
Colorectum	PRS7	rs11636031	15	75815758	C	T	2.92E-04	PRS-CSx
Colorectum	PRS7	rs11636199	15	75825337	G	A	6.01E-05	PRS-CSx
Colorectum	PRS7	rs11637373	15	71631026	A	G	3.74E-04	PRS-CSx
Colorectum	PRS7	rs11638490	15	79007950	T	C	1.47E-03	PRS-CSx
Colorectum	PRS7	rs11638563	15	27232072	G	A	3.89E-04	PRS-CSx
Colorectum	PRS7	rs11638674	15	77891940	T	C	-3.41E-04	PRS-CSx
Colorectum	PRS7	rs11638745	15	82176429	C	A	-8.10E-04	PRS-CSx
Colorectum	PRS7	rs11638802	15	82042226	C	T	-4.69E-04	PRS-CSx
Colorectum	PRS7	rs11638974	15	75799306	A	G	2.66E-04	PRS-CSx
Colorectum	PRS7	rs11639239	15	71647888	T	C	4.84E-04	PRS-CSx
Colorectum	PRS7	rs11639481	15	71657445	A	G	3.76E-04	PRS-CSx
Colorectum	PRS7	rs11640073	16	80019182	C	A	-6.36E-04	PRS-CSx
Colorectum	PRS7	rs11640960	16	80055968	C	T	-2.14E-04	PRS-CSx
Colorectum	PRS7	rs11643123	16	11260440	G	A	-1.86E-03	PRS-CSx
Colorectum	PRS7	rs11644916	16	359567	A	G	-2.00E-04	PRS-CSx
Colorectum	PRS7	rs11645885	16	80032104	A	C	-5.64E-04	PRS-CSx

Colorectum	PRS7	rs11646887	16	69508419	G	A	5.43E-04	PRS-CSx
Colorectum	PRS7	rs11648597	16	80054863	G	A	-5.07E-04	PRS-CSx
Colorectum	PRS7	rs11648673	16	377794	A	G	-1.11E-04	PRS-CSx
Colorectum	PRS7	rs11649061	16	65597500	G	A	-9.64E-04	PRS-CSx
Colorectum	PRS7	rs11649255	16	357140	C	T	-2.97E-04	PRS-CSx
Colorectum	PRS7	rs11651343	17	1885125	T	C	-1.05E-03	PRS-CSx
Colorectum	PRS7	rs11651883	17	835502	T	C	5.00E-03	PRS-CSx
Colorectum	PRS7	rs11651885	17	59286263	G	A	-1.46E-03	PRS-CSx
Colorectum	PRS7	rs11652148	17	46644161	G	A	2.02E-04	PRS-CSx
Colorectum	PRS7	rs11652263	17	59265017	T	C	-1.99E-04	PRS-CSx
Colorectum	PRS7	rs11652440	17	5062571	A	G	1.35E-04	PRS-CSx
Colorectum	PRS7	rs11654492	17	22008649	A	C	-1.08E-03	PRS-CSx
Colorectum	PRS7	rs1165494	1	56222154	C	T	6.53E-04	PRS-CSx
Colorectum	PRS7	rs1165495	1	56222118	A	G	4.05E-04	PRS-CSx
Colorectum	PRS7	rs11659257	18	42907457	A	G	7.21E-04	PRS-CSx
Colorectum	PRS7	rs11659625	18	9479989	G	T	4.73E-04	PRS-CSx
Colorectum	PRS7	rs11659892	18	42823267	C	A	1.91E-03	PRS-CSx
Colorectum	PRS7	rs11659898	18	9748110	A	G	-4.59E-04	PRS-CSx
Colorectum	PRS7	rs11661806	18	42885818	C	A	7.29E-04	PRS-CSx
Colorectum	PRS7	rs11663460	18	40635415	T	C	-3.65E-04	PRS-CSx
Colorectum	PRS7	rs11663505	18	74491325	G	T	3.78E-04	PRS-CSx
Colorectum	PRS7	rs11666394	19	50860608	G	A	7.74E-04	PRS-CSx
Colorectum	PRS7	rs11666933	19	41862253	A	G	4.68E-04	PRS-CSx
Colorectum	PRS7	rs11666981	19	22725568	T	C	1.01E-03	PRS-CSx
Colorectum	PRS7	rs11669089	19	14793596	A	G	-5.38E-04	PRS-CSx
Colorectum	PRS7	rs11669734	19	35794775	T	C	7.07E-04	PRS-CSx
Colorectum	PRS7	rs11670426	19	28497091	T	C	1.02E-03	PRS-CSx
Colorectum	PRS7	rs11670757	19	41913431	T	G	1.94E-04	PRS-CSx
Colorectum	PRS7	rs11671118	19	14837286	C	T	-5.98E-04	PRS-CSx
Colorectum	PRS7	rs1167125	12	43434282	C	A	8.41E-04	PRS-CSx
Colorectum	PRS7	rs11672051	19	33776241	T	C	4.19E-03	PRS-CSx
Colorectum	PRS7	rs11672077	19	38167971	T	C	-3.10E-04	PRS-CSx
Colorectum	PRS7	rs11672788	19	33752731	A	G	1.32E-03	PRS-CSx
Colorectum	PRS7	rs11672955	19	3511709	T	C	-8.05E-04	PRS-CSx
Colorectum	PRS7	rs11674671	2	199621092	T	C	3.76E-04	PRS-CSx
Colorectum	PRS7	rs11675180	2	169076787	C	T	-4.49E-04	PRS-CSx
Colorectum	PRS7	rs11675728	2	230326068	T	C	1.17E-03	PRS-CSx
Colorectum	PRS7	rs11676193	2	237750619	T	C	9.39E-04	PRS-CSx
Colorectum	PRS7	rs11676337	2	183246074	C	T	-3.16E-04	PRS-CSx
Colorectum	PRS7	rs11678046	2	72190463	T	G	3.18E-03	PRS-CSx
Colorectum	PRS7	rs11678110	2	36143814	A	G	2.18E-04	PRS-CSx
Colorectum	PRS7	rs11679657	2	183183111	T	G	-1.44E-04	PRS-CSx
Colorectum	PRS7	rs11680628	2	154130843	G	A	2.90E-04	PRS-CSx
Colorectum	PRS7	rs11680715	2	8728459	A	C	-1.04E-03	PRS-CSx
Colorectum	PRS7	rs11681222	2	228647253	G	A	6.59E-04	PRS-CSx
Colorectum	PRS7	rs11682476	2	176488774	G	A	7.87E-04	PRS-CSx
Colorectum	PRS7	rs11682835	2	144632567	A	G	-6.99E-04	PRS-CSx
Colorectum	PRS7	rs11683782	2	30531189	C	A	1.24E-03	PRS-CSx
Colorectum	PRS7	rs11684308	2	76500077	A	C	-4.06E-04	PRS-CSx
Colorectum	PRS7	rs11685313	2	131854373	C	T	-2.64E-04	PRS-CSx
Colorectum	PRS7	rs11685479	2	42694337	G	A	-2.04E-04	PRS-CSx
Colorectum	PRS7	rs11685834	2	241239911	C	T	5.08E-04	PRS-CSx
Colorectum	PRS7	rs11686538	2	225818564	G	A	-1.91E-04	PRS-CSx
Colorectum	PRS7	rs11686680	2	159778783	G	A	-6.40E-04	PRS-CSx
Colorectum	PRS7	rs11686719	2	42494838	G	A	-1.95E-04	PRS-CSx
Colorectum	PRS7	rs11688572	2	42476635	T	C	-7.15E-05	PRS-CSx

Colorectum	PRS7	rs11688673	2	241244112	T	C	5.59E-04	PRS-CSx
Colorectum	PRS7	rs11689625	2	216727951	A	G	8.46E-04	PRS-CSx
Colorectum	PRS7	rs11690862	2	71737389	G	A	-1.38E-03	PRS-CSx
Colorectum	PRS7	rs11691076	2	71589618	G	A	-7.93E-04	PRS-CSx
Colorectum	PRS7	rs11691304	2	2719353	T	C	3.34E-03	PRS-CSx
Colorectum	PRS7	rs1169300	12	121431225	A	G	3.59E-04	PRS-CSx
Colorectum	PRS7	rs11694981	2	176485683	C	T	6.56E-04	PRS-CSx
Colorectum	PRS7	rs11695862	2	8712608	C	T	-9.69E-04	PRS-CSx
Colorectum	PRS7	rs11696140	20	47240370	G	A	-3.88E-04	PRS-CSx
Colorectum	PRS7	rs11697830	20	8128055	A	G	5.69E-04	PRS-CSx
Colorectum	PRS7	rs11699576	20	47349878	C	A	-1.09E-03	PRS-CSx
Colorectum	PRS7	rs11699725	20	47246181	C	A	-4.62E-04	PRS-CSx
Colorectum	PRS7	rs11700407	21	30223119	T	C	-3.98E-04	PRS-CSx
Colorectum	PRS7	rs11700764	21	30390557	T	C	-2.50E-04	PRS-CSx
Colorectum	PRS7	rs1170102	13	42776117	A	G	2.00E-04	PRS-CSx
Colorectum	PRS7	rs11701133	21	30276147	G	A	-3.16E-04	PRS-CSx
Colorectum	PRS7	rs11701361	21	47764477	C	T	2.38E-04	PRS-CSx
Colorectum	PRS7	rs11701464	21	30285392	C	T	-9.81E-05	PRS-CSx
Colorectum	PRS7	rs1170158	13	42701941	G	T	4.35E-04	PRS-CSx
Colorectum	PRS7	rs1170183	13	42650733	C	A	1.43E-04	PRS-CSx
Colorectum	PRS7	rs1170187	13	42679835	G	A	1.30E-04	PRS-CSx
Colorectum	PRS7	rs1170188	13	42678971	A	G	2.01E-04	PRS-CSx
Colorectum	PRS7	rs1170191	13	42675493	A	G	1.66E-04	PRS-CSx
Colorectum	PRS7	rs11702393	21	40382384	A	G	1.55E-03	PRS-CSx
Colorectum	PRS7	rs11704715	22	36906173	A	G	-3.71E-04	PRS-CSx
Colorectum	PRS7	rs11705299	22	43810480	G	A	-5.13E-04	PRS-CSx
Colorectum	PRS7	rs11707060	3	64614823	T	C	2.78E-04	PRS-CSx
Colorectum	PRS7	rs11710894	3	112991959	T	C	2.98E-04	PRS-CSx
Colorectum	PRS7	rs11711014	3	195820780	A	C	1.00E-03	PRS-CSx
Colorectum	PRS7	rs11711946	3	41287018	C	T	-2.56E-04	PRS-CSx
Colorectum	PRS7	rs11712448	3	64625491	T	G	1.20E-04	PRS-CSx
Colorectum	PRS7	rs11714946	3	73678490	G	A	-4.47E-04	PRS-CSx
Colorectum	PRS7	rs11716220	3	139031115	C	T	-8.09E-04	PRS-CSx
Colorectum	PRS7	rs11716997	3	127551630	G	T	9.92E-04	PRS-CSx
Colorectum	PRS7	rs11717158	3	64619048	C	T	2.31E-04	PRS-CSx
Colorectum	PRS7	rs11717310	3	37175150	T	C	-1.99E-04	PRS-CSx
Colorectum	PRS7	rs11717554	3	64519156	T	C	1.03E-04	PRS-CSx
Colorectum	PRS7	rs11717576	3	112818765	T	C	-2.64E-04	PRS-CSx
Colorectum	PRS7	rs11717731	3	113640036	T	G	-3.70E-04	PRS-CSx
Colorectum	PRS7	rs11717833	3	112991842	G	A	4.66E-04	PRS-CSx
Colorectum	PRS7	rs11718282	3	41337338	A	C	-2.00E-04	PRS-CSx
Colorectum	PRS7	rs11718413	3	112906970	G	A	-6.94E-04	PRS-CSx
Colorectum	PRS7	rs11720064	3	37101519	T	G	-1.42E-05	PRS-CSx
Colorectum	PRS7	rs11720167	3	165486145	G	T	-4.87E-04	PRS-CSx
Colorectum	PRS7	rs11720292	3	41308449	A	C	-4.74E-04	PRS-CSx
Colorectum	PRS7	rs11722430	4	148025714	G	A	-3.43E-04	PRS-CSx
Colorectum	PRS7	rs1172292	1	183174319	G	A	-2.07E-04	PRS-CSx
Colorectum	PRS7	rs11724582	4	123391464	G	A	4.94E-04	PRS-CSx
Colorectum	PRS7	rs11728202	4	18937351	T	G	4.74E-04	PRS-CSx
Colorectum	PRS7	rs11730558	4	39229771	A	G	1.53E-04	PRS-CSx
Colorectum	PRS7	rs11731189	4	129853155	C	T	-1.21E-04	PRS-CSx
Colorectum	PRS7	rs11732437	4	86210281	A	G	7.11E-04	PRS-CSx
Colorectum	PRS7	rs11733122	4	17356226	T	C	-3.48E-04	PRS-CSx
Colorectum	PRS7	rs11734382	4	13954615	C	T	-2.02E-03	PRS-CSx
Colorectum	PRS7	rs11734412	4	105870491	T	C	9.60E-04	PRS-CSx
Colorectum	PRS7	rs11735165	4	148025578	A	G	-3.64E-04	PRS-CSx

Colorectum	PRS7	rs11735414	4	86190233	G	A	6.57E-04	PRS-CSx
Colorectum	PRS7	rs11736215	4	7762502	C	A	-6.66E-04	PRS-CSx
Colorectum	PRS7	rs11736475	4	183892751	A	G	-3.05E-04	PRS-CSx
Colorectum	PRS7	rs11737809	4	40895018	A	G	-1.97E-03	PRS-CSx
Colorectum	PRS7	rs11737840	4	110994243	T	C	7.26E-04	PRS-CSx
Colorectum	PRS7	rs11737895	4	156622727	T	G	4.01E-04	PRS-CSx
Colorectum	PRS7	rs11738550	5	79395455	C	T	1.84E-03	PRS-CSx
Colorectum	PRS7	rs11739126	5	129432552	T	C	-2.59E-04	PRS-CSx
Colorectum	PRS7	rs11739961	5	141508488	T	G	3.96E-04	PRS-CSx
Colorectum	PRS7	rs11740283	5	178136440	C	T	4.41E-04	PRS-CSx
Colorectum	PRS7	rs11740402	5	58537849	A	C	1.33E-03	PRS-CSx
Colorectum	PRS7	rs11741861	5	150277909	G	A	-6.97E-04	PRS-CSx
Colorectum	PRS7	rs11742156	5	178147833	T	C	2.53E-04	PRS-CSx
Colorectum	PRS7	rs11743082	5	143637529	A	G	-2.27E-04	PRS-CSx
Colorectum	PRS7	rs11743317	5	15660058	A	G	7.43E-04	PRS-CSx
Colorectum	PRS7	rs11743326	5	39707555	A	G	-4.21E-04	PRS-CSx
Colorectum	PRS7	rs11743841	5	39713039	T	G	-6.51E-04	PRS-CSx
Colorectum	PRS7	rs11743893	5	178125194	T	C	4.35E-04	PRS-CSx
Colorectum	PRS7	rs11744970	5	40045625	A	G	3.06E-04	PRS-CSx
Colorectum	PRS7	rs11745068	5	134475494	G	T	7.71E-04	PRS-CSx
Colorectum	PRS7	rs11745967	5	159454650	G	A	4.90E-04	PRS-CSx
Colorectum	PRS7	rs11746841	5	39707583	C	T	-5.40E-04	PRS-CSx
Colorectum	PRS7	rs11747270	5	150258867	G	A	-8.98E-04	PRS-CSx
Colorectum	PRS7	rs11747390	5	178155304	G	A	6.52E-04	PRS-CSx
Colorectum	PRS7	rs11748465	5	112614518	A	G	-1.26E-04	PRS-CSx
Colorectum	PRS7	rs11748495	5	178130000	T	C	4.71E-04	PRS-CSx
Colorectum	PRS7	rs11748524	5	112614706	T	G	-4.56E-05	PRS-CSx
Colorectum	PRS7	rs11748907	5	143651620	C	T	-3.16E-04	PRS-CSx
Colorectum	PRS7	rs11749314	5	50473298	T	C	-3.79E-04	PRS-CSx
Colorectum	PRS7	rs11749438	5	178131245	T	C	5.29E-04	PRS-CSx
Colorectum	PRS7	rs11749731	5	141500436	A	C	3.30E-04	PRS-CSx
Colorectum	PRS7	rs11749915	5	57309072	T	C	1.59E-04	PRS-CSx
Colorectum	PRS7	rs11750922	5	120853959	A	C	-3.40E-04	PRS-CSx
Colorectum	PRS7	rs11751659	6	33081632	G	A	-4.57E-04	PRS-CSx
Colorectum	PRS7	rs11751668	6	4125474	A	G	1.72E-03	PRS-CSx
Colorectum	PRS7	rs11751737	6	57160585	C	T	-8.77E-04	PRS-CSx
Colorectum	PRS7	rs11753654	6	29782621	T	C	1.42E-04	PRS-CSx
Colorectum	PRS7	rs11755011	6	24215665	A	C	-6.15E-04	PRS-CSx
Colorectum	PRS7	rs11755555	6	12183952	A	G	7.01E-04	PRS-CSx
Colorectum	PRS7	rs11756365	6	43905005	A	G	-6.94E-04	PRS-CSx
Colorectum	PRS7	rs11756925	6	74756656	C	T	3.34E-03	PRS-CSx
Colorectum	PRS7	rs11757332	6	117004240	C	T	-1.48E-04	PRS-CSx
Colorectum	PRS7	rs11757446	6	2852040	T	C	1.48E-03	PRS-CSx
Colorectum	PRS7	rs11757491	6	412236	A	G	1.91E-03	PRS-CSx
Colorectum	PRS7	rs11757973	6	130275975	A	G	5.34E-04	PRS-CSx
Colorectum	PRS7	rs11762189	7	70585797	C	T	8.02E-04	PRS-CSx
Colorectum	PRS7	rs11763946	7	46218147	G	A	-6.27E-04	PRS-CSx
Colorectum	PRS7	rs11765150	7	16653608	G	A	-4.31E-04	PRS-CSx
Colorectum	PRS7	rs11766061	7	46907524	T	G	3.38E-04	PRS-CSx
Colorectum	PRS7	rs11766093	7	46907622	A	G	2.85E-04	PRS-CSx
Colorectum	PRS7	rs11768244	7	25767091	G	A	-1.86E-04	PRS-CSx
Colorectum	PRS7	rs11769531	7	25763095	G	A	-1.58E-04	PRS-CSx
Colorectum	PRS7	rs11770579	7	33922491	G	A	-1.93E-03	PRS-CSx
Colorectum	PRS7	rs11770622	7	46197266	T	C	9.92E-05	PRS-CSx
Colorectum	PRS7	rs11771139	7	99736059	A	G	-4.14E-04	PRS-CSx
Colorectum	PRS7	rs11771331	7	99605588	G	A	-6.66E-04	PRS-CSx

Colorectum	PRS7	rs11772115	7	25770924	C	T	-5.01E-04	PRS-CSx
Colorectum	PRS7	rs1177301	2	61314020	A	G	-3.63E-04	PRS-CSx
Colorectum	PRS7	rs11776189	8	96036741	G	T	5.51E-04	PRS-CSx
Colorectum	PRS7	rs1177637	13	42692823	G	A	1.78E-04	PRS-CSx
Colorectum	PRS7	rs11776511	8	61360266	G	A	-1.92E-03	PRS-CSx
Colorectum	PRS7	rs1177981	17	70471399	T	C	6.23E-04	PRS-CSx
Colorectum	PRS7	rs11779902	8	4650751	G	T	5.55E-04	PRS-CSx
Colorectum	PRS7	rs11780644	8	131449911	G	A	1.16E-03	PRS-CSx
Colorectum	PRS7	rs11780716	8	83855731	A	G	-1.28E-04	PRS-CSx
Colorectum	PRS7	rs11780931	8	96043829	A	G	3.78E-04	PRS-CSx
Colorectum	PRS7	rs11781643	8	123429470	T	C	1.88E-04	PRS-CSx
Colorectum	PRS7	rs11782904	8	142246550	T	C	-2.27E-03	PRS-CSx
Colorectum	PRS7	rs11784473	8	83856081	C	T	-4.54E-04	PRS-CSx
Colorectum	PRS7	rs11786746	8	117884270	G	A	-5.62E-04	PRS-CSx
Colorectum	PRS7	rs11787841	9	136957308	A	G	1.91E-03	PRS-CSx
Colorectum	PRS7	rs11788920	9	101836786	G	A	3.34E-04	PRS-CSx
Colorectum	PRS7	rs11789439	9	101820718	T	G	8.96E-05	PRS-CSx
Colorectum	PRS7	rs11792012	9	20542139	C	T	4.10E-04	PRS-CSx
Colorectum	PRS7	rs1179384	6	39226854	C	T	-8.40E-04	PRS-CSx
Colorectum	PRS7	rs11793842	9	632149	A	G	-8.31E-04	PRS-CSx
Colorectum	PRS7	rs11801589	1	204807500	G	A	3.54E-04	PRS-CSx
Colorectum	PRS7	rs11802105	1	221932500	G	A	2.95E-04	PRS-CSx
Colorectum	PRS7	rs11809984	1	98654262	T	C	-4.27E-04	PRS-CSx
Colorectum	PRS7	rs11811484	1	53775436	G	A	2.12E-02	PRS-CSx
Colorectum	PRS7	rs11812522	10	118052051	T	C	4.48E-04	PRS-CSx
Colorectum	PRS7	rs11812546	10	49701384	A	G	3.42E-04	PRS-CSx
Colorectum	PRS7	rs11814083	10	71645440	C	T	-2.84E-03	PRS-CSx
Colorectum	PRS7	rs11814574	10	73996534	C	A	-6.42E-04	PRS-CSx
Colorectum	PRS7	rs11816226	10	14812114	C	T	-1.15E-03	PRS-CSx
Colorectum	PRS7	rs11816383	10	118018805	T	G	1.16E-03	PRS-CSx
Colorectum	PRS7	rs11817421	10	118121282	T	G	2.99E-04	PRS-CSx
Colorectum	PRS7	rs11817803	10	118067209	T	C	4.25E-04	PRS-CSx
Colorectum	PRS7	rs11817939	10	64588680	C	T	1.86E-04	PRS-CSx
Colorectum	PRS7	rs11818524	10	106469961	G	A	2.11E-04	PRS-CSx
Colorectum	PRS7	rs1181875	1	3681831	T	C	-9.23E-04	PRS-CSx
Colorectum	PRS7	rs11819881	11	382912	G	T	1.59E-03	PRS-CSx
Colorectum	PRS7	rs11820210	11	19493284	C	A	3.41E-04	PRS-CSx
Colorectum	PRS7	rs1182171	7	2878510	G	A	-2.63E-03	PRS-CSx
Colorectum	PRS7	rs1182179	7	2873648	G	A	-1.80E-03	PRS-CSx
Colorectum	PRS7	rs1182182	7	2873234	T	C	-1.69E-03	PRS-CSx
Colorectum	PRS7	rs11823264	11	120294004	A	G	-2.87E-04	PRS-CSx
Colorectum	PRS7	rs11828059	11	94524656	A	G	4.91E-04	PRS-CSx
Colorectum	PRS7	rs1182865	6	39229521	A	C	-2.47E-04	PRS-CSx
Colorectum	PRS7	rs1182933	12	121454622	T	C	6.04E-04	PRS-CSx
Colorectum	PRS7	rs11831226	12	109994112	C	A	-2.58E-04	PRS-CSx
Colorectum	PRS7	rs11832104	12	111619440	T	C	4.09E-05	PRS-CSx
Colorectum	PRS7	rs11833031	12	79828010	G	A	3.87E-04	PRS-CSx
Colorectum	PRS7	rs11834080	12	12617367	G	A	-4.44E-04	PRS-CSx
Colorectum	PRS7	rs11836523	12	13857884	C	T	-9.69E-04	PRS-CSx
Colorectum	PRS7	rs11837865	12	77340041	G	A	-7.59E-04	PRS-CSx
Colorectum	PRS7	rs11838546	13	78531614	A	G	-1.95E-03	PRS-CSx
Colorectum	PRS7	rs11840092	13	58114748	C	T	7.80E-04	PRS-CSx
Colorectum	PRS7	rs11840813	13	24718148	A	C	-2.49E-03	PRS-CSx
Colorectum	PRS7	rs11841381	13	73583279	A	G	2.98E-04	PRS-CSx
Colorectum	PRS7	rs11844358	14	38080088	G	A	-4.55E-04	PRS-CSx
Colorectum	PRS7	rs11845307	14	51395384	G	T	6.91E-05	PRS-CSx

Colorectum	PRS7	rs11845885	14	54765493	C	T	4.31E-04	PRS-CSx
Colorectum	PRS7	rs11846747	14	26763403	T	C	1.24E-03	PRS-CSx
Colorectum	PRS7	rs1184924	13	42704400	A	G	2.63E-04	PRS-CSx
Colorectum	PRS7	rs11851015	14	57669533	G	A	7.69E-04	PRS-CSx
Colorectum	PRS7	rs11851031	14	52092371	G	A	-7.87E-04	PRS-CSx
Colorectum	PRS7	rs11851552	14	58706750	T	C	3.25E-05	PRS-CSx
Colorectum	PRS7	rs11853142	15	79579510	G	T	-5.96E-04	PRS-CSx
Colorectum	PRS7	rs11854805	15	48675874	C	T	2.36E-03	PRS-CSx
Colorectum	PRS7	rs11854854	15	79582965	T	C	-1.08E-03	PRS-CSx
Colorectum	PRS7	rs11854943	15	48861287	C	A	1.03E-03	PRS-CSx
Colorectum	PRS7	rs11856606	15	75925592	A	G	3.90E-04	PRS-CSx
Colorectum	PRS7	rs11858146	15	47528080	C	T	-3.46E-04	PRS-CSx
Colorectum	PRS7	rs11858490	15	102007443	T	G	4.97E-04	PRS-CSx
Colorectum	PRS7	rs11858491	15	102007456	T	C	7.51E-04	PRS-CSx
Colorectum	PRS7	rs11860820	16	86186412	T	C	1.02E-03	PRS-CSx
Colorectum	PRS7	rs11864054	16	30846134	A	G	2.11E-04	PRS-CSx
Colorectum	PRS7	rs11864982	16	84457263	C	T	7.34E-05	PRS-CSx
Colorectum	PRS7	rs11865026	16	68774283	C	T	-9.35E-04	PRS-CSx
Colorectum	PRS7	rs11868258	17	21995777	C	T	-8.53E-04	PRS-CSx
Colorectum	PRS7	rs11868441	17	59239221	A	G	-2.70E-04	PRS-CSx
Colorectum	PRS7	rs11870439	17	833790	T	G	1.26E-03	PRS-CSx
Colorectum	PRS7	rs11872146	18	3908973	T	G	-6.14E-04	PRS-CSx
Colorectum	PRS7	rs11872226	18	9835419	A	C	-5.33E-04	PRS-CSx
Colorectum	PRS7	rs1187411	16	50034680	G	A	-4.80E-04	PRS-CSx
Colorectum	PRS7	rs11875595	18	42308972	T	C	5.94E-04	PRS-CSx
Colorectum	PRS7	rs11875687	18	12843137	C	T	-1.61E-04	PRS-CSx
Colorectum	PRS7	rs11876996	18	72231480	T	C	-8.69E-04	PRS-CSx
Colorectum	PRS7	rs11878563	19	6670910	A	G	-5.95E-04	PRS-CSx
Colorectum	PRS7	rs11878644	19	45876967	C	T	-4.94E-04	PRS-CSx
Colorectum	PRS7	rs11879872	19	39395331	C	T	5.87E-04	PRS-CSx
Colorectum	PRS7	rs11880539	19	41918949	A	C	1.61E-04	PRS-CSx
Colorectum	PRS7	rs11883623	2	240217116	C	T	-8.86E-04	PRS-CSx
Colorectum	PRS7	rs11884886	2	183357799	G	A	2.69E-04	PRS-CSx
Colorectum	PRS7	rs11886084	2	18206413	G	A	2.93E-04	PRS-CSx
Colorectum	PRS7	rs11887008	2	174091088	T	C	-6.48E-04	PRS-CSx
Colorectum	PRS7	rs11892526	2	191255883	C	T	-1.03E-03	PRS-CSx
Colorectum	PRS7	rs11892712	2	157481260	T	C	4.39E-03	PRS-CSx
Colorectum	PRS7	rs11892761	2	131855231	T	C	-4.93E-04	PRS-CSx
Colorectum	PRS7	rs11893063	2	199601925	A	G	4.56E-04	PRS-CSx
Colorectum	PRS7	rs11893353	2	131823623	C	T	-3.48E-04	PRS-CSx
Colorectum	PRS7	rs11895615	2	26113120	C	T	-4.48E-04	PRS-CSx
Colorectum	PRS7	rs11895872	2	131895230	C	T	-4.36E-04	PRS-CSx
Colorectum	PRS7	rs11897055	2	43109583	G	A	-1.06E-03	PRS-CSx
Colorectum	PRS7	rs11897501	2	240678769	C	T	2.27E-03	PRS-CSx
Colorectum	PRS7	rs11897939	2	154144844	T	C	3.97E-04	PRS-CSx
Colorectum	PRS7	rs11897976	2	96007303	T	C	-2.09E-04	PRS-CSx
Colorectum	PRS7	rs11898330	2	71688588	A	G	-4.58E-04	PRS-CSx
Colorectum	PRS7	rs11899646	2	155330994	A	G	6.11E-04	PRS-CSx
Colorectum	PRS7	rs11900545	2	131833758	C	T	-1.43E-04	PRS-CSx
Colorectum	PRS7	rs11901496	2	225320132	C	T	2.18E-04	PRS-CSx
Colorectum	PRS7	rs11901794	2	131865064	G	A	-7.44E-04	PRS-CSx
Colorectum	PRS7	rs11902329	2	28592116	A	G	5.85E-04	PRS-CSx
Colorectum	PRS7	rs11906291	20	49714321	A	G	-1.81E-03	PRS-CSx
Colorectum	PRS7	rs11907586	20	47374058	A	G	-6.54E-04	PRS-CSx
Colorectum	PRS7	rs1190982	14	58815839	C	T	2.21E-04	PRS-CSx
Colorectum	PRS7	rs11910707	21	48042513	T	G	2.67E-04	PRS-CSx

Colorectum	PRS7	rs11916925	3	182429500	T	C	2.13E-03	PRS-CSx
Colorectum	PRS7	rs11918596	3	134017219	G	A	2.35E-05	PRS-CSx
Colorectum	PRS7	rs11918943	3	193233926	T	C	-3.59E-04	PRS-CSx
Colorectum	PRS7	rs11919134	3	113007525	A	G	2.32E-04	PRS-CSx
Colorectum	PRS7	rs11919979	3	152331312	G	A	2.28E-04	PRS-CSx
Colorectum	PRS7	rs11920873	3	975836	C	T	-1.16E-03	PRS-CSx
Colorectum	PRS7	rs11923297	3	152327018	T	C	3.82E-04	PRS-CSx
Colorectum	PRS7	rs11923426	3	122640406	C	A	-2.88E-04	PRS-CSx
Colorectum	PRS7	rs11923603	3	112875949	G	A	-3.98E-04	PRS-CSx
Colorectum	PRS7	rs11923848	3	64602886	T	C	5.79E-04	PRS-CSx
Colorectum	PRS7	rs11925003	3	66466444	A	G	7.04E-04	PRS-CSx
Colorectum	PRS7	rs11925333	3	157386185	G	A	4.58E-04	PRS-CSx
Colorectum	PRS7	rs11925887	3	8350853	A	G	5.43E-04	PRS-CSx
Colorectum	PRS7	rs11926768	3	36968347	C	T	-1.39E-04	PRS-CSx
Colorectum	PRS7	rs11927855	3	18885181	T	C	5.77E-04	PRS-CSx
Colorectum	PRS7	rs11931103	4	105779464	T	G	-4.99E-04	PRS-CSx
Colorectum	PRS7	rs11931151	4	95622305	G	T	1.24E-03	PRS-CSx
Colorectum	PRS7	rs11931161	4	151444619	T	G	-2.44E-04	PRS-CSx
Colorectum	PRS7	rs11932137	4	101544176	T	C	-2.31E-03	PRS-CSx
Colorectum	PRS7	rs11932351	4	39231457	C	T	2.21E-04	PRS-CSx
Colorectum	PRS7	rs11933465	4	22476571	A	G	-3.45E-04	PRS-CSx
Colorectum	PRS7	rs11938628	4	115458168	T	C	7.88E-05	PRS-CSx
Colorectum	PRS7	rs11939702	4	146487324	C	T	8.61E-05	PRS-CSx
Colorectum	PRS7	rs11939979	4	146488327	C	A	7.23E-05	PRS-CSx
Colorectum	PRS7	rs11944586	4	21556769	A	G	3.22E-04	PRS-CSx
Colorectum	PRS7	rs11944685	4	146414267	G	A	6.71E-04	PRS-CSx
Colorectum	PRS7	rs11946839	4	151382330	T	G	-2.27E-04	PRS-CSx
Colorectum	PRS7	rs11948805	5	75895138	T	C	-4.76E-05	PRS-CSx
Colorectum	PRS7	rs11949868	5	110455899	C	T	9.80E-04	PRS-CSx
Colorectum	PRS7	rs11950121	5	53178957	C	A	4.94E-04	PRS-CSx
Colorectum	PRS7	rs11951528	5	161217476	A	G	9.75E-04	PRS-CSx
Colorectum	PRS7	rs11953143	5	133906527	T	C	8.26E-04	PRS-CSx
Colorectum	PRS7	rs11953880	5	100756024	G	A	1.10E-03	PRS-CSx
Colorectum	PRS7	rs11954280	5	134405982	C	T	6.09E-05	PRS-CSx
Colorectum	PRS7	rs11954404	5	120846718	A	C	-4.62E-04	PRS-CSx
Colorectum	PRS7	rs11957134	5	150230950	A	G	-4.40E-04	PRS-CSx
Colorectum	PRS7	rs11957173	5	40063372	A	G	-1.45E-04	PRS-CSx
Colorectum	PRS7	rs11957690	5	74327436	A	G	-1.77E-04	PRS-CSx
Colorectum	PRS7	rs11958842	5	125963051	C	T	-5.59E-04	PRS-CSx
Colorectum	PRS7	rs11960674	5	8614694	G	A	4.31E-04	PRS-CSx
Colorectum	PRS7	rs11962649	6	131419022	C	T	-2.37E-04	PRS-CSx
Colorectum	PRS7	rs11965538	6	28239915	A	G	5.15E-05	PRS-CSx
Colorectum	PRS7	rs11967989	6	11274670	C	T	6.13E-04	PRS-CSx
Colorectum	PRS7	rs11969445	6	36625382	T	C	-4.22E-04	PRS-CSx
Colorectum	PRS7	rs11969461	6	131339257	T	C	1.01E-03	PRS-CSx
Colorectum	PRS7	rs11972082	7	88021474	G	A	4.27E-04	PRS-CSx
Colorectum	PRS7	rs11972096	7	77433484	C	A	-3.13E-04	PRS-CSx
Colorectum	PRS7	rs11972949	7	15476892	A	C	2.77E-05	PRS-CSx
Colorectum	PRS7	rs11981109	7	46297747	A	G	-2.78E-03	PRS-CSx
Colorectum	PRS7	rs11982860	7	67501241	A	G	-8.42E-04	PRS-CSx
Colorectum	PRS7	rs11983139	7	135483229	A	G	2.52E-03	PRS-CSx
Colorectum	PRS7	rs11985829	8	128409232	T	C	1.65E-03	PRS-CSx
Colorectum	PRS7	rs11987213	8	118442583	A	G	2.12E-04	PRS-CSx
Colorectum	PRS7	rs11987482	8	22261629	G	A	1.11E-03	PRS-CSx
Colorectum	PRS7	rs11988025	8	118442981	A	G	1.68E-04	PRS-CSx
Colorectum	PRS7	rs11993111	8	117846282	G	A	3.38E-04	PRS-CSx

Colorectum	PRS7	rs11993769	8	117843213	T	C	1.63E-04	PRS-CSx
Colorectum	PRS7	rs12001981	9	101820545	A	G	-1.84E-04	PRS-CSx
Colorectum	PRS7	rs12019079	17	70066875	C	T	-6.43E-04	PRS-CSx
Colorectum	PRS7	rs12019516	13	114740605	G	A	4.03E-04	PRS-CSx
Colorectum	PRS7	rs12021686	1	104990198	T	C	-1.14E-03	PRS-CSx
Colorectum	PRS7	rs12022676	1	222146085	A	G	6.86E-03	PRS-CSx
Colorectum	PRS7	rs12022755	1	224387749	T	C	-6.30E-04	PRS-CSx
Colorectum	PRS7	rs12022902	1	105009637	A	G	-4.31E-04	PRS-CSx
Colorectum	PRS7	rs12023545	1	105013220	G	A	-2.66E-04	PRS-CSx
Colorectum	PRS7	rs12030948	1	67701765	G	T	3.70E-04	PRS-CSx
Colorectum	PRS7	rs12034436	1	183114797	A	G	2.53E-04	PRS-CSx
Colorectum	PRS7	rs12035017	1	76685705	C	T	4.36E-04	PRS-CSx
Colorectum	PRS7	rs12036108	1	105184308	A	G	-2.88E-04	PRS-CSx
Colorectum	PRS7	rs12036536	1	222152402	G	A	4.46E-04	PRS-CSx
Colorectum	PRS7	rs12037664	1	242798788	A	G	-1.04E-03	PRS-CSx
Colorectum	PRS7	rs12038198	1	151497202	A	G	-1.66E-03	PRS-CSx
Colorectum	PRS7	rs12038343	1	104988625	C	T	-7.00E-04	PRS-CSx
Colorectum	PRS7	rs12039248	1	88899860	A	C	-7.24E-04	PRS-CSx
Colorectum	PRS7	rs12039865	1	55092069	C	T	1.38E-04	PRS-CSx
Colorectum	PRS7	rs12039964	1	110348040	C	T	-2.09E-04	PRS-CSx
Colorectum	PRS7	rs1204042	16	352736	A	G	-8.67E-04	PRS-CSx
Colorectum	PRS7	rs12040514	1	104991650	G	A	-4.83E-04	PRS-CSx
Colorectum	PRS7	rs12041702	1	104989741	T	G	-9.19E-04	PRS-CSx
Colorectum	PRS7	rs12041707	1	104989780	A	G	-6.88E-04	PRS-CSx
Colorectum	PRS7	rs12042136	1	105063644	G	A	-1.18E-04	PRS-CSx
Colorectum	PRS7	rs12042284	1	159132649	C	A	-2.60E-03	PRS-CSx
Colorectum	PRS7	rs12042611	1	200161325	C	T	6.39E-04	PRS-CSx
Colorectum	PRS7	rs12042703	1	195018786	T	C	-6.51E-04	PRS-CSx
Colorectum	PRS7	rs12044531	1	38453041	A	G	-4.58E-04	PRS-CSx
Colorectum	PRS7	rs12044615	1	88910204	T	C	-5.08E-04	PRS-CSx
Colorectum	PRS7	rs12046182	1	220974174	T	C	-1.44E-03	PRS-CSx
Colorectum	PRS7	rs1204631	20	22400907	T	G	-1.36E-04	PRS-CSx
Colorectum	PRS7	rs12046821	1	88912692	T	C	-6.00E-04	PRS-CSx
Colorectum	PRS7	rs12048995	1	246026850	G	T	-6.13E-04	PRS-CSx
Colorectum	PRS7	rs1205233	14	71132759	G	A	-8.40E-04	PRS-CSx
Colorectum	PRS7	rs12052567	2	18162538	A	C	2.15E-03	PRS-CSx
Colorectum	PRS7	rs1205340	20	32923871	G	A	-1.46E-04	PRS-CSx
Colorectum	PRS7	rs1205345	20	32914699	T	C	-1.43E-04	PRS-CSx
Colorectum	PRS7	rs12059918	1	117430453	T	C	-1.61E-03	PRS-CSx
Colorectum	PRS7	rs12061219	1	183101471	G	A	2.22E-04	PRS-CSx
Colorectum	PRS7	rs12067894	1	178130163	T	C	1.16E-04	PRS-CSx
Colorectum	PRS7	rs12069137	1	76729651	T	C	1.76E-03	PRS-CSx
Colorectum	PRS7	rs12073837	1	221010205	T	C	2.88E-04	PRS-CSx
Colorectum	PRS7	rs12073936	1	183061890	G	T	-1.00E-03	PRS-CSx
Colorectum	PRS7	rs12074264	1	194635480	G	A	-3.52E-04	PRS-CSx
Colorectum	PRS7	rs12076549	1	92201699	C	A	4.00E-04	PRS-CSx
Colorectum	PRS7	rs12077886	1	204413986	C	T	-2.30E-04	PRS-CSx
Colorectum	PRS7	rs12078217	1	247709215	A	G	-8.97E-04	PRS-CSx
Colorectum	PRS7	rs12078447	1	51499525	C	A	1.81E-04	PRS-CSx
Colorectum	PRS7	rs12078646	1	54606522	C	T	1.95E-03	PRS-CSx
Colorectum	PRS7	rs12080256	1	2251357	A	C	-5.70E-04	PRS-CSx
Colorectum	PRS7	rs12080823	1	239259263	A	G	-4.79E-04	PRS-CSx
Colorectum	PRS7	rs12081312	1	55370174	A	G	-2.39E-05	PRS-CSx
Colorectum	PRS7	rs12082516	1	2251160	C	T	-1.17E-03	PRS-CSx
Colorectum	PRS7	rs12087292	1	35742791	A	C	-1.56E-03	PRS-CSx
Colorectum	PRS7	rs1208837	7	88894308	C	T	-1.10E-04	PRS-CSx

Colorectum	PRS7	rs12088480	1	215246836	T	C	-5.74E-04	PRS-CSx
Colorectum	PRS7	rs1208876	7	88911638	G	A	-1.71E-04	PRS-CSx
Colorectum	PRS7	rs12089459	1	194703490	C	T	-8.46E-04	PRS-CSx
Colorectum	PRS7	rs12089560	1	1356550	G	A	4.01E-04	PRS-CSx
Colorectum	PRS7	rs12089727	1	110461748	T	C	1.13E-03	PRS-CSx
Colorectum	PRS7	rs12091137	1	183075732	A	G	3.29E-04	PRS-CSx
Colorectum	PRS7	rs12091281	1	5314737	T	C	-2.27E-03	PRS-CSx
Colorectum	PRS7	rs12095664	1	183101686	G	A	4.21E-04	PRS-CSx
Colorectum	PRS7	rs12096061	1	117440063	A	G	-8.18E-04	PRS-CSx
Colorectum	PRS7	rs12097041	1	179401503	A	G	8.45E-05	PRS-CSx
Colorectum	PRS7	rs12097906	1	194641175	T	C	-1.13E-03	PRS-CSx
Colorectum	PRS7	rs12098070	1	182939544	A	C	9.54E-04	PRS-CSx
Colorectum	PRS7	rs12098424	10	73953261	A	C	-6.05E-04	PRS-CSx
Colorectum	PRS7	rs1209954	21	40181766	A	G	-2.75E-04	PRS-CSx
Colorectum	PRS7	rs12100737	14	71471100	G	A	-2.59E-04	PRS-CSx
Colorectum	PRS7	rs12105526	2	30029875	T	C	-2.62E-03	PRS-CSx
Colorectum	PRS7	rs12105749	2	100500674	C	T	3.62E-04	PRS-CSx
Colorectum	PRS7	rs12106159	20	47337840	A	G	-5.89E-04	PRS-CSx
Colorectum	PRS7	rs12107664	3	32345009	A	G	7.63E-04	PRS-CSx
Colorectum	PRS7	rs12108602	4	115640569	G	A	2.44E-04	PRS-CSx
Colorectum	PRS7	rs12111351	6	20724558	G	T	-1.22E-04	PRS-CSx
Colorectum	PRS7	rs12114331	8	227923	T	C	-2.02E-03	PRS-CSx
Colorectum	PRS7	rs12115297	9	126685609	T	C	2.58E-03	PRS-CSx
Colorectum	PRS7	rs12118726	1	117592149	C	T	-1.44E-03	PRS-CSx
Colorectum	PRS7	rs12121509	1	117582373	A	G	-1.08E-03	PRS-CSx
Colorectum	PRS7	rs12122418	1	213832183	C	T	-6.43E-04	PRS-CSx
Colorectum	PRS7	rs12123688	1	117584322	A	G	-1.07E-03	PRS-CSx
Colorectum	PRS7	rs12124078	1	15869899	A	G	-4.86E-04	PRS-CSx
Colorectum	PRS7	rs12125058	1	221277508	T	C	1.46E-04	PRS-CSx
Colorectum	PRS7	rs12125573	1	46734792	C	T	4.15E-04	PRS-CSx
Colorectum	PRS7	rs12126434	1	182988414	G	A	3.52E-04	PRS-CSx
Colorectum	PRS7	rs1212694	14	58673586	G	A	2.69E-04	PRS-CSx
Colorectum	PRS7	rs12128312	1	22658301	A	G	1.17E-03	PRS-CSx
Colorectum	PRS7	rs12130076	1	240918105	G	A	1.91E-03	PRS-CSx
Colorectum	PRS7	rs12130711	1	247607642	T	C	4.25E-04	PRS-CSx
Colorectum	PRS7	rs12131475	1	179436288	G	A	2.82E-04	PRS-CSx
Colorectum	PRS7	rs12132479	1	11836450	G	A	-1.28E-03	PRS-CSx
Colorectum	PRS7	rs12135243	1	101092955	A	G	5.14E-05	PRS-CSx
Colorectum	PRS7	rs12136088	1	156941412	G	T	-3.95E-04	PRS-CSx
Colorectum	PRS7	rs12136349	1	215251227	A	G	-7.54E-04	PRS-CSx
Colorectum	PRS7	rs12138053	1	182979560	C	T	3.29E-04	PRS-CSx
Colorectum	PRS7	rs12138115	1	38452581	A	G	-4.23E-04	PRS-CSx
Colorectum	PRS7	rs12138805	1	104825468	G	A	2.59E-04	PRS-CSx
Colorectum	PRS7	rs12144639	1	213817311	A	G	1.04E-03	PRS-CSx
Colorectum	PRS7	rs12145497	1	194996485	G	A	-6.32E-04	PRS-CSx
Colorectum	PRS7	rs12145528	1	104813525	C	T	1.82E-04	PRS-CSx
Colorectum	PRS7	rs12145674	1	221000273	G	A	2.63E-04	PRS-CSx
Colorectum	PRS7	rs1214598	1	167426424	A	G	1.81E-04	PRS-CSx
Colorectum	PRS7	rs12147072	14	33910447	C	A	5.58E-04	PRS-CSx
Colorectum	PRS7	rs12148149	15	61206551	C	T	-8.32E-04	PRS-CSx
Colorectum	PRS7	rs12151388	2	177144029	G	A	6.61E-04	PRS-CSx
Colorectum	PRS7	rs12153912	6	27179829	A	G	-5.67E-04	PRS-CSx
Colorectum	PRS7	rs12154976	7	120410201	T	C	-4.55E-04	PRS-CSx
Colorectum	PRS7	rs12155015	7	136646097	C	T	-5.56E-04	PRS-CSx
Colorectum	PRS7	rs12158299	22	21979584	C	T	2.52E-04	PRS-CSx
Colorectum	PRS7	rs12164256	9	34023430	G	A	-3.55E-04	PRS-CSx

Colorectum	PRS7	rs1216493	11	100375968	A	G	8.73E-04	PRS-CSx
Colorectum	PRS7	rs12172839	17	69548753	A	G	2.42E-04	PRS-CSx
Colorectum	PRS7	rs12177	12	113335425	A	G	-1.83E-04	PRS-CSx
Colorectum	PRS7	rs12180820	6	28316478	T	C	3.70E-04	PRS-CSx
Colorectum	PRS7	rs12185951	3	5975580	C	A	-6.90E-04	PRS-CSx
Colorectum	PRS7	rs12185973	3	133995154	A	G	-4.90E-04	PRS-CSx
Colorectum	PRS7	rs12188069	5	112575924	G	A	-2.28E-04	PRS-CSx
Colorectum	PRS7	rs12189066	5	129293427	C	A	-2.04E-04	PRS-CSx
Colorectum	PRS7	rs12189242	5	34627666	A	C	-1.89E-04	PRS-CSx
Colorectum	PRS7	rs12189595	6	12174911	G	A	8.09E-04	PRS-CSx
Colorectum	PRS7	rs12192252	6	12179747	T	C	9.20E-04	PRS-CSx
Colorectum	PRS7	rs12192320	6	27090305	A	G	-3.60E-04	PRS-CSx
Colorectum	PRS7	rs12192502	6	26995720	A	C	-2.46E-04	PRS-CSx
Colorectum	PRS7	rs12193012	6	142085759	A	G	-7.26E-04	PRS-CSx
Colorectum	PRS7	rs12193110	6	29937104	T	C	-3.34E-04	PRS-CSx
Colorectum	PRS7	rs12193820	6	27113811	G	A	-2.81E-04	PRS-CSx
Colorectum	PRS7	rs12194621	6	18872434	G	A	3.46E-03	PRS-CSx
Colorectum	PRS7	rs12196541	6	24140690	C	T	1.32E-03	PRS-CSx
Colorectum	PRS7	rs12198248	6	88482778	T	C	3.91E-04	PRS-CSx
Colorectum	PRS7	rs12200985	6	27004892	T	G	-3.62E-04	PRS-CSx
Colorectum	PRS7	rs12201774	6	27058312	G	A	-3.55E-04	PRS-CSx
Colorectum	PRS7	rs12202381	6	24139942	G	A	1.44E-03	PRS-CSx
Colorectum	PRS7	rs1220789	9	132257783	T	C	9.73E-04	PRS-CSx
Colorectum	PRS7	rs12209253	6	56226033	A	G	-5.60E-04	PRS-CSx
Colorectum	PRS7	rs12209429	6	27054867	A	G	-3.36E-04	PRS-CSx
Colorectum	PRS7	rs12209456	6	27085225	T	C	-4.24E-04	PRS-CSx
Colorectum	PRS7	rs12209800	6	27189517	C	T	-4.08E-04	PRS-CSx
Colorectum	PRS7	rs1221201	2	82008849	G	T	2.29E-04	PRS-CSx
Colorectum	PRS7	rs12214848	6	27063348	T	G	-3.57E-04	PRS-CSx
Colorectum	PRS7	rs12214930	6	27093715	T	C	-4.23E-04	PRS-CSx
Colorectum	PRS7	rs12216307	6	134064162	G	T	4.68E-04	PRS-CSx
Colorectum	PRS7	rs12219789	10	102213778	G	A	-1.53E-04	PRS-CSx
Colorectum	PRS7	rs12220375	10	104901491	C	T	4.06E-04	PRS-CSx
Colorectum	PRS7	rs12221064	10	104677126	T	C	1.68E-04	PRS-CSx
Colorectum	PRS7	rs12230772	12	79962814	G	A	5.65E-04	PRS-CSx
Colorectum	PRS7	rs12231341	12	72409436	T	C	2.79E-03	PRS-CSx
Colorectum	PRS7	rs12233479	3	112567789	A	G	1.17E-03	PRS-CSx
Colorectum	PRS7	rs12233819	4	179499759	T	C	8.20E-04	PRS-CSx
Colorectum	PRS7	rs12236688	9	101622543	C	T	8.31E-04	PRS-CSx
Colorectum	PRS7	rs12241008	10	114280702	C	T	3.37E-03	PRS-CSx
Colorectum	PRS7	rs12241671	10	87489415	T	C	1.36E-03	PRS-CSx
Colorectum	PRS7	rs12243558	10	114940502	A	G	-7.90E-04	PRS-CSx
Colorectum	PRS7	rs12244405	10	52643179	T	C	3.11E-03	PRS-CSx
Colorectum	PRS7	rs12245785	10	1738862	C	T	-1.97E-03	PRS-CSx
Colorectum	PRS7	rs12246635	10	114288619	C	T	2.53E-03	PRS-CSx
Colorectum	PRS7	rs12248966	10	8831436	A	C	3.35E-04	PRS-CSx
Colorectum	PRS7	rs12250379	10	8762195	C	T	7.05E-04	PRS-CSx
Colorectum	PRS7	rs12254971	10	114324710	G	A	2.55E-03	PRS-CSx
Colorectum	PRS7	rs12255141	10	114294892	G	A	2.78E-03	PRS-CSx
Colorectum	PRS7	rs12255557	10	87436348	T	C	5.62E-04	PRS-CSx
Colorectum	PRS7	rs12257464	10	73940491	G	A	-3.81E-04	PRS-CSx
Colorectum	PRS7	rs12259226	10	8766942	C	T	9.46E-04	PRS-CSx
Colorectum	PRS7	rs12261966	10	17143000	T	C	-1.11E-03	PRS-CSx
Colorectum	PRS7	rs12264744	10	60052999	T	C	-7.52E-04	PRS-CSx
Colorectum	PRS7	rs12266147	10	74002564	A	G	-6.00E-04	PRS-CSx
Colorectum	PRS7	rs12266418	10	114318004	A	G	1.62E-03	PRS-CSx

Colorectum	PRS7	rs12268910	10	111878510	G	T	-3.29E-04	PRS-CSx
Colorectum	PRS7	rs12272771	11	5690741	C	T	1.09E-03	PRS-CSx
Colorectum	PRS7	rs12273967	11	83346901	A	G	4.14E-04	PRS-CSx
Colorectum	PRS7	rs12274061	11	15023977	T	C	2.04E-04	PRS-CSx
Colorectum	PRS7	rs12274674	11	15099695	T	C	1.88E-04	PRS-CSx
Colorectum	PRS7	rs12274979	11	125124001	G	A	6.11E-04	PRS-CSx
Colorectum	PRS7	rs12276636	11	23715537	T	C	-3.46E-03	PRS-CSx
Colorectum	PRS7	rs1227763	10	71593257	C	A	1.15E-03	PRS-CSx
Colorectum	PRS7	rs12282262	11	74393938	T	C	-1.04E-03	PRS-CSx
Colorectum	PRS7	rs12284449	11	120148845	G	A	-3.31E-04	PRS-CSx
Colorectum	PRS7	rs12284670	11	73544202	A	C	-5.29E-04	PRS-CSx
Colorectum	PRS7	rs12288326	11	125123973	A	G	7.51E-04	PRS-CSx
Colorectum	PRS7	rs12288391	11	125124082	A	G	9.41E-04	PRS-CSx
Colorectum	PRS7	rs12291412	11	111213349	A	G	-1.13E-03	PRS-CSx
Colorectum	PRS7	rs12294934	11	73581908	G	A	-3.07E-04	PRS-CSx
Colorectum	PRS7	rs12295206	11	73600351	C	T	-4.00E-04	PRS-CSx
Colorectum	PRS7	rs12295727	11	125169364	C	T	3.55E-04	PRS-CSx
Colorectum	PRS7	rs12296076	11	111166504	G	A	1.62E-03	PRS-CSx
Colorectum	PRS7	rs12297526	12	113069225	A	G	2.37E-04	PRS-CSx
Colorectum	PRS7	rs12301701	12	86472798	C	T	-4.95E-05	PRS-CSx
Colorectum	PRS7	rs12302961	12	120462482	G	A	1.85E-04	PRS-CSx
Colorectum	PRS7	rs12307364	12	96382938	T	C	-4.38E-04	PRS-CSx
Colorectum	PRS7	rs12308122	12	79956929	T	C	4.07E-04	PRS-CSx
Colorectum	PRS7	rs12308147	12	83755861	A	C	-8.83E-04	PRS-CSx
Colorectum	PRS7	rs12308241	12	70152268	G	A	4.75E-04	PRS-CSx
Colorectum	PRS7	rs12310098	12	70177488	C	T	3.10E-04	PRS-CSx
Colorectum	PRS7	rs12315181	12	104912789	A	G	1.74E-03	PRS-CSx
Colorectum	PRS7	rs12315725	12	25231235	T	C	-2.80E-04	PRS-CSx
Colorectum	PRS7	rs12321987	12	57288449	A	G	-3.70E-05	PRS-CSx
Colorectum	PRS7	rs12322954	12	23184198	A	G	-1.15E-03	PRS-CSx
Colorectum	PRS7	rs12327024	18	3356405	C	T	2.24E-03	PRS-CSx
Colorectum	PRS7	rs1232965	12	47960471	A	G	1.30E-03	PRS-CSx
Colorectum	PRS7	rs12330209	3	113020075	C	T	-2.89E-04	PRS-CSx
Colorectum	PRS7	rs12330539	3	168545727	A	G	-2.57E-03	PRS-CSx
Colorectum	PRS7	rs12333884	7	157452854	T	C	-7.49E-04	PRS-CSx
Colorectum	PRS7	rs1233710	6	28215446	T	C	1.93E-04	PRS-CSx
Colorectum	PRS7	rs12347614	9	5493906	T	C	-7.88E-04	PRS-CSx
Colorectum	PRS7	rs12350098	9	121159542	A	G	1.03E-03	PRS-CSx
Colorectum	PRS7	rs12355538	10	20431598	T	C	-2.78E-04	PRS-CSx
Colorectum	PRS7	rs12359135	10	94843535	T	C	-4.98E-04	PRS-CSx
Colorectum	PRS7	rs12361305	11	69933614	A	C	-3.86E-04	PRS-CSx
Colorectum	PRS7	rs12361394	11	379097	C	T	-2.65E-04	PRS-CSx
Colorectum	PRS7	rs12361673	11	46723937	A	G	4.54E-04	PRS-CSx
Colorectum	PRS7	rs12367875	12	64407451	G	A	-1.56E-03	PRS-CSx
Colorectum	PRS7	rs12368048	12	52284668	A	C	-5.16E-04	PRS-CSx
Colorectum	PRS7	rs1236810	19	37966661	G	A	-4.31E-04	PRS-CSx
Colorectum	PRS7	rs12368504	12	25377692	C	T	-7.27E-04	PRS-CSx
Colorectum	PRS7	rs12369009	12	112019799	T	G	2.48E-04	PRS-CSx
Colorectum	PRS7	rs12369325	12	58599417	G	A	-6.35E-05	PRS-CSx
Colorectum	PRS7	rs12371600	12	16835982	A	C	-1.31E-03	PRS-CSx
Colorectum	PRS7	rs12372876	14	81630194	T	C	-8.61E-05	PRS-CSx
Colorectum	PRS7	rs12372893	14	77601210	T	C	-1.58E-03	PRS-CSx
Colorectum	PRS7	rs12376596	9	5497531	G	A	-1.23E-03	PRS-CSx
Colorectum	PRS7	rs12379750	9	3202558	T	C	-9.11E-05	PRS-CSx
Colorectum	PRS7	rs1238777	12	47978562	A	G	7.44E-04	PRS-CSx
Colorectum	PRS7	rs12401346	1	82352612	T	C	-2.00E-04	PRS-CSx

Colorectum	PRS7	rs1240259	12	70233934	C	T	2.54E-04	PRS-CSx
Colorectum	PRS7	rs12403507	1	192887993	G	A	-9.87E-04	PRS-CSx
Colorectum	PRS7	rs12403933	1	179423620	G	A	6.19E-05	PRS-CSx
Colorectum	PRS7	rs12406355	1	31417458	C	T	4.23E-05	PRS-CSx
Colorectum	PRS7	rs1240707	1	1335302	T	C	2.19E-04	PRS-CSx
Colorectum	PRS7	rs12408279	1	185039223	G	A	-1.89E-04	PRS-CSx
Colorectum	PRS7	rs12409926	1	185052427	T	C	-2.12E-04	PRS-CSx
Colorectum	PRS7	rs12410250	1	199234743	C	T	-1.10E-03	PRS-CSx
Colorectum	PRS7	rs12411753	10	3887162	A	G	-3.25E-04	PRS-CSx
Colorectum	PRS7	rs12411886	10	104685299	A	C	3.20E-04	PRS-CSx
Colorectum	PRS7	rs12411972	10	8638851	A	C	1.49E-05	PRS-CSx
Colorectum	PRS7	rs12412087	10	8651305	A	G	1.63E-04	PRS-CSx
Colorectum	PRS7	rs12412391	10	101288935	G	A	3.13E-04	PRS-CSx
Colorectum	PRS7	rs12412483	10	91349049	T	G	1.47E-03	PRS-CSx
Colorectum	PRS7	rs12413046	10	104871204	G	A	5.49E-04	PRS-CSx
Colorectum	PRS7	rs12413409	10	104719096	A	G	2.56E-04	PRS-CSx
Colorectum	PRS7	rs12413565	10	101285116	A	C	2.97E-04	PRS-CSx
Colorectum	PRS7	rs12413862	10	85807213	T	G	1.45E-03	PRS-CSx
Colorectum	PRS7	rs1241711	14	92668898	C	T	1.84E-04	PRS-CSx
Colorectum	PRS7	rs1241716	14	92671404	A	G	2.87E-04	PRS-CSx
Colorectum	PRS7	rs1241717	14	92671617	C	T	2.97E-04	PRS-CSx
Colorectum	PRS7	rs12417519	11	47129397	C	T	1.34E-04	PRS-CSx
Colorectum	PRS7	rs12418045	11	94513070	C	T	2.74E-04	PRS-CSx
Colorectum	PRS7	rs12420741	11	94480096	T	C	2.22E-04	PRS-CSx
Colorectum	PRS7	rs1242127	11	117076564	A	G	-2.25E-04	PRS-CSx
Colorectum	PRS7	rs12421941	11	120194357	G	A	-1.21E-03	PRS-CSx
Colorectum	PRS7	rs12422918	12	64312610	T	G	-5.68E-03	PRS-CSx
Colorectum	PRS7	rs12423443	12	25330451	C	T	-1.88E-04	PRS-CSx
Colorectum	PRS7	rs12423521	12	114562870	C	A	2.90E-04	PRS-CSx
Colorectum	PRS7	rs12423811	12	114563147	C	T	3.16E-04	PRS-CSx
Colorectum	PRS7	rs12423873	12	115959934	C	T	4.54E-04	PRS-CSx
Colorectum	PRS7	rs12424847	12	115208179	T	C	1.15E-03	PRS-CSx
Colorectum	PRS7	rs12425190	12	111394929	A	C	5.04E-04	PRS-CSx
Colorectum	PRS7	rs12426927	12	59518859	T	C	-3.82E-04	PRS-CSx
Colorectum	PRS7	rs12427378	12	51074199	C	T	1.56E-04	PRS-CSx
Colorectum	PRS7	rs12427600	13	37460648	C	T	2.88E-03	PRS-CSx
Colorectum	PRS7	rs12427972	13	95894315	A	G	1.01E-03	PRS-CSx
Colorectum	PRS7	rs1242971	10	9254263	A	G	-4.59E-04	PRS-CSx
Colorectum	PRS7	rs12431227	13	73619288	T	C	7.29E-04	PRS-CSx
Colorectum	PRS7	rs12432505	14	81012039	C	T	1.78E-03	PRS-CSx
Colorectum	PRS7	rs12432519	14	51398966	T	G	4.16E-04	PRS-CSx
Colorectum	PRS7	rs12433746	14	105537476	C	A	-3.54E-04	PRS-CSx
Colorectum	PRS7	rs1243441	14	21482691	G	A	-8.52E-04	PRS-CSx
Colorectum	PRS7	rs12435695	14	59430630	C	T	6.12E-04	PRS-CSx
Colorectum	PRS7	rs12437092	14	81005140	G	T	1.97E-03	PRS-CSx
Colorectum	PRS7	rs12440046	15	96446579	C	T	2.49E-03	PRS-CSx
Colorectum	PRS7	rs12442191	15	79561023	T	C	-6.25E-04	PRS-CSx
Colorectum	PRS7	rs12442632	15	71700427	C	T	1.89E-04	PRS-CSx
Colorectum	PRS7	rs12442719	15	25966038	C	A	3.67E-03	PRS-CSx
Colorectum	PRS7	rs12443696	16	69490554	A	C	2.17E-04	PRS-CSx
Colorectum	PRS7	rs12443866	16	86702431	T	C	1.17E-03	PRS-CSx
Colorectum	PRS7	rs1244471	10	7866557	T	C	-4.99E-04	PRS-CSx
Colorectum	PRS7	rs12445755	16	50034945	C	T	-4.51E-04	PRS-CSx
Colorectum	PRS7	rs12446951	16	8315470	A	G	1.28E-03	PRS-CSx
Colorectum	PRS7	rs12447057	16	86168727	C	T	1.21E-03	PRS-CSx
Colorectum	PRS7	rs12447091	16	86168845	C	T	6.51E-04	PRS-CSx

Colorectum	PRS7	rs12447341	16	68873686	T	C	4.61E-04	PRS-CSx
Colorectum	PRS7	rs12449276	16	87580137	T	C	-1.02E-03	PRS-CSx
Colorectum	PRS7	rs12449298	17	5105047	C	T	3.13E-04	PRS-CSx
Colorectum	PRS7	rs12451379	17	70268997	A	G	-1.10E-03	PRS-CSx
Colorectum	PRS7	rs1245371	6	30038352	G	A	-3.32E-05	PRS-CSx
Colorectum	PRS7	rs12454987	18	9518126	G	A	-3.27E-04	PRS-CSx
Colorectum	PRS7	rs12455102	18	42883959	C	A	8.07E-04	PRS-CSx
Colorectum	PRS7	rs1245526	10	73797257	T	C	2.15E-05	PRS-CSx
Colorectum	PRS7	rs1245530	10	73792268	T	C	-3.77E-05	PRS-CSx
Colorectum	PRS7	rs1245531	10	73790819	A	G	-3.17E-04	PRS-CSx
Colorectum	PRS7	rs1245582	10	73778267	T	C	-3.53E-04	PRS-CSx
Colorectum	PRS7	rs12457094	18	9482551	A	G	-3.27E-04	PRS-CSx
Colorectum	PRS7	rs12457670	18	41972228	T	C	3.18E-04	PRS-CSx
Colorectum	PRS7	rs12457729	18	44940201	G	A	8.35E-04	PRS-CSx
Colorectum	PRS7	rs12461902	19	30265235	A	G	1.45E-03	PRS-CSx
Colorectum	PRS7	rs12463769	2	160005259	T	C	-2.80E-04	PRS-CSx
Colorectum	PRS7	rs12464308	2	42527766	T	C	-1.86E-04	PRS-CSx
Colorectum	PRS7	rs12464702	2	42531871	G	T	-5.87E-05	PRS-CSx
Colorectum	PRS7	rs12465160	2	42567290	C	T	-6.89E-05	PRS-CSx
Colorectum	PRS7	rs12465343	2	101370474	T	C	-2.14E-03	PRS-CSx
Colorectum	PRS7	rs12465809	2	42483198	T	C	-1.72E-04	PRS-CSx
Colorectum	PRS7	rs12466303	2	225772004	A	G	3.84E-04	PRS-CSx
Colorectum	PRS7	rs1246642	4	89864446	C	T	1.64E-04	PRS-CSx
Colorectum	PRS7	rs12466841	2	207979735	G	A	-1.15E-04	PRS-CSx
Colorectum	PRS7	rs12468232	2	183339722	C	T	2.34E-04	PRS-CSx
Colorectum	PRS7	rs12468596	2	28503941	A	G	5.60E-04	PRS-CSx
Colorectum	PRS7	rs12468777	2	96038810	G	A	-2.38E-04	PRS-CSx
Colorectum	PRS7	rs12469725	2	469494	G	A	1.35E-03	PRS-CSx
Colorectum	PRS7	rs12470242	2	28546023	G	A	4.69E-04	PRS-CSx
Colorectum	PRS7	rs12470887	2	155324303	C	T	6.43E-04	PRS-CSx
Colorectum	PRS7	rs12474501	2	101642843	T	G	-5.12E-04	PRS-CSx
Colorectum	PRS7	rs12475350	2	42484616	A	G	3.18E-06	PRS-CSx
Colorectum	PRS7	rs12477063	2	215673664	C	T	4.58E-04	PRS-CSx
Colorectum	PRS7	rs12477901	2	48711523	G	A	3.88E-04	PRS-CSx
Colorectum	PRS7	rs12479765	20	33825378	A	G	-1.04E-03	PRS-CSx
Colorectum	PRS7	rs12481108	20	22384894	T	G	6.06E-04	PRS-CSx
Colorectum	PRS7	rs12484776	22	40652873	G	A	-2.80E-03	PRS-CSx
Colorectum	PRS7	rs12487617	3	60242432	A	G	-5.07E-04	PRS-CSx
Colorectum	PRS7	rs12488358	3	133904140	C	T	6.67E-04	PRS-CSx
Colorectum	PRS7	rs12489140	3	151631698	C	T	1.84E-05	PRS-CSx
Colorectum	PRS7	rs12491930	3	112966416	T	C	-5.68E-04	PRS-CSx
Colorectum	PRS7	rs12492273	3	112970775	A	C	1.83E-04	PRS-CSx
Colorectum	PRS7	rs12492700	3	137248353	T	C	-1.64E-03	PRS-CSx
Colorectum	PRS7	rs12493038	3	115296034	A	C	1.22E-03	PRS-CSx
Colorectum	PRS7	rs12494864	3	112973240	T	C	1.50E-04	PRS-CSx
Colorectum	PRS7	rs1249488	18	8715107	G	T	-2.98E-03	PRS-CSx
Colorectum	PRS7	rs12499925	4	148066627	T	G	-1.10E-03	PRS-CSx
Colorectum	PRS7	rs12500053	4	177442955	G	A	6.35E-04	PRS-CSx
Colorectum	PRS7	rs12500273	4	86901572	A	G	-4.94E-04	PRS-CSx
Colorectum	PRS7	rs12501140	4	167433524	G	A	6.62E-04	PRS-CSx
Colorectum	PRS7	rs12501220	4	115366345	A	G	3.56E-04	PRS-CSx
Colorectum	PRS7	rs12501998	4	155407569	T	C	-2.33E-04	PRS-CSx
Colorectum	PRS7	rs1250220	2	216320050	C	A	-2.75E-04	PRS-CSx
Colorectum	PRS7	rs1250252	2	216313591	T	G	-1.51E-04	PRS-CSx
Colorectum	PRS7	rs1250253	2	216313693	C	T	-1.79E-03	PRS-CSx
Colorectum	PRS7	rs1250258	2	216300185	C	T	-1.39E-03	PRS-CSx

Colorectum	PRS7	rs12503242	4	115380868	T	G	-1.22E-04	PRS-CSx
Colorectum	PRS7	rs12503654	4	118024255	A	C	-2.34E-03	PRS-CSx
Colorectum	PRS7	rs12504213	4	145722308	T	G	7.18E-04	PRS-CSx
Colorectum	PRS7	rs12505696	4	89931070	T	C	5.68E-04	PRS-CSx
Colorectum	PRS7	rs12505709	4	163059253	T	C	-3.14E-04	PRS-CSx
Colorectum	PRS7	rs12506201	4	105891467	A	G	3.90E-04	PRS-CSx
Colorectum	PRS7	rs12507442	4	17276304	T	C	-8.25E-04	PRS-CSx
Colorectum	PRS7	rs12507444	4	17276324	T	C	-1.11E-03	PRS-CSx
Colorectum	PRS7	rs12508045	4	75594233	C	T	-9.04E-04	PRS-CSx
Colorectum	PRS7	rs12508215	4	115620259	G	A	4.53E-04	PRS-CSx
Colorectum	PRS7	rs12508524	4	89938362	A	G	1.67E-04	PRS-CSx
Colorectum	PRS7	rs12513313	4	163059187	G	A	-4.58E-04	PRS-CSx
Colorectum	PRS7	rs12513951	5	75840225	C	T	2.50E-04	PRS-CSx
Colorectum	PRS7	rs12514412	5	129266996	C	A	-1.71E-04	PRS-CSx
Colorectum	PRS7	rs12515446	5	145701961	A	G	-1.69E-03	PRS-CSx
Colorectum	PRS7	rs12516641	5	128349782	T	C	-2.42E-04	PRS-CSx
Colorectum	PRS7	rs12516833	5	39735991	T	G	-6.33E-04	PRS-CSx
Colorectum	PRS7	rs12517710	5	8650479	A	C	-1.21E-04	PRS-CSx
Colorectum	PRS7	rs12519356	5	134528928	A	G	1.69E-04	PRS-CSx
Colorectum	PRS7	rs12519783	5	8650225	G	A	-3.55E-05	PRS-CSx
Colorectum	PRS7	rs12520287	5	54847098	C	T	-5.84E-04	PRS-CSx
Colorectum	PRS7	rs12523705	6	22068273	G	A	1.33E-03	PRS-CSx
Colorectum	PRS7	rs12524374	6	122804727	G	A	6.44E-04	PRS-CSx
Colorectum	PRS7	rs12525220	6	32675470	A	G	3.20E-04	PRS-CSx
Colorectum	PRS7	rs12526151	6	32304085	G	A	-1.47E-04	PRS-CSx
Colorectum	PRS7	rs12527158	6	98408610	G	A	2.25E-04	PRS-CSx
Colorectum	PRS7	rs12529894	6	131294299	T	G	7.25E-04	PRS-CSx
Colorectum	PRS7	rs12530233	6	22085658	A	C	3.53E-03	PRS-CSx
Colorectum	PRS7	rs12531817	7	45086038	T	C	-3.45E-04	PRS-CSx
Colorectum	PRS7	rs12532492	7	134565347	C	A	-5.56E-04	PRS-CSx
Colorectum	PRS7	rs12536178	7	45178748	T	C	-3.46E-04	PRS-CSx
Colorectum	PRS7	rs12537271	7	77300726	T	C	-2.61E-04	PRS-CSx
Colorectum	PRS7	rs12540482	7	47437150	T	C	-1.78E-03	PRS-CSx
Colorectum	PRS7	rs12540880	7	117719010	A	C	1.53E-03	PRS-CSx
Colorectum	PRS7	rs12542981	8	117604752	G	A	-3.15E-07	PRS-CSx
Colorectum	PRS7	rs12543106	8	128573298	G	A	-1.74E-03	PRS-CSx
Colorectum	PRS7	rs12544206	8	146235564	C	T	2.60E-04	PRS-CSx
Colorectum	PRS7	rs12544472	8	16134094	A	G	1.65E-03	PRS-CSx
Colorectum	PRS7	rs12546046	8	4164444	G	A	4.03E-04	PRS-CSx
Colorectum	PRS7	rs12548881	8	4165096	A	G	2.62E-03	PRS-CSx
Colorectum	PRS7	rs12549845	8	128402907	G	A	-5.17E-04	PRS-CSx
Colorectum	PRS7	rs125505	14	73709015	C	T	3.80E-04	PRS-CSx
Colorectum	PRS7	rs12550805	8	65745896	C	T	-5.33E-04	PRS-CSx
Colorectum	PRS7	rs12551402	9	118260090	T	C	-2.04E-03	PRS-CSx
Colorectum	PRS7	rs12555238	9	139743497	T	C	-4.42E-04	PRS-CSx
Colorectum	PRS7	rs12555291	9	34011391	A	G	-2.85E-04	PRS-CSx
Colorectum	PRS7	rs1256114	14	64810005	A	G	4.17E-04	PRS-CSx
Colorectum	PRS7	rs12561770	1	242874434	T	C	-1.01E-03	PRS-CSx
Colorectum	PRS7	rs12562614	1	201814994	G	A	-2.56E-04	PRS-CSx
Colorectum	PRS7	rs12563171	1	155579484	C	T	-4.17E-03	PRS-CSx
Colorectum	PRS7	rs12564167	1	41041946	G	A	-1.11E-03	PRS-CSx
Colorectum	PRS7	rs12564336	1	73851675	T	C	2.59E-03	PRS-CSx
Colorectum	PRS7	rs125682	5	37337147	T	C	2.29E-04	PRS-CSx
Colorectum	PRS7	rs12568612	1	223879083	A	G	-4.33E-04	PRS-CSx
Colorectum	PRS7	rs12568930	1	22702231	C	T	-1.85E-03	PRS-CSx
Colorectum	PRS7	rs12569076	1	218351800	A	G	-5.04E-05	PRS-CSx

Colorectum	PRS7	rs12569425	10	111758177	A	G	-1.39E-04	PRS-CSx
Colorectum	PRS7	rs12571674	10	111717608	G	A	-1.94E-04	PRS-CSx
Colorectum	PRS7	rs12572608	10	91406927	T	C	-9.01E-04	PRS-CSx
Colorectum	PRS7	rs12573077	10	104434630	A	C	-3.82E-05	PRS-CSx
Colorectum	PRS7	rs12573128	10	114730797	A	G	-4.14E-03	PRS-CSx
Colorectum	PRS7	rs1257334	14	99783405	G	A	3.92E-04	PRS-CSx
Colorectum	PRS7	rs12574859	11	16026291	A	C	2.92E-04	PRS-CSx
Colorectum	PRS7	rs12577378	11	16028010	C	A	1.92E-04	PRS-CSx
Colorectum	PRS7	rs12577495	11	16072004	C	T	3.60E-04	PRS-CSx
Colorectum	PRS7	rs12578552	12	75278353	G	A	1.04E-03	PRS-CSx
Colorectum	PRS7	rs12578742	12	111622980	A	G	7.84E-05	PRS-CSx
Colorectum	PRS7	rs12579073	12	25375799	C	A	-5.86E-04	PRS-CSx
Colorectum	PRS7	rs12579942	12	25383194	C	T	-7.90E-04	PRS-CSx
Colorectum	PRS7	rs12580046	12	75300518	A	G	5.47E-04	PRS-CSx
Colorectum	PRS7	rs12580054	12	115282829	G	A	3.38E-04	PRS-CSx
Colorectum	PRS7	rs12581952	12	104913221	T	C	1.25E-03	PRS-CSx
Colorectum	PRS7	rs12582427	12	120363678	A	G	-4.66E-04	PRS-CSx
Colorectum	PRS7	rs12583036	13	28759498	A	G	-4.33E-04	PRS-CSx
Colorectum	PRS7	rs12583344	13	38243182	G	A	7.12E-04	PRS-CSx
Colorectum	PRS7	rs12584544	13	42768720	A	G	3.27E-04	PRS-CSx
Colorectum	PRS7	rs12585075	13	42711224	T	C	4.56E-04	PRS-CSx
Colorectum	PRS7	rs12585788	13	41663556	A	G	1.69E-03	PRS-CSx
Colorectum	PRS7	rs12587093	14	95702003	C	T	-2.59E-04	PRS-CSx
Colorectum	PRS7	rs12587308	14	59351925	A	G	4.09E-05	PRS-CSx
Colorectum	PRS7	rs1258746	15	33087738	C	T	9.81E-04	PRS-CSx
Colorectum	PRS7	rs1258763	15	33050423	T	C	-5.64E-04	PRS-CSx
Colorectum	PRS7	rs12588202	14	22744408	A	G	-1.88E-03	PRS-CSx
Colorectum	PRS7	rs12588427	14	63269317	T	C	4.14E-04	PRS-CSx
Colorectum	PRS7	rs12589265	14	92718097	G	A	9.11E-04	PRS-CSx
Colorectum	PRS7	rs12589665	14	51364371	C	T	9.10E-04	PRS-CSx
Colorectum	PRS7	rs12591311	15	85087259	T	C	-1.18E-03	PRS-CSx
Colorectum	PRS7	rs12591400	15	48901313	T	C	9.04E-04	PRS-CSx
Colorectum	PRS7	rs12591780	15	38355543	A	G	-5.60E-04	PRS-CSx
Colorectum	PRS7	rs12592288	15	33006508	G	A	-2.10E-04	PRS-CSx
Colorectum	PRS7	rs12592342	15	31749336	C	T	5.82E-04	PRS-CSx
Colorectum	PRS7	rs12593223	15	33092962	A	G	6.90E-04	PRS-CSx
Colorectum	PRS7	rs12594203	15	51583811	T	C	1.41E-04	PRS-CSx
Colorectum	PRS7	rs12594592	15	66776440	A	G	-2.75E-04	PRS-CSx
Colorectum	PRS7	rs12594918	15	33125483	G	T	1.84E-04	PRS-CSx
Colorectum	PRS7	rs12594923	15	66991665	C	T	-2.34E-03	PRS-CSx
Colorectum	PRS7	rs12595176	15	66825689	T	C	-3.22E-04	PRS-CSx
Colorectum	PRS7	rs12596308	16	29691196	C	T	6.58E-04	PRS-CSx
Colorectum	PRS7	rs12597188	16	68814826	A	G	-3.18E-04	PRS-CSx
Colorectum	PRS7	rs12597511	16	31145219	C	T	3.06E-04	PRS-CSx
Colorectum	PRS7	rs12599832	16	53128056	A	G	9.28E-04	PRS-CSx
Colorectum	PRS7	rs12600425	17	65521722	C	A	7.43E-04	PRS-CSx
Colorectum	PRS7	rs12600474	17	63080162	G	A	1.89E-03	PRS-CSx
Colorectum	PRS7	rs12601438	17	6249301	T	C	-9.28E-05	PRS-CSx
Colorectum	PRS7	rs12602	19	41889748	C	T	1.05E-04	PRS-CSx
Colorectum	PRS7	rs12603614	17	61622703	G	A	-7.16E-04	PRS-CSx
Colorectum	PRS7	rs12603796	17	87761	C	A	1.91E-03	PRS-CSx
Colorectum	PRS7	rs12604216	18	59759334	A	G	-1.51E-04	PRS-CSx
Colorectum	PRS7	rs12605825	18	42068006	C	T	2.54E-04	PRS-CSx
Colorectum	PRS7	rs12605978	18	45570094	T	C	1.03E-03	PRS-CSx
Colorectum	PRS7	rs12606390	18	54013942	G	A	-4.27E-04	PRS-CSx
Colorectum	PRS7	rs12606438	18	42041063	C	A	2.17E-04	PRS-CSx

Colorectum	PRS7	rs12607516	18	41252700	T	G	-1.45E-03	PRS-CSx
Colorectum	PRS7	rs12607519	18	73299345	C	T	4.86E-04	PRS-CSx
Colorectum	PRS7	rs12607624	18	59779231	G	A	-1.42E-04	PRS-CSx
Colorectum	PRS7	rs12609177	19	33705455	G	A	-1.42E-03	PRS-CSx
Colorectum	PRS7	rs12609976	19	55587822	A	G	-1.63E-03	PRS-CSx
Colorectum	PRS7	rs12610760	19	7516243	G	A	1.81E-03	PRS-CSx
Colorectum	PRS7	rs12611771	2	182594588	T	C	-5.42E-04	PRS-CSx
Colorectum	PRS7	rs12611811	2	225318971	T	C	3.44E-04	PRS-CSx
Colorectum	PRS7	rs12612013	2	154115765	C	T	4.08E-04	PRS-CSx
Colorectum	PRS7	rs12612273	2	131867409	C	T	-2.87E-04	PRS-CSx
Colorectum	PRS7	rs12613026	2	43014289	C	T	2.44E-04	PRS-CSx
Colorectum	PRS7	rs12613725	2	225323096	A	G	6.50E-04	PRS-CSx
Colorectum	PRS7	rs12614491	2	144290246	A	G	7.07E-04	PRS-CSx
Colorectum	PRS7	rs12614514	2	228651032	A	G	1.71E-03	PRS-CSx
Colorectum	PRS7	rs12614687	2	182584473	C	T	-9.33E-04	PRS-CSx
Colorectum	PRS7	rs12615167	2	48749047	C	T	-7.53E-05	PRS-CSx
Colorectum	PRS7	rs12615297	2	71431885	G	A	-4.20E-04	PRS-CSx
Colorectum	PRS7	rs12615630	2	174384382	T	C	-2.14E-03	PRS-CSx
Colorectum	PRS7	rs12617051	2	43013772	C	T	3.44E-04	PRS-CSx
Colorectum	PRS7	rs12617500	2	1781022	A	C	3.06E-03	PRS-CSx
Colorectum	PRS7	rs12620183	2	114430089	A	G	2.19E-03	PRS-CSx
Colorectum	PRS7	rs12621402	2	159791735	T	G	-1.04E-04	PRS-CSx
Colorectum	PRS7	rs12623277	2	225548072	T	G	2.80E-04	PRS-CSx
Colorectum	PRS7	rs12624640	20	32952125	A	G	-5.06E-05	PRS-CSx
Colorectum	PRS7	rs12624718	20	22463647	G	A	7.57E-04	PRS-CSx
Colorectum	PRS7	rs12625436	20	57445213	G	A	-4.36E-04	PRS-CSx
Colorectum	PRS7	rs12625521	20	7786257	A	C	6.03E-04	PRS-CSx
Colorectum	PRS7	rs12625704	20	13694028	T	C	2.68E-04	PRS-CSx
Colorectum	PRS7	rs12627144	21	16077304	A	G	-1.96E-04	PRS-CSx
Colorectum	PRS7	rs12630461	3	168361891	T	G	-6.33E-04	PRS-CSx
Colorectum	PRS7	rs12630518	3	113135543	A	G	1.44E-04	PRS-CSx
Colorectum	PRS7	rs12630592	3	119768246	G	T	9.49E-05	PRS-CSx
Colorectum	PRS7	rs12630715	3	112880835	C	T	-2.50E-04	PRS-CSx
Colorectum	PRS7	rs12631199	3	112872821	G	A	-4.79E-04	PRS-CSx
Colorectum	PRS7	rs12631354	3	133913889	C	T	9.00E-04	PRS-CSx
Colorectum	PRS7	rs12631386	3	133800471	G	A	4.32E-04	PRS-CSx
Colorectum	PRS7	rs12631535	3	38731117	C	T	-5.96E-04	PRS-CSx
Colorectum	PRS7	rs12631733	3	112991117	T	C	3.27E-04	PRS-CSx
Colorectum	PRS7	rs12632728	3	168445884	T	C	-1.85E-04	PRS-CSx
Colorectum	PRS7	rs12632905	3	8903181	A	G	-1.73E-03	PRS-CSx
Colorectum	PRS7	rs12633127	3	119567553	G	T	-1.84E-03	PRS-CSx
Colorectum	PRS7	rs12634381	3	96168115	A	G	-6.23E-03	PRS-CSx
Colorectum	PRS7	rs12635144	3	133798207	T	C	9.04E-04	PRS-CSx
Colorectum	PRS7	rs12636082	3	117999221	A	G	-1.90E-03	PRS-CSx
Colorectum	PRS7	rs12636686	3	69263857	C	A	-1.08E-03	PRS-CSx
Colorectum	PRS7	rs1263674	2	208055723	C	T	-1.71E-04	PRS-CSx
Colorectum	PRS7	rs1263686	2	208050149	T	C	-4.00E-04	PRS-CSx
Colorectum	PRS7	rs12637343	3	15389816	C	T	-1.42E-03	PRS-CSx
Colorectum	PRS7	rs12641141	4	26913191	A	C	-3.54E-03	PRS-CSx
Colorectum	PRS7	rs12642740	4	177511710	A	G	1.80E-04	PRS-CSx
Colorectum	PRS7	rs1264420	6	30575603	A	G	-6.66E-05	PRS-CSx
Colorectum	PRS7	rs1264423	6	30571471	T	C	-1.30E-04	PRS-CSx
Colorectum	PRS7	rs12644238	4	115481754	T	G	3.34E-04	PRS-CSx
Colorectum	PRS7	rs1264471	8	102428258	T	G	-8.50E-04	PRS-CSx
Colorectum	PRS7	rs12644889	4	177522973	G	A	5.72E-04	PRS-CSx
Colorectum	PRS7	rs1264569	6	30365320	C	T	-9.34E-04	PRS-CSx

Colorectum	PRS7	rs1264708	6	30057154	G	A	3.43E-04	PRS-CSx
Colorectum	PRS7	rs12647475	4	64299915	T	G	8.95E-04	PRS-CSx
Colorectum	PRS7	rs12648803	4	115467384	T	C	3.03E-04	PRS-CSx
Colorectum	PRS7	rs12648883	4	115497660	A	G	1.81E-04	PRS-CSx
Colorectum	PRS7	rs12649176	4	177527851	G	A	7.14E-04	PRS-CSx
Colorectum	PRS7	rs12649800	4	177527905	G	T	5.21E-04	PRS-CSx
Colorectum	PRS7	rs12650546	4	115499674	T	C	3.83E-04	PRS-CSx
Colorectum	PRS7	rs12651300	4	115656257	C	T	1.83E-03	PRS-CSx
Colorectum	PRS7	rs12652642	5	125972624	G	T	6.62E-04	PRS-CSx
Colorectum	PRS7	rs12653481	5	10237036	A	G	-7.73E-04	PRS-CSx
Colorectum	PRS7	rs12653848	5	141489853	C	T	8.32E-04	PRS-CSx
Colorectum	PRS7	rs1265566	12	111716376	C	T	1.40E-03	PRS-CSx
Colorectum	PRS7	rs12655861	5	125967653	A	G	5.83E-04	PRS-CSx
Colorectum	PRS7	rs12656046	5	72597536	A	G	-1.46E-03	PRS-CSx
Colorectum	PRS7	rs12657313	5	31284709	C	A	3.86E-04	PRS-CSx
Colorectum	PRS7	rs12657484	5	134503751	T	C	4.90E-04	PRS-CSx
Colorectum	PRS7	rs12658795	5	133872704	A	G	-8.09E-04	PRS-CSx
Colorectum	PRS7	rs12659111	5	39652260	A	C	1.67E-04	PRS-CSx
Colorectum	PRS7	rs12662158	6	6726927	C	T	-2.83E-04	PRS-CSx
Colorectum	PRS7	rs12663576	6	1590288	G	A	1.83E-03	PRS-CSx
Colorectum	PRS7	rs12664560	6	151470986	G	A	1.31E-03	PRS-CSx
Colorectum	PRS7	rs12665144	6	39083288	T	C	8.79E-04	PRS-CSx
Colorectum	PRS7	rs12668183	7	16495406	C	T	-7.37E-04	PRS-CSx
Colorectum	PRS7	rs12668221	7	92736179	C	A	-3.70E-04	PRS-CSx
Colorectum	PRS7	rs12668381	7	128790796	G	A	-6.16E-04	PRS-CSx
Colorectum	PRS7	rs12671280	7	136979249	T	C	-1.31E-03	PRS-CSx
Colorectum	PRS7	rs12672022	7	45136423	C	T	-3.76E-04	PRS-CSx
Colorectum	PRS7	rs12672801	7	34542753	A	G	8.40E-04	PRS-CSx
Colorectum	PRS7	rs12672982	7	35424928	C	T	-1.03E-03	PRS-CSx
Colorectum	PRS7	rs12673438	7	35461364	G	T	-5.77E-04	PRS-CSx
Colorectum	PRS7	rs12675093	8	117798198	G	A	5.66E-04	PRS-CSx
Colorectum	PRS7	rs12677319	8	23640272	C	T	-1.55E-03	PRS-CSx
Colorectum	PRS7	rs12678061	8	21457258	T	C	3.65E-04	PRS-CSx
Colorectum	PRS7	rs12678349	8	128198564	T	C	-1.21E-03	PRS-CSx
Colorectum	PRS7	rs12678365	8	23678000	G	A	-1.07E-03	PRS-CSx
Colorectum	PRS7	rs12678791	8	23640529	A	G	-1.22E-03	PRS-CSx
Colorectum	PRS7	rs12679	12	109968427	T	C	-3.23E-04	PRS-CSx
Colorectum	PRS7	rs1267942	6	122793520	A	G	5.18E-04	PRS-CSx
Colorectum	PRS7	rs12679689	8	121244377	G	A	-3.12E-04	PRS-CSx
Colorectum	PRS7	rs12680075	8	129148524	C	T	1.07E-03	PRS-CSx
Colorectum	PRS7	rs12680302	8	23654692	T	C	-1.81E-03	PRS-CSx
Colorectum	PRS7	rs12680722	8	21472776	C	T	-4.70E-04	PRS-CSx
Colorectum	PRS7	rs12680888	8	84097208	T	C	-3.35E-04	PRS-CSx
Colorectum	PRS7	rs12680914	8	61827676	T	G	3.66E-04	PRS-CSx
Colorectum	PRS7	rs12685505	9	88017606	C	T	9.13E-04	PRS-CSx
Colorectum	PRS7	rs12685656	9	85080395	G	A	-2.05E-04	PRS-CSx
Colorectum	PRS7	rs12693068	2	176422005	T	C	9.57E-04	PRS-CSx
Colorectum	PRS7	rs12694049	2	207143804	A	G	-2.35E-04	PRS-CSx
Colorectum	PRS7	rs12694643	2	225446603	A	G	1.32E-04	PRS-CSx
Colorectum	PRS7	rs12699798	7	16500884	A	G	6.55E-04	PRS-CSx
Colorectum	PRS7	rs12701942	7	41915225	G	A	6.78E-04	PRS-CSx
Colorectum	PRS7	rs12702255	7	46878747	A	G	-6.85E-04	PRS-CSx
Colorectum	PRS7	rs12702256	7	46879550	A	C	-5.62E-04	PRS-CSx
Colorectum	PRS7	rs12702340	7	47500360	G	A	-2.27E-04	PRS-CSx
Colorectum	PRS7	rs12704447	7	88839812	A	C	-3.32E-04	PRS-CSx
Colorectum	PRS7	rs12704449	7	88851492	G	A	-1.76E-04	PRS-CSx

Colorectum	PRS7	rs12705070	7	99656927	G	A	-3.54E-04	PRS-CSx
Colorectum	PRS7	rs12705071	7	99682026	C	T	-2.93E-04	PRS-CSx
Colorectum	PRS7	rs12706123	7	116570027	C	A	-6.93E-04	PRS-CSx
Colorectum	PRS7	rs12708519	15	75803200	C	T	3.30E-04	PRS-CSx
Colorectum	PRS7	rs12708901	16	53119733	T	G	9.06E-04	PRS-CSx
Colorectum	PRS7	rs12708960	16	56242477	G	A	-4.01E-04	PRS-CSx
Colorectum	PRS7	rs12709072	16	49911620	A	G	6.38E-04	PRS-CSx
Colorectum	PRS7	rs12710210	19	22702102	A	G	6.50E-04	PRS-CSx
Colorectum	PRS7	rs12710701	2	19344757	T	C	2.31E-05	PRS-CSx
Colorectum	PRS7	rs12712470	2	36135030	T	C	-3.21E-04	PRS-CSx
Colorectum	PRS7	rs12712842	2	42741478	G	A	-1.98E-04	PRS-CSx
Colorectum	PRS7	rs12712867	2	43135815	G	A	1.95E-04	PRS-CSx
Colorectum	PRS7	rs12712872	2	43280324	T	G	5.50E-04	PRS-CSx
Colorectum	PRS7	rs12713007	2	48484467	C	T	2.12E-03	PRS-CSx
Colorectum	PRS7	rs12714232	2	28597305	C	T	7.95E-05	PRS-CSx
Colorectum	PRS7	rs12716080	5	11166948	G	T	6.02E-04	PRS-CSx
Colorectum	PRS7	rs12716881	16	80063680	C	A	3.54E-04	PRS-CSx
Colorectum	PRS7	rs12719801	16	381141	T	C	-1.26E-04	PRS-CSx
Colorectum	PRS7	rs12723117	1	38388708	A	G	-4.62E-04	PRS-CSx
Colorectum	PRS7	rs12725126	1	179427632	A	G	2.54E-04	PRS-CSx
Colorectum	PRS7	rs12727472	1	104813869	C	A	2.09E-04	PRS-CSx
Colorectum	PRS7	rs12728438	1	38349400	A	G	-1.30E-04	PRS-CSx
Colorectum	PRS7	rs12729017	1	62674507	T	C	8.55E-04	PRS-CSx
Colorectum	PRS7	rs1272951	12	70261786	C	T	1.68E-04	PRS-CSx
Colorectum	PRS7	rs12729755	1	218936793	A	G	-2.17E-03	PRS-CSx
Colorectum	PRS7	rs12731309	1	2352146	T	C	-2.99E-03	PRS-CSx
Colorectum	PRS7	rs12733015	1	58019733	A	G	6.48E-04	PRS-CSx
Colorectum	PRS7	rs12733578	1	38380285	A	G	-4.87E-05	PRS-CSx
Colorectum	PRS7	rs12735048	1	117581819	G	T	-1.70E-03	PRS-CSx
Colorectum	PRS7	rs12739026	1	38401933	C	A	7.47E-04	PRS-CSx
Colorectum	PRS7	rs12739316	1	183052080	G	A	7.36E-04	PRS-CSx
Colorectum	PRS7	rs12742756	1	38347417	G	A	-2.10E-05	PRS-CSx
Colorectum	PRS7	rs12743493	1	2224836	A	G	1.35E-03	PRS-CSx
Colorectum	PRS7	rs12743834	1	38318929	C	T	-2.67E-04	PRS-CSx
Colorectum	PRS7	rs1274514	19	50871147	G	A	7.99E-04	PRS-CSx
Colorectum	PRS7	rs1274517	19	50868754	G	A	4.60E-04	PRS-CSx
Colorectum	PRS7	rs12745294	1	38309322	T	C	-1.43E-04	PRS-CSx
Colorectum	PRS7	rs12746518	1	218940037	A	G	-1.25E-03	PRS-CSx
Colorectum	PRS7	rs12746544	1	38389202	G	A	-7.14E-05	PRS-CSx
Colorectum	PRS7	rs12749645	1	105161220	C	T	-5.61E-04	PRS-CSx
Colorectum	PRS7	rs12751325	1	38289383	C	T	-8.30E-05	PRS-CSx
Colorectum	PRS7	rs12763142	10	88503041	G	A	-1.85E-04	PRS-CSx
Colorectum	PRS7	rs12767703	10	118049105	C	A	2.33E-04	PRS-CSx
Colorectum	PRS7	rs12768916	10	52630185	A	G	-1.22E-04	PRS-CSx
Colorectum	PRS7	rs12775302	10	104911356	G	A	-1.73E-04	PRS-CSx
Colorectum	PRS7	rs12775883	10	104485301	A	G	-1.43E-04	PRS-CSx
Colorectum	PRS7	rs12785223	10	104844011	G	A	-2.07E-05	PRS-CSx
Colorectum	PRS7	rs12792221	11	100318275	A	G	6.89E-04	PRS-CSx
Colorectum	PRS7	rs12792445	11	111176351	T	C	-1.51E-03	PRS-CSx
Colorectum	PRS7	rs12793521	11	36455766	T	C	4.82E-04	PRS-CSx
Colorectum	PRS7	rs12795702	11	128156314	G	A	-3.09E-04	PRS-CSx
Colorectum	PRS7	rs12800302	11	111257202	T	C	-9.27E-04	PRS-CSx
Colorectum	PRS7	rs1280049	6	76536333	A	C	-3.09E-04	PRS-CSx
Colorectum	PRS7	rs1280052	6	76538403	G	A	-2.04E-04	PRS-CSx
Colorectum	PRS7	rs12801185	11	33174044	C	T	-2.09E-04	PRS-CSx
Colorectum	PRS7	rs12801632	11	79177447	G	A	6.21E-04	PRS-CSx

Colorectum	PRS7	rs12801636	11	65391317	A	G	6.72E-04	PRS-CSx
Colorectum	PRS7	rs1280207	11	74227450	A	G	-6.22E-04	PRS-CSx
Colorectum	PRS7	rs12803820	11	67187352	T	C	3.18E-04	PRS-CSx
Colorectum	PRS7	rs12806740	11	120203628	A	G	-8.21E-05	PRS-CSx
Colorectum	PRS7	rs1281003	1	10995479	A	G	4.54E-03	PRS-CSx
Colorectum	PRS7	rs12810577	12	25346163	G	A	-1.89E-04	PRS-CSx
Colorectum	PRS7	rs12813551	12	25394779	C	T	-2.02E-04	PRS-CSx
Colorectum	PRS7	rs12813721	12	96096556	T	C	-2.67E-04	PRS-CSx
Colorectum	PRS7	rs12815195	12	111402363	C	T	4.30E-04	PRS-CSx
Colorectum	PRS7	rs12818548	12	111398098	C	T	4.05E-04	PRS-CSx
Colorectum	PRS7	rs12821546	12	113421977	C	A	-6.39E-04	PRS-CSx
Colorectum	PRS7	rs12822102	12	52261255	G	A	-1.37E-03	PRS-CSx
Colorectum	PRS7	rs12822710	12	77333437	C	T	-3.95E-04	PRS-CSx
Colorectum	PRS7	rs12827748	12	80088578	C	T	-2.82E-04	PRS-CSx
Colorectum	PRS7	rs12828640	12	111361298	A	G	-2.30E-04	PRS-CSx
Colorectum	PRS7	rs12828893	12	77338293	T	C	-3.91E-04	PRS-CSx
Colorectum	PRS7	rs12831858	12	51259197	G	A	6.60E-04	PRS-CSx
Colorectum	PRS7	rs12853922	13	27083274	C	A	-7.80E-04	PRS-CSx
Colorectum	PRS7	rs12860899	13	51817495	T	C	-2.05E-03	PRS-CSx
Colorectum	PRS7	rs12863390	13	31403787	G	A	-7.35E-04	PRS-CSx
Colorectum	PRS7	rs1286884	13	28788543	A	G	-1.17E-04	PRS-CSx
Colorectum	PRS7	rs1286885	13	28786877	C	A	-2.24E-04	PRS-CSx
Colorectum	PRS7	rs12870730	13	98525890	T	G	-5.07E-03	PRS-CSx
Colorectum	PRS7	rs12878931	14	54549506	C	A	6.39E-04	PRS-CSx
Colorectum	PRS7	rs12879252	14	54375228	T	C	1.04E-03	PRS-CSx
Colorectum	PRS7	rs12879570	14	54375369	T	C	1.27E-03	PRS-CSx
Colorectum	PRS7	rs12879675	14	58707253	A	G	9.24E-05	PRS-CSx
Colorectum	PRS7	rs12880109	14	68813032	T	C	-3.12E-04	PRS-CSx
Colorectum	PRS7	rs12880795	14	96379833	T	C	-7.47E-05	PRS-CSx
Colorectum	PRS7	rs12881924	14	54363545	A	C	1.05E-03	PRS-CSx
Colorectum	PRS7	rs12883434	14	91806204	A	C	-1.01E-03	PRS-CSx
Colorectum	PRS7	rs12890124	14	68781614	A	C	-1.62E-04	PRS-CSx
Colorectum	PRS7	rs12892302	14	34966936	C	T	-6.82E-04	PRS-CSx
Colorectum	PRS7	rs12894505	14	91808042	C	A	-1.04E-03	PRS-CSx
Colorectum	PRS7	rs12895324	14	54374921	A	G	6.51E-04	PRS-CSx
Colorectum	PRS7	rs12895731	14	96598822	A	G	-5.04E-04	PRS-CSx
Colorectum	PRS7	rs12898572	15	48861517	C	A	1.39E-03	PRS-CSx
Colorectum	PRS7	rs12899579	15	27224321	A	G	7.61E-04	PRS-CSx
Colorectum	PRS7	rs12901270	15	57297513	C	A	4.02E-04	PRS-CSx
Colorectum	PRS7	rs12901416	15	49867122	A	G	3.07E-04	PRS-CSx
Colorectum	PRS7	rs12901499	15	67370445	A	G	-8.26E-04	PRS-CSx
Colorectum	PRS7	rs12902107	15	49867323	G	A	3.71E-04	PRS-CSx
Colorectum	PRS7	rs12902794	15	49867148	T	C	4.20E-04	PRS-CSx
Colorectum	PRS7	rs12903048	15	102006325	G	T	2.84E-04	PRS-CSx
Colorectum	PRS7	rs12903325	15	50353277	G	T	-9.19E-04	PRS-CSx
Colorectum	PRS7	rs12909189	15	48816080	A	G	8.92E-04	PRS-CSx
Colorectum	PRS7	rs12913258	15	82062829	G	T	-4.07E-04	PRS-CSx
Colorectum	PRS7	rs12913724	15	66534180	C	T	-1.83E-04	PRS-CSx
Colorectum	PRS7	rs12914734	15	33001264	T	C	-3.86E-04	PRS-CSx
Colorectum	PRS7	rs12915095	15	47594033	C	T	-3.32E-04	PRS-CSx
Colorectum	PRS7	rs12915166	15	67001327	A	G	-3.39E-04	PRS-CSx
Colorectum	PRS7	rs12915429	15	82105900	G	A	-4.40E-04	PRS-CSx
Colorectum	PRS7	rs12915616	15	71639634	A	G	6.64E-04	PRS-CSx
Colorectum	PRS7	rs12915677	15	48864797	T	C	4.47E-04	PRS-CSx
Colorectum	PRS7	rs12915721	15	38350877	A	G	-6.66E-04	PRS-CSx
Colorectum	PRS7	rs12916179	15	82058618	C	T	-5.64E-04	PRS-CSx

Colorectum	PRS7	rs1291625	12	46307048	C	T	-6.20E-04	PRS-CSx
Colorectum	PRS7	rs1291628	12	46326576	G	T	-4.38E-04	PRS-CSx
Colorectum	PRS7	rs12922275	16	57787027	T	C	2.66E-03	PRS-CSx
Colorectum	PRS7	rs12922462	16	49916173	T	C	6.41E-04	PRS-CSx
Colorectum	PRS7	rs1292504	11	74269630	A	G	4.19E-04	PRS-CSx
Colorectum	PRS7	rs12925474	16	11214655	G	T	-5.70E-04	PRS-CSx
Colorectum	PRS7	rs12927046	16	11242191	T	G	-1.06E-03	PRS-CSx
Colorectum	PRS7	rs12928518	16	86702768	C	T	1.43E-03	PRS-CSx
Colorectum	PRS7	rs12930371	16	68802936	T	C	-7.50E-04	PRS-CSx
Colorectum	PRS7	rs12934952	16	49914323	C	T	5.46E-04	PRS-CSx
Colorectum	PRS7	rs1293739	12	113452481	A	G	3.88E-04	PRS-CSx
Colorectum	PRS7	rs1293746	12	113445768	G	A	-3.34E-04	PRS-CSx
Colorectum	PRS7	rs1293766	12	113425282	G	T	3.88E-04	PRS-CSx
Colorectum	PRS7	rs1294023	1	9392088	A	G	-2.16E-03	PRS-CSx
Colorectum	PRS7	rs12940510	17	59260643	G	A	-2.97E-04	PRS-CSx
Colorectum	PRS7	rs12940987	17	59269257	G	A	-3.39E-04	PRS-CSx
Colorectum	PRS7	rs1294914	7	45232650	A	G	-5.44E-04	PRS-CSx
Colorectum	PRS7	rs12949197	17	61619379	T	C	-3.51E-04	PRS-CSx
Colorectum	PRS7	rs1294924	7	45221712	C	T	-3.30E-04	PRS-CSx
Colorectum	PRS7	rs1294935	7	45218366	T	C	-4.52E-04	PRS-CSx
Colorectum	PRS7	rs1294945	7	45215088	T	C	1.78E-04	PRS-CSx
Colorectum	PRS7	rs1294947	7	45211941	G	A	2.26E-04	PRS-CSx
Colorectum	PRS7	rs12952507	17	59265044	A	G	-2.12E-04	PRS-CSx
Colorectum	PRS7	rs12953717	18	46453929	T	C	1.19E-02	PRS-CSx
Colorectum	PRS7	rs1295882	10	18561134	T	C	1.04E-03	PRS-CSx
Colorectum	PRS7	rs1296112	13	28770458	A	G	-1.24E-04	PRS-CSx
Colorectum	PRS7	rs12963087	18	21632996	G	A	8.71E-04	PRS-CSx
Colorectum	PRS7	rs12963694	18	34116553	A	C	-3.04E-04	PRS-CSx
Colorectum	PRS7	rs12967616	18	34120761	T	C	-7.03E-04	PRS-CSx
Colorectum	PRS7	rs12975577	19	33755364	T	C	5.41E-04	PRS-CSx
Colorectum	PRS7	rs12981408	19	3411646	T	G	1.33E-03	PRS-CSx
Colorectum	PRS7	rs12984247	19	39152908	T	C	5.44E-04	PRS-CSx
Colorectum	PRS7	rs12994442	2	183437156	C	T	3.02E-04	PRS-CSx
Colorectum	PRS7	rs12997662	2	216167244	T	C	8.52E-04	PRS-CSx
Colorectum	PRS7	rs1299913	7	45215376	A	G	3.78E-04	PRS-CSx
Colorectum	PRS7	rs13000757	2	207145906	T	G	-4.14E-04	PRS-CSx
Colorectum	PRS7	rs13002186	2	200189480	C	T	-6.09E-05	PRS-CSx
Colorectum	PRS7	rs13002829	2	47486300	T	C	-3.23E-03	PRS-CSx
Colorectum	PRS7	rs13003153	2	154223883	G	A	3.77E-04	PRS-CSx
Colorectum	PRS7	rs13003464	2	61186829	G	A	-6.29E-04	PRS-CSx
Colorectum	PRS7	rs13004477	2	154224516	C	A	9.25E-04	PRS-CSx
Colorectum	PRS7	rs13004596	2	228644789	G	A	1.15E-03	PRS-CSx
Colorectum	PRS7	rs13005619	2	40558573	C	T	1.36E-03	PRS-CSx
Colorectum	PRS7	rs13006016	2	43082792	T	C	2.59E-03	PRS-CSx
Colorectum	PRS7	rs13008251	2	228640442	T	C	9.76E-04	PRS-CSx
Colorectum	PRS7	rs13010262	2	145781546	G	A	-2.19E-04	PRS-CSx
Colorectum	PRS7	rs130121	22	49095071	A	G	-8.60E-04	PRS-CSx
Colorectum	PRS7	rs13012795	2	42743543	C	T	-2.05E-04	PRS-CSx
Colorectum	PRS7	rs13013681	2	43084026	T	C	1.32E-03	PRS-CSx
Colorectum	PRS7	rs13015791	2	130219398	T	G	-3.27E-04	PRS-CSx
Colorectum	PRS7	rs13016514	2	42492191	A	C	-1.29E-04	PRS-CSx
Colorectum	PRS7	rs13018498	2	42445105	A	G	2.20E-05	PRS-CSx
Colorectum	PRS7	rs13018710	2	225793447	C	T	-4.29E-04	PRS-CSx
Colorectum	PRS7	rs13021690	2	225814772	A	G	-1.13E-04	PRS-CSx
Colorectum	PRS7	rs13024433	2	225774612	C	A	-6.77E-04	PRS-CSx
Colorectum	PRS7	rs13024598	2	43060396	A	G	1.51E-03	PRS-CSx

Colorectum	PRS7	rs13026062	2	155323748	C	A	9.42E-04	PRS-CSx
Colorectum	PRS7	rs13026071	2	107296922	A	G	-1.06E-03	PRS-CSx
Colorectum	PRS7	rs13028337	2	9617647	A	G	8.82E-04	PRS-CSx
Colorectum	PRS7	rs13030483	2	42696855	T	C	4.17E-05	PRS-CSx
Colorectum	PRS7	rs13032680	2	204441557	T	G	3.44E-04	PRS-CSx
Colorectum	PRS7	rs130328	22	43061482	G	A	6.27E-04	PRS-CSx
Colorectum	PRS7	rs13034664	2	228672579	A	G	1.31E-03	PRS-CSx
Colorectum	PRS7	rs130385	22	43082265	T	C	8.27E-05	PRS-CSx
Colorectum	PRS7	rs130389	22	43083045	C	A	1.86E-04	PRS-CSx
Colorectum	PRS7	rs13039439	20	62703490	T	C	-1.68E-03	PRS-CSx
Colorectum	PRS7	rs13043313	20	60958269	C	T	-6.94E-04	PRS-CSx
Colorectum	PRS7	rs13043928	20	49042548	C	T	-5.99E-04	PRS-CSx
Colorectum	PRS7	rs13045364	20	47375591	A	G	-3.03E-04	PRS-CSx
Colorectum	PRS7	rs13047696	21	48027177	G	A	1.35E-04	PRS-CSx
Colorectum	PRS7	rs13052308	21	43347556	T	C	4.70E-04	PRS-CSx
Colorectum	PRS7	rs13064288	3	151619656	A	G	-1.17E-03	PRS-CSx
Colorectum	PRS7	rs13064369	3	77539218	C	T	6.87E-05	PRS-CSx
Colorectum	PRS7	rs13066649	3	181905333	G	T	-7.65E-04	PRS-CSx
Colorectum	PRS7	rs13069553	3	169508272	A	G	8.42E-04	PRS-CSx
Colorectum	PRS7	rs13073804	3	16995165	A	G	-4.27E-04	PRS-CSx
Colorectum	PRS7	rs13076788	3	152311489	A	G	7.49E-04	PRS-CSx
Colorectum	PRS7	rs13079208	3	152116652	T	G	-5.52E-04	PRS-CSx
Colorectum	PRS7	rs13080253	3	130019843	C	T	-4.99E-04	PRS-CSx
Colorectum	PRS7	rs13080875	3	133752968	A	C	-1.94E-04	PRS-CSx
Colorectum	PRS7	rs13082346	3	40929572	T	C	4.73E-03	PRS-CSx
Colorectum	PRS7	rs13084153	3	62055170	T	C	1.50E-03	PRS-CSx
Colorectum	PRS7	rs13086367	3	112903888	G	A	-1.25E-03	PRS-CSx
Colorectum	PRS7	rs13091327	3	122607469	C	T	-1.14E-04	PRS-CSx
Colorectum	PRS7	rs13091753	3	188114589	T	G	-7.32E-04	PRS-CSx
Colorectum	PRS7	rs13093350	3	112993063	T	C	-2.19E-03	PRS-CSx
Colorectum	PRS7	rs13098886	3	157613783	G	A	8.25E-04	PRS-CSx
Colorectum	PRS7	rs13099397	3	61217770	C	A	4.43E-05	PRS-CSx
Colorectum	PRS7	rs13102609	4	145771221	T	G	9.18E-04	PRS-CSx
Colorectum	PRS7	rs13102647	4	22462657	C	T	-3.88E-04	PRS-CSx
Colorectum	PRS7	rs13102818	4	70116088	C	A	1.96E-04	PRS-CSx
Colorectum	PRS7	rs13105493	4	70100375	T	C	2.28E-04	PRS-CSx
Colorectum	PRS7	rs13106936	4	175415991	G	A	-5.53E-04	PRS-CSx
Colorectum	PRS7	rs13109695	4	22430209	T	C	-3.35E-04	PRS-CSx
Colorectum	PRS7	rs13109842	4	26333167	A	G	5.19E-04	PRS-CSx
Colorectum	PRS7	rs13110329	4	86871282	A	G	-4.54E-04	PRS-CSx
Colorectum	PRS7	rs13112066	4	155414179	A	G	-1.92E-04	PRS-CSx
Colorectum	PRS7	rs13116679	4	102199215	A	G	-3.05E-04	PRS-CSx
Colorectum	PRS7	rs13116988	4	58497400	T	C	4.02E-04	PRS-CSx
Colorectum	PRS7	rs13118539	4	23631863	A	G	-1.40E-03	PRS-CSx
Colorectum	PRS7	rs13120552	4	115473674	G	A	3.95E-04	PRS-CSx
Colorectum	PRS7	rs13121545	4	150280221	G	T	-2.34E-04	PRS-CSx
Colorectum	PRS7	rs13122890	4	120601731	T	G	-3.57E-04	PRS-CSx
Colorectum	PRS7	rs13129234	4	58474857	A	G	5.24E-04	PRS-CSx
Colorectum	PRS7	rs13130097	4	17349030	C	T	-4.34E-04	PRS-CSx
Colorectum	PRS7	rs13130903	4	127207959	A	G	1.20E-03	PRS-CSx
Colorectum	PRS7	rs13132855	4	44401267	A	G	-8.22E-04	PRS-CSx
Colorectum	PRS7	rs13135121	4	22430507	G	A	-2.51E-04	PRS-CSx
Colorectum	PRS7	rs13135838	4	175354852	G	A	-2.98E-04	PRS-CSx
Colorectum	PRS7	rs13136634	4	181289806	C	A	1.41E-03	PRS-CSx
Colorectum	PRS7	rs13137393	4	102171725	T	C	-2.25E-04	PRS-CSx
Colorectum	PRS7	rs13140580	4	150272872	A	C	-2.01E-04	PRS-CSx

Colorectum	PRS7	rs13143866	4	123540758	A	G	4.91E-04	PRS-CSx
Colorectum	PRS7	rs13144624	4	137589354	G	A	-8.80E-04	PRS-CSx
Colorectum	PRS7	rs13145010	4	127629723	A	G	-4.46E-04	PRS-CSx
Colorectum	PRS7	rs13145262	4	151477716	A	G	2.40E-04	PRS-CSx
Colorectum	PRS7	rs13148940	4	18892570	A	G	6.93E-05	PRS-CSx
Colorectum	PRS7	rs13150736	4	151446347	C	T	2.35E-04	PRS-CSx
Colorectum	PRS7	rs13151559	4	155407680	A	G	-2.53E-04	PRS-CSx
Colorectum	PRS7	rs13151692	4	151462122	G	T	2.66E-04	PRS-CSx
Colorectum	PRS7	rs1315212	11	74249090	T	G	-2.23E-04	PRS-CSx
Colorectum	PRS7	rs13152998	5	169101801	G	A	4.74E-04	PRS-CSx
Colorectum	PRS7	rs13153056	5	140366901	A	G	5.60E-04	PRS-CSx
Colorectum	PRS7	rs13154867	5	90494075	T	G	-1.52E-03	PRS-CSx
Colorectum	PRS7	rs13155298	5	39668076	C	T	-7.76E-05	PRS-CSx
Colorectum	PRS7	rs13156239	5	39668648	C	T	1.49E-04	PRS-CSx
Colorectum	PRS7	rs13156753	5	172319346	T	C	-5.36E-04	PRS-CSx
Colorectum	PRS7	rs13159095	5	40100134	C	T	3.81E-04	PRS-CSx
Colorectum	PRS7	rs13163241	5	140373888	T	C	6.07E-04	PRS-CSx
Colorectum	PRS7	rs1316502	3	152323637	G	A	4.12E-04	PRS-CSx
Colorectum	PRS7	rs13165386	5	143644639	G	A	-1.94E-04	PRS-CSx
Colorectum	PRS7	rs131654	22	21917190	G	T	5.16E-04	PRS-CSx
Colorectum	PRS7	rs1316607	12	51042890	A	C	2.59E-04	PRS-CSx
Colorectum	PRS7	rs1316611	10	80824689	G	A	-1.07E-02	PRS-CSx
Colorectum	PRS7	rs131665	22	21920903	A	G	5.88E-05	PRS-CSx
Colorectum	PRS7	rs13170207	5	125993349	C	T	1.72E-03	PRS-CSx
Colorectum	PRS7	rs1317082	3	169497585	A	G	9.00E-04	PRS-CSx
Colorectum	PRS7	rs13170944	5	39656895	A	G	3.11E-05	PRS-CSx
Colorectum	PRS7	rs13170960	5	39710129	G	A	-5.09E-04	PRS-CSx
Colorectum	PRS7	rs13171592	5	172314463	G	A	-2.77E-04	PRS-CSx
Colorectum	PRS7	rs13172994	5	50465915	A	G	-5.26E-04	PRS-CSx
Colorectum	PRS7	rs1317326	14	54000094	A	G	6.06E-04	PRS-CSx
Colorectum	PRS7	rs13173741	5	87197535	G	A	-4.93E-04	PRS-CSx
Colorectum	PRS7	rs13173933	5	178125921	A	G	-4.02E-04	PRS-CSx
Colorectum	PRS7	rs13175095	5	140377482	C	A	6.96E-04	PRS-CSx
Colorectum	PRS7	rs1317681	1	218575202	G	A	-1.07E-03	PRS-CSx
Colorectum	PRS7	rs1317816	6	25765390	T	C	1.20E-04	PRS-CSx
Colorectum	PRS7	rs13180157	5	54232372	G	A	-9.65E-04	PRS-CSx
Colorectum	PRS7	rs13182980	5	143644780	T	G	-1.49E-04	PRS-CSx
Colorectum	PRS7	rs13185944	5	133255854	C	T	-6.70E-04	PRS-CSx
Colorectum	PRS7	rs13185971	5	39637885	G	A	1.35E-04	PRS-CSx
Colorectum	PRS7	rs1318631	6	29635508	A	G	-2.90E-04	PRS-CSx
Colorectum	PRS7	rs1318821	5	31319179	A	G	-1.05E-04	PRS-CSx
Colorectum	PRS7	rs13188662	5	52091647	G	A	8.24E-04	PRS-CSx
Colorectum	PRS7	rs13189930	5	14727882	A	C	-6.58E-04	PRS-CSx
Colorectum	PRS7	rs13190060	5	129369375	G	T	-2.77E-04	PRS-CSx
Colorectum	PRS7	rs13195478	6	117814437	A	G	2.69E-04	PRS-CSx
Colorectum	PRS7	rs13196989	6	239373	T	C	-7.63E-04	PRS-CSx
Colorectum	PRS7	rs13198772	6	131443526	T	C	5.22E-04	PRS-CSx
Colorectum	PRS7	rs13202519	6	76584271	C	T	-2.10E-04	PRS-CSx
Colorectum	PRS7	rs1320380	18	69566940	T	C	5.78E-04	PRS-CSx
Colorectum	PRS7	rs13205986	6	117791674	T	C	2.57E-04	PRS-CSx
Colorectum	PRS7	rs13206250	6	56964127	G	A	-1.83E-03	PRS-CSx
Colorectum	PRS7	rs13206320	6	131414757	G	A	1.12E-03	PRS-CSx
Colorectum	PRS7	rs13212082	6	142050556	C	T	-5.25E-04	PRS-CSx
Colorectum	PRS7	rs1321310	6	36623124	C	T	3.43E-03	PRS-CSx
Colorectum	PRS7	rs1321311	6	36622900	A	C	3.76E-03	PRS-CSx
Colorectum	PRS7	rs1321440	20	6538532	A	G	7.03E-04	PRS-CSx

Colorectum	PRS7	rs1321443	20	6548863	C	T	9.92E-04	PRS-CSx
Colorectum	PRS7	rs1321482	6	26575154	T	C	-6.90E-05	PRS-CSx
Colorectum	PRS7	rs13216198	6	148218795	A	G	3.02E-04	PRS-CSx
Colorectum	PRS7	rs1321799	6	117731177	T	G	2.26E-04	PRS-CSx
Colorectum	PRS7	rs1321807	6	117684992	A	G	-2.59E-04	PRS-CSx
Colorectum	PRS7	rs13219670	6	57143175	G	A	-1.04E-03	PRS-CSx
Colorectum	PRS7	rs13219839	6	4940511	C	A	-8.80E-04	PRS-CSx
Colorectum	PRS7	rs132225	22	49095050	C	T	-5.67E-04	PRS-CSx
Colorectum	PRS7	rs13223792	7	99675659	G	T	-3.67E-04	PRS-CSx
Colorectum	PRS7	rs1322540	9	101619191	C	T	2.79E-04	PRS-CSx
Colorectum	PRS7	rs1322541	9	101624837	T	C	4.05E-04	PRS-CSx
Colorectum	PRS7	rs13225805	7	88130171	T	G	-4.33E-04	PRS-CSx
Colorectum	PRS7	rs13226636	7	46295568	A	C	-9.52E-04	PRS-CSx
Colorectum	PRS7	rs13226772	7	150478931	T	G	2.47E-03	PRS-CSx
Colorectum	PRS7	rs1322688	13	102548688	A	G	-5.34E-04	PRS-CSx
Colorectum	PRS7	rs13227011	7	114254728	A	G	1.56E-03	PRS-CSx
Colorectum	PRS7	rs13227328	7	70500643	T	C	-1.83E-03	PRS-CSx
Colorectum	PRS7	rs13233995	7	37108104	C	A	8.78E-04	PRS-CSx
Colorectum	PRS7	rs13236869	7	35533597	A	G	2.23E-03	PRS-CSx
Colorectum	PRS7	rs13240161	7	77804846	T	C	-6.22E-04	PRS-CSx
Colorectum	PRS7	rs1324192	9	13612345	G	A	1.71E-04	PRS-CSx
Colorectum	PRS7	rs13242412	7	88851701	G	A	-2.66E-04	PRS-CSx
Colorectum	PRS7	rs1324299	10	89393463	T	C	5.23E-04	PRS-CSx
Colorectum	PRS7	rs13244720	7	88136931	A	G	-4.55E-04	PRS-CSx
Colorectum	PRS7	rs13244745	7	16647832	G	A	-1.03E-03	PRS-CSx
Colorectum	PRS7	rs13245850	7	95937098	G	A	7.34E-04	PRS-CSx
Colorectum	PRS7	rs13247169	7	46308897	G	A	-1.06E-03	PRS-CSx
Colorectum	PRS7	rs13248944	8	128420558	C	T	4.28E-04	PRS-CSx
Colorectum	PRS7	rs13249356	8	117596023	T	G	1.46E-05	PRS-CSx
Colorectum	PRS7	rs13249563	8	20466541	T	C	-5.96E-04	PRS-CSx
Colorectum	PRS7	rs13249934	8	21460312	T	G	2.90E-04	PRS-CSx
Colorectum	PRS7	rs1325187	1	199217307	A	C	-1.71E-03	PRS-CSx
Colorectum	PRS7	rs13252526	8	21458911	G	A	1.70E-04	PRS-CSx
Colorectum	PRS7	rs13255034	8	58655846	G	T	-4.17E-04	PRS-CSx
Colorectum	PRS7	rs13257697	8	146233840	T	C	5.55E-04	PRS-CSx
Colorectum	PRS7	rs13258281	8	21473286	C	T	-3.68E-04	PRS-CSx
Colorectum	PRS7	rs13260351	8	65570867	T	C	-3.95E-04	PRS-CSx
Colorectum	PRS7	rs1326198	10	91597636	G	A	-2.30E-04	PRS-CSx
Colorectum	PRS7	rs13263036	8	83838243	A	G	-2.73E-04	PRS-CSx
Colorectum	PRS7	rs1326605	6	51618170	T	G	-8.17E-04	PRS-CSx
Colorectum	PRS7	rs13267780	8	128357817	A	G	1.42E-03	PRS-CSx
Colorectum	PRS7	rs13269669	8	4293080	G	A	-3.39E-04	PRS-CSx
Colorectum	PRS7	rs13270674	8	21483253	C	A	3.57E-04	PRS-CSx
Colorectum	PRS7	rs13270948	8	146238281	A	G	3.26E-04	PRS-CSx
Colorectum	PRS7	rs13272708	8	21457312	A	G	2.08E-04	PRS-CSx
Colorectum	PRS7	rs13273954	8	18729459	C	T	7.61E-04	PRS-CSx
Colorectum	PRS7	rs13275957	8	20466105	A	G	-3.55E-04	PRS-CSx
Colorectum	PRS7	rs13276825	8	49203046	A	G	4.69E-04	PRS-CSx
Colorectum	PRS7	rs13277937	8	83299295	C	T	4.87E-04	PRS-CSx
Colorectum	PRS7	rs13278525	8	4309702	T	C	-7.15E-04	PRS-CSx
Colorectum	PRS7	rs13279543	8	117809105	G	A	-3.87E-04	PRS-CSx
Colorectum	PRS7	rs13281508	8	83858931	T	C	-2.49E-04	PRS-CSx
Colorectum	PRS7	rs13282866	8	65561378	A	G	-2.25E-04	PRS-CSx
Colorectum	PRS7	rs1329125	1	234740880	T	C	2.83E-03	PRS-CSx
Colorectum	PRS7	rs1329126	1	234740549	C	A	7.88E-04	PRS-CSx
Colorectum	PRS7	rs1329252	10	25791843	T	G	-6.69E-04	PRS-CSx

Colorectum	PRS7	rs1329383	9	84070426	T	C	-7.52E-04	PRS-CSx
Colorectum	PRS7	rs1329520	13	41638611	G	A	-1.71E-03	PRS-CSx
Colorectum	PRS7	rs13296166	9	101812072	A	G	-4.31E-04	PRS-CSx
Colorectum	PRS7	rs1329730	9	19277320	C	T	9.16E-04	PRS-CSx
Colorectum	PRS7	rs13300482	9	15260948	A	G	-2.12E-03	PRS-CSx
Colorectum	PRS7	rs13302368	9	101807566	A	G	-4.24E-04	PRS-CSx
Colorectum	PRS7	rs1330472	13	92527649	G	A	3.97E-04	PRS-CSx
Colorectum	PRS7	rs1330633	6	57148971	G	A	-9.38E-04	PRS-CSx
Colorectum	PRS7	rs13306731	1	179320578	G	A	3.75E-04	PRS-CSx
Colorectum	PRS7	rs1330888	13	78609792	T	C	-9.26E-04	PRS-CSx
Colorectum	PRS7	rs1330897	13	78611929	G	A	-8.99E-04	PRS-CSx
Colorectum	PRS7	rs13314128	3	122535198	G	T	-7.19E-04	PRS-CSx
Colorectum	PRS7	rs1332100	10	101301346	T	C	-3.50E-04	PRS-CSx
Colorectum	PRS7	rs13321783	3	119615375	T	C	2.67E-04	PRS-CSx
Colorectum	PRS7	rs13321908	3	112923072	G	A	-1.37E-03	PRS-CSx
Colorectum	PRS7	rs1332492	6	131351278	T	C	1.10E-03	PRS-CSx
Colorectum	PRS7	rs1332548	10	20644777	C	T	-4.42E-05	PRS-CSx
Colorectum	PRS7	rs13328226	5	58571915	A	G	4.79E-04	PRS-CSx
Colorectum	PRS7	rs1333040	9	22083404	C	T	-1.42E-05	PRS-CSx
Colorectum	PRS7	rs1333042	9	22103813	A	G	2.04E-03	PRS-CSx
Colorectum	PRS7	rs1333048	9	22125347	A	C	5.79E-04	PRS-CSx
Colorectum	PRS7	rs1333050	9	22125913	C	T	2.06E-04	PRS-CSx
Colorectum	PRS7	rs13332134	16	1679439	T	C	3.03E-04	PRS-CSx
Colorectum	PRS7	rs13333528	16	68790502	T	C	-7.91E-04	PRS-CSx
Colorectum	PRS7	rs13333795	16	80063745	A	G	7.58E-05	PRS-CSx
Colorectum	PRS7	rs1333606	9	31189305	A	G	-1.43E-04	PRS-CSx
Colorectum	PRS7	rs133380	22	42468910	T	C	2.53E-04	PRS-CSx
Colorectum	PRS7	rs13344547	19	41945848	G	T	7.73E-05	PRS-CSx
Colorectum	PRS7	rs1334538	10	8843949	A	C	4.49E-04	PRS-CSx
Colorectum	PRS7	rs1334553	10	8693169	C	T	5.27E-04	PRS-CSx
Colorectum	PRS7	rs1334598	10	72351746	C	A	7.97E-04	PRS-CSx
Colorectum	PRS7	rs1335405	9	119993022	T	C	-1.12E-03	PRS-CSx
Colorectum	PRS7	rs13355305	5	40700139	G	T	1.69E-04	PRS-CSx
Colorectum	PRS7	rs13355881	5	75837834	C	T	2.90E-04	PRS-CSx
Colorectum	PRS7	rs13357117	5	143656916	T	G	-3.08E-04	PRS-CSx
Colorectum	PRS7	rs13358455	5	127365786	T	C	-7.64E-04	PRS-CSx
Colorectum	PRS7	rs13359961	5	129356852	G	A	-2.67E-04	PRS-CSx
Colorectum	PRS7	rs1337285	13	74089256	T	C	-2.77E-05	PRS-CSx
Colorectum	PRS7	rs1337286	13	74089246	G	A	7.46E-05	PRS-CSx
Colorectum	PRS7	rs1337577	9	33756998	T	G	-1.48E-04	PRS-CSx
Colorectum	PRS7	rs1337706	9	104555316	C	T	-5.57E-04	PRS-CSx
Colorectum	PRS7	rs13379229	14	66073711	G	A	9.00E-04	PRS-CSx
Colorectum	PRS7	rs1337937	13	109494871	T	C	6.03E-04	PRS-CSx
Colorectum	PRS7	rs13383278	2	207190048	C	T	-1.24E-04	PRS-CSx
Colorectum	PRS7	rs1338449	1	218351323	T	C	-6.75E-06	PRS-CSx
Colorectum	PRS7	rs13385754	2	199542357	C	T	2.36E-04	PRS-CSx
Colorectum	PRS7	rs13388224	2	33802902	C	T	4.76E-04	PRS-CSx
Colorectum	PRS7	rs13391166	2	33805314	G	A	7.76E-04	PRS-CSx
Colorectum	PRS7	rs13393754	2	199518159	T	C	2.41E-04	PRS-CSx
Colorectum	PRS7	rs13399908	2	67306158	A	G	-2.76E-04	PRS-CSx
Colorectum	PRS7	rs1340130	10	16303877	C	T	2.84E-03	PRS-CSx
Colorectum	PRS7	rs1340363	10	73179616	A	G	1.62E-04	PRS-CSx
Colorectum	PRS7	rs13403634	2	1771963	A	G	1.23E-03	PRS-CSx
Colorectum	PRS7	rs13405122	2	67302866	T	C	-6.29E-04	PRS-CSx
Colorectum	PRS7	rs13406110	2	71685529	T	C	-3.71E-04	PRS-CSx
Colorectum	PRS7	rs1340695	6	24316195	A	C	3.10E-04	PRS-CSx

Colorectum	PRS7	rs13409097	2	29240005	T	C	-8.03E-04	PRS-CSx
Colorectum	PRS7	rs13409196	2	107386238	A	G	4.72E-04	PRS-CSx
Colorectum	PRS7	rs13409622	2	159816803	A	G	-9.24E-04	PRS-CSx
Colorectum	PRS7	rs13411945	2	107265058	G	A	-7.66E-04	PRS-CSx
Colorectum	PRS7	rs13412722	2	101646749	A	G	4.93E-04	PRS-CSx
Colorectum	PRS7	rs13414916	2	159954420	T	C	-2.26E-04	PRS-CSx
Colorectum	PRS7	rs134157	22	26916722	C	T	4.28E-04	PRS-CSx
Colorectum	PRS7	rs13416955	2	177115852	T	C	9.01E-04	PRS-CSx
Colorectum	PRS7	rs134176	22	28624319	T	C	1.63E-04	PRS-CSx
Colorectum	PRS7	rs13418410	2	33800899	A	C	4.54E-04	PRS-CSx
Colorectum	PRS7	rs1342310	10	56203741	G	A	1.19E-03	PRS-CSx
Colorectum	PRS7	rs13424337	2	182629582	G	A	-8.04E-04	PRS-CSx
Colorectum	PRS7	rs13424541	2	71625767	A	G	-3.62E-04	PRS-CSx
Colorectum	PRS7	rs1342455	10	90214051	A	C	-9.22E-04	PRS-CSx
Colorectum	PRS7	rs1342586	1	218597859	C	T	-6.03E-04	PRS-CSx
Colorectum	PRS7	rs13426349	2	200198964	C	T	-2.77E-04	PRS-CSx
Colorectum	PRS7	rs13426764	2	199543380	T	C	2.93E-04	PRS-CSx
Colorectum	PRS7	rs13427625	2	157477855	C	T	5.32E-03	PRS-CSx
Colorectum	PRS7	rs13428951	2	123389473	T	C	-1.21E-03	PRS-CSx
Colorectum	PRS7	rs13429266	2	66228539	A	C	4.08E-03	PRS-CSx
Colorectum	PRS7	rs13436401	5	88338622	T	C	4.32E-04	PRS-CSx
Colorectum	PRS7	rs13436926	5	35825173	T	C	4.47E-04	PRS-CSx
Colorectum	PRS7	rs1343778	1	90158592	C	A	3.92E-04	PRS-CSx
Colorectum	PRS7	rs1343779	1	90158719	C	T	5.27E-04	PRS-CSx
Colorectum	PRS7	rs1344583	15	96106002	A	G	1.05E-03	PRS-CSx
Colorectum	PRS7	rs134529	22	28781758	C	T	4.19E-04	PRS-CSx
Colorectum	PRS7	rs1345778	5	40712797	A	C	4.16E-04	PRS-CSx
Colorectum	PRS7	rs1346214	4	139387890	T	C	-7.88E-04	PRS-CSx
Colorectum	PRS7	rs1346441	5	134530116	A	G	3.27E-05	PRS-CSx
Colorectum	PRS7	rs1346452	5	88387127	C	T	4.23E-04	PRS-CSx
Colorectum	PRS7	rs1346655	20	22394312	T	C	5.94E-04	PRS-CSx
Colorectum	PRS7	rs1346715	4	126535676	C	T	-7.93E-04	PRS-CSx
Colorectum	PRS7	rs1346775	2	183328892	G	A	1.09E-04	PRS-CSx
Colorectum	PRS7	rs1347626	8	117656781	T	C	-6.06E-05	PRS-CSx
Colorectum	PRS7	rs1348812	2	200236425	A	G	-2.23E-05	PRS-CSx
Colorectum	PRS7	rs1349348	10	111794554	T	C	-1.71E-04	PRS-CSx
Colorectum	PRS7	rs134938	22	27432357	A	G	-6.23E-04	PRS-CSx
Colorectum	PRS7	rs134943	22	27519787	G	T	4.86E-04	PRS-CSx
Colorectum	PRS7	rs1349951	15	47533478	A	G	-3.53E-04	PRS-CSx
Colorectum	PRS7	rs135001	22	43492639	A	C	1.90E-04	PRS-CSx
Colorectum	PRS7	rs135002	22	43492962	T	C	2.90E-04	PRS-CSx
Colorectum	PRS7	rs135014	22	43497898	G	A	3.41E-05	PRS-CSx
Colorectum	PRS7	rs1351889	17	53148172	T	C	-1.03E-03	PRS-CSx
Colorectum	PRS7	rs1352609	5	57321413	G	A	2.68E-04	PRS-CSx
Colorectum	PRS7	rs1352610	5	57321586	A	G	2.43E-04	PRS-CSx
Colorectum	PRS7	rs1352900	12	20005490	C	T	5.46E-04	PRS-CSx
Colorectum	PRS7	rs1352902	12	19990847	A	G	3.27E-04	PRS-CSx
Colorectum	PRS7	rs1353251	5	35857207	C	T	3.14E-04	PRS-CSx
Colorectum	PRS7	rs1354096	5	8613262	G	T	4.89E-04	PRS-CSx
Colorectum	PRS7	rs1354106	19	51737991	G	T	7.91E-04	PRS-CSx
Colorectum	PRS7	rs1354738	15	48883696	C	T	5.68E-04	PRS-CSx
Colorectum	PRS7	rs1355469	12	59430303	G	A	-4.33E-04	PRS-CSx
Colorectum	PRS7	rs1356003	12	101235646	A	G	-2.73E-04	PRS-CSx
Colorectum	PRS7	rs1356362	3	152354325	A	G	3.11E-04	PRS-CSx
Colorectum	PRS7	rs1356485	2	199571750	C	T	3.51E-04	PRS-CSx
Colorectum	PRS7	rs1358963	6	131605135	A	G	8.56E-04	PRS-CSx

Colorectum	PRS7	rs1359198	13	94061490	T	C	5.48E-04	PRS-CSx
Colorectum	PRS7	rs1359484	6	164569414	A	G	-4.84E-04	PRS-CSx
Colorectum	PRS7	rs136027	22	46229951	T	C	-1.57E-03	PRS-CSx
Colorectum	PRS7	rs1360660	9	31206171	G	A	-1.14E-04	PRS-CSx
Colorectum	PRS7	rs1360661	9	31206286	G	T	-1.21E-04	PRS-CSx
Colorectum	PRS7	rs1360686	9	101823327	A	G	-8.69E-04	PRS-CSx
Colorectum	PRS7	rs1360704	1	183099380	T	C	6.05E-04	PRS-CSx
Colorectum	PRS7	rs1360879	9	139643023	A	G	-1.09E-03	PRS-CSx
Colorectum	PRS7	rs1361057	13	74055303	C	T	-4.53E-04	PRS-CSx
Colorectum	PRS7	rs1361058	13	74055233	C	T	4.70E-04	PRS-CSx
Colorectum	PRS7	rs1361514	20	17112688	T	C	5.04E-04	PRS-CSx
Colorectum	PRS7	rs136229	22	31214572	C	T	-7.82E-04	PRS-CSx
Colorectum	PRS7	rs136230	22	31214382	G	A	-9.24E-04	PRS-CSx
Colorectum	PRS7	rs1362970	12	96052526	A	C	-1.22E-03	PRS-CSx
Colorectum	PRS7	rs1362971	12	96075523	A	G	-5.09E-04	PRS-CSx
Colorectum	PRS7	rs1363024	11	100029698	G	A	8.18E-04	PRS-CSx
Colorectum	PRS7	rs1363220	5	143822828	G	A	-7.42E-05	PRS-CSx
Colorectum	PRS7	rs1365196	11	125158049	G	A	4.82E-04	PRS-CSx
Colorectum	PRS7	rs1365907	2	159865053	T	G	6.91E-05	PRS-CSx
Colorectum	PRS7	rs1366020	5	40139081	C	T	3.64E-04	PRS-CSx
Colorectum	PRS7	rs1367203	2	183311523	G	A	2.88E-04	PRS-CSx
Colorectum	PRS7	rs1367950	17	3618298	T	C	4.59E-04	PRS-CSx
Colorectum	PRS7	rs1367980	11	83340398	G	A	7.87E-05	PRS-CSx
Colorectum	PRS7	rs1370666	2	183208082	A	G	3.79E-04	PRS-CSx
Colorectum	PRS7	rs1370668	2	183178743	A	G	-1.38E-04	PRS-CSx
Colorectum	PRS7	rs1371404	2	43040577	C	A	3.58E-04	PRS-CSx
Colorectum	PRS7	rs1371867	8	101330209	C	A	-2.14E-04	PRS-CSx
Colorectum	PRS7	rs1372807	11	10138299	A	G	-1.80E-04	PRS-CSx
Colorectum	PRS7	rs1373345	18	63199565	T	C	-2.07E-03	PRS-CSx
Colorectum	PRS7	rs1373356	12	59425354	G	A	-3.78E-04	PRS-CSx
Colorectum	PRS7	rs1374357	2	200201605	A	G	-1.81E-04	PRS-CSx
Colorectum	PRS7	rs1377145	4	19070123	G	A	6.40E-04	PRS-CSx
Colorectum	PRS7	rs1377151	4	18922884	A	C	-3.98E-04	PRS-CSx
Colorectum	PRS7	rs1377153	4	18923066	G	A	-3.16E-04	PRS-CSx
Colorectum	PRS7	rs1378646	7	136635001	T	C	-6.61E-04	PRS-CSx
Colorectum	PRS7	rs1378703	8	29100294	T	C	5.94E-04	PRS-CSx
Colorectum	PRS7	rs1378954	9	141005656	G	A	8.31E-04	PRS-CSx
Colorectum	PRS7	rs1379346	15	37779636	T	C	-6.75E-04	PRS-CSx
Colorectum	PRS7	rs1379809	4	69703527	A	G	1.94E-04	PRS-CSx
Colorectum	PRS7	rs1379932	4	89800892	T	C	1.64E-04	PRS-CSx
Colorectum	PRS7	rs1380396	5	100737045	C	A	9.50E-04	PRS-CSx
Colorectum	PRS7	rs1380992	3	191865260	A	G	1.17E-03	PRS-CSx
Colorectum	PRS7	rs1381837	3	110611201	T	C	-4.22E-04	PRS-CSx
Colorectum	PRS7	rs13831	20	57475191	A	G	-1.70E-03	PRS-CSx
Colorectum	PRS7	rs138601	22	44975213	T	C	1.10E-03	PRS-CSx
Colorectum	PRS7	rs1387353	1	164414741	G	A	-1.78E-03	PRS-CSx
Colorectum	PRS7	rs1387355	1	164414695	G	A	-1.28E-03	PRS-CSx
Colorectum	PRS7	rs1387641	5	57334047	C	T	3.32E-04	PRS-CSx
Colorectum	PRS7	rs1388038	4	105867408	C	T	-1.55E-03	PRS-CSx
Colorectum	PRS7	rs1388201	5	100748138	C	T	2.14E-03	PRS-CSx
Colorectum	PRS7	rs1388837	4	163072871	G	T	-4.21E-04	PRS-CSx
Colorectum	PRS7	rs1388840	4	163073169	G	A	-3.65E-04	PRS-CSx
Colorectum	PRS7	rs1388886	4	167412572	T	C	6.76E-04	PRS-CSx
Colorectum	PRS7	rs1389450	5	39826683	G	T	2.32E-04	PRS-CSx
Colorectum	PRS7	rs1390766	3	60242243	G	T	-6.04E-04	PRS-CSx
Colorectum	PRS7	rs1391801	1	88944445	C	T	1.16E-03	PRS-CSx

Colorectum	PRS7	rs1392962	5	8599488	G	T	2.75E-04	PRS-CSx
Colorectum	PRS7	rs1394888	13	92464226	T	C	2.82E-04	PRS-CSx
Colorectum	PRS7	rs1395484	16	76459974	T	C	4.79E-04	PRS-CSx
Colorectum	PRS7	rs1395820	4	148032488	G	A	-4.25E-04	PRS-CSx
Colorectum	PRS7	rs1395821	4	148047550	C	T	-7.04E-04	PRS-CSx
Colorectum	PRS7	rs1397157	16	86305265	T	C	-9.09E-04	PRS-CSx
Colorectum	PRS7	rs1398942	4	89930392	A	G	4.51E-04	PRS-CSx
Colorectum	PRS7	rs139909	22	40697581	T	C	-1.85E-04	PRS-CSx
Colorectum	PRS7	rs1400611	3	152421205	C	T	1.40E-04	PRS-CSx
Colorectum	PRS7	rs1400671	3	5992687	C	T	-8.74E-04	PRS-CSx
Colorectum	PRS7	rs1400875	1	201821443	C	T	5.13E-04	PRS-CSx
Colorectum	PRS7	rs1402304	3	106495324	A	G	-8.86E-04	PRS-CSx
Colorectum	PRS7	rs1403180	7	96562958	A	G	-7.08E-04	PRS-CSx
Colorectum	PRS7	rs140489	22	21921294	G	A	1.24E-04	PRS-CSx
Colorectum	PRS7	rs140491	22	21922364	T	C	2.23E-04	PRS-CSx
Colorectum	PRS7	rs140492	22	21923144	A	C	1.73E-04	PRS-CSx
Colorectum	PRS7	rs140498	22	21927064	G	A	2.19E-04	PRS-CSx
Colorectum	PRS7	rs1405567	20	45186122	T	C	1.06E-03	PRS-CSx
Colorectum	PRS7	rs1405655	19	50882619	C	T	4.01E-04	PRS-CSx
Colorectum	PRS7	rs1406665	2	168479374	C	T	-7.30E-04	PRS-CSx
Colorectum	PRS7	rs1407033	20	6544657	G	A	8.48E-04	PRS-CSx
Colorectum	PRS7	rs1407038	20	6603820	A	G	1.01E-03	PRS-CSx
Colorectum	PRS7	rs1407039	20	6603948	A	G	1.40E-03	PRS-CSx
Colorectum	PRS7	rs1407184	6	117733717	A	G	1.76E-04	PRS-CSx
Colorectum	PRS7	rs1407220	6	123892432	G	A	-2.43E-03	PRS-CSx
Colorectum	PRS7	rs1407367	11	33177392	G	A	-2.49E-04	PRS-CSx
Colorectum	PRS7	rs1408582	10	91606480	T	G	-1.32E-03	PRS-CSx
Colorectum	PRS7	rs1408617	13	48428515	C	T	-1.95E-04	PRS-CSx
Colorectum	PRS7	rs1408977	6	40247122	G	A	9.25E-04	PRS-CSx
Colorectum	PRS7	rs1409248	13	58015993	A	G	3.55E-04	PRS-CSx
Colorectum	PRS7	rs1409550	9	121559990	T	G	6.41E-04	PRS-CSx
Colorectum	PRS7	rs1410619	13	70798566	A	G	-1.10E-04	PRS-CSx
Colorectum	PRS7	rs1411661	9	85097651	T	G	-3.78E-04	PRS-CSx
Colorectum	PRS7	rs1412622	1	62365217	T	C	1.54E-04	PRS-CSx
Colorectum	PRS7	rs1412829	9	22043926	G	A	5.21E-04	PRS-CSx
Colorectum	PRS7	rs1412832	9	22077543	C	T	-9.67E-05	PRS-CSx
Colorectum	PRS7	rs1413298	9	101823373	A	G	3.52E-04	PRS-CSx
Colorectum	PRS7	rs1413390	1	183096634	G	A	2.82E-04	PRS-CSx
Colorectum	PRS7	rs14134	3	101576175	C	T	6.37E-04	PRS-CSx
Colorectum	PRS7	rs1413678	10	8754677	C	T	2.44E-03	PRS-CSx
Colorectum	PRS7	rs1414402	9	133922072	T	C	-1.17E-03	PRS-CSx
Colorectum	PRS7	rs1415408	13	74088814	A	G	3.00E-05	PRS-CSx
Colorectum	PRS7	rs1415657	9	104574237	C	T	1.31E-03	PRS-CSx
Colorectum	PRS7	rs1416919	6	28302884	G	T	5.43E-05	PRS-CSx
Colorectum	PRS7	rs1416920	6	28302784	G	A	3.79E-04	PRS-CSx
Colorectum	PRS7	rs1417566	20	17117683	T	G	5.06E-04	PRS-CSx
Colorectum	PRS7	rs1417567	20	17117716	C	T	5.05E-04	PRS-CSx
Colorectum	PRS7	rs1417823	10	102227886	G	A	-6.70E-05	PRS-CSx
Colorectum	PRS7	rs1419183	6	28242794	C	A	-2.33E-05	PRS-CSx
Colorectum	PRS7	rs1419642	6	29392235	A	G	1.49E-05	PRS-CSx
Colorectum	PRS7	rs1419643	6	29391936	G	T	-5.42E-05	PRS-CSx
Colorectum	PRS7	rs1419958	2	230033560	G	T	5.97E-04	PRS-CSx
Colorectum	PRS7	rs1420371	2	67191073	T	C	1.28E-03	PRS-CSx
Colorectum	PRS7	rs1421050	10	113754544	G	T	5.09E-04	PRS-CSx
Colorectum	PRS7	rs1421247	8	15646857	T	C	-4.60E-04	PRS-CSx
Colorectum	PRS7	rs1421385	2	159994048	A	G	7.56E-04	PRS-CSx

Colorectum	PRS7	rs1421565	11	100008998	C	T	-1.19E-03	PRS-CSx
Colorectum	PRS7	rs1421761	5	134397724	T	C	-1.59E-06	PRS-CSx
Colorectum	PRS7	rs1421892	5	128220643	G	A	3.93E-04	PRS-CSx
Colorectum	PRS7	rs1422708	5	143629815	T	C	1.01E-04	PRS-CSx
Colorectum	PRS7	rs1423259	5	39901149	C	A	-3.53E-04	PRS-CSx
Colorectum	PRS7	rs1423348	5	40149877	G	A	2.23E-04	PRS-CSx
Colorectum	PRS7	rs1423369	5	58115159	A	G	7.97E-04	PRS-CSx
Colorectum	PRS7	rs14235	16	31121793	G	A	2.89E-04	PRS-CSx
Colorectum	PRS7	rs1423658	5	35887640	G	A	4.43E-04	PRS-CSx
Colorectum	PRS7	rs1424752	11	57479848	A	C	2.75E-04	PRS-CSx
Colorectum	PRS7	rs1424832	14	53702370	T	C	8.89E-04	PRS-CSx
Colorectum	PRS7	rs1425313	4	58500584	C	T	4.89E-04	PRS-CSx
Colorectum	PRS7	rs1425781	8	41314993	T	C	9.20E-04	PRS-CSx
Colorectum	PRS7	rs1426715	15	48741996	A	G	5.43E-04	PRS-CSx
Colorectum	PRS7	rs1426979	4	31358976	G	A	1.85E-03	PRS-CSx
Colorectum	PRS7	rs1427282	15	58914326	G	A	2.19E-04	PRS-CSx
Colorectum	PRS7	rs1427324	14	59364693	C	T	8.12E-05	PRS-CSx
Colorectum	PRS7	rs1427742	5	40136928	C	T	6.13E-04	PRS-CSx
Colorectum	PRS7	rs1427744	5	40154769	G	A	4.19E-04	PRS-CSx
Colorectum	PRS7	rs1427747	5	40151057	T	C	3.37E-04	PRS-CSx
Colorectum	PRS7	rs1428555	5	150257391	T	C	-6.46E-04	PRS-CSx
Colorectum	PRS7	rs1428638	5	118154368	T	C	-2.07E-03	PRS-CSx
Colorectum	PRS7	rs1428856	5	143712855	A	G	-3.00E-04	PRS-CSx
Colorectum	PRS7	rs1430145	2	183358906	C	T	2.32E-04	PRS-CSx
Colorectum	PRS7	rs1430150	2	183383288	T	G	3.22E-04	PRS-CSx
Colorectum	PRS7	rs1430220	2	222325598	C	T	7.95E-04	PRS-CSx
Colorectum	PRS7	rs1431900	2	168477472	C	T	-6.39E-04	PRS-CSx
Colorectum	PRS7	rs1432129	15	27232033	C	A	5.18E-04	PRS-CSx
Colorectum	PRS7	rs1432131	15	27228632	C	T	7.68E-04	PRS-CSx
Colorectum	PRS7	rs1432132	15	27228404	A	G	2.59E-04	PRS-CSx
Colorectum	PRS7	rs1432520	2	183092726	T	G	3.19E-04	PRS-CSx
Colorectum	PRS7	rs1432521	2	183092956	G	A	2.87E-04	PRS-CSx
Colorectum	PRS7	rs1433681	2	174338087	A	G	2.07E-03	PRS-CSx
Colorectum	PRS7	rs1434999	2	163627077	T	C	-5.67E-04	PRS-CSx
Colorectum	PRS7	rs1436091	10	78905738	G	A	-7.06E-05	PRS-CSx
Colorectum	PRS7	rs1437787	2	216318237	T	C	-1.03E-03	PRS-CSx
Colorectum	PRS7	rs1438065	2	183218993	A	G	-2.37E-04	PRS-CSx
Colorectum	PRS7	rs1438507	10	133108761	A	G	2.38E-03	PRS-CSx
Colorectum	PRS7	rs1439226	2	42513098	A	G	-8.54E-05	PRS-CSx
Colorectum	PRS7	rs1439644	2	43021749	C	T	4.87E-04	PRS-CSx
Colorectum	PRS7	rs1439716	2	77385284	C	T	5.62E-03	PRS-CSx
Colorectum	PRS7	rs1439920	1	48209873	A	G	2.60E-03	PRS-CSx
Colorectum	PRS7	rs1440372	15	67033151	T	C	-3.99E-04	PRS-CSx
Colorectum	PRS7	rs1440742	4	68752092	A	G	2.25E-05	PRS-CSx
Colorectum	PRS7	rs1440743	4	68751635	G	T	3.54E-05	PRS-CSx
Colorectum	PRS7	rs1441159	2	182581492	T	C	-1.06E-03	PRS-CSx
Colorectum	PRS7	rs1441355	15	71678178	T	C	3.63E-04	PRS-CSx
Colorectum	PRS7	rs1441383	11	99695545	A	G	-3.41E-03	PRS-CSx
Colorectum	PRS7	rs1441433	4	102209360	T	G	-2.90E-04	PRS-CSx
Colorectum	PRS7	rs1442459	1	221287861	A	C	1.32E-04	PRS-CSx
Colorectum	PRS7	rs1442464	1	221240099	C	A	1.98E-04	PRS-CSx
Colorectum	PRS7	rs1442466	1	221300523	C	T	9.18E-05	PRS-CSx
Colorectum	PRS7	rs1443007	18	27986202	G	A	1.58E-03	PRS-CSx
Colorectum	PRS7	rs1444043	4	55233465	T	C	-8.45E-04	PRS-CSx
Colorectum	PRS7	rs1444584	12	46476201	C	T	5.58E-04	PRS-CSx
Colorectum	PRS7	rs1444588	12	46464417	T	C	6.32E-04	PRS-CSx

Colorectum	PRS7	rs1445400	8	72246768	T	C	-5.62E-04	PRS-CSx
Colorectum	PRS7	rs1446099	2	137348398	A	G	1.55E-03	PRS-CSx
Colorectum	PRS7	rs1447294	8	128437686	T	C	5.50E-05	PRS-CSx
Colorectum	PRS7	rs1447657	3	125929471	G	A	1.30E-04	PRS-CSx
Colorectum	PRS7	rs1448753	12	78455040	C	T	-8.47E-04	PRS-CSx
Colorectum	PRS7	rs1448902	2	207178422	G	A	4.30E-04	PRS-CSx
Colorectum	PRS7	rs1449571	13	58032506	G	A	4.63E-04	PRS-CSx
Colorectum	PRS7	rs1449873	3	24278912	C	T	-1.77E-03	PRS-CSx
Colorectum	PRS7	rs1450144	4	183125630	T	C	-8.08E-04	PRS-CSx
Colorectum	PRS7	rs1452408	15	90891117	C	A	3.56E-04	PRS-CSx
Colorectum	PRS7	rs1452554	4	18920164	C	T	-4.70E-04	PRS-CSx
Colorectum	PRS7	rs1452556	4	18936647	G	A	3.57E-04	PRS-CSx
Colorectum	PRS7	rs1453997	11	21177159	T	C	-4.77E-04	PRS-CSx
Colorectum	PRS7	rs1454247	4	69341579	C	T	-1.83E-03	PRS-CSx
Colorectum	PRS7	rs1454924	5	57334825	C	T	1.41E-04	PRS-CSx
Colorectum	PRS7	rs1454925	5	57336494	G	A	1.60E-04	PRS-CSx
Colorectum	PRS7	rs1455101	11	16177318	A	G	7.73E-04	PRS-CSx
Colorectum	PRS7	rs1455335	2	199542718	T	C	3.76E-04	PRS-CSx
Colorectum	PRS7	rs1456655	1	240407163	C	T	9.07E-04	PRS-CSx
Colorectum	PRS7	rs1456657	1	240407307	C	T	8.47E-04	PRS-CSx
Colorectum	PRS7	rs1458236	4	69782895	C	T	-6.89E-05	PRS-CSx
Colorectum	PRS7	rs1458246	4	69818082	C	A	-8.53E-05	PRS-CSx
Colorectum	PRS7	rs1458562	4	89853598	C	T	2.68E-04	PRS-CSx
Colorectum	PRS7	rs1458992	14	81303497	G	A	-1.42E-03	PRS-CSx
Colorectum	PRS7	rs1459498	18	53999315	C	T	-1.64E-04	PRS-CSx
Colorectum	PRS7	rs1460475	4	21565822	G	A	8.73E-05	PRS-CSx
Colorectum	PRS7	rs1460770	4	115405345	A	C	6.46E-05	PRS-CSx
Colorectum	PRS7	rs1460775	4	115368816	T	C	2.99E-04	PRS-CSx
Colorectum	PRS7	rs1460784	4	115463103	G	A	1.84E-05	PRS-CSx
Colorectum	PRS7	rs1460930	8	101114794	C	T	-2.03E-04	PRS-CSx
Colorectum	PRS7	rs1461729	8	9187242	A	G	-1.63E-03	PRS-CSx
Colorectum	PRS7	rs1462622	4	64238559	G	A	5.87E-04	PRS-CSx
Colorectum	PRS7	rs1462807	3	110645112	C	A	-1.72E-04	PRS-CSx
Colorectum	PRS7	rs1463644	3	113197393	C	A	2.26E-04	PRS-CSx
Colorectum	PRS7	rs1464114	3	157549569	A	G	-3.10E-04	PRS-CSx
Colorectum	PRS7	rs1464369	12	78179248	A	G	4.66E-04	PRS-CSx
Colorectum	PRS7	rs1465543	12	116020922	G	A	-3.76E-04	PRS-CSx
Colorectum	PRS7	rs1466649	2	66450306	G	T	3.53E-04	PRS-CSx
Colorectum	PRS7	rs1466862	5	36791812	G	A	-1.44E-04	PRS-CSx
Colorectum	PRS7	rs1467513	1	15894959	T	C	-4.95E-04	PRS-CSx
Colorectum	PRS7	rs1468052	6	134009694	G	T	4.08E-04	PRS-CSx
Colorectum	PRS7	rs1468603	12	6427052	T	C	1.76E-03	PRS-CSx
Colorectum	PRS7	rs1468889	10	101308656	G	A	-3.73E-04	PRS-CSx
Colorectum	PRS7	rs1469531	4	39088980	A	C	-3.37E-05	PRS-CSx
Colorectum	PRS7	rs1470569	3	77531926	G	A	4.26E-05	PRS-CSx
Colorectum	PRS7	rs1471294	8	101117554	T	G	-7.93E-05	PRS-CSx
Colorectum	PRS7	rs1471884	3	113212041	A	G	-8.53E-05	PRS-CSx
Colorectum	PRS7	rs1472974	11	34775452	T	C	-5.24E-04	PRS-CSx
Colorectum	PRS7	rs1473248	19	41923314	C	T	2.09E-04	PRS-CSx
Colorectum	PRS7	rs1473364	4	39212879	T	C	1.32E-04	PRS-CSx
Colorectum	PRS7	rs1473736	14	54556533	A	C	5.68E-04	PRS-CSx
Colorectum	PRS7	rs1473766	13	34128261	T	C	-2.16E-04	PRS-CSx
Colorectum	PRS7	rs1473953	22	45718041	A	G	-4.26E-04	PRS-CSx
Colorectum	PRS7	rs1474630	6	144619627	A	C	-1.34E-03	PRS-CSx
Colorectum	PRS7	rs1474911	6	85559568	C	T	1.04E-03	PRS-CSx
Colorectum	PRS7	rs1476046	6	12293221	A	G	-1.56E-03	PRS-CSx

Colorectum	PRS7	rs1476572	6	29674741	G	T	-3.30E-06	PRS-CSx
Colorectum	PRS7	rs1476705	19	3610188	C	T	7.88E-04	PRS-CSx
Colorectum	PRS7	rs1477917	8	89129953	C	T	-7.09E-05	PRS-CSx
Colorectum	PRS7	rs1478198	15	54156432	G	T	-1.63E-03	PRS-CSx
Colorectum	PRS7	rs1478507	16	80102633	T	C	1.29E-04	PRS-CSx
Colorectum	PRS7	rs1478975	8	83300429	G	A	5.59E-04	PRS-CSx
Colorectum	PRS7	rs1482189	8	122464572	C	T	-2.20E-03	PRS-CSx
Colorectum	PRS7	rs1482704	4	60529334	G	A	-5.02E-04	PRS-CSx
Colorectum	PRS7	rs1484264	4	27862340	T	C	-3.21E-05	PRS-CSx
Colorectum	PRS7	rs1484266	4	27874225	G	A	-5.09E-05	PRS-CSx
Colorectum	PRS7	rs1485004	7	95270506	G	A	3.82E-04	PRS-CSx
Colorectum	PRS7	rs1486147	7	46236053	C	T	-3.93E-04	PRS-CSx
Colorectum	PRS7	rs1486169	7	46230743	A	G	-5.16E-04	PRS-CSx
Colorectum	PRS7	rs1486404	12	116159774	G	A	2.44E-04	PRS-CSx
Colorectum	PRS7	rs1488553	3	173574517	T	C	-2.90E-04	PRS-CSx
Colorectum	PRS7	rs1489381	6	75015727	A	G	-1.70E-04	PRS-CSx
Colorectum	PRS7	rs1490586	4	105890017	C	A	3.25E-04	PRS-CSx
Colorectum	PRS7	rs1491373	4	23688589	A	G	3.81E-05	PRS-CSx
Colorectum	PRS7	rs149245	17	3579652	T	C	2.87E-04	PRS-CSx
Colorectum	PRS7	rs1492464	4	163079268	A	G	5.85E-04	PRS-CSx
Colorectum	PRS7	rs1492592	9	105490129	T	C	2.83E-04	PRS-CSx
Colorectum	PRS7	rs1493428	12	77326330	T	G	-9.18E-04	PRS-CSx
Colorectum	PRS7	rs149462	20	62223238	T	C	2.47E-04	PRS-CSx
Colorectum	PRS7	rs1494641	5	38041550	C	T	1.95E-04	PRS-CSx
Colorectum	PRS7	rs1494978	4	134947801	G	A	7.50E-04	PRS-CSx
Colorectum	PRS7	rs1495598	3	78106208	A	G	1.14E-03	PRS-CSx
Colorectum	PRS7	rs149592	17	3578122	A	C	4.03E-04	PRS-CSx
Colorectum	PRS7	rs1496162	11	15003073	G	A	2.86E-04	PRS-CSx
Colorectum	PRS7	rs1497675	1	104820780	C	T	2.04E-04	PRS-CSx
Colorectum	PRS7	rs1497676	1	104820635	T	C	1.79E-04	PRS-CSx
Colorectum	PRS7	rs1497732	16	86191464	C	T	3.37E-04	PRS-CSx
Colorectum	PRS7	rs1498754	12	115933207	C	T	5.29E-04	PRS-CSx
Colorectum	PRS7	rs1498914	1	88868236	A	G	-6.77E-04	PRS-CSx
Colorectum	PRS7	rs1499236	5	39698509	T	C	-4.06E-04	PRS-CSx
Colorectum	PRS7	rs1499280	5	52096889	C	A	-1.53E-03	PRS-CSx
Colorectum	PRS7	rs1499369	8	129143680	A	G	6.39E-04	PRS-CSx
Colorectum	PRS7	rs1499417	8	118257185	T	G	3.63E-04	PRS-CSx
Colorectum	PRS7	rs1499899	3	112911260	G	A	4.65E-04	PRS-CSx
Colorectum	PRS7	rs1500023	12	59536329	G	A	-4.39E-04	PRS-CSx
Colorectum	PRS7	rs1500033	12	59520330	G	T	-3.20E-04	PRS-CSx
Colorectum	PRS7	rs1500545	20	6714108	C	T	4.45E-03	PRS-CSx
Colorectum	PRS7	rs1500794	3	37165542	G	A	-3.02E-04	PRS-CSx
Colorectum	PRS7	rs1501320	5	8590980	C	T	3.72E-04	PRS-CSx
Colorectum	PRS7	rs1501841	5	60947483	A	G	4.30E-04	PRS-CSx
Colorectum	PRS7	rs1502567	3	2428189	T	C	-1.62E-03	PRS-CSx
Colorectum	PRS7	rs1505387	4	165363909	A	C	-2.47E-04	PRS-CSx
Colorectum	PRS7	rs1505552	1	99549327	C	T	-6.61E-04	PRS-CSx
Colorectum	PRS7	rs1505553	1	99549346	A	G	-7.34E-04	PRS-CSx
Colorectum	PRS7	rs1506171	5	40048051	T	C	1.80E-04	PRS-CSx
Colorectum	PRS7	rs1506175	5	40135498	T	C	-2.44E-04	PRS-CSx
Colorectum	PRS7	rs1506176	5	40135704	A	G	5.77E-04	PRS-CSx
Colorectum	PRS7	rs1506187	5	40018216	T	G	1.09E-04	PRS-CSx
Colorectum	PRS7	rs1506833	16	76443174	C	T	3.92E-04	PRS-CSx
Colorectum	PRS7	rs1507534	11	13863404	T	G	-2.47E-04	PRS-CSx
Colorectum	PRS7	rs150847	17	3574289	T	C	3.97E-04	PRS-CSx
Colorectum	PRS7	rs1509689	1	239254075	T	C	-4.44E-04	PRS-CSx

Colorectum	PRS7	rs1510597	3	168425733	T	G	-4.82E-04	PRS-CSx
Colorectum	PRS7	rs1511174	3	170075833	C	T	-7.08E-04	PRS-CSx
Colorectum	PRS7	rs1511470	6	23868464	G	A	-5.63E-04	PRS-CSx
Colorectum	PRS7	rs1511471	6	23868594	A	G	-3.30E-04	PRS-CSx
Colorectum	PRS7	rs1511530	3	73769858	G	A	-1.59E-04	PRS-CSx
Colorectum	PRS7	rs1511532	3	73735674	T	C	-2.15E-04	PRS-CSx
Colorectum	PRS7	rs1511770	18	42030763	A	G	2.68E-04	PRS-CSx
Colorectum	PRS7	rs1513098	12	71151880	A	G	-4.42E-04	PRS-CSx
Colorectum	PRS7	rs1513431	3	168564812	A	C	-4.12E-04	PRS-CSx
Colorectum	PRS7	rs1515896	2	182976208	A	G	1.44E-04	PRS-CSx
Colorectum	PRS7	rs1515948	2	26097607	A	G	-3.23E-04	PRS-CSx
Colorectum	PRS7	rs1517328	2	169002104	A	G	-2.35E-04	PRS-CSx
Colorectum	PRS7	rs1517397	2	72189250	C	T	9.57E-04	PRS-CSx
Colorectum	PRS7	rs1517661	12	43131533	T	G	-2.83E-03	PRS-CSx
Colorectum	PRS7	rs151769	16	11340579	A	G	9.71E-04	PRS-CSx
Colorectum	PRS7	rs1517810	1	201850057	C	T	6.99E-04	PRS-CSx
Colorectum	PRS7	rs1518070	2	199653885	A	C	5.80E-04	PRS-CSx
Colorectum	PRS7	rs1518075	2	199644913	T	C	3.40E-04	PRS-CSx
Colorectum	PRS7	rs1519411	11	23680789	A	G	-2.18E-03	PRS-CSx
Colorectum	PRS7	rs1519813	8	121060884	T	C	-4.09E-04	PRS-CSx
Colorectum	PRS7	rs1520173	12	116182525	A	G	8.38E-04	PRS-CSx
Colorectum	PRS7	rs1520711	3	106493366	T	C	-1.11E-03	PRS-CSx
Colorectum	PRS7	rs1520725	12	78187017	T	G	4.20E-04	PRS-CSx
Colorectum	PRS7	rs1520726	12	78188510	T	C	3.60E-04	PRS-CSx
Colorectum	PRS7	rs1520733	12	78217340	A	G	2.64E-04	PRS-CSx
Colorectum	PRS7	rs152266	5	150635429	A	G	-5.60E-04	PRS-CSx
Colorectum	PRS7	rs152269	5	141140597	C	A	5.68E-04	PRS-CSx
Colorectum	PRS7	rs1522695	2	159788547	T	C	-2.86E-04	PRS-CSx
Colorectum	PRS7	rs1522696	2	159788585	T	C	-2.38E-04	PRS-CSx
Colorectum	PRS7	rs1522697	2	159788685	A	C	-1.12E-04	PRS-CSx
Colorectum	PRS7	rs1522983	3	168511943	C	A	-3.85E-04	PRS-CSx
Colorectum	PRS7	rs1523284	3	153355389	T	C	7.97E-04	PRS-CSx
Colorectum	PRS7	rs1523463	3	64883314	A	G	6.45E-05	PRS-CSx
Colorectum	PRS7	rs1523759	3	77604242	G	A	-1.39E-04	PRS-CSx
Colorectum	PRS7	rs1525233	7	46856454	C	T	-1.13E-03	PRS-CSx
Colorectum	PRS7	rs1525645	7	45598469	T	C	4.06E-04	PRS-CSx
Colorectum	PRS7	rs1525756	7	154607817	C	T	2.18E-04	PRS-CSx
Colorectum	PRS7	rs152621	5	14583006	A	G	-2.67E-04	PRS-CSx
Colorectum	PRS7	rs1526620	2	76516021	T	C	-7.02E-04	PRS-CSx
Colorectum	PRS7	rs1526963	12	79822067	C	T	5.05E-04	PRS-CSx
Colorectum	PRS7	rs1527790	12	78123770	G	A	-6.35E-04	PRS-CSx
Colorectum	PRS7	rs152788	5	14689707	G	A	-4.08E-04	PRS-CSx
Colorectum	PRS7	rs1529267	2	36464050	C	T	5.71E-04	PRS-CSx
Colorectum	PRS7	rs1529289	2	124915643	T	G	-5.03E-04	PRS-CSx
Colorectum	PRS7	rs1529943	8	117624483	T	G	1.29E-03	PRS-CSx
Colorectum	PRS7	rs1529958	19	58899466	A	G	6.52E-04	PRS-CSx
Colorectum	PRS7	rs1530104	2	124922297	G	A	-4.81E-04	PRS-CSx
Colorectum	PRS7	rs1530477	3	143819506	T	C	5.58E-04	PRS-CSx
Colorectum	PRS7	rs1530478	3	143819553	T	C	5.41E-04	PRS-CSx
Colorectum	PRS7	rs1530500	19	37823811	A	G	-9.31E-04	PRS-CSx
Colorectum	PRS7	rs153052	16	69446526	A	G	5.48E-04	PRS-CSx
Colorectum	PRS7	rs1531111	2	197184321	C	T	-1.14E-04	PRS-CSx
Colorectum	PRS7	rs1531827	14	57204584	G	T	8.97E-04	PRS-CSx
Colorectum	PRS7	rs1532471	16	86185911	A	G	5.28E-04	PRS-CSx
Colorectum	PRS7	rs1532472	16	86185638	A	G	2.59E-04	PRS-CSx
Colorectum	PRS7	rs1532593	3	112933303	G	A	1.39E-03	PRS-CSx

Colorectum	PRS7	rs1533086	7	47512042	C	T	-5.86E-03	PRS-CSx
Colorectum	PRS7	rs1533310	4	156896345	T	C	9.13E-04	PRS-CSx
Colorectum	PRS7	rs1533535	2	199622708	T	C	4.59E-04	PRS-CSx
Colorectum	PRS7	rs1533827	7	2074290	T	C	-5.31E-04	PRS-CSx
Colorectum	PRS7	rs1533829	7	2082841	G	A	-4.20E-04	PRS-CSx
Colorectum	PRS7	rs1533971	2	36136638	A	G	-2.89E-04	PRS-CSx
Colorectum	PRS7	rs1535	11	61597972	A	G	6.95E-04	PRS-CSx
Colorectum	PRS7	rs1535799	13	28779395	G	T	-4.74E-04	PRS-CSx
Colorectum	PRS7	rs1536168	1	1241529	A	G	5.51E-04	PRS-CSx
Colorectum	PRS7	rs1537370	9	22084310	C	T	2.42E-04	PRS-CSx
Colorectum	PRS7	rs1537375	9	22116071	T	C	1.85E-03	PRS-CSx
Colorectum	PRS7	rs1537504	9	101829542	G	A	2.18E-04	PRS-CSx
Colorectum	PRS7	rs1537506	9	101821169	T	C	-4.34E-04	PRS-CSx
Colorectum	PRS7	rs1537520	1	183084229	C	T	3.67E-04	PRS-CSx
Colorectum	PRS7	rs1537603	10	8734295	T	C	-4.88E-03	PRS-CSx
Colorectum	PRS7	rs1538853	13	51697806	C	A	-1.35E-04	PRS-CSx
Colorectum	PRS7	rs1539089	10	102221424	G	A	-4.54E-04	PRS-CSx
Colorectum	PRS7	rs1540140	11	15100206	A	G	1.87E-04	PRS-CSx
Colorectum	PRS7	rs1540148	11	15095130	G	T	1.51E-04	PRS-CSx
Colorectum	PRS7	rs1540774	3	152360283	T	C	2.95E-05	PRS-CSx
Colorectum	PRS7	rs1541213	10	104885330	G	A	-1.75E-04	PRS-CSx
Colorectum	PRS7	rs1541243	1	164875964	C	T	5.24E-04	PRS-CSx
Colorectum	PRS7	rs1541450	5	129385674	A	G	-1.21E-04	PRS-CSx
Colorectum	PRS7	rs1541947	2	80187452	C	T	8.47E-04	PRS-CSx
Colorectum	PRS7	rs154268	5	40795868	C	T	1.15E-04	PRS-CSx
Colorectum	PRS7	rs154275	5	40778641	C	T	6.13E-05	PRS-CSx
Colorectum	PRS7	rs1543016	1	239255612	A	G	-2.87E-04	PRS-CSx
Colorectum	PRS7	rs1543070	3	73749237	T	C	-2.14E-04	PRS-CSx
Colorectum	PRS7	rs1543071	3	73749316	C	A	-2.81E-04	PRS-CSx
Colorectum	PRS7	rs1543350	6	56212433	G	A	-4.36E-04	PRS-CSx
Colorectum	PRS7	rs1543604	9	34040797	C	A	-4.16E-04	PRS-CSx
Colorectum	PRS7	rs1544396	12	112062875	T	C	2.37E-04	PRS-CSx
Colorectum	PRS7	rs1545127	3	97315448	G	A	-9.69E-05	PRS-CSx
Colorectum	PRS7	rs1545157	10	9258696	C	T	-2.52E-04	PRS-CSx
Colorectum	PRS7	rs1545812	18	60832041	G	A	8.12E-04	PRS-CSx
Colorectum	PRS7	rs1546093	4	163084036	G	A	5.55E-04	PRS-CSx
Colorectum	PRS7	rs1546321	6	54704616	A	G	-1.75E-03	PRS-CSx
Colorectum	PRS7	rs1546424	15	49823227	C	T	4.81E-04	PRS-CSx
Colorectum	PRS7	rs1547714	1	183114119	C	T	3.33E-04	PRS-CSx
Colorectum	PRS7	rs1547715	1	183113952	A	G	1.96E-04	PRS-CSx
Colorectum	PRS7	rs1547786	6	125678114	G	A	-7.44E-04	PRS-CSx
Colorectum	PRS7	rs1549293	16	31141993	C	T	2.62E-04	PRS-CSx
Colorectum	PRS7	rs1549338	12	116162616	G	A	-9.68E-07	PRS-CSx
Colorectum	PRS7	rs1549760	7	150755839	T	C	-1.65E-04	PRS-CSx
Colorectum	PRS7	rs1550186	17	75703690	G	A	-3.06E-04	PRS-CSx
Colorectum	PRS7	rs1550656	17	10707285	C	T	-7.72E-04	PRS-CSx
Colorectum	PRS7	rs1550816	5	60847019	C	T	-1.10E-03	PRS-CSx
Colorectum	PRS7	rs1550856	8	117805397	A	G	1.32E-03	PRS-CSx
Colorectum	PRS7	rs1551184	11	134085771	G	A	-4.27E-04	PRS-CSx
Colorectum	PRS7	rs1551545	8	59227309	A	C	3.09E-03	PRS-CSx
Colorectum	PRS7	rs1551740	4	115377305	C	T	-1.35E-04	PRS-CSx
Colorectum	PRS7	rs1551742	4	115407050	C	T	3.52E-05	PRS-CSx
Colorectum	PRS7	rs1551744	11	46865000	A	G	1.49E-04	PRS-CSx
Colorectum	PRS7	rs1552568	6	75035349	T	G	-5.59E-04	PRS-CSx
Colorectum	PRS7	rs1552741	14	47344432	C	T	-5.83E-04	PRS-CSx
Colorectum	PRS7	rs1553191	3	112936609	C	T	-5.74E-04	PRS-CSx

Colorectum	PRS7	rs1553291	1	3209923	G	A	2.28E-03	PRS-CSx
Colorectum	PRS7	rs1554003	4	90038477	T	C	2.87E-04	PRS-CSx
Colorectum	PRS7	rs1554074	12	101240350	A	G	-2.80E-04	PRS-CSx
Colorectum	PRS7	rs1555318	20	345617	T	C	3.98E-03	PRS-CSx
Colorectum	PRS7	rs1555457	9	117674320	T	C	1.41E-03	PRS-CSx
Colorectum	PRS7	rs1555869	10	95520031	G	A	-3.11E-04	PRS-CSx
Colorectum	PRS7	rs1555870	10	95519577	C	A	-3.84E-04	PRS-CSx
Colorectum	PRS7	rs1556387	6	14202715	G	A	-1.04E-03	PRS-CSx
Colorectum	PRS7	rs1558299	7	151121676	T	C	4.70E-04	PRS-CSx
Colorectum	PRS7	rs1558536	18	53963907	G	A	-3.27E-04	PRS-CSx
Colorectum	PRS7	rs1558747	17	70068053	A	G	-4.25E-04	PRS-CSx
Colorectum	PRS7	rs1558907	12	14100193	T	G	-4.39E-04	PRS-CSx
Colorectum	PRS7	rs1559050	5	134470030	G	A	1.62E-03	PRS-CSx
Colorectum	PRS7	rs1559677	15	47738063	G	A	7.53E-04	PRS-CSx
Colorectum	PRS7	rs1560352	3	122446450	A	G	-5.54E-04	PRS-CSx
Colorectum	PRS7	rs1560502	18	59751715	T	C	-4.31E-04	PRS-CSx
Colorectum	PRS7	rs1561270	2	19361579	T	C	5.71E-04	PRS-CSx
Colorectum	PRS7	rs1561988	3	64543258	A	G	2.72E-04	PRS-CSx
Colorectum	PRS7	rs1561998	18	9478293	T	G	-2.31E-04	PRS-CSx
Colorectum	PRS7	rs1562871	8	128401772	C	T	2.39E-03	PRS-CSx
Colorectum	PRS7	rs1564135	4	22430267	A	G	-3.05E-04	PRS-CSx
Colorectum	PRS7	rs1564138	4	22451781	T	C	-3.66E-04	PRS-CSx
Colorectum	PRS7	rs1564139	4	22452276	G	A	-2.99E-04	PRS-CSx
Colorectum	PRS7	rs1564203	19	39210961	A	G	2.83E-04	PRS-CSx
Colorectum	PRS7	rs1564442	6	28132426	A	G	1.20E-03	PRS-CSx
Colorectum	PRS7	rs156473	5	8723607	G	T	2.32E-05	PRS-CSx
Colorectum	PRS7	rs1565072	2	225545245	A	G	2.34E-04	PRS-CSx
Colorectum	PRS7	rs1565073	2	225634693	T	G	1.99E-04	PRS-CSx
Colorectum	PRS7	rs1565496	15	75529182	A	G	5.37E-04	PRS-CSx
Colorectum	PRS7	rs1565592	12	19997502	T	G	3.11E-04	PRS-CSx
Colorectum	PRS7	rs1565912	4	134975009	G	A	6.41E-04	PRS-CSx
Colorectum	PRS7	rs1566618	5	31319072	C	T	-2.88E-04	PRS-CSx
Colorectum	PRS7	rs1567759	12	53091566	A	C	-7.55E-04	PRS-CSx
Colorectum	PRS7	rs1568751	2	225324391	G	T	5.18E-04	PRS-CSx
Colorectum	PRS7	rs1568814	7	124222007	C	T	4.31E-04	PRS-CSx
Colorectum	PRS7	rs156944	7	132144790	T	C	-2.52E-03	PRS-CSx
Colorectum	PRS7	rs1569454	20	7877416	C	A	2.33E-04	PRS-CSx
Colorectum	PRS7	rs1569507	22	43356699	G	A	9.91E-05	PRS-CSx
Colorectum	PRS7	rs1569510	22	43359448	G	A	-2.55E-05	PRS-CSx
Colorectum	PRS7	rs1569699	6	20679310	G	T	-1.56E-04	PRS-CSx
Colorectum	PRS7	rs1570024	20	60978361	C	T	-2.42E-04	PRS-CSx
Colorectum	PRS7	rs1570027	20	60968596	G	A	-5.48E-03	PRS-CSx
Colorectum	PRS7	rs1570061	6	26575986	T	C	-5.21E-05	PRS-CSx
Colorectum	PRS7	rs1570807	1	179061108	A	G	-4.07E-04	PRS-CSx
Colorectum	PRS7	rs157096	20	56255398	T	C	-9.47E-04	PRS-CSx
Colorectum	PRS7	rs1571217	20	6316526	G	T	1.15E-04	PRS-CSx
Colorectum	PRS7	rs1571218	20	6370053	T	G	3.53E-04	PRS-CSx
Colorectum	PRS7	rs1571556	13	73968916	C	T	1.54E-04	PRS-CSx
Colorectum	PRS7	rs1572069	13	24124492	A	C	-5.25E-05	PRS-CSx
Colorectum	PRS7	rs1572416	1	156952054	A	C	-2.28E-04	PRS-CSx
Colorectum	PRS7	rs1573703	22	37827711	T	G	-8.93E-04	PRS-CSx
Colorectum	PRS7	rs1573741	6	111952375	T	C	-2.72E-04	PRS-CSx
Colorectum	PRS7	rs157411	5	67294907	G	A	4.22E-04	PRS-CSx
Colorectum	PRS7	rs1574381	18	63798827	A	G	4.41E-04	PRS-CSx
Colorectum	PRS7	rs1574430	6	43269029	A	C	-3.51E-04	PRS-CSx
Colorectum	PRS7	rs1574477	3	112830021	A	G	4.43E-04	PRS-CSx

Colorectum	PRS7	rs158123	14	77560478	A	G	1.21E-03	PRS-CSx
Colorectum	PRS7	rs1582416	5	143510824	A	G	-4.65E-04	PRS-CSx
Colorectum	PRS7	rs1583178	18	6766273	A	G	-6.34E-04	PRS-CSx
Colorectum	PRS7	rs1583233	15	27223811	T	C	4.90E-04	PRS-CSx
Colorectum	PRS7	rs1584396	1	105033164	G	A	-5.11E-04	PRS-CSx
Colorectum	PRS7	rs1584545	8	83852033	C	T	-2.02E-04	PRS-CSx
Colorectum	PRS7	rs158622	5	36887895	T	C	-1.02E-04	PRS-CSx
Colorectum	PRS7	rs1586598	6	75011462	T	C	-1.44E-04	PRS-CSx
Colorectum	PRS7	rs1587107	15	102006485	T	C	4.12E-04	PRS-CSx
Colorectum	PRS7	rs1587372	1	88882301	T	C	-2.00E-04	PRS-CSx
Colorectum	PRS7	rs158796	5	36878780	A	G	-1.64E-04	PRS-CSx
Colorectum	PRS7	rs1588352	2	167484730	C	T	-1.03E-03	PRS-CSx
Colorectum	PRS7	rs1588549	6	23895830	T	C	-9.57E-05	PRS-CSx
Colorectum	PRS7	rs1591474	1	101168121	T	C	-5.97E-05	PRS-CSx
Colorectum	PRS7	rs1592410	6	29483968	T	C	-2.55E-05	PRS-CSx
Colorectum	PRS7	rs1592411	6	29483911	T	C	-1.95E-05	PRS-CSx
Colorectum	PRS7	rs1592572	16	11810382	C	T	-4.50E-04	PRS-CSx
Colorectum	PRS7	rs1596623	12	46474068	G	A	8.97E-04	PRS-CSx
Colorectum	PRS7	rs159751	5	36999998	A	G	-1.75E-04	PRS-CSx
Colorectum	PRS7	rs159753	5	37007629	T	C	4.30E-06	PRS-CSx
Colorectum	PRS7	rs1598904	4	69813786	A	G	-1.55E-04	PRS-CSx
Colorectum	PRS7	rs1601723	18	69693682	G	A	-2.60E-04	PRS-CSx
Colorectum	PRS7	rs1602298	3	14914578	G	A	-2.69E-04	PRS-CSx
Colorectum	PRS7	rs1602300	3	14914699	G	A	-3.25E-04	PRS-CSx
Colorectum	PRS7	rs1602955	12	70142793	A	G	4.51E-04	PRS-CSx
Colorectum	PRS7	rs1605279	13	71636909	T	G	7.06E-04	PRS-CSx
Colorectum	PRS7	rs160582	17	3580996	A	G	2.82E-04	PRS-CSx
Colorectum	PRS7	rs160586	17	3578178	G	A	3.13E-04	PRS-CSx
Colorectum	PRS7	rs160587	17	3577273	A	G	3.01E-04	PRS-CSx
Colorectum	PRS7	rs1606973	2	51874700	C	A	-6.36E-04	PRS-CSx
Colorectum	PRS7	rs1607100	18	41680769	T	C	6.70E-04	PRS-CSx
Colorectum	PRS7	rs160876	5	3082643	C	T	6.28E-04	PRS-CSx
Colorectum	PRS7	rs1609761	19	1847698	C	A	3.76E-04	PRS-CSx
Colorectum	PRS7	rs1610584	6	29675615	A	G	-6.82E-05	PRS-CSx
Colorectum	PRS7	rs1610586	6	29676316	T	C	-4.58E-05	PRS-CSx
Colorectum	PRS7	rs1610593	6	29678520	A	G	7.88E-05	PRS-CSx
Colorectum	PRS7	rs1610594	6	29679047	C	T	1.47E-05	PRS-CSx
Colorectum	PRS7	rs1613416	17	17133121	G	A	2.95E-04	PRS-CSx
Colorectum	PRS7	rs161445	3	7823658	C	T	-2.58E-04	PRS-CSx
Colorectum	PRS7	rs161450	3	7831196	A	G	-3.83E-04	PRS-CSx
Colorectum	PRS7	rs1614627	1	20946756	A	C	-8.18E-04	PRS-CSx
Colorectum	PRS7	rs16172	7	24297325	G	A	8.66E-04	PRS-CSx
Colorectum	PRS7	rs161913	3	7822324	T	G	-2.21E-04	PRS-CSx
Colorectum	PRS7	rs16210	7	24267358	C	T	-1.18E-03	PRS-CSx
Colorectum	PRS7	rs1623523	18	3328746	C	T	7.02E-04	PRS-CSx
Colorectum	PRS7	rs1624809	15	76335901	C	A	-4.21E-04	PRS-CSx
Colorectum	PRS7	rs1625579	1	98502934	G	T	-2.59E-04	PRS-CSx
Colorectum	PRS7	rs1625709	3	14914385	T	C	-3.99E-04	PRS-CSx
Colorectum	PRS7	rs16260	16	68771034	A	C	-9.01E-04	PRS-CSx
Colorectum	PRS7	rs1630944	13	60078760	G	A	-1.53E-03	PRS-CSx
Colorectum	PRS7	rs1632812	9	118223430	A	G	1.07E-05	PRS-CSx
Colorectum	PRS7	rs1632933	6	29797933	C	T	-1.28E-04	PRS-CSx
Colorectum	PRS7	rs1633085	6	29715997	T	C	5.02E-05	PRS-CSx
Colorectum	PRS7	rs1635	6	28227604	A	C	5.20E-04	PRS-CSx
Colorectum	PRS7	rs1635135	12	113454896	G	A	6.44E-04	PRS-CSx
Colorectum	PRS7	rs1642037	12	109569394	T	C	1.90E-03	PRS-CSx

Colorectum	PRS7	rs1644148	5	122624900	G	A	-1.10E-03	PRS-CSx
Colorectum	PRS7	rs164700	5	106908733	A	C	-2.45E-03	PRS-CSx
Colorectum	PRS7	rs164855	2	55019651	G	A	-1.47E-03	PRS-CSx
Colorectum	PRS7	rs1653098	6	7917086	A	G	-1.51E-03	PRS-CSx
Colorectum	PRS7	rs16531	17	37349655	C	T	2.05E-04	PRS-CSx
Colorectum	PRS7	rs16541	17	37365138	G	A	5.66E-04	PRS-CSx
Colorectum	PRS7	rs1654873	12	109580205	A	G	-2.15E-04	PRS-CSx
Colorectum	PRS7	rs1654884	12	109574956	T	C	3.72E-04	PRS-CSx
Colorectum	PRS7	rs1655475	11	74230411	C	T	-3.37E-04	PRS-CSx
Colorectum	PRS7	rs1655483	11	74264777	G	A	5.39E-04	PRS-CSx
Colorectum	PRS7	rs1660319	8	101337530	G	A	-1.57E-04	PRS-CSx
Colorectum	PRS7	rs1660331	8	101309456	C	T	-1.94E-04	PRS-CSx
Colorectum	PRS7	rs1660332	8	101309490	T	C	-2.02E-04	PRS-CSx
Colorectum	PRS7	rs1660350	8	101268237	G	A	-2.45E-04	PRS-CSx
Colorectum	PRS7	rs166118	16	69042861	A	G	3.90E-04	PRS-CSx
Colorectum	PRS7	rs1662291	18	3338827	T	C	1.01E-03	PRS-CSx
Colorectum	PRS7	rs1662830	18	3345173	G	A	1.32E-03	PRS-CSx
Colorectum	PRS7	rs166750	9	89765887	A	G	1.26E-04	PRS-CSx
Colorectum	PRS7	rs167013	2	183572496	T	C	1.82E-04	PRS-CSx
Colorectum	PRS7	rs1671163	19	55590671	A	G	-9.25E-04	PRS-CSx
Colorectum	PRS7	rs1672385	3	101631685	G	A	-5.10E-04	PRS-CSx
Colorectum	PRS7	rs1673026	19	50901893	A	G	1.09E-03	PRS-CSx
Colorectum	PRS7	rs1673037	19	50913961	G	T	1.67E-03	PRS-CSx
Colorectum	PRS7	rs1673041	19	50909389	G	T	9.98E-04	PRS-CSx
Colorectum	PRS7	rs1679709	6	28228342	A	G	-1.85E-04	PRS-CSx
Colorectum	PRS7	rs16822651	3	114142624	G	A	-1.32E-03	PRS-CSx
Colorectum	PRS7	rs16822702	2	163623169	G	A	-7.37E-04	PRS-CSx
Colorectum	PRS7	rs16823047	2	183183026	C	T	2.27E-04	PRS-CSx
Colorectum	PRS7	rs16823106	2	183216650	T	G	5.19E-04	PRS-CSx
Colorectum	PRS7	rs16823122	2	183223928	C	A	4.77E-04	PRS-CSx
Colorectum	PRS7	rs16823124	2	183224127	A	G	-3.57E-04	PRS-CSx
Colorectum	PRS7	rs16823254	2	183309282	G	T	3.39E-04	PRS-CSx
Colorectum	PRS7	rs16824518	1	38415315	G	T	1.79E-04	PRS-CSx
Colorectum	PRS7	rs16827691	1	187853939	A	C	-2.98E-03	PRS-CSx
Colorectum	PRS7	rs16830407	2	199625153	A	G	2.84E-03	PRS-CSx
Colorectum	PRS7	rs16830444	2	199635641	A	G	1.04E-03	PRS-CSx
Colorectum	PRS7	rs16830594	3	119606078	G	A	-3.26E-04	PRS-CSx
Colorectum	PRS7	rs16836000	2	137409890	G	A	1.03E-03	PRS-CSx
Colorectum	PRS7	rs16836553	1	164893737	A	C	2.42E-04	PRS-CSx
Colorectum	PRS7	rs16837758	1	239262486	G	A	-6.98E-04	PRS-CSx
Colorectum	PRS7	rs16837761	1	239262781	G	A	-8.02E-04	PRS-CSx
Colorectum	PRS7	rs16837764	1	239263134	A	G	-1.00E-03	PRS-CSx
Colorectum	PRS7	rs16837947	1	156971607	T	G	-4.33E-04	PRS-CSx
Colorectum	PRS7	rs16839690	1	240434513	T	C	6.77E-04	PRS-CSx
Colorectum	PRS7	rs16839858	2	204366776	A	G	5.95E-04	PRS-CSx
Colorectum	PRS7	rs16839922	2	204440345	G	T	2.07E-04	PRS-CSx
Colorectum	PRS7	rs16840287	1	5315321	T	C	-1.43E-03	PRS-CSx
Colorectum	PRS7	rs16840289	1	5315481	C	T	-1.35E-03	PRS-CSx
Colorectum	PRS7	rs1684405	12	39124891	C	T	4.94E-05	PRS-CSx
Colorectum	PRS7	rs16844882	2	161082126	A	G	2.24E-04	PRS-CSx
Colorectum	PRS7	rs16846211	3	172594666	C	A	4.04E-03	PRS-CSx
Colorectum	PRS7	rs16847178	2	212766642	C	T	1.21E-03	PRS-CSx
Colorectum	PRS7	rs16847695	3	168350231	A	C	-3.08E-04	PRS-CSx
Colorectum	PRS7	rs16852299	4	40517871	G	T	1.69E-03	PRS-CSx
Colorectum	PRS7	rs16852538	3	168380976	C	T	-6.13E-04	PRS-CSx
Colorectum	PRS7	rs16852539	3	168381002	C	T	-6.34E-04	PRS-CSx

Colorectum	PRS7	rs16852987	3	106530324	G	A	-5.24E-04	PRS-CSx
Colorectum	PRS7	rs16853666	1	204403856	T	C	2.19E-04	PRS-CSx
Colorectum	PRS7	rs16855349	2	145765851	G	A	-6.51E-04	PRS-CSx
Colorectum	PRS7	rs16856542	2	131903240	G	A	-2.71E-04	PRS-CSx
Colorectum	PRS7	rs16856866	4	44381632	C	A	-5.35E-04	PRS-CSx
Colorectum	PRS7	rs16857060	1	232557568	A	G	1.91E-03	PRS-CSx
Colorectum	PRS7	rs16857339	3	110609715	G	A	-6.38E-04	PRS-CSx
Colorectum	PRS7	rs16859371	2	172241568	C	T	-1.07E-03	PRS-CSx
Colorectum	PRS7	rs16859373	2	172242130	G	A	-1.21E-03	PRS-CSx
Colorectum	PRS7	rs16859380	2	172272444	A	G	-1.45E-03	PRS-CSx
Colorectum	PRS7	rs16859386	2	172283604	C	T	-1.02E-03	PRS-CSx
Colorectum	PRS7	rs16859404	2	172340399	T	G	-8.60E-04	PRS-CSx
Colorectum	PRS7	rs16859405	2	172345151	A	G	-9.62E-04	PRS-CSx
Colorectum	PRS7	rs16859878	1	182938992	T	C	1.68E-04	PRS-CSx
Colorectum	PRS7	rs16861393	2	174092595	A	G	-8.62E-04	PRS-CSx
Colorectum	PRS7	rs16863529	3	188409087	T	C	-2.52E-03	PRS-CSx
Colorectum	PRS7	rs16863663	3	151503678	C	T	1.04E-03	PRS-CSx
Colorectum	PRS7	rs16864142	3	188956822	G	A	1.53E-03	PRS-CSx
Colorectum	PRS7	rs16864242	2	5939613	C	T	-7.96E-04	PRS-CSx
Colorectum	PRS7	rs16864268	3	152124635	G	A	-4.66E-04	PRS-CSx
Colorectum	PRS7	rs16864316	3	152190321	T	C	-1.57E-03	PRS-CSx
Colorectum	PRS7	rs16864539	2	224036595	C	T	1.23E-03	PRS-CSx
Colorectum	PRS7	rs16866119	2	225563591	A	G	-5.26E-04	PRS-CSx
Colorectum	PRS7	rs16866157	2	225613899	T	C	-6.90E-04	PRS-CSx
Colorectum	PRS7	rs168662	5	36925735	C	T	-1.26E-04	PRS-CSx
Colorectum	PRS7	rs16866294	2	225759978	T	C	8.44E-04	PRS-CSx
Colorectum	PRS7	rs16867482	2	182585656	A	G	-7.97E-04	PRS-CSx
Colorectum	PRS7	rs16867483	2	182587034	A	G	-8.29E-04	PRS-CSx
Colorectum	PRS7	rs16868994	7	88851587	C	T	-2.93E-04	PRS-CSx
Colorectum	PRS7	rs16869652	6	33851173	A	G	-5.59E-04	PRS-CSx
Colorectum	PRS7	rs16869663	4	20485683	G	A	4.00E-04	PRS-CSx
Colorectum	PRS7	rs16870224	5	40692940	A	G	-7.01E-04	PRS-CSx
Colorectum	PRS7	rs16870378	5	40831063	C	T	-4.75E-04	PRS-CSx
Colorectum	PRS7	rs16870528	5	40936669	T	C	-4.37E-04	PRS-CSx
Colorectum	PRS7	rs16871186	6	11271992	C	T	7.87E-04	PRS-CSx
Colorectum	PRS7	rs16871188	6	11272557	G	A	8.49E-04	PRS-CSx
Colorectum	PRS7	rs16871191	6	11272676	T	C	9.13E-04	PRS-CSx
Colorectum	PRS7	rs16871204	6	11277525	C	T	3.79E-04	PRS-CSx
Colorectum	PRS7	rs16872669	4	22457478	C	T	-5.68E-04	PRS-CSx
Colorectum	PRS7	rs1687291	3	14912402	A	G	-3.01E-04	PRS-CSx
Colorectum	PRS7	rs16877392	8	30687895	T	C	4.14E-04	PRS-CSx
Colorectum	PRS7	rs16877429	8	30717452	G	A	3.43E-04	PRS-CSx
Colorectum	PRS7	rs16877479	8	30749407	G	A	4.10E-04	PRS-CSx
Colorectum	PRS7	rs16879127	6	16769840	A	C	1.03E-04	PRS-CSx
Colorectum	PRS7	rs16888	3	157375087	A	C	6.08E-04	PRS-CSx
Colorectum	PRS7	rs16888695	8	117735099	T	C	2.40E-03	PRS-CSx
Colorectum	PRS7	rs16888699	8	117735209	G	A	1.77E-03	PRS-CSx
Colorectum	PRS7	rs16888728	8	117783975	T	C	1.41E-03	PRS-CSx
Colorectum	PRS7	rs16888856	8	117819007	C	T	6.17E-04	PRS-CSx
Colorectum	PRS7	rs16888859	8	117820361	G	A	7.39E-04	PRS-CSx
Colorectum	PRS7	rs16888894	6	24209886	G	A	-4.26E-04	PRS-CSx
Colorectum	PRS7	rs16889105	8	117882864	C	T	-4.02E-04	PRS-CSx
Colorectum	PRS7	rs16889286	6	24448804	C	T	9.74E-04	PRS-CSx
Colorectum	PRS7	rs16889698	8	118400549	A	G	4.79E-04	PRS-CSx
Colorectum	PRS7	rs16889708	8	118409963	A	G	4.89E-04	PRS-CSx
Colorectum	PRS7	rs16889712	8	118413509	T	C	1.32E-04	PRS-CSx

Colorectum	PRS7	rs16889779	8	118480245	A	G	-1.98E-03	PRS-CSx
Colorectum	PRS7	rs16891146	6	25903308	A	C	6.47E-04	PRS-CSx
Colorectum	PRS7	rs16892216	8	120201893	A	G	-1.46E-03	PRS-CSx
Colorectum	PRS7	rs16893478	8	121073254	C	T	-2.72E-04	PRS-CSx
Colorectum	PRS7	rs16893917	6	28269407	A	G	1.06E-04	PRS-CSx
Colorectum	PRS7	rs16893975	6	28302278	C	T	1.69E-04	PRS-CSx
Colorectum	PRS7	rs16894908	6	29402435	T	C	3.36E-05	PRS-CSx
Colorectum	PRS7	rs16894909	6	29402687	T	C	2.26E-05	PRS-CSx
Colorectum	PRS7	rs16894932	6	29415075	G	A	1.58E-04	PRS-CSx
Colorectum	PRS7	rs16894948	6	29440257	A	G	4.57E-04	PRS-CSx
Colorectum	PRS7	rs16896467	8	98972117	A	G	-5.69E-04	PRS-CSx
Colorectum	PRS7	rs16896476	8	98987744	G	A	-8.52E-04	PRS-CSx
Colorectum	PRS7	rs16896503	8	98995892	T	C	-5.25E-04	PRS-CSx
Colorectum	PRS7	rs16896518	8	99009024	A	G	-5.33E-04	PRS-CSx
Colorectum	PRS7	rs16896564	8	99032925	T	C	-8.00E-04	PRS-CSx
Colorectum	PRS7	rs16897840	5	67733541	T	G	-1.35E-03	PRS-CSx
Colorectum	PRS7	rs16900628	5	82884168	G	A	-1.26E-03	PRS-CSx
Colorectum	PRS7	rs16901401	5	31666904	A	G	-3.50E-03	PRS-CSx
Colorectum	PRS7	rs16902124	8	128357218	A	G	-1.71E-03	PRS-CSx
Colorectum	PRS7	rs1690370	15	55315942	G	A	6.19E-04	PRS-CSx
Colorectum	PRS7	rs16908737	8	139285073	A	G	5.46E-04	PRS-CSx
Colorectum	PRS7	rs16909962	9	98296648	C	A	5.09E-03	PRS-CSx
Colorectum	PRS7	rs16911103	11	12343740	A	G	-1.40E-03	PRS-CSx
Colorectum	PRS7	rs16911106	11	12344805	G	A	-1.64E-03	PRS-CSx
Colorectum	PRS7	rs16912153	10	60095266	A	G	-1.09E-03	PRS-CSx
Colorectum	PRS7	rs16913947	10	61436118	T	C	-1.86E-03	PRS-CSx
Colorectum	PRS7	rs16913961	10	61443381	T	G	-2.47E-03	PRS-CSx
Colorectum	PRS7	rs1691550	12	77326618	G	A	-5.80E-04	PRS-CSx
Colorectum	PRS7	rs16915883	8	94368227	T	C	1.94E-03	PRS-CSx
Colorectum	PRS7	rs16917233	8	96044967	C	A	3.97E-04	PRS-CSx
Colorectum	PRS7	rs16918163	9	101813552	G	A	-3.75E-04	PRS-CSx
Colorectum	PRS7	rs1691943	10	29160836	A	G	3.71E-04	PRS-CSx
Colorectum	PRS7	rs1691946	10	29160457	T	G	4.31E-04	PRS-CSx
Colorectum	PRS7	rs1692007	8	101268343	T	C	-2.03E-04	PRS-CSx
Colorectum	PRS7	rs16920878	9	104607225	T	C	1.03E-03	PRS-CSx
Colorectum	PRS7	rs16922130	9	105506447	T	C	3.89E-04	PRS-CSx
Colorectum	PRS7	rs16922137	9	105507320	C	T	1.94E-04	PRS-CSx
Colorectum	PRS7	rs16922143	9	105507570	G	A	4.00E-04	PRS-CSx
Colorectum	PRS7	rs16923163	8	59121077	T	C	1.11E-03	PRS-CSx
Colorectum	PRS7	rs16923215	8	59148704	G	T	1.19E-03	PRS-CSx
Colorectum	PRS7	rs16924862	11	16196337	G	A	3.45E-04	PRS-CSx
Colorectum	PRS7	rs16925325	10	70090263	G	T	-1.51E-04	PRS-CSx
Colorectum	PRS7	rs16925917	10	25755263	T	C	-2.48E-03	PRS-CSx
Colorectum	PRS7	rs16926555	8	61804265	C	T	-1.71E-03	PRS-CSx
Colorectum	PRS7	rs169266	1	168824111	T	C	-9.94E-04	PRS-CSx
Colorectum	PRS7	rs16928082	12	6396156	C	T	4.77E-04	PRS-CSx
Colorectum	PRS7	rs16929631	9	12822891	A	G	-3.53E-05	PRS-CSx
Colorectum	PRS7	rs16929633	9	12823504	T	C	8.61E-05	PRS-CSx
Colorectum	PRS7	rs16930370	12	3387697	C	T	-1.06E-03	PRS-CSx
Colorectum	PRS7	rs16931454	9	132549985	A	G	3.87E-04	PRS-CSx
Colorectum	PRS7	rs16932405	11	16012242	C	T	3.77E-04	PRS-CSx
Colorectum	PRS7	rs16932409	11	16013476	T	C	2.84E-04	PRS-CSx
Colorectum	PRS7	rs16932416	11	16023324	G	A	2.01E-04	PRS-CSx
Colorectum	PRS7	rs16932458	11	16040473	T	G	3.16E-04	PRS-CSx
Colorectum	PRS7	rs16932580	11	16144535	G	A	4.01E-04	PRS-CSx
Colorectum	PRS7	rs16932585	11	16148799	G	A	1.90E-04	PRS-CSx

Colorectum	PRS7	rs16932620	11	16171678	G	A	4.75E-04	PRS-CSx
Colorectum	PRS7	rs16932670	11	16195888	G	T	4.43E-04	PRS-CSx
Colorectum	PRS7	rs16934752	12	29837605	T	C	1.60E-03	PRS-CSx
Colorectum	PRS7	rs16936082	9	104588622	C	A	3.17E-03	PRS-CSx
Colorectum	PRS7	rs16936800	10	80836089	T	C	-4.92E-04	PRS-CSx
Colorectum	PRS7	rs16938057	9	20407934	C	T	9.94E-04	PRS-CSx
Colorectum	PRS7	rs16939895	18	12821903	A	G	-3.57E-04	PRS-CSx
Colorectum	PRS7	rs16939904	12	109520769	G	A	1.30E-03	PRS-CSx
Colorectum	PRS7	rs16940641	16	86259036	A	C	-4.26E-04	PRS-CSx
Colorectum	PRS7	rs16940923	15	59254408	C	T	-1.44E-03	PRS-CSx
Colorectum	PRS7	rs16941541	12	111953704	A	G	2.65E-03	PRS-CSx
Colorectum	PRS7	rs16941856	16	86708914	T	C	8.90E-04	PRS-CSx
Colorectum	PRS7	rs16943661	15	90404123	A	C	-5.80E-04	PRS-CSx
Colorectum	PRS7	rs16945023	15	86375173	G	T	7.39E-04	PRS-CSx
Colorectum	PRS7	rs169464	3	193290216	T	C	-6.20E-04	PRS-CSx
Colorectum	PRS7	rs16947785	18	27858545	A	G	1.36E-03	PRS-CSx
Colorectum	PRS7	rs16947786	18	27858581	G	A	8.44E-04	PRS-CSx
Colorectum	PRS7	rs16949946	12	120537273	G	T	3.94E-04	PRS-CSx
Colorectum	PRS7	rs16950087	18	46442183	T	C	4.83E-03	PRS-CSx
Colorectum	PRS7	rs16951777	15	68615959	G	A	3.31E-03	PRS-CSx
Colorectum	PRS7	rs16951842	16	80005995	A	G	-8.47E-04	PRS-CSx
Colorectum	PRS7	rs16951897	16	80013110	T	C	-3.88E-04	PRS-CSx
Colorectum	PRS7	rs16951906	16	80014006	G	A	-3.94E-04	PRS-CSx
Colorectum	PRS7	rs16952464	16	80157826	T	C	-3.68E-04	PRS-CSx
Colorectum	PRS7	rs16952477	16	80159704	G	A	-1.98E-04	PRS-CSx
Colorectum	PRS7	rs16953404	15	59264164	A	G	-1.09E-03	PRS-CSx
Colorectum	PRS7	rs16954586	13	98508878	T	C	-4.98E-04	PRS-CSx
Colorectum	PRS7	rs16954807	15	71047670	T	C	-6.10E-04	PRS-CSx
Colorectum	PRS7	rs16955157	18	9521255	A	G	-4.61E-04	PRS-CSx
Colorectum	PRS7	rs16955499	15	71655079	C	T	3.55E-04	PRS-CSx
Colorectum	PRS7	rs16955598	17	53195683	C	T	-1.08E-03	PRS-CSx
Colorectum	PRS7	rs16955855	17	6334336	G	A	-3.67E-03	PRS-CSx
Colorectum	PRS7	rs16956607	13	100120220	G	A	-1.21E-03	PRS-CSx
Colorectum	PRS7	rs16956940	16	82303212	C	T	-1.54E-04	PRS-CSx
Colorectum	PRS7	rs16959739	15	47892021	G	T	2.14E-03	PRS-CSx
Colorectum	PRS7	rs16961083	16	13170785	A	G	-3.00E-03	PRS-CSx
Colorectum	PRS7	rs16961362	15	35944606	A	G	-7.90E-03	PRS-CSx
Colorectum	PRS7	rs16963973	15	32963205	G	A	1.73E-04	PRS-CSx
Colorectum	PRS7	rs16964201	15	51515351	T	C	2.73E-04	PRS-CSx
Colorectum	PRS7	rs16964879	15	37580294	G	A	-4.23E-04	PRS-CSx
Colorectum	PRS7	rs16966144	13	105883929	T	C	-1.25E-03	PRS-CSx
Colorectum	PRS7	rs16966149	13	105884314	G	A	-1.01E-03	PRS-CSx
Colorectum	PRS7	rs16966170	13	105898497	G	A	6.29E-04	PRS-CSx
Colorectum	PRS7	rs16967366	13	106452651	A	G	2.74E-03	PRS-CSx
Colorectum	PRS7	rs16967398	13	106461440	A	G	4.48E-03	PRS-CSx
Colorectum	PRS7	rs16967529	18	33779078	G	T	3.63E-04	PRS-CSx
Colorectum	PRS7	rs16967548	18	33781725	T	C	4.92E-04	PRS-CSx
Colorectum	PRS7	rs16967863	19	33706293	A	C	-5.59E-04	PRS-CSx
Colorectum	PRS7	rs16967952	19	33795241	A	G	-2.91E-04	PRS-CSx
Colorectum	PRS7	rs16969816	15	32993645	A	G	5.36E-03	PRS-CSx
Colorectum	PRS7	rs16969862	15	32994001	G	A	6.80E-03	PRS-CSx
Colorectum	PRS7	rs16970016	15	32995298	C	A	-5.87E-03	PRS-CSx
Colorectum	PRS7	rs16970288	19	35887129	C	T	9.09E-04	PRS-CSx
Colorectum	PRS7	rs16971746	17	34292939	T	C	-1.83E-03	PRS-CSx
Colorectum	PRS7	rs16972024	19	37645458	A	G	7.94E-04	PRS-CSx
Colorectum	PRS7	rs16973342	19	39846328	G	A	1.26E-03	PRS-CSx

Colorectum	PRS7	rs16974510	15	85278404	C	T	-1.63E-03	PRS-CSx
Colorectum	PRS7	rs16975299	19	38210009	C	T	8.82E-05	PRS-CSx
Colorectum	PRS7	rs16975420	13	110813991	A	C	1.21E-05	PRS-CSx
Colorectum	PRS7	rs16975766	15	96432446	C	T	-1.99E-03	PRS-CSx
Colorectum	PRS7	rs16976756	17	69559865	C	T	7.10E-04	PRS-CSx
Colorectum	PRS7	rs16978026	18	42068053	G	A	3.90E-04	PRS-CSx
Colorectum	PRS7	rs16978042	18	42085756	A	G	1.16E-04	PRS-CSx
Colorectum	PRS7	rs16978310	18	42823514	G	A	1.37E-03	PRS-CSx
Colorectum	PRS7	rs16982345	19	18500722	A	G	-6.42E-04	PRS-CSx
Colorectum	PRS7	rs16983165	20	61318835	A	C	8.86E-04	PRS-CSx
Colorectum	PRS7	rs16983785	21	30525418	G	T	-1.51E-03	PRS-CSx
Colorectum	PRS7	rs169884	1	21640565	T	C	-6.16E-04	PRS-CSx
Colorectum	PRS7	rs16988409	4	39269350	T	C	-1.30E-04	PRS-CSx
Colorectum	PRS7	rs16993969	20	7731498	A	G	7.15E-04	PRS-CSx
Colorectum	PRS7	rs16993982	20	7744461	C	T	6.80E-04	PRS-CSx
Colorectum	PRS7	rs16994091	20	7847156	T	C	4.51E-04	PRS-CSx
Colorectum	PRS7	rs16994111	20	13677405	A	G	1.95E-03	PRS-CSx
Colorectum	PRS7	rs16994265	22	45977415	T	C	-1.08E-03	PRS-CSx
Colorectum	PRS7	rs16995568	20	49500894	C	T	-1.52E-05	PRS-CSx
Colorectum	PRS7	rs16997734	22	37518632	C	T	1.33E-03	PRS-CSx
Colorectum	PRS7	rs17000211	19	10369086	T	C	1.75E-03	PRS-CSx
Colorectum	PRS7	rs17004505	21	47571209	C	T	6.48E-04	PRS-CSx
Colorectum	PRS7	rs17008806	1	220976257	C	A	2.47E-04	PRS-CSx
Colorectum	PRS7	rs17016178	3	25358403	C	T	3.59E-04	PRS-CSx
Colorectum	PRS7	rs17021094	1	213785732	G	A	5.20E-04	PRS-CSx
Colorectum	PRS7	rs17021189	1	213831296	T	C	1.88E-03	PRS-CSx
Colorectum	PRS7	rs1702292	1	98524960	A	G	-4.69E-04	PRS-CSx
Colorectum	PRS7	rs17023158	2	100447926	C	T	-2.31E-03	PRS-CSx
Colorectum	PRS7	rs17023308	12	95198736	A	G	-2.11E-03	PRS-CSx
Colorectum	PRS7	rs17023366	12	95250098	A	G	-4.36E-04	PRS-CSx
Colorectum	PRS7	rs17024935	12	96375627	C	A	-1.31E-03	PRS-CSx
Colorectum	PRS7	rs17024981	12	96388052	A	G	-1.01E-03	PRS-CSx
Colorectum	PRS7	rs17025223	1	110490341	T	C	-2.39E-03	PRS-CSx
Colorectum	PRS7	rs17029516	2	42505302	A	G	-4.38E-05	PRS-CSx
Colorectum	PRS7	rs17029598	2	42565581	A	G	-1.44E-05	PRS-CSx
Colorectum	PRS7	rs17030228	2	43180327	A	G	5.33E-04	PRS-CSx
Colorectum	PRS7	rs17031792	3	65322335	T	C	1.24E-03	PRS-CSx
Colorectum	PRS7	rs17032787	2	67173901	G	A	5.55E-04	PRS-CSx
Colorectum	PRS7	rs17032961	2	67290698	G	A	-1.83E-03	PRS-CSx
Colorectum	PRS7	rs17032980	2	67302743	G	A	-3.48E-04	PRS-CSx
Colorectum	PRS7	rs17033002	2	67305081	T	G	-1.44E-03	PRS-CSx
Colorectum	PRS7	rs17033149	2	107271354	A	G	-1.39E-03	PRS-CSx
Colorectum	PRS7	rs17033243	2	107391406	T	C	5.48E-04	PRS-CSx
Colorectum	PRS7	rs17033866	1	116040016	C	T	8.82E-04	PRS-CSx
Colorectum	PRS7	rs17033969	1	116100276	G	A	7.80E-04	PRS-CSx
Colorectum	PRS7	rs17035692	12	104911761	T	C	1.18E-03	PRS-CSx
Colorectum	PRS7	rs1703824	17	813324	C	A	1.76E-03	PRS-CSx
Colorectum	PRS7	rs17038468	1	12788113	G	A	1.43E-04	PRS-CSx
Colorectum	PRS7	rs17040381	3	14892502	G	A	-3.95E-04	PRS-CSx
Colorectum	PRS7	rs17040460	3	14914965	G	A	-3.08E-04	PRS-CSx
Colorectum	PRS7	rs17040476	3	14917526	C	T	-8.86E-04	PRS-CSx
Colorectum	PRS7	rs17040818	12	108988757	T	G	-5.20E-04	PRS-CSx
Colorectum	PRS7	rs17042031	4	163057153	C	T	-2.84E-04	PRS-CSx
Colorectum	PRS7	rs17042032	4	163057553	G	A	-4.14E-04	PRS-CSx
Colorectum	PRS7	rs17042045	4	163059003	T	C	-4.81E-04	PRS-CSx
Colorectum	PRS7	rs17042061	4	163065087	G	T	-1.82E-04	PRS-CSx

Colorectum	PRS7	rs17042064	4	163065143	C	T	-4.38E-04	PRS-CSx
Colorectum	PRS7	rs17044226	2	53428128	A	G	-1.06E-03	PRS-CSx
Colorectum	PRS7	rs17047693	4	116112491	C	T	-1.39E-03	PRS-CSx
Colorectum	PRS7	rs17048874	4	137614143	C	A	-1.05E-03	PRS-CSx
Colorectum	PRS7	rs17050199	2	59732957	T	C	2.05E-03	PRS-CSx
Colorectum	PRS7	rs17051692	3	40931175	T	C	7.51E-04	PRS-CSx
Colorectum	PRS7	rs17054121	13	58019783	A	C	2.52E-04	PRS-CSx
Colorectum	PRS7	rs17054125	13	58020608	G	A	2.51E-04	PRS-CSx
Colorectum	PRS7	rs17054187	13	58140857	G	A	6.97E-04	PRS-CSx
Colorectum	PRS7	rs17054896	3	55199267	T	C	-3.46E-05	PRS-CSx
Colorectum	PRS7	rs17058157	6	98490015	A	G	5.79E-04	PRS-CSx
Colorectum	PRS7	rs17059678	6	131162265	G	A	3.77E-04	PRS-CSx
Colorectum	PRS7	rs17059709	18	74367462	T	C	-2.89E-03	PRS-CSx
Colorectum	PRS7	rs17059736	6	131190838	A	G	4.52E-04	PRS-CSx
Colorectum	PRS7	rs17059855	8	29109665	C	T	5.84E-04	PRS-CSx
Colorectum	PRS7	rs17059860	8	29115928	T	C	5.24E-04	PRS-CSx
Colorectum	PRS7	rs17060521	4	175410435	A	G	-3.73E-04	PRS-CSx
Colorectum	PRS7	rs17060826	8	22263172	C	T	8.55E-04	PRS-CSx
Colorectum	PRS7	rs17061478	3	59908281	T	C	-3.76E-03	PRS-CSx
Colorectum	PRS7	rs17062158	9	78786218	C	A	4.03E-03	PRS-CSx
Colorectum	PRS7	rs17063390	4	177528508	A	G	6.59E-04	PRS-CSx
Colorectum	PRS7	rs17063641	5	173950287	A	G	-1.10E-03	PRS-CSx
Colorectum	PRS7	rs17064262	6	135465474	C	T	1.57E-03	PRS-CSx
Colorectum	PRS7	rs17064916	18	56042060	T	C	-4.61E-03	PRS-CSx
Colorectum	PRS7	rs17065391	8	222239	C	T	-1.99E-03	PRS-CSx
Colorectum	PRS7	rs17073169	18	62176300	A	C	-1.47E-03	PRS-CSx
Colorectum	PRS7	rs17073578	6	144618513	G	A	-1.11E-03	PRS-CSx
Colorectum	PRS7	rs17074325	3	66006701	T	C	1.50E-03	PRS-CSx
Colorectum	PRS7	rs17074540	13	51101332	T	C	1.31E-03	PRS-CSx
Colorectum	PRS7	rs17074618	13	51153475	T	C	9.01E-04	PRS-CSx
Colorectum	PRS7	rs17077550	6	148221417	G	A	4.71E-04	PRS-CSx
Colorectum	PRS7	rs17078051	6	116866949	G	A	-4.05E-04	PRS-CSx
Colorectum	PRS7	rs17078078	3	45621175	C	A	6.81E-04	PRS-CSx
Colorectum	PRS7	rs17078956	13	85565906	T	C	-3.83E-04	PRS-CSx
Colorectum	PRS7	rs17079281	6	117803138	T	C	1.68E-04	PRS-CSx
Colorectum	PRS7	rs17079343	6	117858304	G	T	8.38E-04	PRS-CSx
Colorectum	PRS7	rs17080575	9	86319414	C	T	8.28E-04	PRS-CSx
Colorectum	PRS7	rs17081262	6	151776164	T	G	9.29E-04	PRS-CSx
Colorectum	PRS7	rs17081270	6	151785551	G	A	9.83E-04	PRS-CSx
Colorectum	PRS7	rs17084655	6	122937413	G	A	1.20E-03	PRS-CSx
Colorectum	PRS7	rs17085315	18	69578321	A	G	1.12E-03	PRS-CSx
Colorectum	PRS7	rs1708551	6	67044197	G	T	-1.14E-03	PRS-CSx
Colorectum	PRS7	rs1708552	6	67044431	T	G	-7.63E-04	PRS-CSx
Colorectum	PRS7	rs1708553	6	67044536	C	A	-1.10E-03	PRS-CSx
Colorectum	PRS7	rs1708618	17	17131869	T	C	7.26E-04	PRS-CSx
Colorectum	PRS7	rs17086366	13	70678558	C	T	-1.03E-03	PRS-CSx
Colorectum	PRS7	rs17086379	5	95958386	A	G	-2.24E-03	PRS-CSx
Colorectum	PRS7	rs17087039	9	86892606	C	T	5.81E-04	PRS-CSx
Colorectum	PRS7	rs17087699	6	156979600	T	C	-1.05E-03	PRS-CSx
Colorectum	PRS7	rs17087884	9	87548647	C	A	3.37E-05	PRS-CSx
Colorectum	PRS7	rs1708933	3	129623239	G	A	7.93E-05	PRS-CSx
Colorectum	PRS7	rs17090128	13	73962540	C	T	-2.77E-05	PRS-CSx
Colorectum	PRS7	rs17091712	12	43094326	G	T	-7.94E-04	PRS-CSx
Colorectum	PRS7	rs17091723	12	43098020	G	A	-6.42E-04	PRS-CSx
Colorectum	PRS7	rs17091780	12	43137088	T	C	-1.22E-03	PRS-CSx
Colorectum	PRS7	rs17092068	14	57209682	A	G	1.14E-03	PRS-CSx

Colorectum	PRS7	rs17093978	14	58406532	C	T	3.76E-03	PRS-CSx
Colorectum	PRS7	rs17094567	10	118102086	T	C	7.78E-04	PRS-CSx
Colorectum	PRS7	rs17094683	10	104851301	T	G	5.54E-04	PRS-CSx
Colorectum	PRS7	rs17095008	14	59204202	A	G	-1.71E-03	PRS-CSx
Colorectum	PRS7	rs17095088	14	59240410	G	A	5.44E-04	PRS-CSx
Colorectum	PRS7	rs17095315	14	59348392	A	C	-2.08E-05	PRS-CSx
Colorectum	PRS7	rs17095496	14	59447241	G	A	9.57E-04	PRS-CSx
Colorectum	PRS7	rs17095507	14	59448252	T	C	-7.18E-04	PRS-CSx
Colorectum	PRS7	rs17098224	5	141567415	T	G	1.51E-03	PRS-CSx
Colorectum	PRS7	rs1709942	3	106589586	C	T	-3.12E-04	PRS-CSx
Colorectum	PRS7	rs17099556	14	62604735	A	G	-1.73E-03	PRS-CSx
Colorectum	PRS7	rs17099560	14	62610213	A	G	-9.30E-04	PRS-CSx
Colorectum	PRS7	rs17099567	14	62642558	T	C	-1.62E-03	PRS-CSx
Colorectum	PRS7	rs17100307	14	72184751	A	G	6.42E-04	PRS-CSx
Colorectum	PRS7	rs17101620	14	103916916	T	C	3.34E-04	PRS-CSx
Colorectum	PRS7	rs1710182	12	101372108	G	A	-3.56E-04	PRS-CSx
Colorectum	PRS7	rs17102844	14	66143780	G	A	1.17E-03	PRS-CSx
Colorectum	PRS7	rs17102896	14	66205671	T	C	1.47E-03	PRS-CSx
Colorectum	PRS7	rs17102945	14	66240283	A	G	8.58E-04	PRS-CSx
Colorectum	PRS7	rs17104820	12	68504321	T	C	2.76E-03	PRS-CSx
Colorectum	PRS7	rs17105896	11	106494775	C	T	-1.25E-03	PRS-CSx
Colorectum	PRS7	rs17106195	14	69189494	T	C	7.02E-04	PRS-CSx
Colorectum	PRS7	rs17109069	14	71615357	C	T	3.76E-03	PRS-CSx
Colorectum	PRS7	rs17109902	12	54661102	T	C	-4.88E-04	PRS-CSx
Colorectum	PRS7	rs17110183	14	80471122	A	G	6.38E-04	PRS-CSx
Colorectum	PRS7	rs17111143	14	81294936	G	T	-1.32E-03	PRS-CSx
Colorectum	PRS7	rs17112021	10	101343726	G	T	4.42E-03	PRS-CSx
Colorectum	PRS7	rs17113281	14	107139583	C	T	-3.69E-03	PRS-CSx
Colorectum	PRS7	rs17114156	12	75121997	C	T	6.81E-04	PRS-CSx
Colorectum	PRS7	rs17115100	10	104591393	T	G	-1.93E-04	PRS-CSx
Colorectum	PRS7	rs17117600	14	47214405	G	A	-5.74E-04	PRS-CSx
Colorectum	PRS7	rs17117612	14	47225891	C	A	-6.62E-04	PRS-CSx
Colorectum	PRS7	rs17119351	5	140378983	G	A	4.92E-04	PRS-CSx
Colorectum	PRS7	rs17119434	10	107505161	G	A	-8.05E-04	PRS-CSx
Colorectum	PRS7	rs17119461	10	107516352	C	T	-8.28E-04	PRS-CSx
Colorectum	PRS7	rs17119466	10	107517666	G	A	-1.47E-03	PRS-CSx
Colorectum	PRS7	rs17119484	10	107522100	T	C	-1.60E-03	PRS-CSx
Colorectum	PRS7	rs17119490	10	107522927	A	G	-8.36E-04	PRS-CSx
Colorectum	PRS7	rs17119824	8	14426310	T	C	-1.42E-03	PRS-CSx
Colorectum	PRS7	rs17119892	8	14464669	C	T	-1.35E-03	PRS-CSx
Colorectum	PRS7	rs17121153	11	117603231	T	C	8.59E-04	PRS-CSx
Colorectum	PRS7	rs17121944	12	59440232	C	T	-2.87E-03	PRS-CSx
Colorectum	PRS7	rs17122165	12	59544203	G	A	-7.55E-04	PRS-CSx
Colorectum	PRS7	rs17123103	14	51368495	A	G	1.22E-03	PRS-CSx
Colorectum	PRS7	rs17123861	11	120256337	C	T	-2.90E-04	PRS-CSx
Colorectum	PRS7	rs17125602	12	62386908	T	C	1.13E-03	PRS-CSx
Colorectum	PRS7	rs17125834	10	91433162	A	G	-8.69E-04	PRS-CSx
Colorectum	PRS7	rs17126346	14	53797293	A	G	6.34E-04	PRS-CSx
Colorectum	PRS7	rs17126883	10	111694696	C	T	-4.03E-04	PRS-CSx
Colorectum	PRS7	rs17126984	10	111812955	C	T	-3.49E-04	PRS-CSx
Colorectum	PRS7	rs17127001	10	91522689	A	G	-8.32E-04	PRS-CSx
Colorectum	PRS7	rs17127261	14	91807544	T	G	-1.05E-03	PRS-CSx
Colorectum	PRS7	rs17127275	10	112117300	C	T	-1.67E-03	PRS-CSx
Colorectum	PRS7	rs17128533	10	112947633	T	C	-3.85E-03	PRS-CSx
Colorectum	PRS7	rs17129837	10	114283116	T	C	5.78E-03	PRS-CSx
Colorectum	PRS7	rs17130470	1	88920905	T	C	-6.20E-04	PRS-CSx

Colorectum	PRS7	rs17135515	5	112598947	C	T	7.65E-05	PRS-CSx
Colorectum	PRS7	rs17135526	5	112606338	A	G	7.77E-06	PRS-CSx
Colorectum	PRS7	rs17135534	5	112608343	T	C	-1.20E-04	PRS-CSx
Colorectum	PRS7	rs17136612	10	8660646	G	A	5.87E-05	PRS-CSx
Colorectum	PRS7	rs17138295	17	34981225	C	T	-1.47E-03	PRS-CSx
Colorectum	PRS7	rs17140779	3	67992898	G	A	8.74E-04	PRS-CSx
Colorectum	PRS7	rs17144102	10	8637090	C	A	2.10E-04	PRS-CSx
Colorectum	PRS7	rs17144396	10	8773922	T	G	3.41E-03	PRS-CSx
Colorectum	PRS7	rs17144411	10	8776296	T	G	-1.30E-03	PRS-CSx
Colorectum	PRS7	rs17145380	16	8238988	A	G	1.11E-03	PRS-CSx
Colorectum	PRS7	rs17146488	7	22312065	T	C	1.41E-03	PRS-CSx
Colorectum	PRS7	rs17152404	7	25762797	G	T	-3.45E-04	PRS-CSx
Colorectum	PRS7	rs17152406	7	25762903	A	C	-3.02E-04	PRS-CSx
Colorectum	PRS7	rs17154375	8	8468811	A	G	-1.81E-03	PRS-CSx
Colorectum	PRS7	rs17156153	11	270514	T	C	-3.35E-03	PRS-CSx
Colorectum	PRS7	rs17156247	7	77297541	T	C	-2.24E-04	PRS-CSx
Colorectum	PRS7	rs17156320	7	77300110	T	C	-1.88E-04	PRS-CSx
Colorectum	PRS7	rs17159928	11	86182941	A	G	-2.56E-04	PRS-CSx
Colorectum	PRS7	rs17161975	1	223369577	G	A	-4.74E-04	PRS-CSx
Colorectum	PRS7	rs17163963	1	116098091	C	T	6.24E-04	PRS-CSx
Colorectum	PRS7	rs17163969	1	116101547	T	C	7.92E-04	PRS-CSx
Colorectum	PRS7	rs171649	5	67569746	A	G	9.85E-04	PRS-CSx
Colorectum	PRS7	rs17167685	7	88127742	G	T	-3.13E-04	PRS-CSx
Colorectum	PRS7	rs17168118	7	134576840	A	G	-1.33E-03	PRS-CSx
Colorectum	PRS7	rs17169559	7	16644141	G	T	6.37E-04	PRS-CSx
Colorectum	PRS7	rs17177644	8	12768021	G	T	1.63E-03	PRS-CSx
Colorectum	PRS7	rs17181798	14	66230656	C	T	3.31E-04	PRS-CSx
Colorectum	PRS7	rs17181982	6	169400285	C	T	7.99E-04	PRS-CSx
Colorectum	PRS7	rs17183456	14	22746254	C	T	-1.12E-03	PRS-CSx
Colorectum	PRS7	rs17184107	6	29455098	A	G	2.18E-04	PRS-CSx
Colorectum	PRS7	rs17184114	6	29455599	G	A	9.35E-04	PRS-CSx
Colorectum	PRS7	rs17186945	14	87188791	A	G	8.52E-04	PRS-CSx
Colorectum	PRS7	rs17187632	18	22076977	G	A	7.78E-04	PRS-CSx
Colorectum	PRS7	rs17188808	14	91122123	C	T	3.09E-03	PRS-CSx
Colorectum	PRS7	rs1719894	3	119590322	G	A	2.10E-04	PRS-CSx
Colorectum	PRS7	rs17199431	2	225810943	A	G	-3.11E-04	PRS-CSx
Colorectum	PRS7	rs17200543	3	16973762	A	G	-5.97E-04	PRS-CSx
Colorectum	PRS7	rs17201047	15	66739670	G	A	-3.83E-04	PRS-CSx
Colorectum	PRS7	rs17201917	6	32235757	G	A	-3.21E-05	PRS-CSx
Colorectum	PRS7	rs17202180	17	6392443	T	C	6.92E-04	PRS-CSx
Colorectum	PRS7	rs172038	20	22361396	G	A	2.40E-04	PRS-CSx
Colorectum	PRS7	rs17204375	9	113084488	A	C	-7.51E-04	PRS-CSx
Colorectum	PRS7	rs17206048	5	82923046	T	C	-1.16E-03	PRS-CSx
Colorectum	PRS7	rs1721279	2	225314761	G	A	3.08E-04	PRS-CSx
Colorectum	PRS7	rs17217480	2	107231903	G	A	6.56E-04	PRS-CSx
Colorectum	PRS7	rs17218399	5	40025962	G	A	-1.40E-04	PRS-CSx
Colorectum	PRS7	rs17218730	4	60560780	A	G	-1.18E-03	PRS-CSx
Colorectum	PRS7	rs17221631	10	1357992	A	G	-3.42E-03	PRS-CSx
Colorectum	PRS7	rs17224911	6	56210315	C	T	-6.53E-04	PRS-CSx
Colorectum	PRS7	rs17225519	6	56217129	C	T	-6.05E-04	PRS-CSx
Colorectum	PRS7	rs17225738	17	5861557	T	C	-6.17E-04	PRS-CSx
Colorectum	PRS7	rs17228317	15	31756833	G	T	4.48E-04	PRS-CSx
Colorectum	PRS7	rs17231256	21	40448889	A	G	3.11E-03	PRS-CSx
Colorectum	PRS7	rs17234460	13	94047270	T	C	5.82E-04	PRS-CSx
Colorectum	PRS7	rs17236768	18	68007621	C	T	1.15E-03	PRS-CSx
Colorectum	PRS7	rs17237212	8	13529635	A	C	4.82E-04	PRS-CSx

Colorectum	PRS7	rs172422	11	30704234	T	C	-9.38E-04	PRS-CSx
Colorectum	PRS7	rs17245504	11	120178753	G	T	-5.15E-04	PRS-CSx
Colorectum	PRS7	rs17249164	8	75055057	A	G	-1.26E-04	PRS-CSx
Colorectum	PRS7	rs17252114	3	41212753	C	T	-1.94E-04	PRS-CSx
Colorectum	PRS7	rs17253354	14	54010575	T	G	-3.31E-04	PRS-CSx
Colorectum	PRS7	rs17254544	14	59186791	A	G	1.08E-03	PRS-CSx
Colorectum	PRS7	rs17254989	14	59442384	A	G	1.19E-03	PRS-CSx
Colorectum	PRS7	rs17258785	15	66662359	C	T	-1.83E-04	PRS-CSx
Colorectum	PRS7	rs17259168	14	87235375	T	C	1.29E-03	PRS-CSx
Colorectum	PRS7	rs17259884	18	22121586	C	T	1.91E-03	PRS-CSx
Colorectum	PRS7	rs17264185	15	66997087	G	A	-5.02E-04	PRS-CSx
Colorectum	PRS7	rs17264887	2	183213152	G	A	3.23E-04	PRS-CSx
Colorectum	PRS7	rs17265482	2	183454853	G	A	2.28E-04	PRS-CSx
Colorectum	PRS7	rs17265503	2	183461217	T	C	1.57E-04	PRS-CSx
Colorectum	PRS7	rs17265559	2	183488214	A	C	2.84E-04	PRS-CSx
Colorectum	PRS7	rs1726773	19	50927570	C	T	1.04E-03	PRS-CSx
Colorectum	PRS7	rs17269115	3	41457429	T	C	-6.85E-04	PRS-CSx
Colorectum	PRS7	rs17269562	3	41472471	G	A	-3.71E-04	PRS-CSx
Colorectum	PRS7	rs17270582	13	47120816	C	T	1.12E-03	PRS-CSx
Colorectum	PRS7	rs17271097	11	34538539	A	G	8.76E-04	PRS-CSx
Colorectum	PRS7	rs17271567	2	225795103	A	G	-5.58E-04	PRS-CSx
Colorectum	PRS7	rs17274866	15	67228359	T	G	4.90E-04	PRS-CSx
Colorectum	PRS7	rs17282873	2	107214400	T	G	5.65E-04	PRS-CSx
Colorectum	PRS7	rs17283262	3	151627671	T	C	5.80E-05	PRS-CSx
Colorectum	PRS7	rs17284610	6	4940123	G	A	-6.73E-04	PRS-CSx
Colorectum	PRS7	rs17287118	15	47603474	C	T	-2.47E-04	PRS-CSx
Colorectum	PRS7	rs17287195	12	96108758	T	C	-3.27E-04	PRS-CSx
Colorectum	PRS7	rs17287497	2	172351233	G	A	-6.55E-04	PRS-CSx
Colorectum	PRS7	rs17304583	19	33306222	T	C	5.64E-04	PRS-CSx
Colorectum	PRS7	rs17304779	19	37711656	G	A	8.86E-04	PRS-CSx
Colorectum	PRS7	rs17305345	3	14285488	T	C	6.73E-04	PRS-CSx
Colorectum	PRS7	rs17312621	8	131659108	C	T	-6.59E-04	PRS-CSx
Colorectum	PRS7	rs17313232	15	69899813	T	C	4.87E-04	PRS-CSx
Colorectum	PRS7	rs17318596	19	41937095	G	A	3.80E-04	PRS-CSx
Colorectum	PRS7	rs1732170	3	119583676	C	T	1.61E-04	PRS-CSx
Colorectum	PRS7	rs1732778	12	113456925	A	G	4.17E-04	PRS-CSx
Colorectum	PRS7	rs17329025	12	25383813	A	G	-5.49E-04	PRS-CSx
Colorectum	PRS7	rs17341612	15	82053795	C	T	-5.88E-04	PRS-CSx
Colorectum	PRS7	rs1734197	16	56215936	C	T	-3.33E-04	PRS-CSx
Colorectum	PRS7	rs1734852	21	41102931	A	G	-1.30E-03	PRS-CSx
Colorectum	PRS7	rs17350396	1	11900838	G	A	-1.25E-03	PRS-CSx
Colorectum	PRS7	rs17350701	8	75369545	G	T	5.43E-04	PRS-CSx
Colorectum	PRS7	rs17356056	11	34539004	A	G	9.94E-04	PRS-CSx
Colorectum	PRS7	rs17356152	2	183292692	G	A	9.94E-05	PRS-CSx
Colorectum	PRS7	rs17356166	2	183300759	C	T	2.56E-04	PRS-CSx
Colorectum	PRS7	rs17356207	2	183307827	G	A	2.27E-04	PRS-CSx
Colorectum	PRS7	rs17356900	6	4942582	A	G	-5.54E-04	PRS-CSx
Colorectum	PRS7	rs17361892	3	65806838	C	T	2.39E-03	PRS-CSx
Colorectum	PRS7	rs1736200	17	17155873	C	T	1.68E-04	PRS-CSx
Colorectum	PRS7	rs1736202	17	17151938	G	A	2.46E-04	PRS-CSx
Colorectum	PRS7	rs1736219	17	17127471	G	A	2.04E-04	PRS-CSx
Colorectum	PRS7	rs17363371	15	48847615	A	C	1.60E-03	PRS-CSx
Colorectum	PRS7	rs17366198	2	183018480	T	G	1.73E-05	PRS-CSx
Colorectum	PRS7	rs17366199	7	22603615	A	G	-4.33E-03	PRS-CSx
Colorectum	PRS7	rs17366503	2	183068844	G	T	1.89E-04	PRS-CSx
Colorectum	PRS7	rs1736913	6	29704400	T	C	9.40E-04	PRS-CSx

Colorectum	PRS7	rs1736936	6	29794317	G	A	-9.76E-05	PRS-CSx
Colorectum	PRS7	rs1737085	6	29716390	T	G	6.15E-05	PRS-CSx
Colorectum	PRS7	rs17371795	21	47855876	G	A	7.00E-04	PRS-CSx
Colorectum	PRS7	rs1737270	6	10806255	C	T	-1.32E-03	PRS-CSx
Colorectum	PRS7	rs17386875	5	107649113	C	T	-6.18E-04	PRS-CSx
Colorectum	PRS7	rs1739091	6	15081252	G	A	9.25E-04	PRS-CSx
Colorectum	PRS7	rs17395011	12	26641513	T	C	9.90E-04	PRS-CSx
Colorectum	PRS7	rs17398305	5	31287406	G	T	5.42E-04	PRS-CSx
Colorectum	PRS7	rs17404544	8	3964539	C	T	1.10E-03	PRS-CSx
Colorectum	PRS7	rs17410797	2	226498635	T	C	-1.43E-03	PRS-CSx
Colorectum	PRS7	rs17421511	1	11857788	A	G	-1.43E-03	PRS-CSx
Colorectum	PRS7	rs1742398	16	1959619	T	C	9.60E-05	PRS-CSx
Colorectum	PRS7	rs17429444	6	32786068	G	A	-1.28E-03	PRS-CSx
Colorectum	PRS7	rs17429538	13	24627618	A	G	7.68E-04	PRS-CSx
Colorectum	PRS7	rs17432497	2	63027337	G	T	8.83E-04	PRS-CSx
Colorectum	PRS7	rs17432675	1	201887721	C	T	-2.09E-04	PRS-CSx
Colorectum	PRS7	rs1743720	14	58925726	C	T	1.64E-04	PRS-CSx
Colorectum	PRS7	rs17440734	22	29409966	C	T	-3.15E-04	PRS-CSx
Colorectum	PRS7	rs17442148	1	222187794	C	T	-1.05E-03	PRS-CSx
Colorectum	PRS7	rs17444059	5	58560717	C	T	1.02E-03	PRS-CSx
Colorectum	PRS7	rs174448	11	61639573	G	A	-6.52E-05	PRS-CSx
Colorectum	PRS7	rs174449	11	61640379	G	A	-4.30E-05	PRS-CSx
Colorectum	PRS7	rs174450	11	61641542	G	T	-2.67E-05	PRS-CSx
Colorectum	PRS7	rs17447271	2	216165244	C	T	6.94E-04	PRS-CSx
Colorectum	PRS7	rs17448695	1	4367810	T	C	1.79E-03	PRS-CSx
Colorectum	PRS7	rs17450705	5	107526936	G	A	-6.31E-04	PRS-CSx
Colorectum	PRS7	rs174534	11	61549458	A	G	5.97E-04	PRS-CSx
Colorectum	PRS7	rs174535	11	61551356	T	C	7.85E-04	PRS-CSx
Colorectum	PRS7	rs174537	11	61552680	G	T	5.78E-04	PRS-CSx
Colorectum	PRS7	rs174538	11	61560081	G	A	5.65E-04	PRS-CSx
Colorectum	PRS7	rs174546	11	61569830	C	T	4.24E-04	PRS-CSx
Colorectum	PRS7	rs174549	11	61571382	G	A	7.31E-04	PRS-CSx
Colorectum	PRS7	rs174555	11	61579760	T	C	6.24E-04	PRS-CSx
Colorectum	PRS7	rs174556	11	61580635	C	T	7.63E-04	PRS-CSx
Colorectum	PRS7	rs174566	11	61592362	A	G	7.70E-04	PRS-CSx
Colorectum	PRS7	rs174568	11	61593816	C	T	6.13E-04	PRS-CSx
Colorectum	PRS7	rs174570	11	61597212	C	T	5.92E-03	PRS-CSx
Colorectum	PRS7	rs174574	11	61600342	C	A	7.00E-04	PRS-CSx
Colorectum	PRS7	rs174576	11	61603510	C	A	1.02E-03	PRS-CSx
Colorectum	PRS7	rs174577	11	61604814	C	A	1.17E-03	PRS-CSx
Colorectum	PRS7	rs174579	11	61605613	T	C	-5.88E-04	PRS-CSx
Colorectum	PRS7	rs174583	11	61609750	C	T	7.38E-04	PRS-CSx
Colorectum	PRS7	rs17459269	3	165439372	C	T	-3.45E-04	PRS-CSx
Colorectum	PRS7	rs174602	11	61624414	C	T	-2.01E-04	PRS-CSx
Colorectum	PRS7	rs17461113	4	44402331	G	A	-2.04E-03	PRS-CSx
Colorectum	PRS7	rs17461459	4	44492235	T	C	-1.21E-03	PRS-CSx
Colorectum	PRS7	rs174616	11	61629122	A	G	8.78E-07	PRS-CSx
Colorectum	PRS7	rs174626	11	61637057	G	A	-1.22E-04	PRS-CSx
Colorectum	PRS7	rs17465420	1	38398588	C	T	-1.10E-03	PRS-CSx
Colorectum	PRS7	rs17465637	1	222823529	A	C	4.80E-04	PRS-CSx
Colorectum	PRS7	rs17469861	9	89813686	A	C	9.42E-04	PRS-CSx
Colorectum	PRS7	rs17471975	1	22726678	G	A	7.35E-04	PRS-CSx
Colorectum	PRS7	rs17473434	1	201922759	C	T	-7.27E-04	PRS-CSx
Colorectum	PRS7	rs17482756	10	78933522	G	T	3.72E-04	PRS-CSx
Colorectum	PRS7	rs17485225	12	101400699	A	G	2.74E-03	PRS-CSx
Colorectum	PRS7	rs1749951	1	1231656	A	G	1.32E-03	PRS-CSx

Colorectum	PRS7	rs17500548	11	117232391	G	A	3.76E-04	PRS-CSx
Colorectum	PRS7	rs17501937	1	92021705	G	A	6.88E-04	PRS-CSx
Colorectum	PRS7	rs17505041	1	218857697	A	C	1.56E-03	PRS-CSx
Colorectum	PRS7	rs1751005	13	95829870	T	C	-4.16E-04	PRS-CSx
Colorectum	PRS7	rs1751008	13	95837662	T	C	-4.94E-04	PRS-CSx
Colorectum	PRS7	rs17516059	1	219053400	C	T	-8.37E-04	PRS-CSx
Colorectum	PRS7	rs17518913	4	167413675	G	A	6.48E-04	PRS-CSx
Colorectum	PRS7	rs17519507	3	165445557	A	G	-4.98E-04	PRS-CSx
Colorectum	PRS7	rs17528473	5	58559042	A	G	5.35E-04	PRS-CSx
Colorectum	PRS7	rs17531077	1	38415507	G	A	-3.29E-04	PRS-CSx
Colorectum	PRS7	rs17533219	18	54047421	C	A	-2.43E-04	PRS-CSx
Colorectum	PRS7	rs1754436	9	97366400	C	T	-6.81E-04	PRS-CSx
Colorectum	PRS7	rs17544690	7	76810993	T	C	8.27E-04	PRS-CSx
Colorectum	PRS7	rs17545474	11	83339781	G	A	3.28E-05	PRS-CSx
Colorectum	PRS7	rs17563223	18	33700007	G	A	5.46E-04	PRS-CSx
Colorectum	PRS7	rs175642	14	75915794	C	T	-1.98E-03	PRS-CSx
Colorectum	PRS7	rs175644	14	75919331	G	T	-1.27E-03	PRS-CSx
Colorectum	PRS7	rs17571491	4	44544146	G	A	-1.07E-03	PRS-CSx
Colorectum	PRS7	rs17574573	1	219024035	C	T	-1.25E-03	PRS-CSx
Colorectum	PRS7	rs17576563	2	160042083	C	T	1.52E-03	PRS-CSx
Colorectum	PRS7	rs17583347	7	10609052	A	G	-1.53E-03	PRS-CSx
Colorectum	PRS7	rs17584162	10	71646222	A	C	9.39E-04	PRS-CSx
Colorectum	PRS7	rs17584516	3	2886527	G	A	7.53E-04	PRS-CSx
Colorectum	PRS7	rs17589633	5	58532861	A	G	1.21E-03	PRS-CSx
Colorectum	PRS7	rs17591964	10	52635240	G	A	4.79E-04	PRS-CSx
Colorectum	PRS7	rs17596850	1	220992343	A	G	1.87E-04	PRS-CSx
Colorectum	PRS7	rs17599675	5	147538478	C	T	-4.34E-03	PRS-CSx
Colorectum	PRS7	rs17606653	8	21107763	G	A	-1.27E-03	PRS-CSx
Colorectum	PRS7	rs17609774	13	31256379	A	C	1.64E-03	PRS-CSx
Colorectum	PRS7	rs17617013	7	88931360	G	T	-5.58E-04	PRS-CSx
Colorectum	PRS7	rs17619106	7	16487126	G	A	6.97E-04	PRS-CSx
Colorectum	PRS7	rs1761985	10	29094608	C	T	7.31E-04	PRS-CSx
Colorectum	PRS7	rs17622815	10	82673699	C	T	-6.11E-04	PRS-CSx
Colorectum	PRS7	rs17623512	17	1026163	T	C	8.11E-04	PRS-CSx
Colorectum	PRS7	rs17624426	4	150226520	C	T	-2.76E-04	PRS-CSx
Colorectum	PRS7	rs17637310	5	153774496	A	G	6.94E-04	PRS-CSx
Colorectum	PRS7	rs17637385	5	153774793	A	C	6.71E-04	PRS-CSx
Colorectum	PRS7	rs17641840	6	148776487	G	A	8.69E-04	PRS-CSx
Colorectum	PRS7	rs17642724	7	28396607	A	C	6.36E-04	PRS-CSx
Colorectum	PRS7	rs17644283	4	26308792	A	G	-1.43E-03	PRS-CSx
Colorectum	PRS7	rs17646221	5	169310213	C	A	3.69E-04	PRS-CSx
Colorectum	PRS7	rs17647707	15	51568020	T	G	6.33E-04	PRS-CSx
Colorectum	PRS7	rs17648779	2	182972124	A	G	4.75E-05	PRS-CSx
Colorectum	PRS7	rs17649451	5	141549536	T	C	2.08E-04	PRS-CSx
Colorectum	PRS7	rs17651163	18	34109848	A	G	3.32E-04	PRS-CSx
Colorectum	PRS7	rs17652097	16	9097525	T	C	-1.34E-03	PRS-CSx
Colorectum	PRS7	rs17654592	9	1792095	G	A	1.05E-03	PRS-CSx
Colorectum	PRS7	rs17655565	12	52691958	C	T	-1.28E-03	PRS-CSx
Colorectum	PRS7	rs17659312	5	134453073	T	C	1.14E-03	PRS-CSx
Colorectum	PRS7	rs17660635	8	9197250	G	A	-4.65E-04	PRS-CSx
Colorectum	PRS7	rs17666653	14	52122477	T	C	-8.57E-04	PRS-CSx
Colorectum	PRS7	rs17668748	13	92501540	T	C	4.09E-04	PRS-CSx
Colorectum	PRS7	rs17681130	15	29301571	G	A	4.29E-04	PRS-CSx
Colorectum	PRS7	rs17681575	1	216941624	C	T	1.07E-03	PRS-CSx
Colorectum	PRS7	rs17682328	6	54710076	A	G	-7.82E-04	PRS-CSx
Colorectum	PRS7	rs17682340	6	54721939	A	G	-2.03E-03	PRS-CSx

Colorectum	PRS7	rs17686333	4	150225278	G	A	-3.70E-04	PRS-CSx
Colorectum	PRS7	rs17687191	17	63074794	T	C	1.70E-03	PRS-CSx
Colorectum	PRS7	rs17687727	2	105865967	A	G	2.00E-03	PRS-CSx
Colorectum	PRS7	rs17688079	7	130655846	A	G	5.27E-04	PRS-CSx
Colorectum	PRS7	rs17688758	4	71997259	C	T	2.18E-03	PRS-CSx
Colorectum	PRS7	rs17697699	6	12027402	G	A	-1.44E-04	PRS-CSx
Colorectum	PRS7	rs17708130	19	15676750	G	T	5.98E-04	PRS-CSx
Colorectum	PRS7	rs17708576	18	61441801	A	G	-5.14E-04	PRS-CSx
Colorectum	PRS7	rs17710730	12	64419744	A	G	-5.92E-04	PRS-CSx
Colorectum	PRS7	rs17712809	12	46182851	G	A	-2.68E-04	PRS-CSx
Colorectum	PRS7	rs17715450	16	68729785	A	C	3.83E-04	PRS-CSx
Colorectum	PRS7	rs17715902	5	134451465	A	G	1.64E-03	PRS-CSx
Colorectum	PRS7	rs17716310	5	134476759	C	A	3.03E-03	PRS-CSx
Colorectum	PRS7	rs17722119	7	42956696	C	T	-5.28E-04	PRS-CSx
Colorectum	PRS7	rs17731311	14	91877257	T	C	8.59E-04	PRS-CSx
Colorectum	PRS7	rs17735330	13	108779750	C	T	7.80E-04	PRS-CSx
Colorectum	PRS7	rs1773860	10	29291556	T	C	2.90E-03	PRS-CSx
Colorectum	PRS7	rs17739785	18	33712354	G	A	7.43E-04	PRS-CSx
Colorectum	PRS7	rs17741623	16	84454763	C	T	1.95E-04	PRS-CSx
Colorectum	PRS7	rs17754383	10	27075698	C	T	5.49E-04	PRS-CSx
Colorectum	PRS7	rs17754431	10	27086497	G	T	6.03E-04	PRS-CSx
Colorectum	PRS7	rs1775908	10	29098958	C	A	3.94E-04	PRS-CSx
Colorectum	PRS7	rs1775911	10	29096831	T	C	1.16E-03	PRS-CSx
Colorectum	PRS7	rs1775929	10	29159348	G	T	2.89E-04	PRS-CSx
Colorectum	PRS7	rs1775933	10	29160876	A	G	3.25E-04	PRS-CSx
Colorectum	PRS7	rs17759413	12	13246210	T	C	1.97E-03	PRS-CSx
Colorectum	PRS7	rs1775983	10	29106668	A	G	6.89E-04	PRS-CSx
Colorectum	PRS7	rs17771891	5	131744202	T	C	-3.56E-04	PRS-CSx
Colorectum	PRS7	rs17774927	12	46211396	C	T	-1.67E-04	PRS-CSx
Colorectum	PRS7	rs1777958	1	59964521	T	C	-5.10E-04	PRS-CSx
Colorectum	PRS7	rs17784294	10	104479385	A	C	-2.94E-04	PRS-CSx
Colorectum	PRS7	rs1778508	6	28229881	C	T	-8.12E-05	PRS-CSx
Colorectum	PRS7	rs17790938	20	49509184	A	G	-1.17E-03	PRS-CSx
Colorectum	PRS7	rs17793170	8	129205679	A	G	-1.55E-05	PRS-CSx
Colorectum	PRS7	rs17793261	8	129210618	T	G	-1.16E-04	PRS-CSx
Colorectum	PRS7	rs17795289	7	43025775	C	A	-7.46E-04	PRS-CSx
Colorectum	PRS7	rs17807186	9	113085232	G	A	-8.64E-04	PRS-CSx
Colorectum	PRS7	rs17811658	3	37214204	C	T	-1.39E-04	PRS-CSx
Colorectum	PRS7	rs17811924	8	117902813	G	A	-3.96E-04	PRS-CSx
Colorectum	PRS7	rs17815747	20	22447995	G	A	7.91E-04	PRS-CSx
Colorectum	PRS7	rs17816317	8	1240646	C	T	-7.91E-04	PRS-CSx
Colorectum	PRS7	rs17817006	15	33489957	T	C	-1.16E-03	PRS-CSx
Colorectum	PRS7	rs17817050	17	10709753	G	A	-3.08E-03	PRS-CSx
Colorectum	PRS7	rs17818670	9	19331571	A	G	7.95E-04	PRS-CSx
Colorectum	PRS7	rs17819315	5	40074082	C	T	-2.37E-04	PRS-CSx
Colorectum	PRS7	rs17823013	5	40243264	G	A	3.60E-03	PRS-CSx
Colorectum	PRS7	rs17823049	5	40243624	G	T	2.49E-03	PRS-CSx
Colorectum	PRS7	rs17823187	5	40251657	A	G	2.62E-03	PRS-CSx
Colorectum	PRS7	rs17823714	5	40274708	T	C	2.90E-03	PRS-CSx
Colorectum	PRS7	rs17825664	6	405873	C	T	1.85E-03	PRS-CSx
Colorectum	PRS7	rs17825829	17	69551489	A	G	5.64E-04	PRS-CSx
Colorectum	PRS7	rs17826222	2	199557725	T	C	4.10E-04	PRS-CSx
Colorectum	PRS7	rs1782812	1	98547502	A	G	-4.02E-04	PRS-CSx
Colorectum	PRS7	rs1783196	11	74236141	A	G	-2.79E-04	PRS-CSx
Colorectum	PRS7	rs17833053	14	59181924	A	G	1.53E-03	PRS-CSx
Colorectum	PRS7	rs1783596	11	72946020	C	T	-1.68E-03	PRS-CSx

Colorectum	PRS7	rs17837127	12	92692806	G	A	-1.09E-03	PRS-CSx
Colorectum	PRS7	rs17837621	7	155902880	T	C	1.26E-03	PRS-CSx
Colorectum	PRS7	rs17841064	14	103900609	T	C	2.55E-04	PRS-CSx
Colorectum	PRS7	rs178452	14	80443279	T	C	2.69E-04	PRS-CSx
Colorectum	PRS7	rs178454	14	80447321	G	A	3.34E-04	PRS-CSx
Colorectum	PRS7	rs178455	14	80447347	A	G	2.62E-04	PRS-CSx
Colorectum	PRS7	rs178458	14	80449781	T	G	2.42E-04	PRS-CSx
Colorectum	PRS7	rs178463	17	3578351	A	G	2.33E-04	PRS-CSx
Colorectum	PRS7	rs1785265	18	3319210	G	A	7.01E-04	PRS-CSx
Colorectum	PRS7	rs1785502	9	34023572	C	T	-1.71E-04	PRS-CSx
Colorectum	PRS7	rs1785506	9	34017106	C	T	-1.20E-04	PRS-CSx
Colorectum	PRS7	rs1785509	9	34028285	G	A	-1.86E-04	PRS-CSx
Colorectum	PRS7	rs1785512	9	34026538	C	T	-2.97E-04	PRS-CSx
Colorectum	PRS7	rs17856896	19	57909827	T	G	1.03E-03	PRS-CSx
Colorectum	PRS7	rs1786172	11	65770658	A	G	-3.15E-04	PRS-CSx
Colorectum	PRS7	rs1787091	11	74233031	G	A	-2.47E-04	PRS-CSx
Colorectum	PRS7	rs1787102	11	74206406	G	A	-9.70E-05	PRS-CSx
Colorectum	PRS7	rs1788173	8	101330939	T	C	-2.74E-04	PRS-CSx
Colorectum	PRS7	rs1788189	8	101249762	G	T	-1.79E-04	PRS-CSx
Colorectum	PRS7	rs1788190	8	101253184	G	A	-3.61E-04	PRS-CSx
Colorectum	PRS7	rs1790019	6	162367823	A	C	-7.23E-04	PRS-CSx
Colorectum	PRS7	rs1790022	6	162365901	C	T	-8.54E-04	PRS-CSx
Colorectum	PRS7	rs1791072	18	3353652	T	C	8.77E-04	PRS-CSx
Colorectum	PRS7	rs1791786	11	61399965	T	C	-6.43E-06	PRS-CSx
Colorectum	PRS7	rs1794815	6	111963703	A	G	-2.55E-04	PRS-CSx
Colorectum	PRS7	rs1798796	3	41190647	A	G	3.94E-03	PRS-CSx
Colorectum	PRS7	rs1799841	20	23860178	G	A	3.41E-04	PRS-CSx
Colorectum	PRS7	rs1800141	12	57588433	A	G	1.64E-03	PRS-CSx
Colorectum	PRS7	rs1800469	19	41860296	G	A	1.08E-03	PRS-CSx
Colorectum	PRS7	rs1800543	6	12294137	C	T	-1.64E-03	PRS-CSx
Colorectum	PRS7	rs1800734	3	37034946	G	A	-6.08E-04	PRS-CSx
Colorectum	PRS7	rs1800792	4	155534408	C	T	-4.22E-04	PRS-CSx
Colorectum	PRS7	rs1800920	8	76319876	C	A	-1.43E-03	PRS-CSx
Colorectum	PRS7	rs1801353	17	43319770	T	C	1.51E-04	PRS-CSx
Colorectum	PRS7	rs1802409	16	11772230	A	C	-4.92E-04	PRS-CSx
Colorectum	PRS7	rs1809822	1	2231595	C	A	-5.10E-04	PRS-CSx
Colorectum	PRS7	rs1809844	16	86188011	C	T	8.89E-04	PRS-CSx
Colorectum	PRS7	rs1810502	20	49057488	C	T	1.89E-03	PRS-CSx
Colorectum	PRS7	rs1810745	1	1257593	A	G	5.32E-04	PRS-CSx
Colorectum	PRS7	rs1811337	5	40140556	C	T	-1.34E-04	PRS-CSx
Colorectum	PRS7	rs1811829	1	12828498	A	G	3.89E-04	PRS-CSx
Colorectum	PRS7	rs1812544	11	100028717	C	T	-1.81E-03	PRS-CSx
Colorectum	PRS7	rs1813100	18	9512985	G	A	-3.11E-04	PRS-CSx
Colorectum	PRS7	rs181359	22	21928641	G	A	9.68E-05	PRS-CSx
Colorectum	PRS7	rs181360	22	21928916	T	G	2.40E-04	PRS-CSx
Colorectum	PRS7	rs181362	22	21932068	C	T	2.21E-04	PRS-CSx
Colorectum	PRS7	rs1814271	16	49913093	A	G	1.30E-03	PRS-CSx
Colorectum	PRS7	rs1814272	16	49913220	T	C	8.75E-04	PRS-CSx
Colorectum	PRS7	rs1820167	5	52119356	C	T	2.74E-04	PRS-CSx
Colorectum	PRS7	rs1820488	15	48713996	G	T	1.75E-04	PRS-CSx
Colorectum	PRS7	rs1820682	5	150262142	C	T	-5.52E-04	PRS-CSx
Colorectum	PRS7	rs1822429	17	52702849	T	C	-2.27E-04	PRS-CSx
Colorectum	PRS7	rs1822738	12	46487024	G	T	5.44E-04	PRS-CSx
Colorectum	PRS7	rs1824212	3	21650648	G	T	-5.17E-04	PRS-CSx
Colorectum	PRS7	rs182532	1	1287040	T	C	6.69E-04	PRS-CSx
Colorectum	PRS7	rs1826106	5	57337744	A	G	4.02E-04	PRS-CSx

Colorectum	PRS7	rs182784	20	55824533	G	A	-4.01E-04	PRS-CSx
Colorectum	PRS7	rs1828705	4	69626404	A	C	-2.46E-04	PRS-CSx
Colorectum	PRS7	rs1831564	13	92457768	G	A	2.68E-04	PRS-CSx
Colorectum	PRS7	rs1832325	9	33662389	G	A	-2.00E-04	PRS-CSx
Colorectum	PRS7	rs1834735	13	49459540	T	C	-6.05E-04	PRS-CSx
Colorectum	PRS7	rs1834903	5	134589155	C	T	3.58E-04	PRS-CSx
Colorectum	PRS7	rs183646	12	46273882	G	T	-1.27E-04	PRS-CSx
Colorectum	PRS7	rs1838164	17	52662654	T	C	-2.85E-04	PRS-CSx
Colorectum	PRS7	rs1838166	17	52662561	G	A	-4.74E-04	PRS-CSx
Colorectum	PRS7	rs1839201	2	207183789	T	C	-2.56E-04	PRS-CSx
Colorectum	PRS7	rs1840422	11	16183252	G	A	4.56E-04	PRS-CSx
Colorectum	PRS7	rs1841828	4	22431406	C	T	-3.09E-04	PRS-CSx
Colorectum	PRS7	rs1842076	5	40237018	C	T	1.51E-03	PRS-CSx
Colorectum	PRS7	rs1842077	5	40236648	A	G	2.85E-03	PRS-CSx
Colorectum	PRS7	rs1843834	2	225558042	A	G	1.93E-04	PRS-CSx
Colorectum	PRS7	rs184580	15	76327581	T	C	-1.26E-04	PRS-CSx
Colorectum	PRS7	rs1846158	1	40781038	A	G	7.47E-04	PRS-CSx
Colorectum	PRS7	rs1846976	5	39644604	G	A	-1.13E-04	PRS-CSx
Colorectum	PRS7	rs1847638	11	49003921	T	C	-1.04E-04	PRS-CSx
Colorectum	PRS7	rs1849536	4	69644013	C	T	-1.55E-04	PRS-CSx
Colorectum	PRS7	rs1852686	2	145809250	C	T	-4.95E-04	PRS-CSx
Colorectum	PRS7	rs185435	5	72348272	G	A	-5.35E-04	PRS-CSx
Colorectum	PRS7	rs1855078	6	131351103	C	T	1.32E-03	PRS-CSx
Colorectum	PRS7	rs1856195	9	33696330	G	A	-1.42E-04	PRS-CSx
Colorectum	PRS7	rs1856197	9	33698114	C	T	-1.12E-04	PRS-CSx
Colorectum	PRS7	rs1856201	9	33661582	A	C	-3.52E-04	PRS-CSx
Colorectum	PRS7	rs1857649	9	21008725	A	G	3.24E-04	PRS-CSx
Colorectum	PRS7	rs1857850	3	72009691	T	G	-1.46E-04	PRS-CSx
Colorectum	PRS7	rs1857871	5	40015616	T	C	2.30E-05	PRS-CSx
Colorectum	PRS7	rs1858167	11	34816531	G	T	6.51E-04	PRS-CSx
Colorectum	PRS7	rs1858169	11	34813187	T	G	4.78E-04	PRS-CSx
Colorectum	PRS7	rs1859337	12	113319041	C	T	-2.82E-04	PRS-CSx
Colorectum	PRS7	rs1859902	20	52489205	G	A	1.08E-03	PRS-CSx
Colorectum	PRS7	rs1859955	19	57822369	A	G	-4.54E-04	PRS-CSx
Colorectum	PRS7	rs1860059	5	133847179	T	C	-2.20E-04	PRS-CSx
Colorectum	PRS7	rs1860067	5	129447248	C	T	-3.75E-05	PRS-CSx
Colorectum	PRS7	rs1860118	7	108237706	C	T	-5.11E-04	PRS-CSx
Colorectum	PRS7	rs1860710	14	73703112	T	C	3.76E-04	PRS-CSx
Colorectum	PRS7	rs1860872	7	21052503	C	T	6.45E-04	PRS-CSx
Colorectum	PRS7	rs1860994	18	53951278	T	G	-2.27E-04	PRS-CSx
Colorectum	PRS7	rs1861422	14	77168503	A	G	-8.77E-05	PRS-CSx
Colorectum	PRS7	rs1861662	16	50107383	T	C	-4.60E-04	PRS-CSx
Colorectum	PRS7	rs1862897	2	96015741	T	C	-1.43E-04	PRS-CSx
Colorectum	PRS7	rs1863808	2	137334985	T	C	5.09E-04	PRS-CSx
Colorectum	PRS7	rs1864167	14	81637074	A	G	-3.26E-04	PRS-CSx
Colorectum	PRS7	rs1864255	5	139974473	G	T	5.71E-04	PRS-CSx
Colorectum	PRS7	rs186451	5	55395112	T	C	6.97E-04	PRS-CSx
Colorectum	PRS7	rs1864864	7	42506780	T	C	1.23E-03	PRS-CSx
Colorectum	PRS7	rs1865147	12	109432616	T	G	1.89E-03	PRS-CSx
Colorectum	PRS7	rs1866136	2	240678202	G	T	1.03E-03	PRS-CSx
Colorectum	PRS7	rs1866282	8	4679752	C	T	-1.42E-03	PRS-CSx
Colorectum	PRS7	rs1866569	7	45165116	C	A	-1.53E-04	PRS-CSx
Colorectum	PRS7	rs1866571	7	45173384	G	T	-4.07E-04	PRS-CSx
Colorectum	PRS7	rs1866572	7	45184555	C	T	-1.00E-03	PRS-CSx
Colorectum	PRS7	rs1866788	8	101226443	A	G	-1.00E-05	PRS-CSx
Colorectum	PRS7	rs1866920	15	71656709	T	C	4.86E-04	PRS-CSx

Colorectum	PRS7	rs1867485	16	86681031	G	A	-4.22E-04	PRS-CSx
Colorectum	PRS7	rs1867746	2	137411077	G	A	1.10E-03	PRS-CSx
Colorectum	PRS7	rs1867840	8	117799012	A	G	1.53E-03	PRS-CSx
Colorectum	PRS7	rs1868444	15	64335225	C	T	9.09E-05	PRS-CSx
Colorectum	PRS7	rs1868582	2	62405081	G	A	-7.53E-05	PRS-CSx
Colorectum	PRS7	rs1868583	2	62404984	G	A	2.41E-05	PRS-CSx
Colorectum	PRS7	rs1869300	2	130217767	C	T	-3.12E-04	PRS-CSx
Colorectum	PRS7	rs1869607	8	98991281	T	C	-4.72E-04	PRS-CSx
Colorectum	PRS7	rs187089	8	15646620	C	T	-3.66E-04	PRS-CSx
Colorectum	PRS7	rs1872020	14	85283933	G	A	1.36E-03	PRS-CSx
Colorectum	PRS7	rs1872621	2	240474582	G	A	1.12E-03	PRS-CSx
Colorectum	PRS7	rs1872896	11	47160623	T	C	3.22E-04	PRS-CSx
Colorectum	PRS7	rs1873038	3	173537641	T	C	2.69E-04	PRS-CSx
Colorectum	PRS7	rs1873039	3	173511292	A	G	2.94E-04	PRS-CSx
Colorectum	PRS7	rs1874894	12	115886306	T	C	1.43E-03	PRS-CSx
Colorectum	PRS7	rs1874903	12	115950227	T	C	4.75E-04	PRS-CSx
Colorectum	PRS7	rs1875402	2	95848354	G	A	-9.13E-05	PRS-CSx
Colorectum	PRS7	rs1875513	5	14657062	G	A	-2.52E-04	PRS-CSx
Colorectum	PRS7	rs1875899	4	151385976	A	G	-3.75E-04	PRS-CSx
Colorectum	PRS7	rs1876206	15	48900586	C	T	1.02E-03	PRS-CSx
Colorectum	PRS7	rs1876811	2	48488501	T	C	6.19E-05	PRS-CSx
Colorectum	PRS7	rs1877207	4	26332690	C	A	5.22E-04	PRS-CSx
Colorectum	PRS7	rs1877256	3	73769502	A	G	-2.71E-04	PRS-CSx
Colorectum	PRS7	rs1877474	1	212757891	C	T	2.70E-04	PRS-CSx
Colorectum	PRS7	rs1878671	2	199594138	A	G	5.42E-04	PRS-CSx
Colorectum	PRS7	rs1878871	12	59435030	C	T	-9.60E-04	PRS-CSx
Colorectum	PRS7	rs1879948	2	176434777	A	G	3.87E-04	PRS-CSx
Colorectum	PRS7	rs1880479	3	41210298	G	T	9.06E-03	PRS-CSx
Colorectum	PRS7	rs1880883	12	80055766	G	A	4.27E-04	PRS-CSx
Colorectum	PRS7	rs1881578	10	13903948	G	A	-1.63E-03	PRS-CSx
Colorectum	PRS7	rs1881712	2	64402689	T	C	8.11E-04	PRS-CSx
Colorectum	PRS7	rs1882119	12	52447808	C	T	8.54E-04	PRS-CSx
Colorectum	PRS7	rs1883655	1	210531438	A	G	-9.21E-04	PRS-CSx
Colorectum	PRS7	rs1883740	20	42527683	C	T	-1.02E-03	PRS-CSx
Colorectum	PRS7	rs1883743	20	47369298	G	A	-8.24E-04	PRS-CSx
Colorectum	PRS7	rs1883744	20	47369447	G	A	-4.33E-04	PRS-CSx
Colorectum	PRS7	rs1884897	20	6612832	A	G	1.14E-03	PRS-CSx
Colorectum	PRS7	rs1885133	14	58737402	T	C	2.19E-04	PRS-CSx
Colorectum	PRS7	rs188541	5	37061261	A	G	-1.06E-04	PRS-CSx
Colorectum	PRS7	rs1885580	20	55880564	C	T	-3.50E-04	PRS-CSx
Colorectum	PRS7	rs1885601	14	81641210	T	C	-2.77E-04	PRS-CSx
Colorectum	PRS7	rs1886233	13	28866296	A	G	-6.82E-05	PRS-CSx
Colorectum	PRS7	rs1886450	13	73986628	A	G	-1.26E-03	PRS-CSx
Colorectum	PRS7	rs1886453	13	73990846	G	A	-6.04E-04	PRS-CSx
Colorectum	PRS7	rs1886532	13	34261344	C	T	-1.88E-04	PRS-CSx
Colorectum	PRS7	rs1886697	16	68716179	T	C	-3.43E-04	PRS-CSx
Colorectum	PRS7	rs1886730	1	2488608	T	C	-4.67E-04	PRS-CSx
Colorectum	PRS7	rs1887162	13	95835633	T	G	-8.98E-04	PRS-CSx
Colorectum	PRS7	rs1888233	20	60330913	G	A	4.59E-04	PRS-CSx
Colorectum	PRS7	rs1888354	13	78606250	G	A	-8.93E-04	PRS-CSx
Colorectum	PRS7	rs1889414	13	82086256	C	A	1.32E-03	PRS-CSx
Colorectum	PRS7	rs1890316	1	232420627	C	A	7.43E-04	PRS-CSx
Colorectum	PRS7	rs1890408	6	98330521	C	T	4.20E-04	PRS-CSx
Colorectum	PRS7	rs1890525	6	75027917	G	A	-2.74E-04	PRS-CSx
Colorectum	PRS7	rs1890535	1	164829035	G	A	1.58E-04	PRS-CSx
Colorectum	PRS7	rs1890705	14	51407894	C	T	-1.65E-04	PRS-CSx

Colorectum	PRS7	rs1890706	14	51407580	C	T	-4.38E-05	PRS-CSx
Colorectum	PRS7	rs1890995	1	218604678	G	A	-5.84E-04	PRS-CSx
Colorectum	PRS7	rs1891550	14	54000297	C	T	4.32E-04	PRS-CSx
Colorectum	PRS7	rs1892246	6	25764408	G	A	9.21E-05	PRS-CSx
Colorectum	PRS7	rs1892600	21	36009296	T	C	-1.54E-03	PRS-CSx
Colorectum	PRS7	rs1892894	11	120254684	T	C	-8.26E-05	PRS-CSx
Colorectum	PRS7	rs1893261	11	120198093	A	G	-6.58E-05	PRS-CSx
Colorectum	PRS7	rs1893414	18	68753328	C	T	-1.14E-03	PRS-CSx
Colorectum	PRS7	rs1894128	11	15098067	T	G	1.38E-04	PRS-CSx
Colorectum	PRS7	rs1895943	12	108240832	C	A	-3.74E-04	PRS-CSx
Colorectum	PRS7	rs1896877	17	52678748	G	A	-2.56E-04	PRS-CSx
Colorectum	PRS7	rs1897806	15	79562108	G	A	-5.48E-04	PRS-CSx
Colorectum	PRS7	rs1898858	4	44560636	A	G	-5.69E-04	PRS-CSx
Colorectum	PRS7	rs1899219	4	68744117	G	A	1.90E-04	PRS-CSx
Colorectum	PRS7	rs1900077	8	72250370	A	G	-8.56E-04	PRS-CSx
Colorectum	PRS7	rs1901187	17	38646147	C	T	-1.19E-03	PRS-CSx
Colorectum	PRS7	rs1901786	3	130021945	A	G	-8.15E-04	PRS-CSx
Colorectum	PRS7	rs1901830	11	34725028	T	G	-2.60E-04	PRS-CSx
Colorectum	PRS7	rs1902955	12	113548243	C	T	-5.98E-04	PRS-CSx
Colorectum	PRS7	rs1903003	4	89886297	C	T	3.62E-04	PRS-CSx
Colorectum	PRS7	rs1903431	3	112852657	G	A	-1.95E-04	PRS-CSx
Colorectum	PRS7	rs1903894	10	78907967	C	T	-9.19E-05	PRS-CSx
Colorectum	PRS7	rs1904863	8	136068443	T	G	6.09E-04	PRS-CSx
Colorectum	PRS7	rs1909043	1	104831653	A	G	2.22E-04	PRS-CSx
Colorectum	PRS7	rs1909359	1	88881202	A	G	-3.25E-04	PRS-CSx
Colorectum	PRS7	rs1910722	4	151323490	C	T	1.31E-04	PRS-CSx
Colorectum	PRS7	rs1914219	5	160443918	C	A	-6.54E-05	PRS-CSx
Colorectum	PRS7	rs1917353	7	124196444	C	T	8.17E-04	PRS-CSx
Colorectum	PRS7	rs1917606	7	46871794	C	A	9.54E-04	PRS-CSx
Colorectum	PRS7	rs191828	20	22373597	C	T	5.46E-04	PRS-CSx
Colorectum	PRS7	rs1919839	2	124909452	T	G	-6.15E-04	PRS-CSx
Colorectum	PRS7	rs1920045	12	54670398	C	T	-6.24E-04	PRS-CSx
Colorectum	PRS7	rs1920048	2	208033676	G	A	-6.13E-04	PRS-CSx
Colorectum	PRS7	rs1921249	2	76682789	G	A	-6.96E-04	PRS-CSx
Colorectum	PRS7	rs1921769	2	154099851	T	C	4.47E-04	PRS-CSx
Colorectum	PRS7	rs1921772	2	154211122	C	T	3.29E-04	PRS-CSx
Colorectum	PRS7	rs192219	5	40831202	T	C	2.81E-04	PRS-CSx
Colorectum	PRS7	rs1924303	13	38247506	T	C	1.34E-03	PRS-CSx
Colorectum	PRS7	rs1924815	13	74010099	T	C	-5.47E-04	PRS-CSx
Colorectum	PRS7	rs1924967	13	73008310	G	A	-6.36E-04	PRS-CSx
Colorectum	PRS7	rs1926029	10	104855670	A	G	-7.96E-05	PRS-CSx
Colorectum	PRS7	rs1927343	13	111053959	G	T	1.39E-04	PRS-CSx
Colorectum	PRS7	rs1928123	13	51119845	C	A	1.10E-03	PRS-CSx
Colorectum	PRS7	rs1928392	13	34131102	C	T	-2.80E-04	PRS-CSx
Colorectum	PRS7	rs1928393	13	34149388	A	C	-4.04E-04	PRS-CSx
Colorectum	PRS7	rs1928515	13	77984669	C	T	3.40E-05	PRS-CSx
Colorectum	PRS7	rs1930171	10	56200276	C	T	8.78E-04	PRS-CSx
Colorectum	PRS7	rs1930364	9	81586080	G	T	7.43E-04	PRS-CSx
Colorectum	PRS7	rs1930368	9	81584799	T	C	8.08E-04	PRS-CSx
Colorectum	PRS7	rs1930940	6	127303905	G	A	1.19E-04	PRS-CSx
Colorectum	PRS7	rs1930941	6	127316843	C	A	8.31E-05	PRS-CSx
Colorectum	PRS7	rs1930959	6	127284605	A	G	8.88E-05	PRS-CSx
Colorectum	PRS7	rs1932150	10	25814295	A	G	2.99E-04	PRS-CSx
Colorectum	PRS7	rs1933718	6	98341457	C	T	4.35E-04	PRS-CSx
Colorectum	PRS7	rs1934477	1	22664774	T	C	2.69E-04	PRS-CSx
Colorectum	PRS7	rs193495	5	127234944	T	C	-2.84E-04	PRS-CSx

Colorectum	PRS7	rs1935323	10	104877035	C	T	6.67E-06	PRS-CSx
Colorectum	PRS7	rs1935515	6	73535759	T	C	-6.49E-04	PRS-CSx
Colorectum	PRS7	rs1936078	1	164879202	G	A	6.64E-04	PRS-CSx
Colorectum	PRS7	rs1936096	1	164899933	A	G	5.42E-04	PRS-CSx
Colorectum	PRS7	rs193689	5	88710519	G	A	-6.97E-04	PRS-CSx
Colorectum	PRS7	rs1937127	6	25769872	A	C	1.21E-04	PRS-CSx
Colorectum	PRS7	rs1937395	10	56391303	A	G	-3.24E-03	PRS-CSx
Colorectum	PRS7	rs1938936	11	86389000	C	A	5.98E-04	PRS-CSx
Colorectum	PRS7	rs1939008	11	102656423	G	A	1.21E-03	PRS-CSx
Colorectum	PRS7	rs1939916	11	123672363	A	G	-1.13E-03	PRS-CSx
Colorectum	PRS7	rs194016	19	39817100	G	A	4.94E-04	PRS-CSx
Colorectum	PRS7	rs1940190	11	123668372	C	T	-7.69E-04	PRS-CSx
Colorectum	PRS7	rs1941036	11	56933839	T	C	2.20E-06	PRS-CSx
Colorectum	PRS7	rs1941410	11	120171165	G	A	-1.36E-03	PRS-CSx
Colorectum	PRS7	rs1942113	11	100334883	A	G	7.84E-04	PRS-CSx
Colorectum	PRS7	rs1942184	18	68746396	C	T	-1.38E-03	PRS-CSx
Colorectum	PRS7	rs1942931	11	117610854	A	G	9.55E-04	PRS-CSx
Colorectum	PRS7	rs1944117	11	111351184	C	T	-4.11E-04	PRS-CSx
Colorectum	PRS7	rs1945146	11	86291818	A	G	-5.45E-04	PRS-CSx
Colorectum	PRS7	rs1945321	11	21196180	T	G	-2.80E-04	PRS-CSx
Colorectum	PRS7	rs1945614	11	15102765	T	C	3.06E-04	PRS-CSx
Colorectum	PRS7	rs1946704	3	149397793	C	T	6.27E-05	PRS-CSx
Colorectum	PRS7	rs1946815	2	183298160	G	A	1.47E-04	PRS-CSx
Colorectum	PRS7	rs1947428	2	14048471	T	C	1.15E-04	PRS-CSx
Colorectum	PRS7	rs1948158	3	173552782	C	A	1.42E-04	PRS-CSx
Colorectum	PRS7	rs1948161	3	173491396	C	T	1.53E-04	PRS-CSx
Colorectum	PRS7	rs1948162	3	173557837	T	C	3.27E-04	PRS-CSx
Colorectum	PRS7	rs1948572	15	47739147	T	C	7.72E-04	PRS-CSx
Colorectum	PRS7	rs1949928	2	52721824	C	T	-3.65E-04	PRS-CSx
Colorectum	PRS7	rs195013	20	58586218	T	C	7.80E-04	PRS-CSx
Colorectum	PRS7	rs1950666	14	59447000	G	A	2.28E-04	PRS-CSx
Colorectum	PRS7	rs1950668	14	59425162	A	G	6.86E-05	PRS-CSx
Colorectum	PRS7	rs1951202	14	58779417	T	C	3.05E-04	PRS-CSx
Colorectum	PRS7	rs195147	4	105990602	A	G	5.41E-04	PRS-CSx
Colorectum	PRS7	rs195148	4	105988057	C	T	8.35E-04	PRS-CSx
Colorectum	PRS7	rs1951614	14	81672106	T	C	-3.49E-04	PRS-CSx
Colorectum	PRS7	rs1951865	14	54372841	T	G	1.27E-03	PRS-CSx
Colorectum	PRS7	rs1952586	14	64759419	C	T	4.30E-05	PRS-CSx
Colorectum	PRS7	rs1955023	11	134580920	A	C	-1.04E-03	PRS-CSx
Colorectum	PRS7	rs1956220	14	33075706	T	C	7.78E-04	PRS-CSx
Colorectum	PRS7	rs1956441	14	38029203	T	C	-2.98E-04	PRS-CSx
Colorectum	PRS7	rs1956526	14	68799787	A	C	2.94E-04	PRS-CSx
Colorectum	PRS7	rs1957292	14	92708728	C	T	7.25E-04	PRS-CSx
Colorectum	PRS7	rs1957604	14	54531723	C	T	9.59E-04	PRS-CSx
Colorectum	PRS7	rs1957632	14	54565438	T	C	7.09E-04	PRS-CSx
Colorectum	PRS7	rs1957641	14	54552557	G	A	6.60E-04	PRS-CSx
Colorectum	PRS7	rs1957842	14	54450284	A	G	5.67E-04	PRS-CSx
Colorectum	PRS7	rs1959046	14	96590183	C	T	-1.97E-03	PRS-CSx
Colorectum	PRS7	rs1959146	14	65923125	G	A	4.04E-04	PRS-CSx
Colorectum	PRS7	rs1959147	14	65923243	C	T	8.03E-05	PRS-CSx
Colorectum	PRS7	rs1959527	14	51372333	A	G	-5.11E-04	PRS-CSx
Colorectum	PRS7	rs196381	3	38412303	T	C	2.18E-03	PRS-CSx
Colorectum	PRS7	rs1965780	17	75734511	G	A	5.30E-04	PRS-CSx
Colorectum	PRS7	rs1966272	2	95926154	C	A	-1.75E-04	PRS-CSx
Colorectum	PRS7	rs1970346	6	141342927	C	T	-9.55E-04	PRS-CSx
Colorectum	PRS7	rs1971412	8	8273043	T	G	-3.88E-04	PRS-CSx

Colorectum	PRS7	rs1972844	8	23629252	T	C	-1.46E-03	PRS-CSx
Colorectum	PRS7	rs1972937	18	21682649	A	G	5.61E-04	PRS-CSx
Colorectum	PRS7	rs1976494	4	151481857	A	G	3.64E-04	PRS-CSx
Colorectum	PRS7	rs1977008	9	33663511	T	C	-2.40E-04	PRS-CSx
Colorectum	PRS7	rs1978487	16	31129942	T	C	3.90E-04	PRS-CSx
Colorectum	PRS7	rs1978707	5	151052659	G	A	4.29E-04	PRS-CSx
Colorectum	PRS7	rs1978947	11	20086764	T	G	1.07E-03	PRS-CSx
Colorectum	PRS7	rs1979368	18	9454478	G	A	-7.15E-04	PRS-CSx
Colorectum	PRS7	rs1979425	3	149779984	A	G	2.66E-03	PRS-CSx
Colorectum	PRS7	rs1979670	14	47358536	A	G	-4.83E-04	PRS-CSx
Colorectum	PRS7	rs1979855	20	6747607	G	A	1.21E-03	PRS-CSx
Colorectum	PRS7	rs1981596	7	16509086	A	C	-4.30E-04	PRS-CSx
Colorectum	PRS7	rs198223	14	57235592	C	T	8.31E-04	PRS-CSx
Colorectum	PRS7	rs1983961	13	109924849	G	A	-8.05E-04	PRS-CSx
Colorectum	PRS7	rs198450	11	61469473	T	C	1.70E-04	PRS-CSx
Colorectum	PRS7	rs198462	11	61524119	A	G	3.54E-05	PRS-CSx
Colorectum	PRS7	rs198476	11	61525730	A	G	4.87E-05	PRS-CSx
Colorectum	PRS7	rs1985268	5	127375931	C	T	-7.49E-04	PRS-CSx
Colorectum	PRS7	rs1986564	9	622545	C	A	3.29E-04	PRS-CSx
Colorectum	PRS7	rs1986684	11	111124487	C	T	-4.86E-04	PRS-CSx
Colorectum	PRS7	rs1988500	13	92462942	T	C	2.28E-04	PRS-CSx
Colorectum	PRS7	rs1990059	14	73569475	G	A	4.37E-04	PRS-CSx
Colorectum	PRS7	rs1990135	7	41924651	G	A	5.16E-04	PRS-CSx
Colorectum	PRS7	rs1990775	2	71610016	A	G	-4.24E-04	PRS-CSx
Colorectum	PRS7	rs1990966	19	33775326	C	T	1.32E-04	PRS-CSx
Colorectum	PRS7	rs1991098	2	41332646	T	G	7.82E-04	PRS-CSx
Colorectum	PRS7	rs1992098	1	64983804	G	A	3.50E-04	PRS-CSx
Colorectum	PRS7	rs1992292	7	45142145	C	T	-3.45E-04	PRS-CSx
Colorectum	PRS7	rs1992294	12	52157487	T	C	4.23E-03	PRS-CSx
Colorectum	PRS7	rs1992855	3	125909669	C	T	2.21E-05	PRS-CSx
Colorectum	PRS7	rs1992928	2	168944228	A	G	-2.61E-04	PRS-CSx
Colorectum	PRS7	rs1993205	11	44897700	C	T	1.03E-03	PRS-CSx
Colorectum	PRS7	rs1994634	4	105847746	T	C	-9.28E-04	PRS-CSx
Colorectum	PRS7	rs1994882	5	39806164	A	C	-9.92E-05	PRS-CSx
Colorectum	PRS7	rs1994988	3	78107336	G	A	-3.81E-04	PRS-CSx
Colorectum	PRS7	rs1995118	1	104820318	G	T	2.17E-04	PRS-CSx
Colorectum	PRS7	rs1995303	12	32647758	A	G	-1.15E-03	PRS-CSx
Colorectum	PRS7	rs1995446	18	75464724	G	A	1.07E-04	PRS-CSx
Colorectum	PRS7	rs1995613	8	128321943	G	T	-2.43E-04	PRS-CSx
Colorectum	PRS7	rs1996121	15	68181578	C	A	-3.45E-04	PRS-CSx
Colorectum	PRS7	rs1997397	3	169420117	C	T	2.42E-04	PRS-CSx
Colorectum	PRS7	rs1997450	2	76696261	G	A	8.37E-04	PRS-CSx
Colorectum	PRS7	rs1997644	22	38715222	A	G	-8.46E-04	PRS-CSx
Colorectum	PRS7	rs199774	20	22385317	G	A	-1.08E-04	PRS-CSx
Colorectum	PRS7	rs199778	20	22388154	T	G	-2.43E-04	PRS-CSx
Colorectum	PRS7	rs1998125	14	38081005	G	T	-4.46E-04	PRS-CSx
Colorectum	PRS7	rs1998151	20	6547287	T	C	5.71E-04	PRS-CSx
Colorectum	PRS7	rs1998190	20	55822649	A	C	-8.22E-04	PRS-CSx
Colorectum	PRS7	rs1998247	9	17631552	C	A	-5.52E-04	PRS-CSx
Colorectum	PRS7	rs199841	20	22361041	C	T	1.59E-04	PRS-CSx
Colorectum	PRS7	rs1998975	9	113087013	G	A	-4.81E-04	PRS-CSx
Colorectum	PRS7	rs1999120	6	158900185	A	G	3.29E-04	PRS-CSx
Colorectum	PRS7	rs1999345	14	54041955	T	C	-5.65E-04	PRS-CSx
Colorectum	PRS7	rs1999426	9	29435030	C	T	-4.40E-04	PRS-CSx
Colorectum	PRS7	rs1999555	9	31204481	A	G	-6.64E-05	PRS-CSx
Colorectum	PRS7	rs1999756	6	134089019	G	A	6.09E-04	PRS-CSx

Colorectum	PRS7	rs1999890	1	210510894	C	A	5.07E-04	PRS-CSx
Colorectum	PRS7	rs2000639	21	40280522	C	A	-5.38E-04	PRS-CSx
Colorectum	PRS7	rs200107	5	8655690	G	A	-5.90E-04	PRS-CSx
Colorectum	PRS7	rs2008612	8	101167695	C	T	-6.59E-05	PRS-CSx
Colorectum	PRS7	rs200956	6	27839746	C	T	-2.20E-04	PRS-CSx
Colorectum	PRS7	rs200991	6	27815494	A	C	-7.95E-05	PRS-CSx
Colorectum	PRS7	rs2010014	8	99068290	T	C	-4.23E-04	PRS-CSx
Colorectum	PRS7	rs2010316	20	17129082	A	G	4.69E-04	PRS-CSx
Colorectum	PRS7	rs2010962	18	53950927	G	T	-2.37E-04	PRS-CSx
Colorectum	PRS7	rs2011186	16	56705869	T	C	9.95E-04	PRS-CSx
Colorectum	PRS7	rs2013084	14	59290964	G	A	2.05E-04	PRS-CSx
Colorectum	PRS7	rs2013867	11	47260272	T	C	9.43E-05	PRS-CSx
Colorectum	PRS7	rs2014828	11	16118679	C	T	-2.66E-04	PRS-CSx
Colorectum	PRS7	rs2015069	8	117639532	C	T	-2.87E-03	PRS-CSx
Colorectum	PRS7	rs2015748	21	40202969	A	G	4.38E-04	PRS-CSx
Colorectum	PRS7	rs2017014	9	105492552	G	A	2.18E-04	PRS-CSx
Colorectum	PRS7	rs2017134	6	51875890	G	A	-1.16E-03	PRS-CSx
Colorectum	PRS7	rs2017143	1	2220649	T	C	-7.32E-04	PRS-CSx
Colorectum	PRS7	rs2017862	5	143819327	A	G	-1.42E-04	PRS-CSx
Colorectum	PRS7	rs2018854	15	48910591	G	A	6.93E-04	PRS-CSx
Colorectum	PRS7	rs2021947	13	32686956	T	C	1.42E-03	PRS-CSx
Colorectum	PRS7	rs2022212	6	69531957	A	G	-1.15E-03	PRS-CSx
Colorectum	PRS7	rs2022544	6	32321004	T	C	-3.23E-04	PRS-CSx
Colorectum	PRS7	rs2024847	6	85564139	C	T	7.37E-04	PRS-CSx
Colorectum	PRS7	rs2024962	20	6613051	G	A	7.08E-04	PRS-CSx
Colorectum	PRS7	rs2024967	11	33091567	C	T	-2.45E-04	PRS-CSx
Colorectum	PRS7	rs2025304	13	24636912	T	G	2.89E-05	PRS-CSx
Colorectum	PRS7	rs2026168	20	6418899	A	G	1.13E-04	PRS-CSx
Colorectum	PRS7	rs2027075	1	183073266	A	G	3.50E-04	PRS-CSx
Colorectum	PRS7	rs2027084	1	183110801	G	A	1.91E-04	PRS-CSx
Colorectum	PRS7	rs2027341	14	51372606	G	A	-3.03E-04	PRS-CSx
Colorectum	PRS7	rs2027342	14	51372590	T	C	-2.35E-04	PRS-CSx
Colorectum	PRS7	rs2027605	21	30726773	A	G	-2.38E-04	PRS-CSx
Colorectum	PRS7	rs2027856	6	32402705	A	G	-2.56E-03	PRS-CSx
Colorectum	PRS7	rs2028261	19	1907479	T	C	1.47E-03	PRS-CSx
Colorectum	PRS7	rs2028465	15	71659358	T	G	4.31E-04	PRS-CSx
Colorectum	PRS7	rs2028467	15	71637848	A	G	2.35E-04	PRS-CSx
Colorectum	PRS7	rs2028570	11	20104669	A	G	-7.96E-04	PRS-CSx
Colorectum	PRS7	rs2028660	18	9508997	T	C	-4.31E-04	PRS-CSx
Colorectum	PRS7	rs2028783	10	114316652	G	A	1.71E-03	PRS-CSx
Colorectum	PRS7	rs2028794	15	22919415	C	T	-1.26E-03	PRS-CSx
Colorectum	PRS7	rs2029298	11	47234718	T	C	9.77E-05	PRS-CSx
Colorectum	PRS7	rs2029335	3	173508116	A	G	2.74E-04	PRS-CSx
Colorectum	PRS7	rs2029393	6	74981125	C	T	-1.12E-04	PRS-CSx
Colorectum	PRS7	rs2029877	11	15029576	G	A	2.24E-04	PRS-CSx
Colorectum	PRS7	rs2030552	2	204433184	T	C	2.69E-04	PRS-CSx
Colorectum	PRS7	rs2030791	7	154621545	C	A	9.77E-04	PRS-CSx
Colorectum	PRS7	rs2030825	12	119472983	C	T	-1.37E-03	PRS-CSx
Colorectum	PRS7	rs203147	6	138589262	G	A	1.69E-04	PRS-CSx
Colorectum	PRS7	rs2031712	1	116063937	T	C	1.52E-03	PRS-CSx
Colorectum	PRS7	rs2032110	21	47904356	G	T	1.55E-04	PRS-CSx
Colorectum	PRS7	rs2032567	6	112457390	C	T	1.02E-03	PRS-CSx
Colorectum	PRS7	rs2032568	6	112457471	G	A	8.48E-04	PRS-CSx
Colorectum	PRS7	rs2032793	5	86450305	A	G	6.78E-05	PRS-CSx
Colorectum	PRS7	rs2032915	16	31117413	C	T	2.12E-04	PRS-CSx
Colorectum	PRS7	rs2033467	5	151039626	T	C	-2.31E-04	PRS-CSx

Colorectum	PRS7	rs2034018	14	85789364	C	T	-6.17E-04	PRS-CSx
Colorectum	PRS7	rs2034332	3	67951340	C	T	-5.22E-04	PRS-CSx
Colorectum	PRS7	rs2034730	2	3882177	G	A	-6.23E-04	PRS-CSx
Colorectum	PRS7	rs2034732	2	3882061	G	A	-6.90E-04	PRS-CSx
Colorectum	PRS7	rs2034733	2	3882007	G	A	-5.85E-04	PRS-CSx
Colorectum	PRS7	rs2034734	2	3881891	C	T	-4.86E-04	PRS-CSx
Colorectum	PRS7	rs2034817	8	29084560	G	A	8.67E-04	PRS-CSx
Colorectum	PRS7	rs2034879	15	72429989	A	G	-5.35E-04	PRS-CSx
Colorectum	PRS7	rs2035826	4	23698717	A	G	-7.19E-05	PRS-CSx
Colorectum	PRS7	rs2035912	4	83007475	C	T	2.48E-03	PRS-CSx
Colorectum	PRS7	rs2036282	3	112970157	G	A	8.29E-05	PRS-CSx
Colorectum	PRS7	rs2036610	5	40066219	C	T	3.62E-04	PRS-CSx
Colorectum	PRS7	rs2037815	2	202101715	A	G	6.70E-04	PRS-CSx
Colorectum	PRS7	rs2038062	22	43397966	A	G	4.43E-05	PRS-CSx
Colorectum	PRS7	rs2038171	20	49025587	G	A	-2.35E-04	PRS-CSx
Colorectum	PRS7	rs2038178	20	47303476	C	T	-7.09E-04	PRS-CSx
Colorectum	PRS7	rs2038931	1	92174415	A	G	-2.75E-04	PRS-CSx
Colorectum	PRS7	rs2039923	9	111598970	T	C	8.99E-04	PRS-CSx
Colorectum	PRS7	rs2039958	1	221351145	G	A	8.77E-05	PRS-CSx
Colorectum	PRS7	rs2040687	7	88828769	A	G	-1.72E-04	PRS-CSx
Colorectum	PRS7	rs2041110	19	57802824	A	G	-1.43E-03	PRS-CSx
Colorectum	PRS7	rs2041314	20	52360560	A	G	4.20E-03	PRS-CSx
Colorectum	PRS7	rs2042342	5	55969146	C	T	3.78E-03	PRS-CSx
Colorectum	PRS7	rs2042746	15	48729648	T	C	2.03E-04	PRS-CSx
Colorectum	PRS7	rs2043368	12	95479922	A	G	4.61E-04	PRS-CSx
Colorectum	PRS7	rs2043422	8	142236337	A	G	1.53E-03	PRS-CSx
Colorectum	PRS7	rs2043640	4	43057081	G	T	1.13E-03	PRS-CSx
Colorectum	PRS7	rs2043779	4	146447789	C	T	5.14E-04	PRS-CSx
Colorectum	PRS7	rs2044644	2	33817559	G	A	4.56E-04	PRS-CSx
Colorectum	PRS7	rs2045158	15	47575054	G	A	-3.12E-04	PRS-CSx
Colorectum	PRS7	rs2045517	4	89870964	C	T	3.94E-04	PRS-CSx
Colorectum	PRS7	rs2045909	19	38059282	C	T	-3.09E-04	PRS-CSx
Colorectum	PRS7	rs2046414	15	25937282	G	A	-1.25E-03	PRS-CSx
Colorectum	PRS7	rs2046720	3	173500571	G	A	3.10E-04	PRS-CSx
Colorectum	PRS7	rs2047220	15	101998264	T	C	2.89E-04	PRS-CSx
Colorectum	PRS7	rs2047264	1	201886402	A	G	8.44E-04	PRS-CSx
Colorectum	PRS7	rs2047397	16	86187534	T	G	6.26E-04	PRS-CSx
Colorectum	PRS7	rs2048011	5	39675505	C	T	5.71E-04	PRS-CSx
Colorectum	PRS7	rs2048968	2	199623902	T	C	3.64E-04	PRS-CSx
Colorectum	PRS7	rs2049589	12	80029516	G	T	3.75E-04	PRS-CSx
Colorectum	PRS7	rs2049590	12	80043160	A	C	2.63E-04	PRS-CSx
Colorectum	PRS7	rs204993	6	32155581	G	A	3.82E-05	PRS-CSx
Colorectum	PRS7	rs2050176	22	44488796	A	G	-8.18E-04	PRS-CSx
Colorectum	PRS7	rs2050778	10	9933991	G	A	2.30E-03	PRS-CSx
Colorectum	PRS7	rs2050780	10	9932748	C	T	2.57E-03	PRS-CSx
Colorectum	PRS7	rs2050789	9	33880690	G	A	2.65E-04	PRS-CSx
Colorectum	PRS7	rs2051934	7	41867259	C	T	5.27E-04	PRS-CSx
Colorectum	PRS7	rs2052074	17	59161646	G	A	-9.02E-05	PRS-CSx
Colorectum	PRS7	rs2052550	5	78272942	C	T	-1.28E-03	PRS-CSx
Colorectum	PRS7	rs205356	16	28108528	A	G	1.12E-04	PRS-CSx
Colorectum	PRS7	rs2054678	12	117824549	G	A	9.56E-04	PRS-CSx
Colorectum	PRS7	rs20558	1	183094547	T	C	2.01E-04	PRS-CSx
Colorectum	PRS7	rs2056123	3	157570795	C	A	3.13E-04	PRS-CSx
Colorectum	PRS7	rs20563	1	183085755	A	G	2.68E-04	PRS-CSx
Colorectum	PRS7	rs2056392	4	17106462	G	A	-5.52E-04	PRS-CSx
Colorectum	PRS7	rs2056393	4	17106331	T	C	-7.67E-04	PRS-CSx

Colorectum	PRS7	rs2056501	15	38576234	G	A	-6.10E-04	PRS-CSx
Colorectum	PRS7	rs2057191	14	99134511	T	C	-3.99E-04	PRS-CSx
Colorectum	PRS7	rs2057291	20	57472043	A	G	-3.24E-04	PRS-CSx
Colorectum	PRS7	rs2058813	16	50129454	T	C	-4.07E-04	PRS-CSx
Colorectum	PRS7	rs2058992	2	214932659	G	A	-1.52E-03	PRS-CSx
Colorectum	PRS7	rs2059202	5	52097183	A	G	-1.08E-03	PRS-CSx
Colorectum	PRS7	rs2059254	16	68817439	T	C	-3.57E-04	PRS-CSx
Colorectum	PRS7	rs2059258	16	86581366	G	A	-5.30E-04	PRS-CSx
Colorectum	PRS7	rs2059405	12	46175469	G	A	-2.27E-04	PRS-CSx
Colorectum	PRS7	rs2060147	11	83341316	G	A	2.48E-04	PRS-CSx
Colorectum	PRS7	rs2060675	10	111773423	C	T	-2.51E-04	PRS-CSx
Colorectum	PRS7	rs2060775	8	128378626	T	C	2.03E-04	PRS-CSx
Colorectum	PRS7	rs2061392	5	11908641	T	C	-6.14E-04	PRS-CSx
Colorectum	PRS7	rs2061742	4	76480880	G	A	-5.39E-04	PRS-CSx
Colorectum	PRS7	rs2061847	8	2158537	C	T	1.31E-03	PRS-CSx
Colorectum	PRS7	rs2062319	3	101050546	A	G	-1.11E-03	PRS-CSx
Colorectum	PRS7	rs2062736	4	40516914	C	T	1.97E-03	PRS-CSx
Colorectum	PRS7	rs2062819	11	19097685	G	A	-7.56E-04	PRS-CSx
Colorectum	PRS7	rs2063224	5	56320577	G	A	1.43E-03	PRS-CSx
Colorectum	PRS7	rs2063826	15	71583413	C	T	3.62E-04	PRS-CSx
Colorectum	PRS7	rs2064084	22	29412257	C	T	-3.09E-04	PRS-CSx
Colorectum	PRS7	rs2064912	14	68807132	G	A	-2.58E-04	PRS-CSx
Colorectum	PRS7	rs2065695	10	29101875	T	G	6.53E-04	PRS-CSx
Colorectum	PRS7	rs2066790	4	39318706	G	A	-9.68E-06	PRS-CSx
Colorectum	PRS7	rs2066951	6	29454489	G	T	9.37E-05	PRS-CSx
Colorectum	PRS7	rs2067693	7	83759570	C	T	-5.43E-04	PRS-CSx
Colorectum	PRS7	rs2067986	9	101816399	T	C	-4.81E-04	PRS-CSx
Colorectum	PRS7	rs2068244	2	225440011	G	A	1.65E-04	PRS-CSx
Colorectum	PRS7	rs2068428	9	1792147	T	C	1.29E-03	PRS-CSx
Colorectum	PRS7	rs2068888	10	94839642	G	A	-1.26E-03	PRS-CSx
Colorectum	PRS7	rs2068943	22	43356311	C	T	9.04E-05	PRS-CSx
Colorectum	PRS7	rs2068944	22	43356442	T	G	1.11E-04	PRS-CSx
Colorectum	PRS7	rs2068991	4	146423515	G	T	8.01E-04	PRS-CSx
Colorectum	PRS7	rs2069456	7	150752608	G	T	-7.06E-04	PRS-CSx
Colorectum	PRS7	rs2069662	5	75916603	A	G	-9.45E-04	PRS-CSx
Colorectum	PRS7	rs2069698	5	75916887	T	C	-2.21E-03	PRS-CSx
Colorectum	PRS7	rs2069776	4	123371976	G	A	5.40E-04	PRS-CSx
Colorectum	PRS7	rs2070011	4	155511897	T	C	3.14E-04	PRS-CSx
Colorectum	PRS7	rs2070063	2	64862055	G	A	1.54E-03	PRS-CSx
Colorectum	PRS7	rs2070215	7	99696797	C	T	-2.03E-04	PRS-CSx
Colorectum	PRS7	rs2070302	10	33411435	T	C	3.23E-04	PRS-CSx
Colorectum	PRS7	rs2070368	21	36080398	T	C	-6.35E-04	PRS-CSx
Colorectum	PRS7	rs2070429	21	47980570	A	G	1.01E-04	PRS-CSx
Colorectum	PRS7	rs2070431	21	47983543	T	C	1.92E-04	PRS-CSx
Colorectum	PRS7	rs2070464	22	24183875	G	A	5.50E-04	PRS-CSx
Colorectum	PRS7	rs2070512	22	21949411	A	C	2.22E-04	PRS-CSx
Colorectum	PRS7	rs2070529	21	40194028	T	C	-7.00E-04	PRS-CSx
Colorectum	PRS7	rs2070699	6	12292772	G	T	-6.69E-03	PRS-CSx
Colorectum	PRS7	rs2071047	14	54418411	A	G	2.55E-03	PRS-CSx
Colorectum	PRS7	rs2071279	6	32164874	A	C	2.08E-05	PRS-CSx
Colorectum	PRS7	rs2071286	6	32179896	T	C	-1.21E-04	PRS-CSx
Colorectum	PRS7	rs2071943	6	12295814	A	G	-1.06E-03	PRS-CSx
Colorectum	PRS7	rs2072114	11	61605215	G	A	-2.72E-04	PRS-CSx
Colorectum	PRS7	rs2072133	12	113409260	C	T	-3.18E-04	PRS-CSx
Colorectum	PRS7	rs2072209	7	107592198	G	A	1.54E-03	PRS-CSx
Colorectum	PRS7	rs2072849	6	25554148	G	T	-6.03E-04	PRS-CSx

Colorectum	PRS7	rs2072858	22	40708679	C	T	-1.11E-04	PRS-CSx
Colorectum	PRS7	rs2072883	22	43435668	G	A	2.58E-04	PRS-CSx
Colorectum	PRS7	rs2072884	22	43436169	G	A	6.10E-04	PRS-CSx
Colorectum	PRS7	rs2072894	6	29713036	T	C	1.62E-04	PRS-CSx
Colorectum	PRS7	rs2072898	6	29692729	G	T	2.36E-04	PRS-CSx
Colorectum	PRS7	rs2073048	6	32335433	A	G	-3.13E-04	PRS-CSx
Colorectum	PRS7	rs2073148	6	29430170	C	T	3.30E-04	PRS-CSx
Colorectum	PRS7	rs2073199	22	43275250	G	A	2.86E-04	PRS-CSx
Colorectum	PRS7	rs2073337	10	101567426	G	A	-9.20E-05	PRS-CSx
Colorectum	PRS7	rs2073818	9	136918662	C	A	-9.87E-04	PRS-CSx
Colorectum	PRS7	rs2074465	6	29408476	G	A	4.77E-04	PRS-CSx
Colorectum	PRS7	rs2074791	19	3614136	T	C	4.08E-04	PRS-CSx
Colorectum	PRS7	rs2074852	19	39880011	A	G	1.51E-03	PRS-CSx
Colorectum	PRS7	rs2074986	10	118028614	C	T	1.16E-03	PRS-CSx
Colorectum	PRS7	rs2075643	14	51383432	A	G	1.60E-04	PRS-CSx
Colorectum	PRS7	rs2075759	17	7292685	A	G	3.29E-03	PRS-CSx
Colorectum	PRS7	rs2076112	22	38538822	T	C	5.87E-05	PRS-CSx
Colorectum	PRS7	rs2076154	22	43465529	C	T	2.62E-04	PRS-CSx
Colorectum	PRS7	rs2076157	22	43289236	C	T	3.77E-04	PRS-CSx
Colorectum	PRS7	rs2076158	22	43289738	C	T	3.39E-04	PRS-CSx
Colorectum	PRS7	rs2076177	6	29693113	T	C	2.70E-04	PRS-CSx
Colorectum	PRS7	rs2076484	6	29524003	G	A	2.89E-04	PRS-CSx
Colorectum	PRS7	rs2076486	6	29523872	G	A	2.82E-04	PRS-CSx
Colorectum	PRS7	rs2076513	6	1591011	G	T	-9.11E-04	PRS-CSx
Colorectum	PRS7	rs2076974	10	111820311	T	C	-2.36E-04	PRS-CSx
Colorectum	PRS7	rs2078203	21	47881309	A	G	4.08E-04	PRS-CSx
Colorectum	PRS7	rs2079112	4	105763065	T	C	1.01E-04	PRS-CSx
Colorectum	PRS7	rs2081045	19	33789010	A	G	-5.26E-04	PRS-CSx
Colorectum	PRS7	rs2081430	8	120186415	A	C	-1.34E-04	PRS-CSx
Colorectum	PRS7	rs2083117	15	97079333	A	G	2.48E-03	PRS-CSx
Colorectum	PRS7	rs2083628	2	76163222	G	A	-6.21E-04	PRS-CSx
Colorectum	PRS7	rs2084007	5	133891282	T	C	2.66E-04	PRS-CSx
Colorectum	PRS7	rs2085525	17	21097276	T	G	-4.82E-04	PRS-CSx
Colorectum	PRS7	rs2085600	4	89779909	A	G	1.68E-04	PRS-CSx
Colorectum	PRS7	rs2087215	11	47144542	A	C	1.69E-04	PRS-CSx
Colorectum	PRS7	rs2090166	1	64750226	C	T	5.06E-04	PRS-CSx
Colorectum	PRS7	rs2091181	19	51592564	T	G	1.09E-03	PRS-CSx
Colorectum	PRS7	rs2091331	10	73741706	G	A	-1.91E-04	PRS-CSx
Colorectum	PRS7	rs2093056	10	33413449	G	A	2.57E-04	PRS-CSx
Colorectum	PRS7	rs2093658	1	167355192	C	T	5.40E-04	PRS-CSx
Colorectum	PRS7	rs2093943	13	74007186	C	T	-1.26E-03	PRS-CSx
Colorectum	PRS7	rs2096884	11	120173354	A	G	-5.41E-04	PRS-CSx
Colorectum	PRS7	rs2097215	19	45875787	C	T	-7.89E-04	PRS-CSx
Colorectum	PRS7	rs2100142	3	71112560	T	C	-1.02E-03	PRS-CSx
Colorectum	PRS7	rs2100782	4	115451608	G	A	1.56E-04	PRS-CSx
Colorectum	PRS7	rs2101582	6	26622734	C	T	-9.81E-05	PRS-CSx
Colorectum	PRS7	rs2101955	16	86199509	A	C	-3.26E-04	PRS-CSx
Colorectum	PRS7	rs210326	14	54070912	T	C	-5.13E-04	PRS-CSx
Colorectum	PRS7	rs210327	14	54068781	A	C	-1.18E-03	PRS-CSx
Colorectum	PRS7	rs210328	14	54065665	T	C	-2.78E-04	PRS-CSx
Colorectum	PRS7	rs210330	14	54059001	C	T	-4.36E-04	PRS-CSx
Colorectum	PRS7	rs210363	14	54162080	G	A	1.03E-03	PRS-CSx
Colorectum	PRS7	rs210364	14	54138623	G	A	5.14E-04	PRS-CSx
Colorectum	PRS7	rs210365	14	54138415	A	G	3.08E-04	PRS-CSx
Colorectum	PRS7	rs210373	14	54156090	C	A	8.05E-04	PRS-CSx
Colorectum	PRS7	rs210388	14	54099700	G	A	-1.13E-03	PRS-CSx

Colorectum	PRS7	rs2103889	14	50538595	C	T	6.80E-04	PRS-CSx
Colorectum	PRS7	rs2104064	6	117793798	C	T	2.11E-04	PRS-CSx
Colorectum	PRS7	rs2105274	14	26310229	A	G	-5.68E-04	PRS-CSx
Colorectum	PRS7	rs210610	6	117834444	T	C	2.64E-04	PRS-CSx
Colorectum	PRS7	rs2106119	9	22017550	A	G	5.85E-04	PRS-CSx
Colorectum	PRS7	rs2106292	7	88122038	G	T	-2.99E-04	PRS-CSx
Colorectum	PRS7	rs2106406	12	111394384	A	G	3.02E-04	PRS-CSx
Colorectum	PRS7	rs2106407	12	111394327	C	T	4.15E-04	PRS-CSx
Colorectum	PRS7	rs210648	6	117844072	G	A	4.49E-04	PRS-CSx
Colorectum	PRS7	rs210653	6	117856627	T	G	7.54E-04	PRS-CSx
Colorectum	PRS7	rs2106854	5	131769174	T	C	-6.49E-04	PRS-CSx
Colorectum	PRS7	rs2106900	7	114122506	T	C	3.67E-04	PRS-CSx
Colorectum	PRS7	rs2107189	6	29451168	A	G	2.65E-04	PRS-CSx
Colorectum	PRS7	rs2107351	7	99535951	C	T	-4.56E-04	PRS-CSx
Colorectum	PRS7	rs2110323	7	76837419	T	C	-4.39E-04	PRS-CSx
Colorectum	PRS7	rs2111592	2	208049581	A	G	-1.41E-04	PRS-CSx
Colorectum	PRS7	rs2111593	2	208046998	T	C	-4.21E-04	PRS-CSx
Colorectum	PRS7	rs2111829	14	78197283	C	T	-7.62E-04	PRS-CSx
Colorectum	PRS7	rs2111862	8	30718597	C	T	2.19E-04	PRS-CSx
Colorectum	PRS7	rs2112068	11	99968225	A	G	2.51E-03	PRS-CSx
Colorectum	PRS7	rs2112157	5	143620425	T	G	7.36E-05	PRS-CSx
Colorectum	PRS7	rs2112176	19	18455679	C	A	1.57E-03	PRS-CSx
Colorectum	PRS7	rs2112637	5	150182434	A	G	3.52E-04	PRS-CSx
Colorectum	PRS7	rs2112843	19	12549655	A	G	6.46E-05	PRS-CSx
Colorectum	PRS7	rs2113417	2	95999856	G	A	-1.35E-04	PRS-CSx
Colorectum	PRS7	rs2114181	4	76015851	T	C	1.02E-03	PRS-CSx
Colorectum	PRS7	rs211455	6	33328518	G	A	-3.55E-05	PRS-CSx
Colorectum	PRS7	rs2114616	14	59384353	C	T	2.41E-04	PRS-CSx
Colorectum	PRS7	rs2114844	12	46393161	T	C	-2.69E-04	PRS-CSx
Colorectum	PRS7	rs2116526	15	27232528	G	T	3.49E-04	PRS-CSx
Colorectum	PRS7	rs2118438	4	146428384	G	A	1.54E-03	PRS-CSx
Colorectum	PRS7	rs2118611	15	67401466	T	C	2.23E-03	PRS-CSx
Colorectum	PRS7	rs2118612	15	67400490	T	C	3.14E-03	PRS-CSx
Colorectum	PRS7	rs2119083	2	35394105	G	T	1.07E-03	PRS-CSx
Colorectum	PRS7	rs2120602	12	10845947	C	T	-1.01E-03	PRS-CSx
Colorectum	PRS7	rs2120857	5	40274458	G	A	7.69E-04	PRS-CSx
Colorectum	PRS7	rs2120991	12	54270228	A	C	-1.13E-03	PRS-CSx
Colorectum	PRS7	rs2121468	11	92932229	C	T	1.54E-04	PRS-CSx
Colorectum	PRS7	rs2124132	15	47553498	T	C	-2.19E-04	PRS-CSx
Colorectum	PRS7	rs2124420	8	29133338	G	A	6.55E-04	PRS-CSx
Colorectum	PRS7	rs2124612	1	99552858	T	C	-8.51E-04	PRS-CSx
Colorectum	PRS7	rs2125617	15	31760986	G	A	4.42E-04	PRS-CSx
Colorectum	PRS7	rs2125737	6	43327536	C	T	-4.48E-04	PRS-CSx
Colorectum	PRS7	rs2125738	6	43327750	A	G	-3.90E-04	PRS-CSx
Colorectum	PRS7	rs2127355	10	62534216	A	G	4.94E-04	PRS-CSx
Colorectum	PRS7	rs2127357	10	73745599	T	C	-3.51E-04	PRS-CSx
Colorectum	PRS7	rs2128045	2	183107413	G	A	2.06E-04	PRS-CSx
Colorectum	PRS7	rs2129588	18	69703287	T	C	-3.59E-04	PRS-CSx
Colorectum	PRS7	rs213011	1	21640395	A	G	-4.26E-04	PRS-CSx
Colorectum	PRS7	rs213012	1	21642413	T	C	-4.65E-04	PRS-CSx
Colorectum	PRS7	rs213023	1	21649908	G	A	-3.95E-04	PRS-CSx
Colorectum	PRS7	rs213025	1	21651575	T	G	-2.54E-04	PRS-CSx
Colorectum	PRS7	rs213037	1	21655884	A	G	-4.52E-04	PRS-CSx
Colorectum	PRS7	rs213052	1	21622403	C	T	-3.83E-04	PRS-CSx
Colorectum	PRS7	rs2131230	4	163086157	G	T	3.51E-04	PRS-CSx
Colorectum	PRS7	rs2131887	1	221007855	T	C	-5.42E-04	PRS-CSx

Colorectum	PRS7	rs213194	6	33195604	A	G	1.05E-04	PRS-CSx
Colorectum	PRS7	rs2132466	11	15012110	T	C	3.71E-04	PRS-CSx
Colorectum	PRS7	rs2133045	1	219005972	G	A	-7.08E-04	PRS-CSx
Colorectum	PRS7	rs2133189	1	222814442	C	T	5.55E-04	PRS-CSx
Colorectum	PRS7	rs2134959	4	151372954	G	A	1.82E-04	PRS-CSx
Colorectum	PRS7	rs2136080	4	98471647	C	T	1.14E-03	PRS-CSx
Colorectum	PRS7	rs2136932	6	165314986	A	G	1.08E-03	PRS-CSx
Colorectum	PRS7	rs2137064	3	73750993	G	A	-3.24E-04	PRS-CSx
Colorectum	PRS7	rs2137111	15	77851535	A	G	-5.60E-04	PRS-CSx
Colorectum	PRS7	rs2138046	12	101255582	G	A	-2.88E-04	PRS-CSx
Colorectum	PRS7	rs2138390	2	26112833	G	A	-3.05E-04	PRS-CSx
Colorectum	PRS7	rs2139930	12	51089287	T	G	5.07E-04	PRS-CSx
Colorectum	PRS7	rs2141026	8	16350300	T	C	1.19E-03	PRS-CSx
Colorectum	PRS7	rs2141601	3	152324110	G	T	3.02E-04	PRS-CSx
Colorectum	PRS7	rs2141602	3	152324136	C	T	2.46E-04	PRS-CSx
Colorectum	PRS7	rs214250	16	348222	T	C	-3.61E-04	PRS-CSx
Colorectum	PRS7	rs214251	16	347326	A	G	-6.49E-04	PRS-CSx
Colorectum	PRS7	rs214252	16	347184	G	A	-7.86E-04	PRS-CSx
Colorectum	PRS7	rs2142648	6	104440108	T	C	2.40E-03	PRS-CSx
Colorectum	PRS7	rs2142739	6	11273577	G	A	6.44E-04	PRS-CSx
Colorectum	PRS7	rs2143250	20	7761983	G	T	4.52E-04	PRS-CSx
Colorectum	PRS7	rs2143462	6	32335204	A	G	1.21E-03	PRS-CSx
Colorectum	PRS7	rs2143563	20	47320211	G	A	-1.18E-04	PRS-CSx
Colorectum	PRS7	rs2144112	14	63717832	C	T	1.02E-03	PRS-CSx
Colorectum	PRS7	rs2144425	6	29344939	G	A	-1.48E-04	PRS-CSx
Colorectum	PRS7	rs2144426	6	29345029	G	A	-2.63E-04	PRS-CSx
Colorectum	PRS7	rs2145270	20	6621685	C	T	2.77E-04	PRS-CSx
Colorectum	PRS7	rs2145598	14	58794001	A	G	8.83E-05	PRS-CSx
Colorectum	PRS7	rs2147102	14	54369046	A	C	1.99E-03	PRS-CSx
Colorectum	PRS7	rs2147167	13	37467404	A	G	3.14E-03	PRS-CSx
Colorectum	PRS7	rs2148198	10	104915310	A	G	-3.08E-04	PRS-CSx
Colorectum	PRS7	rs2148529	13	95838784	G	T	-3.77E-04	PRS-CSx
Colorectum	PRS7	rs2151280	9	22034719	G	A	2.89E-04	PRS-CSx
Colorectum	PRS7	rs215276	7	83193369	G	A	-1.13E-03	PRS-CSx
Colorectum	PRS7	rs2153020	6	88482765	A	G	4.03E-04	PRS-CSx
Colorectum	PRS7	rs215340	12	47732638	A	G	-4.60E-04	PRS-CSx
Colorectum	PRS7	rs2155453	11	69942324	C	T	2.22E-03	PRS-CSx
Colorectum	PRS7	rs2155935	11	74339577	T	G	8.47E-04	PRS-CSx
Colorectum	PRS7	rs2156042	1	116097043	G	A	6.08E-04	PRS-CSx
Colorectum	PRS7	rs2157051	6	32658624	G	A	6.71E-04	PRS-CSx
Colorectum	PRS7	rs2157719	9	22033366	C	T	7.17E-04	PRS-CSx
Colorectum	PRS7	rs2157743	7	92767502	C	T	-5.38E-04	PRS-CSx
Colorectum	PRS7	rs2158241	12	113124351	A	G	4.04E-04	PRS-CSx
Colorectum	PRS7	rs2158564	7	45038881	G	A	-1.82E-04	PRS-CSx
Colorectum	PRS7	rs2158839	7	107495091	T	C	-1.01E-03	PRS-CSx
Colorectum	PRS7	rs2159120	5	129398757	C	T	-1.96E-04	PRS-CSx
Colorectum	PRS7	rs2160519	12	13787846	C	T	8.52E-04	PRS-CSx
Colorectum	PRS7	rs2160989	12	96052228	T	G	-2.47E-03	PRS-CSx
Colorectum	PRS7	rs216123	5	149460553	G	A	7.82E-04	PRS-CSx
Colorectum	PRS7	rs2161877	12	116172987	G	T	2.39E-04	PRS-CSx
Colorectum	PRS7	rs2161936	15	95118265	C	T	-1.83E-03	PRS-CSx
Colorectum	PRS7	rs2162709	5	134526551	C	T	1.73E-04	PRS-CSx
Colorectum	PRS7	rs2162840	6	39093551	T	G	1.09E-03	PRS-CSx
Colorectum	PRS7	rs2163616	14	59198493	C	A	-1.59E-03	PRS-CSx
Colorectum	PRS7	rs216364	9	33818257	C	T	-2.96E-04	PRS-CSx
Colorectum	PRS7	rs2163786	5	139986490	T	C	4.78E-04	PRS-CSx

Colorectum	PRS7	rs2164203	7	130853246	C	T	1.88E-05	PRS-CSx
Colorectum	PRS7	rs216493	11	16834031	A	G	2.86E-03	PRS-CSx
Colorectum	PRS7	rs2165193	1	48213641	C	T	1.68E-03	PRS-CSx
Colorectum	PRS7	rs216546	18	54025483	A	G	-2.99E-04	PRS-CSx
Colorectum	PRS7	rs2165488	15	71678798	G	A	1.66E-04	PRS-CSx
Colorectum	PRS7	rs2166517	2	200190213	T	C	-1.40E-04	PRS-CSx
Colorectum	PRS7	rs2166801	3	77526031	T	G	1.69E-05	PRS-CSx
Colorectum	PRS7	rs2167079	11	47270255	C	T	4.15E-05	PRS-CSx
Colorectum	PRS7	rs2167494	4	39216221	T	C	1.58E-04	PRS-CSx
Colorectum	PRS7	rs2169385	8	9206678	G	A	-1.12E-03	PRS-CSx
Colorectum	PRS7	rs2169703	8	13938197	G	T	4.76E-03	PRS-CSx
Colorectum	PRS7	rs2170006	10	62534614	G	A	6.73E-04	PRS-CSx
Colorectum	PRS7	rs2170169	1	38408164	A	G	-8.15E-04	PRS-CSx
Colorectum	PRS7	rs2170402	2	183107485	A	G	1.02E-04	PRS-CSx
Colorectum	PRS7	rs2170403	2	183106997	G	T	1.98E-04	PRS-CSx
Colorectum	PRS7	rs2170665	18	10952343	G	A	-2.22E-03	PRS-CSx
Colorectum	PRS7	rs2172935	1	201826340	T	C	3.54E-04	PRS-CSx
Colorectum	PRS7	rs2173033	1	59850907	A	G	4.39E-04	PRS-CSx
Colorectum	PRS7	rs2175094	2	28587020	A	G	4.49E-04	PRS-CSx
Colorectum	PRS7	rs217797	5	37318295	T	G	1.45E-04	PRS-CSx
Colorectum	PRS7	rs2178824	9	33765513	C	T	-3.43E-04	PRS-CSx
Colorectum	PRS7	rs2179466	20	7729273	T	C	3.79E-04	PRS-CSx
Colorectum	PRS7	rs2179593	20	42660286	C	A	-1.72E-03	PRS-CSx
Colorectum	PRS7	rs2180427	14	59415933	G	T	2.67E-04	PRS-CSx
Colorectum	PRS7	rs2180686	20	6529470	T	C	4.11E-04	PRS-CSx
Colorectum	PRS7	rs2180871	14	58739537	A	G	2.36E-04	PRS-CSx
Colorectum	PRS7	rs2181398	6	6755091	T	C	4.55E-04	PRS-CSx
Colorectum	PRS7	rs2182435	20	55750204	T	G	2.18E-03	PRS-CSx
Colorectum	PRS7	rs2183275	14	34139994	C	T	-1.24E-03	PRS-CSx
Colorectum	PRS7	rs2185369	1	246022159	A	G	-3.15E-04	PRS-CSx
Colorectum	PRS7	rs2185414	9	107737067	A	G	-8.64E-07	PRS-CSx
Colorectum	PRS7	rs2185770	6	73749955	G	A	-6.69E-04	PRS-CSx
Colorectum	PRS7	rs2186023	10	129031266	A	G	-1.15E-03	PRS-CSx
Colorectum	PRS7	rs2186280	21	34837144	A	G	9.03E-05	PRS-CSx
Colorectum	PRS7	rs2188119	12	46310296	T	C	-2.94E-04	PRS-CSx
Colorectum	PRS7	rs2188399	12	111272553	G	A	6.52E-05	PRS-CSx
Colorectum	PRS7	rs2189181	4	105757128	T	C	1.86E-04	PRS-CSx
Colorectum	PRS7	rs2189698	19	57014071	G	T	-1.08E-03	PRS-CSx
Colorectum	PRS7	rs2192562	2	59478292	C	T	-1.08E-03	PRS-CSx
Colorectum	PRS7	rs2192635	16	9690029	G	T	8.01E-04	PRS-CSx
Colorectum	PRS7	rs2193060	17	70086350	T	C	-1.22E-03	PRS-CSx
Colorectum	PRS7	rs2196	9	33914308	T	C	-3.06E-04	PRS-CSx
Colorectum	PRS7	rs2196158	2	80176806	G	A	-6.85E-04	PRS-CSx
Colorectum	PRS7	rs2196652	2	19336208	G	A	6.65E-05	PRS-CSx
Colorectum	PRS7	rs2198050	7	37906477	C	T	-1.34E-03	PRS-CSx
Colorectum	PRS7	rs2199312	4	115434478	G	A	6.55E-05	PRS-CSx
Colorectum	PRS7	rs2201971	3	112953630	A	G	9.43E-05	PRS-CSx
Colorectum	PRS7	rs2202114	5	75837477	T	C	4.55E-04	PRS-CSx
Colorectum	PRS7	rs2202540	5	40026391	T	C	1.91E-04	PRS-CSx
Colorectum	PRS7	rs2203686	3	152347482	T	C	2.88E-04	PRS-CSx
Colorectum	PRS7	rs220488	17	3591822	G	A	1.66E-03	PRS-CSx
Colorectum	PRS7	rs220502	20	37296788	A	G	-4.74E-04	PRS-CSx
Colorectum	PRS7	rs220504	20	37293595	A	G	-5.20E-04	PRS-CSx
Colorectum	PRS7	rs2205824	20	7849927	A	G	1.00E-03	PRS-CSx
Colorectum	PRS7	rs220593	12	13966773	G	A	2.50E-04	PRS-CSx
Colorectum	PRS7	rs2206734	6	20694884	T	C	-2.80E-04	PRS-CSx

Colorectum	PRS7	rs2209760	20	6364504	T	C	2.93E-04	PRS-CSx
Colorectum	PRS7	rs2209761	20	6371011	C	T	4.21E-04	PRS-CSx
Colorectum	PRS7	rs2209763	20	6381747	T	C	1.46E-03	PRS-CSx
Colorectum	PRS7	rs2213992	7	88141179	G	A	-2.68E-04	PRS-CSx
Colorectum	PRS7	rs221430	14	80068121	C	T	-1.33E-04	PRS-CSx
Colorectum	PRS7	rs2214527	12	47945797	A	G	6.12E-04	PRS-CSx
Colorectum	PRS7	rs2215047	19	57823046	G	A	-4.39E-04	PRS-CSx
Colorectum	PRS7	rs2215679	7	21051042	A	C	9.50E-04	PRS-CSx
Colorectum	PRS7	rs2215762	4	18910602	G	A	1.25E-04	PRS-CSx
Colorectum	PRS7	rs2216373	2	71525478	T	C	-4.75E-04	PRS-CSx
Colorectum	PRS7	rs2216375	2	208079942	C	T	-8.57E-05	PRS-CSx
Colorectum	PRS7	rs2216595	19	33533292	T	C	-2.54E-03	PRS-CSx
Colorectum	PRS7	rs2218090	9	87200556	G	A	-1.41E-03	PRS-CSx
Colorectum	PRS7	rs2218989	7	134561241	A	G	-3.54E-04	PRS-CSx
Colorectum	PRS7	rs221899	14	71605268	G	A	-5.02E-04	PRS-CSx
Colorectum	PRS7	rs2219508	15	31751007	C	T	2.72E-04	PRS-CSx
Colorectum	PRS7	rs2219779	3	28033233	C	T	5.68E-04	PRS-CSx
Colorectum	PRS7	rs2220292	12	129618905	A	G	-1.21E-03	PRS-CSx
Colorectum	PRS7	rs2221096	12	115877631	A	G	-2.99E-04	PRS-CSx
Colorectum	PRS7	rs2221824	6	23877024	C	T	-4.88E-04	PRS-CSx
Colorectum	PRS7	rs2221825	3	73764135	T	C	-1.39E-04	PRS-CSx
Colorectum	PRS7	rs2223276	20	7882106	T	C	-2.97E-03	PRS-CSx
Colorectum	PRS7	rs2224011	6	69555181	A	C	-2.83E-04	PRS-CSx
Colorectum	PRS7	rs2224380	6	26553943	G	A	2.26E-05	PRS-CSx
Colorectum	PRS7	rs2224652	1	90196552	C	T	5.36E-04	PRS-CSx
Colorectum	PRS7	rs222478	2	42656919	A	C	-1.37E-03	PRS-CSx
Colorectum	PRS7	rs2226917	11	15111580	T	C	4.75E-04	PRS-CSx
Colorectum	PRS7	rs222757	17	3569913	T	G	-2.07E-04	PRS-CSx
Colorectum	PRS7	rs222762	17	3578647	T	C	2.85E-04	PRS-CSx
Colorectum	PRS7	rs222765	17	3579858	C	T	2.56E-04	PRS-CSx
Colorectum	PRS7	rs2228396	6	32797809	T	C	6.77E-04	PRS-CSx
Colorectum	PRS7	rs2229575	5	31317952	C	T	-1.42E-04	PRS-CSx
Colorectum	PRS7	rs2229637	6	33643558	A	G	-1.41E-03	PRS-CSx
Colorectum	PRS7	rs2231940	19	41944237	T	C	1.90E-04	PRS-CSx
Colorectum	PRS7	rs223287	6	57104266	C	T	-2.80E-03	PRS-CSx
Colorectum	PRS7	rs223288	6	57105602	T	C	-2.33E-03	PRS-CSx
Colorectum	PRS7	rs2233752	14	58755480	A	G	2.08E-04	PRS-CSx
Colorectum	PRS7	rs2234409	11	57321811	T	G	2.19E-04	PRS-CSx
Colorectum	PRS7	rs2235250	20	7870722	T	C	-3.93E-04	PRS-CSx
Colorectum	PRS7	rs2235383	6	29693499	G	A	3.40E-04	PRS-CSx
Colorectum	PRS7	rs2235493	6	6693320	A	G	-1.50E-03	PRS-CSx
Colorectum	PRS7	rs2235529	1	22450487	T	C	-4.11E-04	PRS-CSx
Colorectum	PRS7	rs2235624	16	1545448	A	G	6.11E-05	PRS-CSx
Colorectum	PRS7	rs2235751	20	1969934	A	G	-5.21E-04	PRS-CSx
Colorectum	PRS7	rs2236066	14	66246682	T	C	8.05E-04	PRS-CSx
Colorectum	PRS7	rs2236436	21	40382042	A	G	2.03E-03	PRS-CSx
Colorectum	PRS7	rs2236544	9	139754110	G	A	-2.16E-04	PRS-CSx
Colorectum	PRS7	rs2236604	1	226825341	A	G	1.46E-03	PRS-CSx
Colorectum	PRS7	rs2237091	5	149549797	A	G	-1.39E-03	PRS-CSx
Colorectum	PRS7	rs2237896	11	2858440	A	G	4.22E-04	PRS-CSx
Colorectum	PRS7	rs2238001	11	61524507	C	T	5.99E-04	PRS-CSx
Colorectum	PRS7	rs2238154	12	111882485	C	A	2.43E-04	PRS-CSx
Colorectum	PRS7	rs2239193	12	113436823	G	A	-2.81E-04	PRS-CSx
Colorectum	PRS7	rs2239200	12	120538651	C	T	2.60E-04	PRS-CSx
Colorectum	PRS7	rs2239201	12	120538952	C	T	3.94E-04	PRS-CSx
Colorectum	PRS7	rs2239282	14	105633499	A	G	1.03E-03	PRS-CSx

Colorectum	PRS7	rs2239397	22	46148001	G	A	-4.50E-04	PRS-CSx
Colorectum	PRS7	rs2239529	6	30078330	T	C	-1.71E-04	PRS-CSx
Colorectum	PRS7	rs2239574	21	48019811	T	C	-3.61E-05	PRS-CSx
Colorectum	PRS7	rs223982	4	12967160	G	A	3.22E-03	PRS-CSx
Colorectum	PRS7	rs2240192	12	113335687	A	G	-1.03E-03	PRS-CSx
Colorectum	PRS7	rs2240193	12	113335661	A	C	-3.01E-04	PRS-CSx
Colorectum	PRS7	rs2240194	12	113319600	C	T	-2.10E-04	PRS-CSx
Colorectum	PRS7	rs2240287	11	61505583	A	G	1.24E-04	PRS-CSx
Colorectum	PRS7	rs2240567	17	12819470	A	C	6.64E-04	PRS-CSx
Colorectum	PRS7	rs2240898	18	3595284	G	A	7.56E-04	PRS-CSx
Colorectum	PRS7	rs2241136	12	96387072	A	G	-8.41E-04	PRS-CSx
Colorectum	PRS7	rs2241461	2	131799563	T	C	-1.10E-03	PRS-CSx
Colorectum	PRS7	rs2241714	19	41869392	C	T	1.37E-03	PRS-CSx
Colorectum	PRS7	rs2241715	19	41856886	C	A	1.21E-03	PRS-CSx
Colorectum	PRS7	rs2241992	2	107399365	G	A	3.26E-04	PRS-CSx
Colorectum	PRS7	rs2242205	10	114318065	A	G	1.00E-03	PRS-CSx
Colorectum	PRS7	rs2242358	1	220977965	T	C	5.30E-04	PRS-CSx
Colorectum	PRS7	rs2242416	6	43273604	A	G	-1.76E-04	PRS-CSx
Colorectum	PRS7	rs2242422	10	1360092	T	C	-1.74E-03	PRS-CSx
Colorectum	PRS7	rs2242936	21	40423471	C	T	3.82E-03	PRS-CSx
Colorectum	PRS7	rs2243191	1	207015957	C	T	-6.04E-04	PRS-CSx
Colorectum	PRS7	rs2243384	6	117678083	G	A	-3.68E-04	PRS-CSx
Colorectum	PRS7	rs2243647	2	145793076	G	A	-4.62E-04	PRS-CSx
Colorectum	PRS7	rs2243792	10	8826990	C	T	2.07E-04	PRS-CSx
Colorectum	PRS7	rs2244010	21	23650356	C	T	1.49E-03	PRS-CSx
Colorectum	PRS7	rs2245007	14	54007026	A	G	-5.96E-04	PRS-CSx
Colorectum	PRS7	rs2245777	6	88424509	C	T	7.94E-04	PRS-CSx
Colorectum	PRS7	rs2246142	2	145784885	C	T	-4.03E-04	PRS-CSx
Colorectum	PRS7	rs2247876	15	48931451	C	T	3.21E-04	PRS-CSx
Colorectum	PRS7	rs2248014	8	99030280	C	T	-8.92E-04	PRS-CSx
Colorectum	PRS7	rs2248071	9	33857803	T	C	-1.25E-04	PRS-CSx
Colorectum	PRS7	rs2248077	9	139649273	A	G	1.35E-03	PRS-CSx
Colorectum	PRS7	rs2248820	21	34837623	C	A	3.95E-05	PRS-CSx
Colorectum	PRS7	rs2248863	11	119207341	A	G	-5.64E-04	PRS-CSx
Colorectum	PRS7	rs2248910	9	33838475	T	C	-2.58E-04	PRS-CSx
Colorectum	PRS7	rs2249060	21	47773177	T	C	8.82E-04	PRS-CSx
Colorectum	PRS7	rs2249098	8	22400098	T	C	1.14E-04	PRS-CSx
Colorectum	PRS7	rs2249099	6	30079307	A	C	-7.10E-05	PRS-CSx
Colorectum	PRS7	rs2249213	5	139937146	G	A	1.82E-05	PRS-CSx
Colorectum	PRS7	rs2249371	1	187436592	A	G	1.18E-03	PRS-CSx
Colorectum	PRS7	rs2249742	6	31240721	C	T	-4.36E-04	PRS-CSx
Colorectum	PRS7	rs2250377	1	201860626	A	G	4.79E-04	PRS-CSx
Colorectum	PRS7	rs2250546	2	145770621	A	G	-5.17E-04	PRS-CSx
Colorectum	PRS7	rs2250702	21	38103630	T	C	-7.95E-06	PRS-CSx
Colorectum	PRS7	rs2250704	1	231486647	A	G	2.78E-04	PRS-CSx
Colorectum	PRS7	rs2250734	1	231485958	G	A	3.56E-04	PRS-CSx
Colorectum	PRS7	rs2251207	9	90827581	G	A	-1.36E-03	PRS-CSx
Colorectum	PRS7	rs225205	17	30894286	G	T	4.90E-04	PRS-CSx
Colorectum	PRS7	rs225212	17	30896455	C	T	6.48E-04	PRS-CSx
Colorectum	PRS7	rs225214	17	30896756	T	C	2.49E-04	PRS-CSx
Colorectum	PRS7	rs2252383	2	145803003	A	G	-3.59E-04	PRS-CSx
Colorectum	PRS7	rs2252519	4	39077904	G	A	-5.61E-05	PRS-CSx
Colorectum	PRS7	rs2252708	4	120612158	C	A	-4.03E-04	PRS-CSx
Colorectum	PRS7	rs2252731	20	44008137	A	G	-8.66E-04	PRS-CSx
Colorectum	PRS7	rs2253215	5	72247868	T	C	-3.58E-04	PRS-CSx
Colorectum	PRS7	rs2253650	13	42656841	C	T	5.10E-04	PRS-CSx

Colorectum	PRS7	rs2253771	1	200068658	T	C	5.90E-04	PRS-CSx
Colorectum	PRS7	rs2254303	6	43276390	G	A	-4.44E-04	PRS-CSx
Colorectum	PRS7	rs2254358	1	22216574	A	C	2.42E-04	PRS-CSx
Colorectum	PRS7	rs2254883	8	101162958	G	A	-3.74E-05	PRS-CSx
Colorectum	PRS7	rs2255464	8	99045978	C	A	-5.90E-04	PRS-CSx
Colorectum	PRS7	rs2255701	15	76196709	T	C	-2.89E-04	PRS-CSx
Colorectum	PRS7	rs2255954	21	19793889	G	A	9.93E-04	PRS-CSx
Colorectum	PRS7	rs2255990	11	67164495	T	C	-6.51E-04	PRS-CSx
Colorectum	PRS7	rs2255994	15	54153623	A	G	-1.03E-03	PRS-CSx
Colorectum	PRS7	rs2256003	15	54153462	G	A	-1.04E-03	PRS-CSx
Colorectum	PRS7	rs2256266	6	29632318	T	C	-2.23E-04	PRS-CSx
Colorectum	PRS7	rs2257136	2	71558924	G	T	-4.01E-04	PRS-CSx
Colorectum	PRS7	rs2257763	1	2490898	C	A	-2.76E-04	PRS-CSx
Colorectum	PRS7	rs2259816	12	121435587	T	G	6.06E-04	PRS-CSx
Colorectum	PRS7	rs2260976	1	2527678	G	A	-2.63E-05	PRS-CSx
Colorectum	PRS7	rs2261360	7	99692993	T	G	3.60E-04	PRS-CSx
Colorectum	PRS7	rs2261988	19	4910889	T	G	2.09E-03	PRS-CSx
Colorectum	PRS7	rs2266959	22	21922904	T	G	-2.04E-04	PRS-CSx
Colorectum	PRS7	rs2267172	22	32339782	A	G	3.84E-03	PRS-CSx
Colorectum	PRS7	rs2267465	22	43299878	G	A	5.73E-04	PRS-CSx
Colorectum	PRS7	rs2268466	14	81484599	C	T	-1.23E-03	PRS-CSx
Colorectum	PRS7	rs2269290	5	83385150	T	C	-1.86E-03	PRS-CSx
Colorectum	PRS7	rs2269424	6	32132233	A	G	-1.09E-04	PRS-CSx
Colorectum	PRS7	rs2269872	12	113302685	G	A	-3.64E-04	PRS-CSx
Colorectum	PRS7	rs2270605	3	41496752	C	A	-2.33E-04	PRS-CSx
Colorectum	PRS7	rs2270676	11	74168411	G	A	2.86E-04	PRS-CSx
Colorectum	PRS7	rs2270781	3	113015660	A	G	2.31E-04	PRS-CSx
Colorectum	PRS7	rs2270783	3	113023675	T	G	-3.03E-04	PRS-CSx
Colorectum	PRS7	rs2270784	3	113024002	A	G	-3.27E-03	PRS-CSx
Colorectum	PRS7	rs2270785	3	113024018	C	T	-4.87E-04	PRS-CSx
Colorectum	PRS7	rs2270981	17	7216540	A	G	1.76E-03	PRS-CSx
Colorectum	PRS7	rs2271424	1	204403311	C	T	-5.56E-04	PRS-CSx
Colorectum	PRS7	rs2271427	1	204418198	T	C	-2.03E-04	PRS-CSx
Colorectum	PRS7	rs2272874	6	29696245	C	T	3.07E-04	PRS-CSx
Colorectum	PRS7	rs2272991	6	29527599	C	T	4.00E-04	PRS-CSx
Colorectum	PRS7	rs2273143	22	43280650	T	C	2.51E-04	PRS-CSx
Colorectum	PRS7	rs2273195	1	40779841	T	G	-1.72E-03	PRS-CSx
Colorectum	PRS7	rs2273331	20	1650577	A	G	8.61E-04	PRS-CSx
Colorectum	PRS7	rs2273602	6	117730592	A	G	1.40E-04	PRS-CSx
Colorectum	PRS7	rs2274405	13	95858978	T	C	-3.94E-04	PRS-CSx
Colorectum	PRS7	rs2274410	13	95860288	C	A	-2.21E-04	PRS-CSx
Colorectum	PRS7	rs2274472	9	4985542	C	T	1.01E-03	PRS-CSx
Colorectum	PRS7	rs2274883	6	158910543	C	T	4.73E-04	PRS-CSx
Colorectum	PRS7	rs2274884	6	158910491	C	T	2.91E-04	PRS-CSx
Colorectum	PRS7	rs2274984	1	183107446	G	T	1.44E-04	PRS-CSx
Colorectum	PRS7	rs2275003	9	34124860	G	A	-3.07E-05	PRS-CSx
Colorectum	PRS7	rs2275206	1	156939067	A	G	-2.32E-04	PRS-CSx
Colorectum	PRS7	rs2275741	1	31426815	G	A	7.27E-05	PRS-CSx
Colorectum	PRS7	rs2275819	1	3689408	G	A	-5.27E-04	PRS-CSx
Colorectum	PRS7	rs2275843	13	110813532	A	G	-1.55E-04	PRS-CSx
Colorectum	PRS7	rs2276035	11	120346360	A	G	-3.59E-04	PRS-CSx
Colorectum	PRS7	rs2276543	1	183155305	A	G	8.81E-05	PRS-CSx
Colorectum	PRS7	rs2277103	6	28109633	T	C	1.34E-03	PRS-CSx
Colorectum	PRS7	rs2277151	9	19376408	A	G	2.62E-04	PRS-CSx
Colorectum	PRS7	rs2277339	12	57146069	G	T	1.70E-03	PRS-CSx
Colorectum	PRS7	rs2277474	14	23874523	T	C	1.27E-04	PRS-CSx

Colorectum	PRS7	rs2277827	21	47986001	T	G	2.41E-04	PRS-CSx
Colorectum	PRS7	rs2277841	22	36900271	C	T	-2.63E-04	PRS-CSx
Colorectum	PRS7	rs2278115	2	131797367	G	A	-1.13E-03	PRS-CSx
Colorectum	PRS7	rs2278186	15	48734216	T	C	7.12E-04	PRS-CSx
Colorectum	PRS7	rs2278581	2	43020914	A	G	3.45E-04	PRS-CSx
Colorectum	PRS7	rs2278604	15	67008737	A	C	-1.80E-03	PRS-CSx
Colorectum	PRS7	rs2278722	2	101620136	G	A	-8.03E-04	PRS-CSx
Colorectum	PRS7	rs2278785	2	168996775	T	C	-3.84E-04	PRS-CSx
Colorectum	PRS7	rs2279234	15	48760391	A	G	7.90E-04	PRS-CSx
Colorectum	PRS7	rs2279237	15	48821048	A	G	6.83E-04	PRS-CSx
Colorectum	PRS7	rs2279439	11	47196982	C	T	1.05E-04	PRS-CSx
Colorectum	PRS7	rs2279482	15	29416901	G	A	1.31E-03	PRS-CSx
Colorectum	PRS7	rs2279638	1	221001787	A	G	3.97E-04	PRS-CSx
Colorectum	PRS7	rs2280028	16	86233413	A	G	-2.18E-03	PRS-CSx
Colorectum	PRS7	rs2280305	3	13660809	C	T	-2.84E-03	PRS-CSx
Colorectum	PRS7	rs2280503	12	51138687	C	A	4.59E-04	PRS-CSx
Colorectum	PRS7	rs2280543	11	203788	T	C	-3.71E-03	PRS-CSx
Colorectum	PRS7	rs2280797	14	105623612	C	T	5.04E-04	PRS-CSx
Colorectum	PRS7	rs2280954	4	87988862	G	A	3.23E-04	PRS-CSx
Colorectum	PRS7	rs2281082	22	36872750	G	T	-4.62E-04	PRS-CSx
Colorectum	PRS7	rs2281272	6	1590396	T	C	1.66E-03	PRS-CSx
Colorectum	PRS7	rs2281850	16	68718750	A	G	-3.85E-04	PRS-CSx
Colorectum	PRS7	rs2281852	1	2490942	C	A	-1.61E-04	PRS-CSx
Colorectum	PRS7	rs2282259	9	139649379	T	C	-6.77E-04	PRS-CSx
Colorectum	PRS7	rs2282537	11	120187971	A	G	-2.05E-03	PRS-CSx
Colorectum	PRS7	rs2282637	11	111228098	A	G	-1.02E-03	PRS-CSx
Colorectum	PRS7	rs2282810	5	149552699	C	T	-1.02E-03	PRS-CSx
Colorectum	PRS7	rs2282812	5	149553747	G	A	-7.64E-04	PRS-CSx
Colorectum	PRS7	rs2283017	7	99574758	A	G	-1.10E-04	PRS-CSx
Colorectum	PRS7	rs2283790	22	21956653	A	G	1.83E-04	PRS-CSx
Colorectum	PRS7	rs2283817	22	26923155	C	T	-3.10E-04	PRS-CSx
Colorectum	PRS7	rs2283820	22	26923907	C	T	-3.74E-04	PRS-CSx
Colorectum	PRS7	rs2283822	22	26924312	T	C	-3.02E-04	PRS-CSx
Colorectum	PRS7	rs2283823	22	26924336	T	C	-3.98E-04	PRS-CSx
Colorectum	PRS7	rs2283827	22	26926047	G	A	-3.13E-04	PRS-CSx
Colorectum	PRS7	rs2283941	22	34160402	G	A	-1.32E-04	PRS-CSx
Colorectum	PRS7	rs2283965	22	36869013	T	C	-5.78E-04	PRS-CSx
Colorectum	PRS7	rs2284555	21	34806288	G	A	6.96E-05	PRS-CSx
Colorectum	PRS7	rs2284556	21	34806305	G	A	-5.75E-05	PRS-CSx
Colorectum	PRS7	rs2284691	12	109639521	A	G	1.76E-03	PRS-CSx
Colorectum	PRS7	rs2284928	2	208023968	C	T	-3.32E-04	PRS-CSx
Colorectum	PRS7	rs2285727	12	112093078	G	T	1.79E-04	PRS-CSx
Colorectum	PRS7	rs2285810	12	112699540	T	C	2.73E-04	PRS-CSx
Colorectum	PRS7	rs2286040	12	120502726	G	A	2.12E-04	PRS-CSx
Colorectum	PRS7	rs2286159	7	76998286	T	C	8.91E-04	PRS-CSx
Colorectum	PRS7	rs2286435	19	3638856	T	C	-7.23E-04	PRS-CSx
Colorectum	PRS7	rs2286486	3	45927741	C	T	-6.16E-04	PRS-CSx
Colorectum	PRS7	rs2286630	10	118084657	C	T	5.00E-04	PRS-CSx
Colorectum	PRS7	rs2286836	14	73442192	T	C	3.01E-04	PRS-CSx
Colorectum	PRS7	rs2286838	14	73448583	A	G	4.40E-04	PRS-CSx
Colorectum	PRS7	rs2287197	16	50106594	T	C	-2.58E-04	PRS-CSx
Colorectum	PRS7	rs2287549	12	108924904	T	C	1.07E-03	PRS-CSx
Colorectum	PRS7	rs228803	6	39098815	A	G	3.69E-04	PRS-CSx
Colorectum	PRS7	rs2288105	2	159992699	T	C	1.07E-03	PRS-CSx
Colorectum	PRS7	rs2288158	11	113133676	G	T	1.16E-03	PRS-CSx
Colorectum	PRS7	rs228818	6	39109707	G	A	1.12E-03	PRS-CSx

Colorectum	PRS7	rs228819	6	39110432	A	C	2.03E-04	PRS-CSx
Colorectum	PRS7	rs2288499	14	81660997	T	C	-3.82E-04	PRS-CSx
Colorectum	PRS7	rs2288645	7	150747746	A	G	1.17E-03	PRS-CSx
Colorectum	PRS7	rs2288970	7	132106953	C	T	-4.16E-04	PRS-CSx
Colorectum	PRS7	rs2288971	7	132107670	G	A	-3.91E-04	PRS-CSx
Colorectum	PRS7	rs2288975	7	132114617	G	A	-4.50E-04	PRS-CSx
Colorectum	PRS7	rs2288998	8	142225990	A	G	-2.64E-04	PRS-CSx
Colorectum	PRS7	rs2289367	7	45113170	A	G	-5.05E-04	PRS-CSx
Colorectum	PRS7	rs2289369	7	45113289	T	C	-3.82E-04	PRS-CSx
Colorectum	PRS7	rs2289737	4	146418847	C	T	5.25E-04	PRS-CSx
Colorectum	PRS7	rs2290206	17	38640992	G	A	-7.18E-04	PRS-CSx
Colorectum	PRS7	rs2290207	17	38640744	T	C	-5.12E-04	PRS-CSx
Colorectum	PRS7	rs2290224	7	37905420	G	A	-3.39E-04	PRS-CSx
Colorectum	PRS7	rs2290519	8	121244066	T	C	-4.11E-04	PRS-CSx
Colorectum	PRS7	rs2290883	11	46890197	G	A	1.84E-04	PRS-CSx
Colorectum	PRS7	rs2291193	17	57272079	A	G	1.05E-04	PRS-CSx
Colorectum	PRS7	rs2291297	1	38272660	A	G	-1.63E-05	PRS-CSx
Colorectum	PRS7	rs2291365	12	25272027	A	G	-2.80E-04	PRS-CSx
Colorectum	PRS7	rs2291366	12	25205801	T	C	-1.20E-03	PRS-CSx
Colorectum	PRS7	rs2291443	11	47179861	C	A	7.43E-05	PRS-CSx
Colorectum	PRS7	rs2291444	11	47179143	C	T	4.56E-05	PRS-CSx
Colorectum	PRS7	rs2291507	4	105890515	A	G	-1.96E-03	PRS-CSx
Colorectum	PRS7	rs2291832	1	222826481	G	A	7.38E-04	PRS-CSx
Colorectum	PRS7	rs2291834	1	222832295	T	C	5.81E-04	PRS-CSx
Colorectum	PRS7	rs2291889	1	1335218	A	G	3.40E-04	PRS-CSx
Colorectum	PRS7	rs2291905	3	113010466	T	C	-1.16E-03	PRS-CSx
Colorectum	PRS7	rs2292264	5	10235490	G	A	-5.84E-04	PRS-CSx
Colorectum	PRS7	rs2292459	1	204413297	T	C	3.36E-04	PRS-CSx
Colorectum	PRS7	rs2292758	18	12884466	T	C	-1.33E-03	PRS-CSx
Colorectum	PRS7	rs2293602	12	54635846	G	T	-6.07E-04	PRS-CSx
Colorectum	PRS7	rs2293829	14	80300677	C	A	-1.59E-03	PRS-CSx
Colorectum	PRS7	rs2293881	17	3620138	G	A	5.97E-04	PRS-CSx
Colorectum	PRS7	rs2294073	8	2065962	C	A	5.46E-04	PRS-CSx
Colorectum	PRS7	rs2294305	20	7869456	C	T	-4.41E-04	PRS-CSx
Colorectum	PRS7	rs2294306	20	7869025	G	A	-1.68E-03	PRS-CSx
Colorectum	PRS7	rs2294444	16	1724693	T	G	4.78E-04	PRS-CSx
Colorectum	PRS7	rs2294884	6	32367259	G	T	-1.45E-04	PRS-CSx
Colorectum	PRS7	rs2294952	20	37279967	C	A	-6.40E-04	PRS-CSx
Colorectum	PRS7	rs2295085	6	88240047	C	T	9.34E-04	PRS-CSx
Colorectum	PRS7	rs2295279	6	108025416	A	G	-1.10E-03	PRS-CSx
Colorectum	PRS7	rs2295426	14	59376455	T	C	3.66E-04	PRS-CSx
Colorectum	PRS7	rs2295444	20	33173883	T	C	-9.39E-05	PRS-CSx
Colorectum	PRS7	rs2295692	6	116956489	T	C	-1.09E-04	PRS-CSx
Colorectum	PRS7	rs2295770	10	102239829	A	G	-1.61E-04	PRS-CSx
Colorectum	PRS7	rs2296053	9	735089	A	G	-8.84E-04	PRS-CSx
Colorectum	PRS7	rs2296170	9	9803954	C	T	6.87E-05	PRS-CSx
Colorectum	PRS7	rs2296288	1	183072590	T	C	2.96E-04	PRS-CSx
Colorectum	PRS7	rs2296292	1	183086757	A	C	2.45E-04	PRS-CSx
Colorectum	PRS7	rs2296293	1	183095477	G	A	1.28E-04	PRS-CSx
Colorectum	PRS7	rs2296300	1	183099701	G	A	1.81E-04	PRS-CSx
Colorectum	PRS7	rs2296370	19	55224785	A	G	-8.32E-04	PRS-CSx
Colorectum	PRS7	rs2296405	16	68721470	T	C	-5.22E-04	PRS-CSx
Colorectum	PRS7	rs2296406	16	68721340	G	A	-3.26E-04	PRS-CSx
Colorectum	PRS7	rs2296408	16	68713823	C	A	-1.70E-04	PRS-CSx
Colorectum	PRS7	rs2296409	16	68713730	G	A	-1.11E-04	PRS-CSx
Colorectum	PRS7	rs2296424	10	81064755	A	G	-1.46E-03	PRS-CSx

Colorectum	PRS7	rs2296443	1	2522392	A	G	-4.40E-04	PRS-CSx
Colorectum	PRS7	rs2296481	1	65845076	C	T	-8.49E-04	PRS-CSx
Colorectum	PRS7	rs2296782	10	114723857	A	G	-4.25E-03	PRS-CSx
Colorectum	PRS7	rs2296783	10	114723650	C	T	-6.46E-03	PRS-CSx
Colorectum	PRS7	rs2296948	9	117433685	C	T	-4.90E-04	PRS-CSx
Colorectum	PRS7	rs2297363	6	160506462	A	G	4.43E-04	PRS-CSx
Colorectum	PRS7	rs2297721	9	139656516	T	C	-9.95E-04	PRS-CSx
Colorectum	PRS7	rs2297722	9	139656670	T	C	6.37E-04	PRS-CSx
Colorectum	PRS7	rs2297723	9	139656706	A	C	6.66E-04	PRS-CSx
Colorectum	PRS7	rs2298075	10	102247408	A	C	-1.82E-04	PRS-CSx
Colorectum	PRS7	rs2298428	22	21982892	T	C	-3.55E-04	PRS-CSx
Colorectum	PRS7	rs2298429	22	21983260	A	G	4.20E-04	PRS-CSx
Colorectum	PRS7	rs2298601	18	33775371	A	G	3.99E-04	PRS-CSx
Colorectum	PRS7	rs2298792	11	74340196	G	A	4.18E-04	PRS-CSx
Colorectum	PRS7	rs2299030	6	28198755	C	T	7.31E-04	PRS-CSx
Colorectum	PRS7	rs2299418	7	94032325	A	G	5.68E-05	PRS-CSx
Colorectum	PRS7	rs229988	12	28218232	C	T	8.34E-04	PRS-CSx
Colorectum	PRS7	rs2299919	14	78049868	G	A	6.63E-04	PRS-CSx
Colorectum	PRS7	rs2300250	12	13860621	G	A	-8.38E-04	PRS-CSx
Colorectum	PRS7	rs2301487	16	547297	C	T	2.28E-03	PRS-CSx
Colorectum	PRS7	rs2301573	3	129305919	C	T	-3.28E-04	PRS-CSx
Colorectum	PRS7	rs2301610	12	111353556	G	A	-8.51E-04	PRS-CSx
Colorectum	PRS7	rs2301877	1	183259125	A	G	-8.60E-05	PRS-CSx
Colorectum	PRS7	rs2301971	7	16511064	A	G	4.86E-04	PRS-CSx
Colorectum	PRS7	rs230202	20	55791458	C	T	-6.97E-04	PRS-CSx
Colorectum	PRS7	rs2302104	5	140047600	T	C	4.74E-04	PRS-CSx
Colorectum	PRS7	rs2302702	12	109921258	T	C	-3.54E-04	PRS-CSx
Colorectum	PRS7	rs2302904	5	31317494	T	C	-1.74E-04	PRS-CSx
Colorectum	PRS7	rs2303010	5	179752031	T	C	-5.43E-03	PRS-CSx
Colorectum	PRS7	rs2303222	16	31085470	T	C	1.94E-04	PRS-CSx
Colorectum	PRS7	rs2303364	7	6441688	C	T	1.02E-03	PRS-CSx
Colorectum	PRS7	rs2303478	12	108226622	T	C	-8.85E-04	PRS-CSx
Colorectum	PRS7	rs2303925	7	150732463	A	G	-3.29E-04	PRS-CSx
Colorectum	PRS7	rs2303933	7	150766799	A	G	9.48E-04	PRS-CSx
Colorectum	PRS7	rs2303982	3	122630323	G	T	-6.39E-05	PRS-CSx
Colorectum	PRS7	rs2304102	19	33467357	A	G	1.84E-04	PRS-CSx
Colorectum	PRS7	rs2304231	19	41762670	C	T	-5.07E-04	PRS-CSx
Colorectum	PRS7	rs2304232	19	41762525	G	A	-5.85E-04	PRS-CSx
Colorectum	PRS7	rs2304693	7	45148667	A	G	-5.68E-04	PRS-CSx
Colorectum	PRS7	rs2304694	7	45148773	A	C	-5.39E-04	PRS-CSx
Colorectum	PRS7	rs2305008	11	120319064	C	A	-1.00E-04	PRS-CSx
Colorectum	PRS7	rs2305115	19	37835658	T	C	6.74E-04	PRS-CSx
Colorectum	PRS7	rs2305158	2	101612051	A	G	2.97E-04	PRS-CSx
Colorectum	PRS7	rs2305159	2	101591443	A	C	8.10E-04	PRS-CSx
Colorectum	PRS7	rs2305160	2	101591304	A	G	5.35E-04	PRS-CSx
Colorectum	PRS7	rs2305825	11	117232492	T	C	5.07E-04	PRS-CSx
Colorectum	PRS7	rs2306021	15	68640855	A	G	1.09E-03	PRS-CSx
Colorectum	PRS7	rs2306026	11	46889713	C	T	2.15E-04	PRS-CSx
Colorectum	PRS7	rs2306027	11	46907827	T	C	3.24E-04	PRS-CSx
Colorectum	PRS7	rs2306028	11	46900659	G	T	6.28E-05	PRS-CSx
Colorectum	PRS7	rs2306029	11	46893108	C	T	4.77E-04	PRS-CSx
Colorectum	PRS7	rs2306033	11	46897446	G	A	9.08E-05	PRS-CSx
Colorectum	PRS7	rs2306036	11	46911704	T	C	1.22E-04	PRS-CSx
Colorectum	PRS7	rs2306365	11	65427346	A	G	5.40E-04	PRS-CSx
Colorectum	PRS7	rs2306427	1	38345896	C	A	-6.85E-05	PRS-CSx
Colorectum	PRS7	rs2306557	4	156617994	A	G	3.17E-04	PRS-CSx

Colorectum	PRS7	rs2306620	5	125936399	G	A	-8.88E-04	PRS-CSx
Colorectum	PRS7	rs2306752	17	80426635	C	T	-2.09E-03	PRS-CSx
Colorectum	PRS7	rs2306792	3	113081835	G	A	-4.90E-03	PRS-CSx
Colorectum	PRS7	rs2306887	1	92200218	C	T	-2.80E-04	PRS-CSx
Colorectum	PRS7	rs2306933	15	29421054	T	C	5.52E-03	PRS-CSx
Colorectum	PRS7	rs2307139	17	5066005	G	A	7.06E-05	PRS-CSx
Colorectum	PRS7	rs2307220	12	79985874	C	A	1.80E-04	PRS-CSx
Colorectum	PRS7	rs2314594	4	17010690	G	A	-9.92E-04	PRS-CSx
Colorectum	PRS7	rs2315656	20	62418337	G	A	1.12E-03	PRS-CSx
Colorectum	PRS7	rs2317215	8	117599967	A	G	-1.27E-05	PRS-CSx
Colorectum	PRS7	rs231916	11	2748368	T	C	9.69E-04	PRS-CSx
Colorectum	PRS7	rs231917	11	2745107	T	C	-2.31E-03	PRS-CSx
Colorectum	PRS7	rs2319398	3	119612942	C	A	1.87E-04	PRS-CSx
Colorectum	PRS7	rs2319399	3	119636207	G	A	2.09E-04	PRS-CSx
Colorectum	PRS7	rs2320431	2	96024876	C	A	-3.08E-04	PRS-CSx
Colorectum	PRS7	rs2320490	13	58078558	A	G	1.39E-03	PRS-CSx
Colorectum	PRS7	rs2320624	2	95856611	A	G	-2.94E-04	PRS-CSx
Colorectum	PRS7	rs2321480	13	34264201	C	T	-2.78E-04	PRS-CSx
Colorectum	PRS7	rs2321566	13	34271418	C	A	-2.06E-04	PRS-CSx
Colorectum	PRS7	rs2323412	4	22485598	T	C	-4.34E-04	PRS-CSx
Colorectum	PRS7	rs2324591	13	40881549	G	T	7.47E-04	PRS-CSx
Colorectum	PRS7	rs2325077	6	88453491	A	G	8.19E-04	PRS-CSx
Colorectum	PRS7	rs2325510	13	73989580	G	A	-6.67E-04	PRS-CSx
Colorectum	PRS7	rs2325512	13	73996850	G	T	1.09E-03	PRS-CSx
Colorectum	PRS7	rs2326613	6	5545046	T	G	-1.03E-03	PRS-CSx
Colorectum	PRS7	rs2326748	20	6293824	C	A	2.53E-05	PRS-CSx
Colorectum	PRS7	rs2326765	6	6393103	A	G	-7.62E-04	PRS-CSx
Colorectum	PRS7	rs2326849	20	6698687	C	T	-3.57E-04	PRS-CSx
Colorectum	PRS7	rs2327003	20	7881107	G	T	2.92E-04	PRS-CSx
Colorectum	PRS7	rs2327006	6	131308551	T	G	5.81E-04	PRS-CSx
Colorectum	PRS7	rs2327506	6	12029424	C	T	-4.61E-04	PRS-CSx
Colorectum	PRS7	rs2327849	6	15075424	G	T	-8.14E-04	PRS-CSx
Colorectum	PRS7	rs2328488	6	20418329	T	C	8.51E-04	PRS-CSx
Colorectum	PRS7	rs2328531	6	20643752	G	A	3.73E-04	PRS-CSx
Colorectum	PRS7	rs232924	12	113104903	T	C	4.84E-04	PRS-CSx
Colorectum	PRS7	rs232925	12	113109378	C	T	3.86E-04	PRS-CSx
Colorectum	PRS7	rs2329353	5	40748268	G	A	5.48E-04	PRS-CSx
Colorectum	PRS7	rs2330701	5	31291402	A	G	1.57E-04	PRS-CSx
Colorectum	PRS7	rs2331562	4	69803179	A	G	-1.68E-04	PRS-CSx
Colorectum	PRS7	rs2332251	17	52673809	A	C	-3.06E-04	PRS-CSx
Colorectum	PRS7	rs2332897	4	175386305	A	C	-2.76E-04	PRS-CSx
Colorectum	PRS7	rs2333465	17	57269428	C	T	1.64E-04	PRS-CSx
Colorectum	PRS7	rs2333620	1	183100555	T	C	-5.81E-04	PRS-CSx
Colorectum	PRS7	rs2337094	18	46403018	T	C	4.58E-04	PRS-CSx
Colorectum	PRS7	rs2337107	18	46459323	T	C	1.51E-03	PRS-CSx
Colorectum	PRS7	rs233716	12	113039943	T	C	5.00E-05	PRS-CSx
Colorectum	PRS7	rs233722	12	113031474	A	G	-7.63E-05	PRS-CSx
Colorectum	PRS7	rs2337237	18	21631244	G	A	7.66E-04	PRS-CSx
Colorectum	PRS7	rs2338680	15	31780133	A	G	5.33E-04	PRS-CSx
Colorectum	PRS7	rs2338819	5	141540017	T	C	2.36E-04	PRS-CSx
Colorectum	PRS7	rs2339345	18	49957255	A	C	6.56E-04	PRS-CSx
Colorectum	PRS7	rs2339404	12	110659314	G	A	8.56E-05	PRS-CSx
Colorectum	PRS7	rs2339818	12	112046476	T	C	2.21E-04	PRS-CSx
Colorectum	PRS7	rs2339973	12	112520883	C	T	2.45E-04	PRS-CSx
Colorectum	PRS7	rs2342390	11	5693181	G	A	7.81E-04	PRS-CSx
Colorectum	PRS7	rs2344397	7	46234504	C	T	-6.60E-04	PRS-CSx

Colorectum	PRS7	rs2345160	2	152819262	A	G	-1.88E-04	PRS-CSx
Colorectum	PRS7	rs2345728	2	200268635	T	C	7.50E-04	PRS-CSx
Colorectum	PRS7	rs234623	20	57488964	A	G	-8.12E-04	PRS-CSx
Colorectum	PRS7	rs234629	20	57483948	A	G	-4.72E-04	PRS-CSx
Colorectum	PRS7	rs2347790	22	29414001	G	A	-5.62E-04	PRS-CSx
Colorectum	PRS7	rs2348075	14	58853140	T	C	1.84E-04	PRS-CSx
Colorectum	PRS7	rs2348462	7	47848243	C	T	-1.92E-03	PRS-CSx
Colorectum	PRS7	rs2352202	13	92456283	T	C	3.19E-04	PRS-CSx
Colorectum	PRS7	rs2352782	12	25285958	G	A	-3.11E-04	PRS-CSx
Colorectum	PRS7	rs2352817	2	241256453	G	A	6.24E-04	PRS-CSx
Colorectum	PRS7	rs2352930	16	86171280	G	A	1.37E-03	PRS-CSx
Colorectum	PRS7	rs2352931	16	86169068	A	G	4.76E-04	PRS-CSx
Colorectum	PRS7	rs2352933	16	86164578	T	G	1.38E-04	PRS-CSx
Colorectum	PRS7	rs2352934	16	86187627	A	G	5.63E-04	PRS-CSx
Colorectum	PRS7	rs2354342	6	117722069	G	A	1.28E-04	PRS-CSx
Colorectum	PRS7	rs2355055	2	8228596	A	G	-3.06E-04	PRS-CSx
Colorectum	PRS7	rs2355056	2	8228731	A	G	-8.92E-04	PRS-CSx
Colorectum	PRS7	rs2355873	1	231477442	A	G	4.09E-04	PRS-CSx
Colorectum	PRS7	rs2356736	16	49897295	C	T	3.70E-04	PRS-CSx
Colorectum	PRS7	rs2356837	16	50120893	C	T	-5.28E-04	PRS-CSx
Colorectum	PRS7	rs235713	20	6711863	C	T	8.97E-04	PRS-CSx
Colorectum	PRS7	rs235735	20	6715443	A	G	1.90E-03	PRS-CSx
Colorectum	PRS7	rs235764	20	6754246	A	G	6.07E-04	PRS-CSx
Colorectum	PRS7	rs235767	20	6755598	G	T	1.23E-03	PRS-CSx
Colorectum	PRS7	rs235771	20	6762025	G	A	-1.91E-03	PRS-CSx
Colorectum	PRS7	rs2358849	10	20430040	C	T	-5.39E-05	PRS-CSx
Colorectum	PRS7	rs2359126	2	206977779	A	G	2.02E-04	PRS-CSx
Colorectum	PRS7	rs2359612	16	31103796	G	A	1.97E-04	PRS-CSx
Colorectum	PRS7	rs2359894	18	42055272	A	C	2.44E-04	PRS-CSx
Colorectum	PRS7	rs2360735	17	503759	A	G	-7.29E-04	PRS-CSx
Colorectum	PRS7	rs2361331	3	67939925	C	T	-8.13E-04	PRS-CSx
Colorectum	PRS7	rs2362295	2	234906526	C	T	-1.99E-03	PRS-CSx
Colorectum	PRS7	rs2362824	3	37199453	A	G	-1.78E-04	PRS-CSx
Colorectum	PRS7	rs2366402	5	89552374	A	G	-1.75E-05	PRS-CSx
Colorectum	PRS7	rs236681	4	87988508	G	T	3.30E-04	PRS-CSx
Colorectum	PRS7	rs2367556	12	96021033	T	C	-2.96E-04	PRS-CSx
Colorectum	PRS7	rs2367563	12	96057184	A	G	-4.89E-04	PRS-CSx
Colorectum	PRS7	rs2367811	2	33827428	C	A	2.47E-04	PRS-CSx
Colorectum	PRS7	rs2368255	2	183039421	T	C	1.81E-04	PRS-CSx
Colorectum	PRS7	rs2370825	14	79029937	T	C	1.41E-03	PRS-CSx
Colorectum	PRS7	rs2371147	14	80441564	G	T	4.83E-04	PRS-CSx
Colorectum	PRS7	rs2371218	5	39718145	T	C	-3.27E-04	PRS-CSx
Colorectum	PRS7	rs2372033	7	83746313	C	T	-2.56E-04	PRS-CSx
Colorectum	PRS7	rs2373926	7	150736382	A	G	-6.11E-04	PRS-CSx
Colorectum	PRS7	rs2374165	12	31789431	G	A	-9.80E-04	PRS-CSx
Colorectum	PRS7	rs2374442	2	43002930	G	A	3.72E-04	PRS-CSx
Colorectum	PRS7	rs2375274	9	31186131	C	T	1.45E-05	PRS-CSx
Colorectum	PRS7	rs237686	20	6277097	A	G	1.03E-04	PRS-CSx
Colorectum	PRS7	rs237691	20	6283026	T	C	-1.11E-04	PRS-CSx
Colorectum	PRS7	rs237693	20	6284995	G	A	6.23E-05	PRS-CSx
Colorectum	PRS7	rs237694	20	6286656	C	A	1.16E-04	PRS-CSx
Colorectum	PRS7	rs2377041	1	2713327	T	C	-1.90E-04	PRS-CSx
Colorectum	PRS7	rs2378436	1	221237584	A	G	1.61E-04	PRS-CSx
Colorectum	PRS7	rs2378489	1	222076208	C	A	-2.11E-04	PRS-CSx
Colorectum	PRS7	rs2379208	9	92853793	A	G	-9.63E-05	PRS-CSx
Colorectum	PRS7	rs2381349	4	39212010	A	G	1.26E-04	PRS-CSx

Colorectum	PRS7	rs2382075	15	38347477	A	G	-9.19E-04	PRS-CSx
Colorectum	PRS7	rs2383207	9	22115959	A	G	1.62E-03	PRS-CSx
Colorectum	PRS7	rs2384035	12	113209185	A	G	1.16E-04	PRS-CSx
Colorectum	PRS7	rs2384068	12	113273531	T	C	-6.00E-04	PRS-CSx
Colorectum	PRS7	rs2384069	12	113309189	T	C	-2.02E-04	PRS-CSx
Colorectum	PRS7	rs2386140	9	139823622	A	G	-3.27E-04	PRS-CSx
Colorectum	PRS7	rs238858	22	34156607	C	T	-5.75E-05	PRS-CSx
Colorectum	PRS7	rs238880	6	29347044	A	G	-1.92E-04	PRS-CSx
Colorectum	PRS7	rs238882	6	29346363	A	G	-1.08E-04	PRS-CSx
Colorectum	PRS7	rs2388822	10	8697320	A	C	5.22E-04	PRS-CSx
Colorectum	PRS7	rs2388828	10	8857172	T	C	2.64E-04	PRS-CSx
Colorectum	PRS7	rs2388975	4	115514055	G	A	3.24E-04	PRS-CSx
Colorectum	PRS7	rs2388996	4	115385540	C	T	-2.14E-04	PRS-CSx
Colorectum	PRS7	rs2389002	4	115388927	C	A	-1.59E-04	PRS-CSx
Colorectum	PRS7	rs2389958	4	120695256	C	T	-6.75E-05	PRS-CSx
Colorectum	PRS7	rs2390279	13	100208705	C	T	-9.01E-04	PRS-CSx
Colorectum	PRS7	rs2390849	7	23934969	T	C	-1.24E-03	PRS-CSx
Colorectum	PRS7	rs2390850	7	23935130	A	G	-1.46E-03	PRS-CSx
Colorectum	PRS7	rs2393791	12	121423956	C	T	6.21E-04	PRS-CSx
Colorectum	PRS7	rs2394160	6	29703262	G	A	-2.01E-04	PRS-CSx
Colorectum	PRS7	rs2394177	6	29787369	G	A	2.80E-04	PRS-CSx
Colorectum	PRS7	rs2394660	6	29672686	A	G	-2.62E-05	PRS-CSx
Colorectum	PRS7	rs2394847	10	73744470	T	C	-2.63E-04	PRS-CSx
Colorectum	PRS7	rs2395110	6	32215876	G	A	1.15E-05	PRS-CSx
Colorectum	PRS7	rs2395141	6	32282068	G	A	-1.23E-04	PRS-CSx
Colorectum	PRS7	rs2395182	6	32413317	G	T	-4.91E-04	PRS-CSx
Colorectum	PRS7	rs2396004	6	43355851	A	G	-8.86E-05	PRS-CSx
Colorectum	PRS7	rs2396053	6	43716429	A	G	9.03E-04	PRS-CSx
Colorectum	PRS7	rs2396110	2	225560219	A	G	3.31E-04	PRS-CSx
Colorectum	PRS7	rs2396441	6	45467765	G	A	4.31E-04	PRS-CSx
Colorectum	PRS7	rs2396751	7	114115049	G	A	3.74E-04	PRS-CSx
Colorectum	PRS7	rs2397178	6	54865906	T	G	-3.06E-04	PRS-CSx
Colorectum	PRS7	rs2397816	15	96119880	G	A	-7.37E-04	PRS-CSx
Colorectum	PRS7	rs2399441	3	112587012	T	C	-3.19E-04	PRS-CSx
Colorectum	PRS7	rs2400090	10	14791298	G	T	-5.66E-04	PRS-CSx
Colorectum	PRS7	rs2400894	12	81628938	T	C	1.39E-04	PRS-CSx
Colorectum	PRS7	rs2401823	14	89889586	G	A	1.30E-03	PRS-CSx
Colorectum	PRS7	rs2404478	4	155408038	C	T	-3.02E-04	PRS-CSx
Colorectum	PRS7	rs2404666	5	79135217	A	G	-7.12E-04	PRS-CSx
Colorectum	PRS7	rs2405027	1	224672835	C	T	6.78E-04	PRS-CSx
Colorectum	PRS7	rs2406341	18	74487868	G	A	-1.79E-04	PRS-CSx
Colorectum	PRS7	rs2406342	18	74488280	T	G	3.29E-04	PRS-CSx
Colorectum	PRS7	rs2407068	5	53176402	C	T	4.53E-04	PRS-CSx
Colorectum	PRS7	rs2408436	12	46202099	G	A	-3.21E-04	PRS-CSx
Colorectum	PRS7	rs2408462	12	46415494	G	A	3.48E-04	PRS-CSx
Colorectum	PRS7	rs2409033	5	127234574	T	C	-1.41E-04	PRS-CSx
Colorectum	PRS7	rs2413403	22	36840508	A	G	-1.09E-03	PRS-CSx
Colorectum	PRS7	rs2413565	22	39438659	A	G	-5.91E-04	PRS-CSx
Colorectum	PRS7	rs2413709	22	43061494	T	C	3.69E-04	PRS-CSx
Colorectum	PRS7	rs2413752	22	43656322	A	G	8.63E-04	PRS-CSx
Colorectum	PRS7	rs2413958	15	49760546	A	G	4.07E-04	PRS-CSx
Colorectum	PRS7	rs2414027	15	50435514	T	C	3.83E-04	PRS-CSx
Colorectum	PRS7	rs2416666	9	101844549	A	G	-6.97E-04	PRS-CSx
Colorectum	PRS7	rs2417767	12	19164264	G	T	-4.49E-04	PRS-CSx
Colorectum	PRS7	rs2418004	12	21837299	A	G	-3.23E-03	PRS-CSx
Colorectum	PRS7	rs2418192	9	114643893	T	C	5.45E-04	PRS-CSx

Colorectum	PRS7	rs2418896	2	71436269	G	A	-1.31E-04	PRS-CSx
Colorectum	PRS7	rs241959	19	38283126	T	C	-9.59E-05	PRS-CSx
Colorectum	PRS7	rs241960	19	38284289	A	G	-1.73E-04	PRS-CSx
Colorectum	PRS7	rs2420255	10	118000945	T	C	5.51E-04	PRS-CSx
Colorectum	PRS7	rs2420257	10	118008342	A	G	3.82E-04	PRS-CSx
Colorectum	PRS7	rs2420559	2	124910001	A	G	-5.80E-04	PRS-CSx
Colorectum	PRS7	rs2421069	4	134975713	C	T	7.95E-04	PRS-CSx
Colorectum	PRS7	rs2421449	3	168520730	A	G	-1.48E-04	PRS-CSx
Colorectum	PRS7	rs2421772	3	169415294	T	C	2.21E-04	PRS-CSx
Colorectum	PRS7	rs2423154	20	6392681	G	A	1.49E-03	PRS-CSx
Colorectum	PRS7	rs2423157	20	6396990	A	G	1.35E-03	PRS-CSx
Colorectum	PRS7	rs2423160	20	6402559	A	G	9.61E-04	PRS-CSx
Colorectum	PRS7	rs2423265	20	7794075	G	A	6.49E-04	PRS-CSx
Colorectum	PRS7	rs2423268	20	7799315	G	A	4.71E-04	PRS-CSx
Colorectum	PRS7	rs2423269	20	7803558	G	A	6.84E-04	PRS-CSx
Colorectum	PRS7	rs2423273	20	7806720	G	A	3.68E-04	PRS-CSx
Colorectum	PRS7	rs2423276	20	7807681	T	C	3.55E-04	PRS-CSx
Colorectum	PRS7	rs2423277	20	7810226	G	T	3.91E-04	PRS-CSx
Colorectum	PRS7	rs2423278	20	7812208	G	A	5.75E-04	PRS-CSx
Colorectum	PRS7	rs2423279	20	7812350	C	T	3.40E-04	PRS-CSx
Colorectum	PRS7	rs2423290	20	7818261	T	G	5.95E-04	PRS-CSx
Colorectum	PRS7	rs2423294	20	7819768	A	G	3.27E-04	PRS-CSx
Colorectum	PRS7	rs2423303	20	7827822	T	G	3.39E-04	PRS-CSx
Colorectum	PRS7	rs2423305	20	7830342	G	T	6.12E-04	PRS-CSx
Colorectum	PRS7	rs2423317	20	7835617	G	A	6.42E-04	PRS-CSx
Colorectum	PRS7	rs2423322	20	7873112	G	A	1.11E-03	PRS-CSx
Colorectum	PRS7	rs2423366	20	8517451	A	G	4.92E-05	PRS-CSx
Colorectum	PRS7	rs2424992	20	33012060	T	C	-1.50E-04	PRS-CSx
Colorectum	PRS7	rs2425141	20	34350123	G	A	3.14E-04	PRS-CSx
Colorectum	PRS7	rs2426090	20	47382056	G	A	-5.87E-04	PRS-CSx
Colorectum	PRS7	rs2426097	20	47385467	T	C	-9.55E-04	PRS-CSx
Colorectum	PRS7	rs2426103	20	47394490	A	C	-5.69E-04	PRS-CSx
Colorectum	PRS7	rs2426729	20	56012674	C	T	-1.40E-03	PRS-CSx
Colorectum	PRS7	rs2427300	20	60946237	T	C	-8.04E-04	PRS-CSx
Colorectum	PRS7	rs2427311	20	60970123	G	A	-1.07E-03	PRS-CSx
Colorectum	PRS7	rs2427323	20	60981155	G	A	-4.78E-04	PRS-CSx
Colorectum	PRS7	rs2427381	20	61314785	T	C	-5.39E-04	PRS-CSx
Colorectum	PRS7	rs2429191	5	129376192	T	G	-1.74E-04	PRS-CSx
Colorectum	PRS7	rs2429387	17	60767015	G	A	-1.58E-03	PRS-CSx
Colorectum	PRS7	rs2429389	17	60759340	A	G	-2.00E-03	PRS-CSx
Colorectum	PRS7	rs2430866	12	80080618	C	T	2.20E-04	PRS-CSx
Colorectum	PRS7	rs2431194	5	129380642	G	A	-8.91E-05	PRS-CSx
Colorectum	PRS7	rs2431197	5	129386095	A	G	-1.90E-04	PRS-CSx
Colorectum	PRS7	rs2434309	5	79302455	C	T	1.30E-04	PRS-CSx
Colorectum	PRS7	rs2437149	1	231490105	C	T	2.23E-04	PRS-CSx
Colorectum	PRS7	rs2437841	8	117620112	A	G	-4.07E-03	PRS-CSx
Colorectum	PRS7	rs2439397	15	67013821	G	A	-6.80E-04	PRS-CSx
Colorectum	PRS7	rs2439411	15	66983982	G	A	-4.17E-03	PRS-CSx
Colorectum	PRS7	rs2444892	8	99017396	T	C	-6.51E-04	PRS-CSx
Colorectum	PRS7	rs244524	5	143728206	C	T	-2.29E-04	PRS-CSx
Colorectum	PRS7	rs244525	5	143728091	A	G	-3.77E-04	PRS-CSx
Colorectum	PRS7	rs244526	5	143727167	A	G	-3.63E-04	PRS-CSx
Colorectum	PRS7	rs244533	5	143706155	G	A	-1.34E-04	PRS-CSx
Colorectum	PRS7	rs2445828	19	50941807	A	G	-7.09E-04	PRS-CSx
Colorectum	PRS7	rs2445830	19	50914613	C	T	7.20E-04	PRS-CSx
Colorectum	PRS7	rs2445837	19	50921272	C	T	7.61E-04	PRS-CSx

Colorectum	PRS7	rs2446916	8	101126733	A	G	-1.29E-04	PRS-CSx
Colorectum	PRS7	rs2446917	8	101145709	T	G	-1.16E-04	PRS-CSx
Colorectum	PRS7	rs244743	5	129259316	G	A	-1.49E-04	PRS-CSx
Colorectum	PRS7	rs244744	5	129259267	G	A	-4.63E-05	PRS-CSx
Colorectum	PRS7	rs2448339	10	62525906	G	A	5.70E-04	PRS-CSx
Colorectum	PRS7	rs2448340	10	62526660	A	G	7.23E-04	PRS-CSx
Colorectum	PRS7	rs2449346	8	22415992	C	T	-5.07E-05	PRS-CSx
Colorectum	PRS7	rs2450113	8	117621213	C	T	-4.79E-03	PRS-CSx
Colorectum	PRS7	rs2451136	8	119238894	T	C	-1.76E-03	PRS-CSx
Colorectum	PRS7	rs2451731	6	26624822	A	C	-8.62E-05	PRS-CSx
Colorectum	PRS7	rs2451741	6	26629404	A	G	-2.22E-04	PRS-CSx
Colorectum	PRS7	rs2451933	5	79306599	A	G	2.02E-04	PRS-CSx
Colorectum	PRS7	rs245237	5	127219305	A	G	-1.69E-04	PRS-CSx
Colorectum	PRS7	rs2453236	1	221016277	T	C	-6.33E-04	PRS-CSx
Colorectum	PRS7	rs2453635	8	101122411	A	G	-2.05E-04	PRS-CSx
Colorectum	PRS7	rs2453639	8	101162134	G	T	-1.85E-04	PRS-CSx
Colorectum	PRS7	rs2453641	8	101163986	T	G	-6.71E-05	PRS-CSx
Colorectum	PRS7	rs2453643	8	101166455	G	A	-3.59E-05	PRS-CSx
Colorectum	PRS7	rs2453645	8	101170413	G	A	-8.45E-05	PRS-CSx
Colorectum	PRS7	rs2453646	8	101170471	A	G	-1.34E-04	PRS-CSx
Colorectum	PRS7	rs2453648	8	101174089	T	G	-8.17E-05	PRS-CSx
Colorectum	PRS7	rs2453656	8	101196824	G	A	-4.34E-05	PRS-CSx
Colorectum	PRS7	rs2453657	8	101197876	A	G	-1.03E-04	PRS-CSx
Colorectum	PRS7	rs2454976	13	85566590	C	T	2.33E-04	PRS-CSx
Colorectum	PRS7	rs2456452	8	128197080	A	G	-9.52E-04	PRS-CSx
Colorectum	PRS7	rs2456761	10	62530542	C	A	4.78E-04	PRS-CSx
Colorectum	PRS7	rs2460290	17	60768846	T	C	-9.79E-04	PRS-CSx
Colorectum	PRS7	rs2460300	17	60759347	A	G	-1.57E-03	PRS-CSx
Colorectum	PRS7	rs2460798	15	76176128	C	T	-1.96E-04	PRS-CSx
Colorectum	PRS7	rs2460950	19	37753239	G	A	-9.02E-04	PRS-CSx
Colorectum	PRS7	rs2461221	10	123781339	A	G	1.51E-04	PRS-CSx
Colorectum	PRS7	rs246134	16	69474517	A	G	5.64E-04	PRS-CSx
Colorectum	PRS7	rs246136	16	69477185	G	A	2.50E-04	PRS-CSx
Colorectum	PRS7	rs246141	16	69459591	T	C	2.82E-04	PRS-CSx
Colorectum	PRS7	rs2461628	7	54564464	T	C	4.03E-05	PRS-CSx
Colorectum	PRS7	rs2462634	7	47433628	T	C	-8.91E-06	PRS-CSx
Colorectum	PRS7	rs2463107	12	80093317	C	A	1.89E-04	PRS-CSx
Colorectum	PRS7	rs2463169	12	80079048	A	G	2.34E-04	PRS-CSx
Colorectum	PRS7	rs2464528	4	89817664	C	A	3.18E-04	PRS-CSx
Colorectum	PRS7	rs2465427	17	60766804	C	T	-1.61E-03	PRS-CSx
Colorectum	PRS7	rs2465428	17	60766786	G	T	-1.39E-03	PRS-CSx
Colorectum	PRS7	rs2465429	17	60766483	A	G	-2.67E-03	PRS-CSx
Colorectum	PRS7	rs2465663	2	201503176	G	A	-1.69E-03	PRS-CSx
Colorectum	PRS7	rs2467013	12	46455445	T	C	-1.50E-04	PRS-CSx
Colorectum	PRS7	rs2469	6	43299323	C	T	-2.81E-04	PRS-CSx
Colorectum	PRS7	rs2469073	15	66979185	C	T	-2.88E-04	PRS-CSx
Colorectum	PRS7	rs2470450	3	10468280	A	G	-8.63E-04	PRS-CSx
Colorectum	PRS7	rs2471023	15	62796859	C	T	-6.07E-04	PRS-CSx
Colorectum	PRS7	rs2472793	6	14748675	T	C	-1.70E-03	PRS-CSx
Colorectum	PRS7	rs2472797	6	14763148	G	A	-6.12E-04	PRS-CSx
Colorectum	PRS7	rs2472802	6	14767419	G	A	-3.81E-04	PRS-CSx
Colorectum	PRS7	rs2473156	6	144660881	G	A	-2.03E-03	PRS-CSx
Colorectum	PRS7	rs2474870	11	33150253	T	C	-2.25E-04	PRS-CSx
Colorectum	PRS7	rs2477185	1	182266517	C	A	2.62E-04	PRS-CSx
Colorectum	PRS7	rs2477777	1	1314172	C	T	2.33E-04	PRS-CSx
Colorectum	PRS7	rs2479319	9	219528	C	T	-2.39E-04	PRS-CSx

Colorectum	PRS7	rs2479328	9	261855	G	A	-3.31E-04	PRS-CSx
Colorectum	PRS7	rs2479960	13	111985936	C	T	-4.49E-04	PRS-CSx
Colorectum	PRS7	rs2480792	10	115773296	G	A	3.81E-04	PRS-CSx
Colorectum	PRS7	rs2481955	13	28583581	A	G	1.28E-03	PRS-CSx
Colorectum	PRS7	rs2483278	1	3252104	C	T	7.80E-04	PRS-CSx
Colorectum	PRS7	rs2484967	9	221874	G	A	-1.16E-04	PRS-CSx
Colorectum	PRS7	rs2485250	13	85551462	A	C	3.85E-04	PRS-CSx
Colorectum	PRS7	rs2486675	9	33845079	T	G	-1.33E-04	PRS-CSx
Colorectum	PRS7	rs2486729	1	231535584	C	T	2.08E-04	PRS-CSx
Colorectum	PRS7	rs2489425	10	129034251	A	G	-1.39E-03	PRS-CSx
Colorectum	PRS7	rs2489556	10	26959202	G	A	2.53E-03	PRS-CSx
Colorectum	PRS7	rs2490036	6	3852794	T	C	1.58E-03	PRS-CSx
Colorectum	PRS7	rs2491101	9	130215249	T	C	4.04E-04	PRS-CSx
Colorectum	PRS7	rs249180	12	95404658	G	T	-2.64E-04	PRS-CSx
Colorectum	PRS7	rs2491878	14	54003552	G	A	-6.24E-04	PRS-CSx
Colorectum	PRS7	rs2492883	16	1902105	A	G	-3.34E-05	PRS-CSx
Colorectum	PRS7	rs2493278	1	3307746	T	C	7.98E-04	PRS-CSx
Colorectum	PRS7	rs2493309	1	3296951	T	C	3.21E-03	PRS-CSx
Colorectum	PRS7	rs2493581	13	102559851	G	A	-3.27E-04	PRS-CSx
Colorectum	PRS7	rs2493582	13	102567809	C	T	-7.92E-04	PRS-CSx
Colorectum	PRS7	rs249414	5	40733694	A	G	2.45E-04	PRS-CSx
Colorectum	PRS7	rs249429	5	40782239	C	T	1.67E-04	PRS-CSx
Colorectum	PRS7	rs2494449	1	182272989	T	C	5.56E-04	PRS-CSx
Colorectum	PRS7	rs2494452	1	182270625	A	G	3.86E-04	PRS-CSx
Colorectum	PRS7	rs2494462	1	182267565	A	G	3.99E-04	PRS-CSx
Colorectum	PRS7	rs2494465	1	182264773	T	C	4.64E-04	PRS-CSx
Colorectum	PRS7	rs2494701	6	26634432	C	T	-1.66E-04	PRS-CSx
Colorectum	PRS7	rs2495365	1	2498027	A	G	-2.03E-04	PRS-CSx
Colorectum	PRS7	rs2496021	10	106491136	C	T	2.70E-04	PRS-CSx
Colorectum	PRS7	rs249733	5	141887906	T	C	7.22E-04	PRS-CSx
Colorectum	PRS7	rs2500081	6	14772897	C	T	-1.74E-03	PRS-CSx
Colorectum	PRS7	rs2501257	1	22228836	A	G	3.41E-04	PRS-CSx
Colorectum	PRS7	rs2501351	1	159816313	G	T	6.04E-04	PRS-CSx
Colorectum	PRS7	rs2501575	10	111849864	A	G	-3.80E-04	PRS-CSx
Colorectum	PRS7	rs2502921	9	30804872	A	G	8.72E-04	PRS-CSx
Colorectum	PRS7	rs2503775	6	98521600	A	G	2.45E-04	PRS-CSx
Colorectum	PRS7	rs2504458	1	108227361	C	T	-5.45E-04	PRS-CSx
Colorectum	PRS7	rs2504565	6	26656890	G	A	-2.36E-04	PRS-CSx
Colorectum	PRS7	rs2504571	6	26643435	C	T	-7.07E-05	PRS-CSx
Colorectum	PRS7	rs2504599	6	26641060	C	T	-1.74E-04	PRS-CSx
Colorectum	PRS7	rs2504600	6	26648334	C	A	-1.38E-04	PRS-CSx
Colorectum	PRS7	rs2507983	6	31345794	G	A	-2.86E-04	PRS-CSx
Colorectum	PRS7	rs2509132	11	69933696	A	G	6.40E-04	PRS-CSx
Colorectum	PRS7	rs2509562	11	74248756	G	A	-2.76E-04	PRS-CSx
Colorectum	PRS7	rs2509563	11	74245483	T	C	-2.27E-04	PRS-CSx
Colorectum	PRS7	rs2509623	11	120109967	T	C	3.24E-04	PRS-CSx
Colorectum	PRS7	rs2509656	11	119208576	A	G	-3.47E-04	PRS-CSx
Colorectum	PRS7	rs2510881	11	72899673	C	T	-4.25E-04	PRS-CSx
Colorectum	PRS7	rs251108	16	69051208	T	C	1.09E-04	PRS-CSx
Colorectum	PRS7	rs2511653	8	117621592	T	C	-2.18E-03	PRS-CSx
Colorectum	PRS7	rs2511847	11	119203359	A	G	-4.34E-04	PRS-CSx
Colorectum	PRS7	rs2511988	11	57379170	G	A	6.09E-04	PRS-CSx
Colorectum	PRS7	rs2512982	11	86415790	C	T	1.64E-04	PRS-CSx
Colorectum	PRS7	rs2512987	11	86414282	G	A	1.71E-04	PRS-CSx
Colorectum	PRS7	rs2512988	11	86414027	G	A	2.69E-04	PRS-CSx
Colorectum	PRS7	rs2514680	8	101222499	A	G	-1.81E-05	PRS-CSx

Colorectum	PRS7	rs2514690	8	101167395	T	C	-3.97E-05	PRS-CSx
Colorectum	PRS7	rs2514778	8	97703256	G	A	-3.05E-04	PRS-CSx
Colorectum	PRS7	rs2515279	11	69923299	T	C	4.32E-04	PRS-CSx
Colorectum	PRS7	rs2515913	12	109546114	T	C	6.06E-04	PRS-CSx
Colorectum	PRS7	rs2516393	6	31506744	A	C	-1.48E-05	PRS-CSx
Colorectum	PRS7	rs2516739	16	2097158	A	G	-1.38E-04	PRS-CSx
Colorectum	PRS7	rs2516740	16	2097110	C	A	-1.14E-04	PRS-CSx
Colorectum	PRS7	rs2517595	6	30084549	A	G	-1.57E-04	PRS-CSx
Colorectum	PRS7	rs2517597	6	30081189	A	G	-2.09E-04	PRS-CSx
Colorectum	PRS7	rs2517598	6	30080274	A	G	-1.85E-04	PRS-CSx
Colorectum	PRS7	rs2517672	6	29937262	G	A	1.23E-06	PRS-CSx
Colorectum	PRS7	rs2517715	6	29917439	C	T	-2.34E-04	PRS-CSx
Colorectum	PRS7	rs2517889	6	29803388	A	G	-2.97E-05	PRS-CSx
Colorectum	PRS7	rs2517892	6	29801474	T	C	-1.08E-04	PRS-CSx
Colorectum	PRS7	rs251796	16	69395434	G	A	6.82E-04	PRS-CSx
Colorectum	PRS7	rs2518580	6	53004757	A	G	-1.17E-05	PRS-CSx
Colorectum	PRS7	rs2519454	9	20625228	A	G	2.09E-03	PRS-CSx
Colorectum	PRS7	rs2522474	4	105745818	G	A	1.76E-04	PRS-CSx
Colorectum	PRS7	rs2522480	4	105764654	C	T	8.97E-05	PRS-CSx
Colorectum	PRS7	rs2522482	4	105768938	C	T	1.75E-04	PRS-CSx
Colorectum	PRS7	rs2522484	4	105771144	C	T	-1.77E-04	PRS-CSx
Colorectum	PRS7	rs2522490	4	105778330	A	C	-4.00E-04	PRS-CSx
Colorectum	PRS7	rs2523395	6	29702510	A	G	-3.18E-05	PRS-CSx
Colorectum	PRS7	rs2523508	6	31508761	C	T	-1.62E-05	PRS-CSx
Colorectum	PRS7	rs2523511	6	31506854	C	T	9.39E-05	PRS-CSx
Colorectum	PRS7	rs2523612	6	31321123	G	T	-1.85E-05	PRS-CSx
Colorectum	PRS7	rs2523638	6	31344273	T	C	-1.71E-06	PRS-CSx
Colorectum	PRS7	rs2523769	6	29811022	A	C	-6.14E-05	PRS-CSx
Colorectum	PRS7	rs2523790	6	29803650	T	G	-3.90E-05	PRS-CSx
Colorectum	PRS7	rs2523933	6	29932292	T	G	-3.41E-05	PRS-CSx
Colorectum	PRS7	rs2523946	6	29941943	T	C	-2.32E-04	PRS-CSx
Colorectum	PRS7	rs2523960	6	29939952	C	T	-2.01E-05	PRS-CSx
Colorectum	PRS7	rs2523962	6	29939544	A	G	-3.40E-05	PRS-CSx
Colorectum	PRS7	rs2523971	6	29938258	A	C	4.19E-06	PRS-CSx
Colorectum	PRS7	rs2523981	6	30083182	T	C	-1.35E-04	PRS-CSx
Colorectum	PRS7	rs2523984	6	30082003	T	C	-1.95E-04	PRS-CSx
Colorectum	PRS7	rs2523985	6	30081334	C	T	-7.56E-05	PRS-CSx
Colorectum	PRS7	rs2523986	6	30081246	T	C	-1.06E-04	PRS-CSx
Colorectum	PRS7	rs2523987	6	30079993	C	A	-2.93E-04	PRS-CSx
Colorectum	PRS7	rs2523988	6	30079129	C	T	-3.09E-04	PRS-CSx
Colorectum	PRS7	rs2523989	6	30078275	T	C	1.18E-04	PRS-CSx
Colorectum	PRS7	rs2523992	6	30075103	G	T	-2.41E-04	PRS-CSx
Colorectum	PRS7	rs2524123	6	31265314	C	T	2.05E-05	PRS-CSx
Colorectum	PRS7	rs2525846	12	113453553	T	C	5.59E-04	PRS-CSx
Colorectum	PRS7	rs2526431	3	62056891	A	G	-9.20E-04	PRS-CSx
Colorectum	PRS7	rs2527886	7	99552168	C	T	-3.67E-04	PRS-CSx
Colorectum	PRS7	rs2527894	7	99538841	G	A	-5.08E-04	PRS-CSx
Colorectum	PRS7	rs2527897	7	99534080	G	A	-3.17E-04	PRS-CSx
Colorectum	PRS7	rs2527923	7	99567455	T	C	-2.29E-04	PRS-CSx
Colorectum	PRS7	rs2529672	2	199176477	C	T	2.34E-03	PRS-CSx
Colorectum	PRS7	rs2530253	5	129416927	T	C	-3.10E-04	PRS-CSx
Colorectum	PRS7	rs2531854	17	9204090	A	G	5.28E-04	PRS-CSx
Colorectum	PRS7	rs2532015	16	4128631	C	T	9.50E-04	PRS-CSx
Colorectum	PRS7	rs2533878	7	2878707	A	G	-1.36E-03	PRS-CSx
Colorectum	PRS7	rs2534733	17	12379980	G	A	-4.63E-04	PRS-CSx
Colorectum	PRS7	rs2535584	17	5633982	G	A	-5.76E-04	PRS-CSx

Colorectum	PRS7	rs2542485	2	71533729	G	A	-6.69E-04	PRS-CSx
Colorectum	PRS7	rs2542489	2	71558113	G	A	-3.58E-04	PRS-CSx
Colorectum	PRS7	rs2542505	2	71587797	C	T	-5.20E-04	PRS-CSx
Colorectum	PRS7	rs2542532	2	71495639	T	C	-2.23E-04	PRS-CSx
Colorectum	PRS7	rs2542547	2	71531413	C	T	-3.39E-04	PRS-CSx
Colorectum	PRS7	rs2544616	5	143484902	C	T	-4.96E-04	PRS-CSx
Colorectum	PRS7	rs2544780	5	127214163	T	C	-1.62E-04	PRS-CSx
Colorectum	PRS7	rs254557	5	134446275	T	G	8.17E-04	PRS-CSx
Colorectum	PRS7	rs254560	5	134443606	A	G	1.36E-03	PRS-CSx
Colorectum	PRS7	rs254562	5	134441457	G	A	1.29E-03	PRS-CSx
Colorectum	PRS7	rs254563	5	134440426	A	G	1.45E-03	PRS-CSx
Colorectum	PRS7	rs254570	5	134436352	A	G	9.86E-04	PRS-CSx
Colorectum	PRS7	rs2546532	5	143595109	G	A	-7.34E-05	PRS-CSx
Colorectum	PRS7	rs2552241	8	5853884	T	C	1.20E-03	PRS-CSx
Colorectum	PRS7	rs2561401	6	39191384	C	T	4.87E-03	PRS-CSx
Colorectum	PRS7	rs2564114	2	61230647	G	A	-5.44E-04	PRS-CSx
Colorectum	PRS7	rs2566114	4	39052210	A	G	-4.80E-05	PRS-CSx
Colorectum	PRS7	rs2566116	4	39052838	T	C	2.55E-05	PRS-CSx
Colorectum	PRS7	rs2566117	4	39053022	G	A	-8.24E-05	PRS-CSx
Colorectum	PRS7	rs2566134	4	39074295	A	G	6.64E-05	PRS-CSx
Colorectum	PRS7	rs2566142	4	39081681	G	A	6.12E-05	PRS-CSx
Colorectum	PRS7	rs2566163	4	39129965	A	G	7.28E-05	PRS-CSx
Colorectum	PRS7	rs2566871	7	128843169	T	C	2.74E-04	PRS-CSx
Colorectum	PRS7	rs256739	19	37858660	G	A	-4.05E-04	PRS-CSx
Colorectum	PRS7	rs2567503	17	70713365	C	T	-1.39E-03	PRS-CSx
Colorectum	PRS7	rs2568664	2	183105424	T	C	1.17E-04	PRS-CSx
Colorectum	PRS7	rs2568667	2	183106758	T	G	2.31E-04	PRS-CSx
Colorectum	PRS7	rs2568923	10	118073895	A	G	1.53E-04	PRS-CSx
Colorectum	PRS7	rs2569343	5	133897103	A	G	2.12E-04	PRS-CSx
Colorectum	PRS7	rs2569432	19	51611073	G	A	7.57E-04	PRS-CSx
Colorectum	PRS7	rs2569433	19	51611263	G	A	7.52E-04	PRS-CSx
Colorectum	PRS7	rs257009	5	40771216	C	T	9.79E-05	PRS-CSx
Colorectum	PRS7	rs2572	14	73426290	T	C	4.46E-04	PRS-CSx
Colorectum	PRS7	rs2575337	16	1943418	A	G	7.38E-05	PRS-CSx
Colorectum	PRS7	rs2577346	10	118059344	G	A	3.77E-04	PRS-CSx
Colorectum	PRS7	rs2577356	10	118024590	C	A	-7.59E-04	PRS-CSx
Colorectum	PRS7	rs2577961	10	9261533	C	T	-5.06E-04	PRS-CSx
Colorectum	PRS7	rs2578160	4	94835289	T	C	2.93E-04	PRS-CSx
Colorectum	PRS7	rs2578162	4	94834868	A	G	3.28E-04	PRS-CSx
Colorectum	PRS7	rs2578246	9	90835589	G	A	-8.30E-04	PRS-CSx
Colorectum	PRS7	rs2580768	2	55214015	C	T	5.22E-04	PRS-CSx
Colorectum	PRS7	rs2580769	2	55214618	A	C	4.45E-04	PRS-CSx
Colorectum	PRS7	rs2580771	2	55215825	G	A	3.77E-04	PRS-CSx
Colorectum	PRS7	rs2581194	3	27927730	T	C	-5.20E-04	PRS-CSx
Colorectum	PRS7	rs2581824	3	53022408	C	A	7.97E-04	PRS-CSx
Colorectum	PRS7	rs2583084	8	70381185	A	G	-2.74E-04	PRS-CSx
Colorectum	PRS7	rs2583086	8	70380084	T	G	-1.98E-04	PRS-CSx
Colorectum	PRS7	rs2583405	4	102172981	A	G	-1.27E-04	PRS-CSx
Colorectum	PRS7	rs2584314	1	222204361	C	T	3.33E-04	PRS-CSx
Colorectum	PRS7	rs2584315	1	222206887	A	G	3.87E-04	PRS-CSx
Colorectum	PRS7	rs2584316	1	222211329	G	A	3.78E-04	PRS-CSx
Colorectum	PRS7	rs2584322	1	222227618	G	T	6.95E-04	PRS-CSx
Colorectum	PRS7	rs2584324	1	222228024	A	G	7.89E-04	PRS-CSx
Colorectum	PRS7	rs2584329	1	222212632	A	G	3.87E-04	PRS-CSx
Colorectum	PRS7	rs258622	5	75388834	T	C	6.38E-04	PRS-CSx
Colorectum	PRS7	rs2586532	17	4795307	T	C	4.37E-04	PRS-CSx

Colorectum	PRS7	rs258892	5	72305846	C	A	-2.85E-04	PRS-CSx
Colorectum	PRS7	rs258895	5	72331687	C	T	-2.60E-04	PRS-CSx
Colorectum	PRS7	rs2589400	5	133913553	G	A	-2.98E-04	PRS-CSx
Colorectum	PRS7	rs2593280	15	76150965	G	A	-4.14E-04	PRS-CSx
Colorectum	PRS7	rs25951	5	14693480	G	A	-4.68E-04	PRS-CSx
Colorectum	PRS7	rs25952	5	14695247	A	C	-7.46E-04	PRS-CSx
Colorectum	PRS7	rs2596250	13	49480391	C	T	2.06E-04	PRS-CSx
Colorectum	PRS7	rs2596446	6	31440195	G	A	-2.83E-04	PRS-CSx
Colorectum	PRS7	rs2596454	6	31436312	A	G	4.63E-05	PRS-CSx
Colorectum	PRS7	rs2598	20	47241618	G	A	-1.86E-04	PRS-CSx
Colorectum	PRS7	rs25986	5	14718036	G	T	-8.32E-04	PRS-CSx
Colorectum	PRS7	rs25988	5	14712514	G	A	-3.63E-04	PRS-CSx
Colorectum	PRS7	rs259919	6	30025503	A	G	8.33E-05	PRS-CSx
Colorectum	PRS7	rs25992	5	14707600	C	T	-5.18E-04	PRS-CSx
Colorectum	PRS7	rs259923	6	29994544	C	A	3.55E-05	PRS-CSx
Colorectum	PRS7	rs259934	6	30006876	T	C	-3.99E-05	PRS-CSx
Colorectum	PRS7	rs259939	6	30011581	C	T	1.35E-05	PRS-CSx
Colorectum	PRS7	rs259940	6	30011934	G	A	2.97E-05	PRS-CSx
Colorectum	PRS7	rs259943	6	30015330	C	T	-3.31E-05	PRS-CSx
Colorectum	PRS7	rs2602710	19	4912729	A	C	1.56E-03	PRS-CSx
Colorectum	PRS7	rs2603751	12	52453282	C	T	8.31E-04	PRS-CSx
Colorectum	PRS7	rs2604558	4	15107677	A	G	-1.01E-04	PRS-CSx
Colorectum	PRS7	rs2609255	4	89811195	G	T	1.78E-04	PRS-CSx
Colorectum	PRS7	rs2609260	4	89836819	C	T	4.02E-04	PRS-CSx
Colorectum	PRS7	rs2609261	4	89835485	A	G	2.71E-04	PRS-CSx
Colorectum	PRS7	rs2609262	4	89835438	A	G	2.98E-04	PRS-CSx
Colorectum	PRS7	rs2609264	4	89828080	C	T	1.71E-04	PRS-CSx
Colorectum	PRS7	rs2609265	4	89826966	T	C	3.29E-04	PRS-CSx
Colorectum	PRS7	rs2609818	10	14808696	T	G	-1.45E-03	PRS-CSx
Colorectum	PRS7	rs261032	5	169287116	T	C	-1.24E-03	PRS-CSx
Colorectum	PRS7	rs261038	5	169283210	A	G	4.35E-04	PRS-CSx
Colorectum	PRS7	rs261307	15	58653820	A	G	1.19E-03	PRS-CSx
Colorectum	PRS7	rs2613946	3	112825573	A	G	-2.39E-04	PRS-CSx
Colorectum	PRS7	rs2613964	3	112860780	A	G	-4.23E-04	PRS-CSx
Colorectum	PRS7	rs2615	8	99048329	T	C	-5.80E-04	PRS-CSx
Colorectum	PRS7	rs2616646	10	78889417	C	T	1.03E-04	PRS-CSx
Colorectum	PRS7	rs2617789	19	37960815	T	C	-7.64E-04	PRS-CSx
Colorectum	PRS7	rs2617805	19	49112295	C	T	-1.10E-03	PRS-CSx
Colorectum	PRS7	rs2623106	14	85264742	C	T	1.41E-03	PRS-CSx
Colorectum	PRS7	rs2623422	2	183118373	G	A	2.33E-04	PRS-CSx
Colorectum	PRS7	rs2623423	2	183120344	A	G	2.95E-04	PRS-CSx
Colorectum	PRS7	rs2624164	5	86460047	G	A	3.55E-05	PRS-CSx
Colorectum	PRS7	rs26307	5	14705665	T	C	-2.26E-04	PRS-CSx
Colorectum	PRS7	rs2636066	15	29420381	C	T	6.25E-04	PRS-CSx
Colorectum	PRS7	rs2637677	6	116837556	G	A	-4.63E-04	PRS-CSx
Colorectum	PRS7	rs2638436	12	20003805	A	G	-3.30E-04	PRS-CSx
Colorectum	PRS7	rs2642421	1	220977615	C	T	4.79E-04	PRS-CSx
Colorectum	PRS7	rs2642444	1	220974218	G	A	-1.36E-03	PRS-CSx
Colorectum	PRS7	rs2643885	1	2221222	C	A	-6.67E-04	PRS-CSx
Colorectum	PRS7	rs2644121	1	201881284	G	A	4.43E-04	PRS-CSx
Colorectum	PRS7	rs2644122	1	201810407	G	A	6.19E-04	PRS-CSx
Colorectum	PRS7	rs264577	2	159879349	T	C	-3.74E-04	PRS-CSx
Colorectum	PRS7	rs264578	2	159878926	G	A	3.71E-04	PRS-CSx
Colorectum	PRS7	rs264581	2	159971363	A	G	-3.52E-04	PRS-CSx
Colorectum	PRS7	rs264586	2	159967513	T	C	-1.36E-04	PRS-CSx
Colorectum	PRS7	rs264587	2	159966767	A	G	-1.43E-04	PRS-CSx

Colorectum	PRS7	rs264590	2	159975153	A	G	-7.06E-04	PRS-CSx
Colorectum	PRS7	rs264591	2	159975516	C	T	-3.30E-05	PRS-CSx
Colorectum	PRS7	rs264592	2	159976285	G	A	-5.20E-04	PRS-CSx
Colorectum	PRS7	rs264593	2	159977433	T	C	-1.71E-05	PRS-CSx
Colorectum	PRS7	rs264594	2	159979253	A	G	-2.01E-04	PRS-CSx
Colorectum	PRS7	rs264601	2	159882932	A	G	-4.23E-04	PRS-CSx
Colorectum	PRS7	rs264604	2	159886836	T	C	-3.75E-04	PRS-CSx
Colorectum	PRS7	rs264607	2	159888711	G	A	1.04E-04	PRS-CSx
Colorectum	PRS7	rs264612	2	159867835	C	A	1.33E-04	PRS-CSx
Colorectum	PRS7	rs264618	2	159873905	A	G	4.40E-04	PRS-CSx
Colorectum	PRS7	rs264636	2	159957058	T	C	-2.59E-04	PRS-CSx
Colorectum	PRS7	rs264639	2	159958620	C	T	-5.35E-04	PRS-CSx
Colorectum	PRS7	rs264653	2	159926849	C	T	7.84E-05	PRS-CSx
Colorectum	PRS7	rs264655	2	159923969	G	A	2.10E-04	PRS-CSx
Colorectum	PRS7	rs2647364	15	25172192	T	C	-1.14E-03	PRS-CSx
Colorectum	PRS7	rs264799	8	78389484	A	G	3.97E-04	PRS-CSx
Colorectum	PRS7	rs264826	8	78370662	A	G	2.92E-04	PRS-CSx
Colorectum	PRS7	rs2648558	3	9367619	A	G	4.74E-04	PRS-CSx
Colorectum	PRS7	rs2649097	8	118381545	A	C	8.28E-04	PRS-CSx
Colorectum	PRS7	rs2649588	1	1314015	C	T	2.72E-04	PRS-CSx
Colorectum	PRS7	rs2649589	1	1311716	G	A	3.32E-04	PRS-CSx
Colorectum	PRS7	rs265009	5	79137556	C	A	-1.54E-03	PRS-CSx
Colorectum	PRS7	rs2651256	14	59192645	T	C	4.38E-04	PRS-CSx
Colorectum	PRS7	rs2654715	4	127644271	T	C	-4.28E-04	PRS-CSx
Colorectum	PRS7	rs2654918	18	53969951	A	C	-2.11E-04	PRS-CSx
Colorectum	PRS7	rs2654976	15	99550237	G	A	-8.74E-04	PRS-CSx
Colorectum	PRS7	rs26576	5	72298256	C	A	-5.10E-04	PRS-CSx
Colorectum	PRS7	rs2660300	1	98528211	A	C	-2.61E-04	PRS-CSx
Colorectum	PRS7	rs266115	19	51333238	G	A	1.64E-03	PRS-CSx
Colorectum	PRS7	rs2664056	1	239350538	T	G	1.21E-07	PRS-CSx
Colorectum	PRS7	rs2664057	1	239350728	A	G	-1.17E-04	PRS-CSx
Colorectum	PRS7	rs266457	11	33339593	T	C	-3.07E-04	PRS-CSx
Colorectum	PRS7	rs266470	11	33362723	G	T	-4.67E-04	PRS-CSx
Colorectum	PRS7	rs266476	11	33358431	G	A	-3.45E-04	PRS-CSx
Colorectum	PRS7	rs2665896	10	34315931	A	C	-6.61E-04	PRS-CSx
Colorectum	PRS7	rs2666013	17	74098875	T	G	4.71E-04	PRS-CSx
Colorectum	PRS7	rs2669833	3	129604169	G	A	1.44E-04	PRS-CSx
Colorectum	PRS7	rs2669909	3	113265727	T	C	-1.32E-04	PRS-CSx
Colorectum	PRS7	rs2670348	3	35846872	A	C	-1.01E-03	PRS-CSx
Colorectum	PRS7	rs2670625	4	89978867	C	T	-1.74E-04	PRS-CSx
Colorectum	PRS7	rs2670726	2	71568282	A	G	-4.54E-04	PRS-CSx
Colorectum	PRS7	rs2670740	2	71531856	A	G	-2.95E-04	PRS-CSx
Colorectum	PRS7	rs2670752	2	71573808	G	A	-5.26E-04	PRS-CSx
Colorectum	PRS7	rs2670901	3	129626226	T	G	3.73E-04	PRS-CSx
Colorectum	PRS7	rs2670902	3	129616006	G	A	1.77E-04	PRS-CSx
Colorectum	PRS7	rs2672680	15	29418573	A	G	2.06E-04	PRS-CSx
Colorectum	PRS7	rs2673087	22	45682975	A	G	7.18E-04	PRS-CSx
Colorectum	PRS7	rs267428	11	33293428	G	A	-3.08E-04	PRS-CSx
Colorectum	PRS7	rs267442	11	33270782	A	G	-6.07E-04	PRS-CSx
Colorectum	PRS7	rs267449	11	33281328	C	T	-2.17E-04	PRS-CSx
Colorectum	PRS7	rs2679648	6	122350496	T	C	3.29E-05	PRS-CSx
Colorectum	PRS7	rs2684908	12	51065617	C	T	1.67E-04	PRS-CSx
Colorectum	PRS7	rs2691678	3	41230323	G	A	1.19E-03	PRS-CSx
Colorectum	PRS7	rs2694661	12	80114135	A	G	1.34E-04	PRS-CSx
Colorectum	PRS7	rs2694831	12	80086780	A	G	3.42E-04	PRS-CSx
Colorectum	PRS7	rs2694978	12	20009962	A	G	3.15E-04	PRS-CSx

Colorectum	PRS7	rs2694989	12	19976210	T	C	3.81E-04	PRS-CSx
Colorectum	PRS7	rs2696346	2	182607223	A	G	-1.64E-03	PRS-CSx
Colorectum	PRS7	rs2696820	16	86304691	A	G	-1.51E-03	PRS-CSx
Colorectum	PRS7	rs2696923	11	49053601	A	G	-2.04E-04	PRS-CSx
Colorectum	PRS7	rs2696935	11	49111467	A	G	-3.52E-04	PRS-CSx
Colorectum	PRS7	rs2697936	15	33053402	G	A	-4.04E-04	PRS-CSx
Colorectum	PRS7	rs2698174	18	68746110	G	A	-8.56E-04	PRS-CSx
Colorectum	PRS7	rs2698177	18	68747115	A	G	-1.22E-03	PRS-CSx
Colorectum	PRS7	rs2698193	2	61231014	T	C	-6.15E-04	PRS-CSx
Colorectum	PRS7	rs2699365	2	124916161	A	C	-4.51E-04	PRS-CSx
Colorectum	PRS7	rs2699367	2	124922818	T	C	-3.73E-04	PRS-CSx
Colorectum	PRS7	rs2700201	3	112829442	G	A	-2.38E-04	PRS-CSx
Colorectum	PRS7	rs2701124	12	52448157	A	G	7.69E-04	PRS-CSx
Colorectum	PRS7	rs2701129	12	52429477	G	T	1.79E-03	PRS-CSx
Colorectum	PRS7	rs2701316	7	128843757	G	A	-4.37E-04	PRS-CSx
Colorectum	PRS7	rs2701618	12	113471860	T	C	7.16E-04	PRS-CSx
Colorectum	PRS7	rs2702550	4	15108645	A	G	-6.46E-05	PRS-CSx
Colorectum	PRS7	rs2704668	7	16520193	C	T	-6.25E-04	PRS-CSx
Colorectum	PRS7	rs270554	5	38037792	C	T	2.22E-04	PRS-CSx
Colorectum	PRS7	rs270558	5	38035570	C	A	1.26E-04	PRS-CSx
Colorectum	PRS7	rs270560	5	38033014	G	A	4.11E-04	PRS-CSx
Colorectum	PRS7	rs270563	5	38028535	G	A	2.36E-04	PRS-CSx
Colorectum	PRS7	rs270567	5	38023641	A	C	3.29E-04	PRS-CSx
Colorectum	PRS7	rs270728	1	45083937	A	G	-1.61E-03	PRS-CSx
Colorectum	PRS7	rs2708696	4	69348883	A	G	-3.73E-04	PRS-CSx
Colorectum	PRS7	rs2708699	4	69341037	T	C	-2.79E-03	PRS-CSx
Colorectum	PRS7	rs2709437	2	6123492	T	C	2.66E-04	PRS-CSx
Colorectum	PRS7	rs2710646	2	63134879	A	C	7.68E-04	PRS-CSx
Colorectum	PRS7	rs2711935	4	39162457	C	T	8.77E-05	PRS-CSx
Colorectum	PRS7	rs2711940	4	39065091	G	A	-7.42E-05	PRS-CSx
Colorectum	PRS7	rs2711941	4	39064162	A	C	-1.77E-04	PRS-CSx
Colorectum	PRS7	rs2711943	4	39062436	T	C	2.14E-05	PRS-CSx
Colorectum	PRS7	rs2711952	4	39056818	A	G	-1.03E-04	PRS-CSx
Colorectum	PRS7	rs2711982	4	39043997	T	C	4.35E-05	PRS-CSx
Colorectum	PRS7	rs2711988	4	39152748	T	C	7.82E-05	PRS-CSx
Colorectum	PRS7	rs2711990	4	39151736	G	T	5.48E-05	PRS-CSx
Colorectum	PRS7	rs2712012	4	39077136	G	A	-4.39E-05	PRS-CSx
Colorectum	PRS7	rs2712355	3	113596326	C	T	-4.34E-04	PRS-CSx
Colorectum	PRS7	rs2712429	3	128336221	A	C	1.57E-03	PRS-CSx
Colorectum	PRS7	rs2715364	18	63744324	A	G	6.66E-04	PRS-CSx
Colorectum	PRS7	rs2715544	4	179493721	G	A	5.51E-04	PRS-CSx
Colorectum	PRS7	rs2716122	1	59430345	C	A	6.65E-04	PRS-CSx
Colorectum	PRS7	rs2722427	8	40483270	A	C	-1.28E-03	PRS-CSx
Colorectum	PRS7	rs2725603	15	54144472	A	G	-1.21E-03	PRS-CSx
Colorectum	PRS7	rs2725749	4	105833288	A	C	-4.55E-04	PRS-CSx
Colorectum	PRS7	rs2725750	4	105747315	A	G	6.15E-05	PRS-CSx
Colorectum	PRS7	rs2725767	4	105753265	A	G	8.11E-05	PRS-CSx
Colorectum	PRS7	rs2725769	4	105756169	G	A	1.15E-04	PRS-CSx
Colorectum	PRS7	rs2725771	4	105758739	G	T	1.40E-04	PRS-CSx
Colorectum	PRS7	rs2726459	4	106184597	C	A	8.89E-04	PRS-CSx
Colorectum	PRS7	rs2726554	8	59784043	T	C	-1.95E-03	PRS-CSx
Colorectum	PRS7	rs272834	1	36622730	T	C	-1.09E-03	PRS-CSx
Colorectum	PRS7	rs2729696	3	14915532	T	C	-2.71E-04	PRS-CSx
Colorectum	PRS7	rs2730985	12	43130624	A	G	-1.65E-03	PRS-CSx
Colorectum	PRS7	rs2731602	12	19955649	A	G	3.64E-04	PRS-CSx
Colorectum	PRS7	rs2732916	2	18177667	A	G	4.57E-04	PRS-CSx

Colorectum	PRS7	rs2734971	6	29834449	A	G	1.03E-04	PRS-CSx
Colorectum	PRS7	rs2735005	6	29808464	G	A	3.33E-05	PRS-CSx
Colorectum	PRS7	rs2735046	6	29734098	A	G	-2.76E-04	PRS-CSx
Colorectum	PRS7	rs2735052	6	29701564	A	G	-2.97E-04	PRS-CSx
Colorectum	PRS7	rs2735067	6	29951106	G	A	5.07E-06	PRS-CSx
Colorectum	PRS7	rs2735078	6	29941400	G	A	-5.87E-05	PRS-CSx
Colorectum	PRS7	rs2735097	6	29915301	A	C	-4.08E-04	PRS-CSx
Colorectum	PRS7	rs27356	5	14722441	C	T	-5.54E-04	PRS-CSx
Colorectum	PRS7	rs2736537	11	5405534	T	C	-3.38E-04	PRS-CSx
Colorectum	PRS7	rs273747	18	23188194	T	C	-4.53E-04	PRS-CSx
Colorectum	PRS7	rs2739137	8	134185523	A	C	-3.84E-04	PRS-CSx
Colorectum	PRS7	rs2743951	6	29709234	T	C	-1.49E-04	PRS-CSx
Colorectum	PRS7	rs2745815	20	17767573	A	G	-1.06E-03	PRS-CSx
Colorectum	PRS7	rs2745952	1	208062260	T	C	-5.37E-04	PRS-CSx
Colorectum	PRS7	rs2749717	1	231489353	G	T	2.04E-04	PRS-CSx
Colorectum	PRS7	rs2750055	10	33405575	T	C	1.27E-03	PRS-CSx
Colorectum	PRS7	rs2755430	10	89415605	C	T	-4.93E-04	PRS-CSx
Colorectum	PRS7	rs2756320	6	105758292	G	A	-1.37E-04	PRS-CSx
Colorectum	PRS7	rs27593	5	76012913	C	T	-4.44E-04	PRS-CSx
Colorectum	PRS7	rs276191	19	31625175	G	A	1.79E-03	PRS-CSx
Colorectum	PRS7	rs2764841	1	2529360	A	G	-2.42E-04	PRS-CSx
Colorectum	PRS7	rs2764845	1	2528999	T	G	-1.30E-04	PRS-CSx
Colorectum	PRS7	rs2765015	1	1308982	A	G	3.97E-04	PRS-CSx
Colorectum	PRS7	rs2765021	1	1297422	C	T	2.68E-04	PRS-CSx
Colorectum	PRS7	rs2765033	1	1310924	T	C	4.35E-04	PRS-CSx
Colorectum	PRS7	rs2766618	10	78896996	A	C	-1.16E-04	PRS-CSx
Colorectum	PRS7	rs2768713	10	14813860	G	A	-1.58E-03	PRS-CSx
Colorectum	PRS7	rs276984	16	86199184	T	C	1.06E-03	PRS-CSx
Colorectum	PRS7	rs2772020	9	97286964	G	T	-6.84E-04	PRS-CSx
Colorectum	PRS7	rs2772021	9	97363354	C	T	-5.02E-04	PRS-CSx
Colorectum	PRS7	rs2772179	13	41662028	G	A	-7.33E-04	PRS-CSx
Colorectum	PRS7	rs2772401	9	19322931	A	G	4.38E-04	PRS-CSx
Colorectum	PRS7	rs2776514	14	54056982	T	C	-2.35E-04	PRS-CSx
Colorectum	PRS7	rs277757	9	89769882	A	G	4.74E-04	PRS-CSx
Colorectum	PRS7	rs2778022	9	90807568	T	C	-5.30E-04	PRS-CSx
Colorectum	PRS7	rs2781008	9	86262537	A	G	4.42E-04	PRS-CSx
Colorectum	PRS7	rs2784070	9	139647074	G	A	1.08E-03	PRS-CSx
Colorectum	PRS7	rs2787933	6	88420902	A	G	5.74E-04	PRS-CSx
Colorectum	PRS7	rs2789948	1	223184459	G	T	2.67E-04	PRS-CSx
Colorectum	PRS7	rs2789951	1	223186683	A	G	-2.00E-05	PRS-CSx
Colorectum	PRS7	rs2790743	1	222113294	A	C	-8.66E-04	PRS-CSx
Colorectum	PRS7	rs2790745	1	222212115	C	T	7.37E-04	PRS-CSx
Colorectum	PRS7	rs2790746	1	222216000	C	T	2.98E-04	PRS-CSx
Colorectum	PRS7	rs2790760	1	222109518	A	G	-7.39E-05	PRS-CSx
Colorectum	PRS7	rs2790897	1	231491712	C	A	2.78E-04	PRS-CSx
Colorectum	PRS7	rs2790898	1	201919674	T	C	1.93E-04	PRS-CSx
Colorectum	PRS7	rs27911	5	14707378	G	A	-7.81E-04	PRS-CSx
Colorectum	PRS7	rs2793639	1	58036812	C	A	-2.90E-04	PRS-CSx
Colorectum	PRS7	rs27952	5	72316437	A	G	-4.42E-04	PRS-CSx
Colorectum	PRS7	rs27958	5	142253686	G	A	-4.37E-04	PRS-CSx
Colorectum	PRS7	rs2797470	10	29105453	G	T	7.50E-04	PRS-CSx
Colorectum	PRS7	rs2798429	9	130257191	A	G	4.18E-04	PRS-CSx
Colorectum	PRS7	rs2799077	6	28234597	T	C	-1.18E-04	PRS-CSx
Colorectum	PRS7	rs2800846	1	222122590	C	T	-6.07E-05	PRS-CSx
Colorectum	PRS7	rs2800866	1	222207647	A	G	3.28E-04	PRS-CSx
Colorectum	PRS7	rs2803213	13	101604450	A	C	-7.20E-04	PRS-CSx

Colorectum	PRS7	rs2804609	10	113847037	T	C	-1.27E-03	PRS-CSx
Colorectum	PRS7	rs2804610	10	113847054	A	G	-4.42E-04	PRS-CSx
Colorectum	PRS7	rs2808611	1	231548480	G	A	2.37E-04	PRS-CSx
Colorectum	PRS7	rs2810098	14	71433911	G	A	-4.31E-04	PRS-CSx
Colorectum	PRS7	rs2810489	9	136922700	G	A	-2.85E-04	PRS-CSx
Colorectum	PRS7	rs2810491	9	136923331	T	C	-4.19E-04	PRS-CSx
Colorectum	PRS7	rs2811768	9	139668443	C	T	4.96E-04	PRS-CSx
Colorectum	PRS7	rs2811915	9	86254293	A	G	5.73E-04	PRS-CSx
Colorectum	PRS7	rs2814837	9	90817188	A	G	-1.54E-04	PRS-CSx
Colorectum	PRS7	rs2814839	9	90819763	T	C	-1.86E-04	PRS-CSx
Colorectum	PRS7	rs2815074	6	39124008	T	C	3.83E-04	PRS-CSx
Colorectum	PRS7	rs2815385	1	67542083	A	G	-9.26E-06	PRS-CSx
Colorectum	PRS7	rs2815496	10	28081288	A	C	-1.34E-03	PRS-CSx
Colorectum	PRS7	rs281797	3	193311756	C	T	-4.17E-04	PRS-CSx
Colorectum	PRS7	rs281808	3	193283519	A	C	-4.13E-04	PRS-CSx
Colorectum	PRS7	rs2819346	1	201882087	C	A	4.91E-04	PRS-CSx
Colorectum	PRS7	rs2819370	1	201915055	T	C	1.85E-04	PRS-CSx
Colorectum	PRS7	rs2820292	1	201784287	C	A	4.32E-04	PRS-CSx
Colorectum	PRS7	rs2820295	1	201800868	A	G	3.39E-04	PRS-CSx
Colorectum	PRS7	rs2820304	1	201918527	G	T	2.82E-04	PRS-CSx
Colorectum	PRS7	rs2820311	1	201841476	G	A	4.61E-04	PRS-CSx
Colorectum	PRS7	rs2820312	1	201869257	A	G	5.44E-04	PRS-CSx
Colorectum	PRS7	rs2820313	1	201870221	G	A	5.19E-04	PRS-CSx
Colorectum	PRS7	rs2820315	1	201872264	T	C	1.94E-04	PRS-CSx
Colorectum	PRS7	rs2822837	21	16064137	C	T	-5.70E-04	PRS-CSx
Colorectum	PRS7	rs2822839	21	16072859	C	T	-3.33E-04	PRS-CSx
Colorectum	PRS7	rs2822841	21	16076547	C	T	-2.72E-04	PRS-CSx
Colorectum	PRS7	rs2822872	21	16091027	A	G	-3.80E-04	PRS-CSx
Colorectum	PRS7	rs2823980	21	18062714	C	A	-1.44E-03	PRS-CSx
Colorectum	PRS7	rs2823984	21	18065768	T	C	-1.39E-03	PRS-CSx
Colorectum	PRS7	rs2824816	21	19791091	C	A	8.91E-04	PRS-CSx
Colorectum	PRS7	rs2827300	21	23615318	G	T	-1.48E-03	PRS-CSx
Colorectum	PRS7	rs2830213	21	27795018	G	A	-1.68E-03	PRS-CSx
Colorectum	PRS7	rs2832115	21	30221409	A	G	-1.27E-04	PRS-CSx
Colorectum	PRS7	rs2832127	21	30274207	T	C	-2.66E-04	PRS-CSx
Colorectum	PRS7	rs2832131	21	30275947	A	C	-1.98E-04	PRS-CSx
Colorectum	PRS7	rs2832132	21	30276353	T	C	-2.48E-04	PRS-CSx
Colorectum	PRS7	rs2832142	21	30311767	G	A	-1.88E-04	PRS-CSx
Colorectum	PRS7	rs2832149	21	30354112	T	C	-2.32E-04	PRS-CSx
Colorectum	PRS7	rs2832157	21	30415629	G	A	-2.07E-04	PRS-CSx
Colorectum	PRS7	rs2832222	21	30527777	C	T	-2.25E-04	PRS-CSx
Colorectum	PRS7	rs2832290	21	30728863	A	G	-2.40E-04	PRS-CSx
Colorectum	PRS7	rs2833306	21	32524232	T	C	1.73E-03	PRS-CSx
Colorectum	PRS7	rs283350	5	109323840	T	C	1.54E-03	PRS-CSx
Colorectum	PRS7	rs2833584	21	33336188	G	A	2.30E-03	PRS-CSx
Colorectum	PRS7	rs2834210	21	34786574	A	G	8.72E-05	PRS-CSx
Colorectum	PRS7	rs2834220	21	34823252	C	T	-1.03E-04	PRS-CSx
Colorectum	PRS7	rs2834222	21	34824771	G	A	-2.76E-06	PRS-CSx
Colorectum	PRS7	rs2834224	21	34825318	T	C	-1.17E-04	PRS-CSx
Colorectum	PRS7	rs2834229	21	34834727	A	G	-2.64E-06	PRS-CSx
Colorectum	PRS7	rs2834549	21	36004539	G	A	-3.11E-04	PRS-CSx
Colorectum	PRS7	rs2834562	21	36020811	A	C	-4.37E-04	PRS-CSx
Colorectum	PRS7	rs2834756	21	36464234	T	C	5.90E-04	PRS-CSx
Colorectum	PRS7	rs2834761	21	36471078	T	C	6.69E-04	PRS-CSx
Colorectum	PRS7	rs28365789	5	125936318	G	A	-2.01E-03	PRS-CSx
Colorectum	PRS7	rs28365931	12	113622716	G	A	2.40E-03	PRS-CSx

Colorectum	PRS7	rs2836701	21	40209798	C	A	2.63E-04	PRS-CSx
Colorectum	PRS7	rs2836706	21	40211489	G	A	7.63E-04	PRS-CSx
Colorectum	PRS7	rs2836714	21	40235290	T	C	3.58E-04	PRS-CSx
Colorectum	PRS7	rs2836718	21	40237243	C	T	6.20E-04	PRS-CSx
Colorectum	PRS7	rs2836737	21	40265938	C	T	5.75E-04	PRS-CSx
Colorectum	PRS7	rs2836824	21	40380823	C	T	-1.33E-03	PRS-CSx
Colorectum	PRS7	rs2836845	21	40395343	T	C	1.48E-03	PRS-CSx
Colorectum	PRS7	rs2836862	21	40425289	T	C	6.87E-04	PRS-CSx
Colorectum	PRS7	rs2837756	21	41995874	C	T	9.74E-04	PRS-CSx
Colorectum	PRS7	rs2839187	21	47690949	A	G	4.99E-04	PRS-CSx
Colorectum	PRS7	rs2839226	21	47786494	C	T	4.15E-04	PRS-CSx
Colorectum	PRS7	rs2839227	21	47786524	G	A	4.11E-04	PRS-CSx
Colorectum	PRS7	rs2839232	21	47792874	A	G	4.15E-04	PRS-CSx
Colorectum	PRS7	rs2839234	21	47796874	A	G	3.75E-04	PRS-CSx
Colorectum	PRS7	rs2839235	21	47800592	C	T	1.00E-03	PRS-CSx
Colorectum	PRS7	rs2839240	21	47819986	G	A	7.01E-04	PRS-CSx
Colorectum	PRS7	rs2839251	21	47841692	T	C	6.90E-04	PRS-CSx
Colorectum	PRS7	rs2839274	21	47895181	A	G	1.32E-04	PRS-CSx
Colorectum	PRS7	rs2839283	21	47902993	T	C	3.49E-04	PRS-CSx
Colorectum	PRS7	rs2839321	21	47973610	A	G	1.53E-04	PRS-CSx
Colorectum	PRS7	rs2839349	21	48017197	G	A	6.57E-05	PRS-CSx
Colorectum	PRS7	rs2840803	6	73535037	G	A	-8.76E-04	PRS-CSx
Colorectum	PRS7	rs28411034	1	38276997	A	G	-1.92E-05	PRS-CSx
Colorectum	PRS7	rs2841642	6	43302285	G	T	-2.23E-04	PRS-CSx
Colorectum	PRS7	rs28419191	5	138844599	T	C	2.62E-03	PRS-CSx
Colorectum	PRS7	rs2842647	6	41645564	A	G	-6.77E-04	PRS-CSx
Colorectum	PRS7	rs28431145	5	68807200	T	C	-6.57E-04	PRS-CSx
Colorectum	PRS7	rs2843159	1	2235672	T	C	-5.29E-04	PRS-CSx
Colorectum	PRS7	rs2843163	1	2227422	T	C	-8.00E-04	PRS-CSx
Colorectum	PRS7	rs2843401	1	2528133	C	T	-1.92E-04	PRS-CSx
Colorectum	PRS7	rs2843402	1	2528682	C	T	-1.96E-04	PRS-CSx
Colorectum	PRS7	rs2843403	1	2529097	C	T	-2.43E-04	PRS-CSx
Colorectum	PRS7	rs2843404	1	2530558	C	T	-2.32E-04	PRS-CSx
Colorectum	PRS7	rs28435150	1	38407014	T	C	-9.93E-04	PRS-CSx
Colorectum	PRS7	rs2844505	6	31439063	C	T	-3.12E-04	PRS-CSx
Colorectum	PRS7	rs2844649	6	30904272	A	G	-3.70E-04	PRS-CSx
Colorectum	PRS7	rs2844793	6	30080496	A	G	-7.92E-05	PRS-CSx
Colorectum	PRS7	rs2844804	6	29942126	G	T	-1.62E-04	PRS-CSx
Colorectum	PRS7	rs2844831	6	29706277	C	T	-1.27E-04	PRS-CSx
Colorectum	PRS7	rs28450036	1	38431554	C	T	1.80E-04	PRS-CSx
Colorectum	PRS7	rs28455716	9	140148189	T	G	6.10E-04	PRS-CSx
Colorectum	PRS7	rs2845573	11	61601908	G	A	-5.71E-04	PRS-CSx
Colorectum	PRS7	rs28461580	12	122605204	A	G	1.81E-04	PRS-CSx
Colorectum	PRS7	rs284652	19	41928652	T	C	2.45E-04	PRS-CSx
Colorectum	PRS7	rs284661	19	41932120	T	C	1.35E-04	PRS-CSx
Colorectum	PRS7	rs284662	19	41932275	C	T	1.15E-04	PRS-CSx
Colorectum	PRS7	rs284663	19	41932612	T	C	2.72E-04	PRS-CSx
Colorectum	PRS7	rs28469609	1	38367404	T	C	-1.15E-04	PRS-CSx
Colorectum	PRS7	rs28477803	1	38398694	T	C	-4.70E-05	PRS-CSx
Colorectum	PRS7	rs2848871	18	68750255	T	C	-8.93E-04	PRS-CSx
Colorectum	PRS7	rs2848872	18	68748610	T	C	-9.56E-04	PRS-CSx
Colorectum	PRS7	rs28489109	5	68808501	A	G	-5.06E-04	PRS-CSx
Colorectum	PRS7	rs2850335	4	102172941	T	C	-1.61E-04	PRS-CSx
Colorectum	PRS7	rs2850336	4	102178058	A	G	-3.58E-04	PRS-CSx
Colorectum	PRS7	rs2850359	4	102164220	T	G	-3.03E-04	PRS-CSx
Colorectum	PRS7	rs2850845	18	75048151	C	T	2.42E-03	PRS-CSx

Colorectum	PRS7	rs2850971	4	102184626	C	T	-2.93E-04	PRS-CSx
Colorectum	PRS7	rs2851572	11	94498275	T	C	4.13E-04	PRS-CSx
Colorectum	PRS7	rs2851582	11	94521573	A	G	3.13E-04	PRS-CSx
Colorectum	PRS7	rs2851589	11	94507137	A	G	2.85E-04	PRS-CSx
Colorectum	PRS7	rs2851682	11	61616012	G	A	-3.41E-04	PRS-CSx
Colorectum	PRS7	rs2852786	11	61514085	C	T	2.84E-04	PRS-CSx
Colorectum	PRS7	rs28536662	3	119688191	G	A	1.57E-04	PRS-CSx
Colorectum	PRS7	rs2853672	5	1292983	C	A	-9.11E-03	PRS-CSx
Colorectum	PRS7	rs2853676	5	1288547	T	C	-8.77E-04	PRS-CSx
Colorectum	PRS7	rs2853677	5	1287194	G	A	-2.67E-04	PRS-CSx
Colorectum	PRS7	rs28558979	5	68820348	G	T	-9.15E-04	PRS-CSx
Colorectum	PRS7	rs28562181	5	68824310	T	C	-8.55E-04	PRS-CSx
Colorectum	PRS7	rs28564871	14	51368610	G	A	8.40E-04	PRS-CSx
Colorectum	PRS7	rs28567783	13	114740268	T	C	7.84E-04	PRS-CSx
Colorectum	PRS7	rs2857596	6	31567422	G	T	-1.32E-04	PRS-CSx
Colorectum	PRS7	rs2857709	6	31532814	A	G	-8.06E-05	PRS-CSx
Colorectum	PRS7	rs28583821	1	38296719	A	G	2.40E-04	PRS-CSx
Colorectum	PRS7	rs28594927	4	167566167	T	C	1.88E-04	PRS-CSx
Colorectum	PRS7	rs2860001	5	40278849	A	G	3.05E-03	PRS-CSx
Colorectum	PRS7	rs28605425	12	122596148	C	T	1.51E-04	PRS-CSx
Colorectum	PRS7	rs28610581	15	75812474	G	A	3.92E-04	PRS-CSx
Colorectum	PRS7	rs2861598	3	173544812	G	A	3.94E-04	PRS-CSx
Colorectum	PRS7	rs2861602	3	173508722	G	A	4.47E-04	PRS-CSx
Colorectum	PRS7	rs28616120	5	68785298	G	A	-4.11E-04	PRS-CSx
Colorectum	PRS7	rs28625842	1	38310786	A	G	-4.04E-05	PRS-CSx
Colorectum	PRS7	rs2863028	11	43985156	G	A	-4.05E-04	PRS-CSx
Colorectum	PRS7	rs28631372	9	140148051	G	A	3.58E-04	PRS-CSx
Colorectum	PRS7	rs2863202	1	67584335	T	C	2.63E-04	PRS-CSx
Colorectum	PRS7	rs2863266	18	53971903	C	A	-2.80E-04	PRS-CSx
Colorectum	PRS7	rs28645661	1	116050880	T	C	1.23E-03	PRS-CSx
Colorectum	PRS7	rs2864680	5	132637766	A	G	1.76E-03	PRS-CSx
Colorectum	PRS7	rs28652632	12	122590465	G	T	2.44E-04	PRS-CSx
Colorectum	PRS7	rs2867776	20	42541246	T	C	8.86E-04	PRS-CSx
Colorectum	PRS7	rs28678708	1	38382611	T	C	-9.12E-05	PRS-CSx
Colorectum	PRS7	rs286872	11	34702703	A	G	4.08E-04	PRS-CSx
Colorectum	PRS7	rs286889	11	34680853	G	T	-1.45E-03	PRS-CSx
Colorectum	PRS7	rs286896	11	34674475	G	A	-2.03E-03	PRS-CSx
Colorectum	PRS7	rs286900	11	34669090	A	C	3.48E-04	PRS-CSx
Colorectum	PRS7	rs2869290	4	86061990	C	T	1.09E-03	PRS-CSx
Colorectum	PRS7	rs2869966	4	89869078	C	T	3.91E-04	PRS-CSx
Colorectum	PRS7	rs2869967	4	89869332	T	C	3.42E-04	PRS-CSx
Colorectum	PRS7	rs2871026	22	19378723	T	C	9.01E-05	PRS-CSx
Colorectum	PRS7	rs2871027	22	19381331	T	C	1.06E-04	PRS-CSx
Colorectum	PRS7	rs2871198	4	26292555	T	C	4.93E-04	PRS-CSx
Colorectum	PRS7	rs2872373	3	78107128	C	A	-5.26E-04	PRS-CSx
Colorectum	PRS7	rs2872795	4	17330381	T	C	-8.50E-04	PRS-CSx
Colorectum	PRS7	rs28739509	1	38366907	C	T	-1.36E-04	PRS-CSx
Colorectum	PRS7	rs2874568	4	165364934	C	T	-2.48E-04	PRS-CSx
Colorectum	PRS7	rs2874670	8	26245658	A	G	-7.92E-05	PRS-CSx
Colorectum	PRS7	rs2875040	4	22482353	T	C	-3.20E-04	PRS-CSx
Colorectum	PRS7	rs28757122	15	51581330	C	T	2.91E-04	PRS-CSx
Colorectum	PRS7	rs2876032	20	6399494	T	C	1.81E-03	PRS-CSx
Colorectum	PRS7	rs2876669	6	148216298	A	C	4.11E-04	PRS-CSx
Colorectum	PRS7	rs2877307	7	45178291	A	G	-4.89E-04	PRS-CSx
Colorectum	PRS7	rs2877617	17	52698425	T	C	-2.88E-04	PRS-CSx
Colorectum	PRS7	rs2877946	17	57281310	T	G	1.80E-04	PRS-CSx

Colorectum	PRS7	rs2880102	6	168583032	T	C	-8.93E-04	PRS-CSx
Colorectum	PRS7	rs2881345	14	57667723	A	C	5.16E-04	PRS-CSx
Colorectum	PRS7	rs2881770	14	58643877	G	A	2.54E-04	PRS-CSx
Colorectum	PRS7	rs2883160	1	239254410	G	A	-3.29E-04	PRS-CSx
Colorectum	PRS7	rs2883250	16	86302131	A	G	-1.05E-03	PRS-CSx
Colorectum	PRS7	rs2883457	17	40590472	T	C	1.20E-03	PRS-CSx
Colorectum	PRS7	rs2884567	10	20501566	A	G	-4.88E-04	PRS-CSx
Colorectum	PRS7	rs2884830	1	213834509	G	A	-1.22E-03	PRS-CSx
Colorectum	PRS7	rs2885821	1	200162783	A	G	1.29E-03	PRS-CSx
Colorectum	PRS7	rs2887043	18	69549683	T	C	1.33E-03	PRS-CSx
Colorectum	PRS7	rs2887208	2	183380753	C	T	6.77E-05	PRS-CSx
Colorectum	PRS7	rs2887217	2	183375853	A	G	2.44E-04	PRS-CSx
Colorectum	PRS7	rs2888	12	65641985	T	C	1.77E-03	PRS-CSx
Colorectum	PRS7	rs2890854	9	9161898	C	T	9.30E-04	PRS-CSx
Colorectum	PRS7	rs2891168	9	22098619	A	G	8.75E-04	PRS-CSx
Colorectum	PRS7	rs2891575	12	116178812	G	A	4.59E-04	PRS-CSx
Colorectum	PRS7	rs2892240	1	247698164	T	C	-6.96E-04	PRS-CSx
Colorectum	PRS7	rs2892491	5	8599225	G	A	6.27E-04	PRS-CSx
Colorectum	PRS7	rs2892592	10	132054599	T	C	-1.16E-03	PRS-CSx
Colorectum	PRS7	rs2892614	10	8857470	G	A	3.31E-04	PRS-CSx
Colorectum	PRS7	rs2893506	7	35441671	T	C	-5.37E-04	PRS-CSx
Colorectum	PRS7	rs2893812	12	120419963	G	A	6.77E-04	PRS-CSx
Colorectum	PRS7	rs2894125	10	72344579	A	G	9.06E-04	PRS-CSx
Colorectum	PRS7	rs2894401	6	35408959	A	G	1.94E-04	PRS-CSx
Colorectum	PRS7	rs289459	15	98267695	T	C	-7.24E-04	PRS-CSx
Colorectum	PRS7	rs2895562	11	94528056	C	A	3.41E-04	PRS-CSx
Colorectum	PRS7	rs2895719	5	100929556	T	C	5.19E-04	PRS-CSx
Colorectum	PRS7	rs2895728	12	79802302	T	C	-5.31E-04	PRS-CSx
Colorectum	PRS7	rs2895873	8	83851852	G	A	-2.93E-04	PRS-CSx
Colorectum	PRS7	rs2896200	14	92711766	T	C	7.30E-04	PRS-CSx
Colorectum	PRS7	rs2898449	21	42814495	T	G	8.33E-04	PRS-CSx
Colorectum	PRS7	rs2899417	15	48700103	T	C	-9.14E-05	PRS-CSx
Colorectum	PRS7	rs2899473	15	51519073	T	C	4.61E-04	PRS-CSx
Colorectum	PRS7	rs2902323	16	68736292	T	C	-1.24E-03	PRS-CSx
Colorectum	PRS7	rs290356	10	8822658	A	G	1.75E-04	PRS-CSx
Colorectum	PRS7	rs2903825	11	61488968	T	C	1.24E-04	PRS-CSx
Colorectum	PRS7	rs2904086	4	86922723	C	T	-6.62E-04	PRS-CSx
Colorectum	PRS7	rs2904185	4	89139832	C	T	2.20E-03	PRS-CSx
Colorectum	PRS7	rs2905722	6	31449327	A	G	-1.60E-04	PRS-CSx
Colorectum	PRS7	rs2907105	5	11086565	G	A	-8.08E-05	PRS-CSx
Colorectum	PRS7	rs2909235	8	129185918	T	G	-1.18E-04	PRS-CSx
Colorectum	PRS7	rs2909569	5	31315377	T	C	-8.05E-05	PRS-CSx
Colorectum	PRS7	rs2909623	8	229694	T	C	-2.06E-03	PRS-CSx
Colorectum	PRS7	rs2910211	5	159876888	A	G	-8.49E-05	PRS-CSx
Colorectum	PRS7	rs2915239	11	94501849	G	A	4.44E-04	PRS-CSx
Colorectum	PRS7	rs2919384	8	32559943	G	A	-4.12E-04	PRS-CSx
Colorectum	PRS7	rs2921755	8	117915557	A	G	1.32E-03	PRS-CSx
Colorectum	PRS7	rs292182	5	36954812	G	A	-1.53E-04	PRS-CSx
Colorectum	PRS7	rs292194	5	36941767	T	C	-2.11E-04	PRS-CSx
Colorectum	PRS7	rs2926539	3	101586824	A	G	7.32E-04	PRS-CSx
Colorectum	PRS7	rs2926585	8	76410861	G	A	7.22E-04	PRS-CSx
Colorectum	PRS7	rs2929528	15	72269337	A	C	-2.19E-04	PRS-CSx
Colorectum	PRS7	rs2933259	3	148518875	C	T	-8.72E-04	PRS-CSx
Colorectum	PRS7	rs2935644	5	2716034	G	T	7.19E-04	PRS-CSx
Colorectum	PRS7	rs2936604	8	118393167	T	C	8.66E-04	PRS-CSx
Colorectum	PRS7	rs2938616	1	110364275	G	T	3.11E-03	PRS-CSx

Colorectum	PRS7	rs293919	3	14919254	G	A	-3.91E-04	PRS-CSx
Colorectum	PRS7	rs293923	3	14920010	T	G	-3.59E-04	PRS-CSx
Colorectum	PRS7	rs293924	3	14920105	A	G	-2.58E-04	PRS-CSx
Colorectum	PRS7	rs2941528	7	47494959	A	G	-1.23E-04	PRS-CSx
Colorectum	PRS7	rs2941549	7	47498364	A	G	-2.41E-04	PRS-CSx
Colorectum	PRS7	rs2943301	3	101658671	T	C	-4.52E-04	PRS-CSx
Colorectum	PRS7	rs294351	11	30718731	G	A	-9.21E-04	PRS-CSx
Colorectum	PRS7	rs2943559	8	76417937	G	A	7.82E-04	PRS-CSx
Colorectum	PRS7	rs2943568	8	76419046	A	C	5.87E-04	PRS-CSx
Colorectum	PRS7	rs2943584	8	76440918	A	C	9.97E-04	PRS-CSx
Colorectum	PRS7	rs294363	11	30707698	A	G	-6.36E-04	PRS-CSx
Colorectum	PRS7	rs294365	11	30710193	A	G	-6.51E-04	PRS-CSx
Colorectum	PRS7	rs2948926	2	7862742	G	T	-4.11E-04	PRS-CSx
Colorectum	PRS7	rs2948927	2	7856399	A	G	-6.58E-04	PRS-CSx
Colorectum	PRS7	rs2949938	17	65503997	A	G	2.96E-03	PRS-CSx
Colorectum	PRS7	rs2951868	8	6716344	T	C	-2.92E-04	PRS-CSx
Colorectum	PRS7	rs2955404	12	25424498	C	T	-5.05E-04	PRS-CSx
Colorectum	PRS7	rs2955725	15	76436248	T	G	-2.69E-03	PRS-CSx
Colorectum	PRS7	rs2957368	15	72329906	C	T	-1.31E-04	PRS-CSx
Colorectum	PRS7	rs2957725	15	72248205	A	C	-3.27E-04	PRS-CSx
Colorectum	PRS7	rs2961911	5	159861575	C	T	-9.39E-04	PRS-CSx
Colorectum	PRS7	rs2962799	5	31299254	T	C	2.59E-04	PRS-CSx
Colorectum	PRS7	rs296533	1	200865768	T	G	7.36E-04	PRS-CSx
Colorectum	PRS7	rs296724	9	93745381	T	C	-1.22E-03	PRS-CSx
Colorectum	PRS7	rs2967320	16	82303724	A	C	6.94E-04	PRS-CSx
Colorectum	PRS7	rs296962	5	36990483	C	T	-7.09E-05	PRS-CSx
Colorectum	PRS7	rs2970627	8	36884712	C	T	6.20E-04	PRS-CSx
Colorectum	PRS7	rs2971772	7	131407670	G	T	5.93E-03	PRS-CSx
Colorectum	PRS7	rs2972597	19	37838010	A	G	7.67E-04	PRS-CSx
Colorectum	PRS7	rs2973519	5	11078958	G	A	-2.46E-04	PRS-CSx
Colorectum	PRS7	rs2973523	5	11075285	T	C	-4.29E-04	PRS-CSx
Colorectum	PRS7	rs29743	5	40755280	G	T	2.05E-04	PRS-CSx
Colorectum	PRS7	rs2975498	8	32552189	C	T	-3.95E-04	PRS-CSx
Colorectum	PRS7	rs2976921	8	8266397	A	C	-5.72E-04	PRS-CSx
Colorectum	PRS7	rs2977904	8	76405582	T	C	5.17E-04	PRS-CSx
Colorectum	PRS7	rs2977918	8	76427727	G	A	4.82E-04	PRS-CSx
Colorectum	PRS7	rs2978487	8	23416034	T	C	4.55E-04	PRS-CSx
Colorectum	PRS7	rs2979107	8	146208961	A	G	6.37E-04	PRS-CSx
Colorectum	PRS7	rs2979111	8	146198217	C	A	4.97E-04	PRS-CSx
Colorectum	PRS7	rs2979146	8	8263945	G	A	-6.25E-04	PRS-CSx
Colorectum	PRS7	rs2980218	8	76306804	A	G	-5.27E-04	PRS-CSx
Colorectum	PRS7	rs2980221	8	76307739	A	C	-3.89E-04	PRS-CSx
Colorectum	PRS7	rs2980945	8	6715751	G	A	-8.36E-04	PRS-CSx
Colorectum	PRS7	rs2985431	1	182269701	A	C	4.04E-04	PRS-CSx
Colorectum	PRS7	rs2985432	1	182269763	C	T	4.22E-04	PRS-CSx
Colorectum	PRS7	rs2985434	1	182269948	G	A	5.56E-04	PRS-CSx
Colorectum	PRS7	rs2985855	1	2531372	C	A	-6.31E-05	PRS-CSx
Colorectum	PRS7	rs2985857	1	2532624	T	C	-1.80E-04	PRS-CSx
Colorectum	PRS7	rs2987325	13	67759847	C	T	-2.44E-03	PRS-CSx
Colorectum	PRS7	rs2988277	1	167431352	T	C	3.15E-04	PRS-CSx
Colorectum	PRS7	rs29897	5	143710896	T	G	-2.85E-04	PRS-CSx
Colorectum	PRS7	rs29916	5	93726134	C	T	-9.39E-04	PRS-CSx
Colorectum	PRS7	rs2991809	10	8821674	T	C	3.58E-04	PRS-CSx
Colorectum	PRS7	rs29924	5	93730489	A	G	-1.21E-03	PRS-CSx
Colorectum	PRS7	rs2995081	1	167568441	T	C	4.93E-04	PRS-CSx
Colorectum	PRS7	rs2996005	1	221496601	C	T	1.28E-04	PRS-CSx

Colorectum	PRS7	rs300035	16	86687834	G	A	4.73E-04	PRS-CSx
Colorectum	PRS7	rs300051	5	37088627	A	G	-1.30E-04	PRS-CSx
Colorectum	PRS7	rs300060	5	37019354	G	A	-1.37E-04	PRS-CSx
Colorectum	PRS7	rs300061	5	37022775	G	A	-1.11E-04	PRS-CSx
Colorectum	PRS7	rs300063	5	37023898	G	T	-1.09E-04	PRS-CSx
Colorectum	PRS7	rs3000922	1	12804402	G	A	2.18E-04	PRS-CSx
Colorectum	PRS7	rs3000929	1	12788406	G	A	2.02E-04	PRS-CSx
Colorectum	PRS7	rs3000930	1	12786070	A	G	2.76E-04	PRS-CSx
Colorectum	PRS7	rs3001179	1	194518771	C	A	-8.28E-04	PRS-CSx
Colorectum	PRS7	rs3001275	1	182268637	G	A	5.57E-04	PRS-CSx
Colorectum	PRS7	rs300272	1	55045983	A	G	7.07E-04	PRS-CSx
Colorectum	PRS7	rs300273	1	55045930	A	C	5.86E-04	PRS-CSx
Colorectum	PRS7	rs3007075	14	51604860	C	T	5.10E-04	PRS-CSx
Colorectum	PRS7	rs3007125	14	47267652	C	T	-2.77E-04	PRS-CSx
Colorectum	PRS7	rs3007168	14	51608293	A	G	3.55E-04	PRS-CSx
Colorectum	PRS7	rs3007169	14	51607897	C	T	3.43E-04	PRS-CSx
Colorectum	PRS7	rs3007285	13	105876544	C	T	-9.89E-04	PRS-CSx
Colorectum	PRS7	rs3008257	8	225590	G	T	-1.38E-03	PRS-CSx
Colorectum	PRS7	rs3008260	8	225168	T	C	-1.49E-03	PRS-CSx
Colorectum	PRS7	rs3008647	1	222759007	T	C	-1.41E-04	PRS-CSx
Colorectum	PRS7	rs300885	2	183133550	A	G	2.52E-04	PRS-CSx
Colorectum	PRS7	rs3009947	1	218689155	C	T	-4.58E-04	PRS-CSx
Colorectum	PRS7	rs3010402	1	116111105	A	G	7.59E-04	PRS-CSx
Colorectum	PRS7	rs3010403	1	116112425	G	A	5.59E-04	PRS-CSx
Colorectum	PRS7	rs3010406	1	116089793	A	G	1.23E-03	PRS-CSx
Colorectum	PRS7	rs3010876	1	12779560	T	C	3.56E-04	PRS-CSx
Colorectum	PRS7	rs3010877	1	12779618	T	C	3.85E-04	PRS-CSx
Colorectum	PRS7	rs3010885	1	12786273	T	C	2.42E-04	PRS-CSx
Colorectum	PRS7	rs3010886	1	12786363	C	T	3.50E-04	PRS-CSx
Colorectum	PRS7	rs3010888	1	12787632	C	T	5.48E-04	PRS-CSx
Colorectum	PRS7	rs3010890	1	12787980	A	G	1.99E-04	PRS-CSx
Colorectum	PRS7	rs3010903	1	12796044	G	T	3.08E-04	PRS-CSx
Colorectum	PRS7	rs301395	6	25486626	C	A	-7.38E-04	PRS-CSx
Colorectum	PRS7	rs3016756	9	34016516	C	T	-2.68E-04	PRS-CSx
Colorectum	PRS7	rs301859	5	37045841	A	G	-2.32E-04	PRS-CSx
Colorectum	PRS7	rs301901	5	37046626	A	G	-1.31E-04	PRS-CSx
Colorectum	PRS7	rs3019091	8	101408103	A	G	3.41E-04	PRS-CSx
Colorectum	PRS7	rs3025657	6	29511216	C	A	4.07E-04	PRS-CSx
Colorectum	PRS7	rs3027288	17	8007416	T	G	1.07E-03	PRS-CSx
Colorectum	PRS7	rs3031	17	5113423	A	G	5.64E-04	PRS-CSx
Colorectum	PRS7	rs303451	10	30777280	G	T	2.06E-03	PRS-CSx
Colorectum	PRS7	rs303795	12	52245938	T	C	-1.37E-03	PRS-CSx
Colorectum	PRS7	rs303940	13	72371415	G	A	4.87E-04	PRS-CSx
Colorectum	PRS7	rs303942	13	72369456	T	C	4.66E-04	PRS-CSx
Colorectum	PRS7	rs30454	19	39844176	A	G	4.42E-04	PRS-CSx
Colorectum	PRS7	rs30455	19	39843623	A	G	4.20E-04	PRS-CSx
Colorectum	PRS7	rs305565	1	64964558	G	A	5.85E-04	PRS-CSx
Colorectum	PRS7	rs305570	1	64981396	T	G	7.64E-04	PRS-CSx
Colorectum	PRS7	rs305576	1	64972899	T	C	7.15E-04	PRS-CSx
Colorectum	PRS7	rs305578	1	64976086	A	G	6.20E-04	PRS-CSx
Colorectum	PRS7	rs305580	1	64976251	G	T	9.23E-04	PRS-CSx
Colorectum	PRS7	rs305581	1	64976717	G	A	5.14E-04	PRS-CSx
Colorectum	PRS7	rs306050	4	23669223	A	C	-1.60E-03	PRS-CSx
Colorectum	PRS7	rs306063	4	23649426	A	G	-1.17E-03	PRS-CSx
Colorectum	PRS7	rs306612	12	27661728	T	C	-1.03E-03	PRS-CSx
Colorectum	PRS7	rs307380	1	1287127	G	A	5.33E-04	PRS-CSx

Colorectum	PRS7	rs307646	9	33932338	G	A	-1.52E-04	PRS-CSx
Colorectum	PRS7	rs307649	9	33932702	T	G	-6.52E-05	PRS-CSx
Colorectum	PRS7	rs307651	9	33935073	A	C	-6.97E-05	PRS-CSx
Colorectum	PRS7	rs307652	9	33935736	A	G	-4.26E-04	PRS-CSx
Colorectum	PRS7	rs307654	9	33948652	T	C	-3.96E-04	PRS-CSx
Colorectum	PRS7	rs307658	9	33941759	C	T	-1.54E-04	PRS-CSx
Colorectum	PRS7	rs307678	9	33971441	A	G	-1.90E-04	PRS-CSx
Colorectum	PRS7	rs307682	9	33973324	G	A	-1.59E-04	PRS-CSx
Colorectum	PRS7	rs307691	9	33928223	C	T	-1.20E-04	PRS-CSx
Colorectum	PRS7	rs307697	9	33982587	C	T	-1.13E-04	PRS-CSx
Colorectum	PRS7	rs30839	16	58745341	C	T	3.01E-04	PRS-CSx
Colorectum	PRS7	rs3087967	11	111156836	T	C	1.68E-03	PRS-CSx
Colorectum	PRS7	rs3091364	22	43414330	G	A	-2.16E-05	PRS-CSx
Colorectum	PRS7	rs3092989	13	32889363	A	G	-1.31E-03	PRS-CSx
Colorectum	PRS7	rs3093553	6	31549556	G	T	9.96E-04	PRS-CSx
Colorectum	PRS7	rs3093662	6	31544189	G	A	7.38E-04	PRS-CSx
Colorectum	PRS7	rs3093948	6	31501413	A	G	-2.47E-05	PRS-CSx
Colorectum	PRS7	rs3093976	6	31502861	A	G	4.38E-05	PRS-CSx
Colorectum	PRS7	rs3093978	6	31498497	C	A	3.45E-05	PRS-CSx
Colorectum	PRS7	rs3093981	6	31497457	A	G	6.68E-05	PRS-CSx
Colorectum	PRS7	rs3093983	6	31496925	G	A	-2.11E-05	PRS-CSx
Colorectum	PRS7	rs3093995	6	31488904	C	T	-6.57E-05	PRS-CSx
Colorectum	PRS7	rs3094014	6	31433558	A	G	-5.11E-04	PRS-CSx
Colorectum	PRS7	rs3094228	6	31429927	C	T	-5.71E-04	PRS-CSx
Colorectum	PRS7	rs3094450	16	71310697	A	G	-6.07E-04	PRS-CSx
Colorectum	PRS7	rs3094682	6	31264461	A	C	-2.87E-04	PRS-CSx
Colorectum	PRS7	rs3095971	4	181468920	A	G	4.46E-04	PRS-CSx
Colorectum	PRS7	rs3095972	4	181468882	C	A	-1.33E-03	PRS-CSx
Colorectum	PRS7	rs309722	4	177399566	T	C	1.23E-03	PRS-CSx
Colorectum	PRS7	rs309732	4	177394679	G	A	-7.36E-04	PRS-CSx
Colorectum	PRS7	rs309771	4	177454040	T	C	4.13E-04	PRS-CSx
Colorectum	PRS7	rs309773	4	177455498	G	T	5.14E-04	PRS-CSx
Colorectum	PRS7	rs309780	4	177462810	A	G	1.04E-03	PRS-CSx
Colorectum	PRS7	rs309791	4	177465478	A	C	1.03E-03	PRS-CSx
Colorectum	PRS7	rs3098183	15	50806469	C	T	-3.02E-04	PRS-CSx
Colorectum	PRS7	rs3098190	15	50812899	C	T	-4.05E-04	PRS-CSx
Colorectum	PRS7	rs3098264	9	107810900	A	G	-1.32E-03	PRS-CSx
Colorectum	PRS7	rs3099840	6	31430721	G	A	-4.22E-04	PRS-CSx
Colorectum	PRS7	rs31042	16	55329601	A	G	-5.43E-04	PRS-CSx
Colorectum	PRS7	rs3105104	2	95816365	G	A	-7.18E-05	PRS-CSx
Colorectum	PRS7	rs3105105	2	95848018	T	C	-1.57E-04	PRS-CSx
Colorectum	PRS7	rs3107669	3	119567101	A	C	1.11E-04	PRS-CSx
Colorectum	PRS7	rs3108408	7	76868598	G	A	-4.82E-04	PRS-CSx
Colorectum	PRS7	rs3108428	7	76885769	G	A	-5.58E-04	PRS-CSx
Colorectum	PRS7	rs3108450	7	76860287	C	T	2.95E-04	PRS-CSx
Colorectum	PRS7	rs3109119	4	69349401	G	A	-4.73E-04	PRS-CSx
Colorectum	PRS7	rs3112127	12	83839760	T	C	-5.93E-04	PRS-CSx
Colorectum	PRS7	rs3112228	2	95835614	C	T	-2.99E-05	PRS-CSx
Colorectum	PRS7	rs3112230	2	95846158	T	C	-1.93E-04	PRS-CSx
Colorectum	PRS7	rs3112996	2	95812372	G	A	-8.39E-05	PRS-CSx
Colorectum	PRS7	rs3112997	2	95818151	G	A	-2.17E-04	PRS-CSx
Colorectum	PRS7	rs3113741	4	181470273	C	T	-4.83E-04	PRS-CSx
Colorectum	PRS7	rs3114398	16	68706933	G	A	-1.36E-04	PRS-CSx
Colorectum	PRS7	rs311497	20	62221249	G	A	2.56E-04	PRS-CSx
Colorectum	PRS7	rs3115570	6	32222706	A	G	-2.63E-04	PRS-CSx
Colorectum	PRS7	rs3116788	6	29674197	C	T	-5.69E-05	PRS-CSx

Colorectum	PRS7	rs3117116	6	32367017	G	A	1.80E-04	PRS-CSx
Colorectum	PRS7	rs3117442	6	29349091	G	A	-3.16E-04	PRS-CSx
Colorectum	PRS7	rs3118111	10	9260186	C	T	-3.59E-04	PRS-CSx
Colorectum	PRS7	rs3118182	1	183167175	G	A	8.10E-07	PRS-CSx
Colorectum	PRS7	rs3118228	16	68710737	G	T	-2.12E-04	PRS-CSx
Colorectum	PRS7	rs3118235	16	68732977	A	G	-7.09E-04	PRS-CSx
Colorectum	PRS7	rs311847	14	59202529	A	C	5.85E-04	PRS-CSx
Colorectum	PRS7	rs311848	14	59201080	G	A	4.33E-04	PRS-CSx
Colorectum	PRS7	rs311855	14	59205524	A	G	-9.32E-04	PRS-CSx
Colorectum	PRS7	rs3122351	10	30952481	G	T	-9.59E-04	PRS-CSx
Colorectum	PRS7	rs3122569	1	12784232	G	A	4.57E-04	PRS-CSx
Colorectum	PRS7	rs3128451	1	12641488	T	G	-4.87E-04	PRS-CSx
Colorectum	PRS7	rs3128465	1	12784047	A	G	2.16E-04	PRS-CSx
Colorectum	PRS7	rs3128562	9	137745210	A	G	7.74E-04	PRS-CSx
Colorectum	PRS7	rs3128570	9	137743309	G	A	5.51E-04	PRS-CSx
Colorectum	PRS7	rs3129596	13	22561178	C	T	-1.10E-03	PRS-CSx
Colorectum	PRS7	rs3129860	6	32401079	A	G	1.93E-04	PRS-CSx
Colorectum	PRS7	rs3129868	6	32404377	A	C	2.39E-04	PRS-CSx
Colorectum	PRS7	rs3129883	6	32410137	T	C	-5.15E-04	PRS-CSx
Colorectum	PRS7	rs3129886	6	32410576	T	C	-6.50E-04	PRS-CSx
Colorectum	PRS7	rs3129888	6	32411726	G	A	-1.85E-03	PRS-CSx
Colorectum	PRS7	rs3129937	6	32336364	A	C	4.82E-04	PRS-CSx
Colorectum	PRS7	rs3129939	6	32336766	G	A	4.75E-04	PRS-CSx
Colorectum	PRS7	rs3129943	6	32338695	G	A	1.16E-03	PRS-CSx
Colorectum	PRS7	rs3130056	6	31499354	C	T	-6.44E-06	PRS-CSx
Colorectum	PRS7	rs3130057	6	31504587	G	T	3.12E-06	PRS-CSx
Colorectum	PRS7	rs3130058	6	31505887	T	C	-6.41E-05	PRS-CSx
Colorectum	PRS7	rs3130299	6	32203537	G	A	-1.39E-04	PRS-CSx
Colorectum	PRS7	rs313048	4	127624935	T	C	-1.20E-04	PRS-CSx
Colorectum	PRS7	rs313066	4	127618829	C	T	-2.23E-04	PRS-CSx
Colorectum	PRS7	rs3130695	6	31211050	A	G	-1.05E-04	PRS-CSx
Colorectum	PRS7	rs3130933	6	31132085	T	C	-4.28E-05	PRS-CSx
Colorectum	PRS7	rs313111	4	127632219	C	T	-5.61E-04	PRS-CSx
Colorectum	PRS7	rs3131115	6	30468791	T	C	1.39E-04	PRS-CSx
Colorectum	PRS7	rs31313	5	16690754	T	C	1.38E-03	PRS-CSx
Colorectum	PRS7	rs3131609	15	50802267	T	C	-1.44E-04	PRS-CSx
Colorectum	PRS7	rs3131628	6	31502767	C	T	3.69E-05	PRS-CSx
Colorectum	PRS7	rs3131863	6	29673483	G	A	4.04E-05	PRS-CSx
Colorectum	PRS7	rs3134883	10	6100725	A	G	-1.60E-05	PRS-CSx
Colorectum	PRS7	rs3134899	6	31473286	C	T	4.22E-06	PRS-CSx
Colorectum	PRS7	rs3135341	6	32398748	C	A	3.68E-04	PRS-CSx
Colorectum	PRS7	rs3135344	6	32395036	C	T	-9.51E-05	PRS-CSx
Colorectum	PRS7	rs3135392	6	32409242	A	C	2.85E-06	PRS-CSx
Colorectum	PRS7	rs3136516	11	46760756	A	G	5.61E-04	PRS-CSx
Colorectum	PRS7	rs3136641	19	39396649	T	C	4.13E-04	PRS-CSx
Colorectum	PRS7	rs314091	4	189446242	C	T	-1.03E-03	PRS-CSx
Colorectum	PRS7	rs315029	1	77042405	A	G	-4.83E-04	PRS-CSx
Colorectum	PRS7	rs3169425	16	1682281	A	G	1.73E-04	PRS-CSx
Colorectum	PRS7	rs316964	1	105064891	C	T	-1.87E-04	PRS-CSx
Colorectum	PRS7	rs3170766	1	55354302	A	C	-1.68E-04	PRS-CSx
Colorectum	PRS7	rs3176820	9	34686143	C	T	1.25E-03	PRS-CSx
Colorectum	PRS7	rs3178250	20	6760201	C	T	1.09E-03	PRS-CSx
Colorectum	PRS7	rs3181097	2	204570139	A	G	6.43E-04	PRS-CSx
Colorectum	PRS7	rs3181113	2	204601910	T	G	9.04E-04	PRS-CSx
Colorectum	PRS7	rs3181301	12	6345784	A	C	-1.01E-03	PRS-CSx
Colorectum	PRS7	rs3182911	17	17168164	G	A	2.73E-04	PRS-CSx

Colorectum	PRS7	rs3184991	10	101515316	C	T	1.73E-04	PRS-CSx
Colorectum	PRS7	rs31868	5	14637272	T	C	-2.24E-04	PRS-CSx
Colorectum	PRS7	rs318837	5	41366434	A	G	-5.57E-04	PRS-CSx
Colorectum	PRS7	rs31925	5	14674999	A	G	-2.62E-04	PRS-CSx
Colorectum	PRS7	rs31929	5	14678369	T	C	-3.35E-04	PRS-CSx
Colorectum	PRS7	rs31968	5	14701183	C	T	-5.69E-04	PRS-CSx
Colorectum	PRS7	rs3197188	16	84212108	T	C	7.07E-04	PRS-CSx
Colorectum	PRS7	rs3197233	1	109749071	T	C	1.02E-03	PRS-CSx
Colorectum	PRS7	rs31975	5	14606841	T	C	-4.28E-04	PRS-CSx
Colorectum	PRS7	rs3205537	3	73433369	A	G	1.59E-03	PRS-CSx
Colorectum	PRS7	rs3211958	7	80304072	G	A	-1.31E-03	PRS-CSx
Colorectum	PRS7	rs321651	18	8656598	T	C	4.32E-04	PRS-CSx
Colorectum	PRS7	rs3217830	12	4392530	T	C	-2.65E-03	PRS-CSx
Colorectum	PRS7	rs3217840	12	4394877	C	T	-1.55E-03	PRS-CSx
Colorectum	PRS7	rs3217862	12	4399087	G	T	-2.36E-03	PRS-CSx
Colorectum	PRS7	rs3217881	12	4402688	G	A	-1.42E-03	PRS-CSx
Colorectum	PRS7	rs3217882	12	4402817	G	A	-2.99E-03	PRS-CSx
Colorectum	PRS7	rs3217896	12	4404158	A	G	-1.12E-03	PRS-CSx
Colorectum	PRS7	rs3217898	12	4404376	G	A	-5.94E-04	PRS-CSx
Colorectum	PRS7	rs3217901	12	4405389	A	G	-1.74E-03	PRS-CSx
Colorectum	PRS7	rs3217926	12	4411683	C	T	-5.69E-05	PRS-CSx
Colorectum	PRS7	rs3217992	9	22003223	C	T	3.28E-04	PRS-CSx
Colorectum	PRS7	rs3218020	9	21997872	G	A	3.70E-04	PRS-CSx
Colorectum	PRS7	rs3219281	19	50887087	T	C	8.00E-04	PRS-CSx
Colorectum	PRS7	rs3219341	19	50898017	T	C	1.05E-03	PRS-CSx
Colorectum	PRS7	rs322019	5	55384562	A	G	6.17E-04	PRS-CSx
Colorectum	PRS7	rs323344	8	30702525	C	A	3.97E-03	PRS-CSx
Colorectum	PRS7	rs323345	8	30702602	C	T	4.43E-03	PRS-CSx
Colorectum	PRS7	rs324015	12	57490100	T	C	-1.25E-03	PRS-CSx
Colorectum	PRS7	rs324540	9	9119349	C	T	6.75E-04	PRS-CSx
Colorectum	PRS7	rs324541	9	9119327	C	T	6.28E-04	PRS-CSx
Colorectum	PRS7	rs324544	9	9108336	C	T	2.19E-04	PRS-CSx
Colorectum	PRS7	rs324546	9	9107265	G	A	8.58E-04	PRS-CSx
Colorectum	PRS7	rs325380	15	100256618	C	A	-4.66E-04	PRS-CSx
Colorectum	PRS7	rs325400	15	100252805	T	G	-6.27E-04	PRS-CSx
Colorectum	PRS7	rs326217	11	47303275	C	T	8.44E-05	PRS-CSx
Colorectum	PRS7	rs326222	11	47259668	C	T	1.38E-04	PRS-CSx
Colorectum	PRS7	rs326860	4	112753826	T	G	-5.31E-04	PRS-CSx
Colorectum	PRS7	rs327433	14	81272751	G	A	-7.34E-04	PRS-CSx
Colorectum	PRS7	rs328144	18	44174553	T	C	-9.05E-04	PRS-CSx
Colorectum	PRS7	rs328166	18	44157506	T	G	-1.21E-03	PRS-CSx
Colorectum	PRS7	rs328881	9	107799042	T	C	-8.23E-04	PRS-CSx
Colorectum	PRS7	rs328999	18	9530408	G	A	-2.75E-04	PRS-CSx
Colorectum	PRS7	rs329006	18	9522808	T	C	-1.94E-04	PRS-CSx
Colorectum	PRS7	rs329007	18	9522606	G	A	-3.62E-04	PRS-CSx
Colorectum	PRS7	rs329125	5	133871101	C	A	2.64E-04	PRS-CSx
Colorectum	PRS7	rs329304	5	133897829	T	C	2.10E-04	PRS-CSx
Colorectum	PRS7	rs33234	12	31030018	A	G	-1.13E-03	PRS-CSx
Colorectum	PRS7	rs333968	1	110460520	C	T	2.70E-03	PRS-CSx
Colorectum	PRS7	rs334733	1	61596658	T	C	-8.57E-04	PRS-CSx
Colorectum	PRS7	rs334734	1	61596298	T	C	-1.65E-03	PRS-CSx
Colorectum	PRS7	rs335581	1	214883726	C	T	-1.28E-04	PRS-CSx
Colorectum	PRS7	rs335681	15	76223268	G	A	-3.46E-04	PRS-CSx
Colorectum	PRS7	rs335697	15	76210367	T	C	-4.09E-04	PRS-CSx
Colorectum	PRS7	rs335711	15	76184740	T	C	-3.23E-04	PRS-CSx
Colorectum	PRS7	rs33914856	4	115485824	A	G	2.64E-04	PRS-CSx

Colorectum	PRS7	rs339758	13	72738783	C	T	4.57E-04	PRS-CSx
Colorectum	PRS7	rs339777	13	72766756	A	G	3.33E-04	PRS-CSx
Colorectum	PRS7	rs34103461	2	183316191	T	C	1.25E-04	PRS-CSx
Colorectum	PRS7	rs341127	6	158865657	A	G	2.54E-04	PRS-CSx
Colorectum	PRS7	rs34133302	13	28737639	G	A	-3.90E-04	PRS-CSx
Colorectum	PRS7	rs34180190	4	146376980	A	G	7.85E-04	PRS-CSx
Colorectum	PRS7	rs34260811	6	29715332	A	C	-8.81E-05	PRS-CSx
Colorectum	PRS7	rs34266	12	109595851	C	T	-3.16E-04	PRS-CSx
Colorectum	PRS7	rs342706	13	92417058	C	T	7.22E-04	PRS-CSx
Colorectum	PRS7	rs34281447	11	67028876	T	C	3.49E-04	PRS-CSx
Colorectum	PRS7	rs34309	5	67564383	A	G	5.12E-04	PRS-CSx
Colorectum	PRS7	rs34354043	1	38396205	G	A	1.17E-03	PRS-CSx
Colorectum	PRS7	rs34389364	1	1295323	G	A	1.51E-04	PRS-CSx
Colorectum	PRS7	rs344117	4	77619296	C	T	3.57E-04	PRS-CSx
Colorectum	PRS7	rs344561	19	6664054	G	A	-1.31E-03	PRS-CSx
Colorectum	PRS7	rs34599807	5	125968843	T	C	6.20E-04	PRS-CSx
Colorectum	PRS7	rs34655914	1	38397341	A	G	-9.86E-04	PRS-CSx
Colorectum	PRS7	rs346778	5	19432141	T	C	-7.91E-04	PRS-CSx
Colorectum	PRS7	rs346820	17	4935832	A	G	-5.33E-04	PRS-CSx
Colorectum	PRS7	rs346822	17	4937575	T	C	-8.60E-04	PRS-CSx
Colorectum	PRS7	rs34693	5	66288741	T	G	1.19E-03	PRS-CSx
Colorectum	PRS7	rs347413	13	42787040	A	G	2.94E-04	PRS-CSx
Colorectum	PRS7	rs34839124	5	172331057	T	C	4.50E-04	PRS-CSx
Colorectum	PRS7	rs34876581	1	1295403	T	C	3.63E-04	PRS-CSx
Colorectum	PRS7	rs35000319	1	84542492	A	C	4.25E-04	PRS-CSx
Colorectum	PRS7	rs350045	5	40238192	T	C	6.99E-04	PRS-CSx
Colorectum	PRS7	rs350047	5	40252294	T	C	7.34E-04	PRS-CSx
Colorectum	PRS7	rs350060	5	40213944	G	T	4.64E-04	PRS-CSx
Colorectum	PRS7	rs350067	5	40219261	G	A	-5.40E-04	PRS-CSx
Colorectum	PRS7	rs35066870	6	29942639	G	A	-1.81E-04	PRS-CSx
Colorectum	PRS7	rs35123	12	46281596	T	C	-1.82E-04	PRS-CSx
Colorectum	PRS7	rs35124	12	46279068	T	C	-4.86E-05	PRS-CSx
Colorectum	PRS7	rs351649	5	40217845	G	A	-9.00E-05	PRS-CSx
Colorectum	PRS7	rs35304796	6	56903750	A	G	-1.80E-03	PRS-CSx
Colorectum	PRS7	rs3531	6	27095313	C	T	-1.63E-04	PRS-CSx
Colorectum	PRS7	rs353159	4	68788694	G	A	1.74E-04	PRS-CSx
Colorectum	PRS7	rs353365	5	40228613	G	A	5.27E-04	PRS-CSx
Colorectum	PRS7	rs353372	5	40226919	T	C	6.21E-04	PRS-CSx
Colorectum	PRS7	rs353373	5	40225965	G	A	5.23E-04	PRS-CSx
Colorectum	PRS7	rs35494908	4	39254194	C	A	-2.04E-04	PRS-CSx
Colorectum	PRS7	rs35513983	1	2252759	G	A	-5.06E-04	PRS-CSx
Colorectum	PRS7	rs355527	20	6388068	T	C	2.06E-03	PRS-CSx
Colorectum	PRS7	rs35621308	12	65656484	G	A	1.07E-03	PRS-CSx
Colorectum	PRS7	rs356954	6	29983538	C	T	1.45E-04	PRS-CSx
Colorectum	PRS7	rs356968	6	29975047	T	G	1.10E-04	PRS-CSx
Colorectum	PRS7	rs35743786	2	176516959	G	A	-2.60E-04	PRS-CSx
Colorectum	PRS7	rs35756786	8	146245372	A	G	1.80E-04	PRS-CSx
Colorectum	PRS7	rs35840880	4	2594978	G	A	-1.01E-03	PRS-CSx
Colorectum	PRS7	rs358839	4	21565222	C	T	-8.12E-04	PRS-CSx
Colorectum	PRS7	rs35905748	8	117755248	A	G	-3.29E-04	PRS-CSx
Colorectum	PRS7	rs35924874	6	4941241	A	G	-8.93E-04	PRS-CSx
Colorectum	PRS7	rs35947998	1	223085331	G	A	4.98E-04	PRS-CSx
Colorectum	PRS7	rs36084352	1	38364396	C	T	-3.22E-05	PRS-CSx
Colorectum	PRS7	rs361844	22	31215815	G	A	-6.46E-04	PRS-CSx
Colorectum	PRS7	rs362340	14	73639138	G	A	3.98E-04	PRS-CSx
Colorectum	PRS7	rs362414	14	73581011	G	A	2.48E-04	PRS-CSx

Colorectum	PRS7	rs362439	14	73582301	T	C	2.82E-04	PRS-CSx
Colorectum	PRS7	rs362531	6	29530455	A	G	4.20E-04	PRS-CSx
Colorectum	PRS7	rs362532	6	29530413	G	A	3.51E-04	PRS-CSx
Colorectum	PRS7	rs36264	19	39861514	C	T	3.78E-04	PRS-CSx
Colorectum	PRS7	rs36270	19	39874945	C	T	3.19E-04	PRS-CSx
Colorectum	PRS7	rs363251	10	119019467	G	A	1.35E-03	PRS-CSx
Colorectum	PRS7	rs363800	15	48739082	A	C	5.15E-04	PRS-CSx
Colorectum	PRS7	rs363830	15	48720652	T	C	6.37E-04	PRS-CSx
Colorectum	PRS7	rs363836	15	48722884	G	A	5.86E-04	PRS-CSx
Colorectum	PRS7	rs365051	4	177391167	C	T	3.22E-04	PRS-CSx
Colorectum	PRS7	rs365053	6	32195988	G	T	-1.25E-04	PRS-CSx
Colorectum	PRS7	rs365525	13	23292562	C	T	-1.52E-03	PRS-CSx
Colorectum	PRS7	rs3656	6	32724043	A	G	-3.25E-04	PRS-CSx
Colorectum	PRS7	rs367910	13	109492416	T	C	5.79E-04	PRS-CSx
Colorectum	PRS7	rs369258	1	77042204	A	G	-4.17E-04	PRS-CSx
Colorectum	PRS7	rs370391	2	159963287	A	G	-5.19E-04	PRS-CSx
Colorectum	PRS7	rs3730168	20	57478939	A	G	-1.03E-03	PRS-CSx
Colorectum	PRS7	rs373038	20	6380637	A	G	1.47E-03	PRS-CSx
Colorectum	PRS7	rs3731217	9	21984661	C	A	2.12E-03	PRS-CSx
Colorectum	PRS7	rs3731239	9	21974218	G	A	-9.19E-06	PRS-CSx
Colorectum	PRS7	rs3731257	9	21966221	G	A	3.70E-04	PRS-CSx
Colorectum	PRS7	rs3731631	2	25359422	A	G	8.80E-04	PRS-CSx
Colorectum	PRS7	rs3732082	2	207041441	A	G	4.56E-04	PRS-CSx
Colorectum	PRS7	rs3732084	2	207174316	C	T	-3.43E-04	PRS-CSx
Colorectum	PRS7	rs3732234	2	71631296	C	T	-5.61E-04	PRS-CSx
Colorectum	PRS7	rs3732235	2	71631109	G	A	-4.57E-04	PRS-CSx
Colorectum	PRS7	rs3732361	3	119542297	G	A	7.00E-05	PRS-CSx
Colorectum	PRS7	rs3732413	3	119133183	G	A	-5.08E-04	PRS-CSx
Colorectum	PRS7	rs3732812	3	112863027	C	T	-6.99E-04	PRS-CSx
Colorectum	PRS7	rs3732832	3	122447574	C	A	-4.14E-04	PRS-CSx
Colorectum	PRS7	rs3733030	3	37170717	C	T	-4.97E-06	PRS-CSx
Colorectum	PRS7	rs3733280	4	39271541	G	A	-3.31E-05	PRS-CSx
Colorectum	PRS7	rs3733448	4	90036463	A	G	4.20E-04	PRS-CSx
Colorectum	PRS7	rs3734173	5	125939409	T	C	-5.64E-04	PRS-CSx
Colorectum	PRS7	rs373489	1	105057555	T	C	2.70E-04	PRS-CSx
Colorectum	PRS7	rs373499	11	33146139	G	A	-1.69E-04	PRS-CSx
Colorectum	PRS7	rs3735486	7	45105117	G	T	-3.04E-05	PRS-CSx
Colorectum	PRS7	rs3735494	7	45124286	G	A	-2.49E-04	PRS-CSx
Colorectum	PRS7	rs3736213	11	33183017	A	G	-1.52E-04	PRS-CSx
Colorectum	PRS7	rs3736491	15	68620177	A	G	9.10E-04	PRS-CSx
Colorectum	PRS7	rs3736694	6	29696849	C	A	3.46E-04	PRS-CSx
Colorectum	PRS7	rs3736762	20	33037336	T	C	-1.56E-04	PRS-CSx
Colorectum	PRS7	rs3737600	1	156956261	A	G	-4.41E-04	PRS-CSx
Colorectum	PRS7	rs373806	8	15648936	C	T	-6.31E-04	PRS-CSx
Colorectum	PRS7	rs3738441	1	92224067	C	T	2.45E-03	PRS-CSx
Colorectum	PRS7	rs3738829	1	183113862	C	T	1.45E-04	PRS-CSx
Colorectum	PRS7	rs3739690	9	33971740	A	G	-3.44E-04	PRS-CSx
Colorectum	PRS7	rs3739709	9	113637854	A	G	5.87E-04	PRS-CSx
Colorectum	PRS7	rs3739799	9	101784777	T	C	-9.02E-05	PRS-CSx
Colorectum	PRS7	rs3739942	9	139776400	C	T	-1.83E-04	PRS-CSx
Colorectum	PRS7	rs3740074	10	101571528	C	T	-1.82E-05	PRS-CSx
Colorectum	PRS7	rs3740078	10	101445591	C	A	2.28E-04	PRS-CSx
Colorectum	PRS7	rs3740169	10	16873547	A	G	3.39E-03	PRS-CSx
Colorectum	PRS7	rs3740297	10	48413273	T	C	-1.76E-03	PRS-CSx
Colorectum	PRS7	rs3740386	10	104931051	T	C	-3.65E-04	PRS-CSx
Colorectum	PRS7	rs3740390	10	104638480	T	C	1.43E-04	PRS-CSx

Colorectum	PRS7	rs3740690	11	47188592	C	T	1.82E-05	PRS-CSx
Colorectum	PRS7	rs3740995	11	5700340	T	C	9.78E-04	PRS-CSx
Colorectum	PRS7	rs3741122	11	74408740	T	C	1.10E-03	PRS-CSx
Colorectum	PRS7	rs3741127	11	74346933	G	A	-3.07E-04	PRS-CSx
Colorectum	PRS7	rs3741128	11	74203004	G	A	1.49E-05	PRS-CSx
Colorectum	PRS7	rs3741139	11	73611520	G	A	-4.41E-04	PRS-CSx
Colorectum	PRS7	rs3741378	11	65408937	T	C	2.66E-04	PRS-CSx
Colorectum	PRS7	rs3741764	12	116019203	A	G	-1.39E-04	PRS-CSx
Colorectum	PRS7	rs3741982	12	113328981	G	A	-3.98E-04	PRS-CSx
Colorectum	PRS7	rs3742001	12	112103148	C	T	1.66E-04	PRS-CSx
Colorectum	PRS7	rs3742003	12	111844218	G	A	3.49E-04	PRS-CSx
Colorectum	PRS7	rs3742035	12	110234658	T	C	5.14E-04	PRS-CSx
Colorectum	PRS7	rs3742062	12	51128832	G	A	3.78E-04	PRS-CSx
Colorectum	PRS7	rs3742269	13	47151963	A	G	1.35E-03	PRS-CSx
Colorectum	PRS7	rs3742553	14	81791046	A	G	-1.48E-03	PRS-CSx
Colorectum	PRS7	rs3742577	14	57672540	C	T	5.22E-04	PRS-CSx
Colorectum	PRS7	rs3742578	14	57672715	T	C	4.05E-04	PRS-CSx
Colorectum	PRS7	rs3742768	14	75759060	T	C	1.46E-03	PRS-CSx
Colorectum	PRS7	rs3743105	15	33023951	T	C	-2.32E-04	PRS-CSx
Colorectum	PRS7	rs3743180	15	100199430	G	A	-3.84E-04	PRS-CSx
Colorectum	PRS7	rs3743293	15	49325081	A	G	-1.15E-03	PRS-CSx
Colorectum	PRS7	rs3743406	15	91161548	A	G	7.72E-04	PRS-CSx
Colorectum	PRS7	rs3743613	16	75269534	C	T	-3.82E-04	PRS-CSx
Colorectum	PRS7	rs3744383	17	57290383	A	G	2.26E-04	PRS-CSx
Colorectum	PRS7	rs3744900	18	33779705	A	G	6.07E-04	PRS-CSx
Colorectum	PRS7	rs3745290	19	41890003	T	C	2.05E-04	PRS-CSx
Colorectum	PRS7	rs3746124	19	3632595	T	C	-6.60E-04	PRS-CSx
Colorectum	PRS7	rs3746125	19	3618928	A	G	-8.91E-04	PRS-CSx
Colorectum	PRS7	rs3746185	19	18474542	T	C	1.49E-03	PRS-CSx
Colorectum	PRS7	rs3746227	19	57804524	C	T	-3.98E-04	PRS-CSx
Colorectum	PRS7	rs3746228	19	57804362	A	G	-7.02E-04	PRS-CSx
Colorectum	PRS7	rs3746429	20	33703607	T	C	-1.86E-04	PRS-CSx
Colorectum	PRS7	rs3746446	20	33574765	C	T	-6.38E-04	PRS-CSx
Colorectum	PRS7	rs3746455	20	32957216	G	A	-2.44E-04	PRS-CSx
Colorectum	PRS7	rs3746804	20	744415	A	G	-4.81E-03	PRS-CSx
Colorectum	PRS7	rs3746807	20	745963	A	G	-7.41E-04	PRS-CSx
Colorectum	PRS7	rs3746820	20	47307618	A	G	-1.09E-03	PRS-CSx
Colorectum	PRS7	rs3747129	22	26862041	A	G	8.03E-04	PRS-CSx
Colorectum	PRS7	rs3747581	16	4462619	A	G	3.66E-04	PRS-CSx
Colorectum	PRS7	rs3747633	1	204415513	G	T	-2.25E-04	PRS-CSx
Colorectum	PRS7	rs3747634	1	204411063	C	T	-4.40E-04	PRS-CSx
Colorectum	PRS7	rs3747636	1	204403659	A	G	1.44E-04	PRS-CSx
Colorectum	PRS7	rs3747926	20	7863999	A	C	-1.03E-03	PRS-CSx
Colorectum	PRS7	rs3748543	1	61595989	C	T	-1.42E-03	PRS-CSx
Colorectum	PRS7	rs3748648	8	61851466	C	T	-1.28E-03	PRS-CSx
Colorectum	PRS7	rs3748682	1	38279987	C	T	-1.18E-04	PRS-CSx
Colorectum	PRS7	rs3748804	1	38340963	C	T	-9.87E-05	PRS-CSx
Colorectum	PRS7	rs3748816	1	2526746	A	G	-1.93E-04	PRS-CSx
Colorectum	PRS7	rs3749734	5	58121059	A	G	8.09E-04	PRS-CSx
Colorectum	PRS7	rs3749751	5	134509677	C	T	6.64E-04	PRS-CSx
Colorectum	PRS7	rs3749970	6	29342825	A	G	-1.71E-04	PRS-CSx
Colorectum	PRS7	rs3749971	6	29342775	A	G	3.23E-04	PRS-CSx
Colorectum	PRS7	rs3750035	7	127946173	C	T	-1.15E-03	PRS-CSx
Colorectum	PRS7	rs3750272	2	48461993	A	G	2.97E-03	PRS-CSx
Colorectum	PRS7	rs3750512	9	139821068	G	A	-8.17E-05	PRS-CSx
Colorectum	PRS7	rs3750835	10	123658618	T	C	1.51E-03	PRS-CSx

Colorectum	PRS7	rs3751603	15	52487466	A	G	-7.01E-04	PRS-CSx
Colorectum	PRS7	rs3751947	17	77914112	A	G	1.40E-03	PRS-CSx
Colorectum	PRS7	rs3754380	1	226832323	A	G	2.49E-04	PRS-CSx
Colorectum	PRS7	rs3754524	1	183153267	C	T	-4.05E-04	PRS-CSx
Colorectum	PRS7	rs3754929	2	183088584	G	A	3.28E-04	PRS-CSx
Colorectum	PRS7	rs3755155	2	216864594	T	C	4.83E-05	PRS-CSx
Colorectum	PRS7	rs3755520	2	95985231	G	A	-9.69E-05	PRS-CSx
Colorectum	PRS7	rs3755523	2	95974818	A	G	1.12E-05	PRS-CSx
Colorectum	PRS7	rs3755541	2	42995848	G	A	2.23E-04	PRS-CSx
Colorectum	PRS7	rs3756021	4	146461534	T	C	5.47E-04	PRS-CSx
Colorectum	PRS7	rs375670	2	183564175	C	A	7.08E-04	PRS-CSx
Colorectum	PRS7	rs3756746	5	128428851	T	C	-4.54E-04	PRS-CSx
Colorectum	PRS7	rs3757186	6	28107662	T	C	7.44E-04	PRS-CSx
Colorectum	PRS7	rs3757324	6	29689127	T	G	3.24E-04	PRS-CSx
Colorectum	PRS7	rs3757572	7	45146656	A	C	-8.10E-04	PRS-CSx
Colorectum	PRS7	rs3758141	8	18727949	G	A	6.93E-04	PRS-CSx
Colorectum	PRS7	rs3758673	11	47278917	C	T	2.68E-04	PRS-CSx
Colorectum	PRS7	rs3759322	12	6415994	T	C	-5.34E-03	PRS-CSx
Colorectum	PRS7	rs3759786	15	66629403	A	C	-3.11E-04	PRS-CSx
Colorectum	PRS7	rs3759900	15	72451827	C	A	-3.55E-04	PRS-CSx
Colorectum	PRS7	rs37602	16	75505046	T	C	6.76E-04	PRS-CSx
Colorectum	PRS7	rs3760490	17	4807926	A	G	9.58E-04	PRS-CSx
Colorectum	PRS7	rs3760685	19	8389011	A	C	1.77E-03	PRS-CSx
Colorectum	PRS7	rs3760746	19	6670529	G	A	-6.11E-04	PRS-CSx
Colorectum	PRS7	rs3760775	19	5841356	T	G	4.60E-03	PRS-CSx
Colorectum	PRS7	rs3761055	19	17835831	G	T	-6.65E-04	PRS-CSx
Colorectum	PRS7	rs3761847	9	123690239	G	A	-2.51E-04	PRS-CSx
Colorectum	PRS7	rs3763700	10	6181709	C	T	-3.51E-04	PRS-CSx
Colorectum	PRS7	rs3764	5	141551128	C	T	2.60E-04	PRS-CSx
Colorectum	PRS7	rs3764205	4	68750801	G	A	1.05E-04	PRS-CSx
Colorectum	PRS7	rs3764221	15	51588847	A	G	1.76E-04	PRS-CSx
Colorectum	PRS7	rs3764276	16	29673203	T	C	1.27E-03	PRS-CSx
Colorectum	PRS7	rs3764286	16	84741343	C	T	9.38E-04	PRS-CSx
Colorectum	PRS7	rs3764423	17	38643780	A	G	5.75E-04	PRS-CSx
Colorectum	PRS7	rs3764424	17	38645125	G	A	-9.46E-04	PRS-CSx
Colorectum	PRS7	rs3764626	19	14829749	C	T	-5.22E-04	PRS-CSx
Colorectum	PRS7	rs3764736	20	62668813	A	G	3.13E-03	PRS-CSx
Colorectum	PRS7	rs3764906	2	42588195	T	C	-5.67E-05	PRS-CSx
Colorectum	PRS7	rs3765334	16	831496	A	G	-8.08E-04	PRS-CSx
Colorectum	PRS7	rs3765863	1	210561713	A	C	3.86E-04	PRS-CSx
Colorectum	PRS7	rs3765871	1	210547213	A	G	3.09E-04	PRS-CSx
Colorectum	PRS7	rs3766160	1	15808872	G	A	-1.34E-04	PRS-CSx
Colorectum	PRS7	rs376699	2	183562648	C	A	1.12E-03	PRS-CSx
Colorectum	PRS7	rs3767139	1	22197336	C	T	3.01E-04	PRS-CSx
Colorectum	PRS7	rs3768371	1	226833608	A	G	6.94E-04	PRS-CSx
Colorectum	PRS7	rs3768617	1	183092500	C	T	9.62E-04	PRS-CSx
Colorectum	PRS7	rs3768622	1	183090011	G	A	2.03E-04	PRS-CSx
Colorectum	PRS7	rs3769393	2	169013590	C	T	-4.84E-04	PRS-CSx
Colorectum	PRS7	rs3769827	2	202102685	G	A	4.20E-04	PRS-CSx
Colorectum	PRS7	rs3772031	2	96014891	G	A	-2.10E-04	PRS-CSx
Colorectum	PRS7	rs3772032	2	96014132	G	A	-1.09E-04	PRS-CSx
Colorectum	PRS7	rs3772033	2	96013828	C	T	-1.80E-04	PRS-CSx
Colorectum	PRS7	rs3772041	2	95972707	G	A	-9.72E-06	PRS-CSx
Colorectum	PRS7	rs3772042	2	95970952	C	T	-1.03E-04	PRS-CSx
Colorectum	PRS7	rs3772044	2	95966046	T	G	-9.12E-05	PRS-CSx
Colorectum	PRS7	rs3772172	3	170077713	A	G	-5.32E-04	PRS-CSx

Colorectum	PRS7	rs3772173	3	170078232	C	T	-6.25E-04	PRS-CSx
Colorectum	PRS7	rs3772453	3	59923514	C	T	9.56E-04	PRS-CSx
Colorectum	PRS7	rs3772845	3	124560890	T	C	5.62E-04	PRS-CSx
Colorectum	PRS7	rs3773697	3	113023509	T	C	-3.02E-04	PRS-CSx
Colorectum	PRS7	rs3775228	4	87985166	T	C	3.44E-04	PRS-CSx
Colorectum	PRS7	rs3775311	4	166386944	T	C	1.40E-03	PRS-CSx
Colorectum	PRS7	rs3775373	4	89743821	G	T	-2.98E-04	PRS-CSx
Colorectum	PRS7	rs3775591	4	174222981	A	G	1.73E-03	PRS-CSx
Colorectum	PRS7	rs3776082	5	149544045	G	A	-1.16E-03	PRS-CSx
Colorectum	PRS7	rs3776107	5	140372001	T	C	3.11E-04	PRS-CSx
Colorectum	PRS7	rs3776110	5	140369390	G	A	4.46E-04	PRS-CSx
Colorectum	PRS7	rs3777411	6	160476945	T	C	8.74E-04	PRS-CSx
Colorectum	PRS7	rs3777438	6	131200102	A	C	1.71E-04	PRS-CSx
Colorectum	PRS7	rs3777464	6	131266757	G	A	6.79E-05	PRS-CSx
Colorectum	PRS7	rs3777980	6	117733973	T	G	2.38E-04	PRS-CSx
Colorectum	PRS7	rs3778007	6	76574145	C	T	-2.80E-04	PRS-CSx
Colorectum	PRS7	rs3778555	6	1917689	A	G	3.48E-04	PRS-CSx
Colorectum	PRS7	rs3779999	8	59744222	T	C	-8.40E-04	PRS-CSx
Colorectum	PRS7	rs378052	8	80764370	C	T	4.91E-04	PRS-CSx
Colorectum	PRS7	rs3780620	9	101799399	T	C	2.74E-04	PRS-CSx
Colorectum	PRS7	rs3780642	9	87553145	G	T	9.79E-05	PRS-CSx
Colorectum	PRS7	rs3781281	10	104852648	A	G	-1.76E-04	PRS-CSx
Colorectum	PRS7	rs3781282	10	104852419	G	A	-1.82E-04	PRS-CSx
Colorectum	PRS7	rs3781556	10	117997871	C	T	6.65E-04	PRS-CSx
Colorectum	PRS7	rs3781713	11	49048198	T	C	-1.94E-04	PRS-CSx
Colorectum	PRS7	rs3781926	11	72350595	C	T	3.13E-04	PRS-CSx
Colorectum	PRS7	rs3782091	11	65336483	G	T	-1.09E-04	PRS-CSx
Colorectum	PRS7	rs3782101	11	64131280	T	C	1.14E-04	PRS-CSx
Colorectum	PRS7	rs3782121	11	205935	A	G	-3.71E-03	PRS-CSx
Colorectum	PRS7	rs3782309	12	26859396	T	G	9.24E-04	PRS-CSx
Colorectum	PRS7	rs3782871	12	113315750	A	G	-2.37E-04	PRS-CSx
Colorectum	PRS7	rs3783273	14	51395083	T	C	6.80E-05	PRS-CSx
Colorectum	PRS7	rs3783274	14	51394991	C	T	1.38E-04	PRS-CSx
Colorectum	PRS7	rs3783694	14	58894577	C	T	8.95E-05	PRS-CSx
Colorectum	PRS7	rs3783712	14	65885275	C	T	-1.12E-04	PRS-CSx
Colorectum	PRS7	rs3784108	14	68789683	C	T	-2.38E-04	PRS-CSx
Colorectum	PRS7	rs3784240	14	105623261	A	G	5.50E-04	PRS-CSx
Colorectum	PRS7	rs3784384	15	71642403	C	A	3.17E-04	PRS-CSx
Colorectum	PRS7	rs3784921	16	11774201	T	G	-5.81E-04	PRS-CSx
Colorectum	PRS7	rs3785026	16	84136395	T	C	-2.18E-03	PRS-CSx
Colorectum	PRS7	rs3785079	16	69145476	A	C	2.27E-04	PRS-CSx
Colorectum	PRS7	rs3785133	16	68728611	A	G	3.35E-04	PRS-CSx
Colorectum	PRS7	rs3785898	17	40515120	A	C	4.19E-04	PRS-CSx
Colorectum	PRS7	rs3786941	19	4913071	A	G	1.66E-03	PRS-CSx
Colorectum	PRS7	rs3786949	19	39826479	A	G	6.32E-04	PRS-CSx
Colorectum	PRS7	rs3788169	21	46580779	T	C	-5.81E-04	PRS-CSx
Colorectum	PRS7	rs3788171	21	46583589	G	A	-7.41E-04	PRS-CSx
Colorectum	PRS7	rs3788594	22	43090249	A	C	4.39E-04	PRS-CSx
Colorectum	PRS7	rs3788601	22	43327526	C	T	2.30E-04	PRS-CSx
Colorectum	PRS7	rs3789336	20	13761657	T	C	4.97E-04	PRS-CSx
Colorectum	PRS7	rs3789994	12	79986655	A	G	3.16E-04	PRS-CSx
Colorectum	PRS7	rs3790254	20	19523056	A	G	5.33E-04	PRS-CSx
Colorectum	PRS7	rs3790626	1	38304912	T	C	-1.67E-04	PRS-CSx
Colorectum	PRS7	rs3790927	1	82339237	A	G	-2.48E-04	PRS-CSx
Colorectum	PRS7	rs3793772	10	102222825	C	T	-1.37E-04	PRS-CSx
Colorectum	PRS7	rs3794105	11	35200916	A	G	-1.83E-03	PRS-CSx

Colorectum	PRS7	rs3794552	15	52506217	A	G	-1.00E-03	PRS-CSx
Colorectum	PRS7	rs3794701	16	3730578	A	G	9.54E-04	PRS-CSx
Colorectum	PRS7	rs3795303	1	24388679	C	T	1.49E-03	PRS-CSx
Colorectum	PRS7	rs3795494	1	38271495	A	G	-2.58E-04	PRS-CSx
Colorectum	PRS7	rs3795996	2	172337974	A	G	-1.27E-03	PRS-CSx
Colorectum	PRS7	rs3796145	3	169524862	A	C	1.58E-03	PRS-CSx
Colorectum	PRS7	rs3796150	3	66502234	A	G	6.87E-04	PRS-CSx
Colorectum	PRS7	rs3796373	3	45960851	T	C	-4.46E-04	PRS-CSx
Colorectum	PRS7	rs3796517	4	39336953	G	A	2.51E-05	PRS-CSx
Colorectum	PRS7	rs3796906	4	7206643	T	C	-1.10E-03	PRS-CSx
Colorectum	PRS7	rs3798111	5	128438147	T	C	-2.67E-04	PRS-CSx
Colorectum	PRS7	rs3798251	6	131255106	A	G	6.97E-04	PRS-CSx
Colorectum	PRS7	rs3798397	6	117019607	G	A	-1.00E-04	PRS-CSx
Colorectum	PRS7	rs3798429	6	76604573	G	T	-4.78E-04	PRS-CSx
Colorectum	PRS7	rs3798465	6	73753109	C	T	-4.89E-04	PRS-CSx
Colorectum	PRS7	rs3800148	6	1947819	T	C	5.19E-04	PRS-CSx
Colorectum	PRS7	rs3800150	6	1955764	G	A	4.97E-04	PRS-CSx
Colorectum	PRS7	rs3800278	6	151765511	T	G	1.45E-03	PRS-CSx
Colorectum	PRS7	rs3800280	6	151766199	G	A	7.16E-04	PRS-CSx
Colorectum	PRS7	rs3800305	6	27185576	C	T	-5.03E-04	PRS-CSx
Colorectum	PRS7	rs3801081	7	47511161	A	G	-4.69E-03	PRS-CSx
Colorectum	PRS7	rs3801408	7	45201045	G	A	-4.21E-04	PRS-CSx
Colorectum	PRS7	rs380168	4	69608887	A	G	-3.44E-04	PRS-CSx
Colorectum	PRS7	rs3802333	9	19232135	T	C	2.52E-04	PRS-CSx
Colorectum	PRS7	rs3802758	11	45936035	A	G	4.78E-04	PRS-CSx
Colorectum	PRS7	rs3802840	11	111171646	T	G	1.85E-03	PRS-CSx
Colorectum	PRS7	rs3802842	11	111171709	C	A	1.69E-03	PRS-CSx
Colorectum	PRS7	rs3802936	11	64896237	A	G	-6.71E-04	PRS-CSx
Colorectum	PRS7	rs380303	4	177448126	C	T	-3.53E-04	PRS-CSx
Colorectum	PRS7	rs3803057	12	113368734	A	G	-3.39E-04	PRS-CSx
Colorectum	PRS7	rs3803059	12	113281488	A	G	-1.37E-04	PRS-CSx
Colorectum	PRS7	rs3803062	12	113196920	A	G	1.29E-04	PRS-CSx
Colorectum	PRS7	rs3803064	12	113173494	G	A	1.14E-04	PRS-CSx
Colorectum	PRS7	rs3803167	12	111785586	T	C	8.18E-05	PRS-CSx
Colorectum	PRS7	rs3803255	13	100201640	A	G	-2.26E-04	PRS-CSx
Colorectum	PRS7	rs3803533	15	90441937	T	C	-1.40E-03	PRS-CSx
Colorectum	PRS7	rs3803538	15	90891771	C	T	2.34E-04	PRS-CSx
Colorectum	PRS7	rs3803833	17	70018911	G	T	1.23E-03	PRS-CSx
Colorectum	PRS7	rs3803900	19	55605705	T	C	-9.26E-04	PRS-CSx
Colorectum	PRS7	rs3804124	6	25568942	C	T	-5.93E-04	PRS-CSx
Colorectum	PRS7	rs3804406	4	102166581	A	G	-4.97E-04	PRS-CSx
Colorectum	PRS7	rs3804408	4	102167216	G	A	-2.46E-04	PRS-CSx
Colorectum	PRS7	rs3804420	4	102181583	G	A	-2.86E-04	PRS-CSx
Colorectum	PRS7	rs3804547	6	4936863	A	G	-5.01E-04	PRS-CSx
Colorectum	PRS7	rs3808873	9	34590463	T	C	2.82E-04	PRS-CSx
Colorectum	PRS7	rs3809117	12	122630285	G	A	2.18E-04	PRS-CSx
Colorectum	PRS7	rs3809162	12	54674235	A	G	-4.81E-04	PRS-CSx
Colorectum	PRS7	rs3809272	12	111800258	A	G	8.04E-04	PRS-CSx
Colorectum	PRS7	rs3809276	12	112073916	A	G	1.60E-04	PRS-CSx
Colorectum	PRS7	rs3809288	12	111652522	G	A	2.47E-05	PRS-CSx
Colorectum	PRS7	rs3809290	12	111647453	C	T	2.66E-06	PRS-CSx
Colorectum	PRS7	rs3809470	14	105531945	G	A	-4.71E-04	PRS-CSx
Colorectum	PRS7	rs3809569	15	66999828	G	A	-3.26E-03	PRS-CSx
Colorectum	PRS7	rs380978	14	59357171	G	A	3.09E-04	PRS-CSx
Colorectum	PRS7	rs3809830	17	7257185	T	C	-1.07E-03	PRS-CSx
Colorectum	PRS7	rs3810174	19	41904165	T	C	2.96E-04	PRS-CSx

Colorectum	PRS7	rs3810175	19	41904396	G	A	1.10E-04	PRS-CSx
Colorectum	PRS7	rs3811519	2	48725610	T	C	-2.79E-04	PRS-CSx
Colorectum	PRS7	rs3811520	2	48667764	A	G	1.27E-04	PRS-CSx
Colorectum	PRS7	rs3811986	5	74324437	A	G	-2.07E-04	PRS-CSx
Colorectum	PRS7	rs3811987	5	74324548	A	G	-1.36E-04	PRS-CSx
Colorectum	PRS7	rs3812172	6	7218187	A	G	-1.01E-03	PRS-CSx
Colorectum	PRS7	rs3812726	11	15095651	A	G	2.11E-04	PRS-CSx
Colorectum	PRS7	rs3812866	13	28862066	G	A	-2.01E-04	PRS-CSx
Colorectum	PRS7	rs3812919	15	29414884	T	C	1.25E-03	PRS-CSx
Colorectum	PRS7	rs3812934	15	33008870	C	T	-4.80E-04	PRS-CSx
Colorectum	PRS7	rs3813145	19	14806162	C	T	-5.66E-04	PRS-CSx
Colorectum	PRS7	rs3814391	2	131812738	T	C	-3.74E-04	PRS-CSx
Colorectum	PRS7	rs3814392	2	131810267	G	T	-3.33E-04	PRS-CSx
Colorectum	PRS7	rs3814398	3	112991312	T	C	4.63E-04	PRS-CSx
Colorectum	PRS7	rs3814507	9	86323309	T	C	1.45E-03	PRS-CSx
Colorectum	PRS7	rs3814871	14	71444788	G	A	-3.01E-04	PRS-CSx
Colorectum	PRS7	rs3815953	14	81685482	A	G	-2.85E-04	PRS-CSx
Colorectum	PRS7	rs3816183	2	43015719	T	C	5.98E-04	PRS-CSx
Colorectum	PRS7	rs3816184	2	43015757	G	A	5.39E-04	PRS-CSx
Colorectum	PRS7	rs3816614	11	46890165	T	C	8.17E-05	PRS-CSx
Colorectum	PRS7	rs3817705	8	2054171	T	C	-3.88E-04	PRS-CSx
Colorectum	PRS7	rs3817711	8	2054814	G	A	-4.79E-04	PRS-CSx
Colorectum	PRS7	rs3818253	20	33596876	A	G	-5.77E-04	PRS-CSx
Colorectum	PRS7	rs3818493	13	95858686	G	A	-2.13E-04	PRS-CSx
Colorectum	PRS7	rs3818558	9	327782	T	C	-1.49E-04	PRS-CSx
Colorectum	PRS7	rs3819299	6	31322367	G	T	-1.08E-03	PRS-CSx
Colorectum	PRS7	rs3820071	1	15808767	G	A	-1.06E-04	PRS-CSx
Colorectum	PRS7	rs3820729	2	46392775	T	C	8.48E-04	PRS-CSx
Colorectum	PRS7	rs3820763	2	225347270	G	A	2.71E-04	PRS-CSx
Colorectum	PRS7	rs3821341	2	95995954	C	T	-1.01E-04	PRS-CSx
Colorectum	PRS7	rs3821688	3	122437834	G	A	-3.84E-04	PRS-CSx
Colorectum	PRS7	rs3823355	6	29942083	T	C	-1.38E-04	PRS-CSx
Colorectum	PRS7	rs3823358	6	29942205	A	G	-2.74E-04	PRS-CSx
Colorectum	PRS7	rs3823375	6	29944158	C	T	-1.36E-04	PRS-CSx
Colorectum	PRS7	rs3823624	7	2110346	C	T	-3.58E-04	PRS-CSx
Colorectum	PRS7	rs3823861	7	120446128	A	C	-1.74E-04	PRS-CSx
Colorectum	PRS7	rs3824106	8	29387385	T	C	-7.15E-04	PRS-CSx
Colorectum	PRS7	rs3824153	8	72754502	A	G	1.54E-03	PRS-CSx
Colorectum	PRS7	rs3824277	8	41347396	A	G	2.27E-03	PRS-CSx
Colorectum	PRS7	rs3824754	10	104614350	T	C	9.06E-05	PRS-CSx
Colorectum	PRS7	rs3824999	11	74345550	G	T	1.02E-03	PRS-CSx
Colorectum	PRS7	rs3825036	11	61516476	A	G	1.06E-04	PRS-CSx
Colorectum	PRS7	rs3825202	12	113281621	A	G	-2.04E-04	PRS-CSx
Colorectum	PRS7	rs3825398	12	111651949	G	A	1.52E-05	PRS-CSx
Colorectum	PRS7	rs3825541	14	51395205	C	T	1.96E-04	PRS-CSx
Colorectum	PRS7	rs3825666	14	89815144	C	A	-9.06E-04	PRS-CSx
Colorectum	PRS7	rs3826198	16	8906539	C	A	-6.75E-04	PRS-CSx
Colorectum	PRS7	rs3826248	16	71373048	G	A	-7.81E-04	PRS-CSx
Colorectum	PRS7	rs3827042	20	47319307	A	G	-6.59E-04	PRS-CSx
Colorectum	PRS7	rs3827505	6	25561914	C	T	-5.23E-04	PRS-CSx
Colorectum	PRS7	rs3828686	5	179751111	G	A	-1.68E-03	PRS-CSx
Colorectum	PRS7	rs3829916	10	3161216	G	A	-5.93E-04	PRS-CSx
Colorectum	PRS7	rs3830041	6	32191339	T	C	8.46E-04	PRS-CSx
Colorectum	PRS7	rs3830076	6	32096244	T	C	-7.77E-04	PRS-CSx
Colorectum	PRS7	rs3844577	17	5078965	G	A	6.91E-05	PRS-CSx
Colorectum	PRS7	rs3845293	1	1258246	A	C	4.33E-04	PRS-CSx

Colorectum	PRS7	rs3845498	1	38268397	C	T	-2.27E-04	PRS-CSx
Colorectum	PRS7	rs3845680	2	26099013	T	C	-1.59E-04	PRS-CSx
Colorectum	PRS7	rs3845905	3	66525963	T	C	8.09E-04	PRS-CSx
Colorectum	PRS7	rs3845906	3	66526602	G	A	4.90E-04	PRS-CSx
Colorectum	PRS7	rs3846046	3	112996316	A	G	3.86E-04	PRS-CSx
Colorectum	PRS7	rs3847137	8	128414498	C	T	2.03E-03	PRS-CSx
Colorectum	PRS7	rs3847595	11	33383085	A	G	-3.06E-04	PRS-CSx
Colorectum	PRS7	rs3847953	12	111765464	C	T	7.50E-05	PRS-CSx
Colorectum	PRS7	rs3849958	10	73431596	A	G	-6.84E-04	PRS-CSx
Colorectum	PRS7	rs3849959	10	73431617	C	T	-8.52E-04	PRS-CSx
Colorectum	PRS7	rs3851179	11	85868640	T	C	-3.14E-04	PRS-CSx
Colorectum	PRS7	rs3851222	6	111374253	G	A	-3.83E-04	PRS-CSx
Colorectum	PRS7	rs385132	11	33152074	T	C	-1.04E-04	PRS-CSx
Colorectum	PRS7	rs3852105	4	71422796	C	T	-1.10E-05	PRS-CSx
Colorectum	PRS7	rs3853166	3	168523282	A	G	-4.49E-04	PRS-CSx
Colorectum	PRS7	rs3853629	12	31760409	A	G	7.59E-04	PRS-CSx
Colorectum	PRS7	rs3856442	2	8728909	A	G	-8.80E-04	PRS-CSx
Colorectum	PRS7	rs3856718	3	112996218	A	G	4.09E-04	PRS-CSx
Colorectum	PRS7	rs3856720	3	112998090	C	T	3.29E-04	PRS-CSx
Colorectum	PRS7	rs3857093	4	155408243	T	C	-2.56E-04	PRS-CSx
Colorectum	PRS7	rs385771	5	79320677	C	T	8.40E-05	PRS-CSx
Colorectum	PRS7	rs3858652	12	116212682	C	A	-2.58E-04	PRS-CSx
Colorectum	PRS7	rs3859631	20	16150006	G	A	9.29E-04	PRS-CSx
Colorectum	PRS7	rs3861422	6	148174588	A	G	-1.03E-03	PRS-CSx
Colorectum	PRS7	rs3861449	6	148300251	T	G	3.28E-04	PRS-CSx
Colorectum	PRS7	rs3863085	3	165489550	T	C	-6.06E-04	PRS-CSx
Colorectum	PRS7	rs3863910	2	238326433	T	C	-9.82E-04	PRS-CSx
Colorectum	PRS7	rs3864028	3	1298121	C	T	3.23E-06	PRS-CSx
Colorectum	PRS7	rs3865515	19	39857418	G	A	8.57E-04	PRS-CSx
Colorectum	PRS7	rs3866900	5	129264789	T	G	-7.88E-05	PRS-CSx
Colorectum	PRS7	rs386736	3	156513408	A	C	-1.36E-03	PRS-CSx
Colorectum	PRS7	rs3873385	6	31269308	T	C	4.11E-04	PRS-CSx
Colorectum	PRS7	rs3884586	1	116136825	C	T	4.77E-04	PRS-CSx
Colorectum	PRS7	rs388707	21	30714776	C	T	-2.06E-04	PRS-CSx
Colorectum	PRS7	rs3887828	6	168788974	C	T	3.34E-04	PRS-CSx
Colorectum	PRS7	rs3887925	3	186665645	T	C	-5.45E-04	PRS-CSx
Colorectum	PRS7	rs3888654	18	3597864	G	A	4.58E-04	PRS-CSx
Colorectum	PRS7	rs388914	14	23873092	A	G	2.11E-04	PRS-CSx
Colorectum	PRS7	rs3889671	6	37064487	C	T	6.21E-04	PRS-CSx
Colorectum	PRS7	rs3890745	1	2553624	T	C	-9.62E-06	PRS-CSx
Colorectum	PRS7	rs3890774	3	133925806	T	C	-2.51E-04	PRS-CSx
Colorectum	PRS7	rs3891334	5	58536461	C	T	7.56E-04	PRS-CSx
Colorectum	PRS7	rs3895825	13	80596435	G	T	-4.17E-03	PRS-CSx
Colorectum	PRS7	rs389703	6	32199239	T	C	-3.41E-04	PRS-CSx
Colorectum	PRS7	rs389715	4	69616575	A	G	-3.75E-04	PRS-CSx
Colorectum	PRS7	rs3897991	10	8788259	A	G	5.00E-04	PRS-CSx
Colorectum	PRS7	rs3903160	6	29932897	A	G	-1.22E-04	PRS-CSx
Colorectum	PRS7	rs3904775	13	74076138	A	G	-3.25E-04	PRS-CSx
Colorectum	PRS7	rs390619	13	109492501	A	G	6.15E-04	PRS-CSx
Colorectum	PRS7	rs3906559	19	17325913	G	A	-6.33E-04	PRS-CSx
Colorectum	PRS7	rs3908218	3	165462643	G	A	-3.71E-04	PRS-CSx
Colorectum	PRS7	rs3908395	11	33383339	A	G	-3.28E-04	PRS-CSx
Colorectum	PRS7	rs3910312	6	30008746	C	A	-1.92E-04	PRS-CSx
Colorectum	PRS7	rs3911529	3	76940455	C	T	1.03E-03	PRS-CSx
Colorectum	PRS7	rs3912999	2	225390089	G	A	1.07E-04	PRS-CSx
Colorectum	PRS7	rs3915033	3	66548271	C	A	2.57E-03	PRS-CSx

Colorectum	PRS7	rs3917018	1	101202222	G	A	6.94E-04	PRS-CSx
Colorectum	PRS7	rs3919447	12	111391799	T	C	2.49E-04	PRS-CSx
Colorectum	PRS7	rs3920487	6	104452633	G	A	-9.71E-04	PRS-CSx
Colorectum	PRS7	rs3922	11	118765600	G	A	4.03E-04	PRS-CSx
Colorectum	PRS7	rs3922559	11	16008235	T	C	-5.06E-04	PRS-CSx
Colorectum	PRS7	rs3922904	13	31405422	T	C	-5.41E-04	PRS-CSx
Colorectum	PRS7	rs3923243	4	75579453	C	T	-8.16E-04	PRS-CSx
Colorectum	PRS7	rs3924223	1	222079194	A	G	4.17E-04	PRS-CSx
Colorectum	PRS7	rs3924549	18	36438591	G	T	3.86E-04	PRS-CSx
Colorectum	PRS7	rs3924909	1	221440464	C	A	6.88E-04	PRS-CSx
Colorectum	PRS7	rs3925857	9	33785963	G	A	-2.90E-04	PRS-CSx
Colorectum	PRS7	rs3928271	13	100186958	G	A	-7.47E-04	PRS-CSx
Colorectum	PRS7	rs3929339	11	46086727	A	G	2.28E-04	PRS-CSx
Colorectum	PRS7	rs3929561	3	5972451	A	G	-4.76E-04	PRS-CSx
Colorectum	PRS7	rs3930087	12	64402510	A	G	-1.48E-03	PRS-CSx
Colorectum	PRS7	rs3930345	5	82881255	T	C	-1.36E-03	PRS-CSx
Colorectum	PRS7	rs3930531	10	6143619	T	G	-4.43E-04	PRS-CSx
Colorectum	PRS7	rs3933785	9	15287389	G	A	-1.07E-03	PRS-CSx
Colorectum	PRS7	rs3934721	20	47243164	T	C	-7.06E-04	PRS-CSx
Colorectum	PRS7	rs3935123	17	70641183	G	A	1.46E-03	PRS-CSx
Colorectum	PRS7	rs393521	16	337678	G	T	-7.53E-04	PRS-CSx
Colorectum	PRS7	rs3935333	1	164951148	G	T	1.95E-04	PRS-CSx
Colorectum	PRS7	rs3935384	1	183028970	C	T	4.33E-04	PRS-CSx
Colorectum	PRS7	rs3936042	2	43107039	T	C	-4.55E-04	PRS-CSx
Colorectum	PRS7	rs3936193	20	47246127	A	G	-5.09E-04	PRS-CSx
Colorectum	PRS7	rs394128	16	337871	C	T	-5.02E-04	PRS-CSx
Colorectum	PRS7	rs3949907	6	40245483	C	T	6.64E-04	PRS-CSx
Colorectum	PRS7	rs3950165	6	56218183	T	C	-4.70E-04	PRS-CSx
Colorectum	PRS7	rs3957146	6	32681530	C	T	-1.21E-04	PRS-CSx
Colorectum	PRS7	rs3957148	6	32682137	G	A	-8.92E-06	PRS-CSx
Colorectum	PRS7	rs395936	1	61605207	C	T	-1.28E-03	PRS-CSx
Colorectum	PRS7	rs396518	11	33136661	C	T	-1.90E-04	PRS-CSx
Colorectum	PRS7	rs397435	16	2010138	G	A	-1.69E-03	PRS-CSx
Colorectum	PRS7	rs3977729	3	153273840	T	G	-1.34E-03	PRS-CSx
Colorectum	PRS7	rs398635	17	4946325	A	G	4.67E-04	PRS-CSx
Colorectum	PRS7	rs398854	18	54006782	C	T	-2.20E-04	PRS-CSx
Colorectum	PRS7	rs399246	19	30595364	T	C	-6.93E-04	PRS-CSx
Colorectum	PRS7	rs3995243	12	80098910	C	T	2.27E-04	PRS-CSx
Colorectum	PRS7	rs3995917	3	112852614	G	A	-3.69E-05	PRS-CSx
Colorectum	PRS7	rs3995932	3	112829792	A	G	-1.94E-03	PRS-CSx
Colorectum	PRS7	rs3998159	6	32682019	C	A	-1.64E-04	PRS-CSx
Colorectum	PRS7	rs400037	16	336396	A	G	-9.83E-04	PRS-CSx
Colorectum	PRS7	rs401618	6	29950210	G	A	4.93E-05	PRS-CSx
Colorectum	PRS7	rs4016435	3	41250129	T	G	1.40E-03	PRS-CSx
Colorectum	PRS7	rs4020801	5	88402973	A	G	2.98E-04	PRS-CSx
Colorectum	PRS7	rs4021409	14	77519874	T	G	-5.40E-04	PRS-CSx
Colorectum	PRS7	rs4021419	14	77519693	T	C	-2.65E-04	PRS-CSx
Colorectum	PRS7	rs403552	4	69613030	C	T	-3.84E-04	PRS-CSx
Colorectum	PRS7	rs40441	19	39878006	A	G	5.31E-04	PRS-CSx
Colorectum	PRS7	rs405698	22	22362353	C	T	-6.16E-04	PRS-CSx
Colorectum	PRS7	rs405875	6	32215188	T	C	-4.65E-05	PRS-CSx
Colorectum	PRS7	rs4060029	8	96442676	C	T	-1.36E-03	PRS-CSx
Colorectum	PRS7	rs406412	1	61605087	A	G	-6.48E-04	PRS-CSx
Colorectum	PRS7	rs407182	4	69606943	A	G	-5.27E-04	PRS-CSx
Colorectum	PRS7	rs407197	14	59367989	C	A	2.25E-04	PRS-CSx
Colorectum	PRS7	rs4072180	3	133868479	A	G	-4.46E-04	PRS-CSx

Colorectum	PRS7	rs4072739	17	17884660	G	A	-6.55E-04	PRS-CSx
Colorectum	PRS7	rs4072980	1	38456106	G	A	-8.06E-04	PRS-CSx
Colorectum	PRS7	rs4073089	16	80036594	T	G	-9.43E-04	PRS-CSx
Colorectum	PRS7	rs4074098	15	100795404	T	C	8.90E-04	PRS-CSx
Colorectum	PRS7	rs4074118	7	199163	G	A	4.75E-04	PRS-CSx
Colorectum	PRS7	rs4074119	7	198903	C	T	7.99E-04	PRS-CSx
Colorectum	PRS7	rs4074526	4	75591981	G	A	-7.87E-04	PRS-CSx
Colorectum	PRS7	rs4074794	11	409815	G	A	1.61E-04	PRS-CSx
Colorectum	PRS7	rs4075311	10	97574655	C	T	-8.48E-04	PRS-CSx
Colorectum	PRS7	rs4075522	15	75864335	A	G	1.43E-04	PRS-CSx
Colorectum	PRS7	rs4076292	20	47245761	A	G	-5.83E-04	PRS-CSx
Colorectum	PRS7	rs4076495	2	159768154	G	T	-1.91E-04	PRS-CSx
Colorectum	PRS7	rs4076557	11	46764649	C	T	3.16E-04	PRS-CSx
Colorectum	PRS7	rs4077140	15	82099365	C	T	-5.64E-04	PRS-CSx
Colorectum	PRS7	rs4077511	10	5578172	A	G	-6.57E-04	PRS-CSx
Colorectum	PRS7	rs4077537	11	15074612	A	G	1.57E-04	PRS-CSx
Colorectum	PRS7	rs4077788	8	65770668	A	C	-5.66E-04	PRS-CSx
Colorectum	PRS7	rs408307	9	136892523	C	T	-5.37E-04	PRS-CSx
Colorectum	PRS7	rs4083220	12	80095182	T	C	1.96E-04	PRS-CSx
Colorectum	PRS7	rs4084586	17	5048294	T	C	1.50E-04	PRS-CSx
Colorectum	PRS7	rs4094478	11	49029807	G	A	-3.36E-04	PRS-CSx
Colorectum	PRS7	rs4099470	11	65319986	T	C	-3.78E-03	PRS-CSx
Colorectum	PRS7	rs4105713	12	120453320	A	C	1.18E-04	PRS-CSx
Colorectum	PRS7	rs410876	9	136893867	C	A	-6.11E-04	PRS-CSx
Colorectum	PRS7	rs411326	6	32211317	T	C	-1.01E-04	PRS-CSx
Colorectum	PRS7	rs4118041	3	41345172	T	C	-1.66E-04	PRS-CSx
Colorectum	PRS7	rs4124581	4	77616748	G	A	1.98E-04	PRS-CSx
Colorectum	PRS7	rs4128264	9	109557941	A	G	-2.49E-04	PRS-CSx
Colorectum	PRS7	rs4129132	10	101284637	T	G	-4.73E-04	PRS-CSx
Colorectum	PRS7	rs4129601	11	42874002	G	A	2.48E-03	PRS-CSx
Colorectum	PRS7	rs4133088	2	229058072	A	G	-3.67E-04	PRS-CSx
Colorectum	PRS7	rs4133136	1	179376896	C	T	3.05E-04	PRS-CSx
Colorectum	PRS7	rs4134903	7	99711040	A	G	-1.30E-04	PRS-CSx
Colorectum	PRS7	rs41366748	1	239261802	A	G	-9.39E-04	PRS-CSx
Colorectum	PRS7	rs413693	2	71403616	G	T	-3.09E-04	PRS-CSx
Colorectum	PRS7	rs4140450	1	192845807	C	T	4.74E-04	PRS-CSx
Colorectum	PRS7	rs4140554	22	43355256	C	T	3.55E-05	PRS-CSx
Colorectum	PRS7	rs4140643	22	29408419	C	T	-5.77E-04	PRS-CSx
Colorectum	PRS7	rs4141153	7	41467946	G	A	1.04E-03	PRS-CSx
Colorectum	PRS7	rs4141252	12	113294547	T	C	-2.36E-04	PRS-CSx
Colorectum	PRS7	rs4141253	12	113325629	T	C	-4.22E-04	PRS-CSx
Colorectum	PRS7	rs4141428	2	208059440	T	C	-1.98E-04	PRS-CSx
Colorectum	PRS7	rs4141487	16	9689261	C	A	8.11E-04	PRS-CSx
Colorectum	PRS7	rs414237	3	193386538	C	T	-4.00E-04	PRS-CSx
Colorectum	PRS7	rs4142600	13	93990222	T	G	-4.43E-04	PRS-CSx
Colorectum	PRS7	rs4142640	11	117604207	A	G	6.44E-04	PRS-CSx
Colorectum	PRS7	rs414346	9	89765635	A	G	2.44E-04	PRS-CSx
Colorectum	PRS7	rs41434745	1	207031792	T	C	-4.13E-04	PRS-CSx
Colorectum	PRS7	rs4143772	1	220988346	A	G	1.25E-04	PRS-CSx
Colorectum	PRS7	rs4143890	14	54569027	A	C	7.85E-04	PRS-CSx
Colorectum	PRS7	rs4143998	14	103955247	T	C	3.83E-04	PRS-CSx
Colorectum	PRS7	rs4144156	14	98451900	T	C	-7.62E-04	PRS-CSx
Colorectum	PRS7	rs4144418	9	113506998	C	T	-4.40E-04	PRS-CSx
Colorectum	PRS7	rs41444546	3	188532083	A	G	4.84E-04	PRS-CSx
Colorectum	PRS7	rs4144617	11	94492207	A	G	4.22E-04	PRS-CSx
Colorectum	PRS7	rs41448945	1	239260094	G	T	-7.12E-04	PRS-CSx

Colorectum	PRS7	rs4145454	6	164572036	T	C	-8.36E-04	PRS-CSx
Colorectum	PRS7	rs4145953	11	74331879	G	A	-8.16E-04	PRS-CSx
Colorectum	PRS7	rs41463745	6	11264084	T	C	5.10E-04	PRS-CSx
Colorectum	PRS7	rs4146454	9	93857721	A	G	8.85E-04	PRS-CSx
Colorectum	PRS7	rs4146658	5	129372263	G	T	-1.50E-04	PRS-CSx
Colorectum	PRS7	rs4146707	1	164889498	T	C	4.55E-04	PRS-CSx
Colorectum	PRS7	rs4147100	4	175393169	G	A	-3.43E-04	PRS-CSx
Colorectum	PRS7	rs4147209	1	213793240	G	A	1.84E-03	PRS-CSx
Colorectum	PRS7	rs4147644	19	8386998	C	T	1.86E-03	PRS-CSx
Colorectum	PRS7	rs4147709	2	207023595	A	C	3.68E-04	PRS-CSx
Colorectum	PRS7	rs4147711	2	207023508	C	T	2.21E-04	PRS-CSx
Colorectum	PRS7	rs4147712	2	207018847	C	T	3.32E-04	PRS-CSx
Colorectum	PRS7	rs4148431	13	95913123	T	C	-6.78E-04	PRS-CSx
Colorectum	PRS7	rs4148477	13	95849817	C	T	4.38E-04	PRS-CSx
Colorectum	PRS7	rs4148478	13	95849680	G	A	6.02E-04	PRS-CSx
Colorectum	PRS7	rs4148479	13	95849374	T	C	5.79E-04	PRS-CSx
Colorectum	PRS7	rs4148850	7	150738624	G	A	-3.56E-04	PRS-CSx
Colorectum	PRS7	rs4148853	7	150742985	A	G	-6.68E-04	PRS-CSx
Colorectum	PRS7	rs4148854	7	150743820	A	G	1.68E-04	PRS-CSx
Colorectum	PRS7	rs4148928	10	73740613	A	G	-1.06E-04	PRS-CSx
Colorectum	PRS7	rs4148929	10	73740837	C	A	-2.19E-04	PRS-CSx
Colorectum	PRS7	rs4148933	10	73759890	C	T	-2.26E-04	PRS-CSx
Colorectum	PRS7	rs4148940	10	73765995	G	A	-1.70E-04	PRS-CSx
Colorectum	PRS7	rs4148946	10	73770073	T	C	-3.68E-04	PRS-CSx
Colorectum	PRS7	rs4148949	10	73770651	C	T	-3.16E-04	PRS-CSx
Colorectum	PRS7	rs4149014	12	21282953	G	T	9.53E-04	PRS-CSx
Colorectum	PRS7	rs4149018	12	21291561	G	T	1.63E-03	PRS-CSx
Colorectum	PRS7	rs4149437	2	109001623	C	T	-9.94E-04	PRS-CSx
Colorectum	PRS7	rs414947	17	30891261	A	G	4.21E-04	PRS-CSx
Colorectum	PRS7	rs415994	2	183558687	T	C	8.41E-04	PRS-CSx
Colorectum	PRS7	rs41621	7	120426790	A	C	-3.61E-04	PRS-CSx
Colorectum	PRS7	rs41622	7	120428607	G	A	-1.10E-04	PRS-CSx
Colorectum	PRS7	rs41623	7	120428799	C	A	-1.50E-04	PRS-CSx
Colorectum	PRS7	rs41624	7	120435638	T	C	-2.27E-04	PRS-CSx
Colorectum	PRS7	rs41629	7	120438889	A	C	-1.48E-04	PRS-CSx
Colorectum	PRS7	rs41632	7	120443185	G	A	-1.91E-04	PRS-CSx
Colorectum	PRS7	rs417968	17	43728376	G	A	-5.61E-04	PRS-CSx
Colorectum	PRS7	rs422249	11	61639488	T	C	-1.60E-05	PRS-CSx
Colorectum	PRS7	rs4233192	1	182970837	G	T	3.86E-04	PRS-CSx
Colorectum	PRS7	rs4233286	1	22688589	T	C	6.17E-04	PRS-CSx
Colorectum	PRS7	rs42333	7	120422982	T	C	-3.19E-04	PRS-CSx
Colorectum	PRS7	rs4233533	1	15829187	G	A	-5.36E-04	PRS-CSx
Colorectum	PRS7	rs4234699	3	129382013	G	A	2.51E-04	PRS-CSx
Colorectum	PRS7	rs4234700	3	129506482	A	G	9.16E-05	PRS-CSx
Colorectum	PRS7	rs4235288	4	18900588	C	T	1.85E-04	PRS-CSx
Colorectum	PRS7	rs423593	4	177388779	G	A	4.36E-04	PRS-CSx
Colorectum	PRS7	rs4236382	7	46889411	G	A	-9.42E-04	PRS-CSx
Colorectum	PRS7	rs4236383	7	46889491	C	T	6.21E-04	PRS-CSx
Colorectum	PRS7	rs423719	4	177392660	G	A	-7.80E-04	PRS-CSx
Colorectum	PRS7	rs4237353	10	20428197	A	G	2.48E-04	PRS-CSx
Colorectum	PRS7	rs4239055	17	5037724	T	C	1.28E-04	PRS-CSx
Colorectum	PRS7	rs4240411	5	129368084	A	G	-1.50E-04	PRS-CSx
Colorectum	PRS7	rs4240498	10	50300179	A	C	-4.05E-04	PRS-CSx
Colorectum	PRS7	rs4240546	1	116138871	T	C	8.90E-04	PRS-CSx
Colorectum	PRS7	rs4241367	3	133793121	T	C	1.33E-04	PRS-CSx
Colorectum	PRS7	rs4241370	3	133794199	T	G	1.89E-04	PRS-CSx

Colorectum	PRS7	rs4241931	4	140552120	T	C	-1.64E-03	PRS-CSx
Colorectum	PRS7	rs4242951	13	73990596	T	C	-8.74E-04	PRS-CSx
Colorectum	PRS7	rs4242952	13	73990613	C	T	-7.09E-04	PRS-CSx
Colorectum	PRS7	rs4242954	13	73990785	A	G	-1.42E-03	PRS-CSx
Colorectum	PRS7	rs4243191	16	79993227	T	C	1.24E-04	PRS-CSx
Colorectum	PRS7	rs4243192	16	79993792	T	C	3.54E-04	PRS-CSx
Colorectum	PRS7	rs4244811	11	65320780	G	A	-1.84E-04	PRS-CSx
Colorectum	PRS7	rs4244972	10	88502670	A	G	4.19E-05	PRS-CSx
Colorectum	PRS7	rs4246045	5	150177097	C	A	3.10E-04	PRS-CSx
Colorectum	PRS7	rs4246215	11	61564299	G	T	5.20E-04	PRS-CSx
Colorectum	PRS7	rs4246778	5	134519345	G	A	4.06E-04	PRS-CSx
Colorectum	PRS7	rs4248166	6	32366421	C	T	-3.21E-04	PRS-CSx
Colorectum	PRS7	rs425989	21	30715275	C	T	-1.56E-04	PRS-CSx
Colorectum	PRS7	rs4260948	9	107734804	T	C	3.51E-05	PRS-CSx
Colorectum	PRS7	rs4261575	16	76449988	T	C	2.73E-04	PRS-CSx
Colorectum	PRS7	rs4261913	3	119546197	T	C	1.47E-04	PRS-CSx
Colorectum	PRS7	rs4263531	5	129358549	G	A	-1.23E-04	PRS-CSx
Colorectum	PRS7	rs4264209	12	95190345	C	T	-3.25E-03	PRS-CSx
Colorectum	PRS7	rs4265516	10	50307461	T	C	-1.46E-03	PRS-CSx
Colorectum	PRS7	rs4267999	6	6381420	G	A	-7.49E-04	PRS-CSx
Colorectum	PRS7	rs4270639	4	6391735	G	A	9.98E-04	PRS-CSx
Colorectum	PRS7	rs4276132	3	24816974	A	C	-3.56E-04	PRS-CSx
Colorectum	PRS7	rs4276648	8	128427372	T	C	2.53E-03	PRS-CSx
Colorectum	PRS7	rs4277310	15	82087320	T	C	-3.65E-04	PRS-CSx
Colorectum	PRS7	rs427743	4	69614973	G	T	-3.08E-04	PRS-CSx
Colorectum	PRS7	rs4277599	20	33008905	T	C	-1.84E-04	PRS-CSx
Colorectum	PRS7	rs4277957	5	40699518	C	T	2.48E-04	PRS-CSx
Colorectum	PRS7	rs4278595	12	14290613	C	T	-4.76E-04	PRS-CSx
Colorectum	PRS7	rs4279094	3	119114693	G	A	3.32E-04	PRS-CSx
Colorectum	PRS7	rs4279480	6	32290927	G	T	-1.53E-04	PRS-CSx
Colorectum	PRS7	rs4279976	11	16059833	T	C	4.11E-04	PRS-CSx
Colorectum	PRS7	rs4283733	4	137613620	G	A	-8.47E-04	PRS-CSx
Colorectum	PRS7	rs428463	2	159964516	T	C	-1.21E-04	PRS-CSx
Colorectum	PRS7	rs4285273	5	40701432	C	A	2.09E-04	PRS-CSx
Colorectum	PRS7	rs4286073	15	77886864	A	G	-4.83E-04	PRS-CSx
Colorectum	PRS7	rs4286721	5	40497604	A	G	-7.03E-04	PRS-CSx
Colorectum	PRS7	rs4288253	7	66925725	G	T	5.08E-03	PRS-CSx
Colorectum	PRS7	rs4288933	14	105534009	A	G	-2.99E-04	PRS-CSx
Colorectum	PRS7	rs4290395	14	34958589	A	G	-5.87E-04	PRS-CSx
Colorectum	PRS7	rs429247	4	69615068	G	A	-5.07E-04	PRS-CSx
Colorectum	PRS7	rs4293602	2	48732944	G	A	-1.96E-03	PRS-CSx
Colorectum	PRS7	rs429439	11	33143126	G	A	-1.94E-04	PRS-CSx
Colorectum	PRS7	rs4294655	13	100216696	A	G	-7.56E-04	PRS-CSx
Colorectum	PRS7	rs4294664	13	104527071	A	G	1.13E-03	PRS-CSx
Colorectum	PRS7	rs429512	11	33157834	C	T	-2.41E-04	PRS-CSx
Colorectum	PRS7	rs4295329	4	115433101	A	C	2.02E-04	PRS-CSx
Colorectum	PRS7	rs4296472	2	48747079	A	G	-1.64E-03	PRS-CSx
Colorectum	PRS7	rs429761	5	38021593	G	A	6.16E-04	PRS-CSx
Colorectum	PRS7	rs4299117	15	75789444	T	C	4.84E-04	PRS-CSx
Colorectum	PRS7	rs4301004	3	133760908	C	A	-5.67E-04	PRS-CSx
Colorectum	PRS7	rs4301813	11	5372189	T	G	-8.34E-04	PRS-CSx
Colorectum	PRS7	rs430255	21	41496605	A	G	-9.52E-04	PRS-CSx
Colorectum	PRS7	rs4305500	4	153716517	A	C	-2.96E-04	PRS-CSx
Colorectum	PRS7	rs4307179	6	98484453	C	T	2.76E-04	PRS-CSx
Colorectum	PRS7	rs4308033	18	55334106	T	G	1.72E-03	PRS-CSx
Colorectum	PRS7	rs4310702	12	14291090	A	G	-6.22E-04	PRS-CSx

Colorectum	PRS7	rs431193	13	109495143	G	A	7.48E-04	PRS-CSx
Colorectum	PRS7	rs4313962	2	66331816	C	T	-4.43E-04	PRS-CSx
Colorectum	PRS7	rs4315958	5	129311522	G	A	-2.54E-04	PRS-CSx
Colorectum	PRS7	rs4316825	18	27864820	C	T	1.31E-03	PRS-CSx
Colorectum	PRS7	rs4317088	3	64601375	C	T	3.30E-04	PRS-CSx
Colorectum	PRS7	rs4317369	5	161228374	G	A	7.59E-04	PRS-CSx
Colorectum	PRS7	rs4317605	8	49194671	T	C	1.56E-03	PRS-CSx
Colorectum	PRS7	rs4319536	11	86303136	A	G	4.02E-04	PRS-CSx
Colorectum	PRS7	rs4321282	18	42091035	A	G	1.53E-04	PRS-CSx
Colorectum	PRS7	rs4321893	7	15463607	C	A	3.68E-05	PRS-CSx
Colorectum	PRS7	rs4322043	8	146235459	T	C	2.27E-04	PRS-CSx
Colorectum	PRS7	rs4325638	18	9450518	C	T	-7.92E-04	PRS-CSx
Colorectum	PRS7	rs4325917	3	133786717	A	G	-5.59E-04	PRS-CSx
Colorectum	PRS7	rs4326353	8	128790616	G	A	-1.11E-03	PRS-CSx
Colorectum	PRS7	rs4327389	3	133701960	A	C	1.34E-03	PRS-CSx
Colorectum	PRS7	rs4327688	6	23890877	C	T	-1.86E-04	PRS-CSx
Colorectum	PRS7	rs4329476	1	38361017	C	T	-1.25E-04	PRS-CSx
Colorectum	PRS7	rs4329999	18	72228269	A	G	2.39E-03	PRS-CSx
Colorectum	PRS7	rs4331375	17	70640082	C	T	1.08E-03	PRS-CSx
Colorectum	PRS7	rs4332645	13	78594589	T	C	-1.15E-03	PRS-CSx
Colorectum	PRS7	rs4333673	9	109610456	A	G	-1.95E-04	PRS-CSx
Colorectum	PRS7	rs4334845	5	6141688	A	G	1.59E-03	PRS-CSx
Colorectum	PRS7	rs4335623	12	20371195	C	T	5.48E-04	PRS-CSx
Colorectum	PRS7	rs4337859	5	33631739	C	A	-1.22E-03	PRS-CSx
Colorectum	PRS7	rs4338432	10	20458202	C	T	-1.28E-04	PRS-CSx
Colorectum	PRS7	rs4339373	5	164483072	G	T	1.46E-04	PRS-CSx
Colorectum	PRS7	rs4340393	18	41630054	C	T	3.07E-04	PRS-CSx
Colorectum	PRS7	rs4340697	3	64603654	T	C	-4.92E-04	PRS-CSx
Colorectum	PRS7	rs4342995	11	16021482	A	C	-7.31E-04	PRS-CSx
Colorectum	PRS7	rs4343599	3	134004200	G	A	-1.99E-04	PRS-CSx
Colorectum	PRS7	rs4345344	5	143775494	T	C	-5.09E-04	PRS-CSx
Colorectum	PRS7	rs4345357	5	134253500	T	C	2.63E-03	PRS-CSx
Colorectum	PRS7	rs4345787	1	164828928	C	T	8.89E-05	PRS-CSx
Colorectum	PRS7	rs4346787	5	164483794	G	A	1.58E-04	PRS-CSx
Colorectum	PRS7	rs4346936	7	96191823	G	A	1.09E-03	PRS-CSx
Colorectum	PRS7	rs4347021	8	49171144	T	G	6.72E-04	PRS-CSx
Colorectum	PRS7	rs4347190	1	164882733	T	G	5.32E-04	PRS-CSx
Colorectum	PRS7	rs434778	5	40726496	A	G	1.58E-04	PRS-CSx
Colorectum	PRS7	rs4348160	4	70017531	G	T	-3.20E-04	PRS-CSx
Colorectum	PRS7	rs4348357	6	32291359	T	G	-4.91E-05	PRS-CSx
Colorectum	PRS7	rs4350019	8	65564428	A	G	-3.97E-04	PRS-CSx
Colorectum	PRS7	rs4354124	6	169324377	C	T	8.68E-04	PRS-CSx
Colorectum	PRS7	rs4354226	7	46880743	C	T	6.44E-04	PRS-CSx
Colorectum	PRS7	rs4356634	2	176651117	T	C	3.66E-04	PRS-CSx
Colorectum	PRS7	rs4356814	3	64595816	A	G	2.77E-04	PRS-CSx
Colorectum	PRS7	rs4357512	1	117587250	A	G	-6.91E-04	PRS-CSx
Colorectum	PRS7	rs435986	9	107809777	G	T	-5.27E-04	PRS-CSx
Colorectum	PRS7	rs4361227	3	24834378	C	T	-9.42E-04	PRS-CSx
Colorectum	PRS7	rs4361998	1	12785093	T	G	2.95E-04	PRS-CSx
Colorectum	PRS7	rs4364199	3	103995187	C	T	8.06E-04	PRS-CSx
Colorectum	PRS7	rs4365278	16	85238018	C	T	7.11E-04	PRS-CSx
Colorectum	PRS7	rs4365299	16	80069915	G	A	3.69E-04	PRS-CSx
Colorectum	PRS7	rs4367844	10	28244079	T	C	7.78E-04	PRS-CSx
Colorectum	PRS7	rs4368798	6	26593471	A	G	-2.32E-04	PRS-CSx
Colorectum	PRS7	rs4371401	2	48727280	C	T	3.42E-04	PRS-CSx
Colorectum	PRS7	rs4371897	7	46880341	C	T	-8.14E-04	PRS-CSx

Colorectum	PRS7	rs4373519	8	22933011	A	G	1.34E-03	PRS-CSx
Colorectum	PRS7	rs4374781	5	143506829	G	T	-3.00E-04	PRS-CSx
Colorectum	PRS7	rs4375897	2	777138	A	G	-1.87E-03	PRS-CSx
Colorectum	PRS7	rs4376143	4	105794365	A	G	6.10E-04	PRS-CSx
Colorectum	PRS7	rs4377507	3	173500259	A	G	3.09E-04	PRS-CSx
Colorectum	PRS7	rs4379286	6	12329292	T	C	-4.42E-04	PRS-CSx
Colorectum	PRS7	rs4379863	11	133660717	T	C	2.07E-03	PRS-CSx
Colorectum	PRS7	rs4381380	11	120273316	A	G	-1.05E-04	PRS-CSx
Colorectum	PRS7	rs4382820	10	73949708	T	C	-5.14E-04	PRS-CSx
Colorectum	PRS7	rs4383619	4	7863508	T	C	7.23E-04	PRS-CSx
Colorectum	PRS7	rs4385334	6	148215688	A	G	5.05E-04	PRS-CSx
Colorectum	PRS7	rs4388862	11	33310265	T	C	4.03E-04	PRS-CSx
Colorectum	PRS7	rs4389703	5	31283420	C	T	4.15E-04	PRS-CSx
Colorectum	PRS7	rs4392090	16	80057311	A	G	6.92E-04	PRS-CSx
Colorectum	PRS7	rs4392177	19	1684137	A	G	1.13E-03	PRS-CSx
Colorectum	PRS7	rs4392402	3	16891568	C	T	-9.28E-04	PRS-CSx
Colorectum	PRS7	rs4394270	6	32709964	C	T	1.96E-03	PRS-CSx
Colorectum	PRS7	rs4394609	1	22732518	C	T	-7.43E-04	PRS-CSx
Colorectum	PRS7	rs4394668	1	12671229	C	T	8.22E-04	PRS-CSx
Colorectum	PRS7	rs4394802	11	46990587	T	C	3.36E-05	PRS-CSx
Colorectum	PRS7	rs4395860	8	128788985	A	G	-1.12E-03	PRS-CSx
Colorectum	PRS7	rs439735	14	23868285	A	G	1.16E-04	PRS-CSx
Colorectum	PRS7	rs4399321	11	10323478	G	A	2.99E-04	PRS-CSx
Colorectum	PRS7	rs4399344	11	125134725	A	C	6.09E-04	PRS-CSx
Colorectum	PRS7	rs4400278	7	15474008	G	A	4.67E-05	PRS-CSx
Colorectum	PRS7	rs4401453	4	99774183	T	C	7.03E-04	PRS-CSx
Colorectum	PRS7	rs4402582	16	80063069	A	G	3.95E-04	PRS-CSx
Colorectum	PRS7	rs440261	6	32198056	G	A	-9.93E-05	PRS-CSx
Colorectum	PRS7	rs4404993	9	19367826	T	G	3.51E-04	PRS-CSx
Colorectum	PRS7	rs4405492	15	52515678	T	G	1.20E-03	PRS-CSx
Colorectum	PRS7	rs4406765	10	48545527	C	T	8.17E-04	PRS-CSx
Colorectum	PRS7	rs4407237	2	66522294	T	C	-9.11E-04	PRS-CSx
Colorectum	PRS7	rs4408942	4	153716718	A	G	-2.55E-04	PRS-CSx
Colorectum	PRS7	rs4409621	1	22732749	A	G	-2.49E-04	PRS-CSx
Colorectum	PRS7	rs4409764	10	101284237	T	G	-2.63E-04	PRS-CSx
Colorectum	PRS7	rs4409766	10	104616663	C	T	5.45E-04	PRS-CSx
Colorectum	PRS7	rs4409950	13	109954478	G	A	-2.02E-03	PRS-CSx
Colorectum	PRS7	rs4412203	6	53036999	C	T	-5.10E-05	PRS-CSx
Colorectum	PRS7	rs441233	9	136876021	G	A	-8.55E-05	PRS-CSx
Colorectum	PRS7	rs4413115	2	160002919	C	T	1.62E-03	PRS-CSx
Colorectum	PRS7	rs441663	11	33139419	G	A	-1.88E-04	PRS-CSx
Colorectum	PRS7	rs4419188	2	192960408	C	T	-1.74E-04	PRS-CSx
Colorectum	PRS7	rs4420319	12	63873009	A	G	1.02E-03	PRS-CSx
Colorectum	PRS7	rs4420479	15	66806517	G	A	-3.46E-04	PRS-CSx
Colorectum	PRS7	rs4420529	16	1931056	C	T	1.13E-04	PRS-CSx
Colorectum	PRS7	rs4422154	2	216725663	T	C	1.48E-03	PRS-CSx
Colorectum	PRS7	rs4422543	5	31283014	G	A	2.84E-04	PRS-CSx
Colorectum	PRS7	rs4422969	1	183020762	G	A	4.16E-04	PRS-CSx
Colorectum	PRS7	rs4423707	3	39314574	T	C	-9.10E-04	PRS-CSx
Colorectum	PRS7	rs4424195	7	99642745	G	A	-2.96E-04	PRS-CSx
Colorectum	PRS7	rs4426363	16	80038459	T	C	4.97E-04	PRS-CSx
Colorectum	PRS7	rs4426678	3	133807007	T	C	-1.74E-04	PRS-CSx
Colorectum	PRS7	rs4426996	6	43887937	A	G	-1.33E-03	PRS-CSx
Colorectum	PRS7	rs4428009	2	229077527	T	C	-3.30E-04	PRS-CSx
Colorectum	PRS7	rs4429520	2	48709988	G	A	2.03E-04	PRS-CSx
Colorectum	PRS7	rs4430760	16	80004773	A	C	-2.74E-04	PRS-CSx

Colorectum	PRS7	rs4430989	2	48717807	G	A	2.41E-04	PRS-CSx
Colorectum	PRS7	rs443198	6	32190406	A	G	-4.58E-04	PRS-CSx
Colorectum	PRS7	rs4432098	12	14109900	T	C	-3.39E-04	PRS-CSx
Colorectum	PRS7	rs443298	14	59299372	A	C	2.43E-04	PRS-CSx
Colorectum	PRS7	rs4433894	18	73299956	C	A	6.22E-04	PRS-CSx
Colorectum	PRS7	rs4434042	2	48666736	G	A	-1.81E-03	PRS-CSx
Colorectum	PRS7	rs4434248	4	18905576	G	A	2.20E-04	PRS-CSx
Colorectum	PRS7	rs4436834	17	4769418	T	C	5.43E-04	PRS-CSx
Colorectum	PRS7	rs4437460	6	26689223	G	A	-1.12E-03	PRS-CSx
Colorectum	PRS7	rs4439449	10	114292303	A	G	2.74E-03	PRS-CSx
Colorectum	PRS7	rs4439776	16	80038715	A	G	-7.87E-04	PRS-CSx
Colorectum	PRS7	rs4441080	12	80030707	C	T	3.17E-04	PRS-CSx
Colorectum	PRS7	rs4443044	2	48722755	C	T	-1.42E-04	PRS-CSx
Colorectum	PRS7	rs4444073	11	10331664	C	A	4.01E-04	PRS-CSx
Colorectum	PRS7	rs4444235	14	54410919	C	T	5.54E-03	PRS-CSx
Colorectum	PRS7	rs4445223	8	83827104	A	C	-1.70E-04	PRS-CSx
Colorectum	PRS7	rs4445406	1	2539400	T	C	-1.05E-04	PRS-CSx
Colorectum	PRS7	rs4446742	8	83827544	A	G	-3.53E-04	PRS-CSx
Colorectum	PRS7	rs4447376	15	96126048	T	G	-5.43E-04	PRS-CSx
Colorectum	PRS7	rs4447442	16	78344445	C	T	6.38E-04	PRS-CSx
Colorectum	PRS7	rs4447844	4	120696623	A	G	-2.34E-04	PRS-CSx
Colorectum	PRS7	rs4449015	17	75741550	T	C	4.13E-04	PRS-CSx
Colorectum	PRS7	rs4449471	4	58475491	T	G	3.77E-04	PRS-CSx
Colorectum	PRS7	rs445074	20	6378809	A	G	1.91E-03	PRS-CSx
Colorectum	PRS7	rs4450812	3	133824778	C	T	-1.43E-04	PRS-CSx
Colorectum	PRS7	rs4450813	3	133874113	C	T	-1.24E-04	PRS-CSx
Colorectum	PRS7	rs4451904	15	94114235	C	T	-1.44E-03	PRS-CSx
Colorectum	PRS7	rs4451914	15	75739824	T	C	4.44E-05	PRS-CSx
Colorectum	PRS7	rs4451977	16	73183339	A	G	1.13E-03	PRS-CSx
Colorectum	PRS7	rs445218	20	6378912	A	G	1.18E-03	PRS-CSx
Colorectum	PRS7	rs4452822	8	65538103	A	G	1.55E-04	PRS-CSx
Colorectum	PRS7	rs4454510	1	182982303	G	A	5.67E-04	PRS-CSx
Colorectum	PRS7	rs4454616	10	8665147	G	A	-8.05E-05	PRS-CSx
Colorectum	PRS7	rs4454916	15	67063830	T	G	-1.36E-03	PRS-CSx
Colorectum	PRS7	rs4455220	20	47335017	G	A	-5.62E-04	PRS-CSx
Colorectum	PRS7	rs4456339	12	4359472	A	G	2.41E-04	PRS-CSx
Colorectum	PRS7	rs4457334	8	83829947	A	G	-1.52E-04	PRS-CSx
Colorectum	PRS7	rs4457591	1	222078600	C	T	2.83E-04	PRS-CSx
Colorectum	PRS7	rs4457720	11	106491841	A	G	-1.15E-03	PRS-CSx
Colorectum	PRS7	rs4459	22	44974493	T	C	6.93E-04	PRS-CSx
Colorectum	PRS7	rs4461087	16	79991016	T	G	4.94E-04	PRS-CSx
Colorectum	PRS7	rs4461899	8	83861575	G	A	-2.11E-04	PRS-CSx
Colorectum	PRS7	rs4464333	2	48673328	A	G	2.42E-04	PRS-CSx
Colorectum	PRS7	rs4465618	16	80057372	G	A	-1.32E-04	PRS-CSx
Colorectum	PRS7	rs4465756	2	208080291	C	T	-2.32E-04	PRS-CSx
Colorectum	PRS7	rs4465931	3	3352713	C	T	-5.56E-04	PRS-CSx
Colorectum	PRS7	rs4468024	9	19294073	T	C	7.83E-04	PRS-CSx
Colorectum	PRS7	rs4470778	5	178147408	A	G	6.70E-04	PRS-CSx
Colorectum	PRS7	rs447106	2	159900278	G	A	2.29E-04	PRS-CSx
Colorectum	PRS7	rs4471853	2	188684507	G	A	3.03E-04	PRS-CSx
Colorectum	PRS7	rs4473655	4	7847468	A	C	6.09E-04	PRS-CSx
Colorectum	PRS7	rs4474198	1	2553758	C	T	-2.20E-04	PRS-CSx
Colorectum	PRS7	rs4476839	6	98342192	G	A	3.12E-04	PRS-CSx
Colorectum	PRS7	rs4477273	1	116128030	A	C	1.00E-03	PRS-CSx
Colorectum	PRS7	rs4477507	12	4373928	T	G	3.00E-03	PRS-CSx
Colorectum	PRS7	rs4477582	13	57712738	G	A	7.23E-04	PRS-CSx

Colorectum	PRS7	rs4478958	11	16028675	T	C	5.05E-04	PRS-CSx
Colorectum	PRS7	rs4480567	11	83341015	C	A	1.13E-04	PRS-CSx
Colorectum	PRS7	rs4481145	3	1175575	A	G	-9.31E-04	PRS-CSx
Colorectum	PRS7	rs4481363	5	164474719	C	A	2.42E-04	PRS-CSx
Colorectum	PRS7	rs4481859	1	222732656	G	T	-1.81E-04	PRS-CSx
Colorectum	PRS7	rs4484269	4	175347444	T	C	-5.15E-04	PRS-CSx
Colorectum	PRS7	rs4484700	8	14429347	C	T	-1.08E-03	PRS-CSx
Colorectum	PRS7	rs4484929	1	221313677	G	A	1.76E-04	PRS-CSx
Colorectum	PRS7	rs448513	2	159964552	T	C	-3.93E-04	PRS-CSx
Colorectum	PRS7	rs4487145	20	47249116	A	G	-6.43E-04	PRS-CSx
Colorectum	PRS7	rs4489034	5	100915706	A	G	6.03E-04	PRS-CSx
Colorectum	PRS7	rs4489283	8	32399662	T	C	-8.24E-04	PRS-CSx
Colorectum	PRS7	rs4490014	16	80035732	C	A	-1.26E-03	PRS-CSx
Colorectum	PRS7	rs4490144	2	183480546	A	G	2.46E-04	PRS-CSx
Colorectum	PRS7	rs4491984	4	89140356	C	T	3.22E-03	PRS-CSx
Colorectum	PRS7	rs449250	1	6797684	G	A	7.93E-05	PRS-CSx
Colorectum	PRS7	rs4494199	1	201884668	A	G	2.10E-03	PRS-CSx
Colorectum	PRS7	rs4494268	11	47038220	G	A	2.74E-04	PRS-CSx
Colorectum	PRS7	rs4496283	2	238197739	C	A	-8.24E-04	PRS-CSx
Colorectum	PRS7	rs4496874	7	46880728	T	C	-5.29E-04	PRS-CSx
Colorectum	PRS7	rs4497915	2	48690596	G	T	-8.42E-04	PRS-CSx
Colorectum	PRS7	rs4498989	11	4740665	G	A	-1.19E-04	PRS-CSx
Colorectum	PRS7	rs4499283	17	69598898	G	A	1.32E-04	PRS-CSx
Colorectum	PRS7	rs450055	20	49036751	A	G	-2.75E-04	PRS-CSx
Colorectum	PRS7	rs4500718	16	68754312	C	T	-8.97E-04	PRS-CSx
Colorectum	PRS7	rs4501195	4	46589847	G	A	-4.66E-04	PRS-CSx
Colorectum	PRS7	rs4501567	8	60568606	A	G	3.69E-04	PRS-CSx
Colorectum	PRS7	rs4502996	7	67002797	C	T	5.51E-04	PRS-CSx
Colorectum	PRS7	rs4505341	16	80034203	A	C	-9.21E-04	PRS-CSx
Colorectum	PRS7	rs4506781	13	71728125	A	G	5.10E-04	PRS-CSx
Colorectum	PRS7	rs450808	21	43706944	T	C	1.43E-03	PRS-CSx
Colorectum	PRS7	rs4508106	10	8709338	T	G	3.66E-04	PRS-CSx
Colorectum	PRS7	rs4509829	12	111396613	T	C	3.13E-04	PRS-CSx
Colorectum	PRS7	rs4512035	4	43042976	G	A	9.32E-04	PRS-CSx
Colorectum	PRS7	rs4513726	5	143504102	A	G	-1.76E-04	PRS-CSx
Colorectum	PRS7	rs4513821	6	131242012	T	C	5.66E-04	PRS-CSx
Colorectum	PRS7	rs4515996	11	69919522	C	A	6.72E-04	PRS-CSx
Colorectum	PRS7	rs4516135	14	51502070	A	G	7.22E-04	PRS-CSx
Colorectum	PRS7	rs4517148	8	49194601	G	T	1.71E-03	PRS-CSx
Colorectum	PRS7	rs4517412	10	70063536	A	G	-1.99E-04	PRS-CSx
Colorectum	PRS7	rs4517640	13	111075916	T	C	-4.32E-05	PRS-CSx
Colorectum	PRS7	rs4518485	6	54979492	G	A	-1.80E-03	PRS-CSx
Colorectum	PRS7	rs4520200	8	65769571	T	G	3.87E-04	PRS-CSx
Colorectum	PRS7	rs4526973	15	72138719	A	G	-5.18E-04	PRS-CSx
Colorectum	PRS7	rs4528911	3	112851081	C	T	-6.62E-04	PRS-CSx
Colorectum	PRS7	rs4528941	3	133760537	G	A	7.46E-04	PRS-CSx
Colorectum	PRS7	rs4529997	13	32689584	C	T	1.34E-03	PRS-CSx
Colorectum	PRS7	rs453114	20	6379635	T	G	6.72E-04	PRS-CSx
Colorectum	PRS7	rs4531729	16	56714520	C	T	-6.88E-04	PRS-CSx
Colorectum	PRS7	rs4531770	17	69653253	T	G	4.54E-04	PRS-CSx
Colorectum	PRS7	rs4532391	5	117063252	G	T	-7.45E-04	PRS-CSx
Colorectum	PRS7	rs4532949	10	73642902	C	T	9.32E-04	PRS-CSx
Colorectum	PRS7	rs4533197	14	75762198	T	C	2.32E-03	PRS-CSx
Colorectum	PRS7	rs4533923	5	129336943	G	A	-1.97E-04	PRS-CSx
Colorectum	PRS7	rs4534647	12	112554962	C	T	1.75E-03	PRS-CSx
Colorectum	PRS7	rs4535199	3	134013144	C	A	6.14E-05	PRS-CSx

Colorectum	PRS7	rs4535222	3	133809608	C	A	-2.87E-04	PRS-CSx
Colorectum	PRS7	rs4537971	15	71701660	G	T	3.26E-04	PRS-CSx
Colorectum	PRS7	rs4539328	11	120218243	C	T	-2.30E-04	PRS-CSx
Colorectum	PRS7	rs4541741	6	41664032	A	C	-1.43E-03	PRS-CSx
Colorectum	PRS7	rs4543091	4	148025559	T	C	-4.09E-04	PRS-CSx
Colorectum	PRS7	rs4543289	5	164484948	T	G	-1.03E-05	PRS-CSx
Colorectum	PRS7	rs4543420	6	6384148	C	T	-6.08E-04	PRS-CSx
Colorectum	PRS7	rs4544355	19	4914117	C	T	1.45E-03	PRS-CSx
Colorectum	PRS7	rs4545520	11	15999644	T	C	-1.66E-03	PRS-CSx
Colorectum	PRS7	rs4547215	13	111056155	G	A	6.11E-05	PRS-CSx
Colorectum	PRS7	rs454748	6	32213210	G	A	-1.83E-04	PRS-CSx
Colorectum	PRS7	rs4552343	3	133842112	C	T	-1.90E-04	PRS-CSx
Colorectum	PRS7	rs4552460	4	7863618	A	G	4.50E-04	PRS-CSx
Colorectum	PRS7	rs4552931	8	49171227	G	A	1.07E-03	PRS-CSx
Colorectum	PRS7	rs455328	21	40230194	A	G	1.45E-04	PRS-CSx
Colorectum	PRS7	rs4554200	5	150249537	A	G	2.53E-04	PRS-CSx
Colorectum	PRS7	rs4554346	6	122918372	A	G	2.07E-03	PRS-CSx
Colorectum	PRS7	rs4554462	8	61854841	G	A	-8.81E-04	PRS-CSx
Colorectum	PRS7	rs4555392	2	43103614	A	C	-2.38E-04	PRS-CSx
Colorectum	PRS7	rs4556852	17	54954666	G	A	-1.56E-03	PRS-CSx
Colorectum	PRS7	rs4558089	10	50309330	C	T	-6.62E-04	PRS-CSx
Colorectum	PRS7	rs4559959	17	21062061	G	A	-8.38E-04	PRS-CSx
Colorectum	PRS7	rs4560628	6	22451820	A	G	-7.76E-04	PRS-CSx
Colorectum	PRS7	rs456180	5	14655699	G	A	-2.76E-04	PRS-CSx
Colorectum	PRS7	rs456238	5	141083824	G	A	-1.10E-03	PRS-CSx
Colorectum	PRS7	rs4566770	5	75576313	T	C	-1.28E-03	PRS-CSx
Colorectum	PRS7	rs4570156	8	83844826	A	G	-3.63E-04	PRS-CSx
Colorectum	PRS7	rs4570460	1	202689028	A	G	-6.42E-03	PRS-CSx
Colorectum	PRS7	rs4571329	4	105785830	G	A	4.50E-04	PRS-CSx
Colorectum	PRS7	rs4572561	2	183316895	T	G	1.36E-04	PRS-CSx
Colorectum	PRS7	rs4572894	4	123472786	G	A	5.30E-04	PRS-CSx
Colorectum	PRS7	rs457420	9	136909261	C	T	-5.10E-04	PRS-CSx
Colorectum	PRS7	rs4576462	8	143467482	T	C	7.85E-04	PRS-CSx
Colorectum	PRS7	rs457705	21	40191431	G	T	5.09E-04	PRS-CSx
Colorectum	PRS7	rs4578040	9	19359195	G	A	2.21E-04	PRS-CSx
Colorectum	PRS7	rs4579119	4	105797476	C	T	1.02E-03	PRS-CSx
Colorectum	PRS7	rs458021	4	187629497	A	G	1.08E-03	PRS-CSx
Colorectum	PRS7	rs4581406	11	46962451	T	C	1.47E-04	PRS-CSx
Colorectum	PRS7	rs4581549	12	24077866	T	C	-2.16E-03	PRS-CSx
Colorectum	PRS7	rs45840	3	7822471	T	C	-2.57E-04	PRS-CSx
Colorectum	PRS7	rs4584486	10	8668698	T	C	3.74E-05	PRS-CSx
Colorectum	PRS7	rs458536	6	95056791	C	T	-6.01E-04	PRS-CSx
Colorectum	PRS7	rs4585948	1	38345021	C	T	-1.63E-04	PRS-CSx
Colorectum	PRS7	rs4587689	11	47129854	A	C	1.36E-04	PRS-CSx
Colorectum	PRS7	rs4587727	11	21194850	T	C	-5.09E-04	PRS-CSx
Colorectum	PRS7	rs4588357	3	133873102	T	G	1.72E-04	PRS-CSx
Colorectum	PRS7	rs4589926	3	64601550	T	C	2.93E-04	PRS-CSx
Colorectum	PRS7	rs4590377	7	77707557	C	T	1.23E-03	PRS-CSx
Colorectum	PRS7	rs4591267	19	41765407	C	A	-3.33E-04	PRS-CSx
Colorectum	PRS7	rs4591420	21	16077143	A	G	-4.62E-04	PRS-CSx
Colorectum	PRS7	rs4595478	10	111800145	T	C	-1.25E-04	PRS-CSx
Colorectum	PRS7	rs4595719	14	58768802	A	G	2.66E-04	PRS-CSx
Colorectum	PRS7	rs4596036	20	47258613	A	G	-1.39E-04	PRS-CSx
Colorectum	PRS7	rs4597580	2	48743933	A	G	-1.51E-04	PRS-CSx
Colorectum	PRS7	rs4598109	6	29344704	T	C	-2.01E-04	PRS-CSx
Colorectum	PRS7	rs4598803	13	42762871	G	A	3.16E-04	PRS-CSx

Colorectum	PRS7	rs4599077	2	176542513	A	G	1.59E-04	PRS-CSx
Colorectum	PRS7	rs4599143	2	239871074	A	G	4.36E-05	PRS-CSx
Colorectum	PRS7	rs460154	9	136909309	G	A	-2.11E-03	PRS-CSx
Colorectum	PRS7	rs4603144	1	12803050	G	A	2.69E-04	PRS-CSx
Colorectum	PRS7	rs4603932	3	152321403	T	G	1.87E-04	PRS-CSx
Colorectum	PRS7	rs4604070	4	130196647	A	G	5.78E-04	PRS-CSx
Colorectum	PRS7	rs4604209	5	177419356	T	C	-2.19E-03	PRS-CSx
Colorectum	PRS7	rs460475	21	40224953	A	G	6.55E-04	PRS-CSx
Colorectum	PRS7	rs4606447	11	46691737	G	A	1.64E-04	PRS-CSx
Colorectum	PRS7	rs4607332	5	100733720	G	A	1.54E-03	PRS-CSx
Colorectum	PRS7	rs4607887	1	104834014	G	A	2.12E-04	PRS-CSx
Colorectum	PRS7	rs4608113	11	111172179	T	C	2.25E-03	PRS-CSx
Colorectum	PRS7	rs460982	21	40192577	C	T	3.83E-04	PRS-CSx
Colorectum	PRS7	rs461155	21	40191638	G	A	2.98E-04	PRS-CSx
Colorectum	PRS7	rs4611698	20	47256479	C	T	-7.54E-05	PRS-CSx
Colorectum	PRS7	rs4612984	14	57670959	C	T	6.50E-04	PRS-CSx
Colorectum	PRS7	rs461404	5	40799540	G	A	5.65E-05	PRS-CSx
Colorectum	PRS7	rs4615151	4	46647279	T	C	-3.75E-04	PRS-CSx
Colorectum	PRS7	rs4617548	11	16133413	A	G	-3.08E-04	PRS-CSx
Colorectum	PRS7	rs4617622	11	111183157	A	G	1.24E-03	PRS-CSx
Colorectum	PRS7	rs4618739	8	65782242	C	T	-6.04E-04	PRS-CSx
Colorectum	PRS7	rs4618985	1	12671324	C	A	5.97E-04	PRS-CSx
Colorectum	PRS7	rs4619890	4	7853160	A	G	-3.01E-04	PRS-CSx
Colorectum	PRS7	rs4621840	8	65780968	A	G	-4.32E-04	PRS-CSx
Colorectum	PRS7	rs4621895	9	20582556	T	C	2.80E-04	PRS-CSx
Colorectum	PRS7	rs462366	5	40821324	T	C	3.75E-04	PRS-CSx
Colorectum	PRS7	rs4624372	2	225553128	A	G	1.96E-04	PRS-CSx
Colorectum	PRS7	rs4625254	1	167555584	C	T	2.88E-04	PRS-CSx
Colorectum	PRS7	rs4627822	4	90024739	G	A	2.36E-04	PRS-CSx
Colorectum	PRS7	rs4629846	8	141068140	A	G	3.15E-04	PRS-CSx
Colorectum	PRS7	rs463064	9	89772284	A	G	2.67E-04	PRS-CSx
Colorectum	PRS7	rs4631962	12	4373132	A	G	4.45E-03	PRS-CSx
Colorectum	PRS7	rs4632520	3	152324643	C	T	2.87E-04	PRS-CSx
Colorectum	PRS7	rs4633317	1	45985157	C	T	2.39E-04	PRS-CSx
Colorectum	PRS7	rs4633725	16	71380320	C	T	-1.84E-03	PRS-CSx
Colorectum	PRS7	rs4634868	1	38465315	A	G	1.87E-03	PRS-CSx
Colorectum	PRS7	rs4636015	6	27097316	T	C	-7.56E-04	PRS-CSx
Colorectum	PRS7	rs4636298	9	139803958	G	A	-1.43E-04	PRS-CSx
Colorectum	PRS7	rs463991	9	89763193	C	A	2.61E-04	PRS-CSx
Colorectum	PRS7	rs464052	21	40234138	G	A	9.31E-04	PRS-CSx
Colorectum	PRS7	rs464074	21	40234202	G	A	3.20E-04	PRS-CSx
Colorectum	PRS7	rs4641991	2	48745736	A	G	5.72E-05	PRS-CSx
Colorectum	PRS7	rs464332	5	67299387	C	T	2.51E-04	PRS-CSx
Colorectum	PRS7	rs4643636	22	36848527	C	T	-1.96E-03	PRS-CSx
Colorectum	PRS7	rs464366	21	40225071	T	C	2.34E-04	PRS-CSx
Colorectum	PRS7	rs464476	21	40225441	T	C	4.74E-04	PRS-CSx
Colorectum	PRS7	rs464582	14	59101448	T	C	4.79E-04	PRS-CSx
Colorectum	PRS7	rs4645989	1	15850343	A	G	-4.41E-04	PRS-CSx
Colorectum	PRS7	rs4646045	1	15831992	A	G	-3.18E-04	PRS-CSx
Colorectum	PRS7	rs4646047	1	15831783	T	C	-3.44E-04	PRS-CSx
Colorectum	PRS7	rs4646093	1	15821400	C	A	-5.22E-04	PRS-CSx
Colorectum	PRS7	rs4647859	17	74498762	C	T	7.92E-04	PRS-CSx
Colorectum	PRS7	rs4648356	1	2709164	C	A	-6.64E-05	PRS-CSx
Colorectum	PRS7	rs4648360	1	2723345	T	C	-1.04E-04	PRS-CSx
Colorectum	PRS7	rs4648390	1	2700372	T	C	1.21E-04	PRS-CSx
Colorectum	PRS7	rs4648648	1	2512452	G	A	-2.81E-04	PRS-CSx

Colorectum	PRS7	rs4650310	1	69445925	G	A	1.29E-03	PRS-CSx
Colorectum	PRS7	rs4651138	1	183001312	C	A	5.16E-04	PRS-CSx
Colorectum	PRS7	rs4652697	1	182275925	C	T	2.82E-04	PRS-CSx
Colorectum	PRS7	rs4652762	1	182993960	T	C	2.37E-04	PRS-CSx
Colorectum	PRS7	rs4652764	1	182994115	G	A	3.95E-04	PRS-CSx
Colorectum	PRS7	rs4652769	1	183009196	T	C	4.92E-04	PRS-CSx
Colorectum	PRS7	rs4652795	1	183266182	C	T	1.51E-04	PRS-CSx
Colorectum	PRS7	rs4653522	1	223858133	T	C	-1.94E-04	PRS-CSx
Colorectum	PRS7	rs4655044	1	22659375	C	T	3.59E-04	PRS-CSx
Colorectum	PRS7	rs4655059	1	22735906	A	C	-3.87E-04	PRS-CSx
Colorectum	PRS7	rs465724	9	136910097	C	T	-9.57E-04	PRS-CSx
Colorectum	PRS7	rs4657674	1	167561258	A	G	3.39E-04	PRS-CSx
Colorectum	PRS7	rs4657678	1	167561752	C	T	3.94E-04	PRS-CSx
Colorectum	PRS7	rs4658238	1	92064082	C	T	2.94E-04	PRS-CSx
Colorectum	PRS7	rs4658243	1	92067746	C	T	5.00E-04	PRS-CSx
Colorectum	PRS7	rs465927	6	95044885	A	G	-1.25E-03	PRS-CSx
Colorectum	PRS7	rs4660436	1	41042068	A	G	8.44E-05	PRS-CSx
Colorectum	PRS7	rs4660919	1	46754486	G	A	5.12E-04	PRS-CSx
Colorectum	PRS7	rs4660921	1	46781602	G	T	3.02E-04	PRS-CSx
Colorectum	PRS7	rs466108	5	40796746	A	C	1.93E-04	PRS-CSx
Colorectum	PRS7	rs4661486	1	14690876	G	A	1.12E-03	PRS-CSx
Colorectum	PRS7	rs466157	5	141082821	T	C	-8.81E-04	PRS-CSx
Colorectum	PRS7	rs4663369	2	235181704	G	A	5.76E-04	PRS-CSx
Colorectum	PRS7	rs4663370	2	235181900	C	T	7.42E-04	PRS-CSx
Colorectum	PRS7	rs4664664	2	154164829	T	G	4.59E-04	PRS-CSx
Colorectum	PRS7	rs4665022	2	159957945	A	G	-1.64E-04	PRS-CSx
Colorectum	PRS7	rs4666062	2	28585156	G	A	6.91E-04	PRS-CSx
Colorectum	PRS7	rs4666586	2	183354876	T	C	2.53E-04	PRS-CSx
Colorectum	PRS7	rs4666587	2	183354887	T	C	1.42E-04	PRS-CSx
Colorectum	PRS7	rs4666841	2	183351622	C	T	2.02E-04	PRS-CSx
Colorectum	PRS7	rs4667514	2	167393816	A	G	-2.54E-03	PRS-CSx
Colorectum	PRS7	rs4667570	2	169047506	T	C	-2.85E-04	PRS-CSx
Colorectum	PRS7	rs4668040	2	169027867	C	T	-2.66E-04	PRS-CSx
Colorectum	PRS7	rs4668578	2	8728607	C	T	-4.97E-04	PRS-CSx
Colorectum	PRS7	rs4669242	2	8213186	T	G	-4.28E-04	PRS-CSx
Colorectum	PRS7	rs467019	5	143880746	T	G	-1.25E-04	PRS-CSx
Colorectum	PRS7	rs4671194	2	67870799	C	T	-8.95E-04	PRS-CSx
Colorectum	PRS7	rs4673259	2	204582623	C	T	9.80E-04	PRS-CSx
Colorectum	PRS7	rs4673313	2	200189323	C	T	-2.17E-04	PRS-CSx
Colorectum	PRS7	rs4673349	2	207153511	G	A	-2.14E-04	PRS-CSx
Colorectum	PRS7	rs4673350	2	207178872	T	C	-2.44E-04	PRS-CSx
Colorectum	PRS7	rs4674	19	41930396	G	A	2.31E-04	PRS-CSx
Colorectum	PRS7	rs4675588	2	206985925	T	G	2.14E-04	PRS-CSx
Colorectum	PRS7	rs467567	21	40248440	G	A	7.72E-04	PRS-CSx
Colorectum	PRS7	rs4676272	2	109937171	G	A	6.32E-05	PRS-CSx
Colorectum	PRS7	rs4676363	2	241256535	T	G	8.37E-04	PRS-CSx
Colorectum	PRS7	rs4677306	3	73632188	T	C	3.21E-04	PRS-CSx
Colorectum	PRS7	rs467940	21	40223037	G	A	4.79E-04	PRS-CSx
Colorectum	PRS7	rs467985	21	40222039	A	G	6.12E-04	PRS-CSx
Colorectum	PRS7	rs4679989	3	152364694	T	G	2.71E-04	PRS-CSx
Colorectum	PRS7	rs4680074	3	153286204	T	C	-2.19E-03	PRS-CSx
Colorectum	PRS7	rs4680887	3	28031787	G	T	5.91E-04	PRS-CSx
Colorectum	PRS7	rs4681449	3	148521601	G	A	-4.25E-04	PRS-CSx
Colorectum	PRS7	rs4682133	3	113062986	A	G	1.69E-04	PRS-CSx
Colorectum	PRS7	rs4682145	3	113614340	A	G	-3.98E-04	PRS-CSx
Colorectum	PRS7	rs4682465	3	112823658	T	C	-1.24E-03	PRS-CSx

Colorectum	PRS7	rs4682475	3	112921268	T	C	2.48E-03	PRS-CSx
Colorectum	PRS7	rs4682479	3	112974641	T	C	3.67E-04	PRS-CSx
Colorectum	PRS7	rs4682511	3	113624931	A	G	-2.50E-04	PRS-CSx
Colorectum	PRS7	rs4682664	3	133919909	C	T	-4.34E-04	PRS-CSx
Colorectum	PRS7	rs4683499	3	140180230	A	G	5.42E-04	PRS-CSx
Colorectum	PRS7	rs4683702	3	142617138	G	T	5.17E-04	PRS-CSx
Colorectum	PRS7	rs4684384	3	3347090	G	A	-1.24E-03	PRS-CSx
Colorectum	PRS7	rs4686997	3	188539861	G	A	5.31E-04	PRS-CSx
Colorectum	PRS7	rs4687853	3	119130360	G	A	2.69E-04	PRS-CSx
Colorectum	PRS7	rs4687855	3	119132554	A	G	-4.68E-04	PRS-CSx
Colorectum	PRS7	rs4688005	3	119197285	A	G	-5.23E-04	PRS-CSx
Colorectum	PRS7	rs4688007	3	119198873	G	A	-4.29E-04	PRS-CSx
Colorectum	PRS7	rs4688084	3	117935444	G	A	-1.02E-03	PRS-CSx
Colorectum	PRS7	rs4688212	3	64586031	G	T	5.06E-04	PRS-CSx
Colorectum	PRS7	rs4688255	3	65965346	T	C	5.09E-04	PRS-CSx
Colorectum	PRS7	rs468837	21	40217036	C	T	4.15E-04	PRS-CSx
Colorectum	PRS7	rs4688374	3	63079501	A	G	-1.04E-03	PRS-CSx
Colorectum	PRS7	rs4688486	3	64582081	C	T	2.42E-04	PRS-CSx
Colorectum	PRS7	rs4688489	3	64594603	T	C	4.71E-04	PRS-CSx
Colorectum	PRS7	rs468849	21	40219605	G	A	3.11E-04	PRS-CSx
Colorectum	PRS7	rs4688490	3	64598760	A	G	3.29E-04	PRS-CSx
Colorectum	PRS7	rs4688772	3	129432347	A	G	2.20E-04	PRS-CSx
Colorectum	PRS7	rs4688810	3	129375422	T	G	4.41E-04	PRS-CSx
Colorectum	PRS7	rs4688813	3	129490835	T	C	1.54E-04	PRS-CSx
Colorectum	PRS7	rs4691817	4	163080189	C	T	4.62E-04	PRS-CSx
Colorectum	PRS7	rs469303	21	40219958	G	A	6.36E-04	PRS-CSx
Colorectum	PRS7	rs4693352	4	94831571	A	G	2.55E-04	PRS-CSx
Colorectum	PRS7	rs469353	21	40219058	T	G	4.44E-04	PRS-CSx
Colorectum	PRS7	rs4693752	4	86905209	T	G	-3.09E-04	PRS-CSx
Colorectum	PRS7	rs4693753	4	87026302	C	T	9.38E-04	PRS-CSx
Colorectum	PRS7	rs4694202	4	70120322	T	C	2.71E-04	PRS-CSx
Colorectum	PRS7	rs4697202	4	18920624	A	G	-3.60E-04	PRS-CSx
Colorectum	PRS7	rs4697204	4	18921835	C	T	-3.35E-04	PRS-CSx
Colorectum	PRS7	rs4697406	4	23666030	A	G	-1.97E-05	PRS-CSx
Colorectum	PRS7	rs4697407	4	23666163	C	T	-1.18E-05	PRS-CSx
Colorectum	PRS7	rs4698929	4	105872720	A	G	-5.02E-04	PRS-CSx
Colorectum	PRS7	rs4699142	4	105872868	G	A	-1.38E-03	PRS-CSx
Colorectum	PRS7	rs4699366	4	99662224	C	T	9.75E-04	PRS-CSx
Colorectum	PRS7	rs4699680	4	99759132	G	A	-5.09E-04	PRS-CSx
Colorectum	PRS7	rs4700243	5	57338357	C	T	2.04E-04	PRS-CSx
Colorectum	PRS7	rs4700434	5	61035802	A	G	-5.51E-04	PRS-CSx
Colorectum	PRS7	rs4702043	5	14661675	C	T	-2.14E-04	PRS-CSx
Colorectum	PRS7	rs4703156	5	100907855	G	T	5.28E-04	PRS-CSx
Colorectum	PRS7	rs4704166	5	74324902	T	C	-9.81E-05	PRS-CSx
Colorectum	PRS7	rs4704330	5	75841187	A	G	1.90E-04	PRS-CSx
Colorectum	PRS7	rs4704333	5	75842335	C	T	2.92E-04	PRS-CSx
Colorectum	PRS7	rs4705739	5	111984920	T	G	-9.96E-04	PRS-CSx
Colorectum	PRS7	rs4706577	6	74983451	C	T	-1.60E-04	PRS-CSx
Colorectum	PRS7	rs4706579	6	75031337	T	C	-3.28E-04	PRS-CSx
Colorectum	PRS7	rs4707403	6	88466990	T	C	8.07E-04	PRS-CSx
Colorectum	PRS7	rs4707490	6	89264664	A	G	9.72E-04	PRS-CSx
Colorectum	PRS7	rs4708015	6	73752010	G	A	-5.03E-04	PRS-CSx
Colorectum	PRS7	rs4708135	6	74877900	G	A	-8.69E-04	PRS-CSx
Colorectum	PRS7	rs4709395	6	160478789	G	A	4.02E-04	PRS-CSx
Colorectum	PRS7	rs4709823	6	164534582	T	C	-9.96E-04	PRS-CSx
Colorectum	PRS7	rs4711167	6	28294888	C	T	2.06E-04	PRS-CSx

Colorectum	PRS7	rs4711206	6	29987184	A	G	-9.25E-05	PRS-CSx
Colorectum	PRS7	rs4711209	6	30047403	A	G	-6.35E-05	PRS-CSx
Colorectum	PRS7	rs4711689	6	41692812	G	A	-1.07E-03	PRS-CSx
Colorectum	PRS7	rs4711757	6	43888710	C	T	-2.42E-03	PRS-CSx
Colorectum	PRS7	rs4711910	6	13066375	C	T	1.13E-03	PRS-CSx
Colorectum	PRS7	rs4712523	6	20657564	G	A	-4.81E-04	PRS-CSx
Colorectum	PRS7	rs4712944	6	25488265	A	C	-7.29E-04	PRS-CSx
Colorectum	PRS7	rs4712970	6	25770707	G	A	3.03E-05	PRS-CSx
Colorectum	PRS7	rs4713159	6	28283772	A	G	8.73E-05	PRS-CSx
Colorectum	PRS7	rs4713240	6	29710726	G	A	-1.65E-04	PRS-CSx
Colorectum	PRS7	rs4713270	6	29934697	A	G	-4.12E-04	PRS-CSx
Colorectum	PRS7	rs4713332	6	11270637	G	A	2.52E-04	PRS-CSx
Colorectum	PRS7	rs4713335	6	11277154	T	G	8.85E-04	PRS-CSx
Colorectum	PRS7	rs4713466	6	31435869	T	C	3.15E-04	PRS-CSx
Colorectum	PRS7	rs471359	1	21656500	C	T	-6.44E-04	PRS-CSx
Colorectum	PRS7	rs471364	9	15289578	C	T	-1.03E-03	PRS-CSx
Colorectum	PRS7	rs471371	9	33870844	T	C	-2.02E-04	PRS-CSx
Colorectum	PRS7	rs4713864	6	35416039	C	A	2.45E-04	PRS-CSx
Colorectum	PRS7	rs4714340	6	12255290	C	T	5.57E-04	PRS-CSx
Colorectum	PRS7	rs4714503	6	41633272	C	T	-2.02E-03	PRS-CSx
Colorectum	PRS7	rs4714560	6	42026190	A	G	-1.25E-03	PRS-CSx
Colorectum	PRS7	rs4714677	6	43327039	G	T	-2.43E-04	PRS-CSx
Colorectum	PRS7	rs4714696	6	43719993	C	T	-3.05E-03	PRS-CSx
Colorectum	PRS7	rs4714717	6	43904880	A	G	-8.99E-04	PRS-CSx
Colorectum	PRS7	rs471620	6	122794677	T	C	5.08E-04	PRS-CSx
Colorectum	PRS7	rs4719466	7	16494457	G	A	3.95E-04	PRS-CSx
Colorectum	PRS7	rs4720409	7	41884913	A	G	6.33E-04	PRS-CSx
Colorectum	PRS7	rs4720490	7	45057134	G	A	-2.00E-04	PRS-CSx
Colorectum	PRS7	rs4720497	7	45161049	A	G	-2.75E-04	PRS-CSx
Colorectum	PRS7	rs4721321	7	2068470	G	A	-7.60E-04	PRS-CSx
Colorectum	PRS7	rs4722120	7	22304800	A	C	7.97E-04	PRS-CSx
Colorectum	PRS7	rs4722508	7	25777597	C	T	-5.30E-04	PRS-CSx
Colorectum	PRS7	rs472324	9	9094805	A	G	3.35E-04	PRS-CSx
Colorectum	PRS7	rs4724335	7	44978350	G	A	3.18E-04	PRS-CSx
Colorectum	PRS7	rs4724342	7	45036860	T	G	-4.72E-04	PRS-CSx
Colorectum	PRS7	rs4724354	7	45104623	C	T	-4.99E-04	PRS-CSx
Colorectum	PRS7	rs4724362	7	45161303	C	A	-1.40E-04	PRS-CSx
Colorectum	PRS7	rs4724508	7	46882173	C	T	6.19E-04	PRS-CSx
Colorectum	PRS7	rs4725288	7	10524535	C	T	-1.33E-03	PRS-CSx
Colorectum	PRS7	rs4725991	7	150801238	G	A	3.01E-03	PRS-CSx
Colorectum	PRS7	rs4727445	7	99639400	T	C	1.60E-04	PRS-CSx
Colorectum	PRS7	rs4729071	7	92745581	C	T	5.76E-04	PRS-CSx
Colorectum	PRS7	rs4729249	7	95919699	A	C	1.55E-03	PRS-CSx
Colorectum	PRS7	rs4729263	7	96156130	G	T	-1.41E-03	PRS-CSx
Colorectum	PRS7	rs4729566	7	99614313	G	A	-5.41E-04	PRS-CSx
Colorectum	PRS7	rs472959	5	172324558	A	G	5.26E-03	PRS-CSx
Colorectum	PRS7	rs4730556	7	112257009	G	A	-1.14E-03	PRS-CSx
Colorectum	PRS7	rs4731568	7	128904801	C	T	2.93E-04	PRS-CSx
Colorectum	PRS7	rs4731772	7	130932392	G	A	8.88E-04	PRS-CSx
Colorectum	PRS7	rs4732060	7	134530381	T	C	-1.90E-04	PRS-CSx
Colorectum	PRS7	rs473279	1	38275802	T	C	2.72E-04	PRS-CSx
Colorectum	PRS7	rs4733807	8	128592256	G	A	-1.11E-03	PRS-CSx
Colorectum	PRS7	rs4735516	8	98984840	C	A	-8.92E-04	PRS-CSx
Colorectum	PRS7	rs4735521	8	99008015	C	T	-3.89E-04	PRS-CSx
Colorectum	PRS7	rs4735679	8	75378266	G	A	8.06E-04	PRS-CSx
Colorectum	PRS7	rs4736805	8	41231323	A	G	6.93E-04	PRS-CSx

Colorectum	PRS7	rs4737472	8	58653248	T	C	4.32E-04	PRS-CSx
Colorectum	PRS7	rs4738610	8	58640353	G	A	-1.92E-04	PRS-CSx
Colorectum	PRS7	rs4738613	8	58653334	A	G	-2.72E-04	PRS-CSx
Colorectum	PRS7	rs4738721	8	59753928	A	G	-9.47E-04	PRS-CSx
Colorectum	PRS7	rs4738723	8	59776637	T	C	-6.48E-04	PRS-CSx
Colorectum	PRS7	rs474122	18	3603112	A	G	-1.95E-03	PRS-CSx
Colorectum	PRS7	rs4742622	9	9805193	G	A	1.85E-05	PRS-CSx
Colorectum	PRS7	rs4742675	9	109581132	A	C	-4.93E-04	PRS-CSx
Colorectum	PRS7	rs4742756	9	101830199	G	A	4.59E-04	PRS-CSx
Colorectum	PRS7	rs4742851	9	105471880	A	G	2.73E-04	PRS-CSx
Colorectum	PRS7	rs4743034	9	109632353	A	G	-3.98E-04	PRS-CSx
Colorectum	PRS7	rs4743322	9	101835165	C	A	3.73E-04	PRS-CSx
Colorectum	PRS7	rs4743325	9	101849727	G	T	4.12E-04	PRS-CSx
Colorectum	PRS7	rs4743486	9	104522208	A	G	1.23E-03	PRS-CSx
Colorectum	PRS7	rs4743501	9	98824475	A	C	1.73E-03	PRS-CSx
Colorectum	PRS7	rs4743577	9	105507920	G	A	2.17E-04	PRS-CSx
Colorectum	PRS7	rs4746104	10	73744987	C	T	-2.35E-04	PRS-CSx
Colorectum	PRS7	rs4746136	10	75300994	G	A	-2.92E-04	PRS-CSx
Colorectum	PRS7	rs4747229	10	73747591	T	C	-1.48E-04	PRS-CSx
Colorectum	PRS7	rs4747880	10	6152967	T	C	-3.05E-04	PRS-CSx
Colorectum	PRS7	rs4748624	10	20188728	A	C	7.74E-04	PRS-CSx
Colorectum	PRS7	rs4749350	10	29102482	A	G	4.66E-04	PRS-CSx
Colorectum	PRS7	rs4749791	10	8633861	T	C	9.33E-05	PRS-CSx
Colorectum	PRS7	rs4749793	10	8636181	A	G	-2.58E-05	PRS-CSx
Colorectum	PRS7	rs4749806	10	8711723	C	T	9.01E-04	PRS-CSx
Colorectum	PRS7	rs4749821	10	8801845	C	T	2.80E-04	PRS-CSx
Colorectum	PRS7	rs4749920	10	6071453	C	T	5.85E-05	PRS-CSx
Colorectum	PRS7	rs4750190	10	6299773	G	A	8.77E-04	PRS-CSx
Colorectum	PRS7	rs4752815	11	46978698	A	G	2.04E-04	PRS-CSx
Colorectum	PRS7	rs4752816	11	46979023	G	A	9.19E-05	PRS-CSx
Colorectum	PRS7	rs4752926	11	46692870	T	C	1.52E-04	PRS-CSx
Colorectum	PRS7	rs4752927	11	46702160	C	T	1.06E-04	PRS-CSx
Colorectum	PRS7	rs4752932	11	46773354	T	C	2.86E-04	PRS-CSx
Colorectum	PRS7	rs4752933	11	46803785	G	A	9.80E-05	PRS-CSx
Colorectum	PRS7	rs4752936	11	46821078	A	C	1.84E-04	PRS-CSx
Colorectum	PRS7	rs4752957	11	47125632	G	T	3.94E-04	PRS-CSx
Colorectum	PRS7	rs4752965	11	47159053	G	A	1.19E-04	PRS-CSx
Colorectum	PRS7	rs4752969	11	47185510	A	G	1.73E-04	PRS-CSx
Colorectum	PRS7	rs4753618	11	94498860	A	G	2.64E-04	PRS-CSx
Colorectum	PRS7	rs4753623	11	94530693	G	A	3.83E-04	PRS-CSx
Colorectum	PRS7	rs4756119	11	34256377	C	T	1.79E-03	PRS-CSx
Colorectum	PRS7	rs4757379	11	16014954	C	T	-9.41E-04	PRS-CSx
Colorectum	PRS7	rs4757752	11	12680889	A	G	-8.42E-04	PRS-CSx
Colorectum	PRS7	rs4758622	11	3009921	A	G	4.96E-04	PRS-CSx
Colorectum	PRS7	rs4758685	12	122626842	A	G	2.88E-04	PRS-CSx
Colorectum	PRS7	rs4758690	12	122610909	G	A	1.85E-04	PRS-CSx
Colorectum	PRS7	rs4759073	12	54653258	A	G	1.34E-04	PRS-CSx
Colorectum	PRS7	rs4759542	12	131576002	T	C	9.84E-04	PRS-CSx
Colorectum	PRS7	rs4760261	12	59513191	A	G	-6.72E-04	PRS-CSx
Colorectum	PRS7	rs4762596	12	96051418	G	A	-1.48E-03	PRS-CSx
Colorectum	PRS7	rs4763567	12	10782116	A	G	4.74E-03	PRS-CSx
Colorectum	PRS7	rs4764039	12	14064461	T	C	-1.87E-04	PRS-CSx
Colorectum	PRS7	rs4764041	12	14095214	T	C	-4.51E-04	PRS-CSx
Colorectum	PRS7	rs4764497	12	6428116	G	T	1.82E-03	PRS-CSx
Colorectum	PRS7	rs4764552	12	6386518	G	A	-2.68E-03	PRS-CSx
Colorectum	PRS7	rs4764758	12	101237883	A	G	-3.02E-04	PRS-CSx

Colorectum	PRS7	rs4765933	12	2558475	A	G	-6.96E-04	PRS-CSx
Colorectum	PRS7	rs4766227	12	4363350	C	T	6.66E-04	PRS-CSx
Colorectum	PRS7	rs4766442	12	111401693	A	G	4.23E-04	PRS-CSx
Colorectum	PRS7	rs4766443	12	111401798	G	A	3.20E-04	PRS-CSx
Colorectum	PRS7	rs4766451	12	111670682	G	T	1.02E-04	PRS-CSx
Colorectum	PRS7	rs4766494	12	109577952	T	C	-3.00E-04	PRS-CSx
Colorectum	PRS7	rs4766521	12	111386961	C	T	2.43E-04	PRS-CSx
Colorectum	PRS7	rs4766522	12	111393519	G	T	2.68E-04	PRS-CSx
Colorectum	PRS7	rs4766524	12	111399446	C	T	3.14E-04	PRS-CSx
Colorectum	PRS7	rs4766525	12	111399660	C	T	3.40E-04	PRS-CSx
Colorectum	PRS7	rs4766526	12	111401316	T	G	3.90E-04	PRS-CSx
Colorectum	PRS7	rs4766527	12	111405470	G	T	5.77E-04	PRS-CSx
Colorectum	PRS7	rs4766548	12	109632758	G	A	-6.70E-04	PRS-CSx
Colorectum	PRS7	rs4766558	12	111664061	T	G	1.64E-05	PRS-CSx
Colorectum	PRS7	rs4766559	12	111666984	C	T	2.99E-04	PRS-CSx
Colorectum	PRS7	rs4766566	12	111706877	C	T	-1.43E-03	PRS-CSx
Colorectum	PRS7	rs4766573	12	111830809	C	A	3.21E-04	PRS-CSx
Colorectum	PRS7	rs4766648	12	113119813	A	G	3.85E-04	PRS-CSx
Colorectum	PRS7	rs4766679	12	113413025	G	A	6.02E-04	PRS-CSx
Colorectum	PRS7	rs4766773	12	116171826	G	A	2.63E-04	PRS-CSx
Colorectum	PRS7	rs4766988	12	113105030	A	G	1.94E-04	PRS-CSx
Colorectum	PRS7	rs4767000	12	113168368	A	G	1.41E-04	PRS-CSx
Colorectum	PRS7	rs4767002	12	113169770	C	T	2.29E-04	PRS-CSx
Colorectum	PRS7	rs4767003	12	113190918	T	C	2.78E-05	PRS-CSx
Colorectum	PRS7	rs4767017	12	113276968	C	A	-2.89E-04	PRS-CSx
Colorectum	PRS7	rs4767019	12	113289070	G	A	-2.00E-04	PRS-CSx
Colorectum	PRS7	rs4767329	12	115929439	A	G	4.87E-04	PRS-CSx
Colorectum	PRS7	rs4767331	12	115931945	C	A	9.69E-04	PRS-CSx
Colorectum	PRS7	rs4767332	12	115932272	A	C	4.18E-04	PRS-CSx
Colorectum	PRS7	rs4767364	12	112521448	G	A	2.38E-04	PRS-CSx
Colorectum	PRS7	rs4767867	12	120421825	C	T	2.84E-04	PRS-CSx
Colorectum	PRS7	rs4767871	12	120468697	G	A	3.70E-04	PRS-CSx
Colorectum	PRS7	rs4768659	12	46115349	A	G	-3.21E-04	PRS-CSx
Colorectum	PRS7	rs4768660	12	46136636	T	C	-2.58E-04	PRS-CSx
Colorectum	PRS7	rs4770463	13	24134623	A	G	6.19E-05	PRS-CSx
Colorectum	PRS7	rs4770993	13	27083463	T	C	-2.02E-03	PRS-CSx
Colorectum	PRS7	rs4771446	13	103811939	A	G	-9.34E-04	PRS-CSx
Colorectum	PRS7	rs4772217	13	100181915	A	G	-1.26E-04	PRS-CSx
Colorectum	PRS7	rs4772426	13	102544949	A	G	-3.65E-04	PRS-CSx
Colorectum	PRS7	rs4772427	13	102554381	C	T	-4.60E-04	PRS-CSx
Colorectum	PRS7	rs4772510	13	103570881	T	C	-1.01E-03	PRS-CSx
Colorectum	PRS7	rs4773184	13	111080231	C	T	-2.96E-04	PRS-CSx
Colorectum	PRS7	rs4774386	15	61378686	C	T	1.11E-03	PRS-CSx
Colorectum	PRS7	rs4774557	15	50433700	C	T	7.94E-04	PRS-CSx
Colorectum	PRS7	rs4775359	15	61388504	A	G	1.65E-03	PRS-CSx
Colorectum	PRS7	rs4775934	15	51518769	C	T	4.24E-04	PRS-CSx
Colorectum	PRS7	rs4776338	15	67359883	T	C	3.35E-04	PRS-CSx
Colorectum	PRS7	rs4776548	15	71650154	A	G	4.77E-04	PRS-CSx
Colorectum	PRS7	rs4776779	15	66635669	T	C	-1.57E-04	PRS-CSx
Colorectum	PRS7	rs4776783	15	66662107	G	A	-2.16E-04	PRS-CSx
Colorectum	PRS7	rs4776990	15	68137364	C	T	-4.46E-04	PRS-CSx
Colorectum	PRS7	rs4777372	15	71643787	C	T	8.23E-04	PRS-CSx
Colorectum	PRS7	rs4777374	15	71658235	G	T	4.91E-04	PRS-CSx
Colorectum	PRS7	rs4777376	15	71672845	C	T	3.28E-04	PRS-CSx
Colorectum	PRS7	rs4777379	15	71673613	G	T	2.97E-04	PRS-CSx
Colorectum	PRS7	rs4777467	15	72138478	C	T	5.12E-04	PRS-CSx

Colorectum	PRS7	rs477859	20	60937597	A	G	-9.17E-03	PRS-CSx
Colorectum	PRS7	rs4780052	15	33154851	A	C	4.22E-03	PRS-CSx
Colorectum	PRS7	rs478103	1	9402403	T	C	-1.12E-03	PRS-CSx
Colorectum	PRS7	rs4781143	16	11809721	G	T	-3.38E-04	PRS-CSx
Colorectum	PRS7	rs4781701	16	16060942	C	T	-1.39E-03	PRS-CSx
Colorectum	PRS7	rs4783057	16	84785672	G	A	-5.27E-04	PRS-CSx
Colorectum	PRS7	rs4783665	16	68720470	A	G	-3.94E-04	PRS-CSx
Colorectum	PRS7	rs4783676	16	68801077	C	A	3.01E-04	PRS-CSx
Colorectum	PRS7	rs4785201	16	50089192	G	A	-2.72E-04	PRS-CSx
Colorectum	PRS7	rs4785386	16	50164049	G	A	-3.44E-04	PRS-CSx
Colorectum	PRS7	rs4786078	16	6111257	A	G	-8.53E-04	PRS-CSx
Colorectum	PRS7	rs478620	12	31770670	G	A	-1.26E-03	PRS-CSx
Colorectum	PRS7	rs4786490	16	4491190	A	G	5.16E-04	PRS-CSx
Colorectum	PRS7	rs4786635	16	1862724	A	G	5.81E-04	PRS-CSx
Colorectum	PRS7	rs4786965	16	1546443	A	G	-6.30E-04	PRS-CSx
Colorectum	PRS7	rs4787846	16	26528099	C	T	2.10E-03	PRS-CSx
Colorectum	PRS7	rs478882	9	205964	A	G	-1.48E-04	PRS-CSx
Colorectum	PRS7	rs4789546	17	76167393	A	G	2.53E-03	PRS-CSx
Colorectum	PRS7	rs4790726	17	4934107	T	C	-5.62E-04	PRS-CSx
Colorectum	PRS7	rs4792105	17	11111361	T	C	1.76E-03	PRS-CSx
Colorectum	PRS7	rs4792217	17	8005905	G	A	1.14E-03	PRS-CSx
Colorectum	PRS7	rs4793324	17	69007761	G	A	-9.74E-04	PRS-CSx
Colorectum	PRS7	rs4793325	17	69008228	T	C	-9.52E-04	PRS-CSx
Colorectum	PRS7	rs4793326	17	69008238	A	G	-7.60E-04	PRS-CSx
Colorectum	PRS7	rs4793427	17	70303024	C	T	-7.14E-04	PRS-CSx
Colorectum	PRS7	rs4793429	17	70304489	T	C	-8.61E-04	PRS-CSx
Colorectum	PRS7	rs4793460	17	70618841	C	T	4.61E-04	PRS-CSx
Colorectum	PRS7	rs4795281	17	36616059	C	T	-4.29E-04	PRS-CSx
Colorectum	PRS7	rs4795282	17	36616089	T	C	-4.82E-04	PRS-CSx
Colorectum	PRS7	rs479564	1	212629935	G	A	-3.53E-04	PRS-CSx
Colorectum	PRS7	rs4796502	17	6378976	C	T	8.07E-04	PRS-CSx
Colorectum	PRS7	rs479707	11	114333923	A	G	-1.22E-04	PRS-CSx
Colorectum	PRS7	rs4797718	18	12917703	T	C	-4.14E-03	PRS-CSx
Colorectum	PRS7	rs4797986	18	2476773	C	A	3.04E-03	PRS-CSx
Colorectum	PRS7	rs4798142	18	3916039	A	G	-7.84E-04	PRS-CSx
Colorectum	PRS7	rs4798494	18	6767965	T	C	-5.22E-04	PRS-CSx
Colorectum	PRS7	rs4798816	18	9473241	C	T	8.67E-04	PRS-CSx
Colorectum	PRS7	rs4798850	18	9752939	T	C	-2.42E-03	PRS-CSx
Colorectum	PRS7	rs4799358	18	31586132	G	T	-1.08E-03	PRS-CSx
Colorectum	PRS7	rs4799422	18	34098972	G	A	1.55E-04	PRS-CSx
Colorectum	PRS7	rs479994	1	183161729	C	T	-5.45E-05	PRS-CSx
Colorectum	PRS7	rs4800943	18	54020536	T	G	-4.07E-04	PRS-CSx
Colorectum	PRS7	rs4801908	19	52574723	A	G	-3.48E-04	PRS-CSx
Colorectum	PRS7	rs4802015	19	39552222	T	C	-5.35E-04	PRS-CSx
Colorectum	PRS7	rs4802066	19	38109301	A	G	-3.17E-04	PRS-CSx
Colorectum	PRS7	rs4803197	19	39552150	G	A	-5.81E-04	PRS-CSx
Colorectum	PRS7	rs4803228	19	38043260	G	T	-2.44E-04	PRS-CSx
Colorectum	PRS7	rs4803457	19	41861359	C	T	7.82E-04	PRS-CSx
Colorectum	PRS7	rs4803465	19	41918158	T	C	1.84E-04	PRS-CSx
Colorectum	PRS7	rs4804	1	212619339	T	C	-3.03E-04	PRS-CSx
Colorectum	PRS7	rs4805815	19	28492962	A	G	3.59E-04	PRS-CSx
Colorectum	PRS7	rs4805827	19	33423502	C	T	3.28E-04	PRS-CSx
Colorectum	PRS7	rs4805868	19	33762301	C	T	-6.44E-04	PRS-CSx
Colorectum	PRS7	rs480599	4	64256184	T	G	6.53E-04	PRS-CSx
Colorectum	PRS7	rs4806815	19	1867912	T	G	4.37E-04	PRS-CSx
Colorectum	PRS7	rs4807152	19	1851084	G	A	4.02E-04	PRS-CSx

Colorectum	PRS7	rs4808	7	116528240	T	C	-5.05E-04	PRS-CSx
Colorectum	PRS7	rs4808583	19	17278979	G	T	-2.85E-04	PRS-CSx
Colorectum	PRS7	rs4808784	19	18451661	T	C	9.90E-04	PRS-CSx
Colorectum	PRS7	rs480933	11	57488160	G	A	2.46E-04	PRS-CSx
Colorectum	PRS7	rs4809375	20	62497022	A	G	-2.15E-03	PRS-CSx
Colorectum	PRS7	rs4809499	20	61317643	T	C	-6.88E-04	PRS-CSx
Colorectum	PRS7	rs4809510	20	61323629	A	G	1.56E-03	PRS-CSx
Colorectum	PRS7	rs4809580	20	62184170	C	T	1.57E-03	PRS-CSx
Colorectum	PRS7	rs4809591	20	45188433	A	C	1.01E-03	PRS-CSx
Colorectum	PRS7	rs4809712	20	47248057	A	G	-4.16E-04	PRS-CSx
Colorectum	PRS7	rs4809715	20	47263751	C	T	-3.88E-04	PRS-CSx
Colorectum	PRS7	rs480989	11	65493992	A	G	-7.98E-04	PRS-CSx
Colorectum	PRS7	rs4810403	20	42548609	T	G	5.88E-04	PRS-CSx
Colorectum	PRS7	rs4810830	20	47219740	C	T	-1.35E-04	PRS-CSx
Colorectum	PRS7	rs4810831	20	47226012	C	T	-6.62E-04	PRS-CSx
Colorectum	PRS7	rs4810845	20	47249455	G	T	-7.63E-04	PRS-CSx
Colorectum	PRS7	rs4810849	20	47289944	T	C	-2.51E-04	PRS-CSx
Colorectum	PRS7	rs4811068	20	49063166	A	C	-4.90E-04	PRS-CSx
Colorectum	PRS7	rs4811154	20	49945122	C	T	4.31E-04	PRS-CSx
Colorectum	PRS7	rs4811155	20	49945761	A	G	2.83E-04	PRS-CSx
Colorectum	PRS7	rs4811835	20	55875968	A	G	-7.70E-05	PRS-CSx
Colorectum	PRS7	rs4811844	20	55993555	G	A	-1.82E-04	PRS-CSx
Colorectum	PRS7	rs4812761	20	42541539	T	C	1.25E-03	PRS-CSx
Colorectum	PRS7	rs4813143	20	13792741	C	A	5.88E-04	PRS-CSx
Colorectum	PRS7	rs4813210	20	1664840	C	T	1.21E-03	PRS-CSx
Colorectum	PRS7	rs4813800	20	6595646	A	G	2.63E-04	PRS-CSx
Colorectum	PRS7	rs4813802	20	6699595	G	T	3.24E-03	PRS-CSx
Colorectum	PRS7	rs4816455	21	34815794	G	A	6.49E-05	PRS-CSx
Colorectum	PRS7	rs4816642	21	41110282	A	G	1.27E-03	PRS-CSx
Colorectum	PRS7	rs4817	9	33917498	C	T	-1.78E-04	PRS-CSx
Colorectum	PRS7	rs4817966	21	40237762	C	A	4.12E-04	PRS-CSx
Colorectum	PRS7	rs4819241	21	47782064	T	C	4.55E-04	PRS-CSx
Colorectum	PRS7	rs4820091	22	21940189	T	G	1.32E-04	PRS-CSx
Colorectum	PRS7	rs4820237	22	36894653	A	C	-2.55E-04	PRS-CSx
Colorectum	PRS7	rs4820291	22	22422274	T	C	-1.88E-03	PRS-CSx
Colorectum	PRS7	rs4821112	22	21964761	G	A	2.00E-04	PRS-CSx
Colorectum	PRS7	rs4821116	22	21973319	T	C	-3.80E-04	PRS-CSx
Colorectum	PRS7	rs4821124	22	21979289	C	T	-3.67E-04	PRS-CSx
Colorectum	PRS7	rs4821130	22	21980894	C	T	2.11E-04	PRS-CSx
Colorectum	PRS7	rs4821667	22	37830081	A	C	-1.12E-03	PRS-CSx
Colorectum	PRS7	rs4821862	22	39441203	C	T	-3.38E-04	PRS-CSx
Colorectum	PRS7	rs4821942	22	40718100	A	G	-5.98E-04	PRS-CSx
Colorectum	PRS7	rs4821943	22	40722745	G	A	-1.78E-04	PRS-CSx
Colorectum	PRS7	rs4822225	22	43310652	G	T	2.10E-04	PRS-CSx
Colorectum	PRS7	rs4822231	22	43326110	C	T	6.87E-04	PRS-CSx
Colorectum	PRS7	rs4822232	22	43326807	G	T	3.44E-04	PRS-CSx
Colorectum	PRS7	rs4823002	22	29410104	C	T	-7.82E-04	PRS-CSx
Colorectum	PRS7	rs4823189	22	44493101	C	T	-5.91E-04	PRS-CSx
Colorectum	PRS7	rs4823297	22	45840721	G	T	3.53E-04	PRS-CSx
Colorectum	PRS7	rs482759	6	32195017	G	A	-5.75E-05	PRS-CSx
Colorectum	PRS7	rs483030	11	57566850	A	G	4.16E-04	PRS-CSx
Colorectum	PRS7	rs4833457	4	115412047	A	G	1.53E-05	PRS-CSx
Colorectum	PRS7	rs4833823	4	123318542	G	A	5.67E-04	PRS-CSx
Colorectum	PRS7	rs4833830	4	123422563	G	A	6.46E-04	PRS-CSx
Colorectum	PRS7	rs4835377	4	148039045	A	G	-4.48E-04	PRS-CSx
Colorectum	PRS7	rs483574	6	32194956	A	G	1.27E-04	PRS-CSx

Colorectum	PRS7	rs4836358	5	127376869	G	A	-5.66E-04	PRS-CSx
Colorectum	PRS7	rs4836439	5	128426433	T	C	-4.16E-04	PRS-CSx
Colorectum	PRS7	rs4836488	5	129368358	C	T	-7.77E-05	PRS-CSx
Colorectum	PRS7	rs483783	1	183162152	G	T	6.82E-05	PRS-CSx
Colorectum	PRS7	rs4839038	1	116102456	C	T	5.05E-04	PRS-CSx
Colorectum	PRS7	rs4839460	1	116154831	G	A	7.69E-04	PRS-CSx
Colorectum	PRS7	rs484020	18	12855440	G	A	-4.73E-04	PRS-CSx
Colorectum	PRS7	rs4842266	12	79951566	A	G	-9.53E-04	PRS-CSx
Colorectum	PRS7	rs4842316	12	79974565	T	C	-5.63E-04	PRS-CSx
Colorectum	PRS7	rs4842318	12	80044167	T	C	-1.88E-04	PRS-CSx
Colorectum	PRS7	rs4843654	16	87687924	G	T	5.36E-04	PRS-CSx
Colorectum	PRS7	rs4843884	16	86030100	C	T	6.08E-03	PRS-CSx
Colorectum	PRS7	rs484443	12	115111318	C	T	-5.72E-04	PRS-CSx
Colorectum	PRS7	rs4845010	1	210565133	G	A	-6.61E-04	PRS-CSx
Colorectum	PRS7	rs4845976	1	9835617	A	C	1.54E-03	PRS-CSx
Colorectum	PRS7	rs4846064	1	11934842	C	T	-4.34E-04	PRS-CSx
Colorectum	PRS7	rs4846066	1	11938050	G	A	-3.94E-04	PRS-CSx
Colorectum	PRS7	rs484611	9	113614978	T	C	-4.80E-05	PRS-CSx
Colorectum	PRS7	rs4846128	1	12670396	C	T	-8.23E-04	PRS-CSx
Colorectum	PRS7	rs4846479	1	218598410	G	T	-5.49E-04	PRS-CSx
Colorectum	PRS7	rs484717	11	34833640	T	G	-1.00E-03	PRS-CSx
Colorectum	PRS7	rs4847178	1	105144179	C	T	2.80E-04	PRS-CSx
Colorectum	PRS7	rs4849387	2	115969880	G	A	-8.49E-04	PRS-CSx
Colorectum	PRS7	rs484959	1	110366083	C	T	1.78E-03	PRS-CSx
Colorectum	PRS7	rs4850918	2	100504599	C	T	6.00E-04	PRS-CSx
Colorectum	PRS7	rs4852030	2	239865114	G	T	1.10E-04	PRS-CSx
Colorectum	PRS7	rs4852398	2	76478744	A	C	4.74E-04	PRS-CSx
Colorectum	PRS7	rs4852410	2	76943205	G	T	7.58E-04	PRS-CSx
Colorectum	PRS7	rs4852787	2	71706697	C	A	-9.00E-05	PRS-CSx
Colorectum	PRS7	rs4854033	2	241233933	C	T	5.25E-04	PRS-CSx
Colorectum	PRS7	rs4854249	2	95930630	A	G	-6.82E-05	PRS-CSx
Colorectum	PRS7	rs4854251	2	96037247	G	T	-4.09E-04	PRS-CSx
Colorectum	PRS7	rs4854253	2	96056112	C	T	-1.26E-04	PRS-CSx
Colorectum	PRS7	rs4854263	2	455588	T	C	-5.87E-04	PRS-CSx
Colorectum	PRS7	rs4854278	2	776605	T	C	-8.34E-04	PRS-CSx
Colorectum	PRS7	rs4854610	3	133805349	C	T	-2.67E-04	PRS-CSx
Colorectum	PRS7	rs4854768	3	133669718	C	T	-1.24E-04	PRS-CSx
Colorectum	PRS7	rs4854769	3	133670391	C	A	-1.29E-04	PRS-CSx
Colorectum	PRS7	rs4854787	3	133730608	T	C	-5.51E-03	PRS-CSx
Colorectum	PRS7	rs4854789	3	133731842	A	C	-1.38E-03	PRS-CSx
Colorectum	PRS7	rs4854812	3	133813757	A	G	-3.98E-04	PRS-CSx
Colorectum	PRS7	rs4854818	3	133830753	T	C	-1.90E-04	PRS-CSx
Colorectum	PRS7	rs4856944	3	66531866	C	T	4.90E-04	PRS-CSx
Colorectum	PRS7	rs4860862	4	68746883	G	T	1.39E-04	PRS-CSx
Colorectum	PRS7	rs4861321	4	40873745	A	G	-6.95E-04	PRS-CSx
Colorectum	PRS7	rs4862742	4	187817303	G	A	-4.44E-04	PRS-CSx
Colorectum	PRS7	rs4863666	4	140566851	G	A	-1.15E-03	PRS-CSx
Colorectum	PRS7	rs4863797	4	139327019	A	G	-8.96E-04	PRS-CSx
Colorectum	PRS7	rs4865243	4	58421116	T	C	2.74E-04	PRS-CSx
Colorectum	PRS7	rs4865744	5	52082774	A	G	-1.52E-03	PRS-CSx
Colorectum	PRS7	rs4866	7	2289586	A	G	4.47E-03	PRS-CSx
Colorectum	PRS7	rs4866566	5	3429364	C	T	-9.35E-04	PRS-CSx
Colorectum	PRS7	rs4870985	8	127855936	C	T	1.38E-03	PRS-CSx
Colorectum	PRS7	rs4871022	8	128427720	C	T	2.33E-03	PRS-CSx
Colorectum	PRS7	rs4871789	8	128428061	A	G	3.44E-03	PRS-CSx
Colorectum	PRS7	rs4871790	8	128441535	A	C	5.19E-04	PRS-CSx

Colorectum	PRS7	rs4876664	8	117600262	T	C	1.07E-05	PRS-CSx
Colorectum	PRS7	rs4876679	8	117763873	G	A	7.87E-04	PRS-CSx
Colorectum	PRS7	rs4877421	9	90838549	A	G	-1.56E-03	PRS-CSx
Colorectum	PRS7	rs4877928	9	88018061	A	G	1.16E-03	PRS-CSx
Colorectum	PRS7	rs4880142	9	139660402	T	C	5.93E-04	PRS-CSx
Colorectum	PRS7	rs4880158	9	139804679	T	C	-2.73E-04	PRS-CSx
Colorectum	PRS7	rs4880160	9	139808639	T	C	-2.12E-04	PRS-CSx
Colorectum	PRS7	rs4880162	9	139810595	C	T	-1.60E-04	PRS-CSx
Colorectum	PRS7	rs4880163	9	139810652	G	A	-8.19E-05	PRS-CSx
Colorectum	PRS7	rs4880168	9	139829109	A	G	-2.50E-04	PRS-CSx
Colorectum	PRS7	rs4880172	9	139844151	C	A	-2.63E-04	PRS-CSx
Colorectum	PRS7	rs4883888	13	71780111	T	C	3.73E-04	PRS-CSx
Colorectum	PRS7	rs4883940	13	73985283	T	C	-1.47E-03	PRS-CSx
Colorectum	PRS7	rs4885049	13	73453581	G	A	-1.11E-04	PRS-CSx
Colorectum	PRS7	rs4885062	13	73645996	G	T	-3.29E-03	PRS-CSx
Colorectum	PRS7	rs4885099	13	73973132	C	T	-4.13E-04	PRS-CSx
Colorectum	PRS7	rs4886018	13	52990717	C	T	1.59E-04	PRS-CSx
Colorectum	PRS7	rs4886443	15	75732227	T	C	3.28E-04	PRS-CSx
Colorectum	PRS7	rs4886595	15	74663265	C	A	1.10E-04	PRS-CSx
Colorectum	PRS7	rs4886615	15	75131661	A	G	2.02E-04	PRS-CSx
Colorectum	PRS7	rs4886670	15	75449674	T	G	-2.79E-04	PRS-CSx
Colorectum	PRS7	rs4886699	15	75692303	C	A	9.85E-05	PRS-CSx
Colorectum	PRS7	rs4886703	15	75730366	T	C	8.41E-05	PRS-CSx
Colorectum	PRS7	rs4886707	15	75755467	T	C	2.21E-04	PRS-CSx
Colorectum	PRS7	rs4886708	15	75762608	T	C	8.64E-05	PRS-CSx
Colorectum	PRS7	rs4886722	15	75921636	A	C	2.22E-04	PRS-CSx
Colorectum	PRS7	rs4886747	15	76151081	G	A	-4.51E-04	PRS-CSx
Colorectum	PRS7	rs4886748	15	76175128	A	C	2.29E-04	PRS-CSx
Colorectum	PRS7	rs4886753	15	76283942	A	G	-4.89E-04	PRS-CSx
Colorectum	PRS7	rs4886874	15	77838086	A	C	-4.29E-04	PRS-CSx
Colorectum	PRS7	rs4886886	15	77889192	G	A	-2.24E-04	PRS-CSx
Colorectum	PRS7	rs4887810	16	75277480	C	A	-3.08E-04	PRS-CSx
Colorectum	PRS7	rs4888058	16	79979392	A	C	2.07E-04	PRS-CSx
Colorectum	PRS7	rs4888064	16	80032531	C	T	-6.97E-04	PRS-CSx
Colorectum	PRS7	rs4888432	16	75510973	A	C	1.92E-04	PRS-CSx
Colorectum	PRS7	rs4888497	16	76440835	G	A	4.26E-04	PRS-CSx
Colorectum	PRS7	rs4889058	16	79993507	T	G	-2.13E-04	PRS-CSx
Colorectum	PRS7	rs4889060	16	80003522	G	A	-3.56E-04	PRS-CSx
Colorectum	PRS7	rs4889066	16	80050270	T	C	-6.41E-04	PRS-CSx
Colorectum	PRS7	rs4889068	16	80060221	T	C	2.78E-04	PRS-CSx
Colorectum	PRS7	rs4889091	16	80103798	C	T	1.47E-05	PRS-CSx
Colorectum	PRS7	rs4889630	16	30877544	T	C	1.88E-04	PRS-CSx
Colorectum	PRS7	rs4890115	17	38640605	T	C	3.88E-04	PRS-CSx
Colorectum	PRS7	rs4890423	18	40646176	G	A	-4.15E-04	PRS-CSx
Colorectum	PRS7	rs4890477	18	42003466	A	G	1.74E-04	PRS-CSx
Colorectum	PRS7	rs4891295	18	73278547	A	G	3.78E-04	PRS-CSx
Colorectum	PRS7	rs4891371	18	63888221	G	T	-3.81E-04	PRS-CSx
Colorectum	PRS7	rs4895441	6	135426573	G	A	7.29E-04	PRS-CSx
Colorectum	PRS7	rs4897198	6	127289089	C	A	1.70E-04	PRS-CSx
Colorectum	PRS7	rs489724	11	78948010	G	A	-8.58E-04	PRS-CSx
Colorectum	PRS7	rs4897506	6	131611309	C	T	8.97E-04	PRS-CSx
Colorectum	PRS7	rs4901065	14	51371121	T	C	9.05E-04	PRS-CSx
Colorectum	PRS7	rs4901066	14	51381286	T	C	-2.34E-04	PRS-CSx
Colorectum	PRS7	rs4901072	14	51502651	A	G	7.78E-04	PRS-CSx
Colorectum	PRS7	rs4901374	14	53707828	T	C	6.02E-04	PRS-CSx
Colorectum	PRS7	rs4901739	14	57345857	C	T	-1.03E-03	PRS-CSx

Colorectum	PRS7	rs4901852	14	58821929	A	G	1.60E-04	PRS-CSx
Colorectum	PRS7	rs4901878	14	59445296	A	G	1.39E-03	PRS-CSx
Colorectum	PRS7	rs4902175	14	63168547	G	A	1.09E-03	PRS-CSx
Colorectum	PRS7	rs490262	11	117222592	A	G	3.42E-04	PRS-CSx
Colorectum	PRS7	rs4906619	15	25946781	G	T	-1.25E-03	PRS-CSx
Colorectum	PRS7	rs4906750	15	25946584	C	T	-9.01E-04	PRS-CSx
Colorectum	PRS7	rs4908563	1	6623499	T	C	7.35E-05	PRS-CSx
Colorectum	PRS7	rs4910832	11	5669228	T	C	7.22E-04	PRS-CSx
Colorectum	PRS7	rs4911153	20	32961547	C	T	-1.31E-04	PRS-CSx
Colorectum	PRS7	rs4911418	20	32994257	G	A	-2.27E-04	PRS-CSx
Colorectum	PRS7	rs4911420	20	32998654	T	C	-1.89E-04	PRS-CSx
Colorectum	PRS7	rs4911421	20	32998948	T	C	-1.99E-04	PRS-CSx
Colorectum	PRS7	rs4911422	20	32999345	T	C	-1.19E-04	PRS-CSx
Colorectum	PRS7	rs4912402	1	59973874	A	C	-1.12E-03	PRS-CSx
Colorectum	PRS7	rs4912485	3	184085058	T	C	1.01E-03	PRS-CSx
Colorectum	PRS7	rs4912622	5	141490587	G	A	5.58E-04	PRS-CSx
Colorectum	PRS7	rs4912969	5	143683902	A	G	-2.22E-04	PRS-CSx
Colorectum	PRS7	rs4915723	1	61433136	C	A	6.52E-04	PRS-CSx
Colorectum	PRS7	rs4915953	1	64699594	G	T	7.19E-04	PRS-CSx
Colorectum	PRS7	rs4915962	1	64751610	A	G	4.95E-04	PRS-CSx
Colorectum	PRS7	rs4916005	1	65309387	C	T	-3.85E-04	PRS-CSx
Colorectum	PRS7	rs4916669	5	88393124	C	T	3.30E-04	PRS-CSx
Colorectum	PRS7	rs4916935	7	197267	G	A	5.53E-04	PRS-CSx
Colorectum	PRS7	rs4921106	5	159445829	G	A	5.39E-04	PRS-CSx
Colorectum	PRS7	rs4921249	5	159445881	C	T	4.78E-04	PRS-CSx
Colorectum	PRS7	rs4922155	8	20153776	T	C	9.13E-04	PRS-CSx
Colorectum	PRS7	rs4922536	10	48548377	T	G	8.76E-04	PRS-CSx
Colorectum	PRS7	rs4923720	15	36909267	A	G	7.31E-04	PRS-CSx
Colorectum	PRS7	rs4926443	1	246942107	A	G	6.40E-04	PRS-CSx
Colorectum	PRS7	rs4926444	1	246942171	A	G	6.93E-04	PRS-CSx
Colorectum	PRS7	rs4926456	1	246941990	G	A	9.41E-04	PRS-CSx
Colorectum	PRS7	rs4927176	1	55354335	T	C	-8.61E-05	PRS-CSx
Colorectum	PRS7	rs492887	9	257973	A	G	-3.79E-04	PRS-CSx
Colorectum	PRS7	rs492934	9	257985	A	G	-3.70E-04	PRS-CSx
Colorectum	PRS7	rs493248	13	37473386	T	C	3.94E-03	PRS-CSx
Colorectum	PRS7	rs4932768	19	22729318	A	C	9.59E-04	PRS-CSx
Colorectum	PRS7	rs4933198	10	82665743	T	G	-8.66E-04	PRS-CSx
Colorectum	PRS7	rs4933449	10	89386442	T	G	8.19E-04	PRS-CSx
Colorectum	PRS7	rs4934439	10	90831991	G	A	8.64E-04	PRS-CSx
Colorectum	PRS7	rs493516	11	117213160	A	G	5.33E-04	PRS-CSx
Colorectum	PRS7	rs493768	6	69549897	C	T	-1.66E-04	PRS-CSx
Colorectum	PRS7	rs4938799	11	120208257	G	T	-1.27E-04	PRS-CSx
Colorectum	PRS7	rs4938801	11	120223399	T	C	-2.40E-04	PRS-CSx
Colorectum	PRS7	rs4938802	11	120228989	G	A	-1.61E-04	PRS-CSx
Colorectum	PRS7	rs4938804	11	120273865	A	G	-5.65E-05	PRS-CSx
Colorectum	PRS7	rs4938807	11	120331701	A	C	-3.45E-04	PRS-CSx
Colorectum	PRS7	rs4938808	11	120336902	T	G	-1.49E-04	PRS-CSx
Colorectum	PRS7	rs4939827	18	46453463	T	C	1.26E-02	PRS-CSx
Colorectum	PRS7	rs4941104	18	59729644	C	T	-6.59E-05	PRS-CSx
Colorectum	PRS7	rs4942023	13	41675611	A	G	-5.11E-04	PRS-CSx
Colorectum	PRS7	rs4942417	13	32887389	C	A	-9.79E-04	PRS-CSx
Colorectum	PRS7	rs4943076	13	33689996	C	T	7.28E-04	PRS-CSx
Colorectum	PRS7	rs4943112	13	34072198	T	C	-7.55E-04	PRS-CSx
Colorectum	PRS7	rs4943114	13	34083781	C	T	-7.78E-04	PRS-CSx
Colorectum	PRS7	rs4944558	11	85829867	C	T	5.20E-04	PRS-CSx
Colorectum	PRS7	rs4944925	11	74359529	A	G	-5.15E-04	PRS-CSx

Colorectum	PRS7	rs4945871	6	111378649	C	A	-3.99E-04	PRS-CSx
Colorectum	PRS7	rs4945872	6	111378759	C	T	-3.44E-04	PRS-CSx
Colorectum	PRS7	rs494615	19	56606584	A	G	1.10E-03	PRS-CSx
Colorectum	PRS7	rs4946259	6	117816093	A	G	-5.91E-04	PRS-CSx
Colorectum	PRS7	rs4946260	6	117822993	T	C	8.17E-04	PRS-CSx
Colorectum	PRS7	rs4946705	6	106348246	A	G	7.38E-04	PRS-CSx
Colorectum	PRS7	rs4947099	6	111378729	G	A	-3.29E-04	PRS-CSx
Colorectum	PRS7	rs4949624	1	76732617	G	A	1.92E-03	PRS-CSx
Colorectum	PRS7	rs4950964	1	204806479	G	T	2.78E-04	PRS-CSx
Colorectum	PRS7	rs4951140	1	204823161	A	G	2.94E-04	PRS-CSx
Colorectum	PRS7	rs4951448	1	212635416	G	A	5.65E-04	PRS-CSx
Colorectum	PRS7	rs4951458	1	212879644	C	T	2.41E-04	PRS-CSx
Colorectum	PRS7	rs4952913	2	48678659	T	C	2.74E-05	PRS-CSx
Colorectum	PRS7	rs4953572	2	48624007	A	G	-5.19E-03	PRS-CSx
Colorectum	PRS7	rs4953588	2	48738042	T	C	5.31E-05	PRS-CSx
Colorectum	PRS7	rs4953589	2	48740195	G	A	-3.03E-04	PRS-CSx
Colorectum	PRS7	rs4953590	2	48745405	A	C	9.73E-05	PRS-CSx
Colorectum	PRS7	rs4953664	2	43024676	C	A	3.92E-04	PRS-CSx
Colorectum	PRS7	rs4953672	2	43100438	C	A	-1.66E-04	PRS-CSx
Colorectum	PRS7	rs4953702	2	43208328	C	T	-4.66E-04	PRS-CSx
Colorectum	PRS7	rs495406	5	134444120	G	T	2.14E-03	PRS-CSx
Colorectum	PRS7	rs495418	1	183159664	A	G	9.70E-05	PRS-CSx
Colorectum	PRS7	rs4957213	5	39656759	T	C	1.06E-04	PRS-CSx
Colorectum	PRS7	rs4957217	5	39676277	T	C	4.75E-04	PRS-CSx
Colorectum	PRS7	rs4957233	5	40049919	C	T	-2.96E-04	PRS-CSx
Colorectum	PRS7	rs4957238	5	40062150	A	C	-1.25E-04	PRS-CSx
Colorectum	PRS7	rs4957254	5	40159642	A	G	-1.31E-04	PRS-CSx
Colorectum	PRS7	rs495828	9	136154867	T	G	2.10E-04	PRS-CSx
Colorectum	PRS7	rs4958427	5	150278587	C	T	4.95E-05	PRS-CSx
Colorectum	PRS7	rs4958847	5	150239587	G	A	1.65E-04	PRS-CSx
Colorectum	PRS7	rs4958852	5	150330631	T	C	2.50E-05	PRS-CSx
Colorectum	PRS7	rs4959079	6	31488879	T	C	8.32E-04	PRS-CSx
Colorectum	PRS7	rs4960332	6	7618662	A	G	-9.12E-04	PRS-CSx
Colorectum	PRS7	rs4960621	7	154616612	A	G	2.23E-03	PRS-CSx
Colorectum	PRS7	rs4961257	8	142232704	A	G	6.05E-04	PRS-CSx
Colorectum	PRS7	rs4961341	8	142235154	A	C	1.01E-03	PRS-CSx
Colorectum	PRS7	rs4963243	11	61494327	A	G	6.65E-05	PRS-CSx
Colorectum	PRS7	rs4963308	11	61456426	A	G	1.18E-04	PRS-CSx
Colorectum	PRS7	rs4964541	12	107639259	A	G	-4.31E-04	PRS-CSx
Colorectum	PRS7	rs4965260	15	100157136	T	C	-6.45E-04	PRS-CSx
Colorectum	PRS7	rs4965446	15	99667686	T	C	-8.26E-04	PRS-CSx
Colorectum	PRS7	rs4965873	15	102004023	C	T	3.86E-04	PRS-CSx
Colorectum	PRS7	rs4968127	17	809643	G	A	1.81E-03	PRS-CSx
Colorectum	PRS7	rs4968427	17	59220844	T	C	-1.01E-04	PRS-CSx
Colorectum	PRS7	rs4968428	17	59248829	T	C	-1.32E-04	PRS-CSx
Colorectum	PRS7	rs4968535	17	59245274	T	C	-5.93E-05	PRS-CSx
Colorectum	PRS7	rs4968656	17	61616959	G	A	-4.49E-04	PRS-CSx
Colorectum	PRS7	rs496892	9	22024351	T	C	3.25E-04	PRS-CSx
Colorectum	PRS7	rs4970432	1	1254136	G	A	6.75E-04	PRS-CSx
Colorectum	PRS7	rs4970827	1	109748547	C	T	1.07E-03	PRS-CSx
Colorectum	PRS7	rs497123	8	102450266	C	A	1.12E-03	PRS-CSx
Colorectum	PRS7	rs497200	9	97289548	A	G	-5.71E-04	PRS-CSx
Colorectum	PRS7	rs4972399	2	174091200	G	A	-8.85E-04	PRS-CSx
Colorectum	PRS7	rs497240	6	69542291	C	T	-4.38E-04	PRS-CSx
Colorectum	PRS7	rs4974986	4	39166928	C	T	1.26E-04	PRS-CSx
Colorectum	PRS7	rs4975009	4	39361551	C	T	5.36E-05	PRS-CSx

Colorectum	PRS7	rs4975612	5	1300310	G	T	-3.72E-03	PRS-CSx
Colorectum	PRS7	rs4975681	5	1638716	T	C	6.41E-04	PRS-CSx
Colorectum	PRS7	rs4976242	5	134390581	C	A	-1.53E-04	PRS-CSx
Colorectum	PRS7	rs4976270	5	134467220	C	T	1.85E-03	PRS-CSx
Colorectum	PRS7	rs4976593	5	167777443	G	A	7.88E-04	PRS-CSx
Colorectum	PRS7	rs4976741	5	177591495	G	T	-1.15E-03	PRS-CSx
Colorectum	PRS7	rs4977574	9	22098574	A	G	7.36E-04	PRS-CSx
Colorectum	PRS7	rs4977756	9	22068652	G	A	2.12E-04	PRS-CSx
Colorectum	PRS7	rs4978964	9	113633647	A	G	4.73E-04	PRS-CSx
Colorectum	PRS7	rs497916	11	118758089	T	C	4.58E-03	PRS-CSx
Colorectum	PRS7	rs4979843	10	80847236	C	T	1.09E-03	PRS-CSx
Colorectum	PRS7	rs4980022	10	80834685	T	C	2.66E-03	PRS-CSx
Colorectum	PRS7	rs4980024	10	80839269	A	G	1.91E-03	PRS-CSx
Colorectum	PRS7	rs4982061	14	33681031	G	A	-2.32E-03	PRS-CSx
Colorectum	PRS7	rs4983579	14	105555396	A	G	5.40E-04	PRS-CSx
Colorectum	PRS7	rs4984265	15	63627439	G	A	5.19E-04	PRS-CSx
Colorectum	PRS7	rs4985692	17	5691801	G	A	2.50E-04	PRS-CSx
Colorectum	PRS7	rs4985752	17	17120812	T	C	4.29E-04	PRS-CSx
Colorectum	PRS7	rs4985761	17	17179481	G	A	3.26E-04	PRS-CSx
Colorectum	PRS7	rs498612	11	72907686	C	A	-6.32E-04	PRS-CSx
Colorectum	PRS7	rs4986221	18	44933214	G	A	-2.52E-03	PRS-CSx
Colorectum	PRS7	rs499691	6	32194339	C	T	-4.50E-04	PRS-CSx
Colorectum	PRS7	rs499932	5	39882232	C	A	-2.61E-04	PRS-CSx
Colorectum	PRS7	rs500037	12	118139540	T	G	1.06E-03	PRS-CSx
Colorectum	PRS7	rs5003265	6	29344036	T	C	-2.36E-04	PRS-CSx
Colorectum	PRS7	rs500745	11	29990325	C	A	7.18E-04	PRS-CSx
Colorectum	PRS7	rs500830	13	72348768	C	T	4.51E-04	PRS-CSx
Colorectum	PRS7	rs501054	11	57561106	G	T	4.24E-04	PRS-CSx
Colorectum	PRS7	rs5011832	10	21782842	C	T	7.71E-04	PRS-CSx
Colorectum	PRS7	rs5012181	4	175376219	A	G	5.46E-04	PRS-CSx
Colorectum	PRS7	rs501426	1	76721164	T	G	-5.73E-04	PRS-CSx
Colorectum	PRS7	rs501776	11	117258198	C	T	-1.43E-04	PRS-CSx
Colorectum	PRS7	rs501941	5	67298770	A	G	5.00E-05	PRS-CSx
Colorectum	PRS7	rs5020305	7	6463051	A	C	1.97E-03	PRS-CSx
Colorectum	PRS7	rs502211	17	4929640	C	A	-3.15E-04	PRS-CSx
Colorectum	PRS7	rs5022242	1	12671703	T	C	-8.68E-04	PRS-CSx
Colorectum	PRS7	rs502363	11	65775592	G	A	-3.08E-04	PRS-CSx
Colorectum	PRS7	rs5025315	6	31343604	A	G	-2.71E-04	PRS-CSx
Colorectum	PRS7	rs502708	10	78979738	C	T	2.25E-04	PRS-CSx
Colorectum	PRS7	rs5028175	3	112829890	A	G	-8.06E-04	PRS-CSx
Colorectum	PRS7	rs503037	11	57436530	C	T	2.02E-04	PRS-CSx
Colorectum	PRS7	rs5030416	11	36532488	G	T	1.46E-03	PRS-CSx
Colorectum	PRS7	rs503473	11	120355230	A	G	-1.62E-04	PRS-CSx
Colorectum	PRS7	rs506444	11	34854295	T	C	-1.43E-03	PRS-CSx
Colorectum	PRS7	rs506498	9	138590138	T	C	1.38E-03	PRS-CSx
Colorectum	PRS7	rs506873	11	65767215	G	T	-1.86E-04	PRS-CSx
Colorectum	PRS7	rs507672	11	65767153	A	G	-1.49E-04	PRS-CSx
Colorectum	PRS7	rs509360	11	61548559	A	G	2.84E-03	PRS-CSx
Colorectum	PRS7	rs509863	4	13969909	C	T	-5.06E-04	PRS-CSx
Colorectum	PRS7	rs510321	6	32194392	A	C	-1.71E-04	PRS-CSx
Colorectum	PRS7	rs512819	11	17894411	G	A	6.03E-04	PRS-CSx
Colorectum	PRS7	rs513041	6	53447934	T	C	-1.96E-04	PRS-CSx
Colorectum	PRS7	rs514518	6	69541114	A	C	-2.94E-04	PRS-CSx
Colorectum	PRS7	rs515746	12	115118997	A	G	-3.57E-04	PRS-CSx
Colorectum	PRS7	rs516022	19	56609371	T	G	1.43E-03	PRS-CSx
Colorectum	PRS7	rs516187	9	113631618	A	C	3.87E-04	PRS-CSx

Colorectum	PRS7	rs516473	6	131443843	C	A	2.44E-04	PRS-CSx
Colorectum	PRS7	rs517242	9	113631766	A	G	4.13E-04	PRS-CSx
Colorectum	PRS7	rs518558	6	70980388	A	G	6.62E-04	PRS-CSx
Colorectum	PRS7	rs519402	9	258561	A	G	-3.71E-04	PRS-CSx
Colorectum	PRS7	rs522468	1	36653677	T	G	-1.09E-03	PRS-CSx
Colorectum	PRS7	rs523096	9	22019129	G	A	3.93E-04	PRS-CSx
Colorectum	PRS7	rs523615	7	16641749	A	C	-2.08E-03	PRS-CSx
Colorectum	PRS7	rs5242	11	14988998	A	G	1.87E-04	PRS-CSx
Colorectum	PRS7	rs524596	9	113627633	C	T	-2.83E-04	PRS-CSx
Colorectum	PRS7	rs524673	13	34002488	C	T	-6.32E-04	PRS-CSx
Colorectum	PRS7	rs524675	4	13966851	A	G	-3.80E-04	PRS-CSx
Colorectum	PRS7	rs525088	9	259224	G	T	-3.41E-04	PRS-CSx
Colorectum	PRS7	rs525374	18	34308481	T	C	4.48E-04	PRS-CSx
Colorectum	PRS7	rs526509	9	268740	G	T	-3.21E-04	PRS-CSx
Colorectum	PRS7	rs527140	11	57500134	A	G	2.71E-04	PRS-CSx
Colorectum	PRS7	rs527162	11	85715736	C	T	-1.03E-04	PRS-CSx
Colorectum	PRS7	rs527386	1	201629249	C	T	-1.22E-03	PRS-CSx
Colorectum	PRS7	rs527717	11	114326834	A	G	6.11E-05	PRS-CSx
Colorectum	PRS7	rs527929	13	37478506	T	C	2.36E-03	PRS-CSx
Colorectum	PRS7	rs529045	9	217397	G	A	-2.93E-04	PRS-CSx
Colorectum	PRS7	rs529976	9	138590347	C	T	2.11E-03	PRS-CSx
Colorectum	PRS7	rs530094	11	57531037	G	A	2.90E-04	PRS-CSx
Colorectum	PRS7	rs530225	1	38264819	G	T	-1.11E-04	PRS-CSx
Colorectum	PRS7	rs531830	13	33699406	G	A	-3.23E-04	PRS-CSx
Colorectum	PRS7	rs532347	6	18476660	A	G	2.51E-03	PRS-CSx
Colorectum	PRS7	rs532701	9	272270	C	T	-2.09E-04	PRS-CSx
Colorectum	PRS7	rs535286	12	101340157	A	G	-2.06E-04	PRS-CSx
Colorectum	PRS7	rs535550	9	245752	A	G	-3.22E-04	PRS-CSx
Colorectum	PRS7	rs535932	4	13988911	A	C	-8.22E-04	PRS-CSx
Colorectum	PRS7	rs536841	11	85787824	T	C	1.23E-04	PRS-CSx
Colorectum	PRS7	rs5370	6	12296255	T	G	-7.52E-04	PRS-CSx
Colorectum	PRS7	rs537323	11	117226849	T	C	3.46E-04	PRS-CSx
Colorectum	PRS7	rs537786	11	65494987	C	T	-1.19E-04	PRS-CSx
Colorectum	PRS7	rs539709	12	115119314	A	G	3.46E-04	PRS-CSx
Colorectum	PRS7	rs540909	9	273160	T	C	-6.84E-04	PRS-CSx
Colorectum	PRS7	rs540917	5	134456005	A	G	7.32E-04	PRS-CSx
Colorectum	PRS7	rs541245	11	57545948	A	G	6.16E-04	PRS-CSx
Colorectum	PRS7	rs541458	11	85788351	T	C	2.71E-04	PRS-CSx
Colorectum	PRS7	rs543969	11	125982079	C	A	7.39E-04	PRS-CSx
Colorectum	PRS7	rs544099	6	122800982	G	T	6.82E-04	PRS-CSx
Colorectum	PRS7	rs544169	9	33956791	G	A	-3.59E-04	PRS-CSx
Colorectum	PRS7	rs544446	11	57470844	T	C	3.15E-04	PRS-CSx
Colorectum	PRS7	rs545076	12	115119903	A	G	-4.95E-04	PRS-CSx
Colorectum	PRS7	rs546614	11	43983973	A	C	-6.19E-04	PRS-CSx
Colorectum	PRS7	rs547905	1	183157924	G	A	-1.30E-04	PRS-CSx
Colorectum	PRS7	rs549022	11	57456244	C	T	1.90E-04	PRS-CSx
Colorectum	PRS7	rs549844	1	183158143	G	A	3.89E-05	PRS-CSx
Colorectum	PRS7	rs550238	1	218690948	A	G	-4.52E-04	PRS-CSx
Colorectum	PRS7	rs551517	9	113636793	T	C	6.31E-04	PRS-CSx
Colorectum	PRS7	rs551876	6	69545429	A	G	-2.56E-04	PRS-CSx
Colorectum	PRS7	rs552498	11	117260075	G	A	-1.45E-04	PRS-CSx
Colorectum	PRS7	rs552848	9	238389	G	A	-2.36E-04	PRS-CSx
Colorectum	PRS7	rs553219	9	113612463	T	C	3.41E-04	PRS-CSx
Colorectum	PRS7	rs555213	1	58726579	A	G	-6.48E-04	PRS-CSx
Colorectum	PRS7	rs555292	13	34015817	C	T	-3.18E-04	PRS-CSx
Colorectum	PRS7	rs555588	6	69545855	C	T	-3.19E-04	PRS-CSx

Colorectum	PRS7	rs556439	6	122745009	A	G	3.66E-04	PRS-CSx
Colorectum	PRS7	rs556709	13	57713178	T	C	2.73E-04	PRS-CSx
Colorectum	PRS7	rs559422	11	248181	C	A	-5.62E-03	PRS-CSx
Colorectum	PRS7	rs560239	12	31771689	A	G	-3.61E-04	PRS-CSx
Colorectum	PRS7	rs560495	9	138588154	A	C	1.68E-03	PRS-CSx
Colorectum	PRS7	rs560630	10	107707902	T	C	-7.13E-05	PRS-CSx
Colorectum	PRS7	rs560708	18	4373198	A	G	5.20E-04	PRS-CSx
Colorectum	PRS7	rs561	19	8376431	A	G	5.15E-04	PRS-CSx
Colorectum	PRS7	rs561655	11	85800279	G	A	-3.26E-04	PRS-CSx
Colorectum	PRS7	rs562638	19	39849789	G	A	4.26E-04	PRS-CSx
Colorectum	PRS7	rs5629	20	48129706	T	G	-5.48E-07	PRS-CSx
Colorectum	PRS7	rs564001	11	78963697	G	A	-8.36E-04	PRS-CSx
Colorectum	PRS7	rs564398	9	22029547	C	T	4.22E-04	PRS-CSx
Colorectum	PRS7	rs565077	21	43356277	T	C	1.41E-03	PRS-CSx
Colorectum	PRS7	rs566625	1	183161441	A	G	-7.26E-05	PRS-CSx
Colorectum	PRS7	rs566750	5	134485285	G	A	2.14E-03	PRS-CSx
Colorectum	PRS7	rs569991	9	113626521	T	C	3.85E-04	PRS-CSx
Colorectum	PRS7	rs571299	6	69540950	G	A	-2.75E-04	PRS-CSx
Colorectum	PRS7	rs571594	11	111359334	G	A	-2.52E-04	PRS-CSx
Colorectum	PRS7	rs571646	5	72367339	A	G	-3.69E-04	PRS-CSx
Colorectum	PRS7	rs573091	9	256995	A	G	-2.59E-04	PRS-CSx
Colorectum	PRS7	rs573687	9	22011642	A	G	4.06E-04	PRS-CSx
Colorectum	PRS7	rs573709	6	122778818	T	C	4.76E-04	PRS-CSx
Colorectum	PRS7	rs573744	1	38252636	G	A	6.95E-05	PRS-CSx
Colorectum	PRS7	rs573827	11	117214091	A	G	4.37E-04	PRS-CSx
Colorectum	PRS7	rs573851	9	266505	A	C	-3.85E-04	PRS-CSx
Colorectum	PRS7	rs574654	5	134454689	A	G	5.82E-04	PRS-CSx
Colorectum	PRS7	rs5750734	22	39449503	G	T	-2.72E-04	PRS-CSx
Colorectum	PRS7	rs5751341	22	43097551	C	T	3.87E-04	PRS-CSx
Colorectum	PRS7	rs5751457	22	43655427	A	G	1.02E-03	PRS-CSx
Colorectum	PRS7	rs5751458	22	43655526	A	G	1.45E-03	PRS-CSx
Colorectum	PRS7	rs5751749	22	24184850	G	A	9.39E-04	PRS-CSx
Colorectum	PRS7	rs5752764	22	29034964	C	T	3.91E-04	PRS-CSx
Colorectum	PRS7	rs5752793	22	29160314	A	G	4.55E-04	PRS-CSx
Colorectum	PRS7	rs5754217	22	21939675	G	T	3.33E-04	PRS-CSx
Colorectum	PRS7	rs575442	9	15283761	C	T	-5.61E-04	PRS-CSx
Colorectum	PRS7	rs5754467	22	21985094	A	G	2.93E-04	PRS-CSx
Colorectum	PRS7	rs575668	5	39906765	C	T	-3.39E-04	PRS-CSx
Colorectum	PRS7	rs5757037	22	38719189	A	G	9.11E-04	PRS-CSx
Colorectum	PRS7	rs5758837	22	43049014	T	G	4.17E-04	PRS-CSx
Colorectum	PRS7	rs5758873	22	43097514	A	G	1.79E-04	PRS-CSx
Colorectum	PRS7	rs5759066	22	43366296	G	A	5.24E-05	PRS-CSx
Colorectum	PRS7	rs5759070	22	43375701	G	A	1.03E-04	PRS-CSx
Colorectum	PRS7	rs5759093	22	43409989	G	A	2.59E-05	PRS-CSx
Colorectum	PRS7	rs5759111	22	43442599	T	C	2.71E-04	PRS-CSx
Colorectum	PRS7	rs5759116	22	43445572	T	C	-6.40E-04	PRS-CSx
Colorectum	PRS7	rs5759120	22	43446888	T	C	-5.76E-04	PRS-CSx
Colorectum	PRS7	rs5759129	22	43459026	C	T	1.39E-04	PRS-CSx
Colorectum	PRS7	rs5759165	22	43494072	C	T	6.79E-04	PRS-CSx
Colorectum	PRS7	rs5759313	22	43784381	T	C	-8.19E-04	PRS-CSx
Colorectum	PRS7	rs5762862	22	29252732	G	A	8.50E-05	PRS-CSx
Colorectum	PRS7	rs5762931	22	29379731	A	C	2.26E-03	PRS-CSx
Colorectum	PRS7	rs5764725	22	45823327	C	T	-1.83E-04	PRS-CSx
Colorectum	PRS7	rs576515	9	113625888	C	A	-3.15E-04	PRS-CSx
Colorectum	PRS7	rs5765356	22	45828033	A	G	-2.08E-04	PRS-CSx
Colorectum	PRS7	rs5765370	22	45838156	A	G	4.93E-04	PRS-CSx

Colorectum	PRS7	rs5765377	22	45853566	G	A	-1.08E-03	PRS-CSx
Colorectum	PRS7	rs576559	9	113642646	T	C	-5.44E-04	PRS-CSx
Colorectum	PRS7	rs5771717	22	49081164	A	G	-1.29E-03	PRS-CSx
Colorectum	PRS7	rs577328	6	69541331	G	A	-2.23E-04	PRS-CSx
Colorectum	PRS7	rs579459	9	136154168	C	T	1.34E-04	PRS-CSx
Colorectum	PRS7	rs580971	19	34603466	A	G	-9.08E-04	PRS-CSx
Colorectum	PRS7	rs581015	11	114349840	C	T	-5.04E-05	PRS-CSx
Colorectum	PRS7	rs582008	6	4104567	A	G	8.51E-04	PRS-CSx
Colorectum	PRS7	rs582147	12	101332838	G	A	-1.61E-04	PRS-CSx
Colorectum	PRS7	rs583058	1	212610755	T	C	-3.38E-04	PRS-CSx
Colorectum	PRS7	rs583301	7	42947886	C	A	-4.81E-04	PRS-CSx
Colorectum	PRS7	rs583881	6	4110734	T	C	3.10E-04	PRS-CSx
Colorectum	PRS7	rs584324	11	93514237	A	G	-8.04E-04	PRS-CSx
Colorectum	PRS7	rs585768	11	93552855	T	C	-7.86E-04	PRS-CSx
Colorectum	PRS7	rs585849	11	117069061	G	A	-3.29E-04	PRS-CSx
Colorectum	PRS7	rs586166	6	148200667	A	G	3.78E-04	PRS-CSx
Colorectum	PRS7	rs586994	9	264347	C	A	-4.32E-04	PRS-CSx
Colorectum	PRS7	rs587847	15	37660049	C	A	-4.29E-04	PRS-CSx
Colorectum	PRS7	rs5896	11	46745003	C	T	2.09E-04	PRS-CSx
Colorectum	PRS7	rs589627	9	224976	T	G	-1.61E-04	PRS-CSx
Colorectum	PRS7	rs590246	7	42968260	G	A	-1.87E-04	PRS-CSx
Colorectum	PRS7	rs591875	7	42960618	C	A	-2.97E-04	PRS-CSx
Colorectum	PRS7	rs592297	11	85725937	C	T	-8.50E-05	PRS-CSx
Colorectum	PRS7	rs592314	11	85673124	A	G	-2.06E-04	PRS-CSx
Colorectum	PRS7	rs592698	6	158889652	G	A	3.41E-04	PRS-CSx
Colorectum	PRS7	rs592779	3	101584944	T	C	9.10E-04	PRS-CSx
Colorectum	PRS7	rs593100	2	4471859	G	A	-7.89E-04	PRS-CSx
Colorectum	PRS7	rs593717	18	4366070	C	T	5.10E-04	PRS-CSx
Colorectum	PRS7	rs594361	11	86386784	G	A	2.73E-04	PRS-CSx
Colorectum	PRS7	rs59515	5	141901400	G	A	4.33E-04	PRS-CSx
Colorectum	PRS7	rs595367	11	117227694	C	T	5.01E-04	PRS-CSx
Colorectum	PRS7	rs595591	6	35896635	C	T	3.31E-04	PRS-CSx
Colorectum	PRS7	rs598405	6	117053071	A	G	-4.73E-04	PRS-CSx
Colorectum	PRS7	rs599329	6	158897794	T	C	1.67E-04	PRS-CSx
Colorectum	PRS7	rs5995843	22	40697377	G	A	-1.70E-04	PRS-CSx
Colorectum	PRS7	rs5998619	22	21945851	G	A	6.82E-05	PRS-CSx
Colorectum	PRS7	rs5998672	22	21966442	G	A	2.52E-04	PRS-CSx
Colorectum	PRS7	rs6001794	22	40529415	T	G	-7.84E-04	PRS-CSx
Colorectum	PRS7	rs6001848	22	40636799	G	A	-2.07E-04	PRS-CSx
Colorectum	PRS7	rs6001862	22	40681788	T	C	-2.04E-04	PRS-CSx
Colorectum	PRS7	rs6001877	22	40712637	A	G	-1.40E-04	PRS-CSx
Colorectum	PRS7	rs6002985	22	43333427	C	T	5.88E-05	PRS-CSx
Colorectum	PRS7	rs6003913	22	24186623	C	T	1.02E-03	PRS-CSx
Colorectum	PRS7	rs6003914	22	24186637	C	T	9.40E-04	PRS-CSx
Colorectum	PRS7	rs6005967	22	29446975	C	T	-1.03E-03	PRS-CSx
Colorectum	PRS7	rs6006743	22	45805523	G	A	-3.48E-04	PRS-CSx
Colorectum	PRS7	rs6007009	22	45781508	G	A	-6.46E-04	PRS-CSx
Colorectum	PRS7	rs6007030	22	45830359	T	G	-1.83E-04	PRS-CSx
Colorectum	PRS7	rs600951	9	224742	A	G	-1.77E-04	PRS-CSx
Colorectum	PRS7	rs601225	3	101580650	G	A	7.07E-04	PRS-CSx
Colorectum	PRS7	rs6012490	20	47216641	G	A	-1.13E-04	PRS-CSx
Colorectum	PRS7	rs6012519	20	47326000	A	G	-1.52E-03	PRS-CSx
Colorectum	PRS7	rs6013844	20	52565526	T	C	8.84E-04	PRS-CSx
Colorectum	PRS7	rs601389	9	97360148	T	C	-3.82E-04	PRS-CSx
Colorectum	PRS7	rs6014100	20	53257989	G	T	-1.20E-03	PRS-CSx
Colorectum	PRS7	rs6017240	20	42634362	C	A	-1.98E-04	PRS-CSx

Colorectum	PRS7	rs6017246	20	42652933	A	G	-2.57E-04	PRS-CSx
Colorectum	PRS7	rs601904	11	74200739	G	A	-1.64E-04	PRS-CSx
Colorectum	PRS7	rs6019348	20	47274137	A	G	-8.59E-04	PRS-CSx
Colorectum	PRS7	rs6019349	20	47275245	G	A	-1.81E-04	PRS-CSx
Colorectum	PRS7	rs6019360	20	47289756	T	C	-6.99E-04	PRS-CSx
Colorectum	PRS7	rs6019361	20	47290884	G	A	-9.25E-04	PRS-CSx
Colorectum	PRS7	rs6019380	20	47318845	G	A	-2.34E-04	PRS-CSx
Colorectum	PRS7	rs6019382	20	47320394	A	G	-1.21E-03	PRS-CSx
Colorectum	PRS7	rs6020487	20	49031318	T	G	6.71E-04	PRS-CSx
Colorectum	PRS7	rs6020488	20	49031332	T	C	4.50E-03	PRS-CSx
Colorectum	PRS7	rs602182	1	183279029	T	G	-2.63E-04	PRS-CSx
Colorectum	PRS7	rs6024549	20	54547988	T	C	4.02E-03	PRS-CSx
Colorectum	PRS7	rs6024978	20	55165067	C	A	4.49E-03	PRS-CSx
Colorectum	PRS7	rs602524	7	42941854	G	A	-3.03E-04	PRS-CSx
Colorectum	PRS7	rs6025552	20	55994630	C	T	-4.17E-04	PRS-CSx
Colorectum	PRS7	rs6026567	20	57444915	A	G	-5.99E-04	PRS-CSx
Colorectum	PRS7	rs6026584	20	57469073	T	C	-4.66E-04	PRS-CSx
Colorectum	PRS7	rs602922	11	65297600	T	C	-1.52E-03	PRS-CSx
Colorectum	PRS7	rs6030438	20	41353289	G	A	-2.24E-03	PRS-CSx
Colorectum	PRS7	rs6031224	20	42547539	T	C	3.29E-04	PRS-CSx
Colorectum	PRS7	rs6031225	20	42547830	C	A	5.59E-04	PRS-CSx
Colorectum	PRS7	rs6031294	20	42640613	T	C	-4.13E-04	PRS-CSx
Colorectum	PRS7	rs6031301	20	42654347	T	C	-9.61E-04	PRS-CSx
Colorectum	PRS7	rs6031755	20	43337155	C	T	-3.00E-04	PRS-CSx
Colorectum	PRS7	rs6035972	20	2240894	A	G	4.76E-04	PRS-CSx
Colorectum	PRS7	rs6035973	20	2240926	T	G	3.73E-04	PRS-CSx
Colorectum	PRS7	rs603834	7	42942126	C	T	-4.12E-04	PRS-CSx
Colorectum	PRS7	rs6038449	20	6337980	C	T	1.06E-04	PRS-CSx
Colorectum	PRS7	rs6038491	20	6410854	T	C	1.26E-03	PRS-CSx
Colorectum	PRS7	rs6038619	20	6826062	A	C	-1.89E-04	PRS-CSx
Colorectum	PRS7	rs6038955	20	7780034	A	G	7.63E-04	PRS-CSx
Colorectum	PRS7	rs6043707	20	16041628	C	T	-6.93E-04	PRS-CSx
Colorectum	PRS7	rs6044641	20	17110620	G	A	4.15E-04	PRS-CSx
Colorectum	PRS7	rs604470	9	223979	A	G	-1.51E-04	PRS-CSx
Colorectum	PRS7	rs6045912	20	1974008	G	A	-2.11E-04	PRS-CSx
Colorectum	PRS7	rs6046169	20	19524558	T	C	-6.00E-04	PRS-CSx
Colorectum	PRS7	rs6054092	20	6303803	A	G	-1.92E-03	PRS-CSx
Colorectum	PRS7	rs6054147	20	6336002	G	A	2.14E-04	PRS-CSx
Colorectum	PRS7	rs6054240	20	6421211	G	A	2.46E-04	PRS-CSx
Colorectum	PRS7	rs6054334	20	6518520	T	C	3.07E-04	PRS-CSx
Colorectum	PRS7	rs6054399	20	6599698	C	T	9.97E-04	PRS-CSx
Colorectum	PRS7	rs6054479	20	6689058	C	T	-2.84E-03	PRS-CSx
Colorectum	PRS7	rs6054545	20	6824496	C	T	-3.05E-04	PRS-CSx
Colorectum	PRS7	rs6054963	20	7370428	G	A	-1.07E-03	PRS-CSx
Colorectum	PRS7	rs6054965	20	7374248	C	T	-1.45E-03	PRS-CSx
Colorectum	PRS7	rs6055279	20	7706428	T	C	5.53E-04	PRS-CSx
Colorectum	PRS7	rs6055363	20	7863543	G	A	-7.24E-04	PRS-CSx
Colorectum	PRS7	rs6055570	20	8129324	A	C	4.95E-04	PRS-CSx
Colorectum	PRS7	rs6058051	20	33044862	A	G	-8.42E-05	PRS-CSx
Colorectum	PRS7	rs6058073	20	33116483	C	T	-1.14E-04	PRS-CSx
Colorectum	PRS7	rs6058322	20	34367277	G	A	7.74E-04	PRS-CSx
Colorectum	PRS7	rs6059827	20	33016358	A	G	-2.36E-04	PRS-CSx
Colorectum	PRS7	rs6059834	20	33019299	G	A	-1.34E-04	PRS-CSx
Colorectum	PRS7	rs605986	6	131431458	C	T	7.38E-04	PRS-CSx
Colorectum	PRS7	rs6059860	20	33063830	C	A	-1.99E-04	PRS-CSx
Colorectum	PRS7	rs6059866	20	33075810	G	T	-1.14E-04	PRS-CSx

Colorectum	PRS7	rs6059867	20	33078103	T	C	-1.61E-04	PRS-CSx
Colorectum	PRS7	rs6059868	20	33079460	G	A	-2.24E-04	PRS-CSx
Colorectum	PRS7	rs6059875	20	33086090	G	T	-5.23E-05	PRS-CSx
Colorectum	PRS7	rs6059880	20	33088669	A	G	-2.15E-04	PRS-CSx
Colorectum	PRS7	rs6059892	20	33105766	G	A	-2.44E-04	PRS-CSx
Colorectum	PRS7	rs6059897	20	33113224	G	A	-2.28E-04	PRS-CSx
Colorectum	PRS7	rs6059909	20	33139691	A	C	-1.14E-04	PRS-CSx
Colorectum	PRS7	rs6059918	20	33150503	T	C	-1.34E-04	PRS-CSx
Colorectum	PRS7	rs6059926	20	33164804	T	C	-5.69E-05	PRS-CSx
Colorectum	PRS7	rs6059932	20	33175466	T	C	-1.40E-04	PRS-CSx
Colorectum	PRS7	rs6059956	20	33220070	C	T	-1.64E-04	PRS-CSx
Colorectum	PRS7	rs6061231	20	60956917	A	C	-1.03E-03	PRS-CSx
Colorectum	PRS7	rs6061762	20	60288508	T	C	8.97E-04	PRS-CSx
Colorectum	PRS7	rs6062342	20	62695878	C	A	-6.68E-04	PRS-CSx
Colorectum	PRS7	rs606242	6	117039223	C	T	-1.61E-04	PRS-CSx
Colorectum	PRS7	rs6062833	20	61317233	G	A	-5.64E-04	PRS-CSx
Colorectum	PRS7	rs6063312	20	47343059	T	G	-9.40E-04	PRS-CSx
Colorectum	PRS7	rs6063314	20	47353106	C	T	-5.39E-04	PRS-CSx
Colorectum	PRS7	rs6063516	20	49057248	G	A	2.79E-03	PRS-CSx
Colorectum	PRS7	rs6064908	20	58778125	G	A	8.35E-04	PRS-CSx
Colorectum	PRS7	rs6065670	20	42535221	A	G	9.48E-04	PRS-CSx
Colorectum	PRS7	rs6066773	20	47218804	A	G	-1.81E-04	PRS-CSx
Colorectum	PRS7	rs6066791	20	47251687	A	G	-1.62E-04	PRS-CSx
Colorectum	PRS7	rs6066804	20	47262322	A	G	-1.09E-04	PRS-CSx
Colorectum	PRS7	rs6066815	20	47299870	C	T	-5.80E-04	PRS-CSx
Colorectum	PRS7	rs6066820	20	47318444	C	T	-5.63E-04	PRS-CSx
Colorectum	PRS7	rs6066825	20	47340117	G	A	-6.78E-03	PRS-CSx
Colorectum	PRS7	rs6066830	20	47353897	T	C	-5.29E-04	PRS-CSx
Colorectum	PRS7	rs6066838	20	47359951	A	G	-6.40E-04	PRS-CSx
Colorectum	PRS7	rs6067448	20	49053871	A	C	-6.32E-04	PRS-CSx
Colorectum	PRS7	rs6068445	20	51675407	A	G	8.54E-04	PRS-CSx
Colorectum	PRS7	rs6068699	20	52492074	A	G	7.81E-04	PRS-CSx
Colorectum	PRS7	rs606995	5	134572661	T	C	1.35E-03	PRS-CSx
Colorectum	PRS7	rs6070035	20	55825693	G	A	-7.88E-04	PRS-CSx
Colorectum	PRS7	rs6073238	20	42532910	A	G	1.61E-03	PRS-CSx
Colorectum	PRS7	rs6073246	20	42537679	T	G	7.14E-04	PRS-CSx
Colorectum	PRS7	rs6073248	20	42537771	T	C	3.64E-04	PRS-CSx
Colorectum	PRS7	rs6073253	20	42548816	A	G	1.54E-03	PRS-CSx
Colorectum	PRS7	rs6073280	20	42638666	G	A	1.30E-05	PRS-CSx
Colorectum	PRS7	rs6073281	20	42646435	A	G	-2.32E-04	PRS-CSx
Colorectum	PRS7	rs6073284	20	42647643	G	A	-1.53E-04	PRS-CSx
Colorectum	PRS7	rs6073964	20	35909294	A	G	-4.44E-04	PRS-CSx
Colorectum	PRS7	rs6074366	20	11896206	G	T	-7.80E-04	PRS-CSx
Colorectum	PRS7	rs6075167	20	17045941	C	T	9.53E-04	PRS-CSx
Colorectum	PRS7	rs6076598	20	4043286	A	G	1.78E-03	PRS-CSx
Colorectum	PRS7	rs6076983	20	6331376	C	T	-3.53E-06	PRS-CSx
Colorectum	PRS7	rs6076987	20	6351417	A	C	3.50E-04	PRS-CSx
Colorectum	PRS7	rs6077004	20	6442961	A	G	2.00E-04	PRS-CSx
Colorectum	PRS7	rs6077251	20	7752366	T	C	9.80E-04	PRS-CSx
Colorectum	PRS7	rs6077264	20	7805987	T	G	6.27E-04	PRS-CSx
Colorectum	PRS7	rs6077265	20	7806068	T	C	3.53E-04	PRS-CSx
Colorectum	PRS7	rs6077270	20	7826418	G	A	7.11E-04	PRS-CSx
Colorectum	PRS7	rs6077276	20	7832380	A	G	3.84E-04	PRS-CSx
Colorectum	PRS7	rs6077277	20	7835427	G	A	1.07E-03	PRS-CSx
Colorectum	PRS7	rs6077284	20	7860917	T	C	2.84E-04	PRS-CSx
Colorectum	PRS7	rs6082431	20	274333	C	T	1.84E-05	PRS-CSx

Colorectum	PRS7	rs6085661	20	6693128	T	C	7.97E-03	PRS-CSx
Colorectum	PRS7	rs6085693	20	6819428	A	C	-1.14E-03	PRS-CSx
Colorectum	PRS7	rs6086208	20	7765463	T	C	7.66E-04	PRS-CSx
Colorectum	PRS7	rs6086234	20	7810380	G	A	5.67E-04	PRS-CSx
Colorectum	PRS7	rs6086235	20	7810660	G	A	4.66E-04	PRS-CSx
Colorectum	PRS7	rs6086243	20	7815941	G	A	3.65E-04	PRS-CSx
Colorectum	PRS7	rs6086249	20	7826249	T	C	5.12E-04	PRS-CSx
Colorectum	PRS7	rs6086260	20	7851039	T	G	5.78E-04	PRS-CSx
Colorectum	PRS7	rs6086377	20	8233444	C	A	-4.12E-04	PRS-CSx
Colorectum	PRS7	rs6087577	20	32955423	A	G	-1.45E-04	PRS-CSx
Colorectum	PRS7	rs6087580	20	32992592	A	G	-1.19E-04	PRS-CSx
Colorectum	PRS7	rs6087588	20	33066369	C	T	-2.28E-04	PRS-CSx
Colorectum	PRS7	rs6087592	20	33114503	G	A	-1.57E-04	PRS-CSx
Colorectum	PRS7	rs6087600	20	33157217	C	T	1.35E-05	PRS-CSx
Colorectum	PRS7	rs6088498	20	33020446	G	A	-9.86E-05	PRS-CSx
Colorectum	PRS7	rs6088512	20	33095891	A	G	-5.46E-05	PRS-CSx
Colorectum	PRS7	rs6088527	20	33155841	T	C	-3.53E-05	PRS-CSx
Colorectum	PRS7	rs6088536	20	33189106	C	T	-7.23E-05	PRS-CSx
Colorectum	PRS7	rs6088667	20	33566722	G	T	-4.22E-04	PRS-CSx
Colorectum	PRS7	rs6088678	20	33607551	T	C	-4.70E-04	PRS-CSx
Colorectum	PRS7	rs6088691	20	33633758	T	C	-3.06E-04	PRS-CSx
Colorectum	PRS7	rs6089354	20	60944803	G	A	-2.51E-03	PRS-CSx
Colorectum	PRS7	rs6089356	20	60963494	T	C	9.71E-06	PRS-CSx
Colorectum	PRS7	rs6091236	20	49516995	T	C	-9.14E-04	PRS-CSx
Colorectum	PRS7	rs6091237	20	49525987	C	A	-1.27E-03	PRS-CSx
Colorectum	PRS7	rs609230	12	112146911	T	C	1.85E-04	PRS-CSx
Colorectum	PRS7	rs6092934	20	58970583	T	C	-1.07E-03	PRS-CSx
Colorectum	PRS7	rs6095228	20	47279876	A	G	-4.50E-04	PRS-CSx
Colorectum	PRS7	rs6095239	20	47297539	A	G	-4.95E-04	PRS-CSx
Colorectum	PRS7	rs6095246	20	47320829	G	A	-1.37E-04	PRS-CSx
Colorectum	PRS7	rs6095247	20	47320835	C	T	-7.50E-04	PRS-CSx
Colorectum	PRS7	rs6095248	20	47320932	C	T	-3.17E-04	PRS-CSx
Colorectum	PRS7	rs6095250	20	47322165	C	T	-2.25E-04	PRS-CSx
Colorectum	PRS7	rs6095276	20	47363230	A	G	-7.76E-04	PRS-CSx
Colorectum	PRS7	rs6095277	20	47363257	A	G	-7.11E-04	PRS-CSx
Colorectum	PRS7	rs6095949	20	49061728	G	A	1.70E-03	PRS-CSx
Colorectum	PRS7	rs6096186	20	49533949	C	T	-1.49E-03	PRS-CSx
Colorectum	PRS7	rs6099626	20	56013994	G	T	-1.03E-03	PRS-CSx
Colorectum	PRS7	rs6103562	20	42638646	G	A	-2.84E-04	PRS-CSx
Colorectum	PRS7	rs6103572	20	42657862	T	C	-2.95E-03	PRS-CSx
Colorectum	PRS7	rs6105151	20	13691752	G	A	3.41E-04	PRS-CSx
Colorectum	PRS7	rs6105163	20	13736230	A	G	4.60E-04	PRS-CSx
Colorectum	PRS7	rs6107836	20	6555461	A	G	2.20E-04	PRS-CSx
Colorectum	PRS7	rs6107853	20	6609877	G	A	8.90E-04	PRS-CSx
Colorectum	PRS7	rs6110019	20	13695680	T	C	2.57E-04	PRS-CSx
Colorectum	PRS7	rs6110023	20	13714109	A	G	5.46E-04	PRS-CSx
Colorectum	PRS7	rs6110037	20	13766447	G	A	4.09E-04	PRS-CSx
Colorectum	PRS7	rs611150	12	4004752	T	C	2.07E-03	PRS-CSx
Colorectum	PRS7	rs6117260	20	6422697	C	T	1.26E-04	PRS-CSx
Colorectum	PRS7	rs6117325	20	6519677	A	C	2.58E-04	PRS-CSx
Colorectum	PRS7	rs6117959	20	7797349	T	C	7.90E-04	PRS-CSx
Colorectum	PRS7	rs6120644	20	33023810	A	G	-1.45E-04	PRS-CSx
Colorectum	PRS7	rs6120650	20	33039973	A	G	-1.61E-04	PRS-CSx
Colorectum	PRS7	rs6120663	20	33081906	A	C	-2.28E-05	PRS-CSx
Colorectum	PRS7	rs6120669	20	33105028	C	T	-2.14E-04	PRS-CSx
Colorectum	PRS7	rs6120804	20	33623701	A	C	-4.90E-04	PRS-CSx

Colorectum	PRS7	rs6120828	20	33674460	C	T	-3.97E-04	PRS-CSx
Colorectum	PRS7	rs6121557	20	60961271	C	T	-1.55E-04	PRS-CSx
Colorectum	PRS7	rs6121558	20	60961365	C	T	-5.00E-03	PRS-CSx
Colorectum	PRS7	rs6122720	20	47351095	A	G	-6.04E-04	PRS-CSx
Colorectum	PRS7	rs612347	7	42981764	G	A	-5.44E-04	PRS-CSx
Colorectum	PRS7	rs6123836	20	57448746	C	T	-4.80E-04	PRS-CSx
Colorectum	PRS7	rs6123837	20	57465571	G	A	-8.44E-04	PRS-CSx
Colorectum	PRS7	rs612617	15	48906577	G	A	6.35E-04	PRS-CSx
Colorectum	PRS7	rs612688	11	57577096	A	G	3.97E-04	PRS-CSx
Colorectum	PRS7	rs6127980	20	55823762	A	G	2.91E-05	PRS-CSx
Colorectum	PRS7	rs6127983	20	55824475	C	T	-1.33E-04	PRS-CSx
Colorectum	PRS7	rs6127984	20	55824593	A	G	-1.46E-04	PRS-CSx
Colorectum	PRS7	rs6127985	20	55824760	A	G	-9.91E-05	PRS-CSx
Colorectum	PRS7	rs6127999	20	55893607	T	C	-3.83E-04	PRS-CSx
Colorectum	PRS7	rs6128407	20	57272057	T	C	1.16E-04	PRS-CSx
Colorectum	PRS7	rs6128461	20	57477090	T	C	-4.98E-04	PRS-CSx
Colorectum	PRS7	rs6130478	20	42533655	G	A	-1.18E-03	PRS-CSx
Colorectum	PRS7	rs6132511	20	22379060	G	A	7.36E-04	PRS-CSx
Colorectum	PRS7	rs6133483	20	7723047	C	T	7.88E-04	PRS-CSx
Colorectum	PRS7	rs6133485	20	7739919	T	G	6.12E-04	PRS-CSx
Colorectum	PRS7	rs6133486	20	7747882	T	C	7.04E-04	PRS-CSx
Colorectum	PRS7	rs6133511	20	7873818	C	T	-3.79E-04	PRS-CSx
Colorectum	PRS7	rs6134030	20	10748899	T	G	-2.26E-03	PRS-CSx
Colorectum	PRS7	rs6134426	20	11965355	C	T	9.80E-06	PRS-CSx
Colorectum	PRS7	rs6135981	20	17111709	G	A	3.62E-04	PRS-CSx
Colorectum	PRS7	rs6136317	20	1870069	A	G	-1.45E-03	PRS-CSx
Colorectum	PRS7	rs6137456	20	2236890	T	C	6.76E-04	PRS-CSx
Colorectum	PRS7	rs6137590	20	22242921	T	C	4.16E-04	PRS-CSx
Colorectum	PRS7	rs6137633	20	22376594	G	A	7.28E-04	PRS-CSx
Colorectum	PRS7	rs6137634	20	22377581	T	C	5.97E-04	PRS-CSx
Colorectum	PRS7	rs6137635	20	22377724	C	T	8.59E-04	PRS-CSx
Colorectum	PRS7	rs613791	11	118764051	T	C	8.08E-04	PRS-CSx
Colorectum	PRS7	rs6140071	20	6706493	T	C	4.37E-03	PRS-CSx
Colorectum	PRS7	rs6140410	20	7737540	C	T	4.96E-04	PRS-CSx
Colorectum	PRS7	rs6140412	20	7739376	T	C	5.88E-04	PRS-CSx
Colorectum	PRS7	rs6140413	20	7742039	T	G	5.99E-04	PRS-CSx
Colorectum	PRS7	rs6140414	20	7742500	T	C	7.68E-04	PRS-CSx
Colorectum	PRS7	rs6140425	20	7777476	A	C	8.88E-04	PRS-CSx
Colorectum	PRS7	rs6140428	20	7781532	G	A	3.59E-04	PRS-CSx
Colorectum	PRS7	rs6140442	20	7829397	A	C	4.44E-04	PRS-CSx
Colorectum	PRS7	rs6140453	20	7856704	T	G	-1.96E-03	PRS-CSx
Colorectum	PRS7	rs6140629	20	8488087	C	T	-6.13E-04	PRS-CSx
Colorectum	PRS7	rs6141465	20	32967314	T	C	-2.46E-04	PRS-CSx
Colorectum	PRS7	rs6142946	20	60673065	G	T	-9.61E-04	PRS-CSx
Colorectum	PRS7	rs615608	13	78405883	C	T	-4.78E-04	PRS-CSx
Colorectum	PRS7	rs616408	1	212716033	A	G	5.82E-04	PRS-CSx
Colorectum	PRS7	rs616513	12	112003383	G	T	3.43E-04	PRS-CSx
Colorectum	PRS7	rs617044	12	112132875	C	T	1.85E-04	PRS-CSx
Colorectum	PRS7	rs617384	5	141417287	A	C	-4.18E-04	PRS-CSx
Colorectum	PRS7	rs617572	9	12830070	G	A	5.43E-04	PRS-CSx
Colorectum	PRS7	rs617989	11	74202033	C	A	-7.93E-05	PRS-CSx
Colorectum	PRS7	rs618465	1	54627830	A	G	1.13E-03	PRS-CSx
Colorectum	PRS7	rs618679	11	85671702	A	C	-1.38E-04	PRS-CSx
Colorectum	PRS7	rs618850	11	63865622	C	T	-7.18E-04	PRS-CSx
Colorectum	PRS7	rs619586	11	65266169	G	A	-1.50E-03	PRS-CSx
Colorectum	PRS7	rs621517	1	59977019	C	T	-1.03E-03	PRS-CSx

Colorectum	PRS7	rs621611	11	31896170	A	G	-3.95E-04	PRS-CSx
Colorectum	PRS7	rs621834	18	34351529	T	C	1.04E-03	PRS-CSx
Colorectum	PRS7	rs622824	5	134461481	G	A	3.16E-03	PRS-CSx
Colorectum	PRS7	rs623053	18	33779855	C	T	8.56E-04	PRS-CSx
Colorectum	PRS7	rs624313	20	60927412	G	A	-2.10E-04	PRS-CSx
Colorectum	PRS7	rs626277	13	72347696	A	C	7.38E-04	PRS-CSx
Colorectum	PRS7	rs627308	12	112146555	C	A	4.20E-04	PRS-CSx
Colorectum	PRS7	rs627953	18	10121949	G	A	-6.32E-04	PRS-CSx
Colorectum	PRS7	rs628825	12	111951850	C	T	5.92E-04	PRS-CSx
Colorectum	PRS7	rs628993	11	61539691	A	G	-1.75E-04	PRS-CSx
Colorectum	PRS7	rs630191	7	42976789	A	G	-4.34E-04	PRS-CSx
Colorectum	PRS7	rs630512	12	111952167	C	T	3.31E-04	PRS-CSx
Colorectum	PRS7	rs630616	12	112158966	C	T	2.98E-04	PRS-CSx
Colorectum	PRS7	rs632137	11	120247033	C	T	-3.91E-04	PRS-CSx
Colorectum	PRS7	rs632463	13	33699655	A	G	-2.53E-04	PRS-CSx
Colorectum	PRS7	rs632650	12	112131698	T	G	2.50E-04	PRS-CSx
Colorectum	PRS7	rs634970	6	4115176	T	G	4.08E-04	PRS-CSx
Colorectum	PRS7	rs635719	19	50693639	A	G	-5.35E-04	PRS-CSx
Colorectum	PRS7	rs637931	17	26863072	C	T	-1.76E-03	PRS-CSx
Colorectum	PRS7	rs638329	6	4134768	G	A	3.23E-04	PRS-CSx
Colorectum	PRS7	rs638476	13	72741545	G	T	4.29E-04	PRS-CSx
Colorectum	PRS7	rs638491	9	15290012	A	G	-8.12E-04	PRS-CSx
Colorectum	PRS7	rs638759	11	33218974	A	G	-1.24E-04	PRS-CSx
Colorectum	PRS7	rs639459	7	42951936	G	T	-4.22E-04	PRS-CSx
Colorectum	PRS7	rs639836	20	60926223	G	A	-1.94E-04	PRS-CSx
Colorectum	PRS7	rs639933	5	134467751	C	A	2.79E-03	PRS-CSx
Colorectum	PRS7	rs64036	6	29527267	A	C	3.23E-04	PRS-CSx
Colorectum	PRS7	rs640496	1	183157036	C	T	1.10E-04	PRS-CSx
Colorectum	PRS7	rs640783	12	112168050	A	G	2.62E-04	PRS-CSx
Colorectum	PRS7	rs6414283	3	119195913	C	T	-4.72E-04	PRS-CSx
Colorectum	PRS7	rs6414331	3	133804070	T	C	-3.91E-04	PRS-CSx
Colorectum	PRS7	rs641455	5	134472374	A	G	1.83E-03	PRS-CSx
Colorectum	PRS7	rs641579	9	15284100	T	C	-5.49E-04	PRS-CSx
Colorectum	PRS7	rs6421210	12	64417900	T	C	-7.84E-04	PRS-CSx
Colorectum	PRS7	rs642245	11	86389536	A	G	3.56E-04	PRS-CSx
Colorectum	PRS7	rs6424092	1	2532899	A	C	-8.59E-05	PRS-CSx
Colorectum	PRS7	rs6424883	1	183037695	T	C	8.98E-04	PRS-CSx
Colorectum	PRS7	rs6424888	1	183085696	A	G	2.36E-04	PRS-CSx
Colorectum	PRS7	rs6426748	1	22707565	T	C	-6.70E-04	PRS-CSx
Colorectum	PRS7	rs6426752	1	22718991	A	G	-4.88E-04	PRS-CSx
Colorectum	PRS7	rs6427761	1	199188907	G	A	1.78E-03	PRS-CSx
Colorectum	PRS7	rs6429195	1	240407383	A	G	8.00E-04	PRS-CSx
Colorectum	PRS7	rs6429746	1	15815579	G	A	-3.09E-04	PRS-CSx
Colorectum	PRS7	rs6430557	2	131822607	G	A	-3.53E-04	PRS-CSx
Colorectum	PRS7	rs6430581	2	131885847	A	C	-2.70E-04	PRS-CSx
Colorectum	PRS7	rs6431114	2	130619051	A	G	2.16E-03	PRS-CSx
Colorectum	PRS7	rs6432191	2	11548589	C	T	-8.07E-04	PRS-CSx
Colorectum	PRS7	rs643319	9	22017836	A	C	5.47E-04	PRS-CSx
Colorectum	PRS7	rs6433777	2	152875744	T	G	-5.64E-04	PRS-CSx
Colorectum	PRS7	rs6434978	2	199567687	T	C	4.38E-04	PRS-CSx
Colorectum	PRS7	rs6434981	2	199573857	C	A	5.16E-04	PRS-CSx
Colorectum	PRS7	rs6436310	2	223167710	G	A	-1.07E-03	PRS-CSx
Colorectum	PRS7	rs6436490	2	225326693	A	C	7.59E-05	PRS-CSx
Colorectum	PRS7	rs6437745	3	107510457	G	A	-2.16E-03	PRS-CSx
Colorectum	PRS7	rs6438535	3	119218714	T	C	-3.51E-04	PRS-CSx
Colorectum	PRS7	rs6438552	3	119631814	A	G	1.25E-04	PRS-CSx

Colorectum	PRS7	rs6439200	3	129521807	G	A	2.18E-04	PRS-CSx
Colorectum	PRS7	rs6439204	3	129631779	C	T	4.56E-05	PRS-CSx
Colorectum	PRS7	rs6439470	3	133980309	G	T	6.80E-04	PRS-CSx
Colorectum	PRS7	rs6439926	3	140176642	A	G	-5.96E-04	PRS-CSx
Colorectum	PRS7	rs644027	7	42950845	C	T	-6.02E-04	PRS-CSx
Colorectum	PRS7	rs644041	5	134498408	A	G	1.13E-03	PRS-CSx
Colorectum	PRS7	rs644106	7	42950905	C	T	-3.11E-04	PRS-CSx
Colorectum	PRS7	rs6441929	3	45924791	G	A	-4.65E-04	PRS-CSx
Colorectum	PRS7	rs6442180	3	10588012	C	T	7.22E-04	PRS-CSx
Colorectum	PRS7	rs6442791	3	3344733	C	T	-4.17E-04	PRS-CSx
Colorectum	PRS7	rs6443978	3	184242854	C	T	-4.65E-03	PRS-CSx
Colorectum	PRS7	rs6443991	3	184416009	T	C	-1.12E-03	PRS-CSx
Colorectum	PRS7	rs6444188	3	186677936	G	T	6.10E-04	PRS-CSx
Colorectum	PRS7	rs6444679	3	192731246	C	T	-4.37E-04	PRS-CSx
Colorectum	PRS7	rs6445122	3	173555343	C	T	4.12E-04	PRS-CSx
Colorectum	PRS7	rs6445138	3	173778940	T	C	9.46E-04	PRS-CSx
Colorectum	PRS7	rs6445421	3	64620371	T	C	3.09E-04	PRS-CSx
Colorectum	PRS7	rs6449108	4	15202033	T	C	-9.44E-04	PRS-CSx
Colorectum	PRS7	rs6449116	4	15223177	C	T	-3.51E-04	PRS-CSx
Colorectum	PRS7	rs6449287	4	17373412	C	T	-4.81E-04	PRS-CSx
Colorectum	PRS7	rs644955	1	55168084	C	T	6.51E-04	PRS-CSx
Colorectum	PRS7	rs6449939	5	67303386	C	T	2.35E-04	PRS-CSx
Colorectum	PRS7	rs6451439	5	39651401	T	G	2.48E-04	PRS-CSx
Colorectum	PRS7	rs6451452	5	39722109	A	C	-2.95E-04	PRS-CSx
Colorectum	PRS7	rs6451535	5	40688031	G	A	1.86E-04	PRS-CSx
Colorectum	PRS7	rs6454396	6	85533497	C	T	9.26E-04	PRS-CSx
Colorectum	PRS7	rs645541	11	74245673	G	A	-7.70E-05	PRS-CSx
Colorectum	PRS7	rs6456369	6	20660365	C	T	-3.04E-04	PRS-CSx
Colorectum	PRS7	rs6456733	6	26566804	C	T	2.80E-05	PRS-CSx
Colorectum	PRS7	rs6456735	6	26574149	G	A	4.19E-05	PRS-CSx
Colorectum	PRS7	rs6457553	6	32330695	G	A	-1.73E-04	PRS-CSx
Colorectum	PRS7	rs6457736	6	33564412	C	T	4.89E-04	PRS-CSx
Colorectum	PRS7	rs645781	3	101562980	G	A	-4.21E-04	PRS-CSx
Colorectum	PRS7	rs6457931	6	36613812	T	G	-2.15E-04	PRS-CSx
Colorectum	PRS7	rs6458238	6	41717705	A	G	3.07E-03	PRS-CSx
Colorectum	PRS7	rs6458544	6	47126369	G	A	1.05E-03	PRS-CSx
Colorectum	PRS7	rs6461115	7	2103668	G	A	-2.95E-04	PRS-CSx
Colorectum	PRS7	rs6463255	7	44989199	G	A	-1.39E-03	PRS-CSx
Colorectum	PRS7	rs6463354	7	46919215	A	G	2.61E-04	PRS-CSx
Colorectum	PRS7	rs6463358	7	46919603	T	G	3.01E-04	PRS-CSx
Colorectum	PRS7	rs6464821	7	147253201	A	G	2.47E-04	PRS-CSx
Colorectum	PRS7	rs646506	5	134458908	A	G	8.33E-04	PRS-CSx
Colorectum	PRS7	rs6465189	7	88838746	A	C	-3.27E-04	PRS-CSx
Colorectum	PRS7	rs6465402	7	93704209	C	T	2.42E-04	PRS-CSx
Colorectum	PRS7	rs6465825	7	77416439	C	T	-1.74E-04	PRS-CSx
Colorectum	PRS7	rs6469132	8	108711440	T	C	-6.17E-04	PRS-CSx
Colorectum	PRS7	rs6469354	8	112489722	T	G	-1.64E-03	PRS-CSx
Colorectum	PRS7	rs6469660	8	117797719	G	T	9.48E-04	PRS-CSx
Colorectum	PRS7	rs6469895	8	121063562	C	A	-4.32E-04	PRS-CSx
Colorectum	PRS7	rs6471687	8	59104101	A	G	-3.17E-03	PRS-CSx
Colorectum	PRS7	rs6472156	8	65771777	A	G	3.33E-04	PRS-CSx
Colorectum	PRS7	rs6472714	8	73815083	G	A	1.17E-04	PRS-CSx
Colorectum	PRS7	rs6473038	8	78351635	T	C	6.81E-04	PRS-CSx
Colorectum	PRS7	rs6473393	8	83826701	G	A	-1.96E-04	PRS-CSx
Colorectum	PRS7	rs6474346	8	41312419	A	G	2.41E-04	PRS-CSx
Colorectum	PRS7	rs6475295	9	18974072	T	C	-5.03E-04	PRS-CSx

Colorectum	PRS7	rs6475326	9	19386565	C	T	1.89E-06	PRS-CSx
Colorectum	PRS7	rs6475606	9	22081850	C	T	2.19E-04	PRS-CSx
Colorectum	PRS7	rs6476018	9	2793659	T	C	-8.52E-04	PRS-CSx
Colorectum	PRS7	rs6476424	9	33778399	G	A	-2.97E-04	PRS-CSx
Colorectum	PRS7	rs6476429	9	33997093	A	G	-1.44E-04	PRS-CSx
Colorectum	PRS7	rs6477056	9	634160	G	A	9.45E-04	PRS-CSx
Colorectum	PRS7	rs6477081	9	649866	A	G	2.48E-04	PRS-CSx
Colorectum	PRS7	rs6477683	9	111592442	A	C	1.67E-03	PRS-CSx
Colorectum	PRS7	rs6478173	9	118251348	A	C	-4.01E-04	PRS-CSx
Colorectum	PRS7	rs6478176	9	118258940	G	A	-9.92E-04	PRS-CSx
Colorectum	PRS7	rs647824	11	74257850	G	A	-1.51E-04	PRS-CSx
Colorectum	PRS7	rs6478924	9	132667075	A	G	-6.14E-04	PRS-CSx
Colorectum	PRS7	rs6478969	9	101819532	T	G	-2.24E-04	PRS-CSx
Colorectum	PRS7	rs6479768	10	52638933	T	G	6.51E-04	PRS-CSx
Colorectum	PRS7	rs6479769	10	52643221	T	C	2.91E-03	PRS-CSx
Colorectum	PRS7	rs6480592	10	73764509	T	C	-4.06E-04	PRS-CSx
Colorectum	PRS7	rs6481493	10	28208262	T	C	9.36E-04	PRS-CSx
Colorectum	PRS7	rs6481494	10	28216167	T	C	6.96E-04	PRS-CSx
Colorectum	PRS7	rs6483747	11	21180977	A	G	-4.48E-04	PRS-CSx
Colorectum	PRS7	rs6484644	11	33112903	A	C	-2.07E-04	PRS-CSx
Colorectum	PRS7	rs6484648	11	33128256	G	T	-1.80E-04	PRS-CSx
Colorectum	PRS7	rs6485690	11	46798631	G	A	3.44E-04	PRS-CSx
Colorectum	PRS7	rs6485696	11	46842045	T	C	6.64E-04	PRS-CSx
Colorectum	PRS7	rs6485702	11	46898771	C	T	4.52E-04	PRS-CSx
Colorectum	PRS7	rs6485715	11	46975125	C	T	1.31E-04	PRS-CSx
Colorectum	PRS7	rs6485726	11	47082122	A	C	3.47E-04	PRS-CSx
Colorectum	PRS7	rs6485739	11	47145072	A	G	3.95E-04	PRS-CSx
Colorectum	PRS7	rs648677	11	74250649	C	T	-2.73E-04	PRS-CSx
Colorectum	PRS7	rs648691	19	50693096	C	T	-5.24E-04	PRS-CSx
Colorectum	PRS7	rs6487068	12	20378156	G	T	4.28E-04	PRS-CSx
Colorectum	PRS7	rs6487464	12	25391456	T	C	-3.51E-04	PRS-CSx
Colorectum	PRS7	rs6487465	12	25410283	C	T	-5.53E-04	PRS-CSx
Colorectum	PRS7	rs6487998	12	31796881	C	T	-4.48E-04	PRS-CSx
Colorectum	PRS7	rs6488619	12	13927896	C	T	5.70E-04	PRS-CSx
Colorectum	PRS7	rs6488626	12	14108755	C	T	-4.53E-04	PRS-CSx
Colorectum	PRS7	rs6489244	12	122613540	T	C	1.82E-04	PRS-CSx
Colorectum	PRS7	rs6489709	12	6405578	T	C	-5.29E-03	PRS-CSx
Colorectum	PRS7	rs6489844	12	111385296	G	A	4.35E-04	PRS-CSx
Colorectum	PRS7	rs6489858	12	113207818	G	T	-7.63E-05	PRS-CSx
Colorectum	PRS7	rs6490020	12	115921518	C	T	7.23E-04	PRS-CSx
Colorectum	PRS7	rs6490021	12	115928920	C	A	6.05E-04	PRS-CSx
Colorectum	PRS7	rs6490029	12	111698457	G	A	-1.31E-03	PRS-CSx
Colorectum	PRS7	rs6490055	12	111768973	A	G	1.42E-04	PRS-CSx
Colorectum	PRS7	rs6490077	12	111791677	C	A	1.44E-04	PRS-CSx
Colorectum	PRS7	rs6490164	12	118520240	G	A	5.31E-04	PRS-CSx
Colorectum	PRS7	rs6490278	12	120428468	T	C	4.46E-04	PRS-CSx
Colorectum	PRS7	rs6490281	12	120452826	G	A	3.73E-04	PRS-CSx
Colorectum	PRS7	rs6490284	12	120526760	G	A	2.24E-04	PRS-CSx
Colorectum	PRS7	rs6493	14	78140329	A	G	-7.68E-04	PRS-CSx
Colorectum	PRS7	rs6493547	15	52526507	G	A	5.43E-04	PRS-CSx
Colorectum	PRS7	rs6493688	15	31772875	A	G	1.28E-03	PRS-CSx
Colorectum	PRS7	rs6494029	15	58923206	T	C	5.38E-04	PRS-CSx
Colorectum	PRS7	rs649406	12	112126065	T	C	2.16E-04	PRS-CSx
Colorectum	PRS7	rs649419	1	59975370	G	A	-3.99E-03	PRS-CSx
Colorectum	PRS7	rs6494627	15	67338914	A	G	3.23E-04	PRS-CSx
Colorectum	PRS7	rs6494906	15	71629569	C	A	2.11E-04	PRS-CSx

Colorectum	PRS7	rs6495182	15	75814388	T	C	3.72E-04	PRS-CSx
Colorectum	PRS7	rs6495616	15	82101383	A	C	-6.29E-04	PRS-CSx
Colorectum	PRS7	rs6495717	15	35103105	G	A	8.62E-04	PRS-CSx
Colorectum	PRS7	rs6496665	15	90891615	A	G	2.76E-04	PRS-CSx
Colorectum	PRS7	rs6496667	15	90893668	A	C	2.09E-04	PRS-CSx
Colorectum	PRS7	rs6497953	16	26529722	G	A	2.30E-03	PRS-CSx
Colorectum	PRS7	rs6499192	16	68707313	A	G	3.82E-05	PRS-CSx
Colorectum	PRS7	rs6499193	16	68727069	T	C	3.55E-04	PRS-CSx
Colorectum	PRS7	rs6499209	16	68964913	A	C	4.96E-04	PRS-CSx
Colorectum	PRS7	rs6499600	16	72979374	T	C	5.09E-04	PRS-CSx
Colorectum	PRS7	rs6500150	16	60846250	A	G	-5.02E-04	PRS-CSx
Colorectum	PRS7	rs6500261	16	49898441	T	C	6.89E-04	PRS-CSx
Colorectum	PRS7	rs6500264	16	49926374	G	T	-5.74E-04	PRS-CSx
Colorectum	PRS7	rs6500288	16	50148408	G	A	-3.54E-04	PRS-CSx
Colorectum	PRS7	rs6500291	16	50154813	C	T	-2.25E-04	PRS-CSx
Colorectum	PRS7	rs6500596	16	4470027	G	T	5.49E-04	PRS-CSx
Colorectum	PRS7	rs6500602	16	4497451	T	C	4.64E-04	PRS-CSx
Colorectum	PRS7	rs6501084	16	8244539	T	C	1.78E-03	PRS-CSx
Colorectum	PRS7	rs6501495	17	69570803	T	C	6.35E-04	PRS-CSx
Colorectum	PRS7	rs6501496	17	69570941	T	C	6.04E-04	PRS-CSx
Colorectum	PRS7	rs6501529	17	70297363	T	C	-8.00E-04	PRS-CSx
Colorectum	PRS7	rs6501985	17	75712159	G	A	3.89E-04	PRS-CSx
Colorectum	PRS7	rs6502831	17	4930987	G	A	-5.00E-04	PRS-CSx
Colorectum	PRS7	rs6503018	17	7292107	G	A	3.62E-03	PRS-CSx
Colorectum	PRS7	rs6503327	17	10718996	A	G	-4.50E-03	PRS-CSx
Colorectum	PRS7	rs6503695	17	40499533	C	T	9.66E-04	PRS-CSx
Colorectum	PRS7	rs6503900	17	57263393	G	A	2.58E-04	PRS-CSx
Colorectum	PRS7	rs6503913	17	57356184	A	G	1.03E-04	PRS-CSx
Colorectum	PRS7	rs6504921	17	52710924	G	T	-1.95E-04	PRS-CSx
Colorectum	PRS7	rs6506671	18	9455828	T	C	-8.84E-04	PRS-CSx
Colorectum	PRS7	rs6507173	18	34118777	T	G	-1.34E-03	PRS-CSx
Colorectum	PRS7	rs6507876	18	46458921	G	T	1.81E-03	PRS-CSx
Colorectum	PRS7	rs6508738	19	38099700	A	G	-1.53E-04	PRS-CSx
Colorectum	PRS7	rs6508813	19	39162230	A	C	3.83E-04	PRS-CSx
Colorectum	PRS7	rs6509286	19	47076278	C	A	6.95E-04	PRS-CSx
Colorectum	PRS7	rs651007	9	136153875	T	C	2.36E-04	PRS-CSx
Colorectum	PRS7	rs6510306	19	33432709	C	T	3.10E-04	PRS-CSx
Colorectum	PRS7	rs6511	8	101252680	T	C	-2.82E-04	PRS-CSx
Colorectum	PRS7	rs651254	2	12787913	G	T	1.38E-03	PRS-CSx
Colorectum	PRS7	rs6517173	21	34813562	A	C	2.59E-05	PRS-CSx
Colorectum	PRS7	rs6517223	21	35662414	G	A	2.03E-03	PRS-CSx
Colorectum	PRS7	rs6517803	21	19661872	T	C	8.21E-04	PRS-CSx
Colorectum	PRS7	rs6517805	21	19662318	T	G	8.31E-04	PRS-CSx
Colorectum	PRS7	rs6518216	21	46610842	C	A	-4.89E-04	PRS-CSx
Colorectum	PRS7	rs6518289	21	47787002	T	C	6.72E-04	PRS-CSx
Colorectum	PRS7	rs6518304	21	48035505	C	T	1.91E-04	PRS-CSx
Colorectum	PRS7	rs653079	7	42965638	T	C	-3.38E-04	PRS-CSx
Colorectum	PRS7	rs6532769	4	99780391	C	T	-1.03E-03	PRS-CSx
Colorectum	PRS7	rs6533163	4	105794260	A	G	4.06E-04	PRS-CSx
Colorectum	PRS7	rs6533171	4	105963240	A	G	8.12E-04	PRS-CSx
Colorectum	PRS7	rs6533787	4	115384090	A	G	6.86E-05	PRS-CSx
Colorectum	PRS7	rs6534012	4	75547732	A	C	-4.50E-04	PRS-CSx
Colorectum	PRS7	rs6534150	4	120685502	A	G	-4.43E-04	PRS-CSx
Colorectum	PRS7	rs6537494	10	50300393	T	C	-5.59E-04	PRS-CSx
Colorectum	PRS7	rs6538356	12	78180871	A	G	3.06E-04	PRS-CSx
Colorectum	PRS7	rs6538534	12	95154502	C	T	-3.42E-04	PRS-CSx

Colorectum	PRS7	rs6538566	12	95296558	A	C	4.28E-04	PRS-CSx
Colorectum	PRS7	rs653881	7	42959220	T	C	-3.50E-04	PRS-CSx
Colorectum	PRS7	rs6539369	12	108228298	A	G	-1.68E-03	PRS-CSx
Colorectum	PRS7	rs6539377	12	108242007	C	T	-1.31E-03	PRS-CSx
Colorectum	PRS7	rs6539471	12	80089395	C	T	1.30E-04	PRS-CSx
Colorectum	PRS7	rs6540277	16	86185024	A	C	4.94E-04	PRS-CSx
Colorectum	PRS7	rs6541059	1	12799736	C	T	3.24E-04	PRS-CSx
Colorectum	PRS7	rs654237	3	136068699	T	C	5.47E-06	PRS-CSx
Colorectum	PRS7	rs6543426	2	107243420	T	G	4.79E-04	PRS-CSx
Colorectum	PRS7	rs654479	11	117225134	A	G	3.89E-04	PRS-CSx
Colorectum	PRS7	rs6545040	2	48676931	A	G	3.24E-04	PRS-CSx
Colorectum	PRS7	rs6545043	2	48723552	G	A	2.96E-04	PRS-CSx
Colorectum	PRS7	rs6545046	2	48750410	C	T	-4.46E-04	PRS-CSx
Colorectum	PRS7	rs6546119	2	65235333	G	T	6.54E-04	PRS-CSx
Colorectum	PRS7	rs6546891	2	74334790	G	A	-1.50E-03	PRS-CSx
Colorectum	PRS7	rs6547846	2	28575961	C	T	1.49E-04	PRS-CSx
Colorectum	PRS7	rs655025	11	74208029	C	T	-1.39E-04	PRS-CSx
Colorectum	PRS7	rs6550459	3	37223735	A	G	-4.06E-04	PRS-CSx
Colorectum	PRS7	rs6550702	3	18867579	A	G	1.50E-03	PRS-CSx
Colorectum	PRS7	rs6550934	3	25052333	G	A	-1.55E-03	PRS-CSx
Colorectum	PRS7	rs6552677	4	184465524	G	A	-1.50E-03	PRS-CSx
Colorectum	PRS7	rs6553796	4	175367789	T	C	-8.49E-04	PRS-CSx
Colorectum	PRS7	rs6555557	5	8590608	T	G	5.84E-04	PRS-CSx
Colorectum	PRS7	rs6555802	5	167773074	A	C	9.54E-04	PRS-CSx
Colorectum	PRS7	rs655630	6	53432210	A	G	-3.16E-04	PRS-CSx
Colorectum	PRS7	rs6557142	6	151784049	T	G	6.87E-04	PRS-CSx
Colorectum	PRS7	rs6558885	8	4321410	A	G	-9.29E-04	PRS-CSx
Colorectum	PRS7	rs6561484	13	49460357	G	A	-5.06E-04	PRS-CSx
Colorectum	PRS7	rs6562616	13	70839073	T	C	-2.63E-04	PRS-CSx
Colorectum	PRS7	rs6563230	13	82096741	T	C	4.36E-04	PRS-CSx
Colorectum	PRS7	rs6564074	16	84786250	C	A	-5.45E-04	PRS-CSx
Colorectum	PRS7	rs6564261	16	75492242	C	T	5.38E-04	PRS-CSx
Colorectum	PRS7	rs6564724	16	80045598	A	G	-1.98E-04	PRS-CSx
Colorectum	PRS7	rs6564728	16	80096832	C	T	9.53E-05	PRS-CSx
Colorectum	PRS7	rs6567328	18	60844089	G	A	-6.49E-04	PRS-CSx
Colorectum	PRS7	rs6569128	6	120809500	C	T	4.95E-04	PRS-CSx
Colorectum	PRS7	rs6569737	6	131613602	G	A	4.02E-04	PRS-CSx
Colorectum	PRS7	rs6569892	6	133988802	C	T	3.42E-04	PRS-CSx
Colorectum	PRS7	rs6569894	6	133989016	G	A	5.40E-04	PRS-CSx
Colorectum	PRS7	rs6569992	6	135452152	A	G	7.02E-04	PRS-CSx
Colorectum	PRS7	rs6570057	6	136230385	A	G	2.72E-03	PRS-CSx
Colorectum	PRS7	rs6571481	14	32355076	T	G	-3.77E-04	PRS-CSx
Colorectum	PRS7	rs657197	12	111965658	C	A	4.53E-04	PRS-CSx
Colorectum	PRS7	rs657235	3	193252724	T	C	-4.12E-04	PRS-CSx
Colorectum	PRS7	rs6573192	14	58678269	G	A	2.27E-04	PRS-CSx
Colorectum	PRS7	rs6573193	14	58684872	C	T	-1.08E-06	PRS-CSx
Colorectum	PRS7	rs6573194	14	58712914	T	C	2.49E-04	PRS-CSx
Colorectum	PRS7	rs6573196	14	58722539	G	A	2.11E-04	PRS-CSx
Colorectum	PRS7	rs6573198	14	58759762	A	G	3.05E-04	PRS-CSx
Colorectum	PRS7	rs6573229	14	59400491	C	T	2.28E-04	PRS-CSx
Colorectum	PRS7	rs657339	11	74208592	G	T	-1.03E-04	PRS-CSx
Colorectum	PRS7	rs6573628	14	66252937	G	A	3.87E-04	PRS-CSx
Colorectum	PRS7	rs6574631	14	81675772	A	G	-2.32E-04	PRS-CSx
Colorectum	PRS7	rs657474	11	74262213	C	T	-1.00E-04	PRS-CSx
Colorectum	PRS7	rs657507	13	78429260	A	G	-2.28E-04	PRS-CSx
Colorectum	PRS7	rs657555	18	12847136	C	T	-1.58E-04	PRS-CSx

Colorectum	PRS7	rs6575988	14	103900827	A	C	2.86E-04	PRS-CSx
Colorectum	PRS7	rs6577012	2	95971422	C	A	-1.00E-04	PRS-CSx
Colorectum	PRS7	rs6577013	2	95984930	G	A	-1.60E-04	PRS-CSx
Colorectum	PRS7	rs6578820	11	7037829	G	A	-2.09E-04	PRS-CSx
Colorectum	PRS7	rs6578882	11	7599391	A	C	-1.01E-03	PRS-CSx
Colorectum	PRS7	rs6579165	20	32994715	A	G	-1.08E-04	PRS-CSx
Colorectum	PRS7	rs6580311	5	143504010	T	C	-2.47E-04	PRS-CSx
Colorectum	PRS7	rs6580317	5	143772718	C	T	-2.89E-04	PRS-CSx
Colorectum	PRS7	rs6580761	12	51115582	T	C	6.59E-05	PRS-CSx
Colorectum	PRS7	rs6581522	12	64415030	G	A	-6.46E-04	PRS-CSx
Colorectum	PRS7	rs6581525	12	64421548	A	G	-9.01E-04	PRS-CSx
Colorectum	PRS7	rs6581527	12	64421995	G	A	-7.26E-04	PRS-CSx
Colorectum	PRS7	rs6584283	10	101290301	T	C	-4.73E-04	PRS-CSx
Colorectum	PRS7	rs6585196	10	114737050	C	T	-3.32E-04	PRS-CSx
Colorectum	PRS7	rs6587781	1	58040173	G	A	-4.51E-04	PRS-CSx
Colorectum	PRS7	rs6589218	11	111167557	A	C	2.11E-03	PRS-CSx
Colorectum	PRS7	rs6589220	11	111173290	C	T	1.78E-03	PRS-CSx
Colorectum	PRS7	rs6589812	11	120278359	C	T	-2.04E-04	PRS-CSx
Colorectum	PRS7	rs6589813	11	120293490	G	A	-1.91E-04	PRS-CSx
Colorectum	PRS7	rs6590735	11	134022087	G	T	-3.61E-04	PRS-CSx
Colorectum	PRS7	rs6591182	11	65349756	G	T	-2.80E-04	PRS-CSx
Colorectum	PRS7	rs6592	7	99668107	T	G	1.93E-04	PRS-CSx
Colorectum	PRS7	rs6592573	11	74312962	A	G	-2.25E-04	PRS-CSx
Colorectum	PRS7	rs6592577	11	74327614	G	A	-9.37E-04	PRS-CSx
Colorectum	PRS7	rs6592590	11	74381029	T	C	4.36E-04	PRS-CSx
Colorectum	PRS7	rs6594321	5	107921232	G	A	-3.93E-04	PRS-CSx
Colorectum	PRS7	rs659581	11	74250987	T	C	-3.10E-04	PRS-CSx
Colorectum	PRS7	rs6595936	5	129425885	G	A	-2.84E-04	PRS-CSx
Colorectum	PRS7	rs6596197	5	134431163	C	A	4.82E-04	PRS-CSx
Colorectum	PRS7	rs6598025	11	392079	T	C	1.91E-03	PRS-CSx
Colorectum	PRS7	rs6598483	15	102014790	C	T	-6.66E-04	PRS-CSx
Colorectum	PRS7	rs6599155	3	41489604	C	A	-2.82E-04	PRS-CSx
Colorectum	PRS7	rs6600900	4	70013537	A	G	-1.42E-04	PRS-CSx
Colorectum	PRS7	rs660855	11	74251112	G	T	-3.93E-04	PRS-CSx
Colorectum	PRS7	rs661356	9	244457	G	A	-6.81E-04	PRS-CSx
Colorectum	PRS7	rs661451	1	183169690	C	T	-9.19E-05	PRS-CSx
Colorectum	PRS7	rs661592	7	42952486	C	T	-2.80E-04	PRS-CSx
Colorectum	PRS7	rs661781	11	74243390	C	T	-2.52E-04	PRS-CSx
Colorectum	PRS7	rs662792	3	101599608	C	T	2.31E-04	PRS-CSx
Colorectum	PRS7	rs663555	11	74251731	C	T	-2.46E-04	PRS-CSx
Colorectum	PRS7	rs663838	9	84088631	T	C	-4.76E-04	PRS-CSx
Colorectum	PRS7	rs664226	11	64789194	C	T	-2.26E-04	PRS-CSx
Colorectum	PRS7	rs664896	1	201628990	C	T	-1.22E-03	PRS-CSx
Colorectum	PRS7	rs665211	3	101600028	T	C	1.81E-04	PRS-CSx
Colorectum	PRS7	rs665237	2	31869943	C	T	-2.70E-04	PRS-CSx
Colorectum	PRS7	rs6656310	1	11002488	C	T	1.83E-03	PRS-CSx
Colorectum	PRS7	rs6656332	1	199121704	C	T	7.57E-04	PRS-CSx
Colorectum	PRS7	rs6656763	1	62661792	G	A	-3.84E-04	PRS-CSx
Colorectum	PRS7	rs6660031	1	110365045	A	G	3.48E-03	PRS-CSx
Colorectum	PRS7	rs6661433	1	208526097	A	G	1.56E-03	PRS-CSx
Colorectum	PRS7	rs6665228	1	194702451	T	C	-8.90E-04	PRS-CSx
Colorectum	PRS7	rs6667605	1	2502780	C	T	-2.74E-04	PRS-CSx
Colorectum	PRS7	rs666951	12	112136583	C	A	2.38E-04	PRS-CSx
Colorectum	PRS7	rs6669870	1	3421586	T	C	-5.38E-04	PRS-CSx
Colorectum	PRS7	rs6669967	1	179435593	A	G	2.59E-04	PRS-CSx
Colorectum	PRS7	rs667085	13	78421358	G	A	-4.97E-04	PRS-CSx

Colorectum	PRS7	rs6671633	1	45500294	A	G	2.77E-04	PRS-CSx
Colorectum	PRS7	rs6671680	1	192888345	T	C	-8.78E-04	PRS-CSx
Colorectum	PRS7	rs667209	7	42959956	A	G	-1.39E-04	PRS-CSx
Colorectum	PRS7	rs6672093	1	183079853	T	C	2.10E-04	PRS-CSx
Colorectum	PRS7	rs6672306	1	183058898	G	A	4.52E-04	PRS-CSx
Colorectum	PRS7	rs6672780	1	164980830	C	T	-3.11E-03	PRS-CSx
Colorectum	PRS7	rs6678531	1	90785241	C	T	6.31E-05	PRS-CSx
Colorectum	PRS7	rs6682391	1	116390772	T	C	4.52E-04	PRS-CSx
Colorectum	PRS7	rs6682619	1	22690923	T	G	2.93E-04	PRS-CSx
Colorectum	PRS7	rs6683506	1	88936206	A	G	8.82E-04	PRS-CSx
Colorectum	PRS7	rs668438	6	35901378	G	A	3.41E-04	PRS-CSx
Colorectum	PRS7	rs6684865	1	2546229	G	A	-1.57E-04	PRS-CSx
Colorectum	PRS7	rs6685648	1	15825195	T	C	-4.55E-04	PRS-CSx
Colorectum	PRS7	rs6685999	1	185451824	G	A	7.43E-04	PRS-CSx
Colorectum	PRS7	rs6686093	1	221310947	G	T	1.02E-04	PRS-CSx
Colorectum	PRS7	rs668718	18	4363364	C	T	3.66E-04	PRS-CSx
Colorectum	PRS7	rs6687497	1	233279256	T	C	7.94E-05	PRS-CSx
Colorectum	PRS7	rs6687758	1	222164948	G	A	3.72E-03	PRS-CSx
Colorectum	PRS7	rs668988	20	753739	A	G	-6.91E-04	PRS-CSx
Colorectum	PRS7	rs669086	18	34377425	C	T	-2.57E-04	PRS-CSx
Colorectum	PRS7	rs6692775	1	46698490	T	G	5.27E-04	PRS-CSx
Colorectum	PRS7	rs6693555	1	88919998	C	A	-7.85E-04	PRS-CSx
Colorectum	PRS7	rs669433	11	57449565	C	T	1.78E-04	PRS-CSx
Colorectum	PRS7	rs669556	11	85781322	C	T	-9.73E-05	PRS-CSx
Colorectum	PRS7	rs6695584	1	222161989	G	A	4.44E-03	PRS-CSx
Colorectum	PRS7	rs6695712	1	213832229	C	T	1.25E-03	PRS-CSx
Colorectum	PRS7	rs6696348	1	226817467	C	T	-4.94E-04	PRS-CSx
Colorectum	PRS7	rs6696981	1	22702858	T	G	-7.62E-04	PRS-CSx
Colorectum	PRS7	rs669822	18	12863142	C	T	-1.19E-03	PRS-CSx
Colorectum	PRS7	rs6698817	1	2564183	C	T	-1.08E-04	PRS-CSx
Colorectum	PRS7	rs6699332	1	246935011	A	G	5.17E-04	PRS-CSx
Colorectum	PRS7	rs6699830	1	223886784	G	T	4.64E-04	PRS-CSx
Colorectum	PRS7	rs6701026	1	201814004	T	C	1.48E-05	PRS-CSx
Colorectum	PRS7	rs6701767	1	49070646	C	T	2.07E-03	PRS-CSx
Colorectum	PRS7	rs6704135	1	22718261	T	C	9.40E-04	PRS-CSx
Colorectum	PRS7	rs6705069	2	42438608	G	T	-1.43E-04	PRS-CSx
Colorectum	PRS7	rs6705911	2	182979076	G	A	1.55E-04	PRS-CSx
Colorectum	PRS7	rs6706646	2	176540403	C	T	2.36E-04	PRS-CSx
Colorectum	PRS7	rs670736	11	86383679	A	G	9.53E-04	PRS-CSx
Colorectum	PRS7	rs6710395	2	168339866	T	G	5.54E-04	PRS-CSx
Colorectum	PRS7	rs6714609	2	169010250	G	A	-2.88E-04	PRS-CSx
Colorectum	PRS7	rs6714707	2	169010297	G	A	-4.70E-04	PRS-CSx
Colorectum	PRS7	rs6715570	2	215673440	T	C	1.03E-03	PRS-CSx
Colorectum	PRS7	rs6715655	2	225582284	A	G	1.89E-04	PRS-CSx
Colorectum	PRS7	rs6715692	2	183195681	T	C	3.66E-04	PRS-CSx
Colorectum	PRS7	rs6715813	2	95970366	G	A	-5.07E-05	PRS-CSx
Colorectum	PRS7	rs6716681	2	159859820	A	G	-3.75E-04	PRS-CSx
Colorectum	PRS7	rs6716875	2	108990797	A	G	-4.25E-04	PRS-CSx
Colorectum	PRS7	rs6716939	2	18282815	C	T	-1.82E-03	PRS-CSx
Colorectum	PRS7	rs6718569	2	59605011	C	T	8.83E-04	PRS-CSx
Colorectum	PRS7	rs6721068	2	107385240	C	T	5.22E-04	PRS-CSx
Colorectum	PRS7	rs6721338	2	107247100	A	G	4.43E-04	PRS-CSx
Colorectum	PRS7	rs6723361	2	42509829	T	C	-1.11E-04	PRS-CSx
Colorectum	PRS7	rs6724796	2	152412813	T	C	-4.45E-04	PRS-CSx
Colorectum	PRS7	rs672525	1	183162450	A	G	-1.67E-05	PRS-CSx
Colorectum	PRS7	rs6725821	2	95897767	T	C	-1.47E-04	PRS-CSx

Colorectum	PRS7	rs6726	3	122449657	G	A	-4.46E-04	PRS-CSx
Colorectum	PRS7	rs6726093	2	228651351	C	T	8.32E-04	PRS-CSx
Colorectum	PRS7	rs6726447	2	131820978	C	T	-3.72E-04	PRS-CSx
Colorectum	PRS7	rs6726882	2	143242870	A	G	1.51E-03	PRS-CSx
Colorectum	PRS7	rs6728684	2	26150773	G	T	-7.09E-04	PRS-CSx
Colorectum	PRS7	rs6729147	2	163639601	T	C	-2.31E-03	PRS-CSx
Colorectum	PRS7	rs6729656	2	455683	T	C	-1.10E-03	PRS-CSx
Colorectum	PRS7	rs6730171	2	204432604	C	T	1.68E-04	PRS-CSx
Colorectum	PRS7	rs6731100	2	225774390	T	C	-1.24E-03	PRS-CSx
Colorectum	PRS7	rs6731486	2	152410732	G	A	-3.23E-04	PRS-CSx
Colorectum	PRS7	rs6731785	2	225491143	A	G	1.26E-04	PRS-CSx
Colorectum	PRS7	rs6732543	2	28562703	T	C	1.14E-03	PRS-CSx
Colorectum	PRS7	rs6734194	2	153466691	T	G	-1.25E-03	PRS-CSx
Colorectum	PRS7	rs6735012	2	100516134	C	T	2.45E-04	PRS-CSx
Colorectum	PRS7	rs6735551	2	182039477	T	C	-1.09E-03	PRS-CSx
Colorectum	PRS7	rs6735824	2	205386242	G	A	-1.03E-03	PRS-CSx
Colorectum	PRS7	rs6736713	2	230366542	T	C	9.20E-04	PRS-CSx
Colorectum	PRS7	rs6737520	2	11692106	C	A	2.39E-03	PRS-CSx
Colorectum	PRS7	rs6737965	2	160036593	A	G	-2.43E-04	PRS-CSx
Colorectum	PRS7	rs6738420	2	159785940	T	C	-2.47E-04	PRS-CSx
Colorectum	PRS7	rs6738625	2	176544182	C	T	3.85E-04	PRS-CSx
Colorectum	PRS7	rs6739402	2	109001646	A	C	-5.02E-04	PRS-CSx
Colorectum	PRS7	rs6739735	2	225405253	C	T	1.06E-04	PRS-CSx
Colorectum	PRS7	rs6740492	2	169027423	C	T	-2.18E-04	PRS-CSx
Colorectum	PRS7	rs6742011	2	183462762	T	G	1.50E-04	PRS-CSx
Colorectum	PRS7	rs674306	11	74251828	A	C	-2.18E-04	PRS-CSx
Colorectum	PRS7	rs674362	9	9085811	A	C	3.74E-04	PRS-CSx
Colorectum	PRS7	rs6743945	2	107240268	T	C	3.83E-04	PRS-CSx
Colorectum	PRS7	rs6744779	2	42511903	A	G	-1.50E-04	PRS-CSx
Colorectum	PRS7	rs6747918	2	202097575	A	G	6.46E-04	PRS-CSx
Colorectum	PRS7	rs6748415	2	42496095	T	C	-1.26E-04	PRS-CSx
Colorectum	PRS7	rs6748967	2	95914095	G	A	-1.68E-04	PRS-CSx
Colorectum	PRS7	rs6749297	2	235493171	A	C	8.73E-04	PRS-CSx
Colorectum	PRS7	rs6749402	2	225443901	T	G	1.18E-04	PRS-CSx
Colorectum	PRS7	rs6750539	2	43037672	T	C	5.45E-04	PRS-CSx
Colorectum	PRS7	rs6750553	2	117064215	T	C	2.19E-03	PRS-CSx
Colorectum	PRS7	rs6752766	2	72188364	T	C	6.15E-04	PRS-CSx
Colorectum	PRS7	rs6752828	2	80187969	C	T	4.62E-04	PRS-CSx
Colorectum	PRS7	rs6752846	2	69740091	C	T	3.32E-05	PRS-CSx
Colorectum	PRS7	rs6754481	2	182623003	G	A	-6.77E-04	PRS-CSx
Colorectum	PRS7	rs675450	11	74217362	C	T	-1.15E-04	PRS-CSx
Colorectum	PRS7	rs6754934	2	159990664	A	G	-1.79E-03	PRS-CSx
Colorectum	PRS7	rs6756820	2	48679839	C	A	1.75E-04	PRS-CSx
Colorectum	PRS7	rs6758398	2	207138479	G	A	-1.69E-04	PRS-CSx
Colorectum	PRS7	rs675931	4	17123778	A	G	-4.69E-04	PRS-CSx
Colorectum	PRS7	rs6759347	2	8227802	C	A	-6.97E-04	PRS-CSx
Colorectum	PRS7	rs6760993	2	202110917	A	G	5.46E-04	PRS-CSx
Colorectum	PRS7	rs676147	18	10124648	G	A	-1.46E-03	PRS-CSx
Colorectum	PRS7	rs6762690	3	133896121	G	A	-6.49E-04	PRS-CSx
Colorectum	PRS7	rs676314	19	50865535	G	A	4.13E-04	PRS-CSx
Colorectum	PRS7	rs6763735	3	15362246	T	C	1.73E-03	PRS-CSx
Colorectum	PRS7	rs6766086	3	15410365	G	A	7.10E-04	PRS-CSx
Colorectum	PRS7	rs6768108	3	36998755	T	C	-1.95E-04	PRS-CSx
Colorectum	PRS7	rs6769604	3	113185064	C	T	-1.81E-04	PRS-CSx
Colorectum	PRS7	rs677011	3	101574503	A	G	7.44E-04	PRS-CSx
Colorectum	PRS7	rs6770152	3	53100214	T	G	9.34E-04	PRS-CSx

Colorectum	PRS7	rs6770546	3	168465419	A	G	-3.47E-04	PRS-CSx
Colorectum	PRS7	rs6773094	3	146853037	A	C	-4.80E-04	PRS-CSx
Colorectum	PRS7	rs6773439	3	15530595	G	A	-5.59E-04	PRS-CSx
Colorectum	PRS7	rs6774109	3	173497332	G	A	2.69E-04	PRS-CSx
Colorectum	PRS7	rs6774597	3	15356646	C	T	1.43E-03	PRS-CSx
Colorectum	PRS7	rs6774933	3	122448914	C	T	-4.10E-04	PRS-CSx
Colorectum	PRS7	rs6776123	3	24795619	C	T	-4.57E-04	PRS-CSx
Colorectum	PRS7	rs6776592	3	133870211	G	A	-2.57E-04	PRS-CSx
Colorectum	PRS7	rs6778307	3	133754267	C	T	-3.26E-03	PRS-CSx
Colorectum	PRS7	rs6779079	3	157409301	G	A	4.02E-04	PRS-CSx
Colorectum	PRS7	rs6779415	3	173563936	A	G	-2.92E-04	PRS-CSx
Colorectum	PRS7	rs6780295	3	133989068	A	G	-9.20E-04	PRS-CSx
Colorectum	PRS7	rs6780325	3	133803956	T	C	-5.14E-04	PRS-CSx
Colorectum	PRS7	rs6780752	3	142849380	T	C	4.35E-04	PRS-CSx
Colorectum	PRS7	rs6780799	3	37162758	A	G	-5.61E-05	PRS-CSx
Colorectum	PRS7	rs6782835	3	64541669	C	T	-1.83E-04	PRS-CSx
Colorectum	PRS7	rs678285	6	4111429	T	C	5.88E-04	PRS-CSx
Colorectum	PRS7	rs6783294	3	133905345	A	G	2.20E-04	PRS-CSx
Colorectum	PRS7	rs6784609	3	64597031	C	T	3.14E-04	PRS-CSx
Colorectum	PRS7	rs6784938	3	143817528	T	C	6.61E-04	PRS-CSx
Colorectum	PRS7	rs6784955	3	96346543	G	A	-1.16E-03	PRS-CSx
Colorectum	PRS7	rs6785206	3	128412024	A	G	1.55E-03	PRS-CSx
Colorectum	PRS7	rs6785284	3	188120643	T	C	-2.70E-04	PRS-CSx
Colorectum	PRS7	rs678560	18	33785093	G	A	5.78E-04	PRS-CSx
Colorectum	PRS7	rs6786374	3	170105233	C	T	-5.28E-04	PRS-CSx
Colorectum	PRS7	rs6786530	3	151607481	C	T	-1.22E-03	PRS-CSx
Colorectum	PRS7	rs6787449	3	67905314	A	G	-5.85E-04	PRS-CSx
Colorectum	PRS7	rs6787539	3	37164647	A	G	-2.89E-05	PRS-CSx
Colorectum	PRS7	rs6787588	3	73434086	G	A	-1.34E-03	PRS-CSx
Colorectum	PRS7	rs6790513	3	28034296	C	T	5.92E-04	PRS-CSx
Colorectum	PRS7	rs6790910	3	113096445	C	T	-3.05E-03	PRS-CSx
Colorectum	PRS7	rs6792572	3	119636096	A	C	2.09E-04	PRS-CSx
Colorectum	PRS7	rs6793110	3	66444615	A	G	6.00E-04	PRS-CSx
Colorectum	PRS7	rs6793635	3	15351965	T	C	1.32E-03	PRS-CSx
Colorectum	PRS7	rs6793667	3	41311387	A	G	-8.23E-05	PRS-CSx
Colorectum	PRS7	rs6793907	3	128391626	C	T	1.35E-03	PRS-CSx
Colorectum	PRS7	rs6794287	3	142602666	T	C	5.02E-04	PRS-CSx
Colorectum	PRS7	rs6795653	3	119610565	T	C	7.81E-05	PRS-CSx
Colorectum	PRS7	rs6797197	3	167537664	G	A	7.92E-04	PRS-CSx
Colorectum	PRS7	rs6797209	3	182430065	G	A	8.36E-04	PRS-CSx
Colorectum	PRS7	rs6797815	3	122533517	G	A	-4.30E-04	PRS-CSx
Colorectum	PRS7	rs6798961	3	15160002	C	A	6.99E-04	PRS-CSx
Colorectum	PRS7	rs6798972	3	146841078	A	C	-6.10E-04	PRS-CSx
Colorectum	PRS7	rs6800143	3	133844946	G	A	-6.81E-04	PRS-CSx
Colorectum	PRS7	rs6800622	3	119580678	C	A	1.02E-04	PRS-CSx
Colorectum	PRS7	rs6800891	3	24251816	A	G	4.94E-03	PRS-CSx
Colorectum	PRS7	rs6801610	3	142848367	G	A	4.81E-04	PRS-CSx
Colorectum	PRS7	rs6802472	3	71036994	A	G	-2.32E-03	PRS-CSx
Colorectum	PRS7	rs6802512	3	73708021	A	C	-1.72E-04	PRS-CSx
Colorectum	PRS7	rs6802863	3	64603581	T	C	8.33E-04	PRS-CSx
Colorectum	PRS7	rs6804266	3	10600095	A	G	-5.69E-03	PRS-CSx
Colorectum	PRS7	rs6805213	3	169448752	A	G	5.99E-05	PRS-CSx
Colorectum	PRS7	rs6805251	3	119560606	C	T	1.11E-04	PRS-CSx
Colorectum	PRS7	rs6806333	3	122578119	T	G	-1.91E-04	PRS-CSx
Colorectum	PRS7	rs6809441	3	41494605	C	T	-3.16E-04	PRS-CSx
Colorectum	PRS7	rs6809621	3	175002708	T	C	1.11E-03	PRS-CSx

Colorectum	PRS7	rs6812305	4	16396682	A	G	-1.44E-04	PRS-CSx
Colorectum	PRS7	rs6812904	4	149196991	A	G	8.55E-04	PRS-CSx
Colorectum	PRS7	rs6813229	4	115503877	C	A	2.76E-04	PRS-CSx
Colorectum	PRS7	rs6813248	4	94830161	C	T	1.27E-04	PRS-CSx
Colorectum	PRS7	rs6813283	4	115503962	C	A	2.44E-04	PRS-CSx
Colorectum	PRS7	rs6813885	4	39045907	T	C	7.49E-05	PRS-CSx
Colorectum	PRS7	rs681573	3	101584861	C	T	7.61E-04	PRS-CSx
Colorectum	PRS7	rs6815946	4	995305	C	T	-2.75E-03	PRS-CSx
Colorectum	PRS7	rs6816196	4	146115364	A	C	4.93E-04	PRS-CSx
Colorectum	PRS7	rs6817766	4	90045776	T	G	3.24E-04	PRS-CSx
Colorectum	PRS7	rs6818267	4	151350823	A	C	7.14E-05	PRS-CSx
Colorectum	PRS7	rs6818437	4	94802707	C	T	3.18E-04	PRS-CSx
Colorectum	PRS7	rs6819328	4	89137318	C	T	2.98E-03	PRS-CSx
Colorectum	PRS7	rs6820812	4	95622462	C	A	1.77E-03	PRS-CSx
Colorectum	PRS7	rs6821728	4	167549840	T	C	2.34E-04	PRS-CSx
Colorectum	PRS7	rs682251	9	271455	T	C	-4.59E-04	PRS-CSx
Colorectum	PRS7	rs6823076	4	82986961	C	T	2.49E-04	PRS-CSx
Colorectum	PRS7	rs6823186	4	123325969	T	C	4.17E-04	PRS-CSx
Colorectum	PRS7	rs682573	13	42708655	G	T	4.02E-04	PRS-CSx
Colorectum	PRS7	rs6826155	4	14280826	A	G	1.40E-03	PRS-CSx
Colorectum	PRS7	rs6827776	4	175389785	A	G	3.36E-04	PRS-CSx
Colorectum	PRS7	rs6828137	4	90059434	G	T	5.59E-05	PRS-CSx
Colorectum	PRS7	rs6828598	4	39261470	A	G	-1.08E-04	PRS-CSx
Colorectum	PRS7	rs682915	11	86383994	C	T	2.41E-04	PRS-CSx
Colorectum	PRS7	rs6829932	4	146478467	T	C	3.68E-04	PRS-CSx
Colorectum	PRS7	rs6830470	4	153840118	C	T	-2.57E-04	PRS-CSx
Colorectum	PRS7	rs6831280	4	996165	A	G	-1.10E-03	PRS-CSx
Colorectum	PRS7	rs6831582	4	130206511	T	G	-3.94E-04	PRS-CSx
Colorectum	PRS7	rs6831700	4	39256964	G	T	-5.38E-05	PRS-CSx
Colorectum	PRS7	rs6835031	4	89995425	G	A	-2.26E-04	PRS-CSx
Colorectum	PRS7	rs683633	5	72400190	C	T	-1.05E-04	PRS-CSx
Colorectum	PRS7	rs6836928	4	130214024	A	G	-1.57E-04	PRS-CSx
Colorectum	PRS7	rs683756	5	134550777	G	A	3.38E-05	PRS-CSx
Colorectum	PRS7	rs6839599	4	115415910	A	G	3.42E-04	PRS-CSx
Colorectum	PRS7	rs6839694	4	115423197	A	G	8.41E-05	PRS-CSx
Colorectum	PRS7	rs6840214	4	39120619	A	G	1.25E-04	PRS-CSx
Colorectum	PRS7	rs6840354	4	23694492	G	A	-6.62E-05	PRS-CSx
Colorectum	PRS7	rs6840978	4	123554707	T	C	8.34E-04	PRS-CSx
Colorectum	PRS7	rs684232	17	618965	T	C	7.21E-04	PRS-CSx
Colorectum	PRS7	rs6846082	4	151419788	A	G	-3.49E-04	PRS-CSx
Colorectum	PRS7	rs6847753	4	129774375	A	G	-1.40E-03	PRS-CSx
Colorectum	PRS7	rs6848107	4	86879664	C	T	-6.12E-04	PRS-CSx
Colorectum	PRS7	rs6848284	4	153708658	A	G	-2.94E-04	PRS-CSx
Colorectum	PRS7	rs6848760	4	146484997	A	C	4.56E-04	PRS-CSx
Colorectum	PRS7	rs6849143	4	89928489	C	T	3.94E-04	PRS-CSx
Colorectum	PRS7	rs6850952	4	105792576	T	G	3.65E-04	PRS-CSx
Colorectum	PRS7	rs6851217	4	99774466	T	C	2.85E-04	PRS-CSx
Colorectum	PRS7	rs685125	19	39850102	C	T	1.73E-04	PRS-CSx
Colorectum	PRS7	rs6851372	4	99774378	A	G	5.33E-04	PRS-CSx
Colorectum	PRS7	rs6852200	4	146452773	C	T	7.99E-04	PRS-CSx
Colorectum	PRS7	rs6852928	4	89926193	T	C	2.68E-04	PRS-CSx
Colorectum	PRS7	rs6856472	4	27871868	T	C	1.75E-04	PRS-CSx
Colorectum	PRS7	rs6857717	4	27872339	A	G	4.16E-05	PRS-CSx
Colorectum	PRS7	rs6860105	5	88348122	A	G	5.01E-04	PRS-CSx
Colorectum	PRS7	rs686030	9	15304782	C	A	-2.38E-03	PRS-CSx
Colorectum	PRS7	rs6861486	5	151049775	T	C	3.39E-04	PRS-CSx

Colorectum	PRS7	rs686173	2	4470836	C	A	-5.54E-04	PRS-CSx
Colorectum	PRS7	rs6863228	5	68104648	A	G	9.85E-04	PRS-CSx
Colorectum	PRS7	rs6864481	5	129315115	A	G	-1.31E-04	PRS-CSx
Colorectum	PRS7	rs6865476	5	40025084	T	G	2.61E-04	PRS-CSx
Colorectum	PRS7	rs6866344	5	178121599	G	T	2.17E-04	PRS-CSx
Colorectum	PRS7	rs6866671	5	159436823	C	A	4.35E-04	PRS-CSx
Colorectum	PRS7	rs6867144	5	88349622	G	A	3.91E-04	PRS-CSx
Colorectum	PRS7	rs6868517	5	40752536	A	G	4.48E-04	PRS-CSx
Colorectum	PRS7	rs6869827	5	129323187	A	G	-7.91E-05	PRS-CSx
Colorectum	PRS7	rs6870197	5	1653418	T	C	1.15E-03	PRS-CSx
Colorectum	PRS7	rs6871139	5	172322142	C	T	-4.20E-04	PRS-CSx
Colorectum	PRS7	rs6872302	5	174338352	A	G	8.28E-04	PRS-CSx
Colorectum	PRS7	rs6873063	5	150187805	A	C	3.12E-04	PRS-CSx
Colorectum	PRS7	rs6873656	5	67296790	A	G	2.13E-04	PRS-CSx
Colorectum	PRS7	rs6874344	5	134244534	A	G	3.69E-03	PRS-CSx
Colorectum	PRS7	rs6874435	5	75574855	G	A	-2.29E-03	PRS-CSx
Colorectum	PRS7	rs6874438	5	40069162	T	C	3.76E-04	PRS-CSx
Colorectum	PRS7	rs6874509	5	128426159	T	C	-5.60E-04	PRS-CSx
Colorectum	PRS7	rs6875213	5	8655185	A	G	-1.77E-04	PRS-CSx
Colorectum	PRS7	rs687553	9	97289359	T	C	-4.89E-04	PRS-CSx
Colorectum	PRS7	rs6876969	5	39829284	G	T	-2.85E-04	PRS-CSx
Colorectum	PRS7	rs6877546	5	133845112	T	C	1.84E-04	PRS-CSx
Colorectum	PRS7	rs6878100	5	129342305	A	G	-1.61E-04	PRS-CSx
Colorectum	PRS7	rs6878443	5	134491443	A	G	2.45E-03	PRS-CSx
Colorectum	PRS7	rs6879439	5	169314869	C	T	3.63E-04	PRS-CSx
Colorectum	PRS7	rs688058	3	101585467	T	G	7.72E-04	PRS-CSx
Colorectum	PRS7	rs6881033	5	67529191	G	A	-2.92E-04	PRS-CSx
Colorectum	PRS7	rs6882015	5	125937190	A	G	-4.43E-04	PRS-CSx
Colorectum	PRS7	rs688325	1	147077283	G	A	-7.51E-05	PRS-CSx
Colorectum	PRS7	rs6884344	5	40023248	A	G	-1.31E-04	PRS-CSx
Colorectum	PRS7	rs6888154	5	150260382	G	T	2.68E-04	PRS-CSx
Colorectum	PRS7	rs6893009	5	150233304	C	A	1.40E-04	PRS-CSx
Colorectum	PRS7	rs6894520	5	81594748	T	C	2.13E-04	PRS-CSx
Colorectum	PRS7	rs6895329	5	39825740	C	T	6.67E-05	PRS-CSx
Colorectum	PRS7	rs6897038	5	33632717	T	C	-7.64E-04	PRS-CSx
Colorectum	PRS7	rs6897597	5	131743465	T	C	1.22E-03	PRS-CSx
Colorectum	PRS7	rs6898994	5	40000651	A	G	3.12E-04	PRS-CSx
Colorectum	PRS7	rs6899384	6	39132435	T	C	-2.56E-04	PRS-CSx
Colorectum	PRS7	rs6901575	6	28250984	A	G	-2.49E-04	PRS-CSx
Colorectum	PRS7	rs690260	21	46173391	G	A	9.31E-05	PRS-CSx
Colorectum	PRS7	rs6902999	6	105759546	T	C	-2.58E-04	PRS-CSx
Colorectum	PRS7	rs6903066	6	131332166	T	G	1.42E-04	PRS-CSx
Colorectum	PRS7	rs6903956	6	11774583	A	G	3.12E-04	PRS-CSx
Colorectum	PRS7	rs6904029	6	29943067	A	G	-2.46E-04	PRS-CSx
Colorectum	PRS7	rs6904320	6	32312082	A	G	5.09E-04	PRS-CSx
Colorectum	PRS7	rs6904837	6	116883953	T	C	-6.17E-04	PRS-CSx
Colorectum	PRS7	rs6904966	6	14197619	A	G	-7.72E-04	PRS-CSx
Colorectum	PRS7	rs6905856	6	4927010	T	C	-6.80E-04	PRS-CSx
Colorectum	PRS7	rs6906128	6	32206304	C	A	-7.49E-05	PRS-CSx
Colorectum	PRS7	rs6906307	6	29463892	G	T	1.63E-04	PRS-CSx
Colorectum	PRS7	rs6906327	6	20659459	A	G	-2.19E-04	PRS-CSx
Colorectum	PRS7	rs6906897	6	29415636	T	C	8.21E-05	PRS-CSx
Colorectum	PRS7	rs6907678	6	35418954	T	C	1.13E-03	PRS-CSx
Colorectum	PRS7	rs6908010	6	12217999	T	G	5.45E-04	PRS-CSx
Colorectum	PRS7	rs6908183	6	23881151	G	A	-3.33E-04	PRS-CSx
Colorectum	PRS7	rs6908544	6	23895019	C	T	-2.29E-04	PRS-CSx

Colorectum	PRS7	rs6911639	6	32978178	C	T	6.21E-04	PRS-CSx
Colorectum	PRS7	rs6911915	6	117809031	C	T	2.27E-03	PRS-CSx
Colorectum	PRS7	rs6914525	6	133988652	A	G	3.69E-04	PRS-CSx
Colorectum	PRS7	rs6914787	6	21443902	C	T	-8.05E-04	PRS-CSx
Colorectum	PRS7	rs6914901	6	156980007	A	G	-1.19E-03	PRS-CSx
Colorectum	PRS7	rs6915177	6	29522588	T	C	3.16E-04	PRS-CSx
Colorectum	PRS7	rs6916422	6	29938110	T	C	-4.70E-04	PRS-CSx
Colorectum	PRS7	rs6918854	6	26577924	G	A	-1.26E-05	PRS-CSx
Colorectum	PRS7	rs6919440	6	43352898	G	A	-1.83E-04	PRS-CSx
Colorectum	PRS7	rs6919504	6	156976650	G	A	-2.65E-03	PRS-CSx
Colorectum	PRS7	rs6920115	6	26988417	C	T	-2.09E-04	PRS-CSx
Colorectum	PRS7	rs6920338	6	32288089	C	T	-1.12E-05	PRS-CSx
Colorectum	PRS7	rs6921861	6	24225296	A	G	-4.15E-04	PRS-CSx
Colorectum	PRS7	rs6921943	6	11961970	C	A	5.96E-04	PRS-CSx
Colorectum	PRS7	rs6922758	6	98318226	C	T	3.78E-04	PRS-CSx
Colorectum	PRS7	rs6924184	6	24151395	T	C	-4.89E-04	PRS-CSx
Colorectum	PRS7	rs6925408	6	29446570	G	A	1.56E-04	PRS-CSx
Colorectum	PRS7	rs6925744	6	29446689	T	C	1.12E-03	PRS-CSx
Colorectum	PRS7	rs6925922	6	116954069	C	T	-1.75E-04	PRS-CSx
Colorectum	PRS7	rs6926506	6	29463968	A	C	3.80E-04	PRS-CSx
Colorectum	PRS7	rs6926578	6	142074024	G	A	-1.28E-03	PRS-CSx
Colorectum	PRS7	rs6926933	6	136232680	G	A	1.50E-03	PRS-CSx
Colorectum	PRS7	rs6927567	6	116950734	A	G	-9.95E-05	PRS-CSx
Colorectum	PRS7	rs6929400	6	11774792	C	T	1.21E-04	PRS-CSx
Colorectum	PRS7	rs6929774	6	33562720	T	C	3.54E-04	PRS-CSx
Colorectum	PRS7	rs6930053	6	45488758	T	C	-1.26E-03	PRS-CSx
Colorectum	PRS7	rs6930292	6	117783770	T	G	1.72E-04	PRS-CSx
Colorectum	PRS7	rs6931175	6	121317076	A	G	-2.07E-03	PRS-CSx
Colorectum	PRS7	rs6931262	6	7217517	T	C	-9.98E-04	PRS-CSx
Colorectum	PRS7	rs6931291	6	141329638	C	A	-9.50E-04	PRS-CSx
Colorectum	PRS7	rs6931702	6	29507571	A	G	5.87E-04	PRS-CSx
Colorectum	PRS7	rs6932992	6	76573743	A	G	-3.42E-04	PRS-CSx
Colorectum	PRS7	rs6933331	6	29495280	A	G	3.88E-04	PRS-CSx
Colorectum	PRS7	rs693342	9	113632279	A	C	-2.79E-04	PRS-CSx
Colorectum	PRS7	rs6933601	6	29407602	A	G	2.29E-04	PRS-CSx
Colorectum	PRS7	rs6934009	6	142156294	G	A	-6.11E-04	PRS-CSx
Colorectum	PRS7	rs6934302	6	39106276	C	A	-2.50E-03	PRS-CSx
Colorectum	PRS7	rs6935708	6	29416652	C	T	5.75E-04	PRS-CSx
Colorectum	PRS7	rs6936346	6	32200754	T	C	-2.19E-04	PRS-CSx
Colorectum	PRS7	rs693669	6	122786835	T	C	5.60E-04	PRS-CSx
Colorectum	PRS7	rs6936993	6	36626322	T	C	-2.48E-04	PRS-CSx
Colorectum	PRS7	rs6937955	6	26962934	T	C	-2.56E-04	PRS-CSx
Colorectum	PRS7	rs6938586	6	131209404	C	T	2.59E-04	PRS-CSx
Colorectum	PRS7	rs693918	2	31868877	G	A	-2.12E-04	PRS-CSx
Colorectum	PRS7	rs6939861	6	41703041	A	G	-7.35E-03	PRS-CSx
Colorectum	PRS7	rs6941022	6	26553531	T	C	-3.33E-05	PRS-CSx
Colorectum	PRS7	rs6942982	7	105318058	A	G	2.83E-03	PRS-CSx
Colorectum	PRS7	rs6945096	7	151115691	C	T	-9.70E-04	PRS-CSx
Colorectum	PRS7	rs6947303	7	45108972	G	A	-4.66E-04	PRS-CSx
Colorectum	PRS7	rs6949013	7	93706220	C	T	4.52E-04	PRS-CSx
Colorectum	PRS7	rs6951528	7	150809164	C	A	4.08E-04	PRS-CSx
Colorectum	PRS7	rs6952573	7	124212465	G	A	5.56E-04	PRS-CSx
Colorectum	PRS7	rs6953826	7	45117538	A	G	-4.11E-04	PRS-CSx
Colorectum	PRS7	rs6954585	7	41922310	C	T	7.48E-04	PRS-CSx
Colorectum	PRS7	rs695648	22	43760187	C	T	5.49E-04	PRS-CSx
Colorectum	PRS7	rs695810	22	43762357	T	C	-7.40E-04	PRS-CSx

Colorectum	PRS7	rs6958168	7	45019814	G	T	-6.06E-04	PRS-CSx
Colorectum	PRS7	rs6961921	7	22046923	G	A	1.24E-03	PRS-CSx
Colorectum	PRS7	rs6963345	7	99618606	A	G	3.43E-04	PRS-CSx
Colorectum	PRS7	rs6963647	7	96170054	C	T	-7.75E-04	PRS-CSx
Colorectum	PRS7	rs6965201	7	92734451	A	G	3.74E-04	PRS-CSx
Colorectum	PRS7	rs6965245	7	77329743	T	C	-3.85E-04	PRS-CSx
Colorectum	PRS7	rs6966840	7	151112726	A	G	-7.09E-04	PRS-CSx
Colorectum	PRS7	rs6967523	7	108415940	A	G	9.30E-04	PRS-CSx
Colorectum	PRS7	rs6968302	7	36116073	G	A	1.96E-03	PRS-CSx
Colorectum	PRS7	rs6969691	7	92734983	G	A	-4.97E-04	PRS-CSx
Colorectum	PRS7	rs697027	12	77334817	A	G	-4.09E-04	PRS-CSx
Colorectum	PRS7	rs6970531	7	88859261	G	A	-2.96E-04	PRS-CSx
Colorectum	PRS7	rs6970698	7	88859094	A	G	-2.61E-04	PRS-CSx
Colorectum	PRS7	rs6970769	7	88859341	T	C	-3.07E-04	PRS-CSx
Colorectum	PRS7	rs6971403	7	45103771	T	C	-5.45E-04	PRS-CSx
Colorectum	PRS7	rs6972359	7	135310205	G	A	1.80E-04	PRS-CSx
Colorectum	PRS7	rs6973656	7	77422583	G	A	-2.57E-04	PRS-CSx
Colorectum	PRS7	rs6973982	7	45143892	G	A	-3.27E-04	PRS-CSx
Colorectum	PRS7	rs6974494	7	83743961	C	T	-5.44E-04	PRS-CSx
Colorectum	PRS7	rs697845	1	226817556	T	C	4.03E-04	PRS-CSx
Colorectum	PRS7	rs6978601	7	28340264	A	G	1.48E-03	PRS-CSx
Colorectum	PRS7	rs697862	12	116250030	T	C	-3.93E-04	PRS-CSx
Colorectum	PRS7	rs6979322	7	41932014	C	T	7.51E-04	PRS-CSx
Colorectum	PRS7	rs6980502	8	30694153	G	A	1.55E-04	PRS-CSx
Colorectum	PRS7	rs6980504	8	117680269	T	C	-1.77E-04	PRS-CSx
Colorectum	PRS7	rs6981389	8	136068182	G	A	7.20E-04	PRS-CSx
Colorectum	PRS7	rs6981928	8	117823255	A	G	4.89E-04	PRS-CSx
Colorectum	PRS7	rs6982631	8	13530101	C	T	5.17E-04	PRS-CSx
Colorectum	PRS7	rs6983235	8	117906006	A	G	-2.23E-04	PRS-CSx
Colorectum	PRS7	rs6983267	8	128413305	G	T	9.32E-03	PRS-CSx
Colorectum	PRS7	rs6983626	8	117802148	T	C	1.04E-03	PRS-CSx
Colorectum	PRS7	rs6983992	8	18728635	C	T	6.06E-04	PRS-CSx
Colorectum	PRS7	rs6984136	8	128320138	T	C	-8.06E-04	PRS-CSx
Colorectum	PRS7	rs6985419	8	128429721	T	C	2.29E-03	PRS-CSx
Colorectum	PRS7	rs6990281	8	120180777	T	C	-1.62E-04	PRS-CSx
Colorectum	PRS7	rs6992462	8	59763797	A	G	-2.53E-03	PRS-CSx
Colorectum	PRS7	rs6993	16	58741367	G	A	1.71E-04	PRS-CSx
Colorectum	PRS7	rs6993678	8	120183910	T	G	-5.94E-05	PRS-CSx
Colorectum	PRS7	rs6993726	8	123444650	T	C	1.89E-04	PRS-CSx
Colorectum	PRS7	rs6994692	8	142239377	A	C	-1.65E-03	PRS-CSx
Colorectum	PRS7	rs6995633	8	128440651	A	G	7.76E-04	PRS-CSx
Colorectum	PRS7	rs6996354	8	78376314	C	T	3.87E-04	PRS-CSx
Colorectum	PRS7	rs6996613	8	83821211	T	C	-2.13E-04	PRS-CSx
Colorectum	PRS7	rs6997499	8	49190984	C	A	1.06E-03	PRS-CSx
Colorectum	PRS7	rs6999093	8	4322748	T	C	-6.67E-04	PRS-CSx
Colorectum	PRS7	rs6999643	8	117909375	G	A	-3.14E-04	PRS-CSx
Colorectum	PRS7	rs7002672	8	65606916	G	A	-3.26E-04	PRS-CSx
Colorectum	PRS7	rs7002898	8	13492542	G	A	-1.26E-03	PRS-CSx
Colorectum	PRS7	rs700309	7	147093040	T	G	-4.12E-04	PRS-CSx
Colorectum	PRS7	rs7003790	8	59105992	T	C	-2.72E-03	PRS-CSx
Colorectum	PRS7	rs7004242	8	29105528	T	C	2.45E-04	PRS-CSx
Colorectum	PRS7	rs7005484	8	41375246	A	C	8.90E-04	PRS-CSx
Colorectum	PRS7	rs7005829	8	128434944	T	C	2.20E-04	PRS-CSx
Colorectum	PRS7	rs700604	5	120713370	A	G	5.14E-04	PRS-CSx
Colorectum	PRS7	rs7007998	8	121095103	A	G	-2.49E-04	PRS-CSx
Colorectum	PRS7	rs7008596	8	123446887	T	C	2.15E-04	PRS-CSx

Colorectum	PRS7	rs7009635	8	141594881	C	T	1.35E-03	PRS-CSx
Colorectum	PRS7	rs7009885	8	29124351	A	G	8.64E-04	PRS-CSx
Colorectum	PRS7	rs7011507	8	49129242	A	G	6.85E-04	PRS-CSx
Colorectum	PRS7	rs7013654	8	14444701	T	G	-1.28E-03	PRS-CSx
Colorectum	PRS7	rs7014054	8	41368500	G	A	1.26E-03	PRS-CSx
Colorectum	PRS7	rs7014328	8	117799487	C	A	1.31E-03	PRS-CSx
Colorectum	PRS7	rs7014346	8	128424792	A	G	1.99E-03	PRS-CSx
Colorectum	PRS7	rs7014358	8	117799586	A	G	1.31E-03	PRS-CSx
Colorectum	PRS7	rs7014359	8	117799587	A	C	1.40E-03	PRS-CSx
Colorectum	PRS7	rs701580	13	110242391	T	C	6.54E-04	PRS-CSx
Colorectum	PRS7	rs7017038	8	108716769	T	G	-5.53E-04	PRS-CSx
Colorectum	PRS7	rs7017464	8	59746240	C	A	-5.95E-04	PRS-CSx
Colorectum	PRS7	rs7018718	9	19302836	G	A	2.99E-04	PRS-CSx
Colorectum	PRS7	rs7019647	9	33893073	G	A	-3.95E-04	PRS-CSx
Colorectum	PRS7	rs702061	12	77335025	A	G	-4.39E-04	PRS-CSx
Colorectum	PRS7	rs7021722	9	87208419	C	T	-5.80E-04	PRS-CSx
Colorectum	PRS7	rs7023634	9	107731789	G	A	-4.18E-05	PRS-CSx
Colorectum	PRS7	rs702489	1	54197688	A	G	3.40E-03	PRS-CSx
Colorectum	PRS7	rs7025486	9	124422403	A	G	-4.81E-04	PRS-CSx
Colorectum	PRS7	rs7027110	9	109599046	A	G	-1.89E-04	PRS-CSx
Colorectum	PRS7	rs7027205	9	109590148	C	T	-3.02E-04	PRS-CSx
Colorectum	PRS7	rs7031164	9	104605562	T	C	8.09E-04	PRS-CSx
Colorectum	PRS7	rs7031588	9	101822302	C	T	3.91E-04	PRS-CSx
Colorectum	PRS7	rs7032586	9	114377921	A	G	-5.48E-04	PRS-CSx
Colorectum	PRS7	rs703327	2	19452022	T	C	3.53E-04	PRS-CSx
Colorectum	PRS7	rs7034847	9	101776106	C	T	-2.50E-04	PRS-CSx
Colorectum	PRS7	rs703704	12	101273368	G	A	-7.56E-05	PRS-CSx
Colorectum	PRS7	rs7037176	9	16514375	A	C	-5.62E-04	PRS-CSx
Colorectum	PRS7	rs7037232	9	34587485	T	C	4.00E-04	PRS-CSx
Colorectum	PRS7	rs7038553	9	33890932	G	A	-3.36E-04	PRS-CSx
Colorectum	PRS7	rs7041637	9	21961866	A	C	-5.46E-05	PRS-CSx
Colorectum	PRS7	rs7043344	9	19253435	C	T	2.71E-04	PRS-CSx
Colorectum	PRS7	rs7044116	9	109595771	G	A	-4.27E-04	PRS-CSx
Colorectum	PRS7	rs704417	3	64252424	C	T	-8.53E-03	PRS-CSx
Colorectum	PRS7	rs7044318	9	34554999	T	C	5.09E-04	PRS-CSx
Colorectum	PRS7	rs704480	6	57091166	G	A	-2.76E-04	PRS-CSx
Colorectum	PRS7	rs7047127	9	635677	T	C	3.22E-04	PRS-CSx
Colorectum	PRS7	rs704730	11	34686005	A	G	-1.85E-03	PRS-CSx
Colorectum	PRS7	rs704736	11	34695895	G	A	-6.05E-04	PRS-CSx
Colorectum	PRS7	rs7047374	9	139797906	T	C	-2.68E-05	PRS-CSx
Colorectum	PRS7	rs7047526	9	138068124	G	T	9.14E-04	PRS-CSx
Colorectum	PRS7	rs7048659	9	117533289	C	T	3.20E-04	PRS-CSx
Colorectum	PRS7	rs7048827	9	105459702	T	G	2.51E-04	PRS-CSx
Colorectum	PRS7	rs7049105	9	22028801	A	G	3.26E-04	PRS-CSx
Colorectum	PRS7	rs705067	2	129293028	C	A	-8.07E-04	PRS-CSx
Colorectum	PRS7	rs705956	6	158870081	G	A	9.13E-05	PRS-CSx
Colorectum	PRS7	rs705958	6	158866510	C	T	1.86E-04	PRS-CSx
Colorectum	PRS7	rs706232	5	11038666	T	C	6.10E-05	PRS-CSx
Colorectum	PRS7	rs706363	1	57493551	C	T	-3.24E-03	PRS-CSx
Colorectum	PRS7	rs7067601	10	70063970	C	T	-2.04E-04	PRS-CSx
Colorectum	PRS7	rs706771	10	8696446	A	G	-1.88E-03	PRS-CSx
Colorectum	PRS7	rs706779	10	6098824	C	T	-2.79E-05	PRS-CSx
Colorectum	PRS7	rs706781	10	6086385	C	T	4.81E-05	PRS-CSx
Colorectum	PRS7	rs7068684	10	106403115	T	C	1.14E-03	PRS-CSx
Colorectum	PRS7	rs7069128	10	111760075	G	A	-2.49E-04	PRS-CSx
Colorectum	PRS7	rs7069870	10	8695637	G	A	9.31E-04	PRS-CSx

Colorectum	PRS7	rs7070020	10	33398036	A	G	8.48E-04	PRS-CSx
Colorectum	PRS7	rs7070167	10	33398128	A	G	7.48E-04	PRS-CSx
Colorectum	PRS7	rs7070343	10	33398251	T	G	8.12E-04	PRS-CSx
Colorectum	PRS7	rs707107	2	155322696	T	G	5.29E-04	PRS-CSx
Colorectum	PRS7	rs7071509	10	111775849	A	G	-3.60E-04	PRS-CSx
Colorectum	PRS7	rs7073323	10	104797423	C	A	-7.74E-05	PRS-CSx
Colorectum	PRS7	rs7074395	10	104844928	T	G	9.30E-05	PRS-CSx
Colorectum	PRS7	rs7075267	10	8831705	C	T	1.92E-04	PRS-CSx
Colorectum	PRS7	rs7075305	10	101364524	C	T	6.25E-04	PRS-CSx
Colorectum	PRS7	rs7076753	10	2401207	G	A	6.26E-04	PRS-CSx
Colorectum	PRS7	rs7077492	10	54717446	T	C	-2.56E-03	PRS-CSx
Colorectum	PRS7	rs7078182	10	95523396	G	A	-9.63E-04	PRS-CSx
Colorectum	PRS7	rs7078219	10	101274365	G	A	-1.04E-03	PRS-CSx
Colorectum	PRS7	rs7078551	10	25174726	T	C	1.36E-03	PRS-CSx
Colorectum	PRS7	rs7079570	10	50308187	T	C	-9.09E-04	PRS-CSx
Colorectum	PRS7	rs7081062	10	114740745	G	A	-2.78E-04	PRS-CSx
Colorectum	PRS7	rs7081330	10	101274465	A	G	-3.24E-04	PRS-CSx
Colorectum	PRS7	rs7082178	10	80834958	A	G	-8.63E-04	PRS-CSx
Colorectum	PRS7	rs7083908	10	74003722	C	A	-3.72E-04	PRS-CSx
Colorectum	PRS7	rs7084597	10	70076490	C	T	-5.99E-04	PRS-CSx
Colorectum	PRS7	rs7084834	10	106403209	C	T	1.17E-03	PRS-CSx
Colorectum	PRS7	rs7085958	10	73755778	A	G	-2.30E-04	PRS-CSx
Colorectum	PRS7	rs7086249	10	25811435	T	C	-6.02E-04	PRS-CSx
Colorectum	PRS7	rs708686	19	5840619	T	C	4.28E-03	PRS-CSx
Colorectum	PRS7	rs7087268	10	95524801	A	G	-6.47E-04	PRS-CSx
Colorectum	PRS7	rs7087529	10	125711882	A	G	-1.38E-03	PRS-CSx
Colorectum	PRS7	rs7087762	10	74900121	T	C	-5.41E-04	PRS-CSx
Colorectum	PRS7	rs7088203	10	73946588	G	A	-5.78E-04	PRS-CSx
Colorectum	PRS7	rs708822	12	116252880	A	G	-7.16E-04	PRS-CSx
Colorectum	PRS7	rs708840	12	116270701	G	A	-3.88E-04	PRS-CSx
Colorectum	PRS7	rs7088921	10	8796848	A	G	2.32E-04	PRS-CSx
Colorectum	PRS7	rs7089680	10	104802071	C	T	-8.64E-05	PRS-CSx
Colorectum	PRS7	rs7090904	10	116775885	T	G	1.55E-03	PRS-CSx
Colorectum	PRS7	rs7091572	10	101327851	C	T	3.79E-04	PRS-CSx
Colorectum	PRS7	rs7092009	10	101277239	A	G	-5.73E-04	PRS-CSx
Colorectum	PRS7	rs7092052	10	123615028	A	G	1.23E-03	PRS-CSx
Colorectum	PRS7	rs7092725	10	90839450	C	T	2.89E-04	PRS-CSx
Colorectum	PRS7	rs709284	12	122631752	T	C	7.05E-04	PRS-CSx
Colorectum	PRS7	rs709398	4	186929226	C	T	-9.27E-04	PRS-CSx
Colorectum	PRS7	rs7094463	10	114711983	G	A	4.74E-04	PRS-CSx
Colorectum	PRS7	rs7095491	10	101274058	T	C	-6.14E-04	PRS-CSx
Colorectum	PRS7	rs7095711	10	8507223	G	A	-4.46E-03	PRS-CSx
Colorectum	PRS7	rs7095953	10	101274425	C	T	3.53E-04	PRS-CSx
Colorectum	PRS7	rs7096296	10	101277816	C	T	-8.91E-04	PRS-CSx
Colorectum	PRS7	rs7098207	10	8686095	C	T	4.18E-04	PRS-CSx
Colorectum	PRS7	rs7098233	10	91422868	T	G	-6.64E-04	PRS-CSx
Colorectum	PRS7	rs7099028	10	7532999	A	C	-5.27E-04	PRS-CSx
Colorectum	PRS7	rs7099380	10	18549016	G	A	6.13E-04	PRS-CSx
Colorectum	PRS7	rs7100942	10	25823753	C	A	-5.11E-04	PRS-CSx
Colorectum	PRS7	rs7101374	11	46824241	A	G	2.05E-04	PRS-CSx
Colorectum	PRS7	rs7101916	11	65431360	T	C	4.06E-04	PRS-CSx
Colorectum	PRS7	rs7102303	11	95441807	T	C	-5.27E-04	PRS-CSx
Colorectum	PRS7	rs7103581	11	47072805	T	G	4.74E-04	PRS-CSx
Colorectum	PRS7	rs7104802	11	74386800	T	G	1.00E-03	PRS-CSx
Colorectum	PRS7	rs7105372	11	8744219	A	C	-5.59E-04	PRS-CSx
Colorectum	PRS7	rs7105709	11	101787112	A	G	-1.75E-04	PRS-CSx

Colorectum	PRS7	rs7106219	11	74359066	C	T	-5.79E-04	PRS-CSx
Colorectum	PRS7	rs7107211	11	120262922	A	G	-8.80E-05	PRS-CSx
Colorectum	PRS7	rs7107344	11	111196011	T	C	-9.69E-04	PRS-CSx
Colorectum	PRS7	rs7107778	11	46860855	T	C	1.63E-04	PRS-CSx
Colorectum	PRS7	rs7108902	11	47091571	T	C	5.80E-04	PRS-CSx
Colorectum	PRS7	rs7111297	11	106398901	A	G	6.22E-04	PRS-CSx
Colorectum	PRS7	rs7111432	11	429659	T	C	1.80E-04	PRS-CSx
Colorectum	PRS7	rs7112171	11	8321802	G	A	3.02E-03	PRS-CSx
Colorectum	PRS7	rs7112529	11	49038697	G	A	-2.90E-04	PRS-CSx
Colorectum	PRS7	rs7112854	11	47116823	T	C	2.37E-04	PRS-CSx
Colorectum	PRS7	rs7113402	11	5979497	C	T	3.03E-03	PRS-CSx
Colorectum	PRS7	rs7114874	11	100314740	T	C	4.75E-04	PRS-CSx
Colorectum	PRS7	rs7115105	11	106399482	A	G	7.93E-04	PRS-CSx
Colorectum	PRS7	rs7115714	11	120294861	A	G	-1.52E-04	PRS-CSx
Colorectum	PRS7	rs7116064	11	17009084	C	T	-2.85E-03	PRS-CSx
Colorectum	PRS7	rs7116087	11	111133005	C	T	5.11E-04	PRS-CSx
Colorectum	PRS7	rs7116194	11	44554064	T	C	1.41E-03	PRS-CSx
Colorectum	PRS7	rs7118159	11	46160551	T	C	2.72E-04	PRS-CSx
Colorectum	PRS7	rs7118193	11	33202539	A	G	1.34E-03	PRS-CSx
Colorectum	PRS7	rs7118200	11	111233450	T	C	-1.26E-03	PRS-CSx
Colorectum	PRS7	rs7119296	11	74391103	G	A	5.56E-04	PRS-CSx
Colorectum	PRS7	rs7120052	11	86335459	C	A	5.27E-04	PRS-CSx
Colorectum	PRS7	rs7120118	11	47286290	T	C	2.63E-05	PRS-CSx
Colorectum	PRS7	rs7121418	11	47165106	A	G	3.55E-04	PRS-CSx
Colorectum	PRS7	rs7121457	11	74205004	G	A	-5.58E-05	PRS-CSx
Colorectum	PRS7	rs7125196	11	61272565	C	T	1.10E-03	PRS-CSx
Colorectum	PRS7	rs712530	3	113015956	T	C	-2.25E-04	PRS-CSx
Colorectum	PRS7	rs7125634	11	16225741	T	C	-5.07E-04	PRS-CSx
Colorectum	PRS7	rs7125773	11	34723573	T	C	-3.41E-04	PRS-CSx
Colorectum	PRS7	rs7125807	11	47041163	C	A	7.32E-04	PRS-CSx
Colorectum	PRS7	rs7126943	11	27303937	T	C	2.63E-04	PRS-CSx
Colorectum	PRS7	rs7127660	11	15113058	A	G	2.92E-04	PRS-CSx
Colorectum	PRS7	rs7128650	11	47047059	G	A	1.84E-04	PRS-CSx
Colorectum	PRS7	rs7129944	11	120299707	C	T	-1.92E-04	PRS-CSx
Colorectum	PRS7	rs7130173	11	111154072	A	C	2.73E-03	PRS-CSx
Colorectum	PRS7	rs7130947	11	47002005	A	C	1.22E-04	PRS-CSx
Colorectum	PRS7	rs7130955	11	65372580	T	C	-1.81E-03	PRS-CSx
Colorectum	PRS7	rs7131597	11	120303939	T	G	-1.73E-04	PRS-CSx
Colorectum	PRS7	rs7132204	12	113204041	A	G	8.39E-05	PRS-CSx
Colorectum	PRS7	rs7132619	12	96809924	A	G	-1.45E-03	PRS-CSx
Colorectum	PRS7	rs7132980	12	25418077	G	A	-4.25E-04	PRS-CSx
Colorectum	PRS7	rs7133343	12	95320950	A	C	6.92E-04	PRS-CSx
Colorectum	PRS7	rs7133974	12	51184577	G	A	9.57E-04	PRS-CSx
Colorectum	PRS7	rs7134535	12	109441249	A	G	7.21E-04	PRS-CSx
Colorectum	PRS7	rs7134679	12	79939504	A	G	4.33E-04	PRS-CSx
Colorectum	PRS7	rs7136874	12	112113658	C	T	4.46E-04	PRS-CSx
Colorectum	PRS7	rs7138514	12	79794558	G	T	2.16E-04	PRS-CSx
Colorectum	PRS7	rs7138644	12	46444759	G	A	-2.67E-04	PRS-CSx
Colorectum	PRS7	rs7138802	12	110157679	G	A	-5.63E-04	PRS-CSx
Colorectum	PRS7	rs7138818	12	53090190	T	C	-3.54E-04	PRS-CSx
Colorectum	PRS7	rs7139667	13	72337943	C	T	7.24E-04	PRS-CSx
Colorectum	PRS7	rs7139757	13	73991242	T	C	-1.20E-03	PRS-CSx
Colorectum	PRS7	rs714022	22	46175530	C	A	4.13E-04	PRS-CSx
Colorectum	PRS7	rs7141911	14	57672871	G	A	4.70E-04	PRS-CSx
Colorectum	PRS7	rs7143281	14	85826542	T	G	-6.88E-04	PRS-CSx
Colorectum	PRS7	rs714378	6	100872787	C	T	-3.38E-04	PRS-CSx

Colorectum	PRS7	rs7145651	14	59206247	G	A	-7.57E-04	PRS-CSx
Colorectum	PRS7	rs7146567	14	79032690	G	A	2.88E-04	PRS-CSx
Colorectum	PRS7	rs7147423	14	59412693	A	G	1.66E-04	PRS-CSx
Colorectum	PRS7	rs7147439	14	105523663	G	A	4.56E-04	PRS-CSx
Colorectum	PRS7	rs7147669	14	105523817	A	G	-1.70E-04	PRS-CSx
Colorectum	PRS7	rs7148715	14	59446235	A	G	-3.77E-04	PRS-CSx
Colorectum	PRS7	rs7149575	14	54459204	C	T	4.66E-04	PRS-CSx
Colorectum	PRS7	rs7149735	14	58756421	G	A	1.91E-04	PRS-CSx
Colorectum	PRS7	rs7149936	14	51408758	G	A	9.44E-05	PRS-CSx
Colorectum	PRS7	rs7150988	14	68793704	T	C	-2.43E-04	PRS-CSx
Colorectum	PRS7	rs7151036	14	58823033	G	A	1.58E-04	PRS-CSx
Colorectum	PRS7	rs7151133	14	34969406	C	T	-2.81E-04	PRS-CSx
Colorectum	PRS7	rs7151149	14	34969433	C	T	-3.18E-04	PRS-CSx
Colorectum	PRS7	rs7151690	14	66146292	G	A	1.14E-03	PRS-CSx
Colorectum	PRS7	rs7151907	14	63213611	A	G	2.89E-04	PRS-CSx
Colorectum	PRS7	rs7152131	14	73657180	A	C	4.65E-04	PRS-CSx
Colorectum	PRS7	rs7152294	14	26775647	T	C	1.33E-03	PRS-CSx
Colorectum	PRS7	rs7153352	14	54535898	A	G	7.31E-04	PRS-CSx
Colorectum	PRS7	rs7153492	14	99104143	C	T	-2.77E-04	PRS-CSx
Colorectum	PRS7	rs7154962	14	54690685	T	C	1.10E-03	PRS-CSx
Colorectum	PRS7	rs7155136	14	59431448	A	G	2.20E-05	PRS-CSx
Colorectum	PRS7	rs7156	7	16639615	C	A	-1.20E-03	PRS-CSx
Colorectum	PRS7	rs7157018	14	92716881	T	C	6.65E-04	PRS-CSx
Colorectum	PRS7	rs7157111	14	26759206	A	G	1.70E-03	PRS-CSx
Colorectum	PRS7	rs7157250	14	71457829	T	C	-3.56E-04	PRS-CSx
Colorectum	PRS7	rs7157437	14	77168798	A	G	-1.01E-05	PRS-CSx
Colorectum	PRS7	rs7157567	14	105522201	T	C	-3.44E-04	PRS-CSx
Colorectum	PRS7	rs7157608	14	57671215	C	T	4.44E-04	PRS-CSx
Colorectum	PRS7	rs7158168	14	53719172	T	G	8.26E-04	PRS-CSx
Colorectum	PRS7	rs7158479	14	105552083	C	T	-3.86E-04	PRS-CSx
Colorectum	PRS7	rs715948	12	57532982	C	T	5.03E-03	PRS-CSx
Colorectum	PRS7	rs7161189	14	59409500	A	G	9.40E-04	PRS-CSx
Colorectum	PRS7	rs7162192	15	71657396	C	A	3.63E-04	PRS-CSx
Colorectum	PRS7	rs7163907	15	75845097	C	T	3.44E-04	PRS-CSx
Colorectum	PRS7	rs7165429	15	91151881	C	T	8.32E-04	PRS-CSx
Colorectum	PRS7	rs7166281	15	75928192	G	T	6.00E-04	PRS-CSx
Colorectum	PRS7	rs7167343	15	51578594	G	A	2.59E-04	PRS-CSx
Colorectum	PRS7	rs7167355	15	27230025	G	A	7.91E-04	PRS-CSx
Colorectum	PRS7	rs7167951	15	71678963	G	T	3.83E-04	PRS-CSx
Colorectum	PRS7	rs717015	11	19487771	A	G	1.16E-03	PRS-CSx
Colorectum	PRS7	rs7170181	15	86367644	G	A	9.31E-04	PRS-CSx
Colorectum	PRS7	rs717052	11	120180479	A	G	-1.71E-03	PRS-CSx
Colorectum	PRS7	rs7171787	15	22922576	G	T	-6.85E-04	PRS-CSx
Colorectum	PRS7	rs7172156	15	51546298	A	G	-3.83E-06	PRS-CSx
Colorectum	PRS7	rs717295	22	45825621	A	G	-1.62E-04	PRS-CSx
Colorectum	PRS7	rs717304	2	61223290	C	T	-1.99E-03	PRS-CSx
Colorectum	PRS7	rs7174882	15	102018845	G	T	-9.40E-04	PRS-CSx
Colorectum	PRS7	rs7175001	15	82042734	T	C	-2.27E-04	PRS-CSx
Colorectum	PRS7	rs7175513	15	47597049	C	A	-2.98E-04	PRS-CSx
Colorectum	PRS7	rs7176445	15	67328417	G	A	1.89E-06	PRS-CSx
Colorectum	PRS7	rs717659	1	210557308	G	A	2.31E-04	PRS-CSx
Colorectum	PRS7	rs7176654	15	73999006	G	A	-6.96E-04	PRS-CSx
Colorectum	PRS7	rs717746	5	149556558	T	G	-2.30E-03	PRS-CSx
Colorectum	PRS7	rs7179195	15	82070366	G	A	-6.42E-04	PRS-CSx
Colorectum	PRS7	rs7181029	15	82088114	T	C	-5.47E-05	PRS-CSx
Colorectum	PRS7	rs7182227	15	67033421	T	C	8.15E-05	PRS-CSx

Colorectum	PRS7	rs718268	6	100859159	T	C	-3.50E-04	PRS-CSx
Colorectum	PRS7	rs7183943	15	55875066	A	G	-8.70E-04	PRS-CSx
Colorectum	PRS7	rs7185080	16	80119504	G	A	1.70E-04	PRS-CSx
Colorectum	PRS7	rs7185647	16	76462538	T	C	3.88E-04	PRS-CSx
Colorectum	PRS7	rs7186373	16	86702158	T	C	1.01E-03	PRS-CSx
Colorectum	PRS7	rs7186693	16	68745841	G	A	1.12E-04	PRS-CSx
Colorectum	PRS7	rs7186861	16	88761282	G	A	-4.85E-04	PRS-CSx
Colorectum	PRS7	rs7186889	16	50092291	G	A	-5.36E-04	PRS-CSx
Colorectum	PRS7	rs7188511	16	11773743	T	G	-4.13E-04	PRS-CSx
Colorectum	PRS7	rs7188536	16	86205067	G	A	-2.50E-04	PRS-CSx
Colorectum	PRS7	rs7189200	16	76438005	A	G	2.32E-04	PRS-CSx
Colorectum	PRS7	rs7189533	16	84392942	A	G	-7.71E-04	PRS-CSx
Colorectum	PRS7	rs7190907	16	80005967	A	G	2.86E-04	PRS-CSx
Colorectum	PRS7	rs7191333	16	68538612	G	A	1.01E-03	PRS-CSx
Colorectum	PRS7	rs7191812	16	4408941	T	C	7.38E-04	PRS-CSx
Colorectum	PRS7	rs719204	20	52564201	A	G	7.80E-04	PRS-CSx
Colorectum	PRS7	rs7192753	16	80098249	G	A	-9.99E-05	PRS-CSx
Colorectum	PRS7	rs7193138	16	50099218	G	T	-2.58E-04	PRS-CSx
Colorectum	PRS7	rs7193204	16	4403141	C	T	6.82E-04	PRS-CSx
Colorectum	PRS7	rs7193465	16	80057803	G	A	6.34E-04	PRS-CSx
Colorectum	PRS7	rs719368	13	28851332	A	G	-4.50E-04	PRS-CSx
Colorectum	PRS7	rs7197164	16	17067283	C	T	-1.57E-03	PRS-CSx
Colorectum	PRS7	rs7197843	16	84742878	T	C	-8.29E-04	PRS-CSx
Colorectum	PRS7	rs7198088	16	68919559	C	T	2.48E-04	PRS-CSx
Colorectum	PRS7	rs7198225	16	365024	A	C	-1.12E-05	PRS-CSx
Colorectum	PRS7	rs719826	13	74041419	T	C	3.49E-04	PRS-CSx
Colorectum	PRS7	rs7199483	16	86697008	T	C	6.81E-04	PRS-CSx
Colorectum	PRS7	rs7199884	16	1217549	C	T	2.42E-03	PRS-CSx
Colorectum	PRS7	rs7199979	16	49813666	T	C	1.54E-03	PRS-CSx
Colorectum	PRS7	rs7199991	16	68818245	G	T	-2.35E-04	PRS-CSx
Colorectum	PRS7	rs7200137	16	1901211	T	G	1.60E-04	PRS-CSx
Colorectum	PRS7	rs7201437	16	68727130	C	A	4.08E-04	PRS-CSx
Colorectum	PRS7	rs7202333	16	68881495	C	T	2.63E-04	PRS-CSx
Colorectum	PRS7	rs7203610	16	87688568	G	A	4.79E-04	PRS-CSx
Colorectum	PRS7	rs7205189	16	4460114	A	G	4.32E-04	PRS-CSx
Colorectum	PRS7	rs7205994	16	11815964	T	C	-3.58E-04	PRS-CSx
Colorectum	PRS7	rs7206735	16	50148508	C	T	-3.11E-04	PRS-CSx
Colorectum	PRS7	rs720682	22	45717859	C	T	-5.28E-04	PRS-CSx
Colorectum	PRS7	rs7206982	17	57273681	T	C	2.10E-04	PRS-CSx
Colorectum	PRS7	rs7207585	17	13302161	T	C	-1.47E-03	PRS-CSx
Colorectum	PRS7	rs7207980	17	1024394	A	C	1.02E-03	PRS-CSx
Colorectum	PRS7	rs7208065	17	17120204	T	C	2.53E-04	PRS-CSx
Colorectum	PRS7	rs7208610	17	63250334	G	T	-3.68E-04	PRS-CSx
Colorectum	PRS7	rs7209430	17	55705224	T	C	-1.13E-03	PRS-CSx
Colorectum	PRS7	rs7209796	17	814320	T	C	-1.17E-03	PRS-CSx
Colorectum	PRS7	rs7210086	17	70641698	C	A	1.18E-03	PRS-CSx
Colorectum	PRS7	rs721048	2	63131731	A	G	9.63E-04	PRS-CSx
Colorectum	PRS7	rs721219	12	115904058	C	T	1.15E-03	PRS-CSx
Colorectum	PRS7	rs7212995	17	57267529	T	G	2.14E-04	PRS-CSx
Colorectum	PRS7	rs7213257	17	21080315	G	A	-2.20E-04	PRS-CSx
Colorectum	PRS7	rs7214193	17	70641865	G	A	2.19E-03	PRS-CSx
Colorectum	PRS7	rs7214311	17	38648346	G	A	-2.13E-03	PRS-CSx
Colorectum	PRS7	rs7214916	17	75695647	C	T	-9.70E-04	PRS-CSx
Colorectum	PRS7	rs7216434	17	69654266	G	T	3.07E-04	PRS-CSx
Colorectum	PRS7	rs7217872	17	88988	T	C	2.09E-03	PRS-CSx
Colorectum	PRS7	rs7219324	17	17909775	A	G	-7.41E-04	PRS-CSx

Colorectum	PRS7	rs7219822	17	5725518	G	A	2.01E-04	PRS-CSx
Colorectum	PRS7	rs7220814	17	7290695	G	A	-3.42E-03	PRS-CSx
Colorectum	PRS7	rs7223491	17	57276710	G	A	1.94E-04	PRS-CSx
Colorectum	PRS7	rs7225332	17	5725241	T	C	2.04E-04	PRS-CSx
Colorectum	PRS7	rs7225859	17	57331105	T	C	1.81E-04	PRS-CSx
Colorectum	PRS7	rs7226599	18	74491132	A	G	3.36E-04	PRS-CSx
Colorectum	PRS7	rs7226855	18	46454048	A	G	1.19E-02	PRS-CSx
Colorectum	PRS7	rs7227673	18	70348353	A	G	3.17E-04	PRS-CSx
Colorectum	PRS7	rs7228637	18	9446098	T	C	-4.65E-04	PRS-CSx
Colorectum	PRS7	rs7229639	18	46450976	A	G	2.40E-02	PRS-CSx
Colorectum	PRS7	rs7229792	18	69493203	A	G	1.10E-03	PRS-CSx
Colorectum	PRS7	rs7230479	18	42044962	C	A	2.13E-04	PRS-CSx
Colorectum	PRS7	rs7231518	18	9456965	C	T	-2.69E-04	PRS-CSx
Colorectum	PRS7	rs7232502	18	74727618	T	C	1.49E-03	PRS-CSx
Colorectum	PRS7	rs723305	2	208073417	A	G	-2.25E-04	PRS-CSx
Colorectum	PRS7	rs7233081	18	6772593	G	A	-7.38E-04	PRS-CSx
Colorectum	PRS7	rs7233990	18	41660811	C	T	3.87E-04	PRS-CSx
Colorectum	PRS7	rs7234029	18	12877060	G	A	-3.82E-04	PRS-CSx
Colorectum	PRS7	rs723475	6	28304841	G	T	6.36E-05	PRS-CSx
Colorectum	PRS7	rs723476	6	28305104	T	C	1.03E-04	PRS-CSx
Colorectum	PRS7	rs7235285	18	62187090	C	A	-2.40E-03	PRS-CSx
Colorectum	PRS7	rs7236653	18	10698648	A	G	9.77E-04	PRS-CSx
Colorectum	PRS7	rs723831	15	36929369	T	C	2.69E-04	PRS-CSx
Colorectum	PRS7	rs723937	11	120312746	A	G	-2.06E-04	PRS-CSx
Colorectum	PRS7	rs7241016	18	12880206	G	A	-4.10E-04	PRS-CSx
Colorectum	PRS7	rs7241650	18	12880533	T	C	-6.01E-04	PRS-CSx
Colorectum	PRS7	rs7242186	18	67981861	G	A	3.49E-04	PRS-CSx
Colorectum	PRS7	rs7242553	18	70348081	A	G	2.16E-04	PRS-CSx
Colorectum	PRS7	rs7242838	18	10933523	G	A	-4.55E-04	PRS-CSx
Colorectum	PRS7	rs7244456	18	36448846	G	A	4.50E-04	PRS-CSx
Colorectum	PRS7	rs7245902	19	28494758	T	C	-2.55E-04	PRS-CSx
Colorectum	PRS7	rs7246147	19	38283984	A	G	7.92E-04	PRS-CSx
Colorectum	PRS7	rs7247087	19	1031212	A	G	-1.86E-04	PRS-CSx
Colorectum	PRS7	rs7249622	19	38202001	C	T	2.22E-04	PRS-CSx
Colorectum	PRS7	rs7251233	19	57796719	C	T	-5.57E-04	PRS-CSx
Colorectum	PRS7	rs7251236	19	33435765	G	A	2.67E-04	PRS-CSx
Colorectum	PRS7	rs7251815	19	17844942	T	G	-8.93E-04	PRS-CSx
Colorectum	PRS7	rs7252154	19	12569270	C	T	1.94E-05	PRS-CSx
Colorectum	PRS7	rs7252333	19	58900747	T	C	7.88E-04	PRS-CSx
Colorectum	PRS7	rs7253081	19	38201712	T	G	5.60E-05	PRS-CSx
Colorectum	PRS7	rs7253540	19	3539488	A	G	-7.50E-04	PRS-CSx
Colorectum	PRS7	rs7254170	19	38123793	T	C	-1.77E-04	PRS-CSx
Colorectum	PRS7	rs7255130	19	12567293	T	C	4.74E-05	PRS-CSx
Colorectum	PRS7	rs7255247	19	4597413	C	T	8.45E-04	PRS-CSx
Colorectum	PRS7	rs7256689	19	17273893	G	T	-2.72E-04	PRS-CSx
Colorectum	PRS7	rs7257754	19	33502256	G	A	9.39E-04	PRS-CSx
Colorectum	PRS7	rs7259371	19	33534641	A	G	-1.98E-03	PRS-CSx
Colorectum	PRS7	rs7260507	19	41947625	A	C	3.84E-04	PRS-CSx
Colorectum	PRS7	rs7260605	19	41947635	C	T	7.40E-04	PRS-CSx
Colorectum	PRS7	rs7262524	20	60983973	T	C	-8.71E-04	PRS-CSx
Colorectum	PRS7	rs7263438	20	5284251	C	T	-9.18E-04	PRS-CSx
Colorectum	PRS7	rs726418	17	70462407	G	A	5.80E-04	PRS-CSx
Colorectum	PRS7	rs7267819	20	52505281	G	A	-8.21E-04	PRS-CSx
Colorectum	PRS7	rs7268357	20	47320033	A	G	-7.09E-04	PRS-CSx
Colorectum	PRS7	rs7270163	20	6751316	G	A	5.69E-04	PRS-CSx
Colorectum	PRS7	rs7271854	20	57447022	C	T	-5.71E-04	PRS-CSx

Colorectum	PRS7	rs7273764	20	55887723	G	A	-2.10E-04	PRS-CSx
Colorectum	PRS7	rs7274202	20	48130982	T	C	-5.04E-05	PRS-CSx
Colorectum	PRS7	rs727453	18	54045333	C	T	-3.78E-04	PRS-CSx
Colorectum	PRS7	rs727644	7	114109349	G	A	3.25E-04	PRS-CSx
Colorectum	PRS7	rs727689	20	6688405	C	T	-5.64E-04	PRS-CSx
Colorectum	PRS7	rs7277617	21	47570652	G	A	2.44E-04	PRS-CSx
Colorectum	PRS7	rs727905	3	119119433	A	C	-4.85E-04	PRS-CSx
Colorectum	PRS7	rs7279052	21	47808617	T	C	9.58E-04	PRS-CSx
Colorectum	PRS7	rs7279193	21	47757792	A	G	5.92E-04	PRS-CSx
Colorectum	PRS7	rs7280126	21	43349040	A	G	1.11E-03	PRS-CSx
Colorectum	PRS7	rs7281894	21	43353648	C	T	9.40E-04	PRS-CSx
Colorectum	PRS7	rs7282280	21	30214959	T	C	-1.62E-04	PRS-CSx
Colorectum	PRS7	rs7282289	21	30214979	T	C	-2.03E-04	PRS-CSx
Colorectum	PRS7	rs7282496	21	34819582	A	C	5.57E-05	PRS-CSx
Colorectum	PRS7	rs7282606	21	48043992	G	A	3.34E-04	PRS-CSx
Colorectum	PRS7	rs7282864	21	47566828	A	G	9.04E-04	PRS-CSx
Colorectum	PRS7	rs728329	13	49462380	A	G	-4.66E-04	PRS-CSx
Colorectum	PRS7	rs7283555	21	19660053	G	A	6.05E-04	PRS-CSx
Colorectum	PRS7	rs7284306	22	45788896	C	T	-6.51E-04	PRS-CSx
Colorectum	PRS7	rs7286140	22	43063246	G	A	4.66E-04	PRS-CSx
Colorectum	PRS7	rs7286402	22	43811592	A	G	-4.10E-04	PRS-CSx
Colorectum	PRS7	rs728661	12	96071320	C	T	2.38E-03	PRS-CSx
Colorectum	PRS7	rs7291153	22	43356499	C	T	7.38E-05	PRS-CSx
Colorectum	PRS7	rs7294911	12	80023110	A	G	3.44E-04	PRS-CSx
Colorectum	PRS7	rs7295468	12	52688766	T	C	-5.53E-04	PRS-CSx
Colorectum	PRS7	rs7295635	12	64425423	G	A	-9.13E-04	PRS-CSx
Colorectum	PRS7	rs7295717	12	120476434	A	G	2.42E-04	PRS-CSx
Colorectum	PRS7	rs729663	20	47304306	G	A	-3.13E-04	PRS-CSx
Colorectum	PRS7	rs7297103	12	25265295	C	T	-2.49E-04	PRS-CSx
Colorectum	PRS7	rs7297808	12	79953757	T	C	1.61E-04	PRS-CSx
Colorectum	PRS7	rs7298174	12	43094424	A	G	-5.24E-04	PRS-CSx
Colorectum	PRS7	rs7298631	12	95457292	C	T	3.58E-04	PRS-CSx
Colorectum	PRS7	rs7299597	12	64426199	G	A	-6.17E-04	PRS-CSx
Colorectum	PRS7	rs7299936	12	115934000	A	G	6.85E-04	PRS-CSx
Colorectum	PRS7	rs7299998	12	25331384	C	T	-2.23E-04	PRS-CSx
Colorectum	PRS7	rs7300145	12	80062156	C	T	2.63E-04	PRS-CSx
Colorectum	PRS7	rs7302422	12	50645471	G	A	1.60E-04	PRS-CSx
Colorectum	PRS7	rs7302529	12	77321581	T	C	-6.18E-04	PRS-CSx
Colorectum	PRS7	rs7302874	12	120420670	C	T	3.56E-04	PRS-CSx
Colorectum	PRS7	rs7302922	12	25349317	C	T	-3.27E-04	PRS-CSx
Colorectum	PRS7	rs7302948	12	78208739	A	C	5.86E-04	PRS-CSx
Colorectum	PRS7	rs7303669	12	25245604	C	T	-7.71E-04	PRS-CSx
Colorectum	PRS7	rs7304325	12	120423472	T	C	3.83E-04	PRS-CSx
Colorectum	PRS7	rs7304462	12	115894190	A	G	7.57E-04	PRS-CSx
Colorectum	PRS7	rs7304470	12	95066388	A	G	1.48E-03	PRS-CSx
Colorectum	PRS7	rs7305668	12	107637820	G	A	-2.89E-04	PRS-CSx
Colorectum	PRS7	rs730631	2	108950832	G	T	-8.97E-04	PRS-CSx
Colorectum	PRS7	rs7306529	12	111780644	T	C	2.32E-04	PRS-CSx
Colorectum	PRS7	rs7306609	12	113282140	C	T	-1.59E-04	PRS-CSx
Colorectum	PRS7	rs7306677	12	51205763	T	C	1.26E-03	PRS-CSx
Colorectum	PRS7	rs7306739	12	120511532	C	T	2.45E-04	PRS-CSx
Colorectum	PRS7	rs7306769	12	25315054	G	A	-3.45E-04	PRS-CSx
Colorectum	PRS7	rs730720	10	73772762	T	C	-1.98E-04	PRS-CSx
Colorectum	PRS7	rs7307647	12	80076418	C	T	3.37E-04	PRS-CSx
Colorectum	PRS7	rs7308303	12	46483918	C	T	6.02E-04	PRS-CSx
Colorectum	PRS7	rs7309577	12	46436825	T	C	-3.21E-05	PRS-CSx

Colorectum	PRS7	rs7309711	12	46326282	T	C	-3.79E-04	PRS-CSx
Colorectum	PRS7	rs730992	11	15985282	A	C	-6.34E-04	PRS-CSx
Colorectum	PRS7	rs730993	11	15985264	G	A	5.53E-04	PRS-CSx
Colorectum	PRS7	rs7309982	12	12550539	A	G	-7.89E-04	PRS-CSx
Colorectum	PRS7	rs7310004	12	71054497	T	C	1.09E-03	PRS-CSx
Colorectum	PRS7	rs7310076	12	16841016	T	C	-1.19E-03	PRS-CSx
Colorectum	PRS7	rs7310151	12	79926158	T	C	2.55E-04	PRS-CSx
Colorectum	PRS7	rs731027	10	73772336	C	T	-2.63E-06	PRS-CSx
Colorectum	PRS7	rs7310376	12	98212884	G	A	-1.83E-03	PRS-CSx
Colorectum	PRS7	rs7310409	12	121424861	A	G	1.01E-03	PRS-CSx
Colorectum	PRS7	rs7311377	12	64403934	T	G	-1.86E-03	PRS-CSx
Colorectum	PRS7	rs7311681	12	112817847	T	G	3.47E-04	PRS-CSx
Colorectum	PRS7	rs7312811	12	120432715	T	C	5.01E-04	PRS-CSx
Colorectum	PRS7	rs7313281	12	46487915	A	G	4.75E-04	PRS-CSx
Colorectum	PRS7	rs7313392	12	115088212	A	G	1.03E-03	PRS-CSx
Colorectum	PRS7	rs7313581	12	25422717	T	C	-4.41E-04	PRS-CSx
Colorectum	PRS7	rs7314004	12	14293118	T	C	-5.54E-04	PRS-CSx
Colorectum	PRS7	rs7314022	12	113176501	C	T	2.94E-04	PRS-CSx
Colorectum	PRS7	rs7314282	12	112502438	A	G	2.51E-04	PRS-CSx
Colorectum	PRS7	rs7314959	12	120498862	A	G	2.29E-04	PRS-CSx
Colorectum	PRS7	rs7315020	12	6409770	T	C	-3.15E-03	PRS-CSx
Colorectum	PRS7	rs7315152	12	113473715	T	C	3.06E-04	PRS-CSx
Colorectum	PRS7	rs7315438	12	115891403	T	C	1.36E-03	PRS-CSx
Colorectum	PRS7	rs7315519	12	113192927	A	G	1.37E-04	PRS-CSx
Colorectum	PRS7	rs7315734	12	113278081	G	A	-1.78E-04	PRS-CSx
Colorectum	PRS7	rs7316938	13	73991408	T	C	-7.45E-04	PRS-CSx
Colorectum	PRS7	rs7316968	13	73991462	T	C	-8.60E-04	PRS-CSx
Colorectum	PRS7	rs7317330	13	100119876	G	T	-7.60E-04	PRS-CSx
Colorectum	PRS7	rs7318674	13	34285848	G	T	-2.16E-04	PRS-CSx
Colorectum	PRS7	rs7318963	13	81566241	A	C	2.59E-04	PRS-CSx
Colorectum	PRS7	rs7320835	13	82088756	C	T	8.23E-04	PRS-CSx
Colorectum	PRS7	rs7323357	13	34115595	T	C	-2.93E-04	PRS-CSx
Colorectum	PRS7	rs7324034	13	34084266	C	A	-3.40E-04	PRS-CSx
Colorectum	PRS7	rs7325027	13	108810083	T	C	1.02E-03	PRS-CSx
Colorectum	PRS7	rs7325697	13	100117793	T	C	-8.33E-04	PRS-CSx
Colorectum	PRS7	rs7326129	13	109929728	C	T	-8.99E-04	PRS-CSx
Colorectum	PRS7	rs7326364	13	33691953	A	G	-3.28E-04	PRS-CSx
Colorectum	PRS7	rs7327118	13	85566745	G	A	-3.20E-04	PRS-CSx
Colorectum	PRS7	rs7327345	13	74022913	T	C	-4.15E-04	PRS-CSx
Colorectum	PRS7	rs7327508	13	28730023	G	T	-2.02E-04	PRS-CSx
Colorectum	PRS7	rs7328345	13	85566663	C	T	-3.31E-04	PRS-CSx
Colorectum	PRS7	rs7328731	13	111056994	A	G	-1.67E-03	PRS-CSx
Colorectum	PRS7	rs7328732	13	28816278	A	G	-1.88E-04	PRS-CSx
Colorectum	PRS7	rs7329068	13	41628282	C	T	-7.04E-04	PRS-CSx
Colorectum	PRS7	rs7329539	13	94052090	C	T	6.39E-04	PRS-CSx
Colorectum	PRS7	rs732998	10	104897901	C	T	4.27E-04	PRS-CSx
Colorectum	PRS7	rs7330759	13	28826983	G	A	-3.84E-04	PRS-CSx
Colorectum	PRS7	rs7331142	13	95860574	C	T	-3.32E-04	PRS-CSx
Colorectum	PRS7	rs7331661	13	76038996	A	G	-6.04E-04	PRS-CSx
Colorectum	PRS7	rs7332041	13	57967735	C	T	6.97E-04	PRS-CSx
Colorectum	PRS7	rs7332533	13	107381543	G	T	-5.53E-04	PRS-CSx
Colorectum	PRS7	rs7333607	13	37462010	G	A	2.66E-03	PRS-CSx
Colorectum	PRS7	rs733381	22	40669648	G	A	-1.30E-03	PRS-CSx
Colorectum	PRS7	rs7334087	13	73999204	T	C	9.88E-04	PRS-CSx
Colorectum	PRS7	rs7334420	13	82121067	G	T	9.56E-04	PRS-CSx
Colorectum	PRS7	rs7335032	13	34172797	C	T	-7.07E-04	PRS-CSx

Colorectum	PRS7	rs7335349	13	72370681	C	A	1.58E-04	PRS-CSx
Colorectum	PRS7	rs7335576	13	76043318	G	A	-7.20E-04	PRS-CSx
Colorectum	PRS7	rs7338537	13	100119581	A	C	-6.59E-04	PRS-CSx
Colorectum	PRS7	rs733920	17	46639703	A	C	1.42E-04	PRS-CSx
Colorectum	PRS7	rs734165	10	111697789	T	C	-1.02E-04	PRS-CSx
Colorectum	PRS7	rs7342574	15	50430183	T	G	5.24E-04	PRS-CSx
Colorectum	PRS7	rs734379	19	58899182	A	G	4.78E-04	PRS-CSx
Colorectum	PRS7	rs734380	19	58898963	G	T	4.80E-04	PRS-CSx
Colorectum	PRS7	rs7349184	1	194961172	G	A	-9.26E-04	PRS-CSx
Colorectum	PRS7	rs734999	1	2513216	C	T	-2.25E-04	PRS-CSx
Colorectum	PRS7	rs7355307	2	48694790	T	C	1.31E-04	PRS-CSx
Colorectum	PRS7	rs7357368	8	128443387	T	C	1.60E-04	PRS-CSx
Colorectum	PRS7	rs7357419	8	143482961	A	G	6.71E-04	PRS-CSx
Colorectum	PRS7	rs7357486	8	128441623	T	C	9.16E-05	PRS-CSx
Colorectum	PRS7	rs735861	8	15633397	T	G	-4.88E-04	PRS-CSx
Colorectum	PRS7	rs7359414	16	362638	G	T	8.09E-05	PRS-CSx
Colorectum	PRS7	rs7362041	20	60975550	G	A	-3.50E-04	PRS-CSx
Colorectum	PRS7	rs736304	2	109935979	A	G	5.47E-05	PRS-CSx
Colorectum	PRS7	rs736365	20	13788882	A	G	4.30E-04	PRS-CSx
Colorectum	PRS7	rs7364152	22	43316667	A	G	3.43E-04	PRS-CSx
Colorectum	PRS7	rs7366048	1	38463504	C	T	-8.18E-04	PRS-CSx
Colorectum	PRS7	rs7366187	1	246912688	C	T	7.89E-04	PRS-CSx
Colorectum	PRS7	rs737148	2	43015119	C	T	2.26E-04	PRS-CSx
Colorectum	PRS7	rs737623	9	107735105	A	G	-6.33E-05	PRS-CSx
Colorectum	PRS7	rs737768	22	27518815	T	C	7.81E-04	PRS-CSx
Colorectum	PRS7	rs737800	22	26863559	T	C	-6.02E-04	PRS-CSx
Colorectum	PRS7	rs7379278	5	177562815	G	A	1.04E-03	PRS-CSx
Colorectum	PRS7	rs738127	22	21968221	G	A	2.30E-04	PRS-CSx
Colorectum	PRS7	rs7382258	6	31345021	G	A	-3.60E-04	PRS-CSx
Colorectum	PRS7	rs738377	22	43389570	C	T	1.07E-05	PRS-CSx
Colorectum	PRS7	rs738378	22	43398962	C	T	1.06E-04	PRS-CSx
Colorectum	PRS7	rs738379	22	43400764	A	C	2.40E-05	PRS-CSx
Colorectum	PRS7	rs738381	22	43404107	T	C	1.08E-04	PRS-CSx
Colorectum	PRS7	rs738386	22	43328419	G	A	2.84E-04	PRS-CSx
Colorectum	PRS7	rs738387	22	43329819	G	A	3.62E-04	PRS-CSx
Colorectum	PRS7	rs738477	22	44489022	G	A	-1.35E-03	PRS-CSx
Colorectum	PRS7	rs738479	22	44489896	T	C	-1.09E-03	PRS-CSx
Colorectum	PRS7	rs738482	22	44480397	C	T	1.97E-04	PRS-CSx
Colorectum	PRS7	rs738803	22	24192879	A	G	8.09E-04	PRS-CSx
Colorectum	PRS7	rs739182	22	40615276	T	G	-1.67E-04	PRS-CSx
Colorectum	PRS7	rs7394572	11	432436	A	G	3.82E-04	PRS-CSx
Colorectum	PRS7	rs739496	12	111887659	A	G	4.56E-04	PRS-CSx
Colorectum	PRS7	rs7396562	11	408352	C	A	4.23E-07	PRS-CSx
Colorectum	PRS7	rs7396812	11	373404	A	G	-5.43E-05	PRS-CSx
Colorectum	PRS7	rs7398343	12	111774068	A	C	1.03E-04	PRS-CSx
Colorectum	PRS7	rs7398833	12	111786892	T	C	1.79E-04	PRS-CSx
Colorectum	PRS7	rs739901	12	113415663	A	C	-8.12E-04	PRS-CSx
Colorectum	PRS7	rs7403275	15	31754008	A	G	3.29E-04	PRS-CSx
Colorectum	PRS7	rs7403458	15	31754025	G	A	4.06E-04	PRS-CSx
Colorectum	PRS7	rs740423	19	1978271	T	C	-1.62E-03	PRS-CSx
Colorectum	PRS7	rs740655	16	5761537	C	T	6.44E-04	PRS-CSx
Colorectum	PRS7	rs740842	12	6421495	A	G	1.68E-03	PRS-CSx
Colorectum	PRS7	rs7408736	19	37796311	T	G	-7.15E-04	PRS-CSx
Colorectum	PRS7	rs7414210	1	97812612	C	A	2.43E-03	PRS-CSx
Colorectum	PRS7	rs741472	2	71436854	G	A	-1.99E-05	PRS-CSx
Colorectum	PRS7	rs742039	6	14764983	A	G	-7.49E-04	PRS-CSx

Colorectum	PRS7	rs742107	6	28290714	G	T	7.69E-05	PRS-CSx
Colorectum	PRS7	rs742144	22	36894622	G	A	-1.94E-04	PRS-CSx
Colorectum	PRS7	rs7423524	2	43233488	C	T	-5.03E-04	PRS-CSx
Colorectum	PRS7	rs742600	20	49058029	G	A	3.11E-03	PRS-CSx
Colorectum	PRS7	rs742643	20	47269874	T	C	-1.06E-03	PRS-CSx
Colorectum	PRS7	rs742697	6	32291542	C	T	-1.44E-04	PRS-CSx
Colorectum	PRS7	rs7429859	3	90253164	T	C	-1.09E-03	PRS-CSx
Colorectum	PRS7	rs7432308	3	18877562	T	C	4.63E-04	PRS-CSx
Colorectum	PRS7	rs7433057	3	65038985	C	T	1.27E-03	PRS-CSx
Colorectum	PRS7	rs7437569	4	115516587	T	G	2.32E-04	PRS-CSx
Colorectum	PRS7	rs744205	11	69929677	A	G	9.40E-04	PRS-CSx
Colorectum	PRS7	rs7443264	5	6153919	T	C	1.21E-03	PRS-CSx
Colorectum	PRS7	rs7444	22	21976934	T	C	2.99E-04	PRS-CSx
Colorectum	PRS7	rs7444022	5	100753906	T	C	2.16E-03	PRS-CSx
Colorectum	PRS7	rs7447025	5	40040287	A	G	-1.39E-04	PRS-CSx
Colorectum	PRS7	rs744748	1	55149254	A	C	7.04E-04	PRS-CSx
Colorectum	PRS7	rs744778	20	60631365	A	G	-2.95E-04	PRS-CSx
Colorectum	PRS7	rs744895	11	36448379	C	T	-2.59E-04	PRS-CSx
Colorectum	PRS7	rs744896	11	36448341	G	A	-4.24E-04	PRS-CSx
Colorectum	PRS7	rs7453967	6	31314243	G	T	-2.79E-04	PRS-CSx
Colorectum	PRS7	rs7458148	7	39787109	G	A	7.62E-04	PRS-CSx
Colorectum	PRS7	rs746210	3	129294568	A	G	-4.48E-04	PRS-CSx
Colorectum	PRS7	rs746939	2	159985486	A	G	-4.37E-04	PRS-CSx
Colorectum	PRS7	rs7473	1	183114634	G	A	3.61E-04	PRS-CSx
Colorectum	PRS7	rs747487	6	14764738	G	A	-7.09E-04	PRS-CSx
Colorectum	PRS7	rs747650	11	47176005	C	T	2.23E-04	PRS-CSx
Colorectum	PRS7	rs7478880	11	120261791	T	C	-1.92E-04	PRS-CSx
Colorectum	PRS7	rs7478973	11	36776618	T	C	1.09E-03	PRS-CSx
Colorectum	PRS7	rs7479309	11	27311814	G	A	1.53E-04	PRS-CSx
Colorectum	PRS7	rs747948	20	60964301	T	C	-7.68E-05	PRS-CSx
Colorectum	PRS7	rs747949	20	60964122	A	G	-7.09E-05	PRS-CSx
Colorectum	PRS7	rs7479880	11	49014990	G	A	-1.91E-04	PRS-CSx
Colorectum	PRS7	rs7480140	11	46856335	G	A	1.57E-04	PRS-CSx
Colorectum	PRS7	rs7480193	11	85861419	A	G	7.18E-04	PRS-CSx
Colorectum	PRS7	rs748037	20	60971327	C	A	-3.35E-04	PRS-CSx
Colorectum	PRS7	rs7480567	11	15069269	G	A	2.46E-04	PRS-CSx
Colorectum	PRS7	rs7481739	11	383855	A	G	1.23E-03	PRS-CSx
Colorectum	PRS7	rs7481929	11	391049	C	T	9.03E-04	PRS-CSx
Colorectum	PRS7	rs748515	2	220311039	A	C	1.52E-03	PRS-CSx
Colorectum	PRS7	rs7485447	12	122611200	G	T	2.36E-04	PRS-CSx
Colorectum	PRS7	rs748651	4	1011115	C	T	-6.83E-04	PRS-CSx
Colorectum	PRS7	rs7487292	12	122614387	T	G	2.81E-04	PRS-CSx
Colorectum	PRS7	rs7487608	12	122614060	G	T	1.07E-04	PRS-CSx
Colorectum	PRS7	rs7488514	12	118520426	C	T	7.48E-04	PRS-CSx
Colorectum	PRS7	rs7488746	12	108254288	A	G	-6.92E-04	PRS-CSx
Colorectum	PRS7	rs7488857	12	122625212	G	A	2.65E-04	PRS-CSx
Colorectum	PRS7	rs7489858	13	49401007	G	A	6.91E-04	PRS-CSx
Colorectum	PRS7	rs748988	14	99786241	A	G	-4.88E-04	PRS-CSx
Colorectum	PRS7	rs749101	9	93729125	T	C	1.12E-03	PRS-CSx
Colorectum	PRS7	rs749103	9	93728502	G	A	1.41E-03	PRS-CSx
Colorectum	PRS7	rs749112	11	65361765	C	T	-4.33E-04	PRS-CSx
Colorectum	PRS7	rs7492599	14	72041458	G	A	4.55E-04	PRS-CSx
Colorectum	PRS7	rs7494781	15	32988138	T	C	1.93E-03	PRS-CSx
Colorectum	PRS7	rs7494978	15	82041299	G	A	-2.55E-04	PRS-CSx
Colorectum	PRS7	rs749671	16	31088347	G	A	2.15E-04	PRS-CSx
Colorectum	PRS7	rs749672	21	42487810	A	G	-5.91E-04	PRS-CSx

Colorectum	PRS7	rs749701	19	39189746	T	C	2.85E-04	PRS-CSx
Colorectum	PRS7	rs7497064	15	66990159	C	T	-1.05E-03	PRS-CSx
Colorectum	PRS7	rs749767	16	31124407	A	G	1.89E-04	PRS-CSx
Colorectum	PRS7	rs7499417	16	76448757	G	A	3.18E-04	PRS-CSx
Colorectum	PRS7	rs7502216	17	36612948	A	C	-3.43E-04	PRS-CSx
Colorectum	PRS7	rs7502442	17	81051007	T	G	1.08E-02	PRS-CSx
Colorectum	PRS7	rs7503053	17	46640290	C	T	5.13E-05	PRS-CSx
Colorectum	PRS7	rs750688	3	159358778	T	C	-8.90E-04	PRS-CSx
Colorectum	PRS7	rs7513079	1	12785494	G	T	3.42E-04	PRS-CSx
Colorectum	PRS7	rs7513496	1	246907206	T	G	9.01E-04	PRS-CSx
Colorectum	PRS7	rs7513712	1	167546460	G	A	4.31E-04	PRS-CSx
Colorectum	PRS7	rs7514366	1	192885481	A	G	-8.69E-04	PRS-CSx
Colorectum	PRS7	rs751450	10	73761015	G	A	-2.98E-04	PRS-CSx
Colorectum	PRS7	rs7514724	1	92173847	T	C	-3.77E-04	PRS-CSx
Colorectum	PRS7	rs7515244	1	15873386	A	G	-3.63E-04	PRS-CSx
Colorectum	PRS7	rs7515568	1	31438545	T	C	1.51E-04	PRS-CSx
Colorectum	PRS7	rs7517044	1	92198162	G	A	-5.80E-04	PRS-CSx
Colorectum	PRS7	rs7517569	1	221208709	T	C	2.83E-04	PRS-CSx
Colorectum	PRS7	rs751984	11	61278246	C	T	1.28E-03	PRS-CSx
Colorectum	PRS7	rs7519947	1	38447725	C	T	2.46E-04	PRS-CSx
Colorectum	PRS7	rs7521048	1	104837082	G	A	1.73E-04	PRS-CSx
Colorectum	PRS7	rs7522218	1	81102914	C	A	-5.09E-04	PRS-CSx
Colorectum	PRS7	rs7524102	1	22698447	G	A	-1.32E-03	PRS-CSx
Colorectum	PRS7	rs7524301	1	88873327	C	T	-9.48E-04	PRS-CSx
Colorectum	PRS7	rs752448	20	33592248	T	C	-5.78E-04	PRS-CSx
Colorectum	PRS7	rs7525553	1	98841829	T	C	-1.62E-03	PRS-CSx
Colorectum	PRS7	rs7526094	1	213833093	A	G	-1.30E-03	PRS-CSx
Colorectum	PRS7	rs7526237	1	246898134	T	C	7.40E-04	PRS-CSx
Colorectum	PRS7	rs7526614	1	101252966	A	G	-8.03E-04	PRS-CSx
Colorectum	PRS7	rs7527017	1	64643277	T	C	-3.43E-04	PRS-CSx
Colorectum	PRS7	rs7527185	1	192883411	T	C	-1.48E-03	PRS-CSx
Colorectum	PRS7	rs7528002	1	246938466	T	C	5.54E-04	PRS-CSx
Colorectum	PRS7	rs7528185	1	38452059	T	C	3.03E-04	PRS-CSx
Colorectum	PRS7	rs7528988	1	215248417	T	C	-1.16E-03	PRS-CSx
Colorectum	PRS7	rs752939	2	67311169	T	G	-9.41E-04	PRS-CSx
Colorectum	PRS7	rs7530027	1	58037504	T	C	6.99E-04	PRS-CSx
Colorectum	PRS7	rs7533274	1	62673225	A	G	1.96E-03	PRS-CSx
Colorectum	PRS7	rs7535528	1	2444414	A	G	-1.81E-04	PRS-CSx
Colorectum	PRS7	rs753643	4	56656956	C	T	-3.88E-03	PRS-CSx
Colorectum	PRS7	rs753693	3	112846786	C	T	-1.44E-04	PRS-CSx
Colorectum	PRS7	rs753719	19	411849	A	G	-8.28E-04	PRS-CSx
Colorectum	PRS7	rs753778	8	142228909	A	G	4.14E-04	PRS-CSx
Colorectum	PRS7	rs7537894	1	178204709	T	C	6.63E-04	PRS-CSx
Colorectum	PRS7	rs7538392	1	222222851	G	T	7.67E-04	PRS-CSx
Colorectum	PRS7	rs7539178	1	65383002	A	C	-4.02E-04	PRS-CSx
Colorectum	PRS7	rs7539245	1	105050416	C	T	3.40E-04	PRS-CSx
Colorectum	PRS7	rs7539994	1	65164851	C	T	-6.86E-04	PRS-CSx
Colorectum	PRS7	rs754005	11	36447430	A	C	-2.94E-04	PRS-CSx
Colorectum	PRS7	rs7542665	1	62673037	C	T	2.21E-03	PRS-CSx
Colorectum	PRS7	rs7543041	1	221934792	G	A	5.27E-05	PRS-CSx
Colorectum	PRS7	rs7543281	1	234743237	A	G	4.37E-04	PRS-CSx
Colorectum	PRS7	rs7543680	1	22731269	A	G	-4.26E-04	PRS-CSx
Colorectum	PRS7	rs7543683	1	167542337	G	A	2.04E-04	PRS-CSx
Colorectum	PRS7	rs7544082	1	48203990	A	C	4.03E-04	PRS-CSx
Colorectum	PRS7	rs7544183	1	101247948	C	T	-5.98E-04	PRS-CSx
Colorectum	PRS7	rs7544735	1	220998913	A	G	2.00E-04	PRS-CSx

Colorectum	PRS7	rs7546398	1	221935450	C	T	1.74E-04	PRS-CSx
Colorectum	PRS7	rs7550872	1	22711378	C	T	-6.28E-04	PRS-CSx
Colorectum	PRS7	rs7551075	1	204239603	T	C	2.89E-04	PRS-CSx
Colorectum	PRS7	rs7551288	1	55338679	G	A	-4.20E-05	PRS-CSx
Colorectum	PRS7	rs7551836	1	246960463	A	G	7.04E-04	PRS-CSx
Colorectum	PRS7	rs7554327	1	11937404	T	C	-6.89E-04	PRS-CSx
Colorectum	PRS7	rs7554445	1	167554337	G	T	9.03E-04	PRS-CSx
Colorectum	PRS7	rs7554730	1	164885860	A	C	5.37E-04	PRS-CSx
Colorectum	PRS7	rs755690	19	39167360	A	G	2.74E-04	PRS-CSx
Colorectum	PRS7	rs755726	20	60962624	C	T	-4.62E-04	PRS-CSx
Colorectum	PRS7	rs755812	15	31765808	T	C	5.47E-04	PRS-CSx
Colorectum	PRS7	rs7559315	2	43281032	C	T	8.58E-04	PRS-CSx
Colorectum	PRS7	rs7560077	2	174377174	C	T	-2.35E-03	PRS-CSx
Colorectum	PRS7	rs7560257	2	161085348	T	C	2.77E-03	PRS-CSx
Colorectum	PRS7	rs7561411	2	101636719	C	T	3.05E-04	PRS-CSx
Colorectum	PRS7	rs756152	11	15121130	G	T	9.20E-04	PRS-CSx
Colorectum	PRS7	rs756175	4	89999041	A	G	-2.43E-04	PRS-CSx
Colorectum	PRS7	rs756228	8	141069131	A	G	3.11E-04	PRS-CSx
Colorectum	PRS7	rs7565424	2	241243780	G	A	5.02E-04	PRS-CSx
Colorectum	PRS7	rs7565830	2	159810691	A	G	-7.34E-04	PRS-CSx
Colorectum	PRS7	rs7566330	2	199530324	A	G	3.03E-04	PRS-CSx
Colorectum	PRS7	rs7567362	2	168338520	A	G	4.85E-04	PRS-CSx
Colorectum	PRS7	rs7568353	2	204439759	G	A	1.47E-04	PRS-CSx
Colorectum	PRS7	rs7568824	2	208025957	A	C	-3.54E-04	PRS-CSx
Colorectum	PRS7	rs7569575	2	48717730	G	A	-1.81E-03	PRS-CSx
Colorectum	PRS7	rs7570877	2	48743051	A	G	1.24E-02	PRS-CSx
Colorectum	PRS7	rs7570950	2	74338942	C	T	-7.82E-04	PRS-CSx
Colorectum	PRS7	rs757169	5	128214819	C	T	3.65E-04	PRS-CSx
Colorectum	PRS7	rs7572023	2	154139453	G	A	3.37E-04	PRS-CSx
Colorectum	PRS7	rs7572685	2	67870225	T	C	-1.97E-03	PRS-CSx
Colorectum	PRS7	rs7574077	2	154151887	A	G	8.08E-04	PRS-CSx
Colorectum	PRS7	rs7576760	2	235491138	C	T	1.00E-03	PRS-CSx
Colorectum	PRS7	rs7577165	2	108968224	T	C	-6.97E-04	PRS-CSx
Colorectum	PRS7	rs7577524	2	67470196	A	C	1.13E-03	PRS-CSx
Colorectum	PRS7	rs7581184	2	11489531	G	A	-4.36E-04	PRS-CSx
Colorectum	PRS7	rs7582582	2	242710079	C	T	9.97E-04	PRS-CSx
Colorectum	PRS7	rs7582864	2	207172627	G	A	-2.54E-04	PRS-CSx
Colorectum	PRS7	rs7582883	2	43023004	C	T	5.64E-04	PRS-CSx
Colorectum	PRS7	rs7583085	2	43022968	G	A	3.42E-04	PRS-CSx
Colorectum	PRS7	rs758335	16	2011126	A	G	-1.30E-03	PRS-CSx
Colorectum	PRS7	rs7583825	2	144293163	T	C	1.31E-03	PRS-CSx
Colorectum	PRS7	rs7584906	2	237736216	T	C	9.05E-04	PRS-CSx
Colorectum	PRS7	rs7585513	2	160406890	A	C	-1.70E-04	PRS-CSx
Colorectum	PRS7	rs758575	10	118047671	G	A	4.34E-04	PRS-CSx
Colorectum	PRS7	rs758577	10	118047298	A	C	-3.84E-04	PRS-CSx
Colorectum	PRS7	rs7585914	2	95945568	G	A	-2.62E-04	PRS-CSx
Colorectum	PRS7	rs758593	5	149577919	G	A	-4.43E-04	PRS-CSx
Colorectum	PRS7	rs7586229	2	95945830	G	T	-1.60E-04	PRS-CSx
Colorectum	PRS7	rs758801	3	52536308	A	C	-4.31E-04	PRS-CSx
Colorectum	PRS7	rs7588654	2	219983030	C	T	1.32E-03	PRS-CSx
Colorectum	PRS7	rs758896	7	88846530	C	A	-5.59E-04	PRS-CSx
Colorectum	PRS7	rs7589259	2	169027044	C	T	-2.96E-04	PRS-CSx
Colorectum	PRS7	rs7590354	2	100497843	C	T	3.45E-04	PRS-CSx
Colorectum	PRS7	rs7592036	2	42604109	C	T	-3.65E-05	PRS-CSx
Colorectum	PRS7	rs7592226	2	12611100	G	A	2.79E-03	PRS-CSx
Colorectum	PRS7	rs7592676	2	216710572	G	A	-6.67E-04	PRS-CSx

Colorectum	PRS7	rs7593466	2	209099479	T	G	-7.17E-04	PRS-CSx
Colorectum	PRS7	rs7594831	2	204439701	T	C	1.64E-04	PRS-CSx
Colorectum	PRS7	rs7594926	2	174342575	C	T	2.05E-03	PRS-CSx
Colorectum	PRS7	rs7595353	2	229232050	C	T	-6.48E-04	PRS-CSx
Colorectum	PRS7	rs7595446	2	159966048	A	G	-2.77E-04	PRS-CSx
Colorectum	PRS7	rs7595502	2	204440081	A	G	2.23E-04	PRS-CSx
Colorectum	PRS7	rs7595630	2	242896724	C	A	-2.55E-04	PRS-CSx
Colorectum	PRS7	rs759663	2	208080975	G	A	-9.99E-05	PRS-CSx
Colorectum	PRS7	rs7597362	2	59656639	T	C	8.99E-04	PRS-CSx
Colorectum	PRS7	rs7598486	2	19375011	C	T	5.51E-04	PRS-CSx
Colorectum	PRS7	rs7598637	2	43102327	T	C	-5.81E-05	PRS-CSx
Colorectum	PRS7	rs7598708	2	124269785	A	G	-4.49E-04	PRS-CSx
Colorectum	PRS7	rs7598890	2	131809197	T	C	-5.94E-04	PRS-CSx
Colorectum	PRS7	rs7599536	2	69396953	G	A	-2.39E-03	PRS-CSx
Colorectum	PRS7	rs7600286	2	208046557	C	T	-3.23E-04	PRS-CSx
Colorectum	PRS7	rs760098	9	33792265	G	A	-2.05E-04	PRS-CSx
Colorectum	PRS7	rs7601314	2	43281211	A	G	5.37E-04	PRS-CSx
Colorectum	PRS7	rs7601633	2	62394127	G	A	-1.32E-04	PRS-CSx
Colorectum	PRS7	rs7602356	2	67793687	A	C	1.41E-03	PRS-CSx
Colorectum	PRS7	rs7602568	2	28584315	T	C	7.50E-04	PRS-CSx
Colorectum	PRS7	rs760316	3	60098968	C	T	9.27E-04	PRS-CSx
Colorectum	PRS7	rs7603172	2	208020941	A	G	-4.25E-04	PRS-CSx
Colorectum	PRS7	rs7603261	2	176483606	C	T	1.55E-03	PRS-CSx
Colorectum	PRS7	rs7605161	2	169059591	A	C	-1.96E-04	PRS-CSx
Colorectum	PRS7	rs7605690	2	77423231	G	A	8.30E-04	PRS-CSx
Colorectum	PRS7	rs760718	22	36900806	G	A	-1.67E-04	PRS-CSx
Colorectum	PRS7	rs7607421	2	220792320	T	C	3.00E-04	PRS-CSx
Colorectum	PRS7	rs760782	6	35448189	T	C	2.45E-04	PRS-CSx
Colorectum	PRS7	rs7608012	2	208037900	C	T	-2.88E-04	PRS-CSx
Colorectum	PRS7	rs7609044	2	107262863	A	G	-1.20E-03	PRS-CSx
Colorectum	PRS7	rs7609267	2	204439982	C	T	2.17E-04	PRS-CSx
Colorectum	PRS7	rs7609304	2	202971733	T	C	-6.75E-04	PRS-CSx
Colorectum	PRS7	rs7611109	3	133823507	T	C	-2.75E-04	PRS-CSx
Colorectum	PRS7	rs7612426	3	3315392	G	A	-4.61E-04	PRS-CSx
Colorectum	PRS7	rs7612448	3	113270402	G	A	-3.07E-04	PRS-CSx
Colorectum	PRS7	rs761272	20	47315581	G	A	-2.94E-04	PRS-CSx
Colorectum	PRS7	rs7613502	3	152328725	G	T	4.87E-05	PRS-CSx
Colorectum	PRS7	rs7613596	3	11690450	T	C	1.08E-03	PRS-CSx
Colorectum	PRS7	rs7614038	3	151635742	T	C	-2.27E-05	PRS-CSx
Colorectum	PRS7	rs7614424	3	52566354	T	C	-7.40E-04	PRS-CSx
Colorectum	PRS7	rs7616160	3	37141977	G	A	-3.88E-04	PRS-CSx
Colorectum	PRS7	rs7616330	3	71115751	C	A	-9.58E-04	PRS-CSx
Colorectum	PRS7	rs7617171	3	25360230	C	T	3.34E-04	PRS-CSx
Colorectum	PRS7	rs7617193	3	112848851	T	C	-1.06E-04	PRS-CSx
Colorectum	PRS7	rs7620307	3	112977050	A	C	-4.77E-04	PRS-CSx
Colorectum	PRS7	rs7620746	3	122528202	A	G	-3.57E-04	PRS-CSx
Colorectum	PRS7	rs7622619	3	152840761	T	G	-5.44E-04	PRS-CSx
Colorectum	PRS7	rs7623286	3	64624377	G	A	3.38E-04	PRS-CSx
Colorectum	PRS7	rs7623860	3	41475001	C	T	-3.78E-04	PRS-CSx
Colorectum	PRS7	rs7624589	3	173495499	A	G	3.58E-04	PRS-CSx
Colorectum	PRS7	rs7627477	3	133704165	T	C	5.49E-03	PRS-CSx
Colorectum	PRS7	rs7628338	3	72432888	G	A	6.59E-04	PRS-CSx
Colorectum	PRS7	rs7628617	3	41209963	T	C	-1.32E-04	PRS-CSx
Colorectum	PRS7	rs7628626	3	119244421	A	C	-7.15E-04	PRS-CSx
Colorectum	PRS7	rs762864	4	3419677	G	A	-1.27E-03	PRS-CSx
Colorectum	PRS7	rs7629340	3	61246997	G	A	-5.21E-05	PRS-CSx

Colorectum	PRS7	rs7629416	3	133730932	T	C	-3.06E-04	PRS-CSx
Colorectum	PRS7	rs7629797	3	173509592	C	T	2.03E-04	PRS-CSx
Colorectum	PRS7	rs7630221	3	37192199	G	A	-1.91E-04	PRS-CSx
Colorectum	PRS7	rs7631034	3	184412473	T	C	-6.84E-04	PRS-CSx
Colorectum	PRS7	rs7631605	3	37234589	T	C	-2.09E-04	PRS-CSx
Colorectum	PRS7	rs7632108	3	37258063	C	A	-1.74E-04	PRS-CSx
Colorectum	PRS7	rs7632691	3	133806218	T	C	-2.97E-04	PRS-CSx
Colorectum	PRS7	rs7632885	3	140178485	G	A	-3.69E-04	PRS-CSx
Colorectum	PRS7	rs763328	14	53707009	G	A	9.68E-04	PRS-CSx
Colorectum	PRS7	rs7634419	3	173860980	G	A	6.08E-04	PRS-CSx
Colorectum	PRS7	rs7634937	3	113611468	T	C	-3.57E-04	PRS-CSx
Colorectum	PRS7	rs763585	19	57821889	A	G	-5.72E-04	PRS-CSx
Colorectum	PRS7	rs763591	16	60844443	A	G	-4.92E-04	PRS-CSx
Colorectum	PRS7	rs763623	9	87539664	A	G	2.68E-04	PRS-CSx
Colorectum	PRS7	rs7636925	3	64616582	A	G	5.90E-04	PRS-CSx
Colorectum	PRS7	rs763713	2	71448455	G	T	-2.43E-04	PRS-CSx
Colorectum	PRS7	rs7637198	3	173861404	G	T	8.66E-04	PRS-CSx
Colorectum	PRS7	rs7637618	3	113176122	A	G	-5.12E-05	PRS-CSx
Colorectum	PRS7	rs7637860	3	122574451	A	G	-1.92E-04	PRS-CSx
Colorectum	PRS7	rs7638936	3	156339632	A	G	-6.93E-04	PRS-CSx
Colorectum	PRS7	rs7640062	3	64617699	A	G	7.81E-04	PRS-CSx
Colorectum	PRS7	rs7640492	3	173514219	G	A	8.82E-05	PRS-CSx
Colorectum	PRS7	rs764192	12	46363027	G	T	-3.54E-04	PRS-CSx
Colorectum	PRS7	rs7642276	3	24797525	T	C	-7.74E-04	PRS-CSx
Colorectum	PRS7	rs7642515	3	67908896	A	G	-6.28E-04	PRS-CSx
Colorectum	PRS7	rs7644234	3	119691611	G	T	2.85E-04	PRS-CSx
Colorectum	PRS7	rs764457	1	64981488	C	T	5.95E-04	PRS-CSx
Colorectum	PRS7	rs7645388	3	73765431	G	A	-2.70E-04	PRS-CSx
Colorectum	PRS7	rs764597	20	33161225	A	G	1.85E-05	PRS-CSx
Colorectum	PRS7	rs764606	12	51313531	A	G	4.41E-04	PRS-CSx
Colorectum	PRS7	rs7646341	3	152329006	A	G	8.09E-05	PRS-CSx
Colorectum	PRS7	rs7646392	3	133699575	T	C	9.39E-04	PRS-CSx
Colorectum	PRS7	rs7647940	3	69246903	A	C	-1.07E-03	PRS-CSx
Colorectum	PRS7	rs7648540	3	64565501	C	A	-6.87E-04	PRS-CSx
Colorectum	PRS7	rs7649025	3	67907431	G	A	-3.70E-04	PRS-CSx
Colorectum	PRS7	rs7649034	3	67907453	G	A	-3.18E-04	PRS-CSx
Colorectum	PRS7	rs7649344	3	37006396	C	T	-1.35E-04	PRS-CSx
Colorectum	PRS7	rs7652387	3	150707822	A	C	1.92E-03	PRS-CSx
Colorectum	PRS7	rs7652820	3	64621779	A	G	2.89E-04	PRS-CSx
Colorectum	PRS7	rs7653175	3	133823275	A	G	-9.01E-05	PRS-CSx
Colorectum	PRS7	rs765392	6	11955091	A	G	3.63E-04	PRS-CSx
Colorectum	PRS7	rs7654860	4	105781402	A	G	-4.55E-04	PRS-CSx
Colorectum	PRS7	rs765516	6	117728118	T	C	1.00E-04	PRS-CSx
Colorectum	PRS7	rs7655641	4	60521175	A	C	-6.89E-04	PRS-CSx
Colorectum	PRS7	rs7655737	4	41203215	T	C	-9.07E-04	PRS-CSx
Colorectum	PRS7	rs765578	2	176663264	T	C	-8.23E-04	PRS-CSx
Colorectum	PRS7	rs7655989	4	115449290	C	T	2.32E-04	PRS-CSx
Colorectum	PRS7	rs7656522	4	155411065	T	C	-3.70E-04	PRS-CSx
Colorectum	PRS7	rs7657160	4	153828846	T	C	-3.63E-04	PRS-CSx
Colorectum	PRS7	rs7657579	4	22447508	T	C	-2.93E-04	PRS-CSx
Colorectum	PRS7	rs765825	15	102014347	A	G	4.37E-04	PRS-CSx
Colorectum	PRS7	rs7660636	4	105869983	T	C	-5.58E-04	PRS-CSx
Colorectum	PRS7	rs7662541	4	146434680	T	C	7.17E-04	PRS-CSx
Colorectum	PRS7	rs7663689	4	142688981	T	C	5.33E-04	PRS-CSx
Colorectum	PRS7	rs766427	21	36003798	G	A	-5.06E-04	PRS-CSx
Colorectum	PRS7	rs7664303	4	153829996	A	G	-3.82E-04	PRS-CSx

Colorectum	PRS7	rs7664748	4	139316303	A	G	-8.67E-04	PRS-CSx
Colorectum	PRS7	rs7664990	4	75564110	T	C	-1.92E-03	PRS-CSx
Colorectum	PRS7	rs7665426	4	175325686	G	T	-3.85E-04	PRS-CSx
Colorectum	PRS7	rs7665770	4	115429539	T	C	4.20E-05	PRS-CSx
Colorectum	PRS7	rs7667406	4	57503077	G	A	-1.68E-03	PRS-CSx
Colorectum	PRS7	rs7668376	4	80984670	C	T	8.78E-04	PRS-CSx
Colorectum	PRS7	rs7668402	4	38380708	G	A	-7.24E-04	PRS-CSx
Colorectum	PRS7	rs7670486	4	146453921	A	G	3.39E-04	PRS-CSx
Colorectum	PRS7	rs7671003	4	41203317	C	T	-7.11E-04	PRS-CSx
Colorectum	PRS7	rs7671026	4	115408892	C	A	1.38E-04	PRS-CSx
Colorectum	PRS7	rs7671167	4	89883979	C	T	2.62E-04	PRS-CSx
Colorectum	PRS7	rs7671690	4	82964509	C	A	3.70E-04	PRS-CSx
Colorectum	PRS7	rs7672789	4	57482846	A	G	-3.90E-04	PRS-CSx
Colorectum	PRS7	rs7673384	4	167540460	C	T	2.49E-04	PRS-CSx
Colorectum	PRS7	rs767441	15	48905816	C	T	1.29E-03	PRS-CSx
Colorectum	PRS7	rs7674464	4	44372045	A	G	-1.45E-03	PRS-CSx
Colorectum	PRS7	rs7674525	4	94804559	C	T	1.35E-04	PRS-CSx
Colorectum	PRS7	rs767471	6	26557854	C	T	-1.16E-06	PRS-CSx
Colorectum	PRS7	rs7675971	4	64285084	C	T	6.96E-04	PRS-CSx
Colorectum	PRS7	rs7676193	4	18889088	A	G	2.43E-04	PRS-CSx
Colorectum	PRS7	rs7676684	4	115413527	T	C	8.93E-05	PRS-CSx
Colorectum	PRS7	rs767707	1	167560542	A	G	4.63E-04	PRS-CSx
Colorectum	PRS7	rs7678120	4	41202583	A	G	-8.70E-04	PRS-CSx
Colorectum	PRS7	rs7678790	4	116091803	T	C	-6.42E-04	PRS-CSx
Colorectum	PRS7	rs7680497	4	18885282	C	T	1.66E-04	PRS-CSx
Colorectum	PRS7	rs768080	16	86192565	T	G	3.99E-04	PRS-CSx
Colorectum	PRS7	rs7681151	4	94809132	C	T	3.07E-04	PRS-CSx
Colorectum	PRS7	rs7681803	4	183410241	T	C	-1.30E-04	PRS-CSx
Colorectum	PRS7	rs7682716	4	156676272	C	A	7.28E-04	PRS-CSx
Colorectum	PRS7	rs7683746	4	41203125	A	G	-8.98E-04	PRS-CSx
Colorectum	PRS7	rs7684187	4	123341159	G	A	4.65E-04	PRS-CSx
Colorectum	PRS7	rs7686589	4	151337243	T	C	4.20E-04	PRS-CSx
Colorectum	PRS7	rs7686909	4	174257693	C	T	2.11E-03	PRS-CSx
Colorectum	PRS7	rs7687343	4	151442374	T	C	-2.70E-04	PRS-CSx
Colorectum	PRS7	rs768825	13	71770610	G	T	4.84E-04	PRS-CSx
Colorectum	PRS7	rs768826	13	71770641	C	T	6.44E-04	PRS-CSx
Colorectum	PRS7	rs7688410	4	151403684	C	T	4.50E-04	PRS-CSx
Colorectum	PRS7	rs768962	19	3615164	G	A	3.27E-04	PRS-CSx
Colorectum	PRS7	rs768974	7	104135160	T	C	6.70E-04	PRS-CSx
Colorectum	PRS7	rs7690080	4	151467872	A	C	3.24E-04	PRS-CSx
Colorectum	PRS7	rs7690426	4	115384107	T	C	-3.10E-04	PRS-CSx
Colorectum	PRS7	rs7692390	4	22509597	C	T	-4.37E-04	PRS-CSx
Colorectum	PRS7	rs7692571	4	115401948	G	A	2.50E-04	PRS-CSx
Colorectum	PRS7	rs7694604	4	146430293	G	T	7.45E-04	PRS-CSx
Colorectum	PRS7	rs7696793	4	118789715	C	T	1.86E-04	PRS-CSx
Colorectum	PRS7	rs7697048	4	22509714	C	T	-9.17E-04	PRS-CSx
Colorectum	PRS7	rs7697323	4	7683417	A	G	9.61E-04	PRS-CSx
Colorectum	PRS7	rs7697724	4	127638432	C	A	-5.45E-04	PRS-CSx
Colorectum	PRS7	rs7697728	4	18922514	C	T	-5.61E-04	PRS-CSx
Colorectum	PRS7	rs7697959	4	22470931	T	C	-2.92E-04	PRS-CSx
Colorectum	PRS7	rs7698391	4	115413613	A	G	1.61E-04	PRS-CSx
Colorectum	PRS7	rs7698460	4	156666219	A	G	4.47E-04	PRS-CSx
Colorectum	PRS7	rs7698790	4	115413803	A	G	1.19E-04	PRS-CSx
Colorectum	PRS7	rs7698944	4	146436351	G	A	6.70E-04	PRS-CSx
Colorectum	PRS7	rs7700023	4	43974714	T	C	-1.79E-03	PRS-CSx
Colorectum	PRS7	rs7700052	4	115656533	A	G	2.28E-04	PRS-CSx

Colorectum	PRS7	rs7701678	5	177553108	C	T	3.31E-04	PRS-CSx
Colorectum	PRS7	rs7701981	5	40136753	A	G	3.18E-04	PRS-CSx
Colorectum	PRS7	rs7705714	5	125971699	T	C	6.94E-04	PRS-CSx
Colorectum	PRS7	rs7707830	5	100914502	T	G	1.50E-04	PRS-CSx
Colorectum	PRS7	rs7708079	5	39648564	C	T	2.94E-04	PRS-CSx
Colorectum	PRS7	rs7708680	5	40697951	C	A	1.28E-04	PRS-CSx
Colorectum	PRS7	rs7709361	5	141513854	T	C	5.62E-04	PRS-CSx
Colorectum	PRS7	rs7709563	5	8603334	C	T	3.26E-04	PRS-CSx
Colorectum	PRS7	rs7711006	5	40151043	T	C	5.09E-04	PRS-CSx
Colorectum	PRS7	rs7711822	5	113185123	A	G	3.34E-03	PRS-CSx
Colorectum	PRS7	rs7712189	5	39807589	T	C	-7.11E-05	PRS-CSx
Colorectum	PRS7	rs7712617	5	139888590	C	A	1.71E-04	PRS-CSx
Colorectum	PRS7	rs7713645	5	67527326	A	C	-2.56E-04	PRS-CSx
Colorectum	PRS7	rs7713805	5	31188433	T	C	5.11E-03	PRS-CSx
Colorectum	PRS7	rs7714584	5	150270420	G	A	-6.66E-04	PRS-CSx
Colorectum	PRS7	rs7714851	5	164475774	T	C	-7.36E-07	PRS-CSx
Colorectum	PRS7	rs7716515	5	39655343	A	G	2.33E-04	PRS-CSx
Colorectum	PRS7	rs7717199	5	134246855	C	T	3.00E-03	PRS-CSx
Colorectum	PRS7	rs7717955	5	35862841	T	C	5.78E-04	PRS-CSx
Colorectum	PRS7	rs7718574	5	125989661	A	G	9.07E-04	PRS-CSx
Colorectum	PRS7	rs7719521	5	151049404	A	C	3.56E-04	PRS-CSx
Colorectum	PRS7	rs7719851	5	61941043	A	G	1.67E-03	PRS-CSx
Colorectum	PRS7	rs7720499	5	56021821	T	C	5.99E-04	PRS-CSx
Colorectum	PRS7	rs7721220	5	177540223	A	G	-1.60E-03	PRS-CSx
Colorectum	PRS7	rs7723887	5	134543554	T	C	1.30E-04	PRS-CSx
Colorectum	PRS7	rs7724036	5	150284808	T	C	-1.53E-04	PRS-CSx
Colorectum	PRS7	rs7724208	5	27528745	A	C	6.50E-04	PRS-CSx
Colorectum	PRS7	rs7724393	5	40104157	A	C	5.94E-04	PRS-CSx
Colorectum	PRS7	rs772445	2	7851303	T	G	-7.61E-04	PRS-CSx
Colorectum	PRS7	rs772457	2	7871951	A	G	-7.60E-04	PRS-CSx
Colorectum	PRS7	rs772458	2	7871194	T	C	-8.93E-04	PRS-CSx
Colorectum	PRS7	rs7725469	5	88340667	A	G	5.03E-04	PRS-CSx
Colorectum	PRS7	rs7726237	5	40688552	G	A	3.22E-04	PRS-CSx
Colorectum	PRS7	rs772731	12	77335215	A	G	-5.76E-04	PRS-CSx
Colorectum	PRS7	rs772735	12	77340586	A	G	-4.50E-04	PRS-CSx
Colorectum	PRS7	rs7728690	5	88411214	C	T	3.04E-04	PRS-CSx
Colorectum	PRS7	rs7730375	5	39666907	G	T	7.40E-05	PRS-CSx
Colorectum	PRS7	rs773239	3	106580208	T	C	-5.21E-04	PRS-CSx
Colorectum	PRS7	rs7732415	5	177572561	G	A	8.97E-04	PRS-CSx
Colorectum	PRS7	rs7732718	5	100668580	T	C	1.62E-03	PRS-CSx
Colorectum	PRS7	rs7733850	5	141515564	C	T	8.18E-04	PRS-CSx
Colorectum	PRS7	rs7734427	5	74328068	T	C	-2.52E-04	PRS-CSx
Colorectum	PRS7	rs7734944	5	39646530	A	C	1.51E-04	PRS-CSx
Colorectum	PRS7	rs773507	9	93974553	G	T	-2.91E-04	PRS-CSx
Colorectum	PRS7	rs773515	9	93982932	T	C	1.03E-03	PRS-CSx
Colorectum	PRS7	rs7738117	6	4939281	A	G	-4.68E-04	PRS-CSx
Colorectum	PRS7	rs7738252	6	13060568	G	A	5.24E-04	PRS-CSx
Colorectum	PRS7	rs7738548	6	29421209	A	G	2.20E-04	PRS-CSx
Colorectum	PRS7	rs7738571	6	25566503	T	C	-3.75E-04	PRS-CSx
Colorectum	PRS7	rs7738722	6	29414286	G	A	1.37E-04	PRS-CSx
Colorectum	PRS7	rs7738742	6	29414346	G	A	1.45E-04	PRS-CSx
Colorectum	PRS7	rs7739243	6	29414613	G	A	5.20E-05	PRS-CSx
Colorectum	PRS7	rs7740572	6	92965004	A	G	-6.21E-04	PRS-CSx
Colorectum	PRS7	rs7742447	6	143712449	C	T	1.37E-03	PRS-CSx
Colorectum	PRS7	rs7743076	6	148787416	C	T	7.14E-04	PRS-CSx
Colorectum	PRS7	rs7743102	6	148787467	G	T	5.81E-04	PRS-CSx

Colorectum	PRS7	rs7743566	6	100822271	G	A	-3.00E-04	PRS-CSx
Colorectum	PRS7	rs7746164	6	162355755	T	C	-3.65E-04	PRS-CSx
Colorectum	PRS7	rs7746517	6	152663929	A	C	6.46E-04	PRS-CSx
Colorectum	PRS7	rs7746536	6	117799572	C	A	3.54E-04	PRS-CSx
Colorectum	PRS7	rs7747189	6	100857869	G	A	-4.26E-04	PRS-CSx
Colorectum	PRS7	rs7749149	6	25781139	G	A	2.58E-05	PRS-CSx
Colorectum	PRS7	rs7749629	6	43714001	C	T	-8.52E-04	PRS-CSx
Colorectum	PRS7	rs7750106	6	28223680	A	C	4.06E-04	PRS-CSx
Colorectum	PRS7	rs7751138	6	127286795	C	T	2.14E-04	PRS-CSx
Colorectum	PRS7	rs7751705	6	29441618	T	C	1.58E-04	PRS-CSx
Colorectum	PRS7	rs775214	3	113010950	C	T	-1.33E-04	PRS-CSx
Colorectum	PRS7	rs775227	3	112995074	C	A	-1.79E-03	PRS-CSx
Colorectum	PRS7	rs7756525	6	41675740	T	C	-1.51E-03	PRS-CSx
Colorectum	PRS7	rs7756687	6	12506386	A	G	1.33E-03	PRS-CSx
Colorectum	PRS7	rs7756992	6	20679709	G	A	-1.05E-04	PRS-CSx
Colorectum	PRS7	rs7757215	6	28212489	A	C	1.11E-03	PRS-CSx
Colorectum	PRS7	rs7757405	6	35423489	A	G	2.69E-04	PRS-CSx
Colorectum	PRS7	rs7757514	6	106834984	C	T	-1.25E-03	PRS-CSx
Colorectum	PRS7	rs7757873	6	39110904	A	G	-2.40E-03	PRS-CSx
Colorectum	PRS7	rs7759272	6	29684994	C	T	3.42E-04	PRS-CSx
Colorectum	PRS7	rs7759474	6	29731635	T	C	-1.95E-04	PRS-CSx
Colorectum	PRS7	rs7760371	6	143712252	A	G	1.60E-03	PRS-CSx
Colorectum	PRS7	rs7760528	6	20442354	C	T	3.64E-04	PRS-CSx
Colorectum	PRS7	rs7763979	6	117795970	A	G	3.97E-04	PRS-CSx
Colorectum	PRS7	rs7765791	6	29410093	C	T	3.76E-04	PRS-CSx
Colorectum	PRS7	rs7766082	6	29468006	C	T	2.74E-05	PRS-CSx
Colorectum	PRS7	rs7766626	6	11256000	C	T	6.43E-04	PRS-CSx
Colorectum	PRS7	rs7767391	6	20725240	C	T	-1.85E-04	PRS-CSx
Colorectum	PRS7	rs7767930	6	25589592	A	G	-4.23E-04	PRS-CSx
Colorectum	PRS7	rs7768491	6	12102615	C	T	4.47E-04	PRS-CSx
Colorectum	PRS7	rs7768854	6	29428831	G	T	3.38E-04	PRS-CSx
Colorectum	PRS7	rs776978	2	229665868	C	T	6.73E-04	PRS-CSx
Colorectum	PRS7	rs7770048	6	32334754	T	C	-1.51E-04	PRS-CSx
Colorectum	PRS7	rs7771335	6	29448038	G	A	3.73E-04	PRS-CSx
Colorectum	PRS7	rs7772827	6	28301143	C	T	1.22E-04	PRS-CSx
Colorectum	PRS7	rs7773358	6	29701800	T	C	1.78E-05	PRS-CSx
Colorectum	PRS7	rs7775228	6	32658079	C	T	3.46E-04	PRS-CSx
Colorectum	PRS7	rs7776083	6	131436888	T	G	6.58E-04	PRS-CSx
Colorectum	PRS7	rs7776973	7	77237390	T	C	-1.34E-04	PRS-CSx
Colorectum	PRS7	rs7777882	7	70511536	C	T	-1.47E-03	PRS-CSx
Colorectum	PRS7	rs7778525	7	46919944	C	T	2.81E-04	PRS-CSx
Colorectum	PRS7	rs7781395	7	45146391	A	C	-4.37E-04	PRS-CSx
Colorectum	PRS7	rs7781467	7	41936908	T	C	6.21E-04	PRS-CSx
Colorectum	PRS7	rs7782249	7	37121150	T	C	7.63E-04	PRS-CSx
Colorectum	PRS7	rs7783016	7	41930721	G	A	5.69E-04	PRS-CSx
Colorectum	PRS7	rs7784041	7	124288203	G	A	3.19E-04	PRS-CSx
Colorectum	PRS7	rs7785365	7	88026221	G	T	2.17E-04	PRS-CSx
Colorectum	PRS7	rs7785613	7	151104603	A	G	9.98E-04	PRS-CSx
Colorectum	PRS7	rs7787218	7	33938388	C	T	-1.81E-03	PRS-CSx
Colorectum	PRS7	rs7788746	7	99612405	G	T	-4.56E-04	PRS-CSx
Colorectum	PRS7	rs7789024	7	90826257	G	A	-1.34E-03	PRS-CSx
Colorectum	PRS7	rs7789805	7	45153306	C	A	-6.23E-04	PRS-CSx
Colorectum	PRS7	rs779105	8	4120070	C	T	-1.08E-03	PRS-CSx
Colorectum	PRS7	rs7792547	7	151123529	A	G	-3.19E-04	PRS-CSx
Colorectum	PRS7	rs7793355	7	124215350	C	T	6.20E-04	PRS-CSx
Colorectum	PRS7	rs7793598	7	83759108	C	T	-3.21E-04	PRS-CSx

Colorectum	PRS7	rs7795102	7	45028992	G	A	-1.69E-04	PRS-CSx
Colorectum	PRS7	rs7795371	7	77362504	G	A	-1.93E-04	PRS-CSx
Colorectum	PRS7	rs7797697	7	46920023	T	C	4.12E-04	PRS-CSx
Colorectum	PRS7	rs7797895	7	93888893	C	A	9.73E-04	PRS-CSx
Colorectum	PRS7	rs7798194	7	151117695	T	C	1.00E-03	PRS-CSx
Colorectum	PRS7	rs7798290	7	88828320	G	A	-2.19E-04	PRS-CSx
Colorectum	PRS7	rs7798679	7	47503866	C	T	-4.43E-04	PRS-CSx
Colorectum	PRS7	rs7798936	7	95413253	A	G	3.86E-04	PRS-CSx
Colorectum	PRS7	rs7799687	7	28397794	A	C	7.28E-04	PRS-CSx
Colorectum	PRS7	rs7799735	7	13883905	C	T	-8.96E-04	PRS-CSx
Colorectum	PRS7	rs7799974	7	28397882	A	G	7.48E-04	PRS-CSx
Colorectum	PRS7	rs7800695	7	45090506	T	G	-5.73E-04	PRS-CSx
Colorectum	PRS7	rs7802116	7	16632086	T	C	-7.47E-04	PRS-CSx
Colorectum	PRS7	rs7803700	7	150464171	T	G	1.43E-03	PRS-CSx
Colorectum	PRS7	rs7806340	7	151122503	T	C	-9.16E-04	PRS-CSx
Colorectum	PRS7	rs7806522	7	34089823	C	T	6.23E-04	PRS-CSx
Colorectum	PRS7	rs7810453	7	46919715	A	C	2.36E-04	PRS-CSx
Colorectum	PRS7	rs7810512	7	45150331	C	A	-1.27E-03	PRS-CSx
Colorectum	PRS7	rs7811892	7	127923778	T	C	-4.08E-04	PRS-CSx
Colorectum	PRS7	rs7812312	8	96020400	C	T	6.32E-04	PRS-CSx
Colorectum	PRS7	rs7815605	8	128590946	G	A	-8.93E-04	PRS-CSx
Colorectum	PRS7	rs7819400	8	22263666	G	A	8.82E-04	PRS-CSx
Colorectum	PRS7	rs7820286	8	65796450	G	T	-8.70E-04	PRS-CSx
Colorectum	PRS7	rs7820543	8	121046348	C	A	-2.75E-04	PRS-CSx
Colorectum	PRS7	rs7820880	8	65762549	G	A	2.24E-04	PRS-CSx
Colorectum	PRS7	rs7820981	8	128400176	T	C	3.11E-03	PRS-CSx
Colorectum	PRS7	rs7822662	8	121064449	A	G	-3.75E-04	PRS-CSx
Colorectum	PRS7	rs7823271	8	117703509	G	T	1.75E-03	PRS-CSx
Colorectum	PRS7	rs7824060	8	96439026	A	G	-7.82E-04	PRS-CSx
Colorectum	PRS7	rs7824313	8	5503800	C	T	1.14E-03	PRS-CSx
Colorectum	PRS7	rs7825233	8	2058991	T	C	-9.05E-04	PRS-CSx
Colorectum	PRS7	rs7826196	8	96029002	G	A	4.86E-04	PRS-CSx
Colorectum	PRS7	rs7828067	8	117796195	C	T	1.20E-03	PRS-CSx
Colorectum	PRS7	rs7829101	8	121053426	G	A	-4.21E-04	PRS-CSx
Colorectum	PRS7	rs7830860	8	117652174	T	C	-1.52E-04	PRS-CSx
Colorectum	PRS7	rs7832345	8	23624725	A	G	-1.83E-03	PRS-CSx
Colorectum	PRS7	rs7833054	8	117681031	A	G	-2.69E-04	PRS-CSx
Colorectum	PRS7	rs7833073	8	121094710	T	C	-4.98E-04	PRS-CSx
Colorectum	PRS7	rs7833302	8	22256310	A	G	1.84E-03	PRS-CSx
Colorectum	PRS7	rs7833587	8	65800457	G	A	-1.44E-03	PRS-CSx
Colorectum	PRS7	rs7834164	8	117644462	G	A	6.22E-05	PRS-CSx
Colorectum	PRS7	rs7835387	8	20150536	C	T	1.22E-03	PRS-CSx
Colorectum	PRS7	rs7836087	8	121072679	T	C	-6.37E-04	PRS-CSx
Colorectum	PRS7	rs7836309	8	116154006	G	T	7.44E-04	PRS-CSx
Colorectum	PRS7	rs7836809	8	41600125	G	T	6.21E-04	PRS-CSx
Colorectum	PRS7	rs7837096	8	120171412	C	A	1.50E-05	PRS-CSx
Colorectum	PRS7	rs7837328	8	128423127	A	G	1.89E-03	PRS-CSx
Colorectum	PRS7	rs7838499	8	59781086	C	T	-8.14E-04	PRS-CSx
Colorectum	PRS7	rs7839361	8	117645687	C	T	-1.98E-04	PRS-CSx
Colorectum	PRS7	rs7840142	8	65796677	G	A	-1.17E-03	PRS-CSx
Colorectum	PRS7	rs7841228	8	128460878	A	G	1.26E-04	PRS-CSx
Colorectum	PRS7	rs7841264	8	128466814	C	T	3.50E-05	PRS-CSx
Colorectum	PRS7	rs7841283	8	131668278	T	C	-1.07E-03	PRS-CSx
Colorectum	PRS7	rs7842055	8	121244089	T	G	-3.10E-04	PRS-CSx
Colorectum	PRS7	rs7842552	8	128431694	G	A	1.30E-03	PRS-CSx
Colorectum	PRS7	rs7843131	8	142366200	C	T	1.04E-03	PRS-CSx

Colorectum	PRS7	rs7843721	8	22917847	T	G	8.56E-04	PRS-CSx
Colorectum	PRS7	rs7843852	8	117676506	C	T	-1.63E-04	PRS-CSx
Colorectum	PRS7	rs7844095	8	21462456	A	G	1.41E-04	PRS-CSx
Colorectum	PRS7	rs7846316	8	117722555	G	T	-4.31E-04	PRS-CSx
Colorectum	PRS7	rs7846979	9	117432944	T	C	-5.21E-04	PRS-CSx
Colorectum	PRS7	rs7848837	9	101785558	G	T	-1.48E-04	PRS-CSx
Colorectum	PRS7	rs7849845	9	33675629	C	A	-1.04E-04	PRS-CSx
Colorectum	PRS7	rs7850032	9	85100694	G	A	-3.56E-04	PRS-CSx
Colorectum	PRS7	rs7851513	9	9842176	A	C	-1.29E-05	PRS-CSx
Colorectum	PRS7	rs7851766	9	653505	G	A	3.10E-04	PRS-CSx
Colorectum	PRS7	rs7851917	9	86890073	C	A	8.26E-04	PRS-CSx
Colorectum	PRS7	rs7852848	9	31192642	A	G	-2.17E-05	PRS-CSx
Colorectum	PRS7	rs7854112	9	101804329	C	T	-3.41E-04	PRS-CSx
Colorectum	PRS7	rs7855135	9	114653439	T	C	3.48E-04	PRS-CSx
Colorectum	PRS7	rs7856117	9	18973795	A	C	-3.66E-04	PRS-CSx
Colorectum	PRS7	rs7856322	9	9831142	T	G	1.06E-04	PRS-CSx
Colorectum	PRS7	rs7858424	9	105502660	T	C	4.22E-04	PRS-CSx
Colorectum	PRS7	rs785847	9	272325	C	T	-2.76E-04	PRS-CSx
Colorectum	PRS7	rs7861925	9	101785598	T	G	-1.93E-04	PRS-CSx
Colorectum	PRS7	rs7863079	9	33666009	T	C	-6.23E-05	PRS-CSx
Colorectum	PRS7	rs7863306	9	109557247	C	T	-4.27E-04	PRS-CSx
Colorectum	PRS7	rs7863624	9	89836173	A	G	8.44E-04	PRS-CSx
Colorectum	PRS7	rs7863680	9	20548238	C	T	2.73E-04	PRS-CSx
Colorectum	PRS7	rs7863987	9	79945104	C	T	1.23E-03	PRS-CSx
Colorectum	PRS7	rs7864059	9	12893356	A	G	-7.21E-04	PRS-CSx
Colorectum	PRS7	rs7864581	9	101619403	A	G	2.46E-04	PRS-CSx
Colorectum	PRS7	rs7865421	9	648961	T	G	1.88E-04	PRS-CSx
Colorectum	PRS7	rs7865618	9	22031005	G	A	5.29E-04	PRS-CSx
Colorectum	PRS7	rs7866416	9	19340521	G	A	2.99E-04	PRS-CSx
Colorectum	PRS7	rs786911	1	89265248	T	C	-2.10E-04	PRS-CSx
Colorectum	PRS7	rs7873360	9	104584465	A	G	4.06E-03	PRS-CSx
Colorectum	PRS7	rs7873419	9	3202370	A	G	5.34E-05	PRS-CSx
Colorectum	PRS7	rs787346	10	29112699	C	T	5.84E-04	PRS-CSx
Colorectum	PRS7	rs7873471	9	104584538	T	G	3.12E-03	PRS-CSx
Colorectum	PRS7	rs787349	10	29110316	T	C	6.58E-04	PRS-CSx
Colorectum	PRS7	rs7874183	9	101848586	G	A	3.62E-04	PRS-CSx
Colorectum	PRS7	rs7874499	9	118250623	T	C	-5.02E-04	PRS-CSx
Colorectum	PRS7	rs7874819	9	101809368	T	C	1.32E-04	PRS-CSx
Colorectum	PRS7	rs78756	10	120140127	T	C	-1.31E-03	PRS-CSx
Colorectum	PRS7	rs7894031	10	29091690	C	T	3.01E-04	PRS-CSx
Colorectum	PRS7	rs7895307	10	114743961	A	G	-2.04E-04	PRS-CSx
Colorectum	PRS7	rs7895362	10	114277173	A	G	2.86E-03	PRS-CSx
Colorectum	PRS7	rs7897180	10	95521457	G	A	-4.29E-04	PRS-CSx
Colorectum	PRS7	rs7897575	10	95521752	G	A	-4.78E-04	PRS-CSx
Colorectum	PRS7	rs7897783	10	95521692	A	G	-8.68E-04	PRS-CSx
Colorectum	PRS7	rs7897847	10	95521958	G	A	-6.06E-04	PRS-CSx
Colorectum	PRS7	rs7898455	10	8738908	T	G	-6.28E-03	PRS-CSx
Colorectum	PRS7	rs7898770	10	104756636	A	G	-6.46E-05	PRS-CSx
Colorectum	PRS7	rs7901275	10	114732906	A	C	-1.51E-03	PRS-CSx
Colorectum	PRS7	rs7902111	10	118082620	T	C	5.43E-04	PRS-CSx
Colorectum	PRS7	rs7902526	10	8820426	G	A	3.69E-04	PRS-CSx
Colorectum	PRS7	rs7904252	10	104456725	T	G	-1.41E-04	PRS-CSx
Colorectum	PRS7	rs7904396	10	104456838	A	G	-5.73E-05	PRS-CSx
Colorectum	PRS7	rs7904473	10	70075569	C	T	-2.36E-04	PRS-CSx
Colorectum	PRS7	rs7904544	10	63553849	C	A	-1.51E-03	PRS-CSx
Colorectum	PRS7	rs7907503	10	104440050	T	C	-1.73E-04	PRS-CSx

Colorectum	PRS7	rs7908815	10	29088649	C	T	3.73E-04	PRS-CSx
Colorectum	PRS7	rs7910940	10	8666008	T	C	-1.16E-04	PRS-CSx
Colorectum	PRS7	rs7912178	10	106440612	T	C	5.34E-04	PRS-CSx
Colorectum	PRS7	rs7912831	10	8731255	T	C	-4.62E-03	PRS-CSx
Colorectum	PRS7	rs7913889	10	16559381	T	C	9.96E-04	PRS-CSx
Colorectum	PRS7	rs7916911	10	8722944	T	G	3.69E-04	PRS-CSx
Colorectum	PRS7	rs7917102	10	48548926	A	G	2.71E-03	PRS-CSx
Colorectum	PRS7	rs791721	1	212613741	G	A	-2.21E-04	PRS-CSx
Colorectum	PRS7	rs7917343	10	123633118	A	G	1.47E-03	PRS-CSx
Colorectum	PRS7	rs7917983	10	114732882	T	C	-6.39E-04	PRS-CSx
Colorectum	PRS7	rs7918220	10	8694069	C	A	1.82E-04	PRS-CSx
Colorectum	PRS7	rs7918658	10	12471105	C	T	1.10E-03	PRS-CSx
Colorectum	PRS7	rs7918749	10	114736470	G	A	-3.77E-03	PRS-CSx
Colorectum	PRS7	rs791881	10	89408246	T	C	5.07E-04	PRS-CSx
Colorectum	PRS7	rs791889	10	89413057	C	T	-3.13E-04	PRS-CSx
Colorectum	PRS7	rs7919079	10	107501893	T	G	-9.84E-04	PRS-CSx
Colorectum	PRS7	rs7920460	10	80838651	T	C	-3.45E-04	PRS-CSx
Colorectum	PRS7	rs7921734	10	8628416	C	T	5.18E-04	PRS-CSx
Colorectum	PRS7	rs7923068	10	8786826	A	G	1.80E-04	PRS-CSx
Colorectum	PRS7	rs7924341	11	440343	C	A	7.44E-04	PRS-CSx
Colorectum	PRS7	rs7924963	11	120233536	A	G	-1.59E-04	PRS-CSx
Colorectum	PRS7	rs7925523	11	61442492	A	G	1.64E-04	PRS-CSx
Colorectum	PRS7	rs7925983	11	69916269	A	G	7.43E-04	PRS-CSx
Colorectum	PRS7	rs7926352	11	69915881	T	C	5.76E-04	PRS-CSx
Colorectum	PRS7	rs792840	3	99471251	T	C	-1.18E-04	PRS-CSx
Colorectum	PRS7	rs792841	3	99471308	G	T	-8.68E-06	PRS-CSx
Colorectum	PRS7	rs7928487	11	33189195	G	A	-2.18E-04	PRS-CSx
Colorectum	PRS7	rs7928813	11	100318818	A	G	4.80E-04	PRS-CSx
Colorectum	PRS7	rs7929240	11	69916008	T	C	4.42E-04	PRS-CSx
Colorectum	PRS7	rs7929294	11	46776176	C	T	3.75E-04	PRS-CSx
Colorectum	PRS7	rs7930269	11	69916793	T	C	5.82E-04	PRS-CSx
Colorectum	PRS7	rs7931260	11	47088303	C	T	4.68E-04	PRS-CSx
Colorectum	PRS7	rs7932579	11	7023688	A	G	-3.95E-04	PRS-CSx
Colorectum	PRS7	rs7932658	11	120211212	A	G	-1.24E-04	PRS-CSx
Colorectum	PRS7	rs7932922	11	74360030	C	T	8.96E-04	PRS-CSx
Colorectum	PRS7	rs7933086	11	120269308	A	G	-1.52E-04	PRS-CSx
Colorectum	PRS7	rs7934404	11	120193477	A	G	-4.43E-04	PRS-CSx
Colorectum	PRS7	rs7935564	11	5718517	A	G	3.66E-04	PRS-CSx
Colorectum	PRS7	rs7935903	11	5718742	A	G	4.11E-04	PRS-CSx
Colorectum	PRS7	rs7935929	11	5718817	A	G	3.26E-04	PRS-CSx
Colorectum	PRS7	rs7936444	11	15052035	C	A	3.62E-04	PRS-CSx
Colorectum	PRS7	rs7937101	11	47226488	G	A	2.50E-04	PRS-CSx
Colorectum	PRS7	rs7937428	11	44022611	G	A	8.00E-04	PRS-CSx
Colorectum	PRS7	rs7938169	11	46971539	C	T	1.28E-04	PRS-CSx
Colorectum	PRS7	rs7939137	11	120343271	T	C	-2.33E-04	PRS-CSx
Colorectum	PRS7	rs7940240	11	47203068	C	T	8.03E-05	PRS-CSx
Colorectum	PRS7	rs7940320	11	34681761	C	T	-4.54E-04	PRS-CSx
Colorectum	PRS7	rs7940441	11	46939027	A	G	1.38E-04	PRS-CSx
Colorectum	PRS7	rs7940578	11	46812227	C	T	2.47E-04	PRS-CSx
Colorectum	PRS7	rs7940880	11	74317414	A	G	7.49E-04	PRS-CSx
Colorectum	PRS7	rs7941496	11	16041305	T	G	-3.93E-04	PRS-CSx
Colorectum	PRS7	rs7942659	11	16026663	C	T	3.45E-04	PRS-CSx
Colorectum	PRS7	rs7942880	11	16026842	T	C	-5.38E-04	PRS-CSx
Colorectum	PRS7	rs7943807	11	72068077	A	G	1.36E-03	PRS-CSx
Colorectum	PRS7	rs7943866	11	46881954	T	C	2.08E-04	PRS-CSx
Colorectum	PRS7	rs7944514	11	74301192	C	T	-3.82E-04	PRS-CSx

Colorectum	PRS7	rs7945315	11	47110652	C	A	2.58E-04	PRS-CSx
Colorectum	PRS7	rs7945558	11	46957808	A	G	1.99E-04	PRS-CSx
Colorectum	PRS7	rs7946257	11	117053165	G	A	-3.26E-04	PRS-CSx
Colorectum	PRS7	rs7946709	11	47100796	T	G	4.38E-04	PRS-CSx
Colorectum	PRS7	rs7947338	11	117613203	C	T	4.73E-04	PRS-CSx
Colorectum	PRS7	rs7948017	11	15000384	C	A	2.21E-04	PRS-CSx
Colorectum	PRS7	rs7950335	11	27322503	T	C	1.49E-04	PRS-CSx
Colorectum	PRS7	rs7950753	11	47071897	T	C	2.91E-04	PRS-CSx
Colorectum	PRS7	rs7951995	11	33170853	A	G	-3.21E-04	PRS-CSx
Colorectum	PRS7	rs7953189	12	95157454	T	C	-4.52E-04	PRS-CSx
Colorectum	PRS7	rs7953236	12	29008173	C	T	-8.53E-04	PRS-CSx
Colorectum	PRS7	rs7953683	12	79993704	T	C	1.88E-04	PRS-CSx
Colorectum	PRS7	rs7954777	12	4373320	C	T	4.03E-03	PRS-CSx
Colorectum	PRS7	rs7955388	12	80006693	A	G	3.01E-04	PRS-CSx
Colorectum	PRS7	rs7955575	12	122616766	A	G	1.75E-04	PRS-CSx
Colorectum	PRS7	rs7956509	12	115910853	A	G	6.36E-04	PRS-CSx
Colorectum	PRS7	rs7958347	12	113325061	C	T	-3.43E-04	PRS-CSx
Colorectum	PRS7	rs7958413	12	57350194	C	A	1.15E-03	PRS-CSx
Colorectum	PRS7	rs7958890	12	47940721	G	A	6.18E-04	PRS-CSx
Colorectum	PRS7	rs7960725	12	120466168	C	T	2.99E-04	PRS-CSx
Colorectum	PRS7	rs7960825	12	111255027	T	C	5.91E-05	PRS-CSx
Colorectum	PRS7	rs7961085	12	46307741	G	T	-3.67E-04	PRS-CSx
Colorectum	PRS7	rs7961187	12	77673046	C	A	-4.27E-04	PRS-CSx
Colorectum	PRS7	rs7961663	12	111399771	A	G	5.66E-04	PRS-CSx
Colorectum	PRS7	rs7962138	12	112180177	A	G	2.00E-04	PRS-CSx
Colorectum	PRS7	rs7962233	12	111655513	C	T	3.35E-06	PRS-CSx
Colorectum	PRS7	rs7962560	12	43287688	C	T	-4.50E-04	PRS-CSx
Colorectum	PRS7	rs7962812	12	120434393	T	G	1.88E-04	PRS-CSx
Colorectum	PRS7	rs7966149	12	113327018	C	T	-2.88E-04	PRS-CSx
Colorectum	PRS7	rs7966335	12	115928220	T	C	5.80E-04	PRS-CSx
Colorectum	PRS7	rs7966469	12	14289567	C	T	-4.16E-04	PRS-CSx
Colorectum	PRS7	rs7968442	12	80025071	A	G	1.34E-04	PRS-CSx
Colorectum	PRS7	rs7968934	12	71153213	T	C	-9.03E-04	PRS-CSx
Colorectum	PRS7	rs7969686	12	111665388	G	A	6.90E-05	PRS-CSx
Colorectum	PRS7	rs7969760	12	79960432	A	C	3.55E-04	PRS-CSx
Colorectum	PRS7	rs7971185	12	111660599	G	T	3.77E-05	PRS-CSx
Colorectum	PRS7	rs7971598	12	115938102	G	T	2.43E-04	PRS-CSx
Colorectum	PRS7	rs7971769	12	79964192	C	A	2.51E-04	PRS-CSx
Colorectum	PRS7	rs7972649	12	3953918	C	T	1.92E-03	PRS-CSx
Colorectum	PRS7	rs7973329	12	46381206	C	T	-2.62E-04	PRS-CSx
Colorectum	PRS7	rs7974633	12	64416284	A	C	-5.51E-04	PRS-CSx
Colorectum	PRS7	rs7974919	12	116174409	T	C	3.32E-04	PRS-CSx
Colorectum	PRS7	rs7975871	12	46432227	T	C	-1.04E-04	PRS-CSx
Colorectum	PRS7	rs7975952	12	107634068	G	T	-3.05E-04	PRS-CSx
Colorectum	PRS7	rs7977210	12	131569989	A	G	6.82E-04	PRS-CSx
Colorectum	PRS7	rs7977281	12	120412925	C	T	3.93E-04	PRS-CSx
Colorectum	PRS7	rs7977670	12	25244383	G	A	-5.59E-04	PRS-CSx
Colorectum	PRS7	rs7977672	12	43138045	T	C	-9.80E-04	PRS-CSx
Colorectum	PRS7	rs797770	12	6303157	C	T	2.00E-03	PRS-CSx
Colorectum	PRS7	rs7977828	12	112094766	A	G	2.24E-04	PRS-CSx
Colorectum	PRS7	rs7978821	12	111400116	G	A	3.37E-04	PRS-CSx
Colorectum	PRS7	rs7979656	12	111376637	T	C	2.81E-04	PRS-CSx
Colorectum	PRS7	rs7981910	13	94014625	C	T	-8.24E-04	PRS-CSx
Colorectum	PRS7	rs7982810	13	47258281	G	A	-2.80E-04	PRS-CSx
Colorectum	PRS7	rs7982828	13	28758037	A	G	-4.41E-04	PRS-CSx
Colorectum	PRS7	rs7982847	13	73533589	C	A	1.96E-04	PRS-CSx

Colorectum	PRS7	rs7983374	13	111050321	C	T	-1.19E-03	PRS-CSx
Colorectum	PRS7	rs7983493	13	73313825	T	G	7.17E-05	PRS-CSx
Colorectum	PRS7	rs798385	2	7873846	G	A	-7.85E-04	PRS-CSx
Colorectum	PRS7	rs798544	7	2763102	T	C	-1.70E-03	PRS-CSx
Colorectum	PRS7	rs798548	7	2760935	C	T	-1.56E-03	PRS-CSx
Colorectum	PRS7	rs798554	7	2759795	T	C	-1.31E-03	PRS-CSx
Colorectum	PRS7	rs7986027	13	92456936	T	G	3.84E-04	PRS-CSx
Colorectum	PRS7	rs7986048	13	74050153	G	A	5.60E-04	PRS-CSx
Colorectum	PRS7	rs7988544	13	47258560	T	C	-3.27E-04	PRS-CSx
Colorectum	PRS7	rs7989425	13	73980468	T	G	-1.47E-03	PRS-CSx
Colorectum	PRS7	rs7991227	13	57708749	T	C	3.41E-04	PRS-CSx
Colorectum	PRS7	rs7991801	13	71600617	C	A	5.46E-04	PRS-CSx
Colorectum	PRS7	rs7992043	13	80600086	G	A	-1.32E-03	PRS-CSx
Colorectum	PRS7	rs7992302	13	73972011	A	C	-8.64E-04	PRS-CSx
Colorectum	PRS7	rs7993044	13	34203051	T	C	-2.76E-04	PRS-CSx
Colorectum	PRS7	rs7993968	13	51153732	T	C	-2.91E-03	PRS-CSx
Colorectum	PRS7	rs7994277	13	93981755	C	T	5.51E-04	PRS-CSx
Colorectum	PRS7	rs7995153	13	73610059	T	C	6.90E-04	PRS-CSx
Colorectum	PRS7	rs7997308	13	108808267	G	A	1.28E-03	PRS-CSx
Colorectum	PRS7	rs7998775	13	78522427	T	C	-2.55E-03	PRS-CSx
Colorectum	PRS7	rs7999869	13	34262060	T	C	5.66E-04	PRS-CSx
Colorectum	PRS7	rs8003636	14	59381328	A	G	1.00E-03	PRS-CSx
Colorectum	PRS7	rs8003773	14	69193327	A	C	8.12E-04	PRS-CSx
Colorectum	PRS7	rs8004217	14	54533857	G	A	8.49E-04	PRS-CSx
Colorectum	PRS7	rs8004768	14	51375797	T	G	-1.80E-04	PRS-CSx
Colorectum	PRS7	rs8004788	14	51375826	T	C	-5.80E-04	PRS-CSx
Colorectum	PRS7	rs8005169	14	54519386	T	C	7.45E-04	PRS-CSx
Colorectum	PRS7	rs8006358	14	92705919	G	A	6.22E-04	PRS-CSx
Colorectum	PRS7	rs8006455	14	51400160	G	A	-4.27E-04	PRS-CSx
Colorectum	PRS7	rs8006497	14	73629732	G	A	3.04E-04	PRS-CSx
Colorectum	PRS7	rs8007059	14	96592891	C	T	-1.42E-03	PRS-CSx
Colorectum	PRS7	rs8007693	14	80561521	T	C	2.73E-04	PRS-CSx
Colorectum	PRS7	rs8008129	14	58829032	T	C	1.10E-04	PRS-CSx
Colorectum	PRS7	rs8009527	14	58752112	A	G	2.79E-04	PRS-CSx
Colorectum	PRS7	rs8009579	14	58713559	A	G	2.32E-04	PRS-CSx
Colorectum	PRS7	rs8010333	14	54358685	C	T	7.08E-04	PRS-CSx
Colorectum	PRS7	rs8011191	14	58717013	T	G	2.35E-04	PRS-CSx
Colorectum	PRS7	rs8011432	14	57659190	C	T	6.71E-04	PRS-CSx
Colorectum	PRS7	rs8012185	14	58668484	A	G	1.64E-04	PRS-CSx
Colorectum	PRS7	rs8013113	14	89897342	C	T	-7.46E-04	PRS-CSx
Colorectum	PRS7	rs8013464	14	58513874	T	C	1.58E-03	PRS-CSx
Colorectum	PRS7	rs8014315	14	26297043	T	C	3.57E-04	PRS-CSx
Colorectum	PRS7	rs801458	5	139953189	C	T	1.47E-04	PRS-CSx
Colorectum	PRS7	rs801460	5	139931930	C	T	2.03E-04	PRS-CSx
Colorectum	PRS7	rs8014637	14	54012258	A	G	-3.37E-04	PRS-CSx
Colorectum	PRS7	rs8015423	14	81719357	T	C	-1.70E-04	PRS-CSx
Colorectum	PRS7	rs8017449	14	57308489	A	C	-1.31E-03	PRS-CSx
Colorectum	PRS7	rs8017797	14	92707795	A	C	6.78E-04	PRS-CSx
Colorectum	PRS7	rs8017931	14	63210951	C	T	4.44E-04	PRS-CSx
Colorectum	PRS7	rs8019025	14	64779438	G	A	-3.92E-05	PRS-CSx
Colorectum	PRS7	rs8020062	14	105538299	T	C	-4.48E-04	PRS-CSx
Colorectum	PRS7	rs8020593	14	79976574	T	C	1.04E-03	PRS-CSx
Colorectum	PRS7	rs8021235	14	54689824	C	A	1.15E-03	PRS-CSx
Colorectum	PRS7	rs8022524	14	63211350	C	T	2.47E-04	PRS-CSx
Colorectum	PRS7	rs8022640	14	77524587	A	G	-5.54E-04	PRS-CSx
Colorectum	PRS7	rs8022952	14	51394480	C	T	-7.14E-04	PRS-CSx

Colorectum	PRS7	rs8023239	14	80525079	T	C	2.97E-04	PRS-CSx
Colorectum	PRS7	rs8023664	15	31762474	C	T	3.69E-04	PRS-CSx
Colorectum	PRS7	rs8023776	15	90897952	T	C	4.21E-04	PRS-CSx
Colorectum	PRS7	rs8024	1	201845575	A	C	5.18E-04	PRS-CSx
Colorectum	PRS7	rs8025898	15	66812208	C	T	-2.35E-04	PRS-CSx
Colorectum	PRS7	rs8027432	15	66662696	T	C	-9.67E-05	PRS-CSx
Colorectum	PRS7	rs8028	1	201865763	A	G	5.08E-04	PRS-CSx
Colorectum	PRS7	rs8028182	15	75718669	T	G	5.47E-05	PRS-CSx
Colorectum	PRS7	rs8028277	15	75684085	G	A	4.96E-05	PRS-CSx
Colorectum	PRS7	rs8029112	15	75808972	C	T	2.96E-04	PRS-CSx
Colorectum	PRS7	rs8029119	15	66592757	G	A	-2.19E-04	PRS-CSx
Colorectum	PRS7	rs8031201	15	71676097	G	A	5.06E-04	PRS-CSx
Colorectum	PRS7	rs8032020	15	71575617	C	T	4.13E-04	PRS-CSx
Colorectum	PRS7	rs8032307	15	48692609	T	C	1.82E-04	PRS-CSx
Colorectum	PRS7	rs8032308	15	48692612	T	G	4.75E-05	PRS-CSx
Colorectum	PRS7	rs8033791	15	71995256	G	T	-3.65E-05	PRS-CSx
Colorectum	PRS7	rs8033972	15	64524980	A	G	-1.05E-03	PRS-CSx
Colorectum	PRS7	rs8034317	15	75764343	A	G	7.93E-05	PRS-CSx
Colorectum	PRS7	rs8034473	15	49829640	A	G	2.11E-04	PRS-CSx
Colorectum	PRS7	rs8035039	15	79129823	A	G	-2.45E-03	PRS-CSx
Colorectum	PRS7	rs8035055	15	96126169	C	A	-4.49E-04	PRS-CSx
Colorectum	PRS7	rs8035321	15	82057290	G	A	-5.69E-04	PRS-CSx
Colorectum	PRS7	rs8035425	15	38558974	G	A	-7.60E-04	PRS-CSx
Colorectum	PRS7	rs8037685	15	37778364	A	G	-6.20E-04	PRS-CSx
Colorectum	PRS7	rs803780	13	71629820	A	G	5.50E-04	PRS-CSx
Colorectum	PRS7	rs803804	13	71599302	C	T	5.81E-04	PRS-CSx
Colorectum	PRS7	rs8038667	15	31753806	T	C	3.03E-04	PRS-CSx
Colorectum	PRS7	rs8038760	15	75742095	C	A	3.83E-04	PRS-CSx
Colorectum	PRS7	rs8040106	15	82182835	C	T	-8.45E-04	PRS-CSx
Colorectum	PRS7	rs8041582	15	102005851	G	A	4.87E-04	PRS-CSx
Colorectum	PRS7	rs8043322	15	82084934	C	A	-2.28E-04	PRS-CSx
Colorectum	PRS7	rs8043542	16	76458238	G	A	3.95E-04	PRS-CSx
Colorectum	PRS7	rs8044524	16	81603771	G	A	-9.69E-04	PRS-CSx
Colorectum	PRS7	rs8045365	16	83262098	G	A	3.32E-03	PRS-CSx
Colorectum	PRS7	rs8046125	16	60822117	C	A	-4.95E-04	PRS-CSx
Colorectum	PRS7	rs8046148	16	50142944	A	G	-3.05E-04	PRS-CSx
Colorectum	PRS7	rs8046518	16	80222886	C	T	4.55E-04	PRS-CSx
Colorectum	PRS7	rs8046794	16	81429819	C	T	-1.23E-03	PRS-CSx
Colorectum	PRS7	rs8047060	16	6816914	T	G	1.14E-03	PRS-CSx
Colorectum	PRS7	rs8047464	16	1856440	C	T	4.42E-04	PRS-CSx
Colorectum	PRS7	rs8047742	16	50117316	G	A	-5.22E-04	PRS-CSx
Colorectum	PRS7	rs8048108	16	50094615	G	T	-3.90E-04	PRS-CSx
Colorectum	PRS7	rs8048393	16	86293962	G	T	-1.11E-03	PRS-CSx
Colorectum	PRS7	rs8050142	16	80093914	A	G	8.58E-05	PRS-CSx
Colorectum	PRS7	rs8050447	16	1681769	C	T	1.44E-04	PRS-CSx
Colorectum	PRS7	rs8051216	16	50113773	C	T	-3.81E-04	PRS-CSx
Colorectum	PRS7	rs8051754	16	79997333	G	A	3.54E-04	PRS-CSx
Colorectum	PRS7	rs8051902	16	50099777	C	T	-3.69E-04	PRS-CSx
Colorectum	PRS7	rs8052110	16	78367814	C	T	-6.59E-04	PRS-CSx
Colorectum	PRS7	rs8052492	16	50099747	G	A	-5.20E-04	PRS-CSx
Colorectum	PRS7	rs8056538	16	68802282	A	G	-5.90E-04	PRS-CSx
Colorectum	PRS7	rs8056862	16	86169419	T	C	1.13E-03	PRS-CSx
Colorectum	PRS7	rs8057084	16	75492706	T	G	6.11E-04	PRS-CSx
Colorectum	PRS7	rs8057091	16	6902148	T	C	-3.89E-04	PRS-CSx
Colorectum	PRS7	rs8057223	16	60815019	A	G	-5.89E-04	PRS-CSx
Colorectum	PRS7	rs8057314	16	8313272	T	C	1.35E-03	PRS-CSx

Colorectum	PRS7	rs8057476	16	80054987	C	A	-3.87E-04	PRS-CSx
Colorectum	PRS7	rs8059194	16	68749663	A	G	4.56E-04	PRS-CSx
Colorectum	PRS7	rs8059251	16	80100312	T	C	1.49E-04	PRS-CSx
Colorectum	PRS7	rs8060329	16	6908864	G	A	-4.53E-04	PRS-CSx
Colorectum	PRS7	rs8060693	16	88757839	T	C	-3.14E-04	PRS-CSx
Colorectum	PRS7	rs8060790	16	68729112	G	A	3.82E-04	PRS-CSx
Colorectum	PRS7	rs8060979	16	74807492	T	C	-6.38E-04	PRS-CSx
Colorectum	PRS7	rs8063712	16	80042806	C	T	-1.07E-03	PRS-CSx
Colorectum	PRS7	rs8063973	16	84460926	A	G	1.85E-04	PRS-CSx
Colorectum	PRS7	rs8065058	17	57303589	C	T	3.21E-04	PRS-CSx
Colorectum	PRS7	rs8065832	17	17122327	G	A	2.07E-04	PRS-CSx
Colorectum	PRS7	rs8067605	17	69587393	T	C	3.24E-04	PRS-CSx
Colorectum	PRS7	rs8068400	17	69985716	G	A	-1.16E-03	PRS-CSx
Colorectum	PRS7	rs8069566	17	811142	T	C	1.80E-03	PRS-CSx
Colorectum	PRS7	rs8069645	17	40494902	G	A	3.40E-04	PRS-CSx
Colorectum	PRS7	rs8072402	17	17185267	C	T	2.71E-04	PRS-CSx
Colorectum	PRS7	rs8072869	17	17194050	C	T	2.90E-04	PRS-CSx
Colorectum	PRS7	rs8077205	17	75673150	C	T	9.48E-04	PRS-CSx
Colorectum	PRS7	rs8077585	17	70066312	A	G	-4.35E-04	PRS-CSx
Colorectum	PRS7	rs8077610	17	12814973	T	C	6.62E-04	PRS-CSx
Colorectum	PRS7	rs8078684	17	10708811	C	T	-1.25E-03	PRS-CSx
Colorectum	PRS7	rs8078776	17	4778487	A	G	6.43E-04	PRS-CSx
Colorectum	PRS7	rs8079173	17	70075796	A	G	-5.76E-04	PRS-CSx
Colorectum	PRS7	rs8079270	17	43414434	T	C	-2.90E-05	PRS-CSx
Colorectum	PRS7	rs8080345	17	69594032	G	T	4.66E-04	PRS-CSx
Colorectum	PRS7	rs8080379	17	21080955	T	C	-4.01E-04	PRS-CSx
Colorectum	PRS7	rs8080610	17	69607546	C	A	2.86E-04	PRS-CSx
Colorectum	PRS7	rs8080886	17	36737020	G	A	-7.21E-04	PRS-CSx
Colorectum	PRS7	rs8081823	17	8965551	A	G	-2.89E-03	PRS-CSx
Colorectum	PRS7	rs8083820	18	42098942	T	G	2.61E-04	PRS-CSx
Colorectum	PRS7	rs8084507	18	42086982	G	A	1.81E-04	PRS-CSx
Colorectum	PRS7	rs8085051	18	9456081	G	A	-6.51E-04	PRS-CSx
Colorectum	PRS7	rs8086342	18	31603023	G	T	-4.87E-04	PRS-CSx
Colorectum	PRS7	rs8087876	18	42015661	T	G	1.37E-04	PRS-CSx
Colorectum	PRS7	rs8088250	18	53977917	C	T	-7.08E-05	PRS-CSx
Colorectum	PRS7	rs8089281	18	73110461	T	C	2.10E-03	PRS-CSx
Colorectum	PRS7	rs808950	3	113015553	A	C	-2.49E-04	PRS-CSx
Colorectum	PRS7	rs808951	3	113002150	T	C	-1.75E-03	PRS-CSx
Colorectum	PRS7	rs8089797	18	2475505	C	T	1.81E-03	PRS-CSx
Colorectum	PRS7	rs8090058	18	33769406	C	A	2.75E-04	PRS-CSx
Colorectum	PRS7	rs8090218	18	73229095	T	C	8.10E-04	PRS-CSx
Colorectum	PRS7	rs8091305	18	12870870	T	C	-1.08E-03	PRS-CSx
Colorectum	PRS7	rs809213	3	129606827	G	A	3.04E-04	PRS-CSx
Colorectum	PRS7	rs8092311	18	6764297	G	A	-5.93E-04	PRS-CSx
Colorectum	PRS7	rs8092661	18	9034182	C	T	-1.14E-03	PRS-CSx
Colorectum	PRS7	rs8092715	18	36443680	T	C	4.23E-04	PRS-CSx
Colorectum	PRS7	rs8092870	18	25562831	T	C	-8.77E-04	PRS-CSx
Colorectum	PRS7	rs8095250	18	70343719	T	C	3.84E-04	PRS-CSx
Colorectum	PRS7	rs8095718	18	33779298	T	C	5.25E-04	PRS-CSx
Colorectum	PRS7	rs8097398	18	66508709	A	G	4.35E-05	PRS-CSx
Colorectum	PRS7	rs8097433	18	34110763	T	C	-3.36E-04	PRS-CSx
Colorectum	PRS7	rs809763	3	129587819	G	A	3.34E-04	PRS-CSx
Colorectum	PRS7	rs8097811	18	34098590	G	A	2.42E-04	PRS-CSx
Colorectum	PRS7	rs8097893	18	74983055	G	A	2.93E-03	PRS-CSx
Colorectum	PRS7	rs8098856	18	40625666	A	C	-3.63E-04	PRS-CSx
Colorectum	PRS7	rs810209	3	113012797	G	A	-9.46E-05	PRS-CSx

Colorectum	PRS7	rs8103698	19	56601298	A	G	8.24E-04	PRS-CSx
Colorectum	PRS7	rs8103890	19	22690374	G	A	-8.04E-04	PRS-CSx
Colorectum	PRS7	rs8104608	19	10370133	A	G	1.11E-03	PRS-CSx
Colorectum	PRS7	rs8106312	19	33447798	T	C	3.14E-04	PRS-CSx
Colorectum	PRS7	rs8106333	19	509569	C	T	1.59E-03	PRS-CSx
Colorectum	PRS7	rs8108650	19	37985422	C	T	-4.99E-04	PRS-CSx
Colorectum	PRS7	rs8108865	19	17872069	T	C	-6.02E-04	PRS-CSx
Colorectum	PRS7	rs8109798	19	33469577	G	T	8.48E-04	PRS-CSx
Colorectum	PRS7	rs8109806	19	37923959	A	C	8.74E-04	PRS-CSx
Colorectum	PRS7	rs8110419	19	14613383	A	G	8.96E-04	PRS-CSx
Colorectum	PRS7	rs8110630	19	34609682	G	A	-4.56E-04	PRS-CSx
Colorectum	PRS7	rs811175	14	54597827	C	A	4.52E-04	PRS-CSx
Colorectum	PRS7	rs8112826	19	42262169	A	G	-2.19E-03	PRS-CSx
Colorectum	PRS7	rs8113160	19	6288357	G	A	9.28E-04	PRS-CSx
Colorectum	PRS7	rs8114358	20	7720594	A	G	1.03E-03	PRS-CSx
Colorectum	PRS7	rs8115156	20	56019846	A	G	-1.47E-03	PRS-CSx
Colorectum	PRS7	rs8117366	20	52364760	G	T	2.36E-03	PRS-CSx
Colorectum	PRS7	rs8121909	20	7711820	C	T	9.10E-04	PRS-CSx
Colorectum	PRS7	rs8123670	20	22460864	C	A	9.55E-04	PRS-CSx
Colorectum	PRS7	rs8123727	20	1972761	A	G	-3.24E-04	PRS-CSx
Colorectum	PRS7	rs8124724	20	6403521	C	T	1.68E-03	PRS-CSx
Colorectum	PRS7	rs8130107	21	40425579	C	T	3.65E-04	PRS-CSx
Colorectum	PRS7	rs8130759	21	16074524	T	C	-2.60E-04	PRS-CSx
Colorectum	PRS7	rs8130926	21	33333331	C	T	1.21E-03	PRS-CSx
Colorectum	PRS7	rs8132320	21	47886796	A	G	3.82E-04	PRS-CSx
Colorectum	PRS7	rs8132865	21	43354828	C	T	1.18E-03	PRS-CSx
Colorectum	PRS7	rs8132871	21	42810820	A	G	6.32E-04	PRS-CSx
Colorectum	PRS7	rs8136533	22	17700284	C	T	8.27E-04	PRS-CSx
Colorectum	PRS7	rs8137254	22	29429997	C	T	-9.53E-04	PRS-CSx
Colorectum	PRS7	rs8138001	22	45785840	A	G	-3.73E-04	PRS-CSx
Colorectum	PRS7	rs8138930	22	43336728	C	T	-1.11E-05	PRS-CSx
Colorectum	PRS7	rs8140112	22	40533130	G	A	-7.65E-04	PRS-CSx
Colorectum	PRS7	rs8142159	22	43490634	G	A	1.49E-04	PRS-CSx
Colorectum	PRS7	rs815710	3	61262149	A	G	3.58E-05	PRS-CSx
Colorectum	PRS7	rs816953	20	62688359	T	C	-5.36E-04	PRS-CSx
Colorectum	PRS7	rs8176875	12	80014188	G	A	3.80E-04	PRS-CSx
Colorectum	PRS7	rs8181047	9	22064465	A	G	-2.59E-04	PRS-CSx
Colorectum	PRS7	rs8181651	12	50601155	C	T	2.07E-04	PRS-CSx
Colorectum	PRS7	rs8182466	19	6285142	G	A	1.04E-03	PRS-CSx
Colorectum	PRS7	rs8182608	19	6284663	G	A	1.05E-03	PRS-CSx
Colorectum	PRS7	rs818702	9	116144973	G	A	-1.25E-03	PRS-CSx
Colorectum	PRS7	rs818706	9	116148630	C	T	-1.46E-03	PRS-CSx
Colorectum	PRS7	rs8191303	16	11767609	T	C	-4.49E-04	PRS-CSx
Colorectum	PRS7	rs8192661	10	118031647	T	C	8.43E-04	PRS-CSx
Colorectum	PRS7	rs8192663	10	117971244	C	T	3.13E-04	PRS-CSx
Colorectum	PRS7	rs8193039	6	52052018	A	G	-2.17E-03	PRS-CSx
Colorectum	PRS7	rs819368	7	17109926	C	T	-1.63E-04	PRS-CSx
Colorectum	PRS7	rs821857	12	101288316	T	C	-4.93E-04	PRS-CSx
Colorectum	PRS7	rs822581	1	156976225	C	T	-4.36E-04	PRS-CSx
Colorectum	PRS7	rs823161	1	231775017	G	A	-3.49E-04	PRS-CSx
Colorectum	PRS7	rs823163	1	231771108	A	G	-3.10E-04	PRS-CSx
Colorectum	PRS7	rs825828	16	73545639	T	C	-1.36E-03	PRS-CSx
Colorectum	PRS7	rs826727	8	59736400	C	T	7.58E-04	PRS-CSx
Colorectum	PRS7	rs827388	10	8706433	A	G	3.69E-04	PRS-CSx
Colorectum	PRS7	rs827392	10	8700934	A	G	2.19E-04	PRS-CSx
Colorectum	PRS7	rs827396	10	8693691	C	T	1.69E-04	PRS-CSx

Colorectum	PRS7	rs827397	10	8693924	T	C	3.81E-04	PRS-CSx
Colorectum	PRS7	rs827401	10	8698830	A	G	-2.20E-03	PRS-CSx
Colorectum	PRS7	rs827405	10	8689003	T	C	2.22E-04	PRS-CSx
Colorectum	PRS7	rs827988	6	158797563	T	G	2.96E-04	PRS-CSx
Colorectum	PRS7	rs830085	11	47251199	A	G	2.60E-05	PRS-CSx
Colorectum	PRS7	rs831229	5	67553016	A	G	1.03E-03	PRS-CSx
Colorectum	PRS7	rs831413	5	143721209	G	A	-2.51E-04	PRS-CSx
Colorectum	PRS7	rs831414	5	143721025	C	T	-2.64E-04	PRS-CSx
Colorectum	PRS7	rs831419	5	143718288	G	A	-1.23E-04	PRS-CSx
Colorectum	PRS7	rs833140	2	183148826	C	T	1.78E-04	PRS-CSx
Colorectum	PRS7	rs834027	9	33988235	T	C	-7.86E-05	PRS-CSx
Colorectum	PRS7	rs835044	12	95397552	T	C	-6.16E-04	PRS-CSx
Colorectum	PRS7	rs835425	1	59860544	A	G	6.33E-04	PRS-CSx
Colorectum	PRS7	rs835431	1	59856404	A	C	5.32E-04	PRS-CSx
Colorectum	PRS7	rs837101	5	40773699	T	C	-2.41E-05	PRS-CSx
Colorectum	PRS7	rs841082	2	129284887	T	C	-1.30E-03	PRS-CSx
Colorectum	PRS7	rs841086	2	129287673	A	G	-6.10E-04	PRS-CSx
Colorectum	PRS7	rs841095	2	129278276	A	G	-9.43E-04	PRS-CSx
Colorectum	PRS7	rs842062	2	159844304	A	G	-2.64E-04	PRS-CSx
Colorectum	PRS7	rs842064	2	159840743	G	T	-5.55E-04	PRS-CSx
Colorectum	PRS7	rs842067	2	159836227	G	T	-3.16E-04	PRS-CSx
Colorectum	PRS7	rs842071	2	159827928	C	T	-4.25E-04	PRS-CSx
Colorectum	PRS7	rs842072	2	159825637	T	G	-3.58E-04	PRS-CSx
Colorectum	PRS7	rs842075	2	159823927	G	A	-4.54E-04	PRS-CSx
Colorectum	PRS7	rs847208	16	86254051	C	A	-4.65E-03	PRS-CSx
Colorectum	PRS7	rs847895	12	112118576	C	T	3.06E-04	PRS-CSx
Colorectum	PRS7	rs847898	12	112163646	T	C	2.67E-04	PRS-CSx
Colorectum	PRS7	rs851332	2	19403510	A	C	3.41E-04	PRS-CSx
Colorectum	PRS7	rs851348	2	19389299	A	G	2.72E-04	PRS-CSx
Colorectum	PRS7	rs851356	2	19474112	C	T	2.48E-04	PRS-CSx
Colorectum	PRS7	rs851386	2	19456196	T	C	2.35E-04	PRS-CSx
Colorectum	PRS7	rs851387	2	19455852	C	T	5.55E-04	PRS-CSx
Colorectum	PRS7	rs851393	2	19451765	G	A	5.59E-04	PRS-CSx
Colorectum	PRS7	rs851420	2	19431168	A	G	2.80E-04	PRS-CSx
Colorectum	PRS7	rs851436	2	19483101	A	C	5.57E-04	PRS-CSx
Colorectum	PRS7	rs851821	7	147242624	G	A	5.52E-04	PRS-CSx
Colorectum	PRS7	rs851823	7	147240610	A	G	2.83E-04	PRS-CSx
Colorectum	PRS7	rs8521	11	117067699	T	C	-3.79E-04	PRS-CSx
Colorectum	PRS7	rs852560	5	10937683	G	A	-1.68E-04	PRS-CSx
Colorectum	PRS7	rs852568	5	10944658	A	G	1.83E-05	PRS-CSx
Colorectum	PRS7	rs852619	5	11011206	T	C	9.37E-05	PRS-CSx
Colorectum	PRS7	rs852621	5	11012130	A	G	9.71E-05	PRS-CSx
Colorectum	PRS7	rs853388	6	14190706	A	G	-1.08E-03	PRS-CSx
Colorectum	PRS7	rs853676	6	28299687	T	C	-3.81E-06	PRS-CSx
Colorectum	PRS7	rs853679	6	28296863	A	C	-1.48E-04	PRS-CSx
Colorectum	PRS7	rs853681	6	28296650	A	C	-2.29E-04	PRS-CSx
Colorectum	PRS7	rs853685	6	28288785	T	C	-1.61E-04	PRS-CSx
Colorectum	PRS7	rs85425	14	59384953	T	C	5.65E-04	PRS-CSx
Colorectum	PRS7	rs856302	19	38259082	T	C	-2.37E-04	PRS-CSx
Colorectum	PRS7	rs856902	11	34704717	G	A	-6.98E-04	PRS-CSx
Colorectum	PRS7	rs858738	6	85523135	T	G	6.55E-04	PRS-CSx
Colorectum	PRS7	rs858985	6	27178028	T	C	-6.33E-04	PRS-CSx
Colorectum	PRS7	rs860434	5	10954186	C	T	4.62E-05	PRS-CSx
Colorectum	PRS7	rs862026	14	74998875	G	A	-7.70E-04	PRS-CSx
Colorectum	PRS7	rs863818	5	67554023	G	A	4.59E-04	PRS-CSx
Colorectum	PRS7	rs8668	20	60964064	G	A	-2.70E-03	PRS-CSx

Colorectum	PRS7	rs868450	12	116179267	G	A	6.34E-04	PRS-CSx
Colorectum	PRS7	rs868452	12	116179325	G	A	8.77E-04	PRS-CSx
Colorectum	PRS7	rs868596	16	84091288	A	G	-7.16E-04	PRS-CSx
Colorectum	PRS7	rs868688	1	3300807	C	T	7.48E-04	PRS-CSx
Colorectum	PRS7	rs868689	1	3301130	T	G	7.68E-04	PRS-CSx
Colorectum	PRS7	rs868933	2	20370926	T	G	-8.83E-04	PRS-CSx
Colorectum	PRS7	rs869724	1	156968568	T	C	-4.66E-04	PRS-CSx
Colorectum	PRS7	rs869776	9	101810339	T	C	4.66E-05	PRS-CSx
Colorectum	PRS7	rs8699	12	96051883	A	G	-6.97E-04	PRS-CSx
Colorectum	PRS7	rs870114	12	43151303	T	C	-1.45E-03	PRS-CSx
Colorectum	PRS7	rs870137	21	44590059	T	C	-1.26E-03	PRS-CSx
Colorectum	PRS7	rs870393	2	95982093	C	T	9.62E-06	PRS-CSx
Colorectum	PRS7	rs871118	10	73184175	A	G	5.13E-04	PRS-CSx
Colorectum	PRS7	rs871443	17	73753503	T	C	-1.63E-03	PRS-CSx
Colorectum	PRS7	rs871540	4	155409030	G	A	-2.83E-04	PRS-CSx
Colorectum	PRS7	rs871541	4	155408604	G	A	-1.37E-04	PRS-CSx
Colorectum	PRS7	rs872331	21	44589215	T	C	-1.08E-03	PRS-CSx
Colorectum	PRS7	rs872463	9	139744807	A	G	-2.12E-04	PRS-CSx
Colorectum	PRS7	rs873165	15	66608190	C	T	1.15E-04	PRS-CSx
Colorectum	PRS7	rs873706	13	95862387	T	C	3.83E-04	PRS-CSx
Colorectum	PRS7	rs873860	11	69941786	T	C	8.89E-04	PRS-CSx
Colorectum	PRS7	rs874286	12	111799681	T	C	2.30E-04	PRS-CSx
Colorectum	PRS7	rs8752	4	175412477	T	C	-4.80E-03	PRS-CSx
Colorectum	PRS7	rs875303	6	74999620	C	T	-1.85E-04	PRS-CSx
Colorectum	PRS7	rs875673	13	53323950	G	A	-4.24E-04	PRS-CSx
Colorectum	PRS7	rs876071	11	128168597	C	A	-3.61E-04	PRS-CSx
Colorectum	PRS7	rs876078	12	51029835	T	G	-1.50E-04	PRS-CSx
Colorectum	PRS7	rs876080	12	51030764	A	G	1.24E-04	PRS-CSx
Colorectum	PRS7	rs876312	12	111366750	A	C	8.18E-05	PRS-CSx
Colorectum	PRS7	rs876479	11	118028094	C	T	1.28E-03	PRS-CSx
Colorectum	PRS7	rs876808	13	94044062	C	T	4.59E-04	PRS-CSx
Colorectum	PRS7	rs877525	14	96357596	T	C	-1.58E-04	PRS-CSx
Colorectum	PRS7	rs877639	16	50118245	G	A	-3.46E-04	PRS-CSx
Colorectum	PRS7	rs877790	11	13247660	T	C	8.92E-04	PRS-CSx
Colorectum	PRS7	rs878082	19	41897674	A	G	2.45E-04	PRS-CSx
Colorectum	PRS7	rs878778	1	11735245	C	A	1.62E-03	PRS-CSx
Colorectum	PRS7	rs878825	22	21982249	T	C	1.31E-04	PRS-CSx
Colorectum	PRS7	rs878939	10	101342941	G	A	2.82E-03	PRS-CSx
Colorectum	PRS7	rs878949	1	22227091	T	C	7.98E-04	PRS-CSx
Colorectum	PRS7	rs878971	18	33046674	A	G	-1.20E-05	PRS-CSx
Colorectum	PRS7	rs879486	11	61475233	C	T	1.04E-04	PRS-CSx
Colorectum	PRS7	rs880026	3	41203895	A	G	-1.83E-04	PRS-CSx
Colorectum	PRS7	rs880603	3	41379517	C	T	-3.69E-04	PRS-CSx
Colorectum	PRS7	rs881805	12	95201831	T	C	-5.27E-04	PRS-CSx
Colorectum	PRS7	rs881807	12	95202145	A	G	-5.17E-04	PRS-CSx
Colorectum	PRS7	rs881835	18	3449310	G	A	7.82E-03	PRS-CSx
Colorectum	PRS7	rs882413	14	51495398	G	A	8.66E-04	PRS-CSx
Colorectum	PRS7	rs883724	11	61457857	T	C	1.69E-04	PRS-CSx
Colorectum	PRS7	rs884437	2	28570398	A	G	1.09E-04	PRS-CSx
Colorectum	PRS7	rs884438	2	28570438	A	C	9.55E-05	PRS-CSx
Colorectum	PRS7	rs884940	1	2223866	T	C	8.77E-04	PRS-CSx
Colorectum	PRS7	rs885156	4	140560388	A	C	-1.10E-03	PRS-CSx
Colorectum	PRS7	rs885640	2	160075183	G	A	-5.84E-04	PRS-CSx
Colorectum	PRS7	rs886001	7	126837463	A	G	3.20E-04	PRS-CSx
Colorectum	PRS7	rs886125	12	111365324	G	A	3.97E-05	PRS-CSx
Colorectum	PRS7	rs886126	12	111679214	C	T	6.60E-04	PRS-CSx

Colorectum	PRS7	rs886476	12	113319471	G	A	-3.06E-04	PRS-CSx
Colorectum	PRS7	rs886479	12	113319105	A	C	-3.14E-04	PRS-CSx
Colorectum	PRS7	rs887344	5	149549022	C	T	-1.85E-03	PRS-CSx
Colorectum	PRS7	rs888208	10	101295863	G	A	3.67E-04	PRS-CSx
Colorectum	PRS7	rs888647	16	87682804	T	C	8.29E-04	PRS-CSx
Colorectum	PRS7	rs888926	5	179738101	A	G	-1.04E-03	PRS-CSx
Colorectum	PRS7	rs889548	16	31137712	C	T	3.04E-04	PRS-CSx
Colorectum	PRS7	rs889851	2	95980340	A	G	-7.31E-05	PRS-CSx
Colorectum	PRS7	rs889852	2	95977790	T	C	-2.00E-04	PRS-CSx
Colorectum	PRS7	rs890058	2	131868107	C	T	-1.58E-04	PRS-CSx
Colorectum	PRS7	rs890898	16	12674329	G	T	6.76E-05	PRS-CSx
Colorectum	PRS7	rs891428	2	109935914	C	T	-8.56E-05	PRS-CSx
Colorectum	PRS7	rs891434	4	39070630	T	C	1.64E-05	PRS-CSx
Colorectum	PRS7	rs891506	7	150742700	A	C	1.22E-04	PRS-CSx
Colorectum	PRS7	rs891509	7	150808109	G	A	7.16E-04	PRS-CSx
Colorectum	PRS7	rs892024	19	33399344	A	G	-2.41E-04	PRS-CSx
Colorectum	PRS7	rs892162	19	4607587	A	G	1.36E-03	PRS-CSx
Colorectum	PRS7	rs892188	19	10409793	T	C	-8.69E-04	PRS-CSx
Colorectum	PRS7	rs892497	12	95191771	T	G	-8.78E-04	PRS-CSx
Colorectum	PRS7	rs892559	5	121705323	T	C	1.57E-03	PRS-CSx
Colorectum	PRS7	rs893179	19	58899056	C	T	-6.75E-04	PRS-CSx
Colorectum	PRS7	rs893902	15	67014742	T	C	-4.19E-04	PRS-CSx
Colorectum	PRS7	rs894141	15	71635244	G	T	2.92E-04	PRS-CSx
Colorectum	PRS7	rs894839	11	120337705	G	A	-2.16E-04	PRS-CSx
Colorectum	PRS7	rs896010	8	83984810	A	G	-3.31E-04	PRS-CSx
Colorectum	PRS7	rs896324	8	128396512	G	A	-6.10E-04	PRS-CSx
Colorectum	PRS7	rs896791	2	130216772	C	T	-3.22E-04	PRS-CSx
Colorectum	PRS7	rs898034	2	28090820	T	C	6.89E-04	PRS-CSx
Colorectum	PRS7	rs898604	11	46917983	A	G	1.00E-04	PRS-CSx
Colorectum	PRS7	rs898888	15	67323244	A	G	1.79E-04	PRS-CSx
Colorectum	PRS7	rs899244	16	86700030	T	C	2.47E-03	PRS-CSx
Colorectum	PRS7	rs901688	3	41377373	G	A	-2.44E-04	PRS-CSx
Colorectum	PRS7	rs901746	11	47260319	A	G	8.18E-05	PRS-CSx
Colorectum	PRS7	rs902146	2	66332914	T	C	-1.68E-03	PRS-CSx
Colorectum	PRS7	rs90268	1	64985855	C	A	5.75E-04	PRS-CSx
Colorectum	PRS7	rs903319	1	220985811	C	T	4.22E-04	PRS-CSx
Colorectum	PRS7	rs903552	15	101997991	T	C	6.44E-04	PRS-CSx
Colorectum	PRS7	rs903678	1	201809918	A	G	4.95E-04	PRS-CSx
Colorectum	PRS7	rs903736	18	34101676	G	T	-4.42E-04	PRS-CSx
Colorectum	PRS7	rs904734	5	1661397	T	C	1.70E-04	PRS-CSx
Colorectum	PRS7	rs905165	5	39639421	T	C	1.40E-04	PRS-CSx
Colorectum	PRS7	rs905167	5	39639610	C	T	3.94E-04	PRS-CSx
Colorectum	PRS7	rs906932	14	99785010	T	C	-9.05E-04	PRS-CSx
Colorectum	PRS7	rs907371	15	77844375	C	T	-4.97E-04	PRS-CSx
Colorectum	PRS7	rs908664	12	43133634	A	C	-2.15E-03	PRS-CSx
Colorectum	PRS7	rs908807	3	156254213	G	A	-5.02E-04	PRS-CSx
Colorectum	PRS7	rs908831	9	139822542	C	T	-1.54E-04	PRS-CSx
Colorectum	PRS7	rs909675	22	36844619	A	C	-6.49E-04	PRS-CSx
Colorectum	PRS7	rs909728	6	29719561	T	C	4.54E-05	PRS-CSx
Colorectum	PRS7	rs909887	20	7805805	A	G	6.24E-04	PRS-CSx
Colorectum	PRS7	rs910049	6	32315727	T	C	4.95E-04	PRS-CSx
Colorectum	PRS7	rs910061	20	45188884	G	A	5.99E-04	PRS-CSx
Colorectum	PRS7	rs911078	20	60990075	G	T	2.89E-04	PRS-CSx
Colorectum	PRS7	rs9130	8	29920825	T	C	4.87E-04	PRS-CSx
Colorectum	PRS7	rs913178	1	109742452	C	T	6.76E-04	PRS-CSx
Colorectum	PRS7	rs913179	1	109742485	G	A	3.86E-04	PRS-CSx

Colorectum	PRS7	rs913835	9	34664488	A	G	1.05E-03	PRS-CSx
Colorectum	PRS7	rs914991	9	101834340	C	A	1.91E-04	PRS-CSx
Colorectum	PRS7	rs915171	6	131184914	T	C	2.43E-04	PRS-CSx
Colorectum	PRS7	rs915172	6	131184770	G	T	1.88E-04	PRS-CSx
Colorectum	PRS7	rs915895	6	32190217	T	C	-2.67E-04	PRS-CSx
Colorectum	PRS7	rs9163	11	117283808	T	C	4.35E-04	PRS-CSx
Colorectum	PRS7	rs916321	22	43041391	A	G	3.88E-04	PRS-CSx
Colorectum	PRS7	rs916682	12	111699146	A	G	-6.83E-04	PRS-CSx
Colorectum	PRS7	rs917065	14	71397846	A	G	-2.61E-04	PRS-CSx
Colorectum	PRS7	rs917427	14	72950368	T	C	-4.23E-04	PRS-CSx
Colorectum	PRS7	rs917571	17	59255231	A	G	-3.35E-04	PRS-CSx
Colorectum	PRS7	rs918119	12	107655354	C	T	7.77E-04	PRS-CSx
Colorectum	PRS7	rs918368	16	87678144	T	C	5.45E-04	PRS-CSx
Colorectum	PRS7	rs918811	2	96003762	T	G	-7.55E-05	PRS-CSx
Colorectum	PRS7	rs919033	8	41377203	G	A	1.42E-03	PRS-CSx
Colorectum	PRS7	rs919034	8	41377399	G	A	1.69E-03	PRS-CSx
Colorectum	PRS7	rs919096	3	41209869	A	G	1.06E-02	PRS-CSx
Colorectum	PRS7	rs919165	13	49393296	T	C	3.80E-04	PRS-CSx
Colorectum	PRS7	rs919197	20	57480933	C	T	-1.22E-04	PRS-CSx
Colorectum	PRS7	rs920285	10	9261962	A	C	-5.93E-04	PRS-CSx
Colorectum	PRS7	rs920390	2	43019144	C	T	6.64E-04	PRS-CSx
Colorectum	PRS7	rs920812	6	160490314	T	C	5.00E-04	PRS-CSx
Colorectum	PRS7	rs920980	5	896983	G	A	-9.07E-04	PRS-CSx
Colorectum	PRS7	rs9210	15	75128501	T	C	5.09E-04	PRS-CSx
Colorectum	PRS7	rs921070	2	43206843	A	G	-6.20E-04	PRS-CSx
Colorectum	PRS7	rs921160	3	63088792	A	G	-6.16E-04	PRS-CSx
Colorectum	PRS7	rs922177	15	64317620	T	C	7.46E-05	PRS-CSx
Colorectum	PRS7	rs924014	15	102017381	G	T	-1.04E-03	PRS-CSx
Colorectum	PRS7	rs925431	3	73769764	G	A	-2.24E-04	PRS-CSx
Colorectum	PRS7	rs9257802	6	29343355	T	C	5.54E-04	PRS-CSx
Colorectum	PRS7	rs9257805	6	29346329	G	A	3.69E-04	PRS-CSx
Colorectum	PRS7	rs9257887	6	29485289	A	G	4.46E-05	PRS-CSx
Colorectum	PRS7	rs9258158	6	29688180	G	A	1.33E-04	PRS-CSx
Colorectum	PRS7	rs9258170	6	29689590	G	T	1.77E-04	PRS-CSx
Colorectum	PRS7	rs9258186	6	29692334	G	A	1.69E-04	PRS-CSx
Colorectum	PRS7	rs9258205	6	29703823	C	T	1.10E-04	PRS-CSx
Colorectum	PRS7	rs9258206	6	29704237	A	G	1.13E-03	PRS-CSx
Colorectum	PRS7	rs9258207	6	29704443	T	C	-7.66E-05	PRS-CSx
Colorectum	PRS7	rs9258209	6	29705402	T	C	1.55E-03	PRS-CSx
Colorectum	PRS7	rs9258211	6	29706180	A	G	3.71E-05	PRS-CSx
Colorectum	PRS7	rs9258213	6	29706691	C	T	7.52E-04	PRS-CSx
Colorectum	PRS7	rs9258215	6	29707307	G	A	1.74E-04	PRS-CSx
Colorectum	PRS7	rs9258218	6	29708028	A	G	1.33E-04	PRS-CSx
Colorectum	PRS7	rs9258220	6	29711041	T	C	9.44E-04	PRS-CSx
Colorectum	PRS7	rs9258225	6	29712704	G	A	1.22E-04	PRS-CSx
Colorectum	PRS7	rs9258260	6	29723161	T	C	1.75E-04	PRS-CSx
Colorectum	PRS7	rs925857	3	5965084	C	T	-6.58E-04	PRS-CSx
Colorectum	PRS7	rs926070	6	32257566	G	A	-1.94E-04	PRS-CSx
Colorectum	PRS7	rs926242	6	14762428	A	G	-1.51E-03	PRS-CSx
Colorectum	PRS7	rs926350	22	43471948	C	T	2.81E-04	PRS-CSx
Colorectum	PRS7	rs9264916	6	31272774	A	G	2.66E-04	PRS-CSx
Colorectum	PRS7	rs9265459	6	31296680	A	C	-8.23E-05	PRS-CSx
Colorectum	PRS7	rs926611	20	49031872	T	C	2.93E-03	PRS-CSx
Colorectum	PRS7	rs926629	20	47335736	G	A	-2.51E-04	PRS-CSx
Colorectum	PRS7	rs9266327	6	31330619	A	G	7.08E-05	PRS-CSx
Colorectum	PRS7	rs9267487	6	31511350	C	T	2.02E-04	PRS-CSx

Colorectum	PRS7	rs9267810	6	32121594	C	T	-1.14E-04	PRS-CSx
Colorectum	PRS7	rs9267820	6	32165583	A	G	-3.99E-05	PRS-CSx
Colorectum	PRS7	rs9268005	6	32224388	C	A	-7.90E-05	PRS-CSx
Colorectum	PRS7	rs9268103	6	32245370	A	G	3.11E-05	PRS-CSx
Colorectum	PRS7	rs9268148	6	32259527	G	A	1.32E-04	PRS-CSx
Colorectum	PRS7	rs9268494	6	32375352	C	A	-5.92E-06	PRS-CSx
Colorectum	PRS7	rs9268499	6	32375695	A	G	-1.21E-04	PRS-CSx
Colorectum	PRS7	rs9275383	6	32668846	T	G	-1.06E-04	PRS-CSx
Colorectum	PRS7	rs9275522	6	32674970	T	C	-2.79E-05	PRS-CSx
Colorectum	PRS7	rs9275523	6	32674994	A	C	-4.93E-05	PRS-CSx
Colorectum	PRS7	rs9275555	6	32677088	T	C	1.54E-07	PRS-CSx
Colorectum	PRS7	rs9275582	6	32680070	T	C	-5.16E-05	PRS-CSx
Colorectum	PRS7	rs9275595	6	32681355	C	T	-6.16E-05	PRS-CSx
Colorectum	PRS7	rs9275602	6	32682812	A	C	4.37E-03	PRS-CSx
Colorectum	PRS7	rs9276171	6	32698918	G	A	9.04E-04	PRS-CSx
Colorectum	PRS7	rs9276826	6	32830568	G	A	-4.43E-03	PRS-CSx
Colorectum	PRS7	rs927737	6	84539678	T	C	8.81E-04	PRS-CSx
Colorectum	PRS7	rs9283502	2	154151468	C	T	5.24E-04	PRS-CSx
Colorectum	PRS7	rs9283588	3	133874566	G	A	-6.32E-04	PRS-CSx
Colorectum	PRS7	rs9283866	6	167096845	C	T	1.46E-03	PRS-CSx
Colorectum	PRS7	rs9288868	3	108077970	C	T	-6.81E-04	PRS-CSx
Colorectum	PRS7	rs9289204	3	122495447	G	T	-3.79E-04	PRS-CSx
Colorectum	PRS7	rs9289462	3	133990426	A	G	-2.23E-05	PRS-CSx
Colorectum	PRS7	rs9290481	3	173531229	G	A	1.32E-04	PRS-CSx
Colorectum	PRS7	rs9291289	4	46685463	A	G	-5.46E-04	PRS-CSx
Colorectum	PRS7	rs9291919	5	67300449	C	T	1.70E-04	PRS-CSx
Colorectum	PRS7	rs9292748	5	39701919	C	A	-4.13E-04	PRS-CSx
Colorectum	PRS7	rs9292756	5	39936006	T	C	2.04E-04	PRS-CSx
Colorectum	PRS7	rs929334	7	83744373	T	C	-3.08E-04	PRS-CSx
Colorectum	PRS7	rs9293511	5	88416354	C	T	2.01E-04	PRS-CSx
Colorectum	PRS7	rs9293687	5	75839124	A	G	4.01E-04	PRS-CSx
Colorectum	PRS7	rs9293688	5	75839165	C	T	1.84E-04	PRS-CSx
Colorectum	PRS7	rs9293946	6	74684983	C	T	-6.76E-04	PRS-CSx
Colorectum	PRS7	rs9293961	6	74985969	T	C	-8.93E-05	PRS-CSx
Colorectum	PRS7	rs9294935	6	169323526	T	C	7.33E-04	PRS-CSx
Colorectum	PRS7	rs9295050	6	168589564	T	C	3.42E-04	PRS-CSx
Colorectum	PRS7	rs9295464	6	20416572	T	G	-8.17E-04	PRS-CSx
Colorectum	PRS7	rs9295475	6	20652765	G	A	-6.68E-04	PRS-CSx
Colorectum	PRS7	rs9295478	6	20716253	A	G	-6.84E-05	PRS-CSx
Colorectum	PRS7	rs9295804	6	29336147	A	G	-2.80E-04	PRS-CSx
Colorectum	PRS7	rs9295829	6	30028800	G	A	2.02E-06	PRS-CSx
Colorectum	PRS7	rs9297191	8	32283471	A	G	-7.36E-04	PRS-CSx
Colorectum	PRS7	rs9297193	8	32283866	C	T	-5.30E-04	PRS-CSx
Colorectum	PRS7	rs9297756	8	128440167	A	C	7.29E-04	PRS-CSx
Colorectum	PRS7	rs9297951	8	96041437	A	G	5.68E-04	PRS-CSx
Colorectum	PRS7	rs929872	16	56265405	A	G	-3.29E-04	PRS-CSx
Colorectum	PRS7	rs9299425	9	97283226	G	A	-7.71E-04	PRS-CSx
Colorectum	PRS7	rs9300551	13	100138079	A	G	-1.45E-04	PRS-CSx
Colorectum	PRS7	rs9300699	13	102539941	T	G	-7.56E-04	PRS-CSx
Colorectum	PRS7	rs9300875	13	104534829	G	A	7.90E-04	PRS-CSx
Colorectum	PRS7	rs9301877	13	94021855	G	A	-4.87E-04	PRS-CSx
Colorectum	PRS7	rs9302160	15	51590011	T	C	2.91E-04	PRS-CSx
Colorectum	PRS7	rs9304998	19	22514551	C	T	5.34E-03	PRS-CSx
Colorectum	PRS7	rs930817	8	98944327	T	C	-3.96E-04	PRS-CSx
Colorectum	PRS7	rs9309141	2	47285714	T	G	8.86E-04	PRS-CSx
Colorectum	PRS7	rs9309332	2	61216165	C	T	-1.57E-03	PRS-CSx

Colorectum	PRS7	rs9309437	2	26102597	C	T	-2.12E-04	PRS-CSx
Colorectum	PRS7	rs9311149	3	37030175	A	C	-2.34E-04	PRS-CSx
Colorectum	PRS7	rs9311184	3	38310091	T	C	2.68E-03	PRS-CSx
Colorectum	PRS7	rs9311900	3	64605948	C	A	8.15E-04	PRS-CSx
Colorectum	PRS7	rs9311904	3	64622841	C	T	6.88E-04	PRS-CSx
Colorectum	PRS7	rs9312555	4	175411953	A	G	-3.25E-04	PRS-CSx
Colorectum	PRS7	rs9312699	4	59375582	A	G	-6.49E-04	PRS-CSx
Colorectum	PRS7	rs9315155	13	32648311	T	C	1.48E-03	PRS-CSx
Colorectum	PRS7	rs9315156	13	32664934	T	G	1.28E-03	PRS-CSx
Colorectum	PRS7	rs9315215	13	34043750	T	C	-9.42E-04	PRS-CSx
Colorectum	PRS7	rs9315226	13	34168454	C	T	-9.80E-04	PRS-CSx
Colorectum	PRS7	rs9315232	13	34204061	T	C	-2.81E-04	PRS-CSx
Colorectum	PRS7	rs931555	5	35803577	T	C	1.45E-04	PRS-CSx
Colorectum	PRS7	rs9315801	13	41586054	A	C	-2.46E-03	PRS-CSx
Colorectum	PRS7	rs931721	3	37211972	C	T	2.28E-05	PRS-CSx
Colorectum	PRS7	rs9318181	13	74050499	T	C	5.34E-04	PRS-CSx
Colorectum	PRS7	rs9319424	13	28847135	T	C	5.08E-04	PRS-CSx
Colorectum	PRS7	rs9319546	16	80023900	G	A	-7.30E-04	PRS-CSx
Colorectum	PRS7	rs9319548	16	80051414	T	G	-2.18E-04	PRS-CSx
Colorectum	PRS7	rs9320373	6	111965272	A	G	-6.18E-04	PRS-CSx
Colorectum	PRS7	rs9320558	6	116644101	T	G	4.69E-04	PRS-CSx
Colorectum	PRS7	rs9320604	6	117816045	A	G	3.39E-04	PRS-CSx
Colorectum	PRS7	rs9320760	6	98331498	A	G	3.35E-04	PRS-CSx
Colorectum	PRS7	rs9321265	6	131304233	T	C	2.93E-04	PRS-CSx
Colorectum	PRS7	rs9322866	14	31386426	T	C	-3.82E-04	PRS-CSx
Colorectum	PRS7	rs9323328	14	58653514	G	A	4.11E-04	PRS-CSx
Colorectum	PRS7	rs9323462	14	66083214	A	G	2.48E-04	PRS-CSx
Colorectum	PRS7	rs9324644	5	139921961	G	A	1.32E-04	PRS-CSx
Colorectum	PRS7	rs9324656	5	150170609	C	T	5.84E-04	PRS-CSx
Colorectum	PRS7	rs932683	14	54629941	A	G	1.41E-03	PRS-CSx
Colorectum	PRS7	rs9327455	5	127249194	A	G	-3.51E-05	PRS-CSx
Colorectum	PRS7	rs9327541	5	129342979	T	G	-2.84E-04	PRS-CSx
Colorectum	PRS7	rs932768	13	72347501	T	C	6.09E-04	PRS-CSx
Colorectum	PRS7	rs9327706	5	134512608	C	A	7.37E-04	PRS-CSx
Colorectum	PRS7	rs9328066	6	1702106	G	A	6.55E-04	PRS-CSx
Colorectum	PRS7	rs9328401	6	7225674	C	A	-2.93E-03	PRS-CSx
Colorectum	PRS7	rs9328722	17	812404	G	A	1.67E-03	PRS-CSx
Colorectum	PRS7	rs9329037	4	69812522	C	T	-1.34E-04	PRS-CSx
Colorectum	PRS7	rs9329570	1	38349606	G	T	2.44E-04	PRS-CSx
Colorectum	PRS7	rs932988	9	132218356	T	C	8.55E-04	PRS-CSx
Colorectum	PRS7	rs933399	12	111807499	G	A	1.32E-04	PRS-CSx
Colorectum	PRS7	rs933573	16	11793395	T	C	-2.81E-04	PRS-CSx
Colorectum	PRS7	rs9341459	6	74976178	G	A	-2.80E-04	PRS-CSx
Colorectum	PRS7	rs9341461	6	74997693	A	G	-1.15E-04	PRS-CSx
Colorectum	PRS7	rs9341464	6	75040446	A	G	-4.65E-04	PRS-CSx
Colorectum	PRS7	rs9342002	6	85534582	G	A	1.09E-03	PRS-CSx
Colorectum	PRS7	rs934299	2	137338940	T	G	1.06E-03	PRS-CSx
Colorectum	PRS7	rs9342997	6	73757091	C	T	-4.74E-04	PRS-CSx
Colorectum	PRS7	rs9343128	6	75001722	C	A	-4.03E-04	PRS-CSx
Colorectum	PRS7	rs9343135	6	75031687	A	C	-3.64E-04	PRS-CSx
Colorectum	PRS7	rs9347562	6	162328273	T	C	-2.12E-04	PRS-CSx
Colorectum	PRS7	rs9347820	6	164563957	A	G	-5.42E-04	PRS-CSx
Colorectum	PRS7	rs9348428	6	20427900	T	C	-4.96E-04	PRS-CSx
Colorectum	PRS7	rs9348440	6	20641336	T	C	-3.10E-04	PRS-CSx
Colorectum	PRS7	rs9350270	6	20667799	G	A	-1.54E-04	PRS-CSx
Colorectum	PRS7	rs9350276	6	20740296	C	T	3.31E-04	PRS-CSx

Colorectum	PRS7	rs9350516	6	74715439	T	C	-6.66E-04	PRS-CSx
Colorectum	PRS7	rs9350532	6	74974439	T	C	-1.77E-04	PRS-CSx
Colorectum	PRS7	rs9351963	6	73749861	C	A	-9.17E-04	PRS-CSx
Colorectum	PRS7	rs935201	17	77911717	G	A	9.22E-04	PRS-CSx
Colorectum	PRS7	rs9352087	6	74987346	T	C	-2.12E-04	PRS-CSx
Colorectum	PRS7	rs9352089	6	74988454	A	G	-2.16E-04	PRS-CSx
Colorectum	PRS7	rs9356743	6	20667688	T	C	-7.80E-05	PRS-CSx
Colorectum	PRS7	rs9356746	6	20720279	C	T	-2.82E-04	PRS-CSx
Colorectum	PRS7	rs9356756	6	20844151	G	A	1.12E-04	PRS-CSx
Colorectum	PRS7	rs9357092	6	29984252	A	G	-1.90E-04	PRS-CSx
Colorectum	PRS7	rs9358029	6	14198772	A	C	-2.08E-03	PRS-CSx
Colorectum	PRS7	rs9360362	6	69525365	G	A	-3.48E-04	PRS-CSx
Colorectum	PRS7	rs9360630	6	73752358	T	C	-6.37E-04	PRS-CSx
Colorectum	PRS7	rs9360745	6	74879308	G	A	-7.06E-04	PRS-CSx
Colorectum	PRS7	rs9360954	6	76609392	A	G	-2.40E-04	PRS-CSx
Colorectum	PRS7	rs936230	15	75145098	T	C	2.10E-04	PRS-CSx
Colorectum	PRS7	rs9362902	6	92390455	C	T	-1.93E-03	PRS-CSx
Colorectum	PRS7	rs9362961	6	92964208	A	G	-4.19E-04	PRS-CSx
Colorectum	PRS7	rs9364627	6	162338927	T	C	-3.64E-04	PRS-CSx
Colorectum	PRS7	rs9365665	6	164565554	T	C	-1.12E-03	PRS-CSx
Colorectum	PRS7	rs9366717	6	28191057	T	C	-1.50E-04	PRS-CSx
Colorectum	PRS7	rs9366752	6	30024677	T	C	-1.53E-04	PRS-CSx
Colorectum	PRS7	rs9367605	6	54917442	T	C	-7.63E-04	PRS-CSx
Colorectum	PRS7	rs9369003	6	11961565	C	T	4.30E-04	PRS-CSx
Colorectum	PRS7	rs9370340	6	54804798	C	T	-6.11E-04	PRS-CSx
Colorectum	PRS7	rs9370341	6	54811826	C	T	-5.66E-04	PRS-CSx
Colorectum	PRS7	rs9370383	6	54981216	C	T	-1.45E-03	PRS-CSx
Colorectum	PRS7	rs9372649	6	98395205	C	T	5.01E-04	PRS-CSx
Colorectum	PRS7	rs9372855	6	127294497	A	G	1.21E-04	PRS-CSx
Colorectum	PRS7	rs9373924	6	107483340	T	G	-1.07E-03	PRS-CSx
Colorectum	PRS7	rs9374615	6	116937342	T	C	-1.91E-05	PRS-CSx
Colorectum	PRS7	rs9374655	6	117709988	A	G	-2.19E-04	PRS-CSx
Colorectum	PRS7	rs9375098	6	98481792	C	T	3.68E-04	PRS-CSx
Colorectum	PRS7	rs9375479	6	127286233	C	T	1.23E-04	PRS-CSx
Colorectum	PRS7	rs9376092	6	135427144	A	C	7.23E-04	PRS-CSx
Colorectum	PRS7	rs9376940	6	100818915	G	A	-3.83E-04	PRS-CSx
Colorectum	PRS7	rs9377010	6	146922544	A	G	1.48E-03	PRS-CSx
Colorectum	PRS7	rs9378664	6	1955398	T	C	4.18E-04	PRS-CSx
Colorectum	PRS7	rs9379030	6	6395922	G	A	-9.80E-04	PRS-CSx
Colorectum	PRS7	rs9379059	6	6726219	T	C	-8.73E-04	PRS-CSx
Colorectum	PRS7	rs9379081	6	7219873	T	G	-1.03E-03	PRS-CSx
Colorectum	PRS7	rs9379400	6	22445521	C	T	-6.85E-04	PRS-CSx
Colorectum	PRS7	rs9379723	6	25145712	T	C	-1.45E-03	PRS-CSx
Colorectum	PRS7	rs9379772	6	25573168	C	A	-4.05E-04	PRS-CSx
Colorectum	PRS7	rs9379892	6	26582414	G	A	-7.20E-04	PRS-CSx
Colorectum	PRS7	rs9380150	6	30010492	C	T	2.54E-05	PRS-CSx
Colorectum	PRS7	rs9380326	6	32798283	C	T	-1.24E-03	PRS-CSx
Colorectum	PRS7	rs9381237	6	43320410	T	C	-2.53E-04	PRS-CSx
Colorectum	PRS7	rs9382424	6	54886014	G	A	-1.44E-03	PRS-CSx
Colorectum	PRS7	rs9383930	6	151914421	A	G	-4.43E-04	PRS-CSx
Colorectum	PRS7	rs9384983	6	116958653	A	G	1.68E-05	PRS-CSx
Colorectum	PRS7	rs9387478	6	117786180	A	C	4.10E-04	PRS-CSx
Colorectum	PRS7	rs9387768	6	98316879	A	G	3.08E-04	PRS-CSx
Colorectum	PRS7	rs9387954	6	98419104	A	G	4.37E-04	PRS-CSx
Colorectum	PRS7	rs938869	2	131019189	C	T	4.71E-04	PRS-CSx
Colorectum	PRS7	rs9388833	6	131032549	T	G	2.14E-04	PRS-CSx

Colorectum	PRS7	rs9389691	6	139816000	A	G	1.41E-03	PRS-CSx
Colorectum	PRS7	rs9389846	6	141327566	C	T	-9.43E-04	PRS-CSx
Colorectum	PRS7	rs9389857	6	141372966	T	C	-1.34E-03	PRS-CSx
Colorectum	PRS7	rs9390325	6	100817828	C	T	-3.00E-04	PRS-CSx
Colorectum	PRS7	rs9390576	6	148820512	G	A	1.31E-03	PRS-CSx
Colorectum	PRS7	rs9392346	6	1929255	G	A	3.27E-04	PRS-CSx
Colorectum	PRS7	rs9392530	6	3842866	T	C	1.11E-03	PRS-CSx
Colorectum	PRS7	rs9392554	6	4144199	T	C	8.25E-04	PRS-CSx
Colorectum	PRS7	rs9393647	6	25494796	A	G	-2.46E-04	PRS-CSx
Colorectum	PRS7	rs9394699	6	40449290	A	G	1.64E-03	PRS-CSx
Colorectum	PRS7	rs939595	1	151783688	A	C	-2.30E-04	PRS-CSx
Colorectum	PRS7	rs9396600	6	15741773	T	C	-1.22E-03	PRS-CSx
Colorectum	PRS7	rs9397064	6	151914951	C	T	1.17E-03	PRS-CSx
Colorectum	PRS7	rs9397065	6	151919377	A	G	-1.00E-03	PRS-CSx
Colorectum	PRS7	rs9397066	6	151919936	G	A	-6.37E-04	PRS-CSx
Colorectum	PRS7	rs9397433	6	151919432	A	C	2.18E-03	PRS-CSx
Colorectum	PRS7	rs9397655	6	154111389	A	G	-4.58E-04	PRS-CSx
Colorectum	PRS7	rs9398302	6	112556666	A	G	8.21E-04	PRS-CSx
Colorectum	PRS7	rs939998	2	159770970	C	T	-1.93E-04	PRS-CSx
Colorectum	PRS7	rs940026	15	38545799	A	G	-8.11E-04	PRS-CSx
Colorectum	PRS7	rs9400957	6	117051934	G	A	-2.41E-04	PRS-CSx
Colorectum	PRS7	rs9401001	6	117700729	A	C	-1.43E-04	PRS-CSx
Colorectum	PRS7	rs9401491	6	98481904	A	G	3.99E-04	PRS-CSx
Colorectum	PRS7	rs9401928	6	127298394	G	A	2.41E-04	PRS-CSx
Colorectum	PRS7	rs9402271	6	131036132	T	C	3.88E-04	PRS-CSx
Colorectum	PRS7	rs9403792	6	146922849	C	T	1.18E-03	PRS-CSx
Colorectum	PRS7	rs9405044	6	30006765	A	G	-8.90E-05	PRS-CSx
Colorectum	PRS7	rs9405505	6	1705317	A	G	4.90E-04	PRS-CSx
Colorectum	PRS7	rs9405702	6	4146704	C	T	7.29E-04	PRS-CSx
Colorectum	PRS7	rs9407333	9	651364	C	A	3.87E-04	PRS-CSx
Colorectum	PRS7	rs9407334	9	651461	T	C	2.17E-04	PRS-CSx
Colorectum	PRS7	rs9407354	9	676692	C	T	2.30E-04	PRS-CSx
Colorectum	PRS7	rs941288	7	99776808	A	G	3.69E-04	PRS-CSx
Colorectum	PRS7	rs941289	7	99678491	G	A	1.88E-04	PRS-CSx
Colorectum	PRS7	rs941752	14	58799361	G	A	1.40E-04	PRS-CSx
Colorectum	PRS7	rs942201	10	6086292	T	G	-3.43E-05	PRS-CSx
Colorectum	PRS7	rs9425569	1	182942202	G	A	1.57E-04	PRS-CSx
Colorectum	PRS7	rs9427965	1	89260091	G	A	-2.36E-04	PRS-CSx
Colorectum	PRS7	rs9428015	1	89269340	G	A	-2.72E-04	PRS-CSx
Colorectum	PRS7	rs9434742	1	9324571	C	T	6.05E-04	PRS-CSx
Colorectum	PRS7	rs944052	14	64805832	A	G	2.73E-04	PRS-CSx
Colorectum	PRS7	rs944141	9	5490522	T	C	-9.18E-04	PRS-CSx
Colorectum	PRS7	rs944378	13	78605893	G	A	-9.54E-04	PRS-CSx
Colorectum	PRS7	rs9446827	6	73746516	A	C	-8.53E-04	PRS-CSx
Colorectum	PRS7	rs9446833	6	73789370	C	T	-1.17E-03	PRS-CSx
Colorectum	PRS7	rs944697	10	20648238	A	G	-8.26E-05	PRS-CSx
Colorectum	PRS7	rs944714	10	6475157	A	G	-5.80E-04	PRS-CSx
Colorectum	PRS7	rs944758	9	139483954	C	T	-7.54E-04	PRS-CSx
Colorectum	PRS7	rs944797	9	22115286	T	C	1.32E-03	PRS-CSx
Colorectum	PRS7	rs944971	1	183112505	T	C	1.37E-04	PRS-CSx
Colorectum	PRS7	rs945385	9	139756477	G	A	-1.23E-04	PRS-CSx
Colorectum	PRS7	rs9455487	6	169248186	A	G	-7.46E-05	PRS-CSx
Colorectum	PRS7	rs9455591	6	168583118	A	G	-1.37E-03	PRS-CSx
Colorectum	PRS7	rs9455756	6	169280650	A	G	-1.47E-04	PRS-CSx
Colorectum	PRS7	rs9455968	6	168581362	A	C	-4.75E-04	PRS-CSx
Colorectum	PRS7	rs9455973	6	168583006	A	G	-7.56E-04	PRS-CSx

Colorectum	PRS7	rs9455975	6	168583749	T	C	-6.44E-04	PRS-CSx
Colorectum	PRS7	rs9458419	6	162344712	C	T	-3.27E-04	PRS-CSx
Colorectum	PRS7	rs945889	6	130321244	C	T	1.75E-03	PRS-CSx
Colorectum	PRS7	rs9460540	6	20648762	G	A	5.49E-04	PRS-CSx
Colorectum	PRS7	rs9460546	6	20663632	G	T	-3.73E-04	PRS-CSx
Colorectum	PRS7	rs9461271	6	26554968	G	A	3.87E-05	PRS-CSx
Colorectum	PRS7	rs9461273	6	26584526	G	A	-2.13E-04	PRS-CSx
Colorectum	PRS7	rs9462209	6	36628042	T	G	-4.75E-04	PRS-CSx
Colorectum	PRS7	rs9462377	6	12021539	T	C	-3.44E-04	PRS-CSx
Colorectum	PRS7	rs9462736	6	41655340	T	C	-5.10E-04	PRS-CSx
Colorectum	PRS7	rs9462738	6	41664501	T	G	-4.46E-04	PRS-CSx
Colorectum	PRS7	rs9463408	6	13063824	G	A	6.77E-04	PRS-CSx
Colorectum	PRS7	rs9465851	6	20640316	T	C	2.99E-04	PRS-CSx
Colorectum	PRS7	rs9465852	6	20641900	A	G	5.57E-04	PRS-CSx
Colorectum	PRS7	rs9465871	6	20717255	C	T	-6.27E-05	PRS-CSx
Colorectum	PRS7	rs9466265	6	22105169	C	T	3.85E-04	PRS-CSx
Colorectum	PRS7	rs9466937	6	23858932	T	C	-3.64E-04	PRS-CSx
Colorectum	PRS7	rs9466949	6	23869466	C	T	-6.35E-04	PRS-CSx
Colorectum	PRS7	rs9466969	6	23897347	C	T	-1.90E-04	PRS-CSx
Colorectum	PRS7	rs9467521	6	25463364	C	T	-6.64E-04	PRS-CSx
Colorectum	PRS7	rs9467810	6	26608261	A	C	-1.48E-04	PRS-CSx
Colorectum	PRS7	rs9468344	6	28309569	G	T	3.40E-04	PRS-CSx
Colorectum	PRS7	rs9470366	6	36625562	A	G	3.98E-03	PRS-CSx
Colorectum	PRS7	rs9470983	6	39127045	T	C	-3.06E-04	PRS-CSx
Colorectum	PRS7	rs9470986	6	39128424	T	C	-7.10E-04	PRS-CSx
Colorectum	PRS7	rs9473427	6	13078644	C	T	9.76E-04	PRS-CSx
Colorectum	PRS7	rs947501	11	34753375	A	G	-5.78E-04	PRS-CSx
Colorectum	PRS7	rs9475687	6	56231402	T	C	-3.93E-04	PRS-CSx
Colorectum	PRS7	rs9479402	6	153135339	C	T	1.94E-03	PRS-CSx
Colorectum	PRS7	rs9481653	6	116986201	C	T	-1.87E-04	PRS-CSx
Colorectum	PRS7	rs948181	11	117208949	T	C	3.31E-04	PRS-CSx
Colorectum	PRS7	rs9482263	6	98539569	C	T	5.79E-04	PRS-CSx
Colorectum	PRS7	rs9489028	6	117050110	C	T	-2.71E-04	PRS-CSx
Colorectum	PRS7	rs9492762	6	131260784	T	G	6.39E-04	PRS-CSx
Colorectum	PRS7	rs9492767	6	131273010	C	T	1.21E-04	PRS-CSx
Colorectum	PRS7	rs949306	18	3327801	A	G	7.64E-04	PRS-CSx
Colorectum	PRS7	rs9494145	6	135432552	C	T	5.71E-04	PRS-CSx
Colorectum	PRS7	rs949827	1	59841785	C	T	2.42E-04	PRS-CSx
Colorectum	PRS7	rs9501624	6	32399286	G	A	2.76E-04	PRS-CSx
Colorectum	PRS7	rs9501626	6	32400344	A	C	-2.48E-03	PRS-CSx
Colorectum	PRS7	rs9501677	6	29497002	A	G	5.95E-04	PRS-CSx
Colorectum	PRS7	rs9503009	6	1701839	T	C	8.00E-04	PRS-CSx
Colorectum	PRS7	rs9503011	6	1704150	T	C	9.79E-04	PRS-CSx
Colorectum	PRS7	rs9503755	6	3842494	G	T	-9.09E-04	PRS-CSx
Colorectum	PRS7	rs9505309	6	7917528	G	T	-1.44E-03	PRS-CSx
Colorectum	PRS7	rs950777	18	34120008	T	C	-6.60E-04	PRS-CSx
Colorectum	PRS7	rs9511023	13	24631796	A	G	1.13E-04	PRS-CSx
Colorectum	PRS7	rs9511834	13	26194059	C	A	-6.95E-04	PRS-CSx
Colorectum	PRS7	rs9515211	13	111074436	A	G	-3.11E-04	PRS-CSx
Colorectum	PRS7	rs9515744	13	91087419	C	T	-9.32E-04	PRS-CSx
Colorectum	PRS7	rs9515975	13	92456597	C	A	3.01E-04	PRS-CSx
Colorectum	PRS7	rs9516225	13	94015230	G	A	1.42E-04	PRS-CSx
Colorectum	PRS7	rs9517759	13	100180108	T	G	-4.39E-04	PRS-CSx
Colorectum	PRS7	rs9517765	13	100202206	C	T	-6.71E-05	PRS-CSx
Colorectum	PRS7	rs9517766	13	100202301	G	A	-1.07E-04	PRS-CSx
Colorectum	PRS7	rs9518872	13	103576289	A	G	-1.57E-03	PRS-CSx

Colorectum	PRS7	rs9518874	13	103577244	A	G	-1.39E-03	PRS-CSx
Colorectum	PRS7	rs9520152	13	107376044	G	A	-5.02E-04	PRS-CSx
Colorectum	PRS7	rs952058	18	73285316	T	G	5.26E-04	PRS-CSx
Colorectum	PRS7	rs9520800	13	108783295	G	A	-1.25E-03	PRS-CSx
Colorectum	PRS7	rs952151	11	121658512	A	G	8.83E-04	PRS-CSx
Colorectum	PRS7	rs9521747	13	111060205	C	T	2.99E-04	PRS-CSx
Colorectum	PRS7	rs9521748	13	111062680	G	A	5.62E-05	PRS-CSx
Colorectum	PRS7	rs9521755	13	111073710	C	T	1.40E-04	PRS-CSx
Colorectum	PRS7	rs9521960	13	111492882	A	G	-1.07E-04	PRS-CSx
Colorectum	PRS7	rs9521997	13	111583422	G	A	-4.29E-05	PRS-CSx
Colorectum	PRS7	rs952318	12	51029266	A	C	-5.59E-05	PRS-CSx
Colorectum	PRS7	rs9524827	13	95861242	C	T	-2.66E-04	PRS-CSx
Colorectum	PRS7	rs9525568	13	42663251	G	A	-1.95E-04	PRS-CSx
Colorectum	PRS7	rs9526061	13	32877888	C	A	-8.86E-04	PRS-CSx
Colorectum	PRS7	rs9526193	13	47113251	C	T	7.27E-04	PRS-CSx
Colorectum	PRS7	rs9527458	13	56493079	C	T	1.79E-03	PRS-CSx
Colorectum	PRS7	rs9527554	13	34025577	G	A	-8.96E-04	PRS-CSx
Colorectum	PRS7	rs9527571	13	34039223	G	A	-8.26E-04	PRS-CSx
Colorectum	PRS7	rs9529782	13	71155658	A	C	1.01E-04	PRS-CSx
Colorectum	PRS7	rs9530136	13	73591265	T	C	2.77E-04	PRS-CSx
Colorectum	PRS7	rs9531693	13	85564579	A	G	-2.73E-04	PRS-CSx
Colorectum	PRS7	rs953201	3	112897695	C	T	2.08E-04	PRS-CSx
Colorectum	PRS7	rs9532580	13	41244260	C	T	-3.71E-04	PRS-CSx
Colorectum	PRS7	rs9532984	13	42634693	A	G	9.14E-05	PRS-CSx
Colorectum	PRS7	rs9532985	13	42644377	A	C	7.59E-05	PRS-CSx
Colorectum	PRS7	rs9534154	13	32886213	A	G	-6.75E-04	PRS-CSx
Colorectum	PRS7	rs953420	20	6416132	T	C	9.72E-05	PRS-CSx
Colorectum	PRS7	rs9534411	13	47076565	G	A	8.96E-04	PRS-CSx
Colorectum	PRS7	rs9536269	13	53318904	C	T	-4.31E-04	PRS-CSx
Colorectum	PRS7	rs9536274	13	53322022	C	T	-4.71E-04	PRS-CSx
Colorectum	PRS7	rs9536277	13	53328934	C	A	-4.36E-04	PRS-CSx
Colorectum	PRS7	rs9537477	13	34020479	G	A	-4.02E-04	PRS-CSx
Colorectum	PRS7	rs9537503	13	34031279	A	G	-6.02E-04	PRS-CSx
Colorectum	PRS7	rs9537647	13	57749024	A	G	3.35E-04	PRS-CSx
Colorectum	PRS7	rs9538300	13	34250978	C	T	-4.50E-04	PRS-CSx
Colorectum	PRS7	rs953945	2	225801484	T	C	-3.10E-04	PRS-CSx
Colorectum	PRS7	rs9543110	13	73324751	T	C	1.20E-04	PRS-CSx
Colorectum	PRS7	rs9543363	13	73982010	T	C	-1.06E-03	PRS-CSx
Colorectum	PRS7	rs9544490	13	77989634	T	C	5.13E-05	PRS-CSx
Colorectum	PRS7	rs9547016	13	85559897	G	A	-4.09E-04	PRS-CSx
Colorectum	PRS7	rs9547030	13	85567636	T	G	-3.18E-04	PRS-CSx
Colorectum	PRS7	rs9548010	13	38244897	A	G	8.00E-04	PRS-CSx
Colorectum	PRS7	rs9548017	13	38261906	T	C	1.41E-03	PRS-CSx
Colorectum	PRS7	rs9551048	13	24636756	A	G	1.53E-04	PRS-CSx
Colorectum	PRS7	rs9551447	13	28717392	T	G	-3.96E-04	PRS-CSx
Colorectum	PRS7	rs9551449	13	28812308	G	A	-5.31E-04	PRS-CSx
Colorectum	PRS7	rs9551453	13	28830997	G	T	-4.75E-04	PRS-CSx
Colorectum	PRS7	rs955203	1	213830592	C	A	1.11E-03	PRS-CSx
Colorectum	PRS7	rs955293	8	121080548	A	G	-4.20E-04	PRS-CSx
Colorectum	PRS7	rs9553631	13	26180090	A	G	-5.33E-04	PRS-CSx
Colorectum	PRS7	rs9554277	13	28728026	A	G	-5.06E-04	PRS-CSx
Colorectum	PRS7	rs9554285	13	28779477	G	A	-2.33E-04	PRS-CSx
Colorectum	PRS7	rs9554296	13	28833143	T	C	-4.57E-04	PRS-CSx
Colorectum	PRS7	rs9554311	13	28867268	C	T	-2.65E-04	PRS-CSx
Colorectum	PRS7	rs9555744	13	111582414	T	C	-1.61E-04	PRS-CSx
Colorectum	PRS7	rs9557251	13	100178627	A	G	-1.71E-04	PRS-CSx

Colorectum	PRS7	rs9557751	13	102543003	G	A	-2.02E-04	PRS-CSx
Colorectum	PRS7	rs9560875	13	92515927	T	C	2.32E-04	PRS-CSx
Colorectum	PRS7	rs9561314	13	93988638	T	C	-5.84E-04	PRS-CSx
Colorectum	PRS7	rs9561328	13	94009312	T	G	-6.92E-04	PRS-CSx
Colorectum	PRS7	rs9561336	13	94034140	A	G	-2.97E-04	PRS-CSx
Colorectum	PRS7	rs9562369	13	42590075	T	C	-1.29E-03	PRS-CSx
Colorectum	PRS7	rs9563522	13	34111581	A	G	-2.97E-04	PRS-CSx
Colorectum	PRS7	rs9563616	13	34193251	T	G	-4.84E-04	PRS-CSx
Colorectum	PRS7	rs9564882	13	72756424	G	T	3.21E-04	PRS-CSx
Colorectum	PRS7	rs9564996	13	74039264	G	A	3.36E-04	PRS-CSx
Colorectum	PRS7	rs9565007	13	74074823	T	C	-1.63E-05	PRS-CSx
Colorectum	PRS7	rs9565011	13	74087542	G	A	1.46E-04	PRS-CSx
Colorectum	PRS7	rs9566690	13	41656159	A	G	-1.12E-03	PRS-CSx
Colorectum	PRS7	rs9566697	13	41676452	A	G	-7.85E-04	PRS-CSx
Colorectum	PRS7	rs9566698	13	41687578	T	C	-1.28E-03	PRS-CSx
Colorectum	PRS7	rs9567163	13	32676605	T	C	1.77E-03	PRS-CSx
Colorectum	PRS7	rs956753	12	11810558	C	T	2.61E-03	PRS-CSx
Colorectum	PRS7	rs9568098	13	49426424	C	T	5.34E-04	PRS-CSx
Colorectum	PRS7	rs9568113	13	49451863	C	T	5.48E-04	PRS-CSx
Colorectum	PRS7	rs9568534	13	51728432	T	G	1.84E-03	PRS-CSx
Colorectum	PRS7	rs9569761	13	34123373	G	T	-1.44E-04	PRS-CSx
Colorectum	PRS7	rs957085	4	17107631	A	G	-5.45E-04	PRS-CSx
Colorectum	PRS7	rs9572782	13	72339141	T	C	8.34E-04	PRS-CSx
Colorectum	PRS7	rs9572784	13	72341727	C	T	4.24E-04	PRS-CSx
Colorectum	PRS7	rs9572786	13	72342605	A	G	6.99E-04	PRS-CSx
Colorectum	PRS7	rs9572787	13	72366606	T	G	2.17E-04	PRS-CSx
Colorectum	PRS7	rs9572788	13	72367372	C	T	3.38E-04	PRS-CSx
Colorectum	PRS7	rs9572899	13	72746142	G	A	5.89E-04	PRS-CSx
Colorectum	PRS7	rs9572900	13	72754543	T	C	8.16E-04	PRS-CSx
Colorectum	PRS7	rs9573073	13	73539853	C	T	-1.87E-05	PRS-CSx
Colorectum	PRS7	rs9573193	13	74004024	C	T	9.05E-04	PRS-CSx
Colorectum	PRS7	rs9573195	13	74004630	A	G	-5.23E-04	PRS-CSx
Colorectum	PRS7	rs9573196	13	74008197	G	A	-4.08E-04	PRS-CSx
Colorectum	PRS7	rs9573215	13	74031332	G	A	8.72E-04	PRS-CSx
Colorectum	PRS7	rs9573216	13	74032735	C	T	2.41E-04	PRS-CSx
Colorectum	PRS7	rs9573218	13	74036922	C	T	2.81E-04	PRS-CSx
Colorectum	PRS7	rs9573220	13	74042621	T	C	-2.26E-04	PRS-CSx
Colorectum	PRS7	rs9573221	13	74047191	C	T	2.77E-04	PRS-CSx
Colorectum	PRS7	rs9573223	13	74056451	G	A	-1.05E-04	PRS-CSx
Colorectum	PRS7	rs9573225	13	74059168	A	G	5.86E-04	PRS-CSx
Colorectum	PRS7	rs9573545	13	76031966	A	G	-1.00E-03	PRS-CSx
Colorectum	PRS7	rs9573546	13	76032533	A	G	-5.13E-04	PRS-CSx
Colorectum	PRS7	rs957566	15	47576969	T	C	-3.95E-04	PRS-CSx
Colorectum	PRS7	rs9578196	13	31311557	T	C	-1.27E-03	PRS-CSx
Colorectum	PRS7	rs9579143	13	28589509	T	C	2.55E-03	PRS-CSx
Colorectum	PRS7	rs9582004	13	28733244	A	G	-2.94E-04	PRS-CSx
Colorectum	PRS7	rs9584701	13	98505630	A	G	-1.08E-03	PRS-CSx
Colorectum	PRS7	rs9589358	13	92496197	C	T	7.12E-04	PRS-CSx
Colorectum	PRS7	rs959024	6	152654160	A	G	6.65E-04	PRS-CSx
Colorectum	PRS7	rs9591873	13	34170118	G	A	-1.80E-03	PRS-CSx
Colorectum	PRS7	rs9593519	13	81608496	A	G	5.03E-04	PRS-CSx
Colorectum	PRS7	rs959606	10	8835711	A	C	2.66E-04	PRS-CSx
Colorectum	PRS7	rs9596486	13	51837299	G	A	1.09E-03	PRS-CSx
Colorectum	PRS7	rs9597711	13	34174766	C	T	-5.73E-04	PRS-CSx
Colorectum	PRS7	rs9600072	13	73711349	A	G	-4.89E-04	PRS-CSx
Colorectum	PRS7	rs9600128	13	74090415	A	C	-4.24E-05	PRS-CSx

Colorectum	PRS7	rs9601511	13	81577275	T	G	5.09E-04	PRS-CSx
Colorectum	PRS7	rs9607601	22	39399438	T	C	5.64E-04	PRS-CSx
Colorectum	PRS7	rs960887	8	121048383	A	G	-2.98E-04	PRS-CSx
Colorectum	PRS7	rs9611069	22	39395612	A	G	4.19E-04	PRS-CSx
Colorectum	PRS7	rs961253	20	6404281	A	C	1.06E-03	PRS-CSx
Colorectum	PRS7	rs961265	5	159437466	T	C	4.48E-04	PRS-CSx
Colorectum	PRS7	rs9613347	22	27420318	G	A	1.22E-03	PRS-CSx
Colorectum	PRS7	rs961341	13	28786220	G	A	-3.12E-04	PRS-CSx
Colorectum	PRS7	rs9613777	22	29420482	A	G	-6.32E-04	PRS-CSx
Colorectum	PRS7	rs961959	9	121550035	A	C	7.20E-04	PRS-CSx
Colorectum	PRS7	rs962079	4	15194361	A	C	-6.21E-04	PRS-CSx
Colorectum	PRS7	rs962375	14	26770873	T	C	1.58E-03	PRS-CSx
Colorectum	PRS7	rs9628999	1	104829326	A	G	3.97E-04	PRS-CSx
Colorectum	PRS7	rs9630218	11	69910840	T	C	5.95E-04	PRS-CSx
Colorectum	PRS7	rs9630219	11	69910890	T	G	1.20E-03	PRS-CSx
Colorectum	PRS7	rs963078	3	37220509	T	G	-4.19E-05	PRS-CSx
Colorectum	PRS7	rs9635117	13	112551202	T	C	2.00E-03	PRS-CSx
Colorectum	PRS7	rs963987	17	40561679	A	G	3.85E-04	PRS-CSx
Colorectum	PRS7	rs9642879	8	128575733	C	A	1.29E-03	PRS-CSx
Colorectum	PRS7	rs964502	11	7050609	C	T	-2.76E-04	PRS-CSx
Colorectum	PRS7	rs964551	11	46897995	T	G	2.63E-04	PRS-CSx
Colorectum	PRS7	rs964610	1	104803458	C	T	2.68E-04	PRS-CSx
Colorectum	PRS7	rs9647159	21	35750975	G	T	8.07E-03	PRS-CSx
Colorectum	PRS7	rs9649524	7	128800696	G	A	-4.16E-04	PRS-CSx
Colorectum	PRS7	rs964958	20	17128766	A	C	5.27E-04	PRS-CSx
Colorectum	PRS7	rs9649875	7	154620262	T	C	4.06E-04	PRS-CSx
Colorectum	PRS7	rs9650068	8	117643773	A	G	7.47E-05	PRS-CSx
Colorectum	PRS7	rs9651170	1	104768887	T	G	1.61E-03	PRS-CSx
Colorectum	PRS7	rs9651573	11	16158420	G	A	-2.94E-04	PRS-CSx
Colorectum	PRS7	rs9651682	11	117240357	A	G	5.54E-04	PRS-CSx
Colorectum	PRS7	rs9651966	12	80028738	T	C	3.67E-04	PRS-CSx
Colorectum	PRS7	rs9652090	13	27983367	T	G	-1.59E-03	PRS-CSx
Colorectum	PRS7	rs9655606	7	151114040	T	C	-2.64E-04	PRS-CSx
Colorectum	PRS7	rs9659310	1	246014673	T	C	-8.31E-04	PRS-CSx
Colorectum	PRS7	rs9660719	1	246003671	T	C	4.13E-04	PRS-CSx
Colorectum	PRS7	rs9661078	1	11936324	A	C	-4.16E-04	PRS-CSx
Colorectum	PRS7	rs9662165	1	246014229	C	A	-6.80E-04	PRS-CSx
Colorectum	PRS7	rs9665552	10	8811034	C	T	2.34E-04	PRS-CSx
Colorectum	PRS7	rs966650	4	23609928	A	G	-6.00E-05	PRS-CSx
Colorectum	PRS7	rs967044	18	53958845	C	T	-2.96E-04	PRS-CSx
Colorectum	PRS7	rs9671414	14	103967299	G	A	-3.84E-04	PRS-CSx
Colorectum	PRS7	rs9675262	17	13395795	T	C	9.22E-04	PRS-CSx
Colorectum	PRS7	rs9676574	19	22678385	A	C	-6.05E-04	PRS-CSx
Colorectum	PRS7	rs9678800	2	96009318	C	A	-9.97E-05	PRS-CSx
Colorectum	PRS7	rs9679392	2	26685633	A	G	-1.02E-03	PRS-CSx
Colorectum	PRS7	rs9679697	2	207156382	G	A	-1.77E-04	PRS-CSx
Colorectum	PRS7	rs9680768	22	43787289	C	T	-1.34E-03	PRS-CSx
Colorectum	PRS7	rs9684357	4	94806875	A	G	3.59E-04	PRS-CSx
Colorectum	PRS7	rs968476	15	29418745	G	T	3.41E-04	PRS-CSx
Colorectum	PRS7	rs968537	16	76445839	T	C	3.16E-04	PRS-CSx
Colorectum	PRS7	rs96894	11	57649243	G	A	2.48E-04	PRS-CSx
Colorectum	PRS7	rs969047	18	53962817	G	A	-4.02E-04	PRS-CSx
Colorectum	PRS7	rs969081	9	104591776	T	G	4.63E-04	PRS-CSx
Colorectum	PRS7	rs9695517	9	117439855	T	C	-7.60E-04	PRS-CSx
Colorectum	PRS7	rs9695897	9	140158650	T	C	4.28E-04	PRS-CSx
Colorectum	PRS7	rs9696384	9	140163033	A	G	6.07E-04	PRS-CSx

Colorectum	PRS7	rs970654	10	33399734	A	G	7.32E-04	PRS-CSx
Colorectum	PRS7	rs971212	5	40275694	C	T	3.64E-03	PRS-CSx
Colorectum	PRS7	rs971330	15	31751664	A	G	2.75E-04	PRS-CSx
Colorectum	PRS7	rs971370	5	112624158	T	C	2.12E-04	PRS-CSx
Colorectum	PRS7	rs971371	5	112624075	C	T	2.03E-04	PRS-CSx
Colorectum	PRS7	rs972345	4	115379817	T	C	1.68E-04	PRS-CSx
Colorectum	PRS7	rs972579	22	43387711	T	C	1.10E-04	PRS-CSx
Colorectum	PRS7	rs9727120	1	38379379	G	A	-1.10E-04	PRS-CSx
Colorectum	PRS7	rs9728268	1	38364089	C	T	-1.44E-04	PRS-CSx
Colorectum	PRS7	rs972936	12	102824921	T	C	4.86E-04	PRS-CSx
Colorectum	PRS7	rs9729851	1	38370259	C	T	1.33E-04	PRS-CSx
Colorectum	PRS7	rs973507	2	108950420	T	C	-6.73E-04	PRS-CSx
Colorectum	PRS7	rs9735635	11	61490880	A	C	2.18E-04	PRS-CSx
Colorectum	PRS7	rs974000	3	122485668	T	C	-2.71E-04	PRS-CSx
Colorectum	PRS7	rs974702	3	113633959	C	T	-1.62E-04	PRS-CSx
Colorectum	PRS7	rs975886	5	52101227	T	C	-9.61E-04	PRS-CSx
Colorectum	PRS7	rs976683	3	173485371	T	C	3.22E-04	PRS-CSx
Colorectum	PRS7	rs977253	2	67222446	G	A	1.31E-03	PRS-CSx
Colorectum	PRS7	rs977321	14	80002534	A	G	-8.13E-04	PRS-CSx
Colorectum	PRS7	rs977547	3	151471137	G	T	8.68E-04	PRS-CSx
Colorectum	PRS7	rs977667	15	47576655	A	G	-1.65E-04	PRS-CSx
Colorectum	PRS7	rs977987	16	75506593	G	A	3.65E-04	PRS-CSx
Colorectum	PRS7	rs977997	5	112616203	C	T	2.88E-04	PRS-CSx
Colorectum	PRS7	rs978373	4	102154344	C	T	-2.45E-04	PRS-CSx
Colorectum	PRS7	rs978458	12	102802239	T	C	2.62E-04	PRS-CSx
Colorectum	PRS7	rs978507	20	7853151	T	C	-2.55E-03	PRS-CSx
Colorectum	PRS7	rs978683	8	128374117	A	G	8.38E-04	PRS-CSx
Colorectum	PRS7	rs9787126	1	156984101	G	A	-7.52E-04	PRS-CSx
Colorectum	PRS7	rs9787692	10	8645163	G	T	5.62E-05	PRS-CSx
Colorectum	PRS7	rs9788566	14	29650365	G	A	1.32E-03	PRS-CSx
Colorectum	PRS7	rs9789420	2	159775621	T	C	-3.73E-05	PRS-CSx
Colorectum	PRS7	rs979012	20	6623374	T	C	4.50E-04	PRS-CSx
Colorectum	PRS7	rs9790655	4	89992347	T	C	3.32E-04	PRS-CSx
Colorectum	PRS7	rs979534	10	8796596	C	T	3.70E-04	PRS-CSx
Colorectum	PRS7	rs979614	6	20662123	G	A	5.81E-04	PRS-CSx
Colorectum	PRS7	rs9796292	13	114740938	A	G	4.21E-04	PRS-CSx
Colorectum	PRS7	rs979867	8	117791502	A	G	2.07E-03	PRS-CSx
Colorectum	PRS7	rs980031	3	96140650	A	G	-1.88E-03	PRS-CSx
Colorectum	PRS7	rs9802576	9	140144649	A	G	5.36E-04	PRS-CSx
Colorectum	PRS7	rs980394	1	231779507	G	A	-2.79E-04	PRS-CSx
Colorectum	PRS7	rs9804135	1	223832277	G	A	-3.78E-04	PRS-CSx
Colorectum	PRS7	rs9804633	11	7132515	C	T	-3.42E-04	PRS-CSx
Colorectum	PRS7	rs9806369	15	102008430	A	C	5.26E-04	PRS-CSx
Colorectum	PRS7	rs9806371	15	51511771	A	G	2.46E-04	PRS-CSx
Colorectum	PRS7	rs9806826	16	1894912	T	C	-9.97E-05	PRS-CSx
Colorectum	PRS7	rs980793	5	39625080	G	A	1.36E-04	PRS-CSx
Colorectum	PRS7	rs9808685	21	34785672	G	T	3.24E-05	PRS-CSx
Colorectum	PRS7	rs9808921	3	133797032	A	G	1.61E-04	PRS-CSx
Colorectum	PRS7	rs9810649	3	41476926	C	T	-2.39E-04	PRS-CSx
Colorectum	PRS7	rs9812025	3	41480879	A	G	-4.29E-04	PRS-CSx
Colorectum	PRS7	rs9812338	3	64611246	A	G	6.99E-04	PRS-CSx
Colorectum	PRS7	rs9813864	3	119590974	T	C	1.31E-04	PRS-CSx
Colorectum	PRS7	rs9814144	3	152746223	C	A	-6.38E-04	PRS-CSx
Colorectum	PRS7	rs9814412	3	186383180	T	C	-8.17E-04	PRS-CSx
Colorectum	PRS7	rs9814976	3	41215796	T	C	-2.07E-04	PRS-CSx
Colorectum	PRS7	rs9815735	3	41216266	A	C	-3.29E-04	PRS-CSx

Colorectum	PRS7	rs9816777	3	173206253	A	G	1.10E-03	PRS-CSx
Colorectum	PRS7	rs98175	22	44975266	T	C	6.49E-04	PRS-CSx
Colorectum	PRS7	rs981794	14	38040755	G	A	-5.37E-04	PRS-CSx
Colorectum	PRS7	rs9818626	3	133809258	C	A	-3.51E-04	PRS-CSx
Colorectum	PRS7	rs9818714	3	133957279	T	C	-2.64E-04	PRS-CSx
Colorectum	PRS7	rs9820182	3	134003345	G	T	-1.32E-04	PRS-CSx
Colorectum	PRS7	rs9820386	3	113095312	T	C	1.63E-04	PRS-CSx
Colorectum	PRS7	rs982042	17	52687500	A	C	-4.19E-04	PRS-CSx
Colorectum	PRS7	rs9820765	3	41312055	A	G	-1.92E-04	PRS-CSx
Colorectum	PRS7	rs982145	7	37906589	T	C	-5.09E-04	PRS-CSx
Colorectum	PRS7	rs9821957	3	173858380	A	G	4.99E-04	PRS-CSx
Colorectum	PRS7	rs9822061	3	123109494	T	C	9.72E-04	PRS-CSx
Colorectum	PRS7	rs9822076	3	133882270	A	G	2.66E-04	PRS-CSx
Colorectum	PRS7	rs9822464	3	14916048	C	T	-3.76E-04	PRS-CSx
Colorectum	PRS7	rs9824172	3	16974123	T	G	-2.52E-04	PRS-CSx
Colorectum	PRS7	rs9824780	3	151594869	C	T	7.68E-05	PRS-CSx
Colorectum	PRS7	rs9825724	3	168573410	A	G	-3.53E-04	PRS-CSx
Colorectum	PRS7	rs9826691	3	151486525	T	C	1.31E-03	PRS-CSx
Colorectum	PRS7	rs9827105	3	133822891	A	G	-2.47E-04	PRS-CSx
Colorectum	PRS7	rs982752	15	72145937	C	T	-3.77E-04	PRS-CSx
Colorectum	PRS7	rs9828860	3	173553737	T	C	2.88E-04	PRS-CSx
Colorectum	PRS7	rs9829309	3	173553783	T	G	1.67E-04	PRS-CSx
Colorectum	PRS7	rs9829651	3	142849107	A	G	3.71E-04	PRS-CSx
Colorectum	PRS7	rs9830510	3	173494302	A	G	5.82E-04	PRS-CSx
Colorectum	PRS7	rs9830798	3	18866539	T	C	1.83E-03	PRS-CSx
Colorectum	PRS7	rs983111	1	67539356	T	C	6.06E-05	PRS-CSx
Colorectum	PRS7	rs9831167	3	112919344	A	G	1.91E-03	PRS-CSx
Colorectum	PRS7	rs9832057	3	64627448	T	C	-2.22E-04	PRS-CSx
Colorectum	PRS7	rs983444	2	167398463	A	G	-1.14E-03	PRS-CSx
Colorectum	PRS7	rs9834727	3	133747404	A	G	-7.33E-03	PRS-CSx
Colorectum	PRS7	rs983589	11	83358400	A	G	9.77E-05	PRS-CSx
Colorectum	PRS7	rs983590	11	83358629	T	C	2.15E-04	PRS-CSx
Colorectum	PRS7	rs9835916	3	32343840	C	T	-9.37E-04	PRS-CSx
Colorectum	PRS7	rs9837076	3	112832851	T	C	4.62E-04	PRS-CSx
Colorectum	PRS7	rs9837200	3	64621249	A	C	3.76E-04	PRS-CSx
Colorectum	PRS7	rs9838262	3	64615873	A	G	3.06E-04	PRS-CSx
Colorectum	PRS7	rs983894	7	93910179	A	G	1.09E-03	PRS-CSx
Colorectum	PRS7	rs9839054	3	151476514	G	A	8.03E-04	PRS-CSx
Colorectum	PRS7	rs9839819	3	133931135	A	C	-5.78E-04	PRS-CSx
Colorectum	PRS7	rs9840170	3	133827144	C	T	2.77E-04	PRS-CSx
Colorectum	PRS7	rs9841380	3	133735861	C	A	-1.20E-03	PRS-CSx
Colorectum	PRS7	rs9841945	3	157581467	T	C	4.10E-04	PRS-CSx
Colorectum	PRS7	rs9842173	3	112951608	A	G	-2.86E-04	PRS-CSx
Colorectum	PRS7	rs9842465	3	159359371	T	G	-9.93E-04	PRS-CSx
Colorectum	PRS7	rs9843415	3	41414528	G	A	-1.64E-04	PRS-CSx
Colorectum	PRS7	rs9843443	3	117392403	C	T	1.06E-03	PRS-CSx
Colorectum	PRS7	rs984399	5	88381894	G	A	4.54E-04	PRS-CSx
Colorectum	PRS7	rs9844111	3	113057481	G	A	1.89E-04	PRS-CSx
Colorectum	PRS7	rs9844263	3	157548790	G	A	-5.14E-04	PRS-CSx
Colorectum	PRS7	rs984578	2	66329532	G	A	-5.27E-04	PRS-CSx
Colorectum	PRS7	rs9846057	3	21826522	A	C	1.00E-03	PRS-CSx
Colorectum	PRS7	rs9846871	3	133852998	G	T	2.74E-04	PRS-CSx
Colorectum	PRS7	rs9846898	3	133797007	G	A	2.07E-04	PRS-CSx
Colorectum	PRS7	rs9847710	3	53062661	T	C	1.67E-03	PRS-CSx
Colorectum	PRS7	rs9848415	3	45828841	A	G	1.32E-03	PRS-CSx
Colorectum	PRS7	rs984926	5	57320343	G	A	2.34E-04	PRS-CSx

Colorectum	PRS7	rs9849546	3	66496592	T	C	1.22E-05	PRS-CSx
Colorectum	PRS7	rs9850653	3	41292736	T	C	2.49E-04	PRS-CSx
Colorectum	PRS7	rs9851577	3	125908310	T	C	-7.42E-05	PRS-CSx
Colorectum	PRS7	rs9852240	3	152427651	G	A	9.73E-05	PRS-CSx
Colorectum	PRS7	rs9852385	3	41458709	T	C	-3.91E-04	PRS-CSx
Colorectum	PRS7	rs9852703	3	113189368	G	A	-2.70E-05	PRS-CSx
Colorectum	PRS7	rs9853452	3	122545494	G	A	-2.93E-04	PRS-CSx
Colorectum	PRS7	rs9854256	3	157340576	C	T	4.29E-04	PRS-CSx
Colorectum	PRS7	rs9855708	3	62620763	C	T	-2.84E-03	PRS-CSx
Colorectum	PRS7	rs9856266	3	45605401	T	C	2.19E-03	PRS-CSx
Colorectum	PRS7	rs9856298	3	133957265	G	A	-6.81E-04	PRS-CSx
Colorectum	PRS7	rs9856301	3	55992015	T	C	1.12E-03	PRS-CSx
Colorectum	PRS7	rs9856724	3	38315626	C	T	1.11E-03	PRS-CSx
Colorectum	PRS7	rs9857718	3	157346370	G	T	4.37E-04	PRS-CSx
Colorectum	PRS7	rs9858450	3	133906086	C	T	-5.23E-04	PRS-CSx
Colorectum	PRS7	rs985849	15	76261993	C	T	-3.55E-04	PRS-CSx
Colorectum	PRS7	rs9859674	3	18882582	T	C	7.30E-04	PRS-CSx
Colorectum	PRS7	rs9860060	3	133845256	A	C	2.22E-04	PRS-CSx
Colorectum	PRS7	rs9860520	3	106416700	A	G	-1.02E-03	PRS-CSx
Colorectum	PRS7	rs9861429	3	128425749	T	C	8.46E-04	PRS-CSx
Colorectum	PRS7	rs9862440	3	113609403	A	G	-4.22E-04	PRS-CSx
Colorectum	PRS7	rs9862599	3	104864331	G	T	8.41E-04	PRS-CSx
Colorectum	PRS7	rs9864196	3	157582078	G	A	-2.55E-04	PRS-CSx
Colorectum	PRS7	rs986617	1	168808752	G	A	-8.85E-04	PRS-CSx
Colorectum	PRS7	rs9866906	3	73405263	A	C	-8.52E-04	PRS-CSx
Colorectum	PRS7	rs9868151	3	73751432	C	A	-2.41E-04	PRS-CSx
Colorectum	PRS7	rs9868353	3	173494682	G	A	3.47E-04	PRS-CSx
Colorectum	PRS7	rs987087	8	118472329	C	T	-2.62E-03	PRS-CSx
Colorectum	PRS7	rs9872164	3	64615559	G	A	2.05E-04	PRS-CSx
Colorectum	PRS7	rs9872216	3	41458638	C	T	-7.40E-04	PRS-CSx
Colorectum	PRS7	rs9873477	3	119645976	A	G	1.73E-04	PRS-CSx
Colorectum	PRS7	rs9873621	3	143357546	A	G	-7.96E-04	PRS-CSx
Colorectum	PRS7	rs9874225	3	173512698	A	G	2.89E-04	PRS-CSx
Colorectum	PRS7	rs9874938	3	74548437	G	A	9.73E-04	PRS-CSx
Colorectum	PRS7	rs9876713	3	173501227	G	A	3.36E-04	PRS-CSx
Colorectum	PRS7	rs9877854	3	119245747	G	T	-2.57E-04	PRS-CSx
Colorectum	PRS7	rs9878131	3	167082989	A	G	1.44E-03	PRS-CSx
Colorectum	PRS7	rs9878394	3	90264056	T	C	9.06E-04	PRS-CSx
Colorectum	PRS7	rs9878473	3	119650788	T	C	8.88E-05	PRS-CSx
Colorectum	PRS7	rs9878812	3	173543451	T	C	1.74E-04	PRS-CSx
Colorectum	PRS7	rs9879266	3	173489454	T	C	3.55E-04	PRS-CSx
Colorectum	PRS7	rs9879481	3	144721908	A	G	5.77E-04	PRS-CSx
Colorectum	PRS7	rs9879792	3	159359429	C	A	-1.33E-03	PRS-CSx
Colorectum	PRS7	rs9881252	3	41479521	T	C	-2.28E-04	PRS-CSx
Colorectum	PRS7	rs9883242	3	169430638	A	G	1.79E-04	PRS-CSx
Colorectum	PRS7	rs9883253	3	184415609	G	A	-5.98E-04	PRS-CSx
Colorectum	PRS7	rs9884405	4	134943881	C	T	7.27E-04	PRS-CSx
Colorectum	PRS7	rs9888701	15	32994843	T	C	-3.51E-05	PRS-CSx
Colorectum	PRS7	rs9890655	17	72033283	G	A	-9.00E-04	PRS-CSx
Colorectum	PRS7	rs9890666	17	52662224	A	G	-2.77E-04	PRS-CSx
Colorectum	PRS7	rs9891526	17	75695351	A	G	-1.00E-03	PRS-CSx
Colorectum	PRS7	rs9891783	17	8516435	G	A	1.35E-03	PRS-CSx
Colorectum	PRS7	rs9892738	17	63081860	C	T	1.24E-03	PRS-CSx
Colorectum	PRS7	rs9892880	17	784636	T	C	-8.96E-05	PRS-CSx
Colorectum	PRS7	rs9892894	17	12815193	T	G	5.01E-04	PRS-CSx
Colorectum	PRS7	rs9893593	17	57352900	T	C	2.33E-04	PRS-CSx

Colorectum	PRS7	rs989365	5	143614503	A	G	4.34E-05	PRS-CSx
Colorectum	PRS7	rs9893674	17	72160724	G	A	-6.75E-04	PRS-CSx
Colorectum	PRS7	rs9894103	17	75695409	G	A	-1.31E-03	PRS-CSx
Colorectum	PRS7	rs989496	6	18938869	T	C	1.93E-03	PRS-CSx
Colorectum	PRS7	rs9895780	17	69627509	A	C	3.79E-04	PRS-CSx
Colorectum	PRS7	rs9897453	17	78634300	G	A	-5.10E-04	PRS-CSx
Colorectum	PRS7	rs9897769	17	81060040	A	G	3.95E-04	PRS-CSx
Colorectum	PRS7	rs9898431	17	4945810	A	C	4.01E-04	PRS-CSx
Colorectum	PRS7	rs9898977	17	36617210	G	A	-3.95E-04	PRS-CSx
Colorectum	PRS7	rs9899355	17	17898243	C	T	-7.00E-04	PRS-CSx
Colorectum	PRS7	rs9899714	17	70094638	G	A	-7.05E-04	PRS-CSx
Colorectum	PRS7	rs9900326	17	10708208	C	T	-1.20E-03	PRS-CSx
Colorectum	PRS7	rs9901206	17	52681292	A	G	-3.20E-04	PRS-CSx
Colorectum	PRS7	rs9901757	17	80963331	T	C	-1.74E-04	PRS-CSx
Colorectum	PRS7	rs9902411	17	52706189	G	A	-1.61E-04	PRS-CSx
Colorectum	PRS7	rs990255	2	67279175	C	A	-1.96E-03	PRS-CSx
Colorectum	PRS7	rs9903227	17	814418	C	T	-6.55E-04	PRS-CSx
Colorectum	PRS7	rs9903344	17	69593238	C	A	3.17E-04	PRS-CSx
Colorectum	PRS7	rs990395	13	102558932	G	A	-4.11E-04	PRS-CSx
Colorectum	PRS7	rs9906155	17	75701697	C	T	-1.72E-03	PRS-CSx
Colorectum	PRS7	rs9907714	17	54112874	C	T	7.58E-04	PRS-CSx
Colorectum	PRS7	rs9908279	17	57280100	A	G	4.64E-05	PRS-CSx
Colorectum	PRS7	rs9909030	17	75718552	A	G	-1.80E-03	PRS-CSx
Colorectum	PRS7	rs9909219	17	4943365	T	C	3.90E-04	PRS-CSx
Colorectum	PRS7	rs9910153	17	70421289	A	G	1.82E-03	PRS-CSx
Colorectum	PRS7	rs9911203	17	12810497	C	T	5.68E-04	PRS-CSx
Colorectum	PRS7	rs9912593	17	5733354	T	C	2.07E-04	PRS-CSx
Colorectum	PRS7	rs9914087	17	4831790	G	A	6.63E-04	PRS-CSx
Colorectum	PRS7	rs991500	2	66330655	A	G	-6.03E-04	PRS-CSx
Colorectum	PRS7	rs991529	4	151495331	G	A	4.15E-04	PRS-CSx
Colorectum	PRS7	rs9915671	17	12823875	A	G	5.83E-04	PRS-CSx
Colorectum	PRS7	rs991817	12	111410537	G	A	4.34E-04	PRS-CSx
Colorectum	PRS7	rs991821	7	25773404	A	G	-5.17E-04	PRS-CSx
Colorectum	PRS7	rs9918266	5	18854429	G	A	1.33E-03	PRS-CSx
Colorectum	PRS7	rs9918832	8	32282597	T	C	-4.47E-04	PRS-CSx
Colorectum	PRS7	rs9920028	15	75927717	A	G	4.74E-04	PRS-CSx
Colorectum	PRS7	rs992021	4	167421242	T	C	6.53E-04	PRS-CSx
Colorectum	PRS7	rs992022	4	167421170	T	C	1.57E-03	PRS-CSx
Colorectum	PRS7	rs9921222	16	375782	T	C	-3.95E-04	PRS-CSx
Colorectum	PRS7	rs9923175	16	11232547	T	G	-5.79E-04	PRS-CSx
Colorectum	PRS7	rs9923231	16	31107689	C	T	2.11E-04	PRS-CSx
Colorectum	PRS7	rs9923667	16	12672518	C	T	1.30E-04	PRS-CSx
Colorectum	PRS7	rs9924628	16	82306551	A	C	-2.16E-03	PRS-CSx
Colorectum	PRS7	rs9925072	16	26883057	G	A	4.46E-04	PRS-CSx
Colorectum	PRS7	rs9925923	16	68819614	T	C	-1.49E-04	PRS-CSx
Colorectum	PRS7	rs9926033	16	80111981	C	T	3.98E-04	PRS-CSx
Colorectum	PRS7	rs9927565	16	49898706	C	T	6.06E-04	PRS-CSx
Colorectum	PRS7	rs9928184	16	8051013	T	C	-2.30E-04	PRS-CSx
Colorectum	PRS7	rs9929218	16	68820946	A	G	-3.10E-04	PRS-CSx
Colorectum	PRS7	rs9931509	16	88744756	A	C	-1.84E-03	PRS-CSx
Colorectum	PRS7	rs9932240	16	50094227	C	T	-2.70E-04	PRS-CSx
Colorectum	PRS7	rs9933359	16	86201589	T	C	-2.80E-04	PRS-CSx
Colorectum	PRS7	rs9934438	16	31104878	G	A	2.17E-04	PRS-CSx
Colorectum	PRS7	rs9934572	16	69058613	G	A	-2.41E-04	PRS-CSx
Colorectum	PRS7	rs993460	4	115431677	A	C	2.14E-04	PRS-CSx
Colorectum	PRS7	rs993483	3	140125714	T	G	1.40E-03	PRS-CSx

Colorectum	PRS7	rs993558	3	157376463	A	G	4.20E-04	PRS-CSx
Colorectum	PRS7	rs9936329	16	31140799	G	T	3.61E-04	PRS-CSx
Colorectum	PRS7	rs9936567	16	71374298	G	A	-1.97E-03	PRS-CSx
Colorectum	PRS7	rs9936884	16	72960230	A	G	-1.62E-03	PRS-CSx
Colorectum	PRS7	rs9936966	16	50090366	T	G	-6.26E-04	PRS-CSx
Colorectum	PRS7	rs9937509	16	53100136	G	A	8.23E-04	PRS-CSx
Colorectum	PRS7	rs9937844	16	80059171	T	C	1.91E-04	PRS-CSx
Colorectum	PRS7	rs9938025	16	72022041	A	G	-4.73E-04	PRS-CSx
Colorectum	PRS7	rs9939417	16	31053467	C	T	2.48E-04	PRS-CSx
Colorectum	PRS7	rs9940076	16	87687614	T	C	7.66E-04	PRS-CSx
Colorectum	PRS7	rs9940390	16	80051931	C	T	4.80E-04	PRS-CSx
Colorectum	PRS7	rs9943111	1	182937401	G	A	1.64E-04	PRS-CSx
Colorectum	PRS7	rs994321	6	29372356	A	G	-2.61E-04	PRS-CSx
Colorectum	PRS7	rs9944037	14	76903733	A	C	6.22E-04	PRS-CSx
Colorectum	PRS7	rs9944229	15	85404969	G	A	-1.35E-03	PRS-CSx
Colorectum	PRS7	rs9944392	17	57341195	C	T	1.67E-04	PRS-CSx
Colorectum	PRS7	rs9944794	18	42004083	T	C	3.10E-04	PRS-CSx
Colorectum	PRS7	rs9945359	18	42004468	A	G	2.98E-04	PRS-CSx
Colorectum	PRS7	rs9947269	18	42008393	C	T	2.69E-04	PRS-CSx
Colorectum	PRS7	rs9948636	18	65020549	A	G	-1.46E-03	PRS-CSx
Colorectum	PRS7	rs994960	3	73731916	G	A	-1.63E-04	PRS-CSx
Colorectum	PRS7	rs9949616	18	42051265	C	A	3.20E-04	PRS-CSx
Colorectum	PRS7	rs9951264	18	42037678	G	T	2.96E-04	PRS-CSx
Colorectum	PRS7	rs9952364	18	6776786	C	T	-6.21E-04	PRS-CSx
Colorectum	PRS7	rs9957037	18	42048214	C	A	2.23E-04	PRS-CSx
Colorectum	PRS7	rs9957453	18	74366206	C	A	-1.50E-03	PRS-CSx
Colorectum	PRS7	rs995805	1	194707591	A	G	-8.87E-04	PRS-CSx
Colorectum	PRS7	rs9959061	18	12870038	T	C	-1.04E-03	PRS-CSx
Colorectum	PRS7	rs9963592	18	41984948	T	G	1.09E-04	PRS-CSx
Colorectum	PRS7	rs9964321	18	6776881	A	C	-9.17E-04	PRS-CSx
Colorectum	PRS7	rs9964359	18	45627633	T	G	6.05E-04	PRS-CSx
Colorectum	PRS7	rs9964538	18	4354518	C	T	8.11E-04	PRS-CSx
Colorectum	PRS7	rs9965539	18	41973779	A	G	3.89E-04	PRS-CSx
Colorectum	PRS7	rs9967648	19	22516884	T	C	4.41E-03	PRS-CSx
Colorectum	PRS7	rs996804	4	86907467	G	A	-3.85E-04	PRS-CSx
Colorectum	PRS7	rs996848	12	106370034	G	A	1.15E-03	PRS-CSx
Colorectum	PRS7	rs9971546	11	64896732	C	T	-5.33E-04	PRS-CSx
Colorectum	PRS7	rs997187	5	35959184	A	G	-1.17E-04	PRS-CSx
Colorectum	PRS7	rs9972246	14	34142565	T	C	-3.45E-04	PRS-CSx
Colorectum	PRS7	rs9973057	18	42824927	G	A	1.47E-03	PRS-CSx
Colorectum	PRS7	rs9974320	21	47569141	A	G	6.11E-04	PRS-CSx
Colorectum	PRS7	rs9976577	21	17536183	C	T	-1.14E-03	PRS-CSx
Colorectum	PRS7	rs9978658	21	48027084	T	G	1.14E-04	PRS-CSx
Colorectum	PRS7	rs9979500	21	48029698	A	C	1.28E-04	PRS-CSx
Colorectum	PRS7	rs9979962	21	47968307	G	A	2.73E-05	PRS-CSx
Colorectum	PRS7	rs998076	5	150924769	A	G	-1.21E-03	PRS-CSx
Colorectum	PRS7	rs9982863	21	48030465	A	G	9.59E-05	PRS-CSx
Colorectum	PRS7	rs9985105	21	17536247	T	C	-1.48E-03	PRS-CSx
Colorectum	PRS7	rs9986145	5	134453390	A	G	3.47E-03	PRS-CSx
Colorectum	PRS7	rs9986241	5	88374038	C	A	2.34E-04	PRS-CSx
Colorectum	PRS7	rs9986382	6	26550619	T	C	4.01E-05	PRS-CSx
Colorectum	PRS7	rs9987580	9	33679273	C	T	-2.41E-04	PRS-CSx
Colorectum	PRS7	rs998896	2	159787793	G	A	-5.91E-05	PRS-CSx
Colorectum	PRS7	rs998915	20	47387699	A	C	-1.56E-04	PRS-CSx
Colorectum	PRS7	rs9989786	2	95967746	A	G	-1.07E-04	PRS-CSx
Colorectum	PRS7	rs9990941	4	86839953	A	G	-5.13E-03	PRS-CSx

Colorectum	PRS7	rs9991568	4	18922645	C	T	-2.84E-04	PRS-CSx
Colorectum	PRS7	rs999367	3	112980483	G	A	-2.76E-04	PRS-CSx
Colorectum	PRS7	rs999480	15	51587860	T	C	2.84E-04	PRS-CSx
Colorectum	PRS7	rs9996560	4	95442692	T	C	9.40E-04	PRS-CSx
Colorectum	PRS7	rs9998015	4	105816898	C	T	-6.94E-04	PRS-CSx
Colorectum	PRS7	rs9999828	4	151534545	A	G	6.74E-04	PRS-CSx
Pancreas	PRS5	rs13303010	1	894573	G	A	0.231	32321713
Pancreas	PRS5	rs10919791	1	199965168	G	A	0.261	32321713
Pancreas	PRS5	rs2816938	1	199985368	A	T	0.191	32321713
Pancreas	PRS5	rs1486134	2	67639769	G	T	0.131	32321713
Pancreas	PRS5	rs9854771	3	189508471	G	A	0.117	32321713
Pancreas	PRS5	rs2736098	5	1294086	C	T	0.174	32321713
Pancreas	PRS5	rs31490	5	1344458	A	G	0.182	32321713
Pancreas	PRS5	rs78417682	7	47488903	G	C	0.186	32321713
Pancreas	PRS5	rs17688601	7	40866663	C	A	0.128	32321713
Pancreas	PRS5	rs6971499	7	130680521	T	C	0.211	32321713
Pancreas	PRS5	rs2941471	8	76470404	A	G	0.117	32321713
Pancreas	PRS5	rs10094872	8	128719884	T	A	0.131	32321713
Pancreas	PRS5	rs1561927	8	129568078	T	C	0.117	32321713
Pancreas	PRS5	rs687289	9	136137106	A	T/C	0.239	32321713
Pancreas	PRS5	rs9581943	13	28493997	A	G	0.140	32321713
Pancreas	PRS5	rs9543325	13	73916628	C	T	0.215	32321713
Pancreas	PRS5	rs7190458	16	75263661	A	G	0.308	32321713
Pancreas	PRS5	rs4795218	17	36078510	G	A	0.128	32321713
Pancreas	PRS5	rs11655237	17	70400166	T	C	0.231	32321713
Pancreas	PRS5	rs1517037	18	56878274	C	T	0.151	32321713
Pancreas	PRS5	rs16986825	22	29300306	T	C	0.140	32321713
Lung	PRS4	rs1885281	10	114492898	A	G	-0.140	31326317
Lung	PRS4	rs11216827	11	118110202	A	T	0.120	31326317
Lung	PRS4	rs72845278	17	65877076	T	C	0.143	31326317
Lung	PRS4	rs2293607	3	169482335	T	C	0.117	31326317
Lung	PRS4	rs36108040	3	189335844	A	G	0.190	31326317
Lung	PRS4	rs13156167	5	1275857	T	C	-0.206	31326317
Lung	PRS4	rs35029535	5	1284976	T	C	-0.261	31326317
Lung	PRS4	rs7705526	5	1285974	A	C	0.268	31326317
Lung	PRS4	rs2736108	5	1297488	T	C	0.166	31326317
Lung	PRS4	rs183257831	5	1307642	A	G	-0.148	31326317
Lung	PRS4	rs401681	5	1322087	T	C	-0.145	31326317
Lung	PRS4	rs2455393	5	1333231	T	G	-0.152	31326317
Lung	PRS4	rs2050188	6	32339897	T	C	0.112	31326317
Lung	PRS4	rs5020945	6	32450134	T	C	-0.122	31326317
Lung	PRS4	rs71536538	6	32523884	T	C	-0.138	31326317
Lung	PRS4	rs9273129	6	32612774	A	T	0.149	31326317
Lung	PRS4	rs2647082	6	32628146	A	G	0.131	31326317
Lung	PRS4	rs2496644	6	41482745	A	C	0.151	31326317
Lung	PRS4	rs13252431	8	32426884	A	G	0.171	31326317
Lung	PRS4	rs10429489	9	21787521	A	G	0.104	31326317
Lung	PRS4	rs11610143	12	52349071	C	G	0.068	31326317
Lung	PRS4	rs1200399	14	35293185	C	T	0.104	31326317
Lung	PRS4	rs17038564	2	65496058	G	A	0.104	31326317
Lung	PRS4	rs17728461	22	30598552	G	C	0.058	31326317
Lung	PRS4	rs2517873	6	29875992	A	G	0.182	31326317
Lung	PRS4	rs2895680	5	146644115	C	T	0.049	31326317
Lung	PRS4	rs3094604	6	31434111	A	G	0.077	31326317
Lung	PRS4	rs35201538	9	33422488	C	CT	0.095	31326317
Lung	PRS4	rs3769821	2	202123430	C	T	0.058	31326317

Lung	PRS4	rs4573350	9	124955115	T	C	0.086	31326317
Lung	PRS4	rs4809957	20	52771171	A	G	0.049	31326317
Lung	PRS4	rs55781567	15	78857986	G	C	0.122	31326317
Lung	PRS4	rs5879422	6	117784658	T	TTG	0.086	31326317
Lung	PRS4	rs6920364	6	167376466	C	G	0.039	31326317
Lung	PRS4	rs72658409	9	22160087	C	T	0.095	31326317
Lung	PRS4	rs75295329	12	111344621	T	G	0.113	31326317
Lung	PRS4	rs753955	13	24293859	G	A	0.058	31326317
Lung	PRS4	rs77468143	15	49376624	T	G	0.077	31326317
Lung	PRS4	rs938682	15	78896547	A	G	0.077	31326317
Lung	PRS4	rs4619206	12	1002857	C	T	0.086	34594039
Lung	PRS4	rs78860372	6	30380366	T	A	0.150	34594039
Lung	PRS4	rs11878604	19	41333284	C	T	-0.147	34594039
Breast	PRS4	rs616488	1	10566215	A	G	0.058	32139696
Breast	PRS4	rs72906468	1	17772093	A	T	0.058	32139696
Breast	PRS4	rs2992756	1	18807339	T	C	0.039	32139696
Breast	PRS4	rs3790585	1	46023356	A	T	0.049	32139696
Breast	PRS4	rs12118297	1	87779217	T	G	-0.051	32139696
Breast	PRS4	rs11249433	1	121280613	A	G	-0.117	32139696
Breast	PRS4	rs2758598	1	156194339	A	G	0.068	32139696
Breast	PRS4	rs4951011	1	203766331	G	A	0.049	32139696
Breast	PRS4	rs12710696	2	19320803	T	C	0.049	32139696
Breast	PRS4	rs6756513	2	70172587	A	G	-0.041	32139696
Breast	PRS4	rs4849887	2	121245122	T	C	-0.083	32139696
Breast	PRS4	rs1830298	2	202181247	T	C	-0.094	32139696
Breast	PRS4	rs4442975	2	217920769	T	G	-0.062	32139696
Breast	PRS4	rs16857609	2	218296508	T	C	0.068	32139696
Breast	PRS4	rs4973768	3	27416013	T	C	0.077	32139696
Breast	PRS4	rs12493607	3	30682939	C	G	0.049	32139696
Breast	PRS4	rs6796502	3	46866866	A	G	-0.051	32139696
Breast	PRS4	rs73006998	3	150464271	A	G	-0.083	32139696
Breast	PRS4	rs11281251	3	156519412	T	TTGTGAC	-0.062	32139696
Breast	PRS4	rs58058861	3	172285237	A	G	0.039	32139696
Breast	PRS4	rs11944638	4	48227719	T	C	0.077	32139696
Breast	PRS4	rs11947923	4	53911337	T	C	-0.041	32139696
Breast	PRS4	rs10022462	4	89243818	T	C	0.113	32139696
Breast	PRS4	rs6828523	4	175846426	A	C	-0.062	32139696
Breast	PRS4	rs10069690	5	1279790	T	C	0.077	32139696
Breast	PRS4	rs3215401	5	1296255	A	AG	0.049	32139696
Breast	PRS4	rs6555134	5	2776483	T	C	-0.051	32139696
Breast	PRS4	rs10941679	5	44706498	A	G	-0.094	32139696
Breast	PRS4	rs62355902	5	56053723	A	T	-0.051	32139696
Breast	PRS4	rs10474352	5	90732225	C	T	0.068	32139696
Breast	PRS4	rs6882649	5	111217786	T	G	0.039	32139696
Breast	PRS4	rs1432679	5	158244083	T	C	-0.073	32139696
Breast	PRS4	rs204247	6	13722523	A	G	-0.030	32139696
Breast	PRS4	rs7765429	6	21904169	T	C	-0.062	32139696
Breast	PRS4	rs12207986	6	81094287	A	G	0.030	32139696
Breast	PRS4	rs7768862	6	85088846	A	T	-0.051	32139696
Breast	PRS4	rs9485372	6	149608874	G	A	0.077	32139696
Breast	PRS4	rs2046210	6	151948366	A	G	0.207	32139696
Breast	PRS4	rs2747652	6	152437016	T	C	-0.041	32139696
Breast	PRS4	rs6940159	6	170332621	T	C	-0.062	32139696
Breast	PRS4	rs6964587	7	91630620	T	G	0.049	32139696
Breast	PRS4	rs17268829	7	94113799	T	C	-0.051	32139696
Breast	PRS4	rs4593472	7	130667121	T	C	-0.051	32139696

Breast	PRS4	rs11977670	7	139942304	A	G	0.049	32139696
Breast	PRS4	rs66823261	8	170692	T	C	-0.051	32139696
Breast	PRS4	rs144145984	8	23644003	CT	C	-0.041	32139696
Breast	PRS4	rs9693444	8	29509616	A	C	0.068	32139696
Breast	PRS4	rs13365225	8	36858483	A	G	0.058	32139696
Breast	PRS4	rs6472903	8	76230301	T	G	0.131	32139696
Breast	PRS4	rs2849506	8	101329134	C	G	-0.041	32139696
Breast	PRS4	rs514192	8	102478959	A	T	0.049	32139696
Breast	PRS4	rs12546444	8	106358620	A	T	0.058	32139696
Breast	PRS4	rs13267382	8	117209548	A	G	0.030	32139696
Breast	PRS4	rs142360995	8	118205719	A	G	0.122	32139696
Breast	PRS4	rs17350191	8	124757661	T	C	0.049	32139696
Breast	PRS4	rs13281615	8	128355618	A	G	-0.041	32139696
Breast	PRS4	rs10820600	9	106856692	T	C	-0.051	32139696
Breast	PRS4	rs10759243	9	110306115	A	C	0.049	32139696
Breast	PRS4	rs10816625	9	110837073	A	G	-0.094	32139696
Breast	PRS4	rs10760444	9	129396434	A	G	-0.073	32139696
Breast	PRS4	rs8176636	9	136151579	T	TGGTGCAGGCGCAGGAAAAAATTGTGGCAATTCCTCA	0.039	32139696
Breast	PRS4	rs541079479	10	22861533	CA	C	0.058	32139696
Breast	PRS4	rs10822013	10	64251977	T	C	0.068	32139696
Breast	PRS4	rs704010	10	80841148	T	C	0.049	32139696
Breast	PRS4	rs2901157	10	119262365	A	G	0.058	32139696
Breast	PRS4	rs11199914	10	123093901	T	C	-0.030	32139696
Breast	PRS4	rs2981578	10	123340311	T	C	-0.186	32139696
Breast	PRS4	rs3817198	11	1909006	T	C	-0.062	32139696
Breast	PRS4	rs10838267	11	44368892	A	G	0.058	32139696
Breast	PRS4	rs7107217	11	129473690	C	A	0.058	32139696
Breast	PRS4	rs12422552	12	14413931	C	G	0.077	32139696
Breast	PRS4	rs7297051	12	28174817	T	C	-0.117	32139696
Breast	PRS4	rs78588049	12	69180907	A	ATTTT	-0.073	32139696
Breast	PRS4	rs17356907	12	96027759	A	G	0.049	32139696
Breast	PRS4	rs855596	12	103045519	T	C	-0.105	32139696
Breast	PRS4	rs1292011	12	115836522	A	G	0.095	32139696
Breast	PRS4	rs9316500	13	51094114	T	G	0.049	32139696
Breast	PRS4	rs2236007	14	37132769	A	G	-0.073	32139696
Breast	PRS4	rs75004998	14	77517786	A	G	-0.041	32139696
Breast	PRS4	rs8027365	15	75808740	A	C	0.049	32139696
Breast	PRS4	rs2290203	15	91512067	G	A	0.095	32139696
Breast	PRS4	rs4784227	16	52599188	T	C	0.199	32139696
Breast	PRS4	rs17817449	16	53813367	T	G	0.068	32139696
Breast	PRS4	rs11075995	16	53855291	A	T	0.049	32139696
Breast	PRS4	rs28539243	16	54682064	A	G	0.030	32139696
Breast	PRS4	rs2432539	16	56420987	A	G	0.049	32139696
Breast	PRS4	rs76535198	16	71892498	A	C	0.077	32139696
Breast	PRS4	rs13329835	16	80650805	A	G	-0.073	32139696
Breast	PRS4	rs4496150	16	87085237	A	C	-0.041	32139696
Breast	PRS4	rs2787486	17	53209774	A	C	0.077	32139696
Breast	PRS4	rs745570	17	77781725	A	G	0.030	32139696
Breast	PRS4	rs527616	18	24337424	C	G	-0.030	32139696
Breast	PRS4	rs1436904	18	24570667	T	G	0.030	32139696
Breast	PRS4	rs6507583	18	42399590	A	G	0.058	32139696
Breast	PRS4	rs2594714	19	13954571	A	G	-0.041	32139696
Breast	PRS4	rs4808801	19	18571141	A	G	0.058	32139696
Breast	PRS4	rs71338792	19	46183031	A	AT	-0.041	32139696
Breast	PRS4	rs6122906	20	48945911	A	G	-0.030	32139696

Breast	PRS4	rs12481286	20	52287610	T	G	0.049	32139696
Breast	PRS4	rs16992204	21	36111201	C	T	0.122	32139696
Breast	PRS4	rs35418111	21	47856670	A	G	0.068	32139696
Breast	PRS4	rs34331122	22	19762428	CTT	C	-0.062	32139696
Breast	PRS4	rs12628403	22	39358037	C	A	0.077	32139696
Breast	PRS4	rs6001930	22	40876234	T	C	-0.051	32139696
Breast	PRS4	rs112199989	6	33308380	A	G	0.136	34594039
Breast	PRS4	rs17743054	18	42900892	C	T	-0.093	34594039
Breast	PRS4	rs2464195	12	121435475	A	G	-0.045	34594039
Breast	PRS4	rs12578576	12	29274538	T	C	-0.083	34594039
Breast	PRS4	rs117515012	2	121179161	A	G	0.273	34594039
Cervix	PRS4	rs13117307	4	56751740	T	C	0.231	23817570
Cervix	PRS4	rs4282438	6	33072172	G	T	-0.288	23817570
Cervix	PRS4	rs9277952	6	33204274	A	G	-0.163	23817570
Cervix	PRS4	rs8067378	17	38051348	G	A	0.166	23817570
Cervix	PRS4	rs59661306	5	90383461	A	G	-0.261	30412241
Cervix	PRS4	rs7457728	7	54447962	C	G	0.174	30412241
Cervix	PRS4	rs586610	6	32572311	C	T	-0.141	34594039
Cervix	PRS4	rs1799964	6	31542308	C	T	-0.120	34594039
Cervix	PRS4	rs28584179	6	32626119	T	C	-0.436	34594039
Cervix	PRS4	rs805257	6	31634193	G	A	-0.141	34594039
Cervix	PRS4	rs2856437	6	32157364	A	G	0.193	34594039
Cervix	PRS4	rs3130169	6	33048102	T	C	0.359	34594039
Cervix	PRS4	rs558000791	6	31495508	A	AAG	-0.318	34594039
Cervix	PRS4	rs578039100	6	31377719	C	T	-0.328	34594039
Cervix	PRS4	rs2858884	6	32700083	C	A	0.328	34594039
Ovary	PRS7	rs10001607	4	56203199	T	C	2.29E-04	PRS-CSx
Ovary	PRS7	rs1000427	22	36890105	A	G	-1.19E-03	PRS-CSx
Ovary	PRS7	rs10009435	4	45524582	A	G	-1.45E-03	PRS-CSx
Ovary	PRS7	rs10010379	4	43982530	A	G	3.27E-04	PRS-CSx
Ovary	PRS7	rs1001301	9	16925586	G	A	9.28E-05	PRS-CSx
Ovary	PRS7	rs10013239	4	7680962	A	C	-4.56E-05	PRS-CSx
Ovary	PRS7	rs1001347	20	50538971	C	T	2.18E-04	PRS-CSx
Ovary	PRS7	rs10015551	4	113961039	T	G	-1.82E-04	PRS-CSx
Ovary	PRS7	rs1001938	6	45927857	A	C	-2.89E-04	PRS-CSx
Ovary	PRS7	rs1002175	15	63686327	G	A	2.62E-05	PRS-CSx
Ovary	PRS7	rs10021878	4	5868984	G	A	-9.25E-05	PRS-CSx
Ovary	PRS7	rs10028700	4	45488193	T	C	-1.17E-03	PRS-CSx
Ovary	PRS7	rs10030434	4	5859510	C	T	8.31E-05	PRS-CSx
Ovary	PRS7	rs10038212	5	63861495	C	T	-3.59E-05	PRS-CSx
Ovary	PRS7	rs1004046	19	30992639	C	A	7.42E-05	PRS-CSx
Ovary	PRS7	rs10050630	5	111807744	T	C	-3.00E-04	PRS-CSx
Ovary	PRS7	rs10053084	5	120328270	G	A	-5.23E-03	PRS-CSx
Ovary	PRS7	rs10058249	5	107323372	A	G	3.95E-04	PRS-CSx
Ovary	PRS7	rs10060831	5	111808908	C	T	-1.06E-04	PRS-CSx
Ovary	PRS7	rs10069382	5	31921265	C	T	1.75E-03	PRS-CSx
Ovary	PRS7	rs10069690	5	1279790	T	C	9.30E-03	PRS-CSx
Ovary	PRS7	rs10077624	5	83907654	A	G	1.86E-04	PRS-CSx
Ovary	PRS7	rs1007937	9	493247	T	C	-1.95E-04	PRS-CSx
Ovary	PRS7	rs10081235	7	158322716	G	A	2.24E-04	PRS-CSx
Ovary	PRS7	rs1008305	14	78711286	G	A	-2.13E-04	PRS-CSx
Ovary	PRS7	rs10084076	18	38533110	A	G	3.37E-03	PRS-CSx
Ovary	PRS7	rs10087840	8	26844269	G	A	-1.07E-04	PRS-CSx
Ovary	PRS7	rs10093618	8	143952214	G	A	3.31E-04	PRS-CSx
Ovary	PRS7	rs10097019	8	29579387	C	T	-1.80E-05	PRS-CSx
Ovary	PRS7	rs10097667	8	93615407	T	C	2.82E-03	PRS-CSx

Ovary	PRS7	rs10098023	8	143949604	T	C	3.35E-04	PRS-CSx
Ovary	PRS7	rs10101514	8	93424545	A	G	7.03E-05	PRS-CSx
Ovary	PRS7	rs10109186	8	5104646	C	T	5.68E-05	PRS-CSx
Ovary	PRS7	rs10110526	8	107412205	C	T	1.24E-05	PRS-CSx
Ovary	PRS7	rs10112382	8	128784397	T	C	-1.28E-03	PRS-CSx
Ovary	PRS7	rs1011529	9	13755487	T	C	-1.90E-04	PRS-CSx
Ovary	PRS7	rs10115913	9	16659259	C	T	-4.88E-05	PRS-CSx
Ovary	PRS7	rs1011595	20	45055061	T	G	9.01E-04	PRS-CSx
Ovary	PRS7	rs10119403	9	109439527	A	C	-7.83E-04	PRS-CSx
Ovary	PRS7	rs10122	12	58350631	C	T	-3.53E-04	PRS-CSx
Ovary	PRS7	rs10124916	9	24756027	G	T	-1.83E-03	PRS-CSx
Ovary	PRS7	rs1013519	22	48088731	T	C	-1.21E-03	PRS-CSx
Ovary	PRS7	rs10135562	14	33128648	T	C	1.80E-05	PRS-CSx
Ovary	PRS7	rs10141122	14	33126484	C	T	-1.12E-04	PRS-CSx
Ovary	PRS7	rs10144339	14	33106344	A	G	-2.04E-04	PRS-CSx
Ovary	PRS7	rs10144370	14	21205679	T	C	-2.21E-03	PRS-CSx
Ovary	PRS7	rs10162491	14	92200291	G	T	-4.44E-05	PRS-CSx
Ovary	PRS7	rs1016522	5	143193326	G	A	-2.29E-04	PRS-CSx
Ovary	PRS7	rs1017821	19	5109701	A	G	1.12E-03	PRS-CSx
Ovary	PRS7	rs1019000	7	50809771	T	C	5.53E-04	PRS-CSx
Ovary	PRS7	rs10191541	2	12901718	C	T	3.45E-04	PRS-CSx
Ovary	PRS7	rs10193758	2	154675607	T	C	-3.42E-04	PRS-CSx
Ovary	PRS7	rs10197366	2	111996785	A	G	1.05E-03	PRS-CSx
Ovary	PRS7	rs10200140	2	133833835	A	C	-1.66E-03	PRS-CSx
Ovary	PRS7	rs10204608	2	31415263	C	T	-3.56E-04	PRS-CSx
Ovary	PRS7	rs1021213	3	154984971	C	A	1.38E-03	PRS-CSx
Ovary	PRS7	rs10212574	3	177639827	C	T	4.07E-03	PRS-CSx
Ovary	PRS7	rs1021341	3	99786289	G	A	2.34E-04	PRS-CSx
Ovary	PRS7	rs1021413	3	70024360	C	T	5.62E-04	PRS-CSx
Ovary	PRS7	rs10216311	7	11560653	C	T	-2.42E-03	PRS-CSx
Ovary	PRS7	rs10218480	1	115666678	C	T	-3.93E-03	PRS-CSx
Ovary	PRS7	rs1023095	8	132022957	C	T	-1.83E-04	PRS-CSx
Ovary	PRS7	rs1023096	8	132035562	C	T	-1.36E-04	PRS-CSx
Ovary	PRS7	rs10232620	7	158327239	C	T	1.96E-04	PRS-CSx
Ovary	PRS7	rs10236127	7	11564201	C	T	-2.39E-03	PRS-CSx
Ovary	PRS7	rs1023635	2	152162727	T	C	-1.67E-04	PRS-CSx
Ovary	PRS7	rs10239340	7	128668510	G	T	-1.38E-03	PRS-CSx
Ovary	PRS7	rs10248619	7	50751090	T	C	5.44E-04	PRS-CSx
Ovary	PRS7	rs10253511	7	130796652	C	T	4.10E-04	PRS-CSx
Ovary	PRS7	rs1025571	9	110887048	A	C	-2.15E-04	PRS-CSx
Ovary	PRS7	rs10256795	7	29195455	A	G	-1.84E-05	PRS-CSx
Ovary	PRS7	rs10259190	7	31947106	T	C	-8.55E-04	PRS-CSx
Ovary	PRS7	rs10260632	7	11564362	A	G	-1.33E-03	PRS-CSx
Ovary	PRS7	rs10261537	7	158327455	G	A	-3.99E-05	PRS-CSx
Ovary	PRS7	rs1026157	4	123623893	G	T	-1.13E-04	PRS-CSx
Ovary	PRS7	rs1026385	14	69786063	G	A	-9.70E-05	PRS-CSx
Ovary	PRS7	rs1026488	8	113894331	C	T	3.21E-04	PRS-CSx
Ovary	PRS7	rs10266729	7	158336474	T	C	7.93E-05	PRS-CSx
Ovary	PRS7	rs10267121	7	44316168	T	C	-8.92E-04	PRS-CSx
Ovary	PRS7	rs10267684	7	71626255	T	C	1.19E-04	PRS-CSx
Ovary	PRS7	rs10276916	7	48740768	T	C	1.13E-03	PRS-CSx
Ovary	PRS7	rs10277665	7	11559580	C	T	-1.41E-03	PRS-CSx
Ovary	PRS7	rs10278909	7	52554637	G	A	1.03E-04	PRS-CSx
Ovary	PRS7	rs10281065	7	50829973	A	C	2.71E-04	PRS-CSx
Ovary	PRS7	rs10281072	7	11535903	C	T	-9.79E-03	PRS-CSx
Ovary	PRS7	rs1028145	2	51928042	G	T	1.47E-03	PRS-CSx

Ovary	PRS7	rs10283307	8	133737802	G	A	-9.39E-04	PRS-CSx
Ovary	PRS7	rs1028945	8	132022101	G	A	-6.48E-05	PRS-CSx
Ovary	PRS7	rs1029967	16	6711165	C	A	-1.63E-04	PRS-CSx
Ovary	PRS7	rs1030021	2	102801478	C	A	-6.78E-04	PRS-CSx
Ovary	PRS7	rs1030065	2	217665415	G	A	-2.28E-05	PRS-CSx
Ovary	PRS7	rs1031977	8	3588100	T	C	1.66E-03	PRS-CSx
Ovary	PRS7	rs1032070	17	40618251	T	C	-6.40E-04	PRS-CSx
Ovary	PRS7	rs1034269	1	179304892	T	C	1.13E-04	PRS-CSx
Ovary	PRS7	rs1035445	19	33694158	T	C	2.53E-04	PRS-CSx
Ovary	PRS7	rs10411036	19	35410798	A	G	4.54E-04	PRS-CSx
Ovary	PRS7	rs10422961	19	35422808	T	C	6.81E-04	PRS-CSx
Ovary	PRS7	rs10425112	19	57392229	C	A	1.21E-03	PRS-CSx
Ovary	PRS7	rs10433805	4	26970376	T	C	7.16E-04	PRS-CSx
Ovary	PRS7	rs10433900	4	113961811	T	C	-1.48E-04	PRS-CSx
Ovary	PRS7	rs10447107	5	66069819	A	G	-1.34E-04	PRS-CSx
Ovary	PRS7	rs10447965	8	90056565	C	T	3.08E-05	PRS-CSx
Ovary	PRS7	rs10448083	8	29565755	T	C	-3.68E-05	PRS-CSx
Ovary	PRS7	rs10453985	10	83240443	C	T	-2.97E-04	PRS-CSx
Ovary	PRS7	rs10454473	10	129478197	G	T	-3.35E-03	PRS-CSx
Ovary	PRS7	rs10467229	13	105970320	T	C	5.00E-06	PRS-CSx
Ovary	PRS7	rs10467230	13	105970508	A	G	-2.94E-05	PRS-CSx
Ovary	PRS7	rs10469872	2	49616270	C	A	-2.73E-03	PRS-CSx
Ovary	PRS7	rs10474077	5	82282153	T	C	-4.60E-05	PRS-CSx
Ovary	PRS7	rs10477360	5	147404281	G	A	2.93E-03	PRS-CSx
Ovary	PRS7	rs1048135	2	152139416	A	G	-8.60E-05	PRS-CSx
Ovary	PRS7	rs10485289	6	49642065	A	G	3.84E-04	PRS-CSx
Ovary	PRS7	rs10486138	7	11555918	T	C	-1.28E-03	PRS-CSx
Ovary	PRS7	rs10486945	7	81773149	A	G	-1.11E-03	PRS-CSx
Ovary	PRS7	rs10488465	7	134561819	T	C	1.61E-03	PRS-CSx
Ovary	PRS7	rs10488466	7	134562744	G	A	8.41E-04	PRS-CSx
Ovary	PRS7	rs10489156	1	20575902	G	A	1.25E-03	PRS-CSx
Ovary	PRS7	rs10490523	2	152085984	G	A	-8.78E-06	PRS-CSx
Ovary	PRS7	rs10490547	2	12809444	T	C	-1.20E-03	PRS-CSx
Ovary	PRS7	rs10490550	2	12805227	T	C	-9.68E-04	PRS-CSx
Ovary	PRS7	rs10490849	3	144079222	G	A	-1.64E-03	PRS-CSx
Ovary	PRS7	rs10490985	10	117655028	A	C	-9.91E-04	PRS-CSx
Ovary	PRS7	rs10492784	16	19366583	A	G	-7.72E-04	PRS-CSx
Ovary	PRS7	rs10494067	1	107830858	C	A	-1.54E-04	PRS-CSx
Ovary	PRS7	rs10494335	1	163506187	A	G	1.87E-04	PRS-CSx
Ovary	PRS7	rs10496095	2	62413613	G	T	1.06E-03	PRS-CSx
Ovary	PRS7	rs10497518	2	179469386	G	A	-1.17E-03	PRS-CSx
Ovary	PRS7	rs10497891	2	208874208	A	G	2.45E-04	PRS-CSx
Ovary	PRS7	rs10497894	2	208916427	G	A	-5.78E-04	PRS-CSx
Ovary	PRS7	rs10501316	11	46090550	T	C	-1.28E-04	PRS-CSx
Ovary	PRS7	rs10501839	11	95793951	C	T	-6.04E-05	PRS-CSx
Ovary	PRS7	rs10507482	13	40755641	G	A	-7.70E-04	PRS-CSx
Ovary	PRS7	rs10507781	13	70728411	T	C	2.33E-03	PRS-CSx
Ovary	PRS7	rs10509190	10	65437155	G	T	1.67E-03	PRS-CSx
Ovary	PRS7	rs10509194	10	65472897	C	T	1.58E-03	PRS-CSx
Ovary	PRS7	rs10510369	3	7649026	T	C	-1.84E-03	PRS-CSx
Ovary	PRS7	rs10511177	3	99724873	A	G	7.90E-05	PRS-CSx
Ovary	PRS7	rs10511826	9	28221934	G	A	2.34E-04	PRS-CSx
Ovary	PRS7	rs10511827	9	28221514	C	T	2.36E-04	PRS-CSx
Ovary	PRS7	rs10512106	9	83106390	C	A	-1.67E-05	PRS-CSx
Ovary	PRS7	rs10512108	9	83116483	A	G	1.47E-04	PRS-CSx
Ovary	PRS7	rs10514496	16	80456403	C	T	7.98E-06	PRS-CSx

Ovary	PRS7	rs10515165	5	73527824	A	G	-5.20E-05	PRS-CSx
Ovary	PRS7	rs10516367	4	20882971	A	C	1.34E-04	PRS-CSx
Ovary	PRS7	rs10517757	4	162933226	T	C	-4.36E-04	PRS-CSx
Ovary	PRS7	rs10518287	19	35399406	T	C	7.86E-04	PRS-CSx
Ovary	PRS7	rs10521	9	139397707	G	A	1.86E-03	PRS-CSx
Ovary	PRS7	rs1052898	4	25679840	A	G	3.31E-03	PRS-CSx
Ovary	PRS7	rs1054885	20	60776182	G	A	-1.23E-04	PRS-CSx
Ovary	PRS7	rs1055075	11	113239512	T	C	2.42E-04	PRS-CSx
Ovary	PRS7	rs1055221	4	95664988	G	A	-1.25E-03	PRS-CSx
Ovary	PRS7	rs1057704	11	122830274	C	T	2.81E-05	PRS-CSx
Ovary	PRS7	rs1057985	6	44185901	C	T	-1.03E-03	PRS-CSx
Ovary	PRS7	rs1069216	1	245578659	A	G	3.17E-04	PRS-CSx
Ovary	PRS7	rs10732253	11	67006400	T	C	1.98E-03	PRS-CSx
Ovary	PRS7	rs10737472	1	23708919	C	A	-1.10E-03	PRS-CSx
Ovary	PRS7	rs10737489	1	162813950	C	T	8.11E-05	PRS-CSx
Ovary	PRS7	rs10741174	10	130659224	G	T	-4.32E-03	PRS-CSx
Ovary	PRS7	rs10745023	19	9413896	A	C	5.13E-04	PRS-CSx
Ovary	PRS7	rs10747786	12	58260601	C	A	-3.85E-04	PRS-CSx
Ovary	PRS7	rs10747787	12	58260657	T	C	-3.34E-04	PRS-CSx
Ovary	PRS7	rs10749771	1	67573730	A	G	-7.74E-05	PRS-CSx
Ovary	PRS7	rs10752721	1	3726326	C	T	-3.41E-05	PRS-CSx
Ovary	PRS7	rs10754377	1	116380172	C	T	-8.27E-05	PRS-CSx
Ovary	PRS7	rs10755201	4	20674761	T	C	5.03E-05	PRS-CSx
Ovary	PRS7	rs10758442	9	37591236	G	T	-8.77E-05	PRS-CSx
Ovary	PRS7	rs10758653	9	489338	G	A	-6.02E-05	PRS-CSx
Ovary	PRS7	rs10758655	9	490071	A	C	-1.69E-04	PRS-CSx
Ovary	PRS7	rs10761346	9	97363620	A	G	1.88E-04	PRS-CSx
Ovary	PRS7	rs1076394	3	47322781	A	G	-1.27E-04	PRS-CSx
Ovary	PRS7	rs10767873	11	30768678	T	C	1.67E-04	PRS-CSx
Ovary	PRS7	rs10770617	12	20280027	G	A	2.35E-03	PRS-CSx
Ovary	PRS7	rs10772893	12	16255325	G	A	-2.29E-04	PRS-CSx
Ovary	PRS7	rs10773622	12	129716348	G	A	-1.27E-03	PRS-CSx
Ovary	PRS7	rs10776845	9	138669976	A	C	1.55E-03	PRS-CSx
Ovary	PRS7	rs10779842	1	230450968	A	C	-3.56E-03	PRS-CSx
Ovary	PRS7	rs10783508	12	52678299	T	C	-1.27E-03	PRS-CSx
Ovary	PRS7	rs10783855	12	58246356	C	A	-2.32E-04	PRS-CSx
Ovary	PRS7	rs10787226	10	111981917	A	G	1.45E-04	PRS-CSx
Ovary	PRS7	rs10792746	11	83976634	T	C	-1.61E-04	PRS-CSx
Ovary	PRS7	rs10795832	10	10946610	T	G	-5.62E-05	PRS-CSx
Ovary	PRS7	rs10798346	1	171374129	C	T	-1.96E-04	PRS-CSx
Ovary	PRS7	rs10798656	1	179264291	A	C	-6.79E-05	PRS-CSx
Ovary	PRS7	rs10802432	1	246854862	C	T	-1.85E-04	PRS-CSx
Ovary	PRS7	rs10804164	2	208919340	G	A	-3.66E-04	PRS-CSx
Ovary	PRS7	rs10804242	2	216256707	T	C	6.20E-04	PRS-CSx
Ovary	PRS7	rs10806414	6	90665969	A	G	-1.70E-03	PRS-CSx
Ovary	PRS7	rs10806993	6	24808283	A	G	1.33E-04	PRS-CSx
Ovary	PRS7	rs10808648	8	93393331	A	G	1.08E-04	PRS-CSx
Ovary	PRS7	rs10810166	9	14495574	T	C	5.19E-04	PRS-CSx
Ovary	PRS7	rs10810856	9	17834342	A	G	9.20E-04	PRS-CSx
Ovary	PRS7	rs10813246	9	30403952	A	G	-9.54E-05	PRS-CSx
Ovary	PRS7	rs10815672	9	7758579	A	G	5.23E-04	PRS-CSx
Ovary	PRS7	rs10817477	9	116075797	C	T	-5.76E-07	PRS-CSx
Ovary	PRS7	rs10818187	9	121325521	G	A	1.24E-03	PRS-CSx
Ovary	PRS7	rs10819395	9	101633082	T	C	-1.69E-03	PRS-CSx
Ovary	PRS7	rs10819508	9	132147913	C	T	4.95E-04	PRS-CSx
Ovary	PRS7	rs10826550	10	29277762	A	G	8.14E-05	PRS-CSx

Ovary	PRS7	rs10833952	11	23169049	C	T	-5.11E-04	PRS-CSx
Ovary	PRS7	rs10833955	11	23179037	G	A	-9.02E-04	PRS-CSx
Ovary	PRS7	rs10836533	11	36329290	T	G	-2.02E-03	PRS-CSx
Ovary	PRS7	rs10838696	11	47363285	A	G	1.58E-03	PRS-CSx
Ovary	PRS7	rs10844071	12	32114175	A	G	1.25E-03	PRS-CSx
Ovary	PRS7	rs10844075	12	32121276	G	T	-1.13E-03	PRS-CSx
Ovary	PRS7	rs10847779	12	129621928	C	T	-1.29E-04	PRS-CSx
Ovary	PRS7	rs10849318	12	5787341	G	T	1.85E-04	PRS-CSx
Ovary	PRS7	rs10854157	19	9606461	T	C	5.59E-04	PRS-CSx
Ovary	PRS7	rs10856860	4	76756059	T	G	-1.38E-04	PRS-CSx
Ovary	PRS7	rs10859789	12	78395973	A	G	-5.23E-05	PRS-CSx
Ovary	PRS7	rs10864734	1	230422210	C	A	-1.64E-04	PRS-CSx
Ovary	PRS7	rs10867333	9	81651602	C	T	-9.36E-04	PRS-CSx
Ovary	PRS7	rs10867902	9	85451187	T	C	2.09E-03	PRS-CSx
Ovary	PRS7	rs10872167	6	118988362	G	A	-8.76E-04	PRS-CSx
Ovary	PRS7	rs10875385	8	135629574	A	G	4.16E-04	PRS-CSx
Ovary	PRS7	rs10876276	12	52713218	T	C	-7.95E-04	PRS-CSx
Ovary	PRS7	rs10876915	12	57049064	T	C	3.69E-04	PRS-CSx
Ovary	PRS7	rs10877028	12	58241028	T	G	-1.87E-04	PRS-CSx
Ovary	PRS7	rs10877029	12	58256010	T	C	-4.04E-04	PRS-CSx
Ovary	PRS7	rs10877030	12	58256714	T	G	-3.23E-04	PRS-CSx
Ovary	PRS7	rs10877036	12	58321650	C	T	-1.07E-03	PRS-CSx
Ovary	PRS7	rs10878964	12	69720691	G	T	3.37E-04	PRS-CSx
Ovary	PRS7	rs10882326	10	95509444	T	C	-3.15E-04	PRS-CSx
Ovary	PRS7	rs10884942	10	112016926	A	G	2.84E-04	PRS-CSx
Ovary	PRS7	rs10886107	10	85066102	T	C	2.45E-04	PRS-CSx
Ovary	PRS7	rs10888614	1	49178446	C	A	-2.89E-03	PRS-CSx
Ovary	PRS7	rs10891546	11	113265292	T	C	2.30E-04	PRS-CSx
Ovary	PRS7	rs10893867	11	128290243	G	A	-1.91E-03	PRS-CSx
Ovary	PRS7	rs10905858	10	10945862	G	A	-2.25E-04	PRS-CSx
Ovary	PRS7	rs10908898	9	92196724	T	G	-1.93E-03	PRS-CSx
Ovary	PRS7	rs10911836	1	186149235	T	C	1.39E-03	PRS-CSx
Ovary	PRS7	rs10924556	1	246378041	A	G	-7.51E-05	PRS-CSx
Ovary	PRS7	rs10924564	1	246380096	A	G	1.17E-04	PRS-CSx
Ovary	PRS7	rs10924576	1	246394175	T	C	-1.46E-04	PRS-CSx
Ovary	PRS7	rs10924577	1	246394232	T	C	4.69E-05	PRS-CSx
Ovary	PRS7	rs10924579	1	246394320	G	A	-7.80E-05	PRS-CSx
Ovary	PRS7	rs10924611	1	246406762	T	C	-1.88E-04	PRS-CSx
Ovary	PRS7	rs10931792	2	154669222	C	T	1.30E-06	PRS-CSx
Ovary	PRS7	rs10932221	2	208901194	C	T	-3.96E-04	PRS-CSx
Ovary	PRS7	rs10932228	2	208919650	A	G	-3.14E-04	PRS-CSx
Ovary	PRS7	rs10932236	2	208951713	A	G	-1.25E-03	PRS-CSx
Ovary	PRS7	rs10936005	3	99674093	G	A	1.42E-04	PRS-CSx
Ovary	PRS7	rs10936008	3	99686665	A	G	2.44E-04	PRS-CSx
Ovary	PRS7	rs10938692	4	8118561	T	C	5.21E-05	PRS-CSx
Ovary	PRS7	rs10941508	5	40459067	A	G	-5.16E-04	PRS-CSx
Ovary	PRS7	rs10947787	6	39174525	T	C	-6.26E-06	PRS-CSx
Ovary	PRS7	rs10949798	7	155843444	T	C	5.96E-04	PRS-CSx
Ovary	PRS7	rs10951881	7	46951569	T	C	-1.10E-03	PRS-CSx
Ovary	PRS7	rs10961543	9	14494785	A	G	1.05E-03	PRS-CSx
Ovary	PRS7	rs10962763	9	16950860	C	A	4.99E-05	PRS-CSx
Ovary	PRS7	rs10963625	9	18530339	G	A	-1.95E-04	PRS-CSx
Ovary	PRS7	rs10965243	9	22130065	G	A	1.02E-03	PRS-CSx
Ovary	PRS7	rs10968382	9	28197129	G	A	2.05E-04	PRS-CSx
Ovary	PRS7	rs10968386	9	28205326	G	T	1.67E-04	PRS-CSx
Ovary	PRS7	rs10970585	9	31895373	G	A	1.16E-03	PRS-CSx

Ovary	PRS7	rs10980112	9	112666483	A	G	-2.74E-03	PRS-CSx
Ovary	PRS7	rs10989591	9	104449098	T	C	-1.02E-03	PRS-CSx
Ovary	PRS7	rs10989747	9	98402621	G	A	-1.57E-03	PRS-CSx
Ovary	PRS7	rs10992480	9	95580376	T	C	1.42E-03	PRS-CSx
Ovary	PRS7	rs10993888	9	136865611	A	C	1.29E-04	PRS-CSx
Ovary	PRS7	rs11002799	10	54385382	T	C	-2.16E-03	PRS-CSx
Ovary	PRS7	rs11007352	10	29290958	C	A	1.89E-04	PRS-CSx
Ovary	PRS7	rs11009462	10	33885610	C	T	-1.23E-03	PRS-CSx
Ovary	PRS7	rs11021396	11	95771968	C	T	1.11E-04	PRS-CSx
Ovary	PRS7	rs11022341	11	12427916	T	C	1.73E-05	PRS-CSx
Ovary	PRS7	rs11027967	11	24537624	C	T	3.82E-03	PRS-CSx
Ovary	PRS7	rs11033457	11	36162848	G	T	1.83E-03	PRS-CSx
Ovary	PRS7	rs11038752	11	46072537	T	C	-3.43E-04	PRS-CSx
Ovary	PRS7	rs11039183	11	47346940	A	G	7.54E-04	PRS-CSx
Ovary	PRS7	rs11051124	12	31026370	C	T	-7.27E-03	PRS-CSx
Ovary	PRS7	rs11072215	15	71096541	G	A	-9.35E-04	PRS-CSx
Ovary	PRS7	rs11072810	15	79132206	C	T	-8.41E-05	PRS-CSx
Ovary	PRS7	rs11073182	15	36868554	C	T	1.05E-03	PRS-CSx
Ovary	PRS7	rs11073183	15	36881360	G	T	-4.29E-04	PRS-CSx
Ovary	PRS7	rs11073337	15	38847763	C	A	4.17E-05	PRS-CSx
Ovary	PRS7	rs1107479	12	57030686	T	C	4.64E-04	PRS-CSx
Ovary	PRS7	rs1107698	2	45355038	C	T	-2.63E-03	PRS-CSx
Ovary	PRS7	rs11077543	17	69071206	A	G	-7.87E-04	PRS-CSx
Ovary	PRS7	rs1107920	19	33690696	A	G	7.09E-05	PRS-CSx
Ovary	PRS7	rs11079394	17	43031392	A	C	-2.27E-04	PRS-CSx
Ovary	PRS7	rs11080090	17	27502029	G	A	-1.66E-04	PRS-CSx
Ovary	PRS7	rs11080138	17	29224542	G	A	2.26E-04	PRS-CSx
Ovary	PRS7	rs11080868	18	1574369	A	C	1.20E-04	PRS-CSx
Ovary	PRS7	rs11081191	18	5415078	T	C	-1.59E-03	PRS-CSx
Ovary	PRS7	rs11081192	18	5415121	T	C	-1.42E-03	PRS-CSx
Ovary	PRS7	rs11083093	18	22393283	A	G	1.49E-03	PRS-CSx
Ovary	PRS7	rs1108845	7	151522715	T	C	1.42E-03	PRS-CSx
Ovary	PRS7	rs11089938	22	39662703	T	C	6.26E-04	PRS-CSx
Ovary	PRS7	rs1110007	11	12160041	T	G	-5.82E-05	PRS-CSx
Ovary	PRS7	rs11102059	1	110690624	A	G	-4.41E-04	PRS-CSx
Ovary	PRS7	rs11105221	12	89647764	G	A	-1.38E-03	PRS-CSx
Ovary	PRS7	rs11107616	12	78364780	G	T	-2.97E-04	PRS-CSx
Ovary	PRS7	rs11107763	12	78391958	C	A	-7.44E-04	PRS-CSx
Ovary	PRS7	rs11111940	12	104628831	C	T	-1.32E-04	PRS-CSx
Ovary	PRS7	rs11113256	12	107690149	C	T	5.92E-04	PRS-CSx
Ovary	PRS7	rs11114401	12	80715764	C	T	5.76E-04	PRS-CSx
Ovary	PRS7	rs11114402	12	80717926	C	T	5.64E-04	PRS-CSx
Ovary	PRS7	rs11114440	12	80827595	T	C	1.22E-04	PRS-CSx
Ovary	PRS7	rs11121472	1	9733221	C	A	-3.88E-04	PRS-CSx
Ovary	PRS7	rs11123201	2	111601478	A	G	7.75E-04	PRS-CSx
Ovary	PRS7	rs11123911	2	102761538	T	C	-4.52E-04	PRS-CSx
Ovary	PRS7	rs11126477	2	75786771	G	A	-1.19E-03	PRS-CSx
Ovary	PRS7	rs11129210	3	25885030	G	A	-3.30E-04	PRS-CSx
Ovary	PRS7	rs11130115	3	47181661	C	A	1.06E-05	PRS-CSx
Ovary	PRS7	rs11130123	3	47285912	A	G	-8.40E-05	PRS-CSx
Ovary	PRS7	rs11130124	3	47292183	T	C	4.16E-05	PRS-CSx
Ovary	PRS7	rs11135445	5	92064034	A	G	-1.43E-03	PRS-CSx
Ovary	PRS7	rs11138681	9	83087110	A	G	3.72E-06	PRS-CSx
Ovary	PRS7	rs11139819	9	85450860	A	G	2.68E-03	PRS-CSx
Ovary	PRS7	rs11147005	12	133262001	T	C	-6.56E-04	PRS-CSx
Ovary	PRS7	rs1114761	8	143928956	T	C	2.60E-04	PRS-CSx

Ovary	PRS7	rs11153000	6	106975536	G	T	-9.05E-04	PRS-CSx
Ovary	PRS7	rs11153777	6	119132164	A	G	1.18E-03	PRS-CSx
Ovary	PRS7	rs11153778	6	119224416	G	T	1.03E-03	PRS-CSx
Ovary	PRS7	rs11153987	6	121338019	T	C	-2.85E-04	PRS-CSx
Ovary	PRS7	rs11155639	6	149637690	C	T	1.47E-03	PRS-CSx
Ovary	PRS7	rs11155641	6	149648595	A	G	4.67E-04	PRS-CSx
Ovary	PRS7	rs11158869	14	71057558	C	T	1.43E-03	PRS-CSx
Ovary	PRS7	rs11160071	14	92938415	T	C	-1.54E-03	PRS-CSx
Ovary	PRS7	rs11164125	2	96057129	T	C	7.25E-04	PRS-CSx
Ovary	PRS7	rs11166629	8	135636964	C	T	2.80E-04	PRS-CSx
Ovary	PRS7	rs11170305	12	53247821	C	T	7.57E-04	PRS-CSx
Ovary	PRS7	rs11172367	12	58272486	T	G	-1.54E-04	PRS-CSx
Ovary	PRS7	rs11172378	12	58298161	C	T	-2.54E-04	PRS-CSx
Ovary	PRS7	rs11177065	12	40923532	A	G	-7.56E-04	PRS-CSx
Ovary	PRS7	rs11177577	12	69671791	A	G	4.48E-04	PRS-CSx
Ovary	PRS7	rs11177644	12	69785748	G	A	4.00E-04	PRS-CSx
Ovary	PRS7	rs11183504	12	46880770	G	A	-1.16E-03	PRS-CSx
Ovary	PRS7	rs11185895	10	91577239	T	C	1.43E-04	PRS-CSx
Ovary	PRS7	rs11189862	10	83192498	A	G	2.73E-04	PRS-CSx
Ovary	PRS7	rs11195062	10	112036975	C	A	5.07E-05	PRS-CSx
Ovary	PRS7	rs11195969	10	114223234	T	C	2.42E-03	PRS-CSx
Ovary	PRS7	rs11198145	10	119606119	C	T	-7.83E-03	PRS-CSx
Ovary	PRS7	rs11198672	10	85193946	T	C	-9.53E-04	PRS-CSx
Ovary	PRS7	rs11209002	1	67590461	T	C	-3.63E-04	PRS-CSx
Ovary	PRS7	rs11209112	1	68018455	A	G	-1.31E-03	PRS-CSx
Ovary	PRS7	rs11212325	11	107555531	G	A	-1.57E-03	PRS-CSx
Ovary	PRS7	rs11214338	11	112614196	A	G	1.58E-04	PRS-CSx
Ovary	PRS7	rs11214343	11	112616071	C	T	-1.11E-05	PRS-CSx
Ovary	PRS7	rs11214769	11	113790668	G	A	5.32E-04	PRS-CSx
Ovary	PRS7	rs11221290	11	128287077	T	C	-2.17E-03	PRS-CSx
Ovary	PRS7	rs11222930	11	131978142	A	G	5.30E-04	PRS-CSx
Ovary	PRS7	rs11222937	11	131980561	G	A	5.32E-04	PRS-CSx
Ovary	PRS7	rs11228368	11	68593258	G	A	2.81E-04	PRS-CSx
Ovary	PRS7	rs11234891	11	86670842	A	G	7.58E-04	PRS-CSx
Ovary	PRS7	rs11239252	10	45320830	C	T	8.06E-05	PRS-CSx
Ovary	PRS7	rs11239953	1	146717564	C	T	5.52E-04	PRS-CSx
Ovary	PRS7	rs11245515	10	126824068	T	C	-2.53E-03	PRS-CSx
Ovary	PRS7	rs11247495	16	46804464	T	C	-8.52E-05	PRS-CSx
Ovary	PRS7	rs11249477	4	70934425	A	G	-2.18E-03	PRS-CSx
Ovary	PRS7	rs11252284	10	4069215	T	C	-1.73E-03	PRS-CSx
Ovary	PRS7	rs1125307	4	8117671	A	G	9.68E-05	PRS-CSx
Ovary	PRS7	rs11259333	10	14817067	C	T	-4.23E-04	PRS-CSx
Ovary	PRS7	rs11265277	1	159742091	C	T	-4.30E-04	PRS-CSx
Ovary	PRS7	rs1134095	8	143954290	G	A	3.75E-04	PRS-CSx
Ovary	PRS7	rs1134096	8	143954223	C	A	2.96E-04	PRS-CSx
Ovary	PRS7	rs1149460	8	64577436	C	A	-5.93E-04	PRS-CSx
Ovary	PRS7	rs1149464	8	64587362	T	G	-1.70E-04	PRS-CSx
Ovary	PRS7	rs1149842	13	50752815	T	C	-2.35E-03	PRS-CSx
Ovary	PRS7	rs11550558	12	56386076	G	A	1.75E-03	PRS-CSx
Ovary	PRS7	rs11576437	1	212442718	G	A	9.23E-05	PRS-CSx
Ovary	PRS7	rs1157840	3	187620527	C	A	-9.47E-05	PRS-CSx
Ovary	PRS7	rs11580295	1	119836236	T	C	1.19E-05	PRS-CSx
Ovary	PRS7	rs11589817	1	212444738	G	A	5.22E-05	PRS-CSx
Ovary	PRS7	rs11595	8	141533818	A	G	1.46E-04	PRS-CSx
Ovary	PRS7	rs11596551	10	45015730	A	G	-8.47E-05	PRS-CSx
Ovary	PRS7	rs11605596	11	125193811	A	G	1.92E-04	PRS-CSx

Ovary	PRS7	rs11606117	11	125194213	T	G	3.48E-04	PRS-CSx
Ovary	PRS7	rs11606683	11	47302733	T	C	1.33E-03	PRS-CSx
Ovary	PRS7	rs1161841	13	62944041	T	C	3.02E-04	PRS-CSx
Ovary	PRS7	rs11627196	14	71875838	A	G	-9.58E-04	PRS-CSx
Ovary	PRS7	rs11629796	15	48045248	G	A	-4.79E-05	PRS-CSx
Ovary	PRS7	rs11636698	15	71053269	C	T	-1.03E-03	PRS-CSx
Ovary	PRS7	rs11637003	15	94445699	T	C	2.53E-03	PRS-CSx
Ovary	PRS7	rs11637242	15	71018293	G	A	-4.09E-04	PRS-CSx
Ovary	PRS7	rs11639262	15	50399646	G	A	8.51E-04	PRS-CSx
Ovary	PRS7	rs11643092	16	10166373	C	T	-6.31E-04	PRS-CSx
Ovary	PRS7	rs11650271	17	29213943	T	C	6.16E-04	PRS-CSx
Ovary	PRS7	rs11650305	17	29229361	C	A	4.89E-04	PRS-CSx
Ovary	PRS7	rs11651802	17	29159660	C	T	5.01E-04	PRS-CSx
Ovary	PRS7	rs11651858	17	29170718	G	A	1.71E-04	PRS-CSx
Ovary	PRS7	rs11652631	17	29060766	G	A	2.99E-04	PRS-CSx
Ovary	PRS7	rs11655623	17	29162173	C	T	2.53E-04	PRS-CSx
Ovary	PRS7	rs11657270	17	29214387	C	T	3.65E-04	PRS-CSx
Ovary	PRS7	rs11658435	17	29228561	G	A	5.11E-04	PRS-CSx
Ovary	PRS7	rs11660127	18	10430734	T	C	8.73E-04	PRS-CSx
Ovary	PRS7	rs11666027	19	52157686	C	T	3.06E-03	PRS-CSx
Ovary	PRS7	rs11670586	19	52184544	T	C	-1.47E-03	PRS-CSx
Ovary	PRS7	rs11676872	2	208874694	G	A	3.06E-04	PRS-CSx
Ovary	PRS7	rs11681351	2	27743423	A	G	4.33E-04	PRS-CSx
Ovary	PRS7	rs11685032	2	11674295	C	A	1.28E-03	PRS-CSx
Ovary	PRS7	rs11686210	2	25433366	A	G	-8.33E-04	PRS-CSx
Ovary	PRS7	rs11690038	2	233410503	C	T	-2.86E-03	PRS-CSx
Ovary	PRS7	rs11690080	2	75845448	C	T	1.84E-04	PRS-CSx
Ovary	PRS7	rs11691838	2	119675587	T	C	-4.64E-04	PRS-CSx
Ovary	PRS7	rs11694842	2	25482970	G	A	-1.28E-03	PRS-CSx
Ovary	PRS7	rs11703249	22	22333025	T	C	1.78E-03	PRS-CSx
Ovary	PRS7	rs11704319	22	39722886	G	A	1.10E-03	PRS-CSx
Ovary	PRS7	rs11705960	3	146494488	G	A	-2.25E-04	PRS-CSx
Ovary	PRS7	rs11708892	3	68337818	A	C	-4.83E-04	PRS-CSx
Ovary	PRS7	rs11709121	3	142371574	C	A	6.26E-05	PRS-CSx
Ovary	PRS7	rs11710013	3	47296921	T	C	1.44E-04	PRS-CSx
Ovary	PRS7	rs11710322	3	47331136	T	C	-1.15E-04	PRS-CSx
Ovary	PRS7	rs11710497	3	11045896	G	A	-8.20E-03	PRS-CSx
Ovary	PRS7	rs11713266	3	7645217	G	T	-2.41E-03	PRS-CSx
Ovary	PRS7	rs11715178	3	20764798	A	G	-1.93E-04	PRS-CSx
Ovary	PRS7	rs11718882	3	70020495	A	C	5.05E-04	PRS-CSx
Ovary	PRS7	rs11719347	3	20432319	A	C	-2.97E-03	PRS-CSx
Ovary	PRS7	rs11725651	4	140413146	T	C	1.72E-04	PRS-CSx
Ovary	PRS7	rs11726503	4	99883163	G	A	9.50E-04	PRS-CSx
Ovary	PRS7	rs11728485	4	113954077	T	C	4.72E-05	PRS-CSx
Ovary	PRS7	rs11730	2	152127011	G	A	-2.66E-05	PRS-CSx
Ovary	PRS7	rs11730600	4	11623160	A	G	4.08E-03	PRS-CSx
Ovary	PRS7	rs11731578	4	7549231	A	G	3.93E-03	PRS-CSx
Ovary	PRS7	rs11737681	4	59630821	C	A	6.75E-05	PRS-CSx
Ovary	PRS7	rs11737692	4	2287421	C	A	-4.15E-05	PRS-CSx
Ovary	PRS7	rs11747607	5	153832289	G	T	-5.47E-05	PRS-CSx
Ovary	PRS7	rs11751090	6	118005198	G	T	-1.58E-03	PRS-CSx
Ovary	PRS7	rs11753185	6	12518688	T	C	1.79E-03	PRS-CSx
Ovary	PRS7	rs1175551	1	3688645	T	C	2.53E-05	PRS-CSx
Ovary	PRS7	rs11764433	7	11545990	C	T	1.17E-03	PRS-CSx
Ovary	PRS7	rs11769599	7	149445992	G	A	3.87E-03	PRS-CSx
Ovary	PRS7	rs11773521	7	115806524	C	T	2.32E-05	PRS-CSx

Ovary	PRS7	rs11774513	8	134158551	A	G	-1.89E-04	PRS-CSx
Ovary	PRS7	rs11774739	8	3987971	C	T	4.81E-05	PRS-CSx
Ovary	PRS7	rs11775009	8	144008181	C	T	2.36E-04	PRS-CSx
Ovary	PRS7	rs11775918	8	93299275	G	A	-7.23E-05	PRS-CSx
Ovary	PRS7	rs11777065	8	1647979	A	G	-1.24E-04	PRS-CSx
Ovary	PRS7	rs11778112	8	134167681	A	G	-4.74E-04	PRS-CSx
Ovary	PRS7	rs11778699	8	29423230	C	T	-1.63E-04	PRS-CSx
Ovary	PRS7	rs11779308	8	48102987	A	G	1.34E-04	PRS-CSx
Ovary	PRS7	rs11784589	8	134170487	C	T	-3.87E-04	PRS-CSx
Ovary	PRS7	rs11803956	1	207803021	C	T	-4.60E-04	PRS-CSx
Ovary	PRS7	rs11813816	10	70902716	A	G	-2.81E-04	PRS-CSx
Ovary	PRS7	rs11814910	10	87659758	C	T	8.55E-04	PRS-CSx
Ovary	PRS7	rs11817569	10	70951346	C	T	-5.43E-04	PRS-CSx
Ovary	PRS7	rs1181871	1	3684321	A	G	1.39E-04	PRS-CSx
Ovary	PRS7	rs1181874	1	3682070	C	A	-7.88E-06	PRS-CSx
Ovary	PRS7	rs11820583	11	21321010	C	T	1.28E-03	PRS-CSx
Ovary	PRS7	rs1182218	1	117361083	C	T	2.80E-03	PRS-CSx
Ovary	PRS7	rs1182530	20	58385800	G	A	-3.33E-05	PRS-CSx
Ovary	PRS7	rs11836770	12	40034699	A	G	-2.43E-03	PRS-CSx
Ovary	PRS7	rs11840785	13	107871944	G	A	-2.40E-03	PRS-CSx
Ovary	PRS7	rs11855134	15	48047676	G	A	-7.41E-05	PRS-CSx
Ovary	PRS7	rs11856607	15	62386695	T	G	1.10E-04	PRS-CSx
Ovary	PRS7	rs1185820	20	58386357	C	A	4.47E-05	PRS-CSx
Ovary	PRS7	rs11862829	16	19350539	C	A	-1.58E-03	PRS-CSx
Ovary	PRS7	rs11871801	17	40570772	C	A	-4.10E-04	PRS-CSx
Ovary	PRS7	rs1188130	8	13173084	C	T	-1.68E-03	PRS-CSx
Ovary	PRS7	rs11881376	19	20010710	A	C	-8.98E-04	PRS-CSx
Ovary	PRS7	rs11891423	2	208905879	T	C	-4.29E-04	PRS-CSx
Ovary	PRS7	rs11897191	2	50443908	A	G	4.52E-04	PRS-CSx
Ovary	PRS7	rs11901892	2	101326608	T	C	1.49E-02	PRS-CSx
Ovary	PRS7	rs11913594	22	37246329	T	C	-1.30E-03	PRS-CSx
Ovary	PRS7	rs11926561	3	156357328	T	C	3.82E-03	PRS-CSx
Ovary	PRS7	rs11930461	4	188590745	A	C	1.89E-03	PRS-CSx
Ovary	PRS7	rs11931074	4	90639515	G	T	-6.79E-06	PRS-CSx
Ovary	PRS7	rs11933172	4	110227513	T	C	2.42E-04	PRS-CSx
Ovary	PRS7	rs11936157	4	24271651	A	G	-1.64E-03	PRS-CSx
Ovary	PRS7	rs11944364	4	32530706	T	C	-8.22E-04	PRS-CSx
Ovary	PRS7	rs11947543	4	45530102	T	C	-1.96E-03	PRS-CSx
Ovary	PRS7	rs11952173	5	177589632	T	C	-5.97E-04	PRS-CSx
Ovary	PRS7	rs11954029	5	11114201	A	G	-7.89E-04	PRS-CSx
Ovary	PRS7	rs11959608	5	146902047	A	G	-2.09E-04	PRS-CSx
Ovary	PRS7	rs11965083	6	45827480	G	A	1.11E-03	PRS-CSx
Ovary	PRS7	rs11965794	6	6495031	A	G	7.92E-05	PRS-CSx
Ovary	PRS7	rs11966294	6	110725061	C	T	2.04E-04	PRS-CSx
Ovary	PRS7	rs11970286	6	118680374	T	C	-4.41E-04	PRS-CSx
Ovary	PRS7	rs11971487	7	11561373	C	T	-2.26E-03	PRS-CSx
Ovary	PRS7	rs11985416	8	135634005	T	C	1.58E-04	PRS-CSx
Ovary	PRS7	rs11988	11	47261260	A	G	1.15E-03	PRS-CSx
Ovary	PRS7	rs11988715	8	76944293	T	C	3.91E-03	PRS-CSx
Ovary	PRS7	rs11991217	8	124817145	A	G	2.21E-03	PRS-CSx
Ovary	PRS7	rs11994326	8	14394225	G	A	-5.41E-04	PRS-CSx
Ovary	PRS7	rs11997795	8	124834384	A	G	6.73E-04	PRS-CSx
Ovary	PRS7	rs12006360	9	16887073	T	C	1.34E-03	PRS-CSx
Ovary	PRS7	rs12006374	9	16887167	T	C	1.74E-03	PRS-CSx
Ovary	PRS7	rs12006443	9	113084316	C	T	-2.12E-03	PRS-CSx
Ovary	PRS7	rs12023455	1	213602132	T	C	-2.95E-05	PRS-CSx

Ovary	PRS7	rs12033048	1	246429681	T	C	-6.84E-06	PRS-CSx
Ovary	PRS7	rs12033636	1	50023716	T	C	8.92E-05	PRS-CSx
Ovary	PRS7	rs12034383	1	207803595	A	G	-8.46E-04	PRS-CSx
Ovary	PRS7	rs12035573	1	18697149	G	A	-2.01E-04	PRS-CSx
Ovary	PRS7	rs12036430	1	89921593	A	G	4.97E-04	PRS-CSx
Ovary	PRS7	rs12036597	1	39529720	C	T	1.43E-03	PRS-CSx
Ovary	PRS7	rs12038775	1	50463136	A	G	-3.75E-04	PRS-CSx
Ovary	PRS7	rs12039250	1	9726705	A	G	-2.16E-04	PRS-CSx
Ovary	PRS7	rs12041676	1	8273392	A	G	-1.08E-03	PRS-CSx
Ovary	PRS7	rs12043973	1	50336842	C	T	-4.92E-04	PRS-CSx
Ovary	PRS7	rs12047614	1	50023580	T	G	-3.99E-04	PRS-CSx
Ovary	PRS7	rs12052336	2	97106451	C	T	-7.77E-04	PRS-CSx
Ovary	PRS7	rs1206147	6	97565173	G	A	7.52E-06	PRS-CSx
Ovary	PRS7	rs1206178	6	97591680	A	G	3.80E-05	PRS-CSx
Ovary	PRS7	rs12070592	1	7151665	T	C	8.18E-04	PRS-CSx
Ovary	PRS7	rs12074459	1	51762087	G	A	-2.27E-04	PRS-CSx
Ovary	PRS7	rs12086457	1	111140160	A	G	-1.03E-03	PRS-CSx
Ovary	PRS7	rs12101469	15	61571546	C	T	1.09E-03	PRS-CSx
Ovary	PRS7	rs12101846	15	61571328	T	G	5.36E-04	PRS-CSx
Ovary	PRS7	rs12103588	17	29197460	T	C	4.36E-04	PRS-CSx
Ovary	PRS7	rs12109416	5	150982156	A	G	-2.55E-03	PRS-CSx
Ovary	PRS7	rs12118197	1	110701174	G	A	-3.42E-04	PRS-CSx
Ovary	PRS7	rs12118760	1	20999804	C	T	-7.16E-04	PRS-CSx
Ovary	PRS7	rs12130070	1	246374741	G	A	-8.71E-05	PRS-CSx
Ovary	PRS7	rs12135034	1	66646184	G	A	-1.99E-03	PRS-CSx
Ovary	PRS7	rs12144933	1	164810239	T	C	2.46E-03	PRS-CSx
Ovary	PRS7	rs12155836	8	93443393	A	G	2.92E-04	PRS-CSx
Ovary	PRS7	rs1217107	8	64579060	T	C	-2.88E-05	PRS-CSx
Ovary	PRS7	rs1217113	8	64594355	A	C	8.16E-05	PRS-CSx
Ovary	PRS7	rs12190861	6	113936188	G	A	1.40E-03	PRS-CSx
Ovary	PRS7	rs12190983	6	114385035	A	G	1.11E-03	PRS-CSx
Ovary	PRS7	rs12191684	6	164071406	T	C	-1.88E-04	PRS-CSx
Ovary	PRS7	rs12201804	6	114396341	T	C	1.37E-03	PRS-CSx
Ovary	PRS7	rs12202381	6	24139942	G	A	1.95E-04	PRS-CSx
Ovary	PRS7	rs12211928	6	114412037	T	C	1.20E-03	PRS-CSx
Ovary	PRS7	rs12213875	6	14587003	G	A	-1.08E-03	PRS-CSx
Ovary	PRS7	rs12217516	10	95338445	C	T	-5.99E-04	PRS-CSx
Ovary	PRS7	rs12219199	10	95332517	T	C	-2.13E-04	PRS-CSx
Ovary	PRS7	rs12220062	10	95336139	A	G	-1.33E-03	PRS-CSx
Ovary	PRS7	rs12222421	11	36161179	A	C	1.49E-03	PRS-CSx
Ovary	PRS7	rs12230560	12	100053656	C	T	-1.18E-04	PRS-CSx
Ovary	PRS7	rs1223954	15	99767071	C	T	7.07E-04	PRS-CSx
Ovary	PRS7	rs12252510	10	19418567	T	C	2.27E-04	PRS-CSx
Ovary	PRS7	rs12256853	10	70930360	G	A	-3.88E-04	PRS-CSx
Ovary	PRS7	rs12257763	10	70939542	C	T	-3.79E-04	PRS-CSx
Ovary	PRS7	rs12258211	10	67114387	T	C	-8.24E-04	PRS-CSx
Ovary	PRS7	rs12260719	10	30577451	C	T	1.10E-03	PRS-CSx
Ovary	PRS7	rs12274537	11	128287850	C	T	-1.60E-03	PRS-CSx
Ovary	PRS7	rs12320133	12	27551457	G	T	-1.00E-03	PRS-CSx
Ovary	PRS7	rs1233556	7	155600417	T	C	2.40E-03	PRS-CSx
Ovary	PRS7	rs12344554	9	92892359	G	A	1.27E-03	PRS-CSx
Ovary	PRS7	rs12346672	9	102868769	G	A	1.41E-03	PRS-CSx
Ovary	PRS7	rs123556	19	30635473	T	C	7.57E-04	PRS-CSx
Ovary	PRS7	rs12359459	10	91223352	G	A	-3.77E-03	PRS-CSx
Ovary	PRS7	rs12365345	11	46089776	C	T	-1.36E-04	PRS-CSx
Ovary	PRS7	rs12373773	2	146095468	C	T	1.08E-04	PRS-CSx

Ovary	PRS7	rs12376289	9	18542869	C	T	-1.64E-04	PRS-CSx
Ovary	PRS7	rs1239932	12	70400569	G	A	2.96E-04	PRS-CSx
Ovary	PRS7	rs12407843	1	156873727	A	G	-4.81E-04	PRS-CSx
Ovary	PRS7	rs12413520	10	98502188	C	T	-2.11E-03	PRS-CSx
Ovary	PRS7	rs12418173	11	112628269	G	A	-1.75E-05	PRS-CSx
Ovary	PRS7	rs12423537	12	88346009	T	G	-1.40E-04	PRS-CSx
Ovary	PRS7	rs12430684	13	91309432	C	T	-1.65E-03	PRS-CSx
Ovary	PRS7	rs12439845	15	41211827	T	C	-1.07E-03	PRS-CSx
Ovary	PRS7	rs12446272	16	6712186	G	A	-1.44E-04	PRS-CSx
Ovary	PRS7	rs12465373	2	224237681	T	G	-4.65E-04	PRS-CSx
Ovary	PRS7	rs12465952	2	201724360	G	A	-2.28E-04	PRS-CSx
Ovary	PRS7	rs12468477	2	50384213	T	G	7.80E-04	PRS-CSx
Ovary	PRS7	rs12468540	2	35448317	G	A	3.55E-04	PRS-CSx
Ovary	PRS7	rs12476568	2	41230862	G	A	-1.54E-03	PRS-CSx
Ovary	PRS7	rs12478958	2	11748037	G	A	-5.25E-04	PRS-CSx
Ovary	PRS7	rs12482307	21	32526525	T	C	3.30E-05	PRS-CSx
Ovary	PRS7	rs12485875	3	47096523	T	C	5.27E-05	PRS-CSx
Ovary	PRS7	rs12486952	3	67040146	A	C	3.72E-04	PRS-CSx
Ovary	PRS7	rs12487923	3	55002066	A	C	-1.83E-04	PRS-CSx
Ovary	PRS7	rs12489232	3	151775979	T	C	-1.48E-05	PRS-CSx
Ovary	PRS7	rs12489750	3	177654829	G	A	1.16E-03	PRS-CSx
Ovary	PRS7	rs12492409	3	177650442	A	G	1.12E-03	PRS-CSx
Ovary	PRS7	rs12494271	3	122585784	C	T	-1.12E-03	PRS-CSx
Ovary	PRS7	rs12496405	3	146520583	G	A	3.65E-04	PRS-CSx
Ovary	PRS7	rs12497387	3	177666644	A	G	1.22E-03	PRS-CSx
Ovary	PRS7	rs12505843	4	160299086	G	A	2.93E-04	PRS-CSx
Ovary	PRS7	rs12509242	4	108388646	A	G	-8.68E-04	PRS-CSx
Ovary	PRS7	rs12520925	5	6961853	A	G	1.58E-04	PRS-CSx
Ovary	PRS7	rs12521560	5	177511022	T	C	1.13E-03	PRS-CSx
Ovary	PRS7	rs12524863	6	17177322	T	C	-9.05E-04	PRS-CSx
Ovary	PRS7	rs12524885	6	45836069	T	C	3.71E-03	PRS-CSx
Ovary	PRS7	rs12528151	6	17177146	A	G	-8.45E-04	PRS-CSx
Ovary	PRS7	rs12530774	7	23914001	G	A	-2.23E-03	PRS-CSx
Ovary	PRS7	rs12532492	7	134565347	C	A	7.02E-04	PRS-CSx
Ovary	PRS7	rs12535925	7	129284182	G	A	7.17E-04	PRS-CSx
Ovary	PRS7	rs12536500	7	50808033	T	C	1.61E-04	PRS-CSx
Ovary	PRS7	rs12536691	7	53255862	C	T	7.49E-04	PRS-CSx
Ovary	PRS7	rs1253752	10	45007131	T	G	5.16E-05	PRS-CSx
Ovary	PRS7	rs1253784	11	66933410	A	C	2.40E-03	PRS-CSx
Ovary	PRS7	rs12538969	7	53250378	A	G	5.57E-04	PRS-CSx
Ovary	PRS7	rs12541086	8	48236907	T	C	3.06E-04	PRS-CSx
Ovary	PRS7	rs12541869	8	93403834	A	G	-1.12E-04	PRS-CSx
Ovary	PRS7	rs12549961	8	84393167	T	C	-7.62E-04	PRS-CSx
Ovary	PRS7	rs12553476	9	89633112	A	G	-1.22E-03	PRS-CSx
Ovary	PRS7	rs12569808	10	65464368	C	T	1.28E-03	PRS-CSx
Ovary	PRS7	rs12573535	10	84976430	T	C	1.07E-03	PRS-CSx
Ovary	PRS7	rs12579380	12	80771335	G	A	4.98E-04	PRS-CSx
Ovary	PRS7	rs12581297	12	43665319	C	A	-1.52E-04	PRS-CSx
Ovary	PRS7	rs12583114	13	98763904	G	A	-3.99E-05	PRS-CSx
Ovary	PRS7	rs12584186	13	91336218	A	G	-1.11E-03	PRS-CSx
Ovary	PRS7	rs12586353	14	27911708	C	A	-8.78E-04	PRS-CSx
Ovary	PRS7	rs12591132	15	99784210	G	A	-4.25E-04	PRS-CSx
Ovary	PRS7	rs12593201	15	38844106	A	G	-1.42E-04	PRS-CSx
Ovary	PRS7	rs12594507	15	71083063	C	T	-1.18E-03	PRS-CSx
Ovary	PRS7	rs12595720	15	56324451	C	T	-1.03E-04	PRS-CSx
Ovary	PRS7	rs12598715	16	80463399	A	G	-2.88E-05	PRS-CSx

Ovary	PRS7	rs12600441	17	70012436	G	T	9.86E-04	PRS-CSx
Ovary	PRS7	rs12603708	17	16847113	G	A	1.33E-03	PRS-CSx
Ovary	PRS7	rs12604758	18	57009769	C	T	6.58E-07	PRS-CSx
Ovary	PRS7	rs12607345	18	3475527	A	G	-3.05E-04	PRS-CSx
Ovary	PRS7	rs12613292	2	165455035	C	T	-1.26E-04	PRS-CSx
Ovary	PRS7	rs12614696	2	97118957	T	C	-1.33E-03	PRS-CSx
Ovary	PRS7	rs1261724	12	78360997	C	T	-9.54E-04	PRS-CSx
Ovary	PRS7	rs12617373	2	62831112	G	A	3.86E-03	PRS-CSx
Ovary	PRS7	rs12620887	2	31387929	C	A	-9.13E-05	PRS-CSx
Ovary	PRS7	rs12622464	2	59524395	T	C	2.30E-05	PRS-CSx
Ovary	PRS7	rs12622868	2	31390023	C	T	-4.02E-04	PRS-CSx
Ovary	PRS7	rs12625651	20	12657429	A	G	-2.40E-03	PRS-CSx
Ovary	PRS7	rs12631599	3	116706630	T	C	-5.28E-04	PRS-CSx
Ovary	PRS7	rs12632736	3	2347409	C	A	1.74E-04	PRS-CSx
Ovary	PRS7	rs12637071	3	3687381	G	A	-1.27E-03	PRS-CSx
Ovary	PRS7	rs12637941	3	171972663	G	A	-5.91E-04	PRS-CSx
Ovary	PRS7	rs12647849	4	112946958	T	C	-9.48E-04	PRS-CSx
Ovary	PRS7	rs12649808	4	112936890	G	A	-4.31E-04	PRS-CSx
Ovary	PRS7	rs1265004	10	105859279	T	C	-1.37E-04	PRS-CSx
Ovary	PRS7	rs12652447	5	15674635	G	A	-9.95E-04	PRS-CSx
Ovary	PRS7	rs12652642	5	125972624	G	T	5.15E-04	PRS-CSx
Ovary	PRS7	rs12654125	5	56215753	A	G	6.79E-05	PRS-CSx
Ovary	PRS7	rs12655019	5	56195790	G	A	-2.26E-04	PRS-CSx
Ovary	PRS7	rs12655861	5	125967653	A	G	3.90E-04	PRS-CSx
Ovary	PRS7	rs12660883	6	30764420	C	T	3.46E-04	PRS-CSx
Ovary	PRS7	rs12669123	7	175991	C	T	6.18E-04	PRS-CSx
Ovary	PRS7	rs12679472	8	5125700	C	T	2.21E-04	PRS-CSx
Ovary	PRS7	rs12683784	9	16884679	G	A	1.68E-03	PRS-CSx
Ovary	PRS7	rs12684385	9	116077694	G	T	-5.35E-04	PRS-CSx
Ovary	PRS7	rs12694027	2	206422140	T	C	3.83E-04	PRS-CSx
Ovary	PRS7	rs12700939	7	29192746	G	A	2.01E-04	PRS-CSx
Ovary	PRS7	rs12718723	7	52995767	T	G	-9.00E-04	PRS-CSx
Ovary	PRS7	rs12740035	1	115555312	T	C	1.52E-03	PRS-CSx
Ovary	PRS7	rs12767507	10	120361816	T	G	3.16E-03	PRS-CSx
Ovary	PRS7	rs12770017	10	130656497	T	C	-5.52E-03	PRS-CSx
Ovary	PRS7	rs1277831	11	83144593	T	C	1.56E-03	PRS-CSx
Ovary	PRS7	rs12778719	10	109579550	C	T	1.17E-03	PRS-CSx
Ovary	PRS7	rs12785570	11	112640338	T	C	5.18E-05	PRS-CSx
Ovary	PRS7	rs1279007	14	32519158	C	A	-6.22E-03	PRS-CSx
Ovary	PRS7	rs12797728	11	112617526	G	A	1.95E-04	PRS-CSx
Ovary	PRS7	rs12812230	12	41720543	C	A	8.85E-04	PRS-CSx
Ovary	PRS7	rs12859433	13	111667024	T	C	-3.81E-04	PRS-CSx
Ovary	PRS7	rs12885928	14	73372969	G	T	1.76E-04	PRS-CSx
Ovary	PRS7	rs12909011	15	50409125	A	G	5.77E-04	PRS-CSx
Ovary	PRS7	rs1292045	17	57947212	T	C	-8.11E-04	PRS-CSx
Ovary	PRS7	rs1292060	17	57913849	A	G	-4.42E-04	PRS-CSx
Ovary	PRS7	rs12922811	16	62959880	G	A	-5.36E-04	PRS-CSx
Ovary	PRS7	rs12923744	16	19355345	T	C	-1.63E-03	PRS-CSx
Ovary	PRS7	rs12927170	16	19361008	A	G	-1.36E-03	PRS-CSx
Ovary	PRS7	rs12945695	17	25956348	C	A	-1.83E-03	PRS-CSx
Ovary	PRS7	rs12948909	17	40570602	C	A	-6.04E-04	PRS-CSx
Ovary	PRS7	rs12953756	18	22659339	T	C	3.99E-03	PRS-CSx
Ovary	PRS7	rs12955584	18	10433382	A	G	1.21E-03	PRS-CSx
Ovary	PRS7	rs12957472	18	19954143	T	C	1.71E-03	PRS-CSx
Ovary	PRS7	rs1295907	14	32527520	T	C	-2.90E-03	PRS-CSx
Ovary	PRS7	rs1295925	17	57910263	C	T	-6.60E-04	PRS-CSx

Ovary	PRS7	rs12965806	18	19967504	C	T	2.25E-03	PRS-CSx
Ovary	PRS7	rs12966945	18	10435574	C	T	1.63E-03	PRS-CSx
Ovary	PRS7	rs12989378	2	182896104	A	G	1.96E-03	PRS-CSx
Ovary	PRS7	rs12992904	2	212237340	C	T	-3.56E-04	PRS-CSx
Ovary	PRS7	rs12997897	2	119695560	A	G	-2.54E-04	PRS-CSx
Ovary	PRS7	rs13002567	2	25465720	C	T	-9.68E-04	PRS-CSx
Ovary	PRS7	rs13007967	2	102732778	G	A	-1.15E-03	PRS-CSx
Ovary	PRS7	rs13012334	2	102736020	G	A	-7.86E-04	PRS-CSx
Ovary	PRS7	rs13019803	2	102776202	T	C	-1.50E-03	PRS-CSx
Ovary	PRS7	rs13020778	2	102784574	C	T	-9.74E-04	PRS-CSx
Ovary	PRS7	rs13021734	2	8118123	G	A	2.61E-03	PRS-CSx
Ovary	PRS7	rs13035227	2	102763837	C	T	-6.64E-04	PRS-CSx
Ovary	PRS7	rs13058814	3	60062636	A	C	9.84E-04	PRS-CSx
Ovary	PRS7	rs13061071	3	47138708	G	A	-2.87E-05	PRS-CSx
Ovary	PRS7	rs13063401	3	102946216	A	G	-6.48E-04	PRS-CSx
Ovary	PRS7	rs13067579	3	124370526	T	C	5.04E-04	PRS-CSx
Ovary	PRS7	rs13071097	3	102938744	C	A	-7.04E-04	PRS-CSx
Ovary	PRS7	rs13076885	3	122617445	T	C	-1.04E-03	PRS-CSx
Ovary	PRS7	rs13084117	3	47183239	C	A	7.81E-06	PRS-CSx
Ovary	PRS7	rs13099331	3	171967050	T	C	-4.19E-04	PRS-CSx
Ovary	PRS7	rs13103693	4	5735817	T	C	2.89E-04	PRS-CSx
Ovary	PRS7	rs13119846	4	154415184	T	C	-5.34E-05	PRS-CSx
Ovary	PRS7	rs13120014	4	110234264	A	G	-1.56E-04	PRS-CSx
Ovary	PRS7	rs13122226	4	8120120	C	T	2.62E-04	PRS-CSx
Ovary	PRS7	rs13124431	4	183071666	A	C	4.05E-03	PRS-CSx
Ovary	PRS7	rs13140239	4	98273255	G	A	-7.53E-04	PRS-CSx
Ovary	PRS7	rs13147033	4	59686485	T	C	1.12E-04	PRS-CSx
Ovary	PRS7	rs13147855	4	10691942	T	C	-2.56E-03	PRS-CSx
Ovary	PRS7	rs13149814	4	176370331	G	T	-1.16E-03	PRS-CSx
Ovary	PRS7	rs13157625	5	104367623	C	T	-1.28E-03	PRS-CSx
Ovary	PRS7	rs13161359	5	160475869	C	A	-1.45E-04	PRS-CSx
Ovary	PRS7	rs13176854	5	176010156	T	G	8.10E-04	PRS-CSx
Ovary	PRS7	rs13181346	5	65828056	T	C	1.04E-06	PRS-CSx
Ovary	PRS7	rs13189047	5	177578875	A	C	-2.40E-04	PRS-CSx
Ovary	PRS7	rs13190616	5	103425505	T	C	8.01E-04	PRS-CSx
Ovary	PRS7	rs13201386	6	90665317	T	C	-1.23E-03	PRS-CSx
Ovary	PRS7	rs13202506	6	118020102	G	T	-2.08E-03	PRS-CSx
Ovary	PRS7	rs132125	22	48070614	A	G	-6.01E-04	PRS-CSx
Ovary	PRS7	rs13212769	6	17302983	T	C	-4.36E-05	PRS-CSx
Ovary	PRS7	rs13225765	7	154125005	T	C	8.62E-04	PRS-CSx
Ovary	PRS7	rs13230379	7	71655407	C	T	2.75E-04	PRS-CSx
Ovary	PRS7	rs1323348	9	14494388	C	T	8.88E-04	PRS-CSx
Ovary	PRS7	rs1323815	1	58177003	T	C	-6.98E-05	PRS-CSx
Ovary	PRS7	rs13249563	8	20466541	T	C	1.91E-04	PRS-CSx
Ovary	PRS7	rs13253380	8	67052744	T	C	-1.36E-03	PRS-CSx
Ovary	PRS7	rs13259479	8	127675332	T	G	3.23E-03	PRS-CSx
Ovary	PRS7	rs13259872	8	48102786	C	T	2.69E-04	PRS-CSx
Ovary	PRS7	rs13269639	8	8517524	C	T	1.17E-03	PRS-CSx
Ovary	PRS7	rs13275957	8	20466105	A	G	-2.64E-04	PRS-CSx
Ovary	PRS7	rs13282248	8	67046982	T	C	-9.81E-04	PRS-CSx
Ovary	PRS7	rs13283164	9	91979438	G	A	-4.01E-04	PRS-CSx
Ovary	PRS7	rs1328606	13	70757536	C	T	-8.06E-04	PRS-CSx
Ovary	PRS7	rs1329803	8	132002576	G	A	-3.77E-04	PRS-CSx
Ovary	PRS7	rs13317725	3	151740395	A	G	4.96E-04	PRS-CSx
Ovary	PRS7	rs13347305	4	60013919	C	T	3.71E-04	PRS-CSx
Ovary	PRS7	rs13376856	10	70924619	G	T	-3.51E-04	PRS-CSx

Ovary	PRS7	rs13377620	12	52712495	A	G	-8.25E-04	PRS-CSx
Ovary	PRS7	rs13379960	15	57133357	A	G	5.27E-05	PRS-CSx
Ovary	PRS7	rs13385731	2	33701890	C	T	-4.97E-03	PRS-CSx
Ovary	PRS7	rs13387203	2	208902466	C	T	-4.01E-04	PRS-CSx
Ovary	PRS7	rs1338762	1	220596516	A	G	2.21E-03	PRS-CSx
Ovary	PRS7	rs1338777	10	105883395	C	A	1.93E-04	PRS-CSx
Ovary	PRS7	rs13388627	2	108883866	A	C	-5.65E-05	PRS-CSx
Ovary	PRS7	rs13400438	2	118881001	A	G	7.29E-05	PRS-CSx
Ovary	PRS7	rs13401731	2	108885340	C	T	-2.75E-04	PRS-CSx
Ovary	PRS7	rs1340452	9	85450078	A	G	1.79E-03	PRS-CSx
Ovary	PRS7	rs1340654	10	61998060	C	A	-5.72E-06	PRS-CSx
Ovary	PRS7	rs1341268	6	105643136	G	A	-3.55E-03	PRS-CSx
Ovary	PRS7	rs13421034	2	208892371	C	T	-2.55E-04	PRS-CSx
Ovary	PRS7	rs13425767	2	227342966	T	C	8.49E-04	PRS-CSx
Ovary	PRS7	rs13431908	2	111957594	T	C	1.09E-03	PRS-CSx
Ovary	PRS7	rs13434090	3	190524192	A	G	-3.11E-04	PRS-CSx
Ovary	PRS7	rs13434876	4	76762674	A	G	8.34E-05	PRS-CSx
Ovary	PRS7	rs13436770	5	83916464	T	C	3.06E-05	PRS-CSx
Ovary	PRS7	rs13436876	5	177556429	A	G	-8.28E-04	PRS-CSx
Ovary	PRS7	rs1345686	5	111807579	T	C	1.88E-04	PRS-CSx
Ovary	PRS7	rs1347226	12	76364611	G	A	-1.67E-05	PRS-CSx
Ovary	PRS7	rs1350166	12	69636314	T	G	4.03E-04	PRS-CSx
Ovary	PRS7	rs1350327	4	180126785	G	A	3.15E-04	PRS-CSx
Ovary	PRS7	rs1357959	7	53254366	C	T	8.68E-04	PRS-CSx
Ovary	PRS7	rs1359974	10	130394371	T	C	1.49E-03	PRS-CSx
Ovary	PRS7	rs1361409	1	246410112	T	C	-2.46E-04	PRS-CSx
Ovary	PRS7	rs1364096	16	79566041	T	C	6.96E-04	PRS-CSx
Ovary	PRS7	rs1364394	2	96195730	T	C	6.53E-04	PRS-CSx
Ovary	PRS7	rs136941	22	27801334	C	A	7.13E-04	PRS-CSx
Ovary	PRS7	rs136951	22	27808478	C	T	-1.13E-03	PRS-CSx
Ovary	PRS7	rs1371404	2	43040577	C	A	1.30E-03	PRS-CSx
Ovary	PRS7	rs1371507	2	35485393	A	G	-3.42E-05	PRS-CSx
Ovary	PRS7	rs1371509	2	35487688	T	C	2.11E-04	PRS-CSx
Ovary	PRS7	rs1372698	9	17429714	T	C	7.80E-05	PRS-CSx
Ovary	PRS7	rs1373340	3	66902287	T	G	8.94E-04	PRS-CSx
Ovary	PRS7	rs1376332	12	16253493	C	T	-5.46E-05	PRS-CSx
Ovary	PRS7	rs1376333	12	16253276	T	C	-2.21E-04	PRS-CSx
Ovary	PRS7	rs1376843	8	82887802	G	A	-1.82E-05	PRS-CSx
Ovary	PRS7	rs1378032	8	14944692	T	C	2.13E-05	PRS-CSx
Ovary	PRS7	rs1378936	15	97831767	T	C	2.62E-03	PRS-CSx
Ovary	PRS7	rs1380422	11	112609873	T	G	1.30E-04	PRS-CSx
Ovary	PRS7	rs138368	22	38790528	C	T	3.22E-03	PRS-CSx
Ovary	PRS7	rs1385965	6	120696495	A	G	-5.20E-04	PRS-CSx
Ovary	PRS7	rs1386364	4	135288617	T	C	-3.40E-04	PRS-CSx
Ovary	PRS7	rs1391767	17	8320988	A	G	-2.15E-04	PRS-CSx
Ovary	PRS7	rs1392696	3	96962733	G	A	6.64E-04	PRS-CSx
Ovary	PRS7	rs1397854	20	41465048	T	C	2.08E-03	PRS-CSx
Ovary	PRS7	rs1397942	4	184228248	G	A	3.09E-03	PRS-CSx
Ovary	PRS7	rs1399564	8	76483856	T	C	-2.81E-04	PRS-CSx
Ovary	PRS7	rs1399774	3	189586182	G	A	-1.18E-03	PRS-CSx
Ovary	PRS7	rs1402499	4	28928041	C	T	1.80E-03	PRS-CSx
Ovary	PRS7	rs1402965	3	179798283	C	T	-9.31E-04	PRS-CSx
Ovary	PRS7	rs1404889	7	53260410	G	A	5.31E-04	PRS-CSx
Ovary	PRS7	rs1408115	9	116809147	A	G	2.60E-04	PRS-CSx
Ovary	PRS7	rs1408299	6	17154680	G	A	-1.17E-03	PRS-CSx
Ovary	PRS7	rs1411179	8	132016011	G	A	-4.44E-05	PRS-CSx

Ovary	PRS7	rs1411180	8	132016686	T	C	-1.02E-04	PRS-CSx
Ovary	PRS7	rs1411187	8	132021963	G	A	-1.18E-04	PRS-CSx
Ovary	PRS7	rs1414128	10	2045821	C	T	2.18E-03	PRS-CSx
Ovary	PRS7	rs1417935	13	90661048	C	T	-9.91E-04	PRS-CSx
Ovary	PRS7	rs1421163	3	177636693	G	A	1.14E-03	PRS-CSx
Ovary	PRS7	rs1426679	18	36085089	C	T	6.64E-05	PRS-CSx
Ovary	PRS7	rs1431972	3	146437202	G	A	-4.06E-04	PRS-CSx
Ovary	PRS7	rs1434889	1	213511231	G	A	1.06E-04	PRS-CSx
Ovary	PRS7	rs1437895	2	133852801	G	A	2.08E-03	PRS-CSx
Ovary	PRS7	rs1441561	13	70593012	T	C	1.44E-04	PRS-CSx
Ovary	PRS7	rs1441875	3	140355104	G	A	1.89E-03	PRS-CSx
Ovary	PRS7	rs1445112	3	27351286	G	A	-3.29E-04	PRS-CSx
Ovary	PRS7	rs1445565	8	81845260	G	A	-1.29E-04	PRS-CSx
Ovary	PRS7	rs1446556	6	149637106	A	C	8.10E-04	PRS-CSx
Ovary	PRS7	rs1451019	7	33690186	T	G	4.23E-04	PRS-CSx
Ovary	PRS7	rs1453122	2	7219384	G	A	4.83E-04	PRS-CSx
Ovary	PRS7	rs1454093	3	84833513	A	G	-1.27E-03	PRS-CSx
Ovary	PRS7	rs1454590	8	14937638	A	G	1.45E-04	PRS-CSx
Ovary	PRS7	rs1454592	8	14935227	T	C	6.77E-06	PRS-CSx
Ovary	PRS7	rs1458388	11	44448938	T	C	-4.64E-04	PRS-CSx
Ovary	PRS7	rs1458999	8	113878100	A	G	3.70E-04	PRS-CSx
Ovary	PRS7	rs1461818	3	28800568	C	T	-1.70E-03	PRS-CSx
Ovary	PRS7	rs1462441	8	76768738	C	T	-1.91E-04	PRS-CSx
Ovary	PRS7	rs1463219	3	17301192	C	T	-2.22E-03	PRS-CSx
Ovary	PRS7	rs1463739	12	40959174	G	A	-9.96E-04	PRS-CSx
Ovary	PRS7	rs1464092	8	76483560	T	C	-4.50E-04	PRS-CSx
Ovary	PRS7	rs1465081	12	57050174	T	C	3.94E-04	PRS-CSx
Ovary	PRS7	rs1466180	2	169224527	A	G	4.57E-05	PRS-CSx
Ovary	PRS7	rs1466382	12	57116834	G	A	2.83E-04	PRS-CSx
Ovary	PRS7	rs1469049	5	120253083	T	C	-8.61E-04	PRS-CSx
Ovary	PRS7	rs1469573	18	38367283	C	A	5.62E-04	PRS-CSx
Ovary	PRS7	rs1470354	3	66914581	A	C	-9.84E-05	PRS-CSx
Ovary	PRS7	rs1472597	3	190530174	G	A	-4.67E-04	PRS-CSx
Ovary	PRS7	rs1480758	4	71103643	A	G	1.97E-03	PRS-CSx
Ovary	PRS7	rs1481966	11	103479435	C	T	-1.09E-04	PRS-CSx
Ovary	PRS7	rs1481975	11	103469531	C	T	2.25E-05	PRS-CSx
Ovary	PRS7	rs1485999	8	82419512	G	A	3.73E-04	PRS-CSx
Ovary	PRS7	rs1486004	8	82425347	C	T	1.81E-04	PRS-CSx
Ovary	PRS7	rs1487623	4	38377756	A	G	-2.05E-04	PRS-CSx
Ovary	PRS7	rs1488342	3	8090865	G	T	1.68E-03	PRS-CSx
Ovary	PRS7	rs1488402	3	3684720	T	G	-1.18E-03	PRS-CSx
Ovary	PRS7	rs1488403	3	3684246	A	G	-6.78E-04	PRS-CSx
Ovary	PRS7	rs1491411	4	175777871	C	T	-4.99E-04	PRS-CSx
Ovary	PRS7	rs149462	20	62223238	T	C	-1.85E-03	PRS-CSx
Ovary	PRS7	rs1498869	12	24166453	C	T	1.39E-03	PRS-CSx
Ovary	PRS7	rs1499370	8	129078467	G	A	-2.97E-03	PRS-CSx
Ovary	PRS7	rs1499374	8	129066074	C	T	-8.65E-04	PRS-CSx
Ovary	PRS7	rs1499433	8	118245643	C	T	-1.51E-03	PRS-CSx
Ovary	PRS7	rs1500700	3	96926548	A	G	1.28E-03	PRS-CSx
Ovary	PRS7	rs1503465	4	27744290	C	T	8.26E-04	PRS-CSx
Ovary	PRS7	rs1504606	14	57446703	C	T	-1.28E-03	PRS-CSx
Ovary	PRS7	rs1506566	12	52761959	T	C	9.49E-04	PRS-CSx
Ovary	PRS7	rs150858	17	3532670	T	C	3.00E-05	PRS-CSx
Ovary	PRS7	rs1511554	3	146423287	C	T	-3.10E-04	PRS-CSx
Ovary	PRS7	rs1511580	3	122897812	G	A	-3.64E-04	PRS-CSx
Ovary	PRS7	rs1515020	8	76477590	T	C	-9.43E-04	PRS-CSx

Ovary	PRS7	rs1516660	5	160412665	G	A	-5.19E-05	PRS-CSx
Ovary	PRS7	rs1516976	8	129548258	C	T	-6.52E-03	PRS-CSx
Ovary	PRS7	rs1517827	2	50377588	A	C	2.82E-04	PRS-CSx
Ovary	PRS7	rs1519541	2	12802363	C	T	-6.49E-04	PRS-CSx
Ovary	PRS7	rs1520058	3	20418421	C	A	-2.23E-03	PRS-CSx
Ovary	PRS7	rs1522118	3	179827381	A	G	-8.77E-04	PRS-CSx
Ovary	PRS7	rs1522122	3	179829075	A	C	-1.14E-03	PRS-CSx
Ovary	PRS7	rs1523358	5	98686197	C	T	-2.55E-04	PRS-CSx
Ovary	PRS7	rs1531460	8	76752696	G	A	-5.62E-04	PRS-CSx
Ovary	PRS7	rs1531461	8	76752763	C	T	-6.31E-04	PRS-CSx
Ovary	PRS7	rs1533299	2	111616141	T	C	1.17E-03	PRS-CSx
Ovary	PRS7	rs1534422	2	12640741	G	A	-8.61E-05	PRS-CSx
Ovary	PRS7	rs1535143	20	60170591	T	C	1.38E-04	PRS-CSx
Ovary	PRS7	rs153516	5	142979183	G	A	-9.20E-05	PRS-CSx
Ovary	PRS7	rs1535546	6	17176032	T	G	-1.05E-03	PRS-CSx
Ovary	PRS7	rs153677	16	65874365	C	T	1.18E-03	PRS-CSx
Ovary	PRS7	rs1537146	9	4859303	G	A	-6.56E-05	PRS-CSx
Ovary	PRS7	rs154035	5	169009060	A	G	5.92E-05	PRS-CSx
Ovary	PRS7	rs154256	5	55819302	G	T	2.05E-03	PRS-CSx
Ovary	PRS7	rs1545099	16	9508047	T	C	1.09E-03	PRS-CSx
Ovary	PRS7	rs1545240	8	135642632	A	C	3.84E-04	PRS-CSx
Ovary	PRS7	rs1545503	2	208903820	T	C	-4.12E-04	PRS-CSx
Ovary	PRS7	rs1547761	9	16918196	C	T	-6.30E-04	PRS-CSx
Ovary	PRS7	rs1552420	12	129657564	A	G	3.37E-03	PRS-CSx
Ovary	PRS7	rs1553497	17	31756983	T	G	-1.42E-03	PRS-CSx
Ovary	PRS7	rs1554849	4	99474774	A	G	-7.56E-05	PRS-CSx
Ovary	PRS7	rs155504	6	7951721	C	T	-1.96E-05	PRS-CSx
Ovary	PRS7	rs155506	6	7952916	C	T	-1.64E-04	PRS-CSx
Ovary	PRS7	rs1558699	7	78092652	G	A	1.97E-03	PRS-CSx
Ovary	PRS7	rs1559714	14	70669002	A	G	-6.92E-04	PRS-CSx
Ovary	PRS7	rs1561927	8	129568078	C	T	-1.12E-03	PRS-CSx
Ovary	PRS7	rs1562608	2	177527873	G	A	-2.80E-04	PRS-CSx
Ovary	PRS7	rs1570541	13	76074865	C	T	-7.08E-04	PRS-CSx
Ovary	PRS7	rs1571317	13	37484693	C	T	-2.61E-04	PRS-CSx
Ovary	PRS7	rs1571376	14	70672989	T	C	-6.62E-04	PRS-CSx
Ovary	PRS7	rs1572202	10	45010160	A	G	-3.85E-05	PRS-CSx
Ovary	PRS7	rs1574108	7	1105805	C	T	-6.72E-04	PRS-CSx
Ovary	PRS7	rs1575067	1	115563866	G	A	2.24E-03	PRS-CSx
Ovary	PRS7	rs1578463	9	102464577	T	C	2.55E-04	PRS-CSx
Ovary	PRS7	rs1591184	13	59364557	G	A	-2.16E-04	PRS-CSx
Ovary	PRS7	rs1597575	12	16256103	G	T	-1.92E-04	PRS-CSx
Ovary	PRS7	rs1598083	3	84809350	C	T	-9.71E-04	PRS-CSx
Ovary	PRS7	rs1599505	11	37651249	T	C	1.02E-04	PRS-CSx
Ovary	PRS7	rs1599751	12	58242849	T	C	-1.00E-04	PRS-CSx
Ovary	PRS7	rs1601648	4	135294098	C	T	-6.30E-04	PRS-CSx
Ovary	PRS7	rs1604870	10	67112930	T	C	-4.39E-04	PRS-CSx
Ovary	PRS7	rs161558	5	143203979	C	T	-1.61E-04	PRS-CSx
Ovary	PRS7	rs1621663	8	107462505	G	A	-3.89E-05	PRS-CSx
Ovary	PRS7	rs1624395	12	66618216	G	A	-8.32E-04	PRS-CSx
Ovary	PRS7	rs1626074	3	99584429	A	G	2.24E-04	PRS-CSx
Ovary	PRS7	rs1628326	12	78360876	C	A	-6.26E-04	PRS-CSx
Ovary	PRS7	rs1629339	15	57171430	A	C	-1.61E-04	PRS-CSx
Ovary	PRS7	rs1630253	1	246423695	C	T	2.68E-05	PRS-CSx
Ovary	PRS7	rs1638676	3	47327651	A	G	-1.04E-04	PRS-CSx
Ovary	PRS7	rs163888	5	106889936	T	C	-1.02E-03	PRS-CSx
Ovary	PRS7	rs1652748	15	57170911	T	C	2.64E-05	PRS-CSx

Ovary	PRS7	rs1657922	15	57178132	C	T	4.21E-09	PRS-CSx
Ovary	PRS7	rs1657934	15	57113810	A	G	-7.78E-06	PRS-CSx
Ovary	PRS7	rs1657938	15	57109533	G	A	6.33E-05	PRS-CSx
Ovary	PRS7	rs1671169	19	55573633	A	G	7.19E-05	PRS-CSx
Ovary	PRS7	rs1672606	4	45550776	A	G	-1.91E-03	PRS-CSx
Ovary	PRS7	rs1673459	2	65810588	C	A	1.21E-03	PRS-CSx
Ovary	PRS7	rs167628	5	41385427	T	C	-6.73E-04	PRS-CSx
Ovary	PRS7	rs16826199	3	156170443	G	A	5.78E-04	PRS-CSx
Ovary	PRS7	rs16826658	1	22485871	G	T	1.14E-03	PRS-CSx
Ovary	PRS7	rs16828811	3	177664921	G	T	7.42E-04	PRS-CSx
Ovary	PRS7	rs16838468	3	96931836	T	C	1.23E-03	PRS-CSx
Ovary	PRS7	rs16841920	3	99783920	C	T	1.05E-03	PRS-CSx
Ovary	PRS7	rs16843928	1	234865288	A	G	-4.30E-04	PRS-CSx
Ovary	PRS7	rs16845236	3	171985304	T	G	-6.74E-04	PRS-CSx
Ovary	PRS7	rs16847317	2	133841199	C	T	-1.94E-03	PRS-CSx
Ovary	PRS7	rs16862648	3	150155120	C	T	-7.91E-04	PRS-CSx
Ovary	PRS7	rs16867844	4	27922256	T	G	1.41E-04	PRS-CSx
Ovary	PRS7	rs16869310	6	49615486	T	C	5.39E-04	PRS-CSx
Ovary	PRS7	rs168705	5	144195653	G	A	-6.14E-05	PRS-CSx
Ovary	PRS7	rs16872714	5	4048841	C	T	-7.63E-04	PRS-CSx
Ovary	PRS7	rs1687626	16	86382120	G	A	-2.39E-04	PRS-CSx
Ovary	PRS7	rs16879521	6	49583871	A	G	9.41E-05	PRS-CSx
Ovary	PRS7	rs168843	5	25739983	A	G	3.14E-05	PRS-CSx
Ovary	PRS7	rs16886496	5	56217529	C	T	2.79E-04	PRS-CSx
Ovary	PRS7	rs16886510	5	56228445	A	G	8.03E-05	PRS-CSx
Ovary	PRS7	rs16886525	5	56237665	A	C	-2.43E-04	PRS-CSx
Ovary	PRS7	rs16892545	4	15890917	G	A	-6.60E-04	PRS-CSx
Ovary	PRS7	rs16898889	8	124820411	T	C	1.78E-03	PRS-CSx
Ovary	PRS7	rs16903097	8	129556356	G	T	-4.70E-03	PRS-CSx
Ovary	PRS7	rs16909244	8	82411481	A	G	3.74E-04	PRS-CSx
Ovary	PRS7	rs16912118	9	125455549	C	T	-9.82E-04	PRS-CSx
Ovary	PRS7	rs16912256	9	125591426	T	C	9.25E-04	PRS-CSx
Ovary	PRS7	rs16912642	11	24525499	A	C	3.86E-03	PRS-CSx
Ovary	PRS7	rs16912674	11	24540890	A	G	2.52E-03	PRS-CSx
Ovary	PRS7	rs16918784	10	65449618	C	A	1.88E-03	PRS-CSx
Ovary	PRS7	rs16918794	10	65461006	C	T	1.60E-03	PRS-CSx
Ovary	PRS7	rs16918796	10	65463446	T	C	1.14E-03	PRS-CSx
Ovary	PRS7	rs16918808	10	65470912	C	T	1.33E-03	PRS-CSx
Ovary	PRS7	rs16920998	8	55907794	T	C	-1.19E-03	PRS-CSx
Ovary	PRS7	rs16921013	8	55912151	G	A	-1.27E-03	PRS-CSx
Ovary	PRS7	rs16930078	9	117317441	A	G	7.44E-04	PRS-CSx
Ovary	PRS7	rs16931937	12	27548191	C	T	-1.91E-03	PRS-CSx
Ovary	PRS7	rs16932806	9	116100368	C	T	-1.39E-04	PRS-CSx
Ovary	PRS7	rs16934273	9	16218382	A	G	1.33E-03	PRS-CSx
Ovary	PRS7	rs16934357	9	37676472	T	C	5.81E-04	PRS-CSx
Ovary	PRS7	rs16934799	10	79208056	C	A	4.24E-04	PRS-CSx
Ovary	PRS7	rs16936700	10	36421152	A	G	-8.34E-04	PRS-CSx
Ovary	PRS7	rs16943205	18	2412278	G	A	1.43E-03	PRS-CSx
Ovary	PRS7	rs16948321	17	45873200	G	T	3.66E-03	PRS-CSx
Ovary	PRS7	rs16951591	13	99293141	T	G	8.36E-05	PRS-CSx
Ovary	PRS7	rs16953272	16	80456733	C	T	-1.89E-04	PRS-CSx
Ovary	PRS7	rs16953285	16	80459551	T	C	-7.25E-05	PRS-CSx
Ovary	PRS7	rs16954683	18	9020352	T	C	-4.13E-03	PRS-CSx
Ovary	PRS7	rs16955490	18	9743635	A	G	-3.36E-03	PRS-CSx
Ovary	PRS7	rs16958589	17	9590409	T	C	7.12E-04	PRS-CSx
Ovary	PRS7	rs16966848	15	33497397	C	T	-1.14E-03	PRS-CSx

Ovary	PRS7	rs16981330	20	19915251	T	G	4.70E-04	PRS-CSx
Ovary	PRS7	rs16981333	20	19916144	C	T	3.60E-04	PRS-CSx
Ovary	PRS7	rs16982013	20	57055152	G	A	6.93E-03	PRS-CSx
Ovary	PRS7	rs16994693	19	19993293	A	G	-1.48E-03	PRS-CSx
Ovary	PRS7	rs16999772	20	53173157	C	A	1.25E-03	PRS-CSx
Ovary	PRS7	rs17013001	2	33674504	G	A	-2.10E-03	PRS-CSx
Ovary	PRS7	rs17016392	2	129236747	G	A	-3.12E-04	PRS-CSx
Ovary	PRS7	rs17018637	3	26803617	T	C	-4.64E-04	PRS-CSx
Ovary	PRS7	rs17026173	1	111141410	T	C	-1.49E-03	PRS-CSx
Ovary	PRS7	rs17032033	12	102286790	C	T	3.33E-04	PRS-CSx
Ovary	PRS7	rs17035982	2	108886171	T	C	-1.44E-04	PRS-CSx
Ovary	PRS7	rs17037102	4	107845794	T	C	-5.63E-04	PRS-CSx
Ovary	PRS7	rs17039932	2	50358840	A	G	4.56E-04	PRS-CSx
Ovary	PRS7	rs17039940	2	50359270	T	C	6.56E-04	PRS-CSx
Ovary	PRS7	rs17043603	2	53088117	T	G	-8.83E-04	PRS-CSx
Ovary	PRS7	rs17043814	2	53143562	G	A	-1.03E-03	PRS-CSx
Ovary	PRS7	rs17043972	3	66907870	T	C	1.96E-03	PRS-CSx
Ovary	PRS7	rs17050030	2	59484780	C	T	-1.09E-03	PRS-CSx
Ovary	PRS7	rs17050128	2	59566809	T	C	-3.58E-04	PRS-CSx
Ovary	PRS7	rs17050219	2	121257128	T	C	4.35E-03	PRS-CSx
Ovary	PRS7	rs17057302	6	97494739	T	C	2.89E-03	PRS-CSx
Ovary	PRS7	rs17058591	5	160399249	A	G	1.07E-04	PRS-CSx
Ovary	PRS7	rs170602	20	56019162	C	T	-1.11E-03	PRS-CSx
Ovary	PRS7	rs17065455	13	76569664	C	T	-2.82E-04	PRS-CSx
Ovary	PRS7	rs17075926	8	5926872	A	C	8.15E-04	PRS-CSx
Ovary	PRS7	rs17078347	5	176017639	A	C	1.08E-03	PRS-CSx
Ovary	PRS7	rs17089447	4	60036914	G	A	5.31E-04	PRS-CSx
Ovary	PRS7	rs17092622	14	57450349	T	G	-2.38E-03	PRS-CSx
Ovary	PRS7	rs17097485	1	70492884	G	T	-3.54E-04	PRS-CSx
Ovary	PRS7	rs17100386	14	74926792	C	T	-1.74E-03	PRS-CSx
Ovary	PRS7	rs17105040	1	81029179	C	T	-5.34E-04	PRS-CSx
Ovary	PRS7	rs17105094	1	81048805	C	T	-1.95E-04	PRS-CSx
Ovary	PRS7	rs17109223	14	71771425	T	C	-1.33E-03	PRS-CSx
Ovary	PRS7	rs17115439	11	113264272	T	C	1.18E-04	PRS-CSx
Ovary	PRS7	rs17133145	7	3228573	G	A	-5.53E-04	PRS-CSx
Ovary	PRS7	rs17133289	7	3451138	C	A	1.09E-03	PRS-CSx
Ovary	PRS7	rs17133292	7	3455249	T	G	8.74E-04	PRS-CSx
Ovary	PRS7	rs17133789	6	209462	T	G	-2.61E-05	PRS-CSx
Ovary	PRS7	rs17134002	7	50808087	A	G	5.61E-04	PRS-CSx
Ovary	PRS7	rs17164772	7	92380438	G	A	5.22E-04	PRS-CSx
Ovary	PRS7	rs17166480	7	132137171	G	A	-1.19E-03	PRS-CSx
Ovary	PRS7	rs1717839	15	23973973	C	T	8.87E-05	PRS-CSx
Ovary	PRS7	rs17191369	6	114440289	A	G	1.49E-03	PRS-CSx
Ovary	PRS7	rs17197676	14	22481451	G	A	4.72E-04	PRS-CSx
Ovary	PRS7	rs17213476	5	66074579	G	A	-5.97E-06	PRS-CSx
Ovary	PRS7	rs17214697	5	66118105	G	A	-9.92E-05	PRS-CSx
Ovary	PRS7	rs17227124	6	118741382	C	T	-2.63E-04	PRS-CSx
Ovary	PRS7	rs17237611	6	100278425	G	A	-5.40E-03	PRS-CSx
Ovary	PRS7	rs17248579	5	14594377	C	T	-1.18E-03	PRS-CSx
Ovary	PRS7	rs17256961	5	67776535	C	T	-1.32E-03	PRS-CSx
Ovary	PRS7	rs17258639	5	67891771	A	G	-3.57E-03	PRS-CSx
Ovary	PRS7	rs17263395	4	174579326	C	T	-1.63E-04	PRS-CSx
Ovary	PRS7	rs17265109	9	31630235	C	T	-3.54E-03	PRS-CSx
Ovary	PRS7	rs17276524	13	19608264	C	A	-3.45E-03	PRS-CSx
Ovary	PRS7	rs17277606	14	21202633	T	G	-2.14E-03	PRS-CSx
Ovary	PRS7	rs1728369	16	86386213	C	A	-1.99E-04	PRS-CSx

Ovary	PRS7	rs172862	9	9032872	C	T	-2.28E-03	PRS-CSx
Ovary	PRS7	rs17288586	6	45819770	A	G	2.25E-03	PRS-CSx
Ovary	PRS7	rs17322850	3	60022109	T	C	1.31E-03	PRS-CSx
Ovary	PRS7	rs17336700	16	74335204	C	T	1.62E-04	PRS-CSx
Ovary	PRS7	rs17345754	1	7141327	C	T	-2.02E-03	PRS-CSx
Ovary	PRS7	rs17356059	1	22531553	T	C	-6.09E-04	PRS-CSx
Ovary	PRS7	rs17368880	1	179276195	A	G	1.09E-04	PRS-CSx
Ovary	PRS7	rs17407080	10	45011363	C	T	2.24E-04	PRS-CSx
Ovary	PRS7	rs17411375	3	47395135	T	C	-8.48E-05	PRS-CSx
Ovary	PRS7	rs17423910	1	66689109	G	A	-1.52E-03	PRS-CSx
Ovary	PRS7	rs17445269	9	92891865	A	G	-3.35E-03	PRS-CSx
Ovary	PRS7	rs17446042	13	108441044	C	A	7.16E-05	PRS-CSx
Ovary	PRS7	rs17446320	13	40765144	C	T	-1.11E-03	PRS-CSx
Ovary	PRS7	rs17447618	19	3797612	T	C	-3.53E-03	PRS-CSx
Ovary	PRS7	rs17463487	1	90625731	G	T	9.18E-06	PRS-CSx
Ovary	PRS7	rs17469499	10	24623632	C	T	2.53E-03	PRS-CSx
Ovary	PRS7	rs17476325	5	159865968	C	T	7.23E-04	PRS-CSx
Ovary	PRS7	rs17477697	14	32534297	A	G	-2.06E-03	PRS-CSx
Ovary	PRS7	rs17485517	10	45011597	G	A	1.90E-05	PRS-CSx
Ovary	PRS7	rs17492667	6	110726236	A	G	4.57E-04	PRS-CSx
Ovary	PRS7	rs1750792	10	738630	T	C	-4.38E-04	PRS-CSx
Ovary	PRS7	rs17515243	14	33112870	C	T	-1.30E-05	PRS-CSx
Ovary	PRS7	rs17524024	2	170365377	C	T	2.10E-04	PRS-CSx
Ovary	PRS7	rs17524893	1	74581311	T	C	3.55E-05	PRS-CSx
Ovary	PRS7	rs17530810	13	40767717	A	G	-4.94E-04	PRS-CSx
Ovary	PRS7	rs17547161	4	132932804	G	A	3.20E-03	PRS-CSx
Ovary	PRS7	rs17547292	4	132938331	G	A	2.52E-03	PRS-CSx
Ovary	PRS7	rs17555250	8	29427765	C	T	-4.08E-06	PRS-CSx
Ovary	PRS7	rs17596765	13	33739427	G	T	-2.87E-04	PRS-CSx
Ovary	PRS7	rs17601515	3	172245403	G	A	-1.20E-03	PRS-CSx
Ovary	PRS7	rs17606924	3	3104951	G	A	3.82E-03	PRS-CSx
Ovary	PRS7	rs17609991	17	75029002	C	T	2.11E-04	PRS-CSx
Ovary	PRS7	rs17631074	13	31902042	C	T	3.41E-04	PRS-CSx
Ovary	PRS7	rs17650643	6	12173498	C	T	-7.53E-03	PRS-CSx
Ovary	PRS7	rs1766531	6	97576033	C	T	2.75E-05	PRS-CSx
Ovary	PRS7	rs17665477	16	6700889	C	T	-3.11E-05	PRS-CSx
Ovary	PRS7	rs17665667	16	6706076	T	C	1.90E-05	PRS-CSx
Ovary	PRS7	rs17665814	16	6707584	T	C	-7.28E-05	PRS-CSx
Ovary	PRS7	rs17668717	7	53239545	T	C	1.10E-03	PRS-CSx
Ovary	PRS7	rs17673882	7	71654175	C	A	-4.97E-05	PRS-CSx
Ovary	PRS7	rs17673922	7	71656002	T	C	3.60E-04	PRS-CSx
Ovary	PRS7	rs1767845	20	58385870	A	C	3.23E-05	PRS-CSx
Ovary	PRS7	rs17681310	6	6499558	A	G	9.00E-05	PRS-CSx
Ovary	PRS7	rs1770011	1	246425984	A	G	-2.57E-04	PRS-CSx
Ovary	PRS7	rs17702625	15	92883303	A	C	-5.24E-04	PRS-CSx
Ovary	PRS7	rs17707869	17	3540042	A	G	-2.66E-04	PRS-CSx
Ovary	PRS7	rs1771445	10	131203805	A	G	2.82E-04	PRS-CSx
Ovary	PRS7	rs17715902	5	134451465	A	G	2.64E-04	PRS-CSx
Ovary	PRS7	rs17722228	16	6709919	C	T	-1.25E-04	PRS-CSx
Ovary	PRS7	rs17724918	14	58124113	G	A	1.55E-03	PRS-CSx
Ovary	PRS7	rs17732496	18	9887857	A	G	-1.68E-03	PRS-CSx
Ovary	PRS7	rs17755066	14	78740186	G	A	-3.41E-04	PRS-CSx
Ovary	PRS7	rs17763934	9	116045088	G	A	-1.01E-03	PRS-CSx
Ovary	PRS7	rs17798175	3	67032596	A	G	3.14E-05	PRS-CSx
Ovary	PRS7	rs1779993	1	162817494	A	C	-8.58E-05	PRS-CSx
Ovary	PRS7	rs17801135	8	13173319	G	A	-1.39E-03	PRS-CSx

Ovary	PRS7	rs17805412	18	9884503	A	G	-3.35E-03	PRS-CSx
Ovary	PRS7	rs17806568	20	51341501	C	A	-8.80E-04	PRS-CSx
Ovary	PRS7	rs17809906	3	20755232	A	C	4.40E-04	PRS-CSx
Ovary	PRS7	rs17823433	6	112505657	A	C	-6.66E-05	PRS-CSx
Ovary	PRS7	rs17825393	6	118772583	C	T	-4.86E-04	PRS-CSx
Ovary	PRS7	rs17830589	18	54675357	T	G	-2.11E-03	PRS-CSx
Ovary	PRS7	rs178490	14	58526602	C	T	1.55E-03	PRS-CSx
Ovary	PRS7	rs178493	14	58527515	G	A	1.17E-03	PRS-CSx
Ovary	PRS7	rs1789967	8	107457255	C	A	-3.28E-05	PRS-CSx
Ovary	PRS7	rs1789973	8	107468418	C	T	-5.21E-06	PRS-CSx
Ovary	PRS7	rs1794275	6	32671248	A	G	-1.22E-03	PRS-CSx
Ovary	PRS7	rs1800651	9	71651605	G	A	5.76E-04	PRS-CSx
Ovary	PRS7	rs1805100	8	76476396	A	G	-7.11E-04	PRS-CSx
Ovary	PRS7	rs1806626	7	129329673	G	A	7.67E-04	PRS-CSx
Ovary	PRS7	rs1815687	1	120377129	A	C	-1.29E-03	PRS-CSx
Ovary	PRS7	rs1820460	12	107704705	C	A	1.82E-03	PRS-CSx
Ovary	PRS7	rs1823013	5	13142487	T	C	7.26E-04	PRS-CSx
Ovary	PRS7	rs1829139	3	76176441	A	G	2.31E-05	PRS-CSx
Ovary	PRS7	rs1830317	2	145856863	A	C	-7.30E-04	PRS-CSx
Ovary	PRS7	rs1832845	10	29233160	A	G	8.23E-05	PRS-CSx
Ovary	PRS7	rs184288	13	62927507	C	T	6.87E-05	PRS-CSx
Ovary	PRS7	rs1860787	7	31918917	A	C	-9.51E-04	PRS-CSx
Ovary	PRS7	rs1861032	7	8268155	C	T	-3.02E-03	PRS-CSx
Ovary	PRS7	rs1863306	4	141812638	C	T	-1.59E-03	PRS-CSx
Ovary	PRS7	rs1863307	4	141812645	A	G	1.00E-03	PRS-CSx
Ovary	PRS7	rs1865365	15	79553571	A	G	1.59E-04	PRS-CSx
Ovary	PRS7	rs1865574	2	11681438	A	G	-7.71E-04	PRS-CSx
Ovary	PRS7	rs1865930	15	62385315	A	C	-4.52E-06	PRS-CSx
Ovary	PRS7	rs1866297	12	104139649	C	T	2.83E-04	PRS-CSx
Ovary	PRS7	rs1870174	10	48428267	G	A	-4.23E-04	PRS-CSx
Ovary	PRS7	rs1870340	2	97116953	G	A	-1.98E-03	PRS-CSx
Ovary	PRS7	rs1870494	11	112567775	C	T	2.39E-04	PRS-CSx
Ovary	PRS7	rs1870496	11	112597566	C	T	4.17E-04	PRS-CSx
Ovary	PRS7	rs1873026	3	3687827	G	A	-2.10E-03	PRS-CSx
Ovary	PRS7	rs1874158	15	58340072	C	T	-7.99E-05	PRS-CSx
Ovary	PRS7	rs1877259	3	146405051	C	A	-6.23E-04	PRS-CSx
Ovary	PRS7	rs1878519	4	8110782	T	C	-1.25E-04	PRS-CSx
Ovary	PRS7	rs1882240	2	67339603	A	G	1.90E-03	PRS-CSx
Ovary	PRS7	rs1882820	14	78688103	A	G	1.15E-03	PRS-CSx
Ovary	PRS7	rs1884619	14	33129814	C	T	1.00E-04	PRS-CSx
Ovary	PRS7	rs1891231	1	240315515	A	G	1.54E-03	PRS-CSx
Ovary	PRS7	rs1891375	10	112001009	T	C	2.57E-04	PRS-CSx
Ovary	PRS7	rs1891733	1	164807830	A	G	1.92E-03	PRS-CSx
Ovary	PRS7	rs1892124	1	162816583	G	A	-1.57E-06	PRS-CSx
Ovary	PRS7	rs1892756	8	102660160	C	T	-4.62E-04	PRS-CSx
Ovary	PRS7	rs1894111	11	67038612	T	C	1.38E-03	PRS-CSx
Ovary	PRS7	rs1902408	10	33873361	T	C	-1.32E-03	PRS-CSx
Ovary	PRS7	rs1902763	12	52657665	T	C	-6.20E-04	PRS-CSx
Ovary	PRS7	rs1902764	12	52657399	T	C	-6.48E-04	PRS-CSx
Ovary	PRS7	rs1912785	3	146402171	A	G	-1.01E-03	PRS-CSx
Ovary	PRS7	rs1915220	2	50443600	G	A	5.14E-04	PRS-CSx
Ovary	PRS7	rs1927693	6	25414846	A	G	-1.34E-04	PRS-CSx
Ovary	PRS7	rs1930777	9	123641168	A	G	-2.48E-04	PRS-CSx
Ovary	PRS7	rs1933758	6	130887052	A	C	-9.21E-04	PRS-CSx
Ovary	PRS7	rs1935465	10	112029300	G	A	2.35E-04	PRS-CSx
Ovary	PRS7	rs1937318	20	60136084	A	G	-2.25E-04	PRS-CSx

Ovary	PRS7	rs1942417	18	47344725	A	C	6.31E-04	PRS-CSx
Ovary	PRS7	rs1946167	3	177668941	G	A	6.83E-04	PRS-CSx
Ovary	PRS7	rs1956223	14	33082029	T	C	7.74E-05	PRS-CSx
Ovary	PRS7	rs1959607	14	20452460	C	T	7.24E-03	PRS-CSx
Ovary	PRS7	rs1962047	12	58292707	G	A	-2.42E-04	PRS-CSx
Ovary	PRS7	rs1962486	2	227239934	C	T	9.62E-04	PRS-CSx
Ovary	PRS7	rs1965112	4	54486795	C	T	-6.64E-05	PRS-CSx
Ovary	PRS7	rs1965239	2	25729721	G	A	1.16E-03	PRS-CSx
Ovary	PRS7	rs1965391	2	34237472	G	T	-2.59E-03	PRS-CSx
Ovary	PRS7	rs1965753	9	116799926	C	T	3.50E-04	PRS-CSx
Ovary	PRS7	rs1968162	19	20071675	T	C	-1.44E-03	PRS-CSx
Ovary	PRS7	rs197833	3	144065468	A	G	-2.92E-04	PRS-CSx
Ovary	PRS7	rs1979187	2	7230988	T	C	4.63E-04	PRS-CSx
Ovary	PRS7	rs1981390	21	36125233	A	G	1.26E-03	PRS-CSx
Ovary	PRS7	rs1981581	7	10620231	G	T	-3.75E-04	PRS-CSx
Ovary	PRS7	rs1985606	4	56207711	C	T	-1.95E-04	PRS-CSx
Ovary	PRS7	rs1989272	3	140328825	A	G	1.79E-03	PRS-CSx
Ovary	PRS7	rs1990224	7	12615698	T	C	2.00E-04	PRS-CSx
Ovary	PRS7	rs1990225	7	12615772	C	A	1.15E-04	PRS-CSx
Ovary	PRS7	rs1991088	15	71004450	T	C	-3.75E-04	PRS-CSx
Ovary	PRS7	rs1991332	3	25920214	T	C	-5.37E-04	PRS-CSx
Ovary	PRS7	rs1992249	2	129230661	T	G	-4.21E-04	PRS-CSx
Ovary	PRS7	rs1993800	4	98249865	A	G	-3.42E-04	PRS-CSx
Ovary	PRS7	rs1994700	4	175791268	C	T	-4.25E-04	PRS-CSx
Ovary	PRS7	rs1996399	7	71652312	A	G	3.72E-04	PRS-CSx
Ovary	PRS7	rs199949	1	181626346	T	C	-1.35E-03	PRS-CSx
Ovary	PRS7	rs2002275	12	58283385	T	C	-2.53E-04	PRS-CSx
Ovary	PRS7	rs2002384	1	110698651	G	A	-4.74E-04	PRS-CSx
Ovary	PRS7	rs2007343	20	60158929	T	C	1.52E-04	PRS-CSx
Ovary	PRS7	rs2011249	11	113768638	A	G	1.16E-03	PRS-CSx
Ovary	PRS7	rs2011511	4	20870791	T	G	7.58E-06	PRS-CSx
Ovary	PRS7	rs2011527	8	129555532	C	T	-4.79E-03	PRS-CSx
Ovary	PRS7	rs2014842	22	39709009	G	A	5.96E-04	PRS-CSx
Ovary	PRS7	rs2019199	9	128798584	T	C	1.96E-03	PRS-CSx
Ovary	PRS7	rs2024488	2	217662968	G	A	1.48E-04	PRS-CSx
Ovary	PRS7	rs2028556	8	135648040	T	C	6.85E-04	PRS-CSx
Ovary	PRS7	rs2028557	8	135648397	G	T	2.89E-04	PRS-CSx
Ovary	PRS7	rs2028677	2	31404001	C	T	-1.12E-04	PRS-CSx
Ovary	PRS7	rs2031236	13	76169777	A	G	-5.58E-04	PRS-CSx
Ovary	PRS7	rs2032828	19	35415021	C	T	3.69E-04	PRS-CSx
Ovary	PRS7	rs2033103	18	36097882	T	C	-7.80E-04	PRS-CSx
Ovary	PRS7	rs203340	12	120236482	T	C	-1.57E-03	PRS-CSx
Ovary	PRS7	rs2035081	12	57125879	A	G	3.27E-04	PRS-CSx
Ovary	PRS7	rs2036737	18	8998614	C	T	-2.12E-04	PRS-CSx
Ovary	PRS7	rs2042248	5	169031218	G	T	1.11E-04	PRS-CSx
Ovary	PRS7	rs2049504	3	143237419	T	C	1.77E-03	PRS-CSx
Ovary	PRS7	rs2054267	11	112634626	A	G	1.68E-05	PRS-CSx
Ovary	PRS7	rs2054724	12	94642865	A	C	-4.29E-04	PRS-CSx
Ovary	PRS7	rs2058123	14	74932776	A	G	-2.37E-03	PRS-CSx
Ovary	PRS7	rs2062009	8	93420294	G	A	2.43E-05	PRS-CSx
Ovary	PRS7	rs2065391	13	104373191	C	T	-2.23E-03	PRS-CSx
Ovary	PRS7	rs2065412	9	107598740	C	T	-2.95E-03	PRS-CSx
Ovary	PRS7	rs2065875	10	95337247	T	C	-1.04E-03	PRS-CSx
Ovary	PRS7	rs2066618	13	50752690	A	G	-1.49E-03	PRS-CSx
Ovary	PRS7	rs2066717	9	107591478	A	G	1.50E-03	PRS-CSx
Ovary	PRS7	rs2069235	22	39747780	G	A	6.68E-04	PRS-CSx

Ovary	PRS7	rs2069650	5	75918113	A	G	-7.02E-04	PRS-CSx
Ovary	PRS7	rs2071918	1	7150577	T	C	1.12E-03	PRS-CSx
Ovary	PRS7	rs2072020	6	112460648	A	G	-8.76E-05	PRS-CSx
Ovary	PRS7	rs2075714	6	49582617	T	C	-2.81E-04	PRS-CSx
Ovary	PRS7	rs2075993	1	23836364	A	G	-9.15E-04	PRS-CSx
Ovary	PRS7	rs2081808	12	105093533	G	A	-2.79E-03	PRS-CSx
Ovary	PRS7	rs2083819	17	10785838	T	C	-2.15E-04	PRS-CSx
Ovary	PRS7	rs2084870	2	208906654	C	T	-3.97E-04	PRS-CSx
Ovary	PRS7	rs2097769	4	7722458	A	G	2.48E-03	PRS-CSx
Ovary	PRS7	rs2100347	8	64499604	C	T	-1.57E-04	PRS-CSx
Ovary	PRS7	rs2102082	3	96979729	G	T	1.32E-03	PRS-CSx
Ovary	PRS7	rs2103598	6	19901452	G	A	2.25E-04	PRS-CSx
Ovary	PRS7	rs2110726	2	102794282	A	G	6.12E-04	PRS-CSx
Ovary	PRS7	rs2114507	18	64579205	G	A	-6.72E-04	PRS-CSx
Ovary	PRS7	rs2114719	15	70933338	A	G	-6.80E-04	PRS-CSx
Ovary	PRS7	rs2114721	15	70936549	C	A	-4.43E-04	PRS-CSx
Ovary	PRS7	rs212051	3	60034455	C	T	8.51E-04	PRS-CSx
Ovary	PRS7	rs2120560	8	53981711	C	A	9.23E-05	PRS-CSx
Ovary	PRS7	rs2120770	1	212509005	C	A	2.12E-04	PRS-CSx
Ovary	PRS7	rs2130937	4	175770916	A	G	-4.26E-04	PRS-CSx
Ovary	PRS7	rs2130938	4	175786729	T	C	-6.22E-04	PRS-CSx
Ovary	PRS7	rs2132349	8	101180248	A	G	-1.03E-03	PRS-CSx
Ovary	PRS7	rs2132545	5	167611655	T	C	-2.32E-05	PRS-CSx
Ovary	PRS7	rs2133738	5	1721003	A	G	1.11E-03	PRS-CSx
Ovary	PRS7	rs2134294	6	55134247	C	T	5.70E-04	PRS-CSx
Ovary	PRS7	rs2138573	5	160436777	A	G	3.12E-05	PRS-CSx
Ovary	PRS7	rs214437	6	9393307	T	G	-1.43E-03	PRS-CSx
Ovary	PRS7	rs214441	6	9394888	A	C	-8.35E-04	PRS-CSx
Ovary	PRS7	rs214443	6	9398094	C	T	-1.21E-03	PRS-CSx
Ovary	PRS7	rs2149380	9	90294574	C	A	-1.00E-04	PRS-CSx
Ovary	PRS7	rs2149891	8	132092099	T	C	-1.38E-04	PRS-CSx
Ovary	PRS7	rs2150721	9	2475688	A	G	2.52E-04	PRS-CSx
Ovary	PRS7	rs2154068	1	3720965	A	G	-1.29E-04	PRS-CSx
Ovary	PRS7	rs2156549	18	40413915	G	A	1.23E-03	PRS-CSx
Ovary	PRS7	rs2159400	3	47375890	C	T	8.94E-05	PRS-CSx
Ovary	PRS7	rs2160227	2	102783355	T	G	-3.26E-04	PRS-CSx
Ovary	PRS7	rs2160441	17	70004689	T	C	1.42E-03	PRS-CSx
Ovary	PRS7	rs2161799	15	71034290	T	C	-7.52E-04	PRS-CSx
Ovary	PRS7	rs2162553	15	70932050	T	G	-6.34E-04	PRS-CSx
Ovary	PRS7	rs2162555	15	70933572	T	C	-8.12E-04	PRS-CSx
Ovary	PRS7	rs2162556	15	70936438	T	C	-6.39E-04	PRS-CSx
Ovary	PRS7	rs217070	11	88067394	C	T	3.78E-04	PRS-CSx
Ovary	PRS7	rs217112	11	88030931	A	G	2.73E-04	PRS-CSx
Ovary	PRS7	rs2171451	5	176106430	G	A	-4.30E-04	PRS-CSx
Ovary	PRS7	rs2172121	8	48291480	C	A	7.26E-04	PRS-CSx
Ovary	PRS7	rs2172211	3	70024751	G	A	5.42E-04	PRS-CSx
Ovary	PRS7	rs2173671	5	73847044	A	G	-1.35E-03	PRS-CSx
Ovary	PRS7	rs2173911	3	190571142	G	A	4.24E-04	PRS-CSx
Ovary	PRS7	rs2174783	3	146429761	C	A	-3.75E-04	PRS-CSx
Ovary	PRS7	rs2180460	22	28964174	A	G	-1.91E-05	PRS-CSx
Ovary	PRS7	rs2182901	9	16406897	A	G	1.07E-03	PRS-CSx
Ovary	PRS7	rs2182973	14	70678222	C	T	-1.16E-03	PRS-CSx
Ovary	PRS7	rs2185608	1	120366283	T	C	-1.23E-03	PRS-CSx
Ovary	PRS7	rs2187160	11	86711494	T	C	1.50E-03	PRS-CSx
Ovary	PRS7	rs218843	6	121346497	G	A	-1.86E-04	PRS-CSx
Ovary	PRS7	rs2189984	7	82207785	G	A	2.18E-06	PRS-CSx

Ovary	PRS7	rs2190503	7	50742617	A	G	3.21E-04	PRS-CSx
Ovary	PRS7	rs2193354	12	107697765	C	T	1.54E-03	PRS-CSx
Ovary	PRS7	rs2197407	2	154789100	T	C	-3.73E-04	PRS-CSx
Ovary	PRS7	rs219765	21	37836711	G	A	-1.44E-03	PRS-CSx
Ovary	PRS7	rs2199197	11	112608137	G	A	-1.58E-05	PRS-CSx
Ovary	PRS7	rs2203817	3	116741261	C	T	8.56E-05	PRS-CSx
Ovary	PRS7	rs2206094	6	97575196	T	C	1.53E-06	PRS-CSx
Ovary	PRS7	rs2206465	20	41986957	G	A	-1.42E-03	PRS-CSx
Ovary	PRS7	rs2207541	20	60180124	A	C	-1.29E-04	PRS-CSx
Ovary	PRS7	rs2207669	10	83283805	T	C	-1.06E-05	PRS-CSx
Ovary	PRS7	rs2208370	1	171955331	A	G	2.31E-04	PRS-CSx
Ovary	PRS7	rs2210111	20	60232675	G	A	-1.58E-04	PRS-CSx
Ovary	PRS7	rs2211647	13	99303411	T	C	-1.84E-04	PRS-CSx
Ovary	PRS7	rs2214681	7	147702692	A	G	2.11E-03	PRS-CSx
Ovary	PRS7	rs2218880	2	152099885	A	G	-6.37E-05	PRS-CSx
Ovary	PRS7	rs2219265	3	66942668	C	T	7.93E-05	PRS-CSx
Ovary	PRS7	rs2224195	6	84307726	T	C	1.64E-04	PRS-CSx
Ovary	PRS7	rs2224396	1	171955221	A	G	9.94E-05	PRS-CSx
Ovary	PRS7	rs2228522	11	67166310	A	G	1.56E-03	PRS-CSx
Ovary	PRS7	rs2229971	9	139407932	A	G	1.35E-03	PRS-CSx
Ovary	PRS7	rs2229974	9	139391636	G	A	1.07E-03	PRS-CSx
Ovary	PRS7	rs2233955	6	31081251	A	G	-1.04E-04	PRS-CSx
Ovary	PRS7	rs2235152	22	45590861	A	G	-2.12E-05	PRS-CSx
Ovary	PRS7	rs2235529	1	22450487	T	C	2.00E-03	PRS-CSx
Ovary	PRS7	rs2235629	16	1546861	A	G	-1.79E-03	PRS-CSx
Ovary	PRS7	rs2237481	7	50748338	T	C	1.72E-03	PRS-CSx
Ovary	PRS7	rs2237488	7	50755995	G	A	7.56E-04	PRS-CSx
Ovary	PRS7	rs2239279	14	72938057	C	T	-3.82E-04	PRS-CSx
Ovary	PRS7	rs2239624	3	186025431	C	T	-6.29E-04	PRS-CSx
Ovary	PRS7	rs224017	9	20316445	C	T	3.32E-05	PRS-CSx
Ovary	PRS7	rs2243597	20	60166005	C	T	7.38E-05	PRS-CSx
Ovary	PRS7	rs2248525	13	78905243	G	T	7.71E-04	PRS-CSx
Ovary	PRS7	rs2249080	5	168986471	C	T	-1.27E-04	PRS-CSx
Ovary	PRS7	rs2249115	21	37849239	C	A	-2.40E-03	PRS-CSx
Ovary	PRS7	rs2249274	20	60141370	C	A	-1.34E-04	PRS-CSx
Ovary	PRS7	rs2252164	20	60142455	G	A	-9.42E-05	PRS-CSx
Ovary	PRS7	rs2252412	20	60144352	A	G	-1.37E-04	PRS-CSx
Ovary	PRS7	rs2252809	20	60147144	T	C	4.18E-05	PRS-CSx
Ovary	PRS7	rs2252915	20	60148268	C	T	-8.92E-06	PRS-CSx
Ovary	PRS7	rs2253214	20	60150821	G	T	-1.89E-04	PRS-CSx
Ovary	PRS7	rs2253803	20	60167718	A	C	-2.33E-04	PRS-CSx
Ovary	PRS7	rs2255074	12	57030026	C	T	3.86E-04	PRS-CSx
Ovary	PRS7	rs2260697	1	62638306	A	C	-2.01E-03	PRS-CSx
Ovary	PRS7	rs2266023	1	120408549	T	G	-1.01E-03	PRS-CSx
Ovary	PRS7	rs2268821	3	186018001	T	G	-7.23E-04	PRS-CSx
Ovary	PRS7	rs226898	16	19620862	T	C	1.97E-03	PRS-CSx
Ovary	PRS7	rs2269916	17	29233763	G	A	7.70E-04	PRS-CSx
Ovary	PRS7	rs2271811	2	152117780	C	T	-2.04E-05	PRS-CSx
Ovary	PRS7	rs2272669	8	76476457	A	G	-5.78E-04	PRS-CSx
Ovary	PRS7	rs2274442	14	69787749	C	T	1.61E-04	PRS-CSx
Ovary	PRS7	rs2275822	1	3761479	T	C	-8.90E-05	PRS-CSx
Ovary	PRS7	rs2276853	3	47282303	G	A	-5.59E-05	PRS-CSx
Ovary	PRS7	rs2276854	3	47276968	C	T	1.32E-04	PRS-CSx
Ovary	PRS7	rs2278089	2	152146672	G	T	4.16E-05	PRS-CSx
Ovary	PRS7	rs2280605	10	95278829	A	G	-3.25E-04	PRS-CSx
Ovary	PRS7	rs2280650	7	3236076	G	A	-2.52E-04	PRS-CSx

Ovary	PRS7	rs2281085	22	36885652	C	T	-1.45E-03	PRS-CSx
Ovary	PRS7	rs2282720	1	23839769	A	G	-4.24E-04	PRS-CSx
Ovary	PRS7	rs2282995	7	92411623	G	A	6.93E-04	PRS-CSx
Ovary	PRS7	rs2283379	14	72928829	C	A	-4.66E-04	PRS-CSx
Ovary	PRS7	rs2285745	19	36590329	T	C	7.47E-04	PRS-CSx
Ovary	PRS7	rs2285975	5	11117583	T	C	-1.27E-03	PRS-CSx
Ovary	PRS7	rs2286248	7	14216609	A	G	3.34E-04	PRS-CSx
Ovary	PRS7	rs228637	1	7917632	G	A	1.30E-03	PRS-CSx
Ovary	PRS7	rs2287047	2	102774054	A	G	-2.74E-04	PRS-CSx
Ovary	PRS7	rs2287049	2	102770738	G	A	-3.16E-04	PRS-CSx
Ovary	PRS7	rs2289909	13	25283596	T	C	6.07E-03	PRS-CSx
Ovary	PRS7	rs2290893	12	57078620	A	G	5.17E-04	PRS-CSx
Ovary	PRS7	rs2291357	3	155009135	A	G	6.37E-04	PRS-CSx
Ovary	PRS7	rs2291578	8	24256579	T	G	-8.64E-05	PRS-CSx
Ovary	PRS7	rs2294996	20	61488785	C	T	8.26E-04	PRS-CSx
Ovary	PRS7	rs2295709	6	119231968	C	T	3.36E-05	PRS-CSx
Ovary	PRS7	rs2295932	9	101634716	C	T	-1.69E-03	PRS-CSx
Ovary	PRS7	rs2296076	9	116079271	C	A	-3.23E-04	PRS-CSx
Ovary	PRS7	rs2296384	1	201058692	C	T	-1.99E-04	PRS-CSx
Ovary	PRS7	rs2297374	6	160575985	T	C	-2.18E-03	PRS-CSx
Ovary	PRS7	rs2298321	8	37456304	A	G	1.97E-04	PRS-CSx
Ovary	PRS7	rs2298489	11	113235419	A	G	-6.61E-06	PRS-CSx
Ovary	PRS7	rs2301059	1	64086901	A	G	3.81E-03	PRS-CSx
Ovary	PRS7	rs2305641	12	69646914	A	G	9.42E-04	PRS-CSx
Ovary	PRS7	rs2306515	12	129715859	G	T	-1.32E-03	PRS-CSx
Ovary	PRS7	rs2306752	17	80426635	C	T	-9.13E-05	PRS-CSx
Ovary	PRS7	rs2310186	2	102743180	G	T	-3.76E-04	PRS-CSx
Ovary	PRS7	rs2315840	3	99829320	A	C	1.91E-03	PRS-CSx
Ovary	PRS7	rs231622	5	54528590	A	G	5.07E-04	PRS-CSx
Ovary	PRS7	rs2316426	2	236358786	T	C	-1.61E-04	PRS-CSx
Ovary	PRS7	rs2317534	8	136944696	G	A	2.14E-04	PRS-CSx
Ovary	PRS7	rs2324909	13	67169478	C	T	9.82E-05	PRS-CSx
Ovary	PRS7	rs2326017	17	46720565	T	C	8.00E-05	PRS-CSx
Ovary	PRS7	rs2326024	16	83793154	A	G	2.39E-04	PRS-CSx
Ovary	PRS7	rs2327587	6	135580953	C	T	-3.70E-05	PRS-CSx
Ovary	PRS7	rs2328962	13	76175865	G	A	-4.17E-04	PRS-CSx
Ovary	PRS7	rs2329072	13	78960814	C	T	7.07E-04	PRS-CSx
Ovary	PRS7	rs2329424	7	50806534	C	A	9.14E-04	PRS-CSx
Ovary	PRS7	rs2331886	1	180733123	T	G	1.37E-04	PRS-CSx
Ovary	PRS7	rs2333563	17	57839568	A	G	-3.03E-04	PRS-CSx
Ovary	PRS7	rs234910	5	78257251	G	A	-1.12E-03	PRS-CSx
Ovary	PRS7	rs2355181	2	52222395	T	C	1.77E-03	PRS-CSx
Ovary	PRS7	rs2363285	7	1091644	T	C	-4.45E-04	PRS-CSx
Ovary	PRS7	rs2364281	3	67050203	T	G	1.22E-04	PRS-CSx
Ovary	PRS7	rs2365680	14	78735426	T	C	-6.05E-04	PRS-CSx
Ovary	PRS7	rs237012	6	149729198	T	C	3.59E-04	PRS-CSx
Ovary	PRS7	rs237018	6	149743930	T	C	3.89E-04	PRS-CSx
Ovary	PRS7	rs237025	6	149721690	G	A	4.33E-04	PRS-CSx
Ovary	PRS7	rs237028	6	149718650	C	T	4.87E-04	PRS-CSx
Ovary	PRS7	rs237035	6	149710848	C	T	5.84E-04	PRS-CSx
Ovary	PRS7	rs2371271	2	212316688	C	T	-1.12E-03	PRS-CSx
Ovary	PRS7	rs2371424	3	28788920	G	T	-6.35E-04	PRS-CSx
Ovary	PRS7	rs2374690	12	107704114	A	G	5.54E-04	PRS-CSx
Ovary	PRS7	rs2376432	8	54003760	G	A	1.31E-04	PRS-CSx
Ovary	PRS7	rs238196	20	47898217	G	A	3.76E-04	PRS-CSx
Ovary	PRS7	rs2383208	9	22132076	G	A	2.02E-03	PRS-CSx

Ovary	PRS7	rs2383933	8	76515057	A	G	-2.88E-04	PRS-CSx
Ovary	PRS7	rs2385867	3	47059323	C	T	-7.41E-05	PRS-CSx
Ovary	PRS7	rs2390866	1	90546731	A	G	-3.25E-05	PRS-CSx
Ovary	PRS7	rs2391166	13	105968712	T	C	-5.81E-06	PRS-CSx
Ovary	PRS7	rs2396332	2	227246379	C	T	1.51E-03	PRS-CSx
Ovary	PRS7	rs2402036	7	115780581	C	T	-1.55E-04	PRS-CSx
Ovary	PRS7	rs2402974	7	129329261	A	G	9.39E-04	PRS-CSx
Ovary	PRS7	rs2403583	11	20378100	C	A	5.16E-04	PRS-CSx
Ovary	PRS7	rs2410229	8	14938404	G	T	-3.08E-04	PRS-CSx
Ovary	PRS7	rs2416742	9	122777758	C	A	8.95E-05	PRS-CSx
Ovary	PRS7	rs2427170	20	60176927	T	C	6.57E-05	PRS-CSx
Ovary	PRS7	rs2427176	20	60178344	C	T	1.43E-04	PRS-CSx
Ovary	PRS7	rs2430081	1	187783367	G	A	1.47E-03	PRS-CSx
Ovary	PRS7	rs2432625	16	12477223	C	T	2.30E-04	PRS-CSx
Ovary	PRS7	rs2432626	16	12476660	A	G	1.10E-04	PRS-CSx
Ovary	PRS7	rs2433	17	29226228	C	T	2.22E-04	PRS-CSx
Ovary	PRS7	rs2439667	13	70591572	C	A	1.99E-05	PRS-CSx
Ovary	PRS7	rs2446469	8	113883082	T	G	3.36E-04	PRS-CSx
Ovary	PRS7	rs2448730	18	65378004	A	G	-2.02E-03	PRS-CSx
Ovary	PRS7	rs2448731	18	65376601	T	C	-2.01E-03	PRS-CSx
Ovary	PRS7	rs2452321	3	99638836	C	T	-1.26E-04	PRS-CSx
Ovary	PRS7	rs2453913	17	57860150	G	T	-4.44E-04	PRS-CSx
Ovary	PRS7	rs2457050	1	48041606	G	T	-1.59E-04	PRS-CSx
Ovary	PRS7	rs245889	7	29190626	C	T	7.27E-05	PRS-CSx
Ovary	PRS7	rs2467712	8	25524964	A	G	3.72E-05	PRS-CSx
Ovary	PRS7	rs2469406	8	3506404	T	G	2.02E-04	PRS-CSx
Ovary	PRS7	rs2469407	8	3506362	C	T	2.73E-04	PRS-CSx
Ovary	PRS7	rs2471254	7	45878544	T	C	-1.74E-03	PRS-CSx
Ovary	PRS7	rs2472280	13	70589228	C	A	-1.64E-05	PRS-CSx
Ovary	PRS7	rs2475230	10	116259930	A	G	2.55E-03	PRS-CSx
Ovary	PRS7	rs247544	5	110895975	A	G	1.71E-05	PRS-CSx
Ovary	PRS7	rs249368	19	48817195	C	T	8.81E-04	PRS-CSx
Ovary	PRS7	rs2504806	6	39824567	G	T	5.18E-04	PRS-CSx
Ovary	PRS7	rs254560	5	134443606	A	G	4.71E-04	PRS-CSx
Ovary	PRS7	rs254562	5	134441457	G	A	4.15E-04	PRS-CSx
Ovary	PRS7	rs254563	5	134440426	A	G	2.79E-04	PRS-CSx
Ovary	PRS7	rs254570	5	134436352	A	G	4.65E-05	PRS-CSx
Ovary	PRS7	rs2545990	19	35405751	C	T	5.58E-04	PRS-CSx
Ovary	PRS7	rs2545995	19	35412315	T	C	6.70E-04	PRS-CSx
Ovary	PRS7	rs2546651	5	135605350	T	C	9.02E-05	PRS-CSx
Ovary	PRS7	rs2546700	5	168943227	C	T	3.89E-04	PRS-CSx
Ovary	PRS7	rs255031	5	25740033	A	G	-7.48E-05	PRS-CSx
Ovary	PRS7	rs255044	5	25720302	A	G	-7.88E-05	PRS-CSx
Ovary	PRS7	rs2553915	8	54038721	A	G	7.90E-05	PRS-CSx
Ovary	PRS7	rs2564	22	36885159	C	A	-1.16E-03	PRS-CSx
Ovary	PRS7	rs25745	5	168998260	G	A	6.59E-05	PRS-CSx
Ovary	PRS7	rs25748	5	169001157	C	T	1.38E-04	PRS-CSx
Ovary	PRS7	rs2586416	1	172270226	A	G	-4.17E-04	PRS-CSx
Ovary	PRS7	rs2598338	12	16254895	T	G	-2.50E-04	PRS-CSx
Ovary	PRS7	rs2602029	15	99706633	C	T	6.60E-04	PRS-CSx
Ovary	PRS7	rs2604256	12	16243058	C	T	1.59E-04	PRS-CSx
Ovary	PRS7	rs2604261	12	16261743	T	C	-2.34E-04	PRS-CSx
Ovary	PRS7	rs2608017	12	52675917	G	A	-9.91E-04	PRS-CSx
Ovary	PRS7	rs2623695	8	3588767	T	C	2.68E-03	PRS-CSx
Ovary	PRS7	rs2623853	8	68671517	G	A	-1.01E-03	PRS-CSx
Ovary	PRS7	rs2629471	6	80175399	A	G	1.03E-03	PRS-CSx

Ovary	PRS7	rs263571	9	17030663	A	G	4.31E-04	PRS-CSx
Ovary	PRS7	rs263648	9	17015867	C	A	-1.93E-04	PRS-CSx
Ovary	PRS7	rs263811	13	55563725	T	C	-4.72E-04	PRS-CSx
Ovary	PRS7	rs2645466	17	57853214	A	C	-2.71E-04	PRS-CSx
Ovary	PRS7	rs264863	5	169060277	A	C	1.92E-04	PRS-CSx
Ovary	PRS7	rs2651085	19	35394338	G	A	1.15E-03	PRS-CSx
Ovary	PRS7	rs2651099	19	35425001	A	G	2.21E-04	PRS-CSx
Ovary	PRS7	rs2651766	2	48044772	T	C	-1.58E-04	PRS-CSx
Ovary	PRS7	rs2656240	8	55216906	C	T	-1.26E-04	PRS-CSx
Ovary	PRS7	rs2672124	2	131538181	T	C	-2.05E-03	PRS-CSx
Ovary	PRS7	rs2676533	17	40690261	G	A	5.19E-05	PRS-CSx
Ovary	PRS7	rs2684878	1	162822771	A	C	2.03E-04	PRS-CSx
Ovary	PRS7	rs268776	3	57934374	C	T	-2.75E-04	PRS-CSx
Ovary	PRS7	rs2696849	16	86375208	G	A	-3.47E-05	PRS-CSx
Ovary	PRS7	rs2707582	7	147734565	A	G	2.44E-04	PRS-CSx
Ovary	PRS7	rs2712162	2	217661788	C	T	1.37E-04	PRS-CSx
Ovary	PRS7	rs2715128	7	50742118	A	G	6.38E-04	PRS-CSx
Ovary	PRS7	rs2715131	7	50749079	C	T	3.81E-04	PRS-CSx
Ovary	PRS7	rs2715133	7	50749425	A	G	4.04E-04	PRS-CSx
Ovary	PRS7	rs2717637	8	54039378	G	A	6.97E-05	PRS-CSx
Ovary	PRS7	rs2726596	8	59710386	T	C	-4.26E-04	PRS-CSx
Ovary	PRS7	rs2726597	8	59712891	T	G	-1.92E-04	PRS-CSx
Ovary	PRS7	rs2726598	8	59713199	A	G	-1.64E-04	PRS-CSx
Ovary	PRS7	rs2728632	12	20146527	C	A	-8.27E-04	PRS-CSx
Ovary	PRS7	rs2731423	12	78357509	G	T	-6.03E-04	PRS-CSx
Ovary	PRS7	rs2739169	8	134095916	G	A	-1.03E-04	PRS-CSx
Ovary	PRS7	rs2739180	8	134108718	A	G	-5.59E-04	PRS-CSx
Ovary	PRS7	rs274168	19	56686801	A	G	-1.29E-03	PRS-CSx
Ovary	PRS7	rs2744708	1	22506851	G	A	-9.74E-04	PRS-CSx
Ovary	PRS7	rs2744721	1	22487036	T	C	-1.14E-03	PRS-CSx
Ovary	PRS7	rs2748787	4	3714659	G	T	-1.52E-04	PRS-CSx
Ovary	PRS7	rs276394	13	40594451	C	T	4.18E-05	PRS-CSx
Ovary	PRS7	rs276420	13	40572070	G	T	9.68E-05	PRS-CSx
Ovary	PRS7	rs276949	16	86244517	G	A	2.59E-03	PRS-CSx
Ovary	PRS7	rs2794496	13	90660090	C	T	-6.86E-04	PRS-CSx
Ovary	PRS7	rs279715	20	45103199	T	C	3.81E-04	PRS-CSx
Ovary	PRS7	rs279727	20	45082339	T	C	6.13E-04	PRS-CSx
Ovary	PRS7	rs2805676	13	90656936	A	C	-9.57E-04	PRS-CSx
Ovary	PRS7	rs2813746	1	90539764	C	A	-1.32E-04	PRS-CSx
Ovary	PRS7	rs2816518	8	5778862	A	C	-2.76E-04	PRS-CSx
Ovary	PRS7	rs2817685	10	98845536	G	A	1.34E-04	PRS-CSx
Ovary	PRS7	rs2817694	10	98866522	A	G	3.09E-05	PRS-CSx
Ovary	PRS7	rs282322	12	95789810	T	G	-8.10E-04	PRS-CSx
Ovary	PRS7	rs282324	12	95790082	C	T	-1.56E-03	PRS-CSx
Ovary	PRS7	rs2835365	21	37865339	T	G	-3.45E-03	PRS-CSx
Ovary	PRS7	rs2845769	21	37856952	C	T	-1.10E-03	PRS-CSx
Ovary	PRS7	rs2850463	18	33135111	T	C	6.18E-05	PRS-CSx
Ovary	PRS7	rs2859486	19	35424504	A	C	3.55E-04	PRS-CSx
Ovary	PRS7	rs2861828	10	98854410	G	A	-1.85E-04	PRS-CSx
Ovary	PRS7	rs2862783	19	35370305	G	A	9.25E-04	PRS-CSx
Ovary	PRS7	rs2863202	1	67584335	T	C	-1.61E-04	PRS-CSx
Ovary	PRS7	rs2867603	7	71598202	G	A	1.85E-04	PRS-CSx
Ovary	PRS7	rs2874792	4	166752233	A	C	-1.50E-03	PRS-CSx
Ovary	PRS7	rs2877847	14	28095823	C	T	3.49E-04	PRS-CSx
Ovary	PRS7	rs2878076	1	246395662	T	C	-7.41E-05	PRS-CSx
Ovary	PRS7	rs2878079	1	246390090	A	G	-2.34E-04	PRS-CSx

Ovary	PRS7	rs288194	5	107318454	C	T	4.06E-04	PRS-CSx
Ovary	PRS7	rs2887115	7	81744432	G	A	9.95E-04	PRS-CSx
Ovary	PRS7	rs288746	7	155606672	G	A	4.44E-04	PRS-CSx
Ovary	PRS7	rs2887961	3	40214049	T	C	-1.02E-03	PRS-CSx
Ovary	PRS7	rs28915400	19	572004	T	G	1.60E-04	PRS-CSx
Ovary	PRS7	rs2893615	19	2458247	T	G	3.83E-04	PRS-CSx
Ovary	PRS7	rs2896769	18	72436704	A	G	1.17E-04	PRS-CSx
Ovary	PRS7	rs289903	2	152084705	G	A	-1.82E-04	PRS-CSx
Ovary	PRS7	rs289904	2	152085643	C	T	-7.72E-06	PRS-CSx
Ovary	PRS7	rs2900167	9	122773855	C	T	-2.64E-04	PRS-CSx
Ovary	PRS7	rs2913530	5	173863445	A	G	1.39E-03	PRS-CSx
Ovary	PRS7	rs2919650	5	78248660	A	C	-4.45E-04	PRS-CSx
Ovary	PRS7	rs2922388	8	136936585	C	A	6.51E-04	PRS-CSx
Ovary	PRS7	rs2926743	12	57114100	A	G	6.68E-04	PRS-CSx
Ovary	PRS7	rs2927234	8	76754904	G	A	-5.49E-04	PRS-CSx
Ovary	PRS7	rs2931095	6	120708891	C	T	-5.69E-04	PRS-CSx
Ovary	PRS7	rs2941422	8	76519834	T	C	-1.44E-04	PRS-CSx
Ovary	PRS7	rs2941475	8	76474404	C	T	-4.82E-04	PRS-CSx
Ovary	PRS7	rs2941479	8	76476959	A	C	-2.96E-04	PRS-CSx
Ovary	PRS7	rs2941481	8	76477368	A	G	-7.31E-04	PRS-CSx
Ovary	PRS7	rs2941483	8	76478616	G	A	-3.31E-04	PRS-CSx
Ovary	PRS7	rs2941484	8	76478768	T	C	-6.41E-04	PRS-CSx
Ovary	PRS7	rs2941489	8	76482263	T	C	-4.93E-04	PRS-CSx
Ovary	PRS7	rs2941492	8	76487628	G	A	-2.89E-04	PRS-CSx
Ovary	PRS7	rs2943561	8	76496638	A	G	-3.01E-04	PRS-CSx
Ovary	PRS7	rs2945767	8	134627403	G	A	1.81E-03	PRS-CSx
Ovary	PRS7	rs2949307	7	46021684	C	T	8.01E-04	PRS-CSx
Ovary	PRS7	rs2950387	12	57021006	C	T	5.06E-04	PRS-CSx
Ovary	PRS7	rs2950390	12	57055291	T	C	2.35E-04	PRS-CSx
Ovary	PRS7	rs295428	3	47359262	G	T	-9.73E-05	PRS-CSx
Ovary	PRS7	rs295442	3	47335881	A	C	9.52E-05	PRS-CSx
Ovary	PRS7	rs295449	3	47375955	G	A	5.28E-05	PRS-CSx
Ovary	PRS7	rs295458	3	47385585	C	A	-5.84E-05	PRS-CSx
Ovary	PRS7	rs295461	3	47368010	T	C	1.40E-04	PRS-CSx
Ovary	PRS7	rs2958139	12	57004668	A	G	5.62E-04	PRS-CSx
Ovary	PRS7	rs2958149	12	57109792	A	G	3.35E-04	PRS-CSx
Ovary	PRS7	rs2958153	12	57081517	A	G	4.95E-04	PRS-CSx
Ovary	PRS7	rs2958154	12	57065713	C	T	3.03E-04	PRS-CSx
Ovary	PRS7	rs2958155	12	57064545	T	G	4.13E-04	PRS-CSx
Ovary	PRS7	rs2961594	10	4847924	T	C	-1.39E-03	PRS-CSx
Ovary	PRS7	rs2967727	19	8932388	A	G	-1.42E-03	PRS-CSx
Ovary	PRS7	rs297304	4	27716653	T	C	1.23E-03	PRS-CSx
Ovary	PRS7	rs297306	4	27715503	G	A	1.91E-03	PRS-CSx
Ovary	PRS7	rs2977332	8	76754934	T	C	-3.45E-04	PRS-CSx
Ovary	PRS7	rs2977944	8	76474719	A	G	-8.09E-04	PRS-CSx
Ovary	PRS7	rs2977952	8	76487541	C	T	-1.82E-04	PRS-CSx
Ovary	PRS7	rs2979025	8	134115737	T	C	-5.21E-04	PRS-CSx
Ovary	PRS7	rs2982285	1	22506253	C	T	-1.14E-03	PRS-CSx
Ovary	PRS7	rs2982608	11	34190762	C	T	1.96E-03	PRS-CSx
Ovary	PRS7	rs2983364	10	52345974	A	G	1.92E-04	PRS-CSx
Ovary	PRS7	rs2988066	9	92144998	A	G	-2.25E-04	PRS-CSx
Ovary	PRS7	rs2994808	1	120350309	A	C	-6.27E-04	PRS-CSx
Ovary	PRS7	rs2994809	1	120351977	C	T	-1.05E-03	PRS-CSx
Ovary	PRS7	rs2994811	1	120355513	T	C	-8.58E-04	PRS-CSx
Ovary	PRS7	rs2994815	1	120365519	T	C	-8.98E-04	PRS-CSx
Ovary	PRS7	rs3006186	6	169798329	C	A	-3.25E-03	PRS-CSx

Ovary	PRS7	rs3009186	1	120380990	T	C	-1.07E-03	PRS-CSx
Ovary	PRS7	rs3009197	1	120359998	G	A	-5.94E-04	PRS-CSx
Ovary	PRS7	rs3026106	17	5282100	T	C	1.68E-04	PRS-CSx
Ovary	PRS7	rs303890	6	25035920	A	G	8.82E-04	PRS-CSx
Ovary	PRS7	rs305641	5	83669315	G	T	-2.11E-04	PRS-CSx
Ovary	PRS7	rs305653	5	83646811	C	T	-1.33E-04	PRS-CSx
Ovary	PRS7	rs3087548	9	116029174	T	C	-2.45E-04	PRS-CSx
Ovary	PRS7	rs3087677	16	69220623	T	G	9.54E-04	PRS-CSx
Ovary	PRS7	rs309091	13	62932015	C	A	-1.09E-04	PRS-CSx
Ovary	PRS7	rs309287	2	7192061	A	G	5.03E-04	PRS-CSx
Ovary	PRS7	rs309309	2	7159485	T	C	7.48E-04	PRS-CSx
Ovary	PRS7	rs309311	2	7156947	A	G	3.65E-04	PRS-CSx
Ovary	PRS7	rs3095646	16	48894046	G	A	-2.26E-03	PRS-CSx
Ovary	PRS7	rs3096425	16	26260788	T	C	-4.49E-04	PRS-CSx
Ovary	PRS7	rs309719	4	177401671	T	G	7.45E-05	PRS-CSx
Ovary	PRS7	rs309722	4	177399566	T	C	4.20E-05	PRS-CSx
Ovary	PRS7	rs3099065	5	179649862	A	G	2.99E-03	PRS-CSx
Ovary	PRS7	rs3105807	5	54392373	C	T	-3.19E-04	PRS-CSx
Ovary	PRS7	rs310583	7	150901609	A	G	7.40E-05	PRS-CSx
Ovary	PRS7	rs310584	7	150896223	A	G	-6.60E-05	PRS-CSx
Ovary	PRS7	rs3108171	19	37117302	A	G	5.62E-04	PRS-CSx
Ovary	PRS7	rs3108543	19	37143992	G	A	4.39E-04	PRS-CSx
Ovary	PRS7	rs310963	13	62972440	C	T	-2.54E-05	PRS-CSx
Ovary	PRS7	rs3110454	17	28651363	T	C	5.20E-04	PRS-CSx
Ovary	PRS7	rs3111519	5	179654415	C	T	1.18E-03	PRS-CSx
Ovary	PRS7	rs3111601	16	86400081	C	T	1.54E-03	PRS-CSx
Ovary	PRS7	rs311497	20	62221249	G	A	-1.49E-03	PRS-CSx
Ovary	PRS7	rs3115900	8	94943615	A	G	2.13E-05	PRS-CSx
Ovary	PRS7	rs3124999	9	139395473	C	T	1.69E-03	PRS-CSx
Ovary	PRS7	rs3128479	9	90297680	A	C	-2.05E-05	PRS-CSx
Ovary	PRS7	rs3129890	6	32414273	C	T	5.23E-04	PRS-CSx
Ovary	PRS7	rs31304	5	52942083	A	C	-8.16E-04	PRS-CSx
Ovary	PRS7	rs3131715	1	58178278	C	T	-6.01E-06	PRS-CSx
Ovary	PRS7	rs3131780	1	58170218	T	G	-4.85E-05	PRS-CSx
Ovary	PRS7	rs313784	19	3293593	G	A	-2.02E-04	PRS-CSx
Ovary	PRS7	rs315419	6	153523383	G	A	7.03E-05	PRS-CSx
Ovary	PRS7	rs3181039	3	46421838	A	C	-2.21E-04	PRS-CSx
Ovary	PRS7	rs319027	11	89249010	T	C	1.21E-03	PRS-CSx
Ovary	PRS7	rs321126	11	112568790	G	A	3.08E-04	PRS-CSx
Ovary	PRS7	rs3214051	12	57119236	G	A	1.38E-04	PRS-CSx
Ovary	PRS7	rs3218069	19	30313576	T	C	2.88E-04	PRS-CSx
Ovary	PRS7	rs328864	9	7711440	G	A	9.88E-04	PRS-CSx
Ovary	PRS7	rs328875	9	107786913	T	C	1.08E-04	PRS-CSx
Ovary	PRS7	rs3340	5	153831867	C	T	-1.30E-04	PRS-CSx
Ovary	PRS7	rs334879	5	54520147	A	G	2.97E-04	PRS-CSx
Ovary	PRS7	rs335739	19	30627316	T	G	5.37E-04	PRS-CSx
Ovary	PRS7	rs335740	19	30627658	A	G	9.93E-04	PRS-CSx
Ovary	PRS7	rs335744	19	30629569	G	A	1.01E-03	PRS-CSx
Ovary	PRS7	rs335745	19	30629595	A	G	1.24E-03	PRS-CSx
Ovary	PRS7	rs335746	19	30630847	A	C	7.45E-04	PRS-CSx
Ovary	PRS7	rs336087	5	54450002	C	T	1.66E-04	PRS-CSx
Ovary	PRS7	rs336115	5	54468921	C	T	1.97E-04	PRS-CSx
Ovary	PRS7	rs336119	5	54472174	A	C	7.01E-04	PRS-CSx
Ovary	PRS7	rs336123	5	54474427	A	G	5.07E-04	PRS-CSx
Ovary	PRS7	rs337887	5	78229036	C	T	-7.27E-04	PRS-CSx
Ovary	PRS7	rs337888	5	78228199	A	G	-5.71E-04	PRS-CSx

Ovary	PRS7	rs34132424	2	152105091	A	C	-6.41E-05	PRS-CSx
Ovary	PRS7	rs34722	19	30366802	C	A	-9.36E-05	PRS-CSx
Ovary	PRS7	rs34724	19	30369010	G	T	2.43E-04	PRS-CSx
Ovary	PRS7	rs34762299	17	40605099	G	A	-1.71E-03	PRS-CSx
Ovary	PRS7	rs35056852	15	58301556	C	T	8.48E-05	PRS-CSx
Ovary	PRS7	rs35124509	3	89521693	C	T	-6.51E-04	PRS-CSx
Ovary	PRS7	rs352766	8	15644559	G	A	5.76E-04	PRS-CSx
Ovary	PRS7	rs353243	5	148827194	C	A	-3.25E-03	PRS-CSx
Ovary	PRS7	rs35329108	5	1216900	A	G	1.13E-03	PRS-CSx
Ovary	PRS7	rs35417544	2	11680403	C	T	-8.84E-04	PRS-CSx
Ovary	PRS7	rs355299	18	33121245	C	T	2.70E-05	PRS-CSx
Ovary	PRS7	rs35539760	9	91963480	A	G	-4.48E-04	PRS-CSx
Ovary	PRS7	rs36023314	17	40649360	T	G	-4.21E-04	PRS-CSx
Ovary	PRS7	rs36054377	10	120353343	C	T	2.46E-03	PRS-CSx
Ovary	PRS7	rs36056619	17	29172284	C	T	6.20E-04	PRS-CSx
Ovary	PRS7	rs364891	2	7137067	A	G	1.16E-03	PRS-CSx
Ovary	PRS7	rs367107	2	7171605	C	T	3.08E-04	PRS-CSx
Ovary	PRS7	rs3731288	7	92423933	C	A	8.20E-04	PRS-CSx
Ovary	PRS7	rs3731303	7	92403859	T	C	6.12E-04	PRS-CSx
Ovary	PRS7	rs3733510	4	20611629	A	G	3.77E-04	PRS-CSx
Ovary	PRS7	rs3734206	6	45922849	T	C	-5.08E-04	PRS-CSx
Ovary	PRS7	rs3735736	8	28194097	A	G	-3.09E-04	PRS-CSx
Ovary	PRS7	rs3740571	10	117705146	C	T	-1.25E-03	PRS-CSx
Ovary	PRS7	rs3741189	11	66995603	T	C	2.40E-03	PRS-CSx
Ovary	PRS7	rs3741255	11	61516910	A	G	2.62E-03	PRS-CSx
Ovary	PRS7	rs3741720	12	52709222	C	T	-1.29E-03	PRS-CSx
Ovary	PRS7	rs3741742	12	65273036	C	T	7.67E-04	PRS-CSx
Ovary	PRS7	rs3744772	17	46713532	G	A	-1.41E-04	PRS-CSx
Ovary	PRS7	rs3746301	19	54074747	A	G	3.87E-04	PRS-CSx
Ovary	PRS7	rs3747163	22	36885026	T	C	-1.24E-03	PRS-CSx
Ovary	PRS7	rs3748962	2	212251864	C	T	5.35E-04	PRS-CSx
Ovary	PRS7	rs3750889	8	132002334	C	T	-5.68E-05	PRS-CSx
Ovary	PRS7	rs3751006	11	5625847	A	G	1.81E-03	PRS-CSx
Ovary	PRS7	rs3751432	13	41044128	T	C	4.57E-03	PRS-CSx
Ovary	PRS7	rs3751434	13	41044340	T	C	1.94E-03	PRS-CSx
Ovary	PRS7	rs3753121	8	143926380	T	C	1.02E-04	PRS-CSx
Ovary	PRS7	rs3754210	1	150954261	T	G	-4.99E-04	PRS-CSx
Ovary	PRS7	rs3754935	2	202119272	C	A	-1.92E-03	PRS-CSx
Ovary	PRS7	rs3756054	4	90674451	T	C	-1.82E-04	PRS-CSx
Ovary	PRS7	rs3760387	17	40608536	T	G	9.81E-05	PRS-CSx
Ovary	PRS7	rs3760994	19	1435771	A	G	-1.48E-04	PRS-CSx
Ovary	PRS7	rs3764420	17	29164154	G	A	3.14E-04	PRS-CSx
Ovary	PRS7	rs3764421	17	29167653	C	A	4.49E-04	PRS-CSx
Ovary	PRS7	rs3765463	20	61485211	G	A	1.10E-03	PRS-CSx
Ovary	PRS7	rs3765474	6	161990483	C	T	-1.82E-03	PRS-CSx
Ovary	PRS7	rs3769541	2	33502505	T	C	1.05E-04	PRS-CSx
Ovary	PRS7	rs3769772	2	170368695	C	T	2.70E-04	PRS-CSx
Ovary	PRS7	rs3769872	2	165565548	T	C	4.04E-04	PRS-CSx
Ovary	PRS7	rs3770388	2	53094042	G	T	-8.98E-04	PRS-CSx
Ovary	PRS7	rs3771199	2	102806613	G	A	5.51E-04	PRS-CSx
Ovary	PRS7	rs3771200	2	102788774	A	G	7.29E-04	PRS-CSx
Ovary	PRS7	rs3771882	2	152126966	A	G	-1.66E-05	PRS-CSx
Ovary	PRS7	rs3776581	5	36661944	A	G	-1.08E-04	PRS-CSx
Ovary	PRS7	rs3777084	5	169031150	T	C	1.55E-05	PRS-CSx
Ovary	PRS7	rs3777591	6	45922402	A	G	-2.15E-04	PRS-CSx
Ovary	PRS7	rs3779341	7	77668115	C	T	-7.47E-06	PRS-CSx

Ovary	PRS7	rs3781226	10	48416258	T	C	-1.91E-03	PRS-CSx
Ovary	PRS7	rs3783914	14	93453676	C	T	1.30E-03	PRS-CSx
Ovary	PRS7	rs3787317	20	48041211	T	C	-1.12E-03	PRS-CSx
Ovary	PRS7	rs3789541	1	6141325	A	G	-4.48E-04	PRS-CSx
Ovary	PRS7	rs3797713	5	169028481	T	C	1.88E-04	PRS-CSx
Ovary	PRS7	rs3800052	6	1773297	T	C	-1.78E-03	PRS-CSx
Ovary	PRS7	rs3802072	7	92403053	A	G	7.42E-04	PRS-CSx
Ovary	PRS7	rs3802073	7	92402480	T	C	5.35E-04	PRS-CSx
Ovary	PRS7	rs3802487	9	125590647	A	G	8.40E-06	PRS-CSx
Ovary	PRS7	rs3803856	17	45942176	C	T	-6.82E-04	PRS-CSx
Ovary	PRS7	rs3807784	7	77669302	A	G	2.29E-04	PRS-CSx
Ovary	PRS7	rs3809485	15	48051456	G	A	-1.75E-04	PRS-CSx
Ovary	PRS7	rs380996	16	85728813	A	G	-3.59E-04	PRS-CSx
Ovary	PRS7	rs3810918	9	116036566	G	A	-2.57E-04	PRS-CSx
Ovary	PRS7	rs3810919	9	116037894	T	C	1.15E-04	PRS-CSx
Ovary	PRS7	rs3810920	9	116091077	G	A	-6.15E-05	PRS-CSx
Ovary	PRS7	rs3813002	16	31470540	T	C	-9.81E-04	PRS-CSx
Ovary	PRS7	rs3813382	7	22149185	A	G	-8.11E-04	PRS-CSx
Ovary	PRS7	rs3816780	17	29161358	T	C	4.42E-04	PRS-CSx
Ovary	PRS7	rs3816852	7	4862259	G	A	-4.99E-04	PRS-CSx
Ovary	PRS7	rs3817578	2	202136595	T	C	-1.63E-03	PRS-CSx
Ovary	PRS7	rs3819857	3	186015438	T	C	1.03E-03	PRS-CSx
Ovary	PRS7	rs3819860	3	186015283	A	G	-1.03E-03	PRS-CSx
Ovary	PRS7	rs3820028	1	23834486	G	A	-1.06E-03	PRS-CSx
Ovary	PRS7	rs3821364	3	70011421	C	T	5.23E-04	PRS-CSx
Ovary	PRS7	rs3825022	11	67013197	G	A	1.37E-03	PRS-CSx
Ovary	PRS7	rs3843360	3	60041687	T	G	1.58E-03	PRS-CSx
Ovary	PRS7	rs3844343	10	65434809	A	G	2.21E-03	PRS-CSx
Ovary	PRS7	rs3845973	3	60027906	G	A	6.93E-04	PRS-CSx
Ovary	PRS7	rs3846145	3	3307984	A	G	1.59E-04	PRS-CSx
Ovary	PRS7	rs3856557	2	152136961	A	G	-1.29E-04	PRS-CSx
Ovary	PRS7	rs3857057	4	90668019	A	G	-1.33E-06	PRS-CSx
Ovary	PRS7	rs3857059	4	90675238	A	G	-8.20E-05	PRS-CSx
Ovary	PRS7	rs3860052	12	76352699	G	T	9.00E-05	PRS-CSx
Ovary	PRS7	rs3862611	11	121831524	A	G	1.82E-03	PRS-CSx
Ovary	PRS7	rs3864095	3	186937037	A	G	-3.29E-04	PRS-CSx
Ovary	PRS7	rs3867335	2	44262001	A	G	-2.13E-03	PRS-CSx
Ovary	PRS7	rs388038	2	7139212	A	G	1.03E-03	PRS-CSx
Ovary	PRS7	rs3889884	12	80757213	T	C	2.96E-04	PRS-CSx
Ovary	PRS7	rs3889940	8	124833311	T	C	2.55E-03	PRS-CSx
Ovary	PRS7	rs3890133	4	5861211	T	C	7.93E-05	PRS-CSx
Ovary	PRS7	rs389496	22	18289204	A	G	-2.59E-03	PRS-CSx
Ovary	PRS7	rs3900188	5	120683069	G	A	-1.19E-03	PRS-CSx
Ovary	PRS7	rs390755	5	73839817	G	A	1.11E-03	PRS-CSx
Ovary	PRS7	rs3909661	4	14161125	T	G	1.53E-03	PRS-CSx
Ovary	PRS7	rs3911870	4	5860283	G	A	-1.18E-04	PRS-CSx
Ovary	PRS7	rs3913598	9	30384151	A	C	-3.02E-04	PRS-CSx
Ovary	PRS7	rs3913649	17	9597456	A	G	4.24E-04	PRS-CSx
Ovary	PRS7	rs3917265	2	102778461	T	C	-6.16E-04	PRS-CSx
Ovary	PRS7	rs3917304	2	102788125	T	G	-4.18E-04	PRS-CSx
Ovary	PRS7	rs3917318	2	102792760	G	A	-1.11E-03	PRS-CSx
Ovary	PRS7	rs3923825	3	171922127	T	C	-4.95E-04	PRS-CSx
Ovary	PRS7	rs3925584	11	30760335	C	T	7.05E-05	PRS-CSx
Ovary	PRS7	rs3931151	19	52165820	A	G	7.15E-04	PRS-CSx
Ovary	PRS7	rs3932334	3	177675859	T	C	8.80E-04	PRS-CSx
Ovary	PRS7	rs3934638	9	91570938	T	C	1.94E-04	PRS-CSx

Ovary	PRS7	rs3991715	16	57951394	G	T	1.33E-03	PRS-CSx
Ovary	PRS7	rs3991716	16	57950985	A	G	2.11E-03	PRS-CSx
Ovary	PRS7	rs401057	13	60544492	C	T	-1.24E-03	PRS-CSx
Ovary	PRS7	rs403026	6	4551432	C	T	-1.10E-03	PRS-CSx
Ovary	PRS7	rs404079	11	36407102	T	C	-1.71E-04	PRS-CSx
Ovary	PRS7	rs4072340	5	7757867	T	C	-1.62E-04	PRS-CSx
Ovary	PRS7	rs407427	2	7140693	G	A	1.30E-03	PRS-CSx
Ovary	PRS7	rs4075464	16	83148686	G	A	-5.30E-04	PRS-CSx
Ovary	PRS7	rs4076168	13	25621910	T	C	1.35E-03	PRS-CSx
Ovary	PRS7	rs4078466	3	47126543	C	T	3.25E-05	PRS-CSx
Ovary	PRS7	rs4082155	3	47125385	G	A	8.03E-05	PRS-CSx
Ovary	PRS7	rs4122861	4	90631947	C	A	-4.88E-04	PRS-CSx
Ovary	PRS7	rs4128707	12	58288363	A	G	-2.64E-04	PRS-CSx
Ovary	PRS7	rs4128999	3	167689083	G	A	-3.12E-04	PRS-CSx
Ovary	PRS7	rs4130423	3	67077961	C	T	2.19E-04	PRS-CSx
Ovary	PRS7	rs4131618	17	29204801	T	C	6.44E-04	PRS-CSx
Ovary	PRS7	rs41394347	4	112941481	C	T	-1.05E-03	PRS-CSx
Ovary	PRS7	rs4143042	13	49493648	T	C	1.89E-04	PRS-CSx
Ovary	PRS7	rs41454650	4	60042050	C	A	4.95E-04	PRS-CSx
Ovary	PRS7	rs41461052	7	92407600	C	T	3.82E-04	PRS-CSx
Ovary	PRS7	rs41507344	14	97905325	T	C	-3.07E-03	PRS-CSx
Ovary	PRS7	rs41509548	5	83639762	C	T	-7.60E-06	PRS-CSx
Ovary	PRS7	rs420013	2	7161586	C	A	3.53E-04	PRS-CSx
Ovary	PRS7	rs421269	13	62936541	C	T	1.27E-04	PRS-CSx
Ovary	PRS7	rs423151	17	28952286	G	A	4.88E-04	PRS-CSx
Ovary	PRS7	rs4233734	2	29637040	T	C	-8.36E-05	PRS-CSx
Ovary	PRS7	rs4234618	3	190550565	A	G	-2.18E-04	PRS-CSx
Ovary	PRS7	rs4234619	3	190550609	A	G	-4.06E-04	PRS-CSx
Ovary	PRS7	rs4235213	4	175769351	T	G	-5.76E-04	PRS-CSx
Ovary	PRS7	rs4235463	5	65849279	A	G	8.24E-05	PRS-CSx
Ovary	PRS7	rs4235464	5	65858406	G	A	-1.81E-06	PRS-CSx
Ovary	PRS7	rs4236051	6	36469821	T	C	2.13E-03	PRS-CSx
Ovary	PRS7	rs4237844	12	58267987	C	T	-2.07E-04	PRS-CSx
Ovary	PRS7	rs4237845	12	58302436	C	T	-2.84E-04	PRS-CSx
Ovary	PRS7	rs4239537	19	46480407	C	T	1.07E-03	PRS-CSx
Ovary	PRS7	rs4243823	1	7088855	A	G	-6.54E-04	PRS-CSx
Ovary	PRS7	rs4247350	16	66448134	C	T	-1.01E-03	PRS-CSx
Ovary	PRS7	rs4252228	5	68662136	A	G	8.71E-05	PRS-CSx
Ovary	PRS7	rs4274939	5	65875938	T	G	-5.73E-05	PRS-CSx
Ovary	PRS7	rs4293672	3	25913415	T	C	-2.76E-04	PRS-CSx
Ovary	PRS7	rs4307206	6	118813320	A	C	-8.45E-04	PRS-CSx
Ovary	PRS7	rs4319280	1	187694044	G	A	-1.70E-03	PRS-CSx
Ovary	PRS7	rs4324867	7	91101805	T	C	2.80E-03	PRS-CSx
Ovary	PRS7	rs4326353	8	128790616	G	A	-1.76E-03	PRS-CSx
Ovary	PRS7	rs4334848	5	7754231	T	C	-1.05E-04	PRS-CSx
Ovary	PRS7	rs4343997	7	3362918	A	G	9.52E-04	PRS-CSx
Ovary	PRS7	rs434658	5	54458904	G	A	8.30E-05	PRS-CSx
Ovary	PRS7	rs4349318	2	173142480	T	G	4.26E-05	PRS-CSx
Ovary	PRS7	rs436282	5	54458763	T	C	1.93E-04	PRS-CSx
Ovary	PRS7	rs4380707	5	41405404	T	C	-1.18E-03	PRS-CSx
Ovary	PRS7	rs4380897	8	130249805	G	A	1.18E-03	PRS-CSx
Ovary	PRS7	rs438531	5	73847077	T	C	4.86E-04	PRS-CSx
Ovary	PRS7	rs4388466	8	48115012	C	T	3.86E-04	PRS-CSx
Ovary	PRS7	rs439046	5	110889580	T	C	2.37E-05	PRS-CSx
Ovary	PRS7	rs4392584	5	65826548	C	A	-7.04E-05	PRS-CSx
Ovary	PRS7	rs4393158	1	209851897	A	G	-1.39E-03	PRS-CSx

Ovary	PRS7	rs4395860	8	128788985	A	G	-1.18E-03	PRS-CSx
Ovary	PRS7	rs4412019	4	76752494	C	T	7.07E-05	PRS-CSx
Ovary	PRS7	rs4415295	8	69430368	A	G	-2.46E-04	PRS-CSx
Ovary	PRS7	rs4422314	3	164548164	A	G	-1.00E-03	PRS-CSx
Ovary	PRS7	rs4428055	2	42012857	T	C	1.82E-03	PRS-CSx
Ovary	PRS7	rs4435255	16	25891829	C	T	3.99E-03	PRS-CSx
Ovary	PRS7	rs4443645	8	134105321	T	C	1.85E-05	PRS-CSx
Ovary	PRS7	rs4447616	2	167163663	T	C	1.41E-07	PRS-CSx
Ovary	PRS7	rs4450343	15	61550270	T	C	9.96E-04	PRS-CSx
Ovary	PRS7	rs4454038	5	83917033	G	A	6.99E-05	PRS-CSx
Ovary	PRS7	rs4457653	10	4943518	T	C	-1.18E-03	PRS-CSx
Ovary	PRS7	rs4457752	11	66970796	G	T	2.92E-03	PRS-CSx
Ovary	PRS7	rs4460395	8	5101602	C	A	-1.73E-04	PRS-CSx
Ovary	PRS7	rs4470571	3	167689432	C	A	-2.51E-04	PRS-CSx
Ovary	PRS7	rs4481118	3	25905593	G	A	-3.71E-04	PRS-CSx
Ovary	PRS7	rs4487583	6	14605878	G	T	2.08E-03	PRS-CSx
Ovary	PRS7	rs4492063	4	189115231	G	A	5.20E-04	PRS-CSx
Ovary	PRS7	rs4492387	8	123409560	C	T	-2.60E-04	PRS-CSx
Ovary	PRS7	rs4493692	5	169021610	C	T	2.53E-04	PRS-CSx
Ovary	PRS7	rs4495224	5	40477515	A	C	-4.84E-04	PRS-CSx
Ovary	PRS7	rs450419	2	70744107	C	T	1.69E-04	PRS-CSx
Ovary	PRS7	rs4507039	2	142199421	A	C	-1.30E-04	PRS-CSx
Ovary	PRS7	rs4508407	16	83145350	A	G	-8.49E-04	PRS-CSx
Ovary	PRS7	rs4512161	5	160491497	G	A	1.10E-04	PRS-CSx
Ovary	PRS7	rs4518720	9	103372647	A	G	-1.36E-04	PRS-CSx
Ovary	PRS7	rs452166	5	73843948	A	G	1.34E-03	PRS-CSx
Ovary	PRS7	rs4522991	5	13246979	G	A	-5.66E-05	PRS-CSx
Ovary	PRS7	rs4524460	4	110254841	T	C	1.75E-04	PRS-CSx
Ovary	PRS7	rs4530122	16	83147179	T	C	-5.45E-04	PRS-CSx
Ovary	PRS7	rs4530786	5	98657224	T	C	-2.84E-04	PRS-CSx
Ovary	PRS7	rs4535497	5	1107428	A	C	1.33E-04	PRS-CSx
Ovary	PRS7	rs4536404	15	61572131	A	G	1.15E-03	PRS-CSx
Ovary	PRS7	rs4536493	16	31503751	A	G	-1.58E-03	PRS-CSx
Ovary	PRS7	rs4539564	15	79128499	A	G	-2.65E-06	PRS-CSx
Ovary	PRS7	rs4539680	18	7184191	A	C	1.56E-03	PRS-CSx
Ovary	PRS7	rs4548164	8	143950501	G	A	4.68E-04	PRS-CSx
Ovary	PRS7	rs455828	20	45102266	T	C	5.68E-04	PRS-CSx
Ovary	PRS7	rs4558382	15	97259985	T	C	2.76E-04	PRS-CSx
Ovary	PRS7	rs456210	20	45081971	G	A	1.29E-03	PRS-CSx
Ovary	PRS7	rs4563558	4	6549294	C	A	6.92E-04	PRS-CSx
Ovary	PRS7	rs4568086	3	25885725	C	T	-5.48E-04	PRS-CSx
Ovary	PRS7	rs4570807	15	34899398	C	T	1.43E-03	PRS-CSx
Ovary	PRS7	rs4577	9	104184022	A	G	7.58E-04	PRS-CSx
Ovary	PRS7	rs4580814	5	1113244	T	G	2.73E-04	PRS-CSx
Ovary	PRS7	rs4581137	9	25655867	A	G	2.34E-03	PRS-CSx
Ovary	PRS7	rs4583845	5	65830624	C	T	-1.95E-04	PRS-CSx
Ovary	PRS7	rs4585781	8	121828919	C	A	1.89E-03	PRS-CSx
Ovary	PRS7	rs4606966	2	177542723	G	A	1.69E-05	PRS-CSx
Ovary	PRS7	rs460869	20	45077974	A	G	7.18E-04	PRS-CSx
Ovary	PRS7	rs4613874	7	15461017	A	C	-5.43E-05	PRS-CSx
Ovary	PRS7	rs461685	5	54494162	C	T	-6.80E-04	PRS-CSx
Ovary	PRS7	rs4618222	3	164586522	T	C	-2.42E-03	PRS-CSx
Ovary	PRS7	rs4622024	1	187703817	C	T	-1.94E-03	PRS-CSx
Ovary	PRS7	rs462467	22	48084299	T	C	-7.81E-04	PRS-CSx
Ovary	PRS7	rs4625679	15	93942456	A	C	9.62E-05	PRS-CSx
Ovary	PRS7	rs4635724	3	161394052	T	C	1.44E-04	PRS-CSx

Ovary	PRS7	rs4641039	8	143977819	T	C	1.83E-04	PRS-CSx
Ovary	PRS7	rs4642534	7	44315541	T	C	1.04E-04	PRS-CSx
Ovary	PRS7	rs4646583	15	58306551	A	G	-2.70E-04	PRS-CSx
Ovary	PRS7	rs4654096	1	246391677	G	A	-1.31E-04	PRS-CSx
Ovary	PRS7	rs4655030	1	22498103	T	C	-9.72E-04	PRS-CSx
Ovary	PRS7	rs4655235	1	21009800	G	A	-8.33E-04	PRS-CSx
Ovary	PRS7	rs4664398	2	161902546	T	G	8.15E-05	PRS-CSx
Ovary	PRS7	rs4665150	2	152134149	A	G	-3.10E-05	PRS-CSx
Ovary	PRS7	rs4669131	2	7232478	T	C	4.39E-04	PRS-CSx
Ovary	PRS7	rs4669746	2	11682018	G	A	-6.53E-04	PRS-CSx
Ovary	PRS7	rs4669747	2	11684315	C	T	-8.12E-04	PRS-CSx
Ovary	PRS7	rs4669868	2	12800502	A	G	-5.77E-04	PRS-CSx
Ovary	PRS7	rs467097	20	45070946	C	T	5.65E-04	PRS-CSx
Ovary	PRS7	rs4672239	2	58628304	T	C	1.70E-04	PRS-CSx
Ovary	PRS7	rs4673618	2	212310843	A	G	-4.74E-04	PRS-CSx
Ovary	PRS7	rs4674192	2	218429292	T	C	-3.54E-04	PRS-CSx
Ovary	PRS7	rs4675722	2	208909308	C	T	-2.55E-04	PRS-CSx
Ovary	PRS7	rs4675723	2	208909332	G	A	-2.63E-04	PRS-CSx
Ovary	PRS7	rs4675726	2	208909561	T	G	-4.41E-04	PRS-CSx
Ovary	PRS7	rs4679515	3	60143877	G	A	-7.60E-04	PRS-CSx
Ovary	PRS7	rs4679616	3	59317981	G	A	2.41E-03	PRS-CSx
Ovary	PRS7	rs4679642	3	60144588	C	A	-1.07E-03	PRS-CSx
Ovary	PRS7	rs4681123	3	146405343	C	T	-6.06E-04	PRS-CSx
Ovary	PRS7	rs4681336	3	146474143	A	G	-2.69E-04	PRS-CSx
Ovary	PRS7	rs4685673	3	3683525	C	T	-8.60E-04	PRS-CSx
Ovary	PRS7	rs4685917	3	5428371	T	C	-3.65E-03	PRS-CSx
Ovary	PRS7	rs4686659	3	193729048	G	A	-6.79E-05	PRS-CSx
Ovary	PRS7	rs4689472	4	6533061	A	G	1.06E-04	PRS-CSx
Ovary	PRS7	rs4695970	4	175777247	C	T	-6.11E-04	PRS-CSx
Ovary	PRS7	rs4698832	4	111249625	A	G	-1.01E-04	PRS-CSx
Ovary	PRS7	rs4698833	4	111253002	T	C	-1.24E-04	PRS-CSx
Ovary	PRS7	rs4702919	5	13141657	A	G	7.37E-04	PRS-CSx
Ovary	PRS7	rs4709528	6	158127600	T	C	-1.41E-04	PRS-CSx
Ovary	PRS7	rs4711930	6	49552535	C	T	-7.92E-05	PRS-CSx
Ovary	PRS7	rs4712373	6	19244975	C	T	-2.24E-04	PRS-CSx
Ovary	PRS7	rs4714892	6	45924967	G	A	-6.36E-05	PRS-CSx
Ovary	PRS7	rs4724039	7	1111633	C	T	-6.59E-04	PRS-CSx
Ovary	PRS7	rs4729049	7	92377183	C	T	2.58E-04	PRS-CSx
Ovary	PRS7	rs4733829	8	129063091	T	C	3.28E-04	PRS-CSx
Ovary	PRS7	rs4735170	8	93414832	C	T	1.18E-04	PRS-CSx
Ovary	PRS7	rs4735171	8	93424363	A	G	-6.41E-05	PRS-CSx
Ovary	PRS7	rs4736311	8	143952700	A	G	1.91E-04	PRS-CSx
Ovary	PRS7	rs4736312	8	143953937	A	C	1.47E-04	PRS-CSx
Ovary	PRS7	rs4736317	8	143982753	A	G	5.68E-04	PRS-CSx
Ovary	PRS7	rs4736691	8	134536088	T	C	-6.63E-05	PRS-CSx
Ovary	PRS7	rs4737806	8	55884105	T	G	-5.97E-04	PRS-CSx
Ovary	PRS7	rs4742037	9	482282	G	T	-1.21E-04	PRS-CSx
Ovary	PRS7	rs474668	1	51750909	G	A	2.27E-04	PRS-CSx
Ovary	PRS7	rs4752177	10	120357856	C	T	1.41E-03	PRS-CSx
Ovary	PRS7	rs475348	9	104179478	G	T	8.23E-04	PRS-CSx
Ovary	PRS7	rs4754957	11	103400088	A	G	1.10E-03	PRS-CSx
Ovary	PRS7	rs4759437	12	3083670	A	G	8.88E-04	PRS-CSx
Ovary	PRS7	rs4760172	12	58254015	T	C	-3.70E-04	PRS-CSx
Ovary	PRS7	rs4760346	12	58334743	A	G	-4.22E-04	PRS-CSx
Ovary	PRS7	rs4761154	12	69705362	A	G	7.17E-04	PRS-CSx
Ovary	PRS7	rs4762917	12	20178064	C	T	-6.24E-04	PRS-CSx

Ovary	PRS7	rs4763263	12	12119414	A	G	-1.06E-03	PRS-CSx
Ovary	PRS7	rs4764839	12	102300347	C	T	-2.15E-03	PRS-CSx
Ovary	PRS7	rs4764841	12	102300677	G	A	-1.99E-03	PRS-CSx
Ovary	PRS7	rs4766020	12	3089560	G	A	1.81E-03	PRS-CSx
Ovary	PRS7	rs476935	6	23958705	C	T	7.33E-04	PRS-CSx
Ovary	PRS7	rs4772190	13	100034116	A	G	9.43E-05	PRS-CSx
Ovary	PRS7	rs4772495	13	103283770	C	T	3.97E-03	PRS-CSx
Ovary	PRS7	rs4773179	13	111061884	G	A	2.26E-04	PRS-CSx
Ovary	PRS7	rs4773539	13	90558300	T	C	9.85E-05	PRS-CSx
Ovary	PRS7	rs4774395	15	61566316	T	C	1.26E-03	PRS-CSx
Ovary	PRS7	rs4777305	15	71045468	C	T	-3.64E-04	PRS-CSx
Ovary	PRS7	rs4778000	15	93066741	G	A	-2.42E-04	PRS-CSx
Ovary	PRS7	rs4778945	15	82209299	T	C	-4.71E-03	PRS-CSx
Ovary	PRS7	rs4780431	16	12498551	T	C	3.74E-04	PRS-CSx
Ovary	PRS7	rs4781223	16	12491754	A	G	1.99E-04	PRS-CSx
Ovary	PRS7	rs4781232	16	12510643	G	A	3.30E-04	PRS-CSx
Ovary	PRS7	rs478265	6	125476813	T	C	1.38E-04	PRS-CSx
Ovary	PRS7	rs478793	2	7177383	G	A	2.63E-04	PRS-CSx
Ovary	PRS7	rs4789604	17	71962659	A	C	4.06E-05	PRS-CSx
Ovary	PRS7	rs4793099	17	40686040	C	T	-3.48E-04	PRS-CSx
Ovary	PRS7	rs4793248	17	41470683	A	G	-7.30E-05	PRS-CSx
Ovary	PRS7	rs4793501	17	68718734	T	C	-4.57E-05	PRS-CSx
Ovary	PRS7	rs479623	15	35093232	T	C	2.01E-04	PRS-CSx
Ovary	PRS7	rs4797750	18	13246486	A	G	-1.33E-03	PRS-CSx
Ovary	PRS7	rs4798416	18	5930979	C	A	7.04E-04	PRS-CSx
Ovary	PRS7	rs4798749	18	8995092	T	C	-4.99E-04	PRS-CSx
Ovary	PRS7	rs4798755	18	9019456	T	G	-3.30E-03	PRS-CSx
Ovary	PRS7	rs479879	10	105945985	C	A	1.33E-04	PRS-CSx
Ovary	PRS7	rs4805303	19	29414631	T	C	-2.06E-03	PRS-CSx
Ovary	PRS7	rs4809261	20	61470725	A	G	7.79E-04	PRS-CSx
Ovary	PRS7	rs4809426	20	61482099	G	T	6.61E-04	PRS-CSx
Ovary	PRS7	rs4810046	20	55437583	T	C	3.14E-04	PRS-CSx
Ovary	PRS7	rs4813250	20	16761605	T	G	-3.01E-04	PRS-CSx
Ovary	PRS7	rs481441	10	741015	G	A	-2.49E-04	PRS-CSx
Ovary	PRS7	rs4817510	21	34361251	T	C	1.95E-03	PRS-CSx
Ovary	PRS7	rs4817511	21	34361790	G	A	-2.19E-03	PRS-CSx
Ovary	PRS7	rs481762	1	6061648	G	A	2.41E-04	PRS-CSx
Ovary	PRS7	rs4823100	22	44298257	C	T	1.95E-04	PRS-CSx
Ovary	PRS7	rs4823103	22	44304419	G	T	-2.24E-03	PRS-CSx
Ovary	PRS7	rs4831679	8	14950895	C	T	-2.18E-05	PRS-CSx
Ovary	PRS7	rs4831904	12	76359853	T	G	2.43E-04	PRS-CSx
Ovary	PRS7	rs4833253	4	123578850	G	A	-5.46E-05	PRS-CSx
Ovary	PRS7	rs4836926	9	126218055	G	A	-3.78E-04	PRS-CSx
Ovary	PRS7	rs4838931	1	110701071	C	T	-4.87E-04	PRS-CSx
Ovary	PRS7	rs4839069	1	111137811	A	G	-1.23E-03	PRS-CSx
Ovary	PRS7	rs4839165	1	110681542	G	T	-7.92E-04	PRS-CSx
Ovary	PRS7	rs4849587	2	118356291	G	A	7.11E-04	PRS-CSx
Ovary	PRS7	rs485142	5	78265876	G	A	-9.85E-04	PRS-CSx
Ovary	PRS7	rs4854033	2	241233933	C	T	2.54E-04	PRS-CSx
Ovary	PRS7	rs4854150	2	3325148	T	C	2.17E-06	PRS-CSx
Ovary	PRS7	rs4855140	3	179826491	C	A	-7.67E-04	PRS-CSx
Ovary	PRS7	rs4855268	3	164580523	T	C	-1.61E-03	PRS-CSx
Ovary	PRS7	rs4855453	3	70023667	T	C	5.67E-04	PRS-CSx
Ovary	PRS7	rs4858888	3	47405305	T	C	2.60E-05	PRS-CSx
Ovary	PRS7	rs4858890	3	47405456	G	A	3.91E-05	PRS-CSx
Ovary	PRS7	rs4865919	5	54507353	A	C	4.48E-04	PRS-CSx

Ovary	PRS7	rs486621	11	78926037	G	A	1.21E-03	PRS-CSx
Ovary	PRS7	rs4868126	5	171283469	T	G	1.61E-03	PRS-CSx
Ovary	PRS7	rs4870302	6	150449865	G	A	-2.86E-04	PRS-CSx
Ovary	PRS7	rs4873106	8	48362889	A	G	5.87E-04	PRS-CSx
Ovary	PRS7	rs4873695	8	53968277	T	C	2.17E-04	PRS-CSx
Ovary	PRS7	rs4873713	8	54001049	T	C	2.33E-04	PRS-CSx
Ovary	PRS7	rs4876978	9	91976520	T	C	-7.61E-04	PRS-CSx
Ovary	PRS7	rs4880878	10	1624284	G	A	2.14E-03	PRS-CSx
Ovary	PRS7	rs4883536	12	133236746	C	T	-3.56E-04	PRS-CSx
Ovary	PRS7	rs4885322	13	76171331	A	G	-5.97E-04	PRS-CSx
Ovary	PRS7	rs4886210	13	60711160	A	G	-5.93E-04	PRS-CSx
Ovary	PRS7	rs4888213	16	74360929	A	G	2.57E-04	PRS-CSx
Ovary	PRS7	rs4894535	3	171995605	T	C	-1.00E-03	PRS-CSx
Ovary	PRS7	rs4900164	14	93416936	A	G	-1.13E-03	PRS-CSx
Ovary	PRS7	rs4909239	7	158326087	A	G	1.20E-05	PRS-CSx
Ovary	PRS7	rs4909330	8	135644369	T	C	5.69E-04	PRS-CSx
Ovary	PRS7	rs4918489	10	112034446	A	G	6.51E-05	PRS-CSx
Ovary	PRS7	rs4920270	1	234875600	T	C	3.55E-05	PRS-CSx
Ovary	PRS7	rs4922504	10	48422754	A	G	-1.08E-03	PRS-CSx
Ovary	PRS7	rs4922505	10	48422148	C	T	-8.84E-04	PRS-CSx
Ovary	PRS7	rs4925042	17	19608773	G	A	1.61E-03	PRS-CSx
Ovary	PRS7	rs4930673	11	69052682	T	C	-1.51E-03	PRS-CSx
Ovary	PRS7	rs4938013	11	113264470	A	C	1.86E-04	PRS-CSx
Ovary	PRS7	rs4938015	11	113264644	T	C	-4.17E-05	PRS-CSx
Ovary	PRS7	rs4938058	11	113789380	G	A	4.52E-04	PRS-CSx
Ovary	PRS7	rs4940437	18	56675757	G	A	1.46E-05	PRS-CSx
Ovary	PRS7	rs4946390	6	119369961	G	A	1.68E-04	PRS-CSx
Ovary	PRS7	rs4947421	7	53244852	A	C	1.14E-03	PRS-CSx
Ovary	PRS7	rs4947861	7	50835893	C	T	5.06E-04	PRS-CSx
Ovary	PRS7	rs4948234	10	61378404	G	A	-1.77E-03	PRS-CSx
Ovary	PRS7	rs494992	11	128649851	A	G	-5.39E-05	PRS-CSx
Ovary	PRS7	rs4949967	1	96150318	G	A	6.11E-04	PRS-CSx
Ovary	PRS7	rs4952053	2	31407458	G	A	-2.42E-04	PRS-CSx
Ovary	PRS7	rs4953236	2	42333345	A	G	-1.30E-03	PRS-CSx
Ovary	PRS7	rs4954702	2	142172366	G	A	1.91E-04	PRS-CSx
Ovary	PRS7	rs4957297	5	40455074	G	A	-4.35E-04	PRS-CSx
Ovary	PRS7	rs4957300	5	40463739	C	T	-6.54E-04	PRS-CSx
Ovary	PRS7	rs4957737	5	107359523	T	C	5.16E-04	PRS-CSx
Ovary	PRS7	rs495828	9	136154867	T	G	2.98E-03	PRS-CSx
Ovary	PRS7	rs496713	1	66644337	G	A	-1.12E-03	PRS-CSx
Ovary	PRS7	rs4971278	1	247098587	C	T	-1.67E-03	PRS-CSx
Ovary	PRS7	rs4974666	4	2277497	C	T	-6.13E-05	PRS-CSx
Ovary	PRS7	rs4980069	10	81145266	G	A	-3.64E-03	PRS-CSx
Ovary	PRS7	rs4981390	14	22390103	A	G	-2.25E-03	PRS-CSx
Ovary	PRS7	rs4993569	8	94908250	G	A	9.09E-05	PRS-CSx
Ovary	PRS7	rs499893	1	153127880	T	C	-1.33E-03	PRS-CSx
Ovary	PRS7	rs5017238	8	143954769	G	A	2.16E-04	PRS-CSx
Ovary	PRS7	rs5018859	10	70955056	G	A	-5.82E-04	PRS-CSx
Ovary	PRS7	rs5030932	10	70892791	T	C	-4.36E-04	PRS-CSx
Ovary	PRS7	rs506571	9	104187400	G	A	6.43E-04	PRS-CSx
Ovary	PRS7	rs507964	6	44186390	G	T	-1.45E-03	PRS-CSx
Ovary	PRS7	rs514483	6	125489124	G	A	-8.62E-08	PRS-CSx
Ovary	PRS7	rs515910	10	105966404	G	A	1.40E-04	PRS-CSx
Ovary	PRS7	rs517403	2	25730439	A	G	6.72E-04	PRS-CSx
Ovary	PRS7	rs530050	11	107555937	T	G	-1.54E-03	PRS-CSx
Ovary	PRS7	rs5303	8	143955095	A	G	3.43E-04	PRS-CSx

Ovary	PRS7	rs537112	6	10395206	T	C	1.72E-03	PRS-CSx
Ovary	PRS7	rs538445	18	40365619	C	T	1.36E-03	PRS-CSx
Ovary	PRS7	rs540742	1	78585086	T	C	-6.80E-06	PRS-CSx
Ovary	PRS7	rs541214	6	125482425	C	T	8.70E-05	PRS-CSx
Ovary	PRS7	rs556292	10	105961957	T	C	-2.92E-06	PRS-CSx
Ovary	PRS7	rs559703	2	12787237	G	A	-2.22E-03	PRS-CSx
Ovary	PRS7	rs574112	10	739162	A	G	-2.12E-04	PRS-CSx
Ovary	PRS7	rs5747079	22	17856960	C	T	5.25E-04	PRS-CSx
Ovary	PRS7	rs5750824	22	39830123	A	G	9.99E-04	PRS-CSx
Ovary	PRS7	rs575125	18	13142415	C	A	-3.40E-04	PRS-CSx
Ovary	PRS7	rs5756202	22	36870845	C	A	-1.04E-03	PRS-CSx
Ovary	PRS7	rs5756326	22	22335639	A	G	2.66E-03	PRS-CSx
Ovary	PRS7	rs576	3	70016467	T	C	7.15E-04	PRS-CSx
Ovary	PRS7	rs5765049	22	44790362	G	T	6.98E-03	PRS-CSx
Ovary	PRS7	rs5768163	22	48317103	A	G	-2.04E-04	PRS-CSx
Ovary	PRS7	rs578597	9	104185258	T	C	6.91E-04	PRS-CSx
Ovary	PRS7	rs579010	4	17154568	C	T	-1.09E-03	PRS-CSx
Ovary	PRS7	rs579459	9	136154168	C	T	1.90E-03	PRS-CSx
Ovary	PRS7	rs581686	10	105983356	T	C	-3.40E-05	PRS-CSx
Ovary	PRS7	rs584309	10	105956776	G	A	-5.13E-05	PRS-CSx
Ovary	PRS7	rs585451	15	50007725	G	A	-3.96E-04	PRS-CSx
Ovary	PRS7	rs585463	1	96143185	A	G	9.22E-04	PRS-CSx
Ovary	PRS7	rs585635	6	158106863	G	T	-1.61E-04	PRS-CSx
Ovary	PRS7	rs593342	4	169191536	C	A	8.87E-04	PRS-CSx
Ovary	PRS7	rs5997110	22	27036505	T	C	-2.82E-04	PRS-CSx
Ovary	PRS7	rs601019	10	105976452	C	T	9.36E-05	PRS-CSx
Ovary	PRS7	rs6014056	20	53176751	G	A	1.18E-03	PRS-CSx
Ovary	PRS7	rs6017787	20	44974696	G	T	6.00E-04	PRS-CSx
Ovary	PRS7	rs6026945	20	58104030	T	G	-3.19E-03	PRS-CSx
Ovary	PRS7	rs6027132	20	58384451	C	T	-1.48E-04	PRS-CSx
Ovary	PRS7	rs6035442	20	19788016	A	G	-1.04E-03	PRS-CSx
Ovary	PRS7	rs6039442	20	9317266	C	T	-9.23E-04	PRS-CSx
Ovary	PRS7	rs6039443	20	9326769	T	G	-8.81E-04	PRS-CSx
Ovary	PRS7	rs6041541	20	12660419	C	A	-1.90E-03	PRS-CSx
Ovary	PRS7	rs6054787	20	7162079	A	G	1.92E-03	PRS-CSx
Ovary	PRS7	rs6056570	20	9334776	A	G	-1.05E-03	PRS-CSx
Ovary	PRS7	rs6064610	20	56596930	C	T	1.09E-03	PRS-CSx
Ovary	PRS7	rs607148	7	42906793	T	C	9.04E-04	PRS-CSx
Ovary	PRS7	rs6071492	20	59686217	A	G	2.65E-05	PRS-CSx
Ovary	PRS7	rs6075114	20	16775848	G	T	-2.92E-04	PRS-CSx
Ovary	PRS7	rs6075479	20	19152492	T	G	-6.42E-04	PRS-CSx
Ovary	PRS7	rs6079894	20	15565634	T	G	2.43E-03	PRS-CSx
Ovary	PRS7	rs6080411	20	16753985	G	A	-1.27E-04	PRS-CSx
Ovary	PRS7	rs6083557	20	24543008	C	T	6.41E-03	PRS-CSx
Ovary	PRS7	rs6097197	20	51645136	G	A	1.34E-03	PRS-CSx
Ovary	PRS7	rs6111277	20	16747738	A	G	-1.34E-04	PRS-CSx
Ovary	PRS7	rs6121722	20	60179532	A	G	2.07E-05	PRS-CSx
Ovary	PRS7	rs6127794	20	55149425	T	C	1.66E-03	PRS-CSx
Ovary	PRS7	rs6128254	20	56597007	C	T	1.16E-03	PRS-CSx
Ovary	PRS7	rs6128258	20	56620246	C	T	-1.51E-03	PRS-CSx
Ovary	PRS7	rs6134639	20	12679627	A	C	-1.09E-03	PRS-CSx
Ovary	PRS7	rs6136836	20	19777525	C	T	-1.21E-03	PRS-CSx
Ovary	PRS7	rs622085	13	37446108	T	C	-1.49E-04	PRS-CSx
Ovary	PRS7	rs622833	11	73365504	G	A	-6.48E-05	PRS-CSx
Ovary	PRS7	rs629427	6	158129974	T	C	-1.57E-04	PRS-CSx
Ovary	PRS7	rs6334	1	156846233	A	G	-7.95E-04	PRS-CSx

Ovary	PRS7	rs6337	1	156848995	T	C	2.31E-03	PRS-CSx
Ovary	PRS7	rs634519	13	62934964	C	T	-4.75E-05	PRS-CSx
Ovary	PRS7	rs637519	2	25760891	A	G	6.68E-04	PRS-CSx
Ovary	PRS7	rs6395	8	143956808	A	C	1.50E-04	PRS-CSx
Ovary	PRS7	rs6414541	3	171920637	C	T	-4.15E-04	PRS-CSx
Ovary	PRS7	rs6424058	1	3747580	T	C	-4.09E-05	PRS-CSx
Ovary	PRS7	rs642743	10	105933417	A	C	1.50E-04	PRS-CSx
Ovary	PRS7	rs6427850	1	200672410	T	G	-1.80E-04	PRS-CSx
Ovary	PRS7	rs6428576	1	90607718	A	G	-1.93E-04	PRS-CSx
Ovary	PRS7	rs6431	8	143993765	G	A	3.33E-04	PRS-CSx
Ovary	PRS7	rs6432322	2	12794840	T	C	-1.63E-03	PRS-CSx
Ovary	PRS7	rs6432323	2	12805980	T	C	-7.49E-04	PRS-CSx
Ovary	PRS7	rs6433	8	143993640	C	T	2.45E-04	PRS-CSx
Ovary	PRS7	rs6433327	2	152154732	A	G	3.27E-05	PRS-CSx
Ovary	PRS7	rs6435290	2	206419858	C	T	-8.37E-04	PRS-CSx
Ovary	PRS7	rs6439340	3	131797500	C	A	6.11E-05	PRS-CSx
Ovary	PRS7	rs6439655	3	136466599	G	A	-9.13E-05	PRS-CSx
Ovary	PRS7	rs6440448	3	146412213	C	T	-3.96E-04	PRS-CSx
Ovary	PRS7	rs6440677	3	150188234	A	C	-8.72E-04	PRS-CSx
Ovary	PRS7	rs6444462	3	190554485	T	C	-3.20E-04	PRS-CSx
Ovary	PRS7	rs6445044	3	171920569	G	A	-3.71E-04	PRS-CSx
Ovary	PRS7	rs6445054	3	171992009	C	T	-1.11E-03	PRS-CSx
Ovary	PRS7	rs6445055	3	171992387	A	G	-1.23E-03	PRS-CSx
Ovary	PRS7	rs6446403	4	5868022	C	T	-4.55E-05	PRS-CSx
Ovary	PRS7	rs6449116	4	15223177	C	T	-8.71E-06	PRS-CSx
Ovary	PRS7	rs645387	11	66960392	G	T	2.07E-03	PRS-CSx
Ovary	PRS7	rs6455622	6	159546872	T	G	-3.10E-03	PRS-CSx
Ovary	PRS7	rs6470637	8	129556163	G	A	-4.21E-03	PRS-CSx
Ovary	PRS7	rs6470879	8	132012870	C	T	-2.47E-04	PRS-CSx
Ovary	PRS7	rs6470883	8	132027184	C	T	-1.56E-04	PRS-CSx
Ovary	PRS7	rs6471570	8	143957405	A	C	2.95E-04	PRS-CSx
Ovary	PRS7	rs6472578	8	72219519	C	T	2.38E-03	PRS-CSx
Ovary	PRS7	rs647397	17	40679398	A	G	-6.65E-04	PRS-CSx
Ovary	PRS7	rs6475391	9	20011821	A	G	1.56E-03	PRS-CSx
Ovary	PRS7	rs6475870	9	25878901	T	C	-1.76E-03	PRS-CSx
Ovary	PRS7	rs6478485	9	123654970	A	G	-1.54E-04	PRS-CSx
Ovary	PRS7	rs6481668	10	30576645	T	C	1.24E-03	PRS-CSx
Ovary	PRS7	rs6483778	11	21539055	T	C	-3.70E-04	PRS-CSx
Ovary	PRS7	rs648511	12	63257225	A	G	-2.20E-03	PRS-CSx
Ovary	PRS7	rs6486394	11	9512121	A	G	1.13E-04	PRS-CSx
Ovary	PRS7	rs6487513	12	25937997	A	G	1.29E-03	PRS-CSx
Ovary	PRS7	rs6489416	12	3093608	A	G	1.19E-03	PRS-CSx
Ovary	PRS7	rs6491410	13	98890164	T	G	-1.36E-03	PRS-CSx
Ovary	PRS7	rs6493893	15	57113881	C	A	4.07E-05	PRS-CSx
Ovary	PRS7	rs6493894	15	57132593	C	T	8.90E-05	PRS-CSx
Ovary	PRS7	rs6494886	15	70933731	A	C	-5.15E-04	PRS-CSx
Ovary	PRS7	rs6495979	15	38847359	C	T	-6.88E-06	PRS-CSx
Ovary	PRS7	rs6499924	16	57970865	G	A	1.37E-03	PRS-CSx
Ovary	PRS7	rs6503638	17	36621604	T	C	8.09E-04	PRS-CSx
Ovary	PRS7	rs6505595	18	10798302	G	A	2.30E-03	PRS-CSx
Ovary	PRS7	rs6507199	18	34909342	G	A	1.26E-03	PRS-CSx
Ovary	PRS7	rs6507386	18	38537628	G	A	2.46E-03	PRS-CSx
Ovary	PRS7	rs6507865	18	46269095	A	G	2.25E-05	PRS-CSx
Ovary	PRS7	rs6507894	18	21338276	C	T	7.88E-04	PRS-CSx
Ovary	PRS7	rs651007	9	136153875	T	C	3.11E-03	PRS-CSx
Ovary	PRS7	rs6518680	22	17846951	A	G	5.89E-04	PRS-CSx

Ovary	PRS7	rs6530833	8	14951943	C	A	-3.60E-05	PRS-CSx
Ovary	PRS7	rs6531960	4	76768762	A	G	-4.89E-04	PRS-CSx
Ovary	PRS7	rs6532746	4	99467624	A	G	1.37E-04	PRS-CSx
Ovary	PRS7	rs6534699	4	129651071	T	C	3.94E-04	PRS-CSx
Ovary	PRS7	rs6535510	4	84831724	T	C	-2.78E-04	PRS-CSx
Ovary	PRS7	rs6540814	1	214436619	A	G	2.89E-03	PRS-CSx
Ovary	PRS7	rs6542759	2	108885895	C	T	-4.64E-05	PRS-CSx
Ovary	PRS7	rs6544852	2	46023371	T	C	-1.16E-05	PRS-CSx
Ovary	PRS7	rs6549254	3	70021899	C	A	8.18E-04	PRS-CSx
Ovary	PRS7	rs6549256	3	70022007	A	G	5.66E-04	PRS-CSx
Ovary	PRS7	rs6549565	3	67055144	A	G	1.71E-04	PRS-CSx
Ovary	PRS7	rs6551000	3	25913883	A	G	-3.40E-04	PRS-CSx
Ovary	PRS7	rs6553831	4	175755487	T	C	-5.61E-04	PRS-CSx
Ovary	PRS7	rs6555869	5	169037291	C	A	1.25E-04	PRS-CSx
Ovary	PRS7	rs6555871	5	169043892	A	C	1.92E-04	PRS-CSx
Ovary	PRS7	rs6558511	8	1640790	T	G	2.32E-04	PRS-CSx
Ovary	PRS7	rs6558551	8	1782495	A	G	2.31E-03	PRS-CSx
Ovary	PRS7	rs6562898	13	76076368	T	C	-6.52E-04	PRS-CSx
Ovary	PRS7	rs6562915	13	76170886	G	T	-7.60E-04	PRS-CSx
Ovary	PRS7	rs6563036	13	78874633	A	G	6.84E-04	PRS-CSx
Ovary	PRS7	rs6563813	13	40858172	C	T	1.28E-03	PRS-CSx
Ovary	PRS7	rs6563976	16	83792732	A	G	1.80E-04	PRS-CSx
Ovary	PRS7	rs6569115	6	120570796	A	G	3.49E-05	PRS-CSx
Ovary	PRS7	rs6570963	6	149691355	G	T	6.82E-04	PRS-CSx
Ovary	PRS7	rs6581889	12	69757429	C	T	5.78E-04	PRS-CSx
Ovary	PRS7	rs6585156	10	114224063	G	A	9.29E-04	PRS-CSx
Ovary	PRS7	rs6585338	10	85134919	T	G	3.36E-04	PRS-CSx
Ovary	PRS7	rs6586198	10	91323777	C	T	-3.04E-04	PRS-CSx
Ovary	PRS7	rs6586894	8	16646624	G	A	-1.74E-03	PRS-CSx
Ovary	PRS7	rs6589664	11	118404804	A	G	1.55E-03	PRS-CSx
Ovary	PRS7	rs6590328	11	128281264	A	G	-1.32E-03	PRS-CSx
Ovary	PRS7	rs6592325	11	86715856	A	G	1.27E-03	PRS-CSx
Ovary	PRS7	rs6593164	7	50824401	C	T	4.14E-04	PRS-CSx
Ovary	PRS7	rs6593174	7	50836486	T	C	2.63E-04	PRS-CSx
Ovary	PRS7	rs6593750	1	146725098	C	A	7.11E-04	PRS-CSx
Ovary	PRS7	rs6593752	1	146729968	T	C	4.29E-04	PRS-CSx
Ovary	PRS7	rs660368	6	158128498	T	C	-4.45E-05	PRS-CSx
Ovary	PRS7	rs661158	6	158128812	G	T	3.63E-05	PRS-CSx
Ovary	PRS7	rs661838	6	149628277	A	G	6.84E-04	PRS-CSx
Ovary	PRS7	rs663214	15	44113782	T	G	-7.14E-04	PRS-CSx
Ovary	PRS7	rs663261	4	169215390	T	C	9.55E-04	PRS-CSx
Ovary	PRS7	rs6650084	1	187697893	C	A	-1.38E-03	PRS-CSx
Ovary	PRS7	rs6651252	8	129567181	C	T	-5.59E-03	PRS-CSx
Ovary	PRS7	rs6651385	8	14962552	A	G	-3.25E-05	PRS-CSx
Ovary	PRS7	rs6660865	1	110701564	G	A	-3.57E-04	PRS-CSx
Ovary	PRS7	rs6667402	1	110679017	G	A	-1.07E-03	PRS-CSx
Ovary	PRS7	rs6683156	1	3766441	T	C	-6.40E-05	PRS-CSx
Ovary	PRS7	rs6684689	1	201062164	G	A	-1.63E-04	PRS-CSx
Ovary	PRS7	rs6686587	1	7099660	A	G	-3.03E-04	PRS-CSx
Ovary	PRS7	rs6687402	1	23031406	A	G	-8.45E-05	PRS-CSx
Ovary	PRS7	rs6691107	1	233262967	T	C	-2.11E-03	PRS-CSx
Ovary	PRS7	rs6694387	1	172292205	T	C	-5.20E-04	PRS-CSx
Ovary	PRS7	rs6698438	1	179291802	T	G	1.16E-04	PRS-CSx
Ovary	PRS7	rs6699698	1	179296008	C	T	1.20E-04	PRS-CSx
Ovary	PRS7	rs6703035	1	3732782	T	C	-2.77E-04	PRS-CSx
Ovary	PRS7	rs6703657	1	96138526	A	G	5.68E-04	PRS-CSx

Ovary	PRS7	rs6705204	2	97098095	A	G	-1.81E-03	PRS-CSx
Ovary	PRS7	rs6707969	2	174105869	T	G	4.78E-04	PRS-CSx
Ovary	PRS7	rs6709607	2	216256615	T	C	9.37E-04	PRS-CSx
Ovary	PRS7	rs6712813	2	102738026	T	C	-6.85E-04	PRS-CSx
Ovary	PRS7	rs6719628	2	62412202	A	G	1.62E-03	PRS-CSx
Ovary	PRS7	rs6720151	2	31399659	C	T	-6.74E-06	PRS-CSx
Ovary	PRS7	rs6720771	2	154753536	A	G	-2.98E-04	PRS-CSx
Ovary	PRS7	rs6723557	2	208909182	T	C	-6.16E-04	PRS-CSx
Ovary	PRS7	rs6724376	2	206399128	G	A	-8.59E-04	PRS-CSx
Ovary	PRS7	rs6726240	2	142194933	C	T	-1.95E-04	PRS-CSx
Ovary	PRS7	rs6726634	2	152159880	G	A	-5.63E-05	PRS-CSx
Ovary	PRS7	rs6727859	2	102738164	G	A	-4.96E-04	PRS-CSx
Ovary	PRS7	rs672786	6	158115787	C	T	-4.45E-04	PRS-CSx
Ovary	PRS7	rs6727985	2	102738275	G	A	-4.88E-04	PRS-CSx
Ovary	PRS7	rs6736078	2	35447208	G	A	-1.26E-04	PRS-CSx
Ovary	PRS7	rs6740315	2	177520106	T	C	-3.15E-04	PRS-CSx
Ovary	PRS7	rs674060	6	158101113	A	G	-2.87E-04	PRS-CSx
Ovary	PRS7	rs6741366	2	208880463	G	A	3.97E-04	PRS-CSx
Ovary	PRS7	rs6747086	2	154747447	T	C	-3.54E-04	PRS-CSx
Ovary	PRS7	rs6752379	2	102746705	A	G	-7.88E-04	PRS-CSx
Ovary	PRS7	rs676017	6	158129715	A	G	-1.33E-05	PRS-CSx
Ovary	PRS7	rs6762330	3	186015714	T	C	7.10E-04	PRS-CSx
Ovary	PRS7	rs6763385	3	67042519	G	A	3.25E-04	PRS-CSx
Ovary	PRS7	rs6765085	3	131801556	C	T	-3.58E-04	PRS-CSx
Ovary	PRS7	rs6766293	3	95797766	A	C	1.25E-03	PRS-CSx
Ovary	PRS7	rs6774177	3	175116207	T	C	6.30E-04	PRS-CSx
Ovary	PRS7	rs6774198	3	69616385	T	C	-2.88E-04	PRS-CSx
Ovary	PRS7	rs6776786	3	6424579	A	G	4.91E-03	PRS-CSx
Ovary	PRS7	rs6778588	3	190527427	C	A	6.80E-04	PRS-CSx
Ovary	PRS7	rs6778915	3	66956170	T	C	3.20E-04	PRS-CSx
Ovary	PRS7	rs677904	10	105957613	A	G	1.71E-04	PRS-CSx
Ovary	PRS7	rs6781449	3	69618736	T	C	-3.45E-04	PRS-CSx
Ovary	PRS7	rs6781552	3	143019749	T	G	1.30E-03	PRS-CSx
Ovary	PRS7	rs6781601	3	95798225	G	A	8.10E-04	PRS-CSx
Ovary	PRS7	rs6783281	3	84791851	A	G	-7.60E-04	PRS-CSx
Ovary	PRS7	rs6785790	3	47146932	C	T	1.35E-05	PRS-CSx
Ovary	PRS7	rs6786168	3	25885177	T	C	-7.01E-05	PRS-CSx
Ovary	PRS7	rs6788821	3	60124580	A	G	6.77E-05	PRS-CSx
Ovary	PRS7	rs6790000	3	122892164	G	A	-2.12E-04	PRS-CSx
Ovary	PRS7	rs6792462	3	23337633	A	G	6.64E-04	PRS-CSx
Ovary	PRS7	rs6794193	3	47114904	T	C	-7.48E-05	PRS-CSx
Ovary	PRS7	rs6794601	3	160946769	C	T	-4.09E-04	PRS-CSx
Ovary	PRS7	rs6799379	3	99762840	T	C	2.39E-04	PRS-CSx
Ovary	PRS7	rs6799514	3	190540022	C	T	-5.32E-04	PRS-CSx
Ovary	PRS7	rs6800444	3	167633424	C	T	-5.59E-05	PRS-CSx
Ovary	PRS7	rs6800537	3	19512074	G	A	1.29E-03	PRS-CSx
Ovary	PRS7	rs6800661	3	88892638	C	A	-8.53E-05	PRS-CSx
Ovary	PRS7	rs6800803	3	25890218	A	C	-4.11E-04	PRS-CSx
Ovary	PRS7	rs6802351	3	103910764	G	A	6.37E-04	PRS-CSx
Ovary	PRS7	rs6803181	3	171932256	C	T	-4.52E-04	PRS-CSx
Ovary	PRS7	rs6806178	3	99780393	C	A	1.95E-04	PRS-CSx
Ovary	PRS7	rs6807176	3	99760864	G	A	3.04E-04	PRS-CSx
Ovary	PRS7	rs6808072	3	194422574	C	A	-2.03E-04	PRS-CSx
Ovary	PRS7	rs6810033	3	25898413	A	G	-7.09E-04	PRS-CSx
Ovary	PRS7	rs6810130	3	25898495	A	G	-4.11E-04	PRS-CSx
Ovary	PRS7	rs6816017	4	110249828	G	T	1.82E-04	PRS-CSx

Ovary	PRS7	rs6817123	4	4776774	A	G	9.31E-06	PRS-CSx
Ovary	PRS7	rs682509	11	66943854	T	C	2.50E-03	PRS-CSx
Ovary	PRS7	rs6827963	4	5856910	G	T	1.59E-04	PRS-CSx
Ovary	PRS7	rs6827985	4	110260176	T	C	2.87E-04	PRS-CSx
Ovary	PRS7	rs6834765	4	90683990	T	C	4.07E-05	PRS-CSx
Ovary	PRS7	rs683735	6	158117108	G	A	-7.37E-04	PRS-CSx
Ovary	PRS7	rs6838020	4	5866819	A	G	-1.06E-04	PRS-CSx
Ovary	PRS7	rs6840119	4	38379495	A	G	-1.78E-04	PRS-CSx
Ovary	PRS7	rs6840524	4	182295766	A	C	1.29E-03	PRS-CSx
Ovary	PRS7	rs6843505	4	60084313	A	C	1.39E-04	PRS-CSx
Ovary	PRS7	rs6858430	4	175816129	C	T	-7.48E-04	PRS-CSx
Ovary	PRS7	rs6859578	5	41404253	A	G	-1.34E-03	PRS-CSx
Ovary	PRS7	rs6860091	5	146896655	A	G	-1.29E-04	PRS-CSx
Ovary	PRS7	rs6860722	5	98666155	G	A	-1.77E-05	PRS-CSx
Ovary	PRS7	rs6862438	5	11024961	G	A	1.08E-03	PRS-CSx
Ovary	PRS7	rs6863117	5	82282526	C	T	4.69E-04	PRS-CSx
Ovary	PRS7	rs686441	10	105950233	T	C	2.80E-05	PRS-CSx
Ovary	PRS7	rs6869734	5	160433250	C	T	-1.09E-05	PRS-CSx
Ovary	PRS7	rs6871834	5	40480187	A	G	-4.61E-04	PRS-CSx
Ovary	PRS7	rs6873523	5	177572927	C	T	-6.72E-04	PRS-CSx
Ovary	PRS7	rs6881380	5	160405275	C	T	1.57E-04	PRS-CSx
Ovary	PRS7	rs6891195	5	31566602	G	T	-6.56E-05	PRS-CSx
Ovary	PRS7	rs6891522	5	65850914	T	C	-2.35E-04	PRS-CSx
Ovary	PRS7	rs6894395	5	111809558	C	A	5.35E-05	PRS-CSx
Ovary	PRS7	rs6904457	6	148274611	T	G	3.56E-04	PRS-CSx
Ovary	PRS7	rs6905027	6	148274932	T	C	6.44E-06	PRS-CSx
Ovary	PRS7	rs6906021	6	32626311	T	C	-7.17E-04	PRS-CSx
Ovary	PRS7	rs6906287	6	118962740	C	T	-6.09E-04	PRS-CSx
Ovary	PRS7	rs6906699	6	19844355	G	A	-3.27E-03	PRS-CSx
Ovary	PRS7	rs6915740	6	9052917	C	A	2.08E-03	PRS-CSx
Ovary	PRS7	rs6916092	6	64269847	A	G	1.90E-03	PRS-CSx
Ovary	PRS7	rs6918122	6	39188691	T	C	-2.56E-04	PRS-CSx
Ovary	PRS7	rs6922310	6	55077184	G	A	-9.63E-04	PRS-CSx
Ovary	PRS7	rs6922684	6	150450034	A	G	2.98E-04	PRS-CSx
Ovary	PRS7	rs6923313	6	31241370	C	T	5.15E-04	PRS-CSx
Ovary	PRS7	rs6925565	6	17149221	G	A	-1.32E-03	PRS-CSx
Ovary	PRS7	rs6936227	6	14605133	C	A	2.10E-03	PRS-CSx
Ovary	PRS7	rs6941524	6	150381667	G	T	1.09E-03	PRS-CSx
Ovary	PRS7	rs6941843	6	71076882	C	T	1.12E-03	PRS-CSx
Ovary	PRS7	rs6943153	7	50791579	T	C	2.41E-04	PRS-CSx
Ovary	PRS7	rs6949446	7	127698403	A	G	4.13E-05	PRS-CSx
Ovary	PRS7	rs6949901	7	11555130	T	C	-1.03E-03	PRS-CSx
Ovary	PRS7	rs6950044	7	107096	A	G	9.74E-04	PRS-CSx
Ovary	PRS7	rs6952158	7	53270914	C	T	2.96E-04	PRS-CSx
Ovary	PRS7	rs6952653	7	92376127	T	C	5.25E-04	PRS-CSx
Ovary	PRS7	rs6954429	7	151512177	T	C	-1.54E-04	PRS-CSx
Ovary	PRS7	rs6954811	7	53981644	T	C	8.17E-04	PRS-CSx
Ovary	PRS7	rs6960169	7	50751829	T	C	5.99E-04	PRS-CSx
Ovary	PRS7	rs6960994	7	128692657	T	C	-1.01E-03	PRS-CSx
Ovary	PRS7	rs6963112	7	46971327	C	A	-7.35E-04	PRS-CSx
Ovary	PRS7	rs6963791	7	4845701	G	A	-6.35E-04	PRS-CSx
Ovary	PRS7	rs6965542	7	128655918	T	C	-1.00E-03	PRS-CSx
Ovary	PRS7	rs696580	3	140338805	G	A	1.08E-03	PRS-CSx
Ovary	PRS7	rs6966081	7	149427728	C	T	1.05E-03	PRS-CSx
Ovary	PRS7	rs6968827	7	50802434	A	G	4.14E-04	PRS-CSx
Ovary	PRS7	rs6969930	7	128630313	C	T	-1.25E-03	PRS-CSx

Ovary	PRS7	rs6971064	7	133740264	A	C	-1.65E-04	PRS-CSx
Ovary	PRS7	rs6976501	7	50760220	G	A	1.73E-04	PRS-CSx
Ovary	PRS7	rs698206	3	3204804	T	C	-5.09E-03	PRS-CSx
Ovary	PRS7	rs6988323	8	143946483	T	C	1.96E-04	PRS-CSx
Ovary	PRS7	rs6989881	8	55908647	G	A	-6.80E-04	PRS-CSx
Ovary	PRS7	rs6991044	8	55909324	G	A	-6.12E-04	PRS-CSx
Ovary	PRS7	rs6992863	8	128269154	T	C	3.48E-03	PRS-CSx
Ovary	PRS7	rs699555	4	45555426	C	T	-2.03E-03	PRS-CSx
Ovary	PRS7	rs7001209	8	57354592	A	C	-4.88E-04	PRS-CSx
Ovary	PRS7	rs7003319	8	143954747	T	C	3.43E-04	PRS-CSx
Ovary	PRS7	rs7003746	8	29552383	A	G	-8.48E-05	PRS-CSx
Ovary	PRS7	rs7004326	8	135610915	A	C	4.28E-04	PRS-CSx
Ovary	PRS7	rs7005638	8	143950214	T	C	3.64E-04	PRS-CSx
Ovary	PRS7	rs7008202	8	132074410	C	A	-7.66E-05	PRS-CSx
Ovary	PRS7	rs7008755	8	72233744	G	A	5.18E-04	PRS-CSx
Ovary	PRS7	rs7011830	8	143982392	A	C	1.70E-04	PRS-CSx
Ovary	PRS7	rs7012866	8	135616959	T	G	6.54E-04	PRS-CSx
Ovary	PRS7	rs701428	22	20228542	A	G	5.19E-06	PRS-CSx
Ovary	PRS7	rs7014689	8	49038732	T	C	1.89E-03	PRS-CSx
Ovary	PRS7	rs7014971	8	66990472	C	T	-1.52E-03	PRS-CSx
Ovary	PRS7	rs7015452	8	1400984	T	C	1.95E-04	PRS-CSx
Ovary	PRS7	rs7015642	8	14949079	A	G	2.15E-04	PRS-CSx
Ovary	PRS7	rs7016088	8	143950026	G	A	3.45E-04	PRS-CSx
Ovary	PRS7	rs7016594	8	143950352	G	A	2.38E-04	PRS-CSx
Ovary	PRS7	rs7017252	8	129950844	T	C	1.77E-04	PRS-CSx
Ovary	PRS7	rs7017310	8	18318883	T	C	-1.32E-03	PRS-CSx
Ovary	PRS7	rs7019530	9	16936082	A	G	-4.03E-05	PRS-CSx
Ovary	PRS7	rs7020876	9	85715039	C	T	-1.14E-03	PRS-CSx
Ovary	PRS7	rs702136	9	1551207	T	C	8.59E-04	PRS-CSx
Ovary	PRS7	rs702140	9	1564810	T	C	6.58E-04	PRS-CSx
Ovary	PRS7	rs7021631	9	7503994	C	T	3.56E-04	PRS-CSx
Ovary	PRS7	rs7027192	9	98406181	T	C	-1.29E-03	PRS-CSx
Ovary	PRS7	rs7034844	9	103585379	A	G	-2.59E-03	PRS-CSx
Ovary	PRS7	rs7035981	9	16936283	C	T	-2.90E-04	PRS-CSx
Ovary	PRS7	rs703667	12	105067844	G	A	2.23E-03	PRS-CSx
Ovary	PRS7	rs7040729	9	91970477	G	A	-6.63E-04	PRS-CSx
Ovary	PRS7	rs704246	3	70016904	T	C	1.00E-03	PRS-CSx
Ovary	PRS7	rs7046355	9	125502869	T	C	-9.70E-04	PRS-CSx
Ovary	PRS7	rs7047256	9	83027012	A	C	1.68E-04	PRS-CSx
Ovary	PRS7	rs705509	1	65531751	A	G	1.54E-03	PRS-CSx
Ovary	PRS7	rs7068676	10	85183729	C	T	-8.13E-04	PRS-CSx
Ovary	PRS7	rs7072870	10	71380320	A	G	-1.40E-04	PRS-CSx
Ovary	PRS7	rs7082255	10	72216011	G	A	1.33E-04	PRS-CSx
Ovary	PRS7	rs708308	3	102615508	G	A	-1.07E-03	PRS-CSx
Ovary	PRS7	rs7085145	10	26764577	G	A	-3.58E-03	PRS-CSx
Ovary	PRS7	rs7088204	10	10947277	G	A	1.78E-04	PRS-CSx
Ovary	PRS7	rs7090372	10	11075133	T	C	2.21E-04	PRS-CSx
Ovary	PRS7	rs7091662	10	121443168	G	A	-3.62E-04	PRS-CSx
Ovary	PRS7	rs709749	1	82799677	A	G	3.77E-04	PRS-CSx
Ovary	PRS7	rs7107502	11	122830214	A	G	-2.97E-05	PRS-CSx
Ovary	PRS7	rs7111734	11	37268971	T	C	5.44E-05	PRS-CSx
Ovary	PRS7	rs7113211	11	112642632	A	G	2.39E-04	PRS-CSx
Ovary	PRS7	rs711619	3	3207754	T	C	-3.75E-03	PRS-CSx
Ovary	PRS7	rs7122009	11	86710510	T	C	1.33E-03	PRS-CSx
Ovary	PRS7	rs7125517	11	117317124	A	C	1.15E-04	PRS-CSx
Ovary	PRS7	rs7130431	11	113238223	T	G	1.78E-04	PRS-CSx

Ovary	PRS7	rs7131741	12	78251906	G	T	-1.25E-03	PRS-CSx
Ovary	PRS7	rs7135092	12	69658330	C	T	6.71E-04	PRS-CSx
Ovary	PRS7	rs7141105	14	70673738	C	T	-7.58E-04	PRS-CSx
Ovary	PRS7	rs714326	10	24622926	C	T	1.44E-03	PRS-CSx
Ovary	PRS7	rs7148858	14	95748715	C	T	6.13E-05	PRS-CSx
Ovary	PRS7	rs7150290	14	53151109	C	T	-1.26E-03	PRS-CSx
Ovary	PRS7	rs7151951	14	72942613	T	C	-4.79E-04	PRS-CSx
Ovary	PRS7	rs716051	4	113964288	G	A	-1.93E-04	PRS-CSx
Ovary	PRS7	rs716168	10	114228163	A	G	1.31E-03	PRS-CSx
Ovary	PRS7	rs716169	10	114228444	C	T	2.14E-03	PRS-CSx
Ovary	PRS7	rs7163859	15	79552585	G	A	9.74E-05	PRS-CSx
Ovary	PRS7	rs716417	1	58178715	T	C	2.59E-05	PRS-CSx
Ovary	PRS7	rs7166062	15	61214322	C	T	1.11E-04	PRS-CSx
Ovary	PRS7	rs7170151	15	38846678	C	T	5.70E-05	PRS-CSx
Ovary	PRS7	rs7173511	15	79550410	T	C	-3.48E-04	PRS-CSx
Ovary	PRS7	rs7173565	15	38850330	T	C	-2.55E-04	PRS-CSx
Ovary	PRS7	rs7177417	15	79547515	T	G	-9.69E-05	PRS-CSx
Ovary	PRS7	rs718079	17	46709835	A	G	-3.17E-04	PRS-CSx
Ovary	PRS7	rs7193889	16	19369306	G	T	-6.20E-04	PRS-CSx
Ovary	PRS7	rs7196583	16	83144850	C	T	-8.36E-04	PRS-CSx
Ovary	PRS7	rs719697	20	15555463	A	G	-3.40E-03	PRS-CSx
Ovary	PRS7	rs719802	11	113234679	T	C	2.28E-04	PRS-CSx
Ovary	PRS7	rs7198093	16	85226863	G	A	5.12E-04	PRS-CSx
Ovary	PRS7	rs7198263	16	83654956	G	T	1.01E-03	PRS-CSx
Ovary	PRS7	rs7201205	16	74323442	T	G	5.24E-04	PRS-CSx
Ovary	PRS7	rs7203024	16	80553903	C	A	-1.30E-03	PRS-CSx
Ovary	PRS7	rs7203154	16	83793018	G	A	-7.89E-05	PRS-CSx
Ovary	PRS7	rs7215998	17	69074425	G	A	-8.79E-04	PRS-CSx
Ovary	PRS7	rs7216570	17	14931979	G	A	2.42E-03	PRS-CSx
Ovary	PRS7	rs7220372	17	40656043	G	T	1.32E-05	PRS-CSx
Ovary	PRS7	rs7220651	17	9590319	T	G	7.64E-04	PRS-CSx
Ovary	PRS7	rs7223784	17	40577580	C	A	-9.85E-04	PRS-CSx
Ovary	PRS7	rs7224006	17	57896338	T	C	-5.54E-04	PRS-CSx
Ovary	PRS7	rs7224721	17	13444373	C	T	6.77E-04	PRS-CSx
Ovary	PRS7	rs7229430	18	29066083	A	G	4.63E-03	PRS-CSx
Ovary	PRS7	rs7229478	18	35606660	A	G	-5.88E-04	PRS-CSx
Ovary	PRS7	rs723053	6	16670698	C	T	3.61E-05	PRS-CSx
Ovary	PRS7	rs7231057	18	55357391	G	A	8.05E-04	PRS-CSx
Ovary	PRS7	rs723297	11	9515319	A	C	2.21E-04	PRS-CSx
Ovary	PRS7	rs7239804	18	73267883	T	C	-1.34E-03	PRS-CSx
Ovary	PRS7	rs7241782	18	10801724	A	G	3.26E-03	PRS-CSx
Ovary	PRS7	rs7242453	18	22624119	C	T	-1.37E-04	PRS-CSx
Ovary	PRS7	rs7251374	19	36628463	T	C	-2.72E-03	PRS-CSx
Ovary	PRS7	rs7253254	19	1027797	C	A	1.67E-03	PRS-CSx
Ovary	PRS7	rs7257330	19	30301823	A	G	3.64E-05	PRS-CSx
Ovary	PRS7	rs727231	2	172278694	T	C	2.76E-03	PRS-CSx
Ovary	PRS7	rs7279594	21	19659074	T	C	4.59E-03	PRS-CSx
Ovary	PRS7	rs729313	11	9501747	A	C	1.06E-04	PRS-CSx
Ovary	PRS7	rs7296742	12	95147249	A	G	3.31E-03	PRS-CSx
Ovary	PRS7	rs729804	7	31939381	C	T	7.29E-04	PRS-CSx
Ovary	PRS7	rs7298751	12	125380232	G	A	-9.02E-05	PRS-CSx
Ovary	PRS7	rs7299249	12	58343710	C	T	-1.09E-03	PRS-CSx
Ovary	PRS7	rs7300266	12	69654187	G	A	7.85E-04	PRS-CSx
Ovary	PRS7	rs7303424	12	16232618	C	T	1.30E-04	PRS-CSx
Ovary	PRS7	rs7306261	12	88273027	T	C	1.89E-05	PRS-CSx
Ovary	PRS7	rs7306587	12	65276032	A	G	1.39E-03	PRS-CSx

Ovary	PRS7	rs7308481	12	69659299	G	A	4.93E-04	PRS-CSx
Ovary	PRS7	rs7310294	12	107690351	T	C	6.61E-04	PRS-CSx
Ovary	PRS7	rs7310707	12	107693844	C	T	1.27E-03	PRS-CSx
Ovary	PRS7	rs7311657	12	65270436	A	G	5.82E-04	PRS-CSx
Ovary	PRS7	rs7312623	12	58262523	G	T	-4.57E-04	PRS-CSx
Ovary	PRS7	rs7313074	12	57154489	G	A	3.68E-04	PRS-CSx
Ovary	PRS7	rs7313171	12	4928871	C	T	1.24E-03	PRS-CSx
Ovary	PRS7	rs7313501	12	53255044	T	C	7.87E-04	PRS-CSx
Ovary	PRS7	rs7314819	12	95794053	A	C	-2.49E-03	PRS-CSx
Ovary	PRS7	rs731582	12	76362129	T	C	6.84E-05	PRS-CSx
Ovary	PRS7	rs7317250	13	76167141	G	A	-5.26E-04	PRS-CSx
Ovary	PRS7	rs7318517	13	40857430	G	A	9.17E-04	PRS-CSx
Ovary	PRS7	rs7319420	13	19640445	T	C	-1.62E-03	PRS-CSx
Ovary	PRS7	rs733190	1	172095226	T	C	-5.83E-04	PRS-CSx
Ovary	PRS7	rs7334658	13	106558376	T	C	-1.26E-03	PRS-CSx
Ovary	PRS7	rs7334785	13	40771985	A	G	-1.71E-03	PRS-CSx
Ovary	PRS7	rs7342938	17	29189830	G	A	2.88E-04	PRS-CSx
Ovary	PRS7	rs7354897	1	162800426	C	T	-2.60E-05	PRS-CSx
Ovary	PRS7	rs7379278	5	177562815	G	A	-8.25E-04	PRS-CSx
Ovary	PRS7	rs738331	22	39714216	A	G	9.44E-04	PRS-CSx
Ovary	PRS7	rs7387547	8	1782876	G	A	2.12E-03	PRS-CSx
Ovary	PRS7	rs740876	19	30626963	T	G	6.88E-04	PRS-CSx
Ovary	PRS7	rs742546	1	40290964	C	T	7.01E-05	PRS-CSx
Ovary	PRS7	rs7426144	2	206405970	G	A	-4.31E-04	PRS-CSx
Ovary	PRS7	rs7444275	5	135699217	G	A	8.34E-05	PRS-CSx
Ovary	PRS7	rs7458839	7	127495797	T	C	3.11E-04	PRS-CSx
Ovary	PRS7	rs7462972	8	66384556	G	A	-4.43E-04	PRS-CSx
Ovary	PRS7	rs7469084	9	91981163	C	T	-5.14E-04	PRS-CSx
Ovary	PRS7	rs7469270	9	16942496	C	T	-3.62E-04	PRS-CSx
Ovary	PRS7	rs747453	13	78881101	C	T	7.19E-04	PRS-CSx
Ovary	PRS7	rs749067	11	47318157	C	T	1.41E-03	PRS-CSx
Ovary	PRS7	rs7501530	17	7903610	T	C	2.32E-04	PRS-CSx
Ovary	PRS7	rs7503542	17	29073830	C	A	3.21E-04	PRS-CSx
Ovary	PRS7	rs751933	6	39164121	T	C	1.17E-04	PRS-CSx
Ovary	PRS7	rs751934	6	39164076	T	C	-2.28E-04	PRS-CSx
Ovary	PRS7	rs7522641	1	179269788	G	A	8.99E-05	PRS-CSx
Ovary	PRS7	rs752335	19	35381406	C	A	6.85E-04	PRS-CSx
Ovary	PRS7	rs7524066	1	92184814	T	G	-7.00E-04	PRS-CSx
Ovary	PRS7	rs7531070	1	246367971	C	T	-2.57E-05	PRS-CSx
Ovary	PRS7	rs7534118	1	104562298	T	C	2.50E-04	PRS-CSx
Ovary	PRS7	rs7535775	1	3719057	G	A	-9.66E-05	PRS-CSx
Ovary	PRS7	rs7539358	1	246369599	A	G	-2.07E-04	PRS-CSx
Ovary	PRS7	rs7541616	1	20580487	C	T	1.59E-03	PRS-CSx
Ovary	PRS7	rs7541663	1	51544465	T	C	-2.69E-04	PRS-CSx
Ovary	PRS7	rs7541874	1	81489305	A	G	1.55E-03	PRS-CSx
Ovary	PRS7	rs7542208	1	4202818	A	G	1.06E-03	PRS-CSx
Ovary	PRS7	rs7557377	2	102736588	T	C	-8.13E-04	PRS-CSx
Ovary	PRS7	rs755859	16	74277844	T	C	6.94E-04	PRS-CSx
Ovary	PRS7	rs7561607	2	121580431	C	T	-2.15E-04	PRS-CSx
Ovary	PRS7	rs7565424	2	241243780	G	A	1.95E-04	PRS-CSx
Ovary	PRS7	rs7567367	2	7231665	A	G	4.92E-04	PRS-CSx
Ovary	PRS7	rs7568108	2	11683324	G	A	-6.79E-04	PRS-CSx
Ovary	PRS7	rs7568330	2	9975557	G	A	-5.69E-05	PRS-CSx
Ovary	PRS7	rs7570373	2	31399358	G	T	-2.96E-04	PRS-CSx
Ovary	PRS7	rs7576602	2	241684692	T	C	1.02E-03	PRS-CSx
Ovary	PRS7	rs758129	15	89900887	A	G	-1.57E-04	PRS-CSx

Ovary	PRS7	rs7582396	2	11664349	C	T	-2.37E-04	PRS-CSx
Ovary	PRS7	rs7583013	2	208901249	C	T	-2.01E-04	PRS-CSx
Ovary	PRS7	rs7583434	2	145858797	G	A	-7.45E-04	PRS-CSx
Ovary	PRS7	rs7585303	2	97114152	G	A	-1.02E-03	PRS-CSx
Ovary	PRS7	rs7587839	2	170360397	T	C	5.11E-05	PRS-CSx
Ovary	PRS7	rs7591598	2	236358314	A	G	-2.18E-04	PRS-CSx
Ovary	PRS7	rs7592930	2	119534331	G	A	-1.69E-03	PRS-CSx
Ovary	PRS7	rs7595499	2	242896889	C	A	1.33E-03	PRS-CSx
Ovary	PRS7	rs7595734	2	208869513	T	C	2.81E-04	PRS-CSx
Ovary	PRS7	rs7599288	2	212216178	G	A	-4.18E-04	PRS-CSx
Ovary	PRS7	rs7605017	2	170575812	A	C	7.90E-06	PRS-CSx
Ovary	PRS7	rs7608941	2	224854068	A	C	-6.61E-06	PRS-CSx
Ovary	PRS7	rs7610636	3	47064436	C	A	-6.41E-05	PRS-CSx
Ovary	PRS7	rs7613282	3	47389409	T	C	3.05E-05	PRS-CSx
Ovary	PRS7	rs7613969	3	23333023	G	A	6.52E-04	PRS-CSx
Ovary	PRS7	rs7614439	3	77521306	C	T	-1.16E-03	PRS-CSx
Ovary	PRS7	rs7614617	3	159198139	C	T	1.79E-04	PRS-CSx
Ovary	PRS7	rs761516	1	186961326	T	G	-2.24E-04	PRS-CSx
Ovary	PRS7	rs761517	1	186961387	G	A	-4.80E-04	PRS-CSx
Ovary	PRS7	rs7616988	3	99701134	G	A	3.98E-04	PRS-CSx
Ovary	PRS7	rs7617994	3	19522955	G	A	1.28E-03	PRS-CSx
Ovary	PRS7	rs7630350	3	69637329	G	A	1.48E-04	PRS-CSx
Ovary	PRS7	rs7632427	3	89534377	C	T	-9.42E-04	PRS-CSx
Ovary	PRS7	rs763254	6	118886615	C	T	-4.26E-04	PRS-CSx
Ovary	PRS7	rs7634770	3	66930228	C	A	1.54E-04	PRS-CSx
Ovary	PRS7	rs7638900	3	99670773	T	C	3.04E-04	PRS-CSx
Ovary	PRS7	rs7639900	3	66906308	T	G	1.65E-03	PRS-CSx
Ovary	PRS7	rs7646131	3	164585832	G	A	-2.03E-03	PRS-CSx
Ovary	PRS7	rs764931	1	229080069	A	G	2.42E-04	PRS-CSx
Ovary	PRS7	rs7654351	4	154441208	A	G	-3.31E-05	PRS-CSx
Ovary	PRS7	rs7657942	4	104540960	A	G	-3.27E-03	PRS-CSx
Ovary	PRS7	rs7661330	4	90663670	T	G	-1.18E-04	PRS-CSx
Ovary	PRS7	rs7664318	4	123599124	G	A	1.15E-04	PRS-CSx
Ovary	PRS7	rs7664871	4	7679806	G	A	1.22E-04	PRS-CSx
Ovary	PRS7	rs7665871	4	23076898	A	G	-3.34E-03	PRS-CSx
Ovary	PRS7	rs766797	6	79059790	A	G	7.62E-04	PRS-CSx
Ovary	PRS7	rs7673867	4	110278483	A	C	9.06E-05	PRS-CSx
Ovary	PRS7	rs7674119	4	176378706	C	T	-5.38E-03	PRS-CSx
Ovary	PRS7	rs7675604	4	135264608	C	T	-3.96E-04	PRS-CSx
Ovary	PRS7	rs7677522	4	12320759	A	C	-1.62E-03	PRS-CSx
Ovary	PRS7	rs7691078	4	5865361	C	A	-1.61E-04	PRS-CSx
Ovary	PRS7	rs7693537	4	135295421	G	A	-3.24E-04	PRS-CSx
Ovary	PRS7	rs7694722	4	184487521	A	G	5.79E-03	PRS-CSx
Ovary	PRS7	rs7694843	4	10712629	T	C	3.67E-04	PRS-CSx
Ovary	PRS7	rs7696758	4	76757884	A	C	-1.60E-05	PRS-CSx
Ovary	PRS7	rs7699155	4	59624687	G	T	-7.52E-05	PRS-CSx
Ovary	PRS7	rs7702184	5	11024270	C	T	1.74E-03	PRS-CSx
Ovary	PRS7	rs7703786	5	160453756	C	T	2.62E-06	PRS-CSx
Ovary	PRS7	rs7705714	5	125971699	T	C	2.47E-04	PRS-CSx
Ovary	PRS7	rs770576	11	99884272	G	A	1.42E-03	PRS-CSx
Ovary	PRS7	rs7712318	5	68653601	C	A	2.03E-04	PRS-CSx
Ovary	PRS7	rs771268	2	7132067	A	G	4.21E-04	PRS-CSx
Ovary	PRS7	rs771920	9	1554754	T	C	7.95E-04	PRS-CSx
Ovary	PRS7	rs771922	9	1555134	C	A	6.12E-04	PRS-CSx
Ovary	PRS7	rs7719941	5	123914126	T	C	3.34E-03	PRS-CSx
Ovary	PRS7	rs7732415	5	177572561	G	A	-7.55E-04	PRS-CSx

Ovary	PRS7	rs7742814	6	119079281	G	A	-5.88E-04	PRS-CSx
Ovary	PRS7	rs7755236	6	113918650	T	C	8.36E-04	PRS-CSx
Ovary	PRS7	rs7757271	6	81417782	C	T	7.49E-04	PRS-CSx
Ovary	PRS7	rs775728	3	77639136	A	G	8.52E-04	PRS-CSx
Ovary	PRS7	rs7759298	6	162514407	A	G	-2.38E-03	PRS-CSx
Ovary	PRS7	rs7765824	6	119084644	G	T	-5.36E-04	PRS-CSx
Ovary	PRS7	rs7766023	6	158132992	A	G	-1.17E-04	PRS-CSx
Ovary	PRS7	rs7779576	7	3365572	T	G	1.01E-03	PRS-CSx
Ovary	PRS7	rs7785159	7	52588348	T	C	2.56E-04	PRS-CSx
Ovary	PRS7	rs778563	4	63328312	A	G	-8.77E-04	PRS-CSx
Ovary	PRS7	rs778939	4	63243787	T	C	-1.20E-03	PRS-CSx
Ovary	PRS7	rs7789596	7	134179315	T	C	-2.90E-03	PRS-CSx
Ovary	PRS7	rs7794299	7	50783191	A	G	6.26E-04	PRS-CSx
Ovary	PRS7	rs7794693	7	147296158	A	C	2.27E-03	PRS-CSx
Ovary	PRS7	rs7796238	7	77670277	A	C	-1.00E-04	PRS-CSx
Ovary	PRS7	rs7799786	7	198440	A	G	4.87E-03	PRS-CSx
Ovary	PRS7	rs7805508	7	8269234	C	T	-3.09E-03	PRS-CSx
Ovary	PRS7	rs7808523	7	71637229	G	A	3.05E-04	PRS-CSx
Ovary	PRS7	rs7808971	7	138125434	A	G	6.23E-03	PRS-CSx
Ovary	PRS7	rs7809828	7	16822054	A	G	-1.75E-03	PRS-CSx
Ovary	PRS7	rs7810629	7	96989624	G	A	1.71E-04	PRS-CSx
Ovary	PRS7	rs7812327	8	62554412	T	C	3.12E-06	PRS-CSx
Ovary	PRS7	rs7815993	8	129951769	T	C	7.70E-05	PRS-CSx
Ovary	PRS7	rs7818331	8	135639653	C	T	4.77E-04	PRS-CSx
Ovary	PRS7	rs7821053	8	14951436	T	C	4.57E-05	PRS-CSx
Ovary	PRS7	rs7822208	8	1194973	T	C	1.93E-03	PRS-CSx
Ovary	PRS7	rs7822849	8	121827840	T	C	2.41E-03	PRS-CSx
Ovary	PRS7	rs7824948	8	14942783	T	C	-4.13E-04	PRS-CSx
Ovary	PRS7	rs7826320	8	19152556	A	G	-3.14E-04	PRS-CSx
Ovary	PRS7	rs7829923	8	93442101	C	T	1.86E-04	PRS-CSx
Ovary	PRS7	rs7835388	8	55911216	C	T	-4.85E-04	PRS-CSx
Ovary	PRS7	rs7835556	8	1643051	A	G	-3.45E-07	PRS-CSx
Ovary	PRS7	rs7838267	8	14951007	A	C	-1.66E-04	PRS-CSx
Ovary	PRS7	rs7848044	9	31928731	G	A	1.26E-03	PRS-CSx
Ovary	PRS7	rs7849050	9	24750179	T	G	-1.32E-03	PRS-CSx
Ovary	PRS7	rs7851988	9	91953252	C	T	-5.74E-04	PRS-CSx
Ovary	PRS7	rs7855043	9	117318726	G	A	7.42E-04	PRS-CSx
Ovary	PRS7	rs7856857	9	104449593	C	A	-1.24E-03	PRS-CSx
Ovary	PRS7	rs7866760	9	107950174	G	A	3.63E-03	PRS-CSx
Ovary	PRS7	rs786772	10	48601573	A	G	4.84E-04	PRS-CSx
Ovary	PRS7	rs786795	10	48624360	A	G	6.51E-04	PRS-CSx
Ovary	PRS7	rs786823	10	48651534	G	A	-2.25E-04	PRS-CSx
Ovary	PRS7	rs789970	10	105977721	C	T	-2.89E-05	PRS-CSx
Ovary	PRS7	rs7902364	10	117701144	A	G	-4.89E-03	PRS-CSx
Ovary	PRS7	rs7905405	10	61385195	T	C	-5.23E-03	PRS-CSx
Ovary	PRS7	rs7909330	10	67103240	C	T	-4.69E-04	PRS-CSx
Ovary	PRS7	rs7918387	10	16018899	C	T	4.75E-04	PRS-CSx
Ovary	PRS7	rs7920142	10	98429594	A	G	-1.16E-03	PRS-CSx
Ovary	PRS7	rs7931135	11	112611579	G	A	5.72E-05	PRS-CSx
Ovary	PRS7	rs7931713	11	103477914	T	C	-3.04E-04	PRS-CSx
Ovary	PRS7	rs7932792	11	9703877	G	T	1.53E-03	PRS-CSx
Ovary	PRS7	rs793440	3	99643176	T	C	2.60E-04	PRS-CSx
Ovary	PRS7	rs793503	3	99588070	G	A	1.89E-04	PRS-CSx
Ovary	PRS7	rs7936389	11	5584496	G	A	3.05E-03	PRS-CSx
Ovary	PRS7	rs7937611	11	66882995	C	T	1.56E-03	PRS-CSx
Ovary	PRS7	rs7944986	11	19841363	G	A	-4.79E-04	PRS-CSx

Ovary	PRS7	rs7945646	11	75885321	G	A	2.07E-03	PRS-CSx
Ovary	PRS7	rs794614	6	119284884	G	A	1.87E-05	PRS-CSx
Ovary	PRS7	rs794728	5	173872986	G	T	2.22E-03	PRS-CSx
Ovary	PRS7	rs7951615	11	112639548	A	G	-4.99E-05	PRS-CSx
Ovary	PRS7	rs7954957	12	58299250	C	T	-4.10E-05	PRS-CSx
Ovary	PRS7	rs7956222	12	101206568	C	T	-1.88E-04	PRS-CSx
Ovary	PRS7	rs7964046	12	20278266	C	T	-1.82E-03	PRS-CSx
Ovary	PRS7	rs7964475	12	131631486	T	C	-2.24E-03	PRS-CSx
Ovary	PRS7	rs7969470	12	80793341	A	G	4.23E-04	PRS-CSx
Ovary	PRS7	rs7971677	12	125411863	G	A	9.14E-05	PRS-CSx
Ovary	PRS7	rs7971877	12	58287630	T	G	-3.30E-04	PRS-CSx
Ovary	PRS7	rs7973157	12	57049193	C	T	1.89E-04	PRS-CSx
Ovary	PRS7	rs7977676	12	24431300	G	A	4.60E-05	PRS-CSx
Ovary	PRS7	rs7978685	12	57103154	T	C	4.82E-04	PRS-CSx
Ovary	PRS7	rs7979569	12	89642933	C	T	-1.71E-03	PRS-CSx
Ovary	PRS7	rs7986566	13	76197931	C	T	-6.40E-04	PRS-CSx
Ovary	PRS7	rs7988257	13	93465539	A	C	-1.94E-03	PRS-CSx
Ovary	PRS7	rs799019	2	34363949	A	G	-9.95E-04	PRS-CSx
Ovary	PRS7	rs7990930	13	108454595	T	C	6.68E-05	PRS-CSx
Ovary	PRS7	rs7995077	13	23827815	A	G	-1.76E-04	PRS-CSx
Ovary	PRS7	rs7996252	13	78876537	C	T	5.57E-04	PRS-CSx
Ovary	PRS7	rs7996348	13	103272017	A	G	2.89E-03	PRS-CSx
Ovary	PRS7	rs8007645	14	22393148	C	T	-1.66E-03	PRS-CSx
Ovary	PRS7	rs8010011	14	90229049	T	G	9.09E-04	PRS-CSx
Ovary	PRS7	rs8016185	14	101533384	C	T	-5.27E-04	PRS-CSx
Ovary	PRS7	rs8022130	14	96542273	C	T	3.81E-03	PRS-CSx
Ovary	PRS7	rs8024685	15	79552364	A	G	-4.05E-04	PRS-CSx
Ovary	PRS7	rs8025761	15	55818610	A	G	1.42E-03	PRS-CSx
Ovary	PRS7	rs8026315	15	61569516	T	G	6.74E-04	PRS-CSx
Ovary	PRS7	rs8032939	15	38834033	T	C	-1.26E-04	PRS-CSx
Ovary	PRS7	rs8035957	15	38838264	T	C	-1.51E-04	PRS-CSx
Ovary	PRS7	rs8043085	15	38828140	T	G	-1.52E-04	PRS-CSx
Ovary	PRS7	rs8043724	16	12490338	C	T	1.71E-04	PRS-CSx
Ovary	PRS7	rs8045581	16	74263384	C	T	6.10E-04	PRS-CSx
Ovary	PRS7	rs8045810	16	66453765	T	C	-1.24E-03	PRS-CSx
Ovary	PRS7	rs8050957	16	66451217	C	T	-6.84E-04	PRS-CSx
Ovary	PRS7	rs8056650	16	59890698	A	G	2.49E-03	PRS-CSx
Ovary	PRS7	rs8057483	16	80456443	T	G	8.19E-07	PRS-CSx
Ovary	PRS7	rs8060246	16	86436817	C	T	2.78E-04	PRS-CSx
Ovary	PRS7	rs8060557	16	63799031	A	C	1.13E-04	PRS-CSx
Ovary	PRS7	rs8067384	17	40672739	A	G	-7.65E-05	PRS-CSx
Ovary	PRS7	rs8069818	17	8323404	T	C	-3.02E-04	PRS-CSx
Ovary	PRS7	rs8074700	17	46749791	A	C	-4.59E-04	PRS-CSx
Ovary	PRS7	rs8076894	17	5996487	G	A	-2.03E-03	PRS-CSx
Ovary	PRS7	rs807931	3	47389317	C	T	-2.07E-04	PRS-CSx
Ovary	PRS7	rs807932	3	47354241	C	T	-1.13E-04	PRS-CSx
Ovary	PRS7	rs807936	3	47322496	C	T	1.22E-04	PRS-CSx
Ovary	PRS7	rs8079830	17	70004026	T	C	2.04E-03	PRS-CSx
Ovary	PRS7	rs808	3	56761650	G	A	1.57E-04	PRS-CSx
Ovary	PRS7	rs8081468	17	57836924	A	G	-7.62E-04	PRS-CSx
Ovary	PRS7	rs8090527	18	48784027	T	C	-9.00E-04	PRS-CSx
Ovary	PRS7	rs8113651	19	35388152	G	A	1.02E-03	PRS-CSx
Ovary	PRS7	rs812545	10	48626479	T	C	3.32E-04	PRS-CSx
Ovary	PRS7	rs8130587	21	41865052	T	C	-9.84E-06	PRS-CSx
Ovary	PRS7	rs8134161	21	42164879	A	G	1.70E-03	PRS-CSx
Ovary	PRS7	rs8134359	21	44104825	T	G	-1.08E-04	PRS-CSx

Ovary	PRS7	rs815867	9	84170701	G	A	-2.61E-03	PRS-CSx
Ovary	PRS7	rs816496	3	39608981	G	A	-1.98E-05	PRS-CSx
Ovary	PRS7	rs816640	10	579869	G	A	-2.82E-04	PRS-CSx
Ovary	PRS7	rs8176720	9	136132873	C	T	-8.53E-04	PRS-CSx
Ovary	PRS7	rs8179252	2	27746832	C	A	3.07E-04	PRS-CSx
Ovary	PRS7	rs821466	16	57035375	C	T	1.99E-03	PRS-CSx
Ovary	PRS7	rs82790	19	30626712	A	C	6.29E-04	PRS-CSx
Ovary	PRS7	rs830131	19	47969748	C	T	2.93E-04	PRS-CSx
Ovary	PRS7	rs830132	19	47969980	G	A	4.42E-04	PRS-CSx
Ovary	PRS7	rs835578	1	120406583	T	C	-9.26E-04	PRS-CSx
Ovary	PRS7	rs840193	4	45556947	T	C	-2.07E-03	PRS-CSx
Ovary	PRS7	rs842387	13	47279454	A	G	-6.40E-05	PRS-CSx
Ovary	PRS7	rs842397	13	47274727	T	C	-1.01E-04	PRS-CSx
Ovary	PRS7	rs842400	13	47271875	G	A	-2.02E-05	PRS-CSx
Ovary	PRS7	rs842405	13	47270592	A	G	6.92E-05	PRS-CSx
Ovary	PRS7	rs847268	14	72705544	C	T	-5.07E-04	PRS-CSx
Ovary	PRS7	rs84839	20	45044830	C	T	6.89E-04	PRS-CSx
Ovary	PRS7	rs852201	6	158112048	A	G	-1.07E-04	PRS-CSx
Ovary	PRS7	rs856797	3	144084535	T	C	-5.64E-04	PRS-CSx
Ovary	PRS7	rs856801	3	144088044	A	G	-7.35E-04	PRS-CSx
Ovary	PRS7	rs860184	1	209841273	A	G	-3.07E-03	PRS-CSx
Ovary	PRS7	rs860674	1	120402764	G	A	-6.58E-04	PRS-CSx
Ovary	PRS7	rs869323	13	100258502	A	G	3.58E-04	PRS-CSx
Ovary	PRS7	rs871097	5	168208107	C	T	2.85E-03	PRS-CSx
Ovary	PRS7	rs871771	9	85209722	T	C	-1.34E-03	PRS-CSx
Ovary	PRS7	rs874547	5	66144813	T	C	1.85E-05	PRS-CSx
Ovary	PRS7	rs874686	12	52765581	T	C	3.49E-04	PRS-CSx
Ovary	PRS7	rs874960	22	48318132	G	A	-1.05E-04	PRS-CSx
Ovary	PRS7	rs875755	2	75844679	C	T	-4.76E-04	PRS-CSx
Ovary	PRS7	rs875780	9	37647741	G	A	2.13E-04	PRS-CSx
Ovary	PRS7	rs877068	1	110698045	T	C	-3.53E-04	PRS-CSx
Ovary	PRS7	rs877138	11	113256508	G	A	1.81E-04	PRS-CSx
Ovary	PRS7	rs877266	14	69779461	G	A	1.66E-04	PRS-CSx
Ovary	PRS7	rs877603	10	67111828	C	A	-2.70E-04	PRS-CSx
Ovary	PRS7	rs879104	8	135609537	G	A	6.44E-04	PRS-CSx
Ovary	PRS7	rs879217	7	4860835	G	A	-8.19E-04	PRS-CSx
Ovary	PRS7	rs884129	1	18700256	A	C	1.02E-04	PRS-CSx
Ovary	PRS7	rs887592	14	74689960	A	G	-1.65E-03	PRS-CSx
Ovary	PRS7	rs887620	7	28421983	A	G	2.70E-03	PRS-CSx
Ovary	PRS7	rs887623	7	28422134	C	T	1.17E-03	PRS-CSx
Ovary	PRS7	rs887847	7	8179782	T	C	-6.36E-04	PRS-CSx
Ovary	PRS7	rs889809	16	12506070	A	G	1.93E-04	PRS-CSx
Ovary	PRS7	rs889811	16	12505469	T	G	1.27E-04	PRS-CSx
Ovary	PRS7	rs894343	8	135612595	A	G	5.06E-04	PRS-CSx
Ovary	PRS7	rs898311	11	112623291	T	C	2.12E-04	PRS-CSx
Ovary	PRS7	rs902557	17	32250107	T	C	1.68E-03	PRS-CSx
Ovary	PRS7	rs902711	3	70032376	T	C	5.49E-04	PRS-CSx
Ovary	PRS7	rs903056	3	14810290	A	G	1.42E-03	PRS-CSx
Ovary	PRS7	rs904053	8	28202936	C	T	1.30E-04	PRS-CSx
Ovary	PRS7	rs904418	3	69616814	C	T	-2.30E-04	PRS-CSx
Ovary	PRS7	rs905849	5	82284721	G	A	2.05E-04	PRS-CSx
Ovary	PRS7	rs905850	5	82284589	G	A	9.80E-06	PRS-CSx
Ovary	PRS7	rs906884	11	24538644	G	A	4.53E-03	PRS-CSx
Ovary	PRS7	rs912069	10	10945351	T	C	7.04E-05	PRS-CSx
Ovary	PRS7	rs912128	13	100074381	A	G	8.57E-06	PRS-CSx
Ovary	PRS7	rs912129	13	100033652	A	G	-2.86E-04	PRS-CSx

Ovary	PRS7	rs913918	10	11069538	C	A	-1.52E-04	PRS-CSx
Ovary	PRS7	rs914428	9	138669261	G	A	-3.06E-03	PRS-CSx
Ovary	PRS7	rs915039	20	56596617	T	C	9.68E-04	PRS-CSx
Ovary	PRS7	rs917132	7	46959491	A	C	-1.36E-03	PRS-CSx
Ovary	PRS7	rs918377	5	143197848	A	G	-3.25E-04	PRS-CSx
Ovary	PRS7	rs918736	16	74302727	C	T	3.60E-04	PRS-CSx
Ovary	PRS7	rs919803	19	30987104	C	T	2.72E-04	PRS-CSx
Ovary	PRS7	rs923677	4	175781686	T	C	-2.18E-03	PRS-CSx
Ovary	PRS7	rs924181	1	110680114	A	G	-5.69E-04	PRS-CSx
Ovary	PRS7	rs924937	4	76769442	G	A	-3.92E-04	PRS-CSx
Ovary	PRS7	rs925193	2	142467595	G	A	1.18E-03	PRS-CSx
Ovary	PRS7	rs925585	15	99315487	G	A	2.11E-03	PRS-CSx
Ovary	PRS7	rs9271588	6	32590953	C	T	-5.35E-04	PRS-CSx
Ovary	PRS7	rs928137	8	132027651	G	A	-4.26E-05	PRS-CSx
Ovary	PRS7	rs9284665	4	157287820	C	T	1.01E-03	PRS-CSx
Ovary	PRS7	rs9288298	2	154775645	A	G	-3.67E-04	PRS-CSx
Ovary	PRS7	rs9290953	3	190546144	T	C	7.73E-04	PRS-CSx
Ovary	PRS7	rs9290954	3	190548613	A	G	-5.09E-04	PRS-CSx
Ovary	PRS7	rs929474	17	68724036	A	G	1.02E-04	PRS-CSx
Ovary	PRS7	rs9296619	6	49641255	C	T	-2.44E-04	PRS-CSx
Ovary	PRS7	rs9297677	8	124832926	T	C	1.65E-03	PRS-CSx
Ovary	PRS7	rs9297909	8	93424621	T	G	-7.70E-06	PRS-CSx
Ovary	PRS7	rs9298891	9	27893210	C	T	8.37E-04	PRS-CSx
Ovary	PRS7	rs9299284	9	125471244	G	A	-1.41E-03	PRS-CSx
Ovary	PRS7	rs9303624	17	27562990	T	C	-1.72E-04	PRS-CSx
Ovary	PRS7	rs9311746	3	60040012	T	C	3.13E-04	PRS-CSx
Ovary	PRS7	rs9317099	13	60696373	C	T	-7.41E-04	PRS-CSx
Ovary	PRS7	rs9322311	6	151605878	G	T	1.07E-04	PRS-CSx
Ovary	PRS7	rs9323465	14	66266677	G	T	-6.62E-04	PRS-CSx
Ovary	PRS7	rs9333594	7	155604941	T	C	8.23E-04	PRS-CSx
Ovary	PRS7	rs933360	7	50758245	C	T	4.80E-04	PRS-CSx
Ovary	PRS7	rs934607	2	212252809	G	A	-6.88E-04	PRS-CSx
Ovary	PRS7	rs9347482	6	158099736	A	G	-7.65E-04	PRS-CSx
Ovary	PRS7	rs9347511	6	161855785	C	T	2.87E-03	PRS-CSx
Ovary	PRS7	rs935119	8	135638353	A	G	3.17E-04	PRS-CSx
Ovary	PRS7	rs9353856	6	92287540	A	G	-2.05E-04	PRS-CSx
Ovary	PRS7	rs9355891	6	158115457	C	T	-2.65E-04	PRS-CSx
Ovary	PRS7	rs9356716	6	19897400	T	C	9.89E-05	PRS-CSx
Ovary	PRS7	rs9368039	6	19216822	G	A	4.36E-05	PRS-CSx
Ovary	PRS7	rs936912	5	176010125	C	A	5.82E-04	PRS-CSx
Ovary	PRS7	rs9379188	6	8362964	T	C	2.34E-03	PRS-CSx
Ovary	PRS7	rs9379691	6	24807829	A	G	-1.28E-04	PRS-CSx
Ovary	PRS7	rs9379692	6	24809771	A	G	-6.13E-05	PRS-CSx
Ovary	PRS7	rs9383877	6	151606123	A	G	1.65E-04	PRS-CSx
Ovary	PRS7	rs938394	3	108871952	G	A	7.55E-04	PRS-CSx
Ovary	PRS7	rs938651	8	129555443	C	A	-4.26E-03	PRS-CSx
Ovary	PRS7	rs938671	17	40673721	C	T	-9.86E-05	PRS-CSx
Ovary	PRS7	rs938672	17	40659216	G	A	3.08E-04	PRS-CSx
Ovary	PRS7	rs9393587	6	24809853	C	T	1.39E-04	PRS-CSx
Ovary	PRS7	rs9396060	6	55079046	C	A	-2.63E-03	PRS-CSx
Ovary	PRS7	rs9405933	6	6496834	A	G	-3.01E-05	PRS-CSx
Ovary	PRS7	rs941984	15	81068593	G	A	8.45E-05	PRS-CSx
Ovary	PRS7	rs941991	6	19268860	C	T	-7.69E-05	PRS-CSx
Ovary	PRS7	rs9421763	10	48556423	A	G	6.66E-04	PRS-CSx
Ovary	PRS7	rs9422352	10	48607823	C	T	1.11E-04	PRS-CSx
Ovary	PRS7	rs9424113	10	10942921	C	T	-1.63E-04	PRS-CSx

Ovary	PRS7	rs9424303	1	3736319	A	G	-1.27E-04	PRS-CSx
Ovary	PRS7	rs9430071	1	211737647	C	T	1.32E-03	PRS-CSx
Ovary	PRS7	rs9434833	1	7144893	T	C	1.50E-03	PRS-CSx
Ovary	PRS7	rs9434834	1	7146809	C	T	8.41E-04	PRS-CSx
Ovary	PRS7	rs9460309	6	19220652	G	A	-2.55E-04	PRS-CSx
Ovary	PRS7	rs9462578	6	39811883	G	T	5.09E-04	PRS-CSx
Ovary	PRS7	rs9462772	6	42084516	C	T	2.60E-04	PRS-CSx
Ovary	PRS7	rs9465283	6	19220684	C	T	-2.24E-04	PRS-CSx
Ovary	PRS7	rs9476596	6	14582008	T	C	-9.67E-04	PRS-CSx
Ovary	PRS7	rs9477205	6	16672810	A	G	2.70E-04	PRS-CSx
Ovary	PRS7	rs9479734	6	150444811	C	T	-5.76E-05	PRS-CSx
Ovary	PRS7	rs948552	18	48667368	C	T	-2.14E-04	PRS-CSx
Ovary	PRS7	rs9488329	6	114447603	G	A	1.21E-03	PRS-CSx
Ovary	PRS7	rs9489143	6	117708971	T	C	-7.72E-04	PRS-CSx
Ovary	PRS7	rs949387	12	52691103	T	C	6.12E-04	PRS-CSx
Ovary	PRS7	rs9502289	6	5306457	T	C	2.17E-03	PRS-CSx
Ovary	PRS7	rs9507368	13	25110139	C	T	1.62E-03	PRS-CSx
Ovary	PRS7	rs9529100	13	67161779	T	G	-4.39E-05	PRS-CSx
Ovary	PRS7	rs9532511	13	40766114	C	T	-9.08E-04	PRS-CSx
Ovary	PRS7	rs9532512	13	40769897	A	G	-1.21E-03	PRS-CSx
Ovary	PRS7	rs9538206	13	59361824	G	A	-1.55E-04	PRS-CSx
Ovary	PRS7	rs9542161	13	70589663	G	A	5.65E-05	PRS-CSx
Ovary	PRS7	rs954753	6	119363976	C	T	7.62E-05	PRS-CSx
Ovary	PRS7	rs9549100	13	40768838	A	G	-9.07E-04	PRS-CSx
Ovary	PRS7	rs9549144	13	40851380	A	G	-2.39E-04	PRS-CSx
Ovary	PRS7	rs9552800	13	23599673	A	G	1.84E-03	PRS-CSx
Ovary	PRS7	rs9554581	13	99962892	T	C	-2.96E-04	PRS-CSx
Ovary	PRS7	rs9557217	13	100068267	C	T	9.87E-05	PRS-CSx
Ovary	PRS7	rs9566503	13	40851098	G	A	-1.61E-04	PRS-CSx
Ovary	PRS7	rs9573903	13	77218884	A	G	8.30E-04	PRS-CSx
Ovary	PRS7	rs9584886	13	99298499	A	G	1.19E-04	PRS-CSx
Ovary	PRS7	rs9592449	13	66732565	A	G	2.10E-03	PRS-CSx
Ovary	PRS7	rs9599076	13	66731477	G	A	1.67E-03	PRS-CSx
Ovary	PRS7	rs9606756	22	31006860	G	A	3.42E-03	PRS-CSx
Ovary	PRS7	rs9607541	22	38849420	T	C	-9.46E-04	PRS-CSx
Ovary	PRS7	rs9615234	22	48321123	A	G	2.05E-04	PRS-CSx
Ovary	PRS7	rs9615235	22	48321429	G	A	2.25E-04	PRS-CSx
Ovary	PRS7	rs962292	12	69670186	A	G	3.53E-04	PRS-CSx
Ovary	PRS7	rs962711	8	76519323	G	A	-1.44E-04	PRS-CSx
Ovary	PRS7	rs9634246	12	57034388	G	A	2.95E-04	PRS-CSx
Ovary	PRS7	rs9643974	8	14199014	C	T	-1.47E-03	PRS-CSx
Ovary	PRS7	rs9652580	16	24308825	T	C	-1.96E-03	PRS-CSx
Ovary	PRS7	rs966226	3	146465376	G	A	4.06E-04	PRS-CSx
Ovary	PRS7	rs966659	8	135639045	T	C	3.55E-04	PRS-CSx
Ovary	PRS7	rs9693229	8	76711112	G	A	-9.02E-04	PRS-CSx
Ovary	PRS7	rs9695	9	97365642	A	G	-1.70E-04	PRS-CSx
Ovary	PRS7	rs972528	14	83294601	A	G	-1.67E-03	PRS-CSx
Ovary	PRS7	rs972594	11	9510715	T	C	1.66E-04	PRS-CSx
Ovary	PRS7	rs972905	14	92199558	T	C	4.29E-05	PRS-CSx
Ovary	PRS7	rs975512	1	110690577	A	G	-7.53E-04	PRS-CSx
Ovary	PRS7	rs977505	8	53996131	C	T	1.43E-04	PRS-CSx
Ovary	PRS7	rs9782928	1	111141682	C	T	-1.91E-03	PRS-CSx
Ovary	PRS7	rs978562	12	17057795	A	C	-5.06E-04	PRS-CSx
Ovary	PRS7	rs978600	2	142470836	C	T	6.96E-04	PRS-CSx
Ovary	PRS7	rs9787509	10	111289799	A	G	-1.35E-03	PRS-CSx
Ovary	PRS7	rs9789678	2	208899477	A	G	-2.01E-04	PRS-CSx

Ovary	PRS7	rs9792165	8	17438490	G	A	-1.85E-03	PRS-CSx
Ovary	PRS7	rs979537	6	123983066	T	C	-7.01E-05	PRS-CSx
Ovary	PRS7	rs9808028	2	212305314	C	T	-1.02E-03	PRS-CSx
Ovary	PRS7	rs9811767	3	156351846	G	A	2.36E-03	PRS-CSx
Ovary	PRS7	rs9817090	3	190524107	G	A	-4.63E-04	PRS-CSx
Ovary	PRS7	rs9817346	3	177680993	T	C	4.51E-03	PRS-CSx
Ovary	PRS7	rs9818956	3	84819147	G	T	-5.76E-04	PRS-CSx
Ovary	PRS7	rs982136	4	141798660	A	G	-4.27E-03	PRS-CSx
Ovary	PRS7	rs9821953	3	84804306	G	A	-9.62E-04	PRS-CSx
Ovary	PRS7	rs9828058	3	67047378	G	A	1.69E-04	PRS-CSx
Ovary	PRS7	rs9830737	3	190537545	A	G	7.32E-04	PRS-CSx
Ovary	PRS7	rs9832475	3	190525217	T	G	-4.68E-04	PRS-CSx
Ovary	PRS7	rs9835908	3	146414837	G	A	-3.24E-04	PRS-CSx
Ovary	PRS7	rs9836760	3	146432172	A	C	-5.01E-04	PRS-CSx
Ovary	PRS7	rs9839064	3	84822412	G	T	-6.64E-04	PRS-CSx
Ovary	PRS7	rs9843503	3	45849270	G	T	1.42E-03	PRS-CSx
Ovary	PRS7	rs9843828	3	190527587	G	A	7.69E-04	PRS-CSx
Ovary	PRS7	rs9845940	3	146499301	A	C	-2.81E-04	PRS-CSx
Ovary	PRS7	rs9850216	3	66926692	G	A	1.45E-04	PRS-CSx
Ovary	PRS7	rs9851021	3	190523757	A	G	-2.94E-04	PRS-CSx
Ovary	PRS7	rs9856351	3	190541920	A	C	6.62E-04	PRS-CSx
Ovary	PRS7	rs9857259	3	190534993	G	A	6.07E-04	PRS-CSx
Ovary	PRS7	rs9861781	3	146495077	G	A	-3.18E-04	PRS-CSx
Ovary	PRS7	rs9870207	3	190525516	A	G	6.81E-04	PRS-CSx
Ovary	PRS7	rs9870349	3	84834126	C	A	-8.98E-04	PRS-CSx
Ovary	PRS7	rs9870986	3	146479144	G	A	2.94E-04	PRS-CSx
Ovary	PRS7	rs9871995	3	146419523	A	G	-3.41E-04	PRS-CSx
Ovary	PRS7	rs9872768	3	146414212	C	T	8.89E-04	PRS-CSx
Ovary	PRS7	rs9873709	3	99711687	A	G	1.18E-04	PRS-CSx
Ovary	PRS7	rs9873800	3	188332618	T	C	-2.37E-04	PRS-CSx
Ovary	PRS7	rs9874421	3	193730859	A	G	-7.07E-05	PRS-CSx
Ovary	PRS7	rs9874501	3	166420044	T	G	1.01E-03	PRS-CSx
Ovary	PRS7	rs9875865	3	66906996	C	A	1.21E-03	PRS-CSx
Ovary	PRS7	rs9877090	3	67008841	A	C	3.21E-04	PRS-CSx
Ovary	PRS7	rs9877250	3	190535183	T	C	6.48E-04	PRS-CSx
Ovary	PRS7	rs9877428	3	67039109	T	C	1.59E-04	PRS-CSx
Ovary	PRS7	rs9878418	3	190539107	A	C	-6.75E-04	PRS-CSx
Ovary	PRS7	rs988016	13	78904481	T	G	8.67E-04	PRS-CSx
Ovary	PRS7	rs9880526	3	195637099	A	G	-3.00E-03	PRS-CSx
Ovary	PRS7	rs9880539	3	25883241	A	G	-4.56E-04	PRS-CSx
Ovary	PRS7	rs9882970	3	88963355	T	C	-1.21E-04	PRS-CSx
Ovary	PRS7	rs9889755	17	29234505	T	C	2.54E-04	PRS-CSx
Ovary	PRS7	rs9890618	17	14936231	A	G	1.44E-03	PRS-CSx
Ovary	PRS7	rs989178	20	56620597	C	T	1.70E-03	PRS-CSx
Ovary	PRS7	rs9895684	17	29242600	A	G	2.78E-04	PRS-CSx
Ovary	PRS7	rs9896095	17	29187497	G	A	2.88E-04	PRS-CSx
Ovary	PRS7	rs989656	12	24166066	T	G	2.12E-03	PRS-CSx
Ovary	PRS7	rs9899349	17	29200122	T	C	5.99E-04	PRS-CSx
Ovary	PRS7	rs9906593	17	9588715	A	C	5.83E-04	PRS-CSx
Ovary	PRS7	rs9907514	17	68731219	A	G	-9.06E-06	PRS-CSx
Ovary	PRS7	rs9909497	17	29173228	A	C	5.32E-04	PRS-CSx
Ovary	PRS7	rs9911989	17	29237702	T	G	5.55E-04	PRS-CSx
Ovary	PRS7	rs9913501	17	40650422	C	T	1.03E-04	PRS-CSx
Ovary	PRS7	rs9913782	17	29237384	G	A	7.30E-04	PRS-CSx
Ovary	PRS7	rs9914220	17	76362890	T	C	9.30E-04	PRS-CSx
Ovary	PRS7	rs9914271	17	29232625	A	G	1.83E-04	PRS-CSx

Ovary	PRS7	rs9915139	17	29158515	G	A	2.66E-04	PRS-CSx
Ovary	PRS7	rs9916351	17	78239532	T	C	4.14E-05	PRS-CSx
Ovary	PRS7	rs9916623	17	13443798	T	C	5.71E-04	PRS-CSx
Ovary	PRS7	rs9917667	3	96973753	T	G	9.41E-04	PRS-CSx
Ovary	PRS7	rs9918275	5	66129815	C	T	-1.99E-05	PRS-CSx
Ovary	PRS7	rs9919069	9	37638972	C	T	-2.33E-04	PRS-CSx
Ovary	PRS7	rs9930051	16	83388692	G	A	1.86E-03	PRS-CSx
Ovary	PRS7	rs9930312	16	83146684	T	G	-6.39E-04	PRS-CSx
Ovary	PRS7	rs9935986	16	74287577	A	G	7.21E-05	PRS-CSx
Ovary	PRS7	rs9946404	18	2391697	T	C	1.86E-03	PRS-CSx
Ovary	PRS7	rs9949574	18	48666626	G	A	-1.17E-03	PRS-CSx
Ovary	PRS7	rs9952591	18	27374311	A	G	-2.57E-03	PRS-CSx
Ovary	PRS7	rs9955940	18	10232227	A	G	-2.31E-03	PRS-CSx
Ovary	PRS7	rs996567	12	100056529	A	G	-5.53E-05	PRS-CSx
Ovary	PRS7	rs9971308	10	114222454	G	A	6.73E-04	PRS-CSx
Ovary	PRS7	rs9972739	16	80462352	A	G	1.50E-04	PRS-CSx
Ovary	PRS7	rs997340	18	22621340	C	A	-4.41E-04	PRS-CSx
Ovary	PRS7	rs9978851	21	15732024	G	A	2.36E-05	PRS-CSx
Ovary	PRS7	rs9979739	21	19561638	C	T	1.03E-03	PRS-CSx
Ovary	PRS7	rs998022	12	69669225	A	G	3.74E-04	PRS-CSx
Ovary	PRS7	rs998791	8	59724602	G	A	-4.69E-04	PRS-CSx
Ovary	PRS7	rs9993317	4	184226121	A	C	8.44E-04	PRS-CSx
Ovary	PRS7	rs9995572	4	86381967	G	T	8.09E-04	PRS-CSx
Ovary	PRS7	rs9995880	4	45517254	C	T	-1.59E-03	PRS-CSx
Ovary	PRS7	rs9995999	4	169209583	T	G	6.50E-04	PRS-CSx
Ovary	PRS7	rs9996190	4	184196516	T	G	1.48E-03	PRS-CSx
Ovary	PRS7	rs999797	17	29159329	A	G	5.58E-04	PRS-CSx
Ovary	PRS7	rs999798	17	29158840	G	A	2.35E-04	PRS-CSx
Prostate	PRS4	rs7542260	1	5743196	T	C	0.138	33398198
Prostate	PRS4	rs1811698	1	150772613	C	T	0.087	33398198
Prostate	PRS4	rs10127983	1	153923276	T	C	0.070	33398198
Prostate	PRS4	rs56103503	1	154980351	T	C	0.056	33398198
Prostate	PRS4	rs80237341	1	157119915	C	G	0.153	33398198
Prostate	PRS4	rs6660538	1	163295678	A	C	0.067	33398198
Prostate	PRS4	rs4075646	1	167135941	T	A	0.070	33398198
Prostate	PRS4	rs507603	1	179897070	A	C	0.092	33398198
Prostate	PRS4	rs4245739	1	204518842	A	C	0.131	33398198
Prostate	PRS4	rs708723	1	205739266	C	T	0.070	33398198
Prostate	PRS4	rs1990613	2	10781975	T	C	0.050	33398198
Prostate	PRS4	rs7602028	2	16016503	C	A	0.098	33398198
Prostate	PRS4	rs9306894	2	20878105	G	A	0.126	33398198
Prostate	PRS4	rs7591218	2	43637998	A	G	0.121	33398198
Prostate	PRS4	rs28514770	2	43851282	C	G	0.112	33398198
Prostate	PRS4	rs11125927	2	62752975	G	A	0.141	33398198
Prostate	PRS4	rs58235267	2	63277843	G	C	0.097	33398198
Prostate	PRS4	rs2028900	2	85767735	C	T	0.089	33398198
Prostate	PRS4	rs11691517	2	111893096	T	G	0.056	33398198
Prostate	PRS4	rs111595856	2	121103598	T	C	0.106	33398198
Prostate	PRS4	rs10206072	2	121373466	G	A	0.044	33398198
Prostate	PRS4	rs16854905	2	169012955	C	T	0.077	33398198
Prostate	PRS4	rs77167534	2	173319930	C	T	0.154	33398198
Prostate	PRS4	rs34925593	2	174234547	C	T	0.074	33398198
Prostate	PRS4	rs1861270	2	202126615	G	A	0.054	33398198
Prostate	PRS4	rs2292884	2	238443226	G	A	0.067	33398198
Prostate	PRS4	rs2074840	2	242141719	C	T	0.056	33398198
Prostate	PRS4	rs6550597	3	18738940	A	G	0.081	33398198

Prostate	PRS4	rs7618603	3	23153062	A	C	0.091	33398198
Prostate	PRS4	rs143745027	3	87144017	G	A	0.179	33398198
Prostate	PRS4	rs7628934	3	87175984	C	T	0.098	33398198
Prostate	PRS4	rs1283104	3	106962521	G	C	0.039	33398198
Prostate	PRS4	rs2271494	3	113300183	A	T	0.083	33398198
Prostate	PRS4	rs2811476	3	127898501	C	A	0.129	33398198
Prostate	PRS4	rs35006112	3	128213994	G	A	0.126	33398198
Prostate	PRS4	rs1457063	3	137562823	A	G	0.060	33398198
Prostate	PRS4	rs7650602	3	141147414	C	T	0.077	33398198
Prostate	PRS4	rs2293607	3	169482335	T	C	0.049	33398198
Prostate	PRS4	rs78416326	3	170074517	G	C	0.150	33398198
Prostate	PRS4	rs6853490	4	95544718	G	A	0.059	33398198
Prostate	PRS4	rs7679673	4	106061534	C	A	0.068	33398198
Prostate	PRS4	rs17035310	4	106064754	C	T	0.166	33398198
Prostate	PRS4	rs77821238	4	140948835	C	T	0.107	33398198
Prostate	PRS4	rs72725734	4	146879237	G	A	0.087	33398198
Prostate	PRS4	rs147762399	4	152030340	T	C	0.071	33398198
Prostate	PRS4	rs2242652	5	1280028	G	A	0.152	33398198
Prostate	PRS4	rs2736098	5	1294086	T	C	0.076	33398198
Prostate	PRS4	rs4975758	5	1891174	G	C	0.204	33398198
Prostate	PRS4	rs10941370	5	37833419	T	C	0.040	33398198
Prostate	PRS4	rs61739424	5	177683905	G	A	0.091	33398198
Prostate	PRS4	rs2672843	5	177891551	G	A	0.081	33398198
Prostate	PRS4	rs6927369	6	21330689	C	T	0.092	33398198
Prostate	PRS4	rs4269363	6	21471490	G	A	0.079	33398198
Prostate	PRS4	rs9469899	6	34793124	A	G	0.054	33398198
Prostate	PRS4	rs4714485	6	41536587	G	T	0.136	33398198
Prostate	PRS4	rs9443189	6	76495882	A	G	0.083	33398198
Prostate	PRS4	rs339351	6	117200434	C	A	0.200	33398198
Prostate	PRS4	rs13215045	6	153447516	C	T	0.068	33398198
Prostate	PRS4	rs963800	6	160150279	C	T	0.083	33398198
Prostate	PRS4	rs4646284	6	160581543	TG	T	0.120	33398198
Prostate	PRS4	rs9655205	7	20999211	C	A	0.069	33398198
Prostate	PRS4	rs6956484	7	27564862	A	C	0.134	33398198
Prostate	PRS4	rs10486567	7	27976563	G	A	0.093	33398198
Prostate	PRS4	rs12701838	7	40877473	A	G	0.188	33398198
Prostate	PRS4	rs834608	7	47451918	A	T	0.043	33398198
Prostate	PRS4	rs6955627	7	92577760	C	T	0.071	33398198
Prostate	PRS4	rs4727386	7	97688440	A	G	0.084	33398198
Prostate	PRS4	rs870167	8	8498803	G	A	0.066	33398198
Prostate	PRS4	rs6557704	8	23470785	A	G	0.106	33398198
Prostate	PRS4	rs1160267	8	23529521	G	A	0.239	33398198
Prostate	PRS4	rs12677206	8	26063165	A	C	0.092	33398198
Prostate	PRS4	rs6984837	8	127901649	G	A	0.128	33398198
Prostate	PRS4	rs7011138	8	127922200	A	T	-0.196	33398198
Prostate	PRS4	rs7463326	8	128027954	G	A	0.235	33398198
Prostate	PRS4	rs72725879	8	128103969	T	C	0.535	33398198
Prostate	PRS4	rs17464492	8	128342866	A	G	0.162	33398198
Prostate	PRS4	rs6983267	8	128413305	G	T	0.138	33398198
Prostate	PRS4	rs10090154	8	128532137	T	C	0.477	33398198
Prostate	PRS4	rs34265760	8	128535543	T	C	0.195	33398198
Prostate	PRS4	rs12549761	8	128540776	C	G	0.093	33398198
Prostate	PRS4	rs10122990	9	19072246	C	A	0.069	33398198
Prostate	PRS4	rs817872	9	110144887	C	T	0.068	33398198
Prostate	PRS4	rs12634	9	132573536	T	G	0.072	33398198
Prostate	PRS4	rs10993994	10	51549496	T	C	0.166	33398198

Prostate	PRS4	rs11817544	10	80236999	C	A	0.153	33398198
Prostate	PRS4	rs12412705	10	80835998	C	T	0.108	33398198
Prostate	PRS4	rs1935581	10	90195149	C	T	0.052	33398198
Prostate	PRS4	rs12262998	10	104428716	C	T	0.077	33398198
Prostate	PRS4	rs10885396	10	114711755	T	C	0.065	33398198
Prostate	PRS4	rs4558107	10	122794926	A	G	0.075	33398198
Prostate	PRS4	rs140783917	10	122834482	C	T	0.197	33398198
Prostate	PRS4	rs10788167	10	123054018	T	A	0.066	33398198
Prostate	PRS4	rs10749415	10	123185303	A	G	0.248	33398198
Prostate	PRS4	rs61890184	11	7547587	A	G	0.139	33398198
Prostate	PRS4	rs1048374	11	58902679	G	A	0.141	33398198
Prostate	PRS4	rs3018690	11	68882926	T	C	0.071	33398198
Prostate	PRS4	rs3918298	11	69463273	A	G	0.149	33398198
Prostate	PRS4	rs56159348	11	76267331	T	G	0.080	33398198
Prostate	PRS4	rs878987	11	134266372	G	A	0.103	33398198
Prostate	PRS4	rs77216612	12	12877983	A	G	0.098	33398198
Prostate	PRS4	rs56222401	12	49672714	G	A	0.090	33398198
Prostate	PRS4	rs187809440	12	53329231	T	C	0.304	33398198
Prostate	PRS4	rs7968403	12	65012824	T	C	0.081	33398198
Prostate	PRS4	rs4842687	12	90156377	A	G	0.113	33398198
Prostate	PRS4	rs77121786	12	102446675	G	T	0.061	33398198
Prostate	PRS4	rs1270884	12	114685571	A	G	0.063	33398198
Prostate	PRS4	rs7295014	12	133067989	G	A	0.103	33398198
Prostate	PRS4	rs1327653	13	51076440	T	C	0.097	33398198
Prostate	PRS4	rs7489409	13	73716861	C	T	0.135	33398198
Prostate	PRS4	rs6571758	14	37136194	G	A	0.093	33398198
Prostate	PRS4	rs8005621	14	61106699	G	A	0.097	33398198
Prostate	PRS4	rs79133931	14	64687926	T	C	0.178	33398198
Prostate	PRS4	rs767127	14	69134264	G	A	0.066	33398198
Prostate	PRS4	rs17565772	14	70756333	G	A	0.074	33398198
Prostate	PRS4	rs11561564	15	40965044	G	A	0.057	33398198
Prostate	PRS4	rs8023793	15	66942093	A	C	0.110	33398198
Prostate	PRS4	rs12913603	15	70668824	A	C	0.074	33398198
Prostate	PRS4	rs13380763	16	54678305	C	T	0.093	33398198
Prostate	PRS4	rs8052913	16	82166181	C	T	0.045	33398198
Prostate	PRS4	rs684232	17	618965	C	T	0.070	33398198
Prostate	PRS4	rs72811270	17	12585459	A	G	0.089	33398198
Prostate	PRS4	rs4795646	17	30092898	G	A	0.054	33398198
Prostate	PRS4	rs3110641	17	36047417	A	G	0.076	33398198
Prostate	PRS4	rs11649743	17	36074979	G	A	0.140	33398198
Prostate	PRS4	rs11263763	17	36103565	A	G	0.270	33398198
Prostate	PRS4	rs2960158	17	47380305	T	C	0.077	33398198
Prostate	PRS4	rs8089411	18	51771322	C	T	0.069	33398198
Prostate	PRS4	rs11876000	18	73035513	T	G	0.041	33398198
Prostate	PRS4	rs59710626	19	38548094	G	T	0.056	33398198
Prostate	PRS4	rs4802297	19	38738130	G	C	0.057	33398198
Prostate	PRS4	rs2659051	19	51345568	G	C	0.135	33398198
Prostate	PRS4	rs6039055	20	867324	A	G	0.047	33398198
Prostate	PRS4	rs73909841	20	49548807	T	C	0.184	33398198
Prostate	PRS4	rs381331	20	62229989	A	G	0.061	33398198
Prostate	PRS4	rs3787099	20	62307517	A	G	0.145	33398198
Prostate	PRS4	rs1058319	20	62374389	C	T	0.108	33398198
Prostate	PRS4	rs1978060	22	19749525	G	A	0.077	33398198
Prostate	PRS4	rs138708	22	39138332	G	A	0.161	33398198
Prostate	PRS4	rs5759167	22	43500212	G	T	0.082	33398198
Prostate	PRS4	rs9615099	22	45698149	T	A	0.053	33398198

Prostate	PRS4	rs960417	X	9811095	A	G	0.038	33398198
Prostate	PRS4	rs5972255	X	30896320	T	C	0.042	33398198
Prostate	PRS4	rs11338635	X	51245276	GA	G	0.118	33398198
Prostate	PRS4	rs4826594	X	54454406	A	G	0.058	33398198
Prostate	PRS4	rs10122495	9	34049779	A	T	-0.051	34594039
Prostate	PRS4	rs10900829	5	133848917	G	A	0.135	34594039
Prostate	PRS4	rs4911252	20	31330115	T	C	-0.074	34594039
Prostate	PRS4	rs10421727	19	17225128	A	G	-0.045	34594039
Prostate	PRS4	rs12776659	10	834057	A	C	0.468	34594039
Prostate	PRS4	rs6899412	6	43700466	T	C	-0.066	34594039
Prostate	PRS4	rs6068691	20	52469400	A	G	-0.045	34594039
Prostate	PRS4	rs58475265	11	134264142	C	T	0.063	34594039
Prostate	PRS4	rs149272765	3	128104469	T	C	0.922	34594039
Prostate	PRS4	rs62451152	7	27993157	C	A	0.297	34594039
Prostate	PRS4	rs61390061	13	73741377	T	C	0.440	34594039
Prostate	PRS4	rs532860474	17	78257346	A	G	0.684	34594039
Prostate	PRS4	rs2823739	21	17661418	G	A	-0.173	34594039
